# Targeting the FNIP2-SERCA2b axis improves metabolic and mitochondrial defects in Ataxia Telangiectasia

**DOI:** 10.1038/s41419-026-08507-5

**Published:** 2026-03-02

**Authors:** Maria Vinciguerra, Catiana El Kharef, Christopher Bruhn, Lucia Falbo, Chiara Milanese, Matteo Audano, Galina V. Beznoussenko, Alexander A. Mironov, Domenico Delia, Marco Foiani, Pier Giorgio Mastroberardino, Nico Mitro, Vincenzo Costanzo

**Affiliations:** 1 IFOM-ETS, The AIRC Institute of Molecular Oncology, Milan, Italy; 2 Department of Oncology and Hematology-Oncology, University of Milan, Milan, Italy; 3 Department of Pharmacological and Biomolecular Sciences “Rodolfo Paoletti”, University of Milan, Milan, Italy; 4 Department of Experimental Oncology, IEO, European Institute of Oncology IRCCS, Milan, Italy

**Keywords:** Spinocerebellar ataxia, Mechanisms of disease

## Abstract

Ataxia telangiectasia (AT) is a rare multisystem disorder caused by the loss of functional ATM protein, leading to immunodeficiency, cancer predisposition, neurodegeneration, diabetes, heart failure, and premature aging. Although ATM’s role as a sensor of DNA double-strand breaks (DSBs) is well established, the mechanisms underlying the diverse AT phenotypes remain incompletely understood, with evidence suggesting they extend beyond DSB sensing. Here, we uncover widespread glycogen accumulation as a key feature of AT cells and tissues, driven by dysregulated glucose metabolism and impaired mitochondrial respiration assessed with a multidimensional approach including metabolomics, flux analysis, histopathology, bioenergetic measurements, and electron tomography. These metabolic defects contribute to reduced cellular viability and premature senescence observed in AT patient-derived cells. Strikingly, inactivation of FNIP2, which controls mitochondrial respiration, partially rescues these defects in AT cellular models. We show that FNIP2 interacts with the SERCA2b calcium channel, and its inactivation enhances cytoplasmic calcium availability, stimulating mitochondrial respiration and increasing glucose consumption. This metabolic reprogramming prevents glycogen accumulation and improves survival in AT primary cells. Our findings provide novel insights into AT pathophysiology and indicate the FNIP2-SERCA2b axis as a novel potential target for mitigating the systemic effects of AT and improving outcomes in this complex disease.

## Introduction

Ataxia Telangiectasia (AT) is a multifaceted autosomal recessive disorder. Affected individuals suffer from neurological deficits, predominantly due to cerebellar atrophy, and exhibit a range of systemic symptoms including ocular telangiectasia, immunodeficiency, premature senescence, heightened sensitivity to ionizing radiation, and a predisposition to cancer, diabetes, and cardiovascular disease [1–3]. ATM, the gene mutated in AT, encodes for a protein kinase that responds to DNA double-strand breaks and oxidative stress by orchestrating a phosphorylation cascade critical for cellular homeostasis [4, 5]. Disruption of ATM function has been implicated in metabolic syndromes in mammalian models, hallmarked by insulin resistance and cardiovascular risk factors [3, 6].

Our investigations have further elucidated the role of ATM in metabolic regulation, specifically highlighting its influence on the pentose phosphate pathway (PPP) [7]. This alternative glucose metabolism route is essential for the generation of nucleotide precursors and the antioxidant cofactor NADPH [8]. We have previously demonstrated ATM’s upregulation of PPP through the activation of glucose-6-phosphate dehydrogenase (G6PD), the pathway’s rate-limiting enzyme [7]. This regulatory mechanism may enhance DNA repair capabilities and promote cellular survival by ensuring a steady supply of nucleotide precursors and antioxidants [7, 9].

A role for ATM in sensing and responding to oxidative stress has now been widely demonstrated [4, 5, 9–11]. Emerging evidence underscores the significance of ATM in maintaining metabolic equilibrium and redox status independent of DNA damage response. Cells from AT individuals exhibit suboptimal growth and accelerated senescence, potentially as a result of metabolic dysregulation and unchecked oxidative stress [1, 12, 13]. However, the interplay between impaired metabolism and the broader implications for AT pathophysiology remains to be fully understood.

In this study, we have conducted comprehensive metabolomic, flux analysis, mitochondrial profiling, and histopathological examinations of cells and tissue samples from several AT patients. Our findings reveal, in addition to oxidative stress, pronounced glycogen accumulation in AT cells and tissues, including brain and heart, linked to glucose homeostasis and mitochondrial respiration impairments.

To discern the impact of aberrant glucose metabolism on AT phenotypes, we explored interventions aimed at normalizing glucose utilization. Remarkably, inhibiting FNIP2, a lesser-studied paralog of FNIP1, which regulates mitochondrial respiration under reductive stress [14], ameliorated glycolytic and respiratory deficiencies, rescuing AT cell viability. Surprisingly, the effects of FNIP2 inactivation are independent of its direct role in cellular metabolism and rely on loss of FNIP2-dependent stimulatory activity on SERCA2b calcium channel, which in turn leads to increased cytoplasmic calcium levels that are able to partially restore normal mitochondrial respiration. These results suggest that FNIP2 contributes to restraining mitochondrial activity under stress conditions in our cell models, possibly to avoid further chronic cellular damage mediated by oxidative stress.

Our insights offer fresh perspectives on AT pathology, with potential implications for diagnostic and therapeutic strategies.

## Results

### Metabolic and oxidative stress response profiling reveals profound disruptions in primary AT cells

**Fig. 1:**
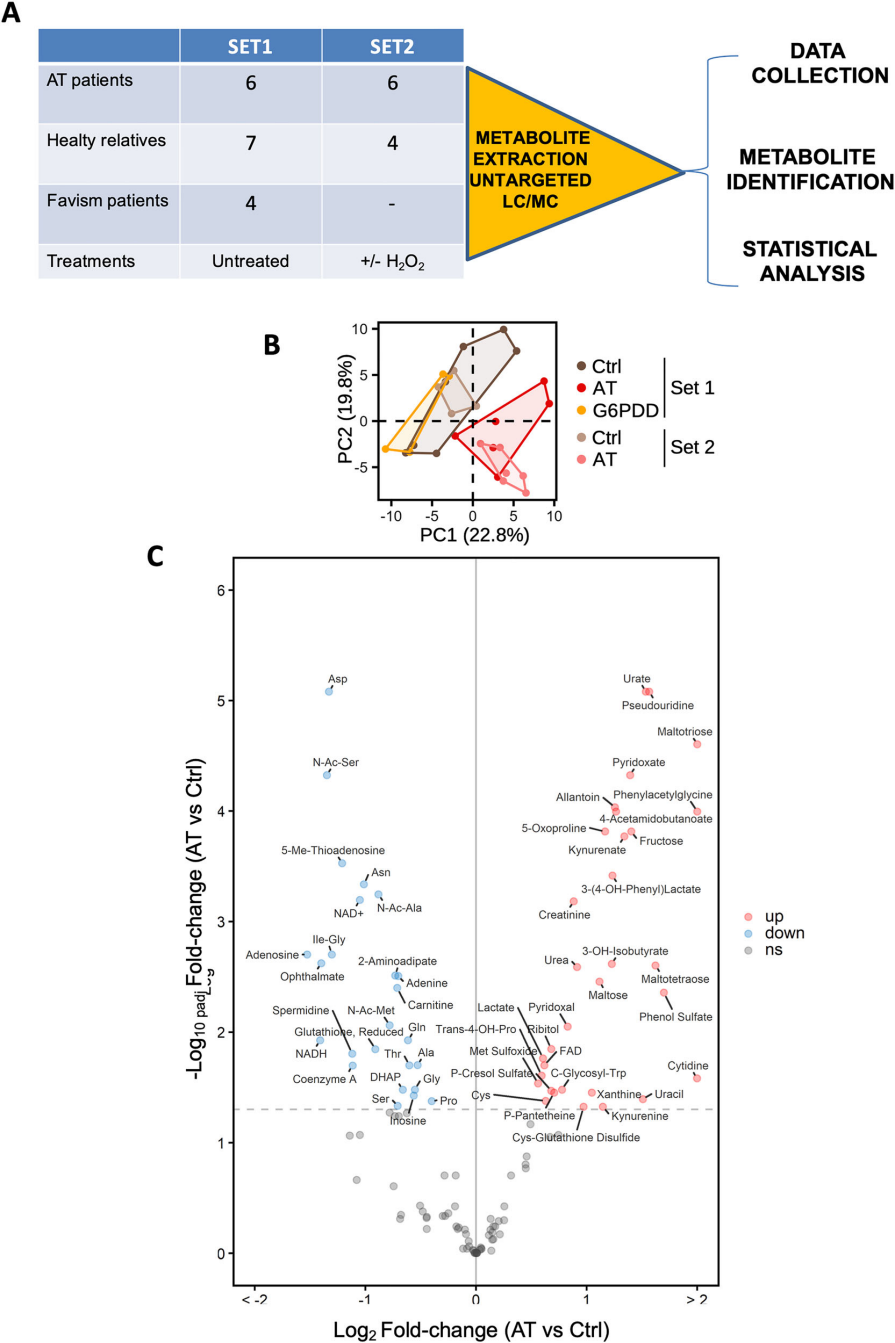
Metabolic and oxidative stress-response profiling reveals profound disruptions in primary AT cells. **A** Diagram illustrating the metabolic analysis conducted on two independent sample sets. Metabolites were identified and quantified by LC–MS and GC–MS. Significant changes were determined using the limma R package, applying an FDR cutoff of 0.05. **B** Principal component analysis of metabolite concentrations in CTRL, AT, and G6PD-deficient (G6PDD) cells. The first two principal components are displayed with the percentage of variance explained. Groups are delineated with convex hulls. **C** Volcano plot showing significantly regulated metabolites in AT vs CTRL cells. Significance analysis was performed by LIMMA, pooling samples from both independent sample sets with batch correction in the statistical model. The *x* and *y* axes show the mean log_2_ metabolite level fold-change in AT vs. CTRL cells, and the Benjamini-Hochberg-adjusted, negative log-transformed *p* value, respectively. Significant metabolites are labeled. Up- and down-regulated metabolites are marked with red and blue fill color, respectively.

We conducted an extensive untargeted metabolomic analysis, comparing primary fibroblasts from AT patients with those from unaffected controls, to identify metabolic irregularities in AT cells. Two experimental sets were processed at different times. Set 1 included independent AT patients and healthy control primary fibroblasts together with primary fibroblasts from favism patients with defective G6PD (Fig. 1A, Set 1; Table S1). Set 2, instead, included fibroblasts treated with chronic H_2_O_2_ to simulate oxidative stress in addition to AT patients and healthy control primary fibroblasts (Fig. 1A, Set 2; Table S2). Standard extraction protocols were employed to isolate water-soluble and non-soluble metabolites, which were then analyzed via LC and GC-MS against standard metabolites [15]. This approach resulted in the robust detection of 120 metabolites across both sample sets (Dataset S1).

Principal component analysis (PCA), incorporating the 120 consistently detectable metabolites, revealed distinct separation between control and AT fibroblasts, indicating reproducible AT-specific metabolic alterations (Fig. 1B). Statistical analysis using the R package ‘limma’ identified significant metabolic changes (false discovery rate = 0.05), delineating an AT metabolite signature comprising 32 up-regulated and 25 downregulated metabolites (Fig. 1C and Dataset S2).

We observed markers of oxidative stress and reduced NAD^+^ levels in AT cell pools. Oxidative stress was evidenced by depleted glutathione and its precursors, and an accumulation of oxidative by-products such as 5-oxoproline [16] (Fig. 1C). Moreover, an aberrant increase in polysaccharide degradation products, including maltotetraose, suggested compromised glucose metabolism (Fig. 1C). We also detected heightened levels of protein and nucleic acid degradation products, indicative of widespread metabolic dysregulation (Fig. 1C).

Further PCA of metabolome datasets from healthy control and AT fibroblast pools, with and without H_2_O_2_ treatment, exhibited similar metabolic perturbations, with H_2_O_2_-induced changes aligning closely with those due to ATM deficiency (Fig. S1A–D). Notably, H_2_O_2_ effects were more pronounced in control cells, suggesting a saturation effect in AT cells (Figs. S1A–D and S2A–D). Given the striking effect of oxidative stress on the AT metabolome, we measured protein thiol disulfides vs. reduced thiols as a measure of protein oxidation [17] and found a significantly reduced ratio in AT cell pools (Fig. S2E), confirming that oxidative stress in AT was indeed high enough to exhaust the endogenous defense system and increase protein oxidation. This led us to hypothesize that oxidative stress plays a central role in metabolic alterations in AT cells.

A major source of NADPH, a powerful antioxidant molecule used by cells to counteract oxidative damage and for lipid synthesis, is the PPP, which is also the major cellular producer of ribose-5-phosphate required for nucleic acid synthesis [8]. We have previously shown that ATM stimulates G6PD, the limiting enzyme of the PPP [7]. As defective G6PD may account for the oxidative stress-induced metabolome alterations, we verified metabolite similarities and differences between AT and G6PD-defective cells derived from patients affected by favism, in which G6PD is defective due to partially inactivating mutations in the G6PD gene [18]. We therefore aligned the AT metabolome with the metabolite profiles of G6PD mutant primary fibroblast cells, normalized with control cells. However, G6PD deficiency alone did not mirror the AT metabolome profile (Figs. S1D and S2C, D), indicating the presence of additional defects underlying AT physiopathology.

Notably, while ATM deficiency and oxidative stress depleted intracellular NAD^+^, which has been mechanistically linked to PARP activation, oxidative stress but not AT deficiency increased the level of the NAD^+^ salvage intermediate nicotinamide riboside. Simultaneously, levels of 1-methyl nicotinamide were reduced, supporting the channeling of nicotinamide mononucleotide towards NAD^+^ biosynthesis rather than methylation to form 1-methyl nicotinamide (Figs. S1D and S2D). Hence, our data show that the oxidative stress phenotype in AT cells is not accompanied by the expected induction of NAD^+^ salvage, which could impede the cells from efficiently counteracting oxidative stress.

Mitochondrial dysfunction is an important internal source of oxidative stress as it leads to significant free radicals’ accumulation. In previous studies, oxygen consumption rate was found to be defective in AT compared to CTRL cells [19–21]. To confirm this behavior we measured mitochondrial respiration activity by monitoring O_2_ consumption in GM02530 (AT) and AG02603 (CTRL) primary fibroblasts, the cells that were used for the experiments shown throughout the paper unless otherwise specified, exploiting the Seahorse platform [22]. Consistent with previous studies, O_2_ consumption rate was found to be defective in AT compared to CTRL cells [19–21]. Standard measurements [23] revealed that basal, ATP-linked (oligomycin), maximal (FCCP), and non-mitochondrial respiration (rotenone/antimycin A) were all reduced in AT cells (Fig. S3A). Consistent with previous reports [20, 21], the mitochondrial-to-nuclear DNA ratio (mtDNA/nDNA) measured by an established assay [24] based on qPCR of mitochondrial NADH dehydrogenase (MT-ND1) and β2-microglobulin (B2M) genes, was slightly increased in AT cells, indicative of an expanded, non-functional mitochondrial mass, likely reflecting a compensatory response to ineffective respiration (Fig. S3B). All functional assays were performed 24-36 h after plating low-passage cultures at low density to avoid artefacts from long-term culture. Under these conditions, a luciferase-based viability and proliferation assay showed no difference in growth or survival between AT and control cells at the time points chosen for the analysis (Fig. S3C). Overall, these experiments confirm that increased oxidation levels in the primary AT fibroblast cells are contributed by defective mitochondrial respiration, which minimally affects cell proliferation and survival at early passage and culture stage.

### ^13^C^6^-Glucose time-course tracing analysis highlights glycolysis and TCA impairments in AT cells

**Fig. 2:**
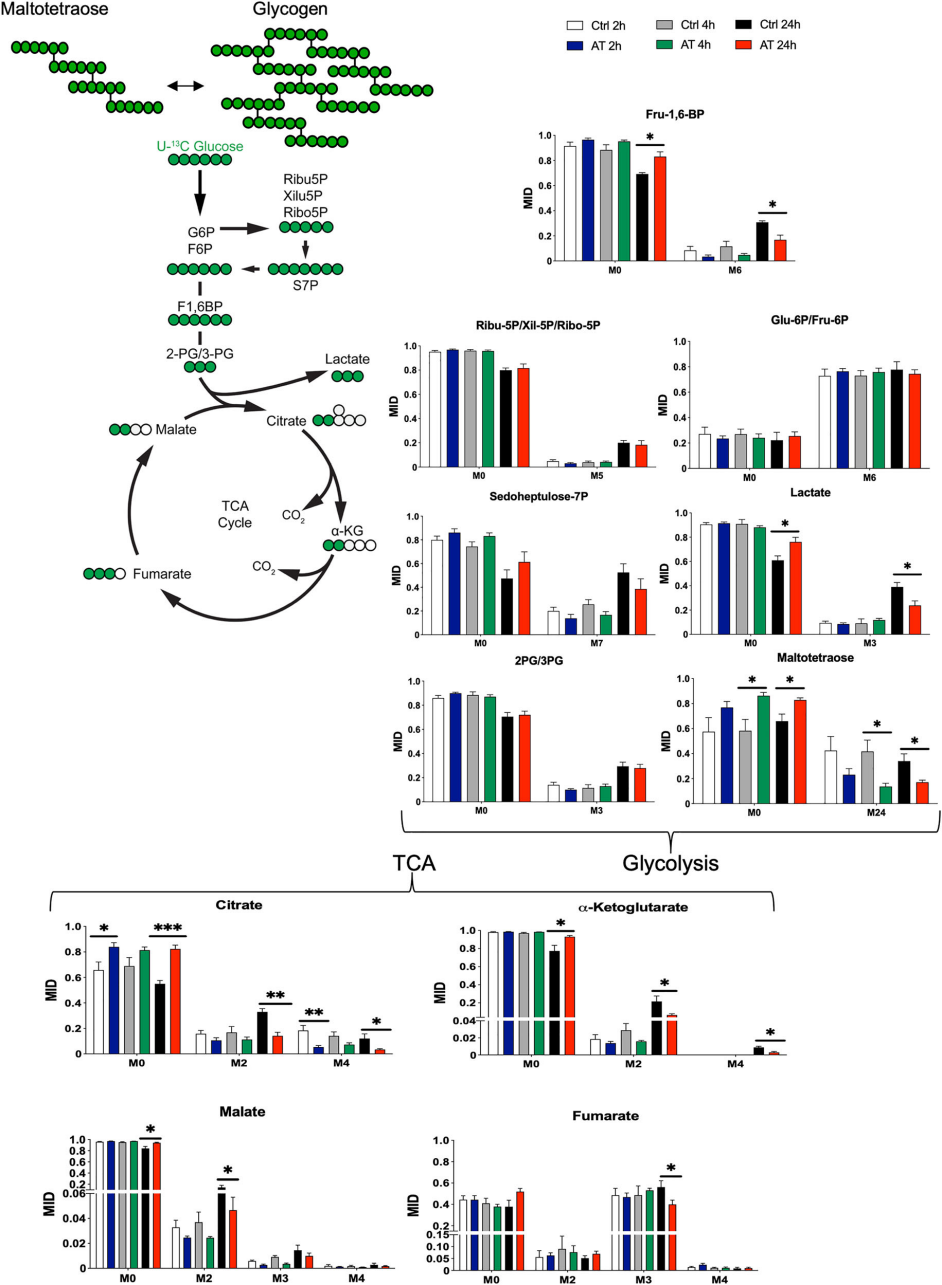
^13^C^6^-Glucose time-course tracing analysis highlights glycolysis and TCA impairments in AT cells. Mass isotopomer distributions (MID) for each intermediate indicated in the graph in low passage CTRL and AT fibroblast cells exposed to U-^13^C^6^-glucose 24–36 h after plating. M0, M2, M3, M4, M5, M6, M7, and M24 indicate the different masses of the isotopomers detected, and the number after M indicates the number of carbons derived from U-^13^C^6^-glucose administered to CTRL and AT cells (M0 represents no labeled carbons). The different time points (2, 4, and 24 h) are indicated in the figure. Statistical analysis was conducted using an unpaired t-test. *P < 0.05; **P < 0.01; ***P < 0.001. *N* = 5 for CTRL and *N* = 6 for AT cells. See “Materials and methods” for more details.

To gain further insight into the metabolic defects of AT cells, we performed a time-course tracing experiment, which provides a dynamic picture of defined metabolic pathways [25, 26]. As the AT metabolic signature identified by the steady-state metabolomic analysis indicated issues with glucose utilization, we monitored the glycolysis and tricarboxylic acids (TCA) cycle activities by uniformly supplementing ^13^C^6^-labelled glucose (U-^13^C^6^-glucose) to low passage live and proliferating primary CTRL and AT fibroblast cells in culture and quantified the percentage of labeled sugar metabolism intermediates at steady-state level (Fig. 2) [27]. The data showed that several metabolites achieved the isotopic steady state, except for those that are changing between CTRL and AT condition (Fig. 2). Strikingly, the analysis revealed AT cells showed reduced levels of M6 fructose-1,6-bisphosphate (Fru-1,6-BP), M3 lactate, M2 citrate, M2 alpha-ketoglutarate and M2 malate after 24 h labeling, all of which are derived by a single round of glycolysis and TCA cycle [27]. Also, the levels of M4 citrate, M4 alpha-ketoglutarate (four glucose-derived carbons), and M3 fumarate (three glucose-derived carbons), a hallmark of multiple rounds of glycolysis coupled to the TCA cycle [27], were decreased in AT cells, indicating that the flux of glucose-derived carbons is impaired in these cells compared to wild type. These results indicate profound defects in glucose usage through glycolysis and TCA in AT cells.

Intriguingly, by monitoring the incorporation of ^13^C-glucose in polysaccharides such as maltotetraose, we detected a statistically significant decreased of labeled-maltotetraose accumulation in AT cells already after 4 h labelling (Fig. 2). The same effect was also observed after 24 h labeling (Fig. 2). The decreased accumulation of labeled maltotetraose in AT cells, revealed by our time-course tracing analysis, was consistent with the elevated levels of released maltotetraose and other oligosaccharides detected in the metabolite profiles of these cells (Figs. 1C and S1D). This impairment in glucose processing may force AT cells to rely more heavily on alternative carbon sources, as evidenced by the increased breakdown products of amino acids and nucleotides (Fig. S1D). This shift in metabolic strategy may represent an adaptation mechanism in AT cells to cope with their glycolytic and TCA cycle deficiencies.

### AT cells and tissues accumulate glycogen

**Fig. 3:**
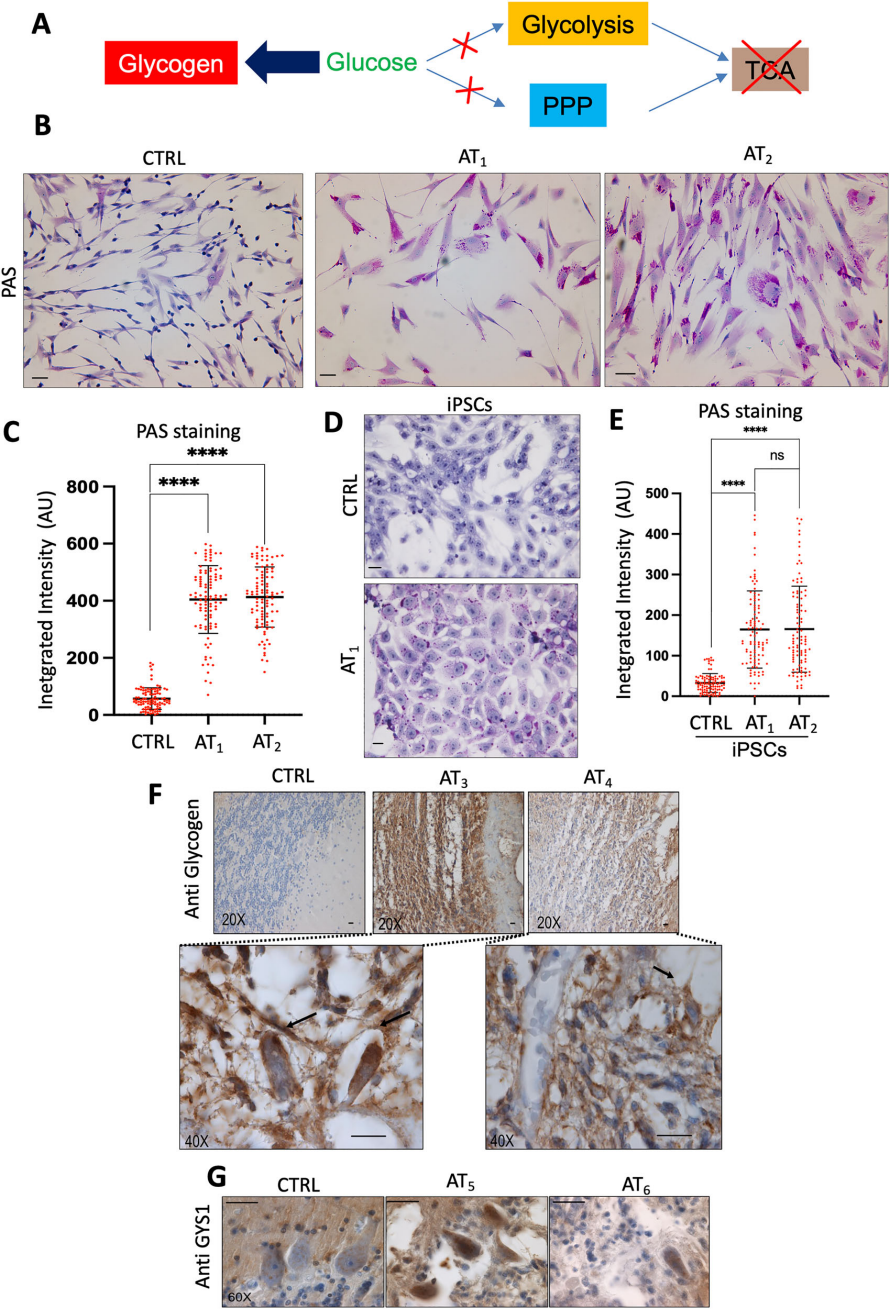
AT cells and tissues accumulate glycogen. **A** Schematic of the metabolic effects leading to altered glucose and glycogen levels due to impaired glycolysis, PPP, and TCA cycle activities. **B** PAS staining of primary fibroblasts from two AT patients compared to CTRL. Cells were at low passage number (12–18) and PAS was performed 24–36 h after plating. Scale bar: 20 μm. **C** Graph showing PAS staining integrated intensity for at least 70 CTRL or AT_1_ and AT_2_ fibroblast cells pooled from three independent experiments, analyzed using Cell Profiler. One-way ANOVA; ****P < 0.001. **D** PAS staining of iPSCs derived from CTRL and AT_1_ fibroblasts. Scale bar: 20 μm. **E** Graph showing PAS staining integrated intensity for at least 70 iPSC cells derived from CTRL and AT_1_ fibroblasts, pooled from three independent experiments. One-way ANOVA; ****P < 0.001; ns non-significant. **F** Anti-glycogen antibody immunostaining of cerebellar sections from a CTRL individual and two AT patients, highlighting the Purkinje cell layer. Scale bar: 40 μm. Magnification of AT patient cells are shown in the rectangles. The images show typical results. **G** Anti-GYS1 immunostaining of cerebellar sections from a CTRL individual and two AT patients, highlighting the Purkinje cell layer. Scale bar: 40 μm. The images show typical results.

The altered production or turnover of maltotetraose, a byproduct in glycogen metabolism, could indicate an overall increase in glycogen levels. Maltotetraose is indeed a product of the partial hydrolysis of starch or glycogen [28, 29]. Maltotetraose can be further broken down into glucose units by enzymes such as maltase and α-glucosidase, which are involved in carbohydrate digestion [28, 29]. Hence, maltotetraose is a breakdown product, and its increased levels are consistent with the accumulation of glycogen. This accumulation might be due to inefficient utilization of glucose through metabolic pathways such as glycolysis, the PPP, and the TCA. As a result of this inefficiency, the excess glucose is potentially converted into glycogen (Fig. 3A).

To test this hypothesis, we analyzed glycogen levels in live proliferating AT primary fibroblast cells using Periodic-acid Schiff (PAS) staining, a classic method for glycogen detection [30]. Strikingly, even at low passage AT cells exhibited a significant increase in PAS-positive material compared to healthy controls, with a diffuse cytoplasmic distribution and localized aggregations (Fig. 3B). PAS specificity for glycogen was confirmed by the loss of staining after pretreatment with diastase, an enzyme that degrades glycogen [31] (Fig. S4A). Quantification of cell staining intensity corroborated the substantial rise in glycogen in cells from two different primary AT patients’ fibroblast cells (AT_1_ = GM02530 and AT_2_ = GM02487, Coriell biobank) compared to CTRL fibroblasts (CTRL = AG02603, Coriell biobank) (Fig. 3C).

The general defect in glucose metabolism was further verified in induced pluripotent stem cells (iPSCs), which we reprogrammed from CTRL and AT fibroblasts using standard procedures [32]. Noticeably, AT-derived iPSCs also displayed elevated glycogen levels compared to CTRL-derived iPSCs (Fig. 3D, E), indicating that this metabolic disruption is not cell-type-specific but rather linked to the absence of functional ATM.

Finally, glycogen stores were not reduced upon inhibition of PARP with olaparib, ruling out a connection with PARP-mediated protein aggregation reported for cells with non-functional ATM [33] (Fig. S4B, C).

To determine whether glycogen accumulation was directly tied to ATM deficiency and not a secondary effect of cellular senescence or proliferation arrest of the AT primary fibroblasts, we probed two different previously characterized ATM knockout HeLa cancer cell clones [34], which showed a marked diastase-sensitive glycogen staining increase (Fig. S5A–C). This suggests that a functional ATM is crucial for normal metabolism and that its loss precipitates metabolic dysfunction, leading to glycogen accumulation.

To validate the in vitro observations, we monitored glycogen accumulation in tissues from AT patients, including muscle and brain. Cardiac muscle from AT patients, which is prone to premature failure [35], likewise showed extensive glycogen accumulation as shown by the PAS staining of AT_4_ cardiac samples (Fig. S6). Background staining was present in cardiac samples derived from a healthy donor (CTRL = NBB5334 and AT_4_ = NBB836, NIH NBB NeuroBiobank) and in the same samples treated with diastase (Fig. S6). To overcome the limitations of PAS staining in detecting neuronal glycogen, which could be obscured by brain cell membrane glycolipids, we employed a glycogen-specific antibody for immunohistochemical staining [36]. The anti-glycogen antibody also facilitated the recognition of the cell types affected by glycogen accumulation. Using this method, we uncovered significant glycogen deposition in cerebellum samples of different AT patients (AT_3_ = NBB1485 and AT_4_ = NBB836, NIH NBB NeuroBioBank). We could recognize some Purkinje cells with dysmorphic changes and loss of dendritic structure (Fig. 3F). Glycogen staining was completely absent in healthy control cerebellum samples (CTRL = NBB1427, NIH NBB NeuroBioBank). The staining levels of glycogen synthase (GYS1), the enzyme catalyzing glycogen synthesis, were also elevated in two different AT patient brains (AT_5_ = NBB1485 and AT_6_ = NBB1459, NIH NBB NeuroBioBank), suggesting that excess GYS1 and glycogen may contribute to the neuropathology of AT (Fig. 3G). GYS1 staining was instead mostly absent from the bodies of CTRL Purkinje cells (Fig. 3G), as expected [37].

These collective findings illustrate a broad metabolic disruption due to defective glucose handling in AT-derived tissues, potentially underlying the complex pathophysiology of AT from diabetes and heart failure to neurodegeneration [11, 35].

### FNIP2 silencing restores glycogen homeostasis and fitness in AT cells

Previous studies have established that ATM activates the PPP, which consumes glucose to produce ribose and NADPH, counteracting oxidative stress [7, 11]. However, as only a small portion of glucose enters the PPP [7–9], this cannot fully explain the widespread glycogen accumulation observed. Furthermore, although AT cells and tissues show increased glycogen, AT features do not match classical glycogen-storage disorders [38], which lack the core AT hallmarks of progressive cerebellar ataxia, telangiectasia, radiosensitivity, immunodeficiency and cancer risk [3]. This AT clinical phenotype aligns more closely with mitochondrial disease biology, which includes cerebellar neurodegeneration, premature aging traits, redox/respiratory impairment rather than with glycogenosis [39]. Given the widespread malfunction of mitochondrial respiration and the TCA cycle we observed in primary AT cells, also documented by other studies [3], it is likely that glucose metabolism is stalled by the inability to process the end-products of glycolysis through mitochondria [40]. It is therefore likely that glycogen accumulation is a secondary adaptation to a primary defect in mitochondrial respiration/TCA flux and associated energy stress. These considerations prompted us to focus on mitochondrial dysfunction as the proximal driver of AT.

**Fig. 4:**
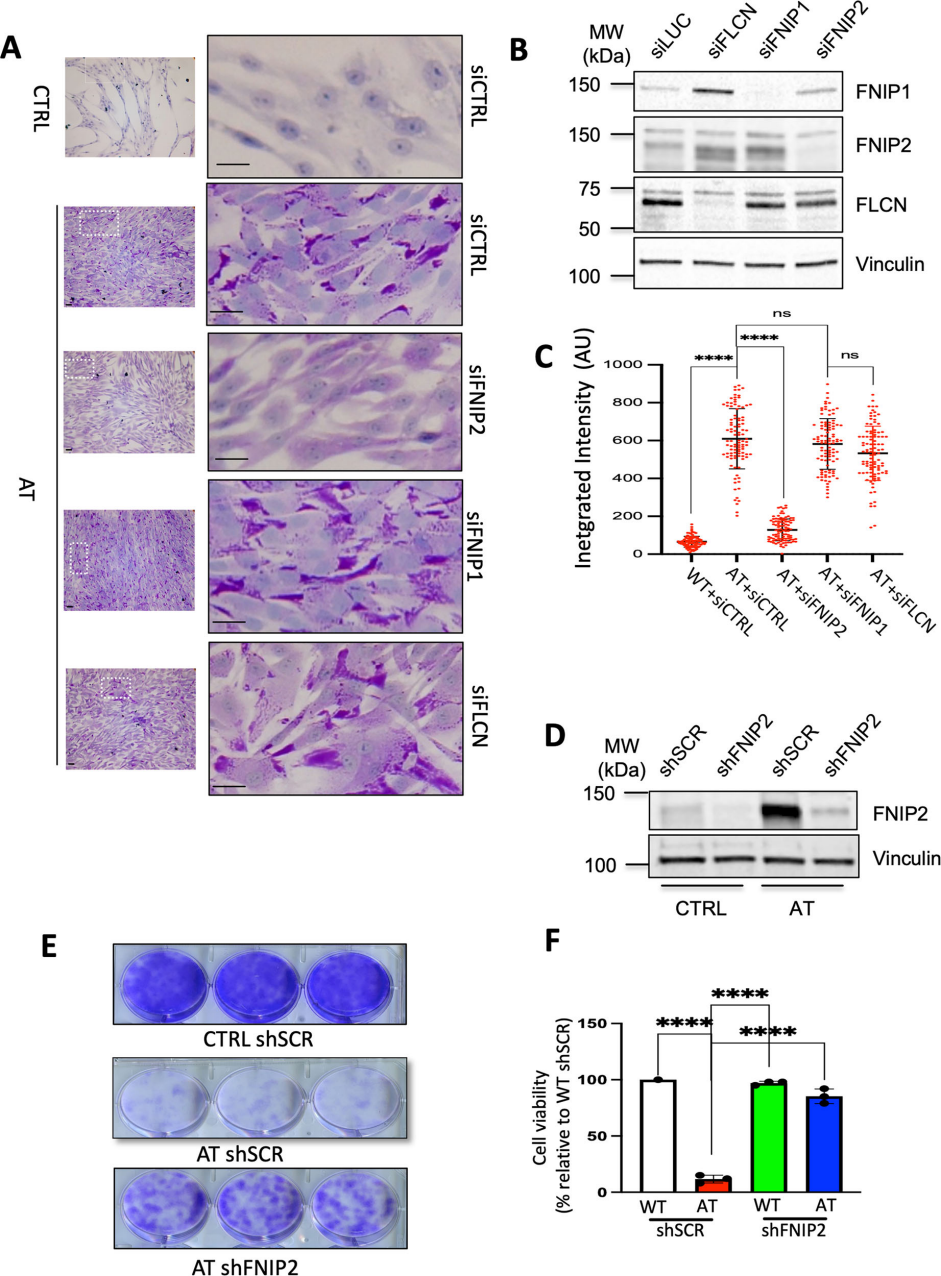
FNIP2 silencing restores glycogen homeostasis and fitness in AT cells. **A** PAS staining of AT cells following treatment with the specified siRNA oligonucleotides. Scale bar: 20 μm. **B** Immunoblot analyses displaying protein expression in CTRL cells post-siRNA-mediated silencing of the indicated target genes. Asterisk (*) indicates non-specific band. **C** Graph showing PAS staining integrated intensity for at least 70 iPSC cells derived from CTRL and AT_1_ fibroblasts, pooled from three independent experiments, and treated as indicated. One-Way ANOVA; ****P < 0.001; ns non-significant. **D** Immunoblots of the indicated proteins from low passage CTRL and AT cells 72 h post infection with shSCR or shFNIP2 encoding lentivirus. **E** Images of crystal violet-stained CTRL and AT fibroblast colonies infected with shSCR or shFNIP2 expressing lentivirus as indicated. **F** Cell survival assessment in shSCR or shFNIP2 expressing cells, normalized to shSCR-infected CTRL cells. Data represent average colony counts after 21 days in culture from three independent experiments. One-Way ANOVA; ****P < 0.001.

Emerging regulators of mitochondrial energy metabolism include FNIP1, FNIP2, and FLCN proteins, which respond to redox and nutrient level changes, limiting glycolysis and mitochondrial respiration under starvation and redox imbalance [14, 41–43]. As these conditions are mimicked by the metabolic and oxidative status induced by the absence of ATM, we tested whether downregulation of FNIP1, FNIP2, and FLCN proteins could have any impact on AT cells’ metabolic alterations. To this end, we monitored glycogen accumulation as a marker of abnormal metabolism following FNIP1, FNIP2, and FLCN protein expression by separately targeting each of these factors with specific siRNAs (Fig. 4A, B and Supplementary material).

Significantly, we found that FNIP2 downregulation alone was sufficient to suppress glycogen accumulation in AT primary fibroblasts, reducing the PAS signal to the background levels observed in CTRL fibroblast cells (Fig. 4A, C). On the contrary, downregulation of FLCN or FNIP1 protein expression (Fig. 4B) did not impact glycogen levels (Fig. 4A, C).

To verify the long-term effects of metabolic rescue obtained by FNIP2 downregulation, we used lentiviruses encoding for short hairpin against FNIP2 (shFNIP2) or an unrelated scrambled control sequence (shSCR). Compared to shSCR-infected cells shFNIP2 lentivirus effectively and specifically decreased FNIP2 protein expression levels (Fig. 4D and Supplementary material), without affecting FNIP1 and FLCN expression (Fig. S7A, B and Supplementary material). Intriguingly, FNIP2 levels were consistently upregulated in AT cells (Figs. 4D and S7B). This observation warrants further experiments to determine whether FNIP2 is normally suppressed by ATM, or whether its upregulation represents an adaptive response to the redox and metabolic defects of AT cells.

We then investigated the effects of FNIP2 downregulation on AT cell survival. At early passages, CTRL primary fibroblasts plated at low number proliferate and form colonies in vitro. However, with continued passaging, colony-forming ability progressively declines and cultures ultimately reach replicative senescence. In AT fibroblast this decline typically begins at passages 12–18, whereas in normal CTRL fibroblasts it occurs later, around passages 20–30. During this phase, AT cells enter premature senescence [1, 12] adopting a flattened morphology with increased cytoplasmic granularity. They then become fully senescent and eventually die (Fig. S8A–C). The progressive decrease in fitness likely reflects cumulative damage and stress accumulated over time in AT cells.

Strikingly, using the colony-formation assay in which cells were plated at low density and cultured for three weeks without passaging we found that stable downregulation of FNIP2 restored normal cell fitness and survival of AT primary fibroblasts as documented by colony-formation assessment (Fig. 4E, F), preventing their premature senescence (Figure S8A–C).

### FNIP2 inhibition improves glycolysis and mitochondrial respiration in AT Cells

To better understand the underlying metabolic effects of FNIP2 downregulation, we explored glucose and oxygen consumption in both CTRL and AT cells in control conditions and following FNIP2 inactivation.

**Fig. 5:**
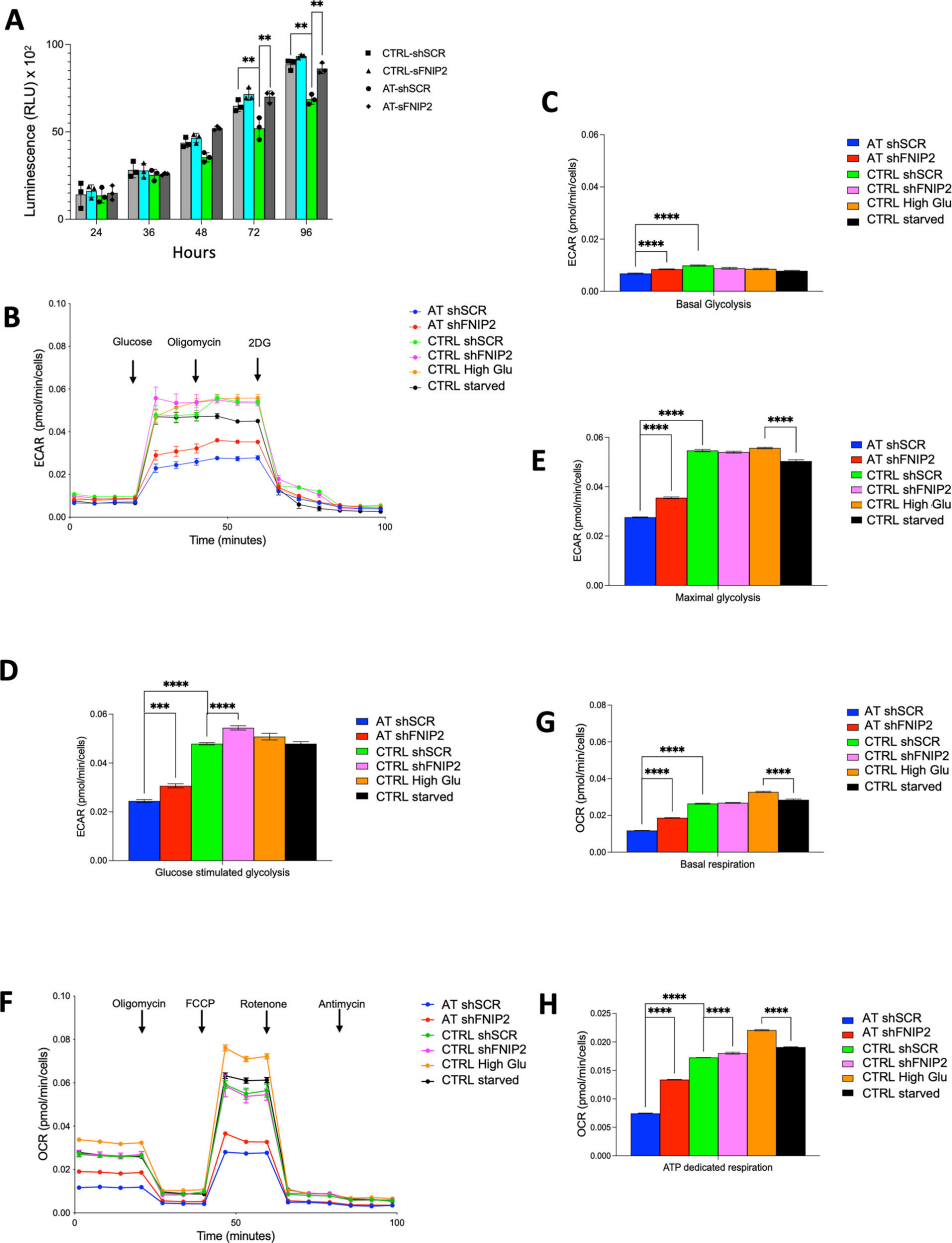
FNIP2 inhibition improves glycolysis and mitochondrial respiration in AT cells. **A** Graph showing cell proliferation of CTRL and AT fibroblasts stably infected with shSCR or shFNIP2 lentivirus after plating for the indicated times and quantified by the cell viability assay CellTiter-Glo. Normalized results from three independent experiments are shown in relative luminescence units (RLU). Error bars show SD. Multiple t-tests were used to calculate significance between the indicated couples for each time point. **P < 0.001. The remaining comparisons show no significant difference. **B** Extracellular acidification rates (ECAR) for shSCR and shFNIP2 encoding CTRL and AT cells 24 h after plating. Analysis included CTRL cells exposed to high glucose and nutrient-starved CTRL cells. **C** Quantification of basal glycolysis in the indicated samples. **D** Assessment of glycolysis upon glucose stimulation in the same set of samples. **E** Evaluation of peak glycolysis capacity in the samples. **F** Oxygen consumption rates (OCR) in the cells detailed in (**B**). **G** Measurement of basal respiration in the indicated samples. **H** Determination of ATP production-linked respiration in the indicated samples. One-way ANOVA was used for statistical calculations for all graphs. For each fibroblast line, two independent Seahorse runs were performed on different days. Within each run, 7 wells per line were measured (technical replicates). Plots display all wells; overlaid symbols show plate-adjusted means with 95% CIs from a model that includes ‘run/plate’ as a factor. For each run/plate groups were compared using one-way ANOVA on normalized per-well values, reporting per-plate *P* values and effect sizes P values (****P < 0.0001; ***P < 0.001) report plate-adjusted comparisons with multiplicity control (Tukey). Exact n: 2 runs per line; 7 technical wells per run.

To avoid confounding effects of cell fitness impairment over long-term culture we used low passage CTRL and AT cells stably infected with shSCR or shFNIP2 after assessing their proliferation and viability using a luciferase-based assay at different times after plating (Fig. 5A). We then used the Seahorse platform to analyze bionergetic parameters in fibroblasts 24 h after plating, a time point at which no proliferation and viability differences were observed between CTRL and AT cells (Fig. 5A), and normalized all the counts to the number of live cells. We took baseline measurements of extracellular acidification rates (ECAR) to understand the initial glycolytic state of the cells (Fig. 5B).

We then sequentially injected glucose to fuel glycolysis, oligomycin to inhibit mitochondrial ATP production, and therefore force cells to rely more on glycolysis, and 2-deoxyglucose (2-DG) to inhibit glycolysis and measure how cells responded [44–46] (Fig. 5B). As a control sample for the technique, we used uninfected CTRL cells that were either starved or exposed to high glucose levels (Fig. 5B). In shSCR-treated AT cells, overall glycolytic activity, comprising basal, maximal, and glucose-stimulated glycolysis, was markedly lower than in shSCR-treated CTRL cells (Fig. 5B–E). However, FNIP2 suppression in shFNIP2-treated AT cells led to a significant enhancement in glycolysis, evidenced by increased glucose metabolism under both basal, maximal, and stimulated conditions (Fig. 5B–E). Instead, FNIP2 downregulation did not affect glycolysis in shFNIP2-treated CTRL cells, except under glucose hyper-loading (Figs. 5B–E and 5E).

We then measured oxygen consumption by mitochondrial respiration. Remarkably, we found that FNIP2 knockdown in AT cells boosted basal respiration and ATP production, indicating a parallel upregulation of mitochondrial function (Fig. 5F-H) [44]. This improvement is likely attributable to the alleviation of FNIP2-mediated repression on mitochondrial activity, a regulatory function akin to the role of FNIP1 [47, 48], which has been previously proposed to act as a brake on mitochondrial respiration. Even if these improvements did not restore the respiration to the same levels as the control, they were able to restore sufficient usage of glucose, preventing glycogen accumulation in AT primary cells (Fig. 4A–C), promoting their viability (Fig. 4E, F), and avoiding senescence (Fig. S8A–C).

Our results also suggest that glucose metabolism impairment and glycogen accumulation are unlikely to result from the activity of the complex formed by FNIP1, FNIP2, and FLCN. This conclusion is supported by the observation that the disruption of this complex due to the inactivation of either FLCN or FNIP1 did not reverse glucose metabolism and glycogen accumulation (Fig. 4A-C).

### FNIP2 interacts with SERCA2b stimulating endoplasmic reticulum (ER) reuptake of Ca^2++^ and mitochondria-ER contacts (MERCs) formation in AT cells

To identify the possible mechanisms of FNIP2 action on mitochondrial respiration, we looked for FNIP2 functions that are independent of the FNIP1-FNIP2-FLCN complex. FNIP1 has been reported to have a FLCN-independent function by binding and stimulating SERCA2b channels, which pump Ca^2++^ into the endoplasmic reticulum (ER) [49]. Loss of FNIP1 limits SERCA2b-mediated Ca^2++^ reuptake in the ER, promoting increased levels of cytoplasmic Ca^2++^ that stimulate mitochondrial respiration [49, 50].

Human FNIP1 and FNIP2 are paralogous co-chaperones that share ~49% overall sequence identity and ~74% similarity, including conserved N-, middle, and C-terminal FNIP domains. Within the C-terminal halves, however, sequence conservation is low except for the last 158 residues, suggesting that divergence in this region contributes to isoform-specific functions [42].

**Fig. 6:**
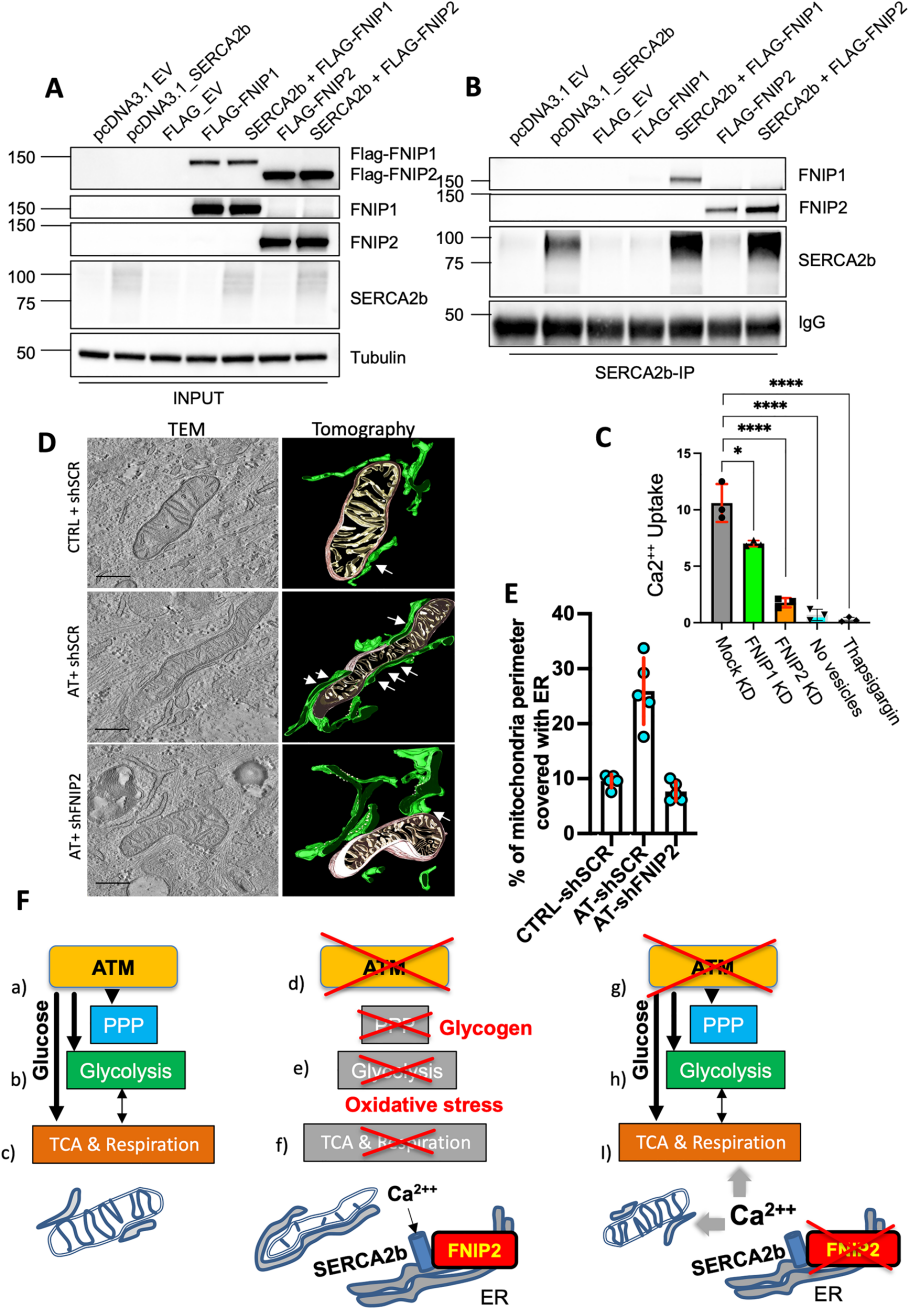
FNIP2 interacts with SERCA2b stimulating ER reuptake of Ca^2++^ and MERC formation in AT cells. **A** Representative immunoblot of the indicated proteins from HEK293 cells transfected with pcDNA3.1-empty vector, pcDNA3.1-SERCA2b, FLAG-empty vector, FLAG-FNIP1 or FLAG-FNIP2, alone or in combination with pcDNA3.1-SERCA2b as indicated. Tubulin was used as a loading control. These blots correspond to the input lysates for the SERCA2b immunoprecipitations shown in (**B**). The experiments were repeated at least three times. **B** Representative immunoblot showing SERCA2b immunoprecipitations from HEK293 cells transfected with pcDNA3.1-empty vector, pcDNA3.1-SERCA2b, FLAG-empty vector, FLAG-FNIP1 or FLAG-FNIP2, alone or in combination with pcDNA3.1-SERCA2b. SERCA2b immunoprecipitates were analyzed by immunoblotting using antibodies against the indicated factors. The IgG heavy chain signal is shown as a control for loading and immunoprecipitation efficiency. All signals were obtained from the same membrane, which was sequentially stripped and re-probed. The image represents a typical results of an experiment repeated at least three times. **C** In vitro ER Ca²⁺ uptake (µM) calculated from the linear range of Fura-2 fluorescence traces recorded in the presence of ER vesicles enriched from mock-, FNIP1-, or FNIP2-knockdown (KD) HEK293 cells, from samples without vesicles, or from HEK293 cells treated with 1 µM thapsigargin, as indicated. **D** Transmission electron microscopy (TEM) and electron tomography reconstruction (ET) of CTRL and AT primary fibroblast cells following the described treatments. Scale bar: 0.5 μm. **E** Quantification of mitochondrial perimeter covered by ER in CTRL or AT cells infected with the indicated lentivirus, expressed as percentage, calculated in five independent experiments. Horizontal bars represented the mean ± SD shown by the vertical red bar from five independent preparations marked by blue circles. **F** Working model: ATM activation (a) leads to efficient glucose utilization (b), enhancing PPP, glycolysis, and mitochondrial function (c), crucial for cell survival. Without functional ATM (d), impaired glucose handling results in glycogen build-up and excessive oxidative stress (e), triggering a FNIP2-mediated metabolic checkpoint that suppresses glycolysis and mitochondrial function, restraining cell growth and proliferation, ultimately compromising cell survival (f).

We tested whether FNIP2 is also able to bind SERCA2b and regulate Ca^2++^ reuptake and mitochondrial respiration. To this end, we expressed equal amounts of Flag-FNIP1 or Flag-FNIP2 and untagged SERCA2b in HEK293 cells (Fig. 6A and Supplementary material). As previously shown [49], immunoprecipitation of SERCA2b also precipitated Flag-FNIP1 when constructs expressing both proteins were co-transfected (Fig. 6B and Supplementary material). Importantly, similar to FNIP1, immunoprecipitation of SERCA2b also precipitated Flag-FNIP2 (Fig. 6B). However, in contrast to Flag-FNIP1, Flag-FNIP2 was precipitated by the sole pulldown of low levels endogenous SERCA2b in the absence of exogenous SERCA2b overexpression (Fig. 6B). These results indicate that low levels of endogenous SERCA2b were sufficient to bind Flag-FNIP2 but not Flag-FNIP1, suggesting that SERCA2b has a much higher affinity for FNIP2 compared to FNIP1. The interactions were specific as no FNIP1 and FNIP2 were detected in SERCA2b immunoprecipitations from cells transfected with empty, SERCA2b or FLAG-tag-only expressing vectors (Fig. 6A, B). These results pointed to a differential functional impact between FNIP1 and FNIP2 towards SERCA2b regulation.

Accordingly, downregulation of FNIP2 in HEK293 cells strongly abolished in vitro Ca^2++^ uptake by ultracentrifuge-enriched endoplasmic reticulum (ER) vesicles, as shown by Fura-2 fluorescence traces (Fig. S9) and relative Ca^2++^ levels quantification (Fig. 6C). Instead, downregulation of FNIP1, as previously shown, only modestly affected Ca^2++^ reuptake (Figs. 6C and S9). No Ca^2++^ uptake was observed with unenriched cell extracts. Importantly, the selective inhibition of Ca^2++^ uptake by SERCA Ca^2++^ pumps inhibitor thapsigargin [51] confirmed specific active Ca^2++^ uptake (Figs. 6C and S9). The strong decrease in Ca^2++^ uptake from the cytoplasm following FNIP2 inactivation might leave more Ca^2++^ available for the stimulation mitochondrial activities, including respiration and TCA cycle enzymes [52]. This stimulation, in turn, improves glucose metabolism and cellular homeostasis (Fig. 5B–H).

To validate mitochondrial activation mechanisms induced by FNIP2 suppression, we performed transmission electron microscopy (TEM) analysis of mitochondrial morphology in primary CTRL and AT fibroblasts (Fig. 6D). Contrary to primary CTRL fibroblast cells, and as previously shown [20, 40], AT fibroblasts displayed mitochondria with variable shape represented by an abnormal elongated morphology with less defined cristae (Figs. 6D and S10A), and a slightly increased mitochondrial mass (Fig. S3B) possibly due a primary defect or a metabolic adaptation. Strikingly, FNIP2 suppression restored the normal appearance of mitochondria in AT cells (Figs. 6D and S10A).

We then applied electron tomography (ET), which uses a series of tilted electron microscope 2D images to reconstruct a 3D model of the mitochondria and their surrounding structures [53]. The high resolution of ET allows for the detailed visualization of organelles, including the mitochondria and the ER [54]. Using this approach, we were able to identify bona-fide mitochondria-ER contacts (MERCs) in CTRL cells (Figs. 6D, E and S10B), which are important for maintaining mitochondrial function through lipid and protein exchanges [55].

CTRL cells displayed normal MERCs with a contact gap ranging from 25 ± 5 nm and a surface interaction area covering ~10% for each mitochondria, on average (Figs. 6D, E and S10B). However, in AT cells, mitochondria showed pervasive increased contacts with ER, covering more than 25% of the available perimeter for each mitochondria and a reduced average gap distance of 10 ± 5 nm (Figs. 6D, E and S10B). These findings could be indicative of an unbuffered oxidative stress response linked to the chronic absence of ATM in primary non-immortalized cells used here [55]. Accordingly, oxidative stress, which we showed to be highly present in AT fibroblasts, is a potent inducer of MERCs [56].

Remarkably, FNIP2 inactivation not only normalized mitochondrial morphology but also reduced the heightened MERCs back to less than 10% of the average individual mitochondrial perimeter in AT cells, restoring 25 ± 5 nm wide contact gaps, indicative of improved mitochondrial function (Figs. 6D, E and S10B) [54, 55].

In summary, our results position FNIP2-SERCA2b axis as a pivotal target to restore normal metabolism in the absence of ATM.

## Discussion

Ataxia Telangiectasia presents with a multifaceted spectrum of symptoms, including cerebellar degeneration, ocular telangiectasia, immunodeficiency, premature aging, and diabetes [11]. These diverse manifestations extend beyond what could be attributed solely to deficiencies in DNA repair, pointing instead towards systemic metabolic dysfunction.

The ATM kinase, a member of the PI3 kinase family, is known to influence various metabolic processes [57]. While ATM dysfunction is associated with metabolic syndrome features such as insulin resistance, dyslipidemia, hypertension, and atherosclerosis, its exact role in cellular metabolism has remained elusive [3, 6]. Our study brings to light ATM’s critical involvement in regulating glucose metabolism. Specifically, we have demonstrated that: (a) AT primary cells exhibit significant deficits in basal and induced glycolysis and mitochondrial respiration; (b) Persistent glucose metabolism impairment leads to the breakdown of alternative carbon sources, like amino acids in AT cells and results in excessive glycogen accumulation, and c) FNIP2 suppression improves glucose processing and mitochondrial function, preventing glycogen accumulation and restoring critical mitochondrial-ER contacts, thereby rescuing the growth and survival of primary AT cells. These insights underscore ATM’s pivotal role in modulating glucose metabolism in response to oxidative stress (Fig. 6F). These findings also indicate that growth arrest triggered by the absence of ATM is not irreversible, as it can be rescued by restoration of normal metabolism.

Our studies validated primary non-immortalized untransformed fibroblasts for metabolic and mitochondrial assessment, as the same alterations were found in several other cell types and patient-derived tissue samples, confirming the value of primary fibroblasts [58] in assessing functional defects in diseases caused by malfunction in housekeeping genes controlling cell metabolism. The inability to efficiently process glucose mirrors metabolic impairments seen across cellular, tissue, and organismal levels in AT, resembling a state of metabolic starvation caused by inefficient usage of glucose, which leads to the accumulation of glycogen. In this context, our tracing experiment data showed that glucose-derived carbon incorporation in maltotetraose, fructose 1,6-bisphosphate, and lactate is decreased in AT cells. Our data clearly indicate that significant effects on glycolysis and the TCA cycle were impaired in AT cells only after 24 h of exposure to labeled glucose. On this basis, the defect in glycogen utilization constitutes a metabolic hallmark in AT cells, and indeed, the cells prefer to use other fuel sources such as amino acids.

A key disruptor of long-term AT cell survival might be the unmitigated oxidative stress linked to mitochondrial defects present in AT cells. Consistent with this interpretation, our results indicate that the metabolic defect of AT cells lies mostly in the impaired mitochondria, as moderate improvement of mitochondrial activity restores AT cell fitness. This effect is obtained through the attenuation of FNIP2-mediated stimulation of SERCA2b-dependent Ca^2++^ reuptake in the ER, which normally decreases the availability of cytoplasmic Ca^2++^ required for mitochondrial stimulation [52]. Deactivating FNIP2 can indeed reboot mitochondrial activity likely by increasing cytoplasmic Ca^2++^ levels, reactivating glucose metabolism, and thus preventing cell growth impairment. In this context FNIP2 might act as a brake on mitochondria activity. Given the increased levels of endogenous FNIP2 protein levels in AT cells this pathway may be activated by chronic oxidative stress arising in the absence of ATM to limit the activity of impaired mitochondria and avoid further ROS accumulation and cellular damage. FNIP2-mediated downregulation of mitochondrial activity could provide a protective brake in AT primary cells, transiently arresting proliferation and inducing a reversible, senescent-like state to prevent additional injury. These effects are specific to FNIP2 and are not reproduced by FNIP1 modulation, consistent with their differential regulation in AT cells and with the distinct potency of these co-chaperones in modulating SERCA2b-mediated ER Ca^2++^ uptake.

While our data are consistent with an association between impaired bioenergetics linked to defective mitochondria and active modulation of AT cell status, they do not establish causality. Additional perturbation and rescue experiments will be required to determine the precise mechanisms underlying these observations.

The therapeutic implications of FNIP2-SERCA2b axis manipulation for non-dividing cells might be useful as its modulation might overcome metabolic shortcomings, especially those resulting from ATM deficiency. In neurons, FNIP2 inhibition could potentially enhance mitochondrial function and glucose metabolism, offering a strategic avenue to improve neuronal function in AT patients. Therefore, our findings provide compelling evidence for ATM’s central role in cellular metabolism and propose the FNIP2-SERCA2b axis as a target for therapeutic intervention to alleviate the metabolic defects in AT.

## Materials and methods

### Metabolite analysis

Metabolite extraction and Ultrahigh Performance Liquid Chromatography-Tandem Mass Spectroscopy analysis were performed by Metabolon (Durham, North Carolina) as previously described [59]. Briefly, raw data were extracted, peak-identified, and quality control processed by Metabolon using proprietary software. Compounds were identified by comparison to library entries of 2400 purified standards or recurrent unknown entities. Data analysis was performed in R (version 4.0.2) running in an RStudio environment (version 1.0.153). Missing metabolite intensities were imputed with the lowest detectable value, and intensities were median-centered. Significance of metabolite alterations was calculated with the maanova R package by pairwise comparison of indicated groups (version 1.58.0), and an FDR threshold of 0.1 was used to identify altered metabolites. Scatter plots and heat maps were generated with the R packages ggplot2 (version 3.3.3) and pheatmap (version 1.0.12), respectively. Principal component analysis calculation and visualization were done with the packages FactoMineR (version 2.4) and factoextra (version 1.0.7) with convex ellipse type. The sum of detectable oligosaccharides was calculated from maltose, maltotriose, and maltotetraose.

### [U-^13^C_6_]-glucose **tracing** time-course analysis

CTRL and AT fibroblast cells at low passage were exposed to 2.5 mM [U-^13^C_6_]-glucose (389374, Sigma-Aldrich) for 2, 4, and 24 h or 24-36 h after plating. Cells were then smashed in a tissue lyser for 1 min at maximum speed in 250 μl of ice-cold methanol/water 50:50. Lysates were spun at 15,000 × *g* for 15 min at 4 °C, and supernatants were then passed through a regenerated cellulose filter (4 mm Ø, Sartorius). Samples were then dried under N2 flow at 40 °C and resuspended in 100 μl of ice-cold methanol for subsequent analyses.

Data were obtained by liquid chromatography coupled to tandem mass spectrometry. We used an API-3500 triple quadrupole mass spectrometer (AB Sciex, Framingham, MA, USA) coupled with an ExionLC^TM^ AC System (AB Sciex). Quantification of isotopomers was performed by using a cyano-phase LUNA column (50 mm × 4.6 mm, 5 μm; Phenomenex) with a 5-min run in negative ion mode. Mobile phase A was water, and phase B was 2 mM ammonium acetate in MeOH. Runs were performed in isocratic mode with 80% B with a flow rate of 500 μl/min. Data are expressed as mass isotopomer distribution (MID). The MID refers to the relative abundance of different isotopomers (molecules that differ only in the isotopic composition of their atoms) in a sample. Isotopomers can differ by the number of heavier isotopes (e.g., ^13^C instead of ^12^C) they contain. M0, M2, M3, M4, M5, M6, M7, and M24 indicate the different masses of the isotopomers detected, and the number after M indicates the number of carbons derived from U-^13^C^6^-glucose administered to CTRL and AT cells (M0 represents no labeled carbons). All analyzed isotopomers are summed, including M0, and then the percentages of each isotopomer are determined on a scale from 0 to 1, where 1 represents 100% labeling.

### Cell culture and tissue samples

The following cell lines were obtained from the NIGMS Human Genetic Cell Repository at the Coriell Institute for Medical Research: GM03492, GM03400, GM03396, AG03059, AG03057, AG02603, GM00024, AG02602, GM03487, GM03395, AG03058, GM05823, GM01588, GM00647, GM02530, and GM00367. Genetic background for each cell is reported in Tables 3 and 4 and at www.Coriell.org. Human tissue samples were obtained from the NIH NeuroBioBank (NBB). The following frozen and fixed tissue samples provided by NeuroBioBank were used: NBB 5334, 836, 4727, 1485 and 1459. HeLa and HEK293 cells were obtained from the IFOM cell culture facility. HeLa cells were maintained in MEM, supplemented with 10% Fetal bovine serum (FBS) in a humid incubator at 5% CO_2_ and 20% O_2_. HEK293 cells were cultured in DMEM supplemented with 10% FBS, 1% penicillin/streptomycin, and 5% L-Glutamine (L-Glu). hiPSC were reprogrammed from AG02603 (CTRL) and GM02530 (AT) primary fibroblasts using the Cytotune^TM^-iPS Sendai reprogramming kit (A16517, Invitrogen) and cultured on Matrigel (354277, SACCO) coated plates in mTESR^TM1^ (85850, Stem Cell Technology) supplemented with ROCK inhibitor, Y27632 (A11001, ADOOQ) in the first 24 h of culture. Assays were performed at controlled passage numbers and confluence to minimize senescence-related artefacts.

### Transfection and siRNA-mediated knockdown

To knock down our genes of interest, primary fibroblasts were transfected with endoribonuclease-prepared small interfering RNA (esiRNA), targeting the mRNA of a specific gene. The transfection reagent Lipofectamine RNAiMax (13778100, Invitrogen) was used, taking advantage of its low toxicity and high efficiency in the delivery of esiRNA into the cells. Once inside the cell, esiRNA induces RNA interference, leading to the degradation of mRNA and the knockdown of its resulting protein. Lipofectamine RNAiMax was diluted 1:20 in Opti-MEM Reduced Serum Medium (51985-042, Life Technologies) and incubated at RT for 5 min. Simultaneously, esiRNA was diluted in Opti-MEM to reach a concentration of 60 nM to 150 nM, depending on the previously optimized concentration. Lipofectamine and esiRNA were mixed 1:1 and incubated for 15 min at RT to allow complex formation. Finally, the lipofectamine/esiRNA mixture was added dropwise to the seeded cells at 60-80% confluency, and cells were incubated for 48 to 72 h before sample collection to be assessed by qPCR, western blot (WB), or PAS staining. In all the experiments with esiRNA, mission esiRNA targeting luciferase (EHURLUC, Sigma-Aldrich) was used as a negative control. The following esiRNA were used: mission esiRNA targeting human FNIP2 (EHU054551, Sigma-Aldrich), mission esiRNA targeting human FLCN (EHU006461, Sigma-Aldrich), and ON-TARGETplus Human FNIP1 (96459) siRNA- SMARTpool (L-032573-02-0020, Horizon Discovery).

### Immunoprecipitation assay and immunoblots

For immunoprecipitation, one day before transfection, HEK293 cells were seeded to reach ~70% confluence. Cells were transiently transfected with 10 µg of FLAG-FNIP1 or FLAG-FNIP2 together with a SERCA2b expression construct or empty vector, using Lipofectamine 2000 in Opti-MEM Reduced Serum Medium according to the manufacturer’s instructions. Medium was refreshed 6–8 h post-transfection to limit cytotoxicity. The following constructs were obtained from Addgene: FLAG-FNIP2 (Plasmid #72294), FLAG-FNIP2 (Plasmid #72292) and SERCA2b (Plasmid #75188). HEK293 cells were harvested 48 h after transfection and lysed using a syringe with IP lysis buffer (50 mM Tris, pH 7.5, 150 mM NaCl, 2 mM EDTA, 1.5% NP-40), supplemented with Protease Inhibitor Cocktail Set III (Cat. No. 539134, Calbiochem) and 1 mM PMSF, incubated for 30 min on ice, and centrifuged at max speed for 30 min at 4 °C. SERCA2b immunoprecipitation was performed by incubating 1 mg of total protein extract with 1 µg of SERCA2b ab (MA3-919) ON at 4 °C on a wheel within 1 ml of complete IP lysis buffer. Fifty microliters Dynabeads® Protein G were washed once with a washing buffer supplemented with a protease inhibitor cocktail and incubated for 2 h with the extract-Ab complex at 4 °C on a wheel. Samples were eluted at RT for 30 min with 50 μl of 0.1 M Glycine pH 3.0 and immediately neutralised with 10 μl of 0.5 M Tris and 1.5 M NaCl pH 8.0. Thirty micrograms of proteins were loaded as input.

For immunoblots, 20–30 μg proteins were loaded on 4–15% Bis-Tris Poly Acrylamide gels (Bio-Rad), and analyzed by standard techniques. Briefly, proteins were transferred from the gel to a 0.2 μm nitrocellulose membrane (Bio-Rad) using the Trans-Blot Turbo Transfer system (Bio-Rad). The filters were blocked for 1 h with 5% milk in TBST buffer (20 mM Tris, 150 mM NaCl, 0.1% Tween 20). After blocking, the corresponding primary antibody was added and membranes were maintained at 4 °C ON, washed 3 times with TBST and incubated for at least 1 h with a suitable secondary antibody conjugated to HRP (Horse-radish peroxidase). Signals were detected using by ECL detection reagent (GE-Healthcare) on Amersham Hyperfilm (GE-Healthcare) or on Bio-Rad ChemiDoc™ XRS + . The following antibodies were used for immunoblots at the concentrations suggested by the manufacturer: ATM (SC-23921, Santa Cruz); Vinculin (PA5-29688, Invitrogen); FNIP2 (HPA042779, Atlas antibody); FNIP1 (Ab134969, Abcam); FLCN (3697 s, Cell Signaling); SERCA2b antibody (clone 2A7-A1; #MA3-919, ThermoFisher Scientific), GAPDH (G8795, Sigma-Aldrich), Tubulin (clone B-5-1-2, #T5168, Sigma-Aldrich), Flag-HRP (A8592, Sigma-Aldrich).

### PAS staining and microscopy

For Periodic acid-Schiff (PAS) staining, low passage (12-18) CTRL and AT fibroblasts and HeLa cells were grown on glass in a 24-well plate or glass chamber. Samples were fixed with ethanol and 37% paraformaldehyde at a ratio of 9:1, respectively, for 10 min and stained using the Periodic acid-Schiff kit (395B, MERCK) according to the manufacturer’s instructions. Briefly, fixed cells were treated with periodic acid solution for 5 min, washed with water, and stained with Schiff reagent for 10 min, then counterstained with hematoxylin provided with the kit for 90 s. The cells were dehydrated with increasing concentration of ethanol (70%, 85% and 100% respectively), air-dried, and mounted with Eukitt medium. For cryosections, as in the case of tissues, samples were fixed with ethanol and chloroform at a ratio of 7:3, respectively, before being stained following the same protocol. To eliminate the possibility of detecting other polysaccharides than glycogen, a negative control treated with 0.5% α-Amylase from porcine pancreas (Diastase, DIE) (A3176, Sigma-Aldrich) for 5 min was used for each condition before staining. The slides were analyzed by widefield microscopy using an Olympus Upright BX51 Full Manual microscope. PAS staining intensity was quantified with CellProfiler image analysis software. Images were imported into Cell Profiler, and a pipeline was created for analysis, which included modules for image adjustment, object identification, and measurement. Specifically, the Color to Gray module was utilized for grayscale conversion, Enhance or Suppress Features for enhancing the PAS staining, and Identify Primary Objects for detecting and masking individual fibroblasts. The Measure Object Intensity module quantified the staining intensity within each cell. Thresholding and settings were iteratively adjusted to accurately capture the magenta color of the PAS staining, representing the glycogen content. The accuracy of the masks and intensity measurements was verified through a systematic review of representative images, and refinements were made accordingly. Final data were exported for statistical analysis, ensuring a robust quantification of PAS staining in fibroblasts. The inhibitor used in the PAS assay was PARPi (Olaparib, SML3705, Sigma-Aldrich)

### Generation of stable FNIP2 knockdown with shRNA vector

Four different constructs of FNIP2-human 29mer shRNA cloned in lentiviral GFP vector (pGFP-C-shLenti, third generation) were obtained from Origene: TL317378A, TL317378B, TL317378C, TL317378D, and one vector containing the scrambled sequence TR30021, for negative control. The dried plasmids were reconstituted in autoclaved MilliQ ddH_2_O, diluted, and added to thawed vials of the transformation competent Top10 *Escherichia coli* strains for transformation, followed by 30 min incubation on ice. The heat shock was performed at 42 °C for 45 s, and the vials were incubated for 2 min on ice. Antibiotic-free LB medium was added to the vials, which were then placed in a shaking incubator at 37 °C, 225 rpm for 1 h. Part of the samples were then spread on 34 μg/ml chloramphenicol plates and incubated at 37 °C ON for colony formation. The plasmids were then expanded and isolated using the NucleoBond Xtra Maxi kit (74041410, Macherey-Nagel).

The viruses were produced in HEK293T cells according to the lentiviral packaging kit instructions (TR30037, Origene). 293T cells were grown in antibiotic-free medium and transfected with a mixture of packaging plasmid (TR30037), one of the shRNA FNIP2 encoding plasmid (TL317378C), or the negative control (TR30021), and Turbofectin in Opti-Mem medium. The mixture was incubated for 15 min at RT and added dropwise to the 293 T plates and incubated ON. The next day medium was changed, and the cells were re-incubated. For the next two days, the medium was collected, filtered through a 0.45 μm filter to and stored at 4 °C. Polyethylene-glycol (PEG) solution was added for precipitation of the virus. The virus was further resuspended in the corresponding fibroblast medium and added to the attached fibroblasts in the presence of polybrene. Three days post-infection, cells were selected with 3 μg/mL of puromycin, and cells were further passaged and collected for knockdown assessment by immunoblots performed as described above.

29-mers shRNA sequences:

TL317378A: TCGGAGTTTGACCTGAATGAGATTCGCCT

TL317378B: CCAAGAAGGTCAACTGATGAGACATTCAG

TL317378C: GCTGTTGAAAGTGGAGATGCCTACAAGAC

TL317378D: AACGACCTGCCTCTGTTGACTGCTATTGC

### Cell proliferation, viability and colony formation assays

For short-term proliferation and viability testing CellTiter-Glo luminescent cell viability assay (Promega) was used to assess proliferation and viability up to 96 h. Briefly, cells were seeded in white 96-well plates at a density of 3–8 × 10³ cells per well in 100 µl of complete growth medium and allowed to adhere overnight at 37 °C in a humidified incubator with 5% CO₂. The following day, medium was replaced with 100 µl of fresh medium. Cells were incubated for the desired treatment period (typically 24–96 h). For background controls, wells containing medium only (no cells). For each time point cells were incubated with the CellTiter-Glo reagent using a multichannel pipette. The plate was placed on an orbital shaker and mixed for 2 min to induce cell lysis. Following mixing, plates were incubated at room temperature for an additional 10 min to stabilize the luminescent signal, protected from strong ambient light. Luminescence was recorded on a Tecan microplate reader equipped for chemiluminescence detection, using an integration time of 0.5–1 s per well and no emission filter. Background signal from wells containing medium plus CellTiter-Glo reagent but no cells was subtracted from all readings. were included on each plate using a Tecan microplater reader to record the produced luminescence. For each condition, luminescence values were averaged across technical replicates (typically 6 wells) and experiments were repeated 3 times.

For long-term survival colony formation assays cells experiments were done with F-10 medium (G&co) containing 20% serum. The cells were seeded at low number (100–600) in 5 cm diameter petri dishes (Nunc, Roskilde, Denmark) and were cultured at 37 °C for 21 days with two medium changes after 7 and 14 days. On the day of staining, medium was removed, cells were washed twice with PBS and fixed with 50% and 100% ethanol solutions for 15 min at RT. Cells were rinsed once with PBS and stained with Crystal Violet solution (0.2% CV in 20% methanol) for 20 min. Excess dye was discarded by washing with water and cells were dried with a filter paper. Colonies composed of 50 cells or more were recorded under a dissecting microscope, using four to eight plates for each point in every experiment. Viability was scored as colony-forming efficiency (CFE), defined as the ratio between the number of colonies recorded and the number of cells inoculated expressed as percent. To confirm colony counting the cell-bound dye was dissolved in 10% glacial acetic acid for 30 min while shaking gently on a rocking shaker, and the absorbance of the solubilized CV was assessed using spectrophotometry. The cells were maintained in MEM minimum essential medium (M2279, Sigma-Aldrich) supplemented with 20% Fetal bovine serum (FBS) in a humid incubator at 5% CO_2_ and 3% O_2_. For these experiments following cell lines were used for the experiments: GM02530 (AT) and AG02603 (CTRL) at passage from 12 to 18 and the same cells 36 h post lentiviral infection with scSCR and shFNIP2 encoding viruses.

### Bioenergetics assays

Oxygen consumption rates (OCR) and extracellular acidification rate (ECAR) were measured using an XFe-96 Extracellular Flux Analyzer (Agilent Technologies), as previously described [45, 46]. Low passage fibroblast cells stably infected with lentiviruses were seeded on the Seahorse plate at a density of 3 × 10^4^ cells per well and grown overnight. Optimal cell density was determined experimentally to ensure a proportional response to the uncoupler (FCCP) with the cell number and resulting in confluent cultures where cell replication was prevented by contact inhibition. On the experimental day, medium was changed to unbuffered DMEM (XF Base assay Medium–Agilent Technologies) supplemented with 2 mM glutamine, 5 mM glucose and 1 mM sodium pyruvate for mitochondrial respiration analysis, or 2 mM glutamine for glycolysis analysis, and incubated 1 h at 37 °C in the absence of CO_2_. Medium and reagents acidity were adjusted to pH 7.4 on the day of the assay, according to the manufacturer’s procedure. Mitochondrial respiration was measured as OCR. After four baseline measurements for mitochondrial stress tests cells were sequentially challenged with injections of mitochondrial toxins: 1 µM oligomycin (ATP-synthase inhibitor, which informs on the level of respiration allocated for ATP production), 1 µM carbonyl cyanide-p-trifluoromethoxyphenylhydrazone (FCCP, oxidative phosphorylation uncoupler, which informs on the maximal achievable level of respiration), 1 μM rotenone (complex I inhibitor, which informs on the level of respiration dependent on complex I) and 1 μM antimycin A (complex III inhibitor, which completely ablates mitochondrial respiration and informs on the level of non-mitochondrial respiration).

Bioenergetic reagents were previously described [45, 46]. Basal respiration was defined as the average OCR values at baseline. Respiration dedicated to ATP production was calculated as the difference between basal respiration and the respiration measured after oligomycin injection.

Glycolysis was measured as extracellular acidification rate (ECAR), which reflects lactate production via glucose catabolism. ECAR was measured in basal conditions, after stimulation with 30 mM glucose, then with 5 μM oligomycin, and finally inhibited with 100 mM 2-deoxyglucose (2-DG). Basal glycolysis was defined as the average ECAR measures at the baseline, glucose-stimulated glycolysis represented the average of measurements after glucose injection, and Maximal glycolysis was obtained after oligomycin injection. At the end of the run, the Seahorse XF data have been normalized to live cells by fluorescent cell counting using DAPI staining (1 μg/ml) and the Evos image acquisition system. The whole well image captured was subsequently analyzed by the FIJI software in an automatic way. A total of 2 Seahorse replicates were performed for each fibroblast line; in each replicate, a total of 7 wells were used for each line.

### β-Gal staining

β-Gal staining was performed using the β-Gal Staining Kit (K1465-01, Invitrogen) according to the manufacturer’s instructions.

### Determination of thiol-disulfide redox equilibrium

The ratiometric disulfide/thiol redox state was calculated as the disulfide/reduced thiols ratio (SS/SH). Reduced and oxidized cysteines were labeled with IRDye 800 maleimide (Li-COR Biosciences) and Alexa Fluor 680-Maleimide (ThermoFisher Scientific); proteins were extracted from samples and resolved by SDS-electrophoresis as previously described [17]. Briefly, equal amounts of cells were harvested and resuspended in lysis buffer containing 50 mM Tris-HCl, 1% SDS, 1 mM EDTA, 10 mM NEM (N-Ethylmaleimide, Sigma-Aldrich, St. Louis, Mo, USA, E3876), 20 μM IRDye 800 Maleimide to label reduced thiols, and proteinase inhibitors mix. Lysates were incubated for 10 min at 70 °C, sonicated, and finally incubated for 30 min at RT in protected from light. Next, unreacted thiols in samples were quenched with 100 mM NEM for 30 min. Lysates were then precipitated with a cold precipitation solution (50% acetone, 25% methanol, and 25% ethanol) and disulfide bonds reduced with 20 mM TCEP (Tris-(2-carboxyethyl)-phosphine; 20490, ThermoFisher Scientific). Finally, newly reduced thiols were labelled with 20 μM Alexa Fluor 680-Maleimide (A20346, ThermoFisher Scientific). After precipitation, samples were diluted in Laemmli sample buffer, and proteins were resolved under reducing conditions. Finally, the gel was fixed with a fixing solution (50% EtOH, 2.5% Ortho-phosphoric acid) overnight under agitation. The next day, the gel was scanned with an Odyssey Platform (Li-COR Biosciences), and its Image Studio Lite software was used to analyze the fluorescent signal of oxidized and reduced cysteines.

### Isolation of ER-enriched microsomal fractions and measurement of Ca^2+^ uptake

Endoplasmic reticulum (ER)-enriched microsomal membranes were prepared from cultured HEK293 cells treated with CTRL, FNIP1 or FNIP2 siRNAs according to standard differential centrifugation steps to isolate cell membranes [60] using an ER isolation kit (SIGMA, ER0100) with minor modifications. Briefly, for crude ER vesicles 3 × 10^6^ HEK293 cells were plated for each condition in 10 cm petri dishes. Once at 50% confluence 10 nM siRNA were transfected in 10 ml complete medium using Lipofectamine RNAiMAX. Cells were incubated for 48 h and then collected for membrane isolation according to the manufacturer’s instructions. Cells were detached by trypsinization, collected by centrifugation at 600 × *g* for 5 min, washed once in ice-cold PBS, and pelleted again. The packed cell volume (PCV) was determined and cells were resuspended in three volumes of ice-cold 1× Hypotonic Extraction Buffer (10 mM HEPES pH 7.8, 0.03 M KCl, 1 mM EGTA) for 20 min at 4 °C to induce swelling. Cells were then centrifuged at 600 × *g* for 5 min, the supernatant was discarded, and the new PCV was measured. The pellet was resuspended in two volumes of 1× Isotonic Extraction Buffer (10 mM HEPES pH 7.8, 500 mM sucrose, 0.03 M KCl, 1 mM EGTA supplemented with 100 mM PMSF and protease inhibitor cocktail from the same kit and disrupted by 10 strokes in a pre-chilled Dounce homogenizer. Cell homogenates were cleared by sequential centrifugation at 1000 × *g* for 10 min and 8000 × *g* for 10 min at 4 °C to remove nuclei, unbroken cells, mitochondria and heavy membranes. The resulting post-mitochondrial supernatant was subjected to ultracentrifugation at 30,000 × *g* for 120 min at 4 °C. The pellet, corresponding to the crude microsomal/ER vesicle fraction, was gently resuspended in 1× Isotonic Extraction Buffer (approximately 0.3 mL per original mL PCV) using a pellet pestle. Protein concentration was determined by Bradford assay.

The capacity of microsomal vesicles to import Ca²⁺ via the SERCA (sarcoplasmic/endoplasmic reticulum Ca²⁺-ATPase) pump was assessed using a Fura-2-based fluorescence assay [61]. Crude ER membrane preparations (50 μg) were incubated in an assay solution containing 100 mM KCl, 10 mM HEPES (pH 7.4), 10 mM potassium oxalate, 5 mM MgCl₂, 10 μM ruthenium red, and 2 μM Fura-2 free acid (Thermo Fisher). The transport reaction was initiated by adding 1 mM ATP and 2 μM CaCl₂. Changes in free Ca²⁺ levels were monitored by measuring Fura-2 fluorescence ratios at 510 nm emission following excitation at 340 and 380 nm wavelengths in an Agilent fluorescence spectrophotometer equipped with monochromators at room temperature. Where indicated thapsigargin (Sigma-Aldrich, SML-1845) was added at 1 μM in the reaction. As negative control not-enriched extract from untreated HEK293 cells was used. Calcium concentrations were calculated using the standard equation, focusing on the linear portion of the fluorescence trace immediately after Ca²⁺ addition as previously shown [61].

Transmission electron microscopy (TEM) and electron tomography (ET) were performed as previously described [62]. Briefly, for embedding, cells grown on MatTek glass bottom dishes (MatTek Corporation, USA) were fixed with of 4% paraformaldehyde and 2.5% glutaraldehyde (EMS, USA) in 0.2 M sodium cacodylate pH 7.2, for 2 h at room temperature (RT), followed by 6 washes in 0.2 M sodium cacodylate pH 7.2 at RT. Then, the cells were incubated in the 1:1 mixture of 2% osmium tetra oxide and 3% potassium ferrocyanide for 1 h at RT followed by 6 times rinsing in cacodylate buffer. Next, the samples were sequentially treated with 0.3% thiocarbohydrazide in 0.2 M cacodylate buffer for 10 min and 1% OsO_4_ in 0.2 M cacodylate buffer (pH 6.9) for 30 min. Then, samples were rinsed with 0.1 M sodium cacodylate (pH 6.9) buffer until all traces of the yellow osmium fixative had been removed, washed in de-ionized water, treated with 1% uranyl acetate in water for 1 h, and washed in water again [41]. The samples were subsequently subjected to dehydration in ethanol and embedded in Epoxy resin at RT and polymerized for at least 72 h in a 60 °C oven.

For sectioning, embedded samples were then sectioned with a diamond knife (Diatome, Switzerland) using a Leica EM UC7 ultra microtome. Sections were analysed with a Tecnai 20 Electron microscope (FEI, now ThermoFisher Scientific; The Netherlands) operating at 200 kV.

For electron tomography, the ultramicrotome (Leica EM UC7; Leica Microsystems, Vienna) was used to cut 200-nm sections. The sections were collected onto 1% Formvar film adhered to slot grids. Both sides of the grids were labelled with the fiduciary gold marks with a diameter of 10 nm (PAG10, CMC, Utrecht, the Netherlands). Tilt-series were collected from the samples from ±65° with 1° increments at 200 kV in Tecnai 20 electron microscopes (FEI, now ThermoFisher Scientific, Eindhoven, the Netherlands). Tilt series were recorded at a magnification (7600–25,000×) using software supplied with the instrument. The nominal resolution in our tomograms was 4 nm, based on section thickness, the number of tilts, tilt increments, and tilt angle range. The IMOD package and its newest viewer, 3DMOD 4.0.11, were used to construct individual tomograms and for the assignment of the outer leaflet of organelle membrane contours, as described [53]. All the analyses were performed in blind.

### Quantification of mtDNA to nuclear DNA ratio by quantitative real-time PCR

Mitochondrial DNA (mtDNA) copy number relative to nuclear DNA (nDNA) was determined by quantitative real-time PCR (qPCR) on total DNA extracted from human CTRL and AT fibroblasts. Relative mitochondrial DNA content was determined by qPCR targeting the mitochondrial MT-ND1 gene and the single-copy nuclear B2M gene, following a previously validated assay for mtDNA copy number quantification with minor modifications [24]. Briefly, human fibroblasts were grown to ~80% confluence, washed twice with ice-cold PBS and harvested by trypsinization. Cell pellets (approximately 10^5^ cells) were resuspended in lysis buffer and genomic DNA was extracted using a commercial kit (Qiagen) according to the manufacturer’s instructions, including RNase A treatment. DNA was eluted in nuclease-free water or low-EDTA buffer, and concentration and purity (A260/280 and A260/230) were assessed by spectrophotometry. Samples with A260/280 between 1.8 and 2.0 were used for qPCR. DNA was diluted to 2-5 ng/µL for all reactions. qPCR reactions were performed in 96-well plates. All quantitative PCR (qPCR) assays used TaqMan Universal Master Mix (Applied Biosystems) and 5 μM primer/probes in 15 μL reactions performed on a thermo cycler at the following standard thermal parameters: 50 °C for 2 min, 95 °C for 10 min and 40 cycles of 95 °C for 15 s and 60 °C for 1 min. Assessment of relative mtDNA copy content was performed on total DNA isolated using MT-ND1 and B2M primer/probes in three independent experiments each with three technical replicates and calculated using the ∆∆Cq method [63]. Serial dilutions were performed to assess linearity of assay.

## Supplementary information

Supplementary Figures

Dataset S1 Metabolomics

Dataset S2 Metabolomic Statistics

Original Western Blots

TableS1

## Data Availability

All datasets have been included in this manuscript. Materials will be shared by the lead author Vincenzo.costanzo@ifom.eu upon request.

## References

[1] Haj M, Levon A, Frey Y, Hourvitz N, Campisi J, Tzfati Y, et al. Accelerated replicative senescence of ataxia-telangiectasia skin fibroblasts is retained at physiologic oxygen levels, with unique and common transcriptional patterns. Aging Cell. 2023;22:e13869. 37254625 10.1111/acel.13869 PMC10410012

[2] Shiloh Y, Lederman HM. Ataxia-telangiectasia (A-T): an emerging dimension of premature ageing. Ageing Res Rev. 2017;33:76–88. 27181190 10.1016/j.arr.2016.05.002

[3] Ambrose M, Gatti RA. Pathogenesis of ataxia-telangiectasia: the next generation of ATM functions. Blood. 2013;121:4036–45. 23440242 10.1182/blood-2012-09-456897 PMC3709651

[4] Shiloh Y, Ziv Y. The ATM protein kinase: regulating the cellular response to genotoxic stress, and more. Nat Rev Mol cell Biol. 2013;14:197–210. 23486281 10.1038/nrm3546

[5] Stagni V, Cirotti C, Barila D. Ataxia-Telangiectasia Mutated Kinase in the Control of Oxidative Stress, Mitochondria, and Autophagy in Cancer: A Maestro With a Large Orchestra. Front Oncol. 2018;8:73. 29616191 10.3389/fonc.2018.00073 PMC5864851

[6] Schneider JG, Finck BN, Ren J, Standley KN, Takagi M, Maclean KH, et al. ATM-dependent suppression of stress signaling reduces vascular disease in metabolic syndrome. Cell Metab. 2006;4:377–89. 17084711 10.1016/j.cmet.2006.10.002

[7] Cosentino C, Grieco D, Costanzo V. ATM activates the pentose phosphate pathway promoting anti-oxidant defence and DNA repair. EMBO J. 2011;30:546–55. 21157431 10.1038/emboj.2010.330 PMC3034007

[8] TeSlaa T, Ralser M, Fan J, Rabinowitz JD. The pentose phosphate pathway in health and disease. Nat Metab. 2023;5:1275–89. 37612403 10.1038/s42255-023-00863-2 PMC11251397

[9] Zhang Y, Lee JH, Paull TT, Gehrke S, D'Alessandro A, Dou Q, et al. Mitochondrial redox sensing by the kinase ATM maintains cellular antioxidant capacity. Sci Signal. 2018;11:eaaq0702. 10.1126/scisignal.aaq0702 PMC6042875 29991649

[10] Guo Z, Kozlov S, Lavin MF, Person MD, Paull TT. ATM activation by oxidative stress. Science. 2010;330:517–21. 20966255 10.1126/science.1192912

[11] Lee JH, Paull TT. Cellular functions of the protein kinase ATM and their relevance to human disease. Nat Rev Mol cell Biol. 2021;22:796–814. 34429537 10.1038/s41580-021-00394-2

[12] Shiloh Y, Tabor E, Becker Y. In vitro phenotype of ataxia-telangiectasia (AT) fibroblast strains: clues to the nature of the “AT DNA lesion” and the molecular defect in AT. Kroc Found Ser. 1985;19:111–21. 3864933

[13] Stagni V, Ferri A, Cirotti C, Barila D. ATM kinase-dependent regulation of autophagy: a key player in senescence? Front Cell Dev Biol. 2020;8:599048. 33490066 10.3389/fcell.2020.599048 PMC7817534

[14] Takagi Y, Kobayashi T, Shiono M, Wang L, Piao X, Sun G, et al. Interaction of folliculin (Birt-Hogg-Dube gene product) with a novel Fnip1-like (FnipL/Fnip2) protein. Oncogene. 2008;27:5339–47. 18663353 10.1038/onc.2008.261

[15] Broeckling CD, Beger RD, Cheng LL, Cumeras R, Cuthbertson DJ, Dasari S, et al. Current Practices in LC-MS Untargeted Metabolomics: A Scoping Review on the Use of Pooled Quality Control Samples. Anal Chem. 2023;95:18645–54. 38055671 10.1021/acs.analchem.3c02924 PMC10753522

[16] Gil A, van der Pol A, van der Meer P, Bischoff R. LC-MS analysis of key components of the glutathione cycle in tissues and body fluids from mice with myocardial infarction. J Pharm Biomed Anal. 2018;160:289–96. 30114606 10.1016/j.jpba.2018.08.001

[17] Mastroberardino PG, Orr AL, Hu X, Na HM, Greenamyre JT. A FRET-based method to study protein thiol oxidation in histological preparations. Free Radic Biol Med. 2008;45:971–81. 18620047 10.1016/j.freeradbiomed.2008.06.018 PMC2605956

[18] Bottini E. Favism: current problems and investigations. J Med Genet. 1973;10:154–7. 4714581 10.1136/jmg.10.2.154 PMC1013006

[19] Yeo AJ, Chong KL, Gatei M, Zou D, Stewart R, Withey S, et al. Impaired endoplasmic reticulum-mitochondrial signaling in ataxia-telangiectasia. iScience. 2021;24:101972. 33437944 10.1016/j.isci.2020.101972 PMC7788243

[20] Valentin-Vega YA, Maclean KH, Tait-Mulder J, Milasta S, Steeves M, Dorsey FC, et al. Mitochondrial dysfunction in ataxia-telangiectasia. Blood. 2012;119:1490–500. 22144182 10.1182/blood-2011-08-373639 PMC3286212

[21] Ambrose M, Goldstine JV, Gatti RA. Intrinsic mitochondrial dysfunction in ATM-deficient lymphoblastoid cells. Hum Mol Genet. 2007;16:2154–64. 17606465 10.1093/hmg/ddm166

[22] Zhang J, Zhang Q. Using seahorse machine to measure OCR and ECAR in cancer cells. Methods Mol Biol. 2019;1928:353–63. 30725464 10.1007/978-1-4939-9027-6_18

[23] Ahn D, Go RE, Choi KC. Oxygen consumption rate to evaluate mitochondrial dysfunction and toxicity in cardiomyocytes. Toxicol Res. 2023;39:333–9. 37398565 10.1007/s43188-023-00183-3 PMC10313613

[24] Belmonte FR, Martin JL, Frescura K, Damas J, Pereira F, Tarnopolsky MA, et al. Digital PCR methods improve detection sensitivity and measurement precision of low abundance mtDNA deletions. Sci Rep. 2016;6:25186. 27122135 10.1038/srep25186 PMC4848546

[25] Locasale JW, Cantley LC. Metabolic flux and the regulation of mammalian cell growth. Cell Metab. 2011;14:443–51. 21982705 10.1016/j.cmet.2011.07.014 PMC3196640

[26] Zamboni N. 13C metabolic flux analysis in complex systems. Curr Opin Biotechnol. 2011;22:103–8. 20833526 10.1016/j.copbio.2010.08.009

[27] Lee WN, Sorou S, Bergner EA. Glucose isotope, carbon recycling, and gluconeogenesis using [U-13C]glucose and mass isotopomer analysis. Biochem Med Metab Biol. 1991;45:298–309. 2049183 10.1016/0885-4505(91)90034-i

[28] An Y, Young SP, Kishnani PS, Millington DS, Amalfitano A, Corz D, et al. Glucose tetrasaccharide as a biomarker for monitoring the therapeutic response to enzyme replacement therapy for Pompe disease. Mol Genet Metab. 2005;85:247–54. 15886040 10.1016/j.ymgme.2005.03.010

[29] Clark AG, Mansford KR. Isolation of maltotriose and maltotetraose from starch hydrolysates. Nature. 1963;200:30–32. 14074622 10.1038/200030a0

[30] Zakout YM, Abdellah MA, Abdallah MA, Batran SA. Optimization of PAS stain and similar Schiff's based methods for glycogen demonstration in liver tissue. Histochem Cell Biol. 2024;161:359–64. 10.1007/s00418-023-02261-x 38147127

[31] Fu DA, Campbell-Thompson M. Periodic acid-schiff staining with diastase. Methods Mol Biol. 2017;1639:145–9. 28752454 10.1007/978-1-4939-7163-3_14

[32] Gonzalez F, Boue S, Izpisua Belmonte JC. Methods for making induced pluripotent stem cells: reprogramming a la carte. Nat Rev Genet. 2011;12:231–42. 21339765 10.1038/nrg2937

[33] Lee JH, Ryu SW, Ender NA, Paull TT. Poly-ADP-ribosylation drives loss of protein homeostasis in ATM and Mre11 deficiency. Mol cell. 2021;81:1515–33 e1515. 33571423 10.1016/j.molcel.2021.01.019 PMC8026623

[34] Bastianello G, Porcella G, Beznoussenko GV, Kidiyoor G, Ascione F, Li Q, et al. Cell stretching activates an ATM mechano-transduction pathway that remodels cytoskeleton and chromatin. Cell Rep. 2023;42:113555. 38088930 10.1016/j.celrep.2023.113555

[35] Wingard MC, Frasier CR, Singh M, Singh K. Heart failure and diabetes: role of ATM. Curr Opin Pharm. 2020;54:27–35. 10.1016/j.coph.2020.06.007 PMC7769978 32745970

[36] Yasuda M, Furuyashiki T, Nakamura T, Kakutani R, Takata H, Ashida H. Immunomodulatory activity of enzymatically synthesized glycogen and its digested metabolite in a co-culture system consisting of differentiated Caco-2 cells and RAW264.7 macrophages. Food Funct. 2013;4:1387–93. 23872795 10.1039/c3fo60035a

[37] Duran J, Tevy MF, Garcia-Rocha M, Calbo J, Milan M, Guinovart JJ. Deleterious effects of neuronal accumulation of glycogen in flies and mice. EMBO Mol Med. 2012;4:719–29. 22549942 10.1002/emmm.201200241 PMC3494072

[38] Gumus E, Ozen H. Glycogen storage diseases: an update. World J Gastroenterol. 2023;29:3932–63. 37476587 10.3748/wjg.v29.i25.3932 PMC10354582

[39] Wen H, Deng H, Li B, Chen J, Zhu J, Zhang X, et al. Mitochondrial diseases: from molecular mechanisms to therapeutic advances. Signal Transduct Target Ther. 2025;10:9. 39788934 10.1038/s41392-024-02044-3 PMC11724432

[40] Subramanian GN, Yeo AJ, Gatei MH, Coman DJ, Lavin MF. Metabolic Stress and Mitochondrial Dysfunction inAtaxia-Telangiectasia. Antioxidants (Basel). 2022;11:653. 10.3390/antiox11040653 PMC9032508 35453338

[41] Baba M, Hong SB, Sharma N, Warren MB, Nickerson ML, Iwamatsu A, et al. Folliculin encoded by the BHD gene interacts with a binding protein, FNIP1, and AMPK, and is involved in AMPK and mTOR signaling. Proc Natl Acad Sci USA. 2006;103:15552–7. 17028174 10.1073/pnas.0603781103 PMC1592464

[42] Hasumi H, Baba M, Hong SB, Hasumi Y, Huang Y, Yao M, et al. Identification and characterization of a novel folliculin-interacting protein FNIP2. Gene. 2008;415:60–67. 18403135 10.1016/j.gene.2008.02.022 PMC2727720

[43] de Martin Garrido N, Aylett CHS. Nutrient signaling and lysosome positioning crosstalk through a multifunctional protein, folliculin. Front Cell Dev Biol. 2020;8:108. 32195250 10.3389/fcell.2020.00108 PMC7063858

[44] Hong X, Munoz-Canoves P. Measuring oxygen consumption rate (OCR) and extracellular acidification rate (ECAR) in muscle stem cells using a seahorse analyzer: applicability for aging studies. Methods Mol Biol. 2023;2640:73–88. 36995588 10.1007/978-1-0716-3036-5_6

[45] Milanese C, Tapias V, Gabriels S, Cerri S, Levandis G, Blandini F, et al. Mitochondrial complex I reversible S-nitrosation improves bioenergetics and is protective in Parkinson’s disease. Antioxid Redox Signal. 2018;28:44–61. 28816057 10.1089/ars.2017.6992 PMC5749586

[46] Milanese C, Cerri S, Ulusoy A, Gornati SV, Plat A, Gabriels S, et al. Activation of the DNA damage response in vivo in synucleinopathy models of Parkinson’s disease. Cell Death Dis. 2018;9:818. 30050065 10.1038/s41419-018-0848-7 PMC6062587

[47] Manford AG, Rodriguez-Perez F, Shih KY, Shi Z, Berdan CA, Choe M, et al. A cellular mechanism to detect and alleviate reductive stress. Cell. 2020;183:46–61. e21. 32941802 10.1016/j.cell.2020.08.034

[48] Malik N, Ferreira BI, Hollstein PE, Curtis SD, Trefts E, Weiser Novak S, et al. Induction of lysosomal and mitochondrial biogenesis by AMPK phosphorylation of FNIP1. Science. 2023;380:eabj5559. 37079666 10.1126/science.abj5559 PMC10794112

[49] Yin Y, Xu D, Mao Y, Xiao L, Sun Z, Liu J, et al. FNIP1 regulates adipocyte browning and systemic glucose homeostasis in mice by shapingintracellular calcium dynamics. J Exp Med. 2022;219:e20212491. 10.1084/jem.20212491 PMC9008465 35412553

[50] Zeng F, Cao J, Li W, Zhou Y, Yuan X. FNIP1: A key regulator of mitochondrial function. Biomed Pharmacother. 2024;177:117146. 39013219 10.1016/j.biopha.2024.117146

[51] Lytton J, Westlin M, Hanley MR. Thapsigargin inhibits the sarcoplasmic or endoplasmic reticulum Ca-ATPase family of calcium pumps. J Biol Chem. 1991;266:17067–71. 1832668

[52] Rizzuto R, Bernardi P, Pozzan T. Mitochondria as all-round players of the calcium game. J Physiol. 2000;529 Pt 1:37–47. 11080249 10.1111/j.1469-7793.2000.00037.x PMC2270183

[53] Beznoussenko GV, Mironov AA. Correlative video-light-electron microscopy of mobile organelles. Methods Mol Biol. 2015;1270:321–46. 25702127 10.1007/978-1-4939-2309-0_23

[54] Biazik J, Yla-Anttila P, Vihinen H, Jokitalo E, Eskelinen EL. Ultrastructural relationship of the phagophore with surrounding organelles. Autophagy. 2015;11:439–51. 25714487 10.1080/15548627.2015.1017178 PMC4502653

[55] Bulow MH, Sellin J. New discoveries in ER-mitochondria communication. Biochem Soc Trans. 2023;51:571–7. 36892405 10.1042/BST20221305

[56] Shiiba I, Ito N, Oshio H, Ishikawa Y, Nagao T, Shimura H, et al. ER-mitochondria contacts mediate lipid radical transfer via RMDN3/PTPIP51 phosphorylation to reduce mitochondrial oxidative stress. Nat Commun. 2025;16:1508. 39929810 10.1038/s41467-025-56666-4 PMC11811300

[57] Fruman DA, Chiu H, Hopkins BD, Bagrodia S, Cantley LC, Abraham RT. The PI3K Pathway in Human Disease. Cell. 2017;170:605–35. 28802037 10.1016/j.cell.2017.07.029 PMC5726441

[58] Hannibal L, Theimer J, Wingert V, Klotz K, Bierschenk I, Nitschke R, et al. Metabolic profiling in human fibroblasts enables subtype clustering in glycogen storage disease. Front Endocrinol. 2020;11:579981. 10.3389/fendo.2020.579981 PMC7719825 33329388

[59] Bruhn C, Ajazi A, Ferrari E, Lanz MC, Batrin R, Choudhary R, et al. The Rad53(CHK1/CHK2)-Spt21(NPAT) and Tel1(ATM) axes couple glucose tolerance to histone dosage and subtelomeric silencing. Nat Commun. 2020;11:4154. 32814778 10.1038/s41467-020-17961-4 PMC7438486

[60] Li CC, Hochstadt J. Transport mechanisms in isolated plasma membranes. Nucleoside processing by membrane vesicles from mouse fibroblast cells grown in defined medium. J Biol Chem. 1976;251:1175–80. 2604

[61] Kargacin ME, Kargacin GJ. Methods for determining cardiac sarcoplasmic reticulum Ca2+ pump kinetics from fura 2 measurements. Am J Physiol. 1994;267:C1145–51. 7943278 10.1152/ajpcell.1994.267.4.C1145

[62] Beznoussenko GV, Kweon HS, Sesorova IS, Mironov AA. Comparison of the Cisterna Maturation-Progression Modelwith the Kiss-and-Run Model of Intra-Golgi Transport: Role of Cisternal Pores and Cargo Domains. Int J Mol Sci. 2022;23:3590. 10.3390/ijms23073590 PMC8999060 35408951

[63] Krishnan KJ, Bender A, Taylor RW, Turnbull DM. A multiplex real-time PCR method to detect and quantify mitochondrial DNA deletions in individual cells. Anal Biochem. 2007;370:127–9. 17662684 10.1016/j.ab.2007.06.024

