# Supplementary material for: Targeting the FNIP2-SERCA2b axis improves metabolic and mitochondrial defects in Ataxia Telangiectasia
Source: Cell Death Dis. 2026 Mar 2;17(1):290. doi: 10.1038/s41419-026-08507-5 (PMC13031930; doi:10.1038/s41419-026-08507-5)
Supplement: Supplementary file 1 — Supplementary Figures [file 41419_2026_8507_MOESM1_ESM.pdf]

Supplementary figures and legend:

Targeting the FNIP2-SERCA2b axis improves metabolic and mitochondrial defects in Ataxia Telangiectasia

Maria Vinciguerra[1#], Catiana El Kharef[1#], Christopher Bruhn[1#], Lucia Falbo[1,2#], Chiara Milanese[1], Matteo Audano[3], Galina V. Beznoussenko[1], Alexander A. Mironov[1], Domenico Delia[1], Marco Foiani[1,2], Pier Giorgio Mastroberardino[1], Nico Mitro[3, 4] and Vincenzo Costanzo[1,2*]

[1]IFOM-ETS, The AIRC Institute of Molecular Oncology

[2]Department of Oncology and Hematology-Oncology, University of Milan, 20133 Milan, Italy

[3]Department of Pharmacological and Biomolecular Sciences "Rodolfo Paoletti", University of Milan, 20133 Milan, Italy

[4]Department of Experimental Oncology, IEO, European Institute of Oncology IRCCS, Milan, Italy.

Keywords: ATM, Ataxia-Telangiectasia, oxidative stress, cell metabolism, glycolysis, PPP, neurodegeneration, mitochondria

# These authors contributed equally

*Corresponding author: Vincenzo.Costanzo@IFOM.EU

Supplementary Fig 1

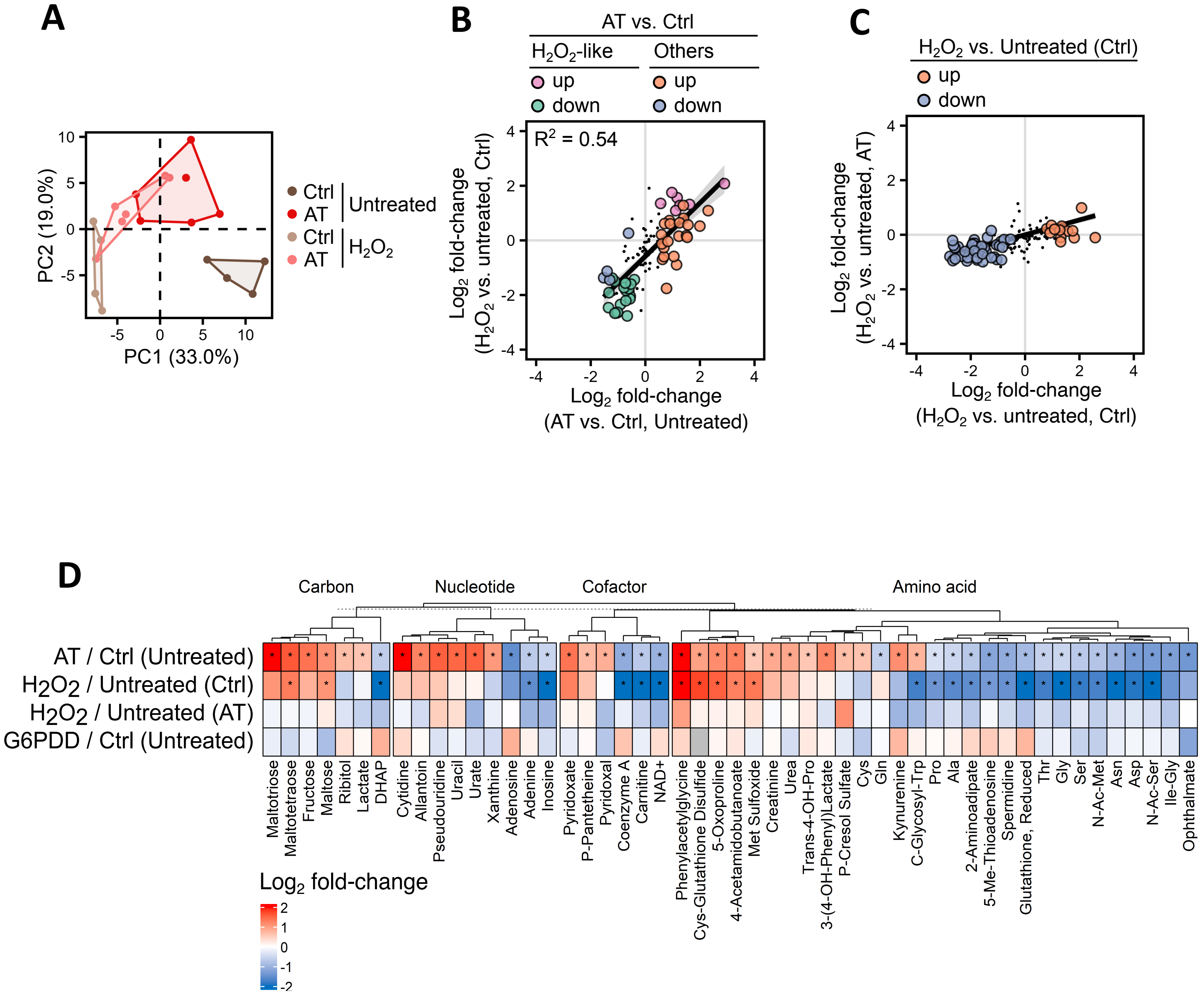

Supplementary Figure 1. **Oxidative stress as a primary driver of metabolic disruption in AT cells.**

A) Principal component analysis contrasting CTRL and AT cells based on metabolite concentrations, with the first two components and explained variance presented. Groups are represented with convex hulls.

B) Comparative analysis of metabolite concentrations in CTRL cells treated with H_2O_2 and AT cells, categorizing metabolites into H_2O_2-specific or AT-specific based on alteration patterns. The squared Pearson correlation coefficient (R^2) is shown as a correlation measure.

C) Graph showing relative levels of metabolites in H_2O_2-treated versus untreated AT cells compared to H_2O_2-treated versus untreated CTRL cells.

D) Heatmap of metabolite fold-changes in AT, H_2O_2, and G6PD deficiency. Metabolites significantly altered in AT vs. CTRL cells are shown and grouped by super-pathway. Fold-changes vs. respective controls are indicated by fill color. Regulation significance with Benjamin-Hochberg-adjusted *p*-value below 0.05 is indicated by asterisks. Comprehensive significance details are in Dataset S2. Volcano plots are shown in Supplementary Figure S2.

Supplementary Fig 2

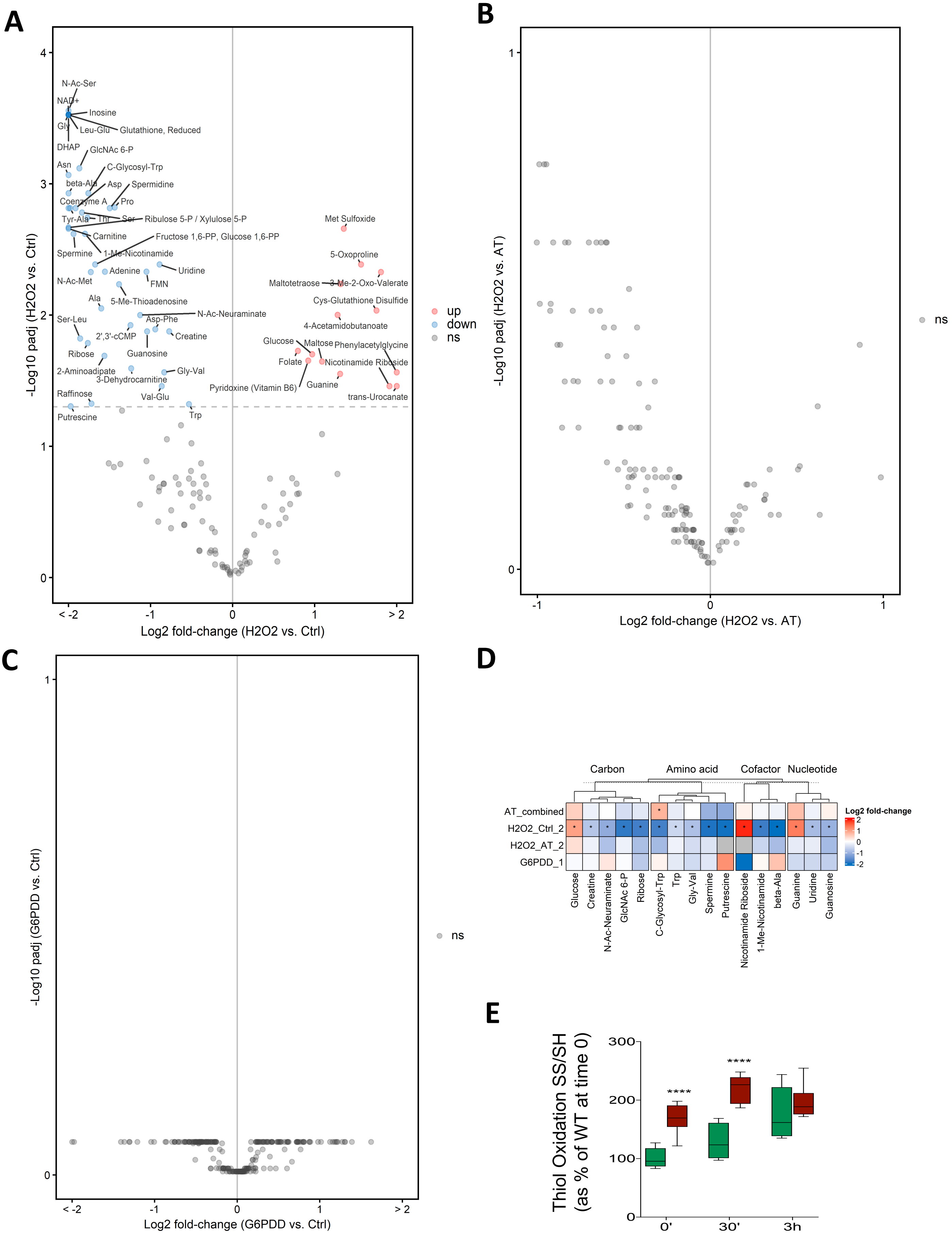

Supplementary Figure 2. Significantly regulated metabolites.

A-C) Volcano plots showing significantly regulated metabolites in the indicated conditions. Significance analysis was performed with LIMMA. The x and y axes show the mean log2 metabolite level fold-changes and the Benjamini-Hochberg-adjusted, negative log-transformed *p* value, respectively. Significant metabolites are labeled. Up- and down-regulated metabolites are marked with red and blue fill color, respectively. ns=non-significant

D) Heatmap of metabolite fold-changes in AT, H_2O_2, and G6PD deficiency. Metabolites significantly altered by H_2O_2 treatment of CTRL cells but not altered in the same direction in AT vs. CTRL cells are shown and grouped by super-pathway. Fold-changes vs. respective controls are indicated by fill color. Regulation significance with Benjamin-Hochberg-adjusted *p*-value below 0.05 is indicated by asterisks. ns=non-significant. Comprehensive significance details are in Dataset S2.

E) Quantification of thiol oxidation in CTRL (green) and AT (red) cells.

Supplementary Fig 3

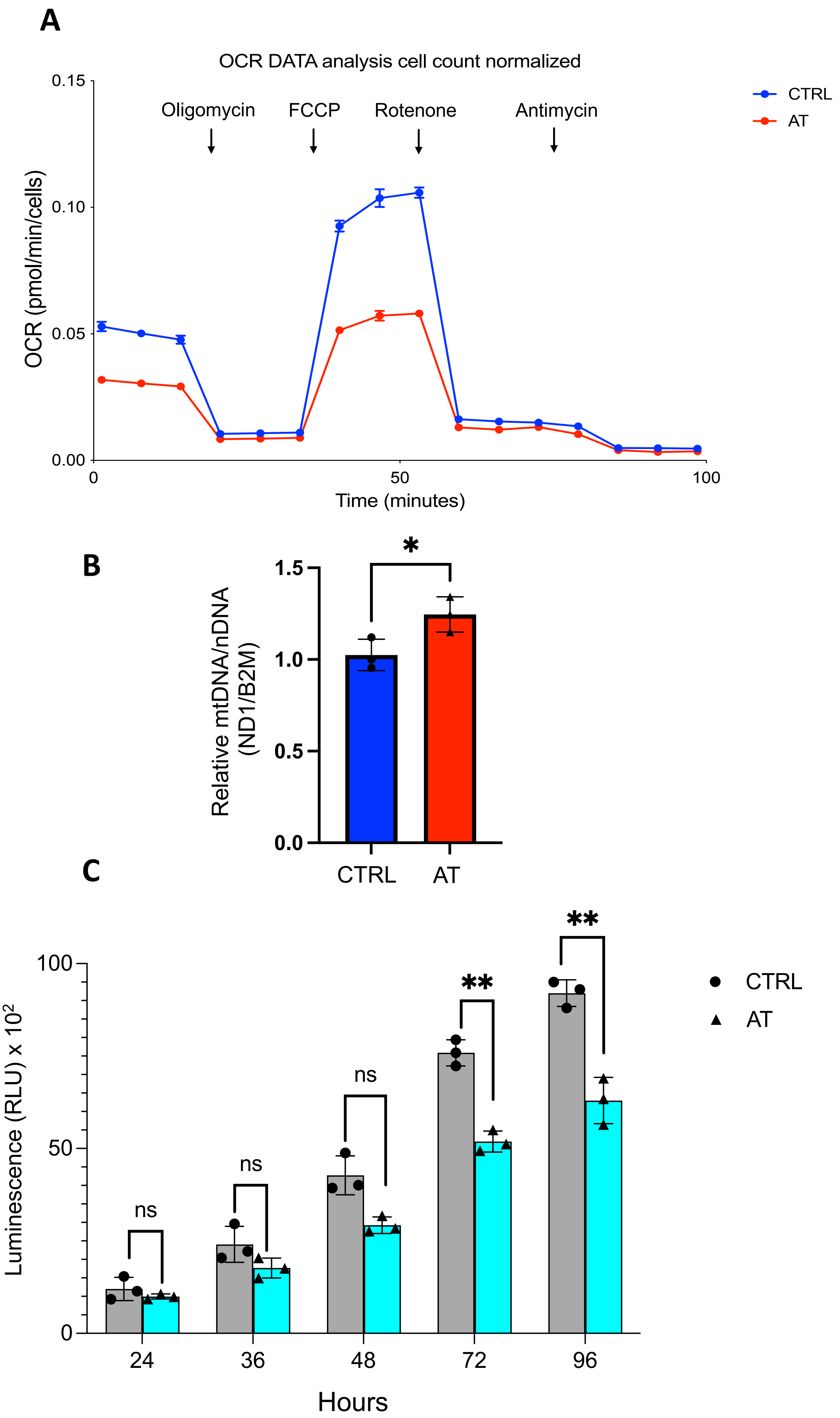

Supplementary Figure 3. Mitochondrial oxygen consumption rates and mass are affected in proliferating AT cells.

A) Oxygen consumption rate and normalized to live cells of performed on CTRL (Green) and AT (Red) fibroblast cells 36 hours after plating. For each fibroblast line, two independent Seahorse runs were performed on different days. Within each run, 7 wells per line were measured. Plots display all wells; overlaid symbols show plate-adjusted means with 95% CIs from a model that includes 'run/plate' as a factor.

B) mtDNA:nDNA ratio in CTRL and AT cells was quantified by qPCR using primers for MT-ND1 and B2M genes. Values were normalized to CTRL fibroblasts set to 1 and are presented as mean ± SD from three independent DNA preparations each measured in technical triplicate. Statistical significance was evaluated using an unpaired two-tailed Student's t test. *$p<0.05$

C) Graph showing cell proliferation of CTRL and AT fibroblasts after plating for the indicated times and quantified by the cell viability assay CellTiter-Glo. Normalized results from three independent experiments are shown in relative luminescence units (RLU). Error bars show SD. Multiple t-tests were used to calculate significance between the indicated couples for each time point. ** $p <0.01$; ns=not significant.

Supplementary Fig 4

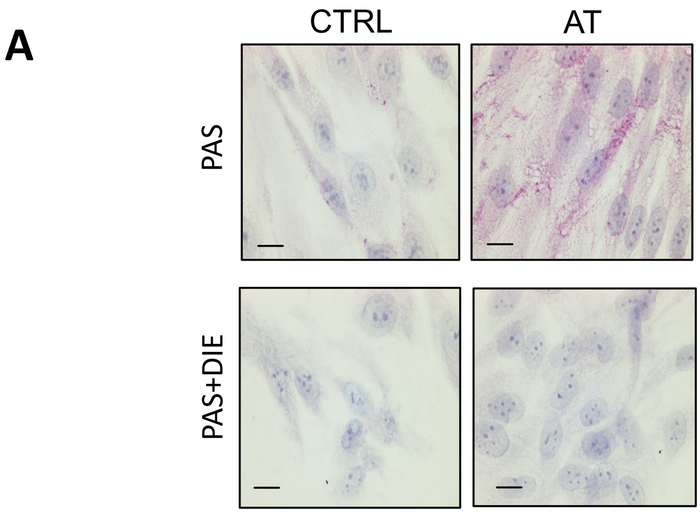

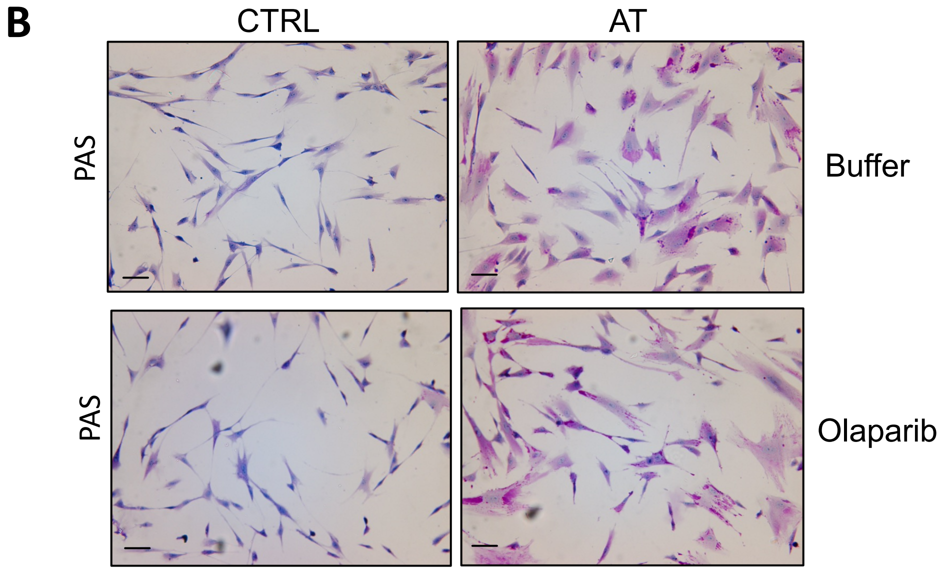

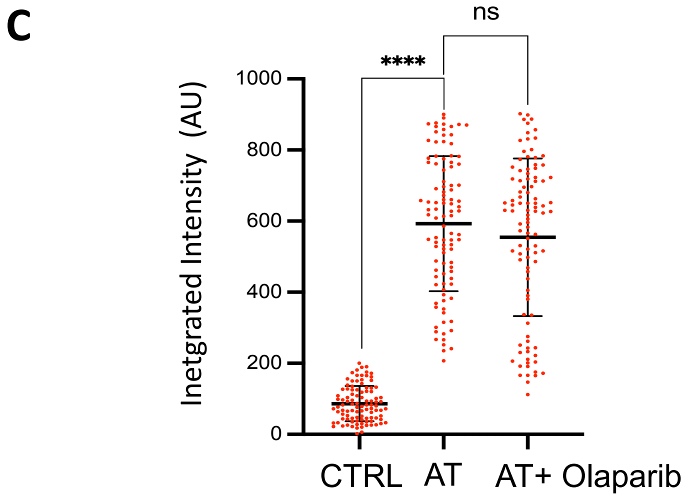

Supplementary Figure 4. Differential glycogen storage in AT cells evidenced by PAS staining is not affected by PARP1 inhibition.

A) PAS staining of CTRL and AT cells pretreated with buffer or diastase (DIE). Scale bar: 5 μm.

B) PAS staining of CTRL and AT cells treated with buffer or 50 μM Olaparib for 36 h. Scale bar: 20μm.

C) Integrated optical density for PAS staining of the samples shown in A. Each dot represents a single cell. Statistical analysis was conducted using One-Way ANOVA; ****$P<0.0001$. At least 70 cells pooled from three independent experiments were scored for each sample.

Supplementary Fig 5

A

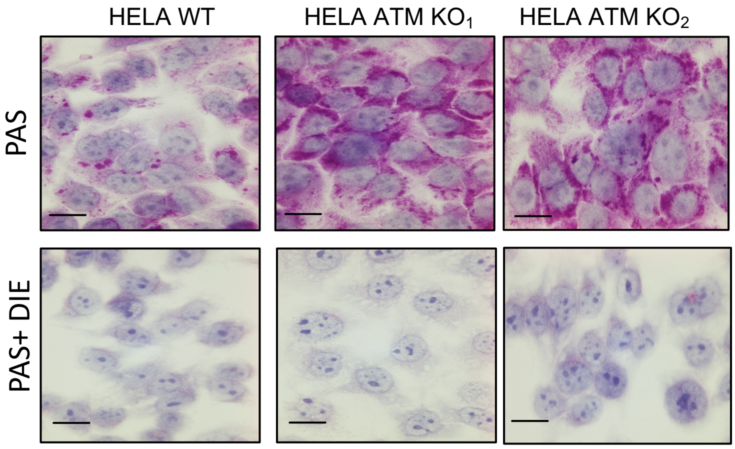

B

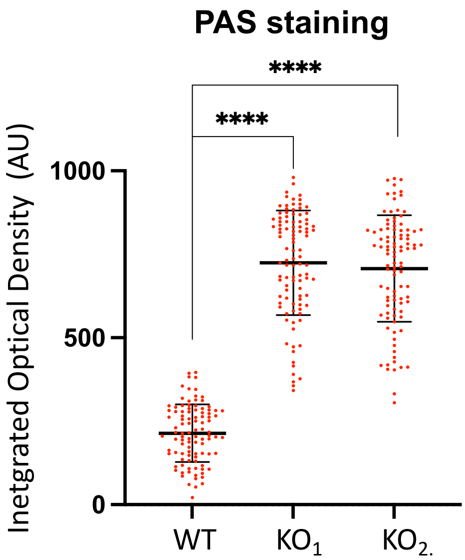

Supplementary Figure 5. PAS staining reveals glycogen accumulation in two different ATM$^{-/-}$ HeLa cell clones .

A) PAS staining of WT and HeLa ATM$^{-/-}$ (KO_1 and KO_2) cells before and after diastase (DIE). Scale bar: 20μm.

B) Integrated optical density for PAS staining of the samples shown in B. Each dot represents a single cell. Statistical analysis was conducted using One-Way ANOVA; ****$P<0.0001$. At least 100 cells pooled from three independent experiments were scored for each sample (n=100).

Supplementary Fig 6

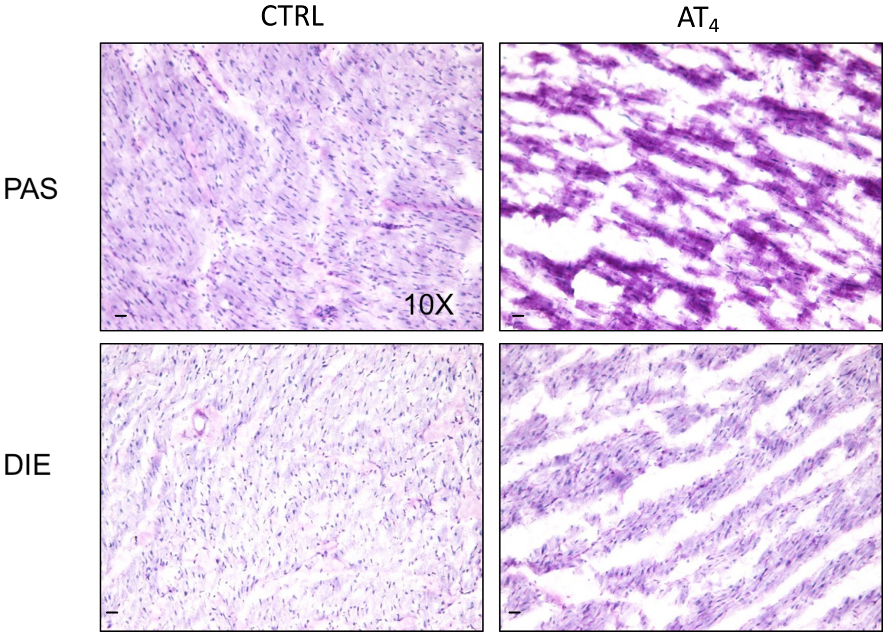

Supplementary Figure 6. Glycogen accumulation in AT heart muscle.

PAS staining of heart muscle sections from CTRL and AT patients before and after diastase (DIE).

Scale bar: 20µm. 10x objective.

Supplementary Fig 7

A

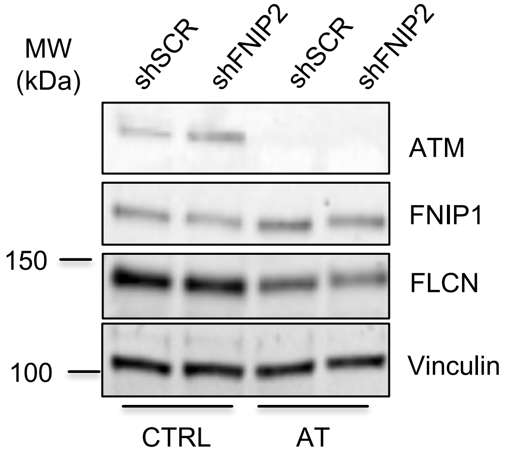

B

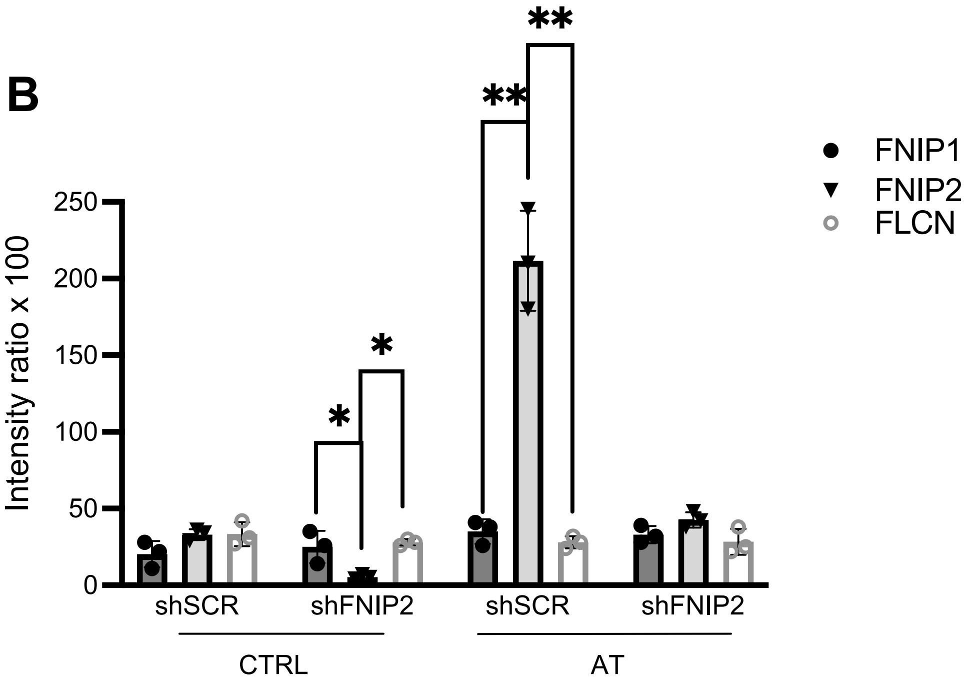

Supplementary Figure 7. FNIP1, FNIP2 and FLCN protein levels in CTRL and AT cells.

A) Immunoblots of the indicated proteins following stable infection of CTRL and AT with shSCR or shFNIP2 lentiviral vector.

B) Quantification of FNIP1, FNIP2 and FLCN expression levels in CTRL and AT cells normalized to Vinculin. Horizontal bars indicate mean ± SD. Symbols indicate $n = 3$ independent biological replicate values. One-way ANOVA, followed by Dunnett's post hoc test for multiple comparisons; $**p < 0.01$; $*p < 0.05$.

Supplementary Fig 8

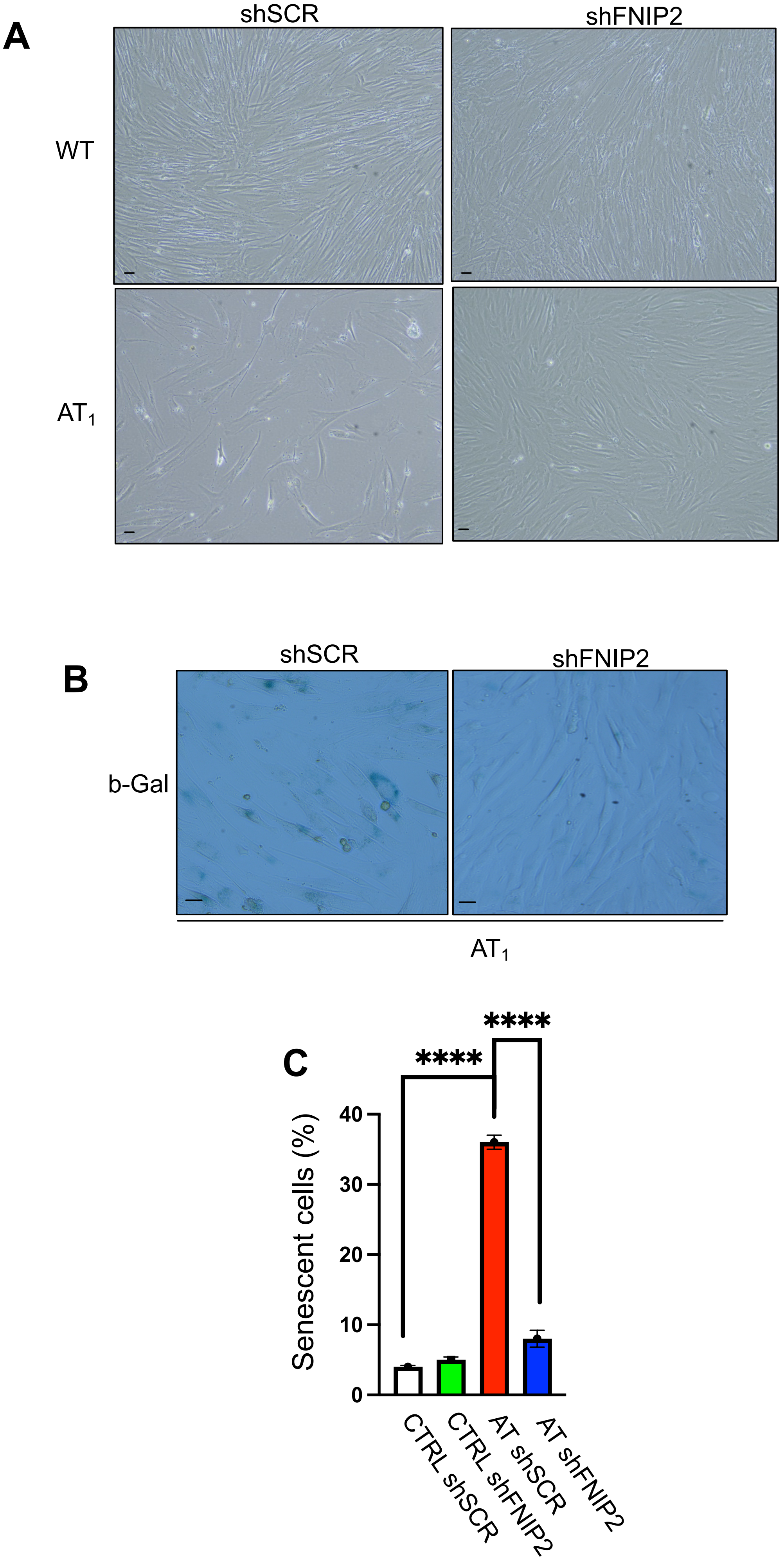

Supplementary Figure 8. FNIP2 stable suppression restores proliferation and prevents senescence in AT cells.

A) Light microscopy of CTRL and AT cells infected with shSCR or shFNIP2 as indicated and cultivated for three weeks. Scale bar: 20µm.

B) β-Galactosidase (β-Gal) staining for the detection of senescent cells was performed on AT cells infected with shSCR or shFNIP2 and cultivated for three weeks. Scale bar: 20µm.

C) Graph showing percentage of senescent cells following infection with the indicated lentivirus after four-week culture. Experiments were repeated three times counting >300 cells for each experiment. Average results are shown. One-way ANOVA; ****$p<0.0001$

Supplementary Fig 9

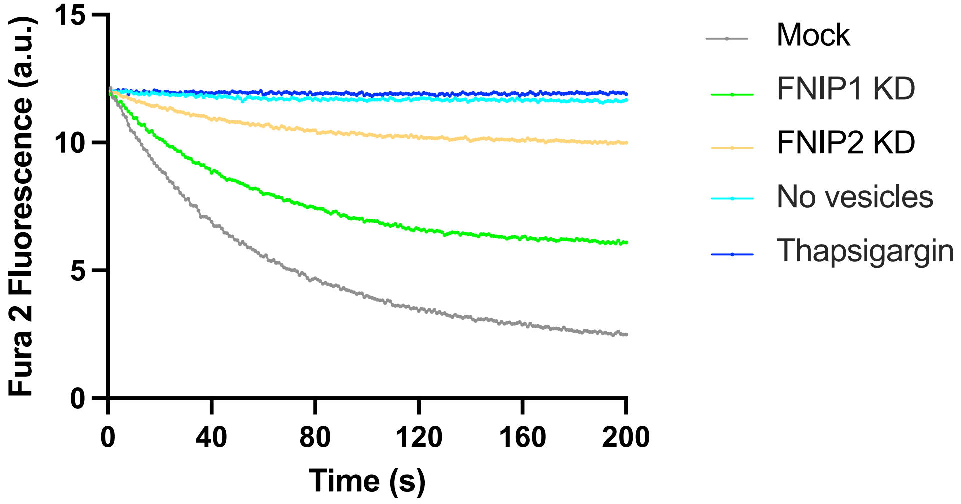

Supplementary Figure 9. Real-time Ca^{2+} uptake measurements

The graph shows Fura-2 fluorescence traces vs. time in arbitrary intensity units (a.u.) related to Ca^{2+} uptake measured in reactions containing ER vesicle preparations derived from CTRL HEK293 cells treated with no siRNA (Mock), siRNA against FNIP1 (FNIP1 KD), siRNA against FNIP2 (FNIP2 KD), 1 μM Thapsigargin or not enriched cell extracts (no vesicles).

Supplementary Fig 10

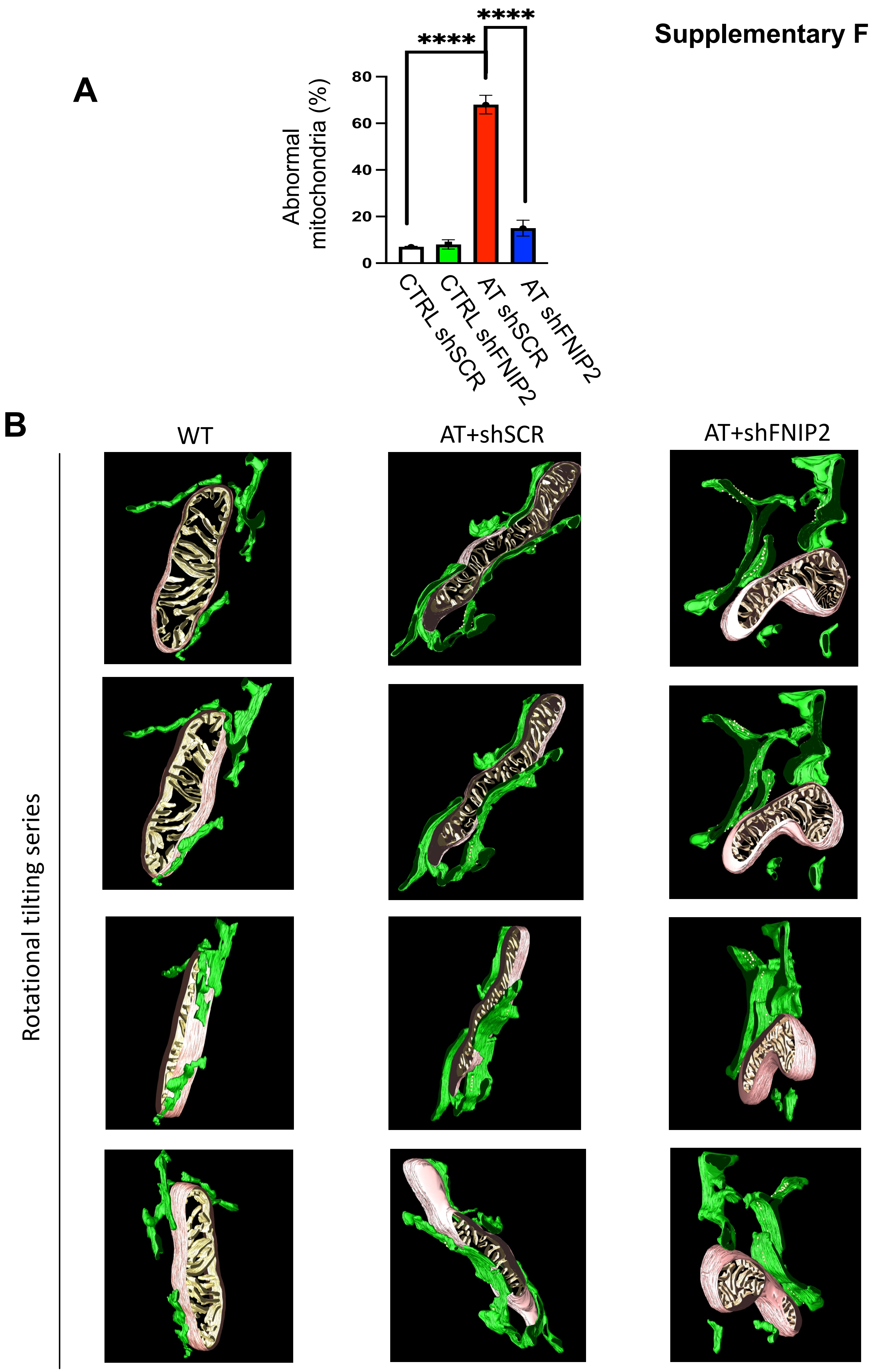

Supplementary Figure 10. Electron tomography of CTRL and AT cells following FNIP2 suppression.

A) Quantification of abnormal mitochondria counted by EM in randomly selected CTRL and AT fibroblasts infected with shSCR or shFNIP2 lentivirus as indicated and expressed as a percentage. Experiments were repeated three times, scoring each time a different cell. Average values are shown. One-way ANOVA; ****P<0.0001.

B) Rotational series of reconstructed electron tomography (ET) images of CTRL and AT fibroblasts infected with shSCR or shFNIP2 lentivirus as indicated. ER Membranes are colored green.
