# Supplementary material for: Targeting the FNIP2-SERCA2b axis improves metabolic and mitochondrial defects in Ataxia Telangiectasia
Source: Cell Death Dis. 2026 Mar 2;17(1):290. doi: 10.1038/s41419-026-08507-5 (PMC13031930; doi:10.1038/s41419-026-08507-5)
Supplement: Supplementary file 2 — Dataset S1 Metabolomics [file 41419_2026_8507_MOESM2_ESM.pdf]

Table S3: Metabolomics Data

Metabolite ID	Name	Super Pathway	Dataset	Sample ID	Group ID	Detection Fraction	Pathway	Detected	Raw Intensity	Log2 Norm Intensity	Norm Imputed	Log2 Ctrl Norm
76	Gln	Amino acid	2	AT_H2O2_2_1	AT_H2O2_2	1.0	Proteinogenic amino acids	True	8.302e+06	6.075	6.075	-0.8773
89	Trp	Amino acid	2	AT_H2O2_2_1	AT_H2O2_2	1.0	Proteinogenic amino acids	True	7.728e+06	5.971	5.971	4.70e-03
723	beta-Ala	Cofactor	2	AT_H2O2_2_1	AT_H2O2_2	1.0	Coenzyme A biosynthesis	True	2.344e+05	0.9282	0.9282	0.602
75	Glu	Amino acid	2	AT_H2O2_2_1	AT_H2O2_2	1.0	Proteinogenic amino acids	True	6.595e+06	5.743	5.743	-0.2052
80	His	Amino acid	2	AT_H2O2_2_1	AT_H2O2_2	1.0	Proteinogenic amino acids	True	1.399e+05	0.1834	0.1834	-0.03076
82	Leu	Amino acid	2	AT_H2O2_2_1	AT_H2O2_2	1.0	Proteinogenic amino acids	True	3.285e+07	8.059	8.059	-0.1011
87	Phe	Amino acid	2	AT_H2O2_2_1	AT_H2O2_2	1.0	Proteinogenic amino acids	True	2.994e+07	7.925	7.925	0.03323
236	Spermidine	Amino acid	2	AT_H2O2_2_1	AT_H2O2_2	1.0	Polyamines	True	3.22e+05	1.386	1.386	-1.441
73	Asn	Amino acid	2	AT_H2O2_2_1	AT_H2O2_2	1.0	Proteinogenic amino acids	True	4.566e+05	1.89	1.89	-0.8587
243	Creatinine	Amino acid	2	AT_H2O2_2_1	AT_H2O2_2	1.0	Creatine degradation	True	9.306e+05	2.917	2.917	1.347
376	Cytidine	Nucleotide	2	AT_H2O2_2_1	AT_H2O2_2	1.0	Pyrimidine nucleosides	True	2.541e+05	1.045	1.045	1.629
41	Lactate	Carbon	2	AT_H2O2_2_1	AT_H2O2_2	1.0	Respiratory carbon sources	True	3.33e+07	8.079	8.079	0.2568
93	3-P-Ser	Amino acid	2	AT_H2O2_2_1	AT_H2O2_2	0.5	Amino acids biosynthesis intermediates	True	1.987e+04	-2.632	-2.632	0.6265
343	Adenine	Nucleotide	2	AT_H2O2_2_1	AT_H2O2_2	0.5	Purine bases	False			-3.45	-1.986
336	Adenosine	Nucleotide	2	AT_H2O2_2_1	AT_H2O2_2	1.0	Purine nucleosides	True	2.341e+05	0.9264	0.9264	-2.211
29	Raffinose	Carbon	2	AT_H2O2_2_1	AT_H2O2_2	0.166666666666667	Sugars and sugar alcohols	False			-5.058	-2.878
717	Nicotinamide	Cofactor	2	AT_H2O2_2_1	AT_H2O2_2	1.0	NAD biosynthesis	True	7.644e+05	2.634	2.634	0.2369
51	PEP	Carbon	2	AT_H2O2_2_1	AT_H2O2_2	1.0	Glycolysis, GNG	True	7.087e+04	-0.7975	-0.7975	0.2745
52	Pyruvate	Carbon	2	AT_H2O2_2_1	AT_H2O2_2	1.0	Glycolysis, GNG	True	2.882e+04	-2.096	-2.096	0.2779
237	Spermine	Amino acid	2	AT_H2O2_2_1	AT_H2O2_2	0.666666666666667	Polyamines	True	4.162e+05	1.756	1.756	-1.632
385	Uracil	Nucleotide	2	AT_H2O2_2_1	AT_H2O2_2	1.0	Pyrimidine bases	True	1.897e+05	0.6229	0.6229	2.35

Metabolite ID	Name	Super Pathway	Dataset	Sample ID	Group ID	Detection Fraction	Pathway	Detected	Raw Intensity	Log2 Norm Intensity	Norm Imputed	Log2 Ctrl Norm
377	Uridine	Nucleotide	2	AT_H2O2_2_1	AT_H2O2_2	1.0	Pyrimidine nucleosides	True	9.529e+05	2.952	2.952	0.06931
112	trans-Urocanate	Amino acid	2	AT_H2O2_2_1	AT_H2O2_2	1.0	Amino acids degradation intermediates	True	1.56e+05	0.3411	0.3411	3.459
737	Pyridoxine (Vitamin B6)	Cofactor	2	AT_H2O2_2_1	AT_H2O2_2	1.0	PLP biosynthesis and salvage	True	1.783e+06	3.856	3.856	0.5557
348	Allantoin	Nucleotide	2	AT_H2O2_2_1	AT_H2O2_2	1.0	Purine degradation	True	2.601e+04	-2.244	-2.244	0.5859
335	Inosine	Nucleotide	2	AT_H2O2_2_1	AT_H2O2_2	1.0	Purine nucleosides	True	4.391e+05	1.834	1.834	-1.027
81	Ile	Amino acid	2	AT_H2O2_2_1	AT_H2O2_2	1.0	Proteinogenic amino acids	True	3.081e+07	7.967	7.967	0.1112
72	Ala	Amino acid	2	AT_H2O2_2_1	AT_H2O2_2	1.0	Proteinogenic amino acids	True	1.806e+07	7.196	7.196	-0.5377
79	Thr	Amino acid	2	AT_H2O2_2_1	AT_H2O2_2	1.0	Proteinogenic amino acids	True	5.405e+06	5.456	5.456	-0.5068
88	Tyr	Amino acid	2	AT_H2O2_2_1	AT_H2O2_2	1.0	Proteinogenic amino acids	True	1.395e+07	6.824	6.824	-0.01881
84	Lys	Amino acid	2	AT_H2O2_2_1	AT_H2O2_2	1.0	Proteinogenic amino acids	True	2.097e+06	4.09	4.09	0.3707
86	Met	Amino acid	2	AT_H2O2_2_1	AT_H2O2_2	1.0	Proteinogenic amino acids	True	5.948e+06	5.594	5.594	-0.1934
61	Malate	Carbon	2	AT_H2O2_2_1	AT_H2O2_2	1.0	TCA cycle	True	2.691e+05	1.127	1.127	0.2305
235	Putrescine	Amino acid	2	AT_H2O2_2_1	AT_H2O2_2	0.0	Polyamines	False			-3.898	-2.331
49	3-P-Glycerate	Carbon	2	AT_H2O2_2_1	AT_H2O2_2	1.0	Glycolysis, GNG	True	8.005e+05	2.7	2.7	0.2376
139	GABA	Amino acid	2	AT_H2O2_2_1	AT_H2O2_2	0.0	Amino acid derivatives	False			-4.963	-1.493
189	Kynurenate	Amino acid	2	AT_H2O2_2_1	AT_H2O2_2	0.833333333333333	Amino acid derivatives	True	1.698e+04	-2.859	-2.859	2.329
234	5-Me-Thioadenosine	Amino acid	2	AT_H2O2_2_1	AT_H2O2_2	1.0	SAM metabolism	True	3.193e+04	-1.948	-1.948	-1.852
59	Succinate	Carbon	2	AT_H2O2_2_1	AT_H2O2_2	0.833333333333333	TCA cycle	False			-2.963	-0.9227
133	Ornithine	Amino acid	2	AT_H2O2_2_1	AT_H2O2_2	1.0	Amino acids degradation intermediates	True	1.033e+06	3.068	3.068	0.5468
313	5-Oxoproline	Amino acid	2	AT_H2O2_2_1	AT_H2O2_2	1.0	Glutathione derivatives	True	1.602e+06	3.701	3.701	1.698
724	Pantothenate	Cofactor	2	AT_H2O2_2_1	AT_H2O2_2	1.0	Coenzyme A biosynthesis	True	1.351e+06	3.455	3.455	0.342
30	Sucrose	Carbon	2	AT_H2O2_2_1	AT_H2O2_2	1.0	Sugars and sugar alcohols	True	7.177e+05	2.543	2.543	0.1659

Metabolite ID	Name	Super Pathway	Dataset	Sample ID	Group ID	Detection Fraction	Pathway	Detected	Raw Intensity	Log2 Norm Intensity	Norm Imputed	Log2 Ctrl Norm
122	3-OH-Isobutyrate	Amino acid	2	AT_H2O2_2_1	AT_H2O2_2	0.5	Amino acids degradation intermediates	True	2.618e+04	-2.234	-2.234	2.394
241	4-Acetamidobutanoate	Amino acid	2	AT_H2O2_2_1	AT_H2O2_2	1.0	Polyamine derivatives	True	1.127e+05	-0.1277	-0.1277	1.38
55	Citrate	Carbon	2	AT_H2O2_2_1	AT_H2O2_2	1.0	TCA cycle	True	1.334e+06	3.437	3.437	0.4022
338	Guanosine	Nucleotide	2	AT_H2O2_2_1	AT_H2O2_2	1.0	Purine nucleosides	True	3.177e+05	1.367	1.367	-0.9937
170	2-Amino-Butyrate	Amino acid	2	AT_H2O2_2_1	AT_H2O2_2	1.0	Amino acid derivatives	True	1.26e+05	0.03318	0.03318	-1.191
209	N-Ac-Ala	Amino acid	2	AT_H2O2_2_1	AT_H2O2_2	0.166666666666667	N-acetylated amino acids	True	8685	-3.826	-3.826	-0.5584
221	N-Ac-Met	Amino acid	2	AT_H2O2_2_1	AT_H2O2_2	1.0	N-acetylated amino acids	True	3.369e+04	-1.87	-1.87	-0.7203
22	N-Ac-Neuraminate	Carbon	2	AT_H2O2_2_1	AT_H2O2_2	0.833333333333333	Aminosugar derivatives	True	1.084e+05	-0.1837	-0.1837	0.0303
346	Urate	Nucleotide	2	AT_H2O2_2_1	AT_H2O2_2	0.833333333333333	Purine degradation	True	2.461e+04	-2.323	-2.323	1.369
90	Arg	Amino acid	2	AT_H2O2_2_1	AT_H2O2_2	1.0	Proteinogenic amino acids	True	3.507e+06	4.832	4.832	0.04957
60	Fumarate	Carbon	2	AT_H2O2_2_1	AT_H2O2_2	1.0	TCA cycle	True	1.669e+05	0.4384	0.4384	0.5951
78	Ser	Amino acid	2	AT_H2O2_2_1	AT_H2O2_2	1.0	Proteinogenic amino acids	True	7.531e+06	5.934	5.934	-0.8926
83	Val	Amino acid	2	AT_H2O2_2_1	AT_H2O2_2	1.0	Proteinogenic amino acids	True	1.944e+07	7.302	7.302	0.06837
734	Pyridoxal	Cofactor	2	AT_H2O2_2_1	AT_H2O2_2	0.666666666666667	PLP biosynthesis and salvage	True	7.391e+04	-0.7369	-0.7369	0.2578
136	Urea	Amino acid	2	AT_H2O2_2_1	AT_H2O2_2	1.0	Amino acids degradation intermediates	True	8.126e+05	2.722	2.722	1.077
742	Folate	Cofactor	2	AT_H2O2_2_1	AT_H2O2_2	1.0	Folate metabolism	True	1.103e+05	-0.1594	-0.1594	0.3576
729	Riboflavin (Vitamin B2)	Cofactor	2	AT_H2O2_2_1	AT_H2O2_2	0.833333333333333	Flavine biosynthesis	True	5.823e+04	-1.081	-1.081	0.4881
91	Pro	Amino acid	2	AT_H2O2_2_1	AT_H2O2_2	1.0	Proteinogenic amino acids	True	7.526e+06	5.933	5.933	-0.7879
308	Glutathione, Reduced	Amino acid	2	AT_H2O2_2_1	AT_H2O2_2	1.0	Glutathione	True	4.404e+06	5.16	5.16	-1.295
706	FAD	Cofactor	2	AT_H2O2_2_1	AT_H2O2_2	0.5	Cofactors	True	1.001e+04	-3.621	-3.621	1.475
299	gamma-Glu-Tyr	Amino acid	2	AT_H2O2_2_1	AT_H2O2_2	0.666666666666667	Gamma-glutamyl dipeptides	True	4.803e+04	-1.359	-1.359	-0.1916
705	Coenzyme A	Cofactor	2	AT_H2O2_2_1	AT_H2O2_2	1.0	Cofactors	True	4211	-4.87	-4.87	-1.164
342	Hypoxanthine	Nucleotide	2	AT_H2O2_2_1	AT_H2O2_2	1.0	Purine bases	True	1.109e+05	-0.1511	-0.1511	-0.153

Metabolite ID	Name	Super Pathway	Dataset	Sample ID	Group ID	Detection Fraction	Pathway	Detected	Raw Intensity	Log2 Norm Intensity	Norm Imputed	Log2 Ctrl Norm
344	Xanthine	Nucleotide	2	AT_H2O2_2_1	AT_H2O2_2	0.833333333333333	Purine bases	True	9.63e+04	-0.355	-0.355	1.884
703	NAD+	Cofactor	2	AT_H2O2_2_1	AT_H2O2_2	1.0	Cofactors	True	5.508e+05	2.161	2.161	-1.398
731	Thiamin (Vitamin B1)	Cofactor	2	AT_H2O2_2_1	AT_H2O2_2	1.0	TPP biosynthesis	True	6.058e+04	-1.024	-1.024	-0.8176
102	2-Aminoadipate	Amino acid	2	AT_H2O2_2_1	AT_H2O2_2	1.0	Amino acids biosynthesis intermediates	True	9.889e+04	-0.3168	-0.3168	-1.073
77	Gly	Amino acid	2	AT_H2O2_2_1	AT_H2O2_2	1.0	Proteinogenic amino acids	True	1.302e+07	6.724	6.724	-0.5038
45	Fructose-6-P	Carbon	2	AT_H2O2_2_1	AT_H2O2_2	0.833333333333333	Glycolysis, GNG	True	1.204e+05	-0.03318	-0.03318	1.402
36	Ribose	Carbon	2	AT_H2O2_2_1	AT_H2O2_2	0.666666666666667	Sugars and sugar alcohols	True	5.803e+04	-1.086	-1.086	-0.08842
4	GlcNAc 6-P	Carbon	2	AT_H2O2_2_1	AT_H2O2_2	1.0	Aminosugar biosynthesis	True	2.712e+05	1.139	1.139	0.7792
188	Kynurenine	Amino acid	2	AT_H2O2_2_1	AT_H2O2_2	0.833333333333333	Amino acid derivatives	True	1.689e+05	0.4552	0.4552	0.4692
63	6-P-Gluconate	Carbon	2	AT_H2O2_2_1	AT_H2O2_2	1.0	Pentose phosphate pathway (PPP)	True	7.276e+04	-0.7595	-0.7595	0.2857
710	Carnitine	Cofactor	2	AT_H2O2_2_1	AT_H2O2_2	1.0	Cofactors	True	3.983e+05	1.693	1.693	-0.8384
725	P-Pantetheine	Cofactor	2	AT_H2O2_2_1	AT_H2O2_2	0.833333333333333	Coenzyme A biosynthesis	True	6934	-4.151	-4.151	0.6616
110	N-alpha-Ac-Ornithine	Amino acid	2	AT_H2O2_2_1	AT_H2O2_2	1.0	Amino acids biosynthesis intermediates	True	1.987e+05	0.6898	0.6898	-0.2842
116	3-Me-2-Oxo-Valerate	Amino acid	2	AT_H2O2_2_1	AT_H2O2_2	1.0	Amino acids degradation intermediates	True	6.373e+04	-0.9506	-0.9506	2.624
155	4-Guanidinobutanoate	Amino acid	2	AT_H2O2_2_1	AT_H2O2_2	0.666666666666667	Amino acid derivatives	True	2.46e+04	-2.324	-2.324	-1.604
310	S-Lactoyl-Glutathione	Amino acid	2	AT_H2O2_2_1	AT_H2O2_2	0.166666666666667	Glutathione derivatives	False			-4.634	-0.962
34	Ribitol	Carbon	2	AT_H2O2_2_1	AT_H2O2_2	1.0	Sugars and sugar alcohols	True	8.643e+04	-0.511	-0.511	1.068
707	FMN	Cofactor	2	AT_H2O2_2_1	AT_H2O2_2	0.666666666666667	Cofactors	True	6084	-4.34	-4.34	-0.9188
17	Maltose	Carbon	2	AT_H2O2_2_1	AT_H2O2_2	1.0	Glycogen degradation	True	2.612e+05	1.085	1.085	1.124
18	Maltotriose	Carbon	2	AT_H2O2_2_1	AT_H2O2_2	1.0	Glycogen degradation	True	1.279e+06	3.377	3.377	1.948
19	Maltotetraose	Carbon	2	AT_H2O2_2_1	AT_H2O2_2	1.0	Glycogen degradation	True	1.45e+05	0.2354	0.2354	0.4258

Metabolite ID	Name	Super Pathway	Dataset	Sample ID	Group ID	Detection Fraction	Pathway	Detected	Raw Intensity	Log2 Norm Intensity	Norm Imputed	Log2 Ctrl Norm
232	SAH	Amino acid	2	AT_H2O2_2_1	AT_H2O2_2	1.0	SAM metabolism	True	1.442e+04	-3.094	-3.094	0.1318
74	Asp	Amino acid	2	AT_H2O2_2_1	AT_H2O2_2	1.0	Proteinogenic amino acids	True	2.8e+06	4.507	4.507	-1.153
129	5-Aminovalerate	Amino acid	2	AT_H2O2_2_1	AT_H2O2_2	0.666666666666667	Amino acids degradation intermediates	True	6.984e+04	-0.8186	-0.8186	-5.57e-03
254	Gly-Val	Amino acid	2	AT_H2O2_2_1	AT_H2O2_2	1.0	Dipeptides	True	1.055e+05	-0.2233	-0.2233	-0.1495
291	gamma-Glu-Leu	Amino acid	2	AT_H2O2_2_1	AT_H2O2_2	0.833333333333333	Gamma-glutamyl dipeptides	True	8.63e+04	-0.5133	-0.5133	0.5687
173	Met Sulfoxide	Amino acid	2	AT_H2O2_2_1	AT_H2O2_2	1.0	Amino acid derivatives	True	2.891e+05	1.231	1.231	1.369
43	Glucose	Carbon	2	AT_H2O2_2_1	AT_H2O2_2	1.0	Glycolysis, GNG	True	2.353e+07	7.578	7.578	0.7579
249	Gly-Gly	Amino acid	2	AT_H2O2_2_1	AT_H2O2_2	0.666666666666667	Dipeptides	True	6.244e+04	-0.9801	-0.9801	-1.197
169	2-OH-Butyrate	Amino acid	2	AT_H2O2_2_1	AT_H2O2_2	0.833333333333333	Amino acid derivatives	True	7.246e+04	-0.7654	-0.7654	0.6685
98	3-Methyl-2-Oxobutyrate	Amino acid	2	AT_H2O2_2_1	AT_H2O2_2	0.666666666666667	Amino acids biosynthesis intermediates	True	3.048e+04	-2.015	-2.015	1.744
100	4-Me-2-Oxo-Pentanoate	Amino acid	2	AT_H2O2_2_1	AT_H2O2_2	1.0	Amino acids biosynthesis intermediates	True	5.462e+04	-1.173	-1.173	2.129
253	Gly-Pro	Amino acid	2	AT_H2O2_2_1	AT_H2O2_2	0.833333333333333	Dipeptides	True	7.044e+04	-0.8062	-0.8062	-0.4851
247	Asp-Phe	Amino acid	2	AT_H2O2_2_1	AT_H2O2_2	1.0	Dipeptides	True	4.58e+04	-1.427	-1.427	-0.486
212	N-Ac-Asp	Amino acid	2	AT_H2O2_2_1	AT_H2O2_2	0.5	N-acetylated amino acids	True	8704	-3.823	-3.823	0.6795
720	1-Me-Nicotinamide	Cofactor	2	AT_H2O2_2_1	AT_H2O2_2	1.0	Derivatives of NA, nicotinamide and NAD	True	1.62e+06	3.717	3.717	0.01383
70	Creatine	Carbon	2	AT_H2O2_2_1	AT_H2O2_2	1.0	Creatine energy storage	True	6.269e+06	5.67	5.67	-0.1109
309	Glutathione, Oxidized	Amino acid	2	AT_H2O2_2_1	AT_H2O2_2	1.0	Glutathione	True	1.486e+06	3.593	3.593	-0.3557
44	Glucose 6-P	Carbon	2	AT_H2O2_2_1	AT_H2O2_2	1.0	Glycolysis, GNG	True	3.057e+05	1.311	1.311	0.4966
24	Fructose	Carbon	2	AT_H2O2_2_1	AT_H2O2_2	1.0	Sugars and sugar alcohols	True	4.25e+06	5.109	5.109	1.748
85	Cys	Amino acid	2	AT_H2O2_2_1	AT_H2O2_2	1.0	Proteinogenic amino acids	True	2.537e+05	1.043	1.043	1.635
704	NADH	Cofactor	2	AT_H2O2_2_1	AT_H2O2_2	0.166666666666667	Cofactors	False			-4.313	-2.383

Metabolite ID	Name	Super Pathway	Dataset	Sample ID	Group ID	Detection Fraction	Pathway	Detected	Raw Intensity	Log2 Norm Intensity	Norm Imputed	Log2 Ctrl Norm
275	Thr-Phe	Amino acid	2	AT_H2O2_2_1	AT_H2O2_2	0.666666666666667	Dipeptides	True	3.899e+04	-1.659	-1.659	-0.7259
738	Pyridoxate	Cofactor	2	AT_H2O2_2_1	AT_H2O2_2	1.0	PLP biosynthesis and salvage	True	1.461e+04	-3.076	-3.076	1.589
177	3-(4-OH-Phenyl)Lactate	Amino acid	2	AT_H2O2_2_1	AT_H2O2_2	1.0	Amino acid derivatives	True	1.678e+04	-2.876	-2.876	1.077
206	Trans-4-OH-Pro	Amino acid	2	AT_H2O2_2_1	AT_H2O2_2	1.0	Amino acid derivatives	True	2.872e+05	1.221	1.221	1.417
329	AMP	Nucleotide	2	AT_H2O2_2_1	AT_H2O2_2	0.5	Purine nucleotides	True	1.971e+04	-2.644	-2.644	-3.064
345	Guanine	Nucleotide	2	AT_H2O2_2_1	AT_H2O2_2	1.0	Purine bases	True	5.37e+05	2.124	2.124	0.5422
271	pyroGlu-Val	Amino acid	2	AT_H2O2_2_1	AT_H2O2_2	0.666666666666667	Dipeptides	True	1.06e+04	-3.538	-3.538	0.1035
279	Val-Glu	Amino acid	2	AT_H2O2_2_1	AT_H2O2_2	0.666666666666667	Dipeptides	True	3.031e+04	-2.023	-2.023	-1.193
183	Phenol Sulfate	Amino acid	2	AT_H2O2_2_1	AT_H2O2_2	0.833333333333333	Amino acid derivatives	True	2.623e+05	1.091	1.091	4.818
740	3-Dehydrocarnitine	Cofactor	2	AT_H2O2_2_1	AT_H2O2_2	0.5	Carnitine biosynthesis	True	9.24e+04	-0.4148	-0.4148	-0.08123
145	Pyro-Gln	Amino acid	2	AT_H2O2_2_1	AT_H2O2_2	1.0	Amino acid derivatives	True	1.617e+05	0.3923	0.3923	0.04631
197	C-Glycosyl-Trp	Amino acid	2	AT_H2O2_2_1	AT_H2O2_2	1.0	Amino acid derivatives	True	2.01e+05	0.7066	0.7066	0.8503
718	Nicotinamide Riboside	Cofactor	2	AT_H2O2_2_1	AT_H2O2_2	0.5	NAD biosynthesis	False			-2.306	-0.5023
295	gamma-Glu-Phe	Amino acid	2	AT_H2O2_2_1	AT_H2O2_2	0.666666666666667	Gamma-glutamyl dipeptides	True	5.285e+04	-1.221	-1.221	-1.119
399	Pseudouridine	Nucleotide	2	AT_H2O2_2_1	AT_H2O2_2	1.0	Pyrimidine derivatives in RNAs	True	3.749e+04	-1.716	-1.716	2.525
375	UTP	Nucleotide	2	AT_H2O2_2_1	AT_H2O2_2	0.0	Pyrimidine nucleotides	False			-5.966	-0.5235
20	Erythronate	Carbon	2	AT_H2O2_2_1	AT_H2O2_2	1.0	Aminosugar derivatives	True	7.13e+04	-0.7887	-0.7887	0.6529
151	Phenylacetylglycine	Amino acid	2	AT_H2O2_2_1	AT_H2O2_2	1.0	Amino acid derivatives	True	4.133e+05	1.746	1.746	6.168
252	Gly-Phe	Amino acid	2	AT_H2O2_2_1	AT_H2O2_2	0.666666666666667	Dipeptides	True	1.157e+05	-0.09005	-0.09005	-0.1942
251	Gly-Leu	Amino acid	2	AT_H2O2_2_1	AT_H2O2_2	1.0	Dipeptides	True	1.64e+05	0.4133	0.4133	0.05069
290	gamma-Glu-Ile	Amino acid	2	AT_H2O2_2_1	AT_H2O2_2	0.666666666666667	Gamma-glutamyl dipeptides	True	4.592e+04	-1.423	-1.423	0.1078
316	Ophthalmate	Amino acid	2	AT_H2O2_2_1	AT_H2O2_2	1.0	Oxidative stress markers	True	9.765e+04	-0.335	-0.335	-0.5892

Metabolite ID	Name	Super Pathway	Dataset	Sample ID	Group ID	Detection Fraction	Pathway	Detected	Raw Intensity	Log2 Norm Intensity	Norm Imputed	Log2 Ctrl Norm
208	Pro-OH-Pro	Amino acid	2	AT_H2O2_2_1	AT_H2O2_2	1.0	Amino acid derivatives	True	1.914e+05	0.6359	0.6359	0.1762
352	3'-AMP	Nucleotide	2	AT_H2O2_2_1	AT_H2O2_2	1.0	Purine derivatives in signaling	True	5.01e+04	-1.298	-1.298	-0.01296
314	Cys-Glutathione Disulfide	Amino acid	2	AT_H2O2_2_1	AT_H2O2_2	1.0	Oxidative stress markers	True	1.062e+05	-0.2134	-0.2134	1.152
39	Threitol	Carbon	2	AT_H2O2_2_1	AT_H2O2_2	0.5	Sugars and sugar alcohols	False			-4.395	-0.3698
31	Ribulose/Xylulose	Carbon	2	AT_H2O2_2_1	AT_H2O2_2	0.0	Sugars and sugar alcohols	False			-5.004	-0.09716
48	DHAP	Carbon	2	AT_H2O2_2_1	AT_H2O2_2	1.0	Glycolysis, GNG	True	1.546e+05	0.3275	0.3275	-1.431
182	P-Cresol Sulfate	Amino acid	2	AT_H2O2_2_1	AT_H2O2_2	1.0	Amino acid derivatives	True	5.334e+04	-1.207	-1.207	2.13
250	Gly-Ile	Amino acid	2	AT_H2O2_2_1	AT_H2O2_2	0.666666666666667	Dipeptides	True	1.061e+05	-0.2147	-0.2147	1.209
286	gamma-Glu-Glu	Amino acid	2	AT_H2O2_2_1	AT_H2O2_2	1.0	Gamma-glutamyl dipeptides	True	4.99e+04	-1.304	-1.304	-1.073
264	Leu-Leu	Amino acid	2	AT_H2O2_2_1	AT_H2O2_2	0.5	Dipeptides	True	4.65e+04	-1.405	-1.405	-0.3321
203	DiMe-Arg	Amino acid	2	AT_H2O2_2_1	AT_H2O2_2	1.0	Amino acid derivatives	True	3.972e+05	1.689	1.689	0.6148
47	Fructose 1,6-PP, Glucose 1,6-PP	Carbon	2	AT_H2O2_2_1	AT_H2O2_2	1.0	Glycolysis, GNG	True	3.762e+04	-1.711	-1.711	-1.421
224	N-Ac-Ser	Amino acid	2	AT_H2O2_2_1	AT_H2O2_2	1.0	N-acetylated amino acids	True	1.344e+05	0.1255	0.1255	-0.7452
244	Ala-Leu	Amino acid	2	AT_H2O2_2_1	AT_H2O2_2	0.166666666666667	Dipeptides	False			-1.778	-2.118
304	Cyclo(Phe-Pro)	Amino acid	2	AT_H2O2_2_1	AT_H2O2_2	0.0	Cyclic dipeptides	False			-1.192	-0.7201
302	Cyclo(Glu-Glu)	Amino acid	2	AT_H2O2_2_1	AT_H2O2_2	0.666666666666667	Cyclic dipeptides	True	6.256e+04	-0.9773	-0.9773	0.1949
303	Cyclo(Leu-Pro)	Amino acid	2	AT_H2O2_2_1	AT_H2O2_2	0.0	Cyclic dipeptides	False			-0.4297	-0.7026
390	2',3'-cUMP	Nucleotide	2	AT_H2O2_2_1	AT_H2O2_2	1.0	Pyrimidine derivatives in signaling	True	3.744e+04	-1.718	-1.718	-0.5893
68	Ribulose 5-P / Xylulose 5-P	Carbon	2	AT_H2O2_2_1	AT_H2O2_2	1.0	Pentose phosphate pathway (PPP)	True	8.356e+04	-0.5599	-0.5599	-1.329
388	2',3'-cCMP	Nucleotide	2	AT_H2O2_2_1	AT_H2O2_2	1.0	Pyrimidine derivatives in signaling	True	6.222e+04	-0.9852	-0.9852	-1.239
33	Arabitol/Xylitol	Carbon	2	AT_H2O2_2_1	AT_H2O2_2	0.166666666666667	Sugars and sugar alcohols	False			-3.559	-0.05408
268	Phe-Phe	Amino acid	2	AT_H2O2_2_1	AT_H2O2_2	0.0	Dipeptides	False			-2.762	-1.674

Metabolite ID	Name	Super Pathway	Dataset	Sample ID	Group ID	Detection Fraction	Pathway	Detected	Raw Intensity	Log2 Norm Intensity	Norm Imputed	Log2 Ctrl Norm
245	Ala-Phe	Amino acid	2	AT_H2O2_2_1	AT_H2O2_2	0.666666666666667	Dipeptides	True	1.899e+04	-2.697	-2.697	-1.242
373	UMP	Nucleotide	2	AT_H2O2_2_1	AT_H2O2_2	0.0	Pyrimidine nucleotides	False			-3.442	-1.402
282	Val-Leu	Amino acid	2	AT_H2O2_2_1	AT_H2O2_2	0.666666666666667	Dipeptides	True	1.135e+05	-0.1185	-0.1185	0.2503
258	Ile-Gly	Amino acid	2	AT_H2O2_2_1	AT_H2O2_2	0.666666666666667	Dipeptides	False			-2.663	-3.077
259	Ile-Ser	Amino acid	2	AT_H2O2_2_1	AT_H2O2_2	0.0	Dipeptides	False			-3.192	-2.329
269	Phe-Ser	Amino acid	2	AT_H2O2_2_1	AT_H2O2_2	0.333333333333333	Dipeptides	True	3.773e+04	-1.707	-1.707	-0.3921
277	Tyr-Ala	Amino acid	2	AT_H2O2_2_1	AT_H2O2_2	0.833333333333333	Dipeptides	True	9.122e+04	-0.4333	-0.4333	-1.003
257	Ile-Gln	Amino acid	2	AT_H2O2_2_1	AT_H2O2_2	0.166666666666667	Dipeptides	True	2.132e+04	-2.53	-2.53	-1.611
261	Leu-Glu	Amino acid	2	AT_H2O2_2_1	AT_H2O2_2	1.0	Dipeptides	True	7.001e+04	-0.8152	-0.8152	-1.605
263	Leu-Gly	Amino acid	2	AT_H2O2_2_1	AT_H2O2_2	1.0	Dipeptides	True	7.673e+04	-0.6828	-0.6828	-1.39
256	Ile-Ala	Amino acid	2	AT_H2O2_2_1	AT_H2O2_2	0.666666666666667	Dipeptides	True	7.328e+04	-0.7492	-0.7492	-0.7075
274	Thr-Leu	Amino acid	2	AT_H2O2_2_1	AT_H2O2_2	0.833333333333333	Dipeptides	True	2.274e+05	0.8844	0.8844	0.03327
273	Ser-Phe	Amino acid	2	AT_H2O2_2_1	AT_H2O2_2	0.833333333333333	Dipeptides	True	2.987e+04	-2.044	-2.044	-0.8219
272	Ser-Leu	Amino acid	2	AT_H2O2_2_1	AT_H2O2_2	1.0	Dipeptides	True	1.464e+05	0.2491	0.2491	-0.4897
246	Asp-Leu	Amino acid	2	AT_H2O2_2_1	AT_H2O2_2	1.0	Dipeptides	True	9.683e+04	-0.3472	-0.3472	-0.2731
76	Gln	Amino acid	2	AT_H2O2_2_2	AT_H2O2_2	1.0	Proteinogenic amino acids	True	8.986e+06	6.498	6.498	-0.4541
89	Trp	Amino acid	2	AT_H2O2_2_2	AT_H2O2_2	1.0	Proteinogenic amino acids	True	4.743e+06	5.576	5.576	-0.3906
723	beta-Ala	Cofactor	2	AT_H2O2_2_2	AT_H2O2_2	1.0	Coenzyme A biosynthesis	True	3.518e+04	-1.499	-1.499	-1.825
75	Glu	Amino acid	2	AT_H2O2_2_2	AT_H2O2_2	1.0	Proteinogenic amino acids	True	5.724e+06	5.847	5.847	-0.1005
80	His	Amino acid	2	AT_H2O2_2_2	AT_H2O2_2	1.0	Proteinogenic amino acids	True	1.002e+05	0.01101	0.01101	-0.2031
82	Leu	Amino acid	2	AT_H2O2_2_2	AT_H2O2_2	1.0	Proteinogenic amino acids	True	2.581e+07	8.02	8.02	-0.1403
87	Phe	Amino acid	2	AT_H2O2_2_2	AT_H2O2_2	1.0	Proteinogenic amino acids	True	2.053e+07	7.69	7.69	-0.2017
236	Spermidine	Amino acid	2	AT_H2O2_2_2	AT_H2O2_2	1.0	Polyamines	True	1.815e+05	0.868	0.868	-1.96

Metabolite ID	Name	Super Pathway	Dataset	Sample ID	Group ID	Detection Fraction	Pathway	Detected	Raw Intensity	Log2 Norm Intensity	Norm Imputed	Log2 Ctrl Norm
73	Asn	Amino acid	2	AT_H2O2_2_2	AT_H2O2_2	1.0	Proteinogenic amino acids	True	2.354e+05	1.243	1.243	-1.506
243	Creatinine	Amino acid	2	AT_H2O2_2_2	AT_H2O2_2	1.0	Creatine degradation	True	8.393e+05	3.077	3.077	1.507
376	Cytidine	Nucleotide	2	AT_H2O2_2_2	AT_H2O2_2	1.0	Pyrimidine nucleosides	True	6.106e+05	2.618	2.618	3.203
41	Lactate	Carbon	2	AT_H2O2_2_2	AT_H2O2_2	1.0	Respiratory carbon sources	True	2.609e+07	8.036	8.036	0.2138
93	3-P-Ser	Amino acid	2	AT_H2O2_2_2	AT_H2O2_2	0.5	Amino acids biosynthesis intermediates	True	9506	-3.387	-3.387	-0.1279
343	Adenine	Nucleotide	2	AT_H2O2_2_2	AT_H2O2_2	0.5	Purine bases	True	2.007e+04	-2.309	-2.309	-0.845
336	Adenosine	Nucleotide	2	AT_H2O2_2_2	AT_H2O2_2	1.0	Purine nucleosides	True	2.981e+05	1.584	1.584	-1.553
29	Raffinose	Carbon	2	AT_H2O2_2_2	AT_H2O2_2	0.166666666666667	Sugars and sugar alcohols	False			-5.058	-2.878
717	Nicotinamide	Cofactor	2	AT_H2O2_2_2	AT_H2O2_2	1.0	NAD biosynthesis	True	8.972e+05	3.174	3.174	0.7769
51	PEP	Carbon	2	AT_H2O2_2_2	AT_H2O2_2	1.0	Glycolysis, GNG	True	6.955e+04	-0.5156	-0.5156	0.5564
52	Pyruvate	Carbon	2	AT_H2O2_2_2	AT_H2O2_2	1.0	Glycolysis, GNG	True	1.73e+04	-2.523	-2.523	-0.1494
237	Spermine	Amino acid	2	AT_H2O2_2_2	AT_H2O2_2	0.666666666666667	Polyamines	True	2.372e+05	1.254	1.254	-2.134
385	Uracil	Nucleotide	2	AT_H2O2_2_2	AT_H2O2_2	1.0	Pyrimidine bases	True	5.593e+04	-0.8301	-0.8301	0.8968
377	Uridine	Nucleotide	2	AT_H2O2_2_2	AT_H2O2_2	1.0	Pyrimidine nucleosides	True	4.232e+05	2.09	2.09	-0.7927
112	trans-Urocanate	Amino acid	2	AT_H2O2_2_2	AT_H2O2_2	1.0	Amino acids degradation intermediates	True	1.261e+05	0.3431	0.3431	3.461
737	Pyridoxine (Vitamin B6)	Cofactor	2	AT_H2O2_2_2	AT_H2O2_2	1.0	PLP biosynthesis and salvage	True	1.56e+06	3.972	3.972	0.6717
348	Allantoin	Nucleotide	2	AT_H2O2_2_2	AT_H2O2_2	1.0	Purine degradation	True	3.777e+04	-1.397	-1.397	1.433
335	Inosine	Nucleotide	2	AT_H2O2_2_2	AT_H2O2_2	1.0	Purine nucleosides	True	2.319e+05	1.222	1.222	-1.64
81	Ile	Amino acid	2	AT_H2O2_2_2	AT_H2O2_2	1.0	Proteinogenic amino acids	True	2.295e+07	7.851	7.851	-4.78e-03
72	Ala	Amino acid	2	AT_H2O2_2_2	AT_H2O2_2	1.0	Proteinogenic amino acids	True	1.147e+07	6.85	6.85	-0.8837
79	Thr	Amino acid	2	AT_H2O2_2_2	AT_H2O2_2	1.0	Proteinogenic amino acids	True	2.399e+06	4.593	4.593	-1.37
88	Tyr	Amino acid	2	AT_H2O2_2_2	AT_H2O2_2	1.0	Proteinogenic amino acids	True	1.042e+07	6.711	6.711	-0.1314
84	Lys	Amino acid	2	AT_H2O2_2_2	AT_H2O2_2	1.0	Proteinogenic amino acids	True	1.733e+06	4.124	4.124	0.4044

Metabolite ID	Name	Super Pathway	Dataset	Sample ID	Group ID	Detection Fraction	Pathway	Detected	Raw Intensity	Log2 Norm Intensity	Norm Imputed	Log2 Ctrl Norm
86	Met	Amino acid	2	AT_H2O2_2_2	AT_H2O2_2	1.0	Proteinogenic amino acids	True	3.922e+06	5.302	5.302	-0.4852
61	Malate	Carbon	2	AT_H2O2_2_2	AT_H2O2_2	1.0	TCA cycle	True	2.029e+05	1.029	1.029	0.132
235	Putrescine	Amino acid	2	AT_H2O2_2_2	AT_H2O2_2	0.0	Polyamines	False			-3.898	-2.331
49	3-P-Glycerate	Carbon	2	AT_H2O2_2_2	AT_H2O2_2	1.0	Glycolysis, GNG	True	5.091e+05	2.356	2.356	-0.1065
139	GABA	Amino acid	2	AT_H2O2_2_2	AT_H2O2_2	0.0	Amino acid derivatives	False			-4.963	-1.493
189	Kynurenate	Amino acid	2	AT_H2O2_2_2	AT_H2O2_2	0.833333333333333	Amino acid derivatives	False			-5.188	0
234	5-Me-Thioadenosine	Amino acid	2	AT_H2O2_2_2	AT_H2O2_2	1.0	SAM metabolism	True	3.219e+04	-1.627	-1.627	-1.531
59	Succinate	Carbon	2	AT_H2O2_2_2	AT_H2O2_2	0.833333333333333	TCA cycle	True	3.823e+04	-1.379	-1.379	0.6609
133	Ornithine	Amino acid	2	AT_H2O2_2_2	AT_H2O2_2	1.0	Amino acids degradation intermediates	True	4.952e+05	2.316	2.316	-0.205
313	5-Oxoproline	Amino acid	2	AT_H2O2_2_2	AT_H2O2_2	1.0	Glutathione derivatives	True	1.492e+06	3.908	3.908	1.904
724	Pantothenate	Cofactor	2	AT_H2O2_2_2	AT_H2O2_2	1.0	Coenzyme A biosynthesis	True	1.105e+06	3.474	3.474	0.3608
30	Sucrose	Carbon	2	AT_H2O2_2_2	AT_H2O2_2	1.0	Sugars and sugar alcohols	True	3.913e+05	1.976	1.976	-0.4002
122	3-OH-Isobutyrate	Amino acid	2	AT_H2O2_2_2	AT_H2O2_2	0.5	Amino acids degradation intermediates	False			-4.628	0
241	4-Acetamidobutanoate	Amino acid	2	AT_H2O2_2_2	AT_H2O2_2	1.0	Polyamine derivatives	True	1.022e+05	0.04027	0.04027	1.548
55	Citrate	Carbon	2	AT_H2O2_2_2	AT_H2O2_2	1.0	TCA cycle	True	5.603e+05	2.494	2.494	-0.54
338	Guanosine	Nucleotide	2	AT_H2O2_2_2	AT_H2O2_2	1.0	Purine nucleosides	True	3.904e+05	1.973	1.973	-0.3874
170	2-Amino-Butyrate	Amino acid	2	AT_H2O2_2_2	AT_H2O2_2	1.0	Amino acid derivatives	True	2.764e+05	1.475	1.475	0.2512
209	N-Ac-Ala	Amino acid	2	AT_H2O2_2_2	AT_H2O2_2	0.166666666666667	N-acetylated amino acids	False			-4.401	-1.133
221	N-Ac-Met	Amino acid	2	AT_H2O2_2_2	AT_H2O2_2	1.0	N-acetylated amino acids	True	2.624e+04	-1.922	-1.922	-0.7715
22	N-Ac-Neuraminate	Carbon	2	AT_H2O2_2_2	AT_H2O2_2	0.833333333333333	Aminosugar derivatives	False			-1.69	-1.476
346	Urate	Nucleotide	2	AT_H2O2_2_2	AT_H2O2_2	0.833333333333333	Purine degradation	True	1.499e+04	-2.729	-2.729	0.9627
90	Arg	Amino acid	2	AT_H2O2_2_2	AT_H2O2_2	1.0	Proteinogenic amino acids	True	3.209e+06	5.013	5.013	0.2305

Metabolite ID	Name	Super Pathway	Dataset	Sample ID	Group ID	Detection Fraction	Pathway	Detected	Raw Intensity	Log2 Norm Intensity	Norm Imputed	Log2 Ctrl Norm
60	Fumarate	Carbon	2	AT_H2O2_2_2	AT_H2O2_2	1.0	TCA cycle	True	7.546e+04	-0.3979	-0.3979	-0.2412
78	Ser	Amino acid	2	AT_H2O2_2_2	AT_H2O2_2	1.0	Proteinogenic amino acids	True	5.453e+06	5.777	5.777	-1.049
83	Val	Amino acid	2	AT_H2O2_2_2	AT_H2O2_2	1.0	Proteinogenic amino acids	True	1.657e+07	7.381	7.381	0.1468
734	Pyridoxal	Cofactor	2	AT_H2O2_2_2	AT_H2O2_2	0.666666666666667	PLP biosynthesis and salvage	True	4.008e+04	-1.311	-1.311	-0.3161
136	Urea	Amino acid	2	AT_H2O2_2_2	AT_H2O2_2	1.0	Amino acids degradation intermediates	True	9.164e+05	3.204	3.204	1.559
742	Folate	Cofactor	2	AT_H2O2_2_2	AT_H2O2_2	1.0	Folate metabolism	True	1.119e+05	0.1702	0.1702	0.6872
729	Riboflavin (Vitamin B2)	Cofactor	2	AT_H2O2_2_2	AT_H2O2_2	0.833333333333333	Flavine biosynthesis	True	3.965e+04	-1.326	-1.326	0.2429
91	Pro	Amino acid	2	AT_H2O2_2_2	AT_H2O2_2	1.0	Proteinogenic amino acids	True	5.661e+06	5.831	5.831	-0.8897
308	Glutathione, Reduced	Amino acid	2	AT_H2O2_2_2	AT_H2O2_2	1.0	Glutathione	True	2.189e+06	4.46	4.46	-1.995
706	FAD	Cofactor	2	AT_H2O2_2_2	AT_H2O2_2	0.5	Cofactors	True	6606	-3.912	-3.912	1.184
299	gamma-Glu-Tyr	Amino acid	2	AT_H2O2_2_2	AT_H2O2_2	0.666666666666667	Gamma-glutamyl dipeptides	False			-3.049	-1.881
705	Coenzyme A	Cofactor	2	AT_H2O2_2_2	AT_H2O2_2	1.0	Cofactors	True	863.3	-6.848	-6.848	-3.141
342	Hypoxanthine	Nucleotide	2	AT_H2O2_2_2	AT_H2O2_2	1.0	Purine bases	True	8.062e+04	-0.3026	-0.3026	-0.3045
344	Xanthine	Nucleotide	2	AT_H2O2_2_2	AT_H2O2_2	0.833333333333333	Purine bases	False			-3.616	-1.376
703	NAD+	Cofactor	2	AT_H2O2_2_2	AT_H2O2_2	1.0	Cofactors	True	2.519e+05	1.341	1.341	-2.218
731	Thiamin (Vitamin B1)	Cofactor	2	AT_H2O2_2_2	AT_H2O2_2	1.0	TPP biosynthesis	True	9.867e+04	-0.01101	-0.01101	0.1951
102	2-Aminoadipate	Amino acid	2	AT_H2O2_2_2	AT_H2O2_2	1.0	Amino acids biosynthesis intermediates	True	6.216e+04	-0.6777	-0.6777	-1.434
77	Gly	Amino acid	2	AT_H2O2_2_2	AT_H2O2_2	1.0	Proteinogenic amino acids	True	6.253e+06	5.975	5.975	-1.254
45	Fructose-6-P	Carbon	2	AT_H2O2_2_2	AT_H2O2_2	0.833333333333333	Glycolysis, GNG	True	4.574e+04	-1.12	-1.12	0.3151
36	Ribose	Carbon	2	AT_H2O2_2_2	AT_H2O2_2	0.666666666666667	Sugars and sugar alcohols	True	2.334e+04	-2.091	-2.091	-1.093
4	GlcNAc 6-P	Carbon	2	AT_H2O2_2_2	AT_H2O2_2	1.0	Aminosugar biosynthesis	True	3.95e+04	-1.332	-1.332	-1.691
188	Kynurenine	Amino acid	2	AT_H2O2_2_2	AT_H2O2_2	0.833333333333333	Amino acid derivatives	True	1.117e+05	0.1681	0.1681	0.182
63	6-P-Gluconate	Carbon	2	AT_H2O2_2_2	AT_H2O2_2	1.0	Pentose phosphate pathway (PPP)	True	4.98e+04	-0.9976	-0.9976	0.04758

Metabolite ID	Name	Super Pathway	Dataset	Sample ID	Group ID	Detection Fraction	Pathway	Detected	Raw Intensity	Log2 Norm Intensity	Norm Imputed	Log2 Ctrl Norm
710	Carnitine	Cofactor	2	AT_H2O2_2_2	AT_H2O2_2	1.0	Cofactors	True	1.922e+05	0.9505	0.9505	-1.581
725	P-Pantetheine	Cofactor	2	AT_H2O2_2_2	AT_H2O2_2	0.833333333333333	Coenzyme A biosynthesis	True	6287	-3.983	-3.983	0.8293
110	N-alpha-Ac-Ornithine	Amino acid	2	AT_H2O2_2_2	AT_H2O2_2	1.0	Amino acids biosynthesis intermediates	True	1.551e+05	0.6415	0.6415	-0.3325
116	3-Me-2-Oxo-Valerate	Amino acid	2	AT_H2O2_2_2	AT_H2O2_2	1.0	Amino acids degradation intermediates	True	3.747e+04	-1.408	-1.408	2.167
155	4-Guanidinobutanoate	Amino acid	2	AT_H2O2_2_2	AT_H2O2_2	0.666666666666667	Amino acid derivatives	False			-2.324	-1.604
310	S-Lactoyl-Glutathione	Amino acid	2	AT_H2O2_2_2	AT_H2O2_2	0.166666666666667	Glutathione derivatives	False			-4.634	-0.962
34	Ribitol	Carbon	2	AT_H2O2_2_2	AT_H2O2_2	1.0	Sugars and sugar alcohols	True	3.137e+04	-1.664	-1.664	-0.0856
707	FMN	Cofactor	2	AT_H2O2_2_2	AT_H2O2_2	0.666666666666667	Cofactors	True	4420	-4.491	-4.491	-1.071
17	Maltose	Carbon	2	AT_H2O2_2_2	AT_H2O2_2	1.0	Glycogen degradation	True	1.455e+05	0.549	0.549	0.5884
18	Maltotriose	Carbon	2	AT_H2O2_2_2	AT_H2O2_2	1.0	Glycogen degradation	True	7.434e+05	2.902	2.902	1.474
19	Maltotetraose	Carbon	2	AT_H2O2_2_2	AT_H2O2_2	1.0	Glycogen degradation	True	2.112e+05	1.087	1.087	1.277
232	SAH	Amino acid	2	AT_H2O2_2_2	AT_H2O2_2	1.0	SAM metabolism	True	1.327e+04	-2.906	-2.906	0.3202
74	Asp	Amino acid	2	AT_H2O2_2_2	AT_H2O2_2	1.0	Proteinogenic amino acids	True	1.483e+06	3.899	3.899	-1.761
129	5-Aminovalerate	Amino acid	2	AT_H2O2_2_2	AT_H2O2_2	0.666666666666667	Amino acids degradation intermediates	True	6.538e+04	-0.6047	-0.6047	0.2084
254	Gly-Val	Amino acid	2	AT_H2O2_2_2	AT_H2O2_2	1.0	Dipeptides	True	6.451e+04	-0.6242	-0.6242	-0.5504
291	gamma-Glu-Leu	Amino acid	2	AT_H2O2_2_2	AT_H2O2_2	0.833333333333333	Gamma-glutamyl dipeptides	True	4.655e+04	-1.095	-1.095	-0.01277
173	Met Sulfoxide	Amino acid	2	AT_H2O2_2_2	AT_H2O2_2	1.0	Amino acid derivatives	True	1.45e+05	0.5446	0.5446	0.6834
43	Glucose	Carbon	2	AT_H2O2_2_2	AT_H2O2_2	1.0	Glycolysis, GNG	True	1.927e+07	7.598	7.598	0.7787
249	Gly-Gly	Amino acid	2	AT_H2O2_2_2	AT_H2O2_2	0.666666666666667	Dipeptides	True	5.147e+04	-0.95	-0.95	-1.167
169	2-OH-Butyrate	Amino acid	2	AT_H2O2_2_2	AT_H2O2_2	0.833333333333333	Amino acid derivatives	True	8.334e+04	-0.2547	-0.2547	1.179
98	3-Methyl-2-Oxobutyrate	Amino acid	2	AT_H2O2_2_2	AT_H2O2_2	0.666666666666667	Amino acids biosynthesis intermediates	True	1.624e+04	-2.614	-2.614	1.145

Metabolite ID	Name	Super Pathway	Dataset	Sample ID	Group ID	Detection Fraction	Pathway	Detected	Raw Intensity	Log2 Norm Intensity	Norm Imputed	Log2 Ctrl Norm
100	4-Me-2-Oxo-Pentanoate	Amino acid	2	AT_H2O2_2_2	AT_H2O2_2	1.0	Amino acids biosynthesis intermediates	True	3.922e+04	-1.342	-1.342	1.96
253	Gly-Pro	Amino acid	2	AT_H2O2_2_2	AT_H2O2_2	0.833333333333333	Dipeptides	True	1.118e+05	0.1691	0.1691	0.4902
247	Asp-Phe	Amino acid	2	AT_H2O2_2_2	AT_H2O2_2	1.0	Dipeptides	True	3.234e+04	-1.62	-1.62	-0.6792
212	N-Ac-Asp	Amino acid	2	AT_H2O2_2_2	AT_H2O2_2	0.5	N-acetylated amino acids	False			-4.689	-0.1861
720	1-Me-Nicotinamide	Cofactor	2	AT_H2O2_2_2	AT_H2O2_2	1.0	Derivatives of NA, nicotinamide and NAD	True	5.822e+05	2.55	2.55	-1.154
70	Creatine	Carbon	2	AT_H2O2_2_2	AT_H2O2_2	1.0	Creatine energy storage	True	4.353e+06	5.452	5.452	-0.328
309	Glutathione, Oxidized	Amino acid	2	AT_H2O2_2_2	AT_H2O2_2	1.0	Glutathione	True	1.67e+06	4.07	4.07	0.122
44	Glucose 6-P	Carbon	2	AT_H2O2_2_2	AT_H2O2_2	1.0	Glycolysis, GNG	True	1.289e+05	0.3742	0.3742	-0.4405
24	Fructose	Carbon	2	AT_H2O2_2_2	AT_H2O2_2	1.0	Sugars and sugar alcohols	True	1.947e+06	4.291	4.291	0.9308
85	Cys	Amino acid	2	AT_H2O2_2_2	AT_H2O2_2	1.0	Proteinogenic amino acids	True	8.255e+04	-0.2684	-0.2684	0.3235
704	NADH	Cofactor	2	AT_H2O2_2_2	AT_H2O2_2	0.166666666666667	Cofactors	False			-4.313	-2.383
275	Thr-Phe	Amino acid	2	AT_H2O2_2_2	AT_H2O2_2	0.666666666666667	Dipeptides	False			-1.922	-0.9888
738	Pyridoxate	Cofactor	2	AT_H2O2_2_2	AT_H2O2_2	1.0	PLP biosynthesis and salvage	True	1.337e+04	-2.894	-2.894	1.771
177	3-(4-OH-Phenyl)Lactate	Amino acid	2	AT_H2O2_2_2	AT_H2O2_2	1.0	Amino acid derivatives	True	1.282e+04	-2.955	-2.955	0.9977
206	Trans-4-OH-Pro	Amino acid	2	AT_H2O2_2_2	AT_H2O2_2	1.0	Amino acid derivatives	True	1.833e+05	0.8829	0.8829	1.078
329	AMP	Nucleotide	2	AT_H2O2_2_2	AT_H2O2_2	0.5	Purine nucleotides	False			-2.644	-3.064
345	Guanine	Nucleotide	2	AT_H2O2_2_2	AT_H2O2_2	1.0	Purine bases	True	5.66e+05	2.509	2.509	0.9271
271	pyroGlu-Val	Amino acid	2	AT_H2O2_2_2	AT_H2O2_2	0.666666666666667	Dipeptides	False			-5.212	-1.57
279	Val-Glu	Amino acid	2	AT_H2O2_2_2	AT_H2O2_2	0.666666666666667	Dipeptides	False			-3	-2.17
183	Phenol Sulfate	Amino acid	2	AT_H2O2_2_2	AT_H2O2_2	0.833333333333333	Amino acid derivatives	True	7215	-3.785	-3.785	-0.05728
740	3-Dehydrocarnitine	Cofactor	2	AT_H2O2_2_2	AT_H2O2_2	0.5	Carnitine biosynthesis	False			-2.199	-1.865
145	Pyro-Gln	Amino acid	2	AT_H2O2_2_2	AT_H2O2_2	1.0	Amino acid derivatives	True	1.024e+05	0.04317	0.04317	-0.3028

Metabolite ID	Name	Super Pathway	Dataset	Sample ID	Group ID	Detection Fraction	Pathway	Detected	Raw Intensity	Log2 Norm Intensity	Norm Imputed	Log2 Ctrl Norm
197	C-Glycosyl-Trp	Amino acid	2	AT_H2O2_2_2	AT_H2O2_2	1.0	Amino acid derivatives	True	5.913e+04	-0.7499	-0.7499	-0.6062
718	Nicotinamide Riboside	Cofactor	2	AT_H2O2_2_2	AT_H2O2_2	0.5	NAD biosynthesis	True	7.345e+04	-0.4368	-0.4368	1.367
295	gamma-Glu-Phe	Amino acid	2	AT_H2O2_2_2	AT_H2O2_2	0.666666666666667	Gamma-glutamyl dipeptides	True	2.599e+04	-1.936	-1.936	-1.834
399	Pseudouridine	Nucleotide	2	AT_H2O2_2_2	AT_H2O2_2	1.0	Pyrimidine derivatives in RNAs	True	1.521e+04	-2.709	-2.709	1.532
375	UTP	Nucleotide	2	AT_H2O2_2_2	AT_H2O2_2	0.0	Pyrimidine nucleotides	False			-5.966	-0.5235
20	Erythronate	Carbon	2	AT_H2O2_2_2	AT_H2O2_2	1.0	Aminosugar derivatives	True	4.399e+04	-1.176	-1.176	0.2652
151	Phenylacetylglycine	Amino acid	2	AT_H2O2_2_2	AT_H2O2_2	1.0	Amino acid derivatives	True	3.651e+04	-1.445	-1.445	2.976
252	Gly-Phe	Amino acid	2	AT_H2O2_2_2	AT_H2O2_2	0.666666666666667	Dipeptides	False			-0.4688	-0.573
251	Gly-Leu	Amino acid	2	AT_H2O2_2_2	AT_H2O2_2	1.0	Dipeptides	True	6.644e+04	-0.5816	-0.5816	-0.9442
290	gamma-Glu-Ile	Amino acid	2	AT_H2O2_2_2	AT_H2O2_2	0.666666666666667	Gamma-glutamyl dipeptides	False			-2.961	-1.429
316	Ophthalmate	Amino acid	2	AT_H2O2_2_2	AT_H2O2_2	1.0	Oxidative stress markers	True	3.356e+04	-1.567	-1.567	-1.821
208	Pro-OH-Pro	Amino acid	2	AT_H2O2_2_2	AT_H2O2_2	1.0	Amino acid derivatives	True	1.884e+05	0.9217	0.9217	0.4621
352	3'-AMP	Nucleotide	2	AT_H2O2_2_2	AT_H2O2_2	1.0	Purine derivatives in signaling	True	2.742e+04	-1.858	-1.858	-0.5736
314	Cys-Glutathione Disulfide	Amino acid	2	AT_H2O2_2_2	AT_H2O2_2	1.0	Oxidative stress markers	True	1.048e+05	0.07523	0.07523	1.441
39	Threitol	Carbon	2	AT_H2O2_2_2	AT_H2O2_2	0.5	Sugars and sugar alcohols	False			-4.395	-0.3698
31	Ribulose/Xylulose	Carbon	2	AT_H2O2_2_2	AT_H2O2_2	0.0	Sugars and sugar alcohols	False			-5.004	-0.09716
48	DHAP	Carbon	2	AT_H2O2_2_2	AT_H2O2_2	1.0	Glycolysis, GNG	True	9.195e+04	-0.1128	-0.1128	-1.871
182	P-Cresol Sulfate	Amino acid	2	AT_H2O2_2_2	AT_H2O2_2	1.0	Amino acid derivatives	True	1.181e+04	-3.074	-3.074	0.2634
250	Gly-Ile	Amino acid	2	AT_H2O2_2_2	AT_H2O2_2	0.666666666666667	Dipeptides	True	2.003e+04	-2.311	-2.311	-0.8874
286	gamma-Glu-Glu	Amino acid	2	AT_H2O2_2_2	AT_H2O2_2	1.0	Gamma-glutamyl dipeptides	True	5.646e+04	-0.8164	-0.8164	-0.5858
264	Leu-Leu	Amino acid	2	AT_H2O2_2_2	AT_H2O2_2	0.5	Dipeptides	False			-2.222	-1.148
203	DiMe-Arg	Amino acid	2	AT_H2O2_2_2	AT_H2O2_2	1.0	Amino acid derivatives	True	6.268e+05	2.656	2.656	1.582

Metabolite ID	Name	Super Pathway	Dataset	Sample ID	Group ID	Detection Fraction	Pathway	Detected	Raw Intensity	Log2 Norm Intensity	Norm Imputed	Log2 Ctrl Norm
47	Fructose 1,6-PP, Glucose 1,6-PP	Carbon	2	AT_H2O2_2_2	AT_H2O2_2	1.0	Glycolysis, GNG	True	2.849e+04	-1.803	-1.803	-1.513
224	N-Ac-Ser	Amino acid	2	AT_H2O2_2_2	AT_H2O2_2	1.0	N-acetylated amino acids	True	3.892e+04	-1.353	-1.353	-2.224
244	Ala-Leu	Amino acid	2	AT_H2O2_2_2	AT_H2O2_2	0.166666666666667	Dipeptides	False			-1.778	-2.118
304	Cyclo(Phe-Pro)	Amino acid	2	AT_H2O2_2_2	AT_H2O2_2	0.0	Cyclic dipeptides	False			-1.192	-0.7201
302	Cyclo(Glu-Glu)	Amino acid	2	AT_H2O2_2_2	AT_H2O2_2	0.666666666666667	Cyclic dipeptides	False			-2.527	-1.355
303	Cyclo(Leu-Pro)	Amino acid	2	AT_H2O2_2_2	AT_H2O2_2	0.0	Cyclic dipeptides	False			-0.4297	-0.7026
390	2',3'-cUMP	Nucleotide	2	AT_H2O2_2_2	AT_H2O2_2	1.0	Pyrimidine derivatives in signaling	True	3.216e+04	-1.629	-1.629	-0.4997
68	Ribulose 5-P / Xylulose 5-P	Carbon	2	AT_H2O2_2_2	AT_H2O2_2	1.0	Pentose phosphate pathway (PPP)	True	4.757e+04	-1.064	-1.064	-1.833
388	2',3'-cCMP	Nucleotide	2	AT_H2O2_2_2	AT_H2O2_2	1.0	Pyrimidine derivatives in signaling	True	4.235e+04	-1.231	-1.231	-1.485
33	Arabitol/Xylitol	Carbon	2	AT_H2O2_2_2	AT_H2O2_2	0.166666666666667	Sugars and sugar alcohols	False			-3.559	-0.05408
268	Phe-Phe	Amino acid	2	AT_H2O2_2_2	AT_H2O2_2	0.0	Dipeptides	False			-2.762	-1.674
245	Ala-Phe	Amino acid	2	AT_H2O2_2_2	AT_H2O2_2	0.666666666666667	Dipeptides	True	3.862e+04	-1.364	-1.364	0.09123
373	UMP	Nucleotide	2	AT_H2O2_2_2	AT_H2O2_2	0.0	Pyrimidine nucleotides	False			-3.442	-1.402
282	Val-Leu	Amino acid	2	AT_H2O2_2_2	AT_H2O2_2	0.666666666666667	Dipeptides	True	3.732e+04	-1.414	-1.414	-1.045
258	Ile-Gly	Amino acid	2	AT_H2O2_2_2	AT_H2O2_2	0.666666666666667	Dipeptides	True	5.122e+04	-0.9568	-0.9568	-1.371
259	Ile-Ser	Amino acid	2	AT_H2O2_2_2	AT_H2O2_2	0.0	Dipeptides	False			-3.192	-2.329
269	Phe-Ser	Amino acid	2	AT_H2O2_2_2	AT_H2O2_2	0.333333333333333	Dipeptides	False			-3.197	-1.882
277	Tyr-Ala	Amino acid	2	AT_H2O2_2_2	AT_H2O2_2	0.833333333333333	Dipeptides	False			-1.848	-2.418
257	Ile-Gln	Amino acid	2	AT_H2O2_2_2	AT_H2O2_2	0.166666666666667	Dipeptides	False			-2.625	-1.706
261	Leu-Glu	Amino acid	2	AT_H2O2_2_2	AT_H2O2_2	1.0	Dipeptides	True	3.059e+04	-1.701	-1.701	-2.491
263	Leu-Gly	Amino acid	2	AT_H2O2_2_2	AT_H2O2_2	1.0	Dipeptides	True	1.924e+04	-2.37	-2.37	-3.077
256	Ile-Ala	Amino acid	2	AT_H2O2_2_2	AT_H2O2_2	0.666666666666667	Dipeptides	False			-2.214	-2.173
274	Thr-Leu	Amino acid	2	AT_H2O2_2_2	AT_H2O2_2	0.833333333333333	Dipeptides	True	3.142e+05	1.66	1.66	0.8089

Metabolite ID	Name	Super Pathway	Dataset	Sample ID	Group ID	Detection Fraction	Pathway	Detected	Raw Intensity	Log2 Norm Intensity	Norm Imputed	Log2 Ctrl Norm
273	Ser-Phe	Amino acid	2	AT_H2O2_2_2	AT_H2O2_2	0.833333333333333	Dipeptides	False			-2.449	-1.227
272	Ser-Leu	Amino acid	2	AT_H2O2_2_2	AT_H2O2_2	1.0	Dipeptides	True	6.043e+04	-0.7183	-0.7183	-1.457
246	Asp-Leu	Amino acid	2	AT_H2O2_2_2	AT_H2O2_2	1.0	Dipeptides	True	7.832e+04	-0.3442	-0.3442	-0.2702
76	Gln	Amino acid	2	AT_H2O2_2_3	AT_H2O2_2	1.0	Proteinogenic amino acids	True	8.736e+06	6.116	6.116	-0.836
89	Trp	Amino acid	2	AT_H2O2_2_3	AT_H2O2_2	1.0	Proteinogenic amino acids	True	5.785e+06	5.521	5.521	-0.4453
723	beta-Ala	Cofactor	2	AT_H2O2_2_3	AT_H2O2_2	1.0	Coenzyme A biosynthesis	True	5.954e+04	-1.081	-1.081	-1.407
75	Glu	Amino acid	2	AT_H2O2_2_3	AT_H2O2_2	1.0	Proteinogenic amino acids	True	7.614e+06	5.918	5.918	-0.03027
80	His	Amino acid	2	AT_H2O2_2_3	AT_H2O2_2	1.0	Proteinogenic amino acids	True	1.029e+05	-0.2911	-0.2911	-0.5052
82	Leu	Amino acid	2	AT_H2O2_2_3	AT_H2O2_2	1.0	Proteinogenic amino acids	True	2.537e+07	7.654	7.654	-0.5061
87	Phe	Amino acid	2	AT_H2O2_2_3	AT_H2O2_2	1.0	Proteinogenic amino acids	True	2.187e+07	7.44	7.44	-0.4518
236	Spermidine	Amino acid	2	AT_H2O2_2_3	AT_H2O2_2	1.0	Polyamines	True	3.121e+05	1.309	1.309	-1.518
73	Asn	Amino acid	2	AT_H2O2_2_3	AT_H2O2_2	1.0	Proteinogenic amino acids	True	3.068e+05	1.284	1.284	-1.465
243	Creatinine	Amino acid	2	AT_H2O2_2_3	AT_H2O2_2	1.0	Creatine degradation	True	6.06e+05	2.266	2.266	0.696
376	Cytidine	Nucleotide	2	AT_H2O2_2_3	AT_H2O2_2	1.0	Pyrimidine nucleosides	True	1.858e+05	0.5608	0.5608	1.145
41	Lactate	Carbon	2	AT_H2O2_2_3	AT_H2O2_2	1.0	Respiratory carbon sources	True	3.793e+07	8.234	8.234	0.4122
93	3-P-Ser	Amino acid	2	AT_H2O2_2_3	AT_H2O2_2	0.5	Amino acids biosynthesis intermediates	False			-4.967	-1.709
343	Adenine	Nucleotide	2	AT_H2O2_2_3	AT_H2O2_2	0.5	Purine bases	True	1.153e+04	-3.45	-3.45	-1.986
336	Adenosine	Nucleotide	2	AT_H2O2_2_3	AT_H2O2_2	1.0	Purine nucleosides	True	3.023e+05	1.263	1.263	-1.874
29	Raffinose	Carbon	2	AT_H2O2_2_3	AT_H2O2_2	0.166666666666667	Sugars and sugar alcohols	False			-5.058	-2.878
717	Nicotinamide	Cofactor	2	AT_H2O2_2_3	AT_H2O2_2	1.0	NAD biosynthesis	True	6.086e+05	2.273	2.273	-0.1242
51	PEP	Carbon	2	AT_H2O2_2_3	AT_H2O2_2	1.0	Glycolysis, GNG	True	4.448e+04	-1.502	-1.502	-0.4299
52	Pyruvate	Carbon	2	AT_H2O2_2_3	AT_H2O2_2	1.0	Glycolysis, GNG	True	1.774e+04	-2.828	-2.828	-0.4546
237	Spermine	Amino acid	2	AT_H2O2_2_3	AT_H2O2_2	0.666666666666667	Polyamines	True	2.888e+05	1.197	1.197	-2.192

Metabolite ID	Name	Super Pathway	Dataset	Sample ID	Group ID	Detection Fraction	Pathway	Detected	Raw Intensity	Log2 Norm Intensity	Norm Imputed	Log2 Ctrl Norm
385	Uracil	Nucleotide	2	AT_H2O2_2_3	AT_H2O2_2	1.0	Pyrimidine bases	True	1.922e+05	0.6097	0.6097	2.337
377	Uridine	Nucleotide	2	AT_H2O2_2_3	AT_H2O2_2	1.0	Pyrimidine nucleosides	True	6.661e+05	2.403	2.403	-0.4795
112	trans-Urocanate	Amino acid	2	AT_H2O2_2_3	AT_H2O2_2	1.0	Amino acids degradation intermediates	True	8.084e+04	-0.6397	-0.6397	2.478
737	Pyridoxine (Vitamin B6)	Cofactor	2	AT_H2O2_2_3	AT_H2O2_2	1.0	PLP biosynthesis and salvage	True	1.568e+06	3.638	3.638	0.3384
348	Allantoin	Nucleotide	2	AT_H2O2_2_3	AT_H2O2_2	1.0	Purine degradation	True	3.125e+04	-2.011	-2.011	0.8185
335	Inosine	Nucleotide	2	AT_H2O2_2_3	AT_H2O2_2	1.0	Purine nucleosides	True	1.894e+05	0.5884	0.5884	-2.273
81	Ile	Amino acid	2	AT_H2O2_2_3	AT_H2O2_2	1.0	Proteinogenic amino acids	True	2.393e+07	7.569	7.569	-0.286
72	Ala	Amino acid	2	AT_H2O2_2_3	AT_H2O2_2	1.0	Proteinogenic amino acids	True	1.202e+07	6.577	6.577	-1.157
79	Thr	Amino acid	2	AT_H2O2_2_3	AT_H2O2_2	1.0	Proteinogenic amino acids	True	4.014e+06	4.994	4.994	-0.9683
88	Tyr	Amino acid	2	AT_H2O2_2_3	AT_H2O2_2	1.0	Proteinogenic amino acids	True	1.075e+07	6.415	6.415	-0.4275
84	Lys	Amino acid	2	AT_H2O2_2_3	AT_H2O2_2	1.0	Proteinogenic amino acids	True	1.275e+06	3.34	3.34	-0.3793
86	Met	Amino acid	2	AT_H2O2_2_3	AT_H2O2_2	1.0	Proteinogenic amino acids	True	3.997e+06	4.988	4.988	-0.7991
61	Malate	Carbon	2	AT_H2O2_2_3	AT_H2O2_2	1.0	TCA cycle	True	2.987e+05	1.246	1.246	0.3492
235	Putrescine	Amino acid	2	AT_H2O2_2_3	AT_H2O2_2	0.0	Polyamines	False			-3.898	-2.331
49	3-P-Glycerate	Carbon	2	AT_H2O2_2_3	AT_H2O2_2	1.0	Glycolysis, GNG	True	4.552e+05	1.853	1.853	-0.6092
139	GABA	Amino acid	2	AT_H2O2_2_3	AT_H2O2_2	0.0	Amino acid derivatives	False			-4.963	-1.493
189	Kynurenate	Amino acid	2	AT_H2O2_2_3	AT_H2O2_2	0.833333333333333	Amino acid derivatives	True	2.299e+04	-2.454	-2.454	2.734
234	5-Me-Thioadenosine	Amino acid	2	AT_H2O2_2_3	AT_H2O2_2	1.0	SAM metabolism	True	6.496e+04	-0.9554	-0.9554	-0.8594
59	Succinate	Carbon	2	AT_H2O2_2_3	AT_H2O2_2	0.833333333333333	TCA cycle	True	2.115e+04	-2.574	-2.574	-0.5345
133	Ornithine	Amino acid	2	AT_H2O2_2_3	AT_H2O2_2	1.0	Amino acids degradation intermediates	True	6.18e+05	2.295	2.295	-0.2264
313	5-Oxoproline	Amino acid	2	AT_H2O2_2_3	AT_H2O2_2	1.0	Glutathione derivatives	True	1.212e+06	3.266	3.266	1.263
724	Pantothenate	Cofactor	2	AT_H2O2_2_3	AT_H2O2_2	1.0	Coenzyme A biosynthesis	True	1.524e+06	3.597	3.597	0.4837

Metabolite ID	Name	Super Pathway	Dataset	Sample ID	Group ID	Detection Fraction	Pathway	Detected	Raw Intensity	Log2 Norm Intensity	Norm Imputed	Log2 Ctrl Norm
30	Sucrose	Carbon	2	AT_H2O2_2_3	AT_H2O2_2	1.0	Sugars and sugar alcohols	True	1.662e+05	0.4	0.4	-1.977
122	3-OH-Isobutyrate	Amino acid	2	AT_H2O2_2_3	AT_H2O2_2	0.5	Amino acids degradation intermediates	True	2.308e+04	-2.448	-2.448	2.179
241	4-Acetamidobutanoate	Amino acid	2	AT_H2O2_2_3	AT_H2O2_2	1.0	Polyamine derivatives	True	1.277e+05	0.02027	0.02027	1.528
55	Citrate	Carbon	2	AT_H2O2_2_3	AT_H2O2_2	1.0	TCA cycle	True	1.679e+06	3.736	3.736	0.702
338	Guanosine	Nucleotide	2	AT_H2O2_2_3	AT_H2O2_2	1.0	Purine nucleosides	True	1.414e+05	0.1667	0.1667	-2.194
170	2-Amino-Butyrate	Amino acid	2	AT_H2O2_2_3	AT_H2O2_2	1.0	Amino acid derivatives	True	3.403e+05	1.434	1.434	0.2099
209	N-Ac-Ala	Amino acid	2	AT_H2O2_2_3	AT_H2O2_2	0.166666666666667	N-acetylated amino acids	False			-4.401	-1.133
221	N-Ac-Met	Amino acid	2	AT_H2O2_2_3	AT_H2O2_2	1.0	N-acetylated amino acids	True	1.982e+04	-2.668	-2.668	-1.517
22	N-Ac-Neuraminate	Carbon	2	AT_H2O2_2_3	AT_H2O2_2	0.833333333333333	Aminosugar derivatives	True	5.901e+04	-1.094	-1.094	-0.8798
346	Urate	Nucleotide	2	AT_H2O2_2_3	AT_H2O2_2	0.833333333333333	Purine degradation	True	1.095e+04	-3.524	-3.524	0.1679
90	Arg	Amino acid	2	AT_H2O2_2_3	AT_H2O2_2	1.0	Proteinogenic amino acids	True	2.432e+06	4.271	4.271	-0.511
60	Fumarate	Carbon	2	AT_H2O2_2_3	AT_H2O2_2	1.0	TCA cycle	True	1.344e+05	0.09391	0.09391	0.2506
78	Ser	Amino acid	2	AT_H2O2_2_3	AT_H2O2_2	1.0	Proteinogenic amino acids	True	5.418e+06	5.427	5.427	-1.4
83	Val	Amino acid	2	AT_H2O2_2_3	AT_H2O2_2	1.0	Proteinogenic amino acids	True	1.506e+07	6.902	6.902	-0.3324
734	Pyridoxal	Cofactor	2	AT_H2O2_2_3	AT_H2O2_2	0.666666666666667	PLP biosynthesis and salvage	True	4.653e+04	-1.437	-1.437	-0.4421
136	Urea	Amino acid	2	AT_H2O2_2_3	AT_H2O2_2	1.0	Amino acids degradation intermediates	True	6.667e+05	2.404	2.404	0.759
742	Folate	Cofactor	2	AT_H2O2_2_3	AT_H2O2_2	1.0	Folate metabolism	True	1.242e+05	-0.02027	-0.02027	0.4967
729	Riboflavin (Vitamin B2)	Cofactor	2	AT_H2O2_2_3	AT_H2O2_2	0.833333333333333	Flavine biosynthesis	True	5.997e+04	-1.071	-1.071	0.4985
91	Pro	Amino acid	2	AT_H2O2_2_3	AT_H2O2_2	1.0	Proteinogenic amino acids	True	5.861e+06	5.54	5.54	-1.181
308	Glutathione, Reduced	Amino acid	2	AT_H2O2_2_3	AT_H2O2_2	1.0	Glutathione	True	2.627e+06	4.382	4.382	-2.073
706	FAD	Cofactor	2	AT_H2O2_2_3	AT_H2O2_2	0.5	Cofactors	True	1.302e+04	-3.275	-3.275	1.822
299	gamma-Glu-Tyr	Amino acid	2	AT_H2O2_2_3	AT_H2O2_2	0.666666666666667	Gamma-glutamyl dipeptides	True	1.522e+04	-3.049	-3.049	-1.881

Metabolite ID	Name	Super Pathway	Dataset	Sample ID	Group ID	Detection Fraction	Pathway	Detected	Raw Intensity	Log2 Norm Intensity	Norm Imputed	Log2 Ctrl Norm
705	Coenzyme A	Cofactor	2	AT_H2O2_2_3	AT_H2O2_2	1.0	Cofactors	True	4848	-4.699	-4.699	-0.9929
342	Hypoxanthine	Nucleotide	2	AT_H2O2_2_3	AT_H2O2_2	1.0	Purine bases	True	1.485e+05	0.2372	0.2372	0.2353
344	Xanthine	Nucleotide	2	AT_H2O2_2_3	AT_H2O2_2	0.833333333333333	Purine bases	True	1.191e+05	-0.0809	-0.0809	2.158
703	NAD+	Cofactor	2	AT_H2O2_2_3	AT_H2O2_2	1.0	Cofactors	True	3.617e+05	1.522	1.522	-2.037
731	Thiamin (Vitamin B1)	Cofactor	2	AT_H2O2_2_3	AT_H2O2_2	1.0	TPP biosynthesis	True	1.03e+05	-0.2905	-0.2905	-0.08439
102	2-Aminoadipate	Amino acid	2	AT_H2O2_2_3	AT_H2O2_2	1.0	Amino acids biosynthesis intermediates	True	8.01e+04	-0.6531	-0.6531	-1.41
77	Gly	Amino acid	2	AT_H2O2_2_3	AT_H2O2_2	1.0	Proteinogenic amino acids	True	8.069e+06	6.001	6.001	-1.227
45	Fructose-6-P	Carbon	2	AT_H2O2_2_3	AT_H2O2_2	0.833333333333333	Glycolysis, GNG	True	1.18e+05	-0.09471	-0.09471	1.341
36	Ribose	Carbon	2	AT_H2O2_2_3	AT_H2O2_2	0.666666666666667	Sugars and sugar alcohols	False			-3.346	-2.349
4	GlcNAc 6-P	Carbon	2	AT_H2O2_2_3	AT_H2O2_2	1.0	Aminosugar biosynthesis	True	9.316e+04	-0.4352	-0.4352	-0.7949
188	Kynurenine	Amino acid	2	AT_H2O2_2_3	AT_H2O2_2	0.833333333333333	Amino acid derivatives	True	6.942e+04	-0.8595	-0.8595	-0.8455
63	6-P-Gluconate	Carbon	2	AT_H2O2_2_3	AT_H2O2_2	1.0	Pentose phosphate pathway (PPP)	True	1.867e+05	0.5675	0.5675	1.613
710	Carnitine	Cofactor	2	AT_H2O2_2_3	AT_H2O2_2	1.0	Cofactors	True	1.977e+05	0.6503	0.6503	-1.881
725	P-Pantetheine	Cofactor	2	AT_H2O2_2_3	AT_H2O2_2	0.833333333333333	Coenzyme A biosynthesis	True	7190	-4.131	-4.131	0.6817
110	N-alpha-Ac-Ornithine	Amino acid	2	AT_H2O2_2_3	AT_H2O2_2	1.0	Amino acids biosynthesis intermediates	True	1.69e+05	0.4239	0.4239	-0.5502
116	3-Me-2-Oxo-Valerate	Amino acid	2	AT_H2O2_2_3	AT_H2O2_2	1.0	Amino acids degradation intermediates	True	3.59e+04	-1.811	-1.811	1.764
155	4-Guanidinobutanoate	Amino acid	2	AT_H2O2_2_3	AT_H2O2_2	0.666666666666667	Amino acid derivatives	True	3.354e+04	-1.909	-1.909	-1.189
310	S-Lactoyl-Glutathione	Amino acid	2	AT_H2O2_2_3	AT_H2O2_2	0.166666666666667	Glutathione derivatives	False			-4.634	-0.962
34	Ribitol	Carbon	2	AT_H2O2_2_3	AT_H2O2_2	1.0	Sugars and sugar alcohols	True	2.533e+04	-2.314	-2.314	-0.7355
707	FMN	Cofactor	2	AT_H2O2_2_3	AT_H2O2_2	0.666666666666667	Cofactors	True	8380	-3.91	-3.91	-0.489
17	Maltose	Carbon	2	AT_H2O2_2_3	AT_H2O2_2	1.0	Glycogen degradation	True	3.33e+05	1.403	1.403	1.442

Metabolite ID	Name	Super Pathway	Dataset	Sample ID	Group ID	Detection Fraction	Pathway	Detected	Raw Intensity	Log2 Norm Intensity	Norm Imputed	Log2 Ctrl Norm
18	Maltotriose	Carbon	2	AT_H2O2_2_3	AT_H2O2_2	1.0	Glycogen degradation	True	1.261e+06	3.323	3.323	1.894
19	Maltotetraose	Carbon	2	AT_H2O2_2_3	AT_H2O2_2	1.0	Glycogen degradation	True	3.115e+05	1.306	1.306	1.497
232	SAH	Amino acid	2	AT_H2O2_2_3	AT_H2O2_2	1.0	SAM metabolism	True	1.286e+04	-3.292	-3.292	-0.06577
74	Asp	Amino acid	2	AT_H2O2_2_3	AT_H2O2_2	1.0	Proteinogenic amino acids	True	1.377e+06	3.45	3.45	-2.209
129	5-Aminovalerate	Amino acid	2	AT_H2O2_2_3	AT_H2O2_2	0.666666666666667	Amino acids degradation intermediates	True	4.643e+04	-1.44	-1.44	-0.6268
254	Gly-Val	Amino acid	2	AT_H2O2_2_3	AT_H2O2_2	1.0	Dipeptides	True	6.162e+04	-1.031	-1.031	-0.9577
291	gamma-Glu-Leu	Amino acid	2	AT_H2O2_2_3	AT_H2O2_2	0.833333333333333	Gamma-glutamyl dipeptides	True	3.471e+04	-1.859	-1.859	-0.7774
173	Met Sulfoxide	Amino acid	2	AT_H2O2_2_3	AT_H2O2_2	1.0	Amino acid derivatives	True	2.144e+05	0.7675	0.7675	0.9063
43	Glucose	Carbon	2	AT_H2O2_2_3	AT_H2O2_2	1.0	Glycolysis, GNG	True	1.594e+07	6.983	6.983	0.1638
249	Gly-Gly	Amino acid	2	AT_H2O2_2_3	AT_H2O2_2	0.666666666666667	Dipeptides	True	5.376e+04	-1.228	-1.228	-1.446
169	2-OH-Butyrate	Amino acid	2	AT_H2O2_2_3	AT_H2O2_2	0.833333333333333	Amino acid derivatives	False			-1.729	-0.2955
98	3-Methyl-2-Oxobutyrate	Amino acid	2	AT_H2O2_2_3	AT_H2O2_2	0.666666666666667	Amino acids biosynthesis intermediates	True	1.809e+04	-2.8	-2.8	0.9587
100	4-Me-2-Oxo-Pentanoate	Amino acid	2	AT_H2O2_2_3	AT_H2O2_2	1.0	Amino acids biosynthesis intermediates	True	3.353e+04	-1.909	-1.909	1.392
253	Gly-Pro	Amino acid	2	AT_H2O2_2_3	AT_H2O2_2	0.833333333333333	Dipeptides	True	4.493e+04	-1.487	-1.487	-1.166
247	Asp-Phe	Amino acid	2	AT_H2O2_2_3	AT_H2O2_2	1.0	Dipeptides	True	2.589e+04	-2.282	-2.282	-1.341
212	N-Ac-Asp	Amino acid	2	AT_H2O2_2_3	AT_H2O2_2	0.5	N-acetylated amino acids	True	1.098e+04	-3.519	-3.519	0.9831
720	1-Me-Nicotinamide	Cofactor	2	AT_H2O2_2_3	AT_H2O2_2	1.0	Derivatives of NA, nicotinamide and NAD	True	1.179e+06	3.226	3.226	-0.4776
70	Creatine	Carbon	2	AT_H2O2_2_3	AT_H2O2_2	1.0	Creatine energy storage	True	5.718e+06	5.504	5.504	-0.2759
309	Glutathione, Oxidized	Amino acid	2	AT_H2O2_2_3	AT_H2O2_2	1.0	Glutathione	True	1.668e+06	3.727	3.727	-0.221
44	Glucose 6-P	Carbon	2	AT_H2O2_2_3	AT_H2O2_2	1.0	Glycolysis, GNG	True	4.284e+05	1.766	1.766	0.9513
24	Fructose	Carbon	2	AT_H2O2_2_3	AT_H2O2_2	1.0	Sugars and sugar alcohols	True	1.623e+06	3.688	3.688	0.327

Metabolite ID	Name	Super Pathway	Dataset	Sample ID	Group ID	Detection Fraction	Pathway	Detected	Raw Intensity	Log2 Norm Intensity	Norm Imputed	Log2 Ctrl Norm
85	Cys	Amino acid	2	AT_H2O2_2_3	AT_H2O2_2	1.0	Proteinogenic amino acids	True	2.201e+05	0.8049	0.8049	1.397
704	NADH	Cofactor	2	AT_H2O2_2_3	AT_H2O2_2	0.166666666666667	Cofactors	False			-4.313	-2.383
275	Thr-Phe	Amino acid	2	AT_H2O2_2_3	AT_H2O2_2	0.666666666666667	Dipeptides	False			-1.922	-0.9888
738	Pyridoxate	Cofactor	2	AT_H2O2_2_3	AT_H2O2_2	1.0	PLP biosynthesis and salvage	True	1.742e+04	-2.854	-2.854	1.811
177	3-(4-OH-Phenyl)Lactate	Amino acid	2	AT_H2O2_2_3	AT_H2O2_2	1.0	Amino acid derivatives	True	1.39e+04	-3.18	-3.18	0.7733
206	Trans-4-OH-Pro	Amino acid	2	AT_H2O2_2_3	AT_H2O2_2	1.0	Amino acid derivatives	True	2.601e+05	1.046	1.046	1.242
329	AMP	Nucleotide	2	AT_H2O2_2_3	AT_H2O2_2	0.5	Purine nucleotides	True	2.538e+04	-2.311	-2.311	-2.732
345	Guanine	Nucleotide	2	AT_H2O2_2_3	AT_H2O2_2	1.0	Purine bases	True	2.176e+05	0.7888	0.7888	-0.7932
271	pyroGlu-Val	Amino acid	2	AT_H2O2_2_3	AT_H2O2_2	0.666666666666667	Dipeptides	True	9118	-3.788	-3.788	-0.1466
279	Val-Glu	Amino acid	2	AT_H2O2_2_3	AT_H2O2_2	0.666666666666667	Dipeptides	True	1.575e+04	-3	-3	-2.17
183	Phenol Sulfate	Amino acid	2	AT_H2O2_2_3	AT_H2O2_2	0.833333333333333	Amino acid derivatives	True	2.387e+05	0.9225	0.9225	4.65
740	3-Dehydrocarnitine	Cofactor	2	AT_H2O2_2_3	AT_H2O2_2	0.5	Carnitine biosynthesis	False			-2.199	-1.865
145	Pyro-Gln	Amino acid	2	AT_H2O2_2_3	AT_H2O2_2	1.0	Amino acid derivatives	True	2.22e+05	0.8177	0.8177	0.4717
197	C-Glycosyl-Trp	Amino acid	2	AT_H2O2_2_3	AT_H2O2_2	1.0	Amino acid derivatives	True	1.582e+05	0.329	0.329	0.4727
718	Nicotinamide Riboside	Cofactor	2	AT_H2O2_2_3	AT_H2O2_2	0.5	NAD biosynthesis	False			-2.306	-0.5023
295	gamma-Glu-Phe	Amino acid	2	AT_H2O2_2_3	AT_H2O2_2	0.666666666666667	Gamma-glutamyl dipeptides	True	4.078e+04	-1.627	-1.627	-1.525
399	Pseudouridine	Nucleotide	2	AT_H2O2_2_3	AT_H2O2_2	1.0	Pyrimidine derivatives in RNAs	True	6.616e+04	-0.929	-0.929	3.312
375	UTP	Nucleotide	2	AT_H2O2_2_3	AT_H2O2_2	0.0	Pyrimidine nucleotides	False			-5.966	-0.5235
20	Erythronate	Carbon	2	AT_H2O2_2_3	AT_H2O2_2	1.0	Aminosugar derivatives	True	3.758e+04	-1.745	-1.745	-0.3033
151	Phenylacetylglycine	Amino acid	2	AT_H2O2_2_3	AT_H2O2_2	1.0	Amino acid derivatives	True	5.747e+05	2.19	2.19	6.611
252	Gly-Phe	Amino acid	2	AT_H2O2_2_3	AT_H2O2_2	0.666666666666667	Dipeptides	True	1.058e+05	-0.2517	-0.2517	-0.3559
251	Gly-Leu	Amino acid	2	AT_H2O2_2_3	AT_H2O2_2	1.0	Dipeptides	True	3.421e+04	-1.88	-1.88	-2.243

Metabolite ID	Name	Super Pathway	Dataset	Sample ID	Group ID	Detection Fraction	Pathway	Detected	Raw Intensity	Log2 Norm Intensity	Norm Imputed	Log2 Ctrl Norm
290	gamma-Glu-Ile	Amino acid	2	AT_H2O2_2_3	AT_H2O2_2	0.666666666666667	Gamma-glutamyl dipeptides	True	1.736e+04	-2.859	-2.859	-1.328
316	Ophthalmate	Amino acid	2	AT_H2O2_2_3	AT_H2O2_2	1.0	Oxidative stress markers	True	5.359e+04	-1.233	-1.233	-1.487
208	Pro-OH-Pro	Amino acid	2	AT_H2O2_2_3	AT_H2O2_2	1.0	Amino acid derivatives	True	1.731e+05	0.4587	0.4587	-8.90e-04
352	3'-AMP	Nucleotide	2	AT_H2O2_2_3	AT_H2O2_2	1.0	Purine derivatives in signaling	True	2.905e+04	-2.116	-2.116	-0.8316
314	Cys-Glutathione Disulfide	Amino acid	2	AT_H2O2_2_3	AT_H2O2_2	1.0	Oxidative stress markers	True	1.628e+05	0.3705	0.3705	1.736
39	Threitol	Carbon	2	AT_H2O2_2_3	AT_H2O2_2	0.5	Sugars and sugar alcohols	True	1.474e+04	-3.095	-3.095	0.9299
31	Ribulose/Xylulose	Carbon	2	AT_H2O2_2_3	AT_H2O2_2	0.0	Sugars and sugar alcohols	False			-5.004	-0.09716
48	DHAP	Carbon	2	AT_H2O2_2_3	AT_H2O2_2	1.0	Glycolysis, GNG	True	1.39e+05	0.1419	0.1419	-1.616
182	P-Cresol Sulfate	Amino acid	2	AT_H2O2_2_3	AT_H2O2_2	1.0	Amino acid derivatives	True	5.954e+04	-1.081	-1.081	2.256
250	Gly-Ile	Amino acid	2	AT_H2O2_2_3	AT_H2O2_2	0.666666666666667	Dipeptides	False			-2.655	-1.231
286	gamma-Glu-Glu	Amino acid	2	AT_H2O2_2_3	AT_H2O2_2	1.0	Gamma-glutamyl dipeptides	True	3.179e+04	-1.986	-1.986	-1.756
264	Leu-Leu	Amino acid	2	AT_H2O2_2_3	AT_H2O2_2	0.5	Dipeptides	False			-2.222	-1.148
203	DiMe-Arg	Amino acid	2	AT_H2O2_2_3	AT_H2O2_2	1.0	Amino acid derivatives	True	4.126e+05	1.712	1.712	0.6375
47	Fructose 1,6-PP, Glucose 1,6-PP	Carbon	2	AT_H2O2_2_3	AT_H2O2_2	1.0	Glycolysis, GNG	True	3.818e+04	-1.722	-1.722	-1.432
224	N-Ac-Ser	Amino acid	2	AT_H2O2_2_3	AT_H2O2_2	1.0	N-acetylated amino acids	True	5.364e+04	-1.232	-1.232	-2.102
244	Ala-Leu	Amino acid	2	AT_H2O2_2_3	AT_H2O2_2	0.166666666666667	Dipeptides	False			-1.778	-2.118
304	Cyclo(Phe-Pro)	Amino acid	2	AT_H2O2_2_3	AT_H2O2_2	0.0	Cyclic dipeptides	False			-1.192	-0.7201
302	Cyclo(Glu-Glu)	Amino acid	2	AT_H2O2_2_3	AT_H2O2_2	0.666666666666667	Cyclic dipeptides	True	7.364e+04	-0.7744	-0.7744	0.3978
303	Cyclo(Leu-Pro)	Amino acid	2	AT_H2O2_2_3	AT_H2O2_2	0.0	Cyclic dipeptides	False			-0.4297	-0.7026
390	2',3'-cUMP	Nucleotide	2	AT_H2O2_2_3	AT_H2O2_2	1.0	Pyrimidine derivatives in signaling	True	3.604e+04	-1.805	-1.805	-0.6764
68	Ribulose 5-P / Xylulose 5-P	Carbon	2	AT_H2O2_2_3	AT_H2O2_2	1.0	Pentose phosphate pathway (PPP)	True	6.661e+04	-0.9191	-0.9191	-1.688
388	2',3'-cCMP	Nucleotide	2	AT_H2O2_2_3	AT_H2O2_2	1.0	Pyrimidine derivatives in signaling	True	4.594e+04	-1.455	-1.455	-1.708

Metabolite ID	Name	Super Pathway	Dataset	Sample ID	Group ID	Detection Fraction	Pathway	Detected	Raw Intensity	Log2 Norm Intensity	Norm Imputed	Log2 Ctrl Norm
33	Arabitol/Xylitol	Carbon	2	AT_H2O2_2_3	AT_H2O2_2	0.166666666666667	Sugars and sugar alcohols	False			-3.559	-0.05408
268	Phe-Phe	Amino acid	2	AT_H2O2_2_3	AT_H2O2_2	0.0	Dipeptides	False			-2.762	-1.674
245	Ala-Phe	Amino acid	2	AT_H2O2_2_3	AT_H2O2_2	0.666666666666667	Dipeptides	False			-2.821	-1.366
373	UMP	Nucleotide	2	AT_H2O2_2_3	AT_H2O2_2	0.0	Pyrimidine nucleotides	False			-3.442	-1.402
282	Val-Leu	Amino acid	2	AT_H2O2_2_3	AT_H2O2_2	0.666666666666667	Dipeptides	False			-1.414	-1.045
258	Ile-Gly	Amino acid	2	AT_H2O2_2_3	AT_H2O2_2	0.666666666666667	Dipeptides	True	1.989e+04	-2.663	-2.663	-3.077
259	Ile-Ser	Amino acid	2	AT_H2O2_2_3	AT_H2O2_2	0.0	Dipeptides	False			-3.192	-2.329
269	Phe-Ser	Amino acid	2	AT_H2O2_2_3	AT_H2O2_2	0.333333333333333	Dipeptides	False			-3.197	-1.882
277	Tyr-Ala	Amino acid	2	AT_H2O2_2_3	AT_H2O2_2	0.833333333333333	Dipeptides	True	4.883e+04	-1.367	-1.367	-1.937
257	Ile-Gln	Amino acid	2	AT_H2O2_2_3	AT_H2O2_2	0.166666666666667	Dipeptides	False			-2.625	-1.706
261	Leu-Glu	Amino acid	2	AT_H2O2_2_3	AT_H2O2_2	1.0	Dipeptides	True	5.315e+04	-1.245	-1.245	-2.035
263	Leu-Gly	Amino acid	2	AT_H2O2_2_3	AT_H2O2_2	1.0	Dipeptides	True	7.317e+04	-0.7836	-0.7836	-1.491
256	Ile-Ala	Amino acid	2	AT_H2O2_2_3	AT_H2O2_2	0.666666666666667	Dipeptides	False			-2.214	-2.173
274	Thr-Leu	Amino acid	2	AT_H2O2_2_3	AT_H2O2_2	0.833333333333333	Dipeptides	False			0.2082	-0.6429
273	Ser-Phe	Amino acid	2	AT_H2O2_2_3	AT_H2O2_2	0.833333333333333	Dipeptides	True	3.147e+04	-2.001	-2.001	-0.779
272	Ser-Leu	Amino acid	2	AT_H2O2_2_3	AT_H2O2_2	1.0	Dipeptides	True	4.302e+04	-1.55	-1.55	-2.289
246	Asp-Leu	Amino acid	2	AT_H2O2_2_3	AT_H2O2_2	1.0	Dipeptides	True	6.141e+04	-1.036	-1.036	-0.9624
76	Gln	Amino acid	2	AT_H2O2_2_4	AT_H2O2_2	1.0	Proteinogenic amino acids	True	8.804e+06	6.03	6.03	-0.9222
89	Trp	Amino acid	2	AT_H2O2_2_4	AT_H2O2_2	1.0	Proteinogenic amino acids	True	6.825e+06	5.662	5.662	-0.3041
723	beta-Ala	Cofactor	2	AT_H2O2_2_4	AT_H2O2_2	1.0	Coenzyme A biosynthesis	True	1.394e+05	0.0489	0.0489	-0.2774
75	Glu	Amino acid	2	AT_H2O2_2_4	AT_H2O2_2	1.0	Proteinogenic amino acids	True	5.68e+06	5.397	5.397	-0.5505
80	His	Amino acid	2	AT_H2O2_2_4	AT_H2O2_2	1.0	Proteinogenic amino acids	True	1.123e+05	-0.2632	-0.2632	-0.4773
82	Leu	Amino acid	2	AT_H2O2_2_4	AT_H2O2_2	1.0	Proteinogenic amino acids	True	3.338e+07	7.953	7.953	-0.2076

Metabolite ID	Name	Super Pathway	Dataset	Sample ID	Group ID	Detection Fraction	Pathway	Detected	Raw Intensity	Log2 Norm Intensity	Norm Imputed	Log2 Ctrl Norm
87	Phe	Amino acid	2	AT_H2O2_2_4	AT_H2O2_2	1.0	Proteinogenic amino acids	True	2.792e+07	7.695	7.695	-0.1971
236	Spermidine	Amino acid	2	AT_H2O2_2_4	AT_H2O2_2	1.0	Polyamines	True	1.381e+05	0.03542	0.03542	-2.792
73	Asn	Amino acid	2	AT_H2O2_2_4	AT_H2O2_2	1.0	Proteinogenic amino acids	True	2.216e+05	0.7178	0.7178	-2.031
243	Creatinine	Amino acid	2	AT_H2O2_2_4	AT_H2O2_2	1.0	Creatine degradation	True	7.633e+05	2.502	2.502	0.9314
376	Cytidine	Nucleotide	2	AT_H2O2_2_4	AT_H2O2_2	1.0	Pyrimidine nucleosides	True	6.64e+05	2.301	2.301	2.886
41	Lactate	Carbon	2	AT_H2O2_2_4	AT_H2O2_2	1.0	Respiratory carbon sources	True	3.764e+07	8.126	8.126	0.3039
93	3-P-Ser	Amino acid	2	AT_H2O2_2_4	AT_H2O2_2	0.5	Amino acids biosynthesis intermediates	False			-4.967	-1.709
343	Adenine	Nucleotide	2	AT_H2O2_2_4	AT_H2O2_2	0.5	Purine bases	True	2.992e+04	-2.171	-2.171	-0.7078
336	Adenosine	Nucleotide	2	AT_H2O2_2_4	AT_H2O2_2	1.0	Purine nucleosides	True	1.889e+05	0.4871	0.4871	-2.65
29	Raffinose	Carbon	2	AT_H2O2_2_4	AT_H2O2_2	0.166666666666667	Sugars and sugar alcohols	True	4223	-4.996	-4.996	-2.816
717	Nicotinamide	Cofactor	2	AT_H2O2_2_4	AT_H2O2_2	1.0	NAD biosynthesis	True	8.332e+05	2.628	2.628	0.2315
51	PEP	Carbon	2	AT_H2O2_2_4	AT_H2O2_2	1.0	Glycolysis, GNG	True	8.33e+04	-0.6939	-0.6939	0.378
52	Pyruvate	Carbon	2	AT_H2O2_2_4	AT_H2O2_2	1.0	Glycolysis, GNG	True	2.585e+04	-2.382	-2.382	-8.50e-03
237	Spermine	Amino acid	2	AT_H2O2_2_4	AT_H2O2_2	0.666666666666667	Polyamines	False			0.9044	-2.484
385	Uracil	Nucleotide	2	AT_H2O2_2_4	AT_H2O2_2	1.0	Pyrimidine bases	True	6.77e+04	-0.9931	-0.9931	0.7338
377	Uridine	Nucleotide	2	AT_H2O2_2_4	AT_H2O2_2	1.0	Pyrimidine nucleosides	True	4.942e+05	1.875	1.875	-1.008
112	trans-Urocanate	Amino acid	2	AT_H2O2_2_4	AT_H2O2_2	1.0	Amino acids degradation intermediates	True	1.746e+05	0.374	0.374	3.492
737	Pyridoxine (Vitamin B6)	Cofactor	2	AT_H2O2_2_4	AT_H2O2_2	1.0	PLP biosynthesis and salvage	True	1.938e+06	3.846	3.846	0.5465
348	Allantoin	Nucleotide	2	AT_H2O2_2_4	AT_H2O2_2	1.0	Purine degradation	True	3.789e+04	-1.83	-1.83	0.999
335	Inosine	Nucleotide	2	AT_H2O2_2_4	AT_H2O2_2	1.0	Purine nucleosides	True	3.202e+05	1.249	1.249	-1.613
81	Ile	Amino acid	2	AT_H2O2_2_4	AT_H2O2_2	1.0	Proteinogenic amino acids	True	3.146e+07	7.867	7.867	0.01158
72	Ala	Amino acid	2	AT_H2O2_2_4	AT_H2O2_2	1.0	Proteinogenic amino acids	True	1.373e+07	6.671	6.671	-1.063
79	Thr	Amino acid	2	AT_H2O2_2_4	AT_H2O2_2	1.0	Proteinogenic amino acids	True	3.467e+06	4.685	4.685	-1.277

Metabolite ID	Name	Super Pathway	Dataset	Sample ID	Group ID	Detection Fraction	Pathway	Detected	Raw Intensity	Log2 Norm Intensity	Norm Imputed	Log2 Ctrl Norm
88	Tyr	Amino acid	2	AT_H2O2_2_4	AT_H2O2_2	1.0	Proteinogenic amino acids	True	1.221e+07	6.502	6.502	-0.3404
84	Lys	Amino acid	2	AT_H2O2_2_4	AT_H2O2_2	1.0	Proteinogenic amino acids	True	1.771e+06	3.716	3.716	-3.39e-03
86	Met	Amino acid	2	AT_H2O2_2_4	AT_H2O2_2	1.0	Proteinogenic amino acids	True	5.066e+06	5.232	5.232	-0.5549
61	Malate	Carbon	2	AT_H2O2_2_4	AT_H2O2_2	1.0	TCA cycle	True	1.505e+05	0.1594	0.1594	-0.7374
235	Putrescine	Amino acid	2	AT_H2O2_2_4	AT_H2O2_2	0.0	Polyamines	False			-3.898	-2.331
49	3-P-Glycerate	Carbon	2	AT_H2O2_2_4	AT_H2O2_2	1.0	Glycolysis, GNG	True	5.939e+05	2.14	2.14	-0.3228
139	GABA	Amino acid	2	AT_H2O2_2_4	AT_H2O2_2	0.0	Amino acid derivatives	False			-4.963	-1.493
189	Kynurenate	Amino acid	2	AT_H2O2_2_4	AT_H2O2_2	0.833333333333333	Amino acid derivatives	True	4.491e+04	-1.585	-1.585	3.603
234	5-Me-Thioadenosine	Amino acid	2	AT_H2O2_2_4	AT_H2O2_2	1.0	SAM metabolism	True	3.086e+04	-2.127	-2.127	-2.031
59	Succinate	Carbon	2	AT_H2O2_2_4	AT_H2O2_2	0.833333333333333	TCA cycle	True	4.945e+04	-1.446	-1.446	0.5936
133	Ornithine	Amino acid	2	AT_H2O2_2_4	AT_H2O2_2	1.0	Amino acids degradation intermediates	True	1.269e+06	3.235	3.235	0.7142
313	5-Oxoproline	Amino acid	2	AT_H2O2_2_4	AT_H2O2_2	1.0	Glutathione derivatives	True	1.877e+06	3.8	3.8	1.796
724	Pantothenate	Cofactor	2	AT_H2O2_2_4	AT_H2O2_2	1.0	Coenzyme A biosynthesis	True	1.42e+06	3.397	3.397	0.2837
30	Sucrose	Carbon	2	AT_H2O2_2_4	AT_H2O2_2	1.0	Sugars and sugar alcohols	True	9.196e+05	2.771	2.771	0.3939
122	3-OH-Isobutyrate	Amino acid	2	AT_H2O2_2_4	AT_H2O2_2	0.5	Amino acids degradation intermediates	False			-4.628	0
241	4-Acetamidobutanoate	Amino acid	2	AT_H2O2_2_4	AT_H2O2_2	1.0	Polyamine derivatives	True	1.337e+05	-0.01103	-0.01103	1.496
55	Citrate	Carbon	2	AT_H2O2_2_4	AT_H2O2_2	1.0	TCA cycle	True	5.862e+05	2.121	2.121	-0.9134
338	Guanosine	Nucleotide	2	AT_H2O2_2_4	AT_H2O2_2	1.0	Purine nucleosides	True	4.368e+05	1.697	1.697	-0.6639
170	2-Amino-Butyrate	Amino acid	2	AT_H2O2_2_4	AT_H2O2_2	1.0	Amino acid derivatives	True	2.701e+05	1.003	1.003	-0.2209
209	N-Ac-Ala	Amino acid	2	AT_H2O2_2_4	AT_H2O2_2	0.166666666666667	N-acetylated amino acids	False			-4.401	-1.133
221	N-Ac-Met	Amino acid	2	AT_H2O2_2_4	AT_H2O2_2	1.0	N-acetylated amino acids	True	1.578e+04	-3.094	-3.094	-1.944
22	N-Ac-Neuraminate	Carbon	2	AT_H2O2_2_4	AT_H2O2_2	0.833333333333333	Aminosugar derivatives	True	6.673e+04	-1.014	-1.014	-0.8

Metabolite ID	Name	Super Pathway	Dataset	Sample ID	Group ID	Detection Fraction	Pathway	Detected	Raw Intensity	Log2 Norm Intensity	Norm Imputed	Log2 Ctrl Norm
346	Urate	Nucleotide	2	AT_H2O2_2_4	AT_H2O2_2	0.833333333333333	Purine degradation	True	1.834e+04	-2.877	-2.877	0.8146
90	Arg	Amino acid	2	AT_H2O2_2_4	AT_H2O2_2	1.0	Proteinogenic amino acids	True	2.672e+06	4.309	4.309	-0.4727
60	Fumarate	Carbon	2	AT_H2O2_2_4	AT_H2O2_2	1.0	TCA cycle	True	6.096e+04	-1.145	-1.145	-0.9878
78	Ser	Amino acid	2	AT_H2O2_2_4	AT_H2O2_2	1.0	Proteinogenic amino acids	True	6.629e+06	5.62	5.62	-1.206
83	Val	Amino acid	2	AT_H2O2_2_4	AT_H2O2_2	1.0	Proteinogenic amino acids	True	2.186e+07	7.342	7.342	0.1077
734	Pyridoxal	Cofactor	2	AT_H2O2_2_4	AT_H2O2_2	0.666666666666667	PLP biosynthesis and salvage	False			-1.437	-0.4421
136	Urea	Amino acid	2	AT_H2O2_2_4	AT_H2O2_2	1.0	Amino acids degradation intermediates	True	6.228e+05	2.208	2.208	0.5634
742	Folate	Cofactor	2	AT_H2O2_2_4	AT_H2O2_2	1.0	Folate metabolism	True	1.204e+05	-0.1627	-0.1627	0.3543
729	Riboflavin (Vitamin B2)	Cofactor	2	AT_H2O2_2_4	AT_H2O2_2	0.833333333333333	Flavine biosynthesis	True	3.597e+04	-1.906	-1.906	-0.3365
91	Pro	Amino acid	2	AT_H2O2_2_4	AT_H2O2_2	1.0	Proteinogenic amino acids	True	7.12e+06	5.723	5.723	-0.9975
308	Glutathione, Reduced	Amino acid	2	AT_H2O2_2_4	AT_H2O2_2	1.0	Glutathione	True	5.856e+06	5.442	5.442	-1.014
706	FAD	Cofactor	2	AT_H2O2_2_4	AT_H2O2_2	0.5	Cofactors	False			-5.096	0
299	gamma-Glu-Tyr	Amino acid	2	AT_H2O2_2_4	AT_H2O2_2	0.666666666666667	Gamma-glutamyl dipeptides	True	2.673e+04	-2.334	-2.334	-1.166
705	Coenzyme A	Cofactor	2	AT_H2O2_2_4	AT_H2O2_2	1.0	Cofactors	True	2181	-5.949	-5.949	-2.243
342	Hypoxanthine	Nucleotide	2	AT_H2O2_2_4	AT_H2O2_2	1.0	Purine bases	True	5.911e+04	-1.189	-1.189	-1.191
344	Xanthine	Nucleotide	2	AT_H2O2_2_4	AT_H2O2_2	0.833333333333333	Purine bases	True	2.242e+04	-2.588	-2.588	-0.3485
703	NAD+	Cofactor	2	AT_H2O2_2_4	AT_H2O2_2	1.0	Cofactors	True	4.462e+05	1.727	1.727	-1.831
731	Thiamin (Vitamin B1)	Cofactor	2	AT_H2O2_2_4	AT_H2O2_2	1.0	TPP biosynthesis	True	1.233e+05	-0.1281	-0.1281	0.07802
102	2-Aminoadipate	Amino acid	2	AT_H2O2_2_4	AT_H2O2_2	1.0	Amino acids biosynthesis intermediates	True	1.091e+05	-0.3041	-0.3041	-1.061
77	Gly	Amino acid	2	AT_H2O2_2_4	AT_H2O2_2	1.0	Proteinogenic amino acids	True	6.334e+06	5.555	5.555	-1.673
45	Fructose-6-P	Carbon	2	AT_H2O2_2_4	AT_H2O2_2	0.833333333333333	Glycolysis, GNG	False			-2.517	-1.082
36	Ribose	Carbon	2	AT_H2O2_2_4	AT_H2O2_2	0.666666666666667	Sugars and sugar alcohols	True	2.033e+04	-2.729	-2.729	-1.731
4	GlcNAc 6-P	Carbon	2	AT_H2O2_2_4	AT_H2O2_2	1.0	Aminosugar biosynthesis	True	7.794e+04	-0.7899	-0.7899	-1.15

Metabolite ID	Name	Super Pathway	Dataset	Sample ID	Group ID	Detection Fraction	Pathway	Detected	Raw Intensity	Log2 Norm Intensity	Norm Imputed	Log2 Ctrl Norm
188	Kynurenine	Amino acid	2	AT_H2O2_2_4	AT_H2O2_2	0.833333333333333	Amino acid derivatives	True	2.316e+05	0.7813	0.7813	0.7953
63	6-P-Gluconate	Carbon	2	AT_H2O2_2_4	AT_H2O2_2	1.0	Pentose phosphate pathway (PPP)	True	4.106e+04	-1.715	-1.715	-0.6694
710	Carnitine	Cofactor	2	AT_H2O2_2_4	AT_H2O2_2	1.0	Cofactors	True	4.578e+05	1.764	1.764	-0.7676
725	P-Pantetheine	Cofactor	2	AT_H2O2_2_4	AT_H2O2_2	0.833333333333333	Coenzyme A biosynthesis	False			-5.267	-0.4543
110	N-alpha-Ac-Ornithine	Amino acid	2	AT_H2O2_2_4	AT_H2O2_2	1.0	Amino acids biosynthesis intermediates	True	1.662e+05	0.3026	0.3026	-0.6714
116	3-Me-2-Oxo-Valerate	Amino acid	2	AT_H2O2_2_4	AT_H2O2_2	1.0	Amino acids degradation intermediates	True	9.134e+04	-0.561	-0.561	3.014
155	4-Guanidinobutanoate	Amino acid	2	AT_H2O2_2_4	AT_H2O2_2	0.666666666666667	Amino acid derivatives	False			-2.324	-1.604
310	S-Lactoyl-Glutathione	Amino acid	2	AT_H2O2_2_4	AT_H2O2_2	0.166666666666667	Glutathione derivatives	False			-4.634	-0.962
34	Ribitol	Carbon	2	AT_H2O2_2_4	AT_H2O2_2	1.0	Sugars and sugar alcohols	True	7.167e+04	-0.9109	-0.9109	0.6678
707	FMN	Cofactor	2	AT_H2O2_2_4	AT_H2O2_2	0.666666666666667	Cofactors	False			-4.56	-1.139
17	Maltose	Carbon	2	AT_H2O2_2_4	AT_H2O2_2	1.0	Glycogen degradation	True	4.03e+05	1.58	1.58	1.62
18	Maltotriose	Carbon	2	AT_H2O2_2_4	AT_H2O2_2	1.0	Glycogen degradation	True	2.711e+06	4.33	4.33	2.901
19	Maltotetraose	Carbon	2	AT_H2O2_2_4	AT_H2O2_2	1.0	Glycogen degradation	True	3.88e+05	1.526	1.526	1.716
232	SAH	Amino acid	2	AT_H2O2_2_4	AT_H2O2_2	1.0	SAM metabolism	True	9592	-3.812	-3.812	-0.5862
74	Asp	Amino acid	2	AT_H2O2_2_4	AT_H2O2_2	1.0	Proteinogenic amino acids	True	1.863e+06	3.789	3.789	-1.87
129	5-Aminovalerate	Amino acid	2	AT_H2O2_2_4	AT_H2O2_2	0.666666666666667	Amino acids degradation intermediates	False			-1.567	-0.7538
254	Gly-Val	Amino acid	2	AT_H2O2_2_4	AT_H2O2_2	1.0	Dipeptides	True	2.12e+05	0.6534	0.6534	0.7271
291	gamma-Glu-Leu	Amino acid	2	AT_H2O2_2_4	AT_H2O2_2	0.833333333333333	Gamma-glutamyl dipeptides	True	1.348e+05	0	0	1.082
173	Met Sulfoxide	Amino acid	2	AT_H2O2_2_4	AT_H2O2_2	1.0	Amino acid derivatives	True	1.756e+05	0.3817	0.3817	0.5205
43	Glucose	Carbon	2	AT_H2O2_2_4	AT_H2O2_2	1.0	Glycolysis, GNG	True	2.555e+07	7.567	7.567	0.7473
249	Gly-Gly	Amino acid	2	AT_H2O2_2_4	AT_H2O2_2	0.666666666666667	Dipeptides	False			-1.272	-1.489

Metabolite ID	Name	Super Pathway	Dataset	Sample ID	Group ID	Detection Fraction	Pathway	Detected	Raw Intensity	Log2 Norm Intensity	Norm Imputed	Log2 Ctrl Norm
169	2-OH-Butyrate	Amino acid	2	AT_H2O2_2_4	AT_H2O2_2	0.833333333333333	Amino acid derivatives	True	5.998e+04	-1.168	-1.168	0.266
98	3-Methyl-2-Oxobutyrate	Amino acid	2	AT_H2O2_2_4	AT_H2O2_2	0.666666666666667	Amino acids biosynthesis intermediates	True	3.445e+04	-1.968	-1.968	1.791
100	4-Me-2-Oxo-Pentanoate	Amino acid	2	AT_H2O2_2_4	AT_H2O2_2	1.0	Amino acids biosynthesis intermediates	True	6.248e+04	-1.109	-1.109	2.193
253	Gly-Pro	Amino acid	2	AT_H2O2_2_4	AT_H2O2_2	0.833333333333333	Dipeptides	True	1.394e+05	0.04859	0.04859	0.3697
247	Asp-Phe	Amino acid	2	AT_H2O2_2_4	AT_H2O2_2	1.0	Dipeptides	True	6.795e+04	-0.9878	-0.9878	-0.0465
212	N-Ac-Asp	Amino acid	2	AT_H2O2_2_4	AT_H2O2_2	0.5	N-acetylated amino acids	False			-4.689	-0.1861
720	1-Me-Nicotinamide	Cofactor	2	AT_H2O2_2_4	AT_H2O2_2	1.0	Derivatives of NA, nicotinamide and NAD	True	1.447e+06	3.425	3.425	-0.2788
70	Creatine	Carbon	2	AT_H2O2_2_4	AT_H2O2_2	1.0	Creatine energy storage	True	6.385e+06	5.566	5.566	-0.214
309	Glutathione, Oxidized	Amino acid	2	AT_H2O2_2_4	AT_H2O2_2	1.0	Glutathione	True	2.309e+06	4.099	4.099	0.1501
44	Glucose 6-P	Carbon	2	AT_H2O2_2_4	AT_H2O2_2	1.0	Glycolysis, GNG	True	8.119e+04	-0.7311	-0.7311	-1.546
24	Fructose	Carbon	2	AT_H2O2_2_4	AT_H2O2_2	1.0	Sugars and sugar alcohols	True	5.064e+06	5.232	5.232	1.871
85	Cys	Amino acid	2	AT_H2O2_2_4	AT_H2O2_2	1.0	Proteinogenic amino acids	True	1.075e+05	-0.3255	-0.3255	0.2664
704	NADH	Cofactor	2	AT_H2O2_2_4	AT_H2O2_2	0.166666666666667	Cofactors	False			-4.313	-2.383
275	Thr-Phe	Amino acid	2	AT_H2O2_2_4	AT_H2O2_2	0.666666666666667	Dipeptides	True	6.749e+04	-0.9977	-0.9977	-0.06412
738	Pyridoxate	Cofactor	2	AT_H2O2_2_4	AT_H2O2_2	1.0	PLP biosynthesis and salvage	True	2.063e+04	-2.708	-2.708	1.957
177	3-(4-OH-Phenyl)Lactate	Amino acid	2	AT_H2O2_2_4	AT_H2O2_2	1.0	Amino acid derivatives	True	1.511e+04	-3.157	-3.157	0.7962
206	Trans-4-OH-Pro	Amino acid	2	AT_H2O2_2_4	AT_H2O2_2	1.0	Amino acid derivatives	True	2.293e+05	0.7667	0.7667	0.9623
329	AMP	Nucleotide	2	AT_H2O2_2_4	AT_H2O2_2	0.5	Purine nucleotides	False			-2.644	-3.064
345	Guanine	Nucleotide	2	AT_H2O2_2_4	AT_H2O2_2	1.0	Purine bases	True	8.089e+05	2.586	2.586	1.004
271	pyroGlu-Val	Amino acid	2	AT_H2O2_2_4	AT_H2O2_2	0.666666666666667	Dipeptides	True	1.208e+04	-3.479	-3.479	0.1623
279	Val-Glu	Amino acid	2	AT_H2O2_2_4	AT_H2O2_2	0.666666666666667	Dipeptides	True	5.631e+04	-1.259	-1.259	-0.4288

Metabolite ID	Name	Super Pathway	Dataset	Sample ID	Group ID	Detection Fraction	Pathway	Detected	Raw Intensity	Log2 Norm Intensity	Norm Imputed	Log2 Ctrl Norm
183	Phenol Sulfate	Amino acid	2	AT_H2O2_2_4	AT_H2O2_2	0.833333333333333	Amino acid derivatives	True	1.212e+05	-0.1528	-0.1528	3.574
740	3-Dehydrocarnitine	Cofactor	2	AT_H2O2_2_4	AT_H2O2_2	0.5	Carnitine biosynthesis	True	9.1e+04	-0.5664	-0.5664	-0.2328
145	Pyro-Gln	Amino acid	2	AT_H2O2_2_4	AT_H2O2_2	1.0	Amino acid derivatives	True	2.515e+05	0.9004	0.9004	0.5544
197	C-Glycosyl-Trp	Amino acid	2	AT_H2O2_2_4	AT_H2O2_2	1.0	Amino acid derivatives	True	1.144e+05	-0.2359	-0.2359	-0.09227
718	Nicotinamide Riboside	Cofactor	2	AT_H2O2_2_4	AT_H2O2_2	0.5	NAD biosynthesis	True	9.845e+04	-0.4529	-0.4529	1.351
295	gamma-Glu-Phe	Amino acid	2	AT_H2O2_2_4	AT_H2O2_2	0.666666666666667	Gamma-glutamyl dipeptides	True	5.093e+04	-1.404	-1.404	-1.302
399	Pseudouridine	Nucleotide	2	AT_H2O2_2_4	AT_H2O2_2	1.0	Pyrimidine derivatives in RNAs	True	7.654e+04	-0.8161	-0.8161	3.425
375	UTP	Nucleotide	2	AT_H2O2_2_4	AT_H2O2_2	0.0	Pyrimidine nucleotides	False			-5.966	-0.5235
20	Erythronate	Carbon	2	AT_H2O2_2_4	AT_H2O2_2	1.0	Aminosugar derivatives	True	5.221e+04	-1.368	-1.368	0.07368
151	Phenylacetylglycine	Amino acid	2	AT_H2O2_2_4	AT_H2O2_2	1.0	Amino acid derivatives	True	5.242e+05	1.96	1.96	6.381
252	Gly-Phe	Amino acid	2	AT_H2O2_2_4	AT_H2O2_2	0.666666666666667	Dipeptides	True	3.815e+05	1.501	1.501	1.397
251	Gly-Leu	Amino acid	2	AT_H2O2_2_4	AT_H2O2_2	1.0	Dipeptides	True	2.904e+05	1.108	1.108	0.7452
290	gamma-Glu-Ile	Amino acid	2	AT_H2O2_2_4	AT_H2O2_2	0.666666666666667	Gamma-glutamyl dipeptides	True	6.282e+04	-1.101	-1.101	0.43
316	Ophthalmate	Amino acid	2	AT_H2O2_2_4	AT_H2O2_2	1.0	Oxidative stress markers	True	9.272e+04	-0.5394	-0.5394	-0.7936
208	Pro-OH-Pro	Amino acid	2	AT_H2O2_2_4	AT_H2O2_2	1.0	Amino acid derivatives	True	1.64e+05	0.2835	0.2835	-0.1762
352	3'-AMP	Nucleotide	2	AT_H2O2_2_4	AT_H2O2_2	1.0	Purine derivatives in signaling	True	2.88e+04	-2.226	-2.226	-0.9415
314	Cys-Glutathione Disulfide	Amino acid	2	AT_H2O2_2_4	AT_H2O2_2	1.0	Oxidative stress markers	True	1.007e+05	-0.4202	-0.4202	0.9452
39	Threitol	Carbon	2	AT_H2O2_2_4	AT_H2O2_2	0.5	Sugars and sugar alcohols	False			-4.395	-0.3698
31	Ribulose/Xylulose	Carbon	2	AT_H2O2_2_4	AT_H2O2_2	0.0	Sugars and sugar alcohols	False			-5.004	-0.09716
48	DHAP	Carbon	2	AT_H2O2_2_4	AT_H2O2_2	1.0	Glycolysis, GNG	True	1.307e+05	-0.0437	-0.0437	-1.802
182	P-Cresol Sulfate	Amino acid	2	AT_H2O2_2_4	AT_H2O2_2	1.0	Amino acid derivatives	True	6.528e+04	-1.046	-1.046	2.292
250	Gly-Ile	Amino acid	2	AT_H2O2_2_4	AT_H2O2_2	0.666666666666667	Dipeptides	True	1.865e+05	0.4689	0.4689	1.893

Metabolite ID	Name	Super Pathway	Dataset	Sample ID	Group ID	Detection Fraction	Pathway	Detected	Raw Intensity	Log2 Norm Intensity	Norm Imputed	Log2 Ctrl Norm
286	gamma-Glu-Glu	Amino acid	2	AT_H2O2_2_4	AT_H2O2_2	1.0	Gamma-glutamyl dipeptides	True	9.206e+04	-0.5498	-0.5498	-0.3192
264	Leu-Leu	Amino acid	2	AT_H2O2_2_4	AT_H2O2_2	0.5	Dipeptides	False			-2.222	-1.148
203	DiMe-Arg	Amino acid	2	AT_H2O2_2_4	AT_H2O2_2	1.0	Amino acid derivatives	True	4.034e+05	1.582	1.582	0.5075
47	Fructose 1,6-PP, Glucose 1,6-PP	Carbon	2	AT_H2O2_2_4	AT_H2O2_2	1.0	Glycolysis, GNG	True	2.322e+04	-2.537	-2.537	-2.247
224	N-Ac-Ser	Amino acid	2	AT_H2O2_2_4	AT_H2O2_2	1.0	N-acetylated amino acids	True	4.585e+04	-1.555	-1.555	-2.426
244	Ala-Leu	Amino acid	2	AT_H2O2_2_4	AT_H2O2_2	0.166666666666667	Dipeptides	False			-1.778	-2.118
304	Cyclo(Phe-Pro)	Amino acid	2	AT_H2O2_2_4	AT_H2O2_2	0.0	Cyclic dipeptides	False			-1.192	-0.7201
302	Cyclo(Glu-Glu)	Amino acid	2	AT_H2O2_2_4	AT_H2O2_2	0.666666666666667	Cyclic dipeptides	False			-2.527	-1.355
303	Cyclo(Leu-Pro)	Amino acid	2	AT_H2O2_2_4	AT_H2O2_2	0.0	Cyclic dipeptides	False			-0.4297	-0.7026
390	2',3'-cUMP	Nucleotide	2	AT_H2O2_2_4	AT_H2O2_2	1.0	Pyrimidine derivatives in signaling	True	2.8e+04	-2.267	-2.267	-1.138
68	Ribulose 5-P / Xylulose 5-P	Carbon	2	AT_H2O2_2_4	AT_H2O2_2	1.0	Pentose phosphate pathway (PPP)	True	6.814e+04	-0.9837	-0.9837	-1.753
388	2',3'-cCMP	Nucleotide	2	AT_H2O2_2_4	AT_H2O2_2	1.0	Pyrimidine derivatives in signaling	True	3.127e+04	-2.108	-2.108	-2.361
33	Arabitol/Xylitol	Carbon	2	AT_H2O2_2_4	AT_H2O2_2	0.166666666666667	Sugars and sugar alcohols	False			-3.559	-0.05408
268	Phe-Phe	Amino acid	2	AT_H2O2_2_4	AT_H2O2_2	0.0	Dipeptides	False			-2.762	-1.674
245	Ala-Phe	Amino acid	2	AT_H2O2_2_4	AT_H2O2_2	0.666666666666667	Dipeptides	True	4.99e+04	-1.433	-1.433	0.02222
373	UMP	Nucleotide	2	AT_H2O2_2_4	AT_H2O2_2	0.0	Pyrimidine nucleotides	False			-3.442	-1.402
282	Val-Leu	Amino acid	2	AT_H2O2_2_4	AT_H2O2_2	0.666666666666667	Dipeptides	True	1.097e+05	-0.2968	-0.2968	0.07199
258	Ile-Gly	Amino acid	2	AT_H2O2_2_4	AT_H2O2_2	0.666666666666667	Dipeptides	True	6.612e+04	-1.027	-1.027	-1.442
259	Ile-Ser	Amino acid	2	AT_H2O2_2_4	AT_H2O2_2	0.0	Dipeptides	False			-3.192	-2.329
269	Phe-Ser	Amino acid	2	AT_H2O2_2_4	AT_H2O2_2	0.333333333333333	Dipeptides	False			-3.197	-1.882
277	Tyr-Ala	Amino acid	2	AT_H2O2_2_4	AT_H2O2_2	0.833333333333333	Dipeptides	True	2.17e+05	0.6876	0.6876	0.1175
257	Ile-Gln	Amino acid	2	AT_H2O2_2_4	AT_H2O2_2	0.166666666666667	Dipeptides	False			-2.625	-1.706
261	Leu-Glu	Amino acid	2	AT_H2O2_2_4	AT_H2O2_2	1.0	Dipeptides	True	8.326e+04	-0.6947	-0.6947	-1.485

Metabolite ID	Name	Super Pathway	Dataset	Sample ID	Group ID	Detection Fraction	Pathway	Detected	Raw Intensity	Log2 Norm Intensity	Norm Imputed	Log2 Ctrl Norm
263	Leu-Gly	Amino acid	2	AT_H2O2_2_4	AT_H2O2_2	1.0	Dipeptides	True	3.71e+04	-1.861	-1.861	-2.568
256	Ile-Ala	Amino acid	2	AT_H2O2_2_4	AT_H2O2_2	0.666666666666667	Dipeptides	True	1.108e+05	-0.2825	-0.2825	-0.2408
274	Thr-Leu	Amino acid	2	AT_H2O2_2_4	AT_H2O2_2	0.833333333333333	Dipeptides	True	2.058e+05	0.6108	0.6108	-0.2403
273	Ser-Phe	Amino acid	2	AT_H2O2_2_4	AT_H2O2_2	0.833333333333333	Dipeptides	True	3.394e+04	-1.989	-1.989	-0.7674
272	Ser-Leu	Amino acid	2	AT_H2O2_2_4	AT_H2O2_2	1.0	Dipeptides	True	1.319e+05	-0.03131	-0.03131	-0.7701
246	Asp-Leu	Amino acid	2	AT_H2O2_2_4	AT_H2O2_2	1.0	Dipeptides	True	1.417e+05	0.07206	0.07206	0.1461
76	Gln	Amino acid	2	AT_H2O2_2_5	AT_H2O2_2	1.0	Proteinogenic amino acids	True	8.952e+06	6.523	6.523	-0.4294
89	Trp	Amino acid	2	AT_H2O2_2_5	AT_H2O2_2	1.0	Proteinogenic amino acids	True	3.62e+06	5.216	5.216	-0.7502
723	beta-Ala	Cofactor	2	AT_H2O2_2_5	AT_H2O2_2	1.0	Coenzyme A biosynthesis	True	3.243e+04	-1.586	-1.586	-1.912
75	Glu	Amino acid	2	AT_H2O2_2_5	AT_H2O2_2	1.0	Proteinogenic amino acids	True	4.567e+06	5.551	5.551	-0.3964
80	His	Amino acid	2	AT_H2O2_2_5	AT_H2O2_2	1.0	Proteinogenic amino acids	True	6.839e+04	-0.5097	-0.5097	-0.7238
82	Leu	Amino acid	2	AT_H2O2_2_5	AT_H2O2_2	1.0	Proteinogenic amino acids	True	1.965e+07	7.657	7.657	-0.5031
87	Phe	Amino acid	2	AT_H2O2_2_5	AT_H2O2_2	1.0	Proteinogenic amino acids	True	1.561e+07	7.325	7.325	-0.5669
236	Spermidine	Amino acid	2	AT_H2O2_2_5	AT_H2O2_2	1.0	Polyamines	True	1.292e+05	0.4085	0.4085	-2.419
73	Asn	Amino acid	2	AT_H2O2_2_5	AT_H2O2_2	1.0	Proteinogenic amino acids	True	9.003e+04	-0.1131	-0.1131	-2.862
243	Creatinine	Amino acid	2	AT_H2O2_2_5	AT_H2O2_2	1.0	Creatine degradation	True	4.479e+05	2.201	2.201	0.6311
376	Cytidine	Nucleotide	2	AT_H2O2_2_5	AT_H2O2_2	1.0	Pyrimidine nucleosides	True	6.456e+05	2.729	2.729	3.314
41	Lactate	Carbon	2	AT_H2O2_2_5	AT_H2O2_2	1.0	Respiratory carbon sources	True	1.865e+07	7.581	7.581	-0.2409
93	3-P-Ser	Amino acid	2	AT_H2O2_2_5	AT_H2O2_2	0.5	Amino acids biosynthesis intermediates	False			-4.967	-1.709
343	Adenine	Nucleotide	2	AT_H2O2_2_5	AT_H2O2_2	0.5	Purine bases	False			-3.45	-1.986
336	Adenosine	Nucleotide	2	AT_H2O2_2_5	AT_H2O2_2	1.0	Purine nucleosides	True	2.437e+05	1.324	1.324	-1.814
29	Raffinose	Carbon	2	AT_H2O2_2_5	AT_H2O2_2	0.166666666666667	Sugars and sugar alcohols	False			-5.058	-2.878
717	Nicotinamide	Cofactor	2	AT_H2O2_2_5	AT_H2O2_2	1.0	NAD biosynthesis	True	4.752e+05	2.287	2.287	-0.1098

Metabolite ID	Name	Super Pathway	Dataset	Sample ID	Group ID	Detection Fraction	Pathway	Detected	Raw Intensity	Log2 Norm Intensity	Norm Imputed	Log2 Ctrl Norm
51	PEP	Carbon	2	AT_H2O2_2_5	AT_H2O2_2	1.0	Glycolysis, GNG	True	5.77e+04	-0.755	-0.755	0.317
52	Pyruvate	Carbon	2	AT_H2O2_2_5	AT_H2O2_2	1.0	Glycolysis, GNG	True	1.086e+04	-3.165	-3.165	-0.7914
237	Spermine	Amino acid	2	AT_H2O2_2_5	AT_H2O2_2	0.666666666666667	Polyamines	False			0.9044	-2.484
385	Uracil	Nucleotide	2	AT_H2O2_2_5	AT_H2O2_2	1.0	Pyrimidine bases	True	4.581e+04	-1.088	-1.088	0.6392
377	Uridine	Nucleotide	2	AT_H2O2_2_5	AT_H2O2_2	1.0	Pyrimidine nucleosides	True	3.869e+05	1.99	1.99	-0.892
112	trans-Urocanate	Amino acid	2	AT_H2O2_2_5	AT_H2O2_2	1.0	Amino acids degradation intermediates	True	1.359e+05	0.4809	0.4809	3.599
737	Pyridoxine (Vitamin B6)	Cofactor	2	AT_H2O2_2_5	AT_H2O2_2	1.0	PLP biosynthesis and salvage	True	1.457e+06	3.903	3.903	0.6034
348	Allantoin	Nucleotide	2	AT_H2O2_2_5	AT_H2O2_2	1.0	Purine degradation	True	2.524e+04	-1.948	-1.948	0.8815
335	Inosine	Nucleotide	2	AT_H2O2_2_5	AT_H2O2_2	1.0	Purine nucleosides	True	2.227e+05	1.193	1.193	-1.668
81	Ile	Amino acid	2	AT_H2O2_2_5	AT_H2O2_2	1.0	Proteinogenic amino acids	True	1.848e+07	7.568	7.568	-0.2871
72	Ala	Amino acid	2	AT_H2O2_2_5	AT_H2O2_2	1.0	Proteinogenic amino acids	True	8.532e+06	6.453	6.453	-1.281
79	Thr	Amino acid	2	AT_H2O2_2_5	AT_H2O2_2	1.0	Proteinogenic amino acids	True	1.696e+06	4.123	4.123	-1.84
88	Tyr	Amino acid	2	AT_H2O2_2_5	AT_H2O2_2	1.0	Proteinogenic amino acids	True	7.947e+06	6.351	6.351	-0.4917
84	Lys	Amino acid	2	AT_H2O2_2_5	AT_H2O2_2	1.0	Proteinogenic amino acids	True	1.463e+06	3.909	3.909	0.1901
86	Met	Amino acid	2	AT_H2O2_2_5	AT_H2O2_2	1.0	Proteinogenic amino acids	True	3.358e+06	5.108	5.108	-0.6791
61	Malate	Carbon	2	AT_H2O2_2_5	AT_H2O2_2	1.0	TCA cycle	True	6.928e+04	-0.4911	-0.4911	-1.388
235	Putrescine	Amino acid	2	AT_H2O2_2_5	AT_H2O2_2	0.0	Polyamines	False			-3.898	-2.331
49	3-P-Glycerate	Carbon	2	AT_H2O2_2_5	AT_H2O2_2	1.0	Glycolysis, GNG	True	4.455e+05	2.194	2.194	-0.2686
139	GABA	Amino acid	2	AT_H2O2_2_5	AT_H2O2_2	0.0	Amino acid derivatives	False			-4.963	-1.493
189	Kynurenate	Amino acid	2	AT_H2O2_2_5	AT_H2O2_2	0.833333333333333	Amino acid derivatives	True	4433	-4.457	-4.457	0.7304
234	5-Me-Thioadenosine	Amino acid	2	AT_H2O2_2_5	AT_H2O2_2	1.0	SAM metabolism	True	5.378e+04	-0.8564	-0.8564	-0.7604
59	Succinate	Carbon	2	AT_H2O2_2_5	AT_H2O2_2	0.833333333333333	TCA cycle	True	3.638e+04	-1.42	-1.42	0.6195
133	Ornithine	Amino acid	2	AT_H2O2_2_5	AT_H2O2_2	1.0	Amino acids degradation intermediates	True	5.809e+05	2.577	2.577	0.0555

Metabolite ID	Name	Super Pathway	Dataset	Sample ID	Group ID	Detection Fraction	Pathway	Detected	Raw Intensity	Log2 Norm Intensity	Norm Imputed	Log2 Ctrl Norm
313	5-Oxoproline	Amino acid	2	AT_H2O2_2_5	AT_H2O2_2	1.0	Glutathione derivatives	True	1.124e+06	3.529	3.529	1.525
724	Pantothenate	Cofactor	2	AT_H2O2_2_5	AT_H2O2_2	1.0	Coenzyme A biosynthesis	True	8.089e+05	3.054	3.054	-0.0591
30	Sucrose	Carbon	2	AT_H2O2_2_5	AT_H2O2_2	1.0	Sugars and sugar alcohols	True	3.133e+05	1.686	1.686	-0.6908
122	3-OH-Isobutyrate	Amino acid	2	AT_H2O2_2_5	AT_H2O2_2	0.5	Amino acids degradation intermediates	False			-4.628	0
241	4-Acetamidobutanoate	Amino acid	2	AT_H2O2_2_5	AT_H2O2_2	1.0	Polyamine derivatives	True	1.231e+05	0.3389	0.3389	1.846
55	Citrate	Carbon	2	AT_H2O2_2_5	AT_H2O2_2	1.0	TCA cycle	True	4.532e+05	2.219	2.219	-0.8159
338	Guanosine	Nucleotide	2	AT_H2O2_2_5	AT_H2O2_2	1.0	Purine nucleosides	True	4.492e+05	2.206	2.206	-0.1547
170	2-Amino-Butyrate	Amino acid	2	AT_H2O2_2_5	AT_H2O2_2	1.0	Amino acid derivatives	True	3.222e+05	1.726	1.726	0.5025
209	N-Ac-Ala	Amino acid	2	AT_H2O2_2_5	AT_H2O2_2	0.166666666666667	N-acetylated amino acids	False			-4.401	-1.133
221	N-Ac-Met	Amino acid	2	AT_H2O2_2_5	AT_H2O2_2	1.0	N-acetylated amino acids	True	1.506e+04	-2.693	-2.693	-1.543
22	N-Ac-Neuraminate	Carbon	2	AT_H2O2_2_5	AT_H2O2_2	0.833333333333333	Aminosugar derivatives	True	3.019e+04	-1.69	-1.69	-1.476
346	Urate	Nucleotide	2	AT_H2O2_2_5	AT_H2O2_2	0.833333333333333	Purine degradation	False			-4.123	-0.4311
90	Arg	Amino acid	2	AT_H2O2_2_5	AT_H2O2_2	1.0	Proteinogenic amino acids	True	2.832e+06	4.862	4.862	0.08018
60	Fumarate	Carbon	2	AT_H2O2_2_5	AT_H2O2_2	1.0	TCA cycle	True	2.376e+04	-2.035	-2.035	-1.879
78	Ser	Amino acid	2	AT_H2O2_2_5	AT_H2O2_2	1.0	Proteinogenic amino acids	True	3.781e+06	5.279	5.279	-1.548
83	Val	Amino acid	2	AT_H2O2_2_5	AT_H2O2_2	1.0	Proteinogenic amino acids	True	1.321e+07	7.084	7.084	-0.15
734	Pyridoxal	Cofactor	2	AT_H2O2_2_5	AT_H2O2_2	0.666666666666667	PLP biosynthesis and salvage	True	1.231e+05	0.3384	0.3384	1.333
136	Urea	Amino acid	2	AT_H2O2_2_5	AT_H2O2_2	1.0	Amino acids degradation intermediates	True	4.911e+05	2.334	2.334	0.6895
742	Folate	Cofactor	2	AT_H2O2_2_5	AT_H2O2_2	1.0	Folate metabolism	True	9.737e+04	0	0	0.517
729	Riboflavin (Vitamin B2)	Cofactor	2	AT_H2O2_2_5	AT_H2O2_2	0.833333333333333	Flavine biosynthesis	False			-2.318	-0.7493
91	Pro	Amino acid	2	AT_H2O2_2_5	AT_H2O2_2	1.0	Proteinogenic amino acids	True	4.292e+06	5.462	5.462	-1.259
308	Glutathione, Reduced	Amino acid	2	AT_H2O2_2_5	AT_H2O2_2	1.0	Glutathione	True	1.465e+06	3.911	3.911	-2.544

Metabolite ID	Name	Super Pathway	Dataset	Sample ID	Group ID	Detection Fraction	Pathway	Detected	Raw Intensity	Log2 Norm Intensity	Norm Imputed	Log2 Ctrl Norm
706	FAD	Cofactor	2	AT_H2O2_2_5	AT_H2O2_2	0.5	Cofactors	False			-5.096	0
299	gamma-Glu-Tyr	Amino acid	2	AT_H2O2_2_5	AT_H2O2_2	0.666666666666667	Gamma-glutamyl dipeptides	False			-3.049	-1.881
705	Coenzyme A	Cofactor	2	AT_H2O2_2_5	AT_H2O2_2	1.0	Cofactors	True	2092	-5.541	-5.541	-1.834
342	Hypoxanthine	Nucleotide	2	AT_H2O2_2_5	AT_H2O2_2	1.0	Purine bases	True	6.301e+04	-0.6279	-0.6279	-0.6297
344	Xanthine	Nucleotide	2	AT_H2O2_2_5	AT_H2O2_2	0.833333333333333	Purine bases	True	1.873e+04	-2.378	-2.378	-0.1389
703	NAD+	Cofactor	2	AT_H2O2_2_5	AT_H2O2_2	1.0	Cofactors	True	2.356e+05	1.275	1.275	-2.284
731	Thiamin (Vitamin B1)	Cofactor	2	AT_H2O2_2_5	AT_H2O2_2	1.0	TPP biosynthesis	True	8.225e+04	-0.2435	-0.2435	-0.03735
102	2-Aminoadipate	Amino acid	2	AT_H2O2_2_5	AT_H2O2_2	1.0	Amino acids biosynthesis intermediates	True	5.827e+04	-0.7407	-0.7407	-1.497
77	Gly	Amino acid	2	AT_H2O2_2_5	AT_H2O2_2	1.0	Proteinogenic amino acids	True	3.986e+06	5.355	5.355	-1.873
45	Fructose-6-P	Carbon	2	AT_H2O2_2_5	AT_H2O2_2	0.833333333333333	Glycolysis, GNG	True	2.598e+04	-1.906	-1.906	-0.4705
36	Ribose	Carbon	2	AT_H2O2_2_5	AT_H2O2_2	0.666666666666667	Sugars and sugar alcohols	False			-3.346	-2.349
4	GlcNAc 6-P	Carbon	2	AT_H2O2_2_5	AT_H2O2_2	1.0	Aminosugar biosynthesis	True	3.493e+04	-1.479	-1.479	-1.839
188	Kynurenine	Amino acid	2	AT_H2O2_2_5	AT_H2O2_2	0.833333333333333	Amino acid derivatives	False			-1.614	-1.6
63	6-P-Gluconate	Carbon	2	AT_H2O2_2_5	AT_H2O2_2	1.0	Pentose phosphate pathway (PPP)	True	4.002e+04	-1.283	-1.283	-0.2374
710	Carnitine	Cofactor	2	AT_H2O2_2_5	AT_H2O2_2	1.0	Cofactors	True	1.292e+05	0.4085	0.4085	-2.123
725	P-Pantetheine	Cofactor	2	AT_H2O2_2_5	AT_H2O2_2	0.833333333333333	Coenzyme A biosynthesis	True	6050	-4.009	-4.009	0.8039
110	N-alpha-Ac-Ornithine	Amino acid	2	AT_H2O2_2_5	AT_H2O2_2	1.0	Amino acids biosynthesis intermediates	True	1.644e+05	0.756	0.756	-0.2181
116	3-Me-2-Oxo-Valerate	Amino acid	2	AT_H2O2_2_5	AT_H2O2_2	1.0	Amino acids degradation intermediates	True	2.252e+04	-2.112	-2.112	1.462
155	4-Guanidinobutanoate	Amino acid	2	AT_H2O2_2_5	AT_H2O2_2	0.666666666666667	Amino acid derivatives	True	4.432e+04	-1.135	-1.135	-0.416
310	S-Lactoyl-Glutathione	Amino acid	2	AT_H2O2_2_5	AT_H2O2_2	0.166666666666667	Glutathione derivatives	False			-4.634	-0.962
34	Ribitol	Carbon	2	AT_H2O2_2_5	AT_H2O2_2	1.0	Sugars and sugar alcohols	True	4.31e+04	-1.176	-1.176	0.4031
707	FMN	Cofactor	2	AT_H2O2_2_5	AT_H2O2_2	0.666666666666667	Cofactors	False			-4.56	-1.139

Metabolite ID	Name	Super Pathway	Dataset	Sample ID	Group ID	Detection Fraction	Pathway	Detected	Raw Intensity	Log2 Norm Intensity	Norm Imputed	Log2 Ctrl Norm
17	Maltose	Carbon	2	AT_H2O2_2_5	AT_H2O2_2	1.0	Glycogen degradation	True	2.632e+05	1.435	1.435	1.474
18	Maltotriose	Carbon	2	AT_H2O2_2_5	AT_H2O2_2	1.0	Glycogen degradation	True	9.456e+05	3.28	3.28	1.851
19	Maltotetraose	Carbon	2	AT_H2O2_2_5	AT_H2O2_2	1.0	Glycogen degradation	True	1.578e+05	0.6966	0.6966	0.8871
232	SAH	Amino acid	2	AT_H2O2_2_5	AT_H2O2_2	1.0	SAM metabolism	True	1.143e+04	-3.09	-3.09	0.1359
74	Asp	Amino acid	2	AT_H2O2_2_5	AT_H2O2_2	1.0	Proteinogenic amino acids	True	1.096e+06	3.493	3.493	-2.167
129	5-Aminovalerate	Amino acid	2	AT_H2O2_2_5	AT_H2O2_2	0.666666666666667	Amino acids degradation intermediates	False			-1.567	-0.7538
254	Gly-Val	Amino acid	2	AT_H2O2_2_5	AT_H2O2_2	1.0	Dipeptides	True	7.863e+04	-0.3085	-0.3085	-0.2347
291	gamma-Glu-Leu	Amino acid	2	AT_H2O2_2_5	AT_H2O2_2	0.833333333333333	Gamma-glutamyl dipeptides	False			-2.038	-0.9559
173	Met Sulfoxide	Amino acid	2	AT_H2O2_2_5	AT_H2O2_2	1.0	Amino acid derivatives	True	2.531e+05	1.378	1.378	1.517
43	Glucose	Carbon	2	AT_H2O2_2_5	AT_H2O2_2	1.0	Glycolysis, GNG	True	1.793e+07	7.525	7.525	0.7052
249	Gly-Gly	Amino acid	2	AT_H2O2_2_5	AT_H2O2_2	0.666666666666667	Dipeptides	True	6.112e+04	-0.672	-0.672	-0.8892
169	2-OH-Butyrate	Amino acid	2	AT_H2O2_2_5	AT_H2O2_2	0.833333333333333	Amino acid derivatives	True	5.755e+04	-0.7588	-0.7588	0.6751
98	3-Methyl-2-Oxobutyrate	Amino acid	2	AT_H2O2_2_5	AT_H2O2_2	0.666666666666667	Amino acids biosynthesis intermediates	False			-3.759	0
100	4-Me-2-Oxo-Pentanoate	Amino acid	2	AT_H2O2_2_5	AT_H2O2_2	1.0	Amino acids biosynthesis intermediates	True	3.585e+04	-1.442	-1.442	1.86
253	Gly-Pro	Amino acid	2	AT_H2O2_2_5	AT_H2O2_2	0.833333333333333	Dipeptides	True	1.381e+05	0.5045	0.5045	0.8256
247	Asp-Phe	Amino acid	2	AT_H2O2_2_5	AT_H2O2_2	1.0	Dipeptides	True	2.422e+04	-2.008	-2.008	-1.066
212	N-Ac-Asp	Amino acid	2	AT_H2O2_2_5	AT_H2O2_2	0.5	N-acetylated amino acids	False			-4.689	-0.1861
720	1-Me-Nicotinamide	Cofactor	2	AT_H2O2_2_5	AT_H2O2_2	1.0	Derivatives of NA, nicotinamide and NAD	True	4.683e+05	2.266	2.266	-1.438
70	Creatine	Carbon	2	AT_H2O2_2_5	AT_H2O2_2	1.0	Creatine energy storage	True	3.999e+06	5.36	5.36	-0.4202
309	Glutathione, Oxidized	Amino acid	2	AT_H2O2_2_5	AT_H2O2_2	1.0	Glutathione	True	1.301e+06	3.74	3.74	-0.2082
44	Glucose 6-P	Carbon	2	AT_H2O2_2_5	AT_H2O2_2	1.0	Glycolysis, GNG	True	8.725e+04	-0.1584	-0.1584	-0.9731

Metabolite ID	Name	Super Pathway	Dataset	Sample ID	Group ID	Detection Fraction	Pathway	Detected	Raw Intensity	Log2 Norm Intensity	Norm Imputed	Log2 Ctrl Norm
24	Fructose	Carbon	2	AT_H2O2_2_5	AT_H2O2_2	1.0	Sugars and sugar alcohols	True	2.616e+06	4.748	4.748	1.387
85	Cys	Amino acid	2	AT_H2O2_2_5	AT_H2O2_2	1.0	Proteinogenic amino acids	True	3.421e+04	-1.509	-1.509	-0.9174
704	NADH	Cofactor	2	AT_H2O2_2_5	AT_H2O2_2	0.166666666666667	Cofactors	False			-4.313	-2.383
275	Thr-Phe	Amino acid	2	AT_H2O2_2_5	AT_H2O2_2	0.666666666666667	Dipeptides	True	5.365e+04	-0.8598	-0.8598	0.07376
738	Pyridoxate	Cofactor	2	AT_H2O2_2_5	AT_H2O2_2	1.0	PLP biosynthesis and salvage	True	8572	-3.506	-3.506	1.159
177	3-(4-OH-Phenyl)Lactate	Amino acid	2	AT_H2O2_2_5	AT_H2O2_2	1.0	Amino acid derivatives	True	1.038e+04	-3.23	-3.23	0.7233
206	Trans-4-OH-Pro	Amino acid	2	AT_H2O2_2_5	AT_H2O2_2	1.0	Amino acid derivatives	True	1.869e+05	0.9407	0.9407	1.136
329	AMP	Nucleotide	2	AT_H2O2_2_5	AT_H2O2_2	0.5	Purine nucleotides	False			-2.644	-3.064
345	Guanine	Nucleotide	2	AT_H2O2_2_5	AT_H2O2_2	1.0	Purine bases	True	4.396e+05	2.175	2.175	0.5927
271	pyroGlu-Val	Amino acid	2	AT_H2O2_2_5	AT_H2O2_2	0.666666666666667	Dipeptides	False			-5.212	-1.57
279	Val-Glu	Amino acid	2	AT_H2O2_2_5	AT_H2O2_2	0.666666666666667	Dipeptides	False			-3	-2.17
183	Phenol Sulfate	Amino acid	2	AT_H2O2_2_5	AT_H2O2_2	0.833333333333333	Amino acid derivatives	False			-4.072	-0.3451
740	3-Dehydrocarnitine	Cofactor	2	AT_H2O2_2_5	AT_H2O2_2	0.5	Carnitine biosynthesis	False			-2.199	-1.865
145	Pyro-Gln	Amino acid	2	AT_H2O2_2_5	AT_H2O2_2	1.0	Amino acid derivatives	True	1.723e+05	0.8232	0.8232	0.4772
197	C-Glycosyl-Trp	Amino acid	2	AT_H2O2_2_5	AT_H2O2_2	1.0	Amino acid derivatives	True	3.689e+04	-1.4	-1.4	-1.257
718	Nicotinamide Riboside	Cofactor	2	AT_H2O2_2_5	AT_H2O2_2	0.5	NAD biosynthesis	True	1.144e+05	0.2327	0.2327	2.036
295	gamma-Glu-Phe	Amino acid	2	AT_H2O2_2_5	AT_H2O2_2	0.666666666666667	Gamma-glutamyl dipeptides	False			-1.936	-1.834
399	Pseudouridine	Nucleotide	2	AT_H2O2_2_5	AT_H2O2_2	1.0	Pyrimidine derivatives in RNAs	True	1.517e+04	-2.682	-2.682	1.559
375	UTP	Nucleotide	2	AT_H2O2_2_5	AT_H2O2_2	0.0	Pyrimidine nucleotides	False			-5.966	-0.5235
20	Erythronate	Carbon	2	AT_H2O2_2_5	AT_H2O2_2	1.0	Aminosugar derivatives	True	2.858e+04	-1.769	-1.769	-0.327
151	Phenylacetylglycine	Amino acid	2	AT_H2O2_2_5	AT_H2O2_2	1.0	Amino acid derivatives	True	2.109e+04	-2.207	-2.207	2.215
252	Gly-Phe	Amino acid	2	AT_H2O2_2_5	AT_H2O2_2	0.666666666666667	Dipeptides	True	1.672e+05	0.7804	0.7804	0.6763

Metabolite ID	Name	Super Pathway	Dataset	Sample ID	Group ID	Detection Fraction	Pathway	Detected	Raw Intensity	Log2 Norm Intensity	Norm Imputed	Log2 Ctrl Norm
251	Gly-Leu	Amino acid	2	AT_H2O2_2_5	AT_H2O2_2	1.0	Dipeptides	True	8.541e+04	-0.1891	-0.1891	-0.5517
290	gamma-Glu-Ile	Amino acid	2	AT_H2O2_2_5	AT_H2O2_2	0.666666666666667	Gamma-glutamyl dipeptides	False			-2.961	-1.429
316	Ophthalmate	Amino acid	2	AT_H2O2_2_5	AT_H2O2_2	1.0	Oxidative stress markers	True	2.73e+04	-1.835	-1.835	-2.089
208	Pro-OH-Pro	Amino acid	2	AT_H2O2_2_5	AT_H2O2_2	1.0	Amino acid derivatives	True	1.349e+05	0.4705	0.4705	0.01089
352	3'-AMP	Nucleotide	2	AT_H2O2_2_5	AT_H2O2_2	1.0	Purine derivatives in signaling	True	2.111e+04	-2.205	-2.205	-0.9205
314	Cys-Glutathione Disulfide	Amino acid	2	AT_H2O2_2_5	AT_H2O2_2	1.0	Oxidative stress markers	True	1.289e+05	0.4048	0.4048	1.77
39	Threitol	Carbon	2	AT_H2O2_2_5	AT_H2O2_2	0.5	Sugars and sugar alcohols	True	9491	-3.359	-3.359	0.6661
31	Ribulose/Xylulose	Carbon	2	AT_H2O2_2_5	AT_H2O2_2	0.0	Sugars and sugar alcohols	False			-5.004	-0.09716
48	DHAP	Carbon	2	AT_H2O2_2_5	AT_H2O2_2	1.0	Glycolysis, GNG	True	1.021e+05	0.06855	0.06855	-1.689
182	P-Cresol Sulfate	Amino acid	2	AT_H2O2_2_5	AT_H2O2_2	1.0	Amino acid derivatives	True	8738	-3.478	-3.478	-0.1407
250	Gly-Ile	Amino acid	2	AT_H2O2_2_5	AT_H2O2_2	0.666666666666667	Dipeptides	False			-2.655	-1.231
286	gamma-Glu-Glu	Amino acid	2	AT_H2O2_2_5	AT_H2O2_2	1.0	Gamma-glutamyl dipeptides	True	4.475e+04	-1.122	-1.122	-0.891
264	Leu-Leu	Amino acid	2	AT_H2O2_2_5	AT_H2O2_2	0.5	Dipeptides	True	3.952e+04	-1.301	-1.301	-0.2276
203	DiMe-Arg	Amino acid	2	AT_H2O2_2_5	AT_H2O2_2	1.0	Amino acid derivatives	True	2.601e+05	1.418	1.418	0.3434
47	Fructose 1,6-PP, Glucose 1,6-PP	Carbon	2	AT_H2O2_2_5	AT_H2O2_2	1.0	Glycolysis, GNG	True	2.573e+04	-1.92	-1.92	-1.63
224	N-Ac-Ser	Amino acid	2	AT_H2O2_2_5	AT_H2O2_2	1.0	N-acetylated amino acids	True	5.218e+04	-0.9001	-0.9001	-1.771
244	Ala-Leu	Amino acid	2	AT_H2O2_2_5	AT_H2O2_2	0.166666666666667	Dipeptides	False			-1.778	-2.118
304	Cyclo(Phe-Pro)	Amino acid	2	AT_H2O2_2_5	AT_H2O2_2	0.0	Cyclic dipeptides	False			-1.192	-0.7201
302	Cyclo(Glu-Glu)	Amino acid	2	AT_H2O2_2_5	AT_H2O2_2	0.666666666666667	Cyclic dipeptides	True	1.689e+04	-2.527	-2.527	-1.355
303	Cyclo(Leu-Pro)	Amino acid	2	AT_H2O2_2_5	AT_H2O2_2	0.0	Cyclic dipeptides	False			-0.4297	-0.7026
390	2',3'-cUMP	Nucleotide	2	AT_H2O2_2_5	AT_H2O2_2	1.0	Pyrimidine derivatives in signaling	True	2.305e+04	-2.079	-2.079	-0.9496
68	Ribulose 5-P / Xylulose 5-P	Carbon	2	AT_H2O2_2_5	AT_H2O2_2	1.0	Pentose phosphate pathway (PPP)	True	3.479e+04	-1.485	-1.485	-2.254

Metabolite ID	Name	Super Pathway	Dataset	Sample ID	Group ID	Detection Fraction	Pathway	Detected	Raw Intensity	Log2 Norm Intensity	Norm Imputed	Log2 Ctrl Norm
388	2',3'-cCMP	Nucleotide	2	AT_H2O2_2_5	AT_H2O2_2	1.0	Pyrimidine derivatives in signaling	True	2.029e+04	-2.263	-2.263	-2.516
33	Arabitol/Xylitol	Carbon	2	AT_H2O2_2_5	AT_H2O2_2	0.166666666666667	Sugars and sugar alcohols	False			-3.559	-0.05408
268	Phe-Phe	Amino acid	2	AT_H2O2_2_5	AT_H2O2_2	0.0	Dipeptides	False			-2.762	-1.674
245	Ala-Phe	Amino acid	2	AT_H2O2_2_5	AT_H2O2_2	0.666666666666667	Dipeptides	False			-2.821	-1.366
373	UMP	Nucleotide	2	AT_H2O2_2_5	AT_H2O2_2	0.0	Pyrimidine nucleotides	False			-3.442	-1.402
282	Val-Leu	Amino acid	2	AT_H2O2_2_5	AT_H2O2_2	0.666666666666667	Dipeptides	False			-1.414	-1.045
258	Ile-Gly	Amino acid	2	AT_H2O2_2_5	AT_H2O2_2	0.666666666666667	Dipeptides	False			-2.663	-3.077
259	Ile-Ser	Amino acid	2	AT_H2O2_2_5	AT_H2O2_2	0.0	Dipeptides	False			-3.192	-2.329
269	Phe-Ser	Amino acid	2	AT_H2O2_2_5	AT_H2O2_2	0.333333333333333	Dipeptides	True	1.73e+04	-2.493	-2.493	-1.178
277	Tyr-Ala	Amino acid	2	AT_H2O2_2_5	AT_H2O2_2	0.833333333333333	Dipeptides	True	9.014e+04	-0.1113	-0.1113	-0.6814
257	Ile-Gln	Amino acid	2	AT_H2O2_2_5	AT_H2O2_2	0.166666666666667	Dipeptides	False			-2.625	-1.706
261	Leu-Glu	Amino acid	2	AT_H2O2_2_5	AT_H2O2_2	1.0	Dipeptides	True	3.94e+04	-1.305	-1.305	-2.096
263	Leu-Gly	Amino acid	2	AT_H2O2_2_5	AT_H2O2_2	1.0	Dipeptides	True	6.123e+04	-0.6692	-0.6692	-1.376
256	Ile-Ala	Amino acid	2	AT_H2O2_2_5	AT_H2O2_2	0.666666666666667	Dipeptides	True	4.696e+04	-1.052	-1.052	-1.01
274	Thr-Leu	Amino acid	2	AT_H2O2_2_5	AT_H2O2_2	0.833333333333333	Dipeptides	True	2.62e+05	1.428	1.428	0.5769
273	Ser-Phe	Amino acid	2	AT_H2O2_2_5	AT_H2O2_2	0.833333333333333	Dipeptides	True	1.783e+04	-2.449	-2.449	-1.227
272	Ser-Leu	Amino acid	2	AT_H2O2_2_5	AT_H2O2_2	1.0	Dipeptides	True	6.71e+04	-0.5371	-0.5371	-1.276
246	Asp-Leu	Amino acid	2	AT_H2O2_2_5	AT_H2O2_2	1.0	Dipeptides	True	6.575e+04	-0.5666	-0.5666	-0.4925
76	Gln	Amino acid	2	AT_H2O2_2_6	AT_H2O2_2	1.0	Proteinogenic amino acids	True	1.023e+07	6.088	6.088	-0.8637
89	Trp	Amino acid	2	AT_H2O2_2_6	AT_H2O2_2	1.0	Proteinogenic amino acids	True	8.98e+06	5.9	5.9	-0.06698
723	beta-Ala	Cofactor	2	AT_H2O2_2_6	AT_H2O2_2	1.0	Coenzyme A biosynthesis	True	1.436e+05	-0.06685	-0.06685	-0.3931
75	Glu	Amino acid	2	AT_H2O2_2_6	AT_H2O2_2	1.0	Proteinogenic amino acids	True	7.023e+06	5.545	5.545	-0.4028
80	His	Amino acid	2	AT_H2O2_2_6	AT_H2O2_2	1.0	Proteinogenic amino acids	True	1.437e+05	-0.06557	-0.06557	-0.2797

Metabolite ID	Name	Super Pathway	Dataset	Sample ID	Group ID	Detection Fraction	Pathway	Detected	Raw Intensity	Log2 Norm Intensity	Norm Imputed	Log2 Ctrl Norm
82	Leu	Amino acid	2	AT_H2O2_2_6	AT_H2O2_2	1.0	Proteinogenic amino acids	True	4.075e+07	8.082	8.082	-0.07866
87	Phe	Amino acid	2	AT_H2O2_2_6	AT_H2O2_2	1.0	Proteinogenic amino acids	True	3.855e+07	8.002	8.002	0.1098
236	Spermidine	Amino acid	2	AT_H2O2_2_6	AT_H2O2_2	1.0	Polyamines	True	1.109e+06	2.882	2.882	0.05462
73	Asn	Amino acid	2	AT_H2O2_2_6	AT_H2O2_2	1.0	Proteinogenic amino acids	True	4.172e+05	1.472	1.472	-1.277
243	Creatinine	Amino acid	2	AT_H2O2_2_6	AT_H2O2_2	1.0	Creatine degradation	True	5.879e+05	1.967	1.967	0.3963
376	Cytidine	Nucleotide	2	AT_H2O2_2_6	AT_H2O2_2	1.0	Pyrimidine nucleosides	True	3.123e+05	1.054	1.054	1.639
41	Lactate	Carbon	2	AT_H2O2_2_6	AT_H2O2_2	1.0	Respiratory carbon sources	True	5.725e+07	8.572	8.572	0.7502
93	3-P-Ser	Amino acid	2	AT_H2O2_2_6	AT_H2O2_2	0.5	Amino acids biosynthesis intermediates	True	1.883e+04	-2.998	-2.998	0.261
343	Adenine	Nucleotide	2	AT_H2O2_2_6	AT_H2O2_2	0.5	Purine bases	False			-3.45	-1.986
336	Adenosine	Nucleotide	2	AT_H2O2_2_6	AT_H2O2_2	1.0	Purine nucleosides	True	5.814e+05	1.95	1.95	-1.187
29	Raffinose	Carbon	2	AT_H2O2_2_6	AT_H2O2_2	0.166666666666667	Sugars and sugar alcohols	False			-5.058	-2.878
717	Nicotinamide	Cofactor	2	AT_H2O2_2_6	AT_H2O2_2	1.0	NAD biosynthesis	True	7.833e+05	2.381	2.381	-0.01611
51	PEP	Carbon	2	AT_H2O2_2_6	AT_H2O2_2	1.0	Glycolysis, GNG	True	1.518e+05	0.01289	0.01289	1.085
52	Pyruvate	Carbon	2	AT_H2O2_2_6	AT_H2O2_2	1.0	Glycolysis, GNG	True	2.673e+04	-2.492	-2.492	-0.1186
237	Spermine	Amino acid	2	AT_H2O2_2_6	AT_H2O2_2	0.666666666666667	Polyamines	True	1.089e+06	2.855	2.855	-0.5332
385	Uracil	Nucleotide	2	AT_H2O2_2_6	AT_H2O2_2	1.0	Pyrimidine bases	True	3.597e+05	1.258	1.258	2.985
377	Uridine	Nucleotide	2	AT_H2O2_2_6	AT_H2O2_2	1.0	Pyrimidine nucleosides	True	9.087e+05	2.595	2.595	-0.2876
112	trans-Urocanate	Amino acid	2	AT_H2O2_2_6	AT_H2O2_2	1.0	Amino acids degradation intermediates	True	1.081e+05	-0.4764	-0.4764	2.641
737	Pyridoxine (Vitamin B6)	Cofactor	2	AT_H2O2_2_6	AT_H2O2_2	1.0	PLP biosynthesis and salvage	True	2.467e+06	4.036	4.036	0.7357
348	Allantoin	Nucleotide	2	AT_H2O2_2_6	AT_H2O2_2	1.0	Purine degradation	True	4.115e+04	-1.87	-1.87	0.9595
335	Inosine	Nucleotide	2	AT_H2O2_2_6	AT_H2O2_2	1.0	Purine nucleosides	True	4.243e+05	1.496	1.496	-1.365
81	Ile	Amino acid	2	AT_H2O2_2_6	AT_H2O2_2	1.0	Proteinogenic amino acids	True	4.026e+07	8.064	8.064	0.2087
72	Ala	Amino acid	2	AT_H2O2_2_6	AT_H2O2_2	1.0	Proteinogenic amino acids	True	1.432e+07	6.573	6.573	-1.161

Metabolite ID	Name	Super Pathway	Dataset	Sample ID	Group ID	Detection Fraction	Pathway	Detected	Raw Intensity	Log2 Norm Intensity	Norm Imputed	Log2 Ctrl Norm
79	Thr	Amino acid	2	AT_H2O2_2_6	AT_H2O2_2	1.0	Proteinogenic amino acids	True	5.199e+06	5.111	5.111	-0.8513
88	Tyr	Amino acid	2	AT_H2O2_2_6	AT_H2O2_2	1.0	Proteinogenic amino acids	True	1.783e+07	6.89	6.89	0.04707
84	Lys	Amino acid	2	AT_H2O2_2_6	AT_H2O2_2	1.0	Proteinogenic amino acids	True	1.588e+06	3.401	3.401	-0.3187
86	Met	Amino acid	2	AT_H2O2_2_6	AT_H2O2_2	1.0	Proteinogenic amino acids	True	6.059e+06	5.332	5.332	-0.4551
61	Malate	Carbon	2	AT_H2O2_2_6	AT_H2O2_2	1.0	TCA cycle	True	3.966e+05	1.399	1.399	0.5019
235	Putrescine	Amino acid	2	AT_H2O2_2_6	AT_H2O2_2	0.0	Polyamines	False			-3.898	-2.331
49	3-P-Glycerate	Carbon	2	AT_H2O2_2_6	AT_H2O2_2	1.0	Glycolysis, GNG	True	1.192e+06	2.987	2.987	0.524
139	GABA	Amino acid	2	AT_H2O2_2_6	AT_H2O2_2	0.0	Amino acid derivatives	False			-4.963	-1.493
189	Kynurenate	Amino acid	2	AT_H2O2_2_6	AT_H2O2_2	0.833333333333333	Amino acid derivatives	True	8504	-4.145	-4.145	1.043
234	5-Me-Thioadenosine	Amino acid	2	AT_H2O2_2_6	AT_H2O2_2	1.0	SAM metabolism	True	1.019e+05	-0.5616	-0.5616	-0.4657
59	Succinate	Carbon	2	AT_H2O2_2_6	AT_H2O2_2	0.833333333333333	TCA cycle	True	3.76e+04	-2	-2	0.03976
133	Ornithine	Amino acid	2	AT_H2O2_2_6	AT_H2O2_2	1.0	Amino acids degradation intermediates	True	1.412e+06	3.231	3.231	0.7093
313	5-Oxoproline	Amino acid	2	AT_H2O2_2_6	AT_H2O2_2	1.0	Glutathione derivatives	True	1.665e+06	3.469	3.469	1.465
724	Pantothenate	Cofactor	2	AT_H2O2_2_6	AT_H2O2_2	1.0	Coenzyme A biosynthesis	True	2.163e+06	3.846	3.846	0.7329
30	Sucrose	Carbon	2	AT_H2O2_2_6	AT_H2O2_2	1.0	Sugars and sugar alcohols	True	3.1e+05	1.043	1.043	-1.333
122	3-OH-Isobutyrate	Amino acid	2	AT_H2O2_2_6	AT_H2O2_2	0.5	Amino acids degradation intermediates	True	3.287e+04	-2.194	-2.194	2.434
241	4-Acetamidobutanoate	Amino acid	2	AT_H2O2_2_6	AT_H2O2_2	1.0	Polyamine derivatives	True	1.33e+05	-0.1773	-0.1773	1.33
55	Citrate	Carbon	2	AT_H2O2_2_6	AT_H2O2_2	1.0	TCA cycle	True	2.506e+06	4.058	4.058	1.024
338	Guanosine	Nucleotide	2	AT_H2O2_2_6	AT_H2O2_2	1.0	Purine nucleosides	True	4.25e+05	1.498	1.498	-0.8621
170	2-Amino-Butyrate	Amino acid	2	AT_H2O2_2_6	AT_H2O2_2	1.0	Amino acid derivatives	True	2.917e+05	0.9557	0.9557	-0.2681
209	N-Ac-Ala	Amino acid	2	AT_H2O2_2_6	AT_H2O2_2	0.166666666666667	N-acetylated amino acids	False			-4.401	-1.133
221	N-Ac-Met	Amino acid	2	AT_H2O2_2_6	AT_H2O2_2	1.0	N-acetylated amino acids	True	1.854e+04	-3.021	-3.021	-1.87

Metabolite ID	Name	Super Pathway	Dataset	Sample ID	Group ID	Detection Fraction	Pathway	Detected	Raw Intensity	Log2 Norm Intensity	Norm Imputed	Log2 Ctrl Norm
22	N-Ac-Neuraminate	Carbon	2	AT_H2O2_2_6	AT_H2O2_2	0.833333333333333	Aminosugar derivatives	True	1.069e+05	-0.4924	-0.4924	-0.2784
346	Urate	Nucleotide	2	AT_H2O2_2_6	AT_H2O2_2	0.833333333333333	Purine degradation	True	2.19e+04	-2.78	-2.78	0.912
90	Arg	Amino acid	2	AT_H2O2_2_6	AT_H2O2_2	1.0	Proteinogenic amino acids	True	2.458e+06	4.03	4.03	-0.7517
60	Fumarate	Carbon	2	AT_H2O2_2_6	AT_H2O2_2	1.0	TCA cycle	True	2.758e+05	0.8748	0.8748	1.032
78	Ser	Amino acid	2	AT_H2O2_2_6	AT_H2O2_2	1.0	Proteinogenic amino acids	True	6.797e+06	5.498	5.498	-1.329
83	Val	Amino acid	2	AT_H2O2_2_6	AT_H2O2_2	1.0	Proteinogenic amino acids	True	2.549e+07	7.405	7.405	0.1712
734	Pyridoxal	Cofactor	2	AT_H2O2_2_6	AT_H2O2_2	0.666666666666667	PLP biosynthesis and salvage	False			-1.437	-0.4421
136	Urea	Amino acid	2	AT_H2O2_2_6	AT_H2O2_2	1.0	Amino acids degradation intermediates	True	1.089e+06	2.856	2.856	1.211
742	Folate	Cofactor	2	AT_H2O2_2_6	AT_H2O2_2	1.0	Folate metabolism	True	1.444e+05	-0.05899	-0.05899	0.458
729	Riboflavin (Vitamin B2)	Cofactor	2	AT_H2O2_2_6	AT_H2O2_2	0.833333333333333	Flavine biosynthesis	True	6.821e+04	-1.141	-1.141	0.4282
91	Pro	Amino acid	2	AT_H2O2_2_6	AT_H2O2_2	1.0	Proteinogenic amino acids	True	7.516e+06	5.643	5.643	-1.078
308	Glutathione, Reduced	Amino acid	2	AT_H2O2_2_6	AT_H2O2_2	1.0	Glutathione	True	6.934e+06	5.527	5.527	-0.9285
706	FAD	Cofactor	2	AT_H2O2_2_6	AT_H2O2_2	0.5	Cofactors	False			-5.096	0
299	gamma-Glu-Tyr	Amino acid	2	AT_H2O2_2_6	AT_H2O2_2	0.666666666666667	Gamma-glutamyl dipeptides	True	3.332e+04	-2.174	-2.174	-1.007
705	Coenzyme A	Cofactor	2	AT_H2O2_2_6	AT_H2O2_2	1.0	Cofactors	True	9093	-4.048	-4.048	-0.3417
342	Hypoxanthine	Nucleotide	2	AT_H2O2_2_6	AT_H2O2_2	1.0	Purine bases	True	1.449e+05	-0.05393	-0.05393	-0.0558
344	Xanthine	Nucleotide	2	AT_H2O2_2_6	AT_H2O2_2	0.833333333333333	Purine bases	True	2.19e+05	0.5421	0.5421	2.781
703	NAD+	Cofactor	2	AT_H2O2_2_6	AT_H2O2_2	1.0	Cofactors	True	6.075e+05	2.014	2.014	-1.545
731	Thiamin (Vitamin B1)	Cofactor	2	AT_H2O2_2_6	AT_H2O2_2	1.0	TPP biosynthesis	True	1.491e+05	-0.01289	-0.01289	0.1932
102	2-Aminoadipate	Amino acid	2	AT_H2O2_2_6	AT_H2O2_2	1.0	Amino acids biosynthesis intermediates	True	5.677e+04	-1.406	-1.406	-2.162
77	Gly	Amino acid	2	AT_H2O2_2_6	AT_H2O2_2	1.0	Proteinogenic amino acids	True	9.917e+06	6.043	6.043	-1.185
45	Fructose-6-P	Carbon	2	AT_H2O2_2_6	AT_H2O2_2	0.833333333333333	Glycolysis, GNG	True	6.883e+04	-1.128	-1.128	0.3075
36	Ribose	Carbon	2	AT_H2O2_2_6	AT_H2O2_2	0.666666666666667	Sugars and sugar alcohols	True	8.69e+04	-0.7915	-0.7915	0.206

Metabolite ID	Name	Super Pathway	Dataset	Sample ID	Group ID	Detection Fraction	Pathway	Detected	Raw Intensity	Log2 Norm Intensity	Norm Imputed	Log2 Ctrl Norm
4	GlcNAc 6-P	Carbon	2	AT_H2O2_2_6	AT_H2O2_2	1.0	Aminosugar biosynthesis	True	1.839e+05	0.2897	0.2897	-0.07003
188	Kynurenine	Amino acid	2	AT_H2O2_2_6	AT_H2O2_2	0.833333333333333	Amino acid derivatives	True	1.436e+05	-0.06724	-0.06724	-0.05327
63	6-P-Gluconate	Carbon	2	AT_H2O2_2_6	AT_H2O2_2	1.0	Pentose phosphate pathway (PPP)	True	1.834e+05	0.286	0.286	1.331
710	Carnitine	Cofactor	2	AT_H2O2_2_6	AT_H2O2_2	1.0	Cofactors	True	3.545e+05	1.237	1.237	-1.295
725	P-Pantetheine	Cofactor	2	AT_H2O2_2_6	AT_H2O2_2	0.833333333333333	Coenzyme A biosynthesis	True	1.52e+04	-3.307	-3.307	1.505
110	N-alpha-Ac-Ornithine	Amino acid	2	AT_H2O2_2_6	AT_H2O2_2	1.0	Amino acids biosynthesis intermediates	True	2.876e+05	0.9352	0.9352	-0.03887
116	3-Me-2-Oxo-Valerate	Amino acid	2	AT_H2O2_2_6	AT_H2O2_2	1.0	Amino acids degradation intermediates	True	3.787e+04	-1.99	-1.99	1.585
155	4-Guanidinobutanoate	Amino acid	2	AT_H2O2_2_6	AT_H2O2_2	0.666666666666667	Amino acid derivatives	True	3.78e+04	-1.993	-1.993	-1.273
310	S-Lactoyl-Glutathione	Amino acid	2	AT_H2O2_2_6	AT_H2O2_2	0.166666666666667	Glutathione derivatives	True	7236	-4.378	-4.378	-0.7057
34	Ribitol	Carbon	2	AT_H2O2_2_6	AT_H2O2_2	1.0	Sugars and sugar alcohols	True	9.852e+04	-0.6106	-0.6106	0.9682
707	FMN	Cofactor	2	AT_H2O2_2_6	AT_H2O2_2	0.666666666666667	Cofactors	True	2.465e+04	-2.61	-2.61	0.8113
17	Maltose	Carbon	2	AT_H2O2_2_6	AT_H2O2_2	1.0	Glycogen degradation	True	3.051e+05	1.02	1.02	1.06
18	Maltotriose	Carbon	2	AT_H2O2_2_6	AT_H2O2_2	1.0	Glycogen degradation	True	2.055e+06	3.772	3.772	2.343
19	Maltotetraose	Carbon	2	AT_H2O2_2_6	AT_H2O2_2	1.0	Glycogen degradation	True	2.523e+05	0.7461	0.7461	0.9366
232	SAH	Amino acid	2	AT_H2O2_2_6	AT_H2O2_2	1.0	SAM metabolism	True	1.765e+04	-3.091	-3.091	0.1349
74	Asp	Amino acid	2	AT_H2O2_2_6	AT_H2O2_2	1.0	Proteinogenic amino acids	True	1.917e+06	3.672	3.672	-1.988
129	5-Aminovalerate	Amino acid	2	AT_H2O2_2_6	AT_H2O2_2	0.666666666666667	Amino acids degradation intermediates	True	7.352e+04	-1.033	-1.033	-0.2197
254	Gly-Val	Amino acid	2	AT_H2O2_2_6	AT_H2O2_2	1.0	Dipeptides	True	5.984e+04	-1.33	-1.33	-1.256
291	gamma-Glu-Leu	Amino acid	2	AT_H2O2_2_6	AT_H2O2_2	0.833333333333333	Gamma-glutamyl dipeptides	True	1.099e+05	-0.4528	-0.4528	0.6291
173	Met Sulfoxide	Amino acid	2	AT_H2O2_2_6	AT_H2O2_2	1.0	Amino acid derivatives	True	1.995e+05	0.4076	0.4076	0.5464
43	Glucose	Carbon	2	AT_H2O2_2_6	AT_H2O2_2	1.0	Glycolysis, GNG	True	2.695e+07	7.485	7.485	0.6654

Metabolite ID	Name	Super Pathway	Dataset	Sample ID	Group ID	Detection Fraction	Pathway	Detected	Raw Intensity	Log2 Norm Intensity	Norm Imputed	Log2 Ctrl Norm
249	Gly-Gly	Amino acid	2	AT_H2O2_2_6	AT_H2O2_2	0.666666666666667	Dipeptides	False			-1.272	-1.489
169	2-OH-Butyrate	Amino acid	2	AT_H2O2_2_6	AT_H2O2_2	0.833333333333333	Amino acid derivatives	True	8.195e+04	-0.8762	-0.8762	0.5577
98	3-Methyl-2-Oxobutyrate	Amino acid	2	AT_H2O2_2_6	AT_H2O2_2	0.666666666666667	Amino acids biosynthesis intermediates	False			-3.759	0
100	4-Me-2-Oxo-Pentanoate	Amino acid	2	AT_H2O2_2_6	AT_H2O2_2	1.0	Amino acids biosynthesis intermediates	True	4.466e+04	-1.752	-1.752	1.55
253	Gly-Pro	Amino acid	2	AT_H2O2_2_6	AT_H2O2_2	0.833333333333333	Dipeptides	False			-1.487	-1.166
247	Asp-Phe	Amino acid	2	AT_H2O2_2_6	AT_H2O2_2	1.0	Dipeptides	True	4.082e+04	-1.882	-1.882	-0.9403
212	N-Ac-Asp	Amino acid	2	AT_H2O2_2_6	AT_H2O2_2	0.5	N-acetylated amino acids	True	1.137e+04	-3.725	-3.725	0.777
720	1-Me-Nicotinamide	Cofactor	2	AT_H2O2_2_6	AT_H2O2_2	1.0	Derivatives of NA, nicotinamide and NAD	True	1.475e+06	3.293	3.293	-0.4103
70	Creatine	Carbon	2	AT_H2O2_2_6	AT_H2O2_2	1.0	Creatine energy storage	True	6.855e+06	5.51	5.51	-0.2703
309	Glutathione, Oxidized	Amino acid	2	AT_H2O2_2_6	AT_H2O2_2	1.0	Glutathione	True	1.677e+06	3.479	3.479	-0.4694
44	Glucose 6-P	Carbon	2	AT_H2O2_2_6	AT_H2O2_2	1.0	Glycolysis, GNG	True	2.882e+05	0.9382	0.9382	0.1234
24	Fructose	Carbon	2	AT_H2O2_2_6	AT_H2O2_2	1.0	Sugars and sugar alcohols	True	4.474e+06	4.895	4.895	1.534
85	Cys	Amino acid	2	AT_H2O2_2_6	AT_H2O2_2	1.0	Proteinogenic amino acids	True	3.051e+05	1.02	1.02	1.612
704	NADH	Cofactor	2	AT_H2O2_2_6	AT_H2O2_2	0.166666666666667	Cofactors	True	7570	-4.313	-4.313	-2.383
275	Thr-Phe	Amino acid	2	AT_H2O2_2_6	AT_H2O2_2	0.666666666666667	Dipeptides	True	6.994e+04	-1.105	-1.105	-0.1711
738	Pyridoxate	Cofactor	2	AT_H2O2_2_6	AT_H2O2_2	1.0	PLP biosynthesis and salvage	True	2.799e+04	-2.426	-2.426	2.239
177	3-(4-OH-Phenyl)Lactate	Amino acid	2	AT_H2O2_2_6	AT_H2O2_2	1.0	Amino acid derivatives	True	1.852e+04	-3.022	-3.022	0.9313
206	Trans-4-OH-Pro	Amino acid	2	AT_H2O2_2_6	AT_H2O2_2	1.0	Amino acid derivatives	True	2.782e+05	0.8869	0.8869	1.083
329	AMP	Nucleotide	2	AT_H2O2_2_6	AT_H2O2_2	0.5	Purine nucleotides	True	6.108e+05	2.022	2.022	1.601
345	Guanine	Nucleotide	2	AT_H2O2_2_6	AT_H2O2_2	1.0	Purine bases	True	5.917e+05	1.976	1.976	0.3939
271	pyroGlu-Val	Amino acid	2	AT_H2O2_2_6	AT_H2O2_2	0.666666666666667	Dipeptides	True	8907	-4.078	-4.078	-0.4363

Metabolite ID	Name	Super Pathway	Dataset	Sample ID	Group ID	Detection Fraction	Pathway	Detected	Raw Intensity	Log2 Norm Intensity	Norm Imputed	Log2 Ctrl Norm
279	Val-Glu	Amino acid	2	AT_H2O2_2_6	AT_H2O2_2	0.666666666666667	Dipeptides	True	3.448e+04	-2.125	-2.125	-1.295
183	Phenol Sulfate	Amino acid	2	AT_H2O2_2_6	AT_H2O2_2	0.833333333333333	Amino acid derivatives	True	7.504e+04	-1.003	-1.003	2.724
740	3-Dehydrocarnitine	Cofactor	2	AT_H2O2_2_6	AT_H2O2_2	0.5	Carnitine biosynthesis	True	1.049e+05	-0.5195	-0.5195	-0.186
145	Pyro-Gln	Amino acid	2	AT_H2O2_2_6	AT_H2O2_2	1.0	Amino acid derivatives	True	1.606e+05	0.09492	0.09492	-0.251
197	C-Glycosyl-Trp	Amino acid	2	AT_H2O2_2_6	AT_H2O2_2	1.0	Amino acid derivatives	True	2.492e+05	0.7282	0.7282	0.8718
718	Nicotinamide Riboside	Cofactor	2	AT_H2O2_2_6	AT_H2O2_2	0.5	NAD biosynthesis	False			-2.306	-0.5023
295	gamma-Glu-Phe	Amino acid	2	AT_H2O2_2_6	AT_H2O2_2	0.666666666666667	Gamma-glutamyl dipeptides	False			-1.936	-1.834
399	Pseudouridine	Nucleotide	2	AT_H2O2_2_6	AT_H2O2_2	1.0	Pyrimidine derivatives in RNAs	True	2.192e+04	-2.779	-2.779	1.462
375	UTP	Nucleotide	2	AT_H2O2_2_6	AT_H2O2_2	0.0	Pyrimidine nucleotides	False			-5.966	-0.5235
20	Erythronate	Carbon	2	AT_H2O2_2_6	AT_H2O2_2	1.0	Aminosugar derivatives	True	5.891e+04	-1.352	-1.352	0.08923
151	Phenylacetylglycine	Amino acid	2	AT_H2O2_2_6	AT_H2O2_2	1.0	Amino acid derivatives	True	8.992e+04	-0.7422	-0.7422	3.679
252	Gly-Phe	Amino acid	2	AT_H2O2_2_6	AT_H2O2_2	0.666666666666667	Dipeptides	False			-0.4688	-0.573
251	Gly-Leu	Amino acid	2	AT_H2O2_2_6	AT_H2O2_2	1.0	Dipeptides	True	7.054e+04	-1.093	-1.093	-1.455
290	gamma-Glu-Ile	Amino acid	2	AT_H2O2_2_6	AT_H2O2_2	0.666666666666667	Gamma-glutamyl dipeptides	True	5.268e+04	-1.514	-1.514	0.01757
316	Ophthalmate	Amino acid	2	AT_H2O2_2_6	AT_H2O2_2	1.0	Oxidative stress markers	True	7.269e+04	-1.049	-1.049	-1.303
208	Pro-OH-Pro	Amino acid	2	AT_H2O2_2_6	AT_H2O2_2	1.0	Amino acid derivatives	True	2.602e+05	0.7907	0.7907	0.3311
352	3'-AMP	Nucleotide	2	AT_H2O2_2_6	AT_H2O2_2	1.0	Purine derivatives in signaling	True	6.22e+04	-1.274	-1.274	0.01098
314	Cys-Glutathione Disulfide	Amino acid	2	AT_H2O2_2_6	AT_H2O2_2	1.0	Oxidative stress markers	True	1.022e+05	-0.557	-0.557	0.8083
39	Threitol	Carbon	2	AT_H2O2_2_6	AT_H2O2_2	0.5	Sugars and sugar alcohols	True	1.508e+04	-3.318	-3.318	0.7066
31	Ribulose/Xylulose	Carbon	2	AT_H2O2_2_6	AT_H2O2_2	0.0	Sugars and sugar alcohols	False			-5.004	-0.09716
48	DHAP	Carbon	2	AT_H2O2_2_6	AT_H2O2_2	1.0	Glycolysis, GNG	True	2.232e+05	0.5692	0.5692	-1.189
182	P-Cresol Sulfate	Amino acid	2	AT_H2O2_2_6	AT_H2O2_2	1.0	Amino acid derivatives	True	1.428e+05	-0.07528	-0.07528	3.262

Metabolite ID	Name	Super Pathway	Dataset	Sample ID	Group ID	Detection Fraction	Pathway	Detected	Raw Intensity	Log2 Norm Intensity	Norm Imputed	Log2 Ctrl Norm
250	Gly-Ile	Amino acid	2	AT_H2O2_2_6	AT_H2O2_2	0.666666666666667	Dipeptides	True	4.814e+04	-1.644	-1.644	-0.2198
286	gamma-Glu-Glu	Amino acid	2	AT_H2O2_2_6	AT_H2O2_2	1.0	Gamma-glutamyl dipeptides	True	2.705e+04	-2.475	-2.475	-2.245
264	Leu-Leu	Amino acid	2	AT_H2O2_2_6	AT_H2O2_2	0.5	Dipeptides	True	5.784e+04	-1.379	-1.379	-0.3056
203	DiMe-Arg	Amino acid	2	AT_H2O2_2_6	AT_H2O2_2	1.0	Amino acid derivatives	True	2.787e+05	0.8897	0.8897	-0.1846
47	Fructose 1,6-PP, Glucose 1,6-PP	Carbon	2	AT_H2O2_2_6	AT_H2O2_2	1.0	Glycolysis, GNG	True	4.617e+04	-1.704	-1.704	-1.414
224	N-Ac-Ser	Amino acid	2	AT_H2O2_2_6	AT_H2O2_2	1.0	N-acetylated amino acids	True	6.971e+04	-1.109	-1.109	-1.98
244	Ala-Leu	Amino acid	2	AT_H2O2_2_6	AT_H2O2_2	0.166666666666667	Dipeptides	True	1.988e+05	0.4021	0.4021	0.06232
304	Cyclo(Phe-Pro)	Amino acid	2	AT_H2O2_2_6	AT_H2O2_2	0.0	Cyclic dipeptides	False			-1.192	-0.7201
302	Cyclo(Glu-Glu)	Amino acid	2	AT_H2O2_2_6	AT_H2O2_2	0.666666666666667	Cyclic dipeptides	True	6.48e+04	-1.215	-1.215	-0.04277
303	Cyclo(Leu-Pro)	Amino acid	2	AT_H2O2_2_6	AT_H2O2_2	0.0	Cyclic dipeptides	False			-0.4297	-0.7026
390	2',3'-cUMP	Nucleotide	2	AT_H2O2_2_6	AT_H2O2_2	1.0	Pyrimidine derivatives in signaling	True	7.011e+04	-1.101	-1.101	0.02759
68	Ribulose 5-P / Xylulose 5-P	Carbon	2	AT_H2O2_2_6	AT_H2O2_2	1.0	Pentose phosphate pathway (PPP)	True	1.708e+05	0.1831	0.1831	-0.586
388	2',3'-cCMP	Nucleotide	2	AT_H2O2_2_6	AT_H2O2_2	1.0	Pyrimidine derivatives in signaling	True	1.755e+05	0.2222	0.2222	-0.03123
33	Arabitol/Xylitol	Carbon	2	AT_H2O2_2_6	AT_H2O2_2	0.166666666666667	Sugars and sugar alcohols	True	2.066e+04	-2.864	-2.864	0.6412
268	Phe-Phe	Amino acid	2	AT_H2O2_2_6	AT_H2O2_2	0.0	Dipeptides	False			-2.762	-1.674
245	Ala-Phe	Amino acid	2	AT_H2O2_2_6	AT_H2O2_2	0.666666666666667	Dipeptides	True	4.766e+04	-1.658	-1.658	-0.2027
373	UMP	Nucleotide	2	AT_H2O2_2_6	AT_H2O2_2	0.0	Pyrimidine nucleotides	False			-3.442	-1.402
282	Val-Leu	Amino acid	2	AT_H2O2_2_6	AT_H2O2_2	0.666666666666667	Dipeptides	True	8.312e+04	-0.8556	-0.8556	-0.4869
258	Ile-Gly	Amino acid	2	AT_H2O2_2_6	AT_H2O2_2	0.666666666666667	Dipeptides	True	7.298e+04	-1.043	-1.043	-1.458
259	Ile-Ser	Amino acid	2	AT_H2O2_2_6	AT_H2O2_2	0.0	Dipeptides	False			-3.192	-2.329
269	Phe-Ser	Amino acid	2	AT_H2O2_2_6	AT_H2O2_2	0.333333333333333	Dipeptides	False			-3.197	-1.882
277	Tyr-Ala	Amino acid	2	AT_H2O2_2_6	AT_H2O2_2	0.833333333333333	Dipeptides	True	4.177e+04	-1.848	-1.848	-2.418

Metabolite ID	Name	Super Pathway	Dataset	Sample ID	Group ID	Detection Fraction	Pathway	Detected	Raw Intensity	Log2 Norm Intensity	Norm Imputed	Log2 Ctrl Norm
257	Ile-Gln	Amino acid	2	AT_H2O2_2_6	AT_H2O2_2	0.166666666666667	Dipeptides	False			-2.625	-1.706
261	Leu-Glu	Amino acid	2	AT_H2O2_2_6	AT_H2O2_2	1.0	Dipeptides	True	1.205e+05	-0.3195	-0.3195	-1.11
263	Leu-Gly	Amino acid	2	AT_H2O2_2_6	AT_H2O2_2	1.0	Dipeptides	True	1.163e+05	-0.3706	-0.3706	-1.078
256	Ile-Ala	Amino acid	2	AT_H2O2_2_6	AT_H2O2_2	0.666666666666667	Dipeptides	True	1.119e+05	-0.4266	-0.4266	-0.3848
274	Thr-Leu	Amino acid	2	AT_H2O2_2_6	AT_H2O2_2	0.833333333333333	Dipeptides	True	2.159e+05	0.5215	0.5215	-0.3297
273	Ser-Phe	Amino acid	2	AT_H2O2_2_6	AT_H2O2_2	0.833333333333333	Dipeptides	True	3.799e+04	-1.985	-1.985	-0.7634
272	Ser-Leu	Amino acid	2	AT_H2O2_2_6	AT_H2O2_2	1.0	Dipeptides	True	1.259e+05	-0.2572	-0.2572	-0.9961
246	Asp-Leu	Amino acid	2	AT_H2O2_2_6	AT_H2O2_2	1.0	Dipeptides	True	4.626e+04	-1.701	-1.701	-1.627
76	Gln	Amino acid	2	AT_Untreated_2_1	AT_Untreated_2	1.0	Proteinogenic amino acids	True	7.722e+06	5.638	5.638	-1.314
89	Trp	Amino acid	2	AT_Untreated_2_1	AT_Untreated_2	1.0	Proteinogenic amino acids	True	1.227e+07	6.306	6.306	0.3397
723	beta-Ala	Cofactor	2	AT_Untreated_2_1	AT_Untreated_2	1.0	Coenzyme A biosynthesis	True	3.69e+05	1.251	1.251	0.9246
75	Glu	Amino acid	2	AT_Untreated_2_1	AT_Untreated_2	1.0	Proteinogenic amino acids	True	7.326e+06	5.562	5.562	-0.3858
80	His	Amino acid	2	AT_Untreated_2_1	AT_Untreated_2	1.0	Proteinogenic amino acids	True	1.88e+05	0.2779	0.2779	0.06376
82	Leu	Amino acid	2	AT_Untreated_2_1	AT_Untreated_2	1.0	Proteinogenic amino acids	True	4.973e+07	8.325	8.325	0.1649
87	Phe	Amino acid	2	AT_Untreated_2_1	AT_Untreated_2	1.0	Proteinogenic amino acids	True	4.827e+07	8.282	8.282	0.3902
236	Spermidine	Amino acid	2	AT_Untreated_2_1	AT_Untreated_2	1.0	Polyamines	True	7.943e+05	2.357	2.357	-0.4709
73	Asn	Amino acid	2	AT_Untreated_2_1	AT_Untreated_2	1.0	Proteinogenic amino acids	True	4.871e+05	1.651	1.651	-1.098
243	Creatinine	Amino acid	2	AT_Untreated_2_1	AT_Untreated_2	1.0	Creatine degradation	True	7.099e+05	2.195	2.195	0.6243
376	Cytidine	Nucleotide	2	AT_Untreated_2_1	AT_Untreated_2	1.0	Pyrimidine nucleosides	True	3.361e+05	1.116	1.116	1.701
41	Lactate	Carbon	2	AT_Untreated_2_1	AT_Untreated_2	1.0	Respiratory carbon sources	True	6.581e+07	8.729	8.729	0.9073
93	3-P-Ser	Amino acid	2	AT_Untreated_2_1	AT_Untreated_2	0.333333333333333	Amino acids biosynthesis intermediates	True	4.247e+04	-1.868	-1.868	1.39
343	Adenine	Nucleotide	2	AT_Untreated_2_1	AT_Untreated_2	1.0	Purine bases	True	3.684e+04	-2.074	-2.074	-0.61
336	Adenosine	Nucleotide	2	AT_Untreated_2_1	AT_Untreated_2	1.0	Purine nucleosides	True	3.382e+05	1.125	1.125	-2.012

Metabolite ID	Name	Super Pathway	Dataset	Sample ID	Group ID	Detection Fraction	Pathway	Detected	Raw Intensity	Log2 Norm Intensity	Norm Imputed	Log2 Ctrl Norm
29	Raffinose	Carbon	2	AT_Untreated_2_1	AT_Untreated_2	0.166666666666667	Sugars and sugar alcohols	False			-5.058	-2.878
717	Nicotinamide	Cofactor	2	AT_Untreated_2_1	AT_Untreated_2	1.0	NAD biosynthesis	True	1.335e+06	3.106	3.106	0.7095
51	PEP	Carbon	2	AT_Untreated_2_1	AT_Untreated_2	1.0	Glycolysis, GNG	True	2.9e+05	0.903	0.903	1.975
52	Pyruvate	Carbon	2	AT_Untreated_2_1	AT_Untreated_2	1.0	Glycolysis, GNG	True	1.782e+04	-3.122	-3.122	-0.7479
237	Spermine	Amino acid	2	AT_Untreated_2_1	AT_Untreated_2	0.666666666666667	Polyamines	True	5.621e+05	1.858	1.858	-1.531
385	Uracil	Nucleotide	2	AT_Untreated_2_1	AT_Untreated_2	1.0	Pyrimidine bases	True	3.381e+05	1.125	1.125	2.852
377	Uridine	Nucleotide	2	AT_Untreated_2_1	AT_Untreated_2	1.0	Pyrimidine nucleosides	True	1.138e+06	2.876	2.876	-6.20e-03
112	trans-Urocanate	Amino acid	2	AT_Untreated_2_1	AT_Untreated_2	1.0	Amino acids degradation intermediates	True	1.762e+05	0.1842	0.1842	3.302
737	Pyridoxine (Vitamin B6)	Cofactor	2	AT_Untreated_2_1	AT_Untreated_2	1.0	PLP biosynthesis and salvage	True	2.397e+06	3.95	3.95	0.6502
348	Allantoin	Nucleotide	2	AT_Untreated_2_1	AT_Untreated_2	1.0	Purine degradation	True	6.914e+04	-1.165	-1.165	1.664
335	Inosine	Nucleotide	2	AT_Untreated_2_1	AT_Untreated_2	1.0	Purine nucleosides	True	6.191e+05	1.997	1.997	-0.864
81	Ile	Amino acid	2	AT_Untreated_2_1	AT_Untreated_2	1.0	Proteinogenic amino acids	True	4.753e+07	8.26	8.26	0.4045
72	Ala	Amino acid	2	AT_Untreated_2_1	AT_Untreated_2	1.0	Proteinogenic amino acids	True	2.408e+07	7.279	7.279	-0.4551
79	Thr	Amino acid	2	AT_Untreated_2_1	AT_Untreated_2	1.0	Proteinogenic amino acids	True	8.284e+06	5.739	5.739	-0.223
88	Tyr	Amino acid	2	AT_Untreated_2_1	AT_Untreated_2	1.0	Proteinogenic amino acids	True	2.164e+07	7.124	7.124	0.2819
84	Lys	Amino acid	2	AT_Untreated_2_1	AT_Untreated_2	1.0	Proteinogenic amino acids	True	1.996e+06	3.686	3.686	-0.03309
86	Met	Amino acid	2	AT_Untreated_2_1	AT_Untreated_2	1.0	Proteinogenic amino acids	True	7.768e+06	5.647	5.647	-0.1406
61	Malate	Carbon	2	AT_Untreated_2_1	AT_Untreated_2	1.0	TCA cycle	True	5.961e+05	1.943	1.943	1.046
235	Putrescine	Amino acid	2	AT_Untreated_2_1	AT_Untreated_2	0.666666666666667	Polyamines	True	2.551e+04	-2.604	-2.604	-1.037
49	3-P-Glycerate	Carbon	2	AT_Untreated_2_1	AT_Untreated_2	1.0	Glycolysis, GNG	True	2.018e+06	3.702	3.702	1.239
139	GABA	Amino acid	2	AT_Untreated_2_1	AT_Untreated_2	0.5	Amino acid derivatives	False			-4.963	-1.493
189	Kynurenate	Amino acid	2	AT_Untreated_2_1	AT_Untreated_2	1.0	Amino acid derivatives	True	1.627e+04	-3.253	-3.253	1.935
234	5-Me-Thioadenosine	Amino acid	2	AT_Untreated_2_1	AT_Untreated_2	1.0	SAM metabolism	True	8.887e+04	-0.8031	-0.8031	-0.7071

Metabolite ID	Name	Super Pathway	Dataset	Sample ID	Group ID	Detection Fraction	Pathway	Detected	Raw Intensity	Log2 Norm Intensity	Norm Imputed	Log2 Ctrl Norm
59	Succinate	Carbon	2	AT_Untreated_2_1	AT_Untreated_2	0.833333333333333	TCA cycle	True	4.202e+04	-1.884	-1.884	0.1561
133	Ornithine	Amino acid	2	AT_Untreated_2_1	AT_Untreated_2	1.0	Amino acids degradation intermediates	True	1.96e+06	3.66	3.66	1.139
313	5-Oxoproline	Amino acid	2	AT_Untreated_2_1	AT_Untreated_2	1.0	Glutathione derivatives	True	2.426e+06	3.968	3.968	1.964
724	Pantothenate	Cofactor	2	AT_Untreated_2_1	AT_Untreated_2	1.0	Coenzyme A biosynthesis	True	2.443e+06	3.978	3.978	0.8641
30	Sucrose	Carbon	2	AT_Untreated_2_1	AT_Untreated_2	1.0	Sugars and sugar alcohols	True	1.009e+06	2.702	2.702	0.3253
122	3-OH-Isobutyrate	Amino acid	2	AT_Untreated_2_1	AT_Untreated_2	0.666666666666667	Amino acids degradation intermediates	True	5.426e+04	-1.515	-1.515	3.113
241	4-Acetamidobutanoate	Amino acid	2	AT_Untreated_2_1	AT_Untreated_2	1.0	Polyamine derivatives	True	1.757e+05	0.1806	0.1806	1.688
55	Citrate	Carbon	2	AT_Untreated_2_1	AT_Untreated_2	1.0	TCA cycle	True	1.748e+06	3.495	3.495	0.4602
338	Guanosine	Nucleotide	2	AT_Untreated_2_1	AT_Untreated_2	1.0	Purine nucleosides	True	5.818e+05	1.908	1.908	-0.4528
170	2-Amino-Butyrate	Amino acid	2	AT_Untreated_2_1	AT_Untreated_2	1.0	Amino acid derivatives	True	4.776e+05	1.623	1.623	0.399
209	N-Ac-Ala	Amino acid	2	AT_Untreated_2_1	AT_Untreated_2	0.666666666666667	N-acetylated amino acids	True	7537	-4.363	-4.363	-1.095
221	N-Ac-Met	Amino acid	2	AT_Untreated_2_1	AT_Untreated_2	1.0	N-acetylated amino acids	True	4.289e+04	-1.854	-1.854	-0.7038
22	N-Ac-Neuraminate	Carbon	2	AT_Untreated_2_1	AT_Untreated_2	1.0	Aminosugar derivatives	True	1.317e+05	-0.2352	-0.2352	-0.02115
346	Urate	Nucleotide	2	AT_Untreated_2_1	AT_Untreated_2	1.0	Purine degradation	True	7.447e+04	-1.058	-1.058	2.634
90	Arg	Amino acid	2	AT_Untreated_2_1	AT_Untreated_2	1.0	Proteinogenic amino acids	True	3.063e+06	4.304	4.304	-0.4779
60	Fumarate	Carbon	2	AT_Untreated_2_1	AT_Untreated_2	1.0	TCA cycle	True	1.697e+05	0.1299	0.1299	0.2867
78	Ser	Amino acid	2	AT_Untreated_2_1	AT_Untreated_2	1.0	Proteinogenic amino acids	True	1.026e+07	6.048	6.048	-0.7784
83	Val	Amino acid	2	AT_Untreated_2_1	AT_Untreated_2	1.0	Proteinogenic amino acids	True	3.058e+07	7.624	7.624	0.3899
734	Pyridoxal	Cofactor	2	AT_Untreated_2_1	AT_Untreated_2	1.0	PLP biosynthesis and salvage	True	9.573e+04	-0.6959	-0.6959	0.2988
136	Urea	Amino acid	2	AT_Untreated_2_1	AT_Untreated_2	1.0	Amino acids degradation intermediates	True	1.288e+06	3.055	3.055	1.41
742	Folate	Cofactor	2	AT_Untreated_2_1	AT_Untreated_2	1.0	Folate metabolism	True	1.519e+05	-0.02933	-0.02933	0.4877
729	Riboflavin (Vitamin B2)	Cofactor	2	AT_Untreated_2_1	AT_Untreated_2	1.0	Flavine biosynthesis	True	6.995e+04	-1.149	-1.149	0.4205

Metabolite ID	Name	Super Pathway	Dataset	Sample ID	Group ID	Detection Fraction	Pathway	Detected	Raw Intensity	Log2 Norm Intensity	Norm Imputed	Log2 Ctrl Norm
91	Pro	Amino acid	2	AT_Untreated_2_1	AT_Untreated_2	1.0	Proteinogenic amino acids	True	1.095e+07	6.142	6.142	-0.5788
308	Glutathione, Reduced	Amino acid	2	AT_Untreated_2_1	AT_Untreated_2	1.0	Glutathione	True	8.77e+06	5.822	5.822	-0.6337
706	FAD	Cofactor	2	AT_Untreated_2_1	AT_Untreated_2	0.833333333333333	Cofactors	True	8438	-4.2	-4.2	0.8962
299	gamma-Glu-Tyr	Amino acid	2	AT_Untreated_2_1	AT_Untreated_2	1.0	Gamma-glutamyl dipeptides	True	3.724e+04	-2.058	-2.058	-0.8909
705	Coenzyme A	Cofactor	2	AT_Untreated_2_1	AT_Untreated_2	1.0	Cofactors	True	3675	-5.399	-5.399	-1.693
342	Hypoxanthine	Nucleotide	2	AT_Untreated_2_1	AT_Untreated_2	1.0	Purine bases	True	1.879e+05	0.277	0.277	0.2751
344	Xanthine	Nucleotide	2	AT_Untreated_2_1	AT_Untreated_2	0.833333333333333	Purine bases	True	1.448e+05	-0.09841	-0.09841	2.141
703	NAD+	Cofactor	2	AT_Untreated_2_1	AT_Untreated_2	1.0	Cofactors	True	1.024e+06	2.724	2.724	-0.8347
731	Thiamin (Vitamin B1)	Cofactor	2	AT_Untreated_2_1	AT_Untreated_2	1.0	TPP biosynthesis	True	1.56e+05	8.62e-03	8.62e-03	0.2147
102	2-Aminoadipate	Amino acid	2	AT_Untreated_2_1	AT_Untreated_2	1.0	Amino acids biosynthesis intermediates	True	1.544e+05	-6.57e-03	-6.57e-03	-0.7631
77	Gly	Amino acid	2	AT_Untreated_2_1	AT_Untreated_2	1.0	Proteinogenic amino acids	True	2.219e+07	7.161	7.161	-0.06754
45	Fructose-6-P	Carbon	2	AT_Untreated_2_1	AT_Untreated_2	0.666666666666667	Glycolysis, GNG	False			-2.517	-1.082
36	Ribose	Carbon	2	AT_Untreated_2_1	AT_Untreated_2	0.833333333333333	Sugars and sugar alcohols	True	5.842e+04	-1.408	-1.408	-0.4109
4	GlcNAc 6-P	Carbon	2	AT_Untreated_2_1	AT_Untreated_2	1.0	Aminosugar biosynthesis	True	2.649e+05	0.7723	0.7723	0.4126
188	Kynurenine	Amino acid	2	AT_Untreated_2_1	AT_Untreated_2	1.0	Amino acid derivatives	True	1.551e+05	0	0	0.01397
63	6-P-Gluconate	Carbon	2	AT_Untreated_2_1	AT_Untreated_2	1.0	Pentose phosphate pathway (PPP)	True	7.189e+04	-1.109	-1.109	-0.06377
710	Carnitine	Cofactor	2	AT_Untreated_2_1	AT_Untreated_2	1.0	Cofactors	True	6.429e+05	2.052	2.052	-0.48
725	P-Pantetheine	Cofactor	2	AT_Untreated_2_1	AT_Untreated_2	1.0	Coenzyme A biosynthesis	True	8476	-4.193	-4.193	0.619
110	N-alpha-Ac-Ornithine	Amino acid	2	AT_Untreated_2_1	AT_Untreated_2	1.0	Amino acids biosynthesis intermediates	True	3.014e+05	0.9587	0.9587	-0.01532
116	3-Me-2-Oxo-Valerate	Amino acid	2	AT_Untreated_2_1	AT_Untreated_2	1.0	Amino acids degradation intermediates	True	1.036e+05	-0.5818	-0.5818	2.993
155	4-Guanidinobutanoate	Amino acid	2	AT_Untreated_2_1	AT_Untreated_2	1.0	Amino acid derivatives	True	8.774e+04	-0.8216	-0.8216	-0.1021
310	S-Lactoyl-Glutathione	Amino acid	2	AT_Untreated_2_1	AT_Untreated_2	0.666666666666667	Glutathione derivatives	True	6928	-4.484	-4.484	-0.8123

Metabolite ID	Name	Super Pathway	Dataset	Sample ID	Group ID	Detection Fraction	Pathway	Detected	Raw Intensity	Log2 Norm Intensity	Norm Imputed	Log2 Ctrl Norm
34	Ribitol	Carbon	2	AT_Untreated_2_1	AT_Untreated_2	1.0	Sugars and sugar alcohols	True	1.282e+05	-0.2749	-0.2749	1.304
707	FMN	Cofactor	2	AT_Untreated_2_1	AT_Untreated_2	0.666666666666667	Cofactors	True	1.181e+04	-3.715	-3.715	-0.294
17	Maltose	Carbon	2	AT_Untreated_2_1	AT_Untreated_2	1.0	Glycogen degradation	True	4.762e+05	1.619	1.619	1.658
18	Maltotriose	Carbon	2	AT_Untreated_2_1	AT_Untreated_2	1.0	Glycogen degradation	True	3.826e+06	4.625	4.625	3.196
19	Maltotetraose	Carbon	2	AT_Untreated_2_1	AT_Untreated_2	1.0	Glycogen degradation	True	3.608e+05	1.218	1.218	1.409
232	SAH	Amino acid	2	AT_Untreated_2_1	AT_Untreated_2	1.0	SAM metabolism	True	1.502e+04	-3.367	-3.367	-0.1413
74	Asp	Amino acid	2	AT_Untreated_2_1	AT_Untreated_2	1.0	Proteinogenic amino acids	True	4.173e+06	4.75	4.75	-0.9093
129	5-Aminovalerate	Amino acid	2	AT_Untreated_2_1	AT_Untreated_2	0.833333333333333	Amino acids degradation intermediates	True	1.399e+05	-0.1486	-0.1486	0.6645
254	Gly-Val	Amino acid	2	AT_Untreated_2_1	AT_Untreated_2	1.0	Dipeptides	True	1.465e+05	-0.08163	-0.08163	-7.89e-03
291	gamma-Glu-Leu	Amino acid	2	AT_Untreated_2_1	AT_Untreated_2	1.0	Gamma-glutamyl dipeptides	True	1.669e+05	0.1059	0.1059	1.188
173	Met Sulfoxide	Amino acid	2	AT_Untreated_2_1	AT_Untreated_2	1.0	Amino acid derivatives	True	1.77e+05	0.1909	0.1909	0.3297
43	Glucose	Carbon	2	AT_Untreated_2_1	AT_Untreated_2	1.0	Glycolysis, GNG	True	3.93e+07	7.985	7.985	1.166
249	Gly-Gly	Amino acid	2	AT_Untreated_2_1	AT_Untreated_2	1.0	Dipeptides	True	8.363e+04	-0.8908	-0.8908	-1.108
169	2-OH-Butyrate	Amino acid	2	AT_Untreated_2_1	AT_Untreated_2	0.833333333333333	Amino acid derivatives	True	1.646e+05	0.08604	0.08604	1.52
98	3-Methyl-2-Oxobutyrate	Amino acid	2	AT_Untreated_2_1	AT_Untreated_2	0.833333333333333	Amino acids biosynthesis intermediates	True	3.361e+04	-2.206	-2.206	1.553
100	4-Me-2-Oxo-Pentanoate	Amino acid	2	AT_Untreated_2_1	AT_Untreated_2	1.0	Amino acids biosynthesis intermediates	True	1.043e+05	-0.5721	-0.5721	2.73
253	Gly-Pro	Amino acid	2	AT_Untreated_2_1	AT_Untreated_2	1.0	Dipeptides	True	1.134e+05	-0.4517	-0.4517	-0.1307
247	Asp-Phe	Amino acid	2	AT_Untreated_2_1	AT_Untreated_2	1.0	Dipeptides	True	5.263e+04	-1.559	-1.559	-0.6178
212	N-Ac-Asp	Amino acid	2	AT_Untreated_2_1	AT_Untreated_2	0.333333333333333	N-acetylated amino acids	True	1.913e+04	-3.019	-3.019	1.483
720	1-Me-Nicotinamide	Cofactor	2	AT_Untreated_2_1	AT_Untreated_2	1.0	Derivatives of NA, nicotinamide and NAD	True	1.793e+06	3.532	3.532	-0.1719
70	Creatine	Carbon	2	AT_Untreated_2_1	AT_Untreated_2	1.0	Creatine energy storage	True	7.814e+06	5.655	5.655	-0.1252
309	Glutathione, Oxidized	Amino acid	2	AT_Untreated_2_1	AT_Untreated_2	1.0	Glutathione	True	2.472e+06	3.995	3.995	0.04646

Metabolite ID	Name	Super Pathway	Dataset	Sample ID	Group ID	Detection Fraction	Pathway	Detected	Raw Intensity	Log2 Norm Intensity	Norm Imputed	Log2 Ctrl Norm
44	Glucose 6-P	Carbon	2	AT_Untreated_2_1	AT_Untreated_2	1.0	Glycolysis, GNG	True	2.491e+05	0.6836	0.6836	-0.1312
24	Fructose	Carbon	2	AT_Untreated_2_1	AT_Untreated_2	1.0	Sugars and sugar alcohols	True	8.887e+06	5.841	5.841	2.48
85	Cys	Amino acid	2	AT_Untreated_2_1	AT_Untreated_2	1.0	Proteinogenic amino acids	True	3.383e+05	1.126	1.126	1.717
704	NADH	Cofactor	2	AT_Untreated_2_1	AT_Untreated_2	0.666666666666667	Cofactors	True	1.096e+04	-3.823	-3.823	-1.893
275	Thr-Phe	Amino acid	2	AT_Untreated_2_1	AT_Untreated_2	0.833333333333333	Dipeptides	True	5.2e+04	-1.576	-1.576	-0.6427
738	Pyridoxate	Cofactor	2	AT_Untreated_2_1	AT_Untreated_2	1.0	PLP biosynthesis and salvage	True	2.58e+04	-2.587	-2.587	2.078
177	3-(4-OH-Phenyl)Lactate	Amino acid	2	AT_Untreated_2_1	AT_Untreated_2	1.0	Amino acid derivatives	True	3.257e+04	-2.251	-2.251	1.702
206	Trans-4-OH-Pro	Amino acid	2	AT_Untreated_2_1	AT_Untreated_2	1.0	Amino acid derivatives	True	2.469e+05	0.6708	0.6708	0.8663
329	AMP	Nucleotide	2	AT_Untreated_2_1	AT_Untreated_2	0.833333333333333	Purine nucleotides	True	1.129e+05	-0.4585	-0.4585	-0.879
345	Guanine	Nucleotide	2	AT_Untreated_2_1	AT_Untreated_2	1.0	Purine bases	True	6.577e+05	2.084	2.084	0.5024
271	pyroGlu-Val	Amino acid	2	AT_Untreated_2_1	AT_Untreated_2	1.0	Dipeptides	True	1.452e+04	-3.417	-3.417	0.2244
279	Val-Glu	Amino acid	2	AT_Untreated_2_1	AT_Untreated_2	1.0	Dipeptides	True	2.759e+04	-2.491	-2.491	-1.661
183	Phenol Sulfate	Amino acid	2	AT_Untreated_2_1	AT_Untreated_2	1.0	Amino acid derivatives	True	1.134e+05	-0.4516	-0.4516	3.276
740	3-Dehydrocarnitine	Cofactor	2	AT_Untreated_2_1	AT_Untreated_2	1.0	Carnitine biosynthesis	True	1.163e+05	-0.4149	-0.4149	-0.08134
145	Pyro-Gln	Amino acid	2	AT_Untreated_2_1	AT_Untreated_2	1.0	Amino acid derivatives	True	2.144e+05	0.4672	0.4672	0.1212
197	C-Glycosyl-Trp	Amino acid	2	AT_Untreated_2_1	AT_Untreated_2	1.0	Amino acid derivatives	True	3.178e+05	1.035	1.035	1.179
718	Nicotinamide Riboside	Cofactor	2	AT_Untreated_2_1	AT_Untreated_2	0.333333333333333	NAD biosynthesis	False			-2.306	-0.5023
295	gamma-Glu-Phe	Amino acid	2	AT_Untreated_2_1	AT_Untreated_2	1.0	Gamma-glutamyl dipeptides	True	8.291e+04	-0.9032	-0.9032	-0.8013
399	Pseudouridine	Nucleotide	2	AT_Untreated_2_1	AT_Untreated_2	1.0	Pyrimidine derivatives in RNAs	True	4.791e+04	-1.694	-1.694	2.546
375	UTP	Nucleotide	2	AT_Untreated_2_1	AT_Untreated_2	0.166666666666667	Pyrimidine nucleotides	False			-5.966	-0.5235
20	Erythronate	Carbon	2	AT_Untreated_2_1	AT_Untreated_2	1.0	Aminosugar derivatives	True	9.895e+04	-0.6481	-0.6481	0.7935
151	Phenylacetylglycine	Amino acid	2	AT_Untreated_2_1	AT_Untreated_2	1.0	Amino acid derivatives	True	3.03e+05	0.9664	0.9664	5.388

Metabolite ID	Name	Super Pathway	Dataset	Sample ID	Group ID	Detection Fraction	Pathway	Detected	Raw Intensity	Log2 Norm Intensity	Norm Imputed	Log2 Ctrl Norm
252	Gly-Phe	Amino acid	2	AT_Untreated_2_1	AT_Untreated_2	0.666666666666667	Dipeptides	False			-0.4688	-0.573
251	Gly-Leu	Amino acid	2	AT_Untreated_2_1	AT_Untreated_2	1.0	Dipeptides	True	2.512e+05	0.696	0.696	0.3334
290	gamma-Glu-Ile	Amino acid	2	AT_Untreated_2_1	AT_Untreated_2	0.833333333333333	Gamma-glutamyl dipeptides	True	6.482e+04	-1.258	-1.258	0.2729
316	Ophthalmate	Amino acid	2	AT_Untreated_2_1	AT_Untreated_2	1.0	Oxidative stress markers	True	8.524e+04	-0.8632	-0.8632	-1.117
208	Pro-OH-Pro	Amino acid	2	AT_Untreated_2_1	AT_Untreated_2	1.0	Amino acid derivatives	True	3.221e+05	1.055	1.055	0.5949
352	3'-AMP	Nucleotide	2	AT_Untreated_2_1	AT_Untreated_2	1.0	Purine derivatives in signaling	True	7.071e+04	-1.133	-1.133	0.152
314	Cys-Glutathione Disulfide	Amino acid	2	AT_Untreated_2_1	AT_Untreated_2	1.0	Oxidative stress markers	True	1.055e+05	-0.5563	-0.5563	0.8091
39	Threitol	Carbon	2	AT_Untreated_2_1	AT_Untreated_2	0.5	Sugars and sugar alcohols	False			-4.395	-0.3698
31	Ribulose/Xylulose	Carbon	2	AT_Untreated_2_1	AT_Untreated_2	0.0	Sugars and sugar alcohols	False			-5.004	-0.09716
48	DHAP	Carbon	2	AT_Untreated_2_1	AT_Untreated_2	1.0	Glycolysis, GNG	True	2.355e+05	0.6029	0.6029	-1.155
182	P-Cresol Sulfate	Amino acid	2	AT_Untreated_2_1	AT_Untreated_2	1.0	Amino acid derivatives	True	3.236e+04	-2.26	-2.26	1.077
250	Gly-Ile	Amino acid	2	AT_Untreated_2_1	AT_Untreated_2	1.0	Dipeptides	True	1.183e+05	-0.39	-0.39	1.034
286	gamma-Glu-Glu	Amino acid	2	AT_Untreated_2_1	AT_Untreated_2	0.833333333333333	Gamma-glutamyl dipeptides	True	8.052e+04	-0.9454	-0.9454	-0.7148
264	Leu-Leu	Amino acid	2	AT_Untreated_2_1	AT_Untreated_2	1.0	Dipeptides	True	6.173e+04	-1.329	-1.329	-0.2556
203	DiMe-Arg	Amino acid	2	AT_Untreated_2_1	AT_Untreated_2	1.0	Amino acid derivatives	True	4.947e+05	1.674	1.674	0.5993
47	Fructose 1,6-PP, Glucose 1,6-PP	Carbon	2	AT_Untreated_2_1	AT_Untreated_2	1.0	Glycolysis, GNG	True	3.236e+04	-2.261	-2.261	-1.971
224	N-Ac-Ser	Amino acid	2	AT_Untreated_2_1	AT_Untreated_2	0.833333333333333	N-acetylated amino acids	True	1.322e+05	-0.2304	-0.2304	-1.101
244	Ala-Leu	Amino acid	2	AT_Untreated_2_1	AT_Untreated_2	0.666666666666667	Dipeptides	True	1.254e+05	-0.3059	-0.3059	-0.6457
304	Cyclo(Phe-Pro)	Amino acid	2	AT_Untreated_2_1	AT_Untreated_2	0.0	Cyclic dipeptides	False			-1.192	-0.7201
302	Cyclo(Glu-Glu)	Amino acid	2	AT_Untreated_2_1	AT_Untreated_2	0.666666666666667	Cyclic dipeptides	True	8.289e+04	-0.9036	-0.9036	0.2686
303	Cyclo(Leu-Pro)	Amino acid	2	AT_Untreated_2_1	AT_Untreated_2	0.0	Cyclic dipeptides	False			-0.4297	-0.7026
390	2',3'-cUMP	Nucleotide	2	AT_Untreated_2_1	AT_Untreated_2	1.0	Pyrimidine derivatives in signaling	True	4.878e+04	-1.668	-1.668	-0.5395
68	Ribulose 5-P / Xylulose 5-P	Carbon	2	AT_Untreated_2_1	AT_Untreated_2	1.0	Pentose phosphate pathway (PPP)	True	2.111e+05	0.4452	0.4452	-0.324

Metabolite ID	Name	Super Pathway	Dataset	Sample ID	Group ID	Detection Fraction	Pathway	Detected	Raw Intensity	Log2 Norm Intensity	Norm Imputed	Log2 Ctrl Norm
388	2',3'-cCMP	Nucleotide	2	AT_Untreated_2_1	AT_Untreated_2	1.0	Pyrimidine derivatives in signaling	True	9.308e+04	-0.7363	-0.7363	-0.9898
33	Arabitol/Xylitol	Carbon	2	AT_Untreated_2_1	AT_Untreated_2	0.5	Sugars and sugar alcohols	True	2.398e+04	-2.693	-2.693	0.8123
268	Phe-Phe	Amino acid	2	AT_Untreated_2_1	AT_Untreated_2	0.5	Dipeptides	True	2.286e+04	-2.762	-2.762	-1.674
245	Ala-Phe	Amino acid	2	AT_Untreated_2_1	AT_Untreated_2	0.666666666666667	Dipeptides	True	3.974e+04	-1.964	-1.964	-0.5086
373	UMP	Nucleotide	2	AT_Untreated_2_1	AT_Untreated_2	0.5	Pyrimidine nucleotides	False			-3.442	-1.402
282	Val-Leu	Amino acid	2	AT_Untreated_2_1	AT_Untreated_2	0.666666666666667	Dipeptides	True	6.758e+04	-1.198	-1.198	-0.8294
258	Ile-Gly	Amino acid	2	AT_Untreated_2_1	AT_Untreated_2	0.833333333333333	Dipeptides	True	5.486e+04	-1.499	-1.499	-1.914
259	Ile-Ser	Amino acid	2	AT_Untreated_2_1	AT_Untreated_2	0.666666666666667	Dipeptides	True	1.697e+04	-3.192	-3.192	-2.329
269	Phe-Ser	Amino acid	2	AT_Untreated_2_1	AT_Untreated_2	0.666666666666667	Dipeptides	True	1.691e+04	-3.197	-3.197	-1.882
277	Tyr-Ala	Amino acid	2	AT_Untreated_2_1	AT_Untreated_2	1.0	Dipeptides	True	6.765e+04	-1.197	-1.197	-1.767
257	Ile-Gln	Amino acid	2	AT_Untreated_2_1	AT_Untreated_2	0.833333333333333	Dipeptides	True	3.318e+04	-2.224	-2.224	-1.306
261	Leu-Glu	Amino acid	2	AT_Untreated_2_1	AT_Untreated_2	1.0	Dipeptides	True	7.877e+04	-0.9771	-0.9771	-1.767
263	Leu-Gly	Amino acid	2	AT_Untreated_2_1	AT_Untreated_2	1.0	Dipeptides	True	4.922e+04	-1.656	-1.656	-2.362
256	Ile-Ala	Amino acid	2	AT_Untreated_2_1	AT_Untreated_2	0.833333333333333	Dipeptides	True	1.374e+05	-0.174	-0.174	-0.1323
274	Thr-Leu	Amino acid	2	AT_Untreated_2_1	AT_Untreated_2	0.833333333333333	Dipeptides	True	3.461e+05	1.158	1.158	0.307
273	Ser-Phe	Amino acid	2	AT_Untreated_2_1	AT_Untreated_2	0.666666666666667	Dipeptides	True	5.109e+04	-1.602	-1.602	-0.3798
272	Ser-Leu	Amino acid	2	AT_Untreated_2_1	AT_Untreated_2	1.0	Dipeptides	True	1.518e+05	-0.03108	-0.03108	-0.7699
246	Asp-Leu	Amino acid	2	AT_Untreated_2_1	AT_Untreated_2	1.0	Dipeptides	True	1.478e+05	-0.06961	-0.06961	4.44e-03
76	Gln	Amino acid	2	AT_Untreated_2_2	AT_Untreated_2	1.0	Proteinogenic amino acids	True	1.607e+07	6.415	6.415	-0.5366
89	Trp	Amino acid	2	AT_Untreated_2_2	AT_Untreated_2	1.0	Proteinogenic amino acids	True	1.047e+07	5.796	5.796	-0.1702
723	beta-Ala	Cofactor	2	AT_Untreated_2_2	AT_Untreated_2	1.0	Coenzyme A biosynthesis	True	3.702e+05	0.9751	0.9751	0.6488
75	Glu	Amino acid	2	AT_Untreated_2_2	AT_Untreated_2	1.0	Proteinogenic amino acids	True	1.246e+07	6.048	6.048	0.09994
80	His	Amino acid	2	AT_Untreated_2_2	AT_Untreated_2	1.0	Proteinogenic amino acids	True	1.786e+05	-0.07666	-0.07666	-0.2908

Metabolite ID	Name	Super Pathway	Dataset	Sample ID	Group ID	Detection Fraction	Pathway	Detected	Raw Intensity	Log2 Norm Intensity	Norm Imputed	Log2 Ctrl Norm
82	Leu	Amino acid	2	AT_Untreated_2_2	AT_Untreated_2	1.0	Proteinogenic amino acids	True	5.547e+07	8.202	8.202	0.04227
87	Phe	Amino acid	2	AT_Untreated_2_2	AT_Untreated_2	1.0	Proteinogenic amino acids	True	4.347e+07	7.851	7.851	-0.04102
236	Spermidine	Amino acid	2	AT_Untreated_2_2	AT_Untreated_2	1.0	Polyamines	True	1.108e+06	2.557	2.557	-0.2707
73	Asn	Amino acid	2	AT_Untreated_2_2	AT_Untreated_2	1.0	Proteinogenic amino acids	True	3.582e+05	0.9277	0.9277	-1.821
243	Creatinine	Amino acid	2	AT_Untreated_2_2	AT_Untreated_2	1.0	Creatine degradation	True	8.035e+05	2.093	2.093	0.5227
376	Cytidine	Nucleotide	2	AT_Untreated_2_2	AT_Untreated_2	1.0	Pyrimidine nucleosides	True	6.908e+05	1.875	1.875	2.46
41	Lactate	Carbon	2	AT_Untreated_2_2	AT_Untreated_2	1.0	Respiratory carbon sources	True	1.158e+08	9.264	9.264	1.442
93	3-P-Ser	Amino acid	2	AT_Untreated_2_2	AT_Untreated_2	0.333333333333333	Amino acids biosynthesis intermediates	False			-4.967	-1.709
343	Adenine	Nucleotide	2	AT_Untreated_2_2	AT_Untreated_2	1.0	Purine bases	True	5.856e+04	-1.685	-1.685	-0.2218
336	Adenosine	Nucleotide	2	AT_Untreated_2_2	AT_Untreated_2	1.0	Purine nucleosides	True	1.282e+06	2.767	2.767	-0.3707
29	Raffinose	Carbon	2	AT_Untreated_2_2	AT_Untreated_2	0.166666666666667	Sugars and sugar alcohols	False			-5.058	-2.878
717	Nicotinamide	Cofactor	2	AT_Untreated_2_2	AT_Untreated_2	1.0	NAD biosynthesis	True	1.4e+06	2.894	2.894	0.4974
51	PEP	Carbon	2	AT_Untreated_2_2	AT_Untreated_2	1.0	Glycolysis, GNG	True	8.184e+04	-1.202	-1.202	-0.1303
52	Pyruvate	Carbon	2	AT_Untreated_2_2	AT_Untreated_2	1.0	Glycolysis, GNG	True	9.79e+04	-0.9439	-0.9439	1.43
237	Spermine	Amino acid	2	AT_Untreated_2_2	AT_Untreated_2	0.666666666666667	Polyamines	True	1.201e+06	2.673	2.673	-0.7158
385	Uracil	Nucleotide	2	AT_Untreated_2_2	AT_Untreated_2	1.0	Pyrimidine bases	True	8.45e+04	-1.156	-1.156	0.5707
377	Uridine	Nucleotide	2	AT_Untreated_2_2	AT_Untreated_2	1.0	Pyrimidine nucleosides	True	9.728e+05	2.369	2.369	-0.5134
112	trans-Urocanate	Amino acid	2	AT_Untreated_2_2	AT_Untreated_2	1.0	Amino acids degradation intermediates	True	1.908e+05	0.01899	0.01899	3.137
737	Pyridoxine (Vitamin B6)	Cofactor	2	AT_Untreated_2_2	AT_Untreated_2	1.0	PLP biosynthesis and salvage	True	2.402e+06	3.673	3.673	0.3728
348	Allantoin	Nucleotide	2	AT_Untreated_2_2	AT_Untreated_2	1.0	Purine degradation	True	7.945e+04	-1.245	-1.245	1.584
335	Inosine	Nucleotide	2	AT_Untreated_2_2	AT_Untreated_2	1.0	Purine nucleosides	True	1.403e+06	2.897	2.897	0.03582
81	Ile	Amino acid	2	AT_Untreated_2_2	AT_Untreated_2	1.0	Proteinogenic amino acids	True	4.678e+07	7.957	7.957	0.1011
72	Ala	Amino acid	2	AT_Untreated_2_2	AT_Untreated_2	1.0	Proteinogenic amino acids	True	3.305e+07	7.455	7.455	-0.2788

Metabolite ID	Name	Super Pathway	Dataset	Sample ID	Group ID	Detection Fraction	Pathway	Detected	Raw Intensity	Log2 Norm Intensity	Norm Imputed	Log2 Ctrl Norm
79	Thr	Amino acid	2	AT_Untreated_2_2	AT_Untreated_2	1.0	Proteinogenic amino acids	True	9.668e+06	5.682	5.682	-0.2806
88	Tyr	Amino acid	2	AT_Untreated_2_2	AT_Untreated_2	1.0	Proteinogenic amino acids	True	2.49e+07	7.047	7.047	0.2041
84	Lys	Amino acid	2	AT_Untreated_2_2	AT_Untreated_2	1.0	Proteinogenic amino acids	True	2.56e+06	3.765	3.765	0.04579
86	Met	Amino acid	2	AT_Untreated_2_2	AT_Untreated_2	1.0	Proteinogenic amino acids	True	8.42e+06	5.483	5.483	-0.3046
61	Malate	Carbon	2	AT_Untreated_2_2	AT_Untreated_2	1.0	TCA cycle	True	5.12e+05	1.443	1.443	0.5462
235	Putrescine	Amino acid	2	AT_Untreated_2_2	AT_Untreated_2	0.666666666666667	Polyamines	False			-3.898	-2.331
49	3-P-Glycerate	Carbon	2	AT_Untreated_2_2	AT_Untreated_2	1.0	Glycolysis, GNG	True	9.791e+05	2.378	2.378	-0.08448
139	GABA	Amino acid	2	AT_Untreated_2_2	AT_Untreated_2	0.5	Amino acid derivatives	True	2.106e+04	-3.16	-3.16	0.3102
189	Kynurenate	Amino acid	2	AT_Untreated_2_2	AT_Untreated_2	1.0	Amino acid derivatives	True	6769	-4.798	-4.798	0.3894
234	5-Me-Thioadenosine	Amino acid	2	AT_Untreated_2_2	AT_Untreated_2	1.0	SAM metabolism	True	1.334e+05	-0.4978	-0.4978	-0.4018
59	Succinate	Carbon	2	AT_Untreated_2_2	AT_Untreated_2	0.833333333333333	TCA cycle	True	6.791e+04	-1.472	-1.472	0.5684
133	Ornithine	Amino acid	2	AT_Untreated_2_2	AT_Untreated_2	1.0	Amino acids degradation intermediates	True	2.055e+06	3.448	3.448	0.9264
313	5-Oxoproline	Amino acid	2	AT_Untreated_2_2	AT_Untreated_2	1.0	Glutathione derivatives	True	1.689e+06	3.165	3.165	1.162
724	Pantothenate	Cofactor	2	AT_Untreated_2_2	AT_Untreated_2	1.0	Coenzyme A biosynthesis	True	2.014e+06	3.419	3.419	0.3052
30	Sucrose	Carbon	2	AT_Untreated_2_2	AT_Untreated_2	1.0	Sugars and sugar alcohols	True	8.922e+05	2.244	2.244	-0.1325
122	3-OH-Isobutyrate	Amino acid	2	AT_Untreated_2_2	AT_Untreated_2	0.666666666666667	Amino acids degradation intermediates	True	2.83e+04	-2.735	-2.735	1.893
241	4-Acetamidobutanoate	Amino acid	2	AT_Untreated_2_2	AT_Untreated_2	1.0	Polyamine derivatives	True	1.594e+05	-0.2403	-0.2403	1.267
55	Citrate	Carbon	2	AT_Untreated_2_2	AT_Untreated_2	1.0	TCA cycle	True	1.765e+06	3.229	3.229	0.1943
338	Guanosine	Nucleotide	2	AT_Untreated_2_2	AT_Untreated_2	1.0	Purine nucleosides	True	1.426e+06	2.921	2.921	0.5602
170	2-Amino-Butyrate	Amino acid	2	AT_Untreated_2_2	AT_Untreated_2	1.0	Amino acid derivatives	True	2.872e+05	0.609	0.609	-0.6148
209	N-Ac-Ala	Amino acid	2	AT_Untreated_2_2	AT_Untreated_2	0.666666666666667	N-acetylated amino acids	True	8917	-4.401	-4.401	-1.133
221	N-Ac-Met	Amino acid	2	AT_Untreated_2_2	AT_Untreated_2	1.0	N-acetylated amino acids	True	7.395e+04	-1.349	-1.349	-0.1984

Metabolite ID	Name	Super Pathway	Dataset	Sample ID	Group ID	Detection Fraction	Pathway	Detected	Raw Intensity	Log2 Norm Intensity	Norm Imputed	Log2 Ctrl Norm
22	N-Ac-Neuraminate	Carbon	2	AT_Untreated_2_2	AT_Untreated_2	1.0	Aminosugar derivatives	True	1.578e+05	-0.2551	-0.2551	-0.0411
346	Urate	Nucleotide	2	AT_Untreated_2_2	AT_Untreated_2	1.0	Purine degradation	True	4.614e+04	-2.029	-2.029	1.663
90	Arg	Amino acid	2	AT_Untreated_2_2	AT_Untreated_2	1.0	Proteinogenic amino acids	True	5.09e+06	4.756	4.756	-0.02554
60	Fumarate	Carbon	2	AT_Untreated_2_2	AT_Untreated_2	1.0	TCA cycle	True	1.785e+05	-0.07731	-0.07731	0.07942
78	Ser	Amino acid	2	AT_Untreated_2_2	AT_Untreated_2	1.0	Proteinogenic amino acids	True	1.447e+07	6.264	6.264	-0.563
83	Val	Amino acid	2	AT_Untreated_2_2	AT_Untreated_2	1.0	Proteinogenic amino acids	True	3.139e+07	7.381	7.381	0.1472
734	Pyridoxal	Cofactor	2	AT_Untreated_2_2	AT_Untreated_2	1.0	PLP biosynthesis and salvage	True	1.623e+05	-0.2143	-0.2143	0.7803
136	Urea	Amino acid	2	AT_Untreated_2_2	AT_Untreated_2	1.0	Amino acids degradation intermediates	True	1.17e+06	2.635	2.635	0.9901
742	Folate	Cofactor	2	AT_Untreated_2_2	AT_Untreated_2	1.0	Folate metabolism	True	1.869e+05	-0.01127	-0.01127	0.5057
729	Riboflavin (Vitamin B2)	Cofactor	2	AT_Untreated_2_2	AT_Untreated_2	1.0	Flavine biosynthesis	True	8.341e+04	-1.175	-1.175	0.3941
91	Pro	Amino acid	2	AT_Untreated_2_2	AT_Untreated_2	1.0	Proteinogenic amino acids	True	2.232e+07	6.889	6.889	0.1677
308	Glutathione, Reduced	Amino acid	2	AT_Untreated_2_2	AT_Untreated_2	1.0	Glutathione	True	1.823e+07	6.597	6.597	0.1413
706	FAD	Cofactor	2	AT_Untreated_2_2	AT_Untreated_2	0.833333333333333	Cofactors	True	5506	-5.096	-5.096	0
299	gamma-Glu-Tyr	Amino acid	2	AT_Untreated_2_2	AT_Untreated_2	1.0	Gamma-glutamyl dipeptides	True	4.574e+04	-2.042	-2.042	-0.8745
705	Coenzyme A	Cofactor	2	AT_Untreated_2_2	AT_Untreated_2	1.0	Cofactors	True	1.161e+04	-4.02	-4.02	-0.3139
342	Hypoxanthine	Nucleotide	2	AT_Untreated_2_2	AT_Untreated_2	1.0	Purine bases	True	2.149e+05	0.1904	0.1904	0.1885
344	Xanthine	Nucleotide	2	AT_Untreated_2_2	AT_Untreated_2	0.833333333333333	Purine bases	True	5.45e+04	-1.789	-1.789	0.45
703	NAD+	Cofactor	2	AT_Untreated_2_2	AT_Untreated_2	1.0	Cofactors	True	1.746e+06	3.213	3.213	-0.3459
731	Thiamin (Vitamin B1)	Cofactor	2	AT_Untreated_2_2	AT_Untreated_2	1.0	TPP biosynthesis	True	1.787e+05	-0.07571	-0.07571	0.1304
102	2-Aminoadipate	Amino acid	2	AT_Untreated_2_2	AT_Untreated_2	1.0	Amino acids biosynthesis intermediates	True	2.994e+05	0.6688	0.6688	-0.08772
77	Gly	Amino acid	2	AT_Untreated_2_2	AT_Untreated_2	1.0	Proteinogenic amino acids	True	2.56e+07	7.087	7.087	-0.1412
45	Fructose-6-P	Carbon	2	AT_Untreated_2_2	AT_Untreated_2	0.666666666666667	Glycolysis, GNG	True	3.29e+04	-2.517	-2.517	-1.082
36	Ribose	Carbon	2	AT_Untreated_2_2	AT_Untreated_2	0.833333333333333	Sugars and sugar alcohols	True	8.719e+04	-1.111	-1.111	-0.1135

Metabolite ID	Name	Super Pathway	Dataset	Sample ID	Group ID	Detection Fraction	Pathway	Detected	Raw Intensity	Log2 Norm Intensity	Norm Imputed	Log2 Ctrl Norm
4	GlcNAc 6-P	Carbon	2	AT_Untreated_2_2	AT_Untreated_2	1.0	Aminosugar biosynthesis	True	1.047e+05	-0.8466	-0.8466	-1.206
188	Kynurenine	Amino acid	2	AT_Untreated_2_2	AT_Untreated_2	1.0	Amino acid derivatives	True	5.961e+05	1.662	1.662	1.676
63	6-P-Gluconate	Carbon	2	AT_Untreated_2_2	AT_Untreated_2	1.0	Pentose phosphate pathway (PPP)	True	6.283e+04	-1.584	-1.584	-0.5386
710	Carnitine	Cofactor	2	AT_Untreated_2_2	AT_Untreated_2	1.0	Cofactors	True	8.721e+05	2.211	2.211	-0.3206
725	P-Pantetheine	Cofactor	2	AT_Untreated_2_2	AT_Untreated_2	1.0	Coenzyme A biosynthesis	True	5540	-5.087	-5.087	-0.2747
110	N-alpha-Ac-Ornithine	Amino acid	2	AT_Untreated_2_2	AT_Untreated_2	1.0	Amino acids biosynthesis intermediates	True	1.813e+05	-0.05512	-0.05512	-1.029
116	3-Me-2-Oxo-Valerate	Amino acid	2	AT_Untreated_2_2	AT_Untreated_2	1.0	Amino acids degradation intermediates	True	8.005e+04	-1.234	-1.234	2.34
155	4-Guanidinobutanoate	Amino acid	2	AT_Untreated_2_2	AT_Untreated_2	1.0	Amino acid derivatives	True	3.938e+04	-2.258	-2.258	-1.538
310	S-Lactoyl-Glutathione	Amino acid	2	AT_Untreated_2_2	AT_Untreated_2	0.666666666666667	Glutathione derivatives	True	1.984e+04	-3.247	-3.247	0.4249
34	Ribitol	Carbon	2	AT_Untreated_2_2	AT_Untreated_2	1.0	Sugars and sugar alcohols	True	1.445e+05	-0.3822	-0.3822	1.197
707	FMN	Cofactor	2	AT_Untreated_2_2	AT_Untreated_2	0.666666666666667	Cofactors	True	1.758e+04	-3.421	-3.421	2.21e-05
17	Maltose	Carbon	2	AT_Untreated_2_2	AT_Untreated_2	1.0	Glycogen degradation	True	3.857e+05	1.034	1.034	1.074
18	Maltotriose	Carbon	2	AT_Untreated_2_2	AT_Untreated_2	1.0	Glycogen degradation	True	1.841e+06	3.289	3.289	1.86
19	Maltotetraose	Carbon	2	AT_Untreated_2_2	AT_Untreated_2	1.0	Glycogen degradation	True	3.878e+05	1.042	1.042	1.232
232	SAH	Amino acid	2	AT_Untreated_2_2	AT_Untreated_2	1.0	SAM metabolism	True	2.761e+04	-2.77	-2.77	0.4563
74	Asp	Amino acid	2	AT_Untreated_2_2	AT_Untreated_2	1.0	Proteinogenic amino acids	True	4.108e+06	4.447	4.447	-1.212
129	5-Aminovalerate	Amino acid	2	AT_Untreated_2_2	AT_Untreated_2	0.833333333333333	Amino acids degradation intermediates	True	8.922e+04	-1.078	-1.078	-0.2648
254	Gly-Val	Amino acid	2	AT_Untreated_2_2	AT_Untreated_2	1.0	Dipeptides	True	2.326e+05	0.3048	0.3048	0.3785
291	gamma-Glu-Leu	Amino acid	2	AT_Untreated_2_2	AT_Untreated_2	1.0	Gamma-glutamyl dipeptides	True	1.212e+05	-0.6355	-0.6355	0.4464
173	Met Sulfoxide	Amino acid	2	AT_Untreated_2_2	AT_Untreated_2	1.0	Amino acid derivatives	True	1.531e+05	-0.2991	-0.2991	-0.1603
43	Glucose	Carbon	2	AT_Untreated_2_2	AT_Untreated_2	1.0	Glycolysis, GNG	True	1.89e+07	6.649	6.649	-0.1706

Metabolite ID	Name	Super Pathway	Dataset	Sample ID	Group ID	Detection Fraction	Pathway	Detected	Raw Intensity	Log2 Norm Intensity	Norm Imputed	Log2 Ctrl Norm
249	Gly-Gly	Amino acid	2	AT_Untreated_2_2	AT_Untreated_2	1.0	Dipeptides	True	4.302e+05	1.192	1.192	0.9745
169	2-OH-Butyrate	Amino acid	2	AT_Untreated_2_2	AT_Untreated_2	0.833333333333333	Amino acid derivatives	True	9.078e+04	-1.053	-1.053	0.3811
98	3-Methyl-2-Oxobutyrate	Amino acid	2	AT_Untreated_2_2	AT_Untreated_2	0.833333333333333	Amino acids biosynthesis intermediates	True	3.004e+04	-2.648	-2.648	1.11
100	4-Me-2-Oxo-Pentanoate	Amino acid	2	AT_Untreated_2_2	AT_Untreated_2	1.0	Amino acids biosynthesis intermediates	True	6.938e+04	-1.441	-1.441	1.861
253	Gly-Pro	Amino acid	2	AT_Untreated_2_2	AT_Untreated_2	1.0	Dipeptides	True	2.541e+05	0.432	0.432	0.7531
247	Asp-Phe	Amino acid	2	AT_Untreated_2_2	AT_Untreated_2	1.0	Dipeptides	True	9.511e+04	-0.9856	-0.9856	-0.04432
212	N-Ac-Asp	Amino acid	2	AT_Untreated_2_2	AT_Untreated_2	0.333333333333333	N-acetylated amino acids	False			-4.689	-0.1861
720	1-Me-Nicotinamide	Cofactor	2	AT_Untreated_2_2	AT_Untreated_2	1.0	Derivatives of NA, nicotinamide and NAD	True	2.751e+06	3.869	3.869	0.1649
70	Creatine	Carbon	2	AT_Untreated_2_2	AT_Untreated_2	1.0	Creatine energy storage	True	1.11e+07	5.881	5.881	0.1006
309	Glutathione, Oxidized	Amino acid	2	AT_Untreated_2_2	AT_Untreated_2	1.0	Glutathione	True	2.393e+06	3.668	3.668	-0.2808
44	Glucose 6-P	Carbon	2	AT_Untreated_2_2	AT_Untreated_2	1.0	Glycolysis, GNG	True	1.103e+05	-0.7715	-0.7715	-1.586
24	Fructose	Carbon	2	AT_Untreated_2_2	AT_Untreated_2	1.0	Sugars and sugar alcohols	True	5.412e+06	4.845	4.845	1.484
85	Cys	Amino acid	2	AT_Untreated_2_2	AT_Untreated_2	1.0	Proteinogenic amino acids	True	1.857e+05	-0.02008	-0.02008	0.5718
704	NADH	Cofactor	2	AT_Untreated_2_2	AT_Untreated_2	0.666666666666667	Cofactors	True	5.411e+04	-1.799	-1.799	0.1302
275	Thr-Phe	Amino acid	2	AT_Untreated_2_2	AT_Untreated_2	0.833333333333333	Dipeptides	True	8.927e+04	-1.077	-1.077	-0.1433
738	Pyridoxate	Cofactor	2	AT_Untreated_2_2	AT_Untreated_2	1.0	PLP biosynthesis and salvage	True	1.755e+04	-3.423	-3.423	1.242
177	3-(4-OH-Phenyl)Lactate	Amino acid	2	AT_Untreated_2_2	AT_Untreated_2	1.0	Amino acid derivatives	True	2.354e+04	-3	-3	0.9532
206	Trans-4-OH-Pro	Amino acid	2	AT_Untreated_2_2	AT_Untreated_2	1.0	Amino acid derivatives	True	2.548e+05	0.4359	0.4359	0.6315
329	AMP	Nucleotide	2	AT_Untreated_2_2	AT_Untreated_2	0.833333333333333	Purine nucleotides	True	8.428e+05	2.162	2.162	1.741
345	Guanine	Nucleotide	2	AT_Untreated_2_2	AT_Untreated_2	1.0	Purine bases	True	8.242e+05	2.13	2.13	0.5478
271	pyroGlu-Val	Amino acid	2	AT_Untreated_2_2	AT_Untreated_2	1.0	Dipeptides	True	5081	-5.212	-5.212	-1.57
279	Val-Glu	Amino acid	2	AT_Untreated_2_2	AT_Untreated_2	1.0	Dipeptides	True	1.147e+05	-0.715	-0.715	0.115

Metabolite ID	Name	Super Pathway	Dataset	Sample ID	Group ID	Detection Fraction	Pathway	Detected	Raw Intensity	Log2 Norm Intensity	Norm Imputed	Log2 Ctrl Norm
183	Phenol Sulfate	Amino acid	2	AT_Untreated_2_2	AT_Untreated_2	1.0	Amino acid derivatives	True	1.323e+04	-3.832	-3.832	-0.1046
740	3-Dehydrocarnitine	Cofactor	2	AT_Untreated_2_2	AT_Untreated_2	1.0	Carnitine biosynthesis	True	1.807e+05	-0.0594	-0.0594	0.2742
145	Pyro-Gln	Amino acid	2	AT_Untreated_2_2	AT_Untreated_2	1.0	Amino acid derivatives	True	1.994e+05	0.08218	0.08218	-0.2638
197	C-Glycosyl-Trp	Amino acid	2	AT_Untreated_2_2	AT_Untreated_2	1.0	Amino acid derivatives	True	6.576e+05	1.804	1.804	1.948
718	Nicotinamide Riboside	Cofactor	2	AT_Untreated_2_2	AT_Untreated_2	0.333333333333333	NAD biosynthesis	False			-2.306	-0.5023
295	gamma-Glu-Phe	Amino acid	2	AT_Untreated_2_2	AT_Untreated_2	1.0	Gamma-glutamyl dipeptides	True	8.834e+04	-1.092	-1.092	-0.9901
399	Pseudouridine	Nucleotide	2	AT_Untreated_2_2	AT_Untreated_2	1.0	Pyrimidine derivatives in RNAs	True	2.252e+04	-3.064	-3.064	1.177
375	UTP	Nucleotide	2	AT_Untreated_2_2	AT_Untreated_2	0.166666666666667	Pyrimidine nucleotides	True	6175	-4.931	-4.931	0.5121
20	Erythronate	Carbon	2	AT_Untreated_2_2	AT_Untreated_2	1.0	Aminosugar derivatives	True	1.255e+05	-0.5854	-0.5854	0.8561
151	Phenylacetylglycine	Amino acid	2	AT_Untreated_2_2	AT_Untreated_2	1.0	Amino acid derivatives	True	2.711e+04	-2.796	-2.796	1.625
252	Gly-Phe	Amino acid	2	AT_Untreated_2_2	AT_Untreated_2	0.666666666666667	Dipeptides	True	2.354e+05	0.3219	0.3219	0.2177
251	Gly-Leu	Amino acid	2	AT_Untreated_2_2	AT_Untreated_2	1.0	Dipeptides	True	2.118e+05	0.1694	0.1694	-0.1932
290	gamma-Glu-Ile	Amino acid	2	AT_Untreated_2_2	AT_Untreated_2	0.833333333333333	Gamma-glutamyl dipeptides	True	5.535e+04	-1.766	-1.766	-0.2353
316	Ophthalmate	Amino acid	2	AT_Untreated_2_2	AT_Untreated_2	1.0	Oxidative stress markers	True	1.235e+05	-0.6085	-0.6085	-0.8627
208	Pro-OH-Pro	Amino acid	2	AT_Untreated_2_2	AT_Untreated_2	1.0	Amino acid derivatives	True	3.436e+05	0.8675	0.8675	0.4078
352	3'-AMP	Nucleotide	2	AT_Untreated_2_2	AT_Untreated_2	1.0	Purine derivatives in signaling	True	9.766e+04	-0.9475	-0.9475	0.3374
314	Cys-Glutathione Disulfide	Amino acid	2	AT_Untreated_2_2	AT_Untreated_2	1.0	Oxidative stress markers	True	1.243e+05	-0.5991	-0.5991	0.7662
39	Threitol	Carbon	2	AT_Untreated_2_2	AT_Untreated_2	0.5	Sugars and sugar alcohols	True	1.168e+04	-4.012	-4.012	0.01319
31	Ribulose/Xylulose	Carbon	2	AT_Untreated_2_2	AT_Untreated_2	0.0	Sugars and sugar alcohols	False			-5.004	-0.09716
48	DHAP	Carbon	2	AT_Untreated_2_2	AT_Untreated_2	1.0	Glycolysis, GNG	True	6.899e+05	1.873	1.873	0.1152
182	P-Cresol Sulfate	Amino acid	2	AT_Untreated_2_2	AT_Untreated_2	1.0	Amino acid derivatives	True	1.211e+04	-3.959	-3.959	-0.622
250	Gly-Ile	Amino acid	2	AT_Untreated_2_2	AT_Untreated_2	1.0	Dipeptides	True	6.46e+04	-1.544	-1.544	-0.1197

Metabolite ID	Name	Super Pathway	Dataset	Sample ID	Group ID	Detection Fraction	Pathway	Detected	Raw Intensity	Log2 Norm Intensity	Norm Imputed	Log2 Ctrl Norm
286	gamma-Glu-Glu	Amino acid	2	AT_Untreated_2_2	AT_Untreated_2	0.833333333333333	Gamma-glutamyl dipeptides	True	1.761e+05	-0.09704	-0.09704	0.1336
264	Leu-Leu	Amino acid	2	AT_Untreated_2_2	AT_Untreated_2	1.0	Dipeptides	True	1.072e+05	-0.8133	-0.8133	0.26
203	DiMe-Arg	Amino acid	2	AT_Untreated_2_2	AT_Untreated_2	1.0	Amino acid derivatives	True	5.452e+05	1.534	1.534	0.4593
47	Fructose 1,6-PP, Glucose 1,6-PP	Carbon	2	AT_Untreated_2_2	AT_Untreated_2	1.0	Glycolysis, GNG	True	1.622e+05	-0.2153	-0.2153	0.07483
224	N-Ac-Ser	Amino acid	2	AT_Untreated_2_2	AT_Untreated_2	0.833333333333333	N-acetylated amino acids	True	3.05e+05	0.6956	0.6956	-0.1751
244	Ala-Leu	Amino acid	2	AT_Untreated_2_2	AT_Untreated_2	0.666666666666667	Dipeptides	True	3.584e+05	0.9283	0.9283	0.5885
304	Cyclo(Phe-Pro)	Amino acid	2	AT_Untreated_2_2	AT_Untreated_2	0.0	Cyclic dipeptides	False			-1.192	-0.7201
302	Cyclo(Glu-Glu)	Amino acid	2	AT_Untreated_2_2	AT_Untreated_2	0.666666666666667	Cyclic dipeptides	False			-2.527	-1.355
303	Cyclo(Leu-Pro)	Amino acid	2	AT_Untreated_2_2	AT_Untreated_2	0.0	Cyclic dipeptides	False			-0.4297	-0.7026
390	2',3'-cUMP	Nucleotide	2	AT_Untreated_2_2	AT_Untreated_2	1.0	Pyrimidine derivatives in signaling	True	7.38e+04	-1.352	-1.352	-0.2227
68	Ribulose 5-P / Xylulose 5-P	Carbon	2	AT_Untreated_2_2	AT_Untreated_2	1.0	Pentose phosphate pathway (PPP)	True	2.023e+05	0.103	0.103	-0.6662
388	2',3'-cCMP	Nucleotide	2	AT_Untreated_2_2	AT_Untreated_2	1.0	Pyrimidine derivatives in signaling	True	1.536e+05	-0.2944	-0.2944	-0.5478
33	Arabitol/Xylitol	Carbon	2	AT_Untreated_2_2	AT_Untreated_2	0.5	Sugars and sugar alcohols	True	1.598e+04	-3.559	-3.559	-0.05408
268	Phe-Phe	Amino acid	2	AT_Untreated_2_2	AT_Untreated_2	0.5	Dipeptides	False			-2.762	-1.674
245	Ala-Phe	Amino acid	2	AT_Untreated_2_2	AT_Untreated_2	0.666666666666667	Dipeptides	True	6.359e+04	-1.566	-1.566	-0.1109
373	UMP	Nucleotide	2	AT_Untreated_2_2	AT_Untreated_2	0.5	Pyrimidine nucleotides	True	1.889e+05	4.23e-03	4.23e-03	2.045
282	Val-Leu	Amino acid	2	AT_Untreated_2_2	AT_Untreated_2	0.666666666666667	Dipeptides	True	1.127e+05	-0.7405	-0.7405	-0.3717
258	Ile-Gly	Amino acid	2	AT_Untreated_2_2	AT_Untreated_2	0.833333333333333	Dipeptides	True	1.883e+05	0	0	-0.4146
259	Ile-Ser	Amino acid	2	AT_Untreated_2_2	AT_Untreated_2	0.666666666666667	Dipeptides	True	1.165e+05	-0.6926	-0.6926	0.1704
269	Phe-Ser	Amino acid	2	AT_Untreated_2_2	AT_Untreated_2	0.666666666666667	Dipeptides	True	8.768e+04	-1.103	-1.103	0.212
277	Tyr-Ala	Amino acid	2	AT_Untreated_2_2	AT_Untreated_2	1.0	Dipeptides	True	5.867e+05	1.639	1.639	1.069
257	Ile-Gln	Amino acid	2	AT_Untreated_2_2	AT_Untreated_2	0.833333333333333	Dipeptides	True	7.906e+04	-1.252	-1.252	-0.3334
261	Leu-Glu	Amino acid	2	AT_Untreated_2_2	AT_Untreated_2	1.0	Dipeptides	True	4.391e+05	1.221	1.221	0.4312

Metabolite ID	Name	Super Pathway	Dataset	Sample ID	Group ID	Detection Fraction	Pathway	Detected	Raw Intensity	Log2 Norm Intensity	Norm Imputed	Log2 Ctrl Norm
263	Leu-Gly	Amino acid	2	AT_Untreated_2_2	AT_Untreated_2	1.0	Dipeptides	True	1.08e+06	2.519	2.519	1.813
256	Ile-Ala	Amino acid	2	AT_Untreated_2_2	AT_Untreated_2	0.833333333333333	Dipeptides	True	1.508e+05	-0.3202	-0.3202	-0.2785
274	Thr-Leu	Amino acid	2	AT_Untreated_2_2	AT_Untreated_2	0.833333333333333	Dipeptides	True	2.363e+05	0.3274	0.3274	-0.5237
273	Ser-Phe	Amino acid	2	AT_Untreated_2_2	AT_Untreated_2	0.666666666666667	Dipeptides	True	6.176e+04	-1.609	-1.609	-0.3867
272	Ser-Leu	Amino acid	2	AT_Untreated_2_2	AT_Untreated_2	1.0	Dipeptides	True	3.509e+05	0.898	0.898	0.1592
246	Asp-Leu	Amino acid	2	AT_Untreated_2_2	AT_Untreated_2	1.0	Dipeptides	True	2.255e+05	0.2599	0.2599	0.3339
76	Gln	Amino acid	2	AT_Untreated_2_3	AT_Untreated_2	1.0	Proteinogenic amino acids	True	8.298e+06	6.492	6.492	-0.4602
89	Trp	Amino acid	2	AT_Untreated_2_3	AT_Untreated_2	1.0	Proteinogenic amino acids	True	4.763e+06	5.691	5.691	-0.2756
723	beta-Ala	Cofactor	2	AT_Untreated_2_3	AT_Untreated_2	1.0	Coenzyme A biosynthesis	True	7.126e+04	-0.3718	-0.3718	-0.6981
75	Glu	Amino acid	2	AT_Untreated_2_3	AT_Untreated_2	1.0	Proteinogenic amino acids	True	7.216e+06	6.29	6.29	0.3423
80	His	Amino acid	2	AT_Untreated_2_3	AT_Untreated_2	1.0	Proteinogenic amino acids	True	9.072e+04	-0.02335	-0.02335	-0.2375
82	Leu	Amino acid	2	AT_Untreated_2_3	AT_Untreated_2	1.0	Proteinogenic amino acids	True	2.349e+07	7.993	7.993	-0.1669
87	Phe	Amino acid	2	AT_Untreated_2_3	AT_Untreated_2	1.0	Proteinogenic amino acids	True	2.2e+07	7.899	7.899	6.88e-03
236	Spermidine	Amino acid	2	AT_Untreated_2_3	AT_Untreated_2	1.0	Polyamines	True	1.389e+05	0.5911	0.5911	-2.237
73	Asn	Amino acid	2	AT_Untreated_2_3	AT_Untreated_2	1.0	Proteinogenic amino acids	True	2.117e+05	1.199	1.199	-1.55
243	Creatinine	Amino acid	2	AT_Untreated_2_3	AT_Untreated_2	1.0	Creatine degradation	True	5.18e+05	2.49	2.49	0.9197
376	Cytidine	Nucleotide	2	AT_Untreated_2_3	AT_Untreated_2	1.0	Pyrimidine nucleosides	True	3.25e+05	1.818	1.818	2.402
41	Lactate	Carbon	2	AT_Untreated_2_3	AT_Untreated_2	1.0	Respiratory carbon sources	True	3.349e+07	8.505	8.505	0.6826
93	3-P-Ser	Amino acid	2	AT_Untreated_2_3	AT_Untreated_2	0.333333333333333	Amino acids biosynthesis intermediates	True	1.382e+04	-2.738	-2.738	0.5209
343	Adenine	Nucleotide	2	AT_Untreated_2_3	AT_Untreated_2	1.0	Purine bases	True	1.87e+04	-2.302	-2.302	-0.8383
336	Adenosine	Nucleotide	2	AT_Untreated_2_3	AT_Untreated_2	1.0	Purine nucleosides	True	2.301e+05	1.319	1.319	-1.818
29	Raffinose	Carbon	2	AT_Untreated_2_3	AT_Untreated_2	0.166666666666667	Sugars and sugar alcohols	False			-5.058	-2.878
717	Nicotinamide	Cofactor	2	AT_Untreated_2_3	AT_Untreated_2	1.0	NAD biosynthesis	True	5.764e+05	2.644	2.644	0.2475

Metabolite ID	Name	Super Pathway	Dataset	Sample ID	Group ID	Detection Fraction	Pathway	Detected	Raw Intensity	Log2 Norm Intensity	Norm Imputed	Log2 Ctrl Norm
51	PEP	Carbon	2	AT_Untreated_2_3	AT_Untreated_2	1.0	Glycolysis, GNG	True	7.596e+04	-0.2796	-0.2796	0.7923
52	Pyruvate	Carbon	2	AT_Untreated_2_3	AT_Untreated_2	1.0	Glycolysis, GNG	True	2.251e+04	-2.034	-2.034	0.3394
237	Spermine	Amino acid	2	AT_Untreated_2_3	AT_Untreated_2	0.666666666666667	Polyamines	False			0.9044	-2.484
385	Uracil	Nucleotide	2	AT_Untreated_2_3	AT_Untreated_2	1.0	Pyrimidine bases	True	7.763e+04	-0.2482	-0.2482	1.479
377	Uridine	Nucleotide	2	AT_Untreated_2_3	AT_Untreated_2	1.0	Pyrimidine nucleosides	True	6.065e+05	2.718	2.718	-0.1648
112	trans-Urocanate	Amino acid	2	AT_Untreated_2_3	AT_Untreated_2	1.0	Amino acids degradation intermediates	True	1.151e+05	0.3203	0.3203	3.438
737	Pyridoxine (Vitamin B6)	Cofactor	2	AT_Untreated_2_3	AT_Untreated_2	1.0	PLP biosynthesis and salvage	True	1.863e+06	4.337	4.337	1.037
348	Allantoin	Nucleotide	2	AT_Untreated_2_3	AT_Untreated_2	1.0	Purine degradation	True	3.318e+04	-1.475	-1.475	1.355
335	Inosine	Nucleotide	2	AT_Untreated_2_3	AT_Untreated_2	1.0	Purine nucleosides	True	3.045e+05	1.723	1.723	-1.138
81	Ile	Amino acid	2	AT_Untreated_2_3	AT_Untreated_2	1.0	Proteinogenic amino acids	True	2.443e+07	8.05	8.05	0.1942
72	Ala	Amino acid	2	AT_Untreated_2_3	AT_Untreated_2	1.0	Proteinogenic amino acids	True	1.043e+07	6.821	6.821	-0.9125
79	Thr	Amino acid	2	AT_Untreated_2_3	AT_Untreated_2	1.0	Proteinogenic amino acids	True	2.189e+06	4.57	4.57	-1.393
88	Tyr	Amino acid	2	AT_Untreated_2_3	AT_Untreated_2	1.0	Proteinogenic amino acids	True	9.955e+06	6.754	6.754	-0.08806
84	Lys	Amino acid	2	AT_Untreated_2_3	AT_Untreated_2	1.0	Proteinogenic amino acids	True	1.498e+06	4.022	4.022	0.3029
86	Met	Amino acid	2	AT_Untreated_2_3	AT_Untreated_2	1.0	Proteinogenic amino acids	True	3.906e+06	5.405	5.405	-0.3825
61	Malate	Carbon	2	AT_Untreated_2_3	AT_Untreated_2	1.0	TCA cycle	True	1.811e+05	0.9742	0.9742	0.07743
235	Putrescine	Amino acid	2	AT_Untreated_2_3	AT_Untreated_2	0.666666666666667	Polyamines	True	2.679e+04	-1.783	-1.783	-0.2155
49	3-P-Glycerate	Carbon	2	AT_Untreated_2_3	AT_Untreated_2	1.0	Glycolysis, GNG	True	7.51e+05	3.026	3.026	0.5633
139	GABA	Amino acid	2	AT_Untreated_2_3	AT_Untreated_2	0.5	Amino acid derivatives	False			-4.963	-1.493
189	Kynurenate	Amino acid	2	AT_Untreated_2_3	AT_Untreated_2	1.0	Amino acid derivatives	True	6920	-3.736	-3.736	1.452
234	5-Me-Thioadenosine	Amino acid	2	AT_Untreated_2_3	AT_Untreated_2	1.0	SAM metabolism	True	2.821e+04	-1.708	-1.708	-1.612
59	Succinate	Carbon	2	AT_Untreated_2_3	AT_Untreated_2	0.833333333333333	TCA cycle	False			-2.963	-0.9227
133	Ornithine	Amino acid	2	AT_Untreated_2_3	AT_Untreated_2	1.0	Amino acids degradation intermediates	True	6.921e+05	2.908	2.908	0.3869

Metabolite ID	Name	Super Pathway	Dataset	Sample ID	Group ID	Detection Fraction	Pathway	Detected	Raw Intensity	Log2 Norm Intensity	Norm Imputed	Log2 Ctrl Norm
313	5-Oxoproline	Amino acid	2	AT_Untreated_2_3	AT_Untreated_2	1.0	Glutathione derivatives	True	1.174e+06	3.671	3.671	1.667
724	Pantothenate	Cofactor	2	AT_Untreated_2_3	AT_Untreated_2	1.0	Coenzyme A biosynthesis	True	1.378e+06	3.902	3.902	0.7887
30	Sucrose	Carbon	2	AT_Untreated_2_3	AT_Untreated_2	1.0	Sugars and sugar alcohols	True	3.886e+05	2.075	2.075	-0.3013
122	3-OH-Isobutyrate	Amino acid	2	AT_Untreated_2_3	AT_Untreated_2	0.666666666666667	Amino acids degradation intermediates	False			-4.628	0
241	4-Acetamidobutanoate	Amino acid	2	AT_Untreated_2_3	AT_Untreated_2	1.0	Polyamine derivatives	True	1.065e+05	0.2086	0.2086	1.716
55	Citrate	Carbon	2	AT_Untreated_2_3	AT_Untreated_2	1.0	TCA cycle	True	7.012e+05	2.927	2.927	-0.1074
338	Guanosine	Nucleotide	2	AT_Untreated_2_3	AT_Untreated_2	1.0	Purine nucleosides	True	4.24e+05	2.201	2.201	-0.1592
170	2-Amino-Butyrate	Amino acid	2	AT_Untreated_2_3	AT_Untreated_2	1.0	Amino acid derivatives	True	2.53e+05	1.456	1.456	0.2324
209	N-Ac-Ala	Amino acid	2	AT_Untreated_2_3	AT_Untreated_2	0.666666666666667	N-acetylated amino acids	False			-4.401	-1.133
221	N-Ac-Met	Amino acid	2	AT_Untreated_2_3	AT_Untreated_2	1.0	N-acetylated amino acids	True	1.603e+04	-2.524	-2.524	-1.373
22	N-Ac-Neuraminate	Carbon	2	AT_Untreated_2_3	AT_Untreated_2	1.0	Aminosugar derivatives	True	6.39e+04	-0.529	-0.529	-0.315
346	Urate	Nucleotide	2	AT_Untreated_2_3	AT_Untreated_2	1.0	Purine degradation	True	1.847e+04	-2.32	-2.32	1.372
90	Arg	Amino acid	2	AT_Untreated_2_3	AT_Untreated_2	1.0	Proteinogenic amino acids	True	2.318e+06	4.652	4.652	-0.1302
60	Fumarate	Carbon	2	AT_Untreated_2_3	AT_Untreated_2	1.0	TCA cycle	True	6.777e+04	-0.4441	-0.4441	-0.2874
78	Ser	Amino acid	2	AT_Untreated_2_3	AT_Untreated_2	1.0	Proteinogenic amino acids	True	2.959e+06	5.004	5.004	-1.823
83	Val	Amino acid	2	AT_Untreated_2_3	AT_Untreated_2	1.0	Proteinogenic amino acids	True	1.529e+07	7.373	7.373	0.1392
734	Pyridoxal	Cofactor	2	AT_Untreated_2_3	AT_Untreated_2	1.0	PLP biosynthesis and salvage	True	1.76e+05	0.9328	0.9328	1.927
136	Urea	Amino acid	2	AT_Untreated_2_3	AT_Untreated_2	1.0	Amino acids degradation intermediates	True	4.096e+05	2.151	2.151	0.5062
742	Folate	Cofactor	2	AT_Untreated_2_3	AT_Untreated_2	1.0	Folate metabolism	True	9.734e+04	0.07829	0.07829	0.5953
729	Riboflavin (Vitamin B2)	Cofactor	2	AT_Untreated_2_3	AT_Untreated_2	1.0	Flavine biosynthesis	True	6.377e+04	-0.5319	-0.5319	1.037
91	Pro	Amino acid	2	AT_Untreated_2_3	AT_Untreated_2	1.0	Proteinogenic amino acids	True	5.193e+06	5.816	5.816	-0.9055
308	Glutathione, Reduced	Amino acid	2	AT_Untreated_2_3	AT_Untreated_2	1.0	Glutathione	True	1.276e+06	3.79	3.79	-2.665

Metabolite ID	Name	Super Pathway	Dataset	Sample ID	Group ID	Detection Fraction	Pathway	Detected	Raw Intensity	Log2 Norm Intensity	Norm Imputed	Log2 Ctrl Norm
706	FAD	Cofactor	2	AT_Untreated_2_3	AT_Untreated_2	0.833333333333333	Cofactors	True	1.149e+04	-3.004	-3.004	2.092
299	gamma-Glu-Tyr	Amino acid	2	AT_Untreated_2_3	AT_Untreated_2	1.0	Gamma-glutamyl dipeptides	True	3.218e+04	-1.519	-1.519	-0.3515
705	Coenzyme A	Cofactor	2	AT_Untreated_2_3	AT_Untreated_2	1.0	Cofactors	True	1193	-6.272	-6.272	-2.566
342	Hypoxanthine	Nucleotide	2	AT_Untreated_2_3	AT_Untreated_2	1.0	Purine bases	True	9.793e+04	0.08695	0.08695	0.08507
344	Xanthine	Nucleotide	2	AT_Untreated_2_3	AT_Untreated_2	0.833333333333333	Purine bases	True	5.989e+04	-0.6225	-0.6225	1.617
703	NAD+	Cofactor	2	AT_Untreated_2_3	AT_Untreated_2	1.0	Cofactors	True	3.369e+05	1.869	1.869	-1.689
731	Thiamin (Vitamin B1)	Cofactor	2	AT_Untreated_2_3	AT_Untreated_2	1.0	TPP biosynthesis	True	9.047e+04	-0.02739	-0.02739	0.1787
102	2-Aminoadipate	Amino acid	2	AT_Untreated_2_3	AT_Untreated_2	1.0	Amino acids biosynthesis intermediates	True	5.508e+04	-0.7432	-0.7432	-1.5
77	Gly	Amino acid	2	AT_Untreated_2_3	AT_Untreated_2	1.0	Proteinogenic amino acids	True	5.363e+06	5.862	5.862	-1.366
45	Fructose-6-P	Carbon	2	AT_Untreated_2_3	AT_Untreated_2	0.666666666666667	Glycolysis, GNG	True	2.545e+04	-1.857	-1.857	-0.4216
36	Ribose	Carbon	2	AT_Untreated_2_3	AT_Untreated_2	0.833333333333333	Sugars and sugar alcohols	True	4.163e+04	-1.147	-1.147	-0.1495
4	GlcNAc 6-P	Carbon	2	AT_Untreated_2_3	AT_Untreated_2	1.0	Aminosugar biosynthesis	True	7.15e+04	-0.367	-0.367	-0.7267
188	Kynurenine	Amino acid	2	AT_Untreated_2_3	AT_Untreated_2	1.0	Amino acid derivatives	True	1.139e+05	0.3051	0.3051	0.3191
63	6-P-Gluconate	Carbon	2	AT_Untreated_2_3	AT_Untreated_2	1.0	Pentose phosphate pathway (PPP)	True	4.59e+04	-1.006	-1.006	0.03892
710	Carnitine	Cofactor	2	AT_Untreated_2_3	AT_Untreated_2	1.0	Cofactors	True	2.037e+05	1.144	1.144	-1.388
725	P-Pantetheine	Cofactor	2	AT_Untreated_2_3	AT_Untreated_2	1.0	Coenzyme A biosynthesis	True	5530	-4.06	-4.06	0.7529
110	N-alpha-Ac-Ornithine	Amino acid	2	AT_Untreated_2_3	AT_Untreated_2	1.0	Amino acids biosynthesis intermediates	True	1.943e+05	1.075	1.075	0.1011
116	3-Me-2-Oxo-Valerate	Amino acid	2	AT_Untreated_2_3	AT_Untreated_2	1.0	Amino acids degradation intermediates	True	7.903e+04	-0.2223	-0.2223	3.352
155	4-Guanidinobutanoate	Amino acid	2	AT_Untreated_2_3	AT_Untreated_2	1.0	Amino acid derivatives	True	7.185e+04	-0.3598	-0.3598	0.3596
310	S-Lactoyl-Glutathione	Amino acid	2	AT_Untreated_2_3	AT_Untreated_2	0.666666666666667	Glutathione derivatives	False			-4.634	-0.962
34	Ribitol	Carbon	2	AT_Untreated_2_3	AT_Untreated_2	1.0	Sugars and sugar alcohols	True	3.254e+04	-1.503	-1.503	0.07625

Metabolite ID	Name	Super Pathway	Dataset	Sample ID	Group ID	Detection Fraction	Pathway	Detected	Raw Intensity	Log2 Norm Intensity	Norm Imputed	Log2 Ctrl Norm
707	FMN	Cofactor	2	AT_Untreated_2_3	AT_Untreated_2	0.666666666666667	Cofactors	False			-4.56	-1.139
17	Maltose	Carbon	2	AT_Untreated_2_3	AT_Untreated_2	1.0	Glycogen degradation	True	1.612e+05	0.8058	0.8058	0.8452
18	Maltotriose	Carbon	2	AT_Untreated_2_3	AT_Untreated_2	1.0	Glycogen degradation	True	1.359e+06	3.881	3.881	2.452
19	Maltotetraose	Carbon	2	AT_Untreated_2_3	AT_Untreated_2	1.0	Glycogen degradation	True	2.278e+05	1.305	1.305	1.495
232	SAH	Amino acid	2	AT_Untreated_2_3	AT_Untreated_2	1.0	SAM metabolism	True	1.143e+04	-3.012	-3.012	0.2146
74	Asp	Amino acid	2	AT_Untreated_2_3	AT_Untreated_2	1.0	Proteinogenic amino acids	True	7.971e+05	3.112	3.112	-2.548
129	5-Aminovalerate	Amino acid	2	AT_Untreated_2_3	AT_Untreated_2	0.833333333333333	Amino acids degradation intermediates	False			-1.567	-0.7538
254	Gly-Val	Amino acid	2	AT_Untreated_2_3	AT_Untreated_2	1.0	Dipeptides	True	8.486e+04	-0.1197	-0.1197	-0.04593
291	gamma-Glu-Leu	Amino acid	2	AT_Untreated_2_3	AT_Untreated_2	1.0	Gamma-glutamyl dipeptides	True	1.037e+05	0.1698	0.1698	1.252
173	Met Sulfoxide	Amino acid	2	AT_Untreated_2_3	AT_Untreated_2	1.0	Amino acid derivatives	True	3.612e+05	1.97	1.97	2.109
43	Glucose	Carbon	2	AT_Untreated_2_3	AT_Untreated_2	1.0	Glycolysis, GNG	True	1.456e+07	7.303	7.303	0.4832
249	Gly-Gly	Amino acid	2	AT_Untreated_2_3	AT_Untreated_2	1.0	Dipeptides	True	5.105e+04	-0.853	-0.853	-1.07
169	2-OH-Butyrate	Amino acid	2	AT_Untreated_2_3	AT_Untreated_2	0.833333333333333	Amino acid derivatives	False			-1.729	-0.2955
98	3-Methyl-2-Oxobutyrate	Amino acid	2	AT_Untreated_2_3	AT_Untreated_2	0.833333333333333	Amino acids biosynthesis intermediates	True	3.158e+04	-1.546	-1.546	2.213
100	4-Me-2-Oxo-Pentanoate	Amino acid	2	AT_Untreated_2_3	AT_Untreated_2	1.0	Amino acids biosynthesis intermediates	True	8.078e+04	-0.1908	-0.1908	3.111
253	Gly-Pro	Amino acid	2	AT_Untreated_2_3	AT_Untreated_2	1.0	Dipeptides	True	7.384e+04	-0.3204	-0.3204	7.04e-04
247	Asp-Phe	Amino acid	2	AT_Untreated_2_3	AT_Untreated_2	1.0	Dipeptides	True	3.389e+04	-1.444	-1.444	-0.5028
212	N-Ac-Asp	Amino acid	2	AT_Untreated_2_3	AT_Untreated_2	0.333333333333333	N-acetylated amino acids	False			-4.689	-0.1861
720	1-Me-Nicotinamide	Cofactor	2	AT_Untreated_2_3	AT_Untreated_2	1.0	Derivatives of NA, nicotinamide and NAD	True	8.463e+05	3.198	3.198	-0.5053
70	Creatine	Carbon	2	AT_Untreated_2_3	AT_Untreated_2	1.0	Creatine energy storage	True	4.471e+06	5.6	5.6	-0.1806
309	Glutathione, Oxidized	Amino acid	2	AT_Untreated_2_3	AT_Untreated_2	1.0	Glutathione	True	1.49e+06	4.015	4.015	0.06607
44	Glucose 6-P	Carbon	2	AT_Untreated_2_3	AT_Untreated_2	1.0	Glycolysis, GNG	True	9.692e+04	0.072	0.072	-0.7428

Metabolite ID	Name	Super Pathway	Dataset	Sample ID	Group ID	Detection Fraction	Pathway	Detected	Raw Intensity	Log2 Norm Intensity	Norm Imputed	Log2 Ctrl Norm
24	Fructose	Carbon	2	AT_Untreated_2_3	AT_Untreated_2	1.0	Sugars and sugar alcohols	True	1.937e+06	4.393	4.393	1.033
85	Cys	Amino acid	2	AT_Untreated_2_3	AT_Untreated_2	1.0	Proteinogenic amino acids	True	9.371e+04	0.02335	0.02335	0.6152
704	NADH	Cofactor	2	AT_Untreated_2_3	AT_Untreated_2	0.666666666666667	Cofactors	False			-4.313	-2.383
275	Thr-Phe	Amino acid	2	AT_Untreated_2_3	AT_Untreated_2	0.833333333333333	Dipeptides	False			-1.922	-0.9888
738	Pyridoxate	Cofactor	2	AT_Untreated_2_3	AT_Untreated_2	1.0	PLP biosynthesis and salvage	True	1.714e+04	-2.427	-2.427	2.238
177	3-(4-OH-Phenyl)Lactate	Amino acid	2	AT_Untreated_2_3	AT_Untreated_2	1.0	Amino acid derivatives	True	1.921e+04	-2.263	-2.263	1.69
206	Trans-4-OH-Pro	Amino acid	2	AT_Untreated_2_3	AT_Untreated_2	1.0	Amino acid derivatives	True	1.71e+05	0.8907	0.8907	1.086
329	AMP	Nucleotide	2	AT_Untreated_2_3	AT_Untreated_2	0.833333333333333	Purine nucleotides	False			-2.644	-3.064
345	Guanine	Nucleotide	2	AT_Untreated_2_3	AT_Untreated_2	1.0	Purine bases	True	3.403e+05	1.884	1.884	0.3018
271	pyroGlu-Val	Amino acid	2	AT_Untreated_2_3	AT_Untreated_2	1.0	Dipeptides	True	5145	-4.164	-4.164	-0.522
279	Val-Glu	Amino acid	2	AT_Untreated_2_3	AT_Untreated_2	1.0	Dipeptides	True	4.103e+04	-1.168	-1.168	-0.3382
183	Phenol Sulfate	Amino acid	2	AT_Untreated_2_3	AT_Untreated_2	1.0	Amino acid derivatives	True	2.591e+04	-1.831	-1.831	1.896
740	3-Dehydrocarnitine	Cofactor	2	AT_Untreated_2_3	AT_Untreated_2	1.0	Carnitine biosynthesis	True	2.008e+04	-2.199	-2.199	-1.865
145	Pyro-Gln	Amino acid	2	AT_Untreated_2_3	AT_Untreated_2	1.0	Amino acid derivatives	True	1.705e+05	0.887	0.887	0.5411
197	C-Glycosyl-Trp	Amino acid	2	AT_Untreated_2_3	AT_Untreated_2	1.0	Amino acid derivatives	True	1.278e+05	0.4708	0.4708	0.6145
718	Nicotinamide Riboside	Cofactor	2	AT_Untreated_2_3	AT_Untreated_2	0.333333333333333	NAD biosynthesis	False			-2.306	-0.5023
295	gamma-Glu-Phe	Amino acid	2	AT_Untreated_2_3	AT_Untreated_2	1.0	Gamma-glutamyl dipeptides	True	6.726e+04	-0.455	-0.455	-0.3531
399	Pseudouridine	Nucleotide	2	AT_Untreated_2_3	AT_Untreated_2	1.0	Pyrimidine derivatives in RNAs	True	1.605e+04	-2.522	-2.522	1.718
375	UTP	Nucleotide	2	AT_Untreated_2_3	AT_Untreated_2	0.166666666666667	Pyrimidine nucleotides	False			-5.966	-0.5235
20	Erythronate	Carbon	2	AT_Untreated_2_3	AT_Untreated_2	1.0	Aminosugar derivatives	True	2.141e+04	-2.107	-2.107	-0.6652
151	Phenylacetylglycine	Amino acid	2	AT_Untreated_2_3	AT_Untreated_2	1.0	Amino acid derivatives	True	3.022e+04	-1.609	-1.609	2.812
252	Gly-Phe	Amino acid	2	AT_Untreated_2_3	AT_Untreated_2	0.666666666666667	Dipeptides	True	1.547e+05	0.7464	0.7464	0.6422

Metabolite ID	Name	Super Pathway	Dataset	Sample ID	Group ID	Detection Fraction	Pathway	Detected	Raw Intensity	Log2 Norm Intensity	Norm Imputed	Log2 Ctrl Norm
251	Gly-Leu	Amino acid	2	AT_Untreated_2_3	AT_Untreated_2	1.0	Dipeptides	True	1.015e+05	0.139	0.139	-0.2236
290	gamma-Glu-Ile	Amino acid	2	AT_Untreated_2_3	AT_Untreated_2	0.833333333333333	Gamma-glutamyl dipeptides	True	3.11e+04	-1.568	-1.568	-0.03684
316	Ophthalmate	Amino acid	2	AT_Untreated_2_3	AT_Untreated_2	1.0	Oxidative stress markers	True	3.06e+04	-1.591	-1.591	-1.845
208	Pro-OH-Pro	Amino acid	2	AT_Untreated_2_3	AT_Untreated_2	1.0	Amino acid derivatives	True	1.613e+05	0.807	0.807	0.3474
352	3'-AMP	Nucleotide	2	AT_Untreated_2_3	AT_Untreated_2	1.0	Purine derivatives in signaling	True	4.469e+04	-1.045	-1.045	0.2401
314	Cys-Glutathione Disulfide	Amino acid	2	AT_Untreated_2_3	AT_Untreated_2	1.0	Oxidative stress markers	True	1.388e+05	0.5902	0.5902	1.956
39	Threitol	Carbon	2	AT_Untreated_2_3	AT_Untreated_2	0.5	Sugars and sugar alcohols	False			-4.395	-0.3698
31	Ribulose/Xylulose	Carbon	2	AT_Untreated_2_3	AT_Untreated_2	0.0	Sugars and sugar alcohols	False			-5.004	-0.09716
48	DHAP	Carbon	2	AT_Untreated_2_3	AT_Untreated_2	1.0	Glycolysis, GNG	True	7.755e+04	-0.2497	-0.2497	-2.008
182	P-Cresol Sulfate	Amino acid	2	AT_Untreated_2_3	AT_Untreated_2	1.0	Amino acid derivatives	True	1.316e+04	-2.808	-2.808	0.5291
250	Gly-Ile	Amino acid	2	AT_Untreated_2_3	AT_Untreated_2	1.0	Dipeptides	True	4.714e+04	-0.9678	-0.9678	0.4562
286	gamma-Glu-Glu	Amino acid	2	AT_Untreated_2_3	AT_Untreated_2	0.833333333333333	Gamma-glutamyl dipeptides	False			-2.475	-2.245
264	Leu-Leu	Amino acid	2	AT_Untreated_2_3	AT_Untreated_2	1.0	Dipeptides	True	2.319e+04	-1.991	-1.991	-0.9182
203	DiMe-Arg	Amino acid	2	AT_Untreated_2_3	AT_Untreated_2	1.0	Amino acid derivatives	True	2.16e+05	1.228	1.228	0.1535
47	Fructose 1,6-PP, Glucose 1,6-PP	Carbon	2	AT_Untreated_2_3	AT_Untreated_2	1.0	Glycolysis, GNG	True	2.335e+04	-1.982	-1.982	-1.691
224	N-Ac-Ser	Amino acid	2	AT_Untreated_2_3	AT_Untreated_2	0.833333333333333	N-acetylated amino acids	True	5.412e+04	-0.7685	-0.7685	-1.639
244	Ala-Leu	Amino acid	2	AT_Untreated_2_3	AT_Untreated_2	0.666666666666667	Dipeptides	False			-1.778	-2.118
304	Cyclo(Phe-Pro)	Amino acid	2	AT_Untreated_2_3	AT_Untreated_2	0.0	Cyclic dipeptides	False			-1.192	-0.7201
302	Cyclo(Glu-Glu)	Amino acid	2	AT_Untreated_2_3	AT_Untreated_2	0.666666666666667	Cyclic dipeptides	True	4.595e+04	-1.005	-1.005	0.1674
303	Cyclo(Leu-Pro)	Amino acid	2	AT_Untreated_2_3	AT_Untreated_2	0.0	Cyclic dipeptides	False			-0.4297	-0.7026
390	2',3'-cUMP	Nucleotide	2	AT_Untreated_2_3	AT_Untreated_2	1.0	Pyrimidine derivatives in signaling	True	1.946e+04	-2.245	-2.245	-1.116
68	Ribulose 5-P / Xylulose 5-P	Carbon	2	AT_Untreated_2_3	AT_Untreated_2	1.0	Pentose phosphate pathway (PPP)	True	5.375e+04	-0.7785	-0.7785	-1.548
388	2',3'-cCMP	Nucleotide	2	AT_Untreated_2_3	AT_Untreated_2	1.0	Pyrimidine derivatives in signaling	True	6738	-3.774	-3.774	-4.028

Metabolite ID	Name	Super Pathway	Dataset	Sample ID	Group ID	Detection Fraction	Pathway	Detected	Raw Intensity	Log2 Norm Intensity	Norm Imputed	Log2 Ctrl Norm
33	Arabitol/Xylitol	Carbon	2	AT_Untreated_2_3	AT_Untreated_2	0.5	Sugars and sugar alcohols	False			-3.559	-0.05408
268	Phe-Phe	Amino acid	2	AT_Untreated_2_3	AT_Untreated_2	0.5	Dipeptides	True	2.617e+04	-1.817	-1.817	-0.729
245	Ala-Phe	Amino acid	2	AT_Untreated_2_3	AT_Untreated_2	0.666666666666667	Dipeptides	False			-2.821	-1.366
373	UMP	Nucleotide	2	AT_Untreated_2_3	AT_Untreated_2	0.5	Pyrimidine nucleotides	False			-3.442	-1.402
282	Val-Leu	Amino acid	2	AT_Untreated_2_3	AT_Untreated_2	0.666666666666667	Dipeptides	False			-1.414	-1.045
258	Ile-Gly	Amino acid	2	AT_Untreated_2_3	AT_Untreated_2	0.833333333333333	Dipeptides	False			-2.663	-3.077
259	Ile-Ser	Amino acid	2	AT_Untreated_2_3	AT_Untreated_2	0.666666666666667	Dipeptides	True	4.015e+04	-1.2	-1.2	-0.3365
269	Phe-Ser	Amino acid	2	AT_Untreated_2_3	AT_Untreated_2	0.666666666666667	Dipeptides	False			-3.197	-1.882
277	Tyr-Ala	Amino acid	2	AT_Untreated_2_3	AT_Untreated_2	1.0	Dipeptides	True	4.375e+04	-1.076	-1.076	-1.646
257	Ile-Gln	Amino acid	2	AT_Untreated_2_3	AT_Untreated_2	0.833333333333333	Dipeptides	False			-2.625	-1.706
261	Leu-Glu	Amino acid	2	AT_Untreated_2_3	AT_Untreated_2	1.0	Dipeptides	True	2.973e+04	-1.633	-1.633	-2.423
263	Leu-Gly	Amino acid	2	AT_Untreated_2_3	AT_Untreated_2	1.0	Dipeptides	True	7.354e+04	-0.3264	-0.3264	-1.033
256	Ile-Ala	Amino acid	2	AT_Untreated_2_3	AT_Untreated_2	0.833333333333333	Dipeptides	True	7.663e+04	-0.2669	-0.2669	-0.2252
274	Thr-Leu	Amino acid	2	AT_Untreated_2_3	AT_Untreated_2	0.833333333333333	Dipeptides	False			0.2082	-0.6429
273	Ser-Phe	Amino acid	2	AT_Untreated_2_3	AT_Untreated_2	0.666666666666667	Dipeptides	False			-2.449	-1.227
272	Ser-Leu	Amino acid	2	AT_Untreated_2_3	AT_Untreated_2	1.0	Dipeptides	True	7.82e+04	-0.2377	-0.2377	-0.9765
246	Asp-Leu	Amino acid	2	AT_Untreated_2_3	AT_Untreated_2	1.0	Dipeptides	True	6.812e+04	-0.4366	-0.4366	-0.3626
76	Gln	Amino acid	2	AT_Untreated_2_4	AT_Untreated_2	1.0	Proteinogenic amino acids	True	8.786e+06	6.449	6.449	-0.503
89	Trp	Amino acid	2	AT_Untreated_2_4	AT_Untreated_2	1.0	Proteinogenic amino acids	True	4.942e+06	5.619	5.619	-0.3477
723	beta-Ala	Cofactor	2	AT_Untreated_2_4	AT_Untreated_2	1.0	Coenzyme A biosynthesis	True	1.068e+05	0.0873	0.0873	-0.239
75	Glu	Amino acid	2	AT_Untreated_2_4	AT_Untreated_2	1.0	Proteinogenic amino acids	True	6.448e+06	6.003	6.003	0.05488
80	His	Amino acid	2	AT_Untreated_2_4	AT_Untreated_2	1.0	Proteinogenic amino acids	True	9.465e+04	-0.0873	-0.0873	-0.3014
82	Leu	Amino acid	2	AT_Untreated_2_4	AT_Untreated_2	1.0	Proteinogenic amino acids	True	2.36e+07	7.875	7.875	-0.2854

Metabolite ID	Name	Super Pathway	Dataset	Sample ID	Group ID	Detection Fraction	Pathway	Detected	Raw Intensity	Log2 Norm Intensity	Norm Imputed	Log2 Ctrl Norm
87	Phe	Amino acid	2	AT_Untreated_2_4	AT_Untreated_2	1.0	Proteinogenic amino acids	True	1.881e+07	7.547	7.547	-0.3445
236	Spermidine	Amino acid	2	AT_Untreated_2_4	AT_Untreated_2	1.0	Polyamines	True	7.834e+04	-0.3602	-0.3602	-3.188
73	Asn	Amino acid	2	AT_Untreated_2_4	AT_Untreated_2	1.0	Proteinogenic amino acids	True	2.906e+05	1.531	1.531	-1.218
243	Creatinine	Amino acid	2	AT_Untreated_2_4	AT_Untreated_2	1.0	Creatine degradation	True	8.657e+05	3.106	3.106	1.535
376	Cytidine	Nucleotide	2	AT_Untreated_2_4	AT_Untreated_2	1.0	Pyrimidine nucleosides	True	6.103e+05	2.601	2.601	3.186
41	Lactate	Carbon	2	AT_Untreated_2_4	AT_Untreated_2	1.0	Respiratory carbon sources	True	2.884e+07	8.164	8.164	0.342
93	3-P-Ser	Amino acid	2	AT_Untreated_2_4	AT_Untreated_2	0.333333333333333	Amino acids biosynthesis intermediates	False			-4.967	-1.709
343	Adenine	Nucleotide	2	AT_Untreated_2_4	AT_Untreated_2	1.0	Purine bases	True	2.09e+04	-2.266	-2.266	-0.8026
336	Adenosine	Nucleotide	2	AT_Untreated_2_4	AT_Untreated_2	1.0	Purine nucleosides	True	1.136e+05	0.1761	0.1761	-2.961
29	Raffinose	Carbon	2	AT_Untreated_2_4	AT_Untreated_2	0.166666666666667	Sugars and sugar alcohols	False			-5.058	-2.878
717	Nicotinamide	Cofactor	2	AT_Untreated_2_4	AT_Untreated_2	1.0	NAD biosynthesis	True	3.793e+05	1.915	1.915	-0.4815
51	PEP	Carbon	2	AT_Untreated_2_4	AT_Untreated_2	1.0	Glycolysis, GNG	True	6.774e+04	-0.5701	-0.5701	0.5019
52	Pyruvate	Carbon	2	AT_Untreated_2_4	AT_Untreated_2	1.0	Glycolysis, GNG	True	8356	-3.589	-3.589	-1.215
237	Spermine	Amino acid	2	AT_Untreated_2_4	AT_Untreated_2	0.666666666666667	Polyamines	False			0.9044	-2.484
385	Uracil	Nucleotide	2	AT_Untreated_2_4	AT_Untreated_2	1.0	Pyrimidine bases	True	3.111e+04	-1.693	-1.693	0.03417
377	Uridine	Nucleotide	2	AT_Untreated_2_4	AT_Untreated_2	1.0	Pyrimidine nucleosides	True	5.26e+05	2.387	2.387	-0.4954
112	trans-Urocanate	Amino acid	2	AT_Untreated_2_4	AT_Untreated_2	1.0	Amino acids degradation intermediates	True	1.414e+05	0.4919	0.4919	3.61
737	Pyridoxine (Vitamin B6)	Cofactor	2	AT_Untreated_2_4	AT_Untreated_2	1.0	PLP biosynthesis and salvage	True	1.533e+06	3.93	3.93	0.63
348	Allantoin	Nucleotide	2	AT_Untreated_2_4	AT_Untreated_2	1.0	Purine degradation	True	3.12e+04	-1.689	-1.689	1.141
335	Inosine	Nucleotide	2	AT_Untreated_2_4	AT_Untreated_2	1.0	Purine nucleosides	True	3.542e+05	1.817	1.817	-1.045
81	Ile	Amino acid	2	AT_Untreated_2_4	AT_Untreated_2	1.0	Proteinogenic amino acids	True	2.199e+07	7.773	7.773	-0.08296
72	Ala	Amino acid	2	AT_Untreated_2_4	AT_Untreated_2	1.0	Proteinogenic amino acids	True	1.297e+07	7.011	7.011	-0.723
79	Thr	Amino acid	2	AT_Untreated_2_4	AT_Untreated_2	1.0	Proteinogenic amino acids	True	2.753e+06	4.775	4.775	-1.187

Metabolite ID	Name	Super Pathway	Dataset	Sample ID	Group ID	Detection Fraction	Pathway	Detected	Raw Intensity	Log2 Norm Intensity	Norm Imputed	Log2 Ctrl Norm
88	Tyr	Amino acid	2	AT_Untreated_2_4	AT_Untreated_2	1.0	Proteinogenic amino acids	True	8.903e+06	6.468	6.468	-0.3743
84	Lys	Amino acid	2	AT_Untreated_2_4	AT_Untreated_2	1.0	Proteinogenic amino acids	True	1.607e+06	3.998	3.998	0.2787
86	Met	Amino acid	2	AT_Untreated_2_4	AT_Untreated_2	1.0	Proteinogenic amino acids	True	4.145e+06	5.365	5.365	-0.422
61	Malate	Carbon	2	AT_Untreated_2_4	AT_Untreated_2	1.0	TCA cycle	True	1.218e+05	0.2768	0.2768	-0.6199
235	Putrescine	Amino acid	2	AT_Untreated_2_4	AT_Untreated_2	0.666666666666667	Polyamines	False			-3.898	-2.331
49	3-P-Glycerate	Carbon	2	AT_Untreated_2_4	AT_Untreated_2	1.0	Glycolysis, GNG	True	5.565e+05	2.468	2.468	5.57e-03
139	GABA	Amino acid	2	AT_Untreated_2_4	AT_Untreated_2	0.5	Amino acid derivatives	False			-4.963	-1.493
189	Kynurenate	Amino acid	2	AT_Untreated_2_4	AT_Untreated_2	1.0	Amino acid derivatives	True	6775	-3.892	-3.892	1.296
234	5-Me-Thioadenosine	Amino acid	2	AT_Untreated_2_4	AT_Untreated_2	1.0	SAM metabolism	True	3.222e+04	-1.642	-1.642	-1.546
59	Succinate	Carbon	2	AT_Untreated_2_4	AT_Untreated_2	0.833333333333333	TCA cycle	True	2.689e+04	-1.903	-1.903	0.137
133	Ornithine	Amino acid	2	AT_Untreated_2_4	AT_Untreated_2	1.0	Amino acids degradation intermediates	True	8.785e+05	3.127	3.127	0.6059
313	5-Oxoproline	Amino acid	2	AT_Untreated_2_4	AT_Untreated_2	1.0	Glutathione derivatives	True	1.289e+06	3.681	3.681	1.677
724	Pantothenate	Cofactor	2	AT_Untreated_2_4	AT_Untreated_2	1.0	Coenzyme A biosynthesis	True	9.795e+05	3.284	3.284	0.1705
30	Sucrose	Carbon	2	AT_Untreated_2_4	AT_Untreated_2	1.0	Sugars and sugar alcohols	True	7.088e+05	2.817	2.817	0.4406
122	3-OH-Isobutyrate	Amino acid	2	AT_Untreated_2_4	AT_Untreated_2	0.666666666666667	Amino acids degradation intermediates	False			-4.628	0
241	4-Acetamidobutanoate	Amino acid	2	AT_Untreated_2_4	AT_Untreated_2	1.0	Polyamine derivatives	True	8.88e+04	-0.1795	-0.1795	1.328
55	Citrate	Carbon	2	AT_Untreated_2_4	AT_Untreated_2	1.0	TCA cycle	True	3.985e+05	1.987	1.987	-1.048
338	Guanosine	Nucleotide	2	AT_Untreated_2_4	AT_Untreated_2	1.0	Purine nucleosides	True	4.286e+05	2.091	2.091	-0.2689
170	2-Amino-Butyrate	Amino acid	2	AT_Untreated_2_4	AT_Untreated_2	1.0	Amino acid derivatives	True	3.504e+05	1.801	1.801	0.5771
209	N-Ac-Ala	Amino acid	2	AT_Untreated_2_4	AT_Untreated_2	0.666666666666667	N-acetylated amino acids	False			-4.401	-1.133
221	N-Ac-Met	Amino acid	2	AT_Untreated_2_4	AT_Untreated_2	1.0	N-acetylated amino acids	True	1.973e+04	-2.349	-2.349	-1.199
22	N-Ac-Neuraminate	Carbon	2	AT_Untreated_2_4	AT_Untreated_2	1.0	Aminosugar derivatives	True	7.351e+04	-0.4521	-0.4521	-0.238

Metabolite ID	Name	Super Pathway	Dataset	Sample ID	Group ID	Detection Fraction	Pathway	Detected	Raw Intensity	Log2 Norm Intensity	Norm Imputed	Log2 Ctrl Norm
346	Urate	Nucleotide	2	AT_Untreated_2_4	AT_Untreated_2	1.0	Purine degradation	True	2.468e+04	-2.026	-2.026	1.666
90	Arg	Amino acid	2	AT_Untreated_2_4	AT_Untreated_2	1.0	Proteinogenic amino acids	True	2.881e+06	4.841	4.841	0.05847
60	Fumarate	Carbon	2	AT_Untreated_2_4	AT_Untreated_2	1.0	TCA cycle	True	6.274e+04	-0.6806	-0.6806	-0.5238
78	Ser	Amino acid	2	AT_Untreated_2_4	AT_Untreated_2	1.0	Proteinogenic amino acids	True	6.385e+06	5.988	5.988	-0.8381
83	Val	Amino acid	2	AT_Untreated_2_4	AT_Untreated_2	1.0	Proteinogenic amino acids	True	1.5e+07	7.221	7.221	-0.01286
734	Pyridoxal	Cofactor	2	AT_Untreated_2_4	AT_Untreated_2	1.0	PLP biosynthesis and salvage	True	4.557e+04	-1.142	-1.142	-0.1472
136	Urea	Amino acid	2	AT_Untreated_2_4	AT_Untreated_2	1.0	Amino acids degradation intermediates	True	5.559e+05	2.467	2.467	0.8218
742	Folate	Cofactor	2	AT_Untreated_2_4	AT_Untreated_2	1.0	Folate metabolism	True	5.766e+04	-0.8023	-0.8023	-0.2853
729	Riboflavin (Vitamin B2)	Cofactor	2	AT_Untreated_2_4	AT_Untreated_2	1.0	Flavine biosynthesis	True	2.016e+04	-2.318	-2.318	-0.7493
91	Pro	Amino acid	2	AT_Untreated_2_4	AT_Untreated_2	1.0	Proteinogenic amino acids	True	6.418e+06	5.996	5.996	-0.7249
308	Glutathione, Reduced	Amino acid	2	AT_Untreated_2_4	AT_Untreated_2	1.0	Glutathione	True	6.004e+06	5.9	5.9	-0.5555
706	FAD	Cofactor	2	AT_Untreated_2_4	AT_Untreated_2	0.833333333333333	Cofactors	True	6643	-3.92	-3.92	1.176
299	gamma-Glu-Tyr	Amino acid	2	AT_Untreated_2_4	AT_Untreated_2	1.0	Gamma-glutamyl dipeptides	True	1.518e+04	-2.727	-2.727	-1.56
705	Coenzyme A	Cofactor	2	AT_Untreated_2_4	AT_Untreated_2	1.0	Cofactors	True	1601	-5.973	-5.973	-2.266
342	Hypoxanthine	Nucleotide	2	AT_Untreated_2_4	AT_Untreated_2	1.0	Purine bases	True	7.447e+04	-0.4334	-0.4334	-0.4353
344	Xanthine	Nucleotide	2	AT_Untreated_2_4	AT_Untreated_2	0.833333333333333	Purine bases	False			-3.616	-1.376
703	NAD+	Cofactor	2	AT_Untreated_2_4	AT_Untreated_2	1.0	Cofactors	True	4.675e+05	2.217	2.217	-1.342
731	Thiamin (Vitamin B1)	Cofactor	2	AT_Untreated_2_4	AT_Untreated_2	1.0	TPP biosynthesis	True	5.888e+04	-0.7722	-0.7722	-0.566
102	2-Aminoadipate	Amino acid	2	AT_Untreated_2_4	AT_Untreated_2	1.0	Amino acids biosynthesis intermediates	True	8.48e+04	-0.2458	-0.2458	-1.002
77	Gly	Amino acid	2	AT_Untreated_2_4	AT_Untreated_2	1.0	Proteinogenic amino acids	True	6.651e+06	6.047	6.047	-1.181
45	Fructose-6-P	Carbon	2	AT_Untreated_2_4	AT_Untreated_2	0.666666666666667	Glycolysis, GNG	True	4.051e+04	-1.312	-1.312	0.1235
36	Ribose	Carbon	2	AT_Untreated_2_4	AT_Untreated_2	0.833333333333333	Sugars and sugar alcohols	False			-3.346	-2.349
4	GlcNAc 6-P	Carbon	2	AT_Untreated_2_4	AT_Untreated_2	1.0	Aminosugar biosynthesis	True	7.671e+04	-0.3906	-0.3906	-0.7503

Metabolite ID	Name	Super Pathway	Dataset	Sample ID	Group ID	Detection Fraction	Pathway	Detected	Raw Intensity	Log2 Norm Intensity	Norm Imputed	Log2 Ctrl Norm
188	Kynurenine	Amino acid	2	AT_Untreated_2_4	AT_Untreated_2	1.0	Amino acid derivatives	True	1.994e+05	0.9875	0.9875	1.001
63	6-P-Gluconate	Carbon	2	AT_Untreated_2_4	AT_Untreated_2	1.0	Pentose phosphate pathway (PPP)	True	4.564e+04	-1.14	-1.14	-0.09442
710	Carnitine	Cofactor	2	AT_Untreated_2_4	AT_Untreated_2	1.0	Cofactors	True	2.88e+05	1.518	1.518	-1.014
725	P-Pantetheine	Cofactor	2	AT_Untreated_2_4	AT_Untreated_2	1.0	Coenzyme A biosynthesis	True	5292	-4.248	-4.248	0.5645
110	N-alpha-Ac-Ornithine	Amino acid	2	AT_Untreated_2_4	AT_Untreated_2	1.0	Amino acids biosynthesis intermediates	True	1.463e+05	0.5412	0.5412	-0.4329
116	3-Me-2-Oxo-Valerate	Amino acid	2	AT_Untreated_2_4	AT_Untreated_2	1.0	Amino acids degradation intermediates	True	3.901e+04	-1.366	-1.366	2.208
155	4-Guanidinobutanoate	Amino acid	2	AT_Untreated_2_4	AT_Untreated_2	1.0	Amino acid derivatives	True	4.189e+04	-1.263	-1.263	-0.544
310	S-Lactoyl-Glutathione	Amino acid	2	AT_Untreated_2_4	AT_Untreated_2	0.666666666666667	Glutathione derivatives	False			-4.634	-0.962
34	Ribitol	Carbon	2	AT_Untreated_2_4	AT_Untreated_2	1.0	Sugars and sugar alcohols	True	4.014e+04	-1.325	-1.325	0.2539
707	FMN	Cofactor	2	AT_Untreated_2_4	AT_Untreated_2	0.666666666666667	Cofactors	False			-4.56	-1.139
17	Maltose	Carbon	2	AT_Untreated_2_4	AT_Untreated_2	1.0	Glycogen degradation	True	2.168e+05	1.108	1.108	1.148
18	Maltotriose	Carbon	2	AT_Untreated_2_4	AT_Untreated_2	1.0	Glycogen degradation	True	9.821e+05	3.288	3.288	1.859
19	Maltotetraose	Carbon	2	AT_Untreated_2_4	AT_Untreated_2	1.0	Glycogen degradation	True	2.111e+05	1.07	1.07	1.26
232	SAH	Amino acid	2	AT_Untreated_2_4	AT_Untreated_2	1.0	SAM metabolism	True	8959	-3.489	-3.489	-0.2625
74	Asp	Amino acid	2	AT_Untreated_2_4	AT_Untreated_2	1.0	Proteinogenic amino acids	True	1.681e+06	4.064	4.064	-1.596
129	5-Aminovalerate	Amino acid	2	AT_Untreated_2_4	AT_Untreated_2	0.833333333333333	Amino acids degradation intermediates	True	6.315e+04	-0.6712	-0.6712	0.1418
254	Gly-Val	Amino acid	2	AT_Untreated_2_4	AT_Untreated_2	1.0	Dipeptides	True	1.249e+05	0.3125	0.3125	0.3862
291	gamma-Glu-Leu	Amino acid	2	AT_Untreated_2_4	AT_Untreated_2	1.0	Gamma-glutamyl dipeptides	True	6.697e+04	-0.5864	-0.5864	0.4955
173	Met Sulfoxide	Amino acid	2	AT_Untreated_2_4	AT_Untreated_2	1.0	Amino acid derivatives	True	1.284e+05	0.352	0.352	0.4908
43	Glucose	Carbon	2	AT_Untreated_2_4	AT_Untreated_2	1.0	Glycolysis, GNG	True	1.566e+07	7.283	7.283	0.4633
249	Gly-Gly	Amino acid	2	AT_Untreated_2_4	AT_Untreated_2	1.0	Dipeptides	True	4.165e+04	-1.272	-1.272	-1.489

Metabolite ID	Name	Super Pathway	Dataset	Sample ID	Group ID	Detection Fraction	Pathway	Detected	Raw Intensity	Log2 Norm Intensity	Norm Imputed	Log2 Ctrl Norm
169	2-OH-Butyrate	Amino acid	2	AT_Untreated_2_4	AT_Untreated_2	0.833333333333333	Amino acid derivatives	True	6.292e+04	-0.6765	-0.6765	0.7574
98	3-Methyl-2-Oxobutyrate	Amino acid	2	AT_Untreated_2_4	AT_Untreated_2	0.833333333333333	Amino acids biosynthesis intermediates	True	1.84e+04	-2.45	-2.45	1.309
100	4-Me-2-Oxo-Pentanoate	Amino acid	2	AT_Untreated_2_4	AT_Untreated_2	1.0	Amino acids biosynthesis intermediates	True	5.108e+04	-0.9771	-0.9771	2.325
253	Gly-Pro	Amino acid	2	AT_Untreated_2_4	AT_Untreated_2	1.0	Dipeptides	True	1.664e+05	0.7267	0.7267	1.048
247	Asp-Phe	Amino acid	2	AT_Untreated_2_4	AT_Untreated_2	1.0	Dipeptides	True	5.587e+04	-0.8479	-0.8479	0.09336
212	N-Ac-Asp	Amino acid	2	AT_Untreated_2_4	AT_Untreated_2	0.333333333333333	N-acetylated amino acids	False			-4.689	-0.1861
720	1-Me-Nicotinamide	Cofactor	2	AT_Untreated_2_4	AT_Untreated_2	1.0	Derivatives of NA, nicotinamide and NAD	True	1.476e+06	3.875	3.875	0.1717
70	Creatine	Carbon	2	AT_Untreated_2_4	AT_Untreated_2	1.0	Creatine energy storage	True	5.547e+06	5.786	5.786	5.27e-03
309	Glutathione, Oxidized	Amino acid	2	AT_Untreated_2_4	AT_Untreated_2	1.0	Glutathione	True	1.966e+06	4.289	4.289	0.3408
44	Glucose 6-P	Carbon	2	AT_Untreated_2_4	AT_Untreated_2	1.0	Glycolysis, GNG	True	1.17e+05	0.2182	0.2182	-0.5965
24	Fructose	Carbon	2	AT_Untreated_2_4	AT_Untreated_2	1.0	Sugars and sugar alcohols	True	2.391e+06	4.571	4.571	1.211
85	Cys	Amino acid	2	AT_Untreated_2_4	AT_Untreated_2	1.0	Proteinogenic amino acids	True	1.075e+05	0.09608	0.09608	0.688
704	NADH	Cofactor	2	AT_Untreated_2_4	AT_Untreated_2	0.666666666666667	Cofactors	False			-4.313	-2.383
275	Thr-Phe	Amino acid	2	AT_Untreated_2_4	AT_Untreated_2	0.833333333333333	Dipeptides	True	4.157e+04	-1.274	-1.274	-0.3408
738	Pyridoxate	Cofactor	2	AT_Untreated_2_4	AT_Untreated_2	1.0	PLP biosynthesis and salvage	True	1.132e+04	-3.151	-3.151	1.514
177	3-(4-OH-Phenyl)Lactate	Amino acid	2	AT_Untreated_2_4	AT_Untreated_2	1.0	Amino acid derivatives	True	1.502e+04	-2.743	-2.743	1.21
206	Trans-4-OH-Pro	Amino acid	2	AT_Untreated_2_4	AT_Untreated_2	1.0	Amino acid derivatives	True	2.263e+05	1.17	1.17	1.366
329	AMP	Nucleotide	2	AT_Untreated_2_4	AT_Untreated_2	0.833333333333333	Purine nucleotides	True	1.911e+04	-2.396	-2.396	-2.816
345	Guanine	Nucleotide	2	AT_Untreated_2_4	AT_Untreated_2	1.0	Purine bases	True	8.122e+05	3.014	3.014	1.432
271	pyroGlu-Val	Amino acid	2	AT_Untreated_2_4	AT_Untreated_2	1.0	Dipeptides	True	8648	-3.54	-3.54	0.102
279	Val-Glu	Amino acid	2	AT_Untreated_2_4	AT_Untreated_2	1.0	Dipeptides	True	3.2e+04	-1.652	-1.652	-0.822
183	Phenol Sulfate	Amino acid	2	AT_Untreated_2_4	AT_Untreated_2	1.0	Amino acid derivatives	True	1.061e+04	-3.245	-3.245	0.4821

Metabolite ID	Name	Super Pathway	Dataset	Sample ID	Group ID	Detection Fraction	Pathway	Detected	Raw Intensity	Log2 Norm Intensity	Norm Imputed	Log2 Ctrl Norm
740	3-Dehydrocarnitine	Cofactor	2	AT_Untreated_2_4	AT_Untreated_2	1.0	Carnitine biosynthesis	True	1.255e+05	0.3192	0.3192	0.6528
145	Pyro-Gln	Amino acid	2	AT_Untreated_2_4	AT_Untreated_2	1.0	Amino acid derivatives	True	1.437e+05	0.5152	0.5152	0.1693
197	C-Glycosyl-Trp	Amino acid	2	AT_Untreated_2_4	AT_Untreated_2	1.0	Amino acid derivatives	True	8.76e+04	-0.1991	-0.1991	-0.05544
718	Nicotinamide Riboside	Cofactor	2	AT_Untreated_2_4	AT_Untreated_2	0.333333333333333	NAD biosynthesis	True	5.305e+04	-0.9227	-0.9227	0.881
295	gamma-Glu-Phe	Amino acid	2	AT_Untreated_2_4	AT_Untreated_2	1.0	Gamma-glutamyl dipeptides	True	5.873e+04	-0.776	-0.776	-0.674
399	Pseudouridine	Nucleotide	2	AT_Untreated_2_4	AT_Untreated_2	1.0	Pyrimidine derivatives in RNAs	True	1.217e+04	-3.047	-3.047	1.194
375	UTP	Nucleotide	2	AT_Untreated_2_4	AT_Untreated_2	0.166666666666667	Pyrimidine nucleotides	False			-5.966	-0.5235
20	Erythronate	Carbon	2	AT_Untreated_2_4	AT_Untreated_2	1.0	Aminosugar derivatives	True	4.08e+04	-1.301	-1.301	0.1401
151	Phenylacetylglycine	Amino acid	2	AT_Untreated_2_4	AT_Untreated_2	1.0	Amino acid derivatives	True	4.398e+04	-1.193	-1.193	3.228
252	Gly-Phe	Amino acid	2	AT_Untreated_2_4	AT_Untreated_2	0.666666666666667	Dipeptides	True	2.486e+05	1.306	1.306	1.202
251	Gly-Leu	Amino acid	2	AT_Untreated_2_4	AT_Untreated_2	1.0	Dipeptides	True	1.613e+05	0.682	0.682	0.3194
290	gamma-Glu-Ile	Amino acid	2	AT_Untreated_2_4	AT_Untreated_2	0.833333333333333	Gamma-glutamyl dipeptides	False			-2.961	-1.429
316	Ophthalmate	Amino acid	2	AT_Untreated_2_4	AT_Untreated_2	1.0	Oxidative stress markers	True	2.69e+04	-1.902	-1.902	-2.156
208	Pro-OH-Pro	Amino acid	2	AT_Untreated_2_4	AT_Untreated_2	1.0	Amino acid derivatives	True	1.411e+05	0.4883	0.4883	0.02866
352	3'-AMP	Nucleotide	2	AT_Untreated_2_4	AT_Untreated_2	1.0	Purine derivatives in signaling	True	3.003e+04	-1.743	-1.743	-0.4585
314	Cys-Glutathione Disulfide	Amino acid	2	AT_Untreated_2_4	AT_Untreated_2	1.0	Oxidative stress markers	True	8.994e+04	-0.161	-0.161	1.204
39	Threitol	Carbon	2	AT_Untreated_2_4	AT_Untreated_2	0.5	Sugars and sugar alcohols	False			-4.395	-0.3698
31	Ribulose/Xylulose	Carbon	2	AT_Untreated_2_4	AT_Untreated_2	0.0	Sugars and sugar alcohols	False			-5.004	-0.09716
48	DHAP	Carbon	2	AT_Untreated_2_4	AT_Untreated_2	1.0	Glycolysis, GNG	True	1.287e+05	0.3558	0.3558	-1.402
182	P-Cresol Sulfate	Amino acid	2	AT_Untreated_2_4	AT_Untreated_2	1.0	Amino acid derivatives	True	1.082e+04	-3.216	-3.216	0.1214
250	Gly-Ile	Amino acid	2	AT_Untreated_2_4	AT_Untreated_2	1.0	Dipeptides	True	1.344e+05	0.4189	0.4189	1.843
286	gamma-Glu-Glu	Amino acid	2	AT_Untreated_2_4	AT_Untreated_2	0.833333333333333	Gamma-glutamyl dipeptides	True	7.906e+04	-0.3471	-0.3471	-0.1164

Metabolite ID	Name	Super Pathway	Dataset	Sample ID	Group ID	Detection Fraction	Pathway	Detected	Raw Intensity	Log2 Norm Intensity	Norm Imputed	Log2 Ctrl Norm
264	Leu-Leu	Amino acid	2	AT_Untreated_2_4	AT_Untreated_2	1.0	Dipeptides	True	3.203e+04	-1.651	-1.651	-0.5774
203	DiMe-Arg	Amino acid	2	AT_Untreated_2_4	AT_Untreated_2	1.0	Amino acid derivatives	True	3.545e+05	1.818	1.818	0.7433
47	Fructose 1,6-PP, Glucose 1,6-PP	Carbon	2	AT_Untreated_2_4	AT_Untreated_2	1.0	Glycolysis, GNG	True	3.513e+04	-1.517	-1.517	-1.227
224	N-Ac-Ser	Amino acid	2	AT_Untreated_2_4	AT_Untreated_2	0.833333333333333	N-acetylated amino acids	False			-1.752	-2.623
244	Ala-Leu	Amino acid	2	AT_Untreated_2_4	AT_Untreated_2	0.666666666666667	Dipeptides	False			-1.778	-2.118
304	Cyclo(Phe-Pro)	Amino acid	2	AT_Untreated_2_4	AT_Untreated_2	0.0	Cyclic dipeptides	False			-1.192	-0.7201
302	Cyclo(Glu-Glu)	Amino acid	2	AT_Untreated_2_4	AT_Untreated_2	0.666666666666667	Cyclic dipeptides	False			-2.527	-1.355
303	Cyclo(Leu-Pro)	Amino acid	2	AT_Untreated_2_4	AT_Untreated_2	0.0	Cyclic dipeptides	False			-0.4297	-0.7026
390	2',3'-cUMP	Nucleotide	2	AT_Untreated_2_4	AT_Untreated_2	1.0	Pyrimidine derivatives in signaling	True	2.035e+04	-2.305	-2.305	-1.176
68	Ribulose 5-P / Xylulose 5-P	Carbon	2	AT_Untreated_2_4	AT_Untreated_2	1.0	Pentose phosphate pathway (PPP)	True	7.106e+04	-0.501	-0.501	-1.27
388	2',3'-cCMP	Nucleotide	2	AT_Untreated_2_4	AT_Untreated_2	1.0	Pyrimidine derivatives in signaling	True	1.316e+04	-2.934	-2.934	-3.187
33	Arabitol/Xylitol	Carbon	2	AT_Untreated_2_4	AT_Untreated_2	0.5	Sugars and sugar alcohols	False			-3.559	-0.05408
268	Phe-Phe	Amino acid	2	AT_Untreated_2_4	AT_Untreated_2	0.5	Dipeptides	True	3.552e+04	-1.501	-1.501	-0.4138
245	Ala-Phe	Amino acid	2	AT_Untreated_2_4	AT_Untreated_2	0.666666666666667	Dipeptides	True	1.526e+04	-2.72	-2.72	-1.265
373	UMP	Nucleotide	2	AT_Untreated_2_4	AT_Untreated_2	0.5	Pyrimidine nucleotides	False			-3.442	-1.402
282	Val-Leu	Amino acid	2	AT_Untreated_2_4	AT_Untreated_2	0.666666666666667	Dipeptides	False			-1.414	-1.045
258	Ile-Gly	Amino acid	2	AT_Untreated_2_4	AT_Untreated_2	0.833333333333333	Dipeptides	True	2.917e+04	-1.785	-1.785	-2.2
259	Ile-Ser	Amino acid	2	AT_Untreated_2_4	AT_Untreated_2	0.666666666666667	Dipeptides	False			-3.192	-2.329
269	Phe-Ser	Amino acid	2	AT_Untreated_2_4	AT_Untreated_2	0.666666666666667	Dipeptides	True	4.28e+04	-1.232	-1.232	0.08251
277	Tyr-Ala	Amino acid	2	AT_Untreated_2_4	AT_Untreated_2	1.0	Dipeptides	True	8.779e+04	-0.1959	-0.1959	-0.766
257	Ile-Gln	Amino acid	2	AT_Untreated_2_4	AT_Untreated_2	0.833333333333333	Dipeptides	True	4.749e+04	-1.082	-1.082	-0.1636
261	Leu-Glu	Amino acid	2	AT_Untreated_2_4	AT_Untreated_2	1.0	Dipeptides	True	2.888e+04	-1.8	-1.8	-2.59
263	Leu-Gly	Amino acid	2	AT_Untreated_2_4	AT_Untreated_2	1.0	Dipeptides	True	3.647e+04	-1.463	-1.463	-2.17

Metabolite ID	Name	Super Pathway	Dataset	Sample ID	Group ID	Detection Fraction	Pathway	Detected	Raw Intensity	Log2 Norm Intensity	Norm Imputed	Log2 Ctrl Norm
256	Ile-Ala	Amino acid	2	AT_Untreated_2_4	AT_Untreated_2	0.833333333333333	Dipeptides	True	1.14e+05	0.181	0.181	0.2227
274	Thr-Leu	Amino acid	2	AT_Untreated_2_4	AT_Untreated_2	0.833333333333333	Dipeptides	True	4.033e+05	2.004	2.004	1.153
273	Ser-Phe	Amino acid	2	AT_Untreated_2_4	AT_Untreated_2	0.666666666666667	Dipeptides	False			-2.449	-1.227
272	Ser-Leu	Amino acid	2	AT_Untreated_2_4	AT_Untreated_2	1.0	Dipeptides	True	1.314e+05	0.3861	0.3861	-0.3527
246	Asp-Leu	Amino acid	2	AT_Untreated_2_4	AT_Untreated_2	1.0	Dipeptides	True	1.169e+05	0.2177	0.2177	0.2917
76	Gln	Amino acid	2	AT_Untreated_2_5	AT_Untreated_2	1.0	Proteinogenic amino acids	True	1.53e+07	6.299	6.299	-0.6529
89	Trp	Amino acid	2	AT_Untreated_2_5	AT_Untreated_2	1.0	Proteinogenic amino acids	True	8.36e+06	5.427	5.427	-0.5395
723	beta-Ala	Cofactor	2	AT_Untreated_2_5	AT_Untreated_2	1.0	Coenzyme A biosynthesis	True	1.98e+05	0.02703	0.02703	-0.2992
75	Glu	Amino acid	2	AT_Untreated_2_5	AT_Untreated_2	1.0	Proteinogenic amino acids	True	1.152e+07	5.89	5.89	-0.0577
80	His	Amino acid	2	AT_Untreated_2_5	AT_Untreated_2	1.0	Proteinogenic amino acids	True	1.718e+05	-0.1772	-0.1772	-0.3914
82	Leu	Amino acid	2	AT_Untreated_2_5	AT_Untreated_2	1.0	Proteinogenic amino acids	True	4.814e+07	7.953	7.953	-0.2076
87	Phe	Amino acid	2	AT_Untreated_2_5	AT_Untreated_2	1.0	Proteinogenic amino acids	True	3.96e+07	7.671	7.671	-0.2209
236	Spermidine	Amino acid	2	AT_Untreated_2_5	AT_Untreated_2	1.0	Polyamines	True	9.612e+05	2.306	2.306	-0.5212
73	Asn	Amino acid	2	AT_Untreated_2_5	AT_Untreated_2	1.0	Proteinogenic amino acids	True	6.85e+05	1.818	1.818	-0.9312
243	Creatinine	Amino acid	2	AT_Untreated_2_5	AT_Untreated_2	1.0	Creatine degradation	True	1.426e+06	2.876	2.876	1.305
376	Cytidine	Nucleotide	2	AT_Untreated_2_5	AT_Untreated_2	1.0	Pyrimidine nucleosides	True	7.72e+05	1.99	1.99	2.575
41	Lactate	Carbon	2	AT_Untreated_2_5	AT_Untreated_2	1.0	Respiratory carbon sources	True	8.467e+07	8.767	8.767	0.9453
93	3-P-Ser	Amino acid	2	AT_Untreated_2_5	AT_Untreated_2	0.333333333333333	Amino acids biosynthesis intermediates	False			-4.967	-1.709
343	Adenine	Nucleotide	2	AT_Untreated_2_5	AT_Untreated_2	1.0	Purine bases	True	4.238e+04	-2.197	-2.197	-0.7334
336	Adenosine	Nucleotide	2	AT_Untreated_2_5	AT_Untreated_2	1.0	Purine nucleosides	True	2.497e+05	0.3617	0.3617	-2.776
29	Raffinose	Carbon	2	AT_Untreated_2_5	AT_Untreated_2	0.166666666666667	Sugars and sugar alcohols	True	5833	-5.058	-5.058	-2.878
717	Nicotinamide	Cofactor	2	AT_Untreated_2_5	AT_Untreated_2	1.0	NAD biosynthesis	True	1.289e+06	2.73	2.73	0.3334
51	PEP	Carbon	2	AT_Untreated_2_5	AT_Untreated_2	1.0	Glycolysis, GNG	True	1.3e+05	-0.5794	-0.5794	0.4925

Metabolite ID	Name	Super Pathway	Dataset	Sample ID	Group ID	Detection Fraction	Pathway	Detected	Raw Intensity	Log2 Norm Intensity	Norm Imputed	Log2 Ctrl Norm
52	Pyruvate	Carbon	2	AT_Untreated_2_5	AT_Untreated_2	1.0	Glycolysis, GNG	True	4.299e+04	-2.176	-2.176	0.1973
237	Spermine	Amino acid	2	AT_Untreated_2_5	AT_Untreated_2	0.666666666666667	Polyamines	True	1.579e+06	3.022	3.022	-0.3663
385	Uracil	Nucleotide	2	AT_Untreated_2_5	AT_Untreated_2	1.0	Pyrimidine bases	True	7.588e+04	-1.357	-1.357	0.3703
377	Uridine	Nucleotide	2	AT_Untreated_2_5	AT_Untreated_2	1.0	Pyrimidine nucleosides	True	9.183e+05	2.241	2.241	-0.6417
112	trans-Urocanate	Amino acid	2	AT_Untreated_2_5	AT_Untreated_2	1.0	Amino acids degradation intermediates	True	1.989e+05	0.03398	0.03398	3.152
737	Pyridoxine (Vitamin B6)	Cofactor	2	AT_Untreated_2_5	AT_Untreated_2	1.0	PLP biosynthesis and salvage	True	1.604e+06	3.045	3.045	-0.255
348	Allantoin	Nucleotide	2	AT_Untreated_2_5	AT_Untreated_2	1.0	Purine degradation	True	6.832e+04	-1.508	-1.508	1.321
335	Inosine	Nucleotide	2	AT_Untreated_2_5	AT_Untreated_2	1.0	Purine nucleosides	True	1.209e+06	2.637	2.637	-0.2238
81	Ile	Amino acid	2	AT_Untreated_2_5	AT_Untreated_2	1.0	Proteinogenic amino acids	True	3.711e+07	7.577	7.577	-0.2781
72	Ala	Amino acid	2	AT_Untreated_2_5	AT_Untreated_2	1.0	Proteinogenic amino acids	True	3.389e+07	7.446	7.446	-0.2875
79	Thr	Amino acid	2	AT_Untreated_2_5	AT_Untreated_2	1.0	Proteinogenic amino acids	True	9.041e+06	5.54	5.54	-0.4225
88	Tyr	Amino acid	2	AT_Untreated_2_5	AT_Untreated_2	1.0	Proteinogenic amino acids	True	2.119e+07	6.769	6.769	-0.0736
84	Lys	Amino acid	2	AT_Untreated_2_5	AT_Untreated_2	1.0	Proteinogenic amino acids	True	2.869e+06	3.884	3.884	0.1651
86	Met	Amino acid	2	AT_Untreated_2_5	AT_Untreated_2	1.0	Proteinogenic amino acids	True	7.509e+06	5.272	5.272	-0.515
61	Malate	Carbon	2	AT_Untreated_2_5	AT_Untreated_2	1.0	TCA cycle	True	5.64e+05	1.537	1.537	0.6405
235	Putrescine	Amino acid	2	AT_Untreated_2_5	AT_Untreated_2	0.666666666666667	Polyamines	True	1.303e+04	-3.898	-3.898	-2.331
49	3-P-Glycerate	Carbon	2	AT_Untreated_2_5	AT_Untreated_2	1.0	Glycolysis, GNG	True	1.444e+06	2.893	2.893	0.4305
139	GABA	Amino acid	2	AT_Untreated_2_5	AT_Untreated_2	0.5	Amino acid derivatives	True	1.25e+04	-3.958	-3.958	-0.4875
189	Kynurenate	Amino acid	2	AT_Untreated_2_5	AT_Untreated_2	1.0	Amino acid derivatives	True	4.226e+04	-2.201	-2.201	2.987
234	5-Me-Thioadenosine	Amino acid	2	AT_Untreated_2_5	AT_Untreated_2	1.0	SAM metabolism	True	6.419e+04	-1.598	-1.598	-1.502
59	Succinate	Carbon	2	AT_Untreated_2_5	AT_Untreated_2	0.833333333333333	TCA cycle	True	3.513e+04	-2.468	-2.468	-0.4277
133	Ornithine	Amino acid	2	AT_Untreated_2_5	AT_Untreated_2	1.0	Amino acids degradation intermediates	True	1.373e+06	2.821	2.821	0.2994

Metabolite ID	Name	Super Pathway	Dataset	Sample ID	Group ID	Detection Fraction	Pathway	Detected	Raw Intensity	Log2 Norm Intensity	Norm Imputed	Log2 Ctrl Norm
313	5-Oxoproline	Amino acid	2	AT_Untreated_2_5	AT_Untreated_2	1.0	Glutathione derivatives	True	1.453e+06	2.903	2.903	0.8991
724	Pantothenate	Cofactor	2	AT_Untreated_2_5	AT_Untreated_2	1.0	Coenzyme A biosynthesis	True	1.718e+06	3.144	3.144	0.03093
30	Sucrose	Carbon	2	AT_Untreated_2_5	AT_Untreated_2	1.0	Sugars and sugar alcohols	True	9.605e+05	2.305	2.305	-0.0712
122	3-OH-Isobutyrate	Amino acid	2	AT_Untreated_2_5	AT_Untreated_2	0.666666666666667	Amino acids degradation intermediates	True	1.677e+04	-3.535	-3.535	1.093
241	4-Acetamidobutanoate	Amino acid	2	AT_Untreated_2_5	AT_Untreated_2	1.0	Polyamine derivatives	True	1.086e+05	-0.8391	-0.8391	0.6682
55	Citrate	Carbon	2	AT_Untreated_2_5	AT_Untreated_2	1.0	TCA cycle	True	8.541e+05	2.136	2.136	-0.8983
338	Guanosine	Nucleotide	2	AT_Untreated_2_5	AT_Untreated_2	1.0	Purine nucleosides	True	1.476e+06	2.925	2.925	0.5646
170	2-Amino-Butyrate	Amino acid	2	AT_Untreated_2_5	AT_Untreated_2	1.0	Amino acid derivatives	True	4.157e+05	1.097	1.097	-0.1266
209	N-Ac-Ala	Amino acid	2	AT_Untreated_2_5	AT_Untreated_2	0.666666666666667	N-acetylated amino acids	True	1.517e+04	-3.679	-3.679	-0.4111
221	N-Ac-Met	Amino acid	2	AT_Untreated_2_5	AT_Untreated_2	1.0	N-acetylated amino acids	True	1.199e+05	-0.6961	-0.6961	0.4541
22	N-Ac-Neuraminate	Carbon	2	AT_Untreated_2_5	AT_Untreated_2	1.0	Aminosugar derivatives	True	2.539e+05	0.386	0.386	0.6
346	Urate	Nucleotide	2	AT_Untreated_2_5	AT_Untreated_2	1.0	Purine degradation	True	3.155e+04	-2.623	-2.623	1.07
90	Arg	Amino acid	2	AT_Untreated_2_5	AT_Untreated_2	1.0	Proteinogenic amino acids	True	6.638e+06	5.094	5.094	0.3122
60	Fumarate	Carbon	2	AT_Untreated_2_5	AT_Untreated_2	1.0	TCA cycle	True	2.521e+05	0.3754	0.3754	0.5322
78	Ser	Amino acid	2	AT_Untreated_2_5	AT_Untreated_2	1.0	Proteinogenic amino acids	True	1.923e+07	6.629	6.629	-0.1979
83	Val	Amino acid	2	AT_Untreated_2_5	AT_Untreated_2	1.0	Proteinogenic amino acids	True	2.666e+07	7.1	7.1	-0.1339
734	Pyridoxal	Cofactor	2	AT_Untreated_2_5	AT_Untreated_2	1.0	PLP biosynthesis and salvage	True	1.238e+05	-0.6498	-0.6498	0.3448
136	Urea	Amino acid	2	AT_Untreated_2_5	AT_Untreated_2	1.0	Amino acids degradation intermediates	True	1.221e+06	2.651	2.651	1.006
742	Folate	Cofactor	2	AT_Untreated_2_5	AT_Untreated_2	1.0	Folate metabolism	True	1.076e+05	-0.8523	-0.8523	-0.3353
729	Riboflavin (Vitamin B2)	Cofactor	2	AT_Untreated_2_5	AT_Untreated_2	1.0	Flavine biosynthesis	True	8.429e+04	-1.205	-1.205	0.3642
91	Pro	Amino acid	2	AT_Untreated_2_5	AT_Untreated_2	1.0	Proteinogenic amino acids	True	2.13e+07	6.776	6.776	0.05536
308	Glutathione, Reduced	Amino acid	2	AT_Untreated_2_5	AT_Untreated_2	1.0	Glutathione	True	1.131e+07	5.862	5.862	-0.5927

Metabolite ID	Name	Super Pathway	Dataset	Sample ID	Group ID	Detection Fraction	Pathway	Detected	Raw Intensity	Log2 Norm Intensity	Norm Imputed	Log2 Ctrl Norm
706	FAD	Cofactor	2	AT_Untreated_2_5	AT_Untreated_2	0.833333333333333	Cofactors	False			-5.096	0
299	gamma-Glu-Tyr	Amino acid	2	AT_Untreated_2_5	AT_Untreated_2	1.0	Gamma-glutamyl dipeptides	True	3.228e+04	-2.59	-2.59	-1.423
705	Coenzyme A	Cofactor	2	AT_Untreated_2_5	AT_Untreated_2	1.0	Cofactors	True	1.059e+04	-4.198	-4.198	-0.4913
342	Hypoxanthine	Nucleotide	2	AT_Untreated_2_5	AT_Untreated_2	1.0	Purine bases	True	2.253e+05	0.2132	0.2132	0.2113
344	Xanthine	Nucleotide	2	AT_Untreated_2_5	AT_Untreated_2	0.833333333333333	Purine bases	True	6.68e+04	-1.54	-1.54	0.6987
703	NAD+	Cofactor	2	AT_Untreated_2_5	AT_Untreated_2	1.0	Cofactors	True	1.409e+06	2.859	2.859	-0.7
731	Thiamin (Vitamin B1)	Cofactor	2	AT_Untreated_2_5	AT_Untreated_2	1.0	TPP biosynthesis	True	1.558e+05	-0.3185	-0.3185	-0.1124
102	2-Aminoadipate	Amino acid	2	AT_Untreated_2_5	AT_Untreated_2	1.0	Amino acids biosynthesis intermediates	True	2.275e+05	0.2277	0.2277	-0.5288
77	Gly	Amino acid	2	AT_Untreated_2_5	AT_Untreated_2	1.0	Proteinogenic amino acids	True	2.526e+07	7.022	7.022	-0.2058
45	Fructose-6-P	Carbon	2	AT_Untreated_2_5	AT_Untreated_2	0.666666666666667	Glycolysis, GNG	True	8.102e+04	-1.262	-1.262	0.1733
36	Ribose	Carbon	2	AT_Untreated_2_5	AT_Untreated_2	0.833333333333333	Sugars and sugar alcohols	True	1.634e+05	-0.2496	-0.2496	0.748
4	GlcNAc 6-P	Carbon	2	AT_Untreated_2_5	AT_Untreated_2	1.0	Aminosugar biosynthesis	True	1.207e+05	-0.6867	-0.6867	-1.046
188	Kynurenine	Amino acid	2	AT_Untreated_2_5	AT_Untreated_2	1.0	Amino acid derivatives	True	4.699e+05	1.274	1.274	1.288
63	6-P-Gluconate	Carbon	2	AT_Untreated_2_5	AT_Untreated_2	1.0	Pentose phosphate pathway (PPP)	True	4.912e+04	-1.984	-1.984	-0.9387
710	Carnitine	Cofactor	2	AT_Untreated_2_5	AT_Untreated_2	1.0	Cofactors	True	8.259e+05	2.088	2.088	-0.4442
725	P-Pantetheine	Cofactor	2	AT_Untreated_2_5	AT_Untreated_2	1.0	Coenzyme A biosynthesis	True	1.444e+04	-3.751	-3.751	1.062
110	N-alpha-Ac-Ornithine	Amino acid	2	AT_Untreated_2_5	AT_Untreated_2	1.0	Amino acids biosynthesis intermediates	True	1.598e+05	-0.2823	-0.2823	-1.256
116	3-Me-2-Oxo-Valerate	Amino acid	2	AT_Untreated_2_5	AT_Untreated_2	1.0	Amino acids degradation intermediates	True	4.229e+04	-2.2	-2.2	1.375
155	4-Guanidinobutanoate	Amino acid	2	AT_Untreated_2_5	AT_Untreated_2	1.0	Amino acid derivatives	True	5.846e+05	1.589	1.589	2.308
310	S-Lactoyl-Glutathione	Amino acid	2	AT_Untreated_2_5	AT_Untreated_2	0.666666666666667	Glutathione derivatives	True	3.691e+04	-2.396	-2.396	1.275
34	Ribitol	Carbon	2	AT_Untreated_2_5	AT_Untreated_2	1.0	Sugars and sugar alcohols	True	9.875e+04	-0.9766	-0.9766	0.6022

Metabolite ID	Name	Super Pathway	Dataset	Sample ID	Group ID	Detection Fraction	Pathway	Detected	Raw Intensity	Log2 Norm Intensity	Norm Imputed	Log2 Ctrl Norm
707	FMN	Cofactor	2	AT_Untreated_2_5	AT_Untreated_2	0.666666666666667	Cofactors	True	2.261e+04	-3.103	-3.103	0.3175
17	Maltose	Carbon	2	AT_Untreated_2_5	AT_Untreated_2	1.0	Glycogen degradation	True	2.089e+05	0.1042	0.1042	0.1436
18	Maltotriose	Carbon	2	AT_Untreated_2_5	AT_Untreated_2	1.0	Glycogen degradation	True	1.193e+06	2.618	2.618	1.189
19	Maltotetraose	Carbon	2	AT_Untreated_2_5	AT_Untreated_2	1.0	Glycogen degradation	True	3.214e+05	0.7259	0.7259	0.9163
232	SAH	Amino acid	2	AT_Untreated_2_5	AT_Untreated_2	1.0	SAM metabolism	True	2.202e+04	-3.142	-3.142	0.08446
74	Asp	Amino acid	2	AT_Untreated_2_5	AT_Untreated_2	1.0	Proteinogenic amino acids	True	3.852e+06	4.309	4.309	-1.35
129	5-Aminovalerate	Amino acid	2	AT_Untreated_2_5	AT_Untreated_2	0.833333333333333	Amino acids degradation intermediates	True	9.309e+04	-1.062	-1.062	-0.2486
254	Gly-Val	Amino acid	2	AT_Untreated_2_5	AT_Untreated_2	1.0	Dipeptides	True	2.011e+05	0.04947	0.04947	0.1232
291	gamma-Glu-Leu	Amino acid	2	AT_Untreated_2_5	AT_Untreated_2	1.0	Gamma-glutamyl dipeptides	True	1.007e+05	-0.9481	-0.9481	0.1338
173	Met Sulfoxide	Amino acid	2	AT_Untreated_2_5	AT_Untreated_2	1.0	Amino acid derivatives	True	1.812e+05	-0.101	-0.101	0.03784
43	Glucose	Carbon	2	AT_Untreated_2_5	AT_Untreated_2	1.0	Glycolysis, GNG	True	1.45e+07	6.222	6.222	-0.5978
249	Gly-Gly	Amino acid	2	AT_Untreated_2_5	AT_Untreated_2	1.0	Dipeptides	True	4.345e+05	1.161	1.161	0.9438
169	2-OH-Butyrate	Amino acid	2	AT_Untreated_2_5	AT_Untreated_2	0.833333333333333	Amino acid derivatives	True	1.058e+05	-0.8765	-0.8765	0.5574
98	3-Methyl-2-Oxobutyrate	Amino acid	2	AT_Untreated_2_5	AT_Untreated_2	0.833333333333333	Amino acids biosynthesis intermediates	True	1.436e+04	-3.759	-3.759	0
100	4-Me-2-Oxo-Pentanoate	Amino acid	2	AT_Untreated_2_5	AT_Untreated_2	1.0	Amino acids biosynthesis intermediates	True	3.37e+04	-2.528	-2.528	0.774
253	Gly-Pro	Amino acid	2	AT_Untreated_2_5	AT_Untreated_2	1.0	Dipeptides	True	1.981e+05	0.028	0.028	0.3491
247	Asp-Phe	Amino acid	2	AT_Untreated_2_5	AT_Untreated_2	1.0	Dipeptides	True	1.119e+05	-0.7962	-0.7962	0.145
212	N-Ac-Asp	Amino acid	2	AT_Untreated_2_5	AT_Untreated_2	0.333333333333333	N-acetylated amino acids	False			-4.689	-0.1861
720	1-Me-Nicotinamide	Cofactor	2	AT_Untreated_2_5	AT_Untreated_2	1.0	Derivatives of NA, nicotinamide and NAD	True	2.539e+06	3.708	3.708	4.30e-03
70	Creatine	Carbon	2	AT_Untreated_2_5	AT_Untreated_2	1.0	Creatine energy storage	True	8.624e+06	5.472	5.472	-0.3085
309	Glutathione, Oxidized	Amino acid	2	AT_Untreated_2_5	AT_Untreated_2	1.0	Glutathione	True	2.235e+06	3.524	3.524	-0.4246
44	Glucose 6-P	Carbon	2	AT_Untreated_2_5	AT_Untreated_2	1.0	Glycolysis, GNG	True	2.234e+05	0.2015	0.2015	-0.6133

Metabolite ID	Name	Super Pathway	Dataset	Sample ID	Group ID	Detection Fraction	Pathway	Detected	Raw Intensity	Log2 Norm Intensity	Norm Imputed	Log2 Ctrl Norm
24	Fructose	Carbon	2	AT_Untreated_2_5	AT_Untreated_2	1.0	Sugars and sugar alcohols	True	3.928e+06	4.337	4.337	0.9767
85	Cys	Amino acid	2	AT_Untreated_2_5	AT_Untreated_2	1.0	Proteinogenic amino acids	True	1.922e+05	-0.01573	-0.01573	0.5762
704	NADH	Cofactor	2	AT_Untreated_2_5	AT_Untreated_2	0.666666666666667	Cofactors	True	2.758e+04	-2.817	-2.817	-0.8876
275	Thr-Phe	Amino acid	2	AT_Untreated_2_5	AT_Untreated_2	0.833333333333333	Dipeptides	True	5.126e+04	-1.922	-1.922	-0.9888
738	Pyridoxate	Cofactor	2	AT_Untreated_2_5	AT_Untreated_2	1.0	PLP biosynthesis and salvage	True	1.997e+04	-3.282	-3.282	1.383
177	3-(4-OH-Phenyl)Lactate	Amino acid	2	AT_Untreated_2_5	AT_Untreated_2	1.0	Amino acid derivatives	True	1.793e+04	-3.438	-3.438	0.515
206	Trans-4-OH-Pro	Amino acid	2	AT_Untreated_2_5	AT_Untreated_2	1.0	Amino acid derivatives	True	2.171e+05	0.16	0.16	0.3556
329	AMP	Nucleotide	2	AT_Untreated_2_5	AT_Untreated_2	0.833333333333333	Purine nucleotides	True	2.345e+05	0.2709	0.2709	-0.1496
345	Guanine	Nucleotide	2	AT_Untreated_2_5	AT_Untreated_2	1.0	Purine bases	True	1.487e+06	2.936	2.936	1.354
271	pyroGlu-Val	Amino acid	2	AT_Untreated_2_5	AT_Untreated_2	1.0	Dipeptides	True	9855	-4.301	-4.301	-0.6599
279	Val-Glu	Amino acid	2	AT_Untreated_2_5	AT_Untreated_2	1.0	Dipeptides	True	1.111e+05	-0.8065	-0.8065	0.02354
183	Phenol Sulfate	Amino acid	2	AT_Untreated_2_5	AT_Untreated_2	1.0	Amino acid derivatives	True	2.773e+05	0.5131	0.5131	4.24
740	3-Dehydrocarnitine	Cofactor	2	AT_Untreated_2_5	AT_Untreated_2	1.0	Carnitine biosynthesis	True	1.647e+05	-0.2389	-0.2389	0.0947
145	Pyro-Gln	Amino acid	2	AT_Untreated_2_5	AT_Untreated_2	1.0	Amino acid derivatives	True	1.517e+05	-0.357	-0.357	-0.7029
197	C-Glycosyl-Trp	Amino acid	2	AT_Untreated_2_5	AT_Untreated_2	1.0	Amino acid derivatives	True	4.229e+05	1.122	1.122	1.265
718	Nicotinamide Riboside	Cofactor	2	AT_Untreated_2_5	AT_Untreated_2	0.333333333333333	NAD biosynthesis	True	1.686e+05	-0.205	-0.205	1.599
295	gamma-Glu-Phe	Amino acid	2	AT_Untreated_2_5	AT_Untreated_2	1.0	Gamma-glutamyl dipeptides	True	6.565e+04	-1.565	-1.565	-1.464
399	Pseudouridine	Nucleotide	2	AT_Untreated_2_5	AT_Untreated_2	1.0	Pyrimidine derivatives in RNAs	True	6.481e+04	-1.584	-1.584	2.657
375	UTP	Nucleotide	2	AT_Untreated_2_5	AT_Untreated_2	0.166666666666667	Pyrimidine nucleotides	False			-5.966	-0.5235
20	Erythronate	Carbon	2	AT_Untreated_2_5	AT_Untreated_2	1.0	Aminosugar derivatives	True	6.022e+04	-1.69	-1.69	-0.2485
151	Phenylacetylglycine	Amino acid	2	AT_Untreated_2_5	AT_Untreated_2	1.0	Amino acid derivatives	True	7.579e+05	1.964	1.964	6.385
252	Gly-Phe	Amino acid	2	AT_Untreated_2_5	AT_Untreated_2	0.666666666666667	Dipeptides	True	1.982e+05	0.02827	0.02827	-0.07587

Metabolite ID	Name	Super Pathway	Dataset	Sample ID	Group ID	Detection Fraction	Pathway	Detected	Raw Intensity	Log2 Norm Intensity	Norm Imputed	Log2 Ctrl Norm
251	Gly-Leu	Amino acid	2	AT_Untreated_2_5	AT_Untreated_2	1.0	Dipeptides	True	1.305e+05	-0.5748	-0.5748	-0.9374
290	gamma-Glu-Ile	Amino acid	2	AT_Untreated_2_5	AT_Untreated_2	0.833333333333333	Gamma-glutamyl dipeptides	True	2.496e+04	-2.961	-2.961	-1.429
316	Ophthalmate	Amino acid	2	AT_Untreated_2_5	AT_Untreated_2	1.0	Oxidative stress markers	True	1.136e+05	-0.775	-0.775	-1.029
208	Pro-OH-Pro	Amino acid	2	AT_Untreated_2_5	AT_Untreated_2	1.0	Amino acid derivatives	True	2.334e+05	0.2642	0.2642	-0.1954
352	3'-AMP	Nucleotide	2	AT_Untreated_2_5	AT_Untreated_2	1.0	Purine derivatives in signaling	True	1.081e+05	-0.8458	-0.8458	0.4391
314	Cys-Glutathione Disulfide	Amino acid	2	AT_Untreated_2_5	AT_Untreated_2	1.0	Oxidative stress markers	True	1.763e+05	-0.1408	-0.1408	1.225
39	Threitol	Carbon	2	AT_Untreated_2_5	AT_Untreated_2	0.5	Sugars and sugar alcohols	True	1.808e+04	-3.426	-3.426	0.5991
31	Ribulose/Xylulose	Carbon	2	AT_Untreated_2_5	AT_Untreated_2	0.0	Sugars and sugar alcohols	False			-5.004	-0.09716
48	DHAP	Carbon	2	AT_Untreated_2_5	AT_Untreated_2	1.0	Glycolysis, GNG	True	5.771e+05	1.57	1.57	-0.1875
182	P-Cresol Sulfate	Amino acid	2	AT_Untreated_2_5	AT_Untreated_2	1.0	Amino acid derivatives	True	7.049e+04	-1.463	-1.463	1.874
250	Gly-Ile	Amino acid	2	AT_Untreated_2_5	AT_Untreated_2	1.0	Dipeptides	True	3.086e+04	-2.655	-2.655	-1.231
286	gamma-Glu-Glu	Amino acid	2	AT_Untreated_2_5	AT_Untreated_2	0.833333333333333	Gamma-glutamyl dipeptides	True	1.462e+05	-0.4104	-0.4104	-0.1798
264	Leu-Leu	Amino acid	2	AT_Untreated_2_5	AT_Untreated_2	1.0	Dipeptides	True	6.773e+04	-1.521	-1.521	-0.4473
203	DiMe-Arg	Amino acid	2	AT_Untreated_2_5	AT_Untreated_2	1.0	Amino acid derivatives	True	9.241e+05	2.25	2.25	1.175
47	Fructose 1,6-PP, Glucose 1,6-PP	Carbon	2	AT_Untreated_2_5	AT_Untreated_2	1.0	Glycolysis, GNG	True	1.197e+05	-0.6991	-0.6991	-0.409
224	N-Ac-Ser	Amino acid	2	AT_Untreated_2_5	AT_Untreated_2	0.833333333333333	N-acetylated amino acids	True	1.325e+05	-0.5529	-0.5529	-1.424
244	Ala-Leu	Amino acid	2	AT_Untreated_2_5	AT_Untreated_2	0.666666666666667	Dipeptides	True	2.127e+05	0.1304	0.1304	-0.2093
304	Cyclo(Phe-Pro)	Amino acid	2	AT_Untreated_2_5	AT_Untreated_2	0.0	Cyclic dipeptides	False			-1.192	-0.7201
302	Cyclo(Glu-Glu)	Amino acid	2	AT_Untreated_2_5	AT_Untreated_2	0.666666666666667	Cyclic dipeptides	True	5.616e+04	-1.791	-1.791	-0.6185
303	Cyclo(Leu-Pro)	Amino acid	2	AT_Untreated_2_5	AT_Untreated_2	0.0	Cyclic dipeptides	False			-0.4297	-0.7026
390	2',3'-cUMP	Nucleotide	2	AT_Untreated_2_5	AT_Untreated_2	1.0	Pyrimidine derivatives in signaling	True	8.115e+04	-1.26	-1.26	-0.1308
68	Ribulose 5-P / Xylulose 5-P	Carbon	2	AT_Untreated_2_5	AT_Untreated_2	1.0	Pentose phosphate pathway (PPP)	True	2.414e+05	0.313	0.313	-0.4562
388	2',3'-cCMP	Nucleotide	2	AT_Untreated_2_5	AT_Untreated_2	1.0	Pyrimidine derivatives in signaling	True	1.89e+05	-0.03968	-0.03968	-0.2931

Metabolite ID	Name	Super Pathway	Dataset	Sample ID	Group ID	Detection Fraction	Pathway	Detected	Raw Intensity	Log2 Norm Intensity	Norm Imputed	Log2 Ctrl Norm
33	Arabitol/Xylitol	Carbon	2	AT_Untreated_2_5	AT_Untreated_2	0.5	Sugars and sugar alcohols	False			-3.559	-0.05408
268	Phe-Phe	Amino acid	2	AT_Untreated_2_5	AT_Untreated_2	0.5	Dipeptides	False			-2.762	-1.674
245	Ala-Phe	Amino acid	2	AT_Untreated_2_5	AT_Untreated_2	0.666666666666667	Dipeptides	False			-2.821	-1.366
373	UMP	Nucleotide	2	AT_Untreated_2_5	AT_Untreated_2	0.5	Pyrimidine nucleotides	True	1.787e+04	-3.442	-3.442	-1.402
282	Val-Leu	Amino acid	2	AT_Untreated_2_5	AT_Untreated_2	0.666666666666667	Dipeptides	True	1.799e+05	-0.1115	-0.1115	0.2572
258	Ile-Gly	Amino acid	2	AT_Untreated_2_5	AT_Untreated_2	0.833333333333333	Dipeptides	True	1.964e+05	0.01573	0.01573	-0.3989
259	Ile-Ser	Amino acid	2	AT_Untreated_2_5	AT_Untreated_2	0.666666666666667	Dipeptides	True	8.767e+04	-1.148	-1.148	-0.2852
269	Phe-Ser	Amino acid	2	AT_Untreated_2_5	AT_Untreated_2	0.666666666666667	Dipeptides	True	6.284e+04	-1.629	-1.629	-0.3137
277	Tyr-Ala	Amino acid	2	AT_Untreated_2_5	AT_Untreated_2	1.0	Dipeptides	True	1.597e+05	-0.2829	-0.2829	-0.853
257	Ile-Gln	Amino acid	2	AT_Untreated_2_5	AT_Untreated_2	0.833333333333333	Dipeptides	True	8.47e+04	-1.198	-1.198	-0.2791
261	Leu-Glu	Amino acid	2	AT_Untreated_2_5	AT_Untreated_2	1.0	Dipeptides	True	3.268e+05	0.7502	0.7502	-0.04
263	Leu-Gly	Amino acid	2	AT_Untreated_2_5	AT_Untreated_2	1.0	Dipeptides	True	3.656e+05	0.912	0.912	0.2051
256	Ile-Ala	Amino acid	2	AT_Untreated_2_5	AT_Untreated_2	0.833333333333333	Dipeptides	True	1.321e+05	-0.5568	-0.5568	-0.5151
274	Thr-Leu	Amino acid	2	AT_Untreated_2_5	AT_Untreated_2	0.833333333333333	Dipeptides	True	3.382e+05	0.7996	0.7996	-0.05159
273	Ser-Phe	Amino acid	2	AT_Untreated_2_5	AT_Untreated_2	0.666666666666667	Dipeptides	True	5.279e+04	-1.88	-1.88	-0.6583
272	Ser-Leu	Amino acid	2	AT_Untreated_2_5	AT_Untreated_2	1.0	Dipeptides	True	1.811e+05	-0.1014	-0.1014	-0.8402
246	Asp-Leu	Amino acid	2	AT_Untreated_2_5	AT_Untreated_2	1.0	Dipeptides	True	2.241e+05	0.2056	0.2056	0.2797
76	Gln	Amino acid	2	AT_Untreated_2_6	AT_Untreated_2	1.0	Proteinogenic amino acids	True	1.249e+07	6.327	6.327	-0.6253
89	Trp	Amino acid	2	AT_Untreated_2_6	AT_Untreated_2	1.0	Proteinogenic amino acids	True	9.997e+06	6.006	6.006	0.03947
723	beta-Ala	Cofactor	2	AT_Untreated_2_6	AT_Untreated_2	1.0	Coenzyme A biosynthesis	True	1.649e+05	0.08411	0.08411	-0.2422
75	Glu	Amino acid	2	AT_Untreated_2_6	AT_Untreated_2	1.0	Proteinogenic amino acids	True	1.092e+07	6.134	6.134	0.1859
80	His	Amino acid	2	AT_Untreated_2_6	AT_Untreated_2	1.0	Proteinogenic amino acids	True	1.515e+05	-0.03771	-0.03771	-0.2518
82	Leu	Amino acid	2	AT_Untreated_2_6	AT_Untreated_2	1.0	Proteinogenic amino acids	True	4.484e+07	8.171	8.171	0.01093

Metabolite ID	Name	Super Pathway	Dataset	Sample ID	Group ID	Detection Fraction	Pathway	Detected	Raw Intensity	Log2 Norm Intensity	Norm Imputed	Log2 Ctrl Norm
87	Phe	Amino acid	2	AT_Untreated_2_6	AT_Untreated_2	1.0	Proteinogenic amino acids	True	4.137e+07	8.055	8.055	0.1632
236	Spermidine	Amino acid	2	AT_Untreated_2_6	AT_Untreated_2	1.0	Polyamines	True	7.674e+05	2.303	2.303	-0.5249
73	Asn	Amino acid	2	AT_Untreated_2_6	AT_Untreated_2	1.0	Proteinogenic amino acids	True	5.032e+05	1.694	1.694	-1.055
243	Creatinine	Amino acid	2	AT_Untreated_2_6	AT_Untreated_2	1.0	Creatine degradation	True	9.244e+05	2.571	2.571	1.001
376	Cytidine	Nucleotide	2	AT_Untreated_2_6	AT_Untreated_2	1.0	Pyrimidine nucleosides	True	5.286e+05	1.765	1.765	2.349
41	Lactate	Carbon	2	AT_Untreated_2_6	AT_Untreated_2	1.0	Respiratory carbon sources	True	6.894e+07	8.792	8.792	0.9697
93	3-P-Ser	Amino acid	2	AT_Untreated_2_6	AT_Untreated_2	0.333333333333333	Amino acids biosynthesis intermediates	False			-4.967	-1.709
343	Adenine	Nucleotide	2	AT_Untreated_2_6	AT_Untreated_2	1.0	Purine bases	True	4.408e+04	-1.819	-1.819	-0.3555
336	Adenosine	Nucleotide	2	AT_Untreated_2_6	AT_Untreated_2	1.0	Purine nucleosides	True	5.012e+05	1.688	1.688	-1.449
29	Raffinose	Carbon	2	AT_Untreated_2_6	AT_Untreated_2	0.166666666666667	Sugars and sugar alcohols	False			-5.058	-2.878
717	Nicotinamide	Cofactor	2	AT_Untreated_2_6	AT_Untreated_2	1.0	NAD biosynthesis	True	1.231e+06	2.984	2.984	0.5874
51	PEP	Carbon	2	AT_Untreated_2_6	AT_Untreated_2	1.0	Glycolysis, GNG	True	2.352e+05	0.5965	0.5965	1.668
52	Pyruvate	Carbon	2	AT_Untreated_2_6	AT_Untreated_2	1.0	Glycolysis, GNG	True	3.433e+04	-2.18	-2.18	0.1938
237	Spermine	Amino acid	2	AT_Untreated_2_6	AT_Untreated_2	0.666666666666667	Polyamines	True	4.901e+05	1.656	1.656	-1.733
385	Uracil	Nucleotide	2	AT_Untreated_2_6	AT_Untreated_2	1.0	Pyrimidine bases	True	2.74e+05	0.817	0.817	2.544
377	Uridine	Nucleotide	2	AT_Untreated_2_6	AT_Untreated_2	1.0	Pyrimidine nucleosides	True	1.095e+06	2.816	2.816	-0.06643
112	trans-Urocanate	Amino acid	2	AT_Untreated_2_6	AT_Untreated_2	1.0	Amino acids degradation intermediates	True	1.56e+05	3.88e-03	3.88e-03	3.121
737	Pyridoxine (Vitamin B6)	Cofactor	2	AT_Untreated_2_6	AT_Untreated_2	1.0	PLP biosynthesis and salvage	True	2.333e+06	3.907	3.907	0.607
348	Allantoin	Nucleotide	2	AT_Untreated_2_6	AT_Untreated_2	1.0	Purine degradation	True	5.915e+04	-1.395	-1.395	1.434
335	Inosine	Nucleotide	2	AT_Untreated_2_6	AT_Untreated_2	1.0	Purine nucleosides	True	7.537e+05	2.277	2.277	-0.5846
81	Ile	Amino acid	2	AT_Untreated_2_6	AT_Untreated_2	1.0	Proteinogenic amino acids	True	4.356e+07	8.13	8.13	0.2741
72	Ala	Amino acid	2	AT_Untreated_2_6	AT_Untreated_2	1.0	Proteinogenic amino acids	True	1.797e+07	6.852	6.852	-0.8818
79	Thr	Amino acid	2	AT_Untreated_2_6	AT_Untreated_2	1.0	Proteinogenic amino acids	True	5.772e+06	5.214	5.214	-0.7489

Metabolite ID	Name	Super Pathway	Dataset	Sample ID	Group ID	Detection Fraction	Pathway	Detected	Raw Intensity	Log2 Norm Intensity	Norm Imputed	Log2 Ctrl Norm
88	Tyr	Amino acid	2	AT_Untreated_2_6	AT_Untreated_2	1.0	Proteinogenic amino acids	True	1.909e+07	6.94	6.94	0.09709
84	Lys	Amino acid	2	AT_Untreated_2_6	AT_Untreated_2	1.0	Proteinogenic amino acids	True	1.786e+06	3.521	3.521	-0.1981
86	Met	Amino acid	2	AT_Untreated_2_6	AT_Untreated_2	1.0	Proteinogenic amino acids	True	6.971e+06	5.486	5.486	-0.3012
61	Malate	Carbon	2	AT_Untreated_2_6	AT_Untreated_2	1.0	TCA cycle	True	4.267e+05	1.456	1.456	0.5592
235	Putrescine	Amino acid	2	AT_Untreated_2_6	AT_Untreated_2	0.666666666666667	Polyamines	True	5.186e+04	-1.585	-1.585	-0.01719
49	3-P-Glycerate	Carbon	2	AT_Untreated_2_6	AT_Untreated_2	1.0	Glycolysis, GNG	True	1.882e+06	3.596	3.596	1.134
139	GABA	Amino acid	2	AT_Untreated_2_6	AT_Untreated_2	0.5	Amino acid derivatives	True	4986	-4.963	-4.963	-1.493
189	Kynurenate	Amino acid	2	AT_Untreated_2_6	AT_Untreated_2	1.0	Amino acid derivatives	True	4268	-5.188	-5.188	0
234	5-Me-Thioadenosine	Amino acid	2	AT_Untreated_2_6	AT_Untreated_2	1.0	SAM metabolism	True	6.529e+04	-1.252	-1.252	-1.156
59	Succinate	Carbon	2	AT_Untreated_2_6	AT_Untreated_2	0.833333333333333	TCA cycle	True	1.995e+04	-2.963	-2.963	-0.9227
133	Ornithine	Amino acid	2	AT_Untreated_2_6	AT_Untreated_2	1.0	Amino acids degradation intermediates	True	1.459e+06	3.23	3.23	0.7085
313	5-Oxoproline	Amino acid	2	AT_Untreated_2_6	AT_Untreated_2	1.0	Glutathione derivatives	True	1.744e+06	3.487	3.487	1.483
724	Pantothenate	Cofactor	2	AT_Untreated_2_6	AT_Untreated_2	1.0	Coenzyme A biosynthesis	True	2.643e+06	4.087	4.087	0.9732
30	Sucrose	Carbon	2	AT_Untreated_2_6	AT_Untreated_2	1.0	Sugars and sugar alcohols	True	3.098e+05	0.9939	0.9939	-1.383
122	3-OH-Isobutyrate	Amino acid	2	AT_Untreated_2_6	AT_Untreated_2	0.666666666666667	Amino acids degradation intermediates	True	2.541e+04	-2.614	-2.614	2.014
241	4-Acetamidobutanoate	Amino acid	2	AT_Untreated_2_6	AT_Untreated_2	1.0	Polyamine derivatives	True	1.486e+05	-0.06561	-0.06561	1.442
55	Citrate	Carbon	2	AT_Untreated_2_6	AT_Untreated_2	1.0	TCA cycle	True	1.436e+06	3.207	3.207	0.1722
338	Guanosine	Nucleotide	2	AT_Untreated_2_6	AT_Untreated_2	1.0	Purine nucleosides	True	7.718e+05	2.311	2.311	-0.04957
170	2-Amino-Butyrate	Amino acid	2	AT_Untreated_2_6	AT_Untreated_2	1.0	Amino acid derivatives	True	2.024e+05	0.3799	0.3799	-0.8439
209	N-Ac-Ala	Amino acid	2	AT_Untreated_2_6	AT_Untreated_2	0.666666666666667	N-acetylated amino acids	True	8553	-4.185	-4.185	-0.9173
221	N-Ac-Met	Amino acid	2	AT_Untreated_2_6	AT_Untreated_2	1.0	N-acetylated amino acids	True	2.422e+04	-2.683	-2.683	-1.533
22	N-Ac-Neuraminate	Carbon	2	AT_Untreated_2_6	AT_Untreated_2	1.0	Aminosugar derivatives	True	1.397e+05	-0.1555	-0.1555	0.05854

Metabolite ID	Name	Super Pathway	Dataset	Sample ID	Group ID	Detection Fraction	Pathway	Detected	Raw Intensity	Log2 Norm Intensity	Norm Imputed	Log2 Ctrl Norm
346	Urate	Nucleotide	2	AT_Untreated_2_6	AT_Untreated_2	1.0	Purine degradation	True	3.206e+04	-2.278	-2.278	1.414
90	Arg	Amino acid	2	AT_Untreated_2_6	AT_Untreated_2	1.0	Proteinogenic amino acids	True	2.984e+06	4.262	4.262	-0.5201
60	Fumarate	Carbon	2	AT_Untreated_2_6	AT_Untreated_2	1.0	TCA cycle	True	1.683e+05	0.1133	0.1133	0.27
78	Ser	Amino acid	2	AT_Untreated_2_6	AT_Untreated_2	1.0	Proteinogenic amino acids	True	7.203e+06	5.533	5.533	-1.293
83	Val	Amino acid	2	AT_Untreated_2_6	AT_Untreated_2	1.0	Proteinogenic amino acids	True	2.68e+07	7.428	7.428	0.1946
734	Pyridoxal	Cofactor	2	AT_Untreated_2_6	AT_Untreated_2	1.0	PLP biosynthesis and salvage	True	3.027e+05	0.9604	0.9604	1.955
136	Urea	Amino acid	2	AT_Untreated_2_6	AT_Untreated_2	1.0	Amino acids degradation intermediates	True	8.72e+05	2.487	2.487	0.8419
742	Folate	Cofactor	2	AT_Untreated_2_6	AT_Untreated_2	1.0	Folate metabolism	True	1.658e+05	0.09188	0.09188	0.6089
729	Riboflavin (Vitamin B2)	Cofactor	2	AT_Untreated_2_6	AT_Untreated_2	1.0	Flavine biosynthesis	True	7.744e+04	-1.006	-1.006	0.5629
91	Pro	Amino acid	2	AT_Untreated_2_6	AT_Untreated_2	1.0	Proteinogenic amino acids	True	1.094e+07	6.136	6.136	-0.5853
308	Glutathione, Reduced	Amino acid	2	AT_Untreated_2_6	AT_Untreated_2	1.0	Glutathione	True	7.87e+06	5.661	5.661	-0.7943
706	FAD	Cofactor	2	AT_Untreated_2_6	AT_Untreated_2	0.833333333333333	Cofactors	True	6051	-4.684	-4.684	0.4121
299	gamma-Glu-Tyr	Amino acid	2	AT_Untreated_2_6	AT_Untreated_2	1.0	Gamma-glutamyl dipeptides	True	2.297e+04	-2.759	-2.759	-1.592
705	Coenzyme A	Cofactor	2	AT_Untreated_2_6	AT_Untreated_2	1.0	Cofactors	True	5464	-4.831	-4.831	-1.125
342	Hypoxanthine	Nucleotide	2	AT_Untreated_2_6	AT_Untreated_2	1.0	Purine bases	True	2.191e+05	0.4942	0.4942	0.4923
344	Xanthine	Nucleotide	2	AT_Untreated_2_6	AT_Untreated_2	0.833333333333333	Purine bases	True	2.132e+05	0.4545	0.4545	2.694
703	NAD+	Cofactor	2	AT_Untreated_2_6	AT_Untreated_2	1.0	Cofactors	True	1.12e+06	2.848	2.848	-0.7107
731	Thiamin (Vitamin B1)	Cofactor	2	AT_Untreated_2_6	AT_Untreated_2	1.0	TPP biosynthesis	True	1.63e+05	0.06737	0.06737	0.2735
102	2-Aminoadipate	Amino acid	2	AT_Untreated_2_6	AT_Untreated_2	1.0	Amino acids biosynthesis intermediates	True	1.808e+05	0.2166	0.2166	-0.5399
77	Gly	Amino acid	2	AT_Untreated_2_6	AT_Untreated_2	1.0	Proteinogenic amino acids	True	1.506e+07	6.597	6.597	-0.6314
45	Fructose-6-P	Carbon	2	AT_Untreated_2_6	AT_Untreated_2	0.666666666666667	Glycolysis, GNG	False			-2.517	-1.082
36	Ribose	Carbon	2	AT_Untreated_2_6	AT_Untreated_2	0.833333333333333	Sugars and sugar alcohols	True	7.879e+04	-0.9814	-0.9814	0.01617
4	GlcNAc 6-P	Carbon	2	AT_Untreated_2_6	AT_Untreated_2	1.0	Aminosugar biosynthesis	True	1.6e+05	0.04061	0.04061	-0.3191

Metabolite ID	Name	Super Pathway	Dataset	Sample ID	Group ID	Detection Fraction	Pathway	Detected	Raw Intensity	Log2 Norm Intensity	Norm Imputed	Log2 Ctrl Norm
188	Kynurenine	Amino acid	2	AT_Untreated_2_6	AT_Untreated_2	1.0	Amino acid derivatives	True	2.276e+05	0.5488	0.5488	0.5627
63	6-P-Gluconate	Carbon	2	AT_Untreated_2_6	AT_Untreated_2	1.0	Pentose phosphate pathway (PPP)	True	8.913e+04	-0.8034	-0.8034	0.2418
710	Carnitine	Cofactor	2	AT_Untreated_2_6	AT_Untreated_2	1.0	Cofactors	True	4.038e+05	1.376	1.376	-1.156
725	P-Pantetheine	Cofactor	2	AT_Untreated_2_6	AT_Untreated_2	1.0	Coenzyme A biosynthesis	True	8046	-4.273	-4.273	0.5395
110	N-alpha-Ac-Ornithine	Amino acid	2	AT_Untreated_2_6	AT_Untreated_2	1.0	Amino acids biosynthesis intermediates	True	2.747e+05	0.8203	0.8203	-0.1537
116	3-Me-2-Oxo-Valerate	Amino acid	2	AT_Untreated_2_6	AT_Untreated_2	1.0	Amino acids degradation intermediates	True	2.644e+04	-2.556	-2.556	1.018
155	4-Guanidinobutanoate	Amino acid	2	AT_Untreated_2_6	AT_Untreated_2	1.0	Amino acid derivatives	True	4.388e+04	-1.826	-1.826	-1.106
310	S-Lactoyl-Glutathione	Amino acid	2	AT_Untreated_2_6	AT_Untreated_2	0.666666666666667	Glutathione derivatives	True	1.274e+04	-3.609	-3.609	0.06239
34	Ribitol	Carbon	2	AT_Untreated_2_6	AT_Untreated_2	1.0	Sugars and sugar alcohols	True	1.061e+05	-0.5524	-0.5524	1.026
707	FMN	Cofactor	2	AT_Untreated_2_6	AT_Untreated_2	0.666666666666667	Cofactors	True	2.116e+04	-2.878	-2.878	0.543
17	Maltose	Carbon	2	AT_Untreated_2_6	AT_Untreated_2	1.0	Glycogen degradation	True	3.562e+05	1.195	1.195	1.235
18	Maltotriose	Carbon	2	AT_Untreated_2_6	AT_Untreated_2	1.0	Glycogen degradation	True	2.68e+06	4.107	4.107	2.678
19	Maltotetraose	Carbon	2	AT_Untreated_2_6	AT_Untreated_2	1.0	Glycogen degradation	True	2.723e+05	0.8078	0.8078	0.9982
232	SAH	Amino acid	2	AT_Untreated_2_6	AT_Untreated_2	1.0	SAM metabolism	True	1.483e+04	-3.391	-3.391	-0.1646
74	Asp	Amino acid	2	AT_Untreated_2_6	AT_Untreated_2	1.0	Proteinogenic amino acids	True	2.554e+06	4.037	4.037	-1.622
129	5-Aminovalerate	Amino acid	2	AT_Untreated_2_6	AT_Untreated_2	0.833333333333333	Amino acids degradation intermediates	True	8.797e+04	-0.8223	-0.8223	-9.29e-03
254	Gly-Val	Amino acid	2	AT_Untreated_2_6	AT_Untreated_2	1.0	Dipeptides	True	1.042e+05	-0.578	-0.578	-0.5043
291	gamma-Glu-Leu	Amino acid	2	AT_Untreated_2_6	AT_Untreated_2	1.0	Gamma-glutamyl dipeptides	True	7.269e+04	-1.098	-1.098	-0.01565
173	Met Sulfoxide	Amino acid	2	AT_Untreated_2_6	AT_Untreated_2	1.0	Amino acid derivatives	True	2.549e+05	0.7123	0.7123	0.8511
43	Glucose	Carbon	2	AT_Untreated_2_6	AT_Untreated_2	1.0	Glycolysis, GNG	True	2.343e+07	7.235	7.235	0.4154
249	Gly-Gly	Amino acid	2	AT_Untreated_2_6	AT_Untreated_2	1.0	Dipeptides	True	7.149e+04	-1.122	-1.122	-1.339

Metabolite ID	Name	Super Pathway	Dataset	Sample ID	Group ID	Detection Fraction	Pathway	Detected	Raw Intensity	Log2 Norm Intensity	Norm Imputed	Log2 Ctrl Norm
169	2-OH-Butyrate	Amino acid	2	AT_Untreated_2_6	AT_Untreated_2	0.833333333333333	Amino acid derivatives	True	1.252e+05	-0.3135	-0.3135	1.12
98	3-Methyl-2-Oxobutyrate	Amino acid	2	AT_Untreated_2_6	AT_Untreated_2	0.833333333333333	Amino acids biosynthesis intermediates	False			-3.759	0
100	4-Me-2-Oxo-Pentanoate	Amino acid	2	AT_Untreated_2_6	AT_Untreated_2	1.0	Amino acids biosynthesis intermediates	True	3.016e+04	-2.367	-2.367	0.935
253	Gly-Pro	Amino acid	2	AT_Untreated_2_6	AT_Untreated_2	1.0	Dipeptides	True	9.702e+04	-0.681	-0.681	-0.36
247	Asp-Phe	Amino acid	2	AT_Untreated_2_6	AT_Untreated_2	1.0	Dipeptides	True	3.6e+04	-2.111	-2.111	-1.17
212	N-Ac-Asp	Amino acid	2	AT_Untreated_2_6	AT_Untreated_2	0.333333333333333	N-acetylated amino acids	True	1.899e+04	-3.034	-3.034	1.468
720	1-Me-Nicotinamide	Cofactor	2	AT_Untreated_2_6	AT_Untreated_2	1.0	Derivatives of NA, nicotinamide and NAD	True	1.949e+06	3.647	3.647	-0.05626
70	Creatine	Carbon	2	AT_Untreated_2_6	AT_Untreated_2	1.0	Creatine energy storage	True	8.307e+06	5.739	5.739	-0.04161
309	Glutathione, Oxidized	Amino acid	2	AT_Untreated_2_6	AT_Untreated_2	1.0	Glutathione	True	2.607e+06	4.067	4.067	0.1185
44	Glucose 6-P	Carbon	2	AT_Untreated_2_6	AT_Untreated_2	1.0	Glycolysis, GNG	True	1.551e+05	-3.88e-03	-3.88e-03	-0.8186
24	Fructose	Carbon	2	AT_Untreated_2_6	AT_Untreated_2	1.0	Sugars and sugar alcohols	True	4.427e+06	4.831	4.831	1.47
85	Cys	Amino acid	2	AT_Untreated_2_6	AT_Untreated_2	1.0	Proteinogenic amino acids	True	2.654e+05	0.7709	0.7709	1.363
704	NADH	Cofactor	2	AT_Untreated_2_6	AT_Untreated_2	0.666666666666667	Cofactors	True	1.203e+04	-3.692	-3.692	-1.763
275	Thr-Phe	Amino acid	2	AT_Untreated_2_6	AT_Untreated_2	0.833333333333333	Dipeptides	True	4.831e+04	-1.687	-1.687	-0.7534
738	Pyridoxate	Cofactor	2	AT_Untreated_2_6	AT_Untreated_2	1.0	PLP biosynthesis and salvage	True	1.711e+04	-3.184	-3.184	1.481
177	3-(4-OH-Phenyl)Lactate	Amino acid	2	AT_Untreated_2_6	AT_Untreated_2	1.0	Amino acid derivatives	True	2.619e+04	-2.571	-2.571	1.383
206	Trans-4-OH-Pro	Amino acid	2	AT_Untreated_2_6	AT_Untreated_2	1.0	Amino acid derivatives	True	3.494e+05	1.167	1.167	1.363
329	AMP	Nucleotide	2	AT_Untreated_2_6	AT_Untreated_2	0.833333333333333	Purine nucleotides	True	2.326e+05	0.5805	0.5805	0.1599
345	Guanine	Nucleotide	2	AT_Untreated_2_6	AT_Untreated_2	1.0	Purine bases	True	4.894e+05	1.654	1.654	0.0716
271	pyroGlu-Val	Amino acid	2	AT_Untreated_2_6	AT_Untreated_2	1.0	Dipeptides	True	7718	-4.333	-4.333	-0.6916
279	Val-Glu	Amino acid	2	AT_Untreated_2_6	AT_Untreated_2	1.0	Dipeptides	True	3.796e+04	-2.035	-2.035	-1.205
183	Phenol Sulfate	Amino acid	2	AT_Untreated_2_6	AT_Untreated_2	1.0	Amino acid derivatives	True	4.034e+04	-1.947	-1.947	1.78

Metabolite ID	Name	Super Pathway	Dataset	Sample ID	Group ID	Detection Fraction	Pathway	Detected	Raw Intensity	Log2 Norm Intensity	Norm Imputed	Log2 Ctrl Norm
740	3-Dehydrocarnitine	Cofactor	2	AT_Untreated_2_6	AT_Untreated_2	1.0	Carnitine biosynthesis	True	1.134e+05	-0.4555	-0.4555	-0.1219
145	Pyro-Gln	Amino acid	2	AT_Untreated_2_6	AT_Untreated_2	1.0	Amino acid derivatives	True	1.515e+05	-0.03803	-0.03803	-0.384
197	C-Glycosyl-Trp	Amino acid	2	AT_Untreated_2_6	AT_Untreated_2	1.0	Amino acid derivatives	True	2.529e+05	0.7012	0.7012	0.8448
718	Nicotinamide Riboside	Cofactor	2	AT_Untreated_2_6	AT_Untreated_2	0.333333333333333	NAD biosynthesis	False			-2.306	-0.5023
295	gamma-Glu-Phe	Amino acid	2	AT_Untreated_2_6	AT_Untreated_2	1.0	Gamma-glutamyl dipeptides	True	5.598e+04	-1.474	-1.474	-1.373
399	Pseudouridine	Nucleotide	2	AT_Untreated_2_6	AT_Untreated_2	1.0	Pyrimidine derivatives in RNAs	True	2.3e+04	-2.758	-2.758	1.483
375	UTP	Nucleotide	2	AT_Untreated_2_6	AT_Untreated_2	0.166666666666667	Pyrimidine nucleotides	False			-5.966	-0.5235
20	Erythronate	Carbon	2	AT_Untreated_2_6	AT_Untreated_2	1.0	Aminosugar derivatives	True	8.245e+04	-0.9159	-0.9159	0.5257
151	Phenylacetylglycine	Amino acid	2	AT_Untreated_2_6	AT_Untreated_2	1.0	Amino acid derivatives	True	4.647e+04	-1.743	-1.743	2.679
252	Gly-Phe	Amino acid	2	AT_Untreated_2_6	AT_Untreated_2	0.666666666666667	Dipeptides	False			-0.4688	-0.573
251	Gly-Leu	Amino acid	2	AT_Untreated_2_6	AT_Untreated_2	1.0	Dipeptides	True	1.42e+05	-0.1314	-0.1314	-0.494
290	gamma-Glu-Ile	Amino acid	2	AT_Untreated_2_6	AT_Untreated_2	0.833333333333333	Gamma-glutamyl dipeptides	True	3.863e+04	-2.01	-2.01	-0.4785
316	Ophthalmate	Amino acid	2	AT_Untreated_2_6	AT_Untreated_2	1.0	Oxidative stress markers	True	9.267e+04	-0.7472	-0.7472	-1.001
208	Pro-OH-Pro	Amino acid	2	AT_Untreated_2_6	AT_Untreated_2	1.0	Amino acid derivatives	True	2.927e+05	0.9118	0.9118	0.4522
352	3'-AMP	Nucleotide	2	AT_Untreated_2_6	AT_Untreated_2	1.0	Purine derivatives in signaling	True	6.266e+04	-1.312	-1.312	-0.02686
314	Cys-Glutathione Disulfide	Amino acid	2	AT_Untreated_2_6	AT_Untreated_2	1.0	Oxidative stress markers	True	1.226e+05	-0.3438	-0.3438	1.022
39	Threitol	Carbon	2	AT_Untreated_2_6	AT_Untreated_2	0.5	Sugars and sugar alcohols	True	2.089e+04	-2.897	-2.897	1.128
31	Ribulose/Xylulose	Carbon	2	AT_Untreated_2_6	AT_Untreated_2	0.0	Sugars and sugar alcohols	False			-5.004	-0.09716
48	DHAP	Carbon	2	AT_Untreated_2_6	AT_Untreated_2	1.0	Glycolysis, GNG	True	4.617e+05	1.57	1.57	-0.1884
182	P-Cresol Sulfate	Amino acid	2	AT_Untreated_2_6	AT_Untreated_2	1.0	Amino acid derivatives	True	1.817e+04	-3.098	-3.098	0.2398
250	Gly-Ile	Amino acid	2	AT_Untreated_2_6	AT_Untreated_2	1.0	Dipeptides	True	4.915e+04	-1.662	-1.662	-0.238
286	gamma-Glu-Glu	Amino acid	2	AT_Untreated_2_6	AT_Untreated_2	0.833333333333333	Gamma-glutamyl dipeptides	True	7.022e+04	-1.147	-1.147	-0.9168

Metabolite ID	Name	Super Pathway	Dataset	Sample ID	Group ID	Detection Fraction	Pathway	Detected	Raw Intensity	Log2 Norm Intensity	Norm Imputed	Log2 Ctrl Norm
264	Leu-Leu	Amino acid	2	AT_Untreated_2_6	AT_Untreated_2	1.0	Dipeptides	True	3.335e+04	-2.222	-2.222	-1.148
203	DiMe-Arg	Amino acid	2	AT_Untreated_2_6	AT_Untreated_2	1.0	Amino acid derivatives	True	2.567e+05	0.7229	0.7229	-0.3514
47	Fructose 1,6-PP, Glucose 1,6-PP	Carbon	2	AT_Untreated_2_6	AT_Untreated_2	1.0	Glycolysis, GNG	True	6.25e+04	-1.316	-1.316	-1.025
224	N-Ac-Ser	Amino acid	2	AT_Untreated_2_6	AT_Untreated_2	0.833333333333333	N-acetylated amino acids	True	9.168e+04	-0.7628	-0.7628	-1.634
244	Ala-Leu	Amino acid	2	AT_Untreated_2_6	AT_Untreated_2	0.666666666666667	Dipeptides	True	1.107e+05	-0.4913	-0.4913	-0.8311
304	Cyclo(Phe-Pro)	Amino acid	2	AT_Untreated_2_6	AT_Untreated_2	0.0	Cyclic dipeptides	False			-1.192	-0.7201
302	Cyclo(Glu-Glu)	Amino acid	2	AT_Untreated_2_6	AT_Untreated_2	0.666666666666667	Cyclic dipeptides	True	7.903e+04	-0.977	-0.977	0.1952
303	Cyclo(Leu-Pro)	Amino acid	2	AT_Untreated_2_6	AT_Untreated_2	0.0	Cyclic dipeptides	False			-0.4297	-0.7026
390	2',3'-cUMP	Nucleotide	2	AT_Untreated_2_6	AT_Untreated_2	1.0	Pyrimidine derivatives in signaling	True	2.542e+04	-2.613	-2.613	-1.484
68	Ribulose 5-P / Xylulose 5-P	Carbon	2	AT_Untreated_2_6	AT_Untreated_2	1.0	Pentose phosphate pathway (PPP)	True	2.41e+05	0.6319	0.6319	-0.1373
388	2',3'-cCMP	Nucleotide	2	AT_Untreated_2_6	AT_Untreated_2	1.0	Pyrimidine derivatives in signaling	True	8.065e+04	-0.9477	-0.9477	-1.201
33	Arabitol/Xylitol	Carbon	2	AT_Untreated_2_6	AT_Untreated_2	0.5	Sugars and sugar alcohols	True	1.467e+04	-3.406	-3.406	0.09923
268	Phe-Phe	Amino acid	2	AT_Untreated_2_6	AT_Untreated_2	0.5	Dipeptides	False			-2.762	-1.674
245	Ala-Phe	Amino acid	2	AT_Untreated_2_6	AT_Untreated_2	0.666666666666667	Dipeptides	True	2.201e+04	-2.821	-2.821	-1.366
373	UMP	Nucleotide	2	AT_Untreated_2_6	AT_Untreated_2	0.5	Pyrimidine nucleotides	True	2.075e+04	-2.907	-2.907	-0.8662
282	Val-Leu	Amino acid	2	AT_Untreated_2_6	AT_Untreated_2	0.666666666666667	Dipeptides	True	1.149e+05	-0.4367	-0.4367	-0.06797
258	Ile-Gly	Amino acid	2	AT_Untreated_2_6	AT_Untreated_2	0.833333333333333	Dipeptides	True	1.091e+05	-0.5112	-0.5112	-0.9257
259	Ile-Ser	Amino acid	2	AT_Untreated_2_6	AT_Untreated_2	0.666666666666667	Dipeptides	False			-3.192	-2.329
269	Phe-Ser	Amino acid	2	AT_Untreated_2_6	AT_Untreated_2	0.666666666666667	Dipeptides	False			-3.197	-1.882
277	Tyr-Ala	Amino acid	2	AT_Untreated_2_6	AT_Untreated_2	1.0	Dipeptides	True	7.593e+04	-1.035	-1.035	-1.605
257	Ile-Gln	Amino acid	2	AT_Untreated_2_6	AT_Untreated_2	0.833333333333333	Dipeptides	True	4.812e+04	-1.693	-1.693	-0.7737
261	Leu-Glu	Amino acid	2	AT_Untreated_2_6	AT_Untreated_2	1.0	Dipeptides	True	9.028e+04	-0.785	-0.785	-1.575
263	Leu-Gly	Amino acid	2	AT_Untreated_2_6	AT_Untreated_2	1.0	Dipeptides	True	1.576e+05	0.01844	0.01844	-0.6885

Metabolite ID	Name	Super Pathway	Dataset	Sample ID	Group ID	Detection Fraction	Pathway	Detected	Raw Intensity	Log2 Norm Intensity	Norm Imputed	Log2 Ctrl Norm
256	Ile-Ala	Amino acid	2	AT_Untreated_2_6	AT_Untreated_2	0.833333333333333	Dipeptides	False			-2.214	-2.173
274	Thr-Leu	Amino acid	2	AT_Untreated_2_6	AT_Untreated_2	0.833333333333333	Dipeptides	True	2.184e+05	0.4893	0.4893	-0.3618
273	Ser-Phe	Amino acid	2	AT_Untreated_2_6	AT_Untreated_2	0.666666666666667	Dipeptides	True	4.893e+04	-1.669	-1.669	-0.4468
272	Ser-Leu	Amino acid	2	AT_Untreated_2_6	AT_Untreated_2	1.0	Dipeptides	True	1.358e+05	-0.1959	-0.1959	-0.9347
246	Asp-Leu	Amino acid	2	AT_Untreated_2_6	AT_Untreated_2	1.0	Dipeptides	True	5.497e+04	-1.501	-1.501	-1.427
76	Gln	Amino acid	2	Ctrl_H2O2_2_1	Ctrl_H2O2_2	1.0	Proteinogenic amino acids	True	1.021e+07	6.85	6.85	-0.1024
89	Trp	Amino acid	2	Ctrl_H2O2_2_1	Ctrl_H2O2_2	1.0	Proteinogenic amino acids	True	3.866e+06	5.448	5.448	-0.5182
723	beta-Ala	Cofactor	2	Ctrl_H2O2_2_1	Ctrl_H2O2_2	0.75	Coenzyme A biosynthesis	True	1.246e+04	-2.829	-2.829	-3.156
75	Glu	Amino acid	2	Ctrl_H2O2_2_1	Ctrl_H2O2_2	1.0	Proteinogenic amino acids	True	3.471e+06	5.293	5.293	-0.6549
80	His	Amino acid	2	Ctrl_H2O2_2_1	Ctrl_H2O2_2	1.0	Proteinogenic amino acids	True	8.538e+04	-0.05228	-0.05228	-0.2664
82	Leu	Amino acid	2	Ctrl_H2O2_2_1	Ctrl_H2O2_2	1.0	Proteinogenic amino acids	True	2.102e+07	7.891	7.891	-0.2688
87	Phe	Amino acid	2	Ctrl_H2O2_2_1	Ctrl_H2O2_2	1.0	Proteinogenic amino acids	True	1.818e+07	7.682	7.682	-0.2099
236	Spermidine	Amino acid	2	Ctrl_H2O2_2_1	Ctrl_H2O2_2	1.0	Polyamines	True	1.837e+05	1.053	1.053	-1.775
73	Asn	Amino acid	2	Ctrl_H2O2_2_1	Ctrl_H2O2_2	1.0	Proteinogenic amino acids	True	8.848e+04	-8.25e-04	-8.25e-04	-2.75
243	Creatinine	Amino acid	2	Ctrl_H2O2_2_1	Ctrl_H2O2_2	0.75	Creatine degradation	True	5.309e+05	2.584	2.584	1.014
376	Cytidine	Nucleotide	2	Ctrl_H2O2_2_1	Ctrl_H2O2_2	0.75	Pyrimidine nucleosides	True	1.586e+05	0.8409	0.8409	1.426
41	Lactate	Carbon	2	Ctrl_H2O2_2_1	Ctrl_H2O2_2	1.0	Respiratory carbon sources	True	2.881e+07	8.346	8.346	0.5241
93	3-P-Ser	Amino acid	2	Ctrl_H2O2_2_1	Ctrl_H2O2_2	0.5	Amino acids biosynthesis intermediates	False			-4.967	-1.709
343	Adenine	Nucleotide	2	Ctrl_H2O2_2_1	Ctrl_H2O2_2	0.5	Purine bases	False			-3.45	-1.986
336	Adenosine	Nucleotide	2	Ctrl_H2O2_2_1	Ctrl_H2O2_2	1.0	Purine nucleosides	True	3.85e+05	2.121	2.121	-1.017
29	Raffinose	Carbon	2	Ctrl_H2O2_2_1	Ctrl_H2O2_2	0.5	Sugars and sugar alcohols	False			-5.058	-2.878
717	Nicotinamide	Cofactor	2	Ctrl_H2O2_2_1	Ctrl_H2O2_2	1.0	NAD biosynthesis	True	7.617e+05	3.105	3.105	0.7083
51	PEP	Carbon	2	Ctrl_H2O2_2_1	Ctrl_H2O2_2	1.0	Glycolysis, GNG	True	3.52e+04	-1.331	-1.331	-0.2588

Metabolite ID	Name	Super Pathway	Dataset	Sample ID	Group ID	Detection Fraction	Pathway	Detected	Raw Intensity	Log2 Norm Intensity	Norm Imputed	Log2 Ctrl Norm
52	Pyruvate	Carbon	2	Ctrl_H2O2_2_1	Ctrl_H2O2_2	0.25	Glycolysis, GNG	False			-3.589	-1.215
237	Spermine	Amino acid	2	Ctrl_H2O2_2_1	Ctrl_H2O2_2	0.75	Polyamines	False			0.9044	-2.484
385	Uracil	Nucleotide	2	Ctrl_H2O2_2_1	Ctrl_H2O2_2	1.0	Pyrimidine bases	True	4.386e+04	-1.013	-1.013	0.7136
377	Uridine	Nucleotide	2	Ctrl_H2O2_2_1	Ctrl_H2O2_2	1.0	Pyrimidine nucleosides	True	3.363e+05	1.925	1.925	-0.9569
112	trans-Urocanate	Amino acid	2	Ctrl_H2O2_2_1	Ctrl_H2O2_2	0.75	Amino acids degradation intermediates	True	1.169e+05	0.401	0.401	3.519
737	Pyridoxine (Vitamin B6)	Cofactor	2	Ctrl_H2O2_2_1	Ctrl_H2O2_2	1.0	PLP biosynthesis and salvage	True	1.808e+06	4.352	4.352	1.052
348	Allantoin	Nucleotide	2	Ctrl_H2O2_2_1	Ctrl_H2O2_2	1.0	Purine degradation	True	4.363e+04	-1.021	-1.021	1.808
335	Inosine	Nucleotide	2	Ctrl_H2O2_2_1	Ctrl_H2O2_2	1.0	Purine nucleosides	True	1.093e+05	0.3045	0.3045	-2.557
81	Ile	Amino acid	2	Ctrl_H2O2_2_1	Ctrl_H2O2_2	1.0	Proteinogenic amino acids	True	2.112e+07	7.898	7.898	0.04284
72	Ala	Amino acid	2	Ctrl_H2O2_2_1	Ctrl_H2O2_2	1.0	Proteinogenic amino acids	True	8.401e+06	6.568	6.568	-1.166
79	Thr	Amino acid	2	Ctrl_H2O2_2_1	Ctrl_H2O2_2	1.0	Proteinogenic amino acids	True	1.787e+06	4.336	4.336	-1.627
88	Tyr	Amino acid	2	Ctrl_H2O2_2_1	Ctrl_H2O2_2	1.0	Proteinogenic amino acids	True	7.773e+06	6.456	6.456	-0.3864
84	Lys	Amino acid	2	Ctrl_H2O2_2_1	Ctrl_H2O2_2	1.0	Proteinogenic amino acids	True	1.21e+06	3.772	3.772	0.0531
86	Met	Amino acid	2	Ctrl_H2O2_2_1	Ctrl_H2O2_2	1.0	Proteinogenic amino acids	True	3.658e+06	5.369	5.369	-0.4184
61	Malate	Carbon	2	Ctrl_H2O2_2_1	Ctrl_H2O2_2	1.0	TCA cycle	True	1.294e+05	0.5479	0.5479	-0.3488
235	Putrescine	Amino acid	2	Ctrl_H2O2_2_1	Ctrl_H2O2_2	0.5	Polyamines	False			-3.898	-2.331
49	3-P-Glycerate	Carbon	2	Ctrl_H2O2_2_1	Ctrl_H2O2_2	1.0	Glycolysis, GNG	True	4.444e+05	2.328	2.328	-0.135
139	GABA	Amino acid	2	Ctrl_H2O2_2_1	Ctrl_H2O2_2	0.25	Amino acid derivatives	False			-4.963	-1.493
189	Kynurenate	Amino acid	2	Ctrl_H2O2_2_1	Ctrl_H2O2_2	0.5	Amino acid derivatives	False			-5.188	0
234	5-Me-Thioadenosine	Amino acid	2	Ctrl_H2O2_2_1	Ctrl_H2O2_2	1.0	SAM metabolism	True	4.628e+04	-0.9359	-0.9359	-0.8399
59	Succinate	Carbon	2	Ctrl_H2O2_2_1	Ctrl_H2O2_2	0.75	TCA cycle	True	2.889e+04	-1.616	-1.616	0.4244
133	Ornithine	Amino acid	2	Ctrl_H2O2_2_1	Ctrl_H2O2_2	1.0	Amino acids degradation intermediates	True	4.626e+05	2.386	2.386	-0.1356
313	5-Oxoproline	Amino acid	2	Ctrl_H2O2_2_1	Ctrl_H2O2_2	1.0	Glutathione derivatives	True	1.66e+06	4.229	4.229	2.225

Metabolite ID	Name	Super Pathway	Dataset	Sample ID	Group ID	Detection Fraction	Pathway	Detected	Raw Intensity	Log2 Norm Intensity	Norm Imputed	Log2 Ctrl Norm
724	Pantothenate	Cofactor	2	Ctrl_H2O2_2_1	Ctrl_H2O2_2	1.0	Coenzyme A biosynthesis	True	1.082e+06	3.612	3.612	0.4981
30	Sucrose	Carbon	2	Ctrl_H2O2_2_1	Ctrl_H2O2_2	1.0	Sugars and sugar alcohols	True	3.006e+05	1.764	1.764	-0.613
122	3-OH-Isobutyrate	Amino acid	2	Ctrl_H2O2_2_1	Ctrl_H2O2_2	0.0	Amino acids degradation intermediates	False			-4.628	0
241	4-Acetamidobutanoate	Amino acid	2	Ctrl_H2O2_2_1	Ctrl_H2O2_2	1.0	Polyamine derivatives	True	9.157e+04	0.04867	0.04867	1.556
55	Citrate	Carbon	2	Ctrl_H2O2_2_1	Ctrl_H2O2_2	1.0	TCA cycle	True	7.787e+05	3.137	3.137	0.1024
338	Guanosine	Nucleotide	2	Ctrl_H2O2_2_1	Ctrl_H2O2_2	1.0	Purine nucleosides	True	1.593e+05	0.8474	0.8474	-1.513
170	2-Amino-Butyrate	Amino acid	2	Ctrl_H2O2_2_1	Ctrl_H2O2_2	1.0	Amino acid derivatives	True	8.858e+04	8.25e-04	8.25e-04	-1.223
209	N-Ac-Ala	Amino acid	2	Ctrl_H2O2_2_1	Ctrl_H2O2_2	0.0	N-acetylated amino acids	False			-4.401	-1.133
221	N-Ac-Met	Amino acid	2	Ctrl_H2O2_2_1	Ctrl_H2O2_2	1.0	N-acetylated amino acids	True	1.779e+04	-2.315	-2.315	-1.165
22	N-Ac-Neuraminate	Carbon	2	Ctrl_H2O2_2_1	Ctrl_H2O2_2	0.5	Aminosugar derivatives	True	4.449e+04	-0.9927	-0.9927	-0.7787
346	Urate	Nucleotide	2	Ctrl_H2O2_2_1	Ctrl_H2O2_2	0.5	Purine degradation	True	1.532e+04	-2.531	-2.531	1.161
90	Arg	Amino acid	2	Ctrl_H2O2_2_1	Ctrl_H2O2_2	1.0	Proteinogenic amino acids	True	2.104e+06	4.571	4.571	-0.2112
60	Fumarate	Carbon	2	Ctrl_H2O2_2_1	Ctrl_H2O2_2	1.0	TCA cycle	True	6.134e+04	-0.5292	-0.5292	-0.3725
78	Ser	Amino acid	2	Ctrl_H2O2_2_1	Ctrl_H2O2_2	1.0	Proteinogenic amino acids	True	2.833e+06	5	5	-1.827
83	Val	Amino acid	2	Ctrl_H2O2_2_1	Ctrl_H2O2_2	1.0	Proteinogenic amino acids	True	1.338e+07	7.24	7.24	5.72e-03
734	Pyridoxal	Cofactor	2	Ctrl_H2O2_2_1	Ctrl_H2O2_2	0.5	PLP biosynthesis and salvage	False			-1.437	-0.4421
136	Urea	Amino acid	2	Ctrl_H2O2_2_1	Ctrl_H2O2_2	1.0	Amino acids degradation intermediates	True	5.939e+05	2.746	2.746	1.101
742	Folate	Cofactor	2	Ctrl_H2O2_2_1	Ctrl_H2O2_2	1.0	Folate metabolism	True	1.055e+05	0.2535	0.2535	0.7705
729	Riboflavin (Vitamin B2)	Cofactor	2	Ctrl_H2O2_2_1	Ctrl_H2O2_2	1.0	Flavine biosynthesis	True	4.081e+04	-1.117	-1.117	0.452
91	Pro	Amino acid	2	Ctrl_H2O2_2_1	Ctrl_H2O2_2	1.0	Proteinogenic amino acids	True	3.215e+06	5.182	5.182	-1.539
308	Glutathione, Reduced	Amino acid	2	Ctrl_H2O2_2_1	Ctrl_H2O2_2	1.0	Glutathione	True	1.377e+06	3.959	3.959	-2.496
706	FAD	Cofactor	2	Ctrl_H2O2_2_1	Ctrl_H2O2_2	0.0	Cofactors	False			-5.096	0
299	gamma-Glu-Tyr	Amino acid	2	Ctrl_H2O2_2_1	Ctrl_H2O2_2	0.5	Gamma-glutamyl dipeptides	False			-3.049	-1.881

Metabolite ID	Name	Super Pathway	Dataset	Sample ID	Group ID	Detection Fraction	Pathway	Detected	Raw Intensity	Log2 Norm Intensity	Norm Imputed	Log2 Ctrl Norm
705	Coenzyme A	Cofactor	2	Ctrl_H2O2_2_1	Ctrl_H2O2_2	0.75	Cofactors	True	818	-6.758	-6.758	-3.051
342	Hypoxanthine	Nucleotide	2	Ctrl_H2O2_2_1	Ctrl_H2O2_2	1.0	Purine bases	True	6.883e+04	-0.363	-0.363	-0.3649
344	Xanthine	Nucleotide	2	Ctrl_H2O2_2_1	Ctrl_H2O2_2	0.75	Purine bases	True	1.938e+04	-2.191	-2.191	0.04764
703	NAD+	Cofactor	2	Ctrl_H2O2_2_1	Ctrl_H2O2_2	1.0	Cofactors	True	1.11e+05	0.3265	0.3265	-3.232
731	Thiamin (Vitamin B1)	Cofactor	2	Ctrl_H2O2_2_1	Ctrl_H2O2_2	1.0	TPP biosynthesis	True	9.534e+04	0.1069	0.1069	0.3131
102	2-Aminoadipate	Amino acid	2	Ctrl_H2O2_2_1	Ctrl_H2O2_2	1.0	Amino acids biosynthesis intermediates	True	1.054e+05	0.2514	0.2514	-0.5052
77	Gly	Amino acid	2	Ctrl_H2O2_2_1	Ctrl_H2O2_2	1.0	Proteinogenic amino acids	True	3.522e+06	5.314	5.314	-1.914
45	Fructose-6-P	Carbon	2	Ctrl_H2O2_2_1	Ctrl_H2O2_2	0.75	Glycolysis, GNG	True	4.492e+04	-0.9787	-0.9787	0.4565
36	Ribose	Carbon	2	Ctrl_H2O2_2_1	Ctrl_H2O2_2	0.75	Sugars and sugar alcohols	True	8704	-3.346	-3.346	-2.349
4	GlcNAc 6-P	Carbon	2	Ctrl_H2O2_2_1	Ctrl_H2O2_2	1.0	Aminosugar biosynthesis	True	2.978e+04	-1.572	-1.572	-1.931
188	Kynurenine	Amino acid	2	Ctrl_H2O2_2_1	Ctrl_H2O2_2	0.75	Amino acid derivatives	True	5.876e+04	-0.5913	-0.5913	-0.5773
63	6-P-Gluconate	Carbon	2	Ctrl_H2O2_2_1	Ctrl_H2O2_2	1.0	Pentose phosphate pathway (PPP)	True	3.517e+04	-1.332	-1.332	-0.2867
710	Carnitine	Cofactor	2	Ctrl_H2O2_2_1	Ctrl_H2O2_2	1.0	Cofactors	True	1.82e+05	1.04	1.04	-1.492
725	P-Pantetheine	Cofactor	2	Ctrl_H2O2_2_1	Ctrl_H2O2_2	0.75	Coenzyme A biosynthesis	True	5667	-3.966	-3.966	0.8469
110	N-alpha-Ac-Ornithine	Amino acid	2	Ctrl_H2O2_2_1	Ctrl_H2O2_2	1.0	Amino acids biosynthesis intermediates	True	1.812e+05	1.033	1.033	0.05906
116	3-Me-2-Oxo-Valerate	Amino acid	2	Ctrl_H2O2_2_1	Ctrl_H2O2_2	1.0	Amino acids degradation intermediates	True	4.777e+04	-0.8902	-0.8902	2.684
155	4-Guanidinobutanoate	Amino acid	2	Ctrl_H2O2_2_1	Ctrl_H2O2_2	0.5	Amino acid derivatives	False			-2.324	-1.604
310	S-Lactoyl-Glutathione	Amino acid	2	Ctrl_H2O2_2_1	Ctrl_H2O2_2	0.0	Glutathione derivatives	False			-4.634	-0.962
34	Ribitol	Carbon	2	Ctrl_H2O2_2_1	Ctrl_H2O2_2	0.75	Sugars and sugar alcohols	True	3.386e+04	-1.386	-1.386	0.1924
707	FMN	Cofactor	2	Ctrl_H2O2_2_1	Ctrl_H2O2_2	0.5	Cofactors	True	4791	-4.208	-4.208	-0.7868
17	Maltose	Carbon	2	Ctrl_H2O2_2_1	Ctrl_H2O2_2	1.0	Glycogen degradation	True	2.526e+05	1.513	1.513	1.552
18	Maltotriose	Carbon	2	Ctrl_H2O2_2_1	Ctrl_H2O2_2	1.0	Glycogen degradation	True	1.064e+06	3.587	3.587	2.158

Metabolite ID	Name	Super Pathway	Dataset	Sample ID	Group ID	Detection Fraction	Pathway	Detected	Raw Intensity	Log2 Norm Intensity	Norm Imputed	Log2 Ctrl Norm
19	Maltotetraose	Carbon	2	Ctrl_H2O2_2_1	Ctrl_H2O2_2	1.0	Glycogen degradation	True	1.794e+05	1.019	1.019	1.21
232	SAH	Amino acid	2	Ctrl_H2O2_2_1	Ctrl_H2O2_2	1.0	SAM metabolism	True	7565	-3.549	-3.549	-0.3227
74	Asp	Amino acid	2	Ctrl_H2O2_2_1	Ctrl_H2O2_2	1.0	Proteinogenic amino acids	True	7.897e+05	3.157	3.157	-2.502
129	5-Aminovalerate	Amino acid	2	Ctrl_H2O2_2_1	Ctrl_H2O2_2	0.0	Amino acids degradation intermediates	False			-1.567	-0.7538
254	Gly-Val	Amino acid	2	Ctrl_H2O2_2_1	Ctrl_H2O2_2	0.75	Dipeptides	True	5.238e+04	-0.7572	-0.7572	-0.6834
291	gamma-Glu-Leu	Amino acid	2	Ctrl_H2O2_2_1	Ctrl_H2O2_2	0.5	Gamma-glutamyl dipeptides	True	7.197e+04	-0.2988	-0.2988	0.7831
173	Met Sulfoxide	Amino acid	2	Ctrl_H2O2_2_1	Ctrl_H2O2_2	1.0	Amino acid derivatives	True	2.462e+05	1.476	1.476	1.615
43	Glucose	Carbon	2	Ctrl_H2O2_2_1	Ctrl_H2O2_2	1.0	Glycolysis, GNG	True	2.526e+07	8.157	8.157	1.337
249	Gly-Gly	Amino acid	2	Ctrl_H2O2_2_1	Ctrl_H2O2_2	0.0	Dipeptides	False			-1.272	-1.489
169	2-OH-Butyrate	Amino acid	2	Ctrl_H2O2_2_1	Ctrl_H2O2_2	0.25	Amino acid derivatives	False			-1.729	-0.2955
98	3-Methyl-2-Oxobutyrate	Amino acid	2	Ctrl_H2O2_2_1	Ctrl_H2O2_2	0.25	Amino acids biosynthesis intermediates	True	1.937e+04	-2.192	-2.192	1.567
100	4-Me-2-Oxo-Pentanoate	Amino acid	2	Ctrl_H2O2_2_1	Ctrl_H2O2_2	1.0	Amino acids biosynthesis intermediates	True	2.963e+04	-1.579	-1.579	1.723
253	Gly-Pro	Amino acid	2	Ctrl_H2O2_2_1	Ctrl_H2O2_2	0.75	Dipeptides	False			-1.487	-1.166
247	Asp-Phe	Amino acid	2	Ctrl_H2O2_2_1	Ctrl_H2O2_2	1.0	Dipeptides	True	2.786e+04	-1.668	-1.668	-0.7268
212	N-Ac-Asp	Amino acid	2	Ctrl_H2O2_2_1	Ctrl_H2O2_2	0.0	N-acetylated amino acids	False			-4.689	-0.1861
720	1-Me-Nicotinamide	Cofactor	2	Ctrl_H2O2_2_1	Ctrl_H2O2_2	1.0	Derivatives of NA, nicotinamide and NAD	True	2.131e+05	1.267	1.267	-2.436
70	Creatine	Carbon	2	Ctrl_H2O2_2_1	Ctrl_H2O2_2	1.0	Creatine energy storage	True	3.017e+06	5.091	5.091	-0.6894
309	Glutathione, Oxidized	Amino acid	2	Ctrl_H2O2_2_1	Ctrl_H2O2_2	1.0	Glutathione	True	1.341e+06	3.921	3.921	-0.02756
44	Glucose 6-P	Carbon	2	Ctrl_H2O2_2_1	Ctrl_H2O2_2	1.0	Glycolysis, GNG	True	1.603e+05	0.8566	0.8566	0.04179
24	Fructose	Carbon	2	Ctrl_H2O2_2_1	Ctrl_H2O2_2	1.0	Sugars and sugar alcohols	True	4.391e+06	5.632	5.632	2.272
85	Cys	Amino acid	2	Ctrl_H2O2_2_1	Ctrl_H2O2_2	1.0	Proteinogenic amino acids	True	3.855e+04	-1.199	-1.199	-0.6075
704	NADH	Cofactor	2	Ctrl_H2O2_2_1	Ctrl_H2O2_2	0.0	Cofactors	False			-4.313	-2.383
275	Thr-Phe	Amino acid	2	Ctrl_H2O2_2_1	Ctrl_H2O2_2	0.0	Dipeptides	False			-1.922	-0.9888

Metabolite ID	Name	Super Pathway	Dataset	Sample ID	Group ID	Detection Fraction	Pathway	Detected	Raw Intensity	Log2 Norm Intensity	Norm Imputed	Log2 Ctrl Norm
738	Pyridoxate	Cofactor	2	Ctrl_H2O2_2_1	Ctrl_H2O2_2	0.75	PLP biosynthesis and salvage	True	2.092e+04	-2.082	-2.082	2.584
177	3-(4-OH-Phenyl)Lactate	Amino acid	2	Ctrl_H2O2_2_1	Ctrl_H2O2_2	0.5	Amino acid derivatives	True	1.119e+04	-2.984	-2.984	0.9692
206	Trans-4-OH-Pro	Amino acid	2	Ctrl_H2O2_2_1	Ctrl_H2O2_2	1.0	Amino acid derivatives	True	1.691e+05	0.9336	0.9336	1.129
329	AMP	Nucleotide	2	Ctrl_H2O2_2_1	Ctrl_H2O2_2	0.25	Purine nucleotides	False			-2.644	-3.064
345	Guanine	Nucleotide	2	Ctrl_H2O2_2_1	Ctrl_H2O2_2	1.0	Purine bases	True	7.087e+05	3.001	3.001	1.419
271	pyroGlu-Val	Amino acid	2	Ctrl_H2O2_2_1	Ctrl_H2O2_2	0.75	Dipeptides	True	8338	-3.408	-3.408	0.2332
279	Val-Glu	Amino acid	2	Ctrl_H2O2_2_1	Ctrl_H2O2_2	1.0	Dipeptides	True	2.503e+04	-1.823	-1.823	-0.9927
183	Phenol Sulfate	Amino acid	2	Ctrl_H2O2_2_1	Ctrl_H2O2_2	0.25	Amino acid derivatives	True	1.59e+04	-2.477	-2.477	1.25
740	3-Dehydrocarnitine	Cofactor	2	Ctrl_H2O2_2_1	Ctrl_H2O2_2	0.5	Carnitine biosynthesis	True	3.885e+04	-1.188	-1.188	-0.8546
145	Pyro-Gln	Amino acid	2	Ctrl_H2O2_2_1	Ctrl_H2O2_2	1.0	Amino acid derivatives	True	1.951e+05	1.14	1.14	0.7941
197	C-Glycosyl-Trp	Amino acid	2	Ctrl_H2O2_2_1	Ctrl_H2O2_2	1.0	Amino acid derivatives	True	2.664e+04	-1.733	-1.733	-1.589
718	Nicotinamide Riboside	Cofactor	2	Ctrl_H2O2_2_1	Ctrl_H2O2_2	1.0	NAD biosynthesis	True	1.007e+05	0.1865	0.1865	1.99
295	gamma-Glu-Phe	Amino acid	2	Ctrl_H2O2_2_1	Ctrl_H2O2_2	0.25	Gamma-glutamyl dipeptides	False			-1.936	-1.834
399	Pseudouridine	Nucleotide	2	Ctrl_H2O2_2_1	Ctrl_H2O2_2	0.5	Pyrimidine derivatives in RNAs	True	1.837e+04	-2.269	-2.269	1.972
375	UTP	Nucleotide	2	Ctrl_H2O2_2_1	Ctrl_H2O2_2	0.0	Pyrimidine nucleotides	False			-5.966	-0.5235
20	Erythronate	Carbon	2	Ctrl_H2O2_2_1	Ctrl_H2O2_2	0.75	Aminosugar derivatives	True	4.563e+04	-0.9562	-0.9562	0.4853
151	Phenylacetylglycine	Amino acid	2	Ctrl_H2O2_2_1	Ctrl_H2O2_2	1.0	Amino acid derivatives	True	3.182e+04	-1.476	-1.476	2.945
252	Gly-Phe	Amino acid	2	Ctrl_H2O2_2_1	Ctrl_H2O2_2	0.75	Dipeptides	True	7.318e+04	-0.2746	-0.2746	-0.3788
251	Gly-Leu	Amino acid	2	Ctrl_H2O2_2_1	Ctrl_H2O2_2	1.0	Dipeptides	True	6.562e+04	-0.432	-0.432	-0.7946
290	gamma-Glu-Ile	Amino acid	2	Ctrl_H2O2_2_1	Ctrl_H2O2_2	0.5	Gamma-glutamyl dipeptides	True	2.652e+04	-1.739	-1.739	-0.2078
316	Ophthalmate	Amino acid	2	Ctrl_H2O2_2_1	Ctrl_H2O2_2	1.0	Oxidative stress markers	True	8.696e+04	-0.02585	-0.02585	-0.2801
208	Pro-OH-Pro	Amino acid	2	Ctrl_H2O2_2_1	Ctrl_H2O2_2	1.0	Amino acid derivatives	True	1.599e+05	0.8533	0.8533	0.3937
352	3'-AMP	Nucleotide	2	Ctrl_H2O2_2_1	Ctrl_H2O2_2	0.75	Purine derivatives in signaling	True	3.255e+04	-1.443	-1.443	-0.1585

Metabolite ID	Name	Super Pathway	Dataset	Sample ID	Group ID	Detection Fraction	Pathway	Detected	Raw Intensity	Log2 Norm Intensity	Norm Imputed	Log2 Ctrl Norm
314	Cys-Glutathione Disulfide	Amino acid	2	Ctrl_H2O2_2_1	Ctrl_H2O2_2	1.0	Oxidative stress markers	True	1.44e+05	0.702	0.702	2.067
39	Threitol	Carbon	2	Ctrl_H2O2_2_1	Ctrl_H2O2_2	0.25	Sugars and sugar alcohols	True	9695	-3.191	-3.191	0.8341
31	Ribulose/Xylulose	Carbon	2	Ctrl_H2O2_2_1	Ctrl_H2O2_2	0.0	Sugars and sugar alcohols	False			-5.004	-0.09716
48	DHAP	Carbon	2	Ctrl_H2O2_2_1	Ctrl_H2O2_2	1.0	Glycolysis, GNG	True	4.946e+04	-0.8399	-0.8399	-2.598
182	P-Cresol Sulfate	Amino acid	2	Ctrl_H2O2_2_1	Ctrl_H2O2_2	1.0	Amino acid derivatives	True	9352	-3.243	-3.243	0.09449
250	Gly-Ile	Amino acid	2	Ctrl_H2O2_2_1	Ctrl_H2O2_2	0.25	Dipeptides	False			-2.655	-1.231
286	gamma-Glu-Glu	Amino acid	2	Ctrl_H2O2_2_1	Ctrl_H2O2_2	1.0	Gamma-glutamyl dipeptides	True	5.488e+04	-0.69	-0.69	-0.4594
264	Leu-Leu	Amino acid	2	Ctrl_H2O2_2_1	Ctrl_H2O2_2	0.25	Dipeptides	True	3.291e+04	-1.428	-1.428	-0.3543
203	DiMe-Arg	Amino acid	2	Ctrl_H2O2_2_1	Ctrl_H2O2_2	1.0	Amino acid derivatives	True	4.857e+05	2.456	2.456	1.382
47	Fructose 1,6-PP, Glucose 1,6-PP	Carbon	2	Ctrl_H2O2_2_1	Ctrl_H2O2_2	1.0	Glycolysis, GNG	True	1.583e+04	-2.484	-2.484	-2.194
224	N-Ac-Ser	Amino acid	2	Ctrl_H2O2_2_1	Ctrl_H2O2_2	0.5	N-acetylated amino acids	True	2.628e+04	-1.752	-1.752	-2.623
244	Ala-Leu	Amino acid	2	Ctrl_H2O2_2_1	Ctrl_H2O2_2	0.25	Dipeptides	False			-1.778	-2.118
304	Cyclo(Phe-Pro)	Amino acid	2	Ctrl_H2O2_2_1	Ctrl_H2O2_2	0.25	Cyclic dipeptides	False			-1.192	-0.7201
302	Cyclo(Glu-Glu)	Amino acid	2	Ctrl_H2O2_2_1	Ctrl_H2O2_2	1.0	Cyclic dipeptides	True	5.022e+04	-0.8179	-0.8179	0.3543
303	Cyclo(Leu-Pro)	Amino acid	2	Ctrl_H2O2_2_1	Ctrl_H2O2_2	0.5	Cyclic dipeptides	False			-0.4297	-0.7026
390	2',3'-cUMP	Nucleotide	2	Ctrl_H2O2_2_1	Ctrl_H2O2_2	1.0	Pyrimidine derivatives in signaling	True	3.516e+04	-1.332	-1.332	-0.2035
68	Ribulose 5-P / Xylulose 5-P	Carbon	2	Ctrl_H2O2_2_1	Ctrl_H2O2_2	1.0	Pentose phosphate pathway (PPP)	True	2.919e+04	-1.601	-1.601	-2.37
388	2',3'-cCMP	Nucleotide	2	Ctrl_H2O2_2_1	Ctrl_H2O2_2	1.0	Pyrimidine derivatives in signaling	True	4.379e+04	-1.015	-1.015	-1.269
33	Arabitol/Xylitol	Carbon	2	Ctrl_H2O2_2_1	Ctrl_H2O2_2	0.0	Sugars and sugar alcohols	False			-3.559	-0.05408
268	Phe-Phe	Amino acid	2	Ctrl_H2O2_2_1	Ctrl_H2O2_2	0.5	Dipeptides	False			-2.762	-1.674
245	Ala-Phe	Amino acid	2	Ctrl_H2O2_2_1	Ctrl_H2O2_2	0.0	Dipeptides	False			-2.821	-1.366
373	UMP	Nucleotide	2	Ctrl_H2O2_2_1	Ctrl_H2O2_2	0.25	Pyrimidine nucleotides	False			-3.442	-1.402
282	Val-Leu	Amino acid	2	Ctrl_H2O2_2_1	Ctrl_H2O2_2	0.75	Dipeptides	True	3.756e+04	-1.237	-1.237	-0.8681
258	Ile-Gly	Amino acid	2	Ctrl_H2O2_2_1	Ctrl_H2O2_2	0.75	Dipeptides	False			-2.663	-3.077
259	Ile-Ser	Amino acid	2	Ctrl_H2O2_2_1	Ctrl_H2O2_2	0.25	Dipeptides	False			-3.192	-2.329

Metabolite ID	Name	Super Pathway	Dataset	Sample ID	Group ID	Detection Fraction	Pathway	Detected	Raw Intensity	Log2 Norm Intensity	Norm Imputed	Log2 Ctrl Norm
269	Phe-Ser	Amino acid	2	Ctrl_H2O2_2_1	Ctrl_H2O2_2	0.5	Dipeptides	False			-3.197	-1.882
277	Tyr-Ala	Amino acid	2	Ctrl_H2O2_2_1	Ctrl_H2O2_2	0.75	Dipeptides	False			-1.848	-2.418
257	Ile-Gln	Amino acid	2	Ctrl_H2O2_2_1	Ctrl_H2O2_2	0.25	Dipeptides	False			-2.625	-1.706
261	Leu-Glu	Amino acid	2	Ctrl_H2O2_2_1	Ctrl_H2O2_2	0.75	Dipeptides	False			-1.868	-2.658
263	Leu-Gly	Amino acid	2	Ctrl_H2O2_2_1	Ctrl_H2O2_2	1.0	Dipeptides	True	3.431e+04	-1.367	-1.367	-2.074
256	Ile-Ala	Amino acid	2	Ctrl_H2O2_2_1	Ctrl_H2O2_2	0.75	Dipeptides	False			-2.214	-2.173
274	Thr-Leu	Amino acid	2	Ctrl_H2O2_2_1	Ctrl_H2O2_2	0.25	Dipeptides	True	2.07e+05	1.226	1.226	0.3744
273	Ser-Phe	Amino acid	2	Ctrl_H2O2_2_1	Ctrl_H2O2_2	1.0	Dipeptides	True	1.823e+04	-2.28	-2.28	-1.058
272	Ser-Leu	Amino acid	2	Ctrl_H2O2_2_1	Ctrl_H2O2_2	1.0	Dipeptides	True	3.814e+04	-1.215	-1.215	-1.954
246	Asp-Leu	Amino acid	2	Ctrl_H2O2_2_1	Ctrl_H2O2_2	1.0	Dipeptides	True	6.375e+04	-0.4737	-0.4737	-0.3996
76	Gln	Amino acid	2	Ctrl_H2O2_2_2	Ctrl_H2O2_2	1.0	Proteinogenic amino acids	True	1.711e+07	7.621	7.621	0.6686
89	Trp	Amino acid	2	Ctrl_H2O2_2_2	Ctrl_H2O2_2	1.0	Proteinogenic amino acids	True	3.804e+06	5.452	5.452	-0.515
723	beta-Ala	Cofactor	2	Ctrl_H2O2_2_2	Ctrl_H2O2_2	0.75	Coenzyme A biosynthesis	True	2.474e+04	-1.813	-1.813	-2.14
75	Glu	Amino acid	2	Ctrl_H2O2_2_2	Ctrl_H2O2_2	1.0	Proteinogenic amino acids	True	4.596e+06	5.724	5.724	-0.2236
80	His	Amino acid	2	Ctrl_H2O2_2_2	Ctrl_H2O2_2	1.0	Proteinogenic amino acids	True	8.456e+04	-0.03988	-0.03988	-0.254
82	Leu	Amino acid	2	Ctrl_H2O2_2_2	Ctrl_H2O2_2	1.0	Proteinogenic amino acids	True	1.888e+07	7.762	7.762	-0.3978
87	Phe	Amino acid	2	Ctrl_H2O2_2_2	Ctrl_H2O2_2	1.0	Proteinogenic amino acids	True	1.591e+07	7.516	7.516	-0.3759
236	Spermidine	Amino acid	2	Ctrl_H2O2_2_2	Ctrl_H2O2_2	1.0	Polyamines	True	2.082e+05	1.26	1.26	-1.568
73	Asn	Amino acid	2	Ctrl_H2O2_2_2	Ctrl_H2O2_2	1.0	Proteinogenic amino acids	True	1.289e+05	0.5681	0.5681	-2.181
243	Creatinine	Amino acid	2	Ctrl_H2O2_2_2	Ctrl_H2O2_2	0.75	Creatine degradation	True	6.646e+05	2.934	2.934	1.364
376	Cytidine	Nucleotide	2	Ctrl_H2O2_2_2	Ctrl_H2O2_2	0.75	Pyrimidine nucleosides	False			-2.11	-1.525
41	Lactate	Carbon	2	Ctrl_H2O2_2_2	Ctrl_H2O2_2	1.0	Respiratory carbon sources	True	1.209e+07	7.119	7.119	-0.7027
93	3-P-Ser	Amino acid	2	Ctrl_H2O2_2_2	Ctrl_H2O2_2	0.5	Amino acids biosynthesis intermediates	True	6.853e+04	-0.3431	-0.3431	2.916
343	Adenine	Nucleotide	2	Ctrl_H2O2_2_2	Ctrl_H2O2_2	0.5	Purine bases	True	1.459e+04	-2.575	-2.575	-1.111
336	Adenosine	Nucleotide	2	Ctrl_H2O2_2_2	Ctrl_H2O2_2	1.0	Purine nucleosides	True	2.235e+05	1.362	1.362	-1.775

Metabolite ID	Name	Super Pathway	Dataset	Sample ID	Group ID	Detection Fraction	Pathway	Detected	Raw Intensity	Log2 Norm Intensity	Norm Imputed	Log2 Ctrl Norm
29	Raffinose	Carbon	2	Ctrl_H2O2_2_2	Ctrl_H2O2_2	0.5	Sugars and sugar alcohols	True	1.438e+04	-2.595	-2.595	-0.4155
717	Nicotinamide	Cofactor	2	Ctrl_H2O2_2_2	Ctrl_H2O2_2	1.0	NAD biosynthesis	True	6.179e+05	2.829	2.829	0.4327
51	PEP	Carbon	2	Ctrl_H2O2_2_2	Ctrl_H2O2_2	1.0	Glycolysis, GNG	True	2.916e+04	-1.576	-1.576	-0.5039
52	Pyruvate	Carbon	2	Ctrl_H2O2_2_2	Ctrl_H2O2_2	0.25	Glycolysis, GNG	False			-3.589	-1.215
237	Spermine	Amino acid	2	Ctrl_H2O2_2_2	Ctrl_H2O2_2	0.75	Polyamines	True	3.104e+05	1.836	1.836	-1.553
385	Uracil	Nucleotide	2	Ctrl_H2O2_2_2	Ctrl_H2O2_2	1.0	Pyrimidine bases	True	2.084e+04	-2.061	-2.061	-0.334
377	Uridine	Nucleotide	2	Ctrl_H2O2_2_2	Ctrl_H2O2_2	1.0	Pyrimidine nucleosides	True	3.515e+05	2.016	2.016	-0.8668
112	trans-Urocanate	Amino acid	2	Ctrl_H2O2_2_2	Ctrl_H2O2_2	0.75	Amino acids degradation intermediates	False			-3.252	-0.1347
737	Pyridoxine (Vitamin B6)	Cofactor	2	Ctrl_H2O2_2_2	Ctrl_H2O2_2	1.0	PLP biosynthesis and salvage	True	1.74e+06	4.323	4.323	1.023
348	Allantoin	Nucleotide	2	Ctrl_H2O2_2_2	Ctrl_H2O2_2	1.0	Purine degradation	True	1.196e+04	-2.862	-2.862	-0.03258
335	Inosine	Nucleotide	2	Ctrl_H2O2_2_2	Ctrl_H2O2_2	1.0	Purine nucleosides	True	1.572e+05	0.8546	0.8546	-2.007
81	Ile	Amino acid	2	Ctrl_H2O2_2_2	Ctrl_H2O2_2	1.0	Proteinogenic amino acids	True	2.062e+07	7.89	7.89	0.03439
72	Ala	Amino acid	2	Ctrl_H2O2_2_2	Ctrl_H2O2_2	1.0	Proteinogenic amino acids	True	6.076e+06	6.127	6.127	-1.607
79	Thr	Amino acid	2	Ctrl_H2O2_2_2	Ctrl_H2O2_2	1.0	Proteinogenic amino acids	True	1.583e+06	4.187	4.187	-1.776
88	Tyr	Amino acid	2	Ctrl_H2O2_2_2	Ctrl_H2O2_2	1.0	Proteinogenic amino acids	True	7.472e+06	6.425	6.425	-0.417
84	Lys	Amino acid	2	Ctrl_H2O2_2_2	Ctrl_H2O2_2	1.0	Proteinogenic amino acids	True	1.373e+06	3.981	3.981	0.2616
86	Met	Amino acid	2	Ctrl_H2O2_2_2	Ctrl_H2O2_2	1.0	Proteinogenic amino acids	True	3.219e+06	5.211	5.211	-0.5765
61	Malate	Carbon	2	Ctrl_H2O2_2_2	Ctrl_H2O2_2	1.0	TCA cycle	True	5.216e+04	-0.7369	-0.7369	-1.634
235	Putrescine	Amino acid	2	Ctrl_H2O2_2_2	Ctrl_H2O2_2	0.5	Polyamines	False			-3.898	-2.331
49	3-P-Glycerate	Carbon	2	Ctrl_H2O2_2_2	Ctrl_H2O2_2	1.0	Glycolysis, GNG	True	4.112e+05	2.242	2.242	-0.2207
139	GABA	Amino acid	2	Ctrl_H2O2_2_2	Ctrl_H2O2_2	0.25	Amino acid derivatives	True	8250	-3.397	-3.397	0.07313
189	Kynurenate	Amino acid	2	Ctrl_H2O2_2_2	Ctrl_H2O2_2	0.5	Amino acid derivatives	True	5131	-4.083	-4.083	1.105
234	5-Me-Thioadenosine	Amino acid	2	Ctrl_H2O2_2_2	Ctrl_H2O2_2	1.0	SAM metabolism	True	1.997e+04	-2.122	-2.122	-2.026
59	Succinate	Carbon	2	Ctrl_H2O2_2_2	Ctrl_H2O2_2	0.75	TCA cycle	True	1.271e+04	-2.774	-2.774	-0.7338

Metabolite ID	Name	Super Pathway	Dataset	Sample ID	Group ID	Detection Fraction	Pathway	Detected	Raw Intensity	Log2 Norm Intensity	Norm Imputed	Log2 Ctrl Norm
133	Ornithine	Amino acid	2	Ctrl_H2O2_2_2	Ctrl_H2O2_2	1.0	Amino acids degradation intermediates	True	1.495e+05	0.7823	0.7823	-1.739
313	5-Oxoproline	Amino acid	2	Ctrl_H2O2_2_2	Ctrl_H2O2_2	1.0	Glutathione derivatives	True	7.721e+05	3.151	3.151	1.147
724	Pantothenate	Cofactor	2	Ctrl_H2O2_2_2	Ctrl_H2O2_2	1.0	Coenzyme A biosynthesis	True	7.573e+05	3.123	3.123	9.35e-03
30	Sucrose	Carbon	2	Ctrl_H2O2_2_2	Ctrl_H2O2_2	1.0	Sugars and sugar alcohols	True	4.828e+05	2.473	2.473	0.09665
122	3-OH-Isobutyrate	Amino acid	2	Ctrl_H2O2_2_2	Ctrl_H2O2_2	0.0	Amino acids degradation intermediates	False			-4.628	0
241	4-Acetamidobutanoate	Amino acid	2	Ctrl_H2O2_2_2	Ctrl_H2O2_2	1.0	Polyamine derivatives	True	5.442e+04	-0.6757	-0.6757	0.8316
55	Citrate	Carbon	2	Ctrl_H2O2_2_2	Ctrl_H2O2_2	1.0	TCA cycle	True	6.058e+05	2.801	2.801	-0.2335
338	Guanosine	Nucleotide	2	Ctrl_H2O2_2_2	Ctrl_H2O2_2	1.0	Purine nucleosides	True	2.756e+05	1.665	1.665	-0.6959
170	2-Amino-Butyrate	Amino acid	2	Ctrl_H2O2_2_2	Ctrl_H2O2_2	1.0	Amino acid derivatives	True	2.258e+05	1.377	1.377	0.1535
209	N-Ac-Ala	Amino acid	2	Ctrl_H2O2_2_2	Ctrl_H2O2_2	0.0	N-acetylated amino acids	False			-4.401	-1.133
221	N-Ac-Met	Amino acid	2	Ctrl_H2O2_2_2	Ctrl_H2O2_2	1.0	N-acetylated amino acids	True	9066	-3.261	-3.261	-2.111
22	N-Ac-Neuraminate	Carbon	2	Ctrl_H2O2_2_2	Ctrl_H2O2_2	0.5	Aminosugar derivatives	False			-1.69	-1.476
346	Urate	Nucleotide	2	Ctrl_H2O2_2_2	Ctrl_H2O2_2	0.5	Purine degradation	False			-4.123	-0.4311
90	Arg	Amino acid	2	Ctrl_H2O2_2_2	Ctrl_H2O2_2	1.0	Proteinogenic amino acids	True	2.539e+06	4.868	4.868	0.08592
60	Fumarate	Carbon	2	Ctrl_H2O2_2_2	Ctrl_H2O2_2	1.0	TCA cycle	True	4.269e+04	-1.026	-1.026	-0.8694
78	Ser	Amino acid	2	Ctrl_H2O2_2_2	Ctrl_H2O2_2	1.0	Proteinogenic amino acids	True	2.766e+06	4.991	4.991	-1.835
83	Val	Amino acid	2	Ctrl_H2O2_2_2	Ctrl_H2O2_2	1.0	Proteinogenic amino acids	True	1.324e+07	7.251	7.251	0.01684
734	Pyridoxal	Cofactor	2	Ctrl_H2O2_2_2	Ctrl_H2O2_2	0.5	PLP biosynthesis and salvage	True	4.486e+04	-0.9544	-0.9544	0.04023
136	Urea	Amino acid	2	Ctrl_H2O2_2_2	Ctrl_H2O2_2	1.0	Amino acids degradation intermediates	True	2.992e+05	1.783	1.783	0.1379
742	Folate	Cofactor	2	Ctrl_H2O2_2_2	Ctrl_H2O2_2	1.0	Folate metabolism	True	1.043e+05	0.2629	0.2629	0.7799
729	Riboflavin (Vitamin B2)	Cofactor	2	Ctrl_H2O2_2_2	Ctrl_H2O2_2	1.0	Flavine biosynthesis	True	2.929e+04	-1.57	-1.57	-5.11e-04
91	Pro	Amino acid	2	Ctrl_H2O2_2_2	Ctrl_H2O2_2	1.0	Proteinogenic amino acids	True	3.803e+06	5.451	5.451	-1.27

Metabolite ID	Name	Super Pathway	Dataset	Sample ID	Group ID	Detection Fraction	Pathway	Detected	Raw Intensity	Log2 Norm Intensity	Norm Imputed	Log2 Ctrl Norm
308	Glutathione, Reduced	Amino acid	2	Ctrl_H2O2_2_2	Ctrl_H2O2_2	1.0	Glutathione	True	1.091e+06	3.65	3.65	-2.805
706	FAD	Cofactor	2	Ctrl_H2O2_2_2	Ctrl_H2O2_2	0.0	Cofactors	False			-5.096	0
299	gamma-Glu-Tyr	Amino acid	2	Ctrl_H2O2_2_2	Ctrl_H2O2_2	0.5	Gamma-glutamyl dipeptides	True	3.805e+04	-1.192	-1.192	-0.02484
705	Coenzyme A	Cofactor	2	Ctrl_H2O2_2_2	Ctrl_H2O2_2	0.75	Cofactors	False			-6.848	-3.141
342	Hypoxanthine	Nucleotide	2	Ctrl_H2O2_2_2	Ctrl_H2O2_2	1.0	Purine bases	True	6.019e+04	-0.5303	-0.5303	-0.5322
344	Xanthine	Nucleotide	2	Ctrl_H2O2_2_2	Ctrl_H2O2_2	0.75	Purine bases	True	1.098e+04	-2.986	-2.986	-0.7465
703	NAD+	Cofactor	2	Ctrl_H2O2_2_2	Ctrl_H2O2_2	1.0	Cofactors	True	2.594e+05	1.577	1.577	-1.982
731	Thiamin (Vitamin B1)	Cofactor	2	Ctrl_H2O2_2_2	Ctrl_H2O2_2	1.0	TPP biosynthesis	True	1.202e+05	0.4678	0.4678	0.6739
102	2-Aminoadipate	Amino acid	2	Ctrl_H2O2_2_2	Ctrl_H2O2_2	1.0	Amino acids biosynthesis intermediates	True	4.598e+04	-0.9191	-0.9191	-1.676
77	Gly	Amino acid	2	Ctrl_H2O2_2_2	Ctrl_H2O2_2	1.0	Proteinogenic amino acids	True	3.195e+06	5.2	5.2	-2.029
45	Fructose-6-P	Carbon	2	Ctrl_H2O2_2_2	Ctrl_H2O2_2	0.75	Glycolysis, GNG	True	2.482e+04	-1.808	-1.808	-0.3729
36	Ribose	Carbon	2	Ctrl_H2O2_2_2	Ctrl_H2O2_2	0.75	Sugars and sugar alcohols	True	1.872e+04	-2.215	-2.215	-1.218
4	GlcNAc 6-P	Carbon	2	Ctrl_H2O2_2_2	Ctrl_H2O2_2	1.0	Aminosugar biosynthesis	True	3.048e+04	-1.512	-1.512	-1.872
188	Kynurenine	Amino acid	2	Ctrl_H2O2_2_2	Ctrl_H2O2_2	0.75	Amino acid derivatives	False			-1.614	-1.6
63	6-P-Gluconate	Carbon	2	Ctrl_H2O2_2_2	Ctrl_H2O2_2	1.0	Pentose phosphate pathway (PPP)	True	1.375e+05	0.6612	0.6612	1.706
710	Carnitine	Cofactor	2	Ctrl_H2O2_2_2	Ctrl_H2O2_2	1.0	Cofactors	True	7.82e+04	-0.1527	-0.1527	-2.684
725	P-Pantetheine	Cofactor	2	Ctrl_H2O2_2_2	Ctrl_H2O2_2	0.75	Coenzyme A biosynthesis	True	6131	-3.826	-3.826	0.9868
110	N-alpha-Ac-Ornithine	Amino acid	2	Ctrl_H2O2_2_2	Ctrl_H2O2_2	1.0	Amino acids biosynthesis intermediates	True	1.404e+05	0.6916	0.6916	-0.2825
116	3-Me-2-Oxo-Valerate	Amino acid	2	Ctrl_H2O2_2_2	Ctrl_H2O2_2	1.0	Amino acids degradation intermediates	True	2.881e+04	-1.594	-1.594	1.981
155	4-Guanidinobutanoate	Amino acid	2	Ctrl_H2O2_2_2	Ctrl_H2O2_2	0.5	Amino acid derivatives	True	3.559e+04	-1.288	-1.288	-0.5689
310	S-Lactoyl-Glutathione	Amino acid	2	Ctrl_H2O2_2_2	Ctrl_H2O2_2	0.0	Glutathione derivatives	False			-4.634	-0.962
34	Ribitol	Carbon	2	Ctrl_H2O2_2_2	Ctrl_H2O2_2	0.75	Sugars and sugar alcohols	False			-2.846	-1.267
707	FMN	Cofactor	2	Ctrl_H2O2_2_2	Ctrl_H2O2_2	0.5	Cofactors	False			-4.56	-1.139

Metabolite ID	Name	Super Pathway	Dataset	Sample ID	Group ID	Detection Fraction	Pathway	Detected	Raw Intensity	Log2 Norm Intensity	Norm Imputed	Log2 Ctrl Norm
17	Maltose	Carbon	2	Ctrl_H2O2_2_2	Ctrl_H2O2_2	1.0	Glycogen degradation	True	1.254e+05	0.5286	0.5286	0.568
18	Maltotriose	Carbon	2	Ctrl_H2O2_2_2	Ctrl_H2O2_2	1.0	Glycogen degradation	True	2.991e+05	1.782	1.782	0.3536
19	Maltotetraose	Carbon	2	Ctrl_H2O2_2_2	Ctrl_H2O2_2	1.0	Glycogen degradation	True	2.24e+05	1.365	1.365	1.556
232	SAH	Amino acid	2	Ctrl_H2O2_2_2	Ctrl_H2O2_2	1.0	SAM metabolism	True	7312	-3.572	-3.572	-0.3455
74	Asp	Amino acid	2	Ctrl_H2O2_2_2	Ctrl_H2O2_2	1.0	Proteinogenic amino acids	True	1.679e+06	4.272	4.272	-1.388
129	5-Aminovalerate	Amino acid	2	Ctrl_H2O2_2_2	Ctrl_H2O2_2	0.0	Amino acids degradation intermediates	False			-1.567	-0.7538
254	Gly-Val	Amino acid	2	Ctrl_H2O2_2_2	Ctrl_H2O2_2	0.75	Dipeptides	True	6.2e+04	-0.4876	-0.4876	-0.4139
291	gamma-Glu-Leu	Amino acid	2	Ctrl_H2O2_2_2	Ctrl_H2O2_2	0.5	Gamma-glutamyl dipeptides	False			-2.038	-0.9559
173	Met Sulfoxide	Amino acid	2	Ctrl_H2O2_2_2	Ctrl_H2O2_2	1.0	Amino acid derivatives	True	2.334e+05	1.425	1.425	1.564
43	Glucose	Carbon	2	Ctrl_H2O2_2_2	Ctrl_H2O2_2	1.0	Glycolysis, GNG	True	2.047e+07	7.879	7.879	1.06
249	Gly-Gly	Amino acid	2	Ctrl_H2O2_2_2	Ctrl_H2O2_2	0.0	Dipeptides	False			-1.272	-1.489
169	2-OH-Butyrate	Amino acid	2	Ctrl_H2O2_2_2	Ctrl_H2O2_2	0.25	Amino acid derivatives	False			-1.729	-0.2955
98	3-Methyl-2-Oxobutyrate	Amino acid	2	Ctrl_H2O2_2_2	Ctrl_H2O2_2	0.25	Amino acids biosynthesis intermediates	False			-3.759	0
100	4-Me-2-Oxo-Pentanoate	Amino acid	2	Ctrl_H2O2_2_2	Ctrl_H2O2_2	1.0	Amino acids biosynthesis intermediates	True	1.346e+04	-2.691	-2.691	0.6106
253	Gly-Pro	Amino acid	2	Ctrl_H2O2_2_2	Ctrl_H2O2_2	0.75	Dipeptides	True	5.877e+04	-0.5648	-0.5648	-0.2437
247	Asp-Phe	Amino acid	2	Ctrl_H2O2_2_2	Ctrl_H2O2_2	1.0	Dipeptides	True	2.933e+04	-1.567	-1.567	-0.6261
212	N-Ac-Asp	Amino acid	2	Ctrl_H2O2_2_2	Ctrl_H2O2_2	0.0	N-acetylated amino acids	False			-4.689	-0.1861
720	1-Me-Nicotinamide	Cofactor	2	Ctrl_H2O2_2_2	Ctrl_H2O2_2	1.0	Derivatives of NA, nicotinamide and NAD	True	4.658e+05	2.422	2.422	-1.282
70	Creatine	Carbon	2	Ctrl_H2O2_2_2	Ctrl_H2O2_2	1.0	Creatine energy storage	True	2.619e+06	4.913	4.913	-0.8674
309	Glutathione, Oxidized	Amino acid	2	Ctrl_H2O2_2_2	Ctrl_H2O2_2	1.0	Glutathione	True	8.963e+05	3.366	3.366	-0.5826
44	Glucose 6-P	Carbon	2	Ctrl_H2O2_2_2	Ctrl_H2O2_2	1.0	Glycolysis, GNG	True	1.595e+05	0.8757	0.8757	0.06095
24	Fructose	Carbon	2	Ctrl_H2O2_2_2	Ctrl_H2O2_2	1.0	Sugars and sugar alcohols	True	1.02e+06	3.553	3.553	0.1925

Metabolite ID	Name	Super Pathway	Dataset	Sample ID	Group ID	Detection Fraction	Pathway	Detected	Raw Intensity	Log2 Norm Intensity	Norm Imputed	Log2 Ctrl Norm
85	Cys	Amino acid	2	Ctrl_H2O2_2_2	Ctrl_H2O2_2	1.0	Proteinogenic amino acids	True	4.075e+04	-1.093	-1.093	-0.5012
704	NADH	Cofactor	2	Ctrl_H2O2_2_2	Ctrl_H2O2_2	0.0	Cofactors	False			-4.313	-2.383
275	Thr-Phe	Amino acid	2	Ctrl_H2O2_2_2	Ctrl_H2O2_2	0.0	Dipeptides	False			-1.922	-0.9888
738	Pyridoxate	Cofactor	2	Ctrl_H2O2_2_2	Ctrl_H2O2_2	0.75	PLP biosynthesis and salvage	True	9072	-3.26	-3.26	1.405
177	3-(4-OH-Phenyl)Lactate	Amino acid	2	Ctrl_H2O2_2_2	Ctrl_H2O2_2	0.5	Amino acid derivatives	False			-4.295	-0.342
206	Trans-4-OH-Pro	Amino acid	2	Ctrl_H2O2_2_2	Ctrl_H2O2_2	1.0	Amino acid derivatives	True	5.5e+04	-0.6605	-0.6605	-0.4649
329	AMP	Nucleotide	2	Ctrl_H2O2_2_2	Ctrl_H2O2_2	0.25	Purine nucleotides	True	1.625e+04	-2.42	-2.42	-2.84
345	Guanine	Nucleotide	2	Ctrl_H2O2_2_2	Ctrl_H2O2_2	1.0	Purine bases	True	3.683e+05	2.083	2.083	0.501
271	pyroGlu-Val	Amino acid	2	Ctrl_H2O2_2_2	Ctrl_H2O2_2	0.75	Dipeptides	True	1.325e+04	-2.714	-2.714	0.9274
279	Val-Glu	Amino acid	2	Ctrl_H2O2_2_2	Ctrl_H2O2_2	1.0	Dipeptides	True	2.207e+04	-1.978	-1.978	-1.148
183	Phenol Sulfate	Amino acid	2	Ctrl_H2O2_2_2	Ctrl_H2O2_2	0.25	Amino acid derivatives	False			-4.072	-0.3451
740	3-Dehydrocarnitine	Cofactor	2	Ctrl_H2O2_2_2	Ctrl_H2O2_2	0.5	Carnitine biosynthesis	False			-2.199	-1.865
145	Pyro-Gln	Amino acid	2	Ctrl_H2O2_2_2	Ctrl_H2O2_2	1.0	Amino acid derivatives	True	1.336e+05	0.6202	0.6202	0.2742
197	C-Glycosyl-Trp	Amino acid	2	Ctrl_H2O2_2_2	Ctrl_H2O2_2	1.0	Amino acid derivatives	True	2.381e+04	-1.868	-1.868	-1.724
718	Nicotinamide Riboside	Cofactor	2	Ctrl_H2O2_2_2	Ctrl_H2O2_2	1.0	NAD biosynthesis	True	3.746e+04	-1.215	-1.215	0.5891
295	gamma-Glu-Phe	Amino acid	2	Ctrl_H2O2_2_2	Ctrl_H2O2_2	0.25	Gamma-glutamyl dipeptides	False			-1.936	-1.834
399	Pseudouridine	Nucleotide	2	Ctrl_H2O2_2_2	Ctrl_H2O2_2	0.5	Pyrimidine derivatives in RNAs	False			-4.566	-0.3249
375	UTP	Nucleotide	2	Ctrl_H2O2_2_2	Ctrl_H2O2_2	0.0	Pyrimidine nucleotides	False			-5.966	-0.5235
20	Erythronate	Carbon	2	Ctrl_H2O2_2_2	Ctrl_H2O2_2	0.75	Aminosugar derivatives	True	1.612e+04	-2.431	-2.431	-0.9891
151	Phenylacetylglycine	Amino acid	2	Ctrl_H2O2_2_2	Ctrl_H2O2_2	1.0	Amino acid derivatives	True	1.335e+04	-2.703	-2.703	1.718
252	Gly-Phe	Amino acid	2	Ctrl_H2O2_2_2	Ctrl_H2O2_2	0.75	Dipeptides	False			-0.4688	-0.573
251	Gly-Leu	Amino acid	2	Ctrl_H2O2_2_2	Ctrl_H2O2_2	1.0	Dipeptides	True	6.977e+04	-0.3173	-0.3173	-0.6799
290	gamma-Glu-Ile	Amino acid	2	Ctrl_H2O2_2_2	Ctrl_H2O2_2	0.5	Gamma-glutamyl dipeptides	False			-2.961	-1.429
316	Ophthalmate	Amino acid	2	Ctrl_H2O2_2_2	Ctrl_H2O2_2	1.0	Oxidative stress markers	True	1.797e+04	-2.274	-2.274	-2.529

Metabolite ID	Name	Super Pathway	Dataset	Sample ID	Group ID	Detection Fraction	Pathway	Detected	Raw Intensity	Log2 Norm Intensity	Norm Imputed	Log2 Ctrl Norm
208	Pro-OH-Pro	Amino acid	2	Ctrl_H2O2_2_2	Ctrl_H2O2_2	1.0	Amino acid derivatives	True	1.227e+05	0.4971	0.4971	0.03747
352	3'-AMP	Nucleotide	2	Ctrl_H2O2_2_2	Ctrl_H2O2_2	0.75	Purine derivatives in signaling	False			-2.226	-0.9415
314	Cys-Glutathione Disulfide	Amino acid	2	Ctrl_H2O2_2_2	Ctrl_H2O2_2	1.0	Oxidative stress markers	True	1.475e+05	0.7624	0.7624	2.128
39	Threitol	Carbon	2	Ctrl_H2O2_2_2	Ctrl_H2O2_2	0.25	Sugars and sugar alcohols	False			-4.395	-0.3698
31	Ribulose/Xylulose	Carbon	2	Ctrl_H2O2_2_2	Ctrl_H2O2_2	0.0	Sugars and sugar alcohols	False			-5.004	-0.09716
48	DHAP	Carbon	2	Ctrl_H2O2_2_2	Ctrl_H2O2_2	1.0	Glycolysis, GNG	True	3.791e+04	-1.197	-1.197	-2.955
182	P-Cresol Sulfate	Amino acid	2	Ctrl_H2O2_2_2	Ctrl_H2O2_2	1.0	Amino acid derivatives	True	6155	-3.82	-3.82	-0.4829
250	Gly-Ile	Amino acid	2	Ctrl_H2O2_2_2	Ctrl_H2O2_2	0.25	Dipeptides	False			-2.655	-1.231
286	gamma-Glu-Glu	Amino acid	2	Ctrl_H2O2_2_2	Ctrl_H2O2_2	1.0	Gamma-glutamyl dipeptides	True	3.937e+04	-1.143	-1.143	-0.9122
264	Leu-Leu	Amino acid	2	Ctrl_H2O2_2_2	Ctrl_H2O2_2	0.25	Dipeptides	False			-2.222	-1.148
203	DiMe-Arg	Amino acid	2	Ctrl_H2O2_2_2	Ctrl_H2O2_2	1.0	Amino acid derivatives	True	2.435e+05	1.486	1.486	0.4113
47	Fructose 1,6-PP, Glucose 1,6-PP	Carbon	2	Ctrl_H2O2_2_2	Ctrl_H2O2_2	1.0	Glycolysis, GNG	True	2.978e+04	-1.545	-1.545	-1.255
224	N-Ac-Ser	Amino acid	2	Ctrl_H2O2_2_2	Ctrl_H2O2_2	0.5	N-acetylated amino acids	False			-1.752	-2.623
244	Ala-Leu	Amino acid	2	Ctrl_H2O2_2_2	Ctrl_H2O2_2	0.25	Dipeptides	False			-1.778	-2.118
304	Cyclo(Phe-Pro)	Amino acid	2	Ctrl_H2O2_2_2	Ctrl_H2O2_2	0.25	Cyclic dipeptides	True	9.898e+04	0.1873	0.1873	0.659
302	Cyclo(Glu-Glu)	Amino acid	2	Ctrl_H2O2_2_2	Ctrl_H2O2_2	1.0	Cyclic dipeptides	True	7.388e+04	-0.2348	-0.2348	0.9374
303	Cyclo(Leu-Pro)	Amino acid	2	Ctrl_H2O2_2_2	Ctrl_H2O2_2	0.5	Cyclic dipeptides	True	1.041e+05	0.2604	0.2604	-0.01247
390	2',3'-cUMP	Nucleotide	2	Ctrl_H2O2_2_2	Ctrl_H2O2_2	1.0	Pyrimidine derivatives in signaling	True	3.377e+04	-1.364	-1.364	-0.2353
68	Ribulose 5-P / Xylulose 5-P	Carbon	2	Ctrl_H2O2_2_2	Ctrl_H2O2_2	1.0	Pentose phosphate pathway (PPP)	True	3.918e+04	-1.15	-1.15	-1.919
388	2',3'-cCMP	Nucleotide	2	Ctrl_H2O2_2_2	Ctrl_H2O2_2	1.0	Pyrimidine derivatives in signaling	True	4.063e+04	-1.097	-1.097	-1.351
33	Arabitol/Xylitol	Carbon	2	Ctrl_H2O2_2_2	Ctrl_H2O2_2	0.0	Sugars and sugar alcohols	False			-3.559	-0.05408
268	Phe-Phe	Amino acid	2	Ctrl_H2O2_2_2	Ctrl_H2O2_2	0.5	Dipeptides	True	2.879e+04	-1.595	-1.595	-0.5069
245	Ala-Phe	Amino acid	2	Ctrl_H2O2_2_2	Ctrl_H2O2_2	0.0	Dipeptides	False			-2.821	-1.366
373	UMP	Nucleotide	2	Ctrl_H2O2_2_2	Ctrl_H2O2_2	0.25	Pyrimidine nucleotides	False			-3.442	-1.402

Metabolite ID	Name	Super Pathway	Dataset	Sample ID	Group ID	Detection Fraction	Pathway	Detected	Raw Intensity	Log2 Norm Intensity	Norm Imputed	Log2 Ctrl Norm
282	Val-Leu	Amino acid	2	Ctrl_H2O2_2_2	Ctrl_H2O2_2	0.75	Dipeptides	False			-1.414	-1.045
258	Ile-Gly	Amino acid	2	Ctrl_H2O2_2_2	Ctrl_H2O2_2	0.75	Dipeptides	True	1.03e+05	0.2453	0.2453	-0.1693
259	Ile-Ser	Amino acid	2	Ctrl_H2O2_2_2	Ctrl_H2O2_2	0.25	Dipeptides	True	2.77e+04	-1.65	-1.65	-0.7872
269	Phe-Ser	Amino acid	2	Ctrl_H2O2_2_2	Ctrl_H2O2_2	0.5	Dipeptides	True	2.169e+04	-2.003	-2.003	-0.6878
277	Tyr-Ala	Amino acid	2	Ctrl_H2O2_2_2	Ctrl_H2O2_2	0.75	Dipeptides	True	3.304e+04	-1.396	-1.396	-1.966
257	Ile-Gln	Amino acid	2	Ctrl_H2O2_2_2	Ctrl_H2O2_2	0.25	Dipeptides	False			-2.625	-1.706
261	Leu-Glu	Amino acid	2	Ctrl_H2O2_2_2	Ctrl_H2O2_2	0.75	Dipeptides	True	2.382e+04	-1.868	-1.868	-2.658
263	Leu-Gly	Amino acid	2	Ctrl_H2O2_2_2	Ctrl_H2O2_2	1.0	Dipeptides	True	7.971e+04	-0.1252	-0.1252	-0.8321
256	Ile-Ala	Amino acid	2	Ctrl_H2O2_2_2	Ctrl_H2O2_2	0.75	Dipeptides	True	1.079e+05	0.3116	0.3116	0.3534
274	Thr-Leu	Amino acid	2	Ctrl_H2O2_2_2	Ctrl_H2O2_2	0.25	Dipeptides	False			0.2082	-0.6429
273	Ser-Phe	Amino acid	2	Ctrl_H2O2_2_2	Ctrl_H2O2_2	1.0	Dipeptides	True	1.774e+04	-2.293	-2.293	-1.071
272	Ser-Leu	Amino acid	2	Ctrl_H2O2_2_2	Ctrl_H2O2_2	1.0	Dipeptides	True	2.56e+04	-1.764	-1.764	-2.503
246	Asp-Leu	Amino acid	2	Ctrl_H2O2_2_2	Ctrl_H2O2_2	1.0	Dipeptides	True	8.937e+04	0.03988	0.03988	0.1139
76	Gln	Amino acid	2	Ctrl_H2O2_2_3	Ctrl_H2O2_2	1.0	Proteinogenic amino acids	True	1.065e+07	6.811	6.811	-0.1407
89	Trp	Amino acid	2	Ctrl_H2O2_2_3	Ctrl_H2O2_2	1.0	Proteinogenic amino acids	True	3.715e+06	5.292	5.292	-0.6742
723	beta-Ala	Cofactor	2	Ctrl_H2O2_2_3	Ctrl_H2O2_2	0.75	Coenzyme A biosynthesis	False			-2.829	-3.156
75	Glu	Amino acid	2	Ctrl_H2O2_2_3	Ctrl_H2O2_2	1.0	Proteinogenic amino acids	True	4.429e+06	5.546	5.546	-0.402
80	His	Amino acid	2	Ctrl_H2O2_2_3	Ctrl_H2O2_2	1.0	Proteinogenic amino acids	True	8.037e+04	-0.2383	-0.2383	-0.4524
82	Leu	Amino acid	2	Ctrl_H2O2_2_3	Ctrl_H2O2_2	1.0	Proteinogenic amino acids	True	1.961e+07	7.693	7.693	-0.4674
87	Phe	Amino acid	2	Ctrl_H2O2_2_3	Ctrl_H2O2_2	1.0	Proteinogenic amino acids	True	1.727e+07	7.509	7.509	-0.3824
236	Spermidine	Amino acid	2	Ctrl_H2O2_2_3	Ctrl_H2O2_2	1.0	Polyamines	True	2.485e+05	1.39	1.39	-1.437
73	Asn	Amino acid	2	Ctrl_H2O2_2_3	Ctrl_H2O2_2	1.0	Proteinogenic amino acids	True	7.039e+04	-0.4295	-0.4295	-3.178
243	Creatinine	Amino acid	2	Ctrl_H2O2_2_3	Ctrl_H2O2_2	0.75	Creatine degradation	True	4.846e+05	2.354	2.354	0.7832
376	Cytidine	Nucleotide	2	Ctrl_H2O2_2_3	Ctrl_H2O2_2	0.75	Pyrimidine nucleosides	True	1.875e+05	0.984	0.984	1.569
41	Lactate	Carbon	2	Ctrl_H2O2_2_3	Ctrl_H2O2_2	1.0	Respiratory carbon sources	True	2.814e+07	8.213	8.213	0.3914

Metabolite ID	Name	Super Pathway	Dataset	Sample ID	Group ID	Detection Fraction	Pathway	Detected	Raw Intensity	Log2 Norm Intensity	Norm Imputed	Log2 Ctrl Norm
93	3-P-Ser	Amino acid	2	Ctrl_H2O2_2_3	Ctrl_H2O2_2	0.5	Amino acids biosynthesis intermediates	False			-4.967	-1.709
343	Adenine	Nucleotide	2	Ctrl_H2O2_2_3	Ctrl_H2O2_2	0.5	Purine bases	True	1.555e+04	-2.608	-2.608	-1.144
336	Adenosine	Nucleotide	2	Ctrl_H2O2_2_3	Ctrl_H2O2_2	1.0	Purine nucleosides	True	4.384e+05	2.209	2.209	-0.9282
29	Raffinose	Carbon	2	Ctrl_H2O2_2_3	Ctrl_H2O2_2	0.5	Sugars and sugar alcohols	False			-5.058	-2.878
717	Nicotinamide	Cofactor	2	Ctrl_H2O2_2_3	Ctrl_H2O2_2	1.0	NAD biosynthesis	True	7.874e+05	3.054	3.054	0.6574
51	PEP	Carbon	2	Ctrl_H2O2_2_3	Ctrl_H2O2_2	1.0	Glycolysis, GNG	True	7.5e+04	-0.338	-0.338	0.7339
52	Pyruvate	Carbon	2	Ctrl_H2O2_2_3	Ctrl_H2O2_2	0.25	Glycolysis, GNG	False			-3.589	-1.215
237	Spermine	Amino acid	2	Ctrl_H2O2_2_3	Ctrl_H2O2_2	0.75	Polyamines	True	1.775e+05	0.9044	0.9044	-2.484
385	Uracil	Nucleotide	2	Ctrl_H2O2_2_3	Ctrl_H2O2_2	1.0	Pyrimidine bases	True	5.013e+04	-0.9194	-0.9194	0.8075
377	Uridine	Nucleotide	2	Ctrl_H2O2_2_3	Ctrl_H2O2_2	1.0	Pyrimidine nucleosides	True	4.026e+05	2.086	2.086	-0.7961
112	trans-Urocanate	Amino acid	2	Ctrl_H2O2_2_3	Ctrl_H2O2_2	0.75	Amino acids degradation intermediates	True	2.298e+05	1.278	1.278	4.395
737	Pyridoxine (Vitamin B6)	Cofactor	2	Ctrl_H2O2_2_3	Ctrl_H2O2_2	1.0	PLP biosynthesis and salvage	True	1.377e+06	3.86	3.86	0.5605
348	Allantoin	Nucleotide	2	Ctrl_H2O2_2_3	Ctrl_H2O2_2	1.0	Purine degradation	True	3.595e+04	-1.399	-1.399	1.431
335	Inosine	Nucleotide	2	Ctrl_H2O2_2_3	Ctrl_H2O2_2	1.0	Purine nucleosides	True	1.401e+05	0.5631	0.5631	-2.298
81	Ile	Amino acid	2	Ctrl_H2O2_2_3	Ctrl_H2O2_2	1.0	Proteinogenic amino acids	True	2.082e+07	7.779	7.779	-0.07662
72	Ala	Amino acid	2	Ctrl_H2O2_2_3	Ctrl_H2O2_2	1.0	Proteinogenic amino acids	True	8.376e+06	6.465	6.465	-1.269
79	Thr	Amino acid	2	Ctrl_H2O2_2_3	Ctrl_H2O2_2	1.0	Proteinogenic amino acids	True	1.872e+06	4.304	4.304	-1.659
88	Tyr	Amino acid	2	Ctrl_H2O2_2_3	Ctrl_H2O2_2	1.0	Proteinogenic amino acids	True	8.358e+06	6.462	6.462	-0.3805
84	Lys	Amino acid	2	Ctrl_H2O2_2_3	Ctrl_H2O2_2	1.0	Proteinogenic amino acids	True	1.385e+06	3.869	3.869	0.15
86	Met	Amino acid	2	Ctrl_H2O2_2_3	Ctrl_H2O2_2	1.0	Proteinogenic amino acids	True	3.808e+06	5.328	5.328	-0.4592
61	Malate	Carbon	2	Ctrl_H2O2_2_3	Ctrl_H2O2_2	1.0	TCA cycle	True	2.093e+05	1.142	1.142	0.2457
235	Putrescine	Amino acid	2	Ctrl_H2O2_2_3	Ctrl_H2O2_2	0.5	Polyamines	True	8906	-3.412	-3.412	-1.845
49	3-P-Glycerate	Carbon	2	Ctrl_H2O2_2_3	Ctrl_H2O2_2	1.0	Glycolysis, GNG	True	6.354e+05	2.745	2.745	0.282
139	GABA	Amino acid	2	Ctrl_H2O2_2_3	Ctrl_H2O2_2	0.25	Amino acid derivatives	False			-4.963	-1.493

Metabolite ID	Name	Super Pathway	Dataset	Sample ID	Group ID	Detection Fraction	Pathway	Detected	Raw Intensity	Log2 Norm Intensity	Norm Imputed	Log2 Ctrl Norm
189	Kynurenate	Amino acid	2	Ctrl_H2O2_2_3	Ctrl_H2O2_2	0.5	Amino acid derivatives	False			-5.188	0
234	5-Me-Thioadenosine	Amino acid	2	Ctrl_H2O2_2_3	Ctrl_H2O2_2	1.0	SAM metabolism	True	4.223e+04	-1.167	-1.167	-1.071
59	Succinate	Carbon	2	Ctrl_H2O2_2_3	Ctrl_H2O2_2	0.75	TCA cycle	False			-2.963	-0.9227
133	Ornithine	Amino acid	2	Ctrl_H2O2_2_3	Ctrl_H2O2_2	1.0	Amino acids degradation intermediates	True	4.984e+05	2.394	2.394	-0.127
313	5-Oxoproline	Amino acid	2	Ctrl_H2O2_2_3	Ctrl_H2O2_2	1.0	Glutathione derivatives	True	1.373e+06	3.856	3.856	1.853
724	Pantothenate	Cofactor	2	Ctrl_H2O2_2_3	Ctrl_H2O2_2	1.0	Coenzyme A biosynthesis	True	9.769e+05	3.365	3.365	0.2517
30	Sucrose	Carbon	2	Ctrl_H2O2_2_3	Ctrl_H2O2_2	1.0	Sugars and sugar alcohols	True	2.526e+05	1.414	1.414	-0.9631
122	3-OH-Isobutyrate	Amino acid	2	Ctrl_H2O2_2_3	Ctrl_H2O2_2	0.0	Amino acids degradation intermediates	False			-4.628	0
241	4-Acetamidobutanoate	Amino acid	2	Ctrl_H2O2_2_3	Ctrl_H2O2_2	1.0	Polyamine derivatives	True	1.047e+05	0.1427	0.1427	1.65
55	Citrate	Carbon	2	Ctrl_H2O2_2_3	Ctrl_H2O2_2	1.0	TCA cycle	True	9.776e+05	3.366	3.366	0.3318
338	Guanosine	Nucleotide	2	Ctrl_H2O2_2_3	Ctrl_H2O2_2	1.0	Purine nucleosides	True	2.154e+05	1.184	1.184	-1.177
170	2-Amino-Butyrate	Amino acid	2	Ctrl_H2O2_2_3	Ctrl_H2O2_2	1.0	Amino acid derivatives	True	3.553e+05	1.906	1.906	0.6822
209	N-Ac-Ala	Amino acid	2	Ctrl_H2O2_2_3	Ctrl_H2O2_2	0.0	N-acetylated amino acids	False			-4.401	-1.133
221	N-Ac-Met	Amino acid	2	Ctrl_H2O2_2_3	Ctrl_H2O2_2	1.0	N-acetylated amino acids	True	1.979e+04	-2.26	-2.26	-1.11
22	N-Ac-Neuraminate	Carbon	2	Ctrl_H2O2_2_3	Ctrl_H2O2_2	0.5	Aminosugar derivatives	True	4.743e+04	-0.9991	-0.9991	-0.7851
346	Urate	Nucleotide	2	Ctrl_H2O2_2_3	Ctrl_H2O2_2	0.5	Purine degradation	True	8148	-3.54	-3.54	0.1517
90	Arg	Amino acid	2	Ctrl_H2O2_2_3	Ctrl_H2O2_2	1.0	Proteinogenic amino acids	True	2.318e+06	4.612	4.612	-0.1701
60	Fumarate	Carbon	2	Ctrl_H2O2_2_3	Ctrl_H2O2_2	1.0	TCA cycle	True	8.798e+04	-0.1077	-0.1077	0.049
78	Ser	Amino acid	2	Ctrl_H2O2_2_3	Ctrl_H2O2_2	1.0	Proteinogenic amino acids	True	3.407e+06	5.167	5.167	-1.659
83	Val	Amino acid	2	Ctrl_H2O2_2_3	Ctrl_H2O2_2	1.0	Proteinogenic amino acids	True	1.237e+07	7.028	7.028	-0.2058
734	Pyridoxal	Cofactor	2	Ctrl_H2O2_2_3	Ctrl_H2O2_2	0.5	PLP biosynthesis and salvage	False			-1.437	-0.4421
136	Urea	Amino acid	2	Ctrl_H2O2_2_3	Ctrl_H2O2_2	1.0	Amino acids degradation intermediates	True	8.916e+05	3.233	3.233	1.588

Metabolite ID	Name	Super Pathway	Dataset	Sample ID	Group ID	Detection Fraction	Pathway	Detected	Raw Intensity	Log2 Norm Intensity	Norm Imputed	Log2 Ctrl Norm
742	Folate	Cofactor	2	Ctrl_H2O2_2_3	Ctrl_H2O2_2	1.0	Folate metabolism	True	1.216e+05	0.3591	0.3591	0.8761
729	Riboflavin (Vitamin B2)	Cofactor	2	Ctrl_H2O2_2_3	Ctrl_H2O2_2	1.0	Flavine biosynthesis	True	3.094e+04	-1.615	-1.615	-0.04631
91	Pro	Amino acid	2	Ctrl_H2O2_2_3	Ctrl_H2O2_2	1.0	Proteinogenic amino acids	True	3.502e+06	5.207	5.207	-1.514
308	Glutathione, Reduced	Amino acid	2	Ctrl_H2O2_2_3	Ctrl_H2O2_2	1.0	Glutathione	True	2.48e+06	4.709	4.709	-1.746
706	FAD	Cofactor	2	Ctrl_H2O2_2_3	Ctrl_H2O2_2	0.0	Cofactors	False			-5.096	0
299	gamma-Glu-Tyr	Amino acid	2	Ctrl_H2O2_2_3	Ctrl_H2O2_2	0.5	Gamma-glutamyl dipeptides	False			-3.049	-1.881
705	Coenzyme A	Cofactor	2	Ctrl_H2O2_2_3	Ctrl_H2O2_2	0.75	Cofactors	True	2751	-5.107	-5.107	-1.401
342	Hypoxanthine	Nucleotide	2	Ctrl_H2O2_2_3	Ctrl_H2O2_2	1.0	Purine bases	True	7.923e+04	-0.2589	-0.2589	-0.2608
344	Xanthine	Nucleotide	2	Ctrl_H2O2_2_3	Ctrl_H2O2_2	0.75	Purine bases	True	1.659e+04	-2.514	-2.514	-0.2753
703	NAD+	Cofactor	2	Ctrl_H2O2_2_3	Ctrl_H2O2_2	1.0	Cofactors	True	1.354e+05	0.5137	0.5137	-3.045
731	Thiamin (Vitamin B1)	Cofactor	2	Ctrl_H2O2_2_3	Ctrl_H2O2_2	1.0	TPP biosynthesis	True	9.53e+04	7.53e-03	7.53e-03	0.2137
102	2-Aminoadipate	Amino acid	2	Ctrl_H2O2_2_3	Ctrl_H2O2_2	1.0	Amino acids biosynthesis intermediates	True	2.276e+04	-2.059	-2.059	-2.815
77	Gly	Amino acid	2	Ctrl_H2O2_2_3	Ctrl_H2O2_2	1.0	Proteinogenic amino acids	True	3.858e+06	5.347	5.347	-1.881
45	Fructose-6-P	Carbon	2	Ctrl_H2O2_2_3	Ctrl_H2O2_2	0.75	Glycolysis, GNG	True	3.854e+04	-1.299	-1.299	0.1367
36	Ribose	Carbon	2	Ctrl_H2O2_2_3	Ctrl_H2O2_2	0.75	Sugars and sugar alcohols	True	2.15e+04	-2.141	-2.141	-1.143
4	GlcNAc 6-P	Carbon	2	Ctrl_H2O2_2_3	Ctrl_H2O2_2	1.0	Aminosugar biosynthesis	True	3.601e+04	-1.397	-1.397	-1.756
188	Kynurenine	Amino acid	2	Ctrl_H2O2_2_3	Ctrl_H2O2_2	0.75	Amino acid derivatives	True	3.097e+04	-1.614	-1.614	-1.6
63	6-P-Gluconate	Carbon	2	Ctrl_H2O2_2_3	Ctrl_H2O2_2	1.0	Pentose phosphate pathway (PPP)	True	4.624e+04	-1.036	-1.036	9.49e-03
710	Carnitine	Cofactor	2	Ctrl_H2O2_2_3	Ctrl_H2O2_2	1.0	Cofactors	True	1.873e+05	0.9822	0.9822	-1.55
725	P-Pantetheine	Cofactor	2	Ctrl_H2O2_2_3	Ctrl_H2O2_2	0.75	Coenzyme A biosynthesis	True	7120	-3.735	-3.735	1.077
110	N-alpha-Ac-Ornithine	Amino acid	2	Ctrl_H2O2_2_3	Ctrl_H2O2_2	1.0	Amino acids biosynthesis intermediates	True	1.656e+05	0.8047	0.8047	-0.1693
116	3-Me-2-Oxo-Valerate	Amino acid	2	Ctrl_H2O2_2_3	Ctrl_H2O2_2	1.0	Amino acids degradation intermediates	True	2.37e+04	-2	-2	1.574
155	4-Guanidinobutanoate	Amino acid	2	Ctrl_H2O2_2_3	Ctrl_H2O2_2	0.5	Amino acid derivatives	False			-2.324	-1.604

Metabolite ID	Name	Super Pathway	Dataset	Sample ID	Group ID	Detection Fraction	Pathway	Detected	Raw Intensity	Log2 Norm Intensity	Norm Imputed	Log2 Ctrl Norm
310	S-Lactoyl-Glutathione	Amino acid	2	Ctrl_H2O2_2_3	Ctrl_H2O2_2	0.0	Glutathione derivatives	False			-4.634	-0.962
34	Ribitol	Carbon	2	Ctrl_H2O2_2_3	Ctrl_H2O2_2	0.75	Sugars and sugar alcohols	True	3.099e+04	-1.613	-1.613	-0.0346
707	FMN	Cofactor	2	Ctrl_H2O2_2_3	Ctrl_H2O2_2	0.5	Cofactors	True	4020	-4.56	-4.56	-1.139
17	Maltose	Carbon	2	Ctrl_H2O2_2_3	Ctrl_H2O2_2	1.0	Glycogen degradation	True	2.54e+05	1.422	1.422	1.461
18	Maltotriose	Carbon	2	Ctrl_H2O2_2_3	Ctrl_H2O2_2	1.0	Glycogen degradation	True	7.311e+05	2.947	2.947	1.518
19	Maltotetraose	Carbon	2	Ctrl_H2O2_2_3	Ctrl_H2O2_2	1.0	Glycogen degradation	True	1.533e+05	0.6936	0.6936	0.884
232	SAH	Amino acid	2	Ctrl_H2O2_2_3	Ctrl_H2O2_2	1.0	SAM metabolism	True	1.223e+04	-2.955	-2.955	0.2715
74	Asp	Amino acid	2	Ctrl_H2O2_2_3	Ctrl_H2O2_2	1.0	Proteinogenic amino acids	True	1.142e+06	3.59	3.59	-2.069
129	5-Aminovalerate	Amino acid	2	Ctrl_H2O2_2_3	Ctrl_H2O2_2	0.0	Amino acids degradation intermediates	False			-1.567	-0.7538
254	Gly-Val	Amino acid	2	Ctrl_H2O2_2_3	Ctrl_H2O2_2	0.75	Dipeptides	False			-1.33	-1.256
291	gamma-Glu-Leu	Amino acid	2	Ctrl_H2O2_2_3	Ctrl_H2O2_2	0.5	Gamma-glutamyl dipeptides	True	3.208e+04	-1.563	-1.563	-0.4815
173	Met Sulfoxide	Amino acid	2	Ctrl_H2O2_2_3	Ctrl_H2O2_2	1.0	Amino acid derivatives	True	2.099e+05	1.146	1.146	1.285
43	Glucose	Carbon	2	Ctrl_H2O2_2_3	Ctrl_H2O2_2	1.0	Glycolysis, GNG	True	1.717e+07	7.5	7.5	0.6808
249	Gly-Gly	Amino acid	2	Ctrl_H2O2_2_3	Ctrl_H2O2_2	0.0	Dipeptides	False			-1.272	-1.489
169	2-OH-Butyrate	Amino acid	2	Ctrl_H2O2_2_3	Ctrl_H2O2_2	0.25	Amino acid derivatives	False			-1.729	-0.2955
98	3-Methyl-2-Oxobutyrate	Amino acid	2	Ctrl_H2O2_2_3	Ctrl_H2O2_2	0.25	Amino acids biosynthesis intermediates	False			-3.759	0
100	4-Me-2-Oxo-Pentanoate	Amino acid	2	Ctrl_H2O2_2_3	Ctrl_H2O2_2	1.0	Amino acids biosynthesis intermediates	True	2.115e+04	-2.164	-2.164	1.138
253	Gly-Pro	Amino acid	2	Ctrl_H2O2_2_3	Ctrl_H2O2_2	0.75	Dipeptides	True	5.231e+04	-0.8578	-0.8578	-0.5368
247	Asp-Phe	Amino acid	2	Ctrl_H2O2_2_3	Ctrl_H2O2_2	1.0	Dipeptides	True	2.348e+04	-2.014	-2.014	-1.072
212	N-Ac-Asp	Amino acid	2	Ctrl_H2O2_2_3	Ctrl_H2O2_2	0.0	N-acetylated amino acids	False			-4.689	-0.1861
720	1-Me-Nicotinamide	Cofactor	2	Ctrl_H2O2_2_3	Ctrl_H2O2_2	1.0	Derivatives of NA, nicotinamide and NAD	True	2.989e+05	1.656	1.656	-2.047
70	Creatine	Carbon	2	Ctrl_H2O2_2_3	Ctrl_H2O2_2	1.0	Creatine energy storage	True	3.293e+06	5.118	5.118	-0.6619

Metabolite ID	Name	Super Pathway	Dataset	Sample ID	Group ID	Detection Fraction	Pathway	Detected	Raw Intensity	Log2 Norm Intensity	Norm Imputed	Log2 Ctrl Norm
309	Glutathione, Oxidized	Amino acid	2	Ctrl_H2O2_2_3	Ctrl_H2O2_2	1.0	Glutathione	True	1.46e+06	3.945	3.945	-3.45e-03
44	Glucose 6-P	Carbon	2	Ctrl_H2O2_2_3	Ctrl_H2O2_2	1.0	Glycolysis, GNG	True	1.707e+05	0.8481	0.8481	0.03335
24	Fructose	Carbon	2	Ctrl_H2O2_2_3	Ctrl_H2O2_2	1.0	Sugars and sugar alcohols	True	2.016e+06	4.411	4.411	1.05
85	Cys	Amino acid	2	Ctrl_H2O2_2_3	Ctrl_H2O2_2	1.0	Proteinogenic amino acids	True	3.038e+04	-1.642	-1.642	-1.05
704	NADH	Cofactor	2	Ctrl_H2O2_2_3	Ctrl_H2O2_2	0.0	Cofactors	False			-4.313	-2.383
275	Thr-Phe	Amino acid	2	Ctrl_H2O2_2_3	Ctrl_H2O2_2	0.0	Dipeptides	False			-1.922	-0.9888
738	Pyridoxate	Cofactor	2	Ctrl_H2O2_2_3	Ctrl_H2O2_2	0.75	PLP biosynthesis and salvage	True	1.217e+04	-2.961	-2.961	1.704
177	3-(4-OH-Phenyl)Lactate	Amino acid	2	Ctrl_H2O2_2_3	Ctrl_H2O2_2	0.5	Amino acid derivatives	True	7655	-3.63	-3.63	0.3226
206	Trans-4-OH-Pro	Amino acid	2	Ctrl_H2O2_2_3	Ctrl_H2O2_2	1.0	Amino acid derivatives	True	1.811e+05	0.9339	0.9339	1.13
329	AMP	Nucleotide	2	Ctrl_H2O2_2_3	Ctrl_H2O2_2	0.25	Purine nucleotides	False			-2.644	-3.064
345	Guanine	Nucleotide	2	Ctrl_H2O2_2_3	Ctrl_H2O2_2	1.0	Purine bases	True	1.092e+06	3.525	3.525	1.943
271	pyroGlu-Val	Amino acid	2	Ctrl_H2O2_2_3	Ctrl_H2O2_2	0.75	Dipeptides	False			-5.212	-1.57
279	Val-Glu	Amino acid	2	Ctrl_H2O2_2_3	Ctrl_H2O2_2	1.0	Dipeptides	True	2.614e+04	-1.859	-1.859	-1.029
183	Phenol Sulfate	Amino acid	2	Ctrl_H2O2_2_3	Ctrl_H2O2_2	0.25	Amino acid derivatives	False			-4.072	-0.3451
740	3-Dehydrocarnitine	Cofactor	2	Ctrl_H2O2_2_3	Ctrl_H2O2_2	0.5	Carnitine biosynthesis	True	5.846e+04	-0.6974	-0.6974	-0.3638
145	Pyro-Gln	Amino acid	2	Ctrl_H2O2_2_3	Ctrl_H2O2_2	1.0	Amino acid derivatives	True	1.343e+05	0.5025	0.5025	0.1565
197	C-Glycosyl-Trp	Amino acid	2	Ctrl_H2O2_2_3	Ctrl_H2O2_2	1.0	Amino acid derivatives	True	1.924e+04	-2.301	-2.301	-2.157
718	Nicotinamide Riboside	Cofactor	2	Ctrl_H2O2_2_3	Ctrl_H2O2_2	1.0	NAD biosynthesis	True	2.325e+05	1.294	1.294	3.098
295	gamma-Glu-Phe	Amino acid	2	Ctrl_H2O2_2_3	Ctrl_H2O2_2	0.25	Gamma-glutamyl dipeptides	False			-1.936	-1.834
399	Pseudouridine	Nucleotide	2	Ctrl_H2O2_2_3	Ctrl_H2O2_2	0.5	Pyrimidine derivatives in RNAs	True	9648	-3.297	-3.297	0.9441
375	UTP	Nucleotide	2	Ctrl_H2O2_2_3	Ctrl_H2O2_2	0.0	Pyrimidine nucleotides	False			-5.966	-0.5235
20	Erythronate	Carbon	2	Ctrl_H2O2_2_3	Ctrl_H2O2_2	0.75	Aminosugar derivatives	True	4.516e+04	-1.07	-1.07	0.3715
151	Phenylacetylglycine	Amino acid	2	Ctrl_H2O2_2_3	Ctrl_H2O2_2	1.0	Amino acid derivatives	True	2.036e+04	-2.219	-2.219	2.202
252	Gly-Phe	Amino acid	2	Ctrl_H2O2_2_3	Ctrl_H2O2_2	0.75	Dipeptides	True	1.313e+05	0.4695	0.4695	0.3654

Metabolite ID	Name	Super Pathway	Dataset	Sample ID	Group ID	Detection Fraction	Pathway	Detected	Raw Intensity	Log2 Norm Intensity	Norm Imputed	Log2 Ctrl Norm
251	Gly-Leu	Amino acid	2	Ctrl_H2O2_2_3	Ctrl_H2O2_2	1.0	Dipeptides	True	7.511e+04	-0.3359	-0.3359	-0.6985
290	gamma-Glu-Ile	Amino acid	2	Ctrl_H2O2_2_3	Ctrl_H2O2_2	0.5	Gamma-glutamyl dipeptides	True	3.443e+04	-1.461	-1.461	0.06987
316	Ophthalmate	Amino acid	2	Ctrl_H2O2_2_3	Ctrl_H2O2_2	1.0	Oxidative stress markers	True	1.205e+05	0.3465	0.3465	0.09226
208	Pro-OH-Pro	Amino acid	2	Ctrl_H2O2_2_3	Ctrl_H2O2_2	1.0	Amino acid derivatives	True	1.243e+05	0.3909	0.3909	-0.06869
352	3'-AMP	Nucleotide	2	Ctrl_H2O2_2_3	Ctrl_H2O2_2	0.75	Purine derivatives in signaling	True	2.896e+04	-1.711	-1.711	-0.4262
314	Cys-Glutathione Disulfide	Amino acid	2	Ctrl_H2O2_2_3	Ctrl_H2O2_2	1.0	Oxidative stress markers	True	9.431e+04	-7.53e-03	-7.53e-03	1.358
39	Threitol	Carbon	2	Ctrl_H2O2_2_3	Ctrl_H2O2_2	0.25	Sugars and sugar alcohols	False			-4.395	-0.3698
31	Ribulose/Xylulose	Carbon	2	Ctrl_H2O2_2_3	Ctrl_H2O2_2	0.0	Sugars and sugar alcohols	False			-5.004	-0.09716
48	DHAP	Carbon	2	Ctrl_H2O2_2_3	Ctrl_H2O2_2	1.0	Glycolysis, GNG	True	6.634e+04	-0.5152	-0.5152	-2.273
182	P-Cresol Sulfate	Amino acid	2	Ctrl_H2O2_2_3	Ctrl_H2O2_2	1.0	Amino acid derivatives	True	6268	-3.919	-3.919	-0.5815
250	Gly-Ile	Amino acid	2	Ctrl_H2O2_2_3	Ctrl_H2O2_2	0.25	Dipeptides	False			-2.655	-1.231
286	gamma-Glu-Glu	Amino acid	2	Ctrl_H2O2_2_3	Ctrl_H2O2_2	1.0	Gamma-glutamyl dipeptides	True	5.844e+04	-0.698	-0.698	-0.4674
264	Leu-Leu	Amino acid	2	Ctrl_H2O2_2_3	Ctrl_H2O2_2	0.25	Dipeptides	False			-2.222	-1.148
203	DiMe-Arg	Amino acid	2	Ctrl_H2O2_2_3	Ctrl_H2O2_2	1.0	Amino acid derivatives	True	4.959e+05	2.387	2.387	1.313
47	Fructose 1,6-PP, Glucose 1,6-PP	Carbon	2	Ctrl_H2O2_2_3	Ctrl_H2O2_2	1.0	Glycolysis, GNG	True	1.972e+04	-2.266	-2.266	-1.975
224	N-Ac-Ser	Amino acid	2	Ctrl_H2O2_2_3	Ctrl_H2O2_2	0.5	N-acetylated amino acids	False			-1.752	-2.623
244	Ala-Leu	Amino acid	2	Ctrl_H2O2_2_3	Ctrl_H2O2_2	0.25	Dipeptides	True	2.765e+04	-1.778	-1.778	-2.118
304	Cyclo(Phe-Pro)	Amino acid	2	Ctrl_H2O2_2_3	Ctrl_H2O2_2	0.25	Cyclic dipeptides	False			-1.192	-0.7201
302	Cyclo(Glu-Glu)	Amino acid	2	Ctrl_H2O2_2_3	Ctrl_H2O2_2	1.0	Cyclic dipeptides	True	3.697e+04	-1.359	-1.359	-0.1865
303	Cyclo(Leu-Pro)	Amino acid	2	Ctrl_H2O2_2_3	Ctrl_H2O2_2	0.5	Cyclic dipeptides	False			-0.4297	-0.7026
390	2',3'-cUMP	Nucleotide	2	Ctrl_H2O2_2_3	Ctrl_H2O2_2	1.0	Pyrimidine derivatives in signaling	True	3.253e+04	-1.543	-1.543	-0.4145
68	Ribulose 5-P / Xylulose 5-P	Carbon	2	Ctrl_H2O2_2_3	Ctrl_H2O2_2	1.0	Pentose phosphate pathway (PPP)	True	5.261e+04	-0.8496	-0.8496	-1.619
388	2',3'-cCMP	Nucleotide	2	Ctrl_H2O2_2_3	Ctrl_H2O2_2	1.0	Pyrimidine derivatives in signaling	True	4.755e+04	-0.9956	-0.9956	-1.249

Metabolite ID	Name	Super Pathway	Dataset	Sample ID	Group ID	Detection Fraction	Pathway	Detected	Raw Intensity	Log2 Norm Intensity	Norm Imputed	Log2 Ctrl Norm
33	Arabitol/Xylitol	Carbon	2	Ctrl_H2O2_2_3	Ctrl_H2O2_2	0.0	Sugars and sugar alcohols	False			-3.559	-0.05408
268	Phe-Phe	Amino acid	2	Ctrl_H2O2_2_3	Ctrl_H2O2_2	0.5	Dipeptides	False			-2.762	-1.674
245	Ala-Phe	Amino acid	2	Ctrl_H2O2_2_3	Ctrl_H2O2_2	0.0	Dipeptides	False			-2.821	-1.366
373	UMP	Nucleotide	2	Ctrl_H2O2_2_3	Ctrl_H2O2_2	0.25	Pyrimidine nucleotides	False			-3.442	-1.402
282	Val-Leu	Amino acid	2	Ctrl_H2O2_2_3	Ctrl_H2O2_2	0.75	Dipeptides	True	4.254e+04	-1.156	-1.156	-0.7875
258	Ile-Gly	Amino acid	2	Ctrl_H2O2_2_3	Ctrl_H2O2_2	0.75	Dipeptides	True	2.1e+04	-2.175	-2.175	-2.589
259	Ile-Ser	Amino acid	2	Ctrl_H2O2_2_3	Ctrl_H2O2_2	0.25	Dipeptides	False			-3.192	-2.329
269	Phe-Ser	Amino acid	2	Ctrl_H2O2_2_3	Ctrl_H2O2_2	0.5	Dipeptides	False			-3.197	-1.882
277	Tyr-Ala	Amino acid	2	Ctrl_H2O2_2_3	Ctrl_H2O2_2	0.75	Dipeptides	True	3.156e+04	-1.587	-1.587	-2.157
257	Ile-Gln	Amino acid	2	Ctrl_H2O2_2_3	Ctrl_H2O2_2	0.25	Dipeptides	True	1.537e+04	-2.625	-2.625	-1.706
261	Leu-Glu	Amino acid	2	Ctrl_H2O2_2_3	Ctrl_H2O2_2	0.75	Dipeptides	True	2.894e+04	-1.712	-1.712	-2.502
263	Leu-Gly	Amino acid	2	Ctrl_H2O2_2_3	Ctrl_H2O2_2	1.0	Dipeptides	True	3.514e+04	-1.432	-1.432	-2.139
256	Ile-Ala	Amino acid	2	Ctrl_H2O2_2_3	Ctrl_H2O2_2	0.75	Dipeptides	True	2.043e+04	-2.214	-2.214	-2.173
274	Thr-Leu	Amino acid	2	Ctrl_H2O2_2_3	Ctrl_H2O2_2	0.25	Dipeptides	False			0.2082	-0.6429
273	Ser-Phe	Amino acid	2	Ctrl_H2O2_2_3	Ctrl_H2O2_2	1.0	Dipeptides	True	2.2e+04	-2.108	-2.108	-0.8857
272	Ser-Leu	Amino acid	2	Ctrl_H2O2_2_3	Ctrl_H2O2_2	1.0	Dipeptides	True	4.601e+04	-1.043	-1.043	-1.782
246	Asp-Leu	Amino acid	2	Ctrl_H2O2_2_3	Ctrl_H2O2_2	1.0	Dipeptides	True	4.712e+04	-1.009	-1.009	-0.9345
76	Gln	Amino acid	2	Ctrl_H2O2_2_4	Ctrl_H2O2_2	1.0	Proteinogenic amino acids	True	1.788e+07	7.581	7.581	0.6287
89	Trp	Amino acid	2	Ctrl_H2O2_2_4	Ctrl_H2O2_2	1.0	Proteinogenic amino acids	True	4.352e+06	5.542	5.542	-0.4243
723	beta-Ala	Cofactor	2	Ctrl_H2O2_2_4	Ctrl_H2O2_2	0.75	Coenzyme A biosynthesis	True	3.3e+04	-1.501	-1.501	-1.827
75	Glu	Amino acid	2	Ctrl_H2O2_2_4	Ctrl_H2O2_2	1.0	Proteinogenic amino acids	True	4.609e+06	5.625	5.625	-0.323
80	His	Amino acid	2	Ctrl_H2O2_2_4	Ctrl_H2O2_2	1.0	Proteinogenic amino acids	True	9.339e+04	0	0	-0.2141
82	Leu	Amino acid	2	Ctrl_H2O2_2_4	Ctrl_H2O2_2	1.0	Proteinogenic amino acids	True	2.187e+07	7.871	7.871	-0.2888
87	Phe	Amino acid	2	Ctrl_H2O2_2_4	Ctrl_H2O2_2	1.0	Proteinogenic amino acids	True	1.762e+07	7.56	7.56	-0.3318
236	Spermidine	Amino acid	2	Ctrl_H2O2_2_4	Ctrl_H2O2_2	1.0	Polyamines	True	2.859e+05	1.614	1.614	-1.214
73	Asn	Amino acid	2	Ctrl_H2O2_2_4	Ctrl_H2O2_2	1.0	Proteinogenic amino acids	True	1.164e+05	0.318	0.318	-2.431

Metabolite ID	Name	Super Pathway	Dataset	Sample ID	Group ID	Detection Fraction	Pathway	Detected	Raw Intensity	Log2 Norm Intensity	Norm Imputed	Log2 Ctrl Norm
243	Creatinine	Amino acid	2	Ctrl_H2O2_2_4	Ctrl_H2O2_2	0.75	Creatine degradation	False			1.309	-0.2613
376	Cytidine	Nucleotide	2	Ctrl_H2O2_2_4	Ctrl_H2O2_2	0.75	Pyrimidine nucleosides	True	9.572e+04	0.03549	0.03549	0.6201
41	Lactate	Carbon	2	Ctrl_H2O2_2_4	Ctrl_H2O2_2	1.0	Respiratory carbon sources	True	1.276e+07	7.094	7.094	-0.7277
93	3-P-Ser	Amino acid	2	Ctrl_H2O2_2_4	Ctrl_H2O2_2	0.5	Amino acids biosynthesis intermediates	True	6.255e+04	-0.5782	-0.5782	2.681
343	Adenine	Nucleotide	2	Ctrl_H2O2_2_4	Ctrl_H2O2_2	0.5	Purine bases	False			-3.45	-1.986
336	Adenosine	Nucleotide	2	Ctrl_H2O2_2_4	Ctrl_H2O2_2	1.0	Purine nucleosides	True	2.436e+05	1.383	1.383	-1.754
29	Raffinose	Carbon	2	Ctrl_H2O2_2_4	Ctrl_H2O2_2	0.5	Sugars and sugar alcohols	True	1.261e+04	-2.888	-2.888	-0.7085
717	Nicotinamide	Cofactor	2	Ctrl_H2O2_2_4	Ctrl_H2O2_2	1.0	NAD biosynthesis	True	4.994e+05	2.419	2.419	0.02195
51	PEP	Carbon	2	Ctrl_H2O2_2_4	Ctrl_H2O2_2	1.0	Glycolysis, GNG	True	5.497e+04	-0.7646	-0.7646	0.3074
52	Pyruvate	Carbon	2	Ctrl_H2O2_2_4	Ctrl_H2O2_2	0.25	Glycolysis, GNG	True	8099	-3.527	-3.527	-1.154
237	Spermine	Amino acid	2	Ctrl_H2O2_2_4	Ctrl_H2O2_2	0.75	Polyamines	True	4.153e+05	2.153	2.153	-1.236
385	Uracil	Nucleotide	2	Ctrl_H2O2_2_4	Ctrl_H2O2_2	1.0	Pyrimidine bases	True	2.009e+04	-2.217	-2.217	-0.4896
377	Uridine	Nucleotide	2	Ctrl_H2O2_2_4	Ctrl_H2O2_2	1.0	Pyrimidine nucleosides	True	3.573e+05	1.936	1.936	-0.9467
112	trans-Urocanate	Amino acid	2	Ctrl_H2O2_2_4	Ctrl_H2O2_2	0.75	Amino acids degradation intermediates	True	6.28e+04	-0.5725	-0.5725	2.545
737	Pyridoxine (Vitamin B6)	Cofactor	2	Ctrl_H2O2_2_4	Ctrl_H2O2_2	1.0	PLP biosynthesis and salvage	True	1.898e+06	4.345	4.345	1.045
348	Allantoin	Nucleotide	2	Ctrl_H2O2_2_4	Ctrl_H2O2_2	1.0	Purine degradation	True	8510	-3.456	-3.456	-0.6267
335	Inosine	Nucleotide	2	Ctrl_H2O2_2_4	Ctrl_H2O2_2	1.0	Purine nucleosides	True	2.097e+05	1.167	1.167	-1.694
81	Ile	Amino acid	2	Ctrl_H2O2_2_4	Ctrl_H2O2_2	1.0	Proteinogenic amino acids	True	2.392e+07	8.001	8.001	0.1453
72	Ala	Amino acid	2	Ctrl_H2O2_2_4	Ctrl_H2O2_2	1.0	Proteinogenic amino acids	True	3.857e+06	5.368	5.368	-2.366
79	Thr	Amino acid	2	Ctrl_H2O2_2_4	Ctrl_H2O2_2	1.0	Proteinogenic amino acids	True	1.441e+06	3.948	3.948	-2.015
88	Tyr	Amino acid	2	Ctrl_H2O2_2_4	Ctrl_H2O2_2	1.0	Proteinogenic amino acids	True	8.388e+06	6.489	6.489	-0.3536
84	Lys	Amino acid	2	Ctrl_H2O2_2_4	Ctrl_H2O2_2	1.0	Proteinogenic amino acids	True	1.445e+06	3.952	3.952	0.2329
86	Met	Amino acid	2	Ctrl_H2O2_2_4	Ctrl_H2O2_2	1.0	Proteinogenic amino acids	True	3.5e+06	5.228	5.228	-0.5594

Metabolite ID	Name	Super Pathway	Dataset	Sample ID	Group ID	Detection Fraction	Pathway	Detected	Raw Intensity	Log2 Norm Intensity	Norm Imputed	Log2 Ctrl Norm
61	Malate	Carbon	2	Ctrl_H2O2_2_4	Ctrl_H2O2_2	1.0	TCA cycle	True	5.676e+04	-0.7185	-0.7185	-1.615
235	Putrescine	Amino acid	2	Ctrl_H2O2_2_4	Ctrl_H2O2_2	0.5	Polyamines	True	1.206e+04	-2.953	-2.953	-1.386
49	3-P-Glycerate	Carbon	2	Ctrl_H2O2_2_4	Ctrl_H2O2_2	1.0	Glycolysis, GNG	True	4.965e+05	2.41	2.41	-0.05227
139	GABA	Amino acid	2	Ctrl_H2O2_2_4	Ctrl_H2O2_2	0.25	Amino acid derivatives	False			-4.963	-1.493
189	Kynurenate	Amino acid	2	Ctrl_H2O2_2_4	Ctrl_H2O2_2	0.5	Amino acid derivatives	True	5827	-4.002	-4.002	1.185
234	5-Me-Thioadenosine	Amino acid	2	Ctrl_H2O2_2_4	Ctrl_H2O2_2	1.0	SAM metabolism	True	2.876e+04	-1.699	-1.699	-1.603
59	Succinate	Carbon	2	Ctrl_H2O2_2_4	Ctrl_H2O2_2	0.75	TCA cycle	True	1.378e+04	-2.761	-2.761	-0.7212
133	Ornithine	Amino acid	2	Ctrl_H2O2_2_4	Ctrl_H2O2_2	1.0	Amino acids degradation intermediates	True	1.403e+05	0.5874	0.5874	-1.934
313	5-Oxoproline	Amino acid	2	Ctrl_H2O2_2_4	Ctrl_H2O2_2	1.0	Glutathione derivatives	True	7.654e+05	3.035	3.035	1.031
724	Pantothenate	Cofactor	2	Ctrl_H2O2_2_4	Ctrl_H2O2_2	1.0	Coenzyme A biosynthesis	True	7.3e+05	2.966	2.966	-0.147
30	Sucrose	Carbon	2	Ctrl_H2O2_2_4	Ctrl_H2O2_2	1.0	Sugars and sugar alcohols	True	4.859e+05	2.379	2.379	2.52e-03
122	3-OH-Isobutyrate	Amino acid	2	Ctrl_H2O2_2_4	Ctrl_H2O2_2	0.0	Amino acids degradation intermediates	False			-4.628	0
241	4-Acetamidobutanoate	Amino acid	2	Ctrl_H2O2_2_4	Ctrl_H2O2_2	1.0	Polyamine derivatives	True	6.917e+04	-0.4332	-0.4332	1.074
55	Citrate	Carbon	2	Ctrl_H2O2_2_4	Ctrl_H2O2_2	1.0	TCA cycle	True	5.625e+05	2.59	2.59	-0.444
338	Guanosine	Nucleotide	2	Ctrl_H2O2_2_4	Ctrl_H2O2_2	1.0	Purine nucleosides	True	2.785e+05	1.576	1.576	-0.7841
170	2-Amino-Butyrate	Amino acid	2	Ctrl_H2O2_2_4	Ctrl_H2O2_2	1.0	Amino acid derivatives	True	2.034e+05	1.123	1.123	-0.1005
209	N-Ac-Ala	Amino acid	2	Ctrl_H2O2_2_4	Ctrl_H2O2_2	0.0	N-acetylated amino acids	False			-4.401	-1.133
221	N-Ac-Met	Amino acid	2	Ctrl_H2O2_2_4	Ctrl_H2O2_2	1.0	N-acetylated amino acids	True	7279	-3.682	-3.682	-2.531
22	N-Ac-Neuraminate	Carbon	2	Ctrl_H2O2_2_4	Ctrl_H2O2_2	0.5	Aminosugar derivatives	False			-1.69	-1.476
346	Urate	Nucleotide	2	Ctrl_H2O2_2_4	Ctrl_H2O2_2	0.5	Purine degradation	False			-4.123	-0.4311
90	Arg	Amino acid	2	Ctrl_H2O2_2_4	Ctrl_H2O2_2	1.0	Proteinogenic amino acids	True	2.619e+06	4.81	4.81	0.02752
60	Fumarate	Carbon	2	Ctrl_H2O2_2_4	Ctrl_H2O2_2	1.0	TCA cycle	True	1.855e+04	-2.332	-2.332	-2.175
78	Ser	Amino acid	2	Ctrl_H2O2_2_4	Ctrl_H2O2_2	1.0	Proteinogenic amino acids	True	2.602e+06	4.8	4.8	-2.027

Metabolite ID	Name	Super Pathway	Dataset	Sample ID	Group ID	Detection Fraction	Pathway	Detected	Raw Intensity	Log2 Norm Intensity	Norm Imputed	Log2 Ctrl Norm
83	Val	Amino acid	2	Ctrl_H2O2_2_4	Ctrl_H2O2_2	1.0	Proteinogenic amino acids	True	1.461e+07	7.29	7.29	0.05562
734	Pyridoxal	Cofactor	2	Ctrl_H2O2_2_4	Ctrl_H2O2_2	0.5	PLP biosynthesis and salvage	True	7.608e+04	-0.2958	-0.2958	0.6988
136	Urea	Amino acid	2	Ctrl_H2O2_2_4	Ctrl_H2O2_2	1.0	Amino acids degradation intermediates	True	2.204e+05	1.239	1.239	-0.4063
742	Folate	Cofactor	2	Ctrl_H2O2_2_4	Ctrl_H2O2_2	1.0	Folate metabolism	True	1.098e+05	0.2341	0.2341	0.7511
729	Riboflavin (Vitamin B2)	Cofactor	2	Ctrl_H2O2_2_4	Ctrl_H2O2_2	1.0	Flavine biosynthesis	True	3.042e+04	-1.618	-1.618	-0.04923
91	Pro	Amino acid	2	Ctrl_H2O2_2_4	Ctrl_H2O2_2	1.0	Proteinogenic amino acids	True	3.655e+06	5.29	5.29	-1.431
308	Glutathione, Reduced	Amino acid	2	Ctrl_H2O2_2_4	Ctrl_H2O2_2	1.0	Glutathione	True	1.618e+06	4.114	4.114	-2.341
706	FAD	Cofactor	2	Ctrl_H2O2_2_4	Ctrl_H2O2_2	0.0	Cofactors	False			-5.096	0
299	gamma-Glu-Tyr	Amino acid	2	Ctrl_H2O2_2_4	Ctrl_H2O2_2	0.5	Gamma-glutamyl dipeptides	True	3.114e+04	-1.584	-1.584	-0.4173
705	Coenzyme A	Cofactor	2	Ctrl_H2O2_2_4	Ctrl_H2O2_2	0.75	Cofactors	True	848.8	-6.782	-6.782	-3.075
342	Hypoxanthine	Nucleotide	2	Ctrl_H2O2_2_4	Ctrl_H2O2_2	1.0	Purine bases	True	4.772e+04	-0.9688	-0.9688	-0.9706
344	Xanthine	Nucleotide	2	Ctrl_H2O2_2_4	Ctrl_H2O2_2	0.75	Purine bases	False			-3.616	-1.376
703	NAD+	Cofactor	2	Ctrl_H2O2_2_4	Ctrl_H2O2_2	1.0	Cofactors	True	2.135e+05	1.193	1.193	-2.366
731	Thiamin (Vitamin B1)	Cofactor	2	Ctrl_H2O2_2_4	Ctrl_H2O2_2	1.0	TPP biosynthesis	True	1.027e+05	0.1372	0.1372	0.3433
102	2-Aminoadipate	Amino acid	2	Ctrl_H2O2_2_4	Ctrl_H2O2_2	1.0	Amino acids biosynthesis intermediates	True	6.598e+04	-0.5014	-0.5014	-1.258
77	Gly	Amino acid	2	Ctrl_H2O2_2_4	Ctrl_H2O2_2	1.0	Proteinogenic amino acids	True	2.766e+06	4.889	4.889	-2.34
45	Fructose-6-P	Carbon	2	Ctrl_H2O2_2_4	Ctrl_H2O2_2	0.75	Glycolysis, GNG	False			-2.517	-1.082
36	Ribose	Carbon	2	Ctrl_H2O2_2_4	Ctrl_H2O2_2	0.75	Sugars and sugar alcohols	False			-3.346	-2.349
4	GlcNAc 6-P	Carbon	2	Ctrl_H2O2_2_4	Ctrl_H2O2_2	1.0	Aminosugar biosynthesis	True	3.179e+04	-1.555	-1.555	-1.914
188	Kynurenine	Amino acid	2	Ctrl_H2O2_2_4	Ctrl_H2O2_2	0.75	Amino acid derivatives	True	1.067e+05	0.1925	0.1925	0.2065
63	6-P-Gluconate	Carbon	2	Ctrl_H2O2_2_4	Ctrl_H2O2_2	1.0	Pentose phosphate pathway (PPP)	True	1.48e+05	0.6642	0.6642	1.709
710	Carnitine	Cofactor	2	Ctrl_H2O2_2_4	Ctrl_H2O2_2	1.0	Cofactors	True	6.214e+04	-0.5878	-0.5878	-3.12
725	P-Pantetheine	Cofactor	2	Ctrl_H2O2_2_4	Ctrl_H2O2_2	0.75	Coenzyme A biosynthesis	False			-5.267	-0.4543

Metabolite ID	Name	Super Pathway	Dataset	Sample ID	Group ID	Detection Fraction	Pathway	Detected	Raw Intensity	Log2 Norm Intensity	Norm Imputed	Log2 Ctrl Norm
110	N-alpha-Ac-Ornithine	Amino acid	2	Ctrl_H2O2_2_4	Ctrl_H2O2_2	1.0	Amino acids biosynthesis intermediates	True	1.32e+05	0.4989	0.4989	-0.4751
116	3-Me-2-Oxo-Valerate	Amino acid	2	Ctrl_H2O2_2_4	Ctrl_H2O2_2	1.0	Amino acids degradation intermediates	True	1.558e+04	-2.583	-2.583	0.9912
155	4-Guanidinobutanoate	Amino acid	2	Ctrl_H2O2_2_4	Ctrl_H2O2_2	0.5	Amino acid derivatives	True	6.444e+04	-0.5354	-0.5354	0.184
310	S-Lactoyl-Glutathione	Amino acid	2	Ctrl_H2O2_2_4	Ctrl_H2O2_2	0.0	Glutathione derivatives	False			-4.634	-0.962
34	Ribitol	Carbon	2	Ctrl_H2O2_2_4	Ctrl_H2O2_2	0.75	Sugars and sugar alcohols	True	1.299e+04	-2.846	-2.846	-1.267
707	FMN	Cofactor	2	Ctrl_H2O2_2_4	Ctrl_H2O2_2	0.5	Cofactors	False			-4.56	-1.139
17	Maltose	Carbon	2	Ctrl_H2O2_2_4	Ctrl_H2O2_2	1.0	Glycogen degradation	True	1.551e+05	0.732	0.732	0.7714
18	Maltotriose	Carbon	2	Ctrl_H2O2_2_4	Ctrl_H2O2_2	1.0	Glycogen degradation	True	3.144e+05	1.751	1.751	0.3222
19	Maltotetraose	Carbon	2	Ctrl_H2O2_2_4	Ctrl_H2O2_2	1.0	Glycogen degradation	True	2.504e+05	1.423	1.423	1.613
232	SAH	Amino acid	2	Ctrl_H2O2_2_4	Ctrl_H2O2_2	1.0	SAM metabolism	True	6493	-3.846	-3.846	-0.6201
74	Asp	Amino acid	2	Ctrl_H2O2_2_4	Ctrl_H2O2_2	1.0	Proteinogenic amino acids	True	1.454e+06	3.961	3.961	-1.698
129	5-Aminovalerate	Amino acid	2	Ctrl_H2O2_2_4	Ctrl_H2O2_2	0.0	Amino acids degradation intermediates	False			-1.567	-0.7538
254	Gly-Val	Amino acid	2	Ctrl_H2O2_2_4	Ctrl_H2O2_2	0.75	Dipeptides	True	4.494e+04	-1.055	-1.055	-0.9817
291	gamma-Glu-Leu	Amino acid	2	Ctrl_H2O2_2_4	Ctrl_H2O2_2	0.5	Gamma-glutamyl dipeptides	False			-2.038	-0.9559
173	Met Sulfoxide	Amino acid	2	Ctrl_H2O2_2_4	Ctrl_H2O2_2	1.0	Amino acid derivatives	True	1.636e+05	0.8088	0.8088	0.9476
43	Glucose	Carbon	2	Ctrl_H2O2_2_4	Ctrl_H2O2_2	1.0	Glycolysis, GNG	True	1.838e+07	7.62	7.62	0.8007
249	Gly-Gly	Amino acid	2	Ctrl_H2O2_2_4	Ctrl_H2O2_2	0.0	Dipeptides	False			-1.272	-1.489
169	2-OH-Butyrate	Amino acid	2	Ctrl_H2O2_2_4	Ctrl_H2O2_2	0.25	Amino acid derivatives	True	2.816e+04	-1.729	-1.729	-0.2955
98	3-Methyl-2-Oxobutyrate	Amino acid	2	Ctrl_H2O2_2_4	Ctrl_H2O2_2	0.25	Amino acids biosynthesis intermediates	False			-3.759	0
100	4-Me-2-Oxo-Pentanoate	Amino acid	2	Ctrl_H2O2_2_4	Ctrl_H2O2_2	1.0	Amino acids biosynthesis intermediates	True	7411	-3.656	-3.656	-0.3539
253	Gly-Pro	Amino acid	2	Ctrl_H2O2_2_4	Ctrl_H2O2_2	0.75	Dipeptides	True	5.023e+04	-0.8947	-0.8947	-0.5737

Metabolite ID	Name	Super Pathway	Dataset	Sample ID	Group ID	Detection Fraction	Pathway	Detected	Raw Intensity	Log2 Norm Intensity	Norm Imputed	Log2 Ctrl Norm
247	Asp-Phe	Amino acid	2	Ctrl_H2O2_2_4	Ctrl_H2O2_2	1.0	Dipeptides	True	1.907e+04	-2.292	-2.292	-1.351
212	N-Ac-Asp	Amino acid	2	Ctrl_H2O2_2_4	Ctrl_H2O2_2	0.0	N-acetylated amino acids	False			-4.689	-0.1861
720	1-Me-Nicotinamide	Cofactor	2	Ctrl_H2O2_2_4	Ctrl_H2O2_2	1.0	Derivatives of NA, nicotinamide and NAD	True	4.501e+05	2.269	2.269	-1.435
70	Creatine	Carbon	2	Ctrl_H2O2_2_4	Ctrl_H2O2_2	1.0	Creatine energy storage	True	2.791e+06	4.901	4.901	-0.8792
309	Glutathione, Oxidized	Amino acid	2	Ctrl_H2O2_2_4	Ctrl_H2O2_2	1.0	Glutathione	True	7.321e+05	2.971	2.971	-0.9778
44	Glucose 6-P	Carbon	2	Ctrl_H2O2_2_4	Ctrl_H2O2_2	1.0	Glycolysis, GNG	True	4.841e+04	-0.9479	-0.9479	-1.763
24	Fructose	Carbon	2	Ctrl_H2O2_2_4	Ctrl_H2O2_2	1.0	Sugars and sugar alcohols	True	7.885e+05	3.078	3.078	-0.2829
85	Cys	Amino acid	2	Ctrl_H2O2_2_4	Ctrl_H2O2_2	1.0	Proteinogenic amino acids	True	4.031e+04	-1.212	-1.212	-0.6201
704	NADH	Cofactor	2	Ctrl_H2O2_2_4	Ctrl_H2O2_2	0.0	Cofactors	False			-4.313	-2.383
275	Thr-Phe	Amino acid	2	Ctrl_H2O2_2_4	Ctrl_H2O2_2	0.0	Dipeptides	False			-1.922	-0.9888
738	Pyridoxate	Cofactor	2	Ctrl_H2O2_2_4	Ctrl_H2O2_2	0.75	PLP biosynthesis and salvage	False			-5.252	-0.5865
177	3-(4-OH-Phenyl)Lactate	Amino acid	2	Ctrl_H2O2_2_4	Ctrl_H2O2_2	0.5	Amino acid derivatives	False			-4.295	-0.342
206	Trans-4-OH-Pro	Amino acid	2	Ctrl_H2O2_2_4	Ctrl_H2O2_2	1.0	Amino acid derivatives	True	4.185e+04	-1.158	-1.158	-0.9624
329	AMP	Nucleotide	2	Ctrl_H2O2_2_4	Ctrl_H2O2_2	0.25	Purine nucleotides	False			-2.644	-3.064
345	Guanine	Nucleotide	2	Ctrl_H2O2_2_4	Ctrl_H2O2_2	1.0	Purine bases	True	7.245e+05	2.956	2.956	1.374
271	pyroGlu-Val	Amino acid	2	Ctrl_H2O2_2_4	Ctrl_H2O2_2	0.75	Dipeptides	True	1.326e+04	-2.816	-2.816	0.8252
279	Val-Glu	Amino acid	2	Ctrl_H2O2_2_4	Ctrl_H2O2_2	1.0	Dipeptides	True	4.293e+04	-1.121	-1.121	-0.2913
183	Phenol Sulfate	Amino acid	2	Ctrl_H2O2_2_4	Ctrl_H2O2_2	0.25	Amino acid derivatives	False			-4.072	-0.3451
740	3-Dehydrocarnitine	Cofactor	2	Ctrl_H2O2_2_4	Ctrl_H2O2_2	0.5	Carnitine biosynthesis	False			-2.199	-1.865
145	Pyro-Gln	Amino acid	2	Ctrl_H2O2_2_4	Ctrl_H2O2_2	1.0	Amino acid derivatives	True	1.69e+05	0.8558	0.8558	0.5098
197	C-Glycosyl-Trp	Amino acid	2	Ctrl_H2O2_2_4	Ctrl_H2O2_2	1.0	Amino acid derivatives	True	2.855e+04	-1.71	-1.71	-1.566
718	Nicotinamide Riboside	Cofactor	2	Ctrl_H2O2_2_4	Ctrl_H2O2_2	1.0	NAD biosynthesis	True	1.043e+05	0.1587	0.1587	1.963
295	gamma-Glu-Phe	Amino acid	2	Ctrl_H2O2_2_4	Ctrl_H2O2_2	0.25	Gamma-glutamyl dipeptides	True	6.133e+04	-0.6067	-0.6067	-0.5048
399	Pseudouridine	Nucleotide	2	Ctrl_H2O2_2_4	Ctrl_H2O2_2	0.5	Pyrimidine derivatives in RNAs	False			-4.566	-0.3249

Metabolite ID	Name	Super Pathway	Dataset	Sample ID	Group ID	Detection Fraction	Pathway	Detected	Raw Intensity	Log2 Norm Intensity	Norm Imputed	Log2 Ctrl Norm
375	UTP	Nucleotide	2	Ctrl_H2O2_2_4	Ctrl_H2O2_2	0.0	Pyrimidine nucleotides	False			-5.966	-0.5235
20	Erythronate	Carbon	2	Ctrl_H2O2_2_4	Ctrl_H2O2_2	0.75	Aminosugar derivatives	False			-2.431	-0.9891
151	Phenylacetylglycine	Amino acid	2	Ctrl_H2O2_2_4	Ctrl_H2O2_2	1.0	Amino acid derivatives	True	1.187e+04	-2.976	-2.976	1.446
252	Gly-Phe	Amino acid	2	Ctrl_H2O2_2_4	Ctrl_H2O2_2	0.75	Dipeptides	True	1.144e+05	0.2922	0.2922	0.188
251	Gly-Leu	Amino acid	2	Ctrl_H2O2_2_4	Ctrl_H2O2_2	1.0	Dipeptides	True	1.319e+05	0.4976	0.4976	0.135
290	gamma-Glu-Ile	Amino acid	2	Ctrl_H2O2_2_4	Ctrl_H2O2_2	0.5	Gamma-glutamyl dipeptides	False			-2.961	-1.429
316	Ophthalmate	Amino acid	2	Ctrl_H2O2_2_4	Ctrl_H2O2_2	1.0	Oxidative stress markers	True	3.186e+04	-1.552	-1.552	-1.806
208	Pro-OH-Pro	Amino acid	2	Ctrl_H2O2_2_4	Ctrl_H2O2_2	1.0	Amino acid derivatives	True	1.559e+05	0.7396	0.7396	0.2799
352	3'-AMP	Nucleotide	2	Ctrl_H2O2_2_4	Ctrl_H2O2_2	0.75	Purine derivatives in signaling	True	2.935e+04	-1.67	-1.67	-0.385
314	Cys-Glutathione Disulfide	Amino acid	2	Ctrl_H2O2_2_4	Ctrl_H2O2_2	1.0	Oxidative stress markers	True	9.919e+04	0.08696	0.08696	1.452
39	Threitol	Carbon	2	Ctrl_H2O2_2_4	Ctrl_H2O2_2	0.25	Sugars and sugar alcohols	False			-4.395	-0.3698
31	Ribulose/Xylulose	Carbon	2	Ctrl_H2O2_2_4	Ctrl_H2O2_2	0.0	Sugars and sugar alcohols	False			-5.004	-0.09716
48	DHAP	Carbon	2	Ctrl_H2O2_2_4	Ctrl_H2O2_2	1.0	Glycolysis, GNG	True	3.345e+04	-1.481	-1.481	-3.239
182	P-Cresol Sulfate	Amino acid	2	Ctrl_H2O2_2_4	Ctrl_H2O2_2	1.0	Amino acid derivatives	True	8689	-3.426	-3.426	-0.08868
250	Gly-Ile	Amino acid	2	Ctrl_H2O2_2_4	Ctrl_H2O2_2	0.25	Dipeptides	True	3.486e+04	-1.422	-1.422	2.41e-03
286	gamma-Glu-Glu	Amino acid	2	Ctrl_H2O2_2_4	Ctrl_H2O2_2	1.0	Gamma-glutamyl dipeptides	True	4.579e+04	-1.028	-1.028	-0.7975
264	Leu-Leu	Amino acid	2	Ctrl_H2O2_2_4	Ctrl_H2O2_2	0.25	Dipeptides	False			-2.222	-1.148
203	DiMe-Arg	Amino acid	2	Ctrl_H2O2_2_4	Ctrl_H2O2_2	1.0	Amino acid derivatives	True	1.588e+05	0.7657	0.7657	-0.3086
47	Fructose 1,6-PP, Glucose 1,6-PP	Carbon	2	Ctrl_H2O2_2_4	Ctrl_H2O2_2	1.0	Glycolysis, GNG	True	3.143e+04	-1.571	-1.571	-1.281
224	N-Ac-Ser	Amino acid	2	Ctrl_H2O2_2_4	Ctrl_H2O2_2	0.5	N-acetylated amino acids	True	4.324e+04	-1.111	-1.111	-1.982
244	Ala-Leu	Amino acid	2	Ctrl_H2O2_2_4	Ctrl_H2O2_2	0.25	Dipeptides	False			-1.778	-2.118
304	Cyclo(Phe-Pro)	Amino acid	2	Ctrl_H2O2_2_4	Ctrl_H2O2_2	0.25	Cyclic dipeptides	False			-1.192	-0.7201
302	Cyclo(Glu-Glu)	Amino acid	2	Ctrl_H2O2_2_4	Ctrl_H2O2_2	1.0	Cyclic dipeptides	True	5.078e+04	-0.879	-0.879	0.2932
303	Cyclo(Leu-Pro)	Amino acid	2	Ctrl_H2O2_2_4	Ctrl_H2O2_2	0.5	Cyclic dipeptides	True	8.065e+04	-0.2116	-0.2116	-0.4845

Metabolite ID	Name	Super Pathway	Dataset	Sample ID	Group ID	Detection Fraction	Pathway	Detected	Raw Intensity	Log2 Norm Intensity	Norm Imputed	Log2 Ctrl Norm
390	2',3'-cUMP	Nucleotide	2	Ctrl_H2O2_2_4	Ctrl_H2O2_2	1.0	Pyrimidine derivatives in signaling	True	4.33e+04	-1.109	-1.109	0.01999
68	Ribulose 5-P / Xylulose 5-P	Carbon	2	Ctrl_H2O2_2_4	Ctrl_H2O2_2	1.0	Pentose phosphate pathway (PPP)	True	2.74e+04	-1.769	-1.769	-2.538
388	2',3'-cCMP	Nucleotide	2	Ctrl_H2O2_2_4	Ctrl_H2O2_2	1.0	Pyrimidine derivatives in signaling	True	5.148e+04	-0.8592	-0.8592	-1.113
33	Arabitol/Xylitol	Carbon	2	Ctrl_H2O2_2_4	Ctrl_H2O2_2	0.0	Sugars and sugar alcohols	False			-3.559	-0.05408
268	Phe-Phe	Amino acid	2	Ctrl_H2O2_2_4	Ctrl_H2O2_2	0.5	Dipeptides	True	5.144e+04	-0.8604	-0.8604	0.2273
245	Ala-Phe	Amino acid	2	Ctrl_H2O2_2_4	Ctrl_H2O2_2	0.0	Dipeptides	False			-2.821	-1.366
373	UMP	Nucleotide	2	Ctrl_H2O2_2_4	Ctrl_H2O2_2	0.25	Pyrimidine nucleotides	True	1.663e+04	-2.489	-2.489	-0.449
282	Val-Leu	Amino acid	2	Ctrl_H2O2_2_4	Ctrl_H2O2_2	0.75	Dipeptides	True	8.104e+04	-0.2047	-0.2047	0.164
258	Ile-Gly	Amino acid	2	Ctrl_H2O2_2_4	Ctrl_H2O2_2	0.75	Dipeptides	True	1.287e+05	0.4629	0.4629	0.04832
259	Ile-Ser	Amino acid	2	Ctrl_H2O2_2_4	Ctrl_H2O2_2	0.25	Dipeptides	False			-3.192	-2.329
269	Phe-Ser	Amino acid	2	Ctrl_H2O2_2_4	Ctrl_H2O2_2	0.5	Dipeptides	True	1.955e+04	-2.256	-2.256	-0.9412
277	Tyr-Ala	Amino acid	2	Ctrl_H2O2_2_4	Ctrl_H2O2_2	0.75	Dipeptides	True	5.314e+04	-0.8136	-0.8136	-1.384
257	Ile-Gln	Amino acid	2	Ctrl_H2O2_2_4	Ctrl_H2O2_2	0.25	Dipeptides	False			-2.625	-1.706
261	Leu-Glu	Amino acid	2	Ctrl_H2O2_2_4	Ctrl_H2O2_2	0.75	Dipeptides	True	2.823e+04	-1.726	-1.726	-2.516
263	Leu-Gly	Amino acid	2	Ctrl_H2O2_2_4	Ctrl_H2O2_2	1.0	Dipeptides	True	7.645e+04	-0.2888	-0.2888	-0.9958
256	Ile-Ala	Amino acid	2	Ctrl_H2O2_2_4	Ctrl_H2O2_2	0.75	Dipeptides	True	1.798e+05	0.9449	0.9449	0.9867
274	Thr-Leu	Amino acid	2	Ctrl_H2O2_2_4	Ctrl_H2O2_2	0.25	Dipeptides	False			0.2082	-0.6429
273	Ser-Phe	Amino acid	2	Ctrl_H2O2_2_4	Ctrl_H2O2_2	1.0	Dipeptides	True	3.503e+04	-1.415	-1.415	-0.1927
272	Ser-Leu	Amino acid	2	Ctrl_H2O2_2_4	Ctrl_H2O2_2	1.0	Dipeptides	True	6.805e+04	-0.4568	-0.4568	-1.196
246	Asp-Leu	Amino acid	2	Ctrl_H2O2_2_4	Ctrl_H2O2_2	1.0	Dipeptides	True	1.135e+05	0.2819	0.2819	0.356
76	Gln	Amino acid	2	Ctrl_Untreated_2_1	Ctrl_Untreated_2	1.0	Proteinogenic amino acids	True	3.148e+07	6.805	6.805	-0.1471
89	Trp	Amino acid	2	Ctrl_Untreated_2_1	Ctrl_Untreated_2	1.0	Proteinogenic amino acids	True	1.571e+07	5.802	5.802	-0.1648
723	beta-Ala	Cofactor	2	Ctrl_Untreated_2_1	Ctrl_Untreated_2	1.0	Coenzyme A biosynthesis	True	2.951e+05	0.06789	0.06789	-0.2584
75	Glu	Amino acid	2	Ctrl_Untreated_2_1	Ctrl_Untreated_2	1.0	Proteinogenic amino acids	True	1.461e+07	5.698	5.698	-0.2501
80	His	Amino acid	2	Ctrl_Untreated_2_1	Ctrl_Untreated_2	1.0	Proteinogenic amino acids	True	2.8e+05	-8.08e-03	-8.08e-03	-0.2222
82	Leu	Amino acid	2	Ctrl_Untreated_2_1	Ctrl_Untreated_2	1.0	Proteinogenic amino acids	True	7.155e+07	7.989	7.989	-0.1708

Metabolite ID	Name	Super Pathway	Dataset	Sample ID	Group ID	Detection Fraction	Pathway	Detected	Raw Intensity	Log2 Norm Intensity	Norm Imputed	Log2 Ctrl Norm
87	Phe	Amino acid	2	Ctrl_Untreated_2_1	Ctrl_Untreated_2	1.0	Proteinogenic amino acids	True	5.939e+07	7.721	7.721	-0.1712
236	Spermidine	Amino acid	2	Ctrl_Untreated_2_1	Ctrl_Untreated_2	1.0	Polyamines	True	2.092e+06	2.893	2.893	0.06558
73	Asn	Amino acid	2	Ctrl_Untreated_2_1	Ctrl_Untreated_2	1.0	Proteinogenic amino acids	True	2.373e+06	3.075	3.075	0.3265
243	Creatinine	Amino acid	2	Ctrl_Untreated_2_1	Ctrl_Untreated_2	1.0	Creatine degradation	True	7.229e+05	1.36	1.36	-0.2101
376	Cytidine	Nucleotide	2	Ctrl_Untreated_2_1	Ctrl_Untreated_2	1.0	Pyrimidine nucleosides	True	3.82e+05	0.4402	0.4402	1.025
41	Lactate	Carbon	2	Ctrl_Untreated_2_1	Ctrl_Untreated_2	1.0	Respiratory carbon sources	True	4.669e+07	7.373	7.373	-0.4485
93	3-P-Ser	Amino acid	2	Ctrl_Untreated_2_1	Ctrl_Untreated_2	1.0	Amino acids biosynthesis intermediates	True	2.407e+04	-3.548	-3.548	-0.2892
343	Adenine	Nucleotide	2	Ctrl_Untreated_2_1	Ctrl_Untreated_2	1.0	Purine bases	True	1.105e+05	-1.35	-1.35	0.1138
336	Adenosine	Nucleotide	2	Ctrl_Untreated_2_1	Ctrl_Untreated_2	1.0	Purine nucleosides	True	6.526e+06	4.535	4.535	1.397
29	Raffinose	Carbon	2	Ctrl_Untreated_2_1	Ctrl_Untreated_2	1.0	Sugars and sugar alcohols	True	8.907e+04	-1.66	-1.66	0.5195
717	Nicotinamide	Cofactor	2	Ctrl_Untreated_2_1	Ctrl_Untreated_2	1.0	NAD biosynthesis	True	1.146e+06	2.025	2.025	-0.3716
51	PEP	Carbon	2	Ctrl_Untreated_2_1	Ctrl_Untreated_2	1.0	Glycolysis, GNG	True	4.404e+04	-2.677	-2.677	-1.605
52	Pyruvate	Carbon	2	Ctrl_Untreated_2_1	Ctrl_Untreated_2	0.75	Glycolysis, GNG	True	4.328e+04	-2.702	-2.702	-0.328
237	Spermine	Amino acid	2	Ctrl_Untreated_2_1	Ctrl_Untreated_2	1.0	Polyamines	True	2.057e+06	2.869	2.869	-0.5199
385	Uracil	Nucleotide	2	Ctrl_Untreated_2_1	Ctrl_Untreated_2	1.0	Pyrimidine bases	True	5.774e+04	-2.286	-2.286	-0.5589
377	Uridine	Nucleotide	2	Ctrl_Untreated_2_1	Ctrl_Untreated_2	1.0	Pyrimidine nucleosides	True	2.014e+06	2.839	2.839	-0.04371
112	trans-Urocanate	Amino acid	2	Ctrl_Untreated_2_1	Ctrl_Untreated_2	0.5	Amino acids degradation intermediates	True	4.292e+04	-2.714	-2.714	0.404
737	Pyridoxine (Vitamin B6)	Cofactor	2	Ctrl_Untreated_2_1	Ctrl_Untreated_2	1.0	PLP biosynthesis and salvage	True	2.097e+06	2.897	2.897	-0.4031
348	Allantoin	Nucleotide	2	Ctrl_Untreated_2_1	Ctrl_Untreated_2	1.0	Purine degradation	True	3.576e+04	-2.977	-2.977	-0.1476
335	Inosine	Nucleotide	2	Ctrl_Untreated_2_1	Ctrl_Untreated_2	1.0	Purine nucleosides	True	2.198e+06	2.965	2.965	0.1036
81	Ile	Amino acid	2	Ctrl_Untreated_2_1	Ctrl_Untreated_2	1.0	Proteinogenic amino acids	True	5.119e+07	7.506	7.506	-0.3492
72	Ala	Amino acid	2	Ctrl_Untreated_2_1	Ctrl_Untreated_2	1.0	Proteinogenic amino acids	True	7.779e+07	8.11	8.11	0.3762
79	Thr	Amino acid	2	Ctrl_Untreated_2_1	Ctrl_Untreated_2	1.0	Proteinogenic amino acids	True	2.187e+07	6.28	6.28	0.3171

Metabolite ID	Name	Super Pathway	Dataset	Sample ID	Group ID	Detection Fraction	Pathway	Detected	Raw Intensity	Log2 Norm Intensity	Norm Imputed	Log2 Ctrl Norm
88	Tyr	Amino acid	2	Ctrl_Untreated_2_1	Ctrl_Untreated_2	1.0	Proteinogenic amino acids	True	2.96e+07	6.716	6.716	-0.1266
84	Lys	Amino acid	2	Ctrl_Untreated_2_1	Ctrl_Untreated_2	1.0	Proteinogenic amino acids	True	2.743e+06	3.284	3.284	-0.4349
86	Met	Amino acid	2	Ctrl_Untreated_2_1	Ctrl_Untreated_2	1.0	Proteinogenic amino acids	True	1.662e+07	5.883	5.883	0.0959
61	Malate	Carbon	2	Ctrl_Untreated_2_1	Ctrl_Untreated_2	1.0	TCA cycle	True	5.479e+05	0.9605	0.9605	0.06373
235	Putrescine	Amino acid	2	Ctrl_Untreated_2_1	Ctrl_Untreated_2	1.0	Polyamines	True	2.328e+05	-0.2742	-0.2742	1.293
49	3-P-Glycerate	Carbon	2	Ctrl_Untreated_2_1	Ctrl_Untreated_2	1.0	Glycolysis, GNG	True	6.175e+05	1.133	1.133	-1.33
139	GABA	Amino acid	2	Ctrl_Untreated_2_1	Ctrl_Untreated_2	0.75	Amino acid derivatives	True	5.8e+04	-2.279	-2.279	1.191
189	Kynurenate	Amino acid	2	Ctrl_Untreated_2_1	Ctrl_Untreated_2	0.0	Amino acid derivatives	False			-5.188	0
234	5-Me-Thioadenosine	Amino acid	2	Ctrl_Untreated_2_1	Ctrl_Untreated_2	1.0	SAM metabolism	True	2.501e+05	-0.1707	-0.1707	-0.0747
59	Succinate	Carbon	2	Ctrl_Untreated_2_1	Ctrl_Untreated_2	1.0	TCA cycle	True	6.547e+04	-2.105	-2.105	-0.06455
133	Ornithine	Amino acid	2	Ctrl_Untreated_2_1	Ctrl_Untreated_2	1.0	Amino acids degradation intermediates	True	2.178e+06	2.951	2.951	0.43
313	5-Oxoproline	Amino acid	2	Ctrl_Untreated_2_1	Ctrl_Untreated_2	1.0	Glutathione derivatives	True	8.996e+05	1.676	1.676	-0.3278
724	Pantothenate	Cofactor	2	Ctrl_Untreated_2_1	Ctrl_Untreated_2	1.0	Coenzyme A biosynthesis	True	1.666e+06	2.565	2.565	-0.5487
30	Sucrose	Carbon	2	Ctrl_Untreated_2_1	Ctrl_Untreated_2	1.0	Sugars and sugar alcohols	True	1.021e+06	1.859	1.859	-0.5178
122	3-OH-Isobutyrate	Amino acid	2	Ctrl_Untreated_2_1	Ctrl_Untreated_2	0.25	Amino acids degradation intermediates	False			-4.628	0
241	4-Acetamidobutanoate	Amino acid	2	Ctrl_Untreated_2_1	Ctrl_Untreated_2	1.0	Polyamine derivatives	True	7.394e+04	-1.929	-1.929	-0.4218
55	Citrate	Carbon	2	Ctrl_Untreated_2_1	Ctrl_Untreated_2	1.0	TCA cycle	True	2.596e+06	3.205	3.205	0.1707
338	Guanosine	Nucleotide	2	Ctrl_Untreated_2_1	Ctrl_Untreated_2	1.0	Purine nucleosides	True	1.651e+06	2.552	2.552	0.1917
170	2-Amino-Butyrate	Amino acid	2	Ctrl_Untreated_2_1	Ctrl_Untreated_2	1.0	Amino acid derivatives	True	6.806e+05	1.273	1.273	0.04959
209	N-Ac-Ala	Amino acid	2	Ctrl_Untreated_2_1	Ctrl_Untreated_2	1.0	N-acetylated amino acids	True	3.138e+04	-3.165	-3.165	0.1021
221	N-Ac-Met	Amino acid	2	Ctrl_Untreated_2_1	Ctrl_Untreated_2	1.0	N-acetylated amino acids	True	1.4e+05	-1.009	-1.009	0.1417
22	N-Ac-Neuraminate	Carbon	2	Ctrl_Untreated_2_1	Ctrl_Untreated_2	1.0	Aminosugar derivatives	True	1.849e+05	-0.6068	-0.6068	-0.3928
346	Urate	Nucleotide	2	Ctrl_Untreated_2_1	Ctrl_Untreated_2	1.0	Purine degradation	True	2.214e+04	-3.668	-3.668	0.02373

Metabolite ID	Name	Super Pathway	Dataset	Sample ID	Group ID	Detection Fraction	Pathway	Detected	Raw Intensity	Log2 Norm Intensity	Norm Imputed	Log2 Ctrl Norm
90	Arg	Amino acid	2	Ctrl_Untreated_2_1	Ctrl_Untreated_2	1.0	Proteinogenic amino acids	True	6.631e+06	4.558	4.558	-0.2244
60	Fumarate	Carbon	2	Ctrl_Untreated_2_1	Ctrl_Untreated_2	1.0	TCA cycle	True	2.178e+05	-0.3707	-0.3707	-0.214
78	Ser	Amino acid	2	Ctrl_Untreated_2_1	Ctrl_Untreated_2	1.0	Proteinogenic amino acids	True	3.945e+07	7.13	7.13	0.3037
83	Val	Amino acid	2	Ctrl_Untreated_2_1	Ctrl_Untreated_2	1.0	Proteinogenic amino acids	True	3.383e+07	6.909	6.909	-0.3253
734	Pyridoxal	Cofactor	2	Ctrl_Untreated_2_1	Ctrl_Untreated_2	0.75	PLP biosynthesis and salvage	True	2.049e+05	-0.4585	-0.4585	0.5361
136	Urea	Amino acid	2	Ctrl_Untreated_2_1	Ctrl_Untreated_2	1.0	Amino acids degradation intermediates	True	6.873e+05	1.287	1.287	-0.3576
742	Folate	Cofactor	2	Ctrl_Untreated_2_1	Ctrl_Untreated_2	1.0	Folate metabolism	True	1.435e+05	-0.9726	-0.9726	-0.4556
729	Riboflavin (Vitamin B2)	Cofactor	2	Ctrl_Untreated_2_1	Ctrl_Untreated_2	1.0	Flavine biosynthesis	True	8.427e+04	-1.74	-1.74	-0.1712
91	Pro	Amino acid	2	Ctrl_Untreated_2_1	Ctrl_Untreated_2	1.0	Proteinogenic amino acids	True	3.412e+07	6.921	6.921	0.2001
308	Glutathione, Reduced	Amino acid	2	Ctrl_Untreated_2_1	Ctrl_Untreated_2	1.0	Glutathione	True	2.695e+07	6.581	6.581	0.1256
706	FAD	Cofactor	2	Ctrl_Untreated_2_1	Ctrl_Untreated_2	0.0	Cofactors	False			-5.096	0
299	gamma-Glu-Tyr	Amino acid	2	Ctrl_Untreated_2_1	Ctrl_Untreated_2	1.0	Gamma-glutamyl dipeptides	True	9.876e+04	-1.511	-1.511	-0.3441
705	Coenzyme A	Cofactor	2	Ctrl_Untreated_2_1	Ctrl_Untreated_2	1.0	Cofactors	True	3.171e+04	-3.151	-3.151	0.5558
342	Hypoxanthine	Nucleotide	2	Ctrl_Untreated_2_1	Ctrl_Untreated_2	1.0	Purine bases	True	2.71e+05	-0.05525	-0.05525	-0.05713
344	Xanthine	Nucleotide	2	Ctrl_Untreated_2_1	Ctrl_Untreated_2	1.0	Purine bases	True	2.297e+04	-3.616	-3.616	-1.376
703	NAD+	Cofactor	2	Ctrl_Untreated_2_1	Ctrl_Untreated_2	1.0	Cofactors	True	3.454e+06	3.617	3.617	0.05803
731	Thiamin (Vitamin B1)	Cofactor	2	Ctrl_Untreated_2_1	Ctrl_Untreated_2	1.0	TPP biosynthesis	True	1.524e+05	-0.8859	-0.8859	-0.6798
102	2-Aminoadipate	Amino acid	2	Ctrl_Untreated_2_1	Ctrl_Untreated_2	1.0	Amino acids biosynthesis intermediates	True	4.531e+05	0.6862	0.6862	-0.07027
77	Gly	Amino acid	2	Ctrl_Untreated_2_1	Ctrl_Untreated_2	1.0	Proteinogenic amino acids	True	4.388e+07	7.284	7.284	0.05571
45	Fructose-6-P	Carbon	2	Ctrl_Untreated_2_1	Ctrl_Untreated_2	0.75	Glycolysis, GNG	True	6.239e+04	-2.174	-2.174	-0.7388
36	Ribose	Carbon	2	Ctrl_Untreated_2_1	Ctrl_Untreated_2	1.0	Sugars and sugar alcohols	True	1.928e+05	-0.5465	-0.5465	0.451
4	GlcNAc 6-P	Carbon	2	Ctrl_Untreated_2_1	Ctrl_Untreated_2	1.0	Aminosugar biosynthesis	True	4.692e+05	0.7368	0.7368	0.3771
188	Kynurenine	Amino acid	2	Ctrl_Untreated_2_1	Ctrl_Untreated_2	1.0	Amino acid derivatives	True	2.184e+05	-0.3662	-0.3662	-0.3522
63	6-P-Gluconate	Carbon	2	Ctrl_Untreated_2_1	Ctrl_Untreated_2	1.0	Pentose phosphate pathway (PPP)	True	1.104e+05	-1.351	-1.351	-0.3057

Metabolite ID	Name	Super Pathway	Dataset	Sample ID	Group ID	Detection Fraction	Pathway	Detected	Raw Intensity	Log2 Norm Intensity	Norm Imputed	Log2 Ctrl Norm
710	Carnitine	Cofactor	2	Ctrl_Untreated_2_1	Ctrl_Untreated_2	1.0	Cofactors	True	1.716e+06	2.608	2.608	0.07591
725	P-Pantetheine	Cofactor	2	Ctrl_Untreated_2_1	Ctrl_Untreated_2	1.0	Coenzyme A biosynthesis	True	8815	-4.997	-4.997	-0.1849
110	N-alpha-Ac-Ornithine	Amino acid	2	Ctrl_Untreated_2_1	Ctrl_Untreated_2	1.0	Amino acids biosynthesis intermediates	True	5.621e+05	0.9975	0.9975	0.02344
116	3-Me-2-Oxo-Valerate	Amino acid	2	Ctrl_Untreated_2_1	Ctrl_Untreated_2	1.0	Amino acids degradation intermediates	True	2.251e+04	-3.645	-3.645	-0.07022
155	4-Guanidinobutanoate	Amino acid	2	Ctrl_Untreated_2_1	Ctrl_Untreated_2	1.0	Amino acid derivatives	True	9.443e+04	-1.576	-1.576	-0.8567
310	S-Lactoyl-Glutathione	Amino acid	2	Ctrl_Untreated_2_1	Ctrl_Untreated_2	1.0	Glutathione derivatives	True	1.134e+04	-4.634	-4.634	-0.962
34	Ribitol	Carbon	2	Ctrl_Untreated_2_1	Ctrl_Untreated_2	0.75	Sugars and sugar alcohols	True	1.519e+05	-0.8904	-0.8904	0.6884
707	FMN	Cofactor	2	Ctrl_Untreated_2_1	Ctrl_Untreated_2	1.0	Cofactors	True	2.248e+04	-3.647	-3.647	-0.2262
17	Maltose	Carbon	2	Ctrl_Untreated_2_1	Ctrl_Untreated_2	1.0	Glycogen degradation	True	1.812e+05	-0.636	-0.636	-0.5966
18	Maltotriose	Carbon	2	Ctrl_Untreated_2_1	Ctrl_Untreated_2	1.0	Glycogen degradation	True	5.801e+05	1.043	1.043	-0.3859
19	Maltotetraose	Carbon	2	Ctrl_Untreated_2_1	Ctrl_Untreated_2	1.0	Glycogen degradation	True	1.748e+05	-0.6881	-0.6881	-0.4977
232	SAH	Amino acid	2	Ctrl_Untreated_2_1	Ctrl_Untreated_2	1.0	SAM metabolism	True	2.8e+04	-3.33	-3.33	-0.1037
74	Asp	Amino acid	2	Ctrl_Untreated_2_1	Ctrl_Untreated_2	1.0	Proteinogenic amino acids	True	1.682e+07	5.9	5.9	0.2409
129	5-Aminovalerate	Amino acid	2	Ctrl_Untreated_2_1	Ctrl_Untreated_2	1.0	Amino acids degradation intermediates	True	2.012e+05	-0.4846	-0.4846	0.3285
254	Gly-Val	Amino acid	2	Ctrl_Untreated_2_1	Ctrl_Untreated_2	1.0	Dipeptides	True	2.14e+05	-0.3957	-0.3957	-0.322
291	gamma-Glu-Leu	Amino acid	2	Ctrl_Untreated_2_1	Ctrl_Untreated_2	0.75	Gamma-glutamyl dipeptides	True	2.702e+05	-0.05952	-0.05952	1.022
173	Met Sulfoxide	Amino acid	2	Ctrl_Untreated_2_1	Ctrl_Untreated_2	1.0	Amino acid derivatives	True	3.028e+05	0.1048	0.1048	0.2436
43	Glucose	Carbon	2	Ctrl_Untreated_2_1	Ctrl_Untreated_2	1.0	Glycolysis, GNG	True	2.372e+07	6.396	6.396	-0.4231
249	Gly-Gly	Amino acid	2	Ctrl_Untreated_2_1	Ctrl_Untreated_2	1.0	Dipeptides	True	4.061e+05	0.5285	0.5285	0.3113
169	2-OH-Butyrate	Amino acid	2	Ctrl_Untreated_2_1	Ctrl_Untreated_2	0.5	Amino acid derivatives	True	1.104e+05	-1.35	-1.35	0.08379
98	3-Methyl-2-Oxobutyrate	Amino acid	2	Ctrl_Untreated_2_1	Ctrl_Untreated_2	0.0	Amino acids biosynthesis intermediates	False			-3.759	0

Metabolite ID	Name	Super Pathway	Dataset	Sample ID	Group ID	Detection Fraction	Pathway	Detected	Raw Intensity	Log2 Norm Intensity	Norm Imputed	Log2 Ctrl Norm
100	4-Me-2-Oxo-Pentanoate	Amino acid	2	Ctrl_Untreated_2_1	Ctrl_Untreated_2	1.0	Amino acids biosynthesis intermediates	True	2.253e+04	-3.643	-3.643	-0.3415
253	Gly-Pro	Amino acid	2	Ctrl_Untreated_2_1	Ctrl_Untreated_2	1.0	Dipeptides	True	1.987e+05	-0.5025	-0.5025	-0.1815
247	Asp-Phe	Amino acid	2	Ctrl_Untreated_2_1	Ctrl_Untreated_2	1.0	Dipeptides	True	1.423e+05	-0.9848	-0.9848	-0.04357
212	N-Ac-Asp	Amino acid	2	Ctrl_Untreated_2_1	Ctrl_Untreated_2	0.5	N-acetylated amino acids	True	1.092e+04	-4.689	-4.689	-0.1861
720	1-Me-Nicotinamide	Cofactor	2	Ctrl_Untreated_2_1	Ctrl_Untreated_2	1.0	Derivatives of NA, nicotinamide and NAD	True	2.949e+06	3.389	3.389	-0.315
70	Creatine	Carbon	2	Ctrl_Untreated_2_1	Ctrl_Untreated_2	1.0	Creatine energy storage	True	1.369e+07	5.604	5.604	-0.1763
309	Glutathione, Oxidized	Amino acid	2	Ctrl_Untreated_2_1	Ctrl_Untreated_2	1.0	Glutathione	True	4.145e+06	3.88	3.88	-0.06843
44	Glucose 6-P	Carbon	2	Ctrl_Untreated_2_1	Ctrl_Untreated_2	1.0	Glycolysis, GNG	True	2.831e+05	8.08e-03	8.08e-03	-0.8067
24	Fructose	Carbon	2	Ctrl_Untreated_2_1	Ctrl_Untreated_2	1.0	Sugars and sugar alcohols	True	2.816e+06	3.322	3.322	-0.03832
85	Cys	Amino acid	2	Ctrl_Untreated_2_1	Ctrl_Untreated_2	1.0	Proteinogenic amino acids	True	3.266e+05	0.2139	0.2139	0.8058
704	NADH	Cofactor	2	Ctrl_Untreated_2_1	Ctrl_Untreated_2	1.0	Cofactors	True	1.02e+05	-1.465	-1.465	0.4644
275	Thr-Phe	Amino acid	2	Ctrl_Untreated_2_1	Ctrl_Untreated_2	1.0	Dipeptides	True	1.962e+05	-0.5214	-0.5214	0.4122
738	Pyridoxate	Cofactor	2	Ctrl_Untreated_2_1	Ctrl_Untreated_2	0.75	PLP biosynthesis and salvage	False			-5.252	-0.5865
177	3-(4-OH-Phenyl)Lactate	Amino acid	2	Ctrl_Untreated_2_1	Ctrl_Untreated_2	1.0	Amino acid derivatives	True	2.126e+04	-3.727	-3.727	0.2261
206	Trans-4-OH-Pro	Amino acid	2	Ctrl_Untreated_2_1	Ctrl_Untreated_2	1.0	Amino acid derivatives	True	3.023e+05	0.1027	0.1027	0.2983
329	AMP	Nucleotide	2	Ctrl_Untreated_2_1	Ctrl_Untreated_2	1.0	Purine nucleotides	True	1.03e+06	1.871	1.871	1.45
345	Guanine	Nucleotide	2	Ctrl_Untreated_2_1	Ctrl_Untreated_2	1.0	Purine bases	True	9.183e+05	1.706	1.706	0.1236
271	pyroGlu-Val	Amino acid	2	Ctrl_Untreated_2_1	Ctrl_Untreated_2	1.0	Dipeptides	True	1.542e+04	-4.191	-4.191	-0.549
279	Val-Glu	Amino acid	2	Ctrl_Untreated_2_1	Ctrl_Untreated_2	1.0	Dipeptides	True	2.299e+05	-0.2922	-0.2922	0.5378
183	Phenol Sulfate	Amino acid	2	Ctrl_Untreated_2_1	Ctrl_Untreated_2	0.5	Amino acid derivatives	True	1.674e+04	-4.072	-4.072	-0.3451
740	3-Dehydrocarnitine	Cofactor	2	Ctrl_Untreated_2_1	Ctrl_Untreated_2	1.0	Carnitine biosynthesis	True	2.278e+05	-0.306	-0.306	0.02763
145	Pyro-Gln	Amino acid	2	Ctrl_Untreated_2_1	Ctrl_Untreated_2	1.0	Amino acid derivatives	True	2.299e+05	-0.2925	-0.2925	-0.6385
197	C-Glycosyl-Trp	Amino acid	2	Ctrl_Untreated_2_1	Ctrl_Untreated_2	1.0	Amino acid derivatives	True	2.112e+05	-0.415	-0.415	-0.2713
718	Nicotinamide Riboside	Cofactor	2	Ctrl_Untreated_2_1	Ctrl_Untreated_2	0.5	NAD biosynthesis	False			-2.306	-0.5023

Metabolite ID	Name	Super Pathway	Dataset	Sample ID	Group ID	Detection Fraction	Pathway	Detected	Raw Intensity	Log2 Norm Intensity	Norm Imputed	Log2 Ctrl Norm
295	gamma-Glu-Phe	Amino acid	2	Ctrl_Untreated_2_1	Ctrl_Untreated_2	1.0	Gamma-glutamyl dipeptides	True	2.155e+05	-0.386	-0.386	-0.2841
399	Pseudouridine	Nucleotide	2	Ctrl_Untreated_2_1	Ctrl_Untreated_2	1.0	Pyrimidine derivatives in RNAs	True	1.382e+04	-4.349	-4.349	-0.1082
375	UTP	Nucleotide	2	Ctrl_Untreated_2_1	Ctrl_Untreated_2	0.75	Pyrimidine nucleotides	True	4504	-5.966	-5.966	-0.5235
20	Erythronate	Carbon	2	Ctrl_Untreated_2_1	Ctrl_Untreated_2	1.0	Aminosugar derivatives	True	1.285e+05	-1.132	-1.132	0.3098
151	Phenylacetylglycine	Amino acid	2	Ctrl_Untreated_2_1	Ctrl_Untreated_2	1.0	Amino acid derivatives	True	1.008e+04	-4.804	-4.804	-0.3822
252	Gly-Phe	Amino acid	2	Ctrl_Untreated_2_1	Ctrl_Untreated_2	1.0	Dipeptides	True	3.045e+05	0.113	0.113	8.84e-03
251	Gly-Leu	Amino acid	2	Ctrl_Untreated_2_1	Ctrl_Untreated_2	1.0	Dipeptides	True	3.858e+05	0.4543	0.4543	0.0917
290	gamma-Glu-Ile	Amino acid	2	Ctrl_Untreated_2_1	Ctrl_Untreated_2	0.75	Gamma-glutamyl dipeptides	True	1.698e+05	-0.7297	-0.7297	0.8015
316	Ophthalmate	Amino acid	2	Ctrl_Untreated_2_1	Ctrl_Untreated_2	1.0	Oxidative stress markers	True	7.328e+05	1.38	1.38	1.126
208	Pro-OH-Pro	Amino acid	2	Ctrl_Untreated_2_1	Ctrl_Untreated_2	1.0	Amino acid derivatives	True	3.221e+05	0.1943	0.1943	-0.2654
352	3'-AMP	Nucleotide	2	Ctrl_Untreated_2_1	Ctrl_Untreated_2	1.0	Purine derivatives in signaling	True	9.324e+04	-1.594	-1.594	-0.3095
314	Cys-Glutathione Disulfide	Amino acid	2	Ctrl_Untreated_2_1	Ctrl_Untreated_2	1.0	Oxidative stress markers	True	1.117e+05	-1.333	-1.333	0.03202
39	Threitol	Carbon	2	Ctrl_Untreated_2_1	Ctrl_Untreated_2	0.75	Sugars and sugar alcohols	True	2.342e+04	-3.587	-3.587	0.4376
31	Ribulose/Xylulose	Carbon	2	Ctrl_Untreated_2_1	Ctrl_Untreated_2	0.5	Sugars and sugar alcohols	True	1.149e+04	-4.615	-4.615	0.2915
48	DHAP	Carbon	2	Ctrl_Untreated_2_1	Ctrl_Untreated_2	1.0	Glycolysis, GNG	True	7.398e+05	1.394	1.394	-0.3644
182	P-Cresol Sulfate	Amino acid	2	Ctrl_Untreated_2_1	Ctrl_Untreated_2	1.0	Amino acid derivatives	True	3.988e+04	-2.82	-2.82	0.5177
250	Gly-Ile	Amino acid	2	Ctrl_Untreated_2_1	Ctrl_Untreated_2	0.75	Dipeptides	True	1.371e+05	-1.038	-1.038	0.3862
286	gamma-Glu-Glu	Amino acid	2	Ctrl_Untreated_2_1	Ctrl_Untreated_2	1.0	Gamma-glutamyl dipeptides	True	3.379e+05	0.263	0.263	0.4936
264	Leu-Leu	Amino acid	2	Ctrl_Untreated_2_1	Ctrl_Untreated_2	1.0	Dipeptides	True	2.799e+05	-8.29e-03	-8.29e-03	1.065
203	DiMe-Arg	Amino acid	2	Ctrl_Untreated_2_1	Ctrl_Untreated_2	1.0	Amino acid derivatives	True	3.797e+05	0.4314	0.4314	-0.6429
47	Fructose 1,6-PP, Glucose 1,6-PP	Carbon	2	Ctrl_Untreated_2_1	Ctrl_Untreated_2	1.0	Glycolysis, GNG	True	2.03e+05	-0.472	-0.472	-0.1818
224	N-Ac-Ser	Amino acid	2	Ctrl_Untreated_2_1	Ctrl_Untreated_2	1.0	N-acetylated amino acids	True	5.095e+05	0.8557	0.8557	-0.01503
244	Ala-Leu	Amino acid	2	Ctrl_Untreated_2_1	Ctrl_Untreated_2	1.0	Dipeptides	True	5.746e+05	1.029	1.029	0.6894

Metabolite ID	Name	Super Pathway	Dataset	Sample ID	Group ID	Detection Fraction	Pathway	Detected	Raw Intensity	Log2 Norm Intensity	Norm Imputed	Log2 Ctrl Norm
304	Cyclo(Phe-Pro)	Amino acid	2	Ctrl_Untreated_2_1	Ctrl_Untreated_2	1.0	Cyclic dipeptides	True	1.233e+05	-1.192	-1.192	-0.7201
302	Cyclo(Glu-Glu)	Amino acid	2	Ctrl_Untreated_2_1	Ctrl_Untreated_2	1.0	Cyclic dipeptides	True	8.921e+04	-1.658	-1.658	-0.4859
303	Cyclo(Leu-Pro)	Amino acid	2	Ctrl_Untreated_2_1	Ctrl_Untreated_2	1.0	Cyclic dipeptides	True	2.09e+05	-0.4297	-0.4297	-0.7026
390	2',3'-cUMP	Nucleotide	2	Ctrl_Untreated_2_1	Ctrl_Untreated_2	1.0	Pyrimidine derivatives in signaling	True	6.858e+04	-2.038	-2.038	-0.9087
68	Ribulose 5-P / Xylulose 5-P	Carbon	2	Ctrl_Untreated_2_1	Ctrl_Untreated_2	1.0	Pentose phosphate pathway (PPP)	True	5.451e+05	0.953	0.953	0.1838
388	2',3'-cCMP	Nucleotide	2	Ctrl_Untreated_2_1	Ctrl_Untreated_2	1.0	Pyrimidine derivatives in signaling	True	1.893e+05	-0.573	-0.573	-0.8265
33	Arabitol/Xylitol	Carbon	2	Ctrl_Untreated_2_1	Ctrl_Untreated_2	0.25	Sugars and sugar alcohols	False			-3.559	-0.05408
268	Phe-Phe	Amino acid	2	Ctrl_Untreated_2_1	Ctrl_Untreated_2	0.75	Dipeptides	True	1.37e+05	-1.039	-1.039	0.04839
245	Ala-Phe	Amino acid	2	Ctrl_Untreated_2_1	Ctrl_Untreated_2	1.0	Dipeptides	True	2.071e+05	-0.4434	-0.4434	1.012
373	UMP	Nucleotide	2	Ctrl_Untreated_2_1	Ctrl_Untreated_2	0.75	Pyrimidine nucleotides	True	1.255e+05	-1.166	-1.166	0.8741
282	Val-Leu	Amino acid	2	Ctrl_Untreated_2_1	Ctrl_Untreated_2	1.0	Dipeptides	True	4.093e+05	0.5399	0.5399	0.9086
258	Ile-Gly	Amino acid	2	Ctrl_Untreated_2_1	Ctrl_Untreated_2	1.0	Dipeptides	True	5.112e+05	0.8605	0.8605	0.4459
259	Ile-Ser	Amino acid	2	Ctrl_Untreated_2_1	Ctrl_Untreated_2	1.0	Dipeptides	True	3.463e+05	0.2985	0.2985	1.162
269	Phe-Ser	Amino acid	2	Ctrl_Untreated_2_1	Ctrl_Untreated_2	1.0	Dipeptides	True	2.485e+05	-0.1799	-0.1799	1.135
277	Tyr-Ala	Amino acid	2	Ctrl_Untreated_2_1	Ctrl_Untreated_2	1.0	Dipeptides	True	4.596e+05	0.707	0.707	0.1369
257	Ile-Gln	Amino acid	2	Ctrl_Untreated_2_1	Ctrl_Untreated_2	1.0	Dipeptides	True	3.695e+05	0.3923	0.3923	1.311
261	Leu-Glu	Amino acid	2	Ctrl_Untreated_2_1	Ctrl_Untreated_2	1.0	Dipeptides	True	7.453e+05	1.404	1.404	0.6143
263	Leu-Gly	Amino acid	2	Ctrl_Untreated_2_1	Ctrl_Untreated_2	1.0	Dipeptides	True	1.838e+06	2.707	2.707	2
256	Ile-Ala	Amino acid	2	Ctrl_Untreated_2_1	Ctrl_Untreated_2	1.0	Dipeptides	True	3.516e+05	0.3203	0.3203	0.3621
274	Thr-Leu	Amino acid	2	Ctrl_Untreated_2_1	Ctrl_Untreated_2	1.0	Dipeptides	True	1.045e+06	1.892	1.892	1.041
273	Ser-Phe	Amino acid	2	Ctrl_Untreated_2_1	Ctrl_Untreated_2	1.0	Dipeptides	True	1.831e+05	-0.6209	-0.6209	0.601
272	Ser-Leu	Amino acid	2	Ctrl_Untreated_2_1	Ctrl_Untreated_2	1.0	Dipeptides	True	1.161e+06	2.044	2.044	1.305
246	Asp-Leu	Amino acid	2	Ctrl_Untreated_2_1	Ctrl_Untreated_2	1.0	Dipeptides	True	3.094e+05	0.1359	0.1359	0.21
76	Gln	Amino acid	2	Ctrl_Untreated_2_2	Ctrl_Untreated_2	1.0	Proteinogenic amino acids	True	1.977e+07	7.403	7.403	0.4514
89	Trp	Amino acid	2	Ctrl_Untreated_2_2	Ctrl_Untreated_2	1.0	Proteinogenic amino acids	True	7.097e+06	5.926	5.926	-0.04089
723	beta-Ala	Cofactor	2	Ctrl_Untreated_2_2	Ctrl_Untreated_2	1.0	Coenzyme A biosynthesis	True	1.168e+05	0	0	-0.3263
75	Glu	Amino acid	2	Ctrl_Untreated_2_2	Ctrl_Untreated_2	1.0	Proteinogenic amino acids	True	1.011e+07	6.436	6.436	0.4885

Metabolite ID	Name	Super Pathway	Dataset	Sample ID	Group ID	Detection Fraction	Pathway	Detected	Raw Intensity	Log2 Norm Intensity	Norm Imputed	Log2 Ctrl Norm
80	His	Amino acid	2	Ctrl_Untreated_2_2	Ctrl_Untreated_2	1.0	Proteinogenic amino acids	True	1.539e+05	0.3987	0.3987	0.1845
82	Leu	Amino acid	2	Ctrl_Untreated_2_2	Ctrl_Untreated_2	1.0	Proteinogenic amino acids	True	3.512e+07	8.233	8.233	0.07262
87	Phe	Amino acid	2	Ctrl_Untreated_2_2	Ctrl_Untreated_2	1.0	Proteinogenic amino acids	True	2.789e+07	7.9	7.9	8.26e-03
236	Spermidine	Amino acid	2	Ctrl_Untreated_2_2	Ctrl_Untreated_2	1.0	Polyamines	True	6.538e+05	2.485	2.485	-0.3423
73	Asn	Amino acid	2	Ctrl_Untreated_2_2	Ctrl_Untreated_2	1.0	Proteinogenic amino acids	True	6.273e+05	2.426	2.426	-0.3232
243	Creatinine	Amino acid	2	Ctrl_Untreated_2_2	Ctrl_Untreated_2	1.0	Creatine degradation	True	3.017e+05	1.37	1.37	-0.2006
376	Cytidine	Nucleotide	2	Ctrl_Untreated_2_2	Ctrl_Untreated_2	1.0	Pyrimidine nucleosides	True	2.705e+04	-2.11	-2.11	-1.525
41	Lactate	Carbon	2	Ctrl_Untreated_2_2	Ctrl_Untreated_2	1.0	Respiratory carbon sources	True	2.365e+07	7.662	7.662	-0.1596
93	3-P-Ser	Amino acid	2	Ctrl_Untreated_2_2	Ctrl_Untreated_2	1.0	Amino acids biosynthesis intermediates	True	3.103e+04	-1.912	-1.912	1.347
343	Adenine	Nucleotide	2	Ctrl_Untreated_2_2	Ctrl_Untreated_2	1.0	Purine bases	True	3.253e+04	-1.844	-1.844	-0.38
336	Adenosine	Nucleotide	2	Ctrl_Untreated_2_2	Ctrl_Untreated_2	1.0	Purine nucleosides	True	2.534e+05	1.118	1.118	-2.019
29	Raffinose	Carbon	2	Ctrl_Untreated_2_2	Ctrl_Untreated_2	1.0	Sugars and sugar alcohols	True	2.257e+04	-2.371	-2.371	-0.1909
717	Nicotinamide	Cofactor	2	Ctrl_Untreated_2_2	Ctrl_Untreated_2	1.0	NAD biosynthesis	True	6.445e+05	2.465	2.465	0.06798
51	PEP	Carbon	2	Ctrl_Untreated_2_2	Ctrl_Untreated_2	1.0	Glycolysis, GNG	True	1.32e+05	0.1771	0.1771	1.249
52	Pyruvate	Carbon	2	Ctrl_Untreated_2_2	Ctrl_Untreated_2	0.75	Glycolysis, GNG	False			-3.589	-1.215
237	Spermine	Amino acid	2	Ctrl_Untreated_2_2	Ctrl_Untreated_2	1.0	Polyamines	True	1.526e+06	3.708	3.708	0.3198
385	Uracil	Nucleotide	2	Ctrl_Untreated_2_2	Ctrl_Untreated_2	1.0	Pyrimidine bases	True	4.583e+04	-1.349	-1.349	0.3779
377	Uridine	Nucleotide	2	Ctrl_Untreated_2_2	Ctrl_Untreated_2	1.0	Pyrimidine nucleosides	True	8.764e+05	2.908	2.908	0.02572
112	trans-Urocanate	Amino acid	2	Ctrl_Untreated_2_2	Ctrl_Untreated_2	0.5	Amino acids degradation intermediates	False			-3.252	-0.1347
737	Pyridoxine (Vitamin B6)	Cofactor	2	Ctrl_Untreated_2_2	Ctrl_Untreated_2	1.0	PLP biosynthesis and salvage	True	1.721e+06	3.881	3.881	0.5815
348	Allantoin	Nucleotide	2	Ctrl_Untreated_2_2	Ctrl_Untreated_2	1.0	Purine degradation	True	1.794e+04	-2.702	-2.702	0.1276
335	Inosine	Nucleotide	2	Ctrl_Untreated_2_2	Ctrl_Untreated_2	1.0	Purine nucleosides	True	6.79e+05	2.54	2.54	-0.3212
81	Ile	Amino acid	2	Ctrl_Untreated_2_2	Ctrl_Untreated_2	1.0	Proteinogenic amino acids	True	3.053e+07	8.03	8.03	0.175

Metabolite ID	Name	Super Pathway	Dataset	Sample ID	Group ID	Detection Fraction	Pathway	Detected	Raw Intensity	Log2 Norm Intensity	Norm Imputed	Log2 Ctrl Norm
72	Ala	Amino acid	2	Ctrl_Untreated_2_2	Ctrl_Untreated_2	1.0	Proteinogenic amino acids	True	1.988e+07	7.412	7.412	-0.3223
79	Thr	Amino acid	2	Ctrl_Untreated_2_2	Ctrl_Untreated_2	1.0	Proteinogenic amino acids	True	5.626e+06	5.591	5.591	-0.3719
88	Tyr	Amino acid	2	Ctrl_Untreated_2_2	Ctrl_Untreated_2	1.0	Proteinogenic amino acids	True	1.331e+07	6.833	6.833	-9.13e-03
84	Lys	Amino acid	2	Ctrl_Untreated_2_2	Ctrl_Untreated_2	1.0	Proteinogenic amino acids	True	2.382e+06	4.35	4.35	0.6313
86	Met	Amino acid	2	Ctrl_Untreated_2_2	Ctrl_Untreated_2	1.0	Proteinogenic amino acids	True	5.85e+06	5.647	5.647	-0.1401
61	Malate	Carbon	2	Ctrl_Untreated_2_2	Ctrl_Untreated_2	1.0	TCA cycle	True	1.857e+05	0.6698	0.6698	-0.227
235	Putrescine	Amino acid	2	Ctrl_Untreated_2_2	Ctrl_Untreated_2	1.0	Polyamines	True	9017	-3.695	-3.695	-2.127
49	3-P-Glycerate	Carbon	2	Ctrl_Untreated_2_2	Ctrl_Untreated_2	1.0	Glycolysis, GNG	True	1.215e+06	3.38	3.38	0.917
139	GABA	Amino acid	2	Ctrl_Untreated_2_2	Ctrl_Untreated_2	0.75	Amino acid derivatives	False			-4.963	-1.493
189	Kynurenate	Amino acid	2	Ctrl_Untreated_2_2	Ctrl_Untreated_2	0.0	Amino acid derivatives	False			-5.188	0
234	5-Me-Thioadenosine	Amino acid	2	Ctrl_Untreated_2_2	Ctrl_Untreated_2	1.0	SAM metabolism	True	9.141e+04	-0.3531	-0.3531	-0.2571
59	Succinate	Carbon	2	Ctrl_Untreated_2_2	Ctrl_Untreated_2	1.0	TCA cycle	True	4.233e+04	-1.464	-1.464	0.5762
133	Ornithine	Amino acid	2	Ctrl_Untreated_2_2	Ctrl_Untreated_2	1.0	Amino acids degradation intermediates	True	4.02e+05	1.784	1.784	-0.7373
313	5-Oxoproline	Amino acid	2	Ctrl_Untreated_2_2	Ctrl_Untreated_2	1.0	Glutathione derivatives	True	5.629e+05	2.27	2.27	0.2659
724	Pantothenate	Cofactor	2	Ctrl_Untreated_2_2	Ctrl_Untreated_2	1.0	Coenzyme A biosynthesis	True	1.309e+06	3.487	3.487	0.3736
30	Sucrose	Carbon	2	Ctrl_Untreated_2_2	Ctrl_Untreated_2	1.0	Sugars and sugar alcohols	True	7.692e+05	2.72	2.72	0.3432
122	3-OH-Isobutyrate	Amino acid	2	Ctrl_Untreated_2_2	Ctrl_Untreated_2	0.25	Amino acids degradation intermediates	False			-4.628	0
241	4-Acetamidobutanoate	Amino acid	2	Ctrl_Untreated_2_2	Ctrl_Untreated_2	1.0	Polyamine derivatives	True	6.43e+04	-0.8605	-0.8605	0.6469
55	Citrate	Carbon	2	Ctrl_Untreated_2_2	Ctrl_Untreated_2	1.0	TCA cycle	True	7.598e+05	2.702	2.702	-0.3322
338	Guanosine	Nucleotide	2	Ctrl_Untreated_2_2	Ctrl_Untreated_2	1.0	Purine nucleosides	True	6.391e+05	2.453	2.453	0.09225
170	2-Amino-Butyrate	Amino acid	2	Ctrl_Untreated_2_2	Ctrl_Untreated_2	1.0	Amino acid derivatives	True	2.667e+05	1.192	1.192	-0.03192
209	N-Ac-Ala	Amino acid	2	Ctrl_Untreated_2_2	Ctrl_Untreated_2	1.0	N-acetylated amino acids	True	1.142e+04	-3.354	-3.354	-0.08653

Metabolite ID	Name	Super Pathway	Dataset	Sample ID	Group ID	Detection Fraction	Pathway	Detected	Raw Intensity	Log2 Norm Intensity	Norm Imputed	Log2 Ctrl Norm
221	N-Ac-Met	Amino acid	2	Ctrl_Untreated_2_2	Ctrl_Untreated_2	1.0	N-acetylated amino acids	True	5.337e+04	-1.129	-1.129	0.02086
22	N-Ac-Neuraminate	Carbon	2	Ctrl_Untreated_2_2	Ctrl_Untreated_2	1.0	Aminosugar derivatives	True	1.216e+05	0.05881	0.05881	0.2728
346	Urate	Nucleotide	2	Ctrl_Untreated_2_2	Ctrl_Untreated_2	1.0	Purine degradation	True	1.212e+04	-3.268	-3.268	0.4237
90	Arg	Amino acid	2	Ctrl_Untreated_2_2	Ctrl_Untreated_2	1.0	Proteinogenic amino acids	True	4.684e+06	5.326	5.326	0.5442
60	Fumarate	Carbon	2	Ctrl_Untreated_2_2	Ctrl_Untreated_2	1.0	TCA cycle	True	1.427e+05	0.2892	0.2892	0.4459
78	Ser	Amino acid	2	Ctrl_Untreated_2_2	Ctrl_Untreated_2	1.0	Proteinogenic amino acids	True	1.031e+07	6.465	6.465	-0.3616
83	Val	Amino acid	2	Ctrl_Untreated_2_2	Ctrl_Untreated_2	1.0	Proteinogenic amino acids	True	2.053e+07	7.458	7.458	0.2246
734	Pyridoxal	Cofactor	2	Ctrl_Untreated_2_2	Ctrl_Untreated_2	0.75	PLP biosynthesis and salvage	True	7.088e+04	-0.72	-0.72	0.2746
136	Urea	Amino acid	2	Ctrl_Untreated_2_2	Ctrl_Untreated_2	1.0	Amino acids degradation intermediates	True	5.377e+05	2.203	2.203	0.5584
742	Folate	Cofactor	2	Ctrl_Untreated_2_2	Ctrl_Untreated_2	1.0	Folate metabolism	True	9.171e+04	-0.3483	-0.3483	0.1687
729	Riboflavin (Vitamin B2)	Cofactor	2	Ctrl_Untreated_2_2	Ctrl_Untreated_2	1.0	Flavine biosynthesis	True	4.457e+04	-1.389	-1.389	0.1796
91	Pro	Amino acid	2	Ctrl_Untreated_2_2	Ctrl_Untreated_2	1.0	Proteinogenic amino acids	True	1.044e+07	6.482	6.482	-0.239
308	Glutathione, Reduced	Amino acid	2	Ctrl_Untreated_2_2	Ctrl_Untreated_2	1.0	Glutathione	True	8.562e+06	6.196	6.196	-0.2588
706	FAD	Cofactor	2	Ctrl_Untreated_2_2	Ctrl_Untreated_2	0.0	Cofactors	False			-5.096	0
299	gamma-Glu-Tyr	Amino acid	2	Ctrl_Untreated_2_2	Ctrl_Untreated_2	1.0	Gamma-glutamyl dipeptides	True	6.384e+04	-0.8708	-0.8708	0.2964
705	Coenzyme A	Cofactor	2	Ctrl_Untreated_2_2	Ctrl_Untreated_2	1.0	Cofactors	True	9943	-3.554	-3.554	0.1528
342	Hypoxanthine	Nucleotide	2	Ctrl_Untreated_2_2	Ctrl_Untreated_2	1.0	Purine bases	True	1.565e+05	0.4224	0.4224	0.4206
344	Xanthine	Nucleotide	2	Ctrl_Untreated_2_2	Ctrl_Untreated_2	1.0	Purine bases	True	4.945e+04	-1.239	-1.239	0.9996
703	NAD+	Cofactor	2	Ctrl_Untreated_2_2	Ctrl_Untreated_2	1.0	Cofactors	True	1.236e+06	3.405	3.405	-0.154
731	Thiamin (Vitamin B1)	Cofactor	2	Ctrl_Untreated_2_2	Ctrl_Untreated_2	1.0	TPP biosynthesis	True	1.395e+05	0.2573	0.2573	0.4634
102	2-Aminoadipate	Amino acid	2	Ctrl_Untreated_2_2	Ctrl_Untreated_2	1.0	Amino acids biosynthesis intermediates	True	1.654e+05	0.5021	0.5021	-0.2544
77	Gly	Amino acid	2	Ctrl_Untreated_2_2	Ctrl_Untreated_2	1.0	Proteinogenic amino acids	True	1.44e+07	6.946	6.946	-0.282
45	Fructose-6-P	Carbon	2	Ctrl_Untreated_2_2	Ctrl_Untreated_2	0.75	Glycolysis, GNG	True	1.278e+05	0.1304	0.1304	1.566
36	Ribose	Carbon	2	Ctrl_Untreated_2_2	Ctrl_Untreated_2	1.0	Sugars and sugar alcohols	True	3.476e+04	-1.748	-1.748	-0.7504

Metabolite ID	Name	Super Pathway	Dataset	Sample ID	Group ID	Detection Fraction	Pathway	Detected	Raw Intensity	Log2 Norm Intensity	Norm Imputed	Log2 Ctrl Norm
4	GlcNAc 6-P	Carbon	2	Ctrl_Untreated_2_2	Ctrl_Untreated_2	1.0	Aminosugar biosynthesis	True	1.021e+05	-0.193	-0.193	-0.5527
188	Kynurenine	Amino acid	2	Ctrl_Untreated_2_2	Ctrl_Untreated_2	1.0	Amino acid derivatives	True	2.167e+05	0.8923	0.8923	0.9062
63	6-P-Gluconate	Carbon	2	Ctrl_Untreated_2_2	Ctrl_Untreated_2	1.0	Pentose phosphate pathway (PPP)	True	7.777e+04	-0.5861	-0.5861	0.4591
710	Carnitine	Cofactor	2	Ctrl_Untreated_2_2	Ctrl_Untreated_2	1.0	Cofactors	True	5.417e+05	2.214	2.214	-0.3177
725	P-Pantetheine	Cofactor	2	Ctrl_Untreated_2_2	Ctrl_Untreated_2	1.0	Coenzyme A biosynthesis	True	5920	-4.302	-4.302	0.5107
110	N-alpha-Ac-Ornithine	Amino acid	2	Ctrl_Untreated_2_2	Ctrl_Untreated_2	1.0	Amino acids biosynthesis intermediates	True	2.503e+05	1.1	1.1	0.126
116	3-Me-2-Oxo-Valerate	Amino acid	2	Ctrl_Untreated_2_2	Ctrl_Untreated_2	1.0	Amino acids degradation intermediates	True	1.283e+04	-3.186	-3.186	0.3882
155	4-Guanidinobutanoate	Amino acid	2	Ctrl_Untreated_2_2	Ctrl_Untreated_2	1.0	Amino acid derivatives	True	9.065e+04	-0.365	-0.365	0.3544
310	S-Lactoyl-Glutathione	Amino acid	2	Ctrl_Untreated_2_2	Ctrl_Untreated_2	1.0	Glutathione derivatives	True	1.829e+04	-2.674	-2.674	0.9974
34	Ribitol	Carbon	2	Ctrl_Untreated_2_2	Ctrl_Untreated_2	0.75	Sugars and sugar alcohols	False			-2.846	-1.267
707	FMN	Cofactor	2	Ctrl_Untreated_2_2	Ctrl_Untreated_2	1.0	Cofactors	True	1.313e+04	-3.153	-3.153	0.2681
17	Maltose	Carbon	2	Ctrl_Untreated_2_2	Ctrl_Untreated_2	1.0	Glycogen degradation	True	1.432e+05	0.295	0.295	0.3343
18	Maltotriose	Carbon	2	Ctrl_Untreated_2_2	Ctrl_Untreated_2	1.0	Glycogen degradation	True	4.269e+05	1.87	1.87	0.4415
19	Maltotetraose	Carbon	2	Ctrl_Untreated_2_2	Ctrl_Untreated_2	1.0	Glycogen degradation	True	1.467e+05	0.3293	0.3293	0.5197
232	SAH	Amino acid	2	Ctrl_Untreated_2_2	Ctrl_Untreated_2	1.0	SAM metabolism	True	1.38e+04	-3.08	-3.08	0.1456
74	Asp	Amino acid	2	Ctrl_Untreated_2_2	Ctrl_Untreated_2	1.0	Proteinogenic amino acids	True	4.47e+06	5.259	5.259	-0.4007
129	5-Aminovalerate	Amino acid	2	Ctrl_Untreated_2_2	Ctrl_Untreated_2	1.0	Amino acids degradation intermediates	True	5.138e+04	-1.184	-1.184	-0.3712
254	Gly-Val	Amino acid	2	Ctrl_Untreated_2_2	Ctrl_Untreated_2	1.0	Dipeptides	True	1.209e+05	0.05004	0.05004	0.1238
291	gamma-Glu-Leu	Amino acid	2	Ctrl_Untreated_2_2	Ctrl_Untreated_2	0.75	Gamma-glutamyl dipeptides	False			-2.038	-0.9559
173	Met Sulfoxide	Amino acid	2	Ctrl_Untreated_2_2	Ctrl_Untreated_2	1.0	Amino acid derivatives	True	9.808e+04	-0.2514	-0.2514	-0.1126
43	Glucose	Carbon	2	Ctrl_Untreated_2_2	Ctrl_Untreated_2	1.0	Glycolysis, GNG	True	1.96e+07	7.391	7.391	0.5715
249	Gly-Gly	Amino acid	2	Ctrl_Untreated_2_2	Ctrl_Untreated_2	1.0	Dipeptides	True	1.102e+05	-0.08333	-0.08333	-0.3005

Metabolite ID	Name	Super Pathway	Dataset	Sample ID	Group ID	Detection Fraction	Pathway	Detected	Raw Intensity	Log2 Norm Intensity	Norm Imputed	Log2 Ctrl Norm
169	2-OH-Butyrate	Amino acid	2	Ctrl_Untreated_2_2	Ctrl_Untreated_2	0.5	Amino acid derivatives	False			-1.729	-0.2955
98	3-Methyl-2-Oxobutyrate	Amino acid	2	Ctrl_Untreated_2_2	Ctrl_Untreated_2	0.0	Amino acids biosynthesis intermediates	False			-3.759	0
100	4-Me-2-Oxo-Pentanoate	Amino acid	2	Ctrl_Untreated_2_2	Ctrl_Untreated_2	1.0	Amino acids biosynthesis intermediates	True	1.342e+04	-3.12	-3.12	0.1812
253	Gly-Pro	Amino acid	2	Ctrl_Untreated_2_2	Ctrl_Untreated_2	1.0	Dipeptides	True	9.924e+04	-0.2344	-0.2344	0.08668
247	Asp-Phe	Amino acid	2	Ctrl_Untreated_2_2	Ctrl_Untreated_2	1.0	Dipeptides	True	5.337e+04	-1.129	-1.129	-0.188
212	N-Ac-Asp	Amino acid	2	Ctrl_Untreated_2_2	Ctrl_Untreated_2	0.5	N-acetylated amino acids	False			-4.689	-0.1861
720	1-Me-Nicotinamide	Cofactor	2	Ctrl_Untreated_2_2	Ctrl_Untreated_2	1.0	Derivatives of NA, nicotinamide and NAD	True	1.954e+06	4.065	4.065	0.3615
70	Creatine	Carbon	2	Ctrl_Untreated_2_2	Ctrl_Untreated_2	1.0	Creatine energy storage	True	7.678e+06	6.039	6.039	0.2588
309	Glutathione, Oxidized	Amino acid	2	Ctrl_Untreated_2_2	Ctrl_Untreated_2	1.0	Glutathione	True	1.814e+06	3.957	3.957	8.89e-03
44	Glucose 6-P	Carbon	2	Ctrl_Untreated_2_2	Ctrl_Untreated_2	1.0	Glycolysis, GNG	True	4.297e+05	1.88	1.88	1.065
24	Fructose	Carbon	2	Ctrl_Untreated_2_2	Ctrl_Untreated_2	1.0	Sugars and sugar alcohols	True	1.09e+06	3.223	3.223	-0.1376
85	Cys	Amino acid	2	Ctrl_Untreated_2_2	Ctrl_Untreated_2	1.0	Proteinogenic amino acids	True	5.625e+04	-1.054	-1.054	-0.4617
704	NADH	Cofactor	2	Ctrl_Untreated_2_2	Ctrl_Untreated_2	1.0	Cofactors	True	1.535e+04	-2.927	-2.927	-0.998
275	Thr-Phe	Amino acid	2	Ctrl_Untreated_2_2	Ctrl_Untreated_2	1.0	Dipeptides	True	5.64e+04	-1.05	-1.05	-0.1161
738	Pyridoxate	Cofactor	2	Ctrl_Untreated_2_2	Ctrl_Untreated_2	0.75	PLP biosynthesis and salvage	True	8166	-3.838	-3.838	0.8276
177	3-(4-OH-Phenyl)Lactate	Amino acid	2	Ctrl_Untreated_2_2	Ctrl_Untreated_2	1.0	Amino acid derivatives	True	7383	-3.983	-3.983	-0.02999
206	Trans-4-OH-Pro	Amino acid	2	Ctrl_Untreated_2_2	Ctrl_Untreated_2	1.0	Amino acid derivatives	True	9.691e+04	-0.2687	-0.2687	-0.07311
329	AMP	Nucleotide	2	Ctrl_Untreated_2_2	Ctrl_Untreated_2	1.0	Purine nucleotides	True	3.893e+04	-1.585	-1.585	-2.005
345	Guanine	Nucleotide	2	Ctrl_Untreated_2_2	Ctrl_Untreated_2	1.0	Purine bases	True	4.208e+05	1.85	1.85	0.2676
271	pyroGlu-Val	Amino acid	2	Ctrl_Untreated_2_2	Ctrl_Untreated_2	1.0	Dipeptides	True	1.164e+04	-3.326	-3.326	0.3156
279	Val-Glu	Amino acid	2	Ctrl_Untreated_2_2	Ctrl_Untreated_2	1.0	Dipeptides	True	5.688e+04	-1.037	-1.037	-0.2074
183	Phenol Sulfate	Amino acid	2	Ctrl_Untreated_2_2	Ctrl_Untreated_2	0.5	Amino acid derivatives	False			-4.072	-0.3451
740	3-Dehydrocarnitine	Cofactor	2	Ctrl_Untreated_2_2	Ctrl_Untreated_2	1.0	Carnitine biosynthesis	True	7.294e+04	-0.6788	-0.6788	-0.3452

Metabolite ID	Name	Super Pathway	Dataset	Sample ID	Group ID	Detection Fraction	Pathway	Detected	Raw Intensity	Log2 Norm Intensity	Norm Imputed	Log2 Ctrl Norm
145	Pyro-Gln	Amino acid	2	Ctrl_Untreated_2_2	Ctrl_Untreated_2	1.0	Amino acid derivatives	True	2.248e+05	0.9449	0.9449	0.599
197	C-Glycosyl-Trp	Amino acid	2	Ctrl_Untreated_2_2	Ctrl_Untreated_2	1.0	Amino acid derivatives	True	9.603e+04	-0.2819	-0.2819	-0.1382
718	Nicotinamide Riboside	Cofactor	2	Ctrl_Untreated_2_2	Ctrl_Untreated_2	0.5	NAD biosynthesis	True	9.505e+04	-0.2967	-0.2967	1.507
295	gamma-Glu-Phe	Amino acid	2	Ctrl_Untreated_2_2	Ctrl_Untreated_2	1.0	Gamma-glutamyl dipeptides	True	1.147e+05	-0.02547	-0.02547	0.07645
399	Pseudouridine	Nucleotide	2	Ctrl_Untreated_2_2	Ctrl_Untreated_2	1.0	Pyrimidine derivatives in RNAs	True	7204	-4.019	-4.019	0.2221
375	UTP	Nucleotide	2	Ctrl_Untreated_2_2	Ctrl_Untreated_2	0.75	Pyrimidine nucleotides	False			-5.966	-0.5235
20	Erythronate	Carbon	2	Ctrl_Untreated_2_2	Ctrl_Untreated_2	1.0	Aminosugar derivatives	True	3.012e+04	-1.955	-1.955	-0.5131
151	Phenylacetylglycine	Amino acid	2	Ctrl_Untreated_2_2	Ctrl_Untreated_2	1.0	Amino acid derivatives	True	4890	-4.578	-4.578	-0.156
252	Gly-Phe	Amino acid	2	Ctrl_Untreated_2_2	Ctrl_Untreated_2	1.0	Dipeptides	True	8.436e+04	-0.4688	-0.4688	-0.573
251	Gly-Leu	Amino acid	2	Ctrl_Untreated_2_2	Ctrl_Untreated_2	1.0	Dipeptides	True	1.48e+05	0.3417	0.3417	-0.02092
290	gamma-Glu-Ile	Amino acid	2	Ctrl_Untreated_2_2	Ctrl_Untreated_2	0.75	Gamma-glutamyl dipeptides	False			-2.961	-1.429
316	Ophthalmate	Amino acid	2	Ctrl_Untreated_2_2	Ctrl_Untreated_2	1.0	Oxidative stress markers	True	4.649e+04	-1.329	-1.329	-1.583
208	Pro-OH-Pro	Amino acid	2	Ctrl_Untreated_2_2	Ctrl_Untreated_2	1.0	Amino acid derivatives	True	2.414e+05	1.048	1.048	0.5886
352	3'-AMP	Nucleotide	2	Ctrl_Untreated_2_2	Ctrl_Untreated_2	1.0	Purine derivatives in signaling	True	5.932e+04	-0.9769	-0.9769	0.308
314	Cys-Glutathione Disulfide	Amino acid	2	Ctrl_Untreated_2_2	Ctrl_Untreated_2	1.0	Oxidative stress markers	True	8.664e+04	-0.4303	-0.4303	0.9351
39	Threitol	Carbon	2	Ctrl_Untreated_2_2	Ctrl_Untreated_2	0.75	Sugars and sugar alcohols	False			-4.395	-0.3698
31	Ribulose/Xylulose	Carbon	2	Ctrl_Untreated_2_2	Ctrl_Untreated_2	0.5	Sugars and sugar alcohols	False			-5.004	-0.09716
48	DHAP	Carbon	2	Ctrl_Untreated_2_2	Ctrl_Untreated_2	1.0	Glycolysis, GNG	True	3.073e+05	1.396	1.396	-0.3618
182	P-Cresol Sulfate	Amino acid	2	Ctrl_Untreated_2_2	Ctrl_Untreated_2	1.0	Amino acid derivatives	True	1.824e+04	-2.678	-2.678	0.6593
250	Gly-Ile	Amino acid	2	Ctrl_Untreated_2_2	Ctrl_Untreated_2	0.75	Dipeptides	False			-2.655	-1.231
286	gamma-Glu-Glu	Amino acid	2	Ctrl_Untreated_2_2	Ctrl_Untreated_2	1.0	Gamma-glutamyl dipeptides	True	6.724e+04	-0.7961	-0.7961	-0.5655
264	Leu-Leu	Amino acid	2	Ctrl_Untreated_2_2	Ctrl_Untreated_2	1.0	Dipeptides	True	4.496e+04	-1.377	-1.377	-0.3034
203	DiMe-Arg	Amino acid	2	Ctrl_Untreated_2_2	Ctrl_Untreated_2	1.0	Amino acid derivatives	True	5.098e+05	2.127	2.127	1.052

Metabolite ID	Name	Super Pathway	Dataset	Sample ID	Group ID	Detection Fraction	Pathway	Detected	Raw Intensity	Log2 Norm Intensity	Norm Imputed	Log2 Ctrl Norm
47	Fructose 1,6-PP, Glucose 1,6-PP	Carbon	2	Ctrl_Untreated_2_2	Ctrl_Untreated_2	1.0	Glycolysis, GNG	True	6.374e+04	-0.8732	-0.8732	-0.583
224	N-Ac-Ser	Amino acid	2	Ctrl_Untreated_2_2	Ctrl_Untreated_2	1.0	N-acetylated amino acids	True	2.344e+05	1.005	1.005	0.1346
244	Ala-Leu	Amino acid	2	Ctrl_Untreated_2_2	Ctrl_Untreated_2	1.0	Dipeptides	True	1.067e+05	-0.1305	-0.1305	-0.4703
304	Cyclo(Phe-Pro)	Amino acid	2	Ctrl_Untreated_2_2	Ctrl_Untreated_2	1.0	Cyclic dipeptides	True	1.075e+05	-0.1189	-0.1189	0.3528
302	Cyclo(Glu-Glu)	Amino acid	2	Ctrl_Untreated_2_2	Ctrl_Untreated_2	1.0	Cyclic dipeptides	True	7.051e+04	-0.7275	-0.7275	0.4447
303	Cyclo(Leu-Pro)	Amino acid	2	Ctrl_Untreated_2_2	Ctrl_Untreated_2	1.0	Cyclic dipeptides	True	1.713e+05	0.5528	0.5528	0.2799
390	2',3'-cUMP	Nucleotide	2	Ctrl_Untreated_2_2	Ctrl_Untreated_2	1.0	Pyrimidine derivatives in signaling	True	8.737e+04	-0.4183	-0.4183	0.7106
68	Ribulose 5-P / Xylulose 5-P	Carbon	2	Ctrl_Untreated_2_2	Ctrl_Untreated_2	1.0	Pentose phosphate pathway (PPP)	True	1.47e+05	0.3328	0.3328	-0.4363
388	2',3'-cCMP	Nucleotide	2	Ctrl_Untreated_2_2	Ctrl_Untreated_2	1.0	Pyrimidine derivatives in signaling	True	2.011e+05	0.7842	0.7842	0.5307
33	Arabitol/Xylitol	Carbon	2	Ctrl_Untreated_2_2	Ctrl_Untreated_2	0.25	Sugars and sugar alcohols	False			-3.559	-0.05408
268	Phe-Phe	Amino acid	2	Ctrl_Untreated_2_2	Ctrl_Untreated_2	0.75	Dipeptides	False			-2.762	-1.674
245	Ala-Phe	Amino acid	2	Ctrl_Untreated_2_2	Ctrl_Untreated_2	1.0	Dipeptides	True	2.92e+04	-2	-2	-0.5442
373	UMP	Nucleotide	2	Ctrl_Untreated_2_2	Ctrl_Untreated_2	0.75	Pyrimidine nucleotides	False			-3.442	-1.402
282	Val-Leu	Amino acid	2	Ctrl_Untreated_2_2	Ctrl_Untreated_2	1.0	Dipeptides	True	5.751e+04	-1.022	-1.022	-0.6527
258	Ile-Gly	Amino acid	2	Ctrl_Untreated_2_2	Ctrl_Untreated_2	1.0	Dipeptides	True	1.326e+05	0.1836	0.1836	-0.231
259	Ile-Ser	Amino acid	2	Ctrl_Untreated_2_2	Ctrl_Untreated_2	1.0	Dipeptides	True	2.96e+04	-1.98	-1.98	-1.117
269	Phe-Ser	Amino acid	2	Ctrl_Untreated_2_2	Ctrl_Untreated_2	1.0	Dipeptides	True	2.567e+04	-2.185	-2.185	-0.8702
277	Tyr-Ala	Amino acid	2	Ctrl_Untreated_2_2	Ctrl_Untreated_2	1.0	Dipeptides	True	1.095e+05	-0.09239	-0.09239	-0.6625
257	Ile-Gln	Amino acid	2	Ctrl_Untreated_2_2	Ctrl_Untreated_2	1.0	Dipeptides	True	3.401e+04	-1.779	-1.779	-0.8604
261	Leu-Glu	Amino acid	2	Ctrl_Untreated_2_2	Ctrl_Untreated_2	1.0	Dipeptides	True	1.312e+05	0.1681	0.1681	-0.6221
263	Leu-Gly	Amino acid	2	Ctrl_Untreated_2_2	Ctrl_Untreated_2	1.0	Dipeptides	True	6.144e+04	-0.9261	-0.9261	-1.633
256	Ile-Ala	Amino acid	2	Ctrl_Untreated_2_2	Ctrl_Untreated_2	1.0	Dipeptides	True	1.123e+05	-0.05654	-0.05654	-0.01481
274	Thr-Leu	Amino acid	2	Ctrl_Untreated_2_2	Ctrl_Untreated_2	1.0	Dipeptides	True	1.349e+05	0.2082	0.2082	-0.6429
273	Ser-Phe	Amino acid	2	Ctrl_Untreated_2_2	Ctrl_Untreated_2	1.0	Dipeptides	True	3.191e+04	-1.871	-1.871	-0.6495
272	Ser-Leu	Amino acid	2	Ctrl_Untreated_2_2	Ctrl_Untreated_2	1.0	Dipeptides	True	1.119e+05	-0.06127	-0.06127	-0.8001
246	Asp-Leu	Amino acid	2	Ctrl_Untreated_2_2	Ctrl_Untreated_2	1.0	Dipeptides	True	1.13e+05	-0.04658	-0.04658	0.02748
76	Gln	Amino acid	2	Ctrl_Untreated_2_3	Ctrl_Untreated_2	1.0	Proteinogenic amino acids	True	2.785e+07	6.806	6.806	-0.1463

Metabolite ID	Name	Super Pathway	Dataset	Sample ID	Group ID	Detection Fraction	Pathway	Detected	Raw Intensity	Log2 Norm Intensity	Norm Imputed	Log2 Ctrl Norm
89	Trp	Amino acid	2	Ctrl_Untreated_2_3	Ctrl_Untreated_2	1.0	Proteinogenic amino acids	True	1.662e+07	6.061	6.061	0.09484
723	beta-Ala	Cofactor	2	Ctrl_Untreated_2_3	Ctrl_Untreated_2	1.0	Coenzyme A biosynthesis	True	3.051e+05	0.2937	0.2937	-0.03257
75	Glu	Amino acid	2	Ctrl_Untreated_2_3	Ctrl_Untreated_2	1.0	Proteinogenic amino acids	True	1.216e+07	5.611	5.611	-0.3372
80	His	Amino acid	2	Ctrl_Untreated_2_3	Ctrl_Untreated_2	1.0	Proteinogenic amino acids	True	3.101e+05	0.3173	0.3173	0.1031
82	Leu	Amino acid	2	Ctrl_Untreated_2_3	Ctrl_Untreated_2	1.0	Proteinogenic amino acids	True	7.554e+07	8.246	8.246	0.08545
87	Phe	Amino acid	2	Ctrl_Untreated_2_3	Ctrl_Untreated_2	1.0	Proteinogenic amino acids	True	6.62e+07	8.055	8.055	0.1633
236	Spermidine	Amino acid	2	Ctrl_Untreated_2_3	Ctrl_Untreated_2	1.0	Polyamines	True	2.376e+06	3.255	3.255	0.427
73	Asn	Amino acid	2	Ctrl_Untreated_2_3	Ctrl_Untreated_2	1.0	Proteinogenic amino acids	True	2.751e+06	3.467	3.467	0.7176
243	Creatinine	Amino acid	2	Ctrl_Untreated_2_3	Ctrl_Untreated_2	1.0	Creatine degradation	True	6.168e+05	1.309	1.309	-0.2613
376	Cytidine	Nucleotide	2	Ctrl_Untreated_2_3	Ctrl_Untreated_2	1.0	Pyrimidine nucleosides	True	4.078e+05	0.7122	0.7122	1.297
41	Lactate	Carbon	2	Ctrl_Untreated_2_3	Ctrl_Untreated_2	1.0	Respiratory carbon sources	True	7.471e+07	8.23	8.23	0.4075
93	3-P-Ser	Amino acid	2	Ctrl_Untreated_2_3	Ctrl_Untreated_2	1.0	Amino acids biosynthesis intermediates	True	7956	-4.967	-4.967	-1.709
343	Adenine	Nucleotide	2	Ctrl_Untreated_2_3	Ctrl_Untreated_2	1.0	Purine bases	True	1.315e+05	-0.9207	-0.9207	0.5429
336	Adenosine	Nucleotide	2	Ctrl_Untreated_2_3	Ctrl_Untreated_2	1.0	Purine nucleosides	True	2.415e+06	3.278	3.278	0.1411
29	Raffinose	Carbon	2	Ctrl_Untreated_2_3	Ctrl_Untreated_2	1.0	Sugars and sugar alcohols	True	5.193e+04	-2.261	-2.261	-0.08105
717	Nicotinamide	Cofactor	2	Ctrl_Untreated_2_3	Ctrl_Untreated_2	1.0	NAD biosynthesis	True	1.335e+06	2.423	2.423	0.02652
51	PEP	Carbon	2	Ctrl_Untreated_2_3	Ctrl_Untreated_2	1.0	Glycolysis, GNG	True	2.479e+05	-6.06e-03	-6.06e-03	1.066
52	Pyruvate	Carbon	2	Ctrl_Untreated_2_3	Ctrl_Untreated_2	0.75	Glycolysis, GNG	True	6.937e+04	-1.843	-1.843	0.5306
237	Spermine	Amino acid	2	Ctrl_Untreated_2_3	Ctrl_Untreated_2	1.0	Polyamines	True	2.736e+06	3.458	3.458	0.06983
385	Uracil	Nucleotide	2	Ctrl_Untreated_2_3	Ctrl_Untreated_2	1.0	Pyrimidine bases	True	1.311e+05	-0.9252	-0.9252	0.8017
377	Uridine	Nucleotide	2	Ctrl_Untreated_2_3	Ctrl_Untreated_2	1.0	Pyrimidine nucleosides	True	1.842e+06	2.887	2.887	4.92e-03
112	trans-Urocanate	Amino acid	2	Ctrl_Untreated_2_3	Ctrl_Untreated_2	0.5	Amino acids degradation intermediates	True	2.612e+04	-3.252	-3.252	-0.1347
737	Pyridoxine (Vitamin B6)	Cofactor	2	Ctrl_Untreated_2_3	Ctrl_Untreated_2	1.0	PLP biosynthesis and salvage	True	2.14e+06	3.104	3.104	-0.196

Metabolite ID	Name	Super Pathway	Dataset	Sample ID	Group ID	Detection Fraction	Pathway	Detected	Raw Intensity	Log2 Norm Intensity	Norm Imputed	Log2 Ctrl Norm
348	Allantoin	Nucleotide	2	Ctrl_Untreated_2_3	Ctrl_Untreated_2	1.0	Purine degradation	True	2.802e+04	-3.151	-3.151	-0.3215
335	Inosine	Nucleotide	2	Ctrl_Untreated_2_3	Ctrl_Untreated_2	1.0	Purine nucleosides	True	2.052e+06	3.043	3.043	0.1823
81	Ile	Amino acid	2	Ctrl_Untreated_2_3	Ctrl_Untreated_2	1.0	Proteinogenic amino acids	True	5.834e+07	7.873	7.873	0.01717
72	Ala	Amino acid	2	Ctrl_Untreated_2_3	Ctrl_Untreated_2	1.0	Proteinogenic amino acids	True	8.028e+07	8.333	8.333	0.5994
79	Thr	Amino acid	2	Ctrl_Untreated_2_3	Ctrl_Untreated_2	1.0	Proteinogenic amino acids	True	2.375e+07	6.576	6.576	0.6139
88	Tyr	Amino acid	2	Ctrl_Untreated_2_3	Ctrl_Untreated_2	1.0	Proteinogenic amino acids	True	3.179e+07	6.997	6.997	0.1545
84	Lys	Amino acid	2	Ctrl_Untreated_2_3	Ctrl_Untreated_2	1.0	Proteinogenic amino acids	True	2.803e+06	3.493	3.493	-0.2258
86	Met	Amino acid	2	Ctrl_Untreated_2_3	Ctrl_Untreated_2	1.0	Proteinogenic amino acids	True	1.754e+07	6.139	6.139	0.3519
61	Malate	Carbon	2	Ctrl_Untreated_2_3	Ctrl_Untreated_2	1.0	TCA cycle	True	7.346e+05	1.561	1.561	0.6647
235	Putrescine	Amino acid	2	Ctrl_Untreated_2_3	Ctrl_Untreated_2	1.0	Polyamines	True	1.79e+05	-0.4753	-0.4753	1.092
49	3-P-Glycerate	Carbon	2	Ctrl_Untreated_2_3	Ctrl_Untreated_2	1.0	Glycolysis, GNG	True	2.207e+06	3.148	3.148	0.6858
139	GABA	Amino acid	2	Ctrl_Untreated_2_3	Ctrl_Untreated_2	0.75	Amino acid derivatives	True	5.057e+04	-2.299	-2.299	1.171
189	Kynurenate	Amino acid	2	Ctrl_Untreated_2_3	Ctrl_Untreated_2	0.0	Amino acid derivatives	False			-5.188	0
234	5-Me-Thioadenosine	Amino acid	2	Ctrl_Untreated_2_3	Ctrl_Untreated_2	1.0	SAM metabolism	True	2.29e+05	-0.1202	-0.1202	-0.02423
59	Succinate	Carbon	2	Ctrl_Untreated_2_3	Ctrl_Untreated_2	1.0	TCA cycle	True	4.676e+04	-2.412	-2.412	-0.3724
133	Ornithine	Amino acid	2	Ctrl_Untreated_2_3	Ctrl_Untreated_2	1.0	Amino acids degradation intermediates	True	2.226e+06	3.161	3.161	0.6395
313	5-Oxoproline	Amino acid	2	Ctrl_Untreated_2_3	Ctrl_Untreated_2	1.0	Glutathione derivatives	True	1.051e+06	2.079	2.079	0.07502
724	Pantothenate	Cofactor	2	Ctrl_Untreated_2_3	Ctrl_Untreated_2	1.0	Coenzyme A biosynthesis	True	1.945e+06	2.966	2.966	-0.147
30	Sucrose	Carbon	2	Ctrl_Untreated_2_3	Ctrl_Untreated_2	1.0	Sugars and sugar alcohols	True	1.282e+06	2.365	2.365	-0.01152
122	3-OH-Isobutyrate	Amino acid	2	Ctrl_Untreated_2_3	Ctrl_Untreated_2	0.25	Amino acids degradation intermediates	True	1.007e+04	-4.628	-4.628	0
241	4-Acetamidobutanoate	Amino acid	2	Ctrl_Untreated_2_3	Ctrl_Untreated_2	1.0	Polyamine derivatives	True	7.35e+04	-1.76	-1.76	-0.2524
55	Citrate	Carbon	2	Ctrl_Untreated_2_3	Ctrl_Untreated_2	1.0	TCA cycle	True	2.806e+06	3.495	3.495	0.4606
338	Guanosine	Nucleotide	2	Ctrl_Untreated_2_3	Ctrl_Untreated_2	1.0	Purine nucleosides	True	1.455e+06	2.548	2.548	0.1873

Metabolite ID	Name	Super Pathway	Dataset	Sample ID	Group ID	Detection Fraction	Pathway	Detected	Raw Intensity	Log2 Norm Intensity	Norm Imputed	Log2 Ctrl Norm
170	2-Amino-Butyrate	Amino acid	2	Ctrl_Untreated_2_3	Ctrl_Untreated_2	1.0	Amino acid derivatives	True	7.878e+05	1.662	1.662	0.4385
209	N-Ac-Ala	Amino acid	2	Ctrl_Untreated_2_3	Ctrl_Untreated_2	1.0	N-acetylated amino acids	True	3.396e+04	-2.874	-2.874	0.3939
221	N-Ac-Met	Amino acid	2	Ctrl_Untreated_2_3	Ctrl_Untreated_2	1.0	N-acetylated amino acids	True	1.329e+05	-0.9053	-0.9053	0.2449
22	N-Ac-Neuraminate	Carbon	2	Ctrl_Untreated_2_3	Ctrl_Untreated_2	1.0	Aminosugar derivatives	True	2.499e+05	6.06e-03	6.06e-03	0.2201
346	Urate	Nucleotide	2	Ctrl_Untreated_2_3	Ctrl_Untreated_2	1.0	Purine degradation	True	1.904e+04	-3.708	-3.708	-0.01631
90	Arg	Amino acid	2	Ctrl_Untreated_2_3	Ctrl_Untreated_2	1.0	Proteinogenic amino acids	True	5.866e+06	4.559	4.559	-0.2233
60	Fumarate	Carbon	2	Ctrl_Untreated_2_3	Ctrl_Untreated_2	1.0	TCA cycle	True	2.688e+05	0.1109	0.1109	0.2676
78	Ser	Amino acid	2	Ctrl_Untreated_2_3	Ctrl_Untreated_2	1.0	Proteinogenic amino acids	True	4.448e+07	7.481	7.481	0.6547
83	Val	Amino acid	2	Ctrl_Untreated_2_3	Ctrl_Untreated_2	1.0	Proteinogenic amino acids	True	3.529e+07	7.147	7.147	-0.08646
734	Pyridoxal	Cofactor	2	Ctrl_Untreated_2_3	Ctrl_Untreated_2	0.75	PLP biosynthesis and salvage	False			-1.437	-0.4421
136	Urea	Amino acid	2	Ctrl_Untreated_2_3	Ctrl_Untreated_2	1.0	Amino acids degradation intermediates	True	8.555e+05	1.781	1.781	0.1362
742	Folate	Cofactor	2	Ctrl_Untreated_2_3	Ctrl_Untreated_2	1.0	Folate metabolism	True	1.76e+05	-0.4997	-0.4997	0.01729
729	Riboflavin (Vitamin B2)	Cofactor	2	Ctrl_Untreated_2_3	Ctrl_Untreated_2	1.0	Flavine biosynthesis	True	6.864e+04	-1.858	-1.858	-0.2893
91	Pro	Amino acid	2	Ctrl_Untreated_2_3	Ctrl_Untreated_2	1.0	Proteinogenic amino acids	True	3.316e+07	7.058	7.058	0.3366
308	Glutathione, Reduced	Amino acid	2	Ctrl_Untreated_2_3	Ctrl_Untreated_2	1.0	Glutathione	True	2.686e+07	6.754	6.754	0.2987
706	FAD	Cofactor	2	Ctrl_Untreated_2_3	Ctrl_Untreated_2	0.0	Cofactors	False			-5.096	0
299	gamma-Glu-Tyr	Amino acid	2	Ctrl_Untreated_2_3	Ctrl_Untreated_2	1.0	Gamma-glutamyl dipeptides	True	1.634e+05	-0.6072	-0.6072	0.5601
705	Coenzyme A	Cofactor	2	Ctrl_Untreated_2_3	Ctrl_Untreated_2	1.0	Cofactors	True	1.45e+04	-4.102	-4.102	-0.3951
342	Hypoxanthine	Nucleotide	2	Ctrl_Untreated_2_3	Ctrl_Untreated_2	1.0	Purine bases	True	2.952e+05	0.246	0.246	0.2442
344	Xanthine	Nucleotide	2	Ctrl_Untreated_2_3	Ctrl_Untreated_2	1.0	Purine bases	True	4.482e+04	-2.473	-2.473	-0.2344
703	NAD+	Cofactor	2	Ctrl_Untreated_2_3	Ctrl_Untreated_2	1.0	Cofactors	True	3.2e+06	3.685	3.685	0.1261
731	Thiamin (Vitamin B1)	Cofactor	2	Ctrl_Untreated_2_3	Ctrl_Untreated_2	1.0	TPP biosynthesis	True	2.233e+05	-0.1568	-0.1568	0.04928
102	2-Aminoadipate	Amino acid	2	Ctrl_Untreated_2_3	Ctrl_Untreated_2	1.0	Amino acids biosynthesis intermediates	True	4.863e+05	0.9663	0.9663	0.2098
77	Gly	Amino acid	2	Ctrl_Untreated_2_3	Ctrl_Untreated_2	1.0	Proteinogenic amino acids	True	4.916e+07	7.626	7.626	0.3976

Metabolite ID	Name	Super Pathway	Dataset	Sample ID	Group ID	Detection Fraction	Pathway	Detected	Raw Intensity	Log2 Norm Intensity	Norm Imputed	Log2 Ctrl Norm
45	Fructose-6-P	Carbon	2	Ctrl_Untreated_2_3	Ctrl_Untreated_2	0.75	Glycolysis, GNG	True	1.098e+05	-1.181	-1.181	0.2547
36	Ribose	Carbon	2	Ctrl_Untreated_2_3	Ctrl_Untreated_2	1.0	Sugars and sugar alcohols	True	2.39e+05	-0.05858	-0.05858	0.9389
4	GlcNAc 6-P	Carbon	2	Ctrl_Untreated_2_3	Ctrl_Untreated_2	1.0	Aminosugar biosynthesis	True	3.934e+05	0.6605	0.6605	0.3008
188	Kynurenine	Amino acid	2	Ctrl_Untreated_2_3	Ctrl_Untreated_2	1.0	Amino acid derivatives	True	1.291e+05	-0.9472	-0.9472	-0.9333
63	6-P-Gluconate	Carbon	2	Ctrl_Untreated_2_3	Ctrl_Untreated_2	1.0	Pentose phosphate pathway (PPP)	True	1.251e+05	-0.992	-0.992	0.05323
710	Carnitine	Cofactor	2	Ctrl_Untreated_2_3	Ctrl_Untreated_2	1.0	Cofactors	True	1.597e+06	2.682	2.682	0.1498
725	P-Pantetheine	Cofactor	2	Ctrl_Untreated_2_3	Ctrl_Untreated_2	1.0	Coenzyme A biosynthesis	True	9683	-4.684	-4.684	0.1285
110	N-alpha-Ac-Ornithine	Amino acid	2	Ctrl_Untreated_2_3	Ctrl_Untreated_2	1.0	Amino acids biosynthesis intermediates	True	5.647e+05	1.182	1.182	0.2078
116	3-Me-2-Oxo-Valerate	Amino acid	2	Ctrl_Untreated_2_3	Ctrl_Untreated_2	1.0	Amino acids degradation intermediates	True	1.496e+04	-4.057	-4.057	-0.482
155	4-Guanidinobutanoate	Amino acid	2	Ctrl_Untreated_2_3	Ctrl_Untreated_2	1.0	Amino acid derivatives	True	1.013e+05	-1.297	-1.297	-0.5777
310	S-Lactoyl-Glutathione	Amino acid	2	Ctrl_Untreated_2_3	Ctrl_Untreated_2	1.0	Glutathione derivatives	True	1.02e+04	-4.609	-4.609	-0.9376
34	Ribitol	Carbon	2	Ctrl_Untreated_2_3	Ctrl_Untreated_2	0.75	Sugars and sugar alcohols	True	1.428e+05	-0.8021	-0.8021	0.7767
707	FMN	Cofactor	2	Ctrl_Untreated_2_3	Ctrl_Untreated_2	1.0	Cofactors	True	2.596e+04	-3.261	-3.261	0.1597
17	Maltose	Carbon	2	Ctrl_Untreated_2_3	Ctrl_Untreated_2	1.0	Glycogen degradation	True	2.561e+05	0.041	0.041	0.08038
18	Maltotriose	Carbon	2	Ctrl_Untreated_2_3	Ctrl_Untreated_2	1.0	Glycogen degradation	True	7.211e+05	1.535	1.535	0.1057
19	Maltotetraose	Carbon	2	Ctrl_Untreated_2_3	Ctrl_Untreated_2	1.0	Glycogen degradation	True	2.017e+05	-0.3037	-0.3037	-0.1133
232	SAH	Amino acid	2	Ctrl_Untreated_2_3	Ctrl_Untreated_2	1.0	SAM metabolism	True	2.974e+04	-3.065	-3.065	0.1612
74	Asp	Amino acid	2	Ctrl_Untreated_2_3	Ctrl_Untreated_2	1.0	Proteinogenic amino acids	True	1.662e+07	6.062	6.062	0.4021
129	5-Aminovalerate	Amino acid	2	Ctrl_Untreated_2_3	Ctrl_Untreated_2	1.0	Amino acids degradation intermediates	True	2.461e+05	-0.01658	-0.01658	0.7965
254	Gly-Val	Amino acid	2	Ctrl_Untreated_2_3	Ctrl_Untreated_2	1.0	Dipeptides	True	2.878e+05	0.2097	0.2097	0.2835
291	gamma-Glu-Leu	Amino acid	2	Ctrl_Untreated_2_3	Ctrl_Untreated_2	0.75	Gamma-glutamyl dipeptides	True	2.178e+05	-0.1927	-0.1927	0.8893

Metabolite ID	Name	Super Pathway	Dataset	Sample ID	Group ID	Detection Fraction	Pathway	Detected	Raw Intensity	Log2 Norm Intensity	Norm Imputed	Log2 Ctrl Norm
173	Met Sulfoxide	Amino acid	2	Ctrl_Untreated_2_3	Ctrl_Untreated_2	1.0	Amino acid derivatives	True	1.847e+05	-0.4306	-0.4306	-0.2918
43	Glucose	Carbon	2	Ctrl_Untreated_2_3	Ctrl_Untreated_2	1.0	Glycolysis, GNG	True	2.633e+07	6.725	6.725	-0.0946
249	Gly-Gly	Amino acid	2	Ctrl_Untreated_2_3	Ctrl_Untreated_2	1.0	Dipeptides	True	3.672e+05	0.5609	0.5609	0.3437
169	2-OH-Butyrate	Amino acid	2	Ctrl_Untreated_2_3	Ctrl_Untreated_2	0.5	Amino acid derivatives	True	1.309e+05	-0.9266	-0.9266	0.5073
98	3-Methyl-2-Oxobutyrate	Amino acid	2	Ctrl_Untreated_2_3	Ctrl_Untreated_2	0.0	Amino acids biosynthesis intermediates	False			-3.759	0
100	4-Me-2-Oxo-Pentanoate	Amino acid	2	Ctrl_Untreated_2_3	Ctrl_Untreated_2	1.0	Amino acids biosynthesis intermediates	True	3.555e+04	-2.808	-2.808	0.494
253	Gly-Pro	Amino acid	2	Ctrl_Untreated_2_3	Ctrl_Untreated_2	1.0	Dipeptides	True	2.179e+05	-0.1916	-0.1916	0.1295
247	Asp-Phe	Amino acid	2	Ctrl_Untreated_2_3	Ctrl_Untreated_2	1.0	Dipeptides	True	1.222e+05	-1.026	-1.026	-0.08505
212	N-Ac-Asp	Amino acid	2	Ctrl_Untreated_2_3	Ctrl_Untreated_2	0.5	N-acetylated amino acids	True	1.617e+04	-3.944	-3.944	0.5583
720	1-Me-Nicotinamide	Cofactor	2	Ctrl_Untreated_2_3	Ctrl_Untreated_2	1.0	Derivatives of NA, nicotinamide and NAD	True	2.54e+06	3.351	3.351	-0.3526
70	Creatine	Carbon	2	Ctrl_Untreated_2_3	Ctrl_Untreated_2	1.0	Creatine energy storage	True	1.228e+07	5.624	5.624	-0.1563
309	Glutathione, Oxidized	Amino acid	2	Ctrl_Untreated_2_3	Ctrl_Untreated_2	1.0	Glutathione	True	4.111e+06	4.046	4.046	0.09751
44	Glucose 6-P	Carbon	2	Ctrl_Untreated_2_3	Ctrl_Untreated_2	1.0	Glycolysis, GNG	True	6.997e+05	1.491	1.491	0.6765
24	Fructose	Carbon	2	Ctrl_Untreated_2_3	Ctrl_Untreated_2	1.0	Sugars and sugar alcohols	True	3.135e+06	3.655	3.655	0.2941
85	Cys	Amino acid	2	Ctrl_Untreated_2_3	Ctrl_Untreated_2	1.0	Proteinogenic amino acids	True	2.21e+05	-0.1716	-0.1716	0.4203
704	NADH	Cofactor	2	Ctrl_Untreated_2_3	Ctrl_Untreated_2	1.0	Cofactors	True	6.403e+04	-1.959	-1.959	-0.02953
275	Thr-Phe	Amino acid	2	Ctrl_Untreated_2_3	Ctrl_Untreated_2	1.0	Dipeptides	True	1.107e+05	-1.168	-1.168	-0.2348
738	Pyridoxate	Cofactor	2	Ctrl_Untreated_2_3	Ctrl_Untreated_2	0.75	PLP biosynthesis and salvage	True	6533	-5.252	-5.252	-0.5865
177	3-(4-OH-Phenyl)Lactate	Amino acid	2	Ctrl_Untreated_2_3	Ctrl_Untreated_2	1.0	Amino acid derivatives	True	1.778e+04	-3.807	-3.807	0.1459
206	Trans-4-OH-Pro	Amino acid	2	Ctrl_Untreated_2_3	Ctrl_Untreated_2	1.0	Amino acid derivatives	True	2.128e+05	-0.2264	-0.2264	-0.03081
329	AMP	Nucleotide	2	Ctrl_Untreated_2_3	Ctrl_Untreated_2	1.0	Purine nucleotides	True	1.713e+05	-0.5391	-0.5391	-0.9597
345	Guanine	Nucleotide	2	Ctrl_Untreated_2_3	Ctrl_Untreated_2	1.0	Purine bases	True	1.087e+06	2.127	2.127	0.5449
271	pyroGlu-Val	Amino acid	2	Ctrl_Untreated_2_3	Ctrl_Untreated_2	1.0	Dipeptides	True	2.024e+04	-3.62	-3.62	0.0213
279	Val-Glu	Amino acid	2	Ctrl_Untreated_2_3	Ctrl_Untreated_2	1.0	Dipeptides	True	1.162e+05	-1.099	-1.099	-0.2688

Metabolite ID	Name	Super Pathway	Dataset	Sample ID	Group ID	Detection Fraction	Pathway	Detected	Raw Intensity	Log2 Norm Intensity	Norm Imputed	Log2 Ctrl Norm
183	Phenol Sulfate	Amino acid	2	Ctrl_Untreated_2_3	Ctrl_Untreated_2	0.5	Amino acid derivatives	False			-4.072	-0.3451
740	3-Dehydrocarnitine	Cofactor	2	Ctrl_Untreated_2_3	Ctrl_Untreated_2	1.0	Carnitine biosynthesis	True	2.129e+05	-0.2252	-0.2252	0.1084
145	Pyro-Gln	Amino acid	2	Ctrl_Untreated_2_3	Ctrl_Untreated_2	1.0	Amino acid derivatives	True	1.847e+05	-0.4307	-0.4307	-0.7767
197	C-Glycosyl-Trp	Amino acid	2	Ctrl_Untreated_2_3	Ctrl_Untreated_2	1.0	Amino acid derivatives	True	2.021e+05	-0.3004	-0.3004	-0.1567
718	Nicotinamide Riboside	Cofactor	2	Ctrl_Untreated_2_3	Ctrl_Untreated_2	0.5	NAD biosynthesis	True	5.033e+04	-2.306	-2.306	-0.5023
295	gamma-Glu-Phe	Amino acid	2	Ctrl_Untreated_2_3	Ctrl_Untreated_2	1.0	Gamma-glutamyl dipeptides	True	2.808e+05	0.1741	0.1741	0.276
399	Pseudouridine	Nucleotide	2	Ctrl_Untreated_2_3	Ctrl_Untreated_2	1.0	Pyrimidine derivatives in RNAs	True	1.051e+04	-4.566	-4.566	-0.3249
375	UTP	Nucleotide	2	Ctrl_Untreated_2_3	Ctrl_Untreated_2	0.75	Pyrimidine nucleotides	True	8690	-4.84	-4.84	0.6028
20	Erythronate	Carbon	2	Ctrl_Untreated_2_3	Ctrl_Untreated_2	1.0	Aminosugar derivatives	True	1.456e+05	-0.7732	-0.7732	0.6683
151	Phenylacetylglycine	Amino acid	2	Ctrl_Untreated_2_3	Ctrl_Untreated_2	1.0	Amino acid derivatives	True	4716	-5.722	-5.722	-1.3
252	Gly-Phe	Amino acid	2	Ctrl_Untreated_2_3	Ctrl_Untreated_2	1.0	Dipeptides	True	2.127e+05	-0.2266	-0.2266	-0.3308
251	Gly-Leu	Amino acid	2	Ctrl_Untreated_2_3	Ctrl_Untreated_2	1.0	Dipeptides	True	2.878e+05	0.2093	0.2093	-0.1533
290	gamma-Glu-Ile	Amino acid	2	Ctrl_Untreated_2_3	Ctrl_Untreated_2	0.75	Gamma-glutamyl dipeptides	True	1.387e+05	-0.8437	-0.8437	0.6875
316	Ophthalmate	Amino acid	2	Ctrl_Untreated_2_3	Ctrl_Untreated_2	1.0	Oxidative stress markers	True	6.907e+05	1.472	1.472	1.218
208	Pro-OH-Pro	Amino acid	2	Ctrl_Untreated_2_3	Ctrl_Untreated_2	1.0	Amino acid derivatives	True	2.776e+05	0.1575	0.1575	-0.3022
352	3'-AMP	Nucleotide	2	Ctrl_Untreated_2_3	Ctrl_Untreated_2	1.0	Purine derivatives in signaling	True	8.093e+04	-1.621	-1.621	-0.3359
314	Cys-Glutathione Disulfide	Amino acid	2	Ctrl_Untreated_2_3	Ctrl_Untreated_2	1.0	Oxidative stress markers	True	4.684e+04	-2.41	-2.41	-1.044
39	Threitol	Carbon	2	Ctrl_Untreated_2_3	Ctrl_Untreated_2	0.75	Sugars and sugar alcohols	True	1.183e+04	-4.395	-4.395	-0.3698
31	Ribulose/Xylulose	Carbon	2	Ctrl_Untreated_2_3	Ctrl_Untreated_2	0.5	Sugars and sugar alcohols	True	7756	-5.004	-5.004	-0.09716
48	DHAP	Carbon	2	Ctrl_Untreated_2_3	Ctrl_Untreated_2	1.0	Glycolysis, GNG	True	9.49e+05	1.931	1.931	0.1728
182	P-Cresol Sulfate	Amino acid	2	Ctrl_Untreated_2_3	Ctrl_Untreated_2	1.0	Amino acid derivatives	True	1.233e+04	-4.335	-4.335	-0.9976
250	Gly-Ile	Amino acid	2	Ctrl_Untreated_2_3	Ctrl_Untreated_2	0.75	Dipeptides	True	8.055e+04	-1.628	-1.628	-0.2036

Metabolite ID	Name	Super Pathway	Dataset	Sample ID	Group ID	Detection Fraction	Pathway	Detected	Raw Intensity	Log2 Norm Intensity	Norm Imputed	Log2 Ctrl Norm
286	gamma-Glu-Glu	Amino acid	2	Ctrl_Untreated_2_3	Ctrl_Untreated_2	1.0	Gamma-glutamyl dipeptides	True	3.742e+05	0.5884	0.5884	0.819
264	Leu-Leu	Amino acid	2	Ctrl_Untreated_2_3	Ctrl_Untreated_2	1.0	Dipeptides	True	7.901e+04	-1.656	-1.656	-0.5822
203	DiMe-Arg	Amino acid	2	Ctrl_Untreated_2_3	Ctrl_Untreated_2	1.0	Amino acid derivatives	True	4.012e+05	0.6888	0.6888	-0.3856
47	Fructose 1,6-PP, Glucose 1,6-PP	Carbon	2	Ctrl_Untreated_2_3	Ctrl_Untreated_2	1.0	Glycolysis, GNG	True	2.321e+05	-0.1009	-0.1009	0.1892
224	N-Ac-Ser	Amino acid	2	Ctrl_Untreated_2_3	Ctrl_Untreated_2	1.0	N-acetylated amino acids	True	4.679e+05	0.9107	0.9107	0.03994
244	Ala-Leu	Amino acid	2	Ctrl_Untreated_2_3	Ctrl_Untreated_2	1.0	Dipeptides	True	3.599e+05	0.5319	0.5319	0.1922
304	Cyclo(Phe-Pro)	Amino acid	2	Ctrl_Untreated_2_3	Ctrl_Untreated_2	1.0	Cyclic dipeptides	True	1.695e+05	-0.5543	-0.5543	-0.0825
302	Cyclo(Glu-Glu)	Amino acid	2	Ctrl_Untreated_2_3	Ctrl_Untreated_2	1.0	Cyclic dipeptides	True	1.131e+05	-1.138	-1.138	0.03433
303	Cyclo(Leu-Pro)	Amino acid	2	Ctrl_Untreated_2_3	Ctrl_Untreated_2	1.0	Cyclic dipeptides	True	3.09e+05	0.312	0.312	0.03913
390	2',3'-cUMP	Nucleotide	2	Ctrl_Untreated_2_3	Ctrl_Untreated_2	1.0	Pyrimidine derivatives in signaling	True	1.001e+05	-1.313	-1.313	-0.1845
68	Ribulose 5-P / Xylulose 5-P	Carbon	2	Ctrl_Untreated_2_3	Ctrl_Untreated_2	1.0	Pentose phosphate pathway (PPP)	True	7.63e+05	1.616	1.616	0.8471
388	2',3'-cCMP	Nucleotide	2	Ctrl_Untreated_2_3	Ctrl_Untreated_2	1.0	Pyrimidine derivatives in signaling	True	2.764e+05	0.1511	0.1511	-0.1023
33	Arabitol/Xylitol	Carbon	2	Ctrl_Untreated_2_3	Ctrl_Untreated_2	0.25	Sugars and sugar alcohols	False			-3.559	-0.05408
268	Phe-Phe	Amino acid	2	Ctrl_Untreated_2_3	Ctrl_Untreated_2	0.75	Dipeptides	True	8.451e+04	-1.558	-1.558	-0.4707
245	Ala-Phe	Amino acid	2	Ctrl_Untreated_2_3	Ctrl_Untreated_2	1.0	Dipeptides	True	1.006e+05	-1.308	-1.308	0.1478
373	UMP	Nucleotide	2	Ctrl_Untreated_2_3	Ctrl_Untreated_2	0.75	Pyrimidine nucleotides	True	2.426e+04	-3.359	-3.359	-1.319
282	Val-Leu	Amino acid	2	Ctrl_Untreated_2_3	Ctrl_Untreated_2	1.0	Dipeptides	True	2.002e+05	-0.3143	-0.3143	0.05448
258	Ile-Gly	Amino acid	2	Ctrl_Untreated_2_3	Ctrl_Untreated_2	1.0	Dipeptides	True	3.357e+05	0.4318	0.4318	0.01716
259	Ile-Ser	Amino acid	2	Ctrl_Untreated_2_3	Ctrl_Untreated_2	1.0	Dipeptides	True	1.53e+05	-0.7017	-0.7017	0.1613
269	Phe-Ser	Amino acid	2	Ctrl_Untreated_2_3	Ctrl_Untreated_2	1.0	Dipeptides	True	1.247e+05	-0.9972	-0.9972	0.3178
277	Tyr-Ala	Amino acid	2	Ctrl_Untreated_2_3	Ctrl_Untreated_2	1.0	Dipeptides	True	3.896e+05	0.6463	0.6463	0.07621
257	Ile-Gln	Amino acid	2	Ctrl_Untreated_2_3	Ctrl_Untreated_2	1.0	Dipeptides	True	1.312e+05	-0.9241	-0.9241	-5.28e-03
261	Leu-Glu	Amino acid	2	Ctrl_Untreated_2_3	Ctrl_Untreated_2	1.0	Dipeptides	True	5.364e+05	1.108	1.108	0.3175
263	Leu-Gly	Amino acid	2	Ctrl_Untreated_2_3	Ctrl_Untreated_2	1.0	Dipeptides	True	4.507e+05	0.8565	0.8565	0.1496
256	Ile-Ala	Amino acid	2	Ctrl_Untreated_2_3	Ctrl_Untreated_2	1.0	Dipeptides	True	1.7e+05	-0.55	-0.55	-0.5083
274	Thr-Leu	Amino acid	2	Ctrl_Untreated_2_3	Ctrl_Untreated_2	1.0	Dipeptides	True	4.285e+05	0.7839	0.7839	-0.06728
273	Ser-Phe	Amino acid	2	Ctrl_Untreated_2_3	Ctrl_Untreated_2	1.0	Dipeptides	True	1.265e+05	-0.9765	-0.9765	0.2454

Metabolite ID	Name	Super Pathway	Dataset	Sample ID	Group ID	Detection Fraction	Pathway	Detected	Raw Intensity	Log2 Norm Intensity	Norm Imputed	Log2 Ctrl Norm
272	Ser-Leu	Amino acid	2	Ctrl_Untreated_2_3	Ctrl_Untreated_2	1.0	Dipeptides	True	4.67e+05	0.9079	0.9079	0.1691
246	Asp-Leu	Amino acid	2	Ctrl_Untreated_2_3	Ctrl_Untreated_2	1.0	Dipeptides	True	2.593e+05	0.05905	0.05905	0.1331
76	Gln	Amino acid	2	Ctrl_Untreated_2_4	Ctrl_Untreated_2	1.0	Proteinogenic amino acids	True	2.02e+07	6.794	6.794	-0.158
89	Trp	Amino acid	2	Ctrl_Untreated_2_4	Ctrl_Untreated_2	1.0	Proteinogenic amino acids	True	1.23e+07	6.077	6.077	0.1109
723	beta-Ala	Cofactor	2	Ctrl_Untreated_2_4	Ctrl_Untreated_2	1.0	Coenzyme A biosynthesis	True	3.502e+05	0.9435	0.9435	0.6172
75	Glu	Amino acid	2	Ctrl_Untreated_2_4	Ctrl_Untreated_2	1.0	Proteinogenic amino acids	True	1.204e+07	6.047	6.047	0.09878
80	His	Amino acid	2	Ctrl_Untreated_2_4	Ctrl_Untreated_2	1.0	Proteinogenic amino acids	True	2.018e+05	0.1487	0.1487	-0.06546
82	Leu	Amino acid	2	Ctrl_Untreated_2_4	Ctrl_Untreated_2	1.0	Proteinogenic amino acids	True	5.254e+07	8.173	8.173	0.01268
87	Phe	Amino acid	2	Ctrl_Untreated_2_4	Ctrl_Untreated_2	1.0	Proteinogenic amino acids	True	4.323e+07	7.891	7.891	-4.25e-04
236	Spermidine	Amino acid	2	Ctrl_Untreated_2_4	Ctrl_Untreated_2	1.0	Polyamines	True	1.165e+06	2.677	2.677	-0.1503
73	Asn	Amino acid	2	Ctrl_Untreated_2_4	Ctrl_Untreated_2	1.0	Proteinogenic amino acids	True	7.426e+05	2.028	2.028	-0.7209
243	Creatinine	Amino acid	2	Ctrl_Untreated_2_4	Ctrl_Untreated_2	1.0	Creatine degradation	True	8.616e+05	2.242	2.242	0.672
376	Cytidine	Nucleotide	2	Ctrl_Untreated_2_4	Ctrl_Untreated_2	1.0	Pyrimidine nucleosides	True	6.989e+04	-1.381	-1.381	-0.7967
41	Lactate	Carbon	2	Ctrl_Untreated_2_4	Ctrl_Untreated_2	1.0	Respiratory carbon sources	True	4.735e+07	8.023	8.023	0.2006
93	3-P-Ser	Amino acid	2	Ctrl_Untreated_2_4	Ctrl_Untreated_2	1.0	Amino acids biosynthesis intermediates	True	2.986e+04	-2.608	-2.608	0.6507
343	Adenine	Nucleotide	2	Ctrl_Untreated_2_4	Ctrl_Untreated_2	1.0	Purine bases	True	5.45e+04	-1.74	-1.74	-0.2767
336	Adenosine	Nucleotide	2	Ctrl_Untreated_2_4	Ctrl_Untreated_2	1.0	Purine nucleosides	True	2.236e+06	3.618	3.618	0.4808
29	Raffinose	Carbon	2	Ctrl_Untreated_2_4	Ctrl_Untreated_2	1.0	Sugars and sugar alcohols	True	3.385e+04	-2.427	-2.427	-0.2475
717	Nicotinamide	Cofactor	2	Ctrl_Untreated_2_4	Ctrl_Untreated_2	1.0	NAD biosynthesis	True	1.162e+06	2.674	2.674	0.2772
51	PEP	Carbon	2	Ctrl_Untreated_2_4	Ctrl_Untreated_2	1.0	Glycolysis, GNG	True	5.294e+04	-1.782	-1.782	-0.7101
52	Pyruvate	Carbon	2	Ctrl_Untreated_2_4	Ctrl_Untreated_2	0.75	Glycolysis, GNG	True	7.09e+04	-1.361	-1.361	1.013
237	Spermine	Amino acid	2	Ctrl_Untreated_2_4	Ctrl_Untreated_2	1.0	Polyamines	True	2.087e+06	3.519	3.519	0.1302
385	Uracil	Nucleotide	2	Ctrl_Untreated_2_4	Ctrl_Untreated_2	1.0	Pyrimidine bases	True	3.577e+04	-2.348	-2.348	-0.6207
377	Uridine	Nucleotide	2	Ctrl_Untreated_2_4	Ctrl_Untreated_2	1.0	Pyrimidine nucleosides	True	1.355e+06	2.895	2.895	0.01308

Metabolite ID	Name	Super Pathway	Dataset	Sample ID	Group ID	Detection Fraction	Pathway	Detected	Raw Intensity	Log2 Norm Intensity	Norm Imputed	Log2 Ctrl Norm
112	trans-Urocanate	Amino acid	2	Ctrl_Untreated_2_4	Ctrl_Untreated_2	0.5	Amino acids degradation intermediates	False			-3.252	-0.1347
737	Pyridoxine (Vitamin B6)	Cofactor	2	Ctrl_Untreated_2_4	Ctrl_Untreated_2	1.0	PLP biosynthesis and salvage	True	1.815e+06	3.318	3.318	0.01762
348	Allantoin	Nucleotide	2	Ctrl_Untreated_2_4	Ctrl_Untreated_2	1.0	Purine degradation	True	3.246e+04	-2.488	-2.488	0.3414
335	Inosine	Nucleotide	2	Ctrl_Untreated_2_4	Ctrl_Untreated_2	1.0	Purine nucleosides	True	1.356e+06	2.897	2.897	0.03539
81	Ile	Amino acid	2	Ctrl_Untreated_2_4	Ctrl_Untreated_2	1.0	Proteinogenic amino acids	True	4.702e+07	8.012	8.012	0.157
72	Ala	Amino acid	2	Ctrl_Untreated_2_4	Ctrl_Untreated_2	1.0	Proteinogenic amino acids	True	2.465e+07	7.081	7.081	-0.6533
79	Thr	Amino acid	2	Ctrl_Untreated_2_4	Ctrl_Untreated_2	1.0	Proteinogenic amino acids	True	7.706e+06	5.403	5.403	-0.5591
88	Tyr	Amino acid	2	Ctrl_Untreated_2_4	Ctrl_Untreated_2	1.0	Proteinogenic amino acids	True	2.063e+07	6.824	6.824	-0.01872
84	Lys	Amino acid	2	Ctrl_Untreated_2_4	Ctrl_Untreated_2	1.0	Proteinogenic amino acids	True	2.447e+06	3.749	3.749	0.02943
86	Met	Amino acid	2	Ctrl_Untreated_2_4	Ctrl_Untreated_2	1.0	Proteinogenic amino acids	True	8.123e+06	5.48	5.48	-0.3076
61	Malate	Carbon	2	Ctrl_Untreated_2_4	Ctrl_Untreated_2	1.0	TCA cycle	True	2.395e+05	0.3952	0.3952	-0.5015
235	Putrescine	Amino acid	2	Ctrl_Untreated_2_4	Ctrl_Untreated_2	1.0	Polyamines	True	5.137e+04	-1.825	-1.825	-0.258
49	3-P-Glycerate	Carbon	2	Ctrl_Untreated_2_4	Ctrl_Untreated_2	1.0	Glycolysis, GNG	True	8.306e+05	2.19	2.19	-0.273
139	GABA	Amino acid	2	Ctrl_Untreated_2_4	Ctrl_Untreated_2	0.75	Amino acid derivatives	True	8988	-4.34	-4.34	-0.8698
189	Kynurenate	Amino acid	2	Ctrl_Untreated_2_4	Ctrl_Untreated_2	0.0	Amino acid derivatives	False			-5.188	0
234	5-Me-Thioadenosine	Amino acid	2	Ctrl_Untreated_2_4	Ctrl_Untreated_2	1.0	SAM metabolism	True	2.18e+05	0.26	0.26	0.356
59	Succinate	Carbon	2	Ctrl_Untreated_2_4	Ctrl_Untreated_2	1.0	TCA cycle	True	4.02e+04	-2.179	-2.179	-0.1393
133	Ornithine	Amino acid	2	Ctrl_Untreated_2_4	Ctrl_Untreated_2	1.0	Amino acids degradation intermediates	True	8.303e+05	2.189	2.189	-0.3321
313	5-Oxoproline	Amino acid	2	Ctrl_Untreated_2_4	Ctrl_Untreated_2	1.0	Glutathione derivatives	True	7.235e+05	1.99	1.99	-0.0132
724	Pantothenate	Cofactor	2	Ctrl_Untreated_2_4	Ctrl_Untreated_2	1.0	Coenzyme A biosynthesis	True	1.97e+06	3.436	3.436	0.3221
30	Sucrose	Carbon	2	Ctrl_Untreated_2_4	Ctrl_Untreated_2	1.0	Sugars and sugar alcohols	True	1.076e+06	2.563	2.563	0.1861
122	3-OH-Isobutyrate	Amino acid	2	Ctrl_Untreated_2_4	Ctrl_Untreated_2	0.25	Amino acids degradation intermediates	False			-4.628	0

Metabolite ID	Name	Super Pathway	Dataset	Sample ID	Group ID	Detection Fraction	Pathway	Detected	Raw Intensity	Log2 Norm Intensity	Norm Imputed	Log2 Ctrl Norm
241	4-Acetamidobutanoate	Amino acid	2	Ctrl_Untreated_2_4	Ctrl_Untreated_2	1.0	Polyamine derivatives	True	6.527e+04	-1.48	-1.48	0.02728
55	Citrate	Carbon	2	Ctrl_Untreated_2_4	Ctrl_Untreated_2	1.0	TCA cycle	True	1.212e+06	2.735	2.735	-0.299
338	Guanosine	Nucleotide	2	Ctrl_Untreated_2_4	Ctrl_Untreated_2	1.0	Purine nucleosides	True	6.744e+05	1.889	1.889	-0.4712
170	2-Amino-Butyrate	Amino acid	2	Ctrl_Untreated_2_4	Ctrl_Untreated_2	1.0	Amino acid derivatives	True	3.1e+05	0.7676	0.7676	-0.4562
209	N-Ac-Ala	Amino acid	2	Ctrl_Untreated_2_4	Ctrl_Untreated_2	1.0	N-acetylated amino acids	True	1.424e+04	-3.677	-3.677	-0.4094
221	N-Ac-Met	Amino acid	2	Ctrl_Untreated_2_4	Ctrl_Untreated_2	1.0	N-acetylated amino acids	True	6.185e+04	-1.558	-1.558	-0.4075
22	N-Ac-Neuraminate	Carbon	2	Ctrl_Untreated_2_4	Ctrl_Untreated_2	1.0	Aminosugar derivatives	True	1.464e+05	-0.3141	-0.3141	-0.1001
346	Urate	Nucleotide	2	Ctrl_Untreated_2_4	Ctrl_Untreated_2	1.0	Purine degradation	True	1.045e+04	-4.123	-4.123	-0.4311
90	Arg	Amino acid	2	Ctrl_Untreated_2_4	Ctrl_Untreated_2	1.0	Proteinogenic amino acids	True	4.685e+06	4.686	4.686	-0.0965
60	Fumarate	Carbon	2	Ctrl_Untreated_2_4	Ctrl_Untreated_2	1.0	TCA cycle	True	1.155e+05	-0.6563	-0.6563	-0.4996
78	Ser	Amino acid	2	Ctrl_Untreated_2_4	Ctrl_Untreated_2	1.0	Proteinogenic amino acids	True	1.366e+07	6.23	6.23	-0.5968
83	Val	Amino acid	2	Ctrl_Untreated_2_4	Ctrl_Untreated_2	1.0	Proteinogenic amino acids	True	3.12e+07	7.421	7.421	0.1872
734	Pyridoxal	Cofactor	2	Ctrl_Untreated_2_4	Ctrl_Untreated_2	0.75	PLP biosynthesis and salvage	True	7.077e+04	-1.363	-1.363	-0.3686
136	Urea	Amino acid	2	Ctrl_Untreated_2_4	Ctrl_Untreated_2	1.0	Amino acids degradation intermediates	True	4.508e+05	1.308	1.308	-0.337
742	Folate	Cofactor	2	Ctrl_Untreated_2_4	Ctrl_Untreated_2	1.0	Folate metabolism	True	1.534e+05	-0.2473	-0.2473	0.2696
729	Riboflavin (Vitamin B2)	Cofactor	2	Ctrl_Untreated_2_4	Ctrl_Untreated_2	1.0	Flavine biosynthesis	True	7.456e+04	-1.288	-1.288	0.2809
91	Pro	Amino acid	2	Ctrl_Untreated_2_4	Ctrl_Untreated_2	1.0	Proteinogenic amino acids	True	1.563e+07	6.423	6.423	-0.2976
308	Glutathione, Reduced	Amino acid	2	Ctrl_Untreated_2_4	Ctrl_Untreated_2	1.0	Glutathione	True	1.424e+07	6.29	6.29	-0.1656
706	FAD	Cofactor	2	Ctrl_Untreated_2_4	Ctrl_Untreated_2	0.0	Cofactors	False			-5.096	0
299	gamma-Glu-Tyr	Amino acid	2	Ctrl_Untreated_2_4	Ctrl_Untreated_2	1.0	Gamma-glutamyl dipeptides	True	5.684e+04	-1.68	-1.68	-0.5123
705	Coenzyme A	Cofactor	2	Ctrl_Untreated_2_4	Ctrl_Untreated_2	1.0	Cofactors	True	1.122e+04	-4.02	-4.02	-0.3134
342	Hypoxanthine	Nucleotide	2	Ctrl_Untreated_2_4	Ctrl_Untreated_2	1.0	Purine bases	True	1.196e+05	-0.6057	-0.6057	-0.6076
344	Xanthine	Nucleotide	2	Ctrl_Untreated_2_4	Ctrl_Untreated_2	1.0	Purine bases	True	5.891e+04	-1.628	-1.628	0.6112
703	NAD+	Cofactor	2	Ctrl_Untreated_2_4	Ctrl_Untreated_2	1.0	Cofactors	True	2.101e+06	3.528	3.528	-0.03013
731	Thiamin (Vitamin B1)	Cofactor	2	Ctrl_Untreated_2_4	Ctrl_Untreated_2	1.0	TPP biosynthesis	True	1.772e+05	-0.03902	-0.03902	0.1671

Metabolite ID	Name	Super Pathway	Dataset	Sample ID	Group ID	Detection Fraction	Pathway	Detected	Raw Intensity	Log2 Norm Intensity	Norm Imputed	Log2 Ctrl Norm
102	2-Aminoadipate	Amino acid	2	Ctrl_Untreated_2_4	Ctrl_Untreated_2	1.0	Amino acids biosynthesis intermediates	True	3.331e+05	0.8714	0.8714	0.1149
77	Gly	Amino acid	2	Ctrl_Untreated_2_4	Ctrl_Untreated_2	1.0	Proteinogenic amino acids	True	2.424e+07	7.057	7.057	-0.1713
45	Fructose-6-P	Carbon	2	Ctrl_Untreated_2_4	Ctrl_Untreated_2	0.75	Glycolysis, GNG	False			-2.517	-1.082
36	Ribose	Carbon	2	Ctrl_Untreated_2_4	Ctrl_Untreated_2	1.0	Sugars and sugar alcohols	True	5.854e+04	-1.637	-1.637	-0.6396
4	GlcNAc 6-P	Carbon	2	Ctrl_Untreated_2_4	Ctrl_Untreated_2	1.0	Aminosugar biosynthesis	True	2.142e+05	0.2345	0.2345	-0.1252
188	Kynurenine	Amino acid	2	Ctrl_Untreated_2_4	Ctrl_Untreated_2	1.0	Amino acid derivatives	True	2.345e+05	0.3653	0.3653	0.3793
63	6-P-Gluconate	Carbon	2	Ctrl_Untreated_2_4	Ctrl_Untreated_2	1.0	Pentose phosphate pathway (PPP)	True	7.646e+04	-1.252	-1.252	-0.2066
710	Carnitine	Cofactor	2	Ctrl_Untreated_2_4	Ctrl_Untreated_2	1.0	Cofactors	True	1.122e+06	2.624	2.624	0.09205
725	P-Pantetheine	Cofactor	2	Ctrl_Untreated_2_4	Ctrl_Untreated_2	1.0	Coenzyme A biosynthesis	True	4729	-5.267	-5.267	-0.4543
110	N-alpha-Ac-Ornithine	Amino acid	2	Ctrl_Untreated_2_4	Ctrl_Untreated_2	1.0	Amino acids biosynthesis intermediates	True	2.792e+05	0.6168	0.6168	-0.3572
116	3-Me-2-Oxo-Valerate	Amino acid	2	Ctrl_Untreated_2_4	Ctrl_Untreated_2	1.0	Amino acids degradation intermediates	True	1.712e+04	-3.411	-3.411	0.1639
155	4-Guanidinobutanoate	Amino acid	2	Ctrl_Untreated_2_4	Ctrl_Untreated_2	1.0	Amino acid derivatives	True	2.338e+05	0.3605	0.3605	1.08
310	S-Lactoyl-Glutathione	Amino acid	2	Ctrl_Untreated_2_4	Ctrl_Untreated_2	1.0	Glutathione derivatives	True	2.67e+04	-2.77	-2.77	0.9022
34	Ribitol	Carbon	2	Ctrl_Untreated_2_4	Ctrl_Untreated_2	0.75	Sugars and sugar alcohols	True	5.314e+04	-1.777	-1.777	-0.1979
707	FMN	Cofactor	2	Ctrl_Untreated_2_4	Ctrl_Untreated_2	1.0	Cofactors	True	1.478e+04	-3.622	-3.622	-0.2016
17	Maltose	Carbon	2	Ctrl_Untreated_2_4	Ctrl_Untreated_2	1.0	Glycogen degradation	True	2.01e+05	0.1425	0.1425	0.1819
18	Maltotriose	Carbon	2	Ctrl_Untreated_2_4	Ctrl_Untreated_2	1.0	Glycogen degradation	True	4.384e+05	1.268	1.268	-0.1612
19	Maltotetraose	Carbon	2	Ctrl_Untreated_2_4	Ctrl_Untreated_2	1.0	Glycogen degradation	True	1.7e+05	-0.09915	-0.09915	0.09128
232	SAH	Amino acid	2	Ctrl_Untreated_2_4	Ctrl_Untreated_2	1.0	SAM metabolism	True	1.69e+04	-3.429	-3.429	-0.2031
74	Asp	Amino acid	2	Ctrl_Untreated_2_4	Ctrl_Untreated_2	1.0	Proteinogenic amino acids	True	7.78e+06	5.417	5.417	-0.2423
129	5-Aminovalerate	Amino acid	2	Ctrl_Untreated_2_4	Ctrl_Untreated_2	1.0	Amino acids degradation intermediates	True	6.146e+04	-1.567	-1.567	-0.7538

Metabolite ID	Name	Super Pathway	Dataset	Sample ID	Group ID	Detection Fraction	Pathway	Detected	Raw Intensity	Log2 Norm Intensity	Norm Imputed	Log2 Ctrl Norm
254	Gly-Val	Amino acid	2	Ctrl_Untreated_2_4	Ctrl_Untreated_2	1.0	Dipeptides	True	1.631e+05	-0.159	-0.159	-0.08526
291	gamma-Glu-Leu	Amino acid	2	Ctrl_Untreated_2_4	Ctrl_Untreated_2	0.75	Gamma-glutamyl dipeptides	True	4.434e+04	-2.038	-2.038	-0.9559
173	Met Sulfoxide	Amino acid	2	Ctrl_Untreated_2_4	Ctrl_Untreated_2	1.0	Amino acid derivatives	True	1.849e+05	0.02203	0.02203	0.1608
43	Glucose	Carbon	2	Ctrl_Untreated_2_4	Ctrl_Untreated_2	1.0	Glycolysis, GNG	True	1.981e+07	6.766	6.766	-0.05383
249	Gly-Gly	Amino acid	2	Ctrl_Untreated_2_4	Ctrl_Untreated_2	1.0	Dipeptides	True	1.655e+05	-0.1373	-0.1373	-0.3545
169	2-OH-Butyrate	Amino acid	2	Ctrl_Untreated_2_4	Ctrl_Untreated_2	0.5	Amino acid derivatives	False			-1.729	-0.2955
98	3-Methyl-2-Oxobutyrate	Amino acid	2	Ctrl_Untreated_2_4	Ctrl_Untreated_2	0.0	Amino acids biosynthesis intermediates	False			-3.759	0
100	4-Me-2-Oxo-Pentanoate	Amino acid	2	Ctrl_Untreated_2_4	Ctrl_Untreated_2	1.0	Amino acids biosynthesis intermediates	True	1.465e+04	-3.635	-3.635	-0.3337
253	Gly-Pro	Amino acid	2	Ctrl_Untreated_2_4	Ctrl_Untreated_2	1.0	Dipeptides	True	1.423e+05	-0.3558	-0.3558	-0.03471
247	Asp-Phe	Amino acid	2	Ctrl_Untreated_2_4	Ctrl_Untreated_2	1.0	Dipeptides	True	1.181e+05	-0.6247	-0.6247	0.3166
212	N-Ac-Asp	Amino acid	2	Ctrl_Untreated_2_4	Ctrl_Untreated_2	0.5	N-acetylated amino acids	False			-4.689	-0.1861
720	1-Me-Nicotinamide	Cofactor	2	Ctrl_Untreated_2_4	Ctrl_Untreated_2	1.0	Derivatives of NA, nicotinamide and NAD	True	2.933e+06	4.01	4.01	0.3061
70	Creatine	Carbon	2	Ctrl_Untreated_2_4	Ctrl_Untreated_2	1.0	Creatine energy storage	True	1.053e+07	5.854	5.854	0.07382
309	Glutathione, Oxidized	Amino acid	2	Ctrl_Untreated_2_4	Ctrl_Untreated_2	1.0	Glutathione	True	2.738e+06	3.91	3.91	-0.03798
44	Glucose 6-P	Carbon	2	Ctrl_Untreated_2_4	Ctrl_Untreated_2	1.0	Glycolysis, GNG	True	1.675e+05	-0.1203	-0.1203	-0.935
24	Fructose	Carbon	2	Ctrl_Untreated_2_4	Ctrl_Untreated_2	1.0	Sugars and sugar alcohols	True	1.723e+06	3.243	3.243	-0.1181
85	Cys	Amino acid	2	Ctrl_Untreated_2_4	Ctrl_Untreated_2	1.0	Proteinogenic amino acids	True	7.112e+04	-1.356	-1.356	-0.7644
704	NADH	Cofactor	2	Ctrl_Untreated_2_4	Ctrl_Untreated_2	1.0	Cofactors	True	7.063e+04	-1.366	-1.366	0.5631
275	Thr-Phe	Amino acid	2	Ctrl_Untreated_2_4	Ctrl_Untreated_2	1.0	Dipeptides	True	9.136e+04	-0.9948	-0.9948	-0.06122
738	Pyridoxate	Cofactor	2	Ctrl_Untreated_2_4	Ctrl_Untreated_2	0.75	PLP biosynthesis and salvage	True	9118	-4.32	-4.32	0.3455
177	3-(4-OH-Phenyl)Lactate	Amino acid	2	Ctrl_Untreated_2_4	Ctrl_Untreated_2	1.0	Amino acid derivatives	True	9275	-4.295	-4.295	-0.342
206	Trans-4-OH-Pro	Amino acid	2	Ctrl_Untreated_2_4	Ctrl_Untreated_2	1.0	Amino acid derivatives	True	1.39e+05	-0.3899	-0.3899	-0.1943
329	AMP	Nucleotide	2	Ctrl_Untreated_2_4	Ctrl_Untreated_2	1.0	Purine nucleotides	True	6.963e+05	1.935	1.935	1.515

Metabolite ID	Name	Super Pathway	Dataset	Sample ID	Group ID	Detection Fraction	Pathway	Detected	Raw Intensity	Log2 Norm Intensity	Norm Imputed	Log2 Ctrl Norm
345	Guanine	Nucleotide	2	Ctrl_Untreated_2_4	Ctrl_Untreated_2	1.0	Purine bases	True	2.849e+05	0.646	0.646	-0.936
271	pyroGlu-Val	Amino acid	2	Ctrl_Untreated_2_4	Ctrl_Untreated_2	1.0	Dipeptides	True	1.69e+04	-3.429	-3.429	0.2121
279	Val-Glu	Amino acid	2	Ctrl_Untreated_2_4	Ctrl_Untreated_2	1.0	Dipeptides	True	9.814e+04	-0.8916	-0.8916	-0.06162
183	Phenol Sulfate	Amino acid	2	Ctrl_Untreated_2_4	Ctrl_Untreated_2	0.5	Amino acid derivatives	True	2.818e+04	-2.692	-2.692	1.035
740	3-Dehydrocarnitine	Cofactor	2	Ctrl_Untreated_2_4	Ctrl_Untreated_2	1.0	Carnitine biosynthesis	True	1.67e+05	-0.1245	-0.1245	0.2091
145	Pyro-Gln	Amino acid	2	Ctrl_Untreated_2_4	Ctrl_Untreated_2	1.0	Amino acid derivatives	True	4.074e+05	1.162	1.162	0.8161
197	C-Glycosyl-Trp	Amino acid	2	Ctrl_Untreated_2_4	Ctrl_Untreated_2	1.0	Amino acid derivatives	True	2.44e+05	0.4226	0.4226	0.5662
718	Nicotinamide Riboside	Cofactor	2	Ctrl_Untreated_2_4	Ctrl_Untreated_2	0.5	NAD biosynthesis	False			-2.306	-0.5023
295	gamma-Glu-Phe	Amino acid	2	Ctrl_Untreated_2_4	Ctrl_Untreated_2	1.0	Gamma-glutamyl dipeptides	True	1.618e+05	-0.1703	-0.1703	-0.06836
399	Pseudouridine	Nucleotide	2	Ctrl_Untreated_2_4	Ctrl_Untreated_2	1.0	Pyrimidine derivatives in RNAs	True	1.115e+04	-4.03	-4.03	0.211
375	UTP	Nucleotide	2	Ctrl_Untreated_2_4	Ctrl_Untreated_2	0.75	Pyrimidine nucleotides	True	5695	-4.999	-4.999	0.4442
20	Erythronate	Carbon	2	Ctrl_Untreated_2_4	Ctrl_Untreated_2	1.0	Aminosugar derivatives	True	4.856e+04	-1.907	-1.907	-0.465
151	Phenylacetylglycine	Amino acid	2	Ctrl_Untreated_2_4	Ctrl_Untreated_2	1.0	Amino acid derivatives	True	3.039e+04	-2.583	-2.583	1.839
252	Gly-Phe	Amino acid	2	Ctrl_Untreated_2_4	Ctrl_Untreated_2	1.0	Dipeptides	True	3.639e+05	0.999	0.999	0.8949
251	Gly-Leu	Amino acid	2	Ctrl_Untreated_2_4	Ctrl_Untreated_2	1.0	Dipeptides	True	2.479e+05	0.4452	0.4452	0.08257
290	gamma-Glu-Ile	Amino acid	2	Ctrl_Untreated_2_4	Ctrl_Untreated_2	0.75	Gamma-glutamyl dipeptides	True	6.045e+04	-1.591	-1.591	-0.05959
316	Ophthalmate	Amino acid	2	Ctrl_Untreated_2_4	Ctrl_Untreated_2	1.0	Oxidative stress markers	True	1.281e+05	-0.507	-0.507	-0.7612
208	Pro-OH-Pro	Amino acid	2	Ctrl_Untreated_2_4	Ctrl_Untreated_2	1.0	Amino acid derivatives	True	2.468e+05	0.4386	0.4386	-0.02104
352	3'-AMP	Nucleotide	2	Ctrl_Untreated_2_4	Ctrl_Untreated_2	1.0	Purine derivatives in signaling	True	9.441e+04	-0.9475	-0.9475	0.3374
314	Cys-Glutathione Disulfide	Amino acid	2	Ctrl_Untreated_2_4	Ctrl_Untreated_2	1.0	Oxidative stress markers	True	7.456e+04	-1.288	-1.288	0.07728
39	Threitol	Carbon	2	Ctrl_Untreated_2_4	Ctrl_Untreated_2	0.75	Sugars and sugar alcohols	True	1.379e+04	-3.723	-3.723	0.3019
31	Ribulose/Xylulose	Carbon	2	Ctrl_Untreated_2_4	Ctrl_Untreated_2	0.5	Sugars and sugar alcohols	False			-5.004	-0.09716
48	DHAP	Carbon	2	Ctrl_Untreated_2_4	Ctrl_Untreated_2	1.0	Glycolysis, GNG	True	9.038e+05	2.311	2.311	0.5534

Metabolite ID	Name	Super Pathway	Dataset	Sample ID	Group ID	Detection Fraction	Pathway	Detected	Raw Intensity	Log2 Norm Intensity	Norm Imputed	Log2 Ctrl Norm
182	P-Cresol Sulfate	Amino acid	2	Ctrl_Untreated_2_4	Ctrl_Untreated_2	1.0	Amino acid derivatives	True	1.591e+04	-3.517	-3.517	-0.1794
250	Gly-Ile	Amino acid	2	Ctrl_Untreated_2_4	Ctrl_Untreated_2	0.75	Dipeptides	True	1.403e+05	-0.3761	-0.3761	1.048
286	gamma-Glu-Glu	Amino acid	2	Ctrl_Untreated_2_4	Ctrl_Untreated_2	1.0	Gamma-glutamyl dipeptides	True	9.245e+04	-0.9777	-0.9777	-0.7471
264	Leu-Leu	Amino acid	2	Ctrl_Untreated_2_4	Ctrl_Untreated_2	1.0	Dipeptides	True	7.641e+04	-1.253	-1.253	-0.1794
203	DiMe-Arg	Amino acid	2	Ctrl_Untreated_2_4	Ctrl_Untreated_2	1.0	Amino acid derivatives	True	3.771e+05	1.051	1.051	-0.02375
47	Fructose 1,6-PP, Glucose 1,6-PP	Carbon	2	Ctrl_Untreated_2_4	Ctrl_Untreated_2	1.0	Glycolysis, GNG	True	2.219e+05	0.2855	0.2855	0.5756
224	N-Ac-Ser	Amino acid	2	Ctrl_Untreated_2_4	Ctrl_Untreated_2	1.0	N-acetylated amino acids	True	2.981e+05	0.7112	0.7112	-0.1595
244	Ala-Leu	Amino acid	2	Ctrl_Untreated_2_4	Ctrl_Untreated_2	1.0	Dipeptides	True	1.733e+05	-0.07149	-0.07149	-0.4112
304	Cyclo(Phe-Pro)	Amino acid	2	Ctrl_Untreated_2_4	Ctrl_Untreated_2	1.0	Cyclic dipeptides	True	1.793e+05	-0.02203	-0.02203	0.4497
302	Cyclo(Glu-Glu)	Amino acid	2	Ctrl_Untreated_2_4	Ctrl_Untreated_2	1.0	Cyclic dipeptides	True	8.118e+04	-1.165	-1.165	6.83e-03
303	Cyclo(Leu-Pro)	Amino acid	2	Ctrl_Untreated_2_4	Ctrl_Untreated_2	1.0	Cyclic dipeptides	True	2.87e+05	0.6564	0.6564	0.3835
390	2',3'-cUMP	Nucleotide	2	Ctrl_Untreated_2_4	Ctrl_Untreated_2	1.0	Pyrimidine derivatives in signaling	True	1.085e+05	-0.7463	-0.7463	0.3826
68	Ribulose 5-P / Xylulose 5-P	Carbon	2	Ctrl_Untreated_2_4	Ctrl_Untreated_2	1.0	Pentose phosphate pathway (PPP)	True	2.055e+05	0.1746	0.1746	-0.5946
388	2',3'-cCMP	Nucleotide	2	Ctrl_Untreated_2_4	Ctrl_Untreated_2	1.0	Pyrimidine derivatives in signaling	True	2.86e+05	0.6515	0.6515	0.3981
33	Arabitol/Xylitol	Carbon	2	Ctrl_Untreated_2_4	Ctrl_Untreated_2	0.25	Sugars and sugar alcohols	True	1.794e+04	-3.343	-3.343	0.1622
268	Phe-Phe	Amino acid	2	Ctrl_Untreated_2_4	Ctrl_Untreated_2	0.75	Dipeptides	True	3.664e+05	1.009	1.009	2.097
245	Ala-Phe	Amino acid	2	Ctrl_Untreated_2_4	Ctrl_Untreated_2	1.0	Dipeptides	True	4.333e+04	-2.071	-2.071	-0.6156
373	UMP	Nucleotide	2	Ctrl_Untreated_2_4	Ctrl_Untreated_2	0.75	Pyrimidine nucleotides	True	1.592e+05	-0.1939	-0.1939	1.847
282	Val-Leu	Amino acid	2	Ctrl_Untreated_2_4	Ctrl_Untreated_2	1.0	Dipeptides	True	1.137e+05	-0.6791	-0.6791	-0.3104
258	Ile-Gly	Amino acid	2	Ctrl_Untreated_2_4	Ctrl_Untreated_2	1.0	Dipeptides	True	2.066e+05	0.1826	0.1826	-0.232
259	Ile-Ser	Amino acid	2	Ctrl_Untreated_2_4	Ctrl_Untreated_2	1.0	Dipeptides	True	8.676e+04	-1.069	-1.069	-0.2064
269	Phe-Ser	Amino acid	2	Ctrl_Untreated_2_4	Ctrl_Untreated_2	1.0	Dipeptides	True	4.887e+04	-1.898	-1.898	-0.5826
277	Tyr-Ala	Amino acid	2	Ctrl_Untreated_2_4	Ctrl_Untreated_2	1.0	Dipeptides	True	3.691e+05	1.02	1.02	0.4494
257	Ile-Gln	Amino acid	2	Ctrl_Untreated_2_4	Ctrl_Untreated_2	1.0	Dipeptides	True	7.072e+04	-1.364	-1.364	-0.4455
261	Leu-Glu	Amino acid	2	Ctrl_Untreated_2_4	Ctrl_Untreated_2	1.0	Dipeptides	True	2.54e+05	0.4805	0.4805	-0.3097
263	Leu-Gly	Amino acid	2	Ctrl_Untreated_2_4	Ctrl_Untreated_2	1.0	Dipeptides	True	2.078e+05	0.1905	0.1905	-0.5164

Metabolite ID	Name	Super Pathway	Dataset	Sample ID	Group ID	Detection Fraction	Pathway	Detected	Raw Intensity	Log2 Norm Intensity	Norm Imputed	Log2 Ctrl Norm
256	Ile-Ala	Amino acid	2	Ctrl_Untreated_2_4	Ctrl_Untreated_2	1.0	Dipeptides	True	1.978e+05	0.1193	0.1193	0.161
274	Thr-Leu	Amino acid	2	Ctrl_Untreated_2_4	Ctrl_Untreated_2	1.0	Dipeptides	True	2.612e+05	0.5208	0.5208	-0.3304
273	Ser-Phe	Amino acid	2	Ctrl_Untreated_2_4	Ctrl_Untreated_2	1.0	Dipeptides	True	6.81e+04	-1.419	-1.419	-0.1969
272	Ser-Leu	Amino acid	2	Ctrl_Untreated_2_4	Ctrl_Untreated_2	1.0	Dipeptides	True	1.904e+05	0.06435	0.06435	-0.6745
246	Asp-Leu	Amino acid	2	Ctrl_Untreated_2_4	Ctrl_Untreated_2	1.0	Dipeptides	True	1.338e+05	-0.4446	-0.4446	-0.3706
76	Gln	Amino acid	1	AT_Untreated_1_1	AT_Untreated_1	1.0	Proteinogenic amino acids	True	2.807e+08	8.031	8.031	-0.6497
89	Trp	Amino acid	1	AT_Untreated_1_1	AT_Untreated_1	1.0	Proteinogenic amino acids	True	6.155e+07	5.842	5.842	0.166
723	beta-Ala	Cofactor	1	AT_Untreated_1_1	AT_Untreated_1	1.0	Coenzyme A biosynthesis	True	4.064e+06	1.921	1.921	-1.088
75	Glu	Amino acid	1	AT_Untreated_1_1	AT_Untreated_1	1.0	Proteinogenic amino acids	True	8.081e+08	9.556	9.556	-1.18
77	Gly	Amino acid	1	AT_Untreated_1_1	AT_Untreated_1	1.0	Proteinogenic amino acids	True	6.207e+07	5.854	5.854	0.2387
80	His	Amino acid	1	AT_Untreated_1_1	AT_Untreated_1	1.0	Proteinogenic amino acids	True	1.252e+07	3.545	3.545	0.1293
82	Leu	Amino acid	1	AT_Untreated_1_1	AT_Untreated_1	1.0	Proteinogenic amino acids	True	1.189e+09	10.11	10.11	0.8924
87	Phe	Amino acid	1	AT_Untreated_1_1	AT_Untreated_1	1.0	Proteinogenic amino acids	True	6.528e+08	9.249	9.249	0.4371
130	Glutarate	Amino acid	1	AT_Untreated_1_1	AT_Untreated_1	1.0	Amino acids degradation intermediates	True	2.298e+05	-2.223	-2.223	0.2032
196	5-OH-Indole-Ac	Amino acid	1	AT_Untreated_1_1	AT_Untreated_1	1.0	Amino acid derivatives	True	1.297e+05	-3.049	-3.049	0.9629
74	Asp	Amino acid	1	AT_Untreated_1_1	AT_Untreated_1	1.0	Proteinogenic amino acids	True	1.144e+08	6.736	6.736	-1.155
236	Spermidine	Amino acid	1	AT_Untreated_1_1	AT_Untreated_1	1.0	Polyamines	True	9.535e+05	-0.1706	-0.1706	-4.032
73	Asn	Amino acid	1	AT_Untreated_1_1	AT_Untreated_1	1.0	Proteinogenic amino acids	True	1.475e+08	7.103	7.103	0.4315
243	Creatinine	Amino acid	1	AT_Untreated_1_1	AT_Untreated_1	1.0	Creatine degradation	True	3.314e+07	4.948	4.948	-0.05385
376	Cytidine	Nucleotide	1	AT_Untreated_1_1	AT_Untreated_1	0.833333333333333	Pyrimidine nucleosides	False			-1.507	-2.235
41	Lactate	Carbon	1	AT_Untreated_1_1	AT_Untreated_1	1.0	Respiratory carbon sources	True	1.079e+08	6.651	6.651	0.1695
58	alpha-Ketoglutarate	Carbon	1	AT_Untreated_1_1	AT_Untreated_1	1.0	TCA cycle	True	7.752e+05	-0.4694	-0.4694	-0.4861
69	3-OH-Butyrate	Carbon	1	AT_Untreated_1_1	AT_Untreated_1	0.833333333333333	Ketone bodies	False			-3.497	-0.5127

Metabolite ID	Name	Super Pathway	Dataset	Sample ID	Group ID	Detection Fraction	Pathway	Detected	Raw Intensity	Log2 Norm Intensity	Norm Imputed	Log2 Ctrl Norm
343	Adenine	Nucleotide	1	AT_Untreated_1_1	AT_Untreated_1	1.0	Purine bases	True	1.103e+06	0.0393	0.0393	-1.418
336	Adenosine	Nucleotide	1	AT_Untreated_1_1	AT_Untreated_1	1.0	Purine nucleosides	True	3.988e+06	1.894	1.894	-1.529
722	ADP-Ribose	Cofactor	1	AT_Untreated_1_1	AT_Untreated_1	1.0	Derivatives of NA, nicotinamide and NAD	True	2.01e+04	-5.738	-5.738	-2.736
383	Cytosine	Nucleotide	1	AT_Untreated_1_1	AT_Untreated_1	0.833333333333333	Pyrimidine bases	False			-5.32	-0.4652
3	Glucosamine 6-P	Carbon	1	AT_Untreated_1_1	AT_Untreated_1	0.833333333333333	Aminosugar biosynthesis	True	1.917e+05	-2.485	-2.485	-0.8173
717	Nicotinamide	Cofactor	1	AT_Untreated_1_1	AT_Untreated_1	1.0	NAD biosynthesis	True	5.025e+07	5.549	5.549	1.034
51	PEP	Carbon	1	AT_Untreated_1_1	AT_Untreated_1	1.0	Glycolysis, GNG	True	2.795e+06	1.381	1.381	0.3295
237	Spermine	Amino acid	1	AT_Untreated_1_1	AT_Untreated_1	1.0	Polyamines	True	1.353e+05	-2.987	-2.987	-1.497
385	Uracil	Nucleotide	1	AT_Untreated_1_1	AT_Untreated_1	1.0	Pyrimidine bases	True	1.364e+07	3.667	3.667	3.403
377	Uridine	Nucleotide	1	AT_Untreated_1_1	AT_Untreated_1	1.0	Pyrimidine nucleosides	True	1.851e+07	4.108	4.108	0.05919
348	Allantoin	Nucleotide	1	AT_Untreated_1_1	AT_Untreated_1	1.0	Purine degradation	True	9.368e+05	-0.1961	-0.1961	-0.02049
335	Inosine	Nucleotide	1	AT_Untreated_1_1	AT_Untreated_1	1.0	Purine nucleosides	True	2.95e+07	4.781	4.781	-0.7184
81	Ile	Amino acid	1	AT_Untreated_1_1	AT_Untreated_1	1.0	Proteinogenic amino acids	True	6.156e+08	9.164	9.164	0.4487
72	Ala	Amino acid	1	AT_Untreated_1_1	AT_Untreated_1	1.0	Proteinogenic amino acids	True	3.323e+08	8.274	8.274	-0.2271
79	Thr	Amino acid	1	AT_Untreated_1_1	AT_Untreated_1	1.0	Proteinogenic amino acids	True	3.655e+08	8.412	8.412	0.4022
88	Tyr	Amino acid	1	AT_Untreated_1_1	AT_Untreated_1	1.0	Proteinogenic amino acids	True	3.605e+08	8.392	8.392	0.8681
84	Lys	Amino acid	1	AT_Untreated_1_1	AT_Untreated_1	1.0	Proteinogenic amino acids	True	2.986e+08	8.12	8.12	0.1301
86	Met	Amino acid	1	AT_Untreated_1_1	AT_Untreated_1	1.0	Proteinogenic amino acids	True	4.748e+08	8.789	8.789	0.5615
61	Malate	Carbon	1	AT_Untreated_1_1	AT_Untreated_1	1.0	TCA cycle	True	9.494e+07	6.467	6.467	-0.7048
235	Putrescine	Amino acid	1	AT_Untreated_1_1	AT_Untreated_1	0.833333333333333	Polyamines	False			-2.846	-2.355
324	2'-dU	Nucleotide	1	AT_Untreated_1_1	AT_Untreated_1	0.666666666666667	Deoxy-nucleosides	True	1.034e+05	-3.376	-3.376	1.004
49	3-P-Glycerate	Carbon	1	AT_Untreated_1_1	AT_Untreated_1	1.0	Glycolysis, GNG	True	9.232e+06	3.105	3.105	-0.1607
189	Kynurenate	Amino acid	1	AT_Untreated_1_1	AT_Untreated_1	1.0	Amino acid derivatives	True	3.282e+04	-5.031	-5.031	0.7668
234	5-Me-Thioadenosine	Amino acid	1	AT_Untreated_1_1	AT_Untreated_1	1.0	SAM metabolism	True	3.812e+05	-1.493	-1.493	-3.379

Metabolite ID	Name	Super Pathway	Dataset	Sample ID	Group ID	Detection Fraction	Pathway	Detected	Raw Intensity	Log2 Norm Intensity	Norm Imputed	Log2 Ctrl Norm
59	Succinate	Carbon	1	AT_Untreated_1_1	AT_Untreated_1	1.0	TCA cycle	True	1.336e+06	0.3162	0.3162	0.01512
36	Ribose	Carbon	1	AT_Untreated_1_1	AT_Untreated_1	1.0	Sugars and sugar alcohols	True	5.914e+05	-0.8597	-0.8597	0.3738
133	Ornithine	Amino acid	1	AT_Untreated_1_1	AT_Untreated_1	1.0	Amino acids degradation intermediates	True	1.094e+07	3.35	3.35	-1.13
313	5-Oxoproline	Amino acid	1	AT_Untreated_1_1	AT_Untreated_1	1.0	Glutathione derivatives	True	8.168e+06	2.928	2.928	0.8405
165	N-6-Tri-Me-Lys	Amino acid	1	AT_Untreated_1_1	AT_Untreated_1	1.0	Amino acid derivatives	True	1.064e+07	3.31	3.31	-0.4535
380	Orotate	Nucleotide	1	AT_Untreated_1_1	AT_Untreated_1	0.666666666666667	Pyrimidine (UMP) biosynthesis	True	3.425e+04	-4.969	-4.969	-1.339
724	Pantothenate	Cofactor	1	AT_Untreated_1_1	AT_Untreated_1	1.0	Coenzyme A biosynthesis	True	4.913e+07	5.517	5.517	-0.7936
150	N-Me-Gly	Amino acid	1	AT_Untreated_1_1	AT_Untreated_1	1.0	Amino acid derivatives	True	1.303e+06	0.2796	0.2796	0.58
122	3-OH-Isobutyrate	Amino acid	1	AT_Untreated_1_1	AT_Untreated_1	0.833333333333333	Amino acids degradation intermediates	False			-3.901	-0.7452
241	4-Acetamidobutanoate	Amino acid	1	AT_Untreated_1_1	AT_Untreated_1	1.0	Polyamine derivatives	True	1.739e+06	0.6963	0.6963	0.2554
711	alpha-Tocopherol	Cofactor	1	AT_Untreated_1_1	AT_Untreated_1	1.0	Cofactors	True	3.823e+06	1.833	1.833	1.052
55	Citrate	Carbon	1	AT_Untreated_1_1	AT_Untreated_1	1.0	TCA cycle	True	1.534e+07	3.837	3.837	1.888
387	3-Aminoisobutyrate	Nucleotide	1	AT_Untreated_1_1	AT_Untreated_1	0.833333333333333	Pyrimidine degradation	True	1.959e+05	-2.454	-2.454	-0.44
338	Guanosine	Nucleotide	1	AT_Untreated_1_1	AT_Untreated_1	1.0	Purine nucleosides	True	7.436e+07	6.114	6.114	1.009
209	N-Ac-Ala	Amino acid	1	AT_Untreated_1_1	AT_Untreated_1	1.0	N-acetylated amino acids	True	4.285e+05	-1.324	-1.324	-0.1233
221	N-Ac-Met	Amino acid	1	AT_Untreated_1_1	AT_Untreated_1	1.0	N-acetylated amino acids	True	2.954e+06	1.461	1.461	-0.2836
228	N-Ac-Val	Amino acid	1	AT_Untreated_1_1	AT_Untreated_1	0.333333333333333	N-acetylated amino acids	False			-5.82	-0.449
346	Urate	Nucleotide	1	AT_Untreated_1_1	AT_Untreated_1	1.0	Purine degradation	True	6.312e+05	-0.7657	-0.7657	0.2971
90	Arg	Amino acid	1	AT_Untreated_1_1	AT_Untreated_1	1.0	Proteinogenic amino acids	True	1.738e+09	10.66	10.66	0.9559
60	Fumarate	Carbon	1	AT_Untreated_1_1	AT_Untreated_1	1.0	TCA cycle	True	2.237e+06	1.059	1.059	0.04421
78	Ser	Amino acid	1	AT_Untreated_1_1	AT_Untreated_1	1.0	Proteinogenic amino acids	True	3.113e+08	8.18	8.18	0.9228
83	Val	Amino acid	1	AT_Untreated_1_1	AT_Untreated_1	1.0	Proteinogenic amino acids	True	4.419e+08	8.686	8.686	0.8133

Metabolite ID	Name	Super Pathway	Dataset	Sample ID	Group ID	Detection Fraction	Pathway	Detected	Raw Intensity	Log2 Norm Intensity	Norm Imputed	Log2 Ctrl Norm
734	Pyridoxal	Cofactor	1	AT_Untreated_1_1	AT_Untreated_1	1.0	PLP biosynthesis and salvage	True	3.497e+06	1.704	1.704	-0.07904
136	Urea	Amino acid	1	AT_Untreated_1_1	AT_Untreated_1	1.0	Amino acids degradation intermediates	True	1.044e+06	-0.03943	-0.03943	-0.1907
67	Ribose 1-P	Carbon	1	AT_Untreated_1_1	AT_Untreated_1	1.0	Pentose phosphate pathway (PPP)	True	2.392e+06	1.156	1.156	0.5349
284	Carnosine	Amino acid	1	AT_Untreated_1_1	AT_Untreated_1	1.0	Dipeptides	True	2.452e+05	-2.13	-2.13	-0.02137
306	gamma-Glu-Cys	Amino acid	1	AT_Untreated_1_1	AT_Untreated_1	1.0	Glutathione biosynthesis	True	1.045e+05	-3.36	-3.36	-3.024
712	Retinol (Vit A)	Cofactor	1	AT_Untreated_1_1	AT_Untreated_1	0.833333333333333	Cofactors	True	2.031e+05	-2.402	-2.402	0.03149
85	Cys	Amino acid	1	AT_Untreated_1_1	AT_Untreated_1	1.0	Proteinogenic amino acids	True	2.047e+07	4.253	4.253	-0.2602
91	Pro	Amino acid	1	AT_Untreated_1_1	AT_Untreated_1	1.0	Proteinogenic amino acids	True	8.509e+08	9.631	9.631	0.223
308	Glutathione, Reduced	Amino acid	1	AT_Untreated_1_1	AT_Untreated_1	1.0	Glutathione	True	2.097e+07	4.289	4.289	-2.668
107	Citrulline	Amino acid	1	AT_Untreated_1_1	AT_Untreated_1	1.0	Amino acids biosynthesis intermediates	True	3.826e+07	5.156	5.156	2.331
328	IMP	Nucleotide	1	AT_Untreated_1_1	AT_Untreated_1	0.333333333333333	Purine nucleotides	False			-4.53	-0.2088
706	FAD	Cofactor	1	AT_Untreated_1_1	AT_Untreated_1	1.0	Cofactors	True	3.514e+05	-1.611	-1.611	0.5786
735	Pyridoxamine	Cofactor	1	AT_Untreated_1_1	AT_Untreated_1	1.0	PLP biosynthesis and salvage	True	1.083e+06	0.0133	0.0133	0.2063
199	Serotonin	Amino acid	1	AT_Untreated_1_1	AT_Untreated_1	1.0	Amino acid derivatives	True	1.991e+06	0.8914	0.8914	-0.3813
370	CMP	Nucleotide	1	AT_Untreated_1_1	AT_Untreated_1	1.0	Pyrimidine nucleotides	True	2.48e+06	1.209	1.209	-0.6303
287	gamma-Glu-Gln	Amino acid	1	AT_Untreated_1_1	AT_Untreated_1	1.0	Gamma-glutamyl dipeptides	True	9.209e+05	-0.2207	-0.2207	-1.062
14	UDP-Glucuronate	Carbon	1	AT_Untreated_1_1	AT_Untreated_1	0.666666666666667	Polysaccharide biosynthesis	False			-4.022	-2.073
229	N-Formyl-Met	Amino acid	1	AT_Untreated_1_1	AT_Untreated_1	0.666666666666667	N-formylated amino acids	True	2.345e+04	-5.516	-5.516	-1.694
350	3',5'-cAMP	Nucleotide	1	AT_Untreated_1_1	AT_Untreated_1	0.666666666666667	Purine derivatives in signaling	True	6.985e+04	-3.942	-3.942	-0.3224
371	CDP	Nucleotide	1	AT_Untreated_1_1	AT_Untreated_1	0.666666666666667	Pyrimidine nucleotides	False			-5.544	-2.224
372	CTP	Nucleotide	1	AT_Untreated_1_1	AT_Untreated_1	0.333333333333333	Pyrimidine nucleotides	False			-4.713	-1.059

Metabolite ID	Name	Super Pathway	Dataset	Sample ID	Group ID	Detection Fraction	Pathway	Detected	Raw Intensity	Log2 Norm Intensity	Norm Imputed	Log2 Ctrl Norm
333	GDP	Nucleotide	1	AT_Untreated_1_1	AT_Untreated_1	0.5	Purine nucleotides	False			-4.484	-1.983
332	GMP	Nucleotide	1	AT_Untreated_1_1	AT_Untreated_1	1.0	Purine nucleotides	True	6.36e+05	-0.7549	-0.7549	-1.454
373	UMP	Nucleotide	1	AT_Untreated_1_1	AT_Untreated_1	0.833333333333333	Pyrimidine nucleotides	False			-4.645	-3.59
389	3'-CMP	Nucleotide	1	AT_Untreated_1_1	AT_Untreated_1	1.0	Pyrimidine derivatives in signaling	True	6.452e+05	-0.7341	-0.7341	0.3172
330	ADP	Nucleotide	1	AT_Untreated_1_1	AT_Untreated_1	0.5	Purine nucleotides	False			-2.826	-2.53
342	Hypoxanthine	Nucleotide	1	AT_Untreated_1_1	AT_Untreated_1	1.0	Purine bases	True	2.226e+07	4.375	4.375	0.4354
736	Pyridoxamine-P	Cofactor	1	AT_Untreated_1_1	AT_Untreated_1	0.833333333333333	PLP biosynthesis and salvage	True	1.303e+05	-3.042	-3.042	-0.06398
148	Betaine	Amino acid	1	AT_Untreated_1_1	AT_Untreated_1	1.0	Amino acid derivatives	True	2.374e+07	4.467	4.467	-0.3384
344	Xanthine	Nucleotide	1	AT_Untreated_1_1	AT_Untreated_1	1.0	Purine bases	True	4.276e+06	1.994	1.994	1.087
386	3-Ureidopropionate	Nucleotide	1	AT_Untreated_1_1	AT_Untreated_1	1.0	Pyrimidine degradation	True	1.063e+06	-0.0133	-0.0133	0.9061
149	DiMe-Gly	Amino acid	1	AT_Untreated_1_1	AT_Untreated_1	1.0	Amino acid derivatives	True	6.334e+05	-0.7608	-0.7608	-0.2405
703	NAD+	Cofactor	1	AT_Untreated_1_1	AT_Untreated_1	1.0	Cofactors	True	2.54e+06	1.243	1.243	-1.494
709	Pyridoxal-P	Cofactor	1	AT_Untreated_1_1	AT_Untreated_1	0.833333333333333	Cofactors	False			-3.351	-1.043
731	Thiamin (Vitamin B1)	Cofactor	1	AT_Untreated_1_1	AT_Untreated_1	1.0	TPP biosynthesis	True	1.099e+06	0.03417	0.03417	-0.7135
374	UDP	Nucleotide	1	AT_Untreated_1_1	AT_Untreated_1	0.666666666666667	Pyrimidine nucleotides	False			-5.339	-3.785
102	2-Aminoadipate	Amino acid	1	AT_Untreated_1_1	AT_Untreated_1	0.833333333333333	Amino acids biosynthesis intermediates	True	1.027e+05	-3.385	-3.385	-1.276
45	Fructose-6-P	Carbon	1	AT_Untreated_1_1	AT_Untreated_1	1.0	Glycolysis, GNG	True	4.412e+05	-1.282	-1.282	-1.358
320	TMP	Nucleotide	1	AT_Untreated_1_1	AT_Untreated_1	0.5	Deoxy-nucleotides	False			-6.992	-2.2
341	XMP	Nucleotide	1	AT_Untreated_1_1	AT_Untreated_1	0.166666666666667	IMP conversion to AMP & GMP	False			-6.353	-1.778
120	beta-OH-Isovalerate	Amino acid	1	AT_Untreated_1_1	AT_Untreated_1	1.0	Amino acids degradation intermediates	True	8.489e+05	-0.3382	-0.3382	2.827
322	2'-dI	Nucleotide	1	AT_Untreated_1_1	AT_Untreated_1	0.5	Deoxy-nucleosides	True	5.587e+04	-4.264	-4.264	-0.1383
4	GlcNAc 6-P	Carbon	1	AT_Untreated_1_1	AT_Untreated_1	1.0	Aminosugar biosynthesis	True	3.909e+06	1.865	1.865	1.536
337	Xanthosine	Nucleotide	1	AT_Untreated_1_1	AT_Untreated_1	1.0	Purine nucleosides	True	6.264e+05	-0.7769	-0.7769	2.298
188	Kynurenine	Amino acid	1	AT_Untreated_1_1	AT_Untreated_1	1.0	Amino acid derivatives	True	5.45e+05	-0.9777	-0.9777	-0.2861

Metabolite ID	Name	Super Pathway	Dataset	Sample ID	Group ID	Detection Fraction	Pathway	Detected	Raw Intensity	Log2 Norm Intensity	Norm Imputed	Log2 Ctrl Norm
63	6-P-Gluconate	Carbon	1	AT_Untreated_1_1	AT_Untreated_1	1.0	Pentose phosphate pathway (PPP)	True	3.987e+05	-1.428	-1.428	-3.462
40	Glucuronate	Carbon	1	AT_Untreated_1_1	AT_Untreated_1	1.0	Sugars and sugar alcohols	True	5.282e+05	-1.023	-1.023	0.5165
108	Argininosuccinate	Amino acid	1	AT_Untreated_1_1	AT_Untreated_1	1.0	Amino acids biosynthesis intermediates	True	3.492e+05	-1.62	-1.62	-1.394
710	Carnitine	Cofactor	1	AT_Untreated_1_1	AT_Untreated_1	1.0	Cofactors	True	3.847e+07	5.164	5.164	-0.8136
725	P-Pantetheine	Cofactor	1	AT_Untreated_1_1	AT_Untreated_1	1.0	Coenzyme A biosynthesis	True	4.101e+05	-1.388	-1.388	2.317
48	DHAP	Carbon	1	AT_Untreated_1_1	AT_Untreated_1	1.0	Glycolysis, GNG	True	9.572e+06	3.157	3.157	0.06034
17	Maltose	Carbon	1	AT_Untreated_1_1	AT_Untreated_1	1.0	Glycogen degradation	True	9.003e+05	-0.2535	-0.2535	1.039
359	N1-Me-Adenosine	Nucleotide	1	AT_Untreated_1_1	AT_Untreated_1	0.5	Purine derivatives in RNAs	False			-2.032	-0.9939
159	3-Me-His	Amino acid	1	AT_Untreated_1_1	AT_Untreated_1	1.0	Amino acid derivatives	True	1.491e+04	-6.169	-6.169	-1.934
155	4-Guanidinobutanoate	Amino acid	1	AT_Untreated_1_1	AT_Untreated_1	1.0	Amino acid derivatives	True	1.619e+05	-2.729	-2.729	-1.295
164	5-OH-Lys	Amino acid	1	AT_Untreated_1_1	AT_Untreated_1	1.0	Amino acid derivatives	True	2.273e+05	-2.239	-2.239	-0.4686
357	Adenosine-3',5'-PP	Nucleotide	1	AT_Untreated_1_1	AT_Untreated_1	0.666666666666667	Purine byproducts of metabolic processes	False			-5.09	-1.612
104	Cystathionine	Amino acid	1	AT_Untreated_1_1	AT_Untreated_1	1.0	Amino acids biosynthesis intermediates	True	1.485e+06	0.4686	0.4686	-1.741
113	Imidazole Lactate	Amino acid	1	AT_Untreated_1_1	AT_Untreated_1	0.666666666666667	Amino acids degradation intermediates	True	2.117e+05	-2.342	-2.342	1.334
215	N-Ac-Glu	Amino acid	1	AT_Untreated_1_1	AT_Untreated_1	1.0	N-acetylated amino acids	True	9.7e+05	-0.1459	-0.1459	-1.889
310	S-Lactoyl-Glutathione	Amino acid	1	AT_Untreated_1_1	AT_Untreated_1	1.0	Glutathione derivatives	True	1.203e+05	-3.157	-3.157	-3.514
5	GlcNAc 1-P	Carbon	1	AT_Untreated_1_1	AT_Untreated_1	0.833333333333333	Aminosugar biosynthesis	True	6.63e+05	-0.6948	-0.6948	1.216
34	Ribitol	Carbon	1	AT_Untreated_1_1	AT_Untreated_1	1.0	Sugars and sugar alcohols	True	7.224e+04	-3.893	-3.893	-0.463
10	UDP-Galactose	Carbon	1	AT_Untreated_1_1	AT_Untreated_1	0.833333333333333	Polysaccharide biosynthesis	False			-1.452	-0.8254
13	Guanosine 5'-PP-Fucose	Carbon	1	AT_Untreated_1_1	AT_Untreated_1	1.0	Polysaccharide biosynthesis	True	1.947e+04	-5.785	-5.785	-3.485

Metabolite ID	Name	Super Pathway	Dataset	Sample ID	Group ID	Detection Fraction	Pathway	Detected	Raw Intensity	Log2 Norm Intensity	Norm Imputed	Log2 Ctrl Norm
19	Maltotetraose	Carbon	1	AT_Untreated_1_1	AT_Untreated_1	1.0	Glycogen degradation	True	7.874e+06	2.875	2.875	2.919
233	SAM	Amino acid	1	AT_Untreated_1_1	AT_Untreated_1	0.333333333333333	SAM metabolism	False			-2.121	-1.965
129	5-Aminovalerate	Amino acid	1	AT_Untreated_1_1	AT_Untreated_1	0.333333333333333	Amino acids degradation intermediates	False			0.04171	-0.96
741	5-Me-THF	Cofactor	1	AT_Untreated_1_1	AT_Untreated_1	0.333333333333333	Folate metabolism	False			-5.366	-0.6085
198	Indolelactate	Amino acid	1	AT_Untreated_1_1	AT_Untreated_1	1.0	Amino acid derivatives	True	9.576e+04	-3.486	-3.486	0.3008
254	Gly-Val	Amino acid	1	AT_Untreated_1_1	AT_Untreated_1	1.0	Dipeptides	True	4.594e+06	2.098	2.098	0.9864
291	gamma-Glu-Leu	Amino acid	1	AT_Untreated_1_1	AT_Untreated_1	1.0	Gamma-glutamyl dipeptides	True	3.252e+05	-1.722	-1.722	-1.234
173	Met Sulfoxide	Amino acid	1	AT_Untreated_1_1	AT_Untreated_1	1.0	Amino acid derivatives	True	1.252e+07	3.544	3.544	0.9765
43	Glucose	Carbon	1	AT_Untreated_1_1	AT_Untreated_1	1.0	Glycolysis, GNG	True	1.315e+08	6.937	6.937	1.885
185	Phenyllactate	Amino acid	1	AT_Untreated_1_1	AT_Untreated_1	0.833333333333333	Amino acid derivatives	True	7.067e+04	-3.925	-3.925	1.887
156	Homo-Arg	Amino acid	1	AT_Untreated_1_1	AT_Untreated_1	1.0	Amino acid derivatives	True	1.097e+06	0.03128	0.03128	-1.149
135	Homocitrulline	Amino acid	1	AT_Untreated_1_1	AT_Untreated_1	1.0	Amino acids degradation intermediates	True	2.021e+05	-2.409	-2.409	-0.7063
719	Nicotinamide MN	Cofactor	1	AT_Untreated_1_1	AT_Untreated_1	1.0	NAD biosynthesis	True	3.023e+06	1.494	1.494	1.308
212	N-Ac-Asp	Amino acid	1	AT_Untreated_1_1	AT_Untreated_1	1.0	N-acetylated amino acids	True	9.585e+05	-0.163	-0.163	-0.8603
720	1-Me-Nicotinamide	Cofactor	1	AT_Untreated_1_1	AT_Untreated_1	1.0	Derivatives of NA, nicotinamide and NAD	True	2.227e+08	7.697	7.697	-0.1933
216	N-Ac-Gly	Amino acid	1	AT_Untreated_1_1	AT_Untreated_1	0.5	N-acetylated amino acids	False			-4.155	-0.6151
70	Creatine	Carbon	1	AT_Untreated_1_1	AT_Untreated_1	1.0	Creatine energy storage	True	4.031e+08	8.553	8.553	-0.8641
26	Galactonate	Carbon	1	AT_Untreated_1_1	AT_Untreated_1	0.666666666666667	Sugars and sugar alcohols	True	7.102e+05	-0.5956	-0.5956	0.209
309	Glutathione, Oxidized	Amino acid	1	AT_Untreated_1_1	AT_Untreated_1	1.0	Glutathione	True	2.486e+06	1.212	1.212	0.01937
35	Ribonate	Carbon	1	AT_Untreated_1_1	AT_Untreated_1	0.833333333333333	Sugars and sugar alcohols	True	4.95e+05	-1.117	-1.117	-0.5893
160	1-Me-His	Amino acid	1	AT_Untreated_1_1	AT_Untreated_1	1.0	Amino acid derivatives	True	7.131e+06	2.732	2.732	-0.709

Metabolite ID	Name	Super Pathway	Dataset	Sample ID	Group ID	Detection Fraction	Pathway	Detected	Raw Intensity	Log2 Norm Intensity	Norm Imputed	Log2 Ctrl Norm
44	Glucose 6-P	Carbon	1	AT_Untreated_1_1	AT_Untreated_1	0.833333333333333	Glycolysis, GNG	True	3.226e+05	-1.734	-1.734	-0.2576
704	NADH	Cofactor	1	AT_Untreated_1_1	AT_Untreated_1	1.0	Cofactors	True	3.926e+04	-4.773	-4.773	-4.127
275	Thr-Phe	Amino acid	1	AT_Untreated_1_1	AT_Untreated_1	0.833333333333333	Dipeptides	True	2.54e+05	-2.079	-2.079	-0.426
738	Pyridoxate	Cofactor	1	AT_Untreated_1_1	AT_Untreated_1	1.0	PLP biosynthesis and salvage	True	1.316e+05	-3.028	-3.028	0.3368
177	3-(4-OH-Phenyl)Lactate	Amino acid	1	AT_Untreated_1_1	AT_Untreated_1	1.0	Amino acid derivatives	True	2.034e+05	-2.4	-2.4	0.06547
206	Trans-4-OH-Pro	Amino acid	1	AT_Untreated_1_1	AT_Untreated_1	1.0	Amino acid derivatives	True	1.838e+07	4.098	4.098	-1.057
329	AMP	Nucleotide	1	AT_Untreated_1_1	AT_Untreated_1	1.0	Purine nucleotides	True	1.029e+06	-0.05999	-0.05999	-3.695
11	UDP-Glucose	Carbon	1	AT_Untreated_1_1	AT_Untreated_1	0.833333333333333	Polysaccharide biosynthesis	False			-3.865	-3.76
158	4-Imidazole-Ac	Amino acid	1	AT_Untreated_1_1	AT_Untreated_1	1.0	Amino acid derivatives	True	1.929e+05	-2.476	-2.476	-0.3295
111	1-Me-Imidazole-Ac	Amino acid	1	AT_Untreated_1_1	AT_Untreated_1	1.0	Amino acids degradation intermediates	True	3.61e+05	-1.572	-1.572	0.3618
345	Guanine	Nucleotide	1	AT_Untreated_1_1	AT_Untreated_1	1.0	Purine bases	True	1.035e+08	6.591	6.591	1.605
22	N-Ac-Neuraminate	Carbon	1	AT_Untreated_1_1	AT_Untreated_1	1.0	Aminosugar derivatives	True	1.93e+06	0.847	0.847	0.9824
721	N'-Methylnicotinate	Cofactor	1	AT_Untreated_1_1	AT_Untreated_1	1.0	Derivatives of NA, nicotinamide and NAD	True	4.559e+05	-1.235	-1.235	-0.2276
183	Phenol Sulfate	Amino acid	1	AT_Untreated_1_1	AT_Untreated_1	1.0	Amino acid derivatives	True	2.012e+04	-5.737	-5.737	-1.097
718	Nicotinamide Riboside	Cofactor	1	AT_Untreated_1_1	AT_Untreated_1	1.0	NAD biosynthesis	True	6.327e+07	5.881	5.881	4.291
297	gamma-Glu-Thr	Amino acid	1	AT_Untreated_1_1	AT_Untreated_1	1.0	Gamma-glutamyl dipeptides	True	4.545e+05	-1.239	-1.239	-1.88
295	gamma-Glu-Phe	Amino acid	1	AT_Untreated_1_1	AT_Untreated_1	0.166666666666667	Gamma-glutamyl dipeptides	False			-5.975	-0.1899
347	Allantoic Acid	Nucleotide	1	AT_Untreated_1_1	AT_Untreated_1	0.666666666666667	Purine degradation	False			-6.474	-0.8345
399	Pseudouridine	Nucleotide	1	AT_Untreated_1_1	AT_Untreated_1	1.0	Pyrimidine derivatives in RNAs	True	4.174e+05	-1.362	-1.362	0.6676
375	UTP	Nucleotide	1	AT_Untreated_1_1	AT_Untreated_1	0.666666666666667	Pyrimidine nucleotides	False			-5.025	-3.049
144	Glu, gamma-Me Ester	Amino acid	1	AT_Untreated_1_1	AT_Untreated_1	1.0	Amino acid derivatives	True	4.593e+05	-1.225	-1.225	-1.802

Metabolite ID	Name	Super Pathway	Dataset	Sample ID	Group ID	Detection Fraction	Pathway	Detected	Raw Intensity	Log2 Norm Intensity	Norm Imputed	Log2 Ctrl Norm
292	gamma-Glu-epsilon-Lysine	Amino acid	1	AT_Untreated_1_1	AT_Untreated_1	1.0	Gamma-glutamyl dipeptides	True	1.309e+06	0.2865	0.2865	0.4779
225	N-Ac-Thr	Amino acid	1	AT_Untreated_1_1	AT_Untreated_1	1.0	N-acetylated amino acids	True	2.2e+05	-2.286	-2.286	-0.7836
211	N-Ac-Asn	Amino acid	1	AT_Untreated_1_1	AT_Untreated_1	1.0	N-acetylated amino acids	True	6.838e+04	-3.972	-3.972	-1.287
151	Phenylacetylglycine	Amino acid	1	AT_Untreated_1_1	AT_Untreated_1	1.0	Amino acid derivatives	True	1.582e+06	0.5595	0.5595	1.346
217	N-Ac-His	Amino acid	1	AT_Untreated_1_1	AT_Untreated_1	0.833333333333333	N-acetylated amino acids	False			-4.477	-0.9917
288	gamma-Glu-Gly	Amino acid	1	AT_Untreated_1_1	AT_Untreated_1	0.333333333333333	Gamma-glutamyl dipeptides	False			-3.092	-1.268
222	N-Ac-Phe	Amino acid	1	AT_Untreated_1_1	AT_Untreated_1	0.333333333333333	N-acetylated amino acids	False			-6.698	-0.4255
71	Creatine-P	Carbon	1	AT_Untreated_1_1	AT_Untreated_1	1.0	Creatine energy storage	True	8.953e+04	-3.583	-3.583	0.6717
210	N-Ac-Arg	Amino acid	1	AT_Untreated_1_1	AT_Untreated_1	0.666666666666667	N-acetylated amino acids	False			-4.025	-1.35
218	N-Ac-Ile	Amino acid	1	AT_Untreated_1_1	AT_Untreated_1	0.166666666666667	N-acetylated amino acids	False			-6.52	-0.5541
251	Gly-Leu	Amino acid	1	AT_Untreated_1_1	AT_Untreated_1	1.0	Dipeptides	True	4.83e+06	2.17	2.17	0.5313
290	gamma-Glu-Ile	Amino acid	1	AT_Untreated_1_1	AT_Untreated_1	1.0	Gamma-glutamyl dipeptides	True	8.34e+04	-3.686	-3.686	-2.347
316	Ophthalmate	Amino acid	1	AT_Untreated_1_1	AT_Untreated_1	1.0	Oxidative stress markers	True	1.337e+06	0.3174	0.3174	-2.025
125	Isovaleryl-Gly	Amino acid	1	AT_Untreated_1_1	AT_Untreated_1	1.0	Amino acids degradation intermediates	True	5.375e+04	-4.319	-4.319	1.09
368	7-Me-Guanine	Nucleotide	1	AT_Untreated_1_1	AT_Untreated_1	1.0	Purine derivatives in RNAs	True	2.279e+05	-2.235	-2.235	-0.04521
208	Pro-OH-Pro	Amino acid	1	AT_Untreated_1_1	AT_Untreated_1	1.0	Amino acid derivatives	True	5.714e+06	2.413	2.413	-0.9167
366	N2,N2-DiMe-Guanosine	Nucleotide	1	AT_Untreated_1_1	AT_Untreated_1	0.833333333333333	Purine derivatives in RNAs	True	9.604e+04	-3.482	-3.482	0.6315
352	3'-AMP	Nucleotide	1	AT_Untreated_1_1	AT_Untreated_1	1.0	Purine derivatives in signaling	True	5.447e+05	-0.9784	-0.9784	0.5084
363	N6-Carbamoyl-Thr-Adenosine	Nucleotide	1	AT_Untreated_1_1	AT_Untreated_1	0.666666666666667	Purine derivatives in RNAs	False			-6.008	-1.323
314	Cys-Glutathione Disulfide	Amino acid	1	AT_Untreated_1_1	AT_Untreated_1	1.0	Oxidative stress markers	True	5.576e+05	-0.9447	-0.9447	2.339
382	Orotidine	Nucleotide	1	AT_Untreated_1_1	AT_Untreated_1	0.166666666666667	Pyrimidine (UMP) biosynthesis	False			-5.287	-0.9952

Metabolite ID	Name	Super Pathway	Dataset	Sample ID	Group ID	Detection Fraction	Pathway	Detected	Raw Intensity	Log2 Norm Intensity	Norm Imputed	Log2 Ctrl Norm
307	Cys-Gly	Amino acid	1	AT_Untreated_1_1	AT_Untreated_1	1.0	Glutathione biosynthesis	True	5.977e+05	-0.8445	-0.8445	-2.451
64	Sedoheptulose-7-P	Carbon	1	AT_Untreated_1_1	AT_Untreated_1	1.0	Pentose phosphate pathway (PPP)	True	1.227e+06	0.1931	0.1931	0.03903
142	N-Ac-Asp-Glu	Amino acid	1	AT_Untreated_1_1	AT_Untreated_1	1.0	Amino acid derivatives	True	2.116e+05	-2.343	-2.343	-1.197
708	Thiamin-PP	Cofactor	1	AT_Untreated_1_1	AT_Untreated_1	0.5	Cofactors	False			-6.331	-0.3472
182	P-Cresol Sulfate	Amino acid	1	AT_Untreated_1_1	AT_Untreated_1	1.0	Amino acid derivatives	True	1.675e+05	-2.68	-2.68	0.5166
250	Gly-Ile	Amino acid	1	AT_Untreated_1_1	AT_Untreated_1	1.0	Dipeptides	True	6.735e+05	-0.6723	-0.6723	0.6449
286	gamma-Glu-Glu	Amino acid	1	AT_Untreated_1_1	AT_Untreated_1	1.0	Gamma-glutamyl dipeptides	True	3.707e+05	-1.534	-1.534	-1.551
739	Deoxycarnitine	Cofactor	1	AT_Untreated_1_1	AT_Untreated_1	1.0	Carnitine biosynthesis	True	5.548e+06	2.37	2.37	-1.381
203	DiMe-Arg	Amino acid	1	AT_Untreated_1_1	AT_Untreated_1	1.0	Amino acid derivatives	True	6.222e+07	5.857	5.857	0.2247
351	2'-AMP	Nucleotide	1	AT_Untreated_1_1	AT_Untreated_1	1.0	Purine derivatives in signaling	True	6.435e+05	-0.738	-0.738	1.531
8	Cytidine 5'-P-N-Ac-Neuraminate	Carbon	1	AT_Untreated_1_1	AT_Untreated_1	1.0	Aminosugar biosynthesis	True	2.958e+05	-1.859	-1.859	-0.1136
285	gamma-Glu-Ala	Amino acid	1	AT_Untreated_1_1	AT_Untreated_1	0.5	Gamma-glutamyl dipeptides	False			-6.022	-2.783
224	N-Ac-Ser	Amino acid	1	AT_Untreated_1_1	AT_Untreated_1	1.0	N-acetylated amino acids	True	2.082e+06	0.9563	0.9563	-0.923
244	Ala-Leu	Amino acid	1	AT_Untreated_1_1	AT_Untreated_1	1.0	Dipeptides	True	2.655e+06	1.307	1.307	0.1686
207	N-Me-Pro	Amino acid	1	AT_Untreated_1_1	AT_Untreated_1	1.0	Amino acid derivatives	True	5.201e+05	-1.045	-1.045	-0.1149
171	Cys Sulfinic Acid	Amino acid	1	AT_Untreated_1_1	AT_Untreated_1	0.333333333333333	Amino acid derivatives	False			-5.211	-1.913
181	O-Me-Tyr	Amino acid	1	AT_Untreated_1_1	AT_Untreated_1	0.5	Amino acid derivatives	False			-4.309	-0.9396
240	N-Ac-Putrescine	Amino acid	1	AT_Untreated_1_1	AT_Untreated_1	0.833333333333333	Polyamine derivatives	False			-5.717	-3.353
176	S-Me-Met	Amino acid	1	AT_Untreated_1_1	AT_Untreated_1	1.0	Amino acid derivatives	True	1.163e+05	-3.205	-3.205	-1.364
339	AICAR	Nucleotide	1	AT_Untreated_1_1	AT_Untreated_1	0.333333333333333	IMP biosynthesis	False			-4.716	-0.4151
141	gamma-Carboxy-Glu	Amino acid	1	AT_Untreated_1_1	AT_Untreated_1	1.0	Amino acid derivatives	True	4.612e+05	-1.218	-1.218	-0.4334
392	3'-UMP	Nucleotide	1	AT_Untreated_1_1	AT_Untreated_1	0.833333333333333	Pyrimidine derivatives in signaling	True	4.966e+05	-1.112	-1.112	2.921

Metabolite ID	Name	Super Pathway	Dataset	Sample ID	Group ID	Detection Fraction	Pathway	Detected	Raw Intensity	Log2 Norm Intensity	Norm Imputed	Log2 Ctrl Norm
355	3'-GMP	Nucleotide	1	AT_Untreated_1_1	AT_Untreated_1	0.833333333333333	Purine derivatives in signaling	True	1.359e+05	-2.981	-2.981	0.956
282	Val-Leu	Amino acid	1	AT_Untreated_1_1	AT_Untreated_1	1.0	Dipeptides	True	1.274e+06	0.2479	0.2479	-0.7238
140	Carboxyethyl-GABA	Amino acid	1	AT_Untreated_1_1	AT_Untreated_1	1.0	Amino acid derivatives	True	2.243e+05	-2.259	-2.259	-0.8275
258	Ile-Gly	Amino acid	1	AT_Untreated_1_1	AT_Untreated_1	1.0	Dipeptides	True	1.262e+07	3.556	3.556	0.244
260	Leu-Ala	Amino acid	1	AT_Untreated_1_1	AT_Untreated_1	1.0	Dipeptides	True	1.89e+06	0.8164	0.8164	-0.4848
265	Lys-Leu	Amino acid	1	AT_Untreated_1_1	AT_Untreated_1	0.833333333333333	Dipeptides	True	1.936e+05	-2.471	-2.471	0.1406
263	Leu-Gly	Amino acid	1	AT_Untreated_1_1	AT_Untreated_1	1.0	Dipeptides	True	2.015e+07	4.231	4.231	1.21
281	Val-Gly	Amino acid	1	AT_Untreated_1_1	AT_Untreated_1	1.0	Dipeptides	True	2.954e+07	4.783	4.783	0.2634
270	Pro-Gly	Amino acid	1	AT_Untreated_1_1	AT_Untreated_1	1.0	Dipeptides	True	1.851e+06	0.7861	0.7861	0.03933
114	Imidazole Propionate	Amino acid	1	AT_Untreated_1_1	AT_Untreated_1	1.0	Amino acids degradation intermediates	True	4.208e+05	-1.351	-1.351	0.9598
267	Phe-Gly	Amino acid	1	AT_Untreated_1_1	AT_Untreated_1	1.0	Dipeptides	True	2.546e+07	4.568	4.568	0.4744
266	Phe-Ala	Amino acid	1	AT_Untreated_1_1	AT_Untreated_1	1.0	Dipeptides	True	2.512e+06	1.227	1.227	0.1501
278	Tyr-Gly	Amino acid	1	AT_Untreated_1_1	AT_Untreated_1	1.0	Dipeptides	True	7.641e+06	2.832	2.832	0.6496
255	His-Ala	Amino acid	1	AT_Untreated_1_1	AT_Untreated_1	0.833333333333333	Dipeptides	True	6.782e+05	-0.6621	-0.6621	0.6753
280	Val-Gln	Amino acid	1	AT_Untreated_1_1	AT_Untreated_1	1.0	Dipeptides	True	2.742e+06	1.353	1.353	-0.265
143	S-1-Pyrroline-5-Carboxylate	Amino acid	1	AT_Untreated_1_1	AT_Untreated_1	0.833333333333333	Amino acid derivatives	True	2.514e+05	-2.094	-2.094	-0.02553
232	SAH	Amino acid	1	AT_Untreated_1_1	AT_Untreated_1	1.0	SAM metabolism	True	6.243e+05	-0.7816	-0.7816	-0.2962
20	Erythronate	Carbon	1	AT_Untreated_1_1	AT_Untreated_1	1.0	Aminosugar derivatives	True	1.112e+07	3.373	3.373	-0.3683
248	Gln-Leu	Amino acid	1	AT_Untreated_1_1	AT_Untreated_1	0.833333333333333	Dipeptides	True	5.48e+05	-0.9697	-0.9697	-0.06451
276	Trp-Gly	Amino acid	1	AT_Untreated_1_1	AT_Untreated_1	0.833333333333333	Dipeptides	True	4.991e+05	-1.104	-1.104	-0.02608
205	N-delta-Ac-Ornithine	Amino acid	1	AT_Untreated_1_1	AT_Untreated_1	1.0	Amino acid derivatives	True	3.141e+05	-1.773	-1.773	-0.6518
163	Formimino-Glu	Amino acid	1	AT_Untreated_1_1	AT_Untreated_1	1.0	Amino acid derivatives	True	5.937e+05	-0.8542	-0.8542	0.3856
204	N-Me-Arg	Amino acid	1	AT_Untreated_1_1	AT_Untreated_1	0.833333333333333	Amino acid derivatives	True	1.214e+07	3.499	3.499	0.9124
242	Guanidino-Ac	Amino acid	1	AT_Untreated_1_1	AT_Untreated_1	1.0	Creatine biosynthesis	True	8.785e+04	-3.611	-3.611	0.2696

Metabolite ID	Name	Super Pathway	Dataset	Sample ID	Group ID	Detection Fraction	Pathway	Detected	Raw Intensity	Log2 Norm Intensity	Norm Imputed	Log2 Ctrl Norm
300	gamma-Glu-Val	Amino acid	1	AT_Untreated_1_1	AT_Untreated_1	0.333333333333333	Gamma-glutamyl dipeptides	False			-0.1053	-0.726
53	Ac-CoA	Carbon	1	AT_Untreated_1_1	AT_Untreated_1	0.166666666666667	Acetyl-CoA	False			-7.274	-0.5114
18	Maltotriose	Carbon	1	AT_Untreated_1_1	AT_Untreated_1	1.0	Glycogen degradation	True	4.449e+06	2.052	2.052	2.209
294	gamma-Glu-Met	Amino acid	1	AT_Untreated_1_1	AT_Untreated_1	0.333333333333333	Gamma-glutamyl dipeptides	False			-4.503	-1.021
174	Met Sulfone	Amino acid	1	AT_Untreated_1_1	AT_Untreated_1	0.833333333333333	Amino acid derivatives	False			-4.307	-1.229
175	N-Ac-Met Sulfoxide	Amino acid	1	AT_Untreated_1_1	AT_Untreated_1	1.0	Amino acid derivatives	True	1.537e+06	0.518	0.518	0.5417
25	Mannitol/Sorbitol	Carbon	1	AT_Untreated_1_1	AT_Untreated_1	1.0	Sugars and sugar alcohols	True	8.392e+06	2.967	2.967	0.7426
6	UDP-GlcNAc	Carbon	1	AT_Untreated_1_1	AT_Untreated_1	0.166666666666667	Aminosugar biosynthesis	False			-3.589	-0.4814
145	Pyro-Gln	Amino acid	1	AT_Untreated_1_1	AT_Untreated_1	1.0	Amino acid derivatives	True	1.9e+06	0.8239	0.8239	-0.01584
705	Coenzyme A	Cofactor	1	AT_Untreated_1_1	AT_Untreated_1	0.666666666666667	Cofactors	False			-4.967	-1.008
319	2'-dAMP	Nucleotide	1	AT_Untreated_1_1	AT_Untreated_1	0.166666666666667	Deoxy-nucleotides	False			-4.337	-0.6568
119	alpha-OH-Isovalerate	Amino acid	1	AT_Untreated_1_1	AT_Untreated_1	1.0	Amino acids degradation intermediates	True	4.052e+05	-1.405	-1.405	1.893
46	Fructose 1,6-PP / Glucose 1,6-PP / Inositol-1,4-PP / Inositol-1,3-PP	Carbon	1	AT_Untreated_1_1	AT_Untreated_1	1.0	Glycolysis, GNG	True	2.941e+06	1.454	1.454	-3.377
137	1-Me-Guanidine	Amino acid	1	AT_Untreated_1_1	AT_Untreated_1	0.666666666666667	Amino acids degradation intermediates	False			-5.942	-0.7895
23	N-GlcNAc-Asn	Carbon	1	AT_Untreated_1_1	AT_Untreated_1	1.0	Aminosugar derivatives	True	7.211e+05	-0.5737	-0.5737	-1.287
262	Leu-Gln	Amino acid	1	AT_Untreated_1_1	AT_Untreated_1	1.0	Dipeptides	True	1.53e+06	0.5112	0.5112	-0.7851
24	Fructose	Carbon	1	AT_Untreated_1_1	AT_Untreated_1	1.0	Sugars and sugar alcohols	True	6.482e+06	2.594	2.594	0.6456
197	C-Glycosyl-Trp	Amino acid	1	AT_Untreated_1_1	AT_Untreated_1	1.0	Amino acid derivatives	True	1.347e+06	0.3281	0.3281	0.6271
33	Arabitol/Xylitol	Carbon	1	AT_Untreated_1_1	AT_Untreated_1	1.0	Sugars and sugar alcohols	True	1.593e+05	-2.752	-2.752	-0.8922

Metabolite ID	Name	Super Pathway	Dataset	Sample ID	Group ID	Detection Fraction	Pathway	Detected	Raw Intensity	Log2 Norm Intensity	Norm Imputed	Log2 Ctrl Norm
128	N2-Ac-Lys/N6-Ac-Lys	Amino acid	1	AT_Untreated_1_1	AT_Untreated_1	1.0	Amino acids degradation intermediates	True	1.525e+06	0.507	0.507	-0.7034
42	2-Me-Citrate	Carbon	1	AT_Untreated_1_1	AT_Untreated_1	0.833333333333333	Propionate metabolism	True	1.698e+05	-2.66	-2.66	1.723
12	Glucuronate 1-P	Carbon	1	AT_Untreated_1_1	AT_Untreated_1	1.0	Polysaccharide biosynthesis	True	2.436e+05	-2.14	-2.14	-0.8967
76	Gln	Amino acid	1	AT_Untreated_1_2	AT_Untreated_1	1.0	Proteinogenic amino acids	True	8.884e+08	8.238	8.238	-0.4424
89	Trp	Amino acid	1	AT_Untreated_1_2	AT_Untreated_1	1.0	Proteinogenic amino acids	True	1.04e+08	5.144	5.144	-0.5319
723	beta-Ala	Cofactor	1	AT_Untreated_1_2	AT_Untreated_1	1.0	Coenzyme A biosynthesis	True	1.275e+07	2.116	2.116	-0.8932
75	Glu	Amino acid	1	AT_Untreated_1_2	AT_Untreated_1	1.0	Proteinogenic amino acids	True	3.693e+09	10.29	10.29	-0.4429
77	Gly	Amino acid	1	AT_Untreated_1_2	AT_Untreated_1	1.0	Proteinogenic amino acids	True	1.099e+08	5.223	5.223	-0.3926
80	His	Amino acid	1	AT_Untreated_1_2	AT_Untreated_1	1.0	Proteinogenic amino acids	True	2.482e+07	3.076	3.076	-0.3388
82	Leu	Amino acid	1	AT_Untreated_1_2	AT_Untreated_1	1.0	Proteinogenic amino acids	True	1.904e+09	9.338	9.338	0.1164
87	Phe	Amino acid	1	AT_Untreated_1_2	AT_Untreated_1	1.0	Proteinogenic amino acids	True	1.354e+09	8.846	8.846	0.0348
130	Glutarate	Amino acid	1	AT_Untreated_1_2	AT_Untreated_1	1.0	Amino acids degradation intermediates	True	5.704e+05	-2.367	-2.367	0.05989
196	5-OH-Indole-Ac	Amino acid	1	AT_Untreated_1_2	AT_Untreated_1	1.0	Amino acid derivatives	True	3.296e+05	-3.158	-3.158	0.8536
74	Asp	Amino acid	1	AT_Untreated_1_2	AT_Untreated_1	1.0	Proteinogenic amino acids	True	4.449e+08	7.241	7.241	-0.6511
236	Spermidine	Amino acid	1	AT_Untreated_1_2	AT_Untreated_1	1.0	Polyamines	True	3.113e+07	3.403	3.403	-0.4583
73	Asn	Amino acid	1	AT_Untreated_1_2	AT_Untreated_1	1.0	Proteinogenic amino acids	True	2.511e+08	6.415	6.415	-0.256
243	Creatinine	Amino acid	1	AT_Untreated_1_2	AT_Untreated_1	1.0	Creatine degradation	True	1.15e+08	5.289	5.289	0.2863
376	Cytidine	Nucleotide	1	AT_Untreated_1_2	AT_Untreated_1	0.833333333333333	Pyrimidine nucleosides	True	1.035e+06	-1.507	-1.507	-2.235
41	Lactate	Carbon	1	AT_Untreated_1_2	AT_Untreated_1	1.0	Respiratory carbon sources	True	1.809e+08	5.942	5.942	-0.5399
58	alpha-Ketoglutarate	Carbon	1	AT_Untreated_1_2	AT_Untreated_1	1.0	TCA cycle	True	3.205e+06	0.1237	0.1237	0.107
69	3-OH-Butyrate	Carbon	1	AT_Untreated_1_2	AT_Untreated_1	0.833333333333333	Ketone bodies	True	1.884e+06	-0.6433	-0.6433	2.341

Metabolite ID	Name	Super Pathway	Dataset	Sample ID	Group ID	Detection Fraction	Pathway	Detected	Raw Intensity	Log2 Norm Intensity	Norm Imputed	Log2 Ctrl Norm
343	Adenine	Nucleotide	1	AT_Untreated_1_2	AT_Untreated_1	1.0	Purine bases	True	3.637e+06	0.306	0.306	-1.151
336	Adenosine	Nucleotide	1	AT_Untreated_1_2	AT_Untreated_1	1.0	Purine nucleosides	True	5.47e+07	4.217	4.217	0.7939
722	ADP-Ribose	Cofactor	1	AT_Untreated_1_2	AT_Untreated_1	1.0	Derivatives of NA, nicotinamide and NAD	True	1.005e+07	1.773	1.773	4.775
383	Cytosine	Nucleotide	1	AT_Untreated_1_2	AT_Untreated_1	0.833333333333333	Pyrimidine bases	True	5.31e+05	-2.47	-2.47	2.385
3	Glucosamine 6-P	Carbon	1	AT_Untreated_1_2	AT_Untreated_1	0.833333333333333	Aminosugar biosynthesis	True	1.702e+06	-0.7899	-0.7899	0.8774
717	Nicotinamide	Cofactor	1	AT_Untreated_1_2	AT_Untreated_1	1.0	NAD biosynthesis	True	3.691e+08	6.971	6.971	2.456
51	PEP	Carbon	1	AT_Untreated_1_2	AT_Untreated_1	1.0	Glycolysis, GNG	True	4.259e+06	0.5336	0.5336	-0.5176
237	Spermine	Amino acid	1	AT_Untreated_1_2	AT_Untreated_1	1.0	Polyamines	True	1.002e+06	-1.553	-1.553	-0.06267
385	Uracil	Nucleotide	1	AT_Untreated_1_2	AT_Untreated_1	1.0	Pyrimidine bases	True	1.208e+07	2.038	2.038	1.773
377	Uridine	Nucleotide	1	AT_Untreated_1_2	AT_Untreated_1	1.0	Pyrimidine nucleosides	True	7.945e+07	4.755	4.755	0.7063
348	Allantoin	Nucleotide	1	AT_Untreated_1_2	AT_Untreated_1	1.0	Purine degradation	True	2.909e+06	-0.0165	-0.0165	0.1591
335	Inosine	Nucleotide	1	AT_Untreated_1_2	AT_Untreated_1	1.0	Purine nucleosides	True	1.473e+08	5.645	5.645	0.1464
81	Ile	Amino acid	1	AT_Untreated_1_2	AT_Untreated_1	1.0	Proteinogenic amino acids	True	1.25e+09	8.73	8.73	0.01527
72	Ala	Amino acid	1	AT_Untreated_1_2	AT_Untreated_1	1.0	Proteinogenic amino acids	True	8.076e+08	8.101	8.101	-0.4008
79	Thr	Amino acid	1	AT_Untreated_1_2	AT_Untreated_1	1.0	Proteinogenic amino acids	True	6.692e+08	7.83	7.83	-0.18
88	Tyr	Amino acid	1	AT_Untreated_1_2	AT_Untreated_1	1.0	Proteinogenic amino acids	True	5.703e+08	7.599	7.599	0.0751
84	Lys	Amino acid	1	AT_Untreated_1_2	AT_Untreated_1	1.0	Proteinogenic amino acids	True	6.256e+08	7.732	7.732	-0.2577
86	Met	Amino acid	1	AT_Untreated_1_2	AT_Untreated_1	1.0	Proteinogenic amino acids	True	9.662e+08	8.359	8.359	0.1317
61	Malate	Carbon	1	AT_Untreated_1_2	AT_Untreated_1	1.0	TCA cycle	True	2.822e+08	6.584	6.584	-0.5879
235	Putrescine	Amino acid	1	AT_Untreated_1_2	AT_Untreated_1	0.833333333333333	Polyamines	True	1.193e+07	2.02	2.02	2.512
324	2'-dU	Nucleotide	1	AT_Untreated_1_2	AT_Untreated_1	0.666666666666667	Deoxy-nucleosides	False			-5.281	-0.9009
49	3-P-Glycerate	Carbon	1	AT_Untreated_1_2	AT_Untreated_1	1.0	Glycolysis, GNG	True	1.627e+07	2.468	2.468	-0.7979
189	Kynurenate	Amino acid	1	AT_Untreated_1_2	AT_Untreated_1	1.0	Amino acid derivatives	True	1.377e+05	-4.417	-4.417	1.381
234	5-Me-Thioadenosine	Amino acid	1	AT_Untreated_1_2	AT_Untreated_1	1.0	SAM metabolism	True	6.07e+06	1.045	1.045	-0.8407

Metabolite ID	Name	Super Pathway	Dataset	Sample ID	Group ID	Detection Fraction	Pathway	Detected	Raw Intensity	Log2 Norm Intensity	Norm Imputed	Log2 Ctrl Norm
59	Succinate	Carbon	1	AT_Untreated_1_2	AT_Untreated_1	1.0	TCA cycle	True	4.723e+06	0.683	0.683	0.3819
36	Ribose	Carbon	1	AT_Untreated_1_2	AT_Untreated_1	1.0	Sugars and sugar alcohols	True	1.272e+06	-1.21	-1.21	0.02374
133	Ornithine	Amino acid	1	AT_Untreated_1_2	AT_Untreated_1	1.0	Amino acids degradation intermediates	True	4.885e+07	4.053	4.053	-0.4261
313	5-Oxoproline	Amino acid	1	AT_Untreated_1_2	AT_Untreated_1	1.0	Glutathione derivatives	True	8.862e+06	1.591	1.591	-0.4967
165	N-6-Tri-Me-Lys	Amino acid	1	AT_Untreated_1_2	AT_Untreated_1	1.0	Amino acid derivatives	True	3.684e+07	3.646	3.646	-0.1173
380	Orotate	Nucleotide	1	AT_Untreated_1_2	AT_Untreated_1	0.666666666666667	Pyrimidine (UMP) biosynthesis	True	2.281e+05	-3.689	-3.689	-0.05873
724	Pantothenate	Cofactor	1	AT_Untreated_1_2	AT_Untreated_1	1.0	Coenzyme A biosynthesis	True	1.415e+08	5.588	5.588	-0.7221
150	N-Me-Gly	Amino acid	1	AT_Untreated_1_2	AT_Untreated_1	1.0	Amino acid derivatives	True	1.733e+06	-0.7635	-0.7635	-0.4631
122	3-OH-Isobutyrate	Amino acid	1	AT_Untreated_1_2	AT_Untreated_1	0.833333333333333	Amino acids degradation intermediates	True	4.035e+05	-2.866	-2.866	0.2898
241	4-Acetamidobutanoate	Amino acid	1	AT_Untreated_1_2	AT_Untreated_1	1.0	Polyamine derivatives	True	7.128e+06	1.277	1.277	0.8356
711	alpha-Tocopherol	Cofactor	1	AT_Untreated_1_2	AT_Untreated_1	1.0	Cofactors	True	3.556e+06	0.2734	0.2734	-0.5069
55	Citrate	Carbon	1	AT_Untreated_1_2	AT_Untreated_1	1.0	TCA cycle	True	1.808e+07	2.62	2.62	0.6705
387	3-Aminoisobutyrate	Nucleotide	1	AT_Untreated_1_2	AT_Untreated_1	0.833333333333333	Pyrimidine degradation	True	6.468e+05	-2.185	-2.185	-0.1716
338	Guanosine	Nucleotide	1	AT_Untreated_1_2	AT_Untreated_1	1.0	Purine nucleosides	True	1.924e+08	6.031	6.031	0.925
209	N-Ac-Ala	Amino acid	1	AT_Untreated_1_2	AT_Untreated_1	1.0	N-acetylated amino acids	True	9.579e+05	-1.619	-1.619	-0.4176
221	N-Ac-Met	Amino acid	1	AT_Untreated_1_2	AT_Untreated_1	1.0	N-acetylated amino acids	True	6.984e+06	1.247	1.247	-0.497
228	N-Ac-Val	Amino acid	1	AT_Untreated_1_2	AT_Untreated_1	0.333333333333333	N-acetylated amino acids	True	7.125e+04	-5.368	-5.368	3.00e-03
346	Urate	Nucleotide	1	AT_Untreated_1_2	AT_Untreated_1	1.0	Purine degradation	True	2.905e+06	-0.01817	-0.01817	1.045
90	Arg	Amino acid	1	AT_Untreated_1_2	AT_Untreated_1	1.0	Proteinogenic amino acids	True	2.572e+09	9.772	9.772	0.06623
60	Fumarate	Carbon	1	AT_Untreated_1_2	AT_Untreated_1	1.0	TCA cycle	True	3.928e+06	0.4172	0.4172	-0.598
78	Ser	Amino acid	1	AT_Untreated_1_2	AT_Untreated_1	1.0	Proteinogenic amino acids	True	4.426e+08	7.233	7.233	-0.02438
83	Val	Amino acid	1	AT_Untreated_1_2	AT_Untreated_1	1.0	Proteinogenic amino acids	True	7.429e+08	7.98	7.98	0.108

Metabolite ID	Name	Super Pathway	Dataset	Sample ID	Group ID	Detection Fraction	Pathway	Detected	Raw Intensity	Log2 Norm Intensity	Norm Imputed	Log2 Ctrl Norm
734	Pyridoxal	Cofactor	1	AT_Untreated_1_2	AT_Untreated_1	1.0	PLP biosynthesis and salvage	True	2.635e+07	3.163	3.163	1.38
136	Urea	Amino acid	1	AT_Untreated_1_2	AT_Untreated_1	1.0	Amino acids degradation intermediates	True	5.033e+06	0.7746	0.7746	0.6234
67	Ribose 1-P	Carbon	1	AT_Untreated_1_2	AT_Untreated_1	1.0	Pentose phosphate pathway (PPP)	True	4.963e+06	0.7543	0.7543	0.1331
284	Carnosine	Amino acid	1	AT_Untreated_1_2	AT_Untreated_1	1.0	Dipeptides	True	7.949e+05	-1.888	-1.888	0.2203
306	gamma-Glu-Cys	Amino acid	1	AT_Untreated_1_2	AT_Untreated_1	1.0	Glutathione biosynthesis	True	1.118e+06	-1.396	-1.396	-1.059
712	Retinol (Vit A)	Cofactor	1	AT_Untreated_1_2	AT_Untreated_1	0.833333333333333	Cofactors	True	6.82e+05	-2.109	-2.109	0.3244
85	Cys	Amino acid	1	AT_Untreated_1_2	AT_Untreated_1	1.0	Proteinogenic amino acids	True	5.623e+07	4.257	4.257	-0.2572
91	Pro	Amino acid	1	AT_Untreated_1_2	AT_Untreated_1	1.0	Proteinogenic amino acids	True	1.714e+09	9.186	9.186	-0.2215
308	Glutathione, Reduced	Amino acid	1	AT_Untreated_1_2	AT_Untreated_1	1.0	Glutathione	True	2.176e+08	6.209	6.209	-0.7472
107	Citrulline	Amino acid	1	AT_Untreated_1_2	AT_Untreated_1	1.0	Amino acids biosynthesis intermediates	True	2.031e+07	2.788	2.788	-0.03678
328	IMP	Nucleotide	1	AT_Untreated_1_2	AT_Untreated_1	0.333333333333333	Purine nucleotides	False			-4.53	-0.2088
706	FAD	Cofactor	1	AT_Untreated_1_2	AT_Untreated_1	1.0	Cofactors	True	9.919e+05	-1.568	-1.568	0.621
735	Pyridoxamine	Cofactor	1	AT_Untreated_1_2	AT_Untreated_1	1.0	PLP biosynthesis and salvage	True	4.033e+06	0.4549	0.4549	0.6479
199	Serotonin	Amino acid	1	AT_Untreated_1_2	AT_Untreated_1	1.0	Amino acid derivatives	True	6.821e+06	1.213	1.213	-0.05951
370	CMP	Nucleotide	1	AT_Untreated_1_2	AT_Untreated_1	1.0	Pyrimidine nucleotides	True	8.6e+06	1.548	1.548	-0.2913
287	gamma-Glu-Gln	Amino acid	1	AT_Untreated_1_2	AT_Untreated_1	1.0	Gamma-glutamyl dipeptides	True	7.998e+06	1.443	1.443	0.6019
14	UDP-Glucuronate	Carbon	1	AT_Untreated_1_2	AT_Untreated_1	0.666666666666667	Polysaccharide biosynthesis	True	1.396e+06	-1.075	-1.075	0.874
229	N-Formyl-Met	Amino acid	1	AT_Untreated_1_2	AT_Untreated_1	0.666666666666667	N-formylated amino acids	True	1.098e+05	-4.744	-4.744	-0.9221
350	3',5'-cAMP	Nucleotide	1	AT_Untreated_1_2	AT_Untreated_1	0.666666666666667	Purine derivatives in signaling	True	2.103e+05	-3.806	-3.806	-0.1871
371	CDP	Nucleotide	1	AT_Untreated_1_2	AT_Untreated_1	0.666666666666667	Pyrimidine nucleotides	True	6.307e+04	-5.544	-5.544	-2.224
372	CTP	Nucleotide	1	AT_Untreated_1_2	AT_Untreated_1	0.333333333333333	Pyrimidine nucleotides	False			-4.713	-1.059

Metabolite ID	Name	Super Pathway	Dataset	Sample ID	Group ID	Detection Fraction	Pathway	Detected	Raw Intensity	Log2 Norm Intensity	Norm Imputed	Log2 Ctrl Norm
333	GDP	Nucleotide	1	AT_Untreated_1_2	AT_Untreated_1	0.5	Purine nucleotides	True	1.315e+05	-4.484	-4.484	-1.983
332	GMP	Nucleotide	1	AT_Untreated_1_2	AT_Untreated_1	1.0	Purine nucleotides	True	6.208e+06	1.077	1.077	0.3787
373	UMP	Nucleotide	1	AT_Untreated_1_2	AT_Untreated_1	0.833333333333333	Pyrimidine nucleotides	True	1.692e+06	-0.7981	-0.7981	0.257
389	3'-CMP	Nucleotide	1	AT_Untreated_1_2	AT_Untreated_1	1.0	Pyrimidine derivatives in signaling	True	1.173e+06	-1.327	-1.327	-0.2754
330	ADP	Nucleotide	1	AT_Untreated_1_2	AT_Untreated_1	0.5	Purine nucleotides	True	7.021e+05	-2.067	-2.067	-1.771
342	Hypoxanthine	Nucleotide	1	AT_Untreated_1_2	AT_Untreated_1	1.0	Purine bases	True	4.769e+07	4.019	4.019	0.0795
736	Pyridoxamine-P	Cofactor	1	AT_Untreated_1_2	AT_Untreated_1	0.833333333333333	PLP biosynthesis and salvage	True	3.277e+05	-3.166	-3.166	-0.1881
148	Betaine	Amino acid	1	AT_Untreated_1_2	AT_Untreated_1	1.0	Amino acid derivatives	True	1.398e+08	5.57	5.57	0.7643
344	Xanthine	Nucleotide	1	AT_Untreated_1_2	AT_Untreated_1	1.0	Purine bases	True	8.613e+06	1.55	1.55	0.6421
386	3-Ureidopropionate	Nucleotide	1	AT_Untreated_1_2	AT_Untreated_1	1.0	Pyrimidine degradation	True	1.269e+06	-1.213	-1.213	-0.2933
149	DiMe-Gly	Amino acid	1	AT_Untreated_1_2	AT_Untreated_1	1.0	Amino acid derivatives	True	2.991e+06	0.02398	0.02398	0.5443
703	NAD+	Cofactor	1	AT_Untreated_1_2	AT_Untreated_1	1.0	Cofactors	True	2.075e+06	-0.5035	-0.5035	-3.24
709	Pyridoxal-P	Cofactor	1	AT_Untreated_1_2	AT_Untreated_1	0.833333333333333	Cofactors	True	5.516e+05	-2.415	-2.415	-0.1073
731	Thiamin (Vitamin B1)	Cofactor	1	AT_Untreated_1_2	AT_Untreated_1	1.0	TPP biosynthesis	True	6.523e+06	1.149	1.149	0.4011
374	UDP	Nucleotide	1	AT_Untreated_1_2	AT_Untreated_1	0.666666666666667	Pyrimidine nucleotides	True	4.302e+05	-2.774	-2.774	-1.22
102	2-Aminoadipate	Amino acid	1	AT_Untreated_1_2	AT_Untreated_1	0.833333333333333	Amino acids biosynthesis intermediates	True	5.956e+05	-2.304	-2.304	-0.1948
45	Fructose-6-P	Carbon	1	AT_Untreated_1_2	AT_Untreated_1	1.0	Glycolysis, GNG	True	1.261e+06	-1.222	-1.222	-1.297
320	TMP	Nucleotide	1	AT_Untreated_1_2	AT_Untreated_1	0.5	Deoxy-nucleotides	True	2.312e+04	-6.992	-6.992	-2.2
341	XMP	Nucleotide	1	AT_Untreated_1_2	AT_Untreated_1	0.166666666666667	IMP conversion to AMP & GMP	True	7.656e+04	-5.264	-5.264	-0.6898
120	beta-OH-Isovalerate	Amino acid	1	AT_Untreated_1_2	AT_Untreated_1	1.0	Amino acids degradation intermediates	True	6.634e+05	-2.149	-2.149	1.017
322	2'-dI	Nucleotide	1	AT_Untreated_1_2	AT_Untreated_1	0.5	Deoxy-nucleosides	True	8.428e+04	-5.126	-5.126	-1
4	GlcNAc 6-P	Carbon	1	AT_Untreated_1_2	AT_Untreated_1	1.0	Aminosugar biosynthesis	True	7.635e+06	1.376	1.376	1.047
337	Xanthosine	Nucleotide	1	AT_Untreated_1_2	AT_Untreated_1	1.0	Purine nucleosides	True	1.33e+06	-1.146	-1.146	1.929
188	Kynurenine	Amino acid	1	AT_Untreated_1_2	AT_Untreated_1	1.0	Amino acid derivatives	True	4.381e+07	3.896	3.896	4.588

Metabolite ID	Name	Super Pathway	Dataset	Sample ID	Group ID	Detection Fraction	Pathway	Detected	Raw Intensity	Log2 Norm Intensity	Norm Imputed	Log2 Ctrl Norm
63	6-P-Gluconate	Carbon	1	AT_Untreated_1_2	AT_Untreated_1	1.0	Pentose phosphate pathway (PPP)	True	9.765e+06	1.731	1.731	-0.3028
40	Glucuronate	Carbon	1	AT_Untreated_1_2	AT_Untreated_1	1.0	Sugars and sugar alcohols	True	7.497e+05	-1.972	-1.972	-0.4334
108	Argininosuccinate	Amino acid	1	AT_Untreated_1_2	AT_Untreated_1	1.0	Amino acids biosynthesis intermediates	True	2.228e+06	-0.4009	-0.4009	-0.1748
710	Carnitine	Cofactor	1	AT_Untreated_1_2	AT_Untreated_1	1.0	Cofactors	True	1.571e+08	5.739	5.739	-0.2389
725	P-Pantetheine	Cofactor	1	AT_Untreated_1_2	AT_Untreated_1	1.0	Coenzyme A biosynthesis	True	5.161e+05	-2.511	-2.511	1.194
48	DHAP	Carbon	1	AT_Untreated_1_2	AT_Untreated_1	1.0	Glycolysis, GNG	True	1.69e+07	2.522	2.522	-0.5744
17	Maltose	Carbon	1	AT_Untreated_1_2	AT_Untreated_1	1.0	Glycogen degradation	True	5.303e+06	0.8499	0.8499	2.143
359	N1-Me-Adenosine	Nucleotide	1	AT_Untreated_1_2	AT_Untreated_1	0.5	Purine derivatives in RNAs	True	1.176e+06	-1.323	-1.323	-0.2852
159	3-Me-His	Amino acid	1	AT_Untreated_1_2	AT_Untreated_1	1.0	Amino acid derivatives	True	1.958e+05	-3.909	-3.909	0.3261
155	4-Guanidinobutanoate	Amino acid	1	AT_Untreated_1_2	AT_Untreated_1	1.0	Amino acid derivatives	True	1.856e+06	-0.6646	-0.6646	0.7693
164	5-OH-Lys	Amino acid	1	AT_Untreated_1_2	AT_Untreated_1	1.0	Amino acid derivatives	True	1.242e+06	-1.244	-1.244	0.5267
357	Adenosine-3',5'-PP	Nucleotide	1	AT_Untreated_1_2	AT_Untreated_1	0.666666666666667	Purine byproducts of metabolic processes	True	8.639e+04	-5.09	-5.09	-1.612
104	Cystathionine	Amino acid	1	AT_Untreated_1_2	AT_Untreated_1	1.0	Amino acids biosynthesis intermediates	True	7.012e+06	1.253	1.253	-0.9565
113	Imidazole Lactate	Amino acid	1	AT_Untreated_1_2	AT_Untreated_1	0.666666666666667	Amino acids degradation intermediates	True	2.249e+05	-3.71	-3.71	-0.0332
215	N-Ac-Glu	Amino acid	1	AT_Untreated_1_2	AT_Untreated_1	1.0	N-acetylated amino acids	True	4.119e+06	0.4855	0.4855	-1.258
310	S-Lactoyl-Glutathione	Amino acid	1	AT_Untreated_1_2	AT_Untreated_1	1.0	Glutathione derivatives	True	7.399e+06	1.33	1.33	0.9741
5	GlcNAc 1-P	Carbon	1	AT_Untreated_1_2	AT_Untreated_1	0.833333333333333	Aminosugar biosynthesis	True	1.341e+06	-1.134	-1.134	0.7769
34	Ribitol	Carbon	1	AT_Untreated_1_2	AT_Untreated_1	1.0	Sugars and sugar alcohols	True	4.379e+05	-2.748	-2.748	0.6819
10	UDP-Galactose	Carbon	1	AT_Untreated_1_2	AT_Untreated_1	0.833333333333333	Polysaccharide biosynthesis	True	2.154e+06	-0.4501	-0.4501	0.1763
13	Guanosine 5'-PP-Fucose	Carbon	1	AT_Untreated_1_2	AT_Untreated_1	1.0	Polysaccharide biosynthesis	True	1.024e+06	-1.522	-1.522	0.7768

Metabolite ID	Name	Super Pathway	Dataset	Sample ID	Group ID	Detection Fraction	Pathway	Detected	Raw Intensity	Log2 Norm Intensity	Norm Imputed	Log2 Ctrl Norm
19	Maltotetraose	Carbon	1	AT_Untreated_1_2	AT_Untreated_1	1.0	Glycogen degradation	True	2.31e+07	2.973	2.973	3.017
233	SAM	Amino acid	1	AT_Untreated_1_2	AT_Untreated_1	0.333333333333333	SAM metabolism	True	9.759e+05	-1.592	-1.592	-1.436
129	5-Aminovalerate	Amino acid	1	AT_Untreated_1_2	AT_Untreated_1	0.333333333333333	Amino acids degradation intermediates	True	8.133e+06	1.467	1.467	0.4653
741	5-Me-THF	Cofactor	1	AT_Untreated_1_2	AT_Untreated_1	0.333333333333333	Folate metabolism	True	9.961e+04	-4.884	-4.884	-0.1265
198	Indolelactate	Amino acid	1	AT_Untreated_1_2	AT_Untreated_1	1.0	Amino acid derivatives	True	5.469e+05	-2.428	-2.428	1.36
254	Gly-Val	Amino acid	1	AT_Untreated_1_2	AT_Untreated_1	1.0	Dipeptides	True	7.385e+06	1.328	1.328	0.2165
291	gamma-Glu-Leu	Amino acid	1	AT_Untreated_1_2	AT_Untreated_1	1.0	Gamma-glutamyl dipeptides	True	1.122e+06	-1.391	-1.391	-0.9025
173	Met Sulfoxide	Amino acid	1	AT_Untreated_1_2	AT_Untreated_1	1.0	Amino acid derivatives	True	3.066e+07	3.381	3.381	0.8141
43	Glucose	Carbon	1	AT_Untreated_1_2	AT_Untreated_1	1.0	Glycolysis, GNG	True	1.158e+08	5.299	5.299	0.2474
185	Phenyllactate	Amino acid	1	AT_Untreated_1_2	AT_Untreated_1	0.833333333333333	Amino acid derivatives	True	1.26e+06	-1.223	-1.223	4.588
156	Homo-Arg	Amino acid	1	AT_Untreated_1_2	AT_Untreated_1	1.0	Amino acid derivatives	True	7.701e+06	1.388	1.388	0.2078
135	Homocitrulline	Amino acid	1	AT_Untreated_1_2	AT_Untreated_1	1.0	Amino acids degradation intermediates	True	8.918e+05	-1.722	-1.722	-0.01943
719	Nicotinamide MN	Cofactor	1	AT_Untreated_1_2	AT_Untreated_1	1.0	NAD biosynthesis	True	1.038e+06	-1.503	-1.503	-1.689
212	N-Ac-Asp	Amino acid	1	AT_Untreated_1_2	AT_Untreated_1	1.0	N-acetylated amino acids	True	1.781e+06	-0.724	-0.724	-1.421
720	1-Me-Nicotinamide	Cofactor	1	AT_Untreated_1_2	AT_Untreated_1	1.0	Derivatives of NA, nicotinamide and NAD	True	6.239e+08	7.728	7.728	-0.1624
216	N-Ac-Gly	Amino acid	1	AT_Untreated_1_2	AT_Untreated_1	0.5	N-acetylated amino acids	True	3.889e+05	-2.919	-2.919	0.6205
70	Creatine	Carbon	1	AT_Untreated_1_2	AT_Untreated_1	1.0	Creatine energy storage	True	1.474e+09	8.969	8.969	-0.4483
26	Galactonate	Carbon	1	AT_Untreated_1_2	AT_Untreated_1	0.666666666666667	Sugars and sugar alcohols	False			-2.211	-1.406
309	Glutathione, Oxidized	Amino acid	1	AT_Untreated_1_2	AT_Untreated_1	1.0	Glutathione	True	2.805e+07	3.253	3.253	2.061
35	Ribonate	Carbon	1	AT_Untreated_1_2	AT_Untreated_1	0.833333333333333	Sugars and sugar alcohols	True	1.088e+06	-1.435	-1.435	-0.9074
160	1-Me-His	Amino acid	1	AT_Untreated_1_2	AT_Untreated_1	1.0	Amino acid derivatives	True	3.881e+07	3.722	3.722	0.2805

Metabolite ID	Name	Super Pathway	Dataset	Sample ID	Group ID	Detection Fraction	Pathway	Detected	Raw Intensity	Log2 Norm Intensity	Norm Imputed	Log2 Ctrl Norm
44	Glucose 6-P	Carbon	1	AT_Untreated_1_2	AT_Untreated_1	0.833333333333333	Glycolysis, GNG	True	4.757e+05	-2.629	-2.629	-1.152
704	NADH	Cofactor	1	AT_Untreated_1_2	AT_Untreated_1	1.0	Cofactors	True	6.547e+05	-2.168	-2.168	-1.522
275	Thr-Phe	Amino acid	1	AT_Untreated_1_2	AT_Untreated_1	0.833333333333333	Dipeptides	True	9.681e+05	-1.604	-1.604	0.04967
738	Pyridoxate	Cofactor	1	AT_Untreated_1_2	AT_Untreated_1	1.0	PLP biosynthesis and salvage	True	2.535e+05	-3.536	-3.536	-0.1721
177	3-(4-OH-Phenyl)Lactate	Amino acid	1	AT_Untreated_1_2	AT_Untreated_1	1.0	Amino acid derivatives	True	4.281e+06	0.5411	0.5411	3.006
206	Trans-4-OH-Pro	Amino acid	1	AT_Untreated_1_2	AT_Untreated_1	1.0	Amino acid derivatives	True	6.387e+07	4.44	4.44	-0.7149
329	AMP	Nucleotide	1	AT_Untreated_1_2	AT_Untreated_1	1.0	Purine nucleotides	True	2.607e+07	3.148	3.148	-0.4875
11	UDP-Glucose	Carbon	1	AT_Untreated_1_2	AT_Untreated_1	0.833333333333333	Polysaccharide biosynthesis	True	5.095e+06	0.7923	0.7923	0.8975
158	4-Imidazole-Ac	Amino acid	1	AT_Untreated_1_2	AT_Untreated_1	1.0	Amino acid derivatives	True	6.256e+05	-2.233	-2.233	-0.08679
111	1-Me-Imidazole-Ac	Amino acid	1	AT_Untreated_1_2	AT_Untreated_1	1.0	Amino acids degradation intermediates	True	1.22e+06	-1.27	-1.27	0.6638
345	Guanine	Nucleotide	1	AT_Untreated_1_2	AT_Untreated_1	1.0	Purine bases	True	9.493e+07	5.012	5.012	0.0256
22	N-Ac-Neuraminate	Carbon	1	AT_Untreated_1_2	AT_Untreated_1	1.0	Aminosugar derivatives	True	1.692e+06	-0.7983	-0.7983	-0.6629
721	N'-Methylnicotinate	Cofactor	1	AT_Untreated_1_2	AT_Untreated_1	1.0	Derivatives of NA, nicotinamide and NAD	True	1.307e+06	-1.171	-1.171	-0.1633
183	Phenol Sulfate	Amino acid	1	AT_Untreated_1_2	AT_Untreated_1	1.0	Amino acid derivatives	True	7.554e+05	-1.962	-1.962	2.679
718	Nicotinamide Riboside	Cofactor	1	AT_Untreated_1_2	AT_Untreated_1	1.0	NAD biosynthesis	True	1.932e+06	-0.607	-0.607	-2.197
297	gamma-Glu-Thr	Amino acid	1	AT_Untreated_1_2	AT_Untreated_1	1.0	Gamma-glutamyl dipeptides	True	3.895e+06	0.405	0.405	-0.2352
295	gamma-Glu-Phe	Amino acid	1	AT_Untreated_1_2	AT_Untreated_1	0.166666666666667	Gamma-glutamyl dipeptides	True	4.818e+04	-5.932	-5.932	-0.1472
347	Allantoic Acid	Nucleotide	1	AT_Untreated_1_2	AT_Untreated_1	0.666666666666667	Purine degradation	True	8.406e+04	-5.129	-5.129	0.5101
399	Pseudouridine	Nucleotide	1	AT_Untreated_1_2	AT_Untreated_1	1.0	Pyrimidine derivatives in RNAs	True	9.74e+05	-1.595	-1.595	0.4352
375	UTP	Nucleotide	1	AT_Untreated_1_2	AT_Untreated_1	0.666666666666667	Pyrimidine nucleotides	True	9.038e+04	-5.025	-5.025	-3.049
144	Glu, gamma-Me Ester	Amino acid	1	AT_Untreated_1_2	AT_Untreated_1	1.0	Amino acid derivatives	True	4.747e+06	0.6903	0.6903	0.1125

Metabolite ID	Name	Super Pathway	Dataset	Sample ID	Group ID	Detection Fraction	Pathway	Detected	Raw Intensity	Log2 Norm Intensity	Norm Imputed	Log2 Ctrl Norm
292	gamma-Glu-epsilon-Lysine	Amino acid	1	AT_Untreated_1_2	AT_Untreated_1	1.0	Gamma-glutamyl dipeptides	True	4.309e+06	0.5506	0.5506	0.742
225	N-Ac-Thr	Amino acid	1	AT_Untreated_1_2	AT_Untreated_1	1.0	N-acetylated amino acids	True	6.842e+05	-2.104	-2.104	-0.6017
211	N-Ac-Asn	Amino acid	1	AT_Untreated_1_2	AT_Untreated_1	1.0	N-acetylated amino acids	True	3.084e+05	-3.254	-3.254	-0.5686
151	Phenylacetylglycine	Amino acid	1	AT_Untreated_1_2	AT_Untreated_1	1.0	Amino acid derivatives	True	9.88e+05	-1.574	-1.574	-0.7873
217	N-Ac-His	Amino acid	1	AT_Untreated_1_2	AT_Untreated_1	0.833333333333333	N-acetylated amino acids	True	2.365e+05	-3.637	-3.637	-0.1519
288	gamma-Glu-Gly	Amino acid	1	AT_Untreated_1_2	AT_Untreated_1	0.333333333333333	Gamma-glutamyl dipeptides	True	3.449e+05	-3.092	-3.092	-1.268
222	N-Ac-Phe	Amino acid	1	AT_Untreated_1_2	AT_Untreated_1	0.333333333333333	N-acetylated amino acids	True	3.664e+04	-6.327	-6.327	-0.05478
71	Creatine-P	Carbon	1	AT_Untreated_1_2	AT_Untreated_1	1.0	Creatine energy storage	True	1.449e+05	-4.344	-4.344	-0.0888
210	N-Ac-Arg	Amino acid	1	AT_Untreated_1_2	AT_Untreated_1	0.666666666666667	N-acetylated amino acids	True	6.142e+05	-2.26	-2.26	0.4155
218	N-Ac-Ile	Amino acid	1	AT_Untreated_1_2	AT_Untreated_1	0.166666666666667	N-acetylated amino acids	True	4.403e+04	-6.062	-6.062	-0.09626
251	Gly-Leu	Amino acid	1	AT_Untreated_1_2	AT_Untreated_1	1.0	Dipeptides	True	1.46e+07	2.311	2.311	0.6725
290	gamma-Glu-Ile	Amino acid	1	AT_Untreated_1_2	AT_Untreated_1	1.0	Gamma-glutamyl dipeptides	True	6.212e+05	-2.244	-2.244	-0.9051
316	Ophthalmate	Amino acid	1	AT_Untreated_1_2	AT_Untreated_1	1.0	Oxidative stress markers	True	6.61e+06	1.168	1.168	-1.175
125	Isovaleryl-Gly	Amino acid	1	AT_Untreated_1_2	AT_Untreated_1	1.0	Amino acids degradation intermediates	True	1.01e+05	-4.865	-4.865	0.5445
368	7-Me-Guanine	Nucleotide	1	AT_Untreated_1_2	AT_Untreated_1	1.0	Purine derivatives in RNAs	True	5.266e+05	-2.482	-2.482	-0.2919
208	Pro-OH-Pro	Amino acid	1	AT_Untreated_1_2	AT_Untreated_1	1.0	Amino acid derivatives	True	3.334e+07	3.503	3.503	0.1733
366	N2,N2-DiMe-Guanosine	Nucleotide	1	AT_Untreated_1_2	AT_Untreated_1	0.833333333333333	Purine derivatives in RNAs	True	2.032e+05	-3.856	-3.856	0.2577
352	3'-AMP	Nucleotide	1	AT_Untreated_1_2	AT_Untreated_1	1.0	Purine derivatives in signaling	True	9.277e+05	-1.665	-1.665	-0.1782
363	N6-Carbamoyl-Thr-Adenosine	Nucleotide	1	AT_Untreated_1_2	AT_Untreated_1	0.666666666666667	Purine derivatives in RNAs	True	1.298e+05	-4.502	-4.502	0.1831
314	Cys-Glutathione Disulfide	Amino acid	1	AT_Untreated_1_2	AT_Untreated_1	1.0	Oxidative stress markers	True	4.125e+05	-2.834	-2.834	0.4497
382	Orotidine	Nucleotide	1	AT_Untreated_1_2	AT_Untreated_1	0.166666666666667	Pyrimidine (UMP) biosynthesis	False			-5.287	-0.9952

Metabolite ID	Name	Super Pathway	Dataset	Sample ID	Group ID	Detection Fraction	Pathway	Detected	Raw Intensity	Log2 Norm Intensity	Norm Imputed	Log2 Ctrl Norm
307	Cys-Gly	Amino acid	1	AT_Untreated_1_2	AT_Untreated_1	1.0	Glutathione biosynthesis	True	1.094e+07	1.895	1.895	0.2875
64	Sedoheptulose-7-P	Carbon	1	AT_Untreated_1_2	AT_Untreated_1	1.0	Pentose phosphate pathway (PPP)	True	2.976e+06	0.0165	0.0165	-0.1376
142	N-Ac-Asp-Glu	Amino acid	1	AT_Untreated_1_2	AT_Untreated_1	1.0	Amino acid derivatives	True	4.854e+05	-2.6	-2.6	-1.454
708	Thiamin-PP	Cofactor	1	AT_Untreated_1_2	AT_Untreated_1	0.5	Cofactors	True	4.713e+04	-5.964	-5.964	0.01955
182	P-Cresol Sulfate	Amino acid	1	AT_Untreated_1_2	AT_Untreated_1	1.0	Amino acid derivatives	True	6.479e+05	-2.183	-2.183	1.013
250	Gly-Ile	Amino acid	1	AT_Untreated_1_2	AT_Untreated_1	1.0	Dipeptides	True	1.807e+06	-0.7034	-0.7034	0.6138
286	gamma-Glu-Glu	Amino acid	1	AT_Untreated_1_2	AT_Untreated_1	1.0	Gamma-glutamyl dipeptides	True	3.146e+06	0.09665	0.09665	0.07883
739	Deoxycarnitine	Cofactor	1	AT_Untreated_1_2	AT_Untreated_1	1.0	Carnitine biosynthesis	True	2.499e+07	3.086	3.086	-0.665
203	DiMe-Arg	Amino acid	1	AT_Untreated_1_2	AT_Untreated_1	1.0	Amino acid derivatives	True	1.169e+08	5.313	5.313	-0.3201
351	2'-AMP	Nucleotide	1	AT_Untreated_1_2	AT_Untreated_1	1.0	Purine derivatives in signaling	True	3.492e+05	-3.075	-3.075	-0.8063
8	Cytidine 5'-P-N-Ac-Neuraminate	Carbon	1	AT_Untreated_1_2	AT_Untreated_1	1.0	Aminosugar biosynthesis	True	4.879e+05	-2.592	-2.592	-0.8466
285	gamma-Glu-Ala	Amino acid	1	AT_Untreated_1_2	AT_Untreated_1	0.5	Gamma-glutamyl dipeptides	True	4.39e+05	-2.745	-2.745	0.4943
224	N-Ac-Ser	Amino acid	1	AT_Untreated_1_2	AT_Untreated_1	1.0	N-acetylated amino acids	True	5.314e+06	0.8531	0.8531	-1.026
244	Ala-Leu	Amino acid	1	AT_Untreated_1_2	AT_Untreated_1	1.0	Dipeptides	True	1.503e+07	2.353	2.353	1.215
207	N-Me-Pro	Amino acid	1	AT_Untreated_1_2	AT_Untreated_1	1.0	Amino acid derivatives	True	2.3e+06	-0.3552	-0.3552	0.5748
171	Cys Sulfinic Acid	Amino acid	1	AT_Untreated_1_2	AT_Untreated_1	0.333333333333333	Amino acid derivatives	True	2.272e+05	-3.695	-3.695	-0.3976
181	O-Me-Tyr	Amino acid	1	AT_Untreated_1_2	AT_Untreated_1	0.5	Amino acid derivatives	True	1.832e+05	-4.005	-4.005	-0.6354
240	N-Ac-Putrescine	Amino acid	1	AT_Untreated_1_2	AT_Untreated_1	0.833333333333333	Polyamine derivatives	True	3.096e+06	0.07342	0.07342	2.437
176	S-Me-Met	Amino acid	1	AT_Untreated_1_2	AT_Untreated_1	1.0	Amino acid derivatives	True	1.089e+06	-1.434	-1.434	0.4073
339	AICAR	Nucleotide	1	AT_Untreated_1_2	AT_Untreated_1	0.333333333333333	IMP biosynthesis	True	1.317e+05	-4.481	-4.481	-0.1802
141	gamma-Carboxy-Glu	Amino acid	1	AT_Untreated_1_2	AT_Untreated_1	1.0	Amino acid derivatives	True	1.535e+06	-0.9383	-0.9383	-0.1532
392	3'-UMP	Nucleotide	1	AT_Untreated_1_2	AT_Untreated_1	0.833333333333333	Pyrimidine derivatives in signaling	True	4.41e+05	-2.738	-2.738	1.295

Metabolite ID	Name	Super Pathway	Dataset	Sample ID	Group ID	Detection Fraction	Pathway	Detected	Raw Intensity	Log2 Norm Intensity	Norm Imputed	Log2 Ctrl Norm
355	3'-GMP	Nucleotide	1	AT_Untreated_1_2	AT_Untreated_1	0.833333333333333	Purine derivatives in signaling	True	1.75e+05	-4.072	-4.072	-0.1343
282	Val-Leu	Amino acid	1	AT_Untreated_1_2	AT_Untreated_1	1.0	Dipeptides	True	7.53e+06	1.356	1.356	0.3842
140	Carboxyethyl-GABA	Amino acid	1	AT_Untreated_1_2	AT_Untreated_1	1.0	Amino acid derivatives	True	7.314e+05	-2.008	-2.008	-0.5769
258	Ile-Gly	Amino acid	1	AT_Untreated_1_2	AT_Untreated_1	1.0	Dipeptides	True	3.965e+07	3.752	3.752	0.4404
260	Leu-Ala	Amino acid	1	AT_Untreated_1_2	AT_Untreated_1	1.0	Dipeptides	True	8.664e+06	1.558	1.558	0.2569
265	Lys-Leu	Amino acid	1	AT_Untreated_1_2	AT_Untreated_1	0.833333333333333	Dipeptides	True	4.342e+05	-2.76	-2.76	-0.1492
263	Leu-Gly	Amino acid	1	AT_Untreated_1_2	AT_Untreated_1	1.0	Dipeptides	True	7.454e+07	4.663	4.663	1.642
281	Val-Gly	Amino acid	1	AT_Untreated_1_2	AT_Untreated_1	1.0	Dipeptides	True	8.599e+07	4.869	4.869	0.35
270	Pro-Gly	Amino acid	1	AT_Untreated_1_2	AT_Untreated_1	1.0	Dipeptides	True	3.95e+06	0.4249	0.4249	-0.3218
114	Imidazole Propionate	Amino acid	1	AT_Untreated_1_2	AT_Untreated_1	1.0	Amino acids degradation intermediates	True	6.307e+05	-2.222	-2.222	0.08882
267	Phe-Gly	Amino acid	1	AT_Untreated_1_2	AT_Untreated_1	1.0	Dipeptides	True	7.327e+07	4.638	4.638	0.5444
266	Phe-Ala	Amino acid	1	AT_Untreated_1_2	AT_Untreated_1	1.0	Dipeptides	True	9.41e+06	1.677	1.677	0.6006
278	Tyr-Gly	Amino acid	1	AT_Untreated_1_2	AT_Untreated_1	1.0	Dipeptides	True	1.678e+07	2.512	2.512	0.3297
255	His-Ala	Amino acid	1	AT_Untreated_1_2	AT_Untreated_1	0.833333333333333	Dipeptides	True	1.902e+06	-0.6297	-0.6297	0.7077
280	Val-Gln	Amino acid	1	AT_Untreated_1_2	AT_Untreated_1	1.0	Dipeptides	True	1.57e+07	2.416	2.416	0.7977
143	S-1-Pyrroline-5-Carboxylate	Amino acid	1	AT_Untreated_1_2	AT_Untreated_1	0.833333333333333	Amino acid derivatives	True	5.935e+05	-2.309	-2.309	-0.2414
232	SAH	Amino acid	1	AT_Untreated_1_2	AT_Untreated_1	1.0	SAM metabolism	True	1.733e+06	-0.7635	-0.7635	-0.2781
20	Erythronate	Carbon	1	AT_Untreated_1_2	AT_Untreated_1	1.0	Aminosugar derivatives	True	4.146e+07	3.817	3.817	0.07546
248	Gln-Leu	Amino acid	1	AT_Untreated_1_2	AT_Untreated_1	0.833333333333333	Dipeptides	True	1.814e+06	-0.6973	-0.6973	0.2079
276	Trp-Gly	Amino acid	1	AT_Untreated_1_2	AT_Untreated_1	0.833333333333333	Dipeptides	True	1.447e+06	-1.023	-1.023	0.05493
205	N-delta-Ac-Ornithine	Amino acid	1	AT_Untreated_1_2	AT_Untreated_1	1.0	Amino acid derivatives	True	1.256e+06	-1.228	-1.228	-0.1074
163	Formimino-Glu	Amino acid	1	AT_Untreated_1_2	AT_Untreated_1	1.0	Amino acid derivatives	True	1.857e+06	-0.6639	-0.6639	0.5759
204	N-Me-Arg	Amino acid	1	AT_Untreated_1_2	AT_Untreated_1	0.833333333333333	Amino acid derivatives	True	2.041e+07	2.794	2.794	0.2072
242	Guanidino-Ac	Amino acid	1	AT_Untreated_1_2	AT_Untreated_1	1.0	Creatine biosynthesis	True	5.168e+05	-2.509	-2.509	1.371

Metabolite ID	Name	Super Pathway	Dataset	Sample ID	Group ID	Detection Fraction	Pathway	Detected	Raw Intensity	Log2 Norm Intensity	Norm Imputed	Log2 Ctrl Norm
300	gamma-Glu-Val	Amino acid	1	AT_Untreated_1_2	AT_Untreated_1	0.333333333333333	Gamma-glutamyl dipeptides	False			-0.1053	-0.726
53	Ac-CoA	Carbon	1	AT_Untreated_1_2	AT_Untreated_1	0.166666666666667	Acetyl-CoA	False			-7.274	-0.5114
18	Maltotriose	Carbon	1	AT_Untreated_1_2	AT_Untreated_1	1.0	Glycogen degradation	True	2.193e+07	2.898	2.898	3.056
294	gamma-Glu-Met	Amino acid	1	AT_Untreated_1_2	AT_Untreated_1	0.333333333333333	Gamma-glutamyl dipeptides	True	4.149e+05	-2.826	-2.826	0.656
174	Met Sulfone	Amino acid	1	AT_Untreated_1_2	AT_Untreated_1	0.833333333333333	Amino acid derivatives	True	3.231e+05	-3.187	-3.187	-0.108
175	N-Ac-Met Sulfoxide	Amino acid	1	AT_Untreated_1_2	AT_Untreated_1	1.0	Amino acid derivatives	True	3.123e+06	0.08591	0.08591	0.1096
25	Mannitol/Sorbitol	Carbon	1	AT_Untreated_1_2	AT_Untreated_1	1.0	Sugars and sugar alcohols	True	1.853e+07	2.655	2.655	0.4303
6	UDP-GlcNAc	Carbon	1	AT_Untreated_1_2	AT_Untreated_1	0.166666666666667	Aminosugar biosynthesis	False			-3.589	-0.4814
145	Pyro-Gln	Amino acid	1	AT_Untreated_1_2	AT_Untreated_1	1.0	Amino acid derivatives	True	4.491e+06	0.6104	0.6104	-0.2294
705	Coenzyme A	Cofactor	1	AT_Untreated_1_2	AT_Untreated_1	0.666666666666667	Cofactors	True	1.233e+05	-4.576	-4.576	-0.617
319	2'-dAMP	Nucleotide	1	AT_Untreated_1_2	AT_Untreated_1	0.166666666666667	Deoxy-nucleotides	False			-4.337	-0.6568
119	alpha-OH-Isovalerate	Amino acid	1	AT_Untreated_1_2	AT_Untreated_1	1.0	Amino acids degradation intermediates	True	5.766e+05	-2.351	-2.351	0.9472
46	Fructose 1,6-PP / Glucose 1,6-PP / Inositol-1,4-PP / Inositol-1,3-PP	Carbon	1	AT_Untreated_1_2	AT_Untreated_1	1.0	Glycolysis, GNG	True	3.649e+07	3.633	3.633	-1.198
137	1-Me-Guanidine	Amino acid	1	AT_Untreated_1_2	AT_Untreated_1	0.666666666666667	Amino acids degradation intermediates	True	2.224e+05	-3.726	-3.726	1.427
23	N-GlcNAc-Asn	Carbon	1	AT_Untreated_1_2	AT_Untreated_1	1.0	Aminosugar derivatives	True	4.928e+06	0.7441	0.7441	0.03116
262	Leu-Gln	Amino acid	1	AT_Untreated_1_2	AT_Untreated_1	1.0	Dipeptides	True	1.038e+07	1.818	1.818	0.522
24	Fructose	Carbon	1	AT_Untreated_1_2	AT_Untreated_1	1.0	Sugars and sugar alcohols	True	1.588e+07	2.433	2.433	0.4839
197	C-Glycosyl-Trp	Amino acid	1	AT_Untreated_1_2	AT_Untreated_1	1.0	Amino acid derivatives	True	5.243e+06	0.8337	0.8337	1.133
33	Arabitol/Xylitol	Carbon	1	AT_Untreated_1_2	AT_Untreated_1	1.0	Sugars and sugar alcohols	True	5.136e+05	-2.518	-2.518	-0.6581

Metabolite ID	Name	Super Pathway	Dataset	Sample ID	Group ID	Detection Fraction	Pathway	Detected	Raw Intensity	Log2 Norm Intensity	Norm Imputed	Log2 Ctrl Norm
128	N2-Ac-Lys/N6-Ac-Lys	Amino acid	1	AT_Untreated_1_2	AT_Untreated_1	1.0	Amino acids degradation intermediates	True	3.433e+06	0.2226	0.2226	-0.9878
42	2-Me-Citrate	Carbon	1	AT_Untreated_1_2	AT_Untreated_1	0.833333333333333	Propionate metabolism	True	1.553e+05	-4.243	-4.243	0.1397
12	Glucuronate 1-P	Carbon	1	AT_Untreated_1_2	AT_Untreated_1	1.0	Polysaccharide biosynthesis	True	1.037e+06	-1.504	-1.504	-0.261
76	Gln	Amino acid	1	AT_Untreated_1_3	AT_Untreated_1	1.0	Proteinogenic amino acids	True	5.768e+08	7.519	7.519	-1.162
89	Trp	Amino acid	1	AT_Untreated_1_3	AT_Untreated_1	1.0	Proteinogenic amino acids	True	2.336e+07	2.893	2.893	-2.782
723	beta-Ala	Cofactor	1	AT_Untreated_1_3	AT_Untreated_1	1.0	Coenzyme A biosynthesis	True	1.616e+07	2.362	2.362	-0.6478
75	Glu	Amino acid	1	AT_Untreated_1_3	AT_Untreated_1	1.0	Proteinogenic amino acids	True	3.065e+09	9.929	9.929	-0.8078
77	Gly	Amino acid	1	AT_Untreated_1_3	AT_Untreated_1	1.0	Proteinogenic amino acids	True	9.897e+07	4.976	4.976	-0.6393
80	His	Amino acid	1	AT_Untreated_1_3	AT_Untreated_1	1.0	Proteinogenic amino acids	True	1.976e+07	2.651	2.651	-0.764
82	Leu	Amino acid	1	AT_Untreated_1_3	AT_Untreated_1	1.0	Proteinogenic amino acids	True	1.539e+09	8.935	8.935	-0.2866
87	Phe	Amino acid	1	AT_Untreated_1_3	AT_Untreated_1	1.0	Proteinogenic amino acids	True	1.125e+09	8.483	8.483	-0.3281
130	Glutarate	Amino acid	1	AT_Untreated_1_3	AT_Untreated_1	1.0	Amino acids degradation intermediates	True	5.834e+05	-2.43	-2.43	-3.76e-03
196	5-OH-Indole-Ac	Amino acid	1	AT_Untreated_1_3	AT_Untreated_1	1.0	Amino acid derivatives	True	1.609e+05	-4.289	-4.289	-0.2773
74	Asp	Amino acid	1	AT_Untreated_1_3	AT_Untreated_1	1.0	Proteinogenic amino acids	True	5.126e+08	7.349	7.349	-0.5429
236	Spermidine	Amino acid	1	AT_Untreated_1_3	AT_Untreated_1	1.0	Polyamines	True	4.306e+07	3.775	3.775	-0.08625
73	Asn	Amino acid	1	AT_Untreated_1_3	AT_Untreated_1	1.0	Proteinogenic amino acids	True	2.348e+08	6.222	6.222	-0.4488
243	Creatinine	Amino acid	1	AT_Untreated_1_3	AT_Untreated_1	1.0	Creatine degradation	True	9.442e+07	4.908	4.908	-0.09409
376	Cytidine	Nucleotide	1	AT_Untreated_1_3	AT_Untreated_1	0.833333333333333	Pyrimidine nucleosides	True	1.307e+06	-1.266	-1.266	-1.994
41	Lactate	Carbon	1	AT_Untreated_1_3	AT_Untreated_1	1.0	Respiratory carbon sources	True	2.569e+08	6.352	6.352	-0.1297
58	alpha-Ketoglutarate	Carbon	1	AT_Untreated_1_3	AT_Untreated_1	1.0	TCA cycle	True	2.899e+06	-0.1175	-0.1175	-0.1342
69	3-OH-Butyrate	Carbon	1	AT_Untreated_1_3	AT_Untreated_1	0.833333333333333	Ketone bodies	True	1.767e+06	-0.8313	-0.8313	2.153

Metabolite ID	Name	Super Pathway	Dataset	Sample ID	Group ID	Detection Fraction	Pathway	Detected	Raw Intensity	Log2 Norm Intensity	Norm Imputed	Log2 Ctrl Norm
343	Adenine	Nucleotide	1	AT_Untreated_1_3	AT_Untreated_1	1.0	Purine bases	True	4.312e+06	0.4556	0.4556	-1.001
336	Adenosine	Nucleotide	1	AT_Untreated_1_3	AT_Untreated_1	1.0	Purine nucleosides	True	1.623e+07	2.367	2.367	-1.056
722	ADP-Ribose	Cofactor	1	AT_Untreated_1_3	AT_Untreated_1	1.0	Derivatives of NA, nicotinamide and NAD	True	3.705e+06	0.2365	0.2365	3.239
383	Cytosine	Nucleotide	1	AT_Untreated_1_3	AT_Untreated_1	0.833333333333333	Pyrimidine bases	True	4.515e+05	-2.8	-2.8	2.055
3	Glucosamine 6-P	Carbon	1	AT_Untreated_1_3	AT_Untreated_1	0.833333333333333	Aminosugar biosynthesis	True	1.597e+06	-0.9779	-0.9779	0.6894
717	Nicotinamide	Cofactor	1	AT_Untreated_1_3	AT_Untreated_1	1.0	NAD biosynthesis	True	1.262e+08	5.326	5.326	0.8112
51	PEP	Carbon	1	AT_Untreated_1_3	AT_Untreated_1	1.0	Glycolysis, GNG	True	7.908e+06	1.33	1.33	0.2793
237	Spermine	Amino acid	1	AT_Untreated_1_3	AT_Untreated_1	1.0	Polyamines	True	5.443e+05	-2.53	-2.53	-1.04
385	Uracil	Nucleotide	1	AT_Untreated_1_3	AT_Untreated_1	1.0	Pyrimidine bases	True	9.02e+06	1.52	1.52	1.255
377	Uridine	Nucleotide	1	AT_Untreated_1_3	AT_Untreated_1	1.0	Pyrimidine nucleosides	True	3.61e+07	3.521	3.521	-0.5281
348	Allantoin	Nucleotide	1	AT_Untreated_1_3	AT_Untreated_1	1.0	Purine degradation	True	2.892e+06	-0.1207	-0.1207	0.05492
335	Inosine	Nucleotide	1	AT_Untreated_1_3	AT_Untreated_1	1.0	Purine nucleosides	True	1.174e+08	5.223	5.223	-0.2763
81	Ile	Amino acid	1	AT_Untreated_1_3	AT_Untreated_1	1.0	Proteinogenic amino acids	True	1.044e+09	8.375	8.375	-0.3398
72	Ala	Amino acid	1	AT_Untreated_1_3	AT_Untreated_1	1.0	Proteinogenic amino acids	True	5.617e+08	7.481	7.481	-1.021
79	Thr	Amino acid	1	AT_Untreated_1_3	AT_Untreated_1	1.0	Proteinogenic amino acids	True	4.831e+08	7.263	7.263	-0.7464
88	Tyr	Amino acid	1	AT_Untreated_1_3	AT_Untreated_1	1.0	Proteinogenic amino acids	True	4.739e+08	7.236	7.236	-0.288
84	Lys	Amino acid	1	AT_Untreated_1_3	AT_Untreated_1	1.0	Proteinogenic amino acids	True	6.045e+08	7.587	7.587	-0.4033
86	Met	Amino acid	1	AT_Untreated_1_3	AT_Untreated_1	1.0	Proteinogenic amino acids	True	7.682e+08	7.932	7.932	-0.2952
61	Malate	Carbon	1	AT_Untreated_1_3	AT_Untreated_1	1.0	TCA cycle	True	2.689e+08	6.418	6.418	-0.7535
235	Putrescine	Amino acid	1	AT_Untreated_1_3	AT_Untreated_1	0.833333333333333	Polyamines	True	4.8e+06	0.6102	0.6102	1.101
324	2'-dU	Nucleotide	1	AT_Untreated_1_3	AT_Untreated_1	0.666666666666667	Deoxy-nucleosides	False			-5.281	-0.9009
49	3-P-Glycerate	Carbon	1	AT_Untreated_1_3	AT_Untreated_1	1.0	Glycolysis, GNG	True	6.104e+07	4.279	4.279	1.013
189	Kynurenate	Amino acid	1	AT_Untreated_1_3	AT_Untreated_1	1.0	Amino acid derivatives	True	1.072e+05	-4.875	-4.875	0.9232
234	5-Me-Thioadenosine	Amino acid	1	AT_Untreated_1_3	AT_Untreated_1	1.0	SAM metabolism	True	3.923e+06	0.3191	0.3191	-1.566

Metabolite ID	Name	Super Pathway	Dataset	Sample ID	Group ID	Detection Fraction	Pathway	Detected	Raw Intensity	Log2 Norm Intensity	Norm Imputed	Log2 Ctrl Norm
59	Succinate	Carbon	1	AT_Untreated_1_3	AT_Untreated_1	1.0	TCA cycle	True	6.215e+06	0.9828	0.9828	0.6818
36	Ribose	Carbon	1	AT_Untreated_1_3	AT_Untreated_1	1.0	Sugars and sugar alcohols	True	2.83e+06	-0.152	-0.152	1.082
133	Ornithine	Amino acid	1	AT_Untreated_1_3	AT_Untreated_1	1.0	Amino acids degradation intermediates	True	3.798e+07	3.594	3.594	-0.8854
313	5-Oxoproline	Amino acid	1	AT_Untreated_1_3	AT_Untreated_1	1.0	Glutathione derivatives	True	9.416e+06	1.582	1.582	-0.5053
165	N-6-Tri-Me-Lys	Amino acid	1	AT_Untreated_1_3	AT_Untreated_1	1.0	Amino acid derivatives	True	4.017e+07	3.675	3.675	-0.08839
380	Orotate	Nucleotide	1	AT_Untreated_1_3	AT_Untreated_1	0.666666666666667	Pyrimidine (UMP) biosynthesis	True	2.13e+05	-3.884	-3.884	-0.2541
724	Pantothenate	Cofactor	1	AT_Untreated_1_3	AT_Untreated_1	1.0	Coenzyme A biosynthesis	True	1.831e+08	5.863	5.863	-0.4467
150	N-Me-Gly	Amino acid	1	AT_Untreated_1_3	AT_Untreated_1	1.0	Amino acid derivatives	True	2.363e+06	-0.4121	-0.4121	-0.1117
122	3-OH-Isobutyrate	Amino acid	1	AT_Untreated_1_3	AT_Untreated_1	0.833333333333333	Amino acids degradation intermediates	True	5.267e+05	-2.578	-2.578	0.578
241	4-Acetamidobutanoate	Amino acid	1	AT_Untreated_1_3	AT_Untreated_1	1.0	Polyamine derivatives	True	4.96e+06	0.6573	0.6573	0.2163
711	alpha-Tocopherol	Cofactor	1	AT_Untreated_1_3	AT_Untreated_1	1.0	Cofactors	True	3.951e+06	0.3292	0.3292	-0.4512
55	Citrate	Carbon	1	AT_Untreated_1_3	AT_Untreated_1	1.0	TCA cycle	True	1.205e+07	1.938	1.938	-0.01101
387	3-Aminoisobutyrate	Nucleotide	1	AT_Untreated_1_3	AT_Untreated_1	0.833333333333333	Pyrimidine degradation	True	5.194e+05	-2.598	-2.598	-0.5842
338	Guanosine	Nucleotide	1	AT_Untreated_1_3	AT_Untreated_1	1.0	Purine nucleosides	True	1.073e+08	5.092	5.092	-0.01392
209	N-Ac-Ala	Amino acid	1	AT_Untreated_1_3	AT_Untreated_1	1.0	N-acetylated amino acids	True	1.313e+06	-1.26	-1.26	-0.05843
221	N-Ac-Met	Amino acid	1	AT_Untreated_1_3	AT_Untreated_1	1.0	N-acetylated amino acids	True	9.981e+06	1.666	1.666	-0.07793
228	N-Ac-Val	Amino acid	1	AT_Untreated_1_3	AT_Untreated_1	0.333333333333333	N-acetylated amino acids	True	1.083e+05	-4.859	-4.859	0.5114
346	Urate	Nucleotide	1	AT_Untreated_1_3	AT_Untreated_1	1.0	Purine degradation	True	3.366e+06	0.09796	0.09796	1.161
90	Arg	Amino acid	1	AT_Untreated_1_3	AT_Untreated_1	1.0	Proteinogenic amino acids	True	2.177e+09	9.435	9.435	-0.2702
60	Fumarate	Carbon	1	AT_Untreated_1_3	AT_Untreated_1	1.0	TCA cycle	True	4.071e+06	0.3724	0.3724	-0.6428
78	Ser	Amino acid	1	AT_Untreated_1_3	AT_Untreated_1	1.0	Proteinogenic amino acids	True	3.73e+08	6.89	6.89	-0.3672
83	Val	Amino acid	1	AT_Untreated_1_3	AT_Untreated_1	1.0	Proteinogenic amino acids	True	5.538e+08	7.46	7.46	-0.4119

Metabolite ID	Name	Super Pathway	Dataset	Sample ID	Group ID	Detection Fraction	Pathway	Detected	Raw Intensity	Log2 Norm Intensity	Norm Imputed	Log2 Ctrl Norm
734	Pyridoxal	Cofactor	1	AT_Untreated_1_3	AT_Untreated_1	1.0	PLP biosynthesis and salvage	True	1.198e+07	1.93	1.93	0.147
136	Urea	Amino acid	1	AT_Untreated_1_3	AT_Untreated_1	1.0	Amino acids degradation intermediates	True	3.291e+06	0.06544	0.06544	-0.08578
67	Ribose 1-P	Carbon	1	AT_Untreated_1_3	AT_Untreated_1	1.0	Pentose phosphate pathway (PPP)	True	9.424e+06	1.583	1.583	0.9622
284	Carnosine	Amino acid	1	AT_Untreated_1_3	AT_Untreated_1	1.0	Dipeptides	True	6.533e+05	-2.267	-2.267	-0.1589
306	gamma-Glu-Cys	Amino acid	1	AT_Untreated_1_3	AT_Untreated_1	1.0	Glutathione biosynthesis	True	1.76e+06	-0.8377	-0.8377	-0.5009
712	Retinol (Vit A)	Cofactor	1	AT_Untreated_1_3	AT_Untreated_1	0.833333333333333	Cofactors	True	7.572e+05	-2.054	-2.054	0.379
85	Cys	Amino acid	1	AT_Untreated_1_3	AT_Untreated_1	1.0	Proteinogenic amino acids	True	6.595e+07	4.39	4.39	-0.1234
91	Pro	Amino acid	1	AT_Untreated_1_3	AT_Untreated_1	1.0	Proteinogenic amino acids	True	1.475e+09	8.873	8.873	-0.5347
308	Glutathione, Reduced	Amino acid	1	AT_Untreated_1_3	AT_Untreated_1	1.0	Glutathione	True	4.765e+08	7.243	7.243	0.2873
107	Citrulline	Amino acid	1	AT_Untreated_1_3	AT_Untreated_1	1.0	Amino acids biosynthesis intermediates	True	1.744e+07	2.471	2.471	-0.3533
328	IMP	Nucleotide	1	AT_Untreated_1_3	AT_Untreated_1	0.333333333333333	Purine nucleotides	True	1.498e+05	-4.392	-4.392	-0.0702
706	FAD	Cofactor	1	AT_Untreated_1_3	AT_Untreated_1	1.0	Cofactors	True	1.133e+06	-1.473	-1.473	0.7167
735	Pyridoxamine	Cofactor	1	AT_Untreated_1_3	AT_Untreated_1	1.0	PLP biosynthesis and salvage	True	4.009e+06	0.3502	0.3502	0.5432
199	Serotonin	Amino acid	1	AT_Untreated_1_3	AT_Untreated_1	1.0	Amino acid derivatives	True	4.597e+06	0.5477	0.5477	-0.725
370	CMP	Nucleotide	1	AT_Untreated_1_3	AT_Untreated_1	1.0	Pyrimidine nucleotides	True	1.375e+07	2.128	2.128	0.2895
287	gamma-Glu-Gln	Amino acid	1	AT_Untreated_1_3	AT_Untreated_1	1.0	Gamma-glutamyl dipeptides	True	2.297e+06	-0.4531	-0.4531	-1.294
14	UDP-Glucuronate	Carbon	1	AT_Untreated_1_3	AT_Untreated_1	0.666666666666667	Polysaccharide biosynthesis	True	3.892e+06	0.3076	0.3076	2.257
229	N-Formyl-Met	Amino acid	1	AT_Untreated_1_3	AT_Untreated_1	0.666666666666667	N-formylated amino acids	True	1.288e+05	-4.61	-4.61	-0.7877
350	3',5'-cAMP	Nucleotide	1	AT_Untreated_1_3	AT_Untreated_1	0.666666666666667	Purine derivatives in signaling	True	2.237e+05	-3.813	-3.813	-0.1939
371	CDP	Nucleotide	1	AT_Untreated_1_3	AT_Untreated_1	0.666666666666667	Pyrimidine nucleotides	True	4.381e+05	-2.844	-2.844	0.4763
372	CTP	Nucleotide	1	AT_Untreated_1_3	AT_Untreated_1	0.333333333333333	Pyrimidine nucleotides	False			-4.713	-1.059

Metabolite ID	Name	Super Pathway	Dataset	Sample ID	Group ID	Detection Fraction	Pathway	Detected	Raw Intensity	Log2 Norm Intensity	Norm Imputed	Log2 Ctrl Norm
333	GDP	Nucleotide	1	AT_Untreated_1_3	AT_Untreated_1	0.5	Purine nucleotides	True	1.64e+05	-4.261	-4.261	-1.761
332	GMP	Nucleotide	1	AT_Untreated_1_3	AT_Untreated_1	1.0	Purine nucleotides	True	1.462e+07	2.217	2.217	1.518
373	UMP	Nucleotide	1	AT_Untreated_1_3	AT_Untreated_1	0.833333333333333	Pyrimidine nucleotides	True	1.134e+07	1.851	1.851	2.906
389	3'-CMP	Nucleotide	1	AT_Untreated_1_3	AT_Untreated_1	1.0	Pyrimidine derivatives in signaling	True	1.32e+06	-1.252	-1.252	-0.2009
330	ADP	Nucleotide	1	AT_Untreated_1_3	AT_Untreated_1	0.5	Purine nucleotides	True	4.693e+06	0.5777	0.5777	0.8741
342	Hypoxanthine	Nucleotide	1	AT_Untreated_1_3	AT_Untreated_1	1.0	Purine bases	True	3.597e+07	3.516	3.516	-0.4235
736	Pyridoxamine-P	Cofactor	1	AT_Untreated_1_3	AT_Untreated_1	0.833333333333333	PLP biosynthesis and salvage	True	5.319e+05	-2.564	-2.564	0.4144
148	Betaine	Amino acid	1	AT_Untreated_1_3	AT_Untreated_1	1.0	Amino acid derivatives	True	1.182e+08	5.232	5.232	0.4262
344	Xanthine	Nucleotide	1	AT_Untreated_1_3	AT_Untreated_1	1.0	Purine bases	True	2.071e+07	2.72	2.72	1.812
386	3-Ureidopropionate	Nucleotide	1	AT_Untreated_1_3	AT_Untreated_1	1.0	Pyrimidine degradation	True	1.246e+06	-1.336	-1.336	-0.4164
149	DiMe-Gly	Amino acid	1	AT_Untreated_1_3	AT_Untreated_1	1.0	Amino acid derivatives	True	2.394e+06	-0.3934	-0.3934	0.1269
703	NAD+	Cofactor	1	AT_Untreated_1_3	AT_Untreated_1	1.0	Cofactors	True	1.556e+07	2.307	2.307	-0.4297
709	Pyridoxal-P	Cofactor	1	AT_Untreated_1_3	AT_Untreated_1	0.833333333333333	Cofactors	True	5.665e+05	-2.473	-2.473	-0.1649
731	Thiamin (Vitamin B1)	Cofactor	1	AT_Untreated_1_3	AT_Untreated_1	1.0	TPP biosynthesis	True	4.133e+06	0.3942	0.3942	-0.3535
374	UDP	Nucleotide	1	AT_Untreated_1_3	AT_Untreated_1	0.666666666666667	Pyrimidine nucleotides	True	4.724e+06	0.587	0.587	2.141
102	2-Aminoadipate	Amino acid	1	AT_Untreated_1_3	AT_Untreated_1	0.833333333333333	Amino acids biosynthesis intermediates	True	6.758e+05	-2.218	-2.218	-0.1087
45	Fructose-6-P	Carbon	1	AT_Untreated_1_3	AT_Untreated_1	1.0	Glycolysis, GNG	True	5.753e+06	0.8714	0.8714	0.7961
320	TMP	Nucleotide	1	AT_Untreated_1_3	AT_Untreated_1	0.5	Deoxy-nucleotides	True	6.845e+04	-5.522	-5.522	-0.7301
341	XMP	Nucleotide	1	AT_Untreated_1_3	AT_Untreated_1	0.166666666666667	IMP conversion to AMP & GMP	False			-6.353	-1.778
120	beta-OH-Isovalerate	Amino acid	1	AT_Untreated_1_3	AT_Untreated_1	1.0	Amino acids degradation intermediates	True	1.151e+06	-1.45	-1.45	1.715
322	2'-dI	Nucleotide	1	AT_Untreated_1_3	AT_Untreated_1	0.5	Deoxy-nucleosides	True	6.071e+04	-5.695	-5.695	-1.57
4	GlcNAc 6-P	Carbon	1	AT_Untreated_1_3	AT_Untreated_1	1.0	Aminosugar biosynthesis	True	8.367e+06	1.412	1.412	1.083
337	Xanthosine	Nucleotide	1	AT_Untreated_1_3	AT_Untreated_1	1.0	Purine nucleosides	True	1.133e+06	-1.473	-1.473	1.602
188	Kynurenine	Amino acid	1	AT_Untreated_1_3	AT_Untreated_1	1.0	Amino acid derivatives	True	1.073e+08	5.092	5.092	5.784

Metabolite ID	Name	Super Pathway	Dataset	Sample ID	Group ID	Detection Fraction	Pathway	Detected	Raw Intensity	Log2 Norm Intensity	Norm Imputed	Log2 Ctrl Norm
63	6-P-Gluconate	Carbon	1	AT_Untreated_1_3	AT_Untreated_1	1.0	Pentose phosphate pathway (PPP)	True	2.115e+07	2.75	2.75	0.716
40	Glucuronate	Carbon	1	AT_Untreated_1_3	AT_Untreated_1	1.0	Sugars and sugar alcohols	True	9.481e+05	-1.73	-1.73	-0.1907
108	Argininosuccinate	Amino acid	1	AT_Untreated_1_3	AT_Untreated_1	1.0	Amino acids biosynthesis intermediates	True	1.308e+06	-1.265	-1.265	-1.039
710	Carnitine	Cofactor	1	AT_Untreated_1_3	AT_Untreated_1	1.0	Cofactors	True	1.859e+08	5.885	5.885	-0.09219
725	P-Pantetheine	Cofactor	1	AT_Untreated_1_3	AT_Untreated_1	1.0	Coenzyme A biosynthesis	True	2.814e+05	-3.482	-3.482	0.2226
48	DHAP	Carbon	1	AT_Untreated_1_3	AT_Untreated_1	1.0	Glycolysis, GNG	True	3.291e+07	3.387	3.387	0.2908
17	Maltose	Carbon	1	AT_Untreated_1_3	AT_Untreated_1	1.0	Glycogen degradation	True	1.537e+06	-1.033	-1.033	0.26
359	N1-Me-Adenosine	Nucleotide	1	AT_Untreated_1_3	AT_Untreated_1	0.5	Purine derivatives in RNAs	True	7.691e+05	-2.032	-2.032	-0.9939
159	3-Me-His	Amino acid	1	AT_Untreated_1_3	AT_Untreated_1	1.0	Amino acid derivatives	True	1.136e+05	-4.79	-4.79	-0.5549
155	4-Guanidinobutanoate	Amino acid	1	AT_Untreated_1_3	AT_Untreated_1	1.0	Amino acid derivatives	True	1.162e+06	-1.436	-1.436	-2.43e-03
164	5-OH-Lys	Amino acid	1	AT_Untreated_1_3	AT_Untreated_1	1.0	Amino acid derivatives	True	1.033e+06	-1.606	-1.606	0.1644
357	Adenosine-3',5'-PP	Nucleotide	1	AT_Untreated_1_3	AT_Untreated_1	0.666666666666667	Purine byproducts of metabolic processes	True	1.743e+05	-4.173	-4.173	-0.6959
104	Cystathionine	Amino acid	1	AT_Untreated_1_3	AT_Untreated_1	1.0	Amino acids biosynthesis intermediates	True	9.456e+06	1.588	1.588	-0.6212
113	Imidazole Lactate	Amino acid	1	AT_Untreated_1_3	AT_Untreated_1	0.666666666666667	Amino acids degradation intermediates	True	2.997e+05	-3.391	-3.391	0.2854
215	N-Ac-Glu	Amino acid	1	AT_Untreated_1_3	AT_Untreated_1	1.0	N-acetylated amino acids	True	4.374e+06	0.4759	0.4759	-1.268
310	S-Lactoyl-Glutathione	Amino acid	1	AT_Untreated_1_3	AT_Untreated_1	1.0	Glutathione derivatives	True	3.081e+06	-0.02972	-0.02972	-0.3861
5	GlcNAc 1-P	Carbon	1	AT_Untreated_1_3	AT_Untreated_1	0.833333333333333	Aminosugar biosynthesis	True	1.742e+06	-0.8523	-0.8523	1.059
34	Ribitol	Carbon	1	AT_Untreated_1_3	AT_Untreated_1	1.0	Sugars and sugar alcohols	True	4.325e+05	-2.862	-2.862	0.5678
10	UDP-Galactose	Carbon	1	AT_Untreated_1_3	AT_Untreated_1	0.833333333333333	Polysaccharide biosynthesis	True	7.427e+06	1.24	1.24	1.866
13	Guanosine 5'-PP-Fucose	Carbon	1	AT_Untreated_1_3	AT_Untreated_1	1.0	Polysaccharide biosynthesis	True	1.406e+06	-1.161	-1.161	1.138

Metabolite ID	Name	Super Pathway	Dataset	Sample ID	Group ID	Detection Fraction	Pathway	Detected	Raw Intensity	Log2 Norm Intensity	Norm Imputed	Log2 Ctrl Norm
19	Maltotetraose	Carbon	1	AT_Untreated_1_3	AT_Untreated_1	1.0	Glycogen degradation	True	1.164e+07	1.888	1.888	1.932
233	SAM	Amino acid	1	AT_Untreated_1_3	AT_Untreated_1	0.333333333333333	SAM metabolism	True	7.228e+05	-2.121	-2.121	-1.965
129	5-Aminovalerate	Amino acid	1	AT_Untreated_1_3	AT_Untreated_1	0.333333333333333	Amino acids degradation intermediates	True	7.654e+06	1.283	1.283	0.2817
741	5-Me-THF	Cofactor	1	AT_Untreated_1_3	AT_Untreated_1	0.333333333333333	Folate metabolism	True	1.433e+05	-4.456	-4.456	0.3023
198	Indolelactate	Amino acid	1	AT_Untreated_1_3	AT_Untreated_1	1.0	Amino acid derivatives	True	3.185e+05	-3.304	-3.304	0.4836
254	Gly-Val	Amino acid	1	AT_Untreated_1_3	AT_Untreated_1	1.0	Dipeptides	True	6.813e+06	1.115	1.115	4.13e-03
291	gamma-Glu-Leu	Amino acid	1	AT_Untreated_1_3	AT_Untreated_1	1.0	Gamma-glutamyl dipeptides	True	1.048e+06	-1.586	-1.586	-1.097
173	Met Sulfoxide	Amino acid	1	AT_Untreated_1_3	AT_Untreated_1	1.0	Amino acid derivatives	True	2.179e+07	2.793	2.793	0.2255
43	Glucose	Carbon	1	AT_Untreated_1_3	AT_Untreated_1	1.0	Glycolysis, GNG	True	1.058e+08	5.072	5.072	0.0207
185	Phenyllactate	Amino acid	1	AT_Untreated_1_3	AT_Untreated_1	0.833333333333333	Amino acid derivatives	True	2.871e+05	-3.453	-3.453	2.359
156	Homo-Arg	Amino acid	1	AT_Untreated_1_3	AT_Untreated_1	1.0	Amino acid derivatives	True	4.492e+06	0.5145	0.5145	-0.666
135	Homocitrulline	Amino acid	1	AT_Untreated_1_3	AT_Untreated_1	1.0	Amino acids degradation intermediates	True	6.34e+05	-2.31	-2.31	-0.6078
719	Nicotinamide MN	Cofactor	1	AT_Untreated_1_3	AT_Untreated_1	1.0	NAD biosynthesis	True	4.047e+06	0.3641	0.3641	0.1785
212	N-Ac-Asp	Amino acid	1	AT_Untreated_1_3	AT_Untreated_1	1.0	N-acetylated amino acids	True	2.172e+06	-0.534	-0.534	-1.231
720	1-Me-Nicotinamide	Cofactor	1	AT_Untreated_1_3	AT_Untreated_1	1.0	Derivatives of NA, nicotinamide and NAD	True	4.633e+08	7.203	7.203	-0.6878
216	N-Ac-Gly	Amino acid	1	AT_Untreated_1_3	AT_Untreated_1	0.5	N-acetylated amino acids	False			-4.155	-0.6151
70	Creatine	Carbon	1	AT_Untreated_1_3	AT_Untreated_1	1.0	Creatine energy storage	True	1.574e+09	8.967	8.967	-0.4498
26	Galactonate	Carbon	1	AT_Untreated_1_3	AT_Untreated_1	0.666666666666667	Sugars and sugar alcohols	False			-2.211	-1.406
309	Glutathione, Oxidized	Amino acid	1	AT_Untreated_1_3	AT_Untreated_1	1.0	Glutathione	True	1.081e+07	1.781	1.781	0.5884
35	Ribonate	Carbon	1	AT_Untreated_1_3	AT_Untreated_1	0.833333333333333	Sugars and sugar alcohols	True	2.554e+06	-0.3002	-0.3002	0.227
160	1-Me-His	Amino acid	1	AT_Untreated_1_3	AT_Untreated_1	1.0	Amino acid derivatives	True	2.496e+07	2.988	2.988	-0.4527

Metabolite ID	Name	Super Pathway	Dataset	Sample ID	Group ID	Detection Fraction	Pathway	Detected	Raw Intensity	Log2 Norm Intensity	Norm Imputed	Log2 Ctrl Norm
44	Glucose 6-P	Carbon	1	AT_Untreated_1_3	AT_Untreated_1	0.833333333333333	Glycolysis, GNG	True	1.031e+06	-1.609	-1.609	-0.132
704	NADH	Cofactor	1	AT_Untreated_1_3	AT_Untreated_1	1.0	Cofactors	True	2.414e+06	-0.3812	-0.3812	0.2648
275	Thr-Phe	Amino acid	1	AT_Untreated_1_3	AT_Untreated_1	0.833333333333333	Dipeptides	True	1.838e+06	-0.7744	-0.7744	0.8788
738	Pyridoxate	Cofactor	1	AT_Untreated_1_3	AT_Untreated_1	1.0	PLP biosynthesis and salvage	True	3.179e+05	-3.306	-3.306	0.05803
177	3-(4-OH-Phenyl)Lactate	Amino acid	1	AT_Untreated_1_3	AT_Untreated_1	1.0	Amino acid derivatives	True	1.326e+06	-1.246	-1.246	1.219
206	Trans-4-OH-Pro	Amino acid	1	AT_Untreated_1_3	AT_Untreated_1	1.0	Amino acid derivatives	True	5.119e+07	4.025	4.025	-1.13
329	AMP	Nucleotide	1	AT_Untreated_1_3	AT_Untreated_1	1.0	Purine nucleotides	True	9.211e+07	4.872	4.872	1.237
11	UDP-Glucose	Carbon	1	AT_Untreated_1_3	AT_Untreated_1	0.833333333333333	Polysaccharide biosynthesis	True	7.206e+06	1.196	1.196	1.302
158	4-Imidazole-Ac	Amino acid	1	AT_Untreated_1_3	AT_Untreated_1	1.0	Amino acid derivatives	True	3.402e+05	-3.208	-3.208	-1.062
111	1-Me-Imidazole-Ac	Amino acid	1	AT_Untreated_1_3	AT_Untreated_1	1.0	Amino acids degradation intermediates	True	7.813e+05	-2.009	-2.009	-0.07513
345	Guanine	Nucleotide	1	AT_Untreated_1_3	AT_Untreated_1	1.0	Purine bases	True	5.075e+07	4.012	4.012	-0.9739
22	N-Ac-Neuraminate	Carbon	1	AT_Untreated_1_3	AT_Untreated_1	1.0	Aminosugar derivatives	True	3.503e+06	0.1557	0.1557	0.2911
721	N'-Methylnicotinate	Cofactor	1	AT_Untreated_1_3	AT_Untreated_1	1.0	Derivatives of NA, nicotinamide and NAD	True	6.732e+05	-2.224	-2.224	-1.216
183	Phenol Sulfate	Amino acid	1	AT_Untreated_1_3	AT_Untreated_1	1.0	Amino acid derivatives	True	4.472e+05	-2.814	-2.814	1.826
718	Nicotinamide Riboside	Cofactor	1	AT_Untreated_1_3	AT_Untreated_1	1.0	NAD biosynthesis	True	1.214e+06	-1.373	-1.373	-2.963
297	gamma-Glu-Thr	Amino acid	1	AT_Untreated_1_3	AT_Untreated_1	1.0	Gamma-glutamyl dipeptides	True	2.624e+06	-0.261	-0.261	-0.9011
295	gamma-Glu-Phe	Amino acid	1	AT_Untreated_1_3	AT_Untreated_1	0.166666666666667	Gamma-glutamyl dipeptides	False			-5.975	-0.1899
347	Allantoic Acid	Nucleotide	1	AT_Untreated_1_3	AT_Untreated_1	0.666666666666667	Purine degradation	True	3.538e+04	-6.474	-6.474	-0.8345
399	Pseudouridine	Nucleotide	1	AT_Untreated_1_3	AT_Untreated_1	1.0	Pyrimidine derivatives in RNAs	True	7.792e+05	-2.013	-2.013	0.01714
375	UTP	Nucleotide	1	AT_Untreated_1_3	AT_Untreated_1	0.666666666666667	Pyrimidine nucleotides	True	1.604e+06	-0.9715	-0.9715	1.004
144	Glu, gamma-Me Ester	Amino acid	1	AT_Untreated_1_3	AT_Untreated_1	1.0	Amino acid derivatives	True	3.669e+06	0.2224	0.2224	-0.3554

Metabolite ID	Name	Super Pathway	Dataset	Sample ID	Group ID	Detection Fraction	Pathway	Detected	Raw Intensity	Log2 Norm Intensity	Norm Imputed	Log2 Ctrl Norm
292	gamma-Glu-epsilon-Lysine	Amino acid	1	AT_Untreated_1_3	AT_Untreated_1	1.0	Gamma-glutamyl dipeptides	True	3.21e+06	0.02972	0.02972	0.2211
225	N-Ac-Thr	Amino acid	1	AT_Untreated_1_3	AT_Untreated_1	1.0	N-acetylated amino acids	True	8.275e+05	-1.926	-1.926	-0.4236
211	N-Ac-Asn	Amino acid	1	AT_Untreated_1_3	AT_Untreated_1	1.0	N-acetylated amino acids	True	2.627e+05	-3.581	-3.581	-0.8961
151	Phenylacetylglycine	Amino acid	1	AT_Untreated_1_3	AT_Untreated_1	1.0	Amino acid derivatives	True	6.961e+05	-2.176	-2.176	-1.389
217	N-Ac-His	Amino acid	1	AT_Untreated_1_3	AT_Untreated_1	0.833333333333333	N-acetylated amino acids	True	2.053e+05	-3.937	-3.937	-0.4519
288	gamma-Glu-Gly	Amino acid	1	AT_Untreated_1_3	AT_Untreated_1	0.333333333333333	Gamma-glutamyl dipeptides	False			-3.092	-1.268
222	N-Ac-Phe	Amino acid	1	AT_Untreated_1_3	AT_Untreated_1	0.333333333333333	N-acetylated amino acids	False			-6.698	-0.4255
71	Creatine-P	Carbon	1	AT_Untreated_1_3	AT_Untreated_1	1.0	Creatine energy storage	True	9.987e+04	-4.977	-4.977	-0.7215
210	N-Ac-Arg	Amino acid	1	AT_Untreated_1_3	AT_Untreated_1	0.666666666666667	N-acetylated amino acids	True	4.253e+05	-2.886	-2.886	-0.2109
218	N-Ac-Ile	Amino acid	1	AT_Untreated_1_3	AT_Untreated_1	0.166666666666667	N-acetylated amino acids	False			-6.52	-0.5541
251	Gly-Leu	Amino acid	1	AT_Untreated_1_3	AT_Untreated_1	1.0	Dipeptides	True	1.086e+07	1.788	1.788	0.1498
290	gamma-Glu-Ile	Amino acid	1	AT_Untreated_1_3	AT_Untreated_1	1.0	Gamma-glutamyl dipeptides	True	2.177e+05	-3.852	-3.852	-2.514
316	Ophthalmate	Amino acid	1	AT_Untreated_1_3	AT_Untreated_1	1.0	Oxidative stress markers	True	1.405e+06	-1.163	-1.163	-3.505
125	Isovaleryl-Gly	Amino acid	1	AT_Untreated_1_3	AT_Untreated_1	1.0	Amino acids degradation intermediates	True	8.823e+04	-5.156	-5.156	0.254
368	7-Me-Guanine	Nucleotide	1	AT_Untreated_1_3	AT_Untreated_1	1.0	Purine derivatives in RNAs	True	6.796e+05	-2.21	-2.21	-0.02009
208	Pro-OH-Pro	Amino acid	1	AT_Untreated_1_3	AT_Untreated_1	1.0	Amino acid derivatives	True	2.729e+07	3.117	3.117	-0.212
366	N2,N2-DiMe-Guanosine	Nucleotide	1	AT_Untreated_1_3	AT_Untreated_1	0.833333333333333	Purine derivatives in RNAs	True	1.18e+05	-4.736	-4.736	-0.6226
352	3'-AMP	Nucleotide	1	AT_Untreated_1_3	AT_Untreated_1	1.0	Purine derivatives in signaling	True	1.642e+06	-0.9373	-0.9373	0.5495
363	N6-Carbamoyl-Thr-Adenosine	Nucleotide	1	AT_Untreated_1_3	AT_Untreated_1	0.666666666666667	Purine derivatives in RNAs	True	8.667e+04	-5.181	-5.181	-0.4959
314	Cys-Glutathione Disulfide	Amino acid	1	AT_Untreated_1_3	AT_Untreated_1	1.0	Oxidative stress markers	True	2.836e+05	-3.471	-3.471	-0.187
382	Orotidine	Nucleotide	1	AT_Untreated_1_3	AT_Untreated_1	0.166666666666667	Pyrimidine (UMP) biosynthesis	False			-5.287	-0.9952

Metabolite ID	Name	Super Pathway	Dataset	Sample ID	Group ID	Detection Fraction	Pathway	Detected	Raw Intensity	Log2 Norm Intensity	Norm Imputed	Log2 Ctrl Norm
307	Cys-Gly	Amino acid	1	AT_Untreated_1_3	AT_Untreated_1	1.0	Glutathione biosynthesis	True	1.464e+07	2.219	2.219	0.6116
64	Sedoheptulose-7-P	Carbon	1	AT_Untreated_1_3	AT_Untreated_1	1.0	Pentose phosphate pathway (PPP)	True	2.958e+06	-0.08809	-0.08809	-0.2422
142	N-Ac-Asp-Glu	Amino acid	1	AT_Untreated_1_3	AT_Untreated_1	1.0	Amino acid derivatives	True	4.954e+05	-2.666	-2.666	-1.521
708	Thiamin-PP	Cofactor	1	AT_Untreated_1_3	AT_Untreated_1	0.5	Cofactors	True	1.144e+05	-4.78	-4.78	1.203
182	P-Cresol Sulfate	Amino acid	1	AT_Untreated_1_3	AT_Untreated_1	1.0	Amino acid derivatives	True	4.62e+05	-2.767	-2.767	0.4293
250	Gly-Ile	Amino acid	1	AT_Untreated_1_3	AT_Untreated_1	1.0	Dipeptides	True	1.325e+06	-1.247	-1.247	0.07022
286	gamma-Glu-Glu	Amino acid	1	AT_Untreated_1_3	AT_Untreated_1	1.0	Gamma-glutamyl dipeptides	True	1.692e+06	-0.8945	-0.8945	-0.9123
739	Deoxycarnitine	Cofactor	1	AT_Untreated_1_3	AT_Untreated_1	1.0	Carnitine biosynthesis	True	2.076e+07	2.723	2.723	-1.029
203	DiMe-Arg	Amino acid	1	AT_Untreated_1_3	AT_Untreated_1	1.0	Amino acid derivatives	True	1.005e+08	4.999	4.999	-0.6338
351	2'-AMP	Nucleotide	1	AT_Untreated_1_3	AT_Untreated_1	1.0	Purine derivatives in signaling	True	6.227e+05	-2.336	-2.336	-0.0678
8	Cytidine 5'-P-N-Ac-Neuraminate	Carbon	1	AT_Untreated_1_3	AT_Untreated_1	1.0	Aminosugar biosynthesis	True	9.763e+05	-1.688	-1.688	0.05792
285	gamma-Glu-Ala	Amino acid	1	AT_Untreated_1_3	AT_Untreated_1	0.5	Gamma-glutamyl dipeptides	True	4.838e+04	-6.022	-6.022	-2.783
224	N-Ac-Ser	Amino acid	1	AT_Untreated_1_3	AT_Untreated_1	1.0	N-acetylated amino acids	True	4.616e+06	0.5536	0.5536	-1.326
244	Ala-Leu	Amino acid	1	AT_Untreated_1_3	AT_Untreated_1	1.0	Dipeptides	True	1.478e+07	2.233	2.233	1.095
207	N-Me-Pro	Amino acid	1	AT_Untreated_1_3	AT_Untreated_1	1.0	Amino acid derivatives	True	1.485e+06	-1.083	-1.083	-0.1529
171	Cys Sulfinic Acid	Amino acid	1	AT_Untreated_1_3	AT_Untreated_1	0.333333333333333	Amino acid derivatives	True	2.948e+05	-3.415	-3.415	-0.1175
181	O-Me-Tyr	Amino acid	1	AT_Untreated_1_3	AT_Untreated_1	0.5	Amino acid derivatives	True	1.71e+05	-4.2	-4.2	-0.8306
240	N-Ac-Putrescine	Amino acid	1	AT_Untreated_1_3	AT_Untreated_1	0.833333333333333	Polyamine derivatives	True	1.394e+06	-1.173	-1.173	1.191
176	S-Me-Met	Amino acid	1	AT_Untreated_1_3	AT_Untreated_1	1.0	Amino acid derivatives	True	8.029e+05	-1.97	-1.97	-0.1279
339	AICAR	Nucleotide	1	AT_Untreated_1_3	AT_Untreated_1	0.333333333333333	IMP biosynthesis	True	1.196e+05	-4.716	-4.716	-0.4151
141	gamma-Carboxy-Glu	Amino acid	1	AT_Untreated_1_3	AT_Untreated_1	1.0	Amino acid derivatives	True	1.551e+06	-1.019	-1.019	-0.2344
392	3'-UMP	Nucleotide	1	AT_Untreated_1_3	AT_Untreated_1	0.833333333333333	Pyrimidine derivatives in signaling	True	1.515e+06	-1.054	-1.054	2.979

Metabolite ID	Name	Super Pathway	Dataset	Sample ID	Group ID	Detection Fraction	Pathway	Detected	Raw Intensity	Log2 Norm Intensity	Norm Imputed	Log2 Ctrl Norm
355	3'-GMP	Nucleotide	1	AT_Untreated_1_3	AT_Untreated_1	0.833333333333333	Purine derivatives in signaling	True	3.655e+05	-3.105	-3.105	0.8324
282	Val-Leu	Amino acid	1	AT_Untreated_1_3	AT_Untreated_1	1.0	Dipeptides	True	1.307e+07	2.056	2.056	1.084
140	Carboxyethyl-GABA	Amino acid	1	AT_Untreated_1_3	AT_Untreated_1	1.0	Amino acid derivatives	True	6.209e+05	-2.34	-2.34	-0.9093
258	Ile-Gly	Amino acid	1	AT_Untreated_1_3	AT_Untreated_1	1.0	Dipeptides	True	3.384e+07	3.428	3.428	0.1156
260	Leu-Ala	Amino acid	1	AT_Untreated_1_3	AT_Untreated_1	1.0	Dipeptides	True	1.112e+07	1.822	1.822	0.521
265	Lys-Leu	Amino acid	1	AT_Untreated_1_3	AT_Untreated_1	0.833333333333333	Dipeptides	True	3.683e+05	-3.094	-3.094	-0.4827
263	Leu-Gly	Amino acid	1	AT_Untreated_1_3	AT_Untreated_1	1.0	Dipeptides	True	1.008e+08	5.002	5.002	1.981
281	Val-Gly	Amino acid	1	AT_Untreated_1_3	AT_Untreated_1	1.0	Dipeptides	True	7.334e+07	4.544	4.544	0.02446
270	Pro-Gly	Amino acid	1	AT_Untreated_1_3	AT_Untreated_1	1.0	Dipeptides	True	2.873e+06	-0.1302	-0.1302	-0.8769
114	Imidazole Propionate	Amino acid	1	AT_Untreated_1_3	AT_Untreated_1	1.0	Amino acids degradation intermediates	True	4.382e+05	-2.843	-2.843	-0.5326
267	Phe-Gly	Amino acid	1	AT_Untreated_1_3	AT_Untreated_1	1.0	Dipeptides	True	6.001e+07	4.254	4.254	0.1603
266	Phe-Ala	Amino acid	1	AT_Untreated_1_3	AT_Untreated_1	1.0	Dipeptides	True	7.46e+06	1.246	1.246	0.1696
278	Tyr-Gly	Amino acid	1	AT_Untreated_1_3	AT_Untreated_1	1.0	Dipeptides	True	1.445e+07	2.201	2.201	0.01827
255	His-Ala	Amino acid	1	AT_Untreated_1_3	AT_Untreated_1	0.833333333333333	Dipeptides	True	1.493e+06	-1.075	-1.075	0.2624
280	Val-Gln	Amino acid	1	AT_Untreated_1_3	AT_Untreated_1	1.0	Dipeptides	True	2.06e+07	2.712	2.712	1.094
143	S-1-Pyrroline-5-Carboxylate	Amino acid	1	AT_Untreated_1_3	AT_Untreated_1	0.833333333333333	Amino acid derivatives	False			-3.202	-1.133
232	SAH	Amino acid	1	AT_Untreated_1_3	AT_Untreated_1	1.0	SAM metabolism	True	1.855e+06	-0.7618	-0.7618	-0.2764
20	Erythronate	Carbon	1	AT_Untreated_1_3	AT_Untreated_1	1.0	Aminosugar derivatives	True	3.911e+07	3.637	3.637	-0.1046
248	Gln-Leu	Amino acid	1	AT_Untreated_1_3	AT_Untreated_1	0.833333333333333	Dipeptides	True	2.2e+06	-0.5152	-0.5152	0.39
276	Trp-Gly	Amino acid	1	AT_Untreated_1_3	AT_Untreated_1	0.833333333333333	Dipeptides	True	1.362e+06	-1.207	-1.207	-0.1287
205	N-delta-Ac-Ornithine	Amino acid	1	AT_Untreated_1_3	AT_Untreated_1	1.0	Amino acid derivatives	True	8.638e+05	-1.864	-1.864	-0.7431
163	Formimino-Glu	Amino acid	1	AT_Untreated_1_3	AT_Untreated_1	1.0	Amino acid derivatives	True	1.16e+06	-1.438	-1.438	-0.1984
204	N-Me-Arg	Amino acid	1	AT_Untreated_1_3	AT_Untreated_1	0.833333333333333	Amino acid derivatives	True	1.946e+07	2.63	2.63	0.0427
242	Guanidino-Ac	Amino acid	1	AT_Untreated_1_3	AT_Untreated_1	1.0	Creatine biosynthesis	True	2.195e+05	-3.841	-3.841	0.03974

Metabolite ID	Name	Super Pathway	Dataset	Sample ID	Group ID	Detection Fraction	Pathway	Detected	Raw Intensity	Log2 Norm Intensity	Norm Imputed	Log2 Ctrl Norm
300	gamma-Glu-Val	Amino acid	1	AT_Untreated_1_3	AT_Untreated_1	0.333333333333333	Gamma-glutamyl dipeptides	False			-0.1053	-0.726
53	Ac-CoA	Carbon	1	AT_Untreated_1_3	AT_Untreated_1	0.166666666666667	Acetyl-CoA	True	2.032e+04	-7.274	-7.274	-0.5114
18	Maltotriose	Carbon	1	AT_Untreated_1_3	AT_Untreated_1	1.0	Glycogen degradation	True	6.944e+06	1.143	1.143	1.301
294	gamma-Glu-Met	Amino acid	1	AT_Untreated_1_3	AT_Untreated_1	0.333333333333333	Gamma-glutamyl dipeptides	True	2.177e+05	-3.853	-3.853	-0.3708
174	Met Sulfone	Amino acid	1	AT_Untreated_1_3	AT_Untreated_1	0.833333333333333	Amino acid derivatives	True	1.998e+05	-3.976	-3.976	-0.8974
175	N-Ac-Met Sulfoxide	Amino acid	1	AT_Untreated_1_3	AT_Untreated_1	1.0	Amino acid derivatives	True	3.743e+06	0.2513	0.2513	0.275
25	Mannitol/Sorbitol	Carbon	1	AT_Untreated_1_3	AT_Untreated_1	1.0	Sugars and sugar alcohols	True	2.297e+07	2.869	2.869	0.644
6	UDP-GlcNAc	Carbon	1	AT_Untreated_1_3	AT_Untreated_1	0.166666666666667	Aminosugar biosynthesis	True	2.766e+05	-3.507	-3.507	-0.3993
145	Pyro-Gln	Amino acid	1	AT_Untreated_1_3	AT_Untreated_1	1.0	Amino acid derivatives	True	3.373e+06	0.101	0.101	-0.7388
705	Coenzyme A	Cofactor	1	AT_Untreated_1_3	AT_Untreated_1	0.666666666666667	Cofactors	True	2.37e+05	-3.73	-3.73	0.2288
319	2'-dAMP	Nucleotide	1	AT_Untreated_1_3	AT_Untreated_1	0.166666666666667	Deoxy-nucleotides	False			-4.337	-0.6568
119	alpha-OH-Isovalerate	Amino acid	1	AT_Untreated_1_3	AT_Untreated_1	1.0	Amino acids degradation intermediates	True	5.965e+05	-2.398	-2.398	0.9001
46	Fructose 1,6-PP / Glucose 1,6-PP / Inositol-1,4-PP / Inositol-1,3-PP	Carbon	1	AT_Untreated_1_3	AT_Untreated_1	1.0	Glycolysis, GNG	True	1.013e+08	5.009	5.009	0.1784
137	1-Me-Guanidine	Amino acid	1	AT_Untreated_1_3	AT_Untreated_1	0.666666666666667	Amino acids degradation intermediates	True	7.806e+04	-5.332	-5.332	-0.1796
23	N-GlcNAc-Asn	Carbon	1	AT_Untreated_1_3	AT_Untreated_1	1.0	Aminosugar derivatives	True	4.476e+06	0.5093	0.5093	-0.2036
262	Leu-Gln	Amino acid	1	AT_Untreated_1_3	AT_Untreated_1	1.0	Dipeptides	True	1.385e+07	2.139	2.139	0.8422
24	Fructose	Carbon	1	AT_Untreated_1_3	AT_Untreated_1	1.0	Sugars and sugar alcohols	True	1.722e+07	2.453	2.453	0.5042
197	C-Glycosyl-Trp	Amino acid	1	AT_Untreated_1_3	AT_Untreated_1	1.0	Amino acid derivatives	True	6.312e+06	1.005	1.005	1.304
33	Arabitol/Xylitol	Carbon	1	AT_Untreated_1_3	AT_Untreated_1	1.0	Sugars and sugar alcohols	True	4.501e+05	-2.805	-2.805	-0.9448

Metabolite ID	Name	Super Pathway	Dataset	Sample ID	Group ID	Detection Fraction	Pathway	Detected	Raw Intensity	Log2 Norm Intensity	Norm Imputed	Log2 Ctrl Norm
128	N2-Ac-Lys/N6-Ac-Lys	Amino acid	1	AT_Untreated_1_3	AT_Untreated_1	1.0	Amino acids degradation intermediates	True	3.006e+06	-0.06505	-0.06505	-1.275
42	2-Me-Citrate	Carbon	1	AT_Untreated_1_3	AT_Untreated_1	0.833333333333333	Propionate metabolism	True	1.434e+05	-4.455	-4.455	-0.07227
12	Glucuronate 1-P	Carbon	1	AT_Untreated_1_3	AT_Untreated_1	1.0	Polysaccharide biosynthesis	True	9.039e+05	-1.799	-1.799	-0.5558
76	Gln	Amino acid	1	AT_Untreated_1_4	AT_Untreated_1	1.0	Proteinogenic amino acids	True	9.135e+08	9.454	9.454	0.7738
89	Trp	Amino acid	1	AT_Untreated_1_4	AT_Untreated_1	1.0	Proteinogenic amino acids	True	8.153e+07	5.968	5.968	0.2926
723	beta-Ala	Cofactor	1	AT_Untreated_1_4	AT_Untreated_1	1.0	Coenzyme A biosynthesis	True	6.858e+06	2.397	2.397	-0.6124
75	Glu	Amino acid	1	AT_Untreated_1_4	AT_Untreated_1	1.0	Proteinogenic amino acids	True	2.074e+09	10.64	10.64	-0.09942
77	Gly	Amino acid	1	AT_Untreated_1_4	AT_Untreated_1	1.0	Proteinogenic amino acids	True	6.742e+07	5.694	5.694	0.079
80	His	Amino acid	1	AT_Untreated_1_4	AT_Untreated_1	1.0	Proteinogenic amino acids	True	1.836e+07	3.817	3.817	0.402
82	Leu	Amino acid	1	AT_Untreated_1_4	AT_Untreated_1	1.0	Proteinogenic amino acids	True	1.085e+09	9.703	9.703	0.4816
87	Phe	Amino acid	1	AT_Untreated_1_4	AT_Untreated_1	1.0	Proteinogenic amino acids	True	7.669e+08	9.202	9.202	0.3906
130	Glutarate	Amino acid	1	AT_Untreated_1_4	AT_Untreated_1	1.0	Amino acids degradation intermediates	True	4.847e+05	-1.426	-1.426	1.001
196	5-OH-Indole-Ac	Amino acid	1	AT_Untreated_1_4	AT_Untreated_1	1.0	Amino acid derivatives	True	3.211e+05	-2.02	-2.02	1.992
74	Asp	Amino acid	1	AT_Untreated_1_4	AT_Untreated_1	1.0	Proteinogenic amino acids	True	2.583e+08	7.632	7.632	-0.2598
236	Spermidine	Amino acid	1	AT_Untreated_1_4	AT_Untreated_1	1.0	Polyamines	True	1.845e+07	3.825	3.825	-0.03675
73	Asn	Amino acid	1	AT_Untreated_1_4	AT_Untreated_1	1.0	Proteinogenic amino acids	True	1.706e+08	7.033	7.033	0.3624
243	Creatinine	Amino acid	1	AT_Untreated_1_4	AT_Untreated_1	1.0	Creatine degradation	True	8.402e+07	6.012	6.012	1.01
376	Cytidine	Nucleotide	1	AT_Untreated_1_4	AT_Untreated_1	0.833333333333333	Pyrimidine nucleosides	True	7.5e+07	5.848	5.848	5.12
41	Lactate	Carbon	1	AT_Untreated_1_4	AT_Untreated_1	1.0	Respiratory carbon sources	True	6.389e+07	5.617	5.617	-0.8652
58	alpha-Ketoglutarate	Carbon	1	AT_Untreated_1_4	AT_Untreated_1	1.0	TCA cycle	True	2.698e+06	1.051	1.051	1.034
69	3-OH-Butyrate	Carbon	1	AT_Untreated_1_4	AT_Untreated_1	0.833333333333333	Ketone bodies	True	8.832e+05	-0.5601	-0.5601	2.424

Metabolite ID	Name	Super Pathway	Dataset	Sample ID	Group ID	Detection Fraction	Pathway	Detected	Raw Intensity	Log2 Norm Intensity	Norm Imputed	Log2 Ctrl Norm
343	Adenine	Nucleotide	1	AT_Untreated_1_4	AT_Untreated_1	1.0	Purine bases	True	1.505e+06	0.2093	0.2093	-1.248
336	Adenosine	Nucleotide	1	AT_Untreated_1_4	AT_Untreated_1	1.0	Purine nucleosides	True	1.098e+07	3.076	3.076	-0.3464
722	ADP-Ribose	Cofactor	1	AT_Untreated_1_4	AT_Untreated_1	1.0	Derivatives of NA, nicotinamide and NAD	True	3.887e+04	-5.066	-5.066	-2.064
383	Cytosine	Nucleotide	1	AT_Untreated_1_4	AT_Untreated_1	0.833333333333333	Pyrimidine bases	True	8.835e+04	-3.882	-3.882	0.9737
3	Glucosamine 6-P	Carbon	1	AT_Untreated_1_4	AT_Untreated_1	0.833333333333333	Aminosugar biosynthesis	True	1.298e+05	-3.327	-3.327	-1.659
717	Nicotinamide	Cofactor	1	AT_Untreated_1_4	AT_Untreated_1	1.0	NAD biosynthesis	True	6.778e+07	5.702	5.702	1.187
51	PEP	Carbon	1	AT_Untreated_1_4	AT_Untreated_1	1.0	Glycolysis, GNG	True	6.954e+06	2.417	2.417	1.366
237	Spermine	Amino acid	1	AT_Untreated_1_4	AT_Untreated_1	1.0	Polyamines	True	4.246e+06	1.705	1.705	3.196
385	Uracil	Nucleotide	1	AT_Untreated_1_4	AT_Untreated_1	1.0	Pyrimidine bases	True	2.349e+07	4.173	4.173	3.908
377	Uridine	Nucleotide	1	AT_Untreated_1_4	AT_Untreated_1	1.0	Pyrimidine nucleosides	True	3.339e+07	4.68	4.68	0.6315
348	Allantoin	Nucleotide	1	AT_Untreated_1_4	AT_Untreated_1	1.0	Purine degradation	True	2.731e+06	1.069	1.069	1.244
335	Inosine	Nucleotide	1	AT_Untreated_1_4	AT_Untreated_1	1.0	Purine nucleosides	True	5.221e+07	5.325	5.325	-0.1737
81	Ile	Amino acid	1	AT_Untreated_1_4	AT_Untreated_1	1.0	Proteinogenic amino acids	True	8.836e+08	9.406	9.406	0.6911
72	Ala	Amino acid	1	AT_Untreated_1_4	AT_Untreated_1	1.0	Proteinogenic amino acids	True	7.414e+08	9.153	9.153	0.6518
79	Thr	Amino acid	1	AT_Untreated_1_4	AT_Untreated_1	1.0	Proteinogenic amino acids	True	4.273e+08	8.358	8.358	0.3485
88	Tyr	Amino acid	1	AT_Untreated_1_4	AT_Untreated_1	1.0	Proteinogenic amino acids	True	3.487e+08	8.065	8.065	0.5414
84	Lys	Amino acid	1	AT_Untreated_1_4	AT_Untreated_1	1.0	Proteinogenic amino acids	True	3.851e+08	8.208	8.208	0.2182
86	Met	Amino acid	1	AT_Untreated_1_4	AT_Untreated_1	1.0	Proteinogenic amino acids	True	4.494e+08	8.431	8.431	0.2033
61	Malate	Carbon	1	AT_Untreated_1_4	AT_Untreated_1	1.0	TCA cycle	True	2.715e+08	7.704	7.704	0.5322
235	Putrescine	Amino acid	1	AT_Untreated_1_4	AT_Untreated_1	0.833333333333333	Polyamines	True	1.811e+05	-2.846	-2.846	-2.355
324	2'-dU	Nucleotide	1	AT_Untreated_1_4	AT_Untreated_1	0.666666666666667	Deoxy-nucleosides	True	7.252e+04	-4.166	-4.166	0.214
49	3-P-Glycerate	Carbon	1	AT_Untreated_1_4	AT_Untreated_1	1.0	Glycolysis, GNG	True	2.332e+07	4.163	4.163	0.8973
189	Kynurenate	Amino acid	1	AT_Untreated_1_4	AT_Untreated_1	1.0	Amino acid derivatives	True	7.651e+04	-4.089	-4.089	1.709
234	5-Me-Thioadenosine	Amino acid	1	AT_Untreated_1_4	AT_Untreated_1	1.0	SAM metabolism	True	3.281e+06	1.333	1.333	-0.5525

Metabolite ID	Name	Super Pathway	Dataset	Sample ID	Group ID	Detection Fraction	Pathway	Detected	Raw Intensity	Log2 Norm Intensity	Norm Imputed	Log2 Ctrl Norm
59	Succinate	Carbon	1	AT_Untreated_1_4	AT_Untreated_1	1.0	TCA cycle	True	2.687e+06	1.045	1.045	0.7438
36	Ribose	Carbon	1	AT_Untreated_1_4	AT_Untreated_1	1.0	Sugars and sugar alcohols	True	4.685e+05	-1.475	-1.475	-0.2412
133	Ornithine	Amino acid	1	AT_Untreated_1_4	AT_Untreated_1	1.0	Amino acids degradation intermediates	True	3.286e+07	4.657	4.657	0.1779
313	5-Oxoproline	Amino acid	1	AT_Untreated_1_4	AT_Untreated_1	1.0	Glutathione derivatives	True	7.095e+06	2.446	2.446	0.3583
165	N-6-Tri-Me-Lys	Amino acid	1	AT_Untreated_1_4	AT_Untreated_1	1.0	Amino acid derivatives	True	1.855e+07	3.832	3.832	0.06867
380	Orotate	Nucleotide	1	AT_Untreated_1_4	AT_Untreated_1	0.666666666666667	Pyrimidine (UMP) biosynthesis	True	2.4e+05	-2.44	-2.44	1.19
724	Pantothenate	Cofactor	1	AT_Untreated_1_4	AT_Untreated_1	1.0	Coenzyme A biosynthesis	True	8.019e+07	5.944	5.944	-0.3658
150	N-Me-Gly	Amino acid	1	AT_Untreated_1_4	AT_Untreated_1	1.0	Amino acid derivatives	True	9.73e+05	-0.4204	-0.4204	-0.12
122	3-OH-Isobutyrate	Amino acid	1	AT_Untreated_1_4	AT_Untreated_1	0.833333333333333	Amino acids degradation intermediates	True	5.076e+05	-1.359	-1.359	1.797
241	4-Acetamidobutanoate	Amino acid	1	AT_Untreated_1_4	AT_Untreated_1	1.0	Polyamine derivatives	True	5.153e+06	1.985	1.985	1.544
711	alpha-Tocopherol	Cofactor	1	AT_Untreated_1_4	AT_Untreated_1	1.0	Cofactors	True	4.749e+06	1.867	1.867	1.086
55	Citrate	Carbon	1	AT_Untreated_1_4	AT_Untreated_1	1.0	TCA cycle	True	7.21e+06	2.469	2.469	0.5199
387	3-Aminoisobutyrate	Nucleotide	1	AT_Untreated_1_4	AT_Untreated_1	0.833333333333333	Pyrimidine degradation	True	3.655e+05	-1.833	-1.833	0.1806
338	Guanosine	Nucleotide	1	AT_Untreated_1_4	AT_Untreated_1	1.0	Purine nucleosides	True	7.586e+07	5.864	5.864	0.7584
209	N-Ac-Ala	Amino acid	1	AT_Untreated_1_4	AT_Untreated_1	1.0	N-acetylated amino acids	True	4.002e+05	-1.702	-1.702	-0.501
221	N-Ac-Met	Amino acid	1	AT_Untreated_1_4	AT_Untreated_1	1.0	N-acetylated amino acids	True	2.058e+06	0.6604	0.6604	-1.084
228	N-Ac-Val	Amino acid	1	AT_Untreated_1_4	AT_Untreated_1	0.333333333333333	N-acetylated amino acids	False			-5.82	-0.449
346	Urate	Nucleotide	1	AT_Untreated_1_4	AT_Untreated_1	1.0	Purine degradation	True	1.755e+06	0.4309	0.4309	1.494
90	Arg	Amino acid	1	AT_Untreated_1_4	AT_Untreated_1	1.0	Proteinogenic amino acids	True	1.741e+09	10.38	10.38	0.6788
60	Fumarate	Carbon	1	AT_Untreated_1_4	AT_Untreated_1	1.0	TCA cycle	True	3.797e+06	1.544	1.544	0.5288
78	Ser	Amino acid	1	AT_Untreated_1_4	AT_Untreated_1	1.0	Proteinogenic amino acids	True	3.145e+08	7.916	7.916	0.6588
83	Val	Amino acid	1	AT_Untreated_1_4	AT_Untreated_1	1.0	Proteinogenic amino acids	True	5.016e+08	8.59	8.59	0.7174

Metabolite ID	Name	Super Pathway	Dataset	Sample ID	Group ID	Detection Fraction	Pathway	Detected	Raw Intensity	Log2 Norm Intensity	Norm Imputed	Log2 Ctrl Norm
734	Pyridoxal	Cofactor	1	AT_Untreated_1_4	AT_Untreated_1	1.0	PLP biosynthesis and salvage	True	1.141e+07	3.132	3.132	1.349
136	Urea	Amino acid	1	AT_Untreated_1_4	AT_Untreated_1	1.0	Amino acids degradation intermediates	True	4.214e+06	1.694	1.694	1.543
67	Ribose 1-P	Carbon	1	AT_Untreated_1_4	AT_Untreated_1	1.0	Pentose phosphate pathway (PPP)	True	3.798e+06	1.544	1.544	0.9232
284	Carnosine	Amino acid	1	AT_Untreated_1_4	AT_Untreated_1	1.0	Dipeptides	True	7.644e+05	-0.7685	-0.7685	1.34
306	gamma-Glu-Cys	Amino acid	1	AT_Untreated_1_4	AT_Untreated_1	1.0	Glutathione biosynthesis	True	6.366e+05	-1.033	-1.033	-0.6958
712	Retinol (Vit A)	Cofactor	1	AT_Untreated_1_4	AT_Untreated_1	0.833333333333333	Cofactors	True	2.065e+05	-2.656	-2.656	-0.2231
85	Cys	Amino acid	1	AT_Untreated_1_4	AT_Untreated_1	1.0	Proteinogenic amino acids	True	2.625e+07	4.333	4.333	-0.1804
91	Pro	Amino acid	1	AT_Untreated_1_4	AT_Untreated_1	1.0	Proteinogenic amino acids	True	8.602e+08	9.368	9.368	-0.0404
308	Glutathione, Reduced	Amino acid	1	AT_Untreated_1_4	AT_Untreated_1	1.0	Glutathione	True	1.189e+08	6.513	6.513	-0.4432
107	Citrulline	Amino acid	1	AT_Untreated_1_4	AT_Untreated_1	1.0	Amino acids biosynthesis intermediates	True	2.46e+07	4.24	4.24	1.415
328	IMP	Nucleotide	1	AT_Untreated_1_4	AT_Untreated_1	0.333333333333333	Purine nucleotides	True	8.998e+04	-3.855	-3.855	0.4665
706	FAD	Cofactor	1	AT_Untreated_1_4	AT_Untreated_1	1.0	Cofactors	True	2.374e+05	-2.456	-2.456	-0.2661
735	Pyridoxamine	Cofactor	1	AT_Untreated_1_4	AT_Untreated_1	1.0	PLP biosynthesis and salvage	True	1.302e+06	0	0	0.193
199	Serotonin	Amino acid	1	AT_Untreated_1_4	AT_Untreated_1	1.0	Amino acid derivatives	True	6.947e+06	2.415	2.415	1.143
370	CMP	Nucleotide	1	AT_Untreated_1_4	AT_Untreated_1	1.0	Pyrimidine nucleotides	True	3.227e+06	1.309	1.309	-0.5299
287	gamma-Glu-Gln	Amino acid	1	AT_Untreated_1_4	AT_Untreated_1	1.0	Gamma-glutamyl dipeptides	True	1.617e+06	0.312	0.312	-0.5289
14	UDP-Glucuronate	Carbon	1	AT_Untreated_1_4	AT_Untreated_1	0.666666666666667	Polysaccharide biosynthesis	False			-4.022	-2.073
229	N-Formyl-Met	Amino acid	1	AT_Untreated_1_4	AT_Untreated_1	0.666666666666667	N-formylated amino acids	True	5.533e+04	-4.557	-4.557	-0.7345
350	3',5'-cAMP	Nucleotide	1	AT_Untreated_1_4	AT_Untreated_1	0.666666666666667	Purine derivatives in signaling	True	8.682e+04	-3.907	-3.907	-0.2875
371	CDP	Nucleotide	1	AT_Untreated_1_4	AT_Untreated_1	0.666666666666667	Pyrimidine nucleotides	False			-5.544	-2.224
372	CTP	Nucleotide	1	AT_Untreated_1_4	AT_Untreated_1	0.333333333333333	Pyrimidine nucleotides	False			-4.713	-1.059

Metabolite ID	Name	Super Pathway	Dataset	Sample ID	Group ID	Detection Fraction	Pathway	Detected	Raw Intensity	Log2 Norm Intensity	Norm Imputed	Log2 Ctrl Norm
333	GDP	Nucleotide	1	AT_Untreated_1_4	AT_Untreated_1	0.5	Purine nucleotides	False			-4.484	-1.983
332	GMP	Nucleotide	1	AT_Untreated_1_4	AT_Untreated_1	1.0	Purine nucleotides	True	4.963e+04	-4.714	-4.714	-5.412
373	UMP	Nucleotide	1	AT_Untreated_1_4	AT_Untreated_1	0.833333333333333	Pyrimidine nucleotides	True	5.036e+05	-1.371	-1.371	-0.3154
389	3'-CMP	Nucleotide	1	AT_Untreated_1_4	AT_Untreated_1	1.0	Pyrimidine derivatives in signaling	True	2.682e+05	-2.279	-2.279	-1.228
330	ADP	Nucleotide	1	AT_Untreated_1_4	AT_Untreated_1	0.5	Purine nucleotides	False			-2.826	-2.53
342	Hypoxanthine	Nucleotide	1	AT_Untreated_1_4	AT_Untreated_1	1.0	Purine bases	True	2.504e+07	4.265	4.265	0.3261
736	Pyridoxamine-P	Cofactor	1	AT_Untreated_1_4	AT_Untreated_1	0.833333333333333	PLP biosynthesis and salvage	True	6.406e+04	-4.345	-4.345	-1.367
148	Betaine	Amino acid	1	AT_Untreated_1_4	AT_Untreated_1	1.0	Amino acid derivatives	True	6.324e+07	5.602	5.602	0.796
344	Xanthine	Nucleotide	1	AT_Untreated_1_4	AT_Untreated_1	1.0	Purine bases	True	5.007e+06	1.943	1.943	1.036
386	3-Ureidopropionate	Nucleotide	1	AT_Untreated_1_4	AT_Untreated_1	1.0	Pyrimidine degradation	True	1.523e+06	0.2264	0.2264	1.146
149	DiMe-Gly	Amino acid	1	AT_Untreated_1_4	AT_Untreated_1	1.0	Amino acid derivatives	True	2.159e+06	0.7297	0.7297	1.25
703	NAD+	Cofactor	1	AT_Untreated_1_4	AT_Untreated_1	1.0	Cofactors	True	3.851e+06	1.564	1.564	-1.172
709	Pyridoxal-P	Cofactor	1	AT_Untreated_1_4	AT_Untreated_1	0.833333333333333	Cofactors	True	2.261e+05	-2.526	-2.526	-0.2177
731	Thiamin (Vitamin B1)	Cofactor	1	AT_Untreated_1_4	AT_Untreated_1	1.0	TPP biosynthesis	True	2.924e+06	1.167	1.167	0.4192
374	UDP	Nucleotide	1	AT_Untreated_1_4	AT_Untreated_1	0.666666666666667	Pyrimidine nucleotides	False			-5.339	-3.785
102	2-Aminoadipate	Amino acid	1	AT_Untreated_1_4	AT_Untreated_1	0.833333333333333	Amino acids biosynthesis intermediates	True	2.135e+05	-2.608	-2.608	-0.4989
45	Fructose-6-P	Carbon	1	AT_Untreated_1_4	AT_Untreated_1	1.0	Glycolysis, GNG	True	3.407e+05	-1.934	-1.934	-2.01
320	TMP	Nucleotide	1	AT_Untreated_1_4	AT_Untreated_1	0.5	Deoxy-nucleotides	False			-6.992	-2.2
341	XMP	Nucleotide	1	AT_Untreated_1_4	AT_Untreated_1	0.166666666666667	IMP conversion to AMP & GMP	False			-6.353	-1.778
120	beta-OH-Isovalerate	Amino acid	1	AT_Untreated_1_4	AT_Untreated_1	1.0	Amino acids degradation intermediates	True	6.407e+05	-1.023	-1.023	2.142
322	2'-dI	Nucleotide	1	AT_Untreated_1_4	AT_Untreated_1	0.5	Deoxy-nucleosides	False			-5.917	-1.791
4	GlcNAc 6-P	Carbon	1	AT_Untreated_1_4	AT_Untreated_1	1.0	Aminosugar biosynthesis	True	3.161e+06	1.28	1.28	0.9509
337	Xanthosine	Nucleotide	1	AT_Untreated_1_4	AT_Untreated_1	1.0	Purine nucleosides	True	2.414e+05	-2.431	-2.431	0.643
188	Kynurenine	Amino acid	1	AT_Untreated_1_4	AT_Untreated_1	1.0	Amino acid derivatives	True	1.958e+06	0.5885	0.5885	1.28

Metabolite ID	Name	Super Pathway	Dataset	Sample ID	Group ID	Detection Fraction	Pathway	Detected	Raw Intensity	Log2 Norm Intensity	Norm Imputed	Log2 Ctrl Norm
63	6-P-Gluconate	Carbon	1	AT_Untreated_1_4	AT_Untreated_1	1.0	Pentose phosphate pathway (PPP)	True	3.368e+05	-1.951	-1.951	-3.984
40	Glucuronate	Carbon	1	AT_Untreated_1_4	AT_Untreated_1	1.0	Sugars and sugar alcohols	True	3.748e+05	-1.797	-1.797	-0.2576
108	Argininosuccinate	Amino acid	1	AT_Untreated_1_4	AT_Untreated_1	1.0	Amino acids biosynthesis intermediates	True	9.15e+05	-0.509	-0.509	-0.2829
710	Carnitine	Cofactor	1	AT_Untreated_1_4	AT_Untreated_1	1.0	Cofactors	True	9.782e+07	6.231	6.231	0.2536
725	P-Pantetheine	Cofactor	1	AT_Untreated_1_4	AT_Untreated_1	1.0	Coenzyme A biosynthesis	True	4.854e+05	-1.424	-1.424	2.281
48	DHAP	Carbon	1	AT_Untreated_1_4	AT_Untreated_1	1.0	Glycolysis, GNG	True	7.493e+06	2.525	2.525	-0.572
17	Maltose	Carbon	1	AT_Untreated_1_4	AT_Untreated_1	1.0	Glycogen degradation	True	2.3e+06	0.8207	0.8207	2.113
359	N1-Me-Adenosine	Nucleotide	1	AT_Untreated_1_4	AT_Untreated_1	0.5	Purine derivatives in RNAs	True	5.625e+05	-1.211	-1.211	-0.1732
159	3-Me-His	Amino acid	1	AT_Untreated_1_4	AT_Untreated_1	1.0	Amino acid derivatives	True	1.351e+05	-3.269	-3.269	0.9663
155	4-Guanidinobutanoate	Amino acid	1	AT_Untreated_1_4	AT_Untreated_1	1.0	Amino acid derivatives	True	1.53e+06	0.2322	0.2322	1.666
164	5-OH-Lys	Amino acid	1	AT_Untreated_1_4	AT_Untreated_1	1.0	Amino acid derivatives	True	3.945e+05	-1.723	-1.723	0.04746
357	Adenosine-3',5'-PP	Nucleotide	1	AT_Untreated_1_4	AT_Untreated_1	0.666666666666667	Purine byproducts of metabolic processes	True	1.324e+05	-3.298	-3.298	0.1792
104	Cystathionine	Amino acid	1	AT_Untreated_1_4	AT_Untreated_1	1.0	Amino acids biosynthesis intermediates	True	3.16e+06	1.279	1.279	-0.9303
113	Imidazole Lactate	Amino acid	1	AT_Untreated_1_4	AT_Untreated_1	0.666666666666667	Amino acids degradation intermediates	False			-4.646	-0.9699
215	N-Ac-Glu	Amino acid	1	AT_Untreated_1_4	AT_Untreated_1	1.0	N-acetylated amino acids	True	1.837e+06	0.4961	0.4961	-1.247
310	S-Lactoyl-Glutathione	Amino acid	1	AT_Untreated_1_4	AT_Untreated_1	1.0	Glutathione derivatives	True	6.913e+06	2.408	2.408	2.052
5	GlcNAc 1-P	Carbon	1	AT_Untreated_1_4	AT_Untreated_1	0.833333333333333	Aminosugar biosynthesis	True	4.297e+05	-1.599	-1.599	0.3114
34	Ribitol	Carbon	1	AT_Untreated_1_4	AT_Untreated_1	1.0	Sugars and sugar alcohols	True	2.373e+05	-2.456	-2.456	0.9737
10	UDP-Galactose	Carbon	1	AT_Untreated_1_4	AT_Untreated_1	0.833333333333333	Polysaccharide biosynthesis	True	4.76e+05	-1.452	-1.452	-0.8254
13	Guanosine 5'-PP-Fucose	Carbon	1	AT_Untreated_1_4	AT_Untreated_1	1.0	Polysaccharide biosynthesis	True	7.082e+04	-4.201	-4.201	-1.901

Metabolite ID	Name	Super Pathway	Dataset	Sample ID	Group ID	Detection Fraction	Pathway	Detected	Raw Intensity	Log2 Norm Intensity	Norm Imputed	Log2 Ctrl Norm
19	Maltotetraose	Carbon	1	AT_Untreated_1_4	AT_Untreated_1	1.0	Glycogen degradation	True	1.812e+07	3.798	3.798	3.842
233	SAM	Amino acid	1	AT_Untreated_1_4	AT_Untreated_1	0.333333333333333	SAM metabolism	False			-2.121	-1.965
129	5-Aminovalerate	Amino acid	1	AT_Untreated_1_4	AT_Untreated_1	0.333333333333333	Amino acids degradation intermediates	False			0.04171	-0.96
741	5-Me-THF	Cofactor	1	AT_Untreated_1_4	AT_Untreated_1	0.333333333333333	Folate metabolism	False			-5.366	-0.6085
198	Indolelactate	Amino acid	1	AT_Untreated_1_4	AT_Untreated_1	1.0	Amino acid derivatives	True	3.413e+05	-1.932	-1.932	1.855
254	Gly-Val	Amino acid	1	AT_Untreated_1_4	AT_Untreated_1	1.0	Dipeptides	True	1.779e+06	0.4499	0.4499	-0.6614
291	gamma-Glu-Leu	Amino acid	1	AT_Untreated_1_4	AT_Untreated_1	1.0	Gamma-glutamyl dipeptides	True	6.231e+05	-1.063	-1.063	-0.5746
173	Met Sulfoxide	Amino acid	1	AT_Untreated_1_4	AT_Untreated_1	1.0	Amino acid derivatives	True	8.372e+06	2.685	2.685	0.1173
43	Glucose	Carbon	1	AT_Untreated_1_4	AT_Untreated_1	1.0	Glycolysis, GNG	True	1.373e+08	6.72	6.72	1.668
185	Phenyllactate	Amino acid	1	AT_Untreated_1_4	AT_Untreated_1	0.833333333333333	Amino acid derivatives	True	8.428e+04	-3.95	-3.95	1.862
156	Homo-Arg	Amino acid	1	AT_Untreated_1_4	AT_Untreated_1	1.0	Amino acid derivatives	True	5.049e+06	1.955	1.955	0.7746
135	Homocitrulline	Amino acid	1	AT_Untreated_1_4	AT_Untreated_1	1.0	Amino acids degradation intermediates	True	2.374e+05	-2.456	-2.456	-0.753
719	Nicotinamide MN	Cofactor	1	AT_Untreated_1_4	AT_Untreated_1	1.0	NAD biosynthesis	True	2.469e+06	0.9228	0.9228	0.7372
212	N-Ac-Asp	Amino acid	1	AT_Untreated_1_4	AT_Untreated_1	1.0	N-acetylated amino acids	True	7.37e+06	2.501	2.501	1.803
720	1-Me-Nicotinamide	Cofactor	1	AT_Untreated_1_4	AT_Untreated_1	1.0	Derivatives of NA, nicotinamide and NAD	True	3.933e+08	8.239	8.239	0.348
216	N-Ac-Gly	Amino acid	1	AT_Untreated_1_4	AT_Untreated_1	0.5	N-acetylated amino acids	True	2.254e+05	-2.53	-2.53	1.01
70	Creatine	Carbon	1	AT_Untreated_1_4	AT_Untreated_1	1.0	Creatine energy storage	True	6.322e+08	8.923	8.923	-0.4939
26	Galactonate	Carbon	1	AT_Untreated_1_4	AT_Untreated_1	0.666666666666667	Sugars and sugar alcohols	True	1.111e+06	-0.2286	-0.2286	0.576
309	Glutathione, Oxidized	Amino acid	1	AT_Untreated_1_4	AT_Untreated_1	1.0	Glutathione	True	8.864e+06	2.767	2.767	1.574
35	Ribonate	Carbon	1	AT_Untreated_1_4	AT_Untreated_1	0.833333333333333	Sugars and sugar alcohols	False			-1.435	-0.9074
160	1-Me-His	Amino acid	1	AT_Untreated_1_4	AT_Untreated_1	1.0	Amino acid derivatives	True	2.433e+07	4.224	4.224	0.7825

Metabolite ID	Name	Super Pathway	Dataset	Sample ID	Group ID	Detection Fraction	Pathway	Detected	Raw Intensity	Log2 Norm Intensity	Norm Imputed	Log2 Ctrl Norm
44	Glucose 6-P	Carbon	1	AT_Untreated_1_4	AT_Untreated_1	0.833333333333333	Glycolysis, GNG	True	1.215e+05	-3.422	-3.422	-1.946
704	NADH	Cofactor	1	AT_Untreated_1_4	AT_Untreated_1	1.0	Cofactors	True	2.581e+05	-2.335	-2.335	-1.689
275	Thr-Phe	Amino acid	1	AT_Untreated_1_4	AT_Untreated_1	0.833333333333333	Dipeptides	False			-3.909	-2.255
738	Pyridoxate	Cofactor	1	AT_Untreated_1_4	AT_Untreated_1	1.0	PLP biosynthesis and salvage	True	1.615e+05	-3.011	-3.011	0.3532
177	3-(4-OH-Phenyl)Lactate	Amino acid	1	AT_Untreated_1_4	AT_Untreated_1	1.0	Amino acid derivatives	True	4.728e+05	-1.462	-1.462	1.004
206	Trans-4-OH-Pro	Amino acid	1	AT_Untreated_1_4	AT_Untreated_1	1.0	Amino acid derivatives	True	3.903e+07	4.905	4.905	-0.2497
329	AMP	Nucleotide	1	AT_Untreated_1_4	AT_Untreated_1	1.0	Purine nucleotides	True	8.463e+05	-0.6216	-0.6216	-4.257
11	UDP-Glucose	Carbon	1	AT_Untreated_1_4	AT_Untreated_1	0.833333333333333	Polysaccharide biosynthesis	True	7.964e+05	-0.7094	-0.7094	-0.6042
158	4-Imidazole-Ac	Amino acid	1	AT_Untreated_1_4	AT_Untreated_1	1.0	Amino acid derivatives	True	3.414e+05	-1.931	-1.931	0.2154
111	1-Me-Imidazole-Ac	Amino acid	1	AT_Untreated_1_4	AT_Untreated_1	1.0	Amino acids degradation intermediates	True	8.593e+05	-0.5996	-0.5996	1.334
345	Guanine	Nucleotide	1	AT_Untreated_1_4	AT_Untreated_1	1.0	Purine bases	True	1.106e+08	6.408	6.408	1.421
22	N-Ac-Neuraminate	Carbon	1	AT_Untreated_1_4	AT_Untreated_1	1.0	Aminosugar derivatives	True	1.204e+06	-0.1132	-0.1132	0.02216
721	N'-Methylnicotinate	Cofactor	1	AT_Untreated_1_4	AT_Untreated_1	1.0	Derivatives of NA, nicotinamide and NAD	True	7.63e+05	-0.7711	-0.7711	0.2365
183	Phenol Sulfate	Amino acid	1	AT_Untreated_1_4	AT_Untreated_1	1.0	Amino acid derivatives	True	8.603e+04	-3.92	-3.92	0.7202
718	Nicotinamide Riboside	Cofactor	1	AT_Untreated_1_4	AT_Untreated_1	1.0	NAD biosynthesis	True	5.794e+07	5.476	5.476	3.886
297	gamma-Glu-Thr	Amino acid	1	AT_Untreated_1_4	AT_Untreated_1	1.0	Gamma-glutamyl dipeptides	True	9.997e+05	-0.3814	-0.3814	-1.022
295	gamma-Glu-Phe	Amino acid	1	AT_Untreated_1_4	AT_Untreated_1	0.166666666666667	Gamma-glutamyl dipeptides	False			-5.975	-0.1899
347	Allantoic Acid	Nucleotide	1	AT_Untreated_1_4	AT_Untreated_1	0.666666666666667	Purine degradation	True	8.268e+04	-3.977	-3.977	1.662
399	Pseudouridine	Nucleotide	1	AT_Untreated_1_4	AT_Untreated_1	1.0	Pyrimidine derivatives in RNAs	True	7.757e+05	-0.7474	-0.7474	1.283
375	UTP	Nucleotide	1	AT_Untreated_1_4	AT_Untreated_1	0.666666666666667	Pyrimidine nucleotides	False			-5.025	-3.049
144	Glu, gamma-Me Ester	Amino acid	1	AT_Untreated_1_4	AT_Untreated_1	1.0	Amino acid derivatives	True	7.152e+05	-0.8645	-0.8645	-1.442

Metabolite ID	Name	Super Pathway	Dataset	Sample ID	Group ID	Detection Fraction	Pathway	Detected	Raw Intensity	Log2 Norm Intensity	Norm Imputed	Log2 Ctrl Norm
292	gamma-Glu-epsilon-Lysine	Amino acid	1	AT_Untreated_1_4	AT_Untreated_1	1.0	Gamma-glutamyl dipeptides	True	1.225e+06	-0.08767	-0.08767	0.1037
225	N-Ac-Thr	Amino acid	1	AT_Untreated_1_4	AT_Untreated_1	1.0	N-acetylated amino acids	True	3.027e+05	-2.105	-2.105	-0.6025
211	N-Ac-Asn	Amino acid	1	AT_Untreated_1_4	AT_Untreated_1	1.0	N-acetylated amino acids	True	1.767e+05	-2.882	-2.882	-0.1966
151	Phenylacetylglycine	Amino acid	1	AT_Untreated_1_4	AT_Untreated_1	1.0	Amino acid derivatives	True	1.673e+06	0.3618	0.3618	1.149
217	N-Ac-His	Amino acid	1	AT_Untreated_1_4	AT_Untreated_1	0.833333333333333	N-acetylated amino acids	True	1.381e+05	-3.238	-3.238	0.2473
288	gamma-Glu-Gly	Amino acid	1	AT_Untreated_1_4	AT_Untreated_1	0.333333333333333	Gamma-glutamyl dipeptides	True	4.026e+05	-1.693	-1.693	0.1311
222	N-Ac-Phe	Amino acid	1	AT_Untreated_1_4	AT_Untreated_1	0.333333333333333	N-acetylated amino acids	True	2.932e+04	-5.473	-5.473	0.7998
71	Creatine-P	Carbon	1	AT_Untreated_1_4	AT_Untreated_1	1.0	Creatine energy storage	True	7.168e+05	-0.8613	-0.8613	3.394
210	N-Ac-Arg	Amino acid	1	AT_Untreated_1_4	AT_Untreated_1	0.666666666666667	N-acetylated amino acids	True	2.416e+05	-2.43	-2.43	0.2452
218	N-Ac-Ile	Amino acid	1	AT_Untreated_1_4	AT_Untreated_1	0.166666666666667	N-acetylated amino acids	False			-6.52	-0.5541
251	Gly-Leu	Amino acid	1	AT_Untreated_1_4	AT_Untreated_1	1.0	Dipeptides	True	9.898e+05	-0.3958	-0.3958	-2.034
290	gamma-Glu-Ile	Amino acid	1	AT_Untreated_1_4	AT_Untreated_1	1.0	Gamma-glutamyl dipeptides	True	4.638e+05	-1.489	-1.489	-0.1508
316	Ophthalmate	Amino acid	1	AT_Untreated_1_4	AT_Untreated_1	1.0	Oxidative stress markers	True	2.938e+06	1.174	1.174	-1.169
125	Isovaleryl-Gly	Amino acid	1	AT_Untreated_1_4	AT_Untreated_1	1.0	Amino acids degradation intermediates	True	6.048e+04	-4.428	-4.428	0.9811
368	7-Me-Guanine	Nucleotide	1	AT_Untreated_1_4	AT_Untreated_1	1.0	Purine derivatives in RNAs	True	3.967e+05	-1.715	-1.715	0.4751
208	Pro-OH-Pro	Amino acid	1	AT_Untreated_1_4	AT_Untreated_1	1.0	Amino acid derivatives	True	1.777e+07	3.77	3.77	0.4411
366	N2,N2-DiMe-Guanosine	Nucleotide	1	AT_Untreated_1_4	AT_Untreated_1	0.833333333333333	Purine derivatives in RNAs	True	9.554e+04	-3.769	-3.769	0.3449
352	3'-AMP	Nucleotide	1	AT_Untreated_1_4	AT_Untreated_1	1.0	Purine derivatives in signaling	True	2.624e+05	-2.311	-2.311	-0.8245
363	N6-Carbamoyl-Thr-Adenosine	Nucleotide	1	AT_Untreated_1_4	AT_Untreated_1	0.666666666666667	Purine derivatives in RNAs	True	6.463e+04	-4.333	-4.333	0.3529
314	Cys-Glutathione Disulfide	Amino acid	1	AT_Untreated_1_4	AT_Untreated_1	1.0	Oxidative stress markers	True	9.628e+04	-3.758	-3.758	-0.4735
382	Orotidine	Nucleotide	1	AT_Untreated_1_4	AT_Untreated_1	0.166666666666667	Pyrimidine (UMP) biosynthesis	True	9.011e+04	-3.853	-3.853	0.4388

Metabolite ID	Name	Super Pathway	Dataset	Sample ID	Group ID	Detection Fraction	Pathway	Detected	Raw Intensity	Log2 Norm Intensity	Norm Imputed	Log2 Ctrl Norm
307	Cys-Gly	Amino acid	1	AT_Untreated_1_4	AT_Untreated_1	1.0	Glutathione biosynthesis	True	1.682e+06	0.3692	0.3692	-1.238
64	Sedoheptulose-7-P	Carbon	1	AT_Untreated_1_4	AT_Untreated_1	1.0	Pentose phosphate pathway (PPP)	True	1.3e+06	-2.77e-03	-2.77e-03	-0.1569
142	N-Ac-Asp-Glu	Amino acid	1	AT_Untreated_1_4	AT_Untreated_1	1.0	Amino acid derivatives	True	6.99e+05	-0.8976	-0.8976	0.2478
708	Thiamin-PP	Cofactor	1	AT_Untreated_1_4	AT_Untreated_1	0.5	Cofactors	True	3.854e+04	-5.078	-5.078	0.9051
182	P-Cresol Sulfate	Amino acid	1	AT_Untreated_1_4	AT_Untreated_1	1.0	Amino acid derivatives	True	4.32e+05	-1.592	-1.592	1.604
250	Gly-Ile	Amino acid	1	AT_Untreated_1_4	AT_Untreated_1	1.0	Dipeptides	True	2.746e+05	-2.246	-2.246	-0.9284
286	gamma-Glu-Glu	Amino acid	1	AT_Untreated_1_4	AT_Untreated_1	1.0	Gamma-glutamyl dipeptides	True	1.996e+06	0.616	0.616	0.5982
739	Deoxycarnitine	Cofactor	1	AT_Untreated_1_4	AT_Untreated_1	1.0	Carnitine biosynthesis	True	1.642e+07	3.656	3.656	-0.0948
203	DiMe-Arg	Amino acid	1	AT_Untreated_1_4	AT_Untreated_1	1.0	Amino acid derivatives	True	7.258e+07	5.801	5.801	0.168
351	2'-AMP	Nucleotide	1	AT_Untreated_1_4	AT_Untreated_1	1.0	Purine derivatives in signaling	True	1.28e+06	-0.02426	-0.02426	2.244
8	Cytidine 5'-P-N-Ac-Neuraminate	Carbon	1	AT_Untreated_1_4	AT_Untreated_1	1.0	Aminosugar biosynthesis	True	3.969e+05	-1.714	-1.714	0.03136
285	gamma-Glu-Ala	Amino acid	1	AT_Untreated_1_4	AT_Untreated_1	0.5	Gamma-glutamyl dipeptides	False			-6.022	-2.783
224	N-Ac-Ser	Amino acid	1	AT_Untreated_1_4	AT_Untreated_1	1.0	N-acetylated amino acids	True	4.472e+06	1.78	1.78	-0.09927
244	Ala-Leu	Amino acid	1	AT_Untreated_1_4	AT_Untreated_1	1.0	Dipeptides	True	1.02e+05	-3.674	-3.674	-4.812
207	N-Me-Pro	Amino acid	1	AT_Untreated_1_4	AT_Untreated_1	1.0	Amino acid derivatives	True	1.353e+06	0.05527	0.05527	0.9853
171	Cys Sulfinic Acid	Amino acid	1	AT_Untreated_1_4	AT_Untreated_1	0.333333333333333	Amino acid derivatives	False			-5.211	-1.913
181	O-Me-Tyr	Amino acid	1	AT_Untreated_1_4	AT_Untreated_1	0.5	Amino acid derivatives	True	8.773e+04	-3.892	-3.892	-0.5218
240	N-Ac-Putrescine	Amino acid	1	AT_Untreated_1_4	AT_Untreated_1	0.833333333333333	Polyamine derivatives	True	2.475e+04	-5.717	-5.717	-3.353
176	S-Me-Met	Amino acid	1	AT_Untreated_1_4	AT_Untreated_1	1.0	Amino acid derivatives	True	7.057e+05	-0.8838	-0.8838	0.9579
339	AICAR	Nucleotide	1	AT_Untreated_1_4	AT_Untreated_1	0.333333333333333	IMP biosynthesis	False			-4.716	-0.4151
141	gamma-Carboxy-Glu	Amino acid	1	AT_Untreated_1_4	AT_Untreated_1	1.0	Amino acid derivatives	True	7.663e+05	-0.7649	-0.7649	0.02007
392	3'-UMP	Nucleotide	1	AT_Untreated_1_4	AT_Untreated_1	0.833333333333333	Pyrimidine derivatives in signaling	True	3.525e+04	-5.207	-5.207	-1.174

Metabolite ID	Name	Super Pathway	Dataset	Sample ID	Group ID	Detection Fraction	Pathway	Detected	Raw Intensity	Log2 Norm Intensity	Norm Imputed	Log2 Ctrl Norm
355	3'-GMP	Nucleotide	1	AT_Untreated_1_4	AT_Untreated_1	0.833333333333333	Purine derivatives in signaling	False			-5.834	-1.897
282	Val-Leu	Amino acid	1	AT_Untreated_1_4	AT_Untreated_1	1.0	Dipeptides	True	1.83e+05	-2.831	-2.831	-3.803
140	Carboxyethyl-GABA	Amino acid	1	AT_Untreated_1_4	AT_Untreated_1	1.0	Amino acid derivatives	True	3.544e+05	-1.877	-1.877	-0.4463
258	Ile-Gly	Amino acid	1	AT_Untreated_1_4	AT_Untreated_1	1.0	Dipeptides	True	2.101e+06	0.6902	0.6902	-2.622
260	Leu-Ala	Amino acid	1	AT_Untreated_1_4	AT_Untreated_1	1.0	Dipeptides	True	2.491e+05	-2.386	-2.386	-3.687
265	Lys-Leu	Amino acid	1	AT_Untreated_1_4	AT_Untreated_1	0.833333333333333	Dipeptides	False			-3.814	-1.203
263	Leu-Gly	Amino acid	1	AT_Untreated_1_4	AT_Untreated_1	1.0	Dipeptides	True	6.323e+05	-1.042	-1.042	-4.064
281	Val-Gly	Amino acid	1	AT_Untreated_1_4	AT_Untreated_1	1.0	Dipeptides	True	3.215e+06	1.304	1.304	-3.215
270	Pro-Gly	Amino acid	1	AT_Untreated_1_4	AT_Untreated_1	1.0	Dipeptides	True	7.562e+06	2.538	2.538	1.791
114	Imidazole Propionate	Amino acid	1	AT_Untreated_1_4	AT_Untreated_1	1.0	Amino acids degradation intermediates	True	5.32e+05	-1.292	-1.292	1.019
267	Phe-Gly	Amino acid	1	AT_Untreated_1_4	AT_Untreated_1	1.0	Dipeptides	True	3.802e+06	1.546	1.546	-2.548
266	Phe-Ala	Amino acid	1	AT_Untreated_1_4	AT_Untreated_1	1.0	Dipeptides	True	3.614e+05	-1.849	-1.849	-2.926
278	Tyr-Gly	Amino acid	1	AT_Untreated_1_4	AT_Untreated_1	1.0	Dipeptides	True	1.584e+06	0.2827	0.2827	-1.9
255	His-Ala	Amino acid	1	AT_Untreated_1_4	AT_Untreated_1	0.833333333333333	Dipeptides	False			-2.805	-1.468
280	Val-Gln	Amino acid	1	AT_Untreated_1_4	AT_Untreated_1	1.0	Dipeptides	True	2.444e+05	-2.413	-2.413	-4.031
143	S-1-Pyrroline-5-Carboxylate	Amino acid	1	AT_Untreated_1_4	AT_Untreated_1	0.833333333333333	Amino acid derivatives	True	3.863e+05	-1.753	-1.753	0.3151
232	SAH	Amino acid	1	AT_Untreated_1_4	AT_Untreated_1	1.0	SAM metabolism	True	8.02e+05	-0.6992	-0.6992	-0.2138
20	Erythronate	Carbon	1	AT_Untreated_1_4	AT_Untreated_1	1.0	Aminosugar derivatives	True	3.285e+07	4.657	4.657	0.9156
248	Gln-Leu	Amino acid	1	AT_Untreated_1_4	AT_Untreated_1	0.833333333333333	Dipeptides	False			-2.935	-2.03
276	Trp-Gly	Amino acid	1	AT_Untreated_1_4	AT_Untreated_1	0.833333333333333	Dipeptides	False			-3.017	-1.938
205	N-delta-Ac-Ornithine	Amino acid	1	AT_Untreated_1_4	AT_Untreated_1	1.0	Amino acid derivatives	True	4.365e+05	-1.577	-1.577	-0.4559
163	Formimino-Glu	Amino acid	1	AT_Untreated_1_4	AT_Untreated_1	1.0	Amino acid derivatives	True	8.085e+05	-0.6875	-0.6875	0.5523
204	N-Me-Arg	Amino acid	1	AT_Untreated_1_4	AT_Untreated_1	0.833333333333333	Amino acid derivatives	True	1.375e+07	3.401	3.401	0.8135
242	Guanidino-Ac	Amino acid	1	AT_Untreated_1_4	AT_Untreated_1	1.0	Creatine biosynthesis	True	3.667e+05	-1.828	-1.828	2.052

Metabolite ID	Name	Super Pathway	Dataset	Sample ID	Group ID	Detection Fraction	Pathway	Detected	Raw Intensity	Log2 Norm Intensity	Norm Imputed	Log2 Ctrl Norm
300	gamma-Glu-Val	Amino acid	1	AT_Untreated_1_4	AT_Untreated_1	0.333333333333333	Gamma-glutamyl dipeptides	True	2.132e+06	0.7115	0.7115	0.09075
53	Ac-CoA	Carbon	1	AT_Untreated_1_4	AT_Untreated_1	0.166666666666667	Acetyl-CoA	False			-7.274	-0.5114
18	Maltotriose	Carbon	1	AT_Untreated_1_4	AT_Untreated_1	1.0	Glycogen degradation	True	1.719e+07	3.722	3.722	3.88
294	gamma-Glu-Met	Amino acid	1	AT_Untreated_1_4	AT_Untreated_1	0.333333333333333	Gamma-glutamyl dipeptides	False			-4.503	-1.021
174	Met Sulfone	Amino acid	1	AT_Untreated_1_4	AT_Untreated_1	0.833333333333333	Amino acid derivatives	True	2.079e+05	-2.647	-2.647	0.4316
175	N-Ac-Met Sulfoxide	Amino acid	1	AT_Untreated_1_4	AT_Untreated_1	1.0	Amino acid derivatives	True	8.91e+05	-0.5474	-0.5474	-0.5238
25	Mannitol/Sorbitol	Carbon	1	AT_Untreated_1_4	AT_Untreated_1	1.0	Sugars and sugar alcohols	True	1.514e+07	3.539	3.539	1.315
6	UDP-GlcNAc	Carbon	1	AT_Untreated_1_4	AT_Untreated_1	0.166666666666667	Aminosugar biosynthesis	False			-3.589	-0.4814
145	Pyro-Gln	Amino acid	1	AT_Untreated_1_4	AT_Untreated_1	1.0	Amino acid derivatives	True	3.206e+06	1.3	1.3	0.4601
705	Coenzyme A	Cofactor	1	AT_Untreated_1_4	AT_Untreated_1	0.666666666666667	Cofactors	True	1.183e+05	-3.461	-3.461	0.4983
319	2'-dAMP	Nucleotide	1	AT_Untreated_1_4	AT_Untreated_1	0.166666666666667	Deoxy-nucleotides	False			-4.337	-0.6568
119	alpha-OH-Isovalerate	Amino acid	1	AT_Untreated_1_4	AT_Untreated_1	1.0	Amino acids degradation intermediates	True	5.915e+05	-1.138	-1.138	2.16
46	Fructose 1,6-PP / Glucose 1,6-PP / Inositol-1,4-PP / Inositol-1,3-PP	Carbon	1	AT_Untreated_1_4	AT_Untreated_1	1.0	Glycolysis, GNG	True	6.769e+06	2.378	2.378	-2.453
137	1-Me-Guanidine	Amino acid	1	AT_Untreated_1_4	AT_Untreated_1	0.666666666666667	Amino acids degradation intermediates	False			-5.942	-0.7895
23	N-GlcNAc-Asn	Carbon	1	AT_Untreated_1_4	AT_Untreated_1	1.0	Aminosugar derivatives	True	1.212e+06	-0.1031	-0.1031	-0.8161
262	Leu-Gln	Amino acid	1	AT_Untreated_1_4	AT_Untreated_1	1.0	Dipeptides	True	3.933e+05	-1.727	-1.727	-3.024
24	Fructose	Carbon	1	AT_Untreated_1_4	AT_Untreated_1	1.0	Sugars and sugar alcohols	True	1.849e+07	3.828	3.828	1.879
197	C-Glycosyl-Trp	Amino acid	1	AT_Untreated_1_4	AT_Untreated_1	1.0	Amino acid derivatives	True	1.333e+06	0.03364	0.03364	0.3327
33	Arabitol/Xylitol	Carbon	1	AT_Untreated_1_4	AT_Untreated_1	1.0	Sugars and sugar alcohols	True	3.971e+05	-1.713	-1.713	0.1465

Metabolite ID	Name	Super Pathway	Dataset	Sample ID	Group ID	Detection Fraction	Pathway	Detected	Raw Intensity	Log2 Norm Intensity	Norm Imputed	Log2 Ctrl Norm
128	N2-Ac-Lys/N6-Ac-Lys	Amino acid	1	AT_Untreated_1_4	AT_Untreated_1	1.0	Amino acids degradation intermediates	True	2.154e+06	0.7263	0.7263	-0.4841
42	2-Me-Citrate	Carbon	1	AT_Untreated_1_4	AT_Untreated_1	0.833333333333333	Propionate metabolism	True	1.311e+05	-3.312	-3.312	1.071
12	Glucuronate 1-P	Carbon	1	AT_Untreated_1_4	AT_Untreated_1	1.0	Polysaccharide biosynthesis	True	4.693e+05	-1.472	-1.472	-0.2296
76	Gln	Amino acid	1	AT_Untreated_1_5	AT_Untreated_1	1.0	Proteinogenic amino acids	True	2.994e+08	8.084	8.084	-0.5961
89	Trp	Amino acid	1	AT_Untreated_1_5	AT_Untreated_1	1.0	Proteinogenic amino acids	True	3.973e+07	5.17	5.17	-0.5054
723	beta-Ala	Cofactor	1	AT_Untreated_1_5	AT_Untreated_1	1.0	Coenzyme A biosynthesis	True	5.243e+06	2.249	2.249	-0.7605
75	Glu	Amino acid	1	AT_Untreated_1_5	AT_Untreated_1	1.0	Proteinogenic amino acids	True	1.371e+09	10.28	10.28	-0.4576
77	Gly	Amino acid	1	AT_Untreated_1_5	AT_Untreated_1	1.0	Proteinogenic amino acids	True	2.701e+07	4.614	4.614	-1.002
80	His	Amino acid	1	AT_Untreated_1_5	AT_Untreated_1	1.0	Proteinogenic amino acids	True	9.856e+06	3.159	3.159	-0.2558
82	Leu	Amino acid	1	AT_Untreated_1_5	AT_Untreated_1	1.0	Proteinogenic amino acids	True	6.001e+08	9.087	9.087	-0.1338
87	Phe	Amino acid	1	AT_Untreated_1_5	AT_Untreated_1	1.0	Proteinogenic amino acids	True	3.76e+08	8.413	8.413	-0.3985
130	Glutarate	Amino acid	1	AT_Untreated_1_5	AT_Untreated_1	1.0	Amino acids degradation intermediates	True	2.485e+05	-2.15	-2.15	0.2763
196	5-OH-Indole-Ac	Amino acid	1	AT_Untreated_1_5	AT_Untreated_1	1.0	Amino acid derivatives	True	1.578e+05	-2.806	-2.806	1.206
74	Asp	Amino acid	1	AT_Untreated_1_5	AT_Untreated_1	1.0	Proteinogenic amino acids	True	1.648e+08	7.223	7.223	-0.6686
236	Spermidine	Amino acid	1	AT_Untreated_1_5	AT_Untreated_1	1.0	Polyamines	True	1.125e+07	3.35	3.35	-0.5118
73	Asn	Amino acid	1	AT_Untreated_1_5	AT_Untreated_1	1.0	Proteinogenic amino acids	True	7.617e+07	6.109	6.109	-0.5616
243	Creatinine	Amino acid	1	AT_Untreated_1_5	AT_Untreated_1	1.0	Creatine degradation	True	5.425e+07	5.62	5.62	0.6176
376	Cytidine	Nucleotide	1	AT_Untreated_1_5	AT_Untreated_1	0.833333333333333	Pyrimidine nucleosides	True	9.395e+06	3.09	3.09	2.363
41	Lactate	Carbon	1	AT_Untreated_1_5	AT_Untreated_1	1.0	Respiratory carbon sources	True	4.607e+07	5.384	5.384	-1.098
58	alpha-Ketoglutarate	Carbon	1	AT_Untreated_1_5	AT_Untreated_1	1.0	TCA cycle	True	1.281e+06	0.2153	0.2153	0.1986
69	3-OH-Butyrate	Carbon	1	AT_Untreated_1_5	AT_Untreated_1	0.833333333333333	Ketone bodies	True	4.649e+05	-1.247	-1.247	1.738

Metabolite ID	Name	Super Pathway	Dataset	Sample ID	Group ID	Detection Fraction	Pathway	Detected	Raw Intensity	Log2 Norm Intensity	Norm Imputed	Log2 Ctrl Norm
343	Adenine	Nucleotide	1	AT_Untreated_1_5	AT_Untreated_1	1.0	Purine bases	True	9.508e+05	-0.2144	-0.2144	-1.671
336	Adenosine	Nucleotide	1	AT_Untreated_1_5	AT_Untreated_1	1.0	Purine nucleosides	True	1.174e+07	3.411	3.411	-0.01149
722	ADP-Ribose	Cofactor	1	AT_Untreated_1_5	AT_Untreated_1	1.0	Derivatives of NA, nicotinamide and NAD	True	5.555e+04	-4.312	-4.312	-1.31
383	Cytosine	Nucleotide	1	AT_Untreated_1_5	AT_Untreated_1	0.833333333333333	Pyrimidine bases	True	9.77e+04	-3.497	-3.497	1.358
3	Glucosamine 6-P	Carbon	1	AT_Untreated_1_5	AT_Untreated_1	0.833333333333333	Aminosugar biosynthesis	True	2.362e+05	-2.224	-2.224	-0.5564
717	Nicotinamide	Cofactor	1	AT_Untreated_1_5	AT_Untreated_1	1.0	NAD biosynthesis	True	1.713e+07	3.957	3.957	-0.5586
51	PEP	Carbon	1	AT_Untreated_1_5	AT_Untreated_1	1.0	Glycolysis, GNG	True	1.015e+06	-0.1208	-0.1208	-1.172
237	Spermine	Amino acid	1	AT_Untreated_1_5	AT_Untreated_1	1.0	Polyamines	True	1.772e+06	0.6833	0.6833	2.174
385	Uracil	Nucleotide	1	AT_Untreated_1_5	AT_Untreated_1	1.0	Pyrimidine bases	True	4.284e+06	1.957	1.957	1.693
377	Uridine	Nucleotide	1	AT_Untreated_1_5	AT_Untreated_1	1.0	Pyrimidine nucleosides	True	2.392e+07	4.439	4.439	0.3898
348	Allantoin	Nucleotide	1	AT_Untreated_1_5	AT_Untreated_1	1.0	Purine degradation	True	1.752e+06	0.6671	0.6671	0.8427
335	Inosine	Nucleotide	1	AT_Untreated_1_5	AT_Untreated_1	1.0	Purine nucleosides	True	2.961e+07	4.746	4.746	-0.7527
81	Ile	Amino acid	1	AT_Untreated_1_5	AT_Untreated_1	1.0	Proteinogenic amino acids	True	4.782e+08	8.76	8.76	0.04463
72	Ala	Amino acid	1	AT_Untreated_1_5	AT_Untreated_1	1.0	Proteinogenic amino acids	True	2.533e+08	7.843	7.843	-0.6581
79	Thr	Amino acid	1	AT_Untreated_1_5	AT_Untreated_1	1.0	Proteinogenic amino acids	True	1.855e+08	7.394	7.394	-0.6159
88	Tyr	Amino acid	1	AT_Untreated_1_5	AT_Untreated_1	1.0	Proteinogenic amino acids	True	1.845e+08	7.386	7.386	-0.1379
84	Lys	Amino acid	1	AT_Untreated_1_5	AT_Untreated_1	1.0	Proteinogenic amino acids	True	1.863e+08	7.4	7.4	-0.59
86	Met	Amino acid	1	AT_Untreated_1_5	AT_Untreated_1	1.0	Proteinogenic amino acids	True	2.269e+08	7.684	7.684	-0.5436
61	Malate	Carbon	1	AT_Untreated_1_5	AT_Untreated_1	1.0	TCA cycle	True	6.045e+07	5.776	5.776	-1.396
235	Putrescine	Amino acid	1	AT_Untreated_1_5	AT_Untreated_1	0.833333333333333	Polyamines	True	3.295e+05	-1.743	-1.743	-1.252
324	2'-dU	Nucleotide	1	AT_Untreated_1_5	AT_Untreated_1	0.666666666666667	Deoxy-nucleosides	True	7.142e+04	-3.949	-3.949	0.4312
49	3-P-Glycerate	Carbon	1	AT_Untreated_1_5	AT_Untreated_1	1.0	Glycolysis, GNG	True	4.926e+06	2.159	2.159	-1.107
189	Kynurenate	Amino acid	1	AT_Untreated_1_5	AT_Untreated_1	1.0	Amino acid derivatives	True	3.283e+04	-5.071	-5.071	0.7274
234	5-Me-Thioadenosine	Amino acid	1	AT_Untreated_1_5	AT_Untreated_1	1.0	SAM metabolism	True	1.864e+06	0.7564	0.7564	-1.129

Metabolite ID	Name	Super Pathway	Dataset	Sample ID	Group ID	Detection Fraction	Pathway	Detected	Raw Intensity	Log2 Norm Intensity	Norm Imputed	Log2 Ctrl Norm
59	Succinate	Carbon	1	AT_Untreated_1_5	AT_Untreated_1	1.0	TCA cycle	True	2.527e+06	1.196	1.196	0.8947
36	Ribose	Carbon	1	AT_Untreated_1_5	AT_Untreated_1	1.0	Sugars and sugar alcohols	True	1.819e+05	-2.6	-2.6	-1.367
133	Ornithine	Amino acid	1	AT_Untreated_1_5	AT_Untreated_1	1.0	Amino acids degradation intermediates	True	1.847e+07	4.065	4.065	-0.4141
313	5-Oxoproline	Amino acid	1	AT_Untreated_1_5	AT_Untreated_1	1.0	Glutathione derivatives	True	3.363e+06	1.608	1.608	-0.4792
165	N-6-Tri-Me-Lys	Amino acid	1	AT_Untreated_1_5	AT_Untreated_1	1.0	Amino acid derivatives	True	7.361e+06	2.738	2.738	-1.025
380	Orotate	Nucleotide	1	AT_Untreated_1_5	AT_Untreated_1	0.666666666666667	Pyrimidine (UMP) biosynthesis	False			-4.969	-1.339
724	Pantothenate	Cofactor	1	AT_Untreated_1_5	AT_Untreated_1	1.0	Coenzyme A biosynthesis	True	7.789e+07	6.142	6.142	-0.1685
150	N-Me-Gly	Amino acid	1	AT_Untreated_1_5	AT_Untreated_1	1.0	Amino acid derivatives	True	8.419e+05	-0.3899	-0.3899	-0.08946
122	3-OH-Isobutyrate	Amino acid	1	AT_Untreated_1_5	AT_Untreated_1	0.833333333333333	Amino acids degradation intermediates	True	2.808e+05	-1.974	-1.974	1.182
241	4-Acetamidobutanoate	Amino acid	1	AT_Untreated_1_5	AT_Untreated_1	1.0	Polyamine derivatives	True	3.003e+06	1.445	1.445	1.004
711	alpha-Tocopherol	Cofactor	1	AT_Untreated_1_5	AT_Untreated_1	1.0	Cofactors	True	1.183e+06	0.1011	0.1011	-0.6792
55	Citrate	Carbon	1	AT_Untreated_1_5	AT_Untreated_1	1.0	TCA cycle	True	9.568e+06	3.117	3.117	1.168
387	3-Aminoisobutyrate	Nucleotide	1	AT_Untreated_1_5	AT_Untreated_1	0.833333333333333	Pyrimidine degradation	False			-2.626	-0.6123
338	Guanosine	Nucleotide	1	AT_Untreated_1_5	AT_Untreated_1	1.0	Purine nucleosides	True	3.409e+07	4.95	4.95	-0.1563
209	N-Ac-Ala	Amino acid	1	AT_Untreated_1_5	AT_Untreated_1	1.0	N-acetylated amino acids	True	1.38e+05	-2.999	-2.999	-1.798
221	N-Ac-Met	Amino acid	1	AT_Untreated_1_5	AT_Untreated_1	1.0	N-acetylated amino acids	True	8.541e+05	-0.3692	-0.3692	-2.113
228	N-Ac-Val	Amino acid	1	AT_Untreated_1_5	AT_Untreated_1	0.333333333333333	N-acetylated amino acids	False			-5.82	-0.449
346	Urate	Nucleotide	1	AT_Untreated_1_5	AT_Untreated_1	1.0	Purine degradation	True	1.08e+06	-0.03058	-0.03058	1.032
90	Arg	Amino acid	1	AT_Untreated_1_5	AT_Untreated_1	1.0	Proteinogenic amino acids	True	1.081e+09	9.936	9.936	0.2307
60	Fumarate	Carbon	1	AT_Untreated_1_5	AT_Untreated_1	1.0	TCA cycle	True	1.308e+06	0.2459	0.2459	-0.7693
78	Ser	Amino acid	1	AT_Untreated_1_5	AT_Untreated_1	1.0	Proteinogenic amino acids	True	1.529e+08	7.115	7.115	-0.1428
83	Val	Amino acid	1	AT_Untreated_1_5	AT_Untreated_1	1.0	Proteinogenic amino acids	True	2.217e+08	7.651	7.651	-0.2216

Metabolite ID	Name	Super Pathway	Dataset	Sample ID	Group ID	Detection Fraction	Pathway	Detected	Raw Intensity	Log2 Norm Intensity	Norm Imputed	Log2 Ctrl Norm
734	Pyridoxal	Cofactor	1	AT_Untreated_1_5	AT_Untreated_1	1.0	PLP biosynthesis and salvage	True	5.478e+06	2.312	2.312	0.529
136	Urea	Amino acid	1	AT_Untreated_1_5	AT_Untreated_1	1.0	Amino acids degradation intermediates	True	2.409e+06	1.127	1.127	0.9755
67	Ribose 1-P	Carbon	1	AT_Untreated_1_5	AT_Untreated_1	1.0	Pentose phosphate pathway (PPP)	True	4.307e+06	1.965	1.965	1.344
284	Carnosine	Amino acid	1	AT_Untreated_1_5	AT_Untreated_1	1.0	Dipeptides	True	3.196e+05	-1.787	-1.787	0.3208
306	gamma-Glu-Cys	Amino acid	1	AT_Untreated_1_5	AT_Untreated_1	1.0	Glutathione biosynthesis	True	3.543e+05	-1.639	-1.639	-1.302
712	Retinol (Vit A)	Cofactor	1	AT_Untreated_1_5	AT_Untreated_1	0.833333333333333	Cofactors	False			-3.112	-0.6785
85	Cys	Amino acid	1	AT_Untreated_1_5	AT_Untreated_1	1.0	Proteinogenic amino acids	True	1.384e+07	3.649	3.649	-0.8642
91	Pro	Amino acid	1	AT_Untreated_1_5	AT_Untreated_1	1.0	Proteinogenic amino acids	True	5.001e+08	8.824	8.824	-0.5837
308	Glutathione, Reduced	Amino acid	1	AT_Untreated_1_5	AT_Untreated_1	1.0	Glutathione	True	6.574e+07	5.897	5.897	-1.059
107	Citrulline	Amino acid	1	AT_Untreated_1_5	AT_Untreated_1	1.0	Amino acids biosynthesis intermediates	True	1.147e+07	3.378	3.378	0.5531
328	IMP	Nucleotide	1	AT_Untreated_1_5	AT_Untreated_1	0.333333333333333	Purine nucleotides	False			-4.53	-0.2088
706	FAD	Cofactor	1	AT_Untreated_1_5	AT_Untreated_1	1.0	Cofactors	True	1.268e+05	-3.121	-3.121	-0.9319
735	Pyridoxamine	Cofactor	1	AT_Untreated_1_5	AT_Untreated_1	1.0	PLP biosynthesis and salvage	True	6.745e+05	-0.7098	-0.7098	-0.5168
199	Serotonin	Amino acid	1	AT_Untreated_1_5	AT_Untreated_1	1.0	Amino acid derivatives	True	3.617e+06	1.713	1.713	0.4403
370	CMP	Nucleotide	1	AT_Untreated_1_5	AT_Untreated_1	1.0	Pyrimidine nucleotides	True	2.141e+06	0.9569	0.9569	-0.8821
287	gamma-Glu-Gln	Amino acid	1	AT_Untreated_1_5	AT_Untreated_1	1.0	Gamma-glutamyl dipeptides	True	1.543e+06	0.484	0.484	-0.357
14	UDP-Glucuronate	Carbon	1	AT_Untreated_1_5	AT_Untreated_1	0.666666666666667	Polysaccharide biosynthesis	True	4.469e+05	-1.303	-1.303	0.6457
229	N-Formyl-Met	Amino acid	1	AT_Untreated_1_5	AT_Untreated_1	0.666666666666667	N-formylated amino acids	False			-5.516	-1.694
350	3',5'-cAMP	Nucleotide	1	AT_Untreated_1_5	AT_Untreated_1	0.666666666666667	Purine derivatives in signaling	False			-4.527	-0.9083
371	CDP	Nucleotide	1	AT_Untreated_1_5	AT_Untreated_1	0.666666666666667	Pyrimidine nucleotides	True	4.942e+04	-4.48	-4.48	-1.16
372	CTP	Nucleotide	1	AT_Untreated_1_5	AT_Untreated_1	0.333333333333333	Pyrimidine nucleotides	True	4.207e+04	-4.713	-4.713	-1.059

Metabolite ID	Name	Super Pathway	Dataset	Sample ID	Group ID	Detection Fraction	Pathway	Detected	Raw Intensity	Log2 Norm Intensity	Norm Imputed	Log2 Ctrl Norm
333	GDP	Nucleotide	1	AT_Untreated_1_5	AT_Untreated_1	0.5	Purine nucleotides	False			-4.484	-1.983
332	GMP	Nucleotide	1	AT_Untreated_1_5	AT_Untreated_1	1.0	Purine nucleotides	True	5.169e+05	-1.094	-1.094	-1.792
373	UMP	Nucleotide	1	AT_Untreated_1_5	AT_Untreated_1	0.833333333333333	Pyrimidine nucleotides	True	7.114e+04	-3.955	-3.955	-2.9
389	3'-CMP	Nucleotide	1	AT_Untreated_1_5	AT_Untreated_1	1.0	Pyrimidine derivatives in signaling	True	1.246e+06	0.1761	0.1761	1.227
330	ADP	Nucleotide	1	AT_Untreated_1_5	AT_Untreated_1	0.5	Purine nucleotides	False			-2.826	-2.53
342	Hypoxanthine	Nucleotide	1	AT_Untreated_1_5	AT_Untreated_1	1.0	Purine bases	True	1.34e+07	3.603	3.603	-0.3364
736	Pyridoxamine-P	Cofactor	1	AT_Untreated_1_5	AT_Untreated_1	0.833333333333333	PLP biosynthesis and salvage	False			-4.345	-1.367
148	Betaine	Amino acid	1	AT_Untreated_1_5	AT_Untreated_1	1.0	Amino acid derivatives	True	3.672e+07	5.057	5.057	0.251
344	Xanthine	Nucleotide	1	AT_Untreated_1_5	AT_Untreated_1	1.0	Purine bases	True	3.023e+06	1.455	1.455	0.547
386	3-Ureidopropionate	Nucleotide	1	AT_Untreated_1_5	AT_Untreated_1	1.0	Pyrimidine degradation	True	2.133e+05	-2.371	-2.371	-1.451
149	DiMe-Gly	Amino acid	1	AT_Untreated_1_5	AT_Untreated_1	1.0	Amino acid derivatives	True	1.301e+06	0.2384	0.2384	0.7587
703	NAD+	Cofactor	1	AT_Untreated_1_5	AT_Untreated_1	1.0	Cofactors	True	2.719e+06	1.301	1.301	-1.435
709	Pyridoxal-P	Cofactor	1	AT_Untreated_1_5	AT_Untreated_1	0.833333333333333	Cofactors	True	1.506e+05	-2.872	-2.872	-0.5645
731	Thiamin (Vitamin B1)	Cofactor	1	AT_Untreated_1_5	AT_Untreated_1	1.0	TPP biosynthesis	True	1.366e+06	0.3083	0.3083	-0.4394
374	UDP	Nucleotide	1	AT_Untreated_1_5	AT_Untreated_1	0.666666666666667	Pyrimidine nucleotides	True	1.127e+05	-3.292	-3.292	-1.738
102	2-Aminoadipate	Amino acid	1	AT_Untreated_1_5	AT_Untreated_1	0.833333333333333	Amino acids biosynthesis intermediates	False			-3.385	-1.276
45	Fructose-6-P	Carbon	1	AT_Untreated_1_5	AT_Untreated_1	1.0	Glycolysis, GNG	True	2.368e+05	-2.22	-2.22	-2.295
320	TMP	Nucleotide	1	AT_Untreated_1_5	AT_Untreated_1	0.5	Deoxy-nucleotides	False			-6.992	-2.2
341	XMP	Nucleotide	1	AT_Untreated_1_5	AT_Untreated_1	0.166666666666667	IMP conversion to AMP & GMP	False			-6.353	-1.778
120	beta-OH-Isovalerate	Amino acid	1	AT_Untreated_1_5	AT_Untreated_1	1.0	Amino acids degradation intermediates	True	1.444e+05	-2.933	-2.933	0.2323
322	2'-dI	Nucleotide	1	AT_Untreated_1_5	AT_Untreated_1	0.5	Deoxy-nucleosides	False			-5.917	-1.791
4	GlcNAc 6-P	Carbon	1	AT_Untreated_1_5	AT_Untreated_1	1.0	Aminosugar biosynthesis	True	1.127e+06	0.03058	0.03058	-0.298
337	Xanthosine	Nucleotide	1	AT_Untreated_1_5	AT_Untreated_1	1.0	Purine nucleosides	True	1.086e+05	-3.344	-3.344	-0.27
188	Kynurenine	Amino acid	1	AT_Untreated_1_5	AT_Untreated_1	1.0	Amino acid derivatives	True	2.092e+06	0.9232	0.9232	1.615

Metabolite ID	Name	Super Pathway	Dataset	Sample ID	Group ID	Detection Fraction	Pathway	Detected	Raw Intensity	Log2 Norm Intensity	Norm Imputed	Log2 Ctrl Norm
63	6-P-Gluconate	Carbon	1	AT_Untreated_1_5	AT_Untreated_1	1.0	Pentose phosphate pathway (PPP)	True	1.316e+06	0.2546	0.2546	-1.779
40	Glucuronate	Carbon	1	AT_Untreated_1_5	AT_Untreated_1	1.0	Sugars and sugar alcohols	True	2.656e+05	-2.055	-2.055	-0.5154
108	Argininosuccinate	Amino acid	1	AT_Untreated_1_5	AT_Untreated_1	1.0	Amino acids biosynthesis intermediates	True	1.392e+05	-2.987	-2.987	-2.761
710	Carnitine	Cofactor	1	AT_Untreated_1_5	AT_Untreated_1	1.0	Cofactors	True	3.388e+07	4.941	4.941	-1.037
725	P-Pantetheine	Cofactor	1	AT_Untreated_1_5	AT_Untreated_1	1.0	Coenzyme A biosynthesis	True	2.156e+05	-2.355	-2.355	1.35
48	DHAP	Carbon	1	AT_Untreated_1_5	AT_Untreated_1	1.0	Glycolysis, GNG	True	4.706e+06	2.093	2.093	-1.004
17	Maltose	Carbon	1	AT_Untreated_1_5	AT_Untreated_1	1.0	Glycogen degradation	True	1.738e+06	0.656	0.656	1.949
359	N1-Me-Adenosine	Nucleotide	1	AT_Untreated_1_5	AT_Untreated_1	0.5	Purine derivatives in RNAs	False			-2.032	-0.9939
159	3-Me-His	Amino acid	1	AT_Untreated_1_5	AT_Untreated_1	1.0	Amino acid derivatives	True	2.793e+04	-5.304	-5.304	-1.068
155	4-Guanidinobutanoate	Amino acid	1	AT_Untreated_1_5	AT_Untreated_1	1.0	Amino acid derivatives	True	1.538e+05	-2.842	-2.842	-1.409
164	5-OH-Lys	Amino acid	1	AT_Untreated_1_5	AT_Untreated_1	1.0	Amino acid derivatives	True	1.215e+05	-3.183	-3.183	-1.412
357	Adenosine-3',5'-PP	Nucleotide	1	AT_Untreated_1_5	AT_Untreated_1	0.6666666666666667	Purine byproducts of metabolic processes	True	9.495e+04	-3.538	-3.538	-0.06101
104	Cystathionine	Amino acid	1	AT_Untreated_1_5	AT_Untreated_1	1.0	Amino acids biosynthesis intermediates	True	1.918e+06	0.798	0.798	-1.411
113	Imidazole Lactate	Amino acid	1	AT_Untreated_1_5	AT_Untreated_1	0.6666666666666667	Amino acids degradation intermediates	False			-4.646	-0.9699
215	N-Ac-Glu	Amino acid	1	AT_Untreated_1_5	AT_Untreated_1	1.0	N-acetylated amino acids	True	5.064e+06	2.199	2.199	0.4551
310	S-Lactoyl-Glutathione	Amino acid	1	AT_Untreated_1_5	AT_Untreated_1	1.0	Glutathione derivatives	True	1.449e+06	0.3936	0.3936	0.0372
5	GlcNAc 1-P	Carbon	1	AT_Untreated_1_5	AT_Untreated_1	0.8333333333333333	Aminosugar biosynthesis	True	2.138e+05	-2.367	-2.367	-0.4566
34	Ribitol	Carbon	1	AT_Untreated_1_5	AT_Untreated_1	1.0	Sugars and sugar alcohols	True	1.19e+05	-3.213	-3.213	0.2172
10	UDP-Galactose	Carbon	1	AT_Untreated_1_5	AT_Untreated_1	0.8333333333333333	Polysaccharide biosynthesis	True	8.699e+05	-0.3428	-0.3428	0.2837
13	Guanosine 5'-PP-Fucose	Carbon	1	AT_Untreated_1_5	AT_Untreated_1	1.0	Polysaccharide biosynthesis	True	3.622e+04	-4.929	-4.929	-2.63

Metabolite ID	Name	Super Pathway	Dataset	Sample ID	Group ID	Detection Fraction	Pathway	Detected	Raw Intensity	Log2 Norm Intensity	Norm Imputed	Log2 Ctrl Norm
19	Maltotetraose	Carbon	1	AT_Untreated_1_5	AT_Untreated_1	1.0	Glycogen degradation	True	1.705e+07	3.95	3.95	3.994
233	SAM	Amino acid	1	AT_Untreated_1_5	AT_Untreated_1	0.333333333333333	SAM metabolism	False			-2.121	-1.965
129	5-Aminovalerate	Amino acid	1	AT_Untreated_1_5	AT_Untreated_1	0.333333333333333	Amino acids degradation intermediates	False			0.04171	-0.96
741	5-Me-THF	Cofactor	1	AT_Untreated_1_5	AT_Untreated_1	0.333333333333333	Folate metabolism	False			-5.366	-0.6085
198	Indolelactate	Amino acid	1	AT_Untreated_1_5	AT_Untreated_1	1.0	Amino acid derivatives	True	1.278e+05	-3.11	-3.11	0.6772
254	Gly-Val	Amino acid	1	AT_Untreated_1_5	AT_Untreated_1	1.0	Dipeptides	True	1.392e+06	0.3353	0.3353	-0.776
291	gamma-Glu-Leu	Amino acid	1	AT_Untreated_1_5	AT_Untreated_1	1.0	Gamma-glutamyl dipeptides	True	3.637e+05	-1.601	-1.601	-1.112
173	Met Sulfoxide	Amino acid	1	AT_Untreated_1_5	AT_Untreated_1	1.0	Amino acid derivatives	True	5.622e+06	2.349	2.349	-0.2179
43	Glucose	Carbon	1	AT_Untreated_1_5	AT_Untreated_1	1.0	Glycolysis, GNG	True	1.046e+08	6.567	6.567	1.515
185	Phenyllactate	Amino acid	1	AT_Untreated_1_5	AT_Untreated_1	0.833333333333333	Amino acid derivatives	False			-5.917	-0.1051
156	Homo-Arg	Amino acid	1	AT_Untreated_1_5	AT_Untreated_1	1.0	Amino acid derivatives	True	2.171e+06	0.9766	0.9766	-0.2039
135	Homocitrulline	Amino acid	1	AT_Untreated_1_5	AT_Untreated_1	1.0	Amino acids degradation intermediates	True	4.377e+05	-1.334	-1.334	0.3689
719	Nicotinamide MN	Cofactor	1	AT_Untreated_1_5	AT_Untreated_1	1.0	NAD biosynthesis	True	3.883e+06	1.816	1.816	1.63
212	N-Ac-Asp	Amino acid	1	AT_Untreated_1_5	AT_Untreated_1	1.0	N-acetylated amino acids	True	9.792e+05	-0.172	-0.172	-0.8693
720	1-Me-Nicotinamide	Cofactor	1	AT_Untreated_1_5	AT_Untreated_1	1.0	Derivatives of NA, nicotinamide and NAD	True	2.626e+08	7.895	7.895	4.61e-03
216	N-Ac-Gly	Amino acid	1	AT_Untreated_1_5	AT_Untreated_1	0.5	N-acetylated amino acids	False			-4.155	-0.6151
70	Creatine	Carbon	1	AT_Untreated_1_5	AT_Untreated_1	1.0	Creatine energy storage	True	5.543e+08	8.973	8.973	-0.4442
26	Galactonate	Carbon	1	AT_Untreated_1_5	AT_Untreated_1	0.666666666666667	Sugars and sugar alcohols	True	4.128e+05	-1.418	-1.418	-0.6135
309	Glutathione, Oxidized	Amino acid	1	AT_Untreated_1_5	AT_Untreated_1	1.0	Glutathione	True	3.251e+06	1.559	1.559	0.3665
35	Ribonate	Carbon	1	AT_Untreated_1_5	AT_Untreated_1	0.833333333333333	Sugars and sugar alcohols	True	7.212e+05	-0.6132	-0.6132	-0.08597
160	1-Me-His	Amino acid	1	AT_Untreated_1_5	AT_Untreated_1	1.0	Amino acid derivatives	True	1.122e+07	3.346	3.346	-0.09466

Metabolite ID	Name	Super Pathway	Dataset	Sample ID	Group ID	Detection Fraction	Pathway	Detected	Raw Intensity	Log2 Norm Intensity	Norm Imputed	Log2 Ctrl Norm
44	Glucose 6-P	Carbon	1	AT_Untreated_1_5	AT_Untreated_1	0.833333333333333	Glycolysis, GNG	True	4.207e+05	-1.391	-1.391	0.08585
704	NADH	Cofactor	1	AT_Untreated_1_5	AT_Untreated_1	1.0	Cofactors	True	8.042e+05	-0.4561	-0.4561	0.19
275	Thr-Phe	Amino acid	1	AT_Untreated_1_5	AT_Untreated_1	0.833333333333333	Dipeptides	True	7.328e+05	-0.5901	-0.5901	1.063
738	Pyridoxate	Cofactor	1	AT_Untreated_1_5	AT_Untreated_1	1.0	PLP biosynthesis and salvage	True	1.503e+05	-2.876	-2.876	0.4883
177	3-(4-OH-Phenyl)Lactate	Amino acid	1	AT_Untreated_1_5	AT_Untreated_1	1.0	Amino acid derivatives	True	2.655e+05	-2.055	-2.055	0.4105
206	Trans-4-OH-Pro	Amino acid	1	AT_Untreated_1_5	AT_Untreated_1	1.0	Amino acid derivatives	True	2.706e+07	4.617	4.617	-0.5385
329	AMP	Nucleotide	1	AT_Untreated_1_5	AT_Untreated_1	1.0	Purine nucleotides	True	7.405e+05	-0.575	-0.575	-4.21
11	UDP-Glucose	Carbon	1	AT_Untreated_1_5	AT_Untreated_1	0.833333333333333	Polysaccharide biosynthesis	True	8.848e+05	-0.3183	-0.3183	-0.2131
158	4-Imidazole-Ac	Amino acid	1	AT_Untreated_1_5	AT_Untreated_1	1.0	Amino acid derivatives	True	3.329e+05	-1.729	-1.729	0.4181
111	1-Me-Imidazole-Ac	Amino acid	1	AT_Untreated_1_5	AT_Untreated_1	1.0	Amino acids degradation intermediates	True	5.186e+05	-1.089	-1.089	0.8448
345	Guanine	Nucleotide	1	AT_Untreated_1_5	AT_Untreated_1	1.0	Purine bases	True	4.36e+07	5.304	5.304	0.3181
22	N-Ac-Neuraminate	Carbon	1	AT_Untreated_1_5	AT_Untreated_1	1.0	Aminosugar derivatives	True	6.963e+05	-0.6639	-0.6639	-0.5285
721	N'-Methylnicotinate	Cofactor	1	AT_Untreated_1_5	AT_Untreated_1	1.0	Derivatives of NA, nicotinamide and NAD	True	4.518e+05	-1.288	-1.288	-0.2802
183	Phenol Sulfate	Amino acid	1	AT_Untreated_1_5	AT_Untreated_1	1.0	Amino acid derivatives	True	4.525e+04	-4.607	-4.607	0.03266
718	Nicotinamide Riboside	Cofactor	1	AT_Untreated_1_5	AT_Untreated_1	1.0	NAD biosynthesis	True	2.129e+07	4.27	4.27	2.68
297	gamma-Glu-Thr	Amino acid	1	AT_Untreated_1_5	AT_Untreated_1	1.0	Gamma-glutamyl dipeptides	True	8.115e+05	-0.4429	-0.4429	-1.083
295	gamma-Glu-Phe	Amino acid	1	AT_Untreated_1_5	AT_Untreated_1	0.166666666666667	Gamma-glutamyl dipeptides	False			-5.975	-0.1899
347	Allantoic Acid	Nucleotide	1	AT_Untreated_1_5	AT_Untreated_1	0.666666666666667	Purine degradation	False			-6.474	-0.8345
399	Pseudouridine	Nucleotide	1	AT_Untreated_1_5	AT_Untreated_1	1.0	Pyrimidine derivatives in RNAs	True	3.922e+05	-1.492	-1.492	0.5379
375	UTP	Nucleotide	1	AT_Untreated_1_5	AT_Untreated_1	0.666666666666667	Pyrimidine nucleotides	True	4.014e+04	-4.78	-4.78	-2.804
144	Glu, gamma-Me Ester	Amino acid	1	AT_Untreated_1_5	AT_Untreated_1	1.0	Amino acid derivatives	True	4.604e+05	-1.261	-1.261	-1.838

Metabolite ID	Name	Super Pathway	Dataset	Sample ID	Group ID	Detection Fraction	Pathway	Detected	Raw Intensity	Log2 Norm Intensity	Norm Imputed	Log2 Ctrl Norm
292	gamma-Glu-epsilon-Lysine	Amino acid	1	AT_Untreated_1_5	AT_Untreated_1	1.0	Gamma-glutamyl dipeptides	True	4.933e+05	-1.161	-1.161	-0.9696
225	N-Ac-Thr	Amino acid	1	AT_Untreated_1_5	AT_Untreated_1	1.0	N-acetylated amino acids	True	9.137e+04	-3.594	-3.594	-2.091
211	N-Ac-Asn	Amino acid	1	AT_Untreated_1_5	AT_Untreated_1	1.0	N-acetylated amino acids	True	1.124e+05	-3.295	-3.295	-0.61
151	Phenylacetylglycine	Amino acid	1	AT_Untreated_1_5	AT_Untreated_1	1.0	Amino acid derivatives	True	6.145e+05	-0.8442	-0.8442	-0.05725
217	N-Ac-His	Amino acid	1	AT_Untreated_1_5	AT_Untreated_1	0.833333333333333	N-acetylated amino acids	True	1.034e+05	-3.415	-3.415	0.06961
288	gamma-Glu-Gly	Amino acid	1	AT_Untreated_1_5	AT_Untreated_1	0.333333333333333	Gamma-glutamyl dipeptides	False			-3.092	-1.268
222	N-Ac-Phe	Amino acid	1	AT_Untreated_1_5	AT_Untreated_1	0.333333333333333	N-acetylated amino acids	False			-6.698	-0.4255
71	Creatine-P	Carbon	1	AT_Untreated_1_5	AT_Untreated_1	1.0	Creatine energy storage	True	5.164e+04	-4.417	-4.417	-0.1618
210	N-Ac-Arg	Amino acid	1	AT_Untreated_1_5	AT_Untreated_1	0.666666666666667	N-acetylated amino acids	False			-4.025	-1.35
218	N-Ac-Ile	Amino acid	1	AT_Untreated_1_5	AT_Untreated_1	0.166666666666667	N-acetylated amino acids	False			-6.52	-0.5541
251	Gly-Leu	Amino acid	1	AT_Untreated_1_5	AT_Untreated_1	1.0	Dipeptides	True	1.056e+06	-0.06324	-0.06324	-1.702
290	gamma-Glu-Ile	Amino acid	1	AT_Untreated_1_5	AT_Untreated_1	1.0	Gamma-glutamyl dipeptides	True	2.626e+05	-2.071	-2.071	-0.7321
316	Ophthalmate	Amino acid	1	AT_Untreated_1_5	AT_Untreated_1	1.0	Oxidative stress markers	True	4.731e+06	2.1	2.1	-0.2422
125	Isovaleryl-Gly	Amino acid	1	AT_Untreated_1_5	AT_Untreated_1	1.0	Amino acids degradation intermediates	True	5.046e+04	-4.45	-4.45	0.9592
368	7-Me-Guanine	Nucleotide	1	AT_Untreated_1_5	AT_Untreated_1	1.0	Purine derivatives in RNAs	True	2.268e+05	-2.282	-2.282	-0.09196
208	Pro-OH-Pro	Amino acid	1	AT_Untreated_1_5	AT_Untreated_1	1.0	Amino acid derivatives	True	8.668e+06	2.974	2.974	-0.3553
366	N2,N2-DiMe-Guanosine	Nucleotide	1	AT_Untreated_1_5	AT_Untreated_1	0.833333333333333	Purine derivatives in RNAs	False			-5.009	-0.8957
352	3'-AMP	Nucleotide	1	AT_Untreated_1_5	AT_Untreated_1	1.0	Purine derivatives in signaling	True	2.272e+05	-2.28	-2.28	-0.7929
363	N6-Carbamoyl-Thr-Adenosine	Nucleotide	1	AT_Untreated_1_5	AT_Untreated_1	0.666666666666667	Purine derivatives in RNAs	False			-6.008	-1.323
314	Cys-Glutathione Disulfide	Amino acid	1	AT_Untreated_1_5	AT_Untreated_1	1.0	Oxidative stress markers	True	6.809e+04	-4.018	-4.018	-0.734
382	Orotidine	Nucleotide	1	AT_Untreated_1_5	AT_Untreated_1	0.166666666666667	Pyrimidine (UMP) biosynthesis	False			-5.287	-0.9952

Metabolite ID	Name	Super Pathway	Dataset	Sample ID	Group ID	Detection Fraction	Pathway	Detected	Raw Intensity	Log2 Norm Intensity	Norm Imputed	Log2 Ctrl Norm
307	Cys-Gly	Amino acid	1	AT_Untreated_1_5	AT_Untreated_1	1.0	Glutathione biosynthesis	True	2.82e+06	1.354	1.354	-0.253
64	Sedoheptulose-7-P	Carbon	1	AT_Untreated_1_5	AT_Untreated_1	1.0	Pentose phosphate pathway (PPP)	True	4.325e+05	-1.351	-1.351	-1.505
142	N-Ac-Asp-Glu	Amino acid	1	AT_Untreated_1_5	AT_Untreated_1	1.0	Amino acid derivatives	True	3.988e+05	-1.468	-1.468	-0.3227
708	Thiamin-PP	Cofactor	1	AT_Untreated_1_5	AT_Untreated_1	0.5	Cofactors	False			-6.331	-0.3472
182	P-Cresol Sulfate	Amino acid	1	AT_Untreated_1_5	AT_Untreated_1	1.0	Amino acid derivatives	True	2.518e+05	-2.131	-2.131	1.065
250	Gly-Ile	Amino acid	1	AT_Untreated_1_5	AT_Untreated_1	1.0	Dipeptides	True	9.492e+04	-3.539	-3.539	-2.222
286	gamma-Glu-Glu	Amino acid	1	AT_Untreated_1_5	AT_Untreated_1	1.0	Gamma-glutamyl dipeptides	True	7.832e+05	-0.4942	-0.4942	-0.512
739	Deoxycarnitine	Cofactor	1	AT_Untreated_1_5	AT_Untreated_1	1.0	Carnitine biosynthesis	True	4.698e+06	2.09	2.09	-1.661
203	DiMe-Arg	Amino acid	1	AT_Untreated_1_5	AT_Untreated_1	1.0	Amino acid derivatives	True	1.649e+07	3.902	3.902	-1.731
351	2'-AMP	Nucleotide	1	AT_Untreated_1_5	AT_Untreated_1	1.0	Purine derivatives in signaling	True	3.602e+05	-1.615	-1.615	0.6539
8	Cytidine 5'-P-N-Ac-Neuraminate	Carbon	1	AT_Untreated_1_5	AT_Untreated_1	1.0	Aminosugar biosynthesis	True	2.441e+05	-2.176	-2.176	-0.4309
285	gamma-Glu-Ala	Amino acid	1	AT_Untreated_1_5	AT_Untreated_1	0.5	Gamma-glutamyl dipeptides	True	1.392e+05	-2.986	-2.986	0.2524
224	N-Ac-Ser	Amino acid	1	AT_Untreated_1_5	AT_Untreated_1	1.0	N-acetylated amino acids	True	1.315e+06	0.2534	0.2534	-1.626
244	Ala-Leu	Amino acid	1	AT_Untreated_1_5	AT_Untreated_1	1.0	Dipeptides	True	2.812e+06	1.35	1.35	0.2119
207	N-Me-Pro	Amino acid	1	AT_Untreated_1_5	AT_Untreated_1	1.0	Amino acid derivatives	True	9.073e+05	-0.282	-0.282	0.648
171	Cys Sulfinic Acid	Amino acid	1	AT_Untreated_1_5	AT_Untreated_1	0.333333333333333	Amino acid derivatives	False			-5.211	-1.913
181	O-Me-Tyr	Amino acid	1	AT_Untreated_1_5	AT_Untreated_1	0.5	Amino acid derivatives	False			-4.309	-0.9396
240	N-Ac-Putrescine	Amino acid	1	AT_Untreated_1_5	AT_Untreated_1	0.833333333333333	Polyamine derivatives	True	1.444e+05	-2.934	-2.934	-0.5701
176	S-Me-Met	Amino acid	1	AT_Untreated_1_5	AT_Untreated_1	1.0	Amino acid derivatives	True	2.509e+05	-2.137	-2.137	-0.2949
339	AICAR	Nucleotide	1	AT_Untreated_1_5	AT_Untreated_1	0.333333333333333	IMP biosynthesis	False			-4.716	-0.4151
141	gamma-Carboxy-Glu	Amino acid	1	AT_Untreated_1_5	AT_Untreated_1	1.0	Amino acid derivatives	True	9.118e+05	-0.2749	-0.2749	0.5101
392	3'-UMP	Nucleotide	1	AT_Untreated_1_5	AT_Untreated_1	0.833333333333333	Pyrimidine derivatives in signaling	True	1.173e+05	-3.234	-3.234	0.7997

Metabolite ID	Name	Super Pathway	Dataset	Sample ID	Group ID	Detection Fraction	Pathway	Detected	Raw Intensity	Log2 Norm Intensity	Norm Imputed	Log2 Ctrl Norm
355	3'-GMP	Nucleotide	1	AT_Untreated_1_5	AT_Untreated_1	0.833333333333333	Purine derivatives in signaling	True	8.725e+04	-3.66	-3.66	0.2771
282	Val-Leu	Amino acid	1	AT_Untreated_1_5	AT_Untreated_1	1.0	Dipeptides	True	3.086e+06	1.484	1.484	0.5123
140	Carboxyethyl-GABA	Amino acid	1	AT_Untreated_1_5	AT_Untreated_1	1.0	Amino acid derivatives	True	2.437e+05	-2.178	-2.178	-0.7472
258	Ile-Gly	Amino acid	1	AT_Untreated_1_5	AT_Untreated_1	1.0	Dipeptides	True	3.818e+06	1.791	1.791	-1.521
260	Leu-Ala	Amino acid	1	AT_Untreated_1_5	AT_Untreated_1	1.0	Dipeptides	True	4.78e+06	2.115	2.115	0.8142
265	Lys-Leu	Amino acid	1	AT_Untreated_1_5	AT_Untreated_1	0.833333333333333	Dipeptides	True	1.638e+05	-2.752	-2.752	-0.1408
263	Leu-Gly	Amino acid	1	AT_Untreated_1_5	AT_Untreated_1	1.0	Dipeptides	True	3.291e+07	4.899	4.899	1.877
281	Val-Gly	Amino acid	1	AT_Untreated_1_5	AT_Untreated_1	1.0	Dipeptides	True	1.123e+07	3.348	3.348	-1.171
270	Pro-Gly	Amino acid	1	AT_Untreated_1_5	AT_Untreated_1	1.0	Dipeptides	True	7.102e+04	-3.957	-3.957	-4.704
114	Imidazole Propionate	Amino acid	1	AT_Untreated_1_5	AT_Untreated_1	1.0	Amino acids degradation intermediates	True	4.408e+05	-1.323	-1.323	0.9871
267	Phe-Gly	Amino acid	1	AT_Untreated_1_5	AT_Untreated_1	1.0	Dipeptides	True	8.703e+06	2.98	2.98	-1.114
266	Phe-Ala	Amino acid	1	AT_Untreated_1_5	AT_Untreated_1	1.0	Dipeptides	True	2.012e+06	0.8667	0.8667	-0.21
278	Tyr-Gly	Amino acid	1	AT_Untreated_1_5	AT_Untreated_1	1.0	Dipeptides	True	2.413e+06	1.129	1.129	-1.053
255	His-Ala	Amino acid	1	AT_Untreated_1_5	AT_Untreated_1	0.833333333333333	Dipeptides	True	1.83e+05	-2.592	-2.592	-1.254
280	Val-Gln	Amino acid	1	AT_Untreated_1_5	AT_Untreated_1	1.0	Dipeptides	True	2.624e+06	1.25	1.25	-0.3681
143	S-1-Pyrroline-5-Carboxylate	Amino acid	1	AT_Untreated_1_5	AT_Untreated_1	0.833333333333333	Amino acid derivatives	True	3.296e+05	-1.743	-1.743	0.3252
232	SAH	Amino acid	1	AT_Untreated_1_5	AT_Untreated_1	1.0	SAM metabolism	True	3.179e+05	-1.795	-1.795	-1.31
20	Erythronate	Carbon	1	AT_Untreated_1_5	AT_Untreated_1	1.0	Aminosugar derivatives	True	1.912e+07	4.116	4.116	0.3742
248	Gln-Leu	Amino acid	1	AT_Untreated_1_5	AT_Untreated_1	0.833333333333333	Dipeptides	True	9.018e+05	-0.2907	-0.2907	0.6145
276	Trp-Gly	Amino acid	1	AT_Untreated_1_5	AT_Untreated_1	0.833333333333333	Dipeptides	True	1.363e+05	-3.017	-3.017	-1.938
205	N-delta-Ac-Ornithine	Amino acid	1	AT_Untreated_1_5	AT_Untreated_1	1.0	Amino acid derivatives	True	1.173e+06	0.08901	0.08901	1.21
163	Formimino-Glu	Amino acid	1	AT_Untreated_1_5	AT_Untreated_1	1.0	Amino acid derivatives	True	4.826e+05	-1.193	-1.193	0.04708
204	N-Me-Arg	Amino acid	1	AT_Untreated_1_5	AT_Untreated_1	0.833333333333333	Amino acid derivatives	True	3.455e+06	1.647	1.647	-0.94
242	Guanidino-Ac	Amino acid	1	AT_Untreated_1_5	AT_Untreated_1	1.0	Creatine biosynthesis	True	1.756e+05	-2.651	-2.651	1.229

Metabolite ID	Name	Super Pathway	Dataset	Sample ID	Group ID	Detection Fraction	Pathway	Detected	Raw Intensity	Log2 Norm Intensity	Norm Imputed	Log2 Ctrl Norm
300	gamma-Glu-Val	Amino acid	1	AT_Untreated_1_5	AT_Untreated_1	0.333333333333333	Gamma-glutamyl dipeptides	False			-0.1053	-0.726
53	Ac-CoA	Carbon	1	AT_Untreated_1_5	AT_Untreated_1	0.166666666666667	Acetyl-CoA	False			-7.274	-0.5114
18	Maltotriose	Carbon	1	AT_Untreated_1_5	AT_Untreated_1	1.0	Glycogen degradation	True	1.21e+07	3.456	3.456	3.613
294	gamma-Glu-Met	Amino acid	1	AT_Untreated_1_5	AT_Untreated_1	0.333333333333333	Gamma-glutamyl dipeptides	False			-4.503	-1.021
174	Met Sulfone	Amino acid	1	AT_Untreated_1_5	AT_Untreated_1	0.833333333333333	Amino acid derivatives	True	8.357e+04	-3.723	-3.723	-0.6438
175	N-Ac-Met Sulfoxide	Amino acid	1	AT_Untreated_1_5	AT_Untreated_1	1.0	Amino acid derivatives	True	2.644e+05	-2.061	-2.061	-2.037
25	Mannitol/Sorbitol	Carbon	1	AT_Untreated_1_5	AT_Untreated_1	1.0	Sugars and sugar alcohols	True	1.106e+07	3.325	3.325	1.101
6	UDP-GlcNAc	Carbon	1	AT_Untreated_1_5	AT_Untreated_1	0.166666666666667	Aminosugar biosynthesis	False			-3.589	-0.4814
145	Pyro-Gln	Amino acid	1	AT_Untreated_1_5	AT_Untreated_1	1.0	Amino acid derivatives	True	1.773e+06	0.6849	0.6849	-0.1549
705	Coenzyme A	Cofactor	1	AT_Untreated_1_5	AT_Untreated_1	0.666666666666667	Cofactors	False			-4.967	-1.008
319	2'-dAMP	Nucleotide	1	AT_Untreated_1_5	AT_Untreated_1	0.166666666666667	Deoxy-nucleotides	False			-4.337	-0.6568
119	alpha-OH-Isovalerate	Amino acid	1	AT_Untreated_1_5	AT_Untreated_1	1.0	Amino acids degradation intermediates	True	1.236e+05	-3.158	-3.158	0.1402
46	Fructose 1,6-PP / Glucose 1,6-PP / Inositol-1,4-PP / Inositol-1,3-PP	Carbon	1	AT_Untreated_1_5	AT_Untreated_1	1.0	Glycolysis, GNG	True	4.072e+06	1.884	1.884	-2.947
137	1-Me-Guanidine	Amino acid	1	AT_Untreated_1_5	AT_Untreated_1	0.666666666666667	Amino acids degradation intermediates	True	3.561e+04	-4.953	-4.953	0.1995
23	N-GlcNAc-Asn	Carbon	1	AT_Untreated_1_5	AT_Untreated_1	1.0	Aminosugar derivatives	True	1.143e+06	0.05062	0.05062	-0.6623
262	Leu-Gln	Amino acid	1	AT_Untreated_1_5	AT_Untreated_1	1.0	Dipeptides	True	3.26e+06	1.563	1.563	0.2668
24	Fructose	Carbon	1	AT_Untreated_1_5	AT_Untreated_1	1.0	Sugars and sugar alcohols	True	1.267e+07	3.521	3.521	1.573
197	C-Glycosyl-Trp	Amino acid	1	AT_Untreated_1_5	AT_Untreated_1	1.0	Amino acid derivatives	True	3.479e+05	-1.665	-1.665	-1.366
33	Arabitol/Xylitol	Carbon	1	AT_Untreated_1_5	AT_Untreated_1	1.0	Sugars and sugar alcohols	True	2.717e+05	-2.022	-2.022	-0.1618

Metabolite ID	Name	Super Pathway	Dataset	Sample ID	Group ID	Detection Fraction	Pathway	Detected	Raw Intensity	Log2 Norm Intensity	Norm Imputed	Log2 Ctrl Norm
128	N2-Ac-Lys/N6-Ac-Lys	Amino acid	1	AT_Untreated_1_5	AT_Untreated_1	1.0	Amino acids degradation intermediates	True	8.153e+05	-0.4362	-0.4362	-1.647
42	2-Me-Citrate	Carbon	1	AT_Untreated_1_5	AT_Untreated_1	0.833333333333333	Propionate metabolism	False			-5.211	-0.8276
12	Glucuronate 1-P	Carbon	1	AT_Untreated_1_5	AT_Untreated_1	1.0	Polysaccharide biosynthesis	True	5.608e+05	-0.976	-0.976	0.2668
76	Gln	Amino acid	1	AT_Untreated_1_6	AT_Untreated_1	1.0	Proteinogenic amino acids	True	3.217e+08	8.418	8.418	-0.2629
89	Trp	Amino acid	1	AT_Untreated_1_6	AT_Untreated_1	1.0	Proteinogenic amino acids	True	4.748e+07	5.657	5.657	-0.01829
723	beta-Ala	Cofactor	1	AT_Untreated_1_6	AT_Untreated_1	1.0	Coenzyme A biosynthesis	True	5.757e+06	2.614	2.614	-0.3957
75	Glu	Amino acid	1	AT_Untreated_1_6	AT_Untreated_1	1.0	Proteinogenic amino acids	True	1.153e+09	10.26	10.26	-0.4772
77	Gly	Amino acid	1	AT_Untreated_1_6	AT_Untreated_1	1.0	Proteinogenic amino acids	True	2.884e+07	4.938	4.938	-0.6772
80	His	Amino acid	1	AT_Untreated_1_6	AT_Untreated_1	1.0	Proteinogenic amino acids	True	1.5e+07	3.996	3.996	0.5803
82	Leu	Amino acid	1	AT_Untreated_1_6	AT_Untreated_1	1.0	Proteinogenic amino acids	True	4.885e+08	9.021	9.021	-0.2007
87	Phe	Amino acid	1	AT_Untreated_1_6	AT_Untreated_1	1.0	Proteinogenic amino acids	True	3.879e+08	8.688	8.688	-0.1238
130	Glutarate	Amino acid	1	AT_Untreated_1_6	AT_Untreated_1	1.0	Amino acids degradation intermediates	True	4.204e+05	-1.162	-1.162	1.265
196	5-OH-Indole-Ac	Amino acid	1	AT_Untreated_1_6	AT_Untreated_1	1.0	Amino acid derivatives	True	2.62e+05	-1.844	-1.844	2.168
74	Asp	Amino acid	1	AT_Untreated_1_6	AT_Untreated_1	1.0	Proteinogenic amino acids	True	1.618e+08	7.426	7.426	-0.4654
236	Spermidine	Amino acid	1	AT_Untreated_1_6	AT_Untreated_1	1.0	Polyamines	True	1.403e+07	3.898	3.898	0.03689
73	Asn	Amino acid	1	AT_Untreated_1_6	AT_Untreated_1	1.0	Proteinogenic amino acids	True	7.873e+07	6.387	6.387	-0.2841
243	Creatinine	Amino acid	1	AT_Untreated_1_6	AT_Untreated_1	1.0	Creatine degradation	True	8.254e+07	6.455	6.455	1.453
376	Cytidine	Nucleotide	1	AT_Untreated_1_6	AT_Untreated_1	0.833333333333333	Pyrimidine nucleosides	True	6.006e+06	2.675	2.675	1.947
41	Lactate	Carbon	1	AT_Untreated_1_6	AT_Untreated_1	1.0	Respiratory carbon sources	True	1.185e+08	6.977	6.977	0.4953
58	alpha-Ketoglutarate	Carbon	1	AT_Untreated_1_6	AT_Untreated_1	1.0	TCA cycle	True	1.107e+06	0.2353	0.2353	0.2186
69	3-OH-Butyrate	Carbon	1	AT_Untreated_1_6	AT_Untreated_1	0.833333333333333	Ketone bodies	True	3.742e+05	-1.33	-1.33	1.655

Metabolite ID	Name	Super Pathway	Dataset	Sample ID	Group ID	Detection Fraction	Pathway	Detected	Raw Intensity	Log2 Norm Intensity	Norm Imputed	Log2 Ctrl Norm
343	Adenine	Nucleotide	1	AT_Untreated_1_6	AT_Untreated_1	1.0	Purine bases	True	2.7e+06	1.521	1.521	0.06402
336	Adenosine	Nucleotide	1	AT_Untreated_1_6	AT_Untreated_1	1.0	Purine nucleosides	True	1.414e+07	3.91	3.91	0.4868
722	ADP-Ribose	Cofactor	1	AT_Untreated_1_6	AT_Untreated_1	1.0	Derivatives of NA, nicotinamide and NAD	True	2.759e+05	-1.77	-1.77	1.233
383	Cytosine	Nucleotide	1	AT_Untreated_1_6	AT_Untreated_1	0.833333333333333	Pyrimidine bases	True	1.091e+05	-3.108	-3.108	1.748
3	Glucosamine 6-P	Carbon	1	AT_Untreated_1_6	AT_Untreated_1	0.833333333333333	Aminosugar biosynthesis	False			-3.737	-2.07
717	Nicotinamide	Cofactor	1	AT_Untreated_1_6	AT_Untreated_1	1.0	NAD biosynthesis	True	2.797e+07	4.894	4.894	0.3786
51	PEP	Carbon	1	AT_Untreated_1_6	AT_Untreated_1	1.0	Glycolysis, GNG	True	5.085e+06	2.434	2.434	1.383
237	Spermine	Amino acid	1	AT_Untreated_1_6	AT_Untreated_1	1.0	Polyamines	True	6.87e+05	-0.4535	-0.4535	1.037
385	Uracil	Nucleotide	1	AT_Untreated_1_6	AT_Untreated_1	1.0	Pyrimidine bases	True	2.33e+06	1.309	1.309	1.044
377	Uridine	Nucleotide	1	AT_Untreated_1_6	AT_Untreated_1	1.0	Pyrimidine nucleosides	True	2.578e+07	4.777	4.777	0.7276
348	Allantoin	Nucleotide	1	AT_Untreated_1_6	AT_Untreated_1	1.0	Purine degradation	True	3.253e+06	1.79	1.79	1.966
335	Inosine	Nucleotide	1	AT_Untreated_1_6	AT_Untreated_1	1.0	Purine nucleosides	True	3.929e+07	5.384	5.384	-0.1146
81	Ile	Amino acid	1	AT_Untreated_1_6	AT_Untreated_1	1.0	Proteinogenic amino acids	True	5.538e+08	9.201	9.201	0.4862
72	Ala	Amino acid	1	AT_Untreated_1_6	AT_Untreated_1	1.0	Proteinogenic amino acids	True	2.796e+08	8.215	8.215	-0.2861
79	Thr	Amino acid	1	AT_Untreated_1_6	AT_Untreated_1	1.0	Proteinogenic amino acids	True	1.487e+08	7.305	7.305	-0.7048
88	Tyr	Amino acid	1	AT_Untreated_1_6	AT_Untreated_1	1.0	Proteinogenic amino acids	True	1.338e+08	7.152	7.152	-0.3721
84	Lys	Amino acid	1	AT_Untreated_1_6	AT_Untreated_1	1.0	Proteinogenic amino acids	True	2.624e+08	8.124	8.124	0.134
86	Met	Amino acid	1	AT_Untreated_1_6	AT_Untreated_1	1.0	Proteinogenic amino acids	True	1.88e+08	7.643	7.643	-0.5847
61	Malate	Carbon	1	AT_Untreated_1_6	AT_Untreated_1	1.0	TCA cycle	True	1.197e+08	6.991	6.991	-0.1806
235	Putrescine	Amino acid	1	AT_Untreated_1_6	AT_Untreated_1	0.833333333333333	Polyamines	True	7.911e+05	-0.2498	-0.2498	0.2414
324	2'-dU	Nucleotide	1	AT_Untreated_1_6	AT_Untreated_1	0.666666666666667	Deoxy-nucleosides	True	1.2e+05	-2.971	-2.971	1.409
49	3-P-Glycerate	Carbon	1	AT_Untreated_1_6	AT_Untreated_1	1.0	Glycolysis, GNG	True	1.644e+07	4.127	4.127	0.8618
189	Kynurenate	Amino acid	1	AT_Untreated_1_6	AT_Untreated_1	1.0	Amino acid derivatives	True	9.304e+04	-3.338	-3.338	2.46
234	5-Me-Thioadenosine	Amino acid	1	AT_Untreated_1_6	AT_Untreated_1	1.0	SAM metabolism	True	1.815e+06	0.9485	0.9485	-0.937

Metabolite ID	Name	Super Pathway	Dataset	Sample ID	Group ID	Detection Fraction	Pathway	Detected	Raw Intensity	Log2 Norm Intensity	Norm Imputed	Log2 Ctrl Norm
59	Succinate	Carbon	1	AT_Untreated_1_6	AT_Untreated_1	1.0	TCA cycle	True	3.6e+06	1.936	1.936	1.635
36	Ribose	Carbon	1	AT_Untreated_1_6	AT_Untreated_1	1.0	Sugars and sugar alcohols	True	3.495e+05	-1.428	-1.428	-0.1949
133	Ornithine	Amino acid	1	AT_Untreated_1_6	AT_Untreated_1	1.0	Amino acids degradation intermediates	True	4.138e+07	5.459	5.459	0.9796
313	5-Oxoproline	Amino acid	1	AT_Untreated_1_6	AT_Untreated_1	1.0	Glutathione derivatives	True	8.28e+06	3.138	3.138	1.05
165	N-6-Tri-Me-Lys	Amino acid	1	AT_Untreated_1_6	AT_Untreated_1	1.0	Amino acid derivatives	True	1.03e+07	3.453	3.453	-0.3109
380	Orotate	Nucleotide	1	AT_Untreated_1_6	AT_Untreated_1	0.666666666666667	Pyrimidine (UMP) biosynthesis	False			-4.969	-1.339
724	Pantothenate	Cofactor	1	AT_Untreated_1_6	AT_Untreated_1	1.0	Coenzyme A biosynthesis	True	8.691e+07	6.53	6.53	0.2195
150	N-Me-Gly	Amino acid	1	AT_Untreated_1_6	AT_Untreated_1	1.0	Amino acid derivatives	True	6.773e+05	-0.4739	-0.4739	-0.1735
122	3-OH-Isobutyrate	Amino acid	1	AT_Untreated_1_6	AT_Untreated_1	0.833333333333333	Amino acids degradation intermediates	True	3.446e+05	-1.449	-1.449	1.707
241	4-Acetamidobutanoate	Amino acid	1	AT_Untreated_1_6	AT_Untreated_1	1.0	Polyamine derivatives	True	5.104e+06	2.44	2.44	1.999
711	alpha-Tocopherol	Cofactor	1	AT_Untreated_1_6	AT_Untreated_1	1.0	Cofactors	True	1.976e+06	1.071	1.071	0.2906
55	Citrate	Carbon	1	AT_Untreated_1_6	AT_Untreated_1	1.0	TCA cycle	True	7.67e+06	3.027	3.027	1.078
387	3-Aminoisobutyrate	Nucleotide	1	AT_Untreated_1_6	AT_Untreated_1	0.833333333333333	Pyrimidine degradation	True	2.488e+05	-1.919	-1.919	0.09516
338	Guanosine	Nucleotide	1	AT_Untreated_1_6	AT_Untreated_1	1.0	Purine nucleosides	True	5.204e+07	5.79	5.79	0.6836
209	N-Ac-Ala	Amino acid	1	AT_Untreated_1_6	AT_Untreated_1	1.0	N-acetylated amino acids	True	2.661e+05	-1.822	-1.822	-0.6206
221	N-Ac-Met	Amino acid	1	AT_Untreated_1_6	AT_Untreated_1	1.0	N-acetylated amino acids	True	1.487e+06	0.661	0.661	-1.083
228	N-Ac-Val	Amino acid	1	AT_Untreated_1_6	AT_Untreated_1	0.333333333333333	N-acetylated amino acids	False			-5.82	-0.449
346	Urate	Nucleotide	1	AT_Untreated_1_6	AT_Untreated_1	1.0	Purine degradation	True	1.958e+06	1.057	1.057	2.12
90	Arg	Amino acid	1	AT_Untreated_1_6	AT_Untreated_1	1.0	Proteinogenic amino acids	True	8.943e+08	9.893	9.893	0.1872
60	Fumarate	Carbon	1	AT_Untreated_1_6	AT_Untreated_1	1.0	TCA cycle	True	2.579e+06	1.455	1.455	0.4396
78	Ser	Amino acid	1	AT_Untreated_1_6	AT_Untreated_1	1.0	Proteinogenic amino acids	True	6.66e+07	6.146	6.146	-1.112
83	Val	Amino acid	1	AT_Untreated_1_6	AT_Untreated_1	1.0	Proteinogenic amino acids	True	2.099e+08	7.802	7.802	-0.07037

Metabolite ID	Name	Super Pathway	Dataset	Sample ID	Group ID	Detection Fraction	Pathway	Detected	Raw Intensity	Log2 Norm Intensity	Norm Imputed	Log2 Ctrl Norm
734	Pyridoxal	Cofactor	1	AT_Untreated_1_6	AT_Untreated_1	1.0	PLP biosynthesis and salvage	True	6.357e+06	2.757	2.757	0.9734
136	Urea	Amino acid	1	AT_Untreated_1_6	AT_Untreated_1	1.0	Amino acids degradation intermediates	True	5.055e+06	2.426	2.426	2.275
67	Ribose 1-P	Carbon	1	AT_Untreated_1_6	AT_Untreated_1	1.0	Pentose phosphate pathway (PPP)	True	9.066e+05	-0.0533	-0.0533	-0.6745
284	Carnosine	Amino acid	1	AT_Untreated_1_6	AT_Untreated_1	1.0	Dipeptides	True	7.231e+05	-0.3795	-0.3795	1.729
306	gamma-Glu-Cys	Amino acid	1	AT_Untreated_1_6	AT_Untreated_1	1.0	Glutathione biosynthesis	True	2.158e+05	-2.124	-2.124	-1.787
712	Retinol (Vit A)	Cofactor	1	AT_Untreated_1_6	AT_Untreated_1	0.833333333333333	Cofactors	True	1.088e+05	-3.112	-3.112	-0.6785
85	Cys	Amino acid	1	AT_Untreated_1_6	AT_Untreated_1	1.0	Proteinogenic amino acids	True	1.557e+07	4.049	4.049	-0.4646
91	Pro	Amino acid	1	AT_Untreated_1_6	AT_Untreated_1	1.0	Proteinogenic amino acids	True	3.9e+08	8.695	8.695	-0.7126
308	Glutathione, Reduced	Amino acid	1	AT_Untreated_1_6	AT_Untreated_1	1.0	Glutathione	True	2.787e+07	4.889	4.889	-2.067
107	Citrulline	Amino acid	1	AT_Untreated_1_6	AT_Untreated_1	1.0	Amino acids biosynthesis intermediates	True	1.972e+07	4.39	4.39	1.565
328	IMP	Nucleotide	1	AT_Untreated_1_6	AT_Untreated_1	0.333333333333333	Purine nucleotides	False			-4.53	-0.2088
706	FAD	Cofactor	1	AT_Untreated_1_6	AT_Untreated_1	1.0	Cofactors	True	2.117e+05	-2.151	-2.151	0.03809
735	Pyridoxamine	Cofactor	1	AT_Untreated_1_6	AT_Untreated_1	1.0	PLP biosynthesis and salvage	True	9.079e+05	-0.0512	-0.0512	0.1418
199	Serotonin	Amino acid	1	AT_Untreated_1_6	AT_Untreated_1	1.0	Amino acid derivatives	True	3.54e+06	1.912	1.912	0.6392
370	CMP	Nucleotide	1	AT_Untreated_1_6	AT_Untreated_1	1.0	Pyrimidine nucleotides	True	5.727e+06	2.606	2.606	0.767
287	gamma-Glu-Gln	Amino acid	1	AT_Untreated_1_6	AT_Untreated_1	1.0	Gamma-glutamyl dipeptides	True	9.29e+05	-0.01806	-0.01806	-0.859
14	UDP-Glucuronate	Carbon	1	AT_Untreated_1_6	AT_Untreated_1	0.666666666666667	Polysaccharide biosynthesis	True	1.294e+06	0.46	0.46	2.409
229	N-Formyl-Met	Amino acid	1	AT_Untreated_1_6	AT_Untreated_1	0.666666666666667	N-formylated amino acids	False			-5.516	-1.694
350	3',5'-cAMP	Nucleotide	1	AT_Untreated_1_6	AT_Untreated_1	0.666666666666667	Purine derivatives in signaling	False			-4.527	-0.9083
371	CDP	Nucleotide	1	AT_Untreated_1_6	AT_Untreated_1	0.666666666666667	Pyrimidine nucleotides	True	4.41e+05	-1.093	-1.093	2.227
372	CTP	Nucleotide	1	AT_Untreated_1_6	AT_Untreated_1	0.333333333333333	Pyrimidine nucleotides	True	1.309e+05	-2.846	-2.846	0.8081

Metabolite ID	Name	Super Pathway	Dataset	Sample ID	Group ID	Detection Fraction	Pathway	Detected	Raw Intensity	Log2 Norm Intensity	Norm Imputed	Log2 Ctrl Norm
333	GDP	Nucleotide	1	AT_Untreated_1_6	AT_Untreated_1	0.5	Purine nucleotides	True	1.697e+05	-2.471	-2.471	0.02932
332	GMP	Nucleotide	1	AT_Untreated_1_6	AT_Untreated_1	1.0	Purine nucleotides	True	3.007e+06	1.677	1.677	0.978
373	UMP	Nucleotide	1	AT_Untreated_1_6	AT_Untreated_1	0.833333333333333	Pyrimidine nucleotides	True	6.587e+05	-0.5142	-0.5142	0.541
389	3'-CMP	Nucleotide	1	AT_Untreated_1_6	AT_Untreated_1	1.0	Pyrimidine derivatives in signaling	True	2.636e+05	-1.835	-1.835	-0.784
330	ADP	Nucleotide	1	AT_Untreated_1_6	AT_Untreated_1	0.5	Purine nucleotides	True	9.451e+05	6.75e-03	6.75e-03	0.3031
342	Hypoxanthine	Nucleotide	1	AT_Untreated_1_6	AT_Untreated_1	1.0	Purine bases	True	1.603e+07	4.091	4.091	0.152
736	Pyridoxamine-P	Cofactor	1	AT_Untreated_1_6	AT_Untreated_1	0.833333333333333	PLP biosynthesis and salvage	True	7.443e+04	-3.66	-3.66	-0.6815
148	Betaine	Amino acid	1	AT_Untreated_1_6	AT_Untreated_1	1.0	Amino acid derivatives	True	6.021e+07	6	6	1.194
344	Xanthine	Nucleotide	1	AT_Untreated_1_6	AT_Untreated_1	1.0	Purine bases	True	4.429e+06	2.235	2.235	1.328
386	3-Ureidopropionate	Nucleotide	1	AT_Untreated_1_6	AT_Untreated_1	1.0	Pyrimidine degradation	True	6.558e+05	-0.5204	-0.5204	0.399
149	DiMe-Gly	Amino acid	1	AT_Untreated_1_6	AT_Untreated_1	1.0	Amino acid derivatives	True	1.985e+06	1.078	1.078	1.598
703	NAD+	Cofactor	1	AT_Untreated_1_6	AT_Untreated_1	1.0	Cofactors	True	3.584e+06	1.93	1.93	-0.8067
709	Pyridoxal-P	Cofactor	1	AT_Untreated_1_6	AT_Untreated_1	0.833333333333333	Cofactors	True	1.137e+05	-3.048	-3.048	-0.7404
731	Thiamin (Vitamin B1)	Cofactor	1	AT_Untreated_1_6	AT_Untreated_1	1.0	TPP biosynthesis	True	1.981e+06	1.074	1.074	0.3267
374	UDP	Nucleotide	1	AT_Untreated_1_6	AT_Untreated_1	0.666666666666667	Pyrimidine nucleotides	True	8.022e+05	-0.2298	-0.2298	1.324
102	2-Aminoadipate	Amino acid	1	AT_Untreated_1_6	AT_Untreated_1	0.833333333333333	Amino acids biosynthesis intermediates	True	1.136e+05	-3.05	-3.05	-0.9402
45	Fructose-6-P	Carbon	1	AT_Untreated_1_6	AT_Untreated_1	1.0	Glycolysis, GNG	True	3.058e+05	-1.621	-1.621	-1.697
320	TMP	Nucleotide	1	AT_Untreated_1_6	AT_Untreated_1	0.5	Deoxy-nucleotides	True	2.956e+04	-4.992	-4.992	-0.2005
341	XMP	Nucleotide	1	AT_Untreated_1_6	AT_Untreated_1	0.166666666666667	IMP conversion to AMP & GMP	False			-6.353	-1.778
120	beta-OH-Isovalerate	Amino acid	1	AT_Untreated_1_6	AT_Untreated_1	1.0	Amino acids degradation intermediates	True	4.019e+05	-1.227	-1.227	1.939
322	2'-dI	Nucleotide	1	AT_Untreated_1_6	AT_Untreated_1	0.5	Deoxy-nucleosides	False			-5.917	-1.791
4	GlcNAc 6-P	Carbon	1	AT_Untreated_1_6	AT_Untreated_1	1.0	Aminosugar biosynthesis	True	8.712e+04	-3.433	-3.433	-3.761
337	Xanthosine	Nucleotide	1	AT_Untreated_1_6	AT_Untreated_1	1.0	Purine nucleosides	True	3.023e+05	-1.638	-1.638	1.437
188	Kynurenine	Amino acid	1	AT_Untreated_1_6	AT_Untreated_1	1.0	Amino acid derivatives	True	8.963e+05	-0.06979	-0.06979	0.6218

Metabolite ID	Name	Super Pathway	Dataset	Sample ID	Group ID	Detection Fraction	Pathway	Detected	Raw Intensity	Log2 Norm Intensity	Norm Imputed	Log2 Ctrl Norm
63	6-P-Gluconate	Carbon	1	AT_Untreated_1_6	AT_Untreated_1	1.0	Pentose phosphate pathway (PPP)	True	6.578e+06	2.806	2.806	0.7722
40	Glucuronate	Carbon	1	AT_Untreated_1_6	AT_Untreated_1	1.0	Sugars and sugar alcohols	True	3.331e+05	-1.498	-1.498	0.04115
108	Argininosuccinate	Amino acid	1	AT_Untreated_1_6	AT_Untreated_1	1.0	Amino acids biosynthesis intermediates	True	2.82e+05	-1.738	-1.738	-1.512
710	Carnitine	Cofactor	1	AT_Untreated_1_6	AT_Untreated_1	1.0	Cofactors	True	3.878e+07	5.366	5.366	-0.6119
725	P-Pantetheine	Cofactor	1	AT_Untreated_1_6	AT_Untreated_1	1.0	Coenzyme A biosynthesis	True	7.838e+04	-3.585	-3.585	0.1194
48	DHAP	Carbon	1	AT_Untreated_1_6	AT_Untreated_1	1.0	Glycolysis, GNG	True	1.288e+07	3.776	3.776	0.6792
17	Maltose	Carbon	1	AT_Untreated_1_6	AT_Untreated_1	1.0	Glycogen degradation	True	8.372e+05	-0.1682	-0.1682	1.125
359	N1-Me-Adenosine	Nucleotide	1	AT_Untreated_1_6	AT_Untreated_1	0.5	Purine derivatives in RNAs	False			-2.032	-0.9939
159	3-Me-His	Amino acid	1	AT_Untreated_1_6	AT_Untreated_1	1.0	Amino acid derivatives	True	9.533e+04	-3.303	-3.303	0.9326
155	4-Guanidinobutanoate	Amino acid	1	AT_Untreated_1_6	AT_Untreated_1	1.0	Amino acid derivatives	True	3.961e+05	-1.248	-1.248	0.1861
164	5-OH-Lys	Amino acid	1	AT_Untreated_1_6	AT_Untreated_1	1.0	Amino acid derivatives	True	6.203e+05	-0.6007	-0.6007	1.17
357	Adenosine-3',5'-PP	Nucleotide	1	AT_Untreated_1_6	AT_Untreated_1	0.666666666666667	Purine byproducts of metabolic processes	False			-5.09	-1.612
104	Cystathionine	Amino acid	1	AT_Untreated_1_6	AT_Untreated_1	1.0	Amino acids biosynthesis intermediates	True	2.658e+06	1.498	1.498	-0.7112
113	Imidazole Lactate	Amino acid	1	AT_Untreated_1_6	AT_Untreated_1	0.666666666666667	Amino acids degradation intermediates	True	1.472e+05	-2.676	-2.676	1.001
215	N-Ac-Glu	Amino acid	1	AT_Untreated_1_6	AT_Untreated_1	1.0	N-acetylated amino acids	True	1.461e+06	0.635	0.635	-1.109
310	S-Lactoyl-Glutathione	Amino acid	1	AT_Untreated_1_6	AT_Untreated_1	1.0	Glutathione derivatives	True	2.412e+06	1.359	1.359	1.002
5	GlcNAc 1-P	Carbon	1	AT_Untreated_1_6	AT_Untreated_1	0.833333333333333	Aminosugar biosynthesis	False			-3.236	-1.325
34	Ribitol	Carbon	1	AT_Untreated_1_6	AT_Untreated_1	1.0	Sugars and sugar alcohols	True	1.522e+05	-2.628	-2.628	0.8023
10	UDP-Galactose	Carbon	1	AT_Untreated_1_6	AT_Untreated_1	0.833333333333333	Polysaccharide biosynthesis	True	1.414e+06	0.5876	0.5876	1.214
13	Guanosine 5'-PP-Fucose	Carbon	1	AT_Untreated_1_6	AT_Untreated_1	1.0	Polysaccharide biosynthesis	True	1.735e+05	-2.439	-2.439	-0.1394

Metabolite ID	Name	Super Pathway	Dataset	Sample ID	Group ID	Detection Fraction	Pathway	Detected	Raw Intensity	Log2 Norm Intensity	Norm Imputed	Log2 Ctrl Norm
19	Maltotetraose	Carbon	1	AT_Untreated_1_6	AT_Untreated_1	1.0	Glycogen degradation	True	3.969e+06	2.077	2.077	2.121
233	SAM	Amino acid	1	AT_Untreated_1_6	AT_Untreated_1	0.333333333333333	SAM metabolism	False			-2.121	-1.965
129	5-Aminovalerate	Amino acid	1	AT_Untreated_1_6	AT_Untreated_1	0.333333333333333	Amino acids degradation intermediates	False			0.04171	-0.96
741	5-Me-THF	Cofactor	1	AT_Untreated_1_6	AT_Untreated_1	0.333333333333333	Folate metabolism	False			-5.366	-0.6085
198	Indolelactate	Amino acid	1	AT_Untreated_1_6	AT_Untreated_1	1.0	Amino acid derivatives	True	2.776e+05	-1.761	-1.761	2.026
254	Gly-Val	Amino acid	1	AT_Untreated_1_6	AT_Untreated_1	1.0	Dipeptides	True	8.934e+05	-0.07441	-0.07441	-1.186
291	gamma-Glu-Leu	Amino acid	1	AT_Untreated_1_6	AT_Untreated_1	1.0	Gamma-glutamyl dipeptides	True	5.708e+05	-0.7209	-0.7209	-0.2321
173	Met Sulfoxide	Amino acid	1	AT_Untreated_1_6	AT_Untreated_1	1.0	Amino acid derivatives	True	7.689e+06	3.031	3.031	0.4637
43	Glucose	Carbon	1	AT_Untreated_1_6	AT_Untreated_1	1.0	Glycolysis, GNG	True	9.585e+07	6.671	6.671	1.619
185	Phenyllactate	Amino acid	1	AT_Untreated_1_6	AT_Untreated_1	0.833333333333333	Amino acid derivatives	True	9.899e+04	-3.248	-3.248	2.563
156	Homo-Arg	Amino acid	1	AT_Untreated_1_6	AT_Untreated_1	1.0	Amino acid derivatives	True	5.835e+06	2.633	2.633	1.452
135	Homocitrulline	Amino acid	1	AT_Untreated_1_6	AT_Untreated_1	1.0	Amino acids degradation intermediates	True	6.114e+05	-0.6217	-0.6217	1.081
719	Nicotinamide MN	Cofactor	1	AT_Untreated_1_6	AT_Untreated_1	1.0	NAD biosynthesis	True	1.462e+05	-2.686	-2.686	-2.871
212	N-Ac-Asp	Amino acid	1	AT_Untreated_1_6	AT_Untreated_1	1.0	N-acetylated amino acids	True	7.381e+05	-0.3499	-0.3499	-1.047
720	1-Me-Nicotinamide	Cofactor	1	AT_Untreated_1_6	AT_Untreated_1	1.0	Derivatives of NA, nicotinamide and NAD	True	1.73e+08	7.523	7.523	-0.3678
216	N-Ac-Gly	Amino acid	1	AT_Untreated_1_6	AT_Untreated_1	0.5	N-acetylated amino acids	True	3.602e+05	-1.385	-1.385	2.155
70	Creatine	Carbon	1	AT_Untreated_1_6	AT_Untreated_1	1.0	Creatine energy storage	True	5.296e+08	9.137	9.137	-0.2802
26	Galactonate	Carbon	1	AT_Untreated_1_6	AT_Untreated_1	0.666666666666667	Sugars and sugar alcohols	True	7.276e+05	-0.3705	-0.3705	0.4341
309	Glutathione, Oxidized	Amino acid	1	AT_Untreated_1_6	AT_Untreated_1	1.0	Glutathione	True	2.006e+06	1.093	1.093	-0.1
35	Ribonate	Carbon	1	AT_Untreated_1_6	AT_Untreated_1	0.833333333333333	Sugars and sugar alcohols	True	6.671e+05	-0.4958	-0.4958	0.03149
160	1-Me-His	Amino acid	1	AT_Untreated_1_6	AT_Untreated_1	1.0	Amino acid derivatives	True	1.828e+07	4.28	4.28	0.8389

Metabolite ID	Name	Super Pathway	Dataset	Sample ID	Group ID	Detection Fraction	Pathway	Detected	Raw Intensity	Log2 Norm Intensity	Norm Imputed	Log2 Ctrl Norm
44	Glucose 6-P	Carbon	1	AT_Untreated_1_6	AT_Untreated_1	0.833333333333333	Glycolysis, GNG	False			-3.422	-1.946
704	NADH	Cofactor	1	AT_Untreated_1_6	AT_Untreated_1	1.0	Cofactors	True	1.08e+06	0.199	0.199	0.8451
275	Thr-Phe	Amino acid	1	AT_Untreated_1_6	AT_Untreated_1	0.833333333333333	Dipeptides	True	3.958e+05	-1.249	-1.249	0.4042
738	Pyridoxate	Cofactor	1	AT_Untreated_1_6	AT_Untreated_1	1.0	PLP biosynthesis and salvage	True	3.578e+05	-1.394	-1.394	1.97
177	3-(4-OH-Phenyl)Lactate	Amino acid	1	AT_Untreated_1_6	AT_Untreated_1	1.0	Amino acid derivatives	True	4.7e+05	-1.001	-1.001	1.464
206	Trans-4-OH-Pro	Amino acid	1	AT_Untreated_1_6	AT_Untreated_1	1.0	Amino acid derivatives	True	3.76e+07	5.321	5.321	0.1658
329	AMP	Nucleotide	1	AT_Untreated_1_6	AT_Untreated_1	1.0	Purine nucleotides	True	1.154e+07	3.617	3.617	-0.01843
11	UDP-Glucose	Carbon	1	AT_Untreated_1_6	AT_Untreated_1	0.833333333333333	Polysaccharide biosynthesis	True	1.916e+06	1.026	1.026	1.132
158	4-Imidazole-Ac	Amino acid	1	AT_Untreated_1_6	AT_Untreated_1	1.0	Amino acid derivatives	True	2.81e+05	-1.743	-1.743	0.4036
111	1-Me-Imidazole-Ac	Amino acid	1	AT_Untreated_1_6	AT_Untreated_1	1.0	Amino acids degradation intermediates	True	8.388e+05	-0.1653	-0.1653	1.768
345	Guanine	Nucleotide	1	AT_Untreated_1_6	AT_Untreated_1	1.0	Purine bases	True	5.645e+07	5.907	5.907	0.9208
22	N-Ac-Neuraminate	Carbon	1	AT_Untreated_1_6	AT_Untreated_1	1.0	Aminosugar derivatives	True	7.93e+05	-0.2465	-0.2465	-0.1111
721	N'-Methylnicotinate	Cofactor	1	AT_Untreated_1_6	AT_Untreated_1	1.0	Derivatives of NA, nicotinamide and NAD	True	4.046e+05	-1.217	-1.217	-0.2095
183	Phenol Sulfate	Amino acid	1	AT_Untreated_1_6	AT_Untreated_1	1.0	Amino acid derivatives	True	9.736e+04	-3.272	-3.272	1.368
718	Nicotinamide Riboside	Cofactor	1	AT_Untreated_1_6	AT_Untreated_1	1.0	NAD biosynthesis	True	2.215e+05	-2.086	-2.086	-3.676
297	gamma-Glu-Thr	Amino acid	1	AT_Untreated_1_6	AT_Untreated_1	1.0	Gamma-glutamyl dipeptides	True	7.417e+05	-0.3429	-0.3429	-0.983
295	gamma-Glu-Phe	Amino acid	1	AT_Untreated_1_6	AT_Untreated_1	0.166666666666667	Gamma-glutamyl dipeptides	False			-5.975	-0.1899
347	Allantoic Acid	Nucleotide	1	AT_Untreated_1_6	AT_Untreated_1	0.666666666666667	Purine degradation	True	7.138e+04	-3.72	-3.72	1.919
399	Pseudouridine	Nucleotide	1	AT_Untreated_1_6	AT_Untreated_1	1.0	Pyrimidine derivatives in RNAs	True	7.738e+05	-0.2817	-0.2817	1.748
375	UTP	Nucleotide	1	AT_Untreated_1_6	AT_Untreated_1	0.666666666666667	Pyrimidine nucleotides	True	2.842e+05	-1.727	-1.727	0.249
144	Glu, gamma-Me Ester	Amino acid	1	AT_Untreated_1_6	AT_Untreated_1	1.0	Amino acid derivatives	True	6.279e+05	-0.5831	-0.5831	-1.161

Metabolite ID	Name	Super Pathway	Dataset	Sample ID	Group ID	Detection Fraction	Pathway	Detected	Raw Intensity	Log2 Norm Intensity	Norm Imputed	Log2 Ctrl Norm
292	gamma-Glu-epsilon-Lysine	Amino acid	1	AT_Untreated_1_6	AT_Untreated_1	1.0	Gamma-glutamyl dipeptides	True	8.918e+05	-0.07701	-0.07701	0.1143
225	N-Ac-Thr	Amino acid	1	AT_Untreated_1_6	AT_Untreated_1	1.0	N-acetylated amino acids	True	1.992e+05	-2.239	-2.239	-0.7368
211	N-Ac-Asn	Amino acid	1	AT_Untreated_1_6	AT_Untreated_1	1.0	N-acetylated amino acids	True	1.966e+05	-2.258	-2.258	0.4269
151	Phenylacetylglycine	Amino acid	1	AT_Untreated_1_6	AT_Untreated_1	1.0	Amino acid derivatives	True	1.261e+06	0.4228	0.4228	1.21
217	N-Ac-His	Amino acid	1	AT_Untreated_1_6	AT_Untreated_1	0.833333333333333	N-acetylated amino acids	True	4.871e+04	-4.271	-4.271	-0.7866
288	gamma-Glu-Gly	Amino acid	1	AT_Untreated_1_6	AT_Untreated_1	0.333333333333333	Gamma-glutamyl dipeptides	False			-3.092	-1.268
222	N-Ac-Phe	Amino acid	1	AT_Untreated_1_6	AT_Untreated_1	0.333333333333333	N-acetylated amino acids	False			-6.698	-0.4255
71	Creatine-P	Carbon	1	AT_Untreated_1_6	AT_Untreated_1	1.0	Creatine energy storage	True	1.313e+05	-2.841	-2.841	1.415
210	N-Ac-Arg	Amino acid	1	AT_Untreated_1_6	AT_Untreated_1	0.666666666666667	N-acetylated amino acids	True	1.081e+05	-3.121	-3.121	-0.4458
218	N-Ac-Ile	Amino acid	1	AT_Untreated_1_6	AT_Untreated_1	0.166666666666667	N-acetylated amino acids	False			-6.52	-0.5541
251	Gly-Leu	Amino acid	1	AT_Untreated_1_6	AT_Untreated_1	1.0	Dipeptides	True	1.555e+06	0.7249	0.7249	-0.9138
290	gamma-Glu-Ile	Amino acid	1	AT_Untreated_1_6	AT_Untreated_1	1.0	Gamma-glutamyl dipeptides	True	2.741e+05	-1.779	-1.779	-0.4402
316	Ophthalmate	Amino acid	1	AT_Untreated_1_6	AT_Untreated_1	1.0	Oxidative stress markers	True	1.589e+06	0.7559	0.7559	-1.587
125	Isovaleryl-Gly	Amino acid	1	AT_Untreated_1_6	AT_Untreated_1	1.0	Amino acids degradation intermediates	True	6.156e+04	-3.934	-3.934	1.476
368	7-Me-Guanine	Nucleotide	1	AT_Untreated_1_6	AT_Untreated_1	1.0	Purine derivatives in RNAs	True	4.66e+05	-1.013	-1.013	1.177
208	Pro-OH-Pro	Amino acid	1	AT_Untreated_1_6	AT_Untreated_1	1.0	Amino acid derivatives	True	1.541e+07	4.034	4.034	0.7049
366	N2,N2-DiMe-Guanosine	Nucleotide	1	AT_Untreated_1_6	AT_Untreated_1	0.833333333333333	Purine derivatives in RNAs	True	7.562e+04	-3.637	-3.637	0.4768
352	3'-AMP	Nucleotide	1	AT_Untreated_1_6	AT_Untreated_1	1.0	Purine derivatives in signaling	True	2.711e+05	-1.795	-1.795	-0.3081
363	N6-Carbamoyl-Thr-Adenosine	Nucleotide	1	AT_Untreated_1_6	AT_Untreated_1	0.666666666666667	Purine derivatives in RNAs	True	3.878e+04	-4.6	-4.6	0.08497
314	Cys-Glutathione Disulfide	Amino acid	1	AT_Untreated_1_6	AT_Untreated_1	1.0	Oxidative stress markers	True	1.434e+05	-2.714	-2.714	0.5704
382	Orotidine	Nucleotide	1	AT_Untreated_1_6	AT_Untreated_1	0.166666666666667	Pyrimidine (UMP) biosynthesis	False			-5.287	-0.9952

Metabolite ID	Name	Super Pathway	Dataset	Sample ID	Group ID	Detection Fraction	Pathway	Detected	Raw Intensity	Log2 Norm Intensity	Norm Imputed	Log2 Ctrl Norm
307	Cys-Gly	Amino acid	1	AT_Untreated_1_6	AT_Untreated_1	1.0	Glutathione biosynthesis	True	2.424e+06	1.365	1.365	-0.2416
64	Sedoheptulose-7-P	Carbon	1	AT_Untreated_1_6	AT_Untreated_1	1.0	Pentose phosphate pathway (PPP)	True	8.637e+05	-0.1231	-0.1231	-0.2772
142	N-Ac-Asp-Glu	Amino acid	1	AT_Untreated_1_6	AT_Untreated_1	1.0	Amino acid derivatives	True	1.792e+05	-2.392	-2.392	-1.247
708	Thiamin-PP	Cofactor	1	AT_Untreated_1_6	AT_Untreated_1	0.5	Cofactors	False			-6.331	-0.3472
182	P-Cresol Sulfate	Amino acid	1	AT_Untreated_1_6	AT_Untreated_1	1.0	Amino acid derivatives	True	5.092e+05	-0.8856	-0.8856	2.311
250	Gly-Ile	Amino acid	1	AT_Untreated_1_6	AT_Untreated_1	1.0	Dipeptides	True	3.893e+05	-1.273	-1.273	0.04437
286	gamma-Glu-Glu	Amino acid	1	AT_Untreated_1_6	AT_Untreated_1	1.0	Gamma-glutamyl dipeptides	True	6.735e+05	-0.482	-0.482	-0.4998
739	Deoxycarnitine	Cofactor	1	AT_Untreated_1_6	AT_Untreated_1	1.0	Carnitine biosynthesis	True	5.504e+06	2.549	2.549	-1.203
203	DiMe-Arg	Amino acid	1	AT_Untreated_1_6	AT_Untreated_1	1.0	Amino acid derivatives	True	1.702e+07	4.178	4.178	-1.455
351	2'-AMP	Nucleotide	1	AT_Untreated_1_6	AT_Untreated_1	1.0	Purine derivatives in signaling	True	4.133e+04	-4.509	-4.509	-2.24
8	Cytidine 5'-P-N-Ac-Neuraminate	Carbon	1	AT_Untreated_1_6	AT_Untreated_1	1.0	Aminosugar biosynthesis	True	2.009e+05	-2.227	-2.227	-0.4819
285	gamma-Glu-Ala	Amino acid	1	AT_Untreated_1_6	AT_Untreated_1	0.5	Gamma-glutamyl dipeptides	False			-6.022	-2.783
224	N-Ac-Ser	Amino acid	1	AT_Untreated_1_6	AT_Untreated_1	1.0	N-acetylated amino acids	True	1.311e+06	0.4792	0.4792	-1.4
244	Ala-Leu	Amino acid	1	AT_Untreated_1_6	AT_Untreated_1	1.0	Dipeptides	True	3.06e+06	1.702	1.702	0.5636
207	N-Me-Pro	Amino acid	1	AT_Untreated_1_6	AT_Untreated_1	1.0	Amino acid derivatives	True	1.502e+06	0.6754	0.6754	1.605
171	Cys Sulfinic Acid	Amino acid	1	AT_Untreated_1_6	AT_Untreated_1	0.333333333333333	Amino acid derivatives	False			-5.211	-1.913
181	O-Me-Tyr	Amino acid	1	AT_Untreated_1_6	AT_Untreated_1	0.5	Amino acid derivatives	False			-4.309	-0.9396
240	N-Ac-Putrescine	Amino acid	1	AT_Untreated_1_6	AT_Untreated_1	0.833333333333333	Polyamine derivatives	True	4.733e+05	-0.991	-0.991	1.373
176	S-Me-Met	Amino acid	1	AT_Untreated_1_6	AT_Untreated_1	1.0	Amino acid derivatives	True	6.707e+05	-0.4881	-0.4881	1.354
339	AICAR	Nucleotide	1	AT_Untreated_1_6	AT_Untreated_1	0.333333333333333	IMP biosynthesis	False			-4.716	-0.4151
141	gamma-Carboxy-Glu	Amino acid	1	AT_Untreated_1_6	AT_Untreated_1	1.0	Amino acid derivatives	True	4.949e+05	-0.9265	-0.9265	-0.1415
392	3'-UMP	Nucleotide	1	AT_Untreated_1_6	AT_Untreated_1	0.833333333333333	Pyrimidine derivatives in signaling	False			-5.207	-1.174

Metabolite ID	Name	Super Pathway	Dataset	Sample ID	Group ID	Detection Fraction	Pathway	Detected	Raw Intensity	Log2 Norm Intensity	Norm Imputed	Log2 Ctrl Norm
355	3'-GMP	Nucleotide	1	AT_Untreated_1_6	AT_Untreated_1	0.833333333333333	Purine derivatives in signaling	True	9.393e+04	-3.324	-3.324	0.6132
282	Val-Leu	Amino acid	1	AT_Untreated_1_6	AT_Untreated_1	1.0	Dipeptides	True	4.386e+06	2.221	2.221	1.249
140	Carboxyethyl-GABA	Amino acid	1	AT_Untreated_1_6	AT_Untreated_1	1.0	Amino acid derivatives	True	3.507e+05	-1.424	-1.424	7.55e-03
258	Ile-Gly	Amino acid	1	AT_Untreated_1_6	AT_Untreated_1	1.0	Dipeptides	True	5.591e+06	2.571	2.571	-0.7407
260	Leu-Ala	Amino acid	1	AT_Untreated_1_6	AT_Untreated_1	1.0	Dipeptides	True	3.551e+06	1.916	1.916	0.6151
265	Lys-Leu	Amino acid	1	AT_Untreated_1_6	AT_Untreated_1	0.833333333333333	Dipeptides	True	2.529e+05	-1.895	-1.895	0.7161
263	Leu-Gly	Amino acid	1	AT_Untreated_1_6	AT_Untreated_1	1.0	Dipeptides	True	7.345e+06	2.965	2.965	-0.0562
281	Val-Gly	Amino acid	1	AT_Untreated_1_6	AT_Untreated_1	1.0	Dipeptides	True	1.488e+07	3.984	3.984	-0.5354
270	Pro-Gly	Amino acid	1	AT_Untreated_1_6	AT_Untreated_1	1.0	Dipeptides	True	1.157e+06	0.2982	0.2982	-0.4485
114	Imidazole Propionate	Amino acid	1	AT_Untreated_1_6	AT_Untreated_1	1.0	Amino acids degradation intermediates	True	5.825e+05	-0.6915	-0.6915	1.619
267	Phe-Gly	Amino acid	1	AT_Untreated_1_6	AT_Untreated_1	1.0	Dipeptides	True	7.962e+06	3.081	3.081	-1.012
266	Phe-Ala	Amino acid	1	AT_Untreated_1_6	AT_Untreated_1	1.0	Dipeptides	True	1.746e+06	0.8924	0.8924	-0.1843
278	Tyr-Gly	Amino acid	1	AT_Untreated_1_6	AT_Untreated_1	1.0	Dipeptides	True	2.82e+06	1.584	1.584	-0.5981
255	His-Ala	Amino acid	1	AT_Untreated_1_6	AT_Untreated_1	0.833333333333333	Dipeptides	True	1.346e+05	-2.805	-2.805	-1.468
280	Val-Gln	Amino acid	1	AT_Untreated_1_6	AT_Untreated_1	1.0	Dipeptides	True	2.834e+06	1.591	1.591	-0.02701
143	S-1-Pyrroline-5-Carboxylate	Amino acid	1	AT_Untreated_1_6	AT_Untreated_1	0.833333333333333	Amino acid derivatives	True	2.682e+05	-1.81	-1.81	0.2579
232	SAH	Amino acid	1	AT_Untreated_1_6	AT_Untreated_1	1.0	SAM metabolism	True	6.626e+05	-0.5056	-0.5056	-0.02021
20	Erythronate	Carbon	1	AT_Untreated_1_6	AT_Untreated_1	1.0	Aminosugar derivatives	True	2.61e+07	4.794	4.794	1.053
248	Gln-Leu	Amino acid	1	AT_Untreated_1_6	AT_Untreated_1	0.833333333333333	Dipeptides	True	4.362e+05	-1.109	-1.109	-0.2035
276	Trp-Gly	Amino acid	1	AT_Untreated_1_6	AT_Untreated_1	0.833333333333333	Dipeptides	True	2.033e+05	-2.21	-2.21	-1.132
205	N-delta-Ac-Ornithine	Amino acid	1	AT_Untreated_1_6	AT_Untreated_1	1.0	Amino acid derivatives	True	1.344e+06	0.5146	0.5146	1.636
163	Formimino-Glu	Amino acid	1	AT_Untreated_1_6	AT_Untreated_1	1.0	Amino acid derivatives	True	9.363e+05	-6.75e-03	-6.75e-03	1.233
204	N-Me-Arg	Amino acid	1	AT_Untreated_1_6	AT_Untreated_1	0.833333333333333	Amino acid derivatives	False			0.5113	-2.076
242	Guanidino-Ac	Amino acid	1	AT_Untreated_1_6	AT_Untreated_1	1.0	Creatine biosynthesis	True	3.617e+05	-1.379	-1.379	2.502

Metabolite ID	Name	Super Pathway	Dataset	Sample ID	Group ID	Detection Fraction	Pathway	Detected	Raw Intensity	Log2 Norm Intensity	Norm Imputed	Log2 Ctrl Norm
300	gamma-Glu-Val	Amino acid	1	AT_Untreated_1_6	AT_Untreated_1	0.333333333333333	Gamma-glutamyl dipeptides	True	9.564e+05	0.02383	0.02383	-0.5969
53	Ac-CoA	Carbon	1	AT_Untreated_1_6	AT_Untreated_1	0.166666666666667	Acetyl-CoA	False			-7.274	-0.5114
18	Maltotriose	Carbon	1	AT_Untreated_1_6	AT_Untreated_1	1.0	Glycogen degradation	True	2.741e+06	1.543	1.543	1.701
294	gamma-Glu-Met	Amino acid	1	AT_Untreated_1_6	AT_Untreated_1	0.333333333333333	Gamma-glutamyl dipeptides	False			-4.503	-1.021
174	Met Sulfone	Amino acid	1	AT_Untreated_1_6	AT_Untreated_1	0.833333333333333	Amino acid derivatives	True	1.237e+05	-2.927	-2.927	0.1515
175	N-Ac-Met Sulfoxide	Amino acid	1	AT_Untreated_1_6	AT_Untreated_1	1.0	Amino acid derivatives	True	9.207e+05	-0.03095	-0.03095	-7.28e-03
25	Mannitol/Sorbitol	Carbon	1	AT_Untreated_1_6	AT_Untreated_1	1.0	Sugars and sugar alcohols	True	1.561e+07	4.052	4.052	1.828
6	UDP-GlcNAc	Carbon	1	AT_Untreated_1_6	AT_Untreated_1	0.166666666666667	Aminosugar biosynthesis	False			-3.589	-0.4814
145	Pyro-Gln	Amino acid	1	AT_Untreated_1_6	AT_Untreated_1	1.0	Amino acid derivatives	True	2.877e+06	1.613	1.613	0.7728
705	Coenzyme A	Cofactor	1	AT_Untreated_1_6	AT_Untreated_1	0.666666666666667	Cofactors	True	1.32e+05	-2.833	-2.833	1.125
319	2'-dAMP	Nucleotide	1	AT_Untreated_1_6	AT_Untreated_1	0.166666666666667	Deoxy-nucleotides	True	1.772e+05	-2.408	-2.408	1.272
119	alpha-OH-Isovalerate	Amino acid	1	AT_Untreated_1_6	AT_Untreated_1	1.0	Amino acids degradation intermediates	True	5.764e+05	-0.7068	-0.7068	2.592
46	Fructose 1,6-PP / Glucose 1,6-PP / Inositol-1,4-PP / Inositol-1,3-PP	Carbon	1	AT_Untreated_1_6	AT_Untreated_1	1.0	Glycolysis, GNG	True	4.915e+07	5.707	5.707	0.8761
137	1-Me-Guanidine	Amino acid	1	AT_Untreated_1_6	AT_Untreated_1	0.666666666666667	Amino acids degradation intermediates	True	7.159e+04	-3.716	-3.716	1.437
23	N-GlcNAc-Asn	Carbon	1	AT_Untreated_1_6	AT_Untreated_1	1.0	Aminosugar derivatives	True	6.599e+05	-0.5115	-0.5115	-1.224
262	Leu-Gln	Amino acid	1	AT_Untreated_1_6	AT_Untreated_1	1.0	Dipeptides	True	3.515e+06	1.902	1.902	0.6056
24	Fructose	Carbon	1	AT_Untreated_1_6	AT_Untreated_1	1.0	Sugars and sugar alcohols	True	2.065e+07	4.457	4.457	2.508
197	C-Glycosyl-Trp	Amino acid	1	AT_Untreated_1_6	AT_Untreated_1	1.0	Amino acid derivatives	True	3.343e+05	-1.493	-1.493	-1.194
33	Arabitol/Xylitol	Carbon	1	AT_Untreated_1_6	AT_Untreated_1	1.0	Sugars and sugar alcohols	True	3.213e+05	-1.55	-1.55	0.3101

Metabolite ID	Name	Super Pathway	Dataset	Sample ID	Group ID	Detection Fraction	Pathway	Detected	Raw Intensity	Log2 Norm Intensity	Norm Imputed	Log2 Ctrl Norm
128	N2-Ac-Lys/N6-Ac-Lys	Amino acid	1	AT_Untreated_1_6	AT_Untreated_1	1.0	Amino acids degradation intermediates	True	6.939e+05	-0.4391	-0.4391	-1.649
42	2-Me-Citrate	Carbon	1	AT_Untreated_1_6	AT_Untreated_1	0.833333333333333	Propionate metabolism	True	4.507e+04	-4.383	-4.383	-5.03e-04
12	Glucuronate 1-P	Carbon	1	AT_Untreated_1_6	AT_Untreated_1	1.0	Polysaccharide biosynthesis	True	3.663e+05	-1.361	-1.361	-0.1178
76	Gln	Amino acid	1	Ctrl_Untreated_1_3	Ctrl_Untreated_1	1.0	Proteinogenic amino acids	True	6.557e+08	8.223	8.223	-0.4579
89	Trp	Amino acid	1	Ctrl_Untreated_1_3	Ctrl_Untreated_1	1.0	Proteinogenic amino acids	True	9.609e+07	5.452	5.452	-0.2237
723	beta-Ala	Cofactor	1	Ctrl_Untreated_1_3	Ctrl_Untreated_1	1.0	Coenzyme A biosynthesis	True	1.672e+07	2.93	2.93	-0.07963
75	Glu	Amino acid	1	Ctrl_Untreated_1_3	Ctrl_Untreated_1	1.0	Proteinogenic amino acids	True	2.863e+09	10.35	10.35	-0.3877
77	Gly	Amino acid	1	Ctrl_Untreated_1_3	Ctrl_Untreated_1	1.0	Proteinogenic amino acids	True	1.072e+08	5.61	5.61	-5.23e-03
80	His	Amino acid	1	Ctrl_Untreated_1_3	Ctrl_Untreated_1	1.0	Proteinogenic amino acids	True	1.89e+07	3.106	3.106	-0.3093
82	Leu	Amino acid	1	Ctrl_Untreated_1_3	Ctrl_Untreated_1	1.0	Proteinogenic amino acids	True	1.469e+09	9.386	9.386	0.1648
87	Phe	Amino acid	1	Ctrl_Untreated_1_3	Ctrl_Untreated_1	1.0	Proteinogenic amino acids	True	9.49e+08	8.756	8.756	-0.05536
130	Glutarate	Amino acid	1	Ctrl_Untreated_1_3	Ctrl_Untreated_1	1.0	Amino acids degradation intermediates	True	2.363e+05	-3.216	-3.216	-0.7889
196	5-OH-Indole-Ac	Amino acid	1	Ctrl_Untreated_1_3	Ctrl_Untreated_1	0.857142857142857	Amino acid derivatives	False			-5.134	-1.122
74	Asp	Amino acid	1	Ctrl_Untreated_1_3	Ctrl_Untreated_1	1.0	Proteinogenic amino acids	True	5.175e+08	7.881	7.881	-0.01044
236	Spermidine	Amino acid	1	Ctrl_Untreated_1_3	Ctrl_Untreated_1	1.0	Polyamines	True	2.952e+07	3.749	3.749	-0.1124
73	Asn	Amino acid	1	Ctrl_Untreated_1_3	Ctrl_Untreated_1	1.0	Proteinogenic amino acids	True	2.033e+08	6.533	6.533	-0.1381
243	Creatinine	Amino acid	1	Ctrl_Untreated_1_3	Ctrl_Untreated_1	1.0	Creatine degradation	True	3.545e+07	4.013	4.013	-0.9889
376	Cytidine	Nucleotide	1	Ctrl_Untreated_1_3	Ctrl_Untreated_1	0.714285714285714	Pyrimidine nucleosides	True	2.868e+07	3.708	3.708	2.98
41	Lactate	Carbon	1	Ctrl_Untreated_1_3	Ctrl_Untreated_1	1.0	Respiratory carbon sources	True	1.318e+08	5.908	5.908	-0.5733
58	alpha-Ketoglutarate	Carbon	1	Ctrl_Untreated_1_3	Ctrl_Untreated_1	1.0	TCA cycle	True	9.592e+05	-1.194	-1.194	-1.211
69	3-OH-Butyrate	Carbon	1	Ctrl_Untreated_1_3	Ctrl_Untreated_1	0.571428571428571	Ketone bodies	False			-3.497	-0.5127

Metabolite ID	Name	Super Pathway	Dataset	Sample ID	Group ID	Detection Fraction	Pathway	Detected	Raw Intensity	Log2 Norm Intensity	Norm Imputed	Log2 Ctrl Norm
343	Adenine	Nucleotide	1	Ctrl_Untreated_1_3	Ctrl_Untreated_1	1.0	Purine bases	True	4.175e+06	0.9274	0.9274	-0.5295
336	Adenosine	Nucleotide	1	Ctrl_Untreated_1_3	Ctrl_Untreated_1	1.0	Purine nucleosides	True	1.507e+07	2.779	2.779	-0.6439
722	ADP-Ribose	Cofactor	1	Ctrl_Untreated_1_3	Ctrl_Untreated_1	1.0	Derivatives of NA, nicotinamide and NAD	True	5.108e+04	-5.425	-5.425	-2.423
383	Cytosine	Nucleotide	1	Ctrl_Untreated_1_3	Ctrl_Untreated_1	0.571428571428571	Pyrimidine bases	True	5.493e+04	-5.32	-5.32	-0.4652
3	Glucosamine 6-P	Carbon	1	Ctrl_Untreated_1_3	Ctrl_Untreated_1	1.0	Aminosugar biosynthesis	True	1.646e+05	-3.737	-3.737	-2.07
717	Nicotinamide	Cofactor	1	Ctrl_Untreated_1_3	Ctrl_Untreated_1	1.0	NAD biosynthesis	True	2.496e+07	3.507	3.507	-1.008
51	PEP	Carbon	1	Ctrl_Untreated_1_3	Ctrl_Untreated_1	1.0	Glycolysis, GNG	True	8.342e+06	1.926	1.926	0.875
237	Spermine	Amino acid	1	Ctrl_Untreated_1_3	Ctrl_Untreated_1	1.0	Polyamines	True	1.814e+06	-0.2747	-0.2747	1.216
385	Uracil	Nucleotide	1	Ctrl_Untreated_1_3	Ctrl_Untreated_1	1.0	Pyrimidine bases	True	1.588e+07	2.854	2.854	2.59
377	Uridine	Nucleotide	1	Ctrl_Untreated_1_3	Ctrl_Untreated_1	1.0	Pyrimidine nucleosides	True	7.268e+07	5.049	5.049	1
348	Allantoin	Nucleotide	1	Ctrl_Untreated_1_3	Ctrl_Untreated_1	1.0	Purine degradation	True	7.208e+05	-1.607	-1.607	-1.431
335	Inosine	Nucleotide	1	Ctrl_Untreated_1_3	Ctrl_Untreated_1	1.0	Purine nucleosides	True	1.104e+08	5.652	5.652	0.153
81	Ile	Amino acid	1	Ctrl_Untreated_1_3	Ctrl_Untreated_1	1.0	Proteinogenic amino acids	True	8.413e+08	8.582	8.582	-0.1329
72	Ala	Amino acid	1	Ctrl_Untreated_1_3	Ctrl_Untreated_1	1.0	Proteinogenic amino acids	True	6.066e+08	8.11	8.11	-0.3909
79	Thr	Amino acid	1	Ctrl_Untreated_1_3	Ctrl_Untreated_1	1.0	Proteinogenic amino acids	True	5.244e+08	7.9	7.9	-0.1093
88	Tyr	Amino acid	1	Ctrl_Untreated_1_3	Ctrl_Untreated_1	1.0	Proteinogenic amino acids	True	4.582e+08	7.706	7.706	0.1819
84	Lys	Amino acid	1	Ctrl_Untreated_1_3	Ctrl_Untreated_1	1.0	Proteinogenic amino acids	True	5.425e+08	7.949	7.949	-0.0408
86	Met	Amino acid	1	Ctrl_Untreated_1_3	Ctrl_Untreated_1	1.0	Proteinogenic amino acids	True	7.351e+08	8.388	8.388	0.1598
61	Malate	Carbon	1	Ctrl_Untreated_1_3	Ctrl_Untreated_1	1.0	TCA cycle	True	2.473e+08	6.816	6.816	-0.356
235	Putrescine	Amino acid	1	Ctrl_Untreated_1_3	Ctrl_Untreated_1	1.0	Polyamines	True	2.402e+06	0.1298	0.1298	0.621
324	2'-dU	Nucleotide	1	Ctrl_Untreated_1_3	Ctrl_Untreated_1	0.714285714285714	Deoxy-nucleosides	True	2.741e+05	-3.001	-3.001	1.379
49	3-P-Glycerate	Carbon	1	Ctrl_Untreated_1_3	Ctrl_Untreated_1	1.0	Glycolysis, GNG	True	3.16e+07	3.848	3.848	0.5822
189	Kynurenate	Amino acid	1	Ctrl_Untreated_1_3	Ctrl_Untreated_1	0.714285714285714	Amino acid derivatives	False			-6.319	-0.5206
234	5-Me-Thioadenosine	Amino acid	1	Ctrl_Untreated_1_3	Ctrl_Untreated_1	1.0	SAM metabolism	True	4.265e+06	0.9582	0.9582	-0.9273
59	Succinate	Carbon	1	Ctrl_Untreated_1_3	Ctrl_Untreated_1	1.0	TCA cycle	True	1.281e+06	-0.7767	-0.7767	-1.078

Metabolite ID	Name	Super Pathway	Dataset	Sample ID	Group ID	Detection Fraction	Pathway	Detected	Raw Intensity	Log2 Norm Intensity	Norm Imputed	Log2 Ctrl Norm
36	Ribose	Carbon	1	Ctrl_Untreated_1_3	Ctrl_Untreated_1	1.0	Sugars and sugar alcohols	True	1.641e+06	-0.4198	-0.4198	0.8136
133	Ornithine	Amino acid	1	Ctrl_Untreated_1_3	Ctrl_Untreated_1	1.0	Amino acids degradation intermediates	True	2.098e+07	3.257	3.257	-1.223
313	5-Oxoproline	Amino acid	1	Ctrl_Untreated_1_3	Ctrl_Untreated_1	1.0	Glutathione derivatives	True	6.986e+06	1.67	1.67	-0.4173
165	N-6-Tri-Me-Lys	Amino acid	1	Ctrl_Untreated_1_3	Ctrl_Untreated_1	1.0	Amino acid derivatives	True	3.829e+07	4.125	4.125	0.361
380	Orotate	Nucleotide	1	Ctrl_Untreated_1_3	Ctrl_Untreated_1	0.857142857142857	Pyrimidine (UMP) biosynthesis	True	9.428e+04	-4.541	-4.541	-0.9111
724	Pantothenate	Cofactor	1	Ctrl_Untreated_1_3	Ctrl_Untreated_1	1.0	Coenzyme A biosynthesis	True	8.188e+07	5.221	5.221	-1.089
150	N-Me-Gly	Amino acid	1	Ctrl_Untreated_1_3	Ctrl_Untreated_1	1.0	Amino acid derivatives	True	1.596e+06	-0.4597	-0.4597	-0.1593
122	3-OH-Isobutyrate	Amino acid	1	Ctrl_Untreated_1_3	Ctrl_Untreated_1	0.857142857142857	Amino acids degradation intermediates	False			-3.901	-0.7452
241	4-Acetamidobutanoate	Amino acid	1	Ctrl_Untreated_1_3	Ctrl_Untreated_1	1.0	Polyamine derivatives	True	1.11e+06	-0.9839	-0.9839	-1.425
711	alpha-Tocopherol	Cofactor	1	Ctrl_Untreated_1_3	Ctrl_Untreated_1	1.0	Cofactors	True	5.775e+06	1.395	1.395	0.6151
55	Citrate	Carbon	1	Ctrl_Untreated_1_3	Ctrl_Untreated_1	1.0	TCA cycle	True	8.985e+06	2.033	2.033	0.08414
387	3-Aminoisobutyrate	Nucleotide	1	Ctrl_Untreated_1_3	Ctrl_Untreated_1	0.857142857142857	Pyrimidine degradation	False			-2.626	-0.6123
338	Guanosine	Nucleotide	1	Ctrl_Untreated_1_3	Ctrl_Untreated_1	1.0	Purine nucleosides	True	1.208e+08	5.782	5.782	0.6758
209	N-Ac-Ala	Amino acid	1	Ctrl_Untreated_1_3	Ctrl_Untreated_1	1.0	N-acetylated amino acids	True	9.984e+05	-1.137	-1.137	0.06464
221	N-Ac-Met	Amino acid	1	Ctrl_Untreated_1_3	Ctrl_Untreated_1	1.0	N-acetylated amino acids	True	8.87e+06	2.015	2.015	0.2705
228	N-Ac-Val	Amino acid	1	Ctrl_Untreated_1_3	Ctrl_Untreated_1	0.571428571428571	N-acetylated amino acids	False			-5.82	-0.449
346	Urate	Nucleotide	1	Ctrl_Untreated_1_3	Ctrl_Untreated_1	1.0	Purine degradation	True	3.913e+05	-2.488	-2.488	-1.425
90	Arg	Amino acid	1	Ctrl_Untreated_1_3	Ctrl_Untreated_1	1.0	Proteinogenic amino acids	True	2.195e+09	9.966	9.966	0.26
60	Fumarate	Carbon	1	Ctrl_Untreated_1_3	Ctrl_Untreated_1	1.0	TCA cycle	True	3.782e+06	0.7851	0.7851	-0.2301
78	Ser	Amino acid	1	Ctrl_Untreated_1_3	Ctrl_Untreated_1	1.0	Proteinogenic amino acids	True	4.546e+08	7.694	7.694	0.4369
83	Val	Amino acid	1	Ctrl_Untreated_1_3	Ctrl_Untreated_1	1.0	Proteinogenic amino acids	True	5.533e+08	7.978	7.978	0.1053
734	Pyridoxal	Cofactor	1	Ctrl_Untreated_1_3	Ctrl_Untreated_1	1.0	PLP biosynthesis and salvage	True	3.982e+06	0.8593	0.8593	-0.9238

Metabolite ID	Name	Super Pathway	Dataset	Sample ID	Group ID	Detection Fraction	Pathway	Detected	Raw Intensity	Log2 Norm Intensity	Norm Imputed	Log2 Ctrl Norm
136	Urea	Amino acid	1	Ctrl_Untreated_1_3	Ctrl_Untreated_1	0.857142857142857	Amino acids degradation intermediates	False			-0.9089	-1.06
67	Ribose 1-P	Carbon	1	Ctrl_Untreated_1_3	Ctrl_Untreated_1	1.0	Pentose phosphate pathway (PPP)	True	2.821e+06	0.362	0.362	-0.2592
284	Carnosine	Amino acid	1	Ctrl_Untreated_1_3	Ctrl_Untreated_1	1.0	Dipeptides	True	2.005e+05	-3.452	-3.452	-1.344
306	gamma-Glu-Cys	Amino acid	1	Ctrl_Untreated_1_3	Ctrl_Untreated_1	1.0	Glutathione biosynthesis	True	8.718e+05	-1.332	-1.332	-0.9954
712	Retinol (Vit A)	Cofactor	1	Ctrl_Untreated_1_3	Ctrl_Untreated_1	1.0	Cofactors	True	3.902e+05	-2.492	-2.492	-0.05885
85	Cys	Amino acid	1	Ctrl_Untreated_1_3	Ctrl_Untreated_1	1.0	Proteinogenic amino acids	True	3.935e+07	4.164	4.164	-0.3495
91	Pro	Amino acid	1	Ctrl_Untreated_1_3	Ctrl_Untreated_1	1.0	Proteinogenic amino acids	True	1.256e+09	9.161	9.161	-0.2473
308	Glutathione, Reduced	Amino acid	1	Ctrl_Untreated_1_3	Ctrl_Untreated_1	1.0	Glutathione	True	2.028e+08	6.53	6.53	-0.4265
107	Citrulline	Amino acid	1	Ctrl_Untreated_1_3	Ctrl_Untreated_1	1.0	Amino acids biosynthesis intermediates	True	8.735e+06	1.993	1.993	-0.8318
328	IMP	Nucleotide	1	Ctrl_Untreated_1_3	Ctrl_Untreated_1	0.285714285714286	Purine nucleotides	False			-4.53	-0.2088
706	FAD	Cofactor	1	Ctrl_Untreated_1_3	Ctrl_Untreated_1	1.0	Cofactors	True	3.649e+05	-2.588	-2.588	-0.3989
735	Pyridoxamine	Cofactor	1	Ctrl_Untreated_1_3	Ctrl_Untreated_1	1.0	PLP biosynthesis and salvage	True	1.127e+06	-0.9618	-0.9618	-0.7688
199	Serotonin	Amino acid	1	Ctrl_Untreated_1_3	Ctrl_Untreated_1	1.0	Amino acid derivatives	True	4.036e+06	0.8788	0.8788	-0.3939
370	CMP	Nucleotide	1	Ctrl_Untreated_1_3	Ctrl_Untreated_1	1.0	Pyrimidine nucleotides	True	3.902e+06	0.8299	0.8299	-1.009
287	gamma-Glu-Gln	Amino acid	1	Ctrl_Untreated_1_3	Ctrl_Untreated_1	1.0	Gamma-glutamyl dipeptides	True	1.217e+07	2.471	2.471	1.63
14	UDP-Glucuronate	Carbon	1	Ctrl_Untreated_1_3	Ctrl_Untreated_1	0.857142857142857	Polysaccharide biosynthesis	True	2.632e+05	-3.06	-3.06	-1.111
229	N-Formyl-Met	Amino acid	1	Ctrl_Untreated_1_3	Ctrl_Untreated_1	1.0	N-formylated amino acids	True	1.532e+05	-3.841	-3.841	-0.01877
350	3',5'-cAMP	Nucleotide	1	Ctrl_Untreated_1_3	Ctrl_Untreated_1	1.0	Purine derivatives in signaling	True	2.238e+05	-3.294	-3.294	0.3253
371	CDP	Nucleotide	1	Ctrl_Untreated_1_3	Ctrl_Untreated_1	0.571428571428571	Pyrimidine nucleotides	False			-5.544	-2.224
372	CTP	Nucleotide	1	Ctrl_Untreated_1_3	Ctrl_Untreated_1	0.571428571428571	Pyrimidine nucleotides	False			-4.713	-1.059
333	GDP	Nucleotide	1	Ctrl_Untreated_1_3	Ctrl_Untreated_1	0.571428571428571	Purine nucleotides	False			-4.484	-1.983
332	GMP	Nucleotide	1	Ctrl_Untreated_1_3	Ctrl_Untreated_1	1.0	Purine nucleotides	True	1.047e+06	-1.067	-1.067	-1.766

Metabolite ID	Name	Super Pathway	Dataset	Sample ID	Group ID	Detection Fraction	Pathway	Detected	Raw Intensity	Log2 Norm Intensity	Norm Imputed	Log2 Ctrl Norm
373	UMP	Nucleotide	1	Ctrl_Untreated_1_3	Ctrl_Untreated_1	1.0	Pyrimidine nucleotides	True	1.04e+05	-4.4	-4.4	-3.345
389	3'-CMP	Nucleotide	1	Ctrl_Untreated_1_3	Ctrl_Untreated_1	1.0	Pyrimidine derivatives in signaling	True	2.743e+06	0.3213	0.3213	1.373
330	ADP	Nucleotide	1	Ctrl_Untreated_1_3	Ctrl_Untreated_1	0.714285714285714	Purine nucleotides	False			-2.826	-2.53
342	Hypoxanthine	Nucleotide	1	Ctrl_Untreated_1_3	Ctrl_Untreated_1	1.0	Purine bases	True	5.745e+07	4.71	4.71	0.7706
736	Pyridoxamine-P	Cofactor	1	Ctrl_Untreated_1_3	Ctrl_Untreated_1	1.0	PLP biosynthesis and salvage	True	2.171e+05	-3.338	-3.338	-0.3596
148	Betaine	Amino acid	1	Ctrl_Untreated_1_3	Ctrl_Untreated_1	1.0	Amino acid derivatives	True	3.441e+07	3.97	3.97	-0.8354
344	Xanthine	Nucleotide	1	Ctrl_Untreated_1_3	Ctrl_Untreated_1	1.0	Purine bases	True	8.854e+05	-1.31	-1.31	-2.217
386	3-Ureidopropionate	Nucleotide	1	Ctrl_Untreated_1_3	Ctrl_Untreated_1	1.0	Pyrimidine degradation	True	5.081e+05	-2.111	-2.111	-1.192
149	DiMe-Gly	Amino acid	1	Ctrl_Untreated_1_3	Ctrl_Untreated_1	1.0	Amino acid derivatives	True	6.658e+05	-1.721	-1.721	-1.201
703	NAD+	Cofactor	1	Ctrl_Untreated_1_3	Ctrl_Untreated_1	1.0	Cofactors	True	8.725e+06	1.991	1.991	-0.7454
709	Pyridoxal-P	Cofactor	1	Ctrl_Untreated_1_3	Ctrl_Untreated_1	1.0	Cofactors	True	2.339e+05	-3.231	-3.231	-0.9226
731	Thiamin (Vitamin B1)	Cofactor	1	Ctrl_Untreated_1_3	Ctrl_Untreated_1	1.0	TPP biosynthesis	True	2.245e+06	0.03267	0.03267	-0.715
374	UDP	Nucleotide	1	Ctrl_Untreated_1_3	Ctrl_Untreated_1	0.714285714285714	Pyrimidine nucleotides	False			-5.339	-3.785
102	2-Aminoadipate	Amino acid	1	Ctrl_Untreated_1_3	Ctrl_Untreated_1	1.0	Amino acids biosynthesis intermediates	True	2.725e+05	-3.01	-3.01	-0.9003
45	Fructose-6-P	Carbon	1	Ctrl_Untreated_1_3	Ctrl_Untreated_1	1.0	Glycolysis, GNG	True	1.101e+06	-0.9958	-0.9958	-1.071
320	TMP	Nucleotide	1	Ctrl_Untreated_1_3	Ctrl_Untreated_1	0.571428571428571	Deoxy-nucleotides	False			-6.992	-2.2
341	XMP	Nucleotide	1	Ctrl_Untreated_1_3	Ctrl_Untreated_1	0.857142857142857	IMP conversion to AMP & GMP	True	9.858e+04	-4.477	-4.477	0.09761
120	beta-OH-Isovalerate	Amino acid	1	Ctrl_Untreated_1_3	Ctrl_Untreated_1	0.857142857142857	Amino acids degradation intermediates	False			-4.68	-1.515
322	2'-dI	Nucleotide	1	Ctrl_Untreated_1_3	Ctrl_Untreated_1	0.857142857142857	Deoxy-nucleosides	True	3.692e+05	-2.572	-2.572	1.554
4	GlcNAc 6-P	Carbon	1	Ctrl_Untreated_1_3	Ctrl_Untreated_1	1.0	Aminosugar biosynthesis	True	3.735e+06	0.7669	0.7669	0.4383
337	Xanthosine	Nucleotide	1	Ctrl_Untreated_1_3	Ctrl_Untreated_1	1.0	Purine nucleosides	True	1.351e+05	-4.022	-4.022	-0.9479
188	Kynurenine	Amino acid	1	Ctrl_Untreated_1_3	Ctrl_Untreated_1	1.0	Amino acid derivatives	True	7.536e+05	-1.542	-1.542	-0.8509

Metabolite ID	Name	Super Pathway	Dataset	Sample ID	Group ID	Detection Fraction	Pathway	Detected	Raw Intensity	Log2 Norm Intensity	Norm Imputed	Log2 Ctrl Norm
63	6-P-Gluconate	Carbon	1	Ctrl_Untreated_1_3	Ctrl_Untreated_1	1.0	Pentose phosphate pathway (PPP)	True	3.824e+06	0.8008	0.8008	-1.233
40	Glucuronate	Carbon	1	Ctrl_Untreated_1_3	Ctrl_Untreated_1	1.0	Sugars and sugar alcohols	True	8.383e+05	-1.389	-1.389	0.1504
108	Argininosuccinate	Amino acid	1	Ctrl_Untreated_1_3	Ctrl_Untreated_1	1.0	Amino acids biosynthesis intermediates	True	1.163e+06	-0.9159	-0.9159	-0.6897
710	Carnitine	Cofactor	1	Ctrl_Untreated_1_3	Ctrl_Untreated_1	1.0	Cofactors	True	9.872e+07	5.491	5.491	-0.4864
725	P-Pantetheine	Cofactor	1	Ctrl_Untreated_1_3	Ctrl_Untreated_1	1.0	Coenzyme A biosynthesis	True	8.013e+04	-4.776	-4.776	-1.071
48	DHAP	Carbon	1	Ctrl_Untreated_1_3	Ctrl_Untreated_1	1.0	Glycolysis, GNG	True	2.757e+07	3.651	3.651	0.5543
17	Maltose	Carbon	1	Ctrl_Untreated_1_3	Ctrl_Untreated_1	1.0	Glycogen degradation	True	4.139e+05	-2.407	-2.407	-1.114
359	N1-Me-Adenosine	Nucleotide	1	Ctrl_Untreated_1_3	Ctrl_Untreated_1	0.857142857142857	Purine derivatives in RNAs	True	8.695e+05	-1.336	-1.336	-0.2983
159	3-Me-His	Amino acid	1	Ctrl_Untreated_1_3	Ctrl_Untreated_1	1.0	Amino acid derivatives	True	3.647e+04	-5.911	-5.911	-1.676
155	4-Guanidinobutanoate	Amino acid	1	Ctrl_Untreated_1_3	Ctrl_Untreated_1	1.0	Amino acid derivatives	True	6.06e+05	-1.857	-1.857	-0.4228
164	5-OH-Lys	Amino acid	1	Ctrl_Untreated_1_3	Ctrl_Untreated_1	1.0	Amino acid derivatives	True	2.036e+05	-3.43	-3.43	-1.66
357	Adenosine-3',5'-PP	Nucleotide	1	Ctrl_Untreated_1_3	Ctrl_Untreated_1	0.857142857142857	Purine byproducts of metabolic processes	True	3.63e+05	-2.596	-2.596	0.8811
104	Cystathionine	Amino acid	1	Ctrl_Untreated_1_3	Ctrl_Untreated_1	1.0	Amino acids biosynthesis intermediates	True	5.892e+06	1.425	1.425	-0.785
113	Imidazole Lactate	Amino acid	1	Ctrl_Untreated_1_3	Ctrl_Untreated_1	0.857142857142857	Amino acids degradation intermediates	False			-4.646	-0.9699
215	N-Ac-Glu	Amino acid	1	Ctrl_Untreated_1_3	Ctrl_Untreated_1	1.0	N-acetylated amino acids	True	1.187e+07	2.435	2.435	0.691
310	S-Lactoyl-Glutathione	Amino acid	1	Ctrl_Untreated_1_3	Ctrl_Untreated_1	0.857142857142857	Glutathione derivatives	True	4.181e+06	0.9295	0.9295	0.5731
5	GlcNAc 1-P	Carbon	1	Ctrl_Untreated_1_3	Ctrl_Untreated_1	1.0	Aminosugar biosynthesis	True	5.696e+05	-1.946	-1.946	-0.03525
34	Ribitol	Carbon	1	Ctrl_Untreated_1_3	Ctrl_Untreated_1	1.0	Sugars and sugar alcohols	True	1.05e+05	-4.385	-4.385	-0.9553
10	UDP-Galactose	Carbon	1	Ctrl_Untreated_1_3	Ctrl_Untreated_1	0.857142857142857	Polysaccharide biosynthesis	True	1.716e+06	-0.3555	-0.3555	0.271
13	Guanosine 5'-PP-Fucose	Carbon	1	Ctrl_Untreated_1_3	Ctrl_Untreated_1	1.0	Polysaccharide biosynthesis	True	2.8e+05	-2.971	-2.971	-0.6717

Metabolite ID	Name	Super Pathway	Dataset	Sample ID	Group ID	Detection Fraction	Pathway	Detected	Raw Intensity	Log2 Norm Intensity	Norm Imputed	Log2 Ctrl Norm
19	Maltotetraose	Carbon	1	Ctrl_Untreated_1_3	Ctrl_Untreated_1	1.0	Glycogen degradation	True	6.42e+05	-1.774	-1.774	-1.73
233	SAM	Amino acid	1	Ctrl_Untreated_1_3	Ctrl_Untreated_1	1.0	SAM metabolism	True	9.283e+05	-1.242	-1.242	-1.085
129	5-Aminovalerate	Amino acid	1	Ctrl_Untreated_1_3	Ctrl_Untreated_1	1.0	Amino acids degradation intermediates	True	7.058e+06	1.685	1.685	0.6834
741	5-Me-THF	Cofactor	1	Ctrl_Untreated_1_3	Ctrl_Untreated_1	0.714285714285714	Folate metabolism	False			-5.366	-0.6085
198	Indolelactate	Amino acid	1	Ctrl_Untreated_1_3	Ctrl_Untreated_1	0.857142857142857	Amino acid derivatives	False			-4.719	-0.9322
254	Gly-Val	Amino acid	1	Ctrl_Untreated_1_3	Ctrl_Untreated_1	1.0	Dipeptides	True	6.504e+06	1.567	1.567	0.4557
291	gamma-Glu-Leu	Amino acid	1	Ctrl_Untreated_1_3	Ctrl_Untreated_1	1.0	Gamma-glutamyl dipeptides	True	1.022e+06	-1.103	-1.103	-0.6145
173	Met Sulfoxide	Amino acid	1	Ctrl_Untreated_1_3	Ctrl_Untreated_1	1.0	Amino acid derivatives	True	1.469e+07	2.743	2.743	0.1752
43	Glucose	Carbon	1	Ctrl_Untreated_1_3	Ctrl_Untreated_1	1.0	Glycolysis, GNG	True	1.169e+08	5.735	5.735	0.6834
185	Phenyllactate	Amino acid	1	Ctrl_Untreated_1_3	Ctrl_Untreated_1	0.285714285714286	Amino acid derivatives	False			-5.917	-0.1051
156	Homo-Arg	Amino acid	1	Ctrl_Untreated_1_3	Ctrl_Untreated_1	1.0	Amino acid derivatives	True	2.226e+06	0.02017	0.02017	-1.16
135	Homocitrulline	Amino acid	1	Ctrl_Untreated_1_3	Ctrl_Untreated_1	1.0	Amino acids degradation intermediates	True	3.282e+05	-2.742	-2.742	-1.039
719	Nicotinamide MN	Cofactor	1	Ctrl_Untreated_1_3	Ctrl_Untreated_1	1.0	NAD biosynthesis	True	9.054e+06	2.044	2.044	1.859
212	N-Ac-Asp	Amino acid	1	Ctrl_Untreated_1_3	Ctrl_Untreated_1	1.0	N-acetylated amino acids	True	3.408e+06	0.6345	0.6345	-0.06285
720	1-Me-Nicotinamide	Cofactor	1	Ctrl_Untreated_1_3	Ctrl_Untreated_1	1.0	Derivatives of NA, nicotinamide and NAD	True	3.575e+08	7.348	7.348	-0.5431
216	N-Ac-Gly	Amino acid	1	Ctrl_Untreated_1_3	Ctrl_Untreated_1	0.714285714285714	N-acetylated amino acids	False			-4.155	-0.6151
70	Creatine	Carbon	1	Ctrl_Untreated_1_3	Ctrl_Untreated_1	1.0	Creatine energy storage	True	1.348e+09	9.262	9.262	-0.1551
26	Galactonate	Carbon	1	Ctrl_Untreated_1_3	Ctrl_Untreated_1	0.857142857142857	Sugars and sugar alcohols	True	6.149e+05	-1.836	-1.836	-1.031
309	Glutathione, Oxidized	Amino acid	1	Ctrl_Untreated_1_3	Ctrl_Untreated_1	1.0	Glutathione	True	4.994e+06	1.186	1.186	-6.83e-03
35	Ribonate	Carbon	1	Ctrl_Untreated_1_3	Ctrl_Untreated_1	1.0	Sugars and sugar alcohols	True	1.021e+06	-1.104	-1.104	-0.5764
160	1-Me-His	Amino acid	1	Ctrl_Untreated_1_3	Ctrl_Untreated_1	1.0	Amino acid derivatives	True	1.04e+07	2.244	2.244	-1.197

Metabolite ID	Name	Super Pathway	Dataset	Sample ID	Group ID	Detection Fraction	Pathway	Detected	Raw Intensity	Log2 Norm Intensity	Norm Imputed	Log2 Ctrl Norm
44	Glucose 6-P	Carbon	1	Ctrl_Untreated_1_3	Ctrl_Untreated_1	0.857142857142857	Glycolysis, GNG	False			-3.422	-1.946
704	NADH	Cofactor	1	Ctrl_Untreated_1_3	Ctrl_Untreated_1	1.0	Cofactors	True	1.728e+06	-0.3451	-0.3451	0.301
275	Thr-Phe	Amino acid	1	Ctrl_Untreated_1_3	Ctrl_Untreated_1	0.857142857142857	Dipeptides	True	1.513e+06	-0.5366	-0.5366	1.117
738	Pyridoxate	Cofactor	1	Ctrl_Untreated_1_3	Ctrl_Untreated_1	1.0	PLP biosynthesis and salvage	True	2.214e+05	-3.31	-3.31	0.05475
177	3-(4-OH-Phenyl)Lactate	Amino acid	1	Ctrl_Untreated_1_3	Ctrl_Untreated_1	1.0	Amino acid derivatives	True	1.529e+05	-3.843	-3.843	-1.378
206	Trans-4-OH-Pro	Amino acid	1	Ctrl_Untreated_1_3	Ctrl_Untreated_1	1.0	Amino acid derivatives	True	5.141e+07	4.55	4.55	-0.6056
329	AMP	Nucleotide	1	Ctrl_Untreated_1_3	Ctrl_Untreated_1	1.0	Purine nucleotides	True	3.739e+06	0.7683	0.7683	-2.867
11	UDP-Glucose	Carbon	1	Ctrl_Untreated_1_3	Ctrl_Untreated_1	1.0	Polysaccharide biosynthesis	True	8.634e+05	-1.346	-1.346	-1.241
158	4-Imidazole-Ac	Amino acid	1	Ctrl_Untreated_1_3	Ctrl_Untreated_1	1.0	Amino acid derivatives	True	5.15e+05	-2.091	-2.091	0.05512
111	1-Me-Imidazole-Ac	Amino acid	1	Ctrl_Untreated_1_3	Ctrl_Untreated_1	1.0	Amino acids degradation intermediates	True	2.233e+05	-3.297	-3.297	-1.363
345	Guanine	Nucleotide	1	Ctrl_Untreated_1_3	Ctrl_Untreated_1	1.0	Purine bases	True	1.676e+08	6.255	6.255	1.268
22	N-Ac-Neuraminate	Carbon	1	Ctrl_Untreated_1_3	Ctrl_Untreated_1	1.0	Aminosugar derivatives	True	1.84e+06	-0.2542	-0.2542	-0.1188
721	N'-Methylnicotinate	Cofactor	1	Ctrl_Untreated_1_3	Ctrl_Untreated_1	1.0	Derivatives of NA, nicotinamide and NAD	True	7.774e+05	-1.498	-1.498	-0.49
183	Phenol Sulfate	Amino acid	1	Ctrl_Untreated_1_3	Ctrl_Untreated_1	0.857142857142857	Amino acid derivatives	False			-5.737	-1.097
718	Nicotinamide Riboside	Cofactor	1	Ctrl_Untreated_1_3	Ctrl_Untreated_1	1.0	NAD biosynthesis	True	1.334e+08	5.925	5.925	4.335
297	gamma-Glu-Thr	Amino acid	1	Ctrl_Untreated_1_3	Ctrl_Untreated_1	1.0	Gamma-glutamyl dipeptides	True	2.598e+06	0.243	0.243	-0.3972
295	gamma-Glu-Phe	Amino acid	1	Ctrl_Untreated_1_3	Ctrl_Untreated_1	0.428571428571429	Gamma-glutamyl dipeptides	False			-5.975	-0.1899
347	Allantoic Acid	Nucleotide	1	Ctrl_Untreated_1_3	Ctrl_Untreated_1	0.571428571428571	Purine degradation	False			-6.474	-0.8345
399	Pseudouridine	Nucleotide	1	Ctrl_Untreated_1_3	Ctrl_Untreated_1	1.0	Pyrimidine derivatives in RNAs	True	2.747e+05	-2.998	-2.998	-0.9682
375	UTP	Nucleotide	1	Ctrl_Untreated_1_3	Ctrl_Untreated_1	0.571428571428571	Pyrimidine nucleotides	False			-5.025	-3.049
144	Glu, gamma-Me Ester	Amino acid	1	Ctrl_Untreated_1_3	Ctrl_Untreated_1	1.0	Amino acid derivatives	True	1.987e+06	-0.1437	-0.1437	-0.7214

Metabolite ID	Name	Super Pathway	Dataset	Sample ID	Group ID	Detection Fraction	Pathway	Detected	Raw Intensity	Log2 Norm Intensity	Norm Imputed	Log2 Ctrl Norm
292	gamma-Glu-epsilon-Lysine	Amino acid	1	Ctrl_Untreated_1_3	Ctrl_Untreated_1	1.0	Gamma-glutamyl dipeptides	True	8.967e+05	-1.292	-1.292	-1.1
225	N-Ac-Thr	Amino acid	1	Ctrl_Untreated_1_3	Ctrl_Untreated_1	1.0	N-acetylated amino acids	True	7.366e+05	-1.575	-1.575	-0.0727
211	N-Ac-Asn	Amino acid	1	Ctrl_Untreated_1_3	Ctrl_Untreated_1	1.0	N-acetylated amino acids	True	2.599e+05	-3.078	-3.078	-0.3929
151	Phenylacetylglycine	Amino acid	1	Ctrl_Untreated_1_3	Ctrl_Untreated_1	1.0	Amino acid derivatives	True	1.501e+06	-0.548	-0.548	0.239
217	N-Ac-His	Amino acid	1	Ctrl_Untreated_1_3	Ctrl_Untreated_1	1.0	N-acetylated amino acids	True	1.398e+05	-3.973	-3.973	-0.4876
288	gamma-Glu-Gly	Amino acid	1	Ctrl_Untreated_1_3	Ctrl_Untreated_1	0.571428571428571	Gamma-glutamyl dipeptides	False			-3.092	-1.268
222	N-Ac-Phe	Amino acid	1	Ctrl_Untreated_1_3	Ctrl_Untreated_1	0.571428571428571	N-acetylated amino acids	False			-6.698	-0.4255
71	Creatine-P	Carbon	1	Ctrl_Untreated_1_3	Ctrl_Untreated_1	1.0	Creatine energy storage	True	1.026e+05	-4.419	-4.419	-0.1636
210	N-Ac-Arg	Amino acid	1	Ctrl_Untreated_1_3	Ctrl_Untreated_1	1.0	N-acetylated amino acids	True	2.697e+05	-3.025	-3.025	-0.3493
218	N-Ac-Ile	Amino acid	1	Ctrl_Untreated_1_3	Ctrl_Untreated_1	0.428571428571429	N-acetylated amino acids	False			-6.52	-0.5541
251	Gly-Leu	Amino acid	1	Ctrl_Untreated_1_3	Ctrl_Untreated_1	1.0	Dipeptides	True	7.097e+06	1.693	1.693	0.05417
290	gamma-Glu-Ile	Amino acid	1	Ctrl_Untreated_1_3	Ctrl_Untreated_1	1.0	Gamma-glutamyl dipeptides	True	5.399e+05	-2.023	-2.023	-0.6849
316	Ophthalmate	Amino acid	1	Ctrl_Untreated_1_3	Ctrl_Untreated_1	1.0	Oxidative stress markers	True	8.032e+06	1.871	1.871	-0.4711
125	Isovaleryl-Gly	Amino acid	1	Ctrl_Untreated_1_3	Ctrl_Untreated_1	0.857142857142857	Amino acids degradation intermediates	True	6.27e+04	-5.13	-5.13	0.2798
368	7-Me-Guanine	Nucleotide	1	Ctrl_Untreated_1_3	Ctrl_Untreated_1	1.0	Purine derivatives in RNAs	True	1.888e+05	-3.54	-3.54	-1.349
208	Pro-OH-Pro	Amino acid	1	Ctrl_Untreated_1_3	Ctrl_Untreated_1	1.0	Amino acid derivatives	True	6.11e+06	1.477	1.477	-1.852
366	N2,N2-DiMe-Guanosine	Nucleotide	1	Ctrl_Untreated_1_3	Ctrl_Untreated_1	0.857142857142857	Purine derivatives in RNAs	False			-5.009	-0.8957
352	3'-AMP	Nucleotide	1	Ctrl_Untreated_1_3	Ctrl_Untreated_1	1.0	Purine derivatives in signaling	True	1.083e+06	-1.019	-1.019	0.4677
363	N6-Carbamoyl-Thr-Adenosine	Nucleotide	1	Ctrl_Untreated_1_3	Ctrl_Untreated_1	0.857142857142857	Purine derivatives in RNAs	False			-6.008	-1.323
314	Cys-Glutathione Disulfide	Amino acid	1	Ctrl_Untreated_1_3	Ctrl_Untreated_1	1.0	Oxidative stress markers	True	1.294e+05	-4.085	-4.085	-0.8006
382	Orotidine	Nucleotide	1	Ctrl_Untreated_1_3	Ctrl_Untreated_1	0.428571428571429	Pyrimidine (UMP) biosynthesis	False			-5.287	-0.9952

Metabolite ID	Name	Super Pathway	Dataset	Sample ID	Group ID	Detection Fraction	Pathway	Detected	Raw Intensity	Log2 Norm Intensity	Norm Imputed	Log2 Ctrl Norm
307	Cys-Gly	Amino acid	1	Ctrl_Untreated_1_3	Ctrl_Untreated_1	1.0	Glutathione biosynthesis	True	8.812e+06	2.005	2.005	0.3982
64	Sedoheptulose-7-P	Carbon	1	Ctrl_Untreated_1_3	Ctrl_Untreated_1	1.0	Pentose phosphate pathway (PPP)	True	1.986e+06	-0.1444	-0.1444	-0.2985
142	N-Ac-Asp-Glu	Amino acid	1	Ctrl_Untreated_1_3	Ctrl_Untreated_1	1.0	Amino acid derivatives	True	6.285e+05	-1.804	-1.804	-0.6589
708	Thiamin-PP	Cofactor	1	Ctrl_Untreated_1_3	Ctrl_Untreated_1	0.571428571428571	Cofactors	False			-6.331	-0.3472
182	P-Cresol Sulfate	Amino acid	1	Ctrl_Untreated_1_3	Ctrl_Untreated_1	0.857142857142857	Amino acid derivatives	False			-4.093	-0.8968
250	Gly-Ile	Amino acid	1	Ctrl_Untreated_1_3	Ctrl_Untreated_1	1.0	Dipeptides	True	7.522e+05	-1.545	-1.545	-0.2278
286	gamma-Glu-Glu	Amino acid	1	Ctrl_Untreated_1_3	Ctrl_Untreated_1	1.0	Gamma-glutamyl dipeptides	True	1.901e+06	-0.2074	-0.2074	-0.2253
739	Deoxycarnitine	Cofactor	1	Ctrl_Untreated_1_3	Ctrl_Untreated_1	1.0	Carnitine biosynthesis	True	1.887e+07	3.104	3.104	-0.6471
203	DiMe-Arg	Amino acid	1	Ctrl_Untreated_1_3	Ctrl_Untreated_1	1.0	Amino acid derivatives	True	1.275e+08	5.86	5.86	0.2278
351	2'-AMP	Nucleotide	1	Ctrl_Untreated_1_3	Ctrl_Untreated_1	1.0	Purine derivatives in signaling	True	1.605e+06	-0.4519	-0.4519	1.817
8	Cytidine 5'-P-N-Ac-Neuraminate	Carbon	1	Ctrl_Untreated_1_3	Ctrl_Untreated_1	1.0	Aminosugar biosynthesis	True	5.444e+05	-2.011	-2.011	-0.2659
285	gamma-Glu-Ala	Amino acid	1	Ctrl_Untreated_1_3	Ctrl_Untreated_1	0.857142857142857	Gamma-glutamyl dipeptides	True	4.434e+05	-2.307	-2.307	0.9313
224	N-Ac-Ser	Amino acid	1	Ctrl_Untreated_1_3	Ctrl_Untreated_1	1.0	N-acetylated amino acids	True	6.239e+06	1.507	1.507	-0.3721
244	Ala-Leu	Amino acid	1	Ctrl_Untreated_1_3	Ctrl_Untreated_1	1.0	Dipeptides	True	5.395e+06	1.297	1.297	0.1594
207	N-Me-Pro	Amino acid	1	Ctrl_Untreated_1_3	Ctrl_Untreated_1	1.0	Amino acid derivatives	True	5.536e+05	-1.987	-1.987	-1.057
171	Cys Sulfinic Acid	Amino acid	1	Ctrl_Untreated_1_3	Ctrl_Untreated_1	0.857142857142857	Amino acid derivatives	True	3.381e+05	-2.699	-2.699	0.5987
181	O-Me-Tyr	Amino acid	1	Ctrl_Untreated_1_3	Ctrl_Untreated_1	0.857142857142857	Amino acid derivatives	False			-4.309	-0.9396
240	N-Ac-Putrescine	Amino acid	1	Ctrl_Untreated_1_3	Ctrl_Untreated_1	1.0	Polyamine derivatives	True	6.607e+05	-1.732	-1.732	0.6316
176	S-Me-Met	Amino acid	1	Ctrl_Untreated_1_3	Ctrl_Untreated_1	0.857142857142857	Amino acid derivatives	False			-3.205	-1.364
339	AICAR	Nucleotide	1	Ctrl_Untreated_1_3	Ctrl_Untreated_1	0.571428571428571	IMP biosynthesis	True	1.484e+05	-3.887	-3.887	0.4143
141	gamma-Carboxy-Glu	Amino acid	1	Ctrl_Untreated_1_3	Ctrl_Untreated_1	1.0	Amino acid derivatives	True	1.207e+06	-0.8634	-0.8634	-0.0784

Metabolite ID	Name	Super Pathway	Dataset	Sample ID	Group ID	Detection Fraction	Pathway	Detected	Raw Intensity	Log2 Norm Intensity	Norm Imputed	Log2 Ctrl Norm
392	3'-UMP	Nucleotide	1	Ctrl_Untreated_1_3	Ctrl_Untreated_1	0.571428571428571	Pyrimidine derivatives in signaling	True	2.215e+05	-3.309	-3.309	0.7244
355	3'-GMP	Nucleotide	1	Ctrl_Untreated_1_3	Ctrl_Untreated_1	0.857142857142857	Purine derivatives in signaling	True	3.124e+05	-2.813	-2.813	1.125
282	Val-Leu	Amino acid	1	Ctrl_Untreated_1_3	Ctrl_Untreated_1	1.0	Dipeptides	True	2.908e+06	0.4056	0.4056	-0.5661
140	Carboxyethyl-GABA	Amino acid	1	Ctrl_Untreated_1_3	Ctrl_Untreated_1	1.0	Amino acid derivatives	True	9.643e+05	-1.187	-1.187	0.2445
258	Ile-Gly	Amino acid	1	Ctrl_Untreated_1_3	Ctrl_Untreated_1	1.0	Dipeptides	True	1.589e+07	2.856	2.856	-0.4564
260	Leu-Ala	Amino acid	1	Ctrl_Untreated_1_3	Ctrl_Untreated_1	1.0	Dipeptides	True	5.779e+06	1.397	1.397	0.09533
265	Lys-Leu	Amino acid	1	Ctrl_Untreated_1_3	Ctrl_Untreated_1	0.857142857142857	Dipeptides	True	1.035e+06	-1.085	-1.085	1.526
263	Leu-Gly	Amino acid	1	Ctrl_Untreated_1_3	Ctrl_Untreated_1	1.0	Dipeptides	True	1.766e+07	3.009	3.009	-0.01266
281	Val-Gly	Amino acid	1	Ctrl_Untreated_1_3	Ctrl_Untreated_1	1.0	Dipeptides	True	4.064e+07	4.21	4.21	-0.3087
270	Pro-Gly	Amino acid	1	Ctrl_Untreated_1_3	Ctrl_Untreated_1	1.0	Dipeptides	True	2.565e+06	0.2247	0.2247	-0.522
114	Imidazole Propionate	Amino acid	1	Ctrl_Untreated_1_3	Ctrl_Untreated_1	1.0	Amino acids degradation intermediates	True	2.424e+05	-3.179	-3.179	-0.868
267	Phe-Gly	Amino acid	1	Ctrl_Untreated_1_3	Ctrl_Untreated_1	1.0	Dipeptides	True	3.227e+07	3.878	3.878	-0.216
266	Phe-Ala	Amino acid	1	Ctrl_Untreated_1_3	Ctrl_Untreated_1	1.0	Dipeptides	True	5.944e+06	1.437	1.437	0.3606
278	Tyr-Gly	Amino acid	1	Ctrl_Untreated_1_3	Ctrl_Untreated_1	1.0	Dipeptides	True	9.403e+06	2.099	2.099	-0.08333
255	His-Ala	Amino acid	1	Ctrl_Untreated_1_3	Ctrl_Untreated_1	1.0	Dipeptides	True	1.5e+06	-0.5496	-0.5496	0.7878
280	Val-Gln	Amino acid	1	Ctrl_Untreated_1_3	Ctrl_Untreated_1	1.0	Dipeptides	True	5.177e+06	1.238	1.238	-0.3801
143	S-1-Pyrroline-5-Carboxylate	Amino acid	1	Ctrl_Untreated_1_3	Ctrl_Untreated_1	0.857142857142857	Amino acid derivatives	False			-3.202	-1.133
232	SAH	Amino acid	1	Ctrl_Untreated_1_3	Ctrl_Untreated_1	1.0	SAM metabolism	True	1.246e+06	-0.8172	-0.8172	-0.3318
20	Erythronate	Carbon	1	Ctrl_Untreated_1_3	Ctrl_Untreated_1	1.0	Aminosugar derivatives	True	1.418e+07	2.692	2.692	-1.049
248	Gln-Leu	Amino acid	1	Ctrl_Untreated_1_3	Ctrl_Untreated_1	1.0	Dipeptides	True	2.268e+06	0.0473	0.0473	0.9525
276	Trp-Gly	Amino acid	1	Ctrl_Untreated_1_3	Ctrl_Untreated_1	1.0	Dipeptides	True	1.035e+06	-1.085	-1.085	-6.80e-03
205	N-delta-Ac-Ornithine	Amino acid	1	Ctrl_Untreated_1_3	Ctrl_Untreated_1	1.0	Amino acid derivatives	True	4.317e+05	-2.346	-2.346	-1.225
163	Formimino-Glu	Amino acid	1	Ctrl_Untreated_1_3	Ctrl_Untreated_1	1.0	Amino acid derivatives	True	4.8e+05	-2.193	-2.193	-0.9532
204	N-Me-Arg	Amino acid	1	Ctrl_Untreated_1_3	Ctrl_Untreated_1	1.0	Amino acid derivatives	True	1.982e+07	3.175	3.175	0.5876
242	Guanidino-Ac	Amino acid	1	Ctrl_Untreated_1_3	Ctrl_Untreated_1	0.857142857142857	Creatine biosynthesis	False			-4.685	-0.8041

Metabolite ID	Name	Super Pathway	Dataset	Sample ID	Group ID	Detection Fraction	Pathway	Detected	Raw Intensity	Log2 Norm Intensity	Norm Imputed	Log2 Ctrl Norm
300	gamma-Glu-Val	Amino acid	1	Ctrl_Untreated_1_3	Ctrl_Untreated_1	0.857142857142857	Gamma-glutamyl dipeptides	True	2.165e+06	-0.02017	-0.02017	-0.6409
53	Ac-CoA	Carbon	1	Ctrl_Untreated_1_3	Ctrl_Untreated_1	0.285714285714286	Acetyl-CoA	False			-7.274	-0.5114
18	Maltotriose	Carbon	1	Ctrl_Untreated_1_3	Ctrl_Untreated_1	1.0	Glycogen degradation	True	4.627e+05	-2.246	-2.246	-2.088
294	gamma-Glu-Met	Amino acid	1	Ctrl_Untreated_1_3	Ctrl_Untreated_1	0.714285714285714	Gamma-glutamyl dipeptides	True	3.697e+05	-2.57	-2.57	0.9122
174	Met Sulfone	Amino acid	1	Ctrl_Untreated_1_3	Ctrl_Untreated_1	1.0	Amino acid derivatives	True	1.109e+05	-4.307	-4.307	-1.229
175	N-Ac-Met Sulfoxide	Amino acid	1	Ctrl_Untreated_1_3	Ctrl_Untreated_1	1.0	Amino acid derivatives	True	3.038e+06	0.4689	0.4689	0.4926
25	Mannitol/Sorbitol	Carbon	1	Ctrl_Untreated_1_3	Ctrl_Untreated_1	1.0	Sugars and sugar alcohols	True	3.454e+06	0.6541	0.6541	-1.571
6	UDP-GlcNAc	Carbon	1	Ctrl_Untreated_1_3	Ctrl_Untreated_1	0.428571428571429	Aminosugar biosynthesis	False			-3.589	-0.4814
145	Pyro-Gln	Amino acid	1	Ctrl_Untreated_1_3	Ctrl_Untreated_1	1.0	Amino acid derivatives	True	2.745e+06	0.3226	0.3226	-0.5172
705	Coenzyme A	Cofactor	1	Ctrl_Untreated_1_3	Ctrl_Untreated_1	0.714285714285714	Cofactors	False			-4.967	-1.008
319	2'-dAMP	Nucleotide	1	Ctrl_Untreated_1_3	Ctrl_Untreated_1	0.571428571428571	Deoxy-nucleotides	False			-4.337	-0.6568
119	alpha-OH-Isovalerate	Amino acid	1	Ctrl_Untreated_1_3	Ctrl_Untreated_1	0.714285714285714	Amino acids degradation intermediates	False			-5.298	-1.999
46	Fructose 1,6-PP / Glucose 1,6-PP / Inositol-1,4-PP / Inositol-1,3-PP	Carbon	1	Ctrl_Untreated_1_3	Ctrl_Untreated_1	1.0	Glycolysis, GNG	True	5.826e+07	4.73	4.73	-0.101
137	1-Me-Guanidine	Amino acid	1	Ctrl_Untreated_1_3	Ctrl_Untreated_1	0.857142857142857	Amino acids degradation intermediates	True	5.138e+04	-5.417	-5.417	-0.2641
23	N-GlcNAc-Asn	Carbon	1	Ctrl_Untreated_1_3	Ctrl_Untreated_1	1.0	Aminosugar derivatives	True	2.466e+06	0.168	0.168	-0.5449
262	Leu-Gln	Amino acid	1	Ctrl_Untreated_1_3	Ctrl_Untreated_1	1.0	Dipeptides	True	3.934e+06	0.8419	0.8419	-0.4544
24	Fructose	Carbon	1	Ctrl_Untreated_1_3	Ctrl_Untreated_1	1.0	Sugars and sugar alcohols	True	2.122e+06	-0.04871	-0.04871	-1.998
197	C-Glycosyl-Trp	Amino acid	1	Ctrl_Untreated_1_3	Ctrl_Untreated_1	1.0	Amino acid derivatives	True	1.135e+06	-0.9512	-0.9512	-0.6522
33	Arabitol/Xylitol	Carbon	1	Ctrl_Untreated_1_3	Ctrl_Untreated_1	1.0	Sugars and sugar alcohols	True	2.139e+05	-3.36	-3.36	-1.5

Metabolite ID	Name	Super Pathway	Dataset	Sample ID	Group ID	Detection Fraction	Pathway	Detected	Raw Intensity	Log2 Norm Intensity	Norm Imputed	Log2 Ctrl Norm
128	N2-Ac-Lys/N6-Ac-Lys	Amino acid	1	Ctrl_Untreated_1_3	Ctrl_Untreated_1	1.0	Amino acids degradation intermediates	True	1.111e+07	2.339	2.339	1.129
42	2-Me-Citrate	Carbon	1	Ctrl_Untreated_1_3	Ctrl_Untreated_1	0.857142857142857	Propionate metabolism	True	9.651e+04	-4.507	-4.507	-0.1245
12	Glucuronate 1-P	Carbon	1	Ctrl_Untreated_1_3	Ctrl_Untreated_1	1.0	Polysaccharide biosynthesis	True	1.912e+06	-0.1995	-0.1995	1.043
76	Gln	Amino acid	1	Ctrl_Untreated_1_4	Ctrl_Untreated_1	1.0	Proteinogenic amino acids	True	1.354e+09	9.179	9.179	0.4985
89	Trp	Amino acid	1	Ctrl_Untreated_1_4	Ctrl_Untreated_1	1.0	Proteinogenic amino acids	True	1.809e+08	6.275	6.275	0.5992
723	beta-Ala	Cofactor	1	Ctrl_Untreated_1_4	Ctrl_Untreated_1	1.0	Coenzyme A biosynthesis	True	1.826e+07	2.966	2.966	-0.04337
75	Glu	Amino acid	1	Ctrl_Untreated_1_4	Ctrl_Untreated_1	1.0	Proteinogenic amino acids	True	4.859e+09	11.02	11.02	0.2855
77	Gly	Amino acid	1	Ctrl_Untreated_1_4	Ctrl_Untreated_1	1.0	Proteinogenic amino acids	True	2.554e+08	6.772	6.772	1.157
80	His	Amino acid	1	Ctrl_Untreated_1_4	Ctrl_Untreated_1	1.0	Proteinogenic amino acids	True	3.964e+07	4.085	4.085	0.6694
82	Leu	Amino acid	1	Ctrl_Untreated_1_4	Ctrl_Untreated_1	1.0	Proteinogenic amino acids	True	2.523e+09	10.08	10.08	0.8554
87	Phe	Amino acid	1	Ctrl_Untreated_1_4	Ctrl_Untreated_1	1.0	Proteinogenic amino acids	True	1.755e+09	9.553	9.553	0.7414
130	Glutarate	Amino acid	1	Ctrl_Untreated_1_4	Ctrl_Untreated_1	1.0	Amino acids degradation intermediates	True	3.462e+05	-2.755	-2.755	-0.3282
196	5-OH-Indole-Ac	Amino acid	1	Ctrl_Untreated_1_4	Ctrl_Untreated_1	0.857142857142857	Amino acid derivatives	True	1.167e+05	-4.323	-4.323	-0.3113
74	Asp	Amino acid	1	Ctrl_Untreated_1_4	Ctrl_Untreated_1	1.0	Proteinogenic amino acids	True	1.01e+09	8.756	8.756	0.8642
236	Spermidine	Amino acid	1	Ctrl_Untreated_1_4	Ctrl_Untreated_1	1.0	Polyamines	True	4.4e+07	4.235	4.235	0.3736
73	Asn	Amino acid	1	Ctrl_Untreated_1_4	Ctrl_Untreated_1	1.0	Proteinogenic amino acids	True	4.53e+08	7.599	7.599	0.9279
243	Creatinine	Amino acid	1	Ctrl_Untreated_1_4	Ctrl_Untreated_1	1.0	Creatine degradation	True	7.432e+07	4.991	4.991	-0.01096
376	Cytidine	Nucleotide	1	Ctrl_Untreated_1_4	Ctrl_Untreated_1	0.714285714285714	Pyrimidine nucleosides	True	3.374e+07	3.852	3.852	3.124
41	Lactate	Carbon	1	Ctrl_Untreated_1_4	Ctrl_Untreated_1	1.0	Respiratory carbon sources	True	2.472e+08	6.725	6.725	0.2434
58	alpha-Ketoglutarate	Carbon	1	Ctrl_Untreated_1_4	Ctrl_Untreated_1	1.0	TCA cycle	True	3.054e+06	0.3865	0.3865	0.3698
69	3-OH-Butyrate	Carbon	1	Ctrl_Untreated_1_4	Ctrl_Untreated_1	0.571428571428571	Ketone bodies	True	2.069e+05	-3.497	-3.497	-0.5127

Metabolite ID	Name	Super Pathway	Dataset	Sample ID	Group ID	Detection Fraction	Pathway	Detected	Raw Intensity	Log2 Norm Intensity	Norm Imputed	Log2 Ctrl Norm
343	Adenine	Nucleotide	1	Ctrl_Untreated_1_4	Ctrl_Untreated_1	1.0	Purine bases	True	8.38e+06	1.843	1.843	0.3857
336	Adenosine	Nucleotide	1	Ctrl_Untreated_1_4	Ctrl_Untreated_1	1.0	Purine nucleosides	True	3.178e+07	3.766	3.766	0.343
722	ADP-Ribose	Cofactor	1	Ctrl_Untreated_1_4	Ctrl_Untreated_1	1.0	Derivatives of NA, nicotinamide and NAD	True	1.959e+05	-3.576	-3.576	-0.5739
383	Cytosine	Nucleotide	1	Ctrl_Untreated_1_4	Ctrl_Untreated_1	0.571428571428571	Pyrimidine bases	True	1.504e+05	-3.957	-3.957	0.8978
3	Glucosamine 6-P	Carbon	1	Ctrl_Untreated_1_4	Ctrl_Untreated_1	1.0	Aminosugar biosynthesis	True	1.827e+05	-3.677	-3.677	-2.01
717	Nicotinamide	Cofactor	1	Ctrl_Untreated_1_4	Ctrl_Untreated_1	1.0	NAD biosynthesis	True	8.19e+07	5.132	5.132	0.6163
51	PEP	Carbon	1	Ctrl_Untreated_1_4	Ctrl_Untreated_1	1.0	Glycolysis, GNG	True	5.916e+06	1.34	1.34	0.2891
237	Spermine	Amino acid	1	Ctrl_Untreated_1_4	Ctrl_Untreated_1	1.0	Polyamines	True	2.322e+06	-8.91e-03	-8.91e-03	1.482
385	Uracil	Nucleotide	1	Ctrl_Untreated_1_4	Ctrl_Untreated_1	1.0	Pyrimidine bases	True	1.62e+07	2.793	2.793	2.529
377	Uridine	Nucleotide	1	Ctrl_Untreated_1_4	Ctrl_Untreated_1	1.0	Pyrimidine nucleosides	True	8.482e+07	5.182	5.182	1.133
348	Allantoin	Nucleotide	1	Ctrl_Untreated_1_4	Ctrl_Untreated_1	1.0	Purine degradation	True	1.949e+06	-0.2614	-0.2614	-0.0858
335	Inosine	Nucleotide	1	Ctrl_Untreated_1_4	Ctrl_Untreated_1	1.0	Purine nucleosides	True	2.181e+08	6.544	6.544	1.045
81	Ile	Amino acid	1	Ctrl_Untreated_1_4	Ctrl_Untreated_1	1.0	Proteinogenic amino acids	True	1.395e+09	9.222	9.222	0.5066
72	Ala	Amino acid	1	Ctrl_Untreated_1_4	Ctrl_Untreated_1	1.0	Proteinogenic amino acids	True	1.115e+09	8.899	8.899	0.3974
79	Thr	Amino acid	1	Ctrl_Untreated_1_4	Ctrl_Untreated_1	1.0	Proteinogenic amino acids	True	1.17e+09	8.968	8.968	0.9584
88	Tyr	Amino acid	1	Ctrl_Untreated_1_4	Ctrl_Untreated_1	1.0	Proteinogenic amino acids	True	8.323e+08	8.477	8.477	0.953
84	Lys	Amino acid	1	Ctrl_Untreated_1_4	Ctrl_Untreated_1	1.0	Proteinogenic amino acids	True	8.994e+08	8.589	8.589	0.5985
86	Met	Amino acid	1	Ctrl_Untreated_1_4	Ctrl_Untreated_1	1.0	Proteinogenic amino acids	True	1.329e+09	9.152	9.152	0.9243
61	Malate	Carbon	1	Ctrl_Untreated_1_4	Ctrl_Untreated_1	1.0	TCA cycle	True	3.921e+08	7.391	7.391	0.219
235	Putrescine	Amino acid	1	Ctrl_Untreated_1_4	Ctrl_Untreated_1	1.0	Polyamines	True	5.197e+05	-2.169	-2.169	-1.677
324	2'-dU	Nucleotide	1	Ctrl_Untreated_1_4	Ctrl_Untreated_1	0.714285714285714	Deoxy-nucleosides	True	8.895e+04	-4.715	-4.715	-0.3348
49	3-P-Glycerate	Carbon	1	Ctrl_Untreated_1_4	Ctrl_Untreated_1	1.0	Glycolysis, GNG	True	1.96e+07	3.069	3.069	-0.1967
189	Kynurenate	Amino acid	1	Ctrl_Untreated_1_4	Ctrl_Untreated_1	0.714285714285714	Amino acid derivatives	True	3.166e+04	-6.206	-6.206	-0.4077
234	5-Me-Thioadenosine	Amino acid	1	Ctrl_Untreated_1_4	Ctrl_Untreated_1	1.0	SAM metabolism	True	9.312e+06	1.995	1.995	0.1092
59	Succinate	Carbon	1	Ctrl_Untreated_1_4	Ctrl_Untreated_1	1.0	TCA cycle	True	1.467e+06	-0.6719	-0.6719	-0.9729

Metabolite ID	Name	Super Pathway	Dataset	Sample ID	Group ID	Detection Fraction	Pathway	Detected	Raw Intensity	Log2 Norm Intensity	Norm Imputed	Log2 Ctrl Norm
36	Ribose	Carbon	1	Ctrl_Untreated_1_4	Ctrl_Untreated_1	1.0	Sugars and sugar alcohols	True	1.139e+06	-1.036	-1.036	0.1971
133	Ornithine	Amino acid	1	Ctrl_Untreated_1_4	Ctrl_Untreated_1	1.0	Amino acids degradation intermediates	True	3.866e+07	4.048	4.048	-0.4312
313	5-Oxoproline	Amino acid	1	Ctrl_Untreated_1_4	Ctrl_Untreated_1	1.0	Glutathione derivatives	True	8.557e+06	1.873	1.873	-0.2147
165	N-6-Tri-Me-Lys	Amino acid	1	Ctrl_Untreated_1_4	Ctrl_Untreated_1	1.0	Amino acid derivatives	True	6.942e+07	4.893	4.893	1.129
380	Orotate	Nucleotide	1	Ctrl_Untreated_1_4	Ctrl_Untreated_1	0.857142857142857	Pyrimidine (UMP) biosynthesis	True	1.669e+05	-3.807	-3.807	-0.177
724	Pantothenate	Cofactor	1	Ctrl_Untreated_1_4	Ctrl_Untreated_1	1.0	Coenzyme A biosynthesis	True	1.401e+08	5.906	5.906	-0.4038
150	N-Me-Gly	Amino acid	1	Ctrl_Untreated_1_4	Ctrl_Untreated_1	1.0	Amino acid derivatives	True	2.315e+06	-0.01333	-0.01333	0.2871
122	3-OH-Isobutyrate	Amino acid	1	Ctrl_Untreated_1_4	Ctrl_Untreated_1	0.857142857142857	Amino acids degradation intermediates	True	2.723e+05	-3.101	-3.101	0.05484
241	4-Acetamidobutanoate	Amino acid	1	Ctrl_Untreated_1_4	Ctrl_Untreated_1	1.0	Polyamine derivatives	True	2.428e+06	0.05548	0.05548	-0.3855
711	alpha-Tocopherol	Cofactor	1	Ctrl_Untreated_1_4	Ctrl_Untreated_1	1.0	Cofactors	True	2.391e+06	0.03325	0.03325	-0.7471
55	Citrate	Carbon	1	Ctrl_Untreated_1_4	Ctrl_Untreated_1	1.0	TCA cycle	True	1.291e+07	2.466	2.466	0.5167
387	3-Aminoisobutyrate	Nucleotide	1	Ctrl_Untreated_1_4	Ctrl_Untreated_1	0.857142857142857	Pyrimidine degradation	True	1.221e+06	-0.9366	-0.9366	1.077
338	Guanosine	Nucleotide	1	Ctrl_Untreated_1_4	Ctrl_Untreated_1	1.0	Purine nucleosides	True	1.727e+08	6.208	6.208	1.102
209	N-Ac-Ala	Amino acid	1	Ctrl_Untreated_1_4	Ctrl_Untreated_1	1.0	N-acetylated amino acids	True	1.299e+06	-0.8473	-0.8473	0.3539
221	N-Ac-Met	Amino acid	1	Ctrl_Untreated_1_4	Ctrl_Untreated_1	1.0	N-acetylated amino acids	True	1.214e+07	2.377	2.377	0.6327
228	N-Ac-Val	Amino acid	1	Ctrl_Untreated_1_4	Ctrl_Untreated_1	0.571428571428571	N-acetylated amino acids	True	9.773e+04	-4.579	-4.579	0.7914
346	Urate	Nucleotide	1	Ctrl_Untreated_1_4	Ctrl_Untreated_1	1.0	Purine degradation	True	1.407e+06	-0.7312	-0.7312	0.3316
90	Arg	Amino acid	1	Ctrl_Untreated_1_4	Ctrl_Untreated_1	1.0	Proteinogenic amino acids	True	3.104e+09	10.38	10.38	0.6699
60	Fumarate	Carbon	1	Ctrl_Untreated_1_4	Ctrl_Untreated_1	1.0	TCA cycle	True	4.989e+06	1.094	1.094	0.07917
78	Ser	Amino acid	1	Ctrl_Untreated_1_4	Ctrl_Untreated_1	1.0	Proteinogenic amino acids	True	9.213e+08	8.623	8.623	1.366
83	Val	Amino acid	1	Ctrl_Untreated_1_4	Ctrl_Untreated_1	1.0	Proteinogenic amino acids	True	1.106e+09	8.887	8.887	1.015
734	Pyridoxal	Cofactor	1	Ctrl_Untreated_1_4	Ctrl_Untreated_1	1.0	PLP biosynthesis and salvage	True	1.295e+07	2.471	2.471	0.6877

Metabolite ID	Name	Super Pathway	Dataset	Sample ID	Group ID	Detection Fraction	Pathway	Detected	Raw Intensity	Log2 Norm Intensity	Norm Imputed	Log2 Ctrl Norm
136	Urea	Amino acid	1	Ctrl_Untreated_1_4	Ctrl_Untreated_1	0.857142857142857	Amino acids degradation intermediates	True	1.895e+06	-0.3018	-0.3018	-0.453
67	Ribose 1-P	Carbon	1	Ctrl_Untreated_1_4	Ctrl_Untreated_1	1.0	Pentose phosphate pathway (PPP)	True	3.887e+06	0.7342	0.7342	0.113
284	Carnosine	Amino acid	1	Ctrl_Untreated_1_4	Ctrl_Untreated_1	1.0	Dipeptides	True	8.287e+05	-1.495	-1.495	0.6129
306	gamma-Glu-Cys	Amino acid	1	Ctrl_Untreated_1_4	Ctrl_Untreated_1	1.0	Glutathione biosynthesis	True	3.093e+06	0.4046	0.4046	0.7414
712	Retinol (Vit A)	Cofactor	1	Ctrl_Untreated_1_4	Ctrl_Untreated_1	1.0	Cofactors	True	5.256e+05	-2.152	-2.152	0.2811
85	Cys	Amino acid	1	Ctrl_Untreated_1_4	Ctrl_Untreated_1	1.0	Proteinogenic amino acids	True	8.768e+07	5.23	5.23	0.7162
91	Pro	Amino acid	1	Ctrl_Untreated_1_4	Ctrl_Untreated_1	1.0	Proteinogenic amino acids	True	2.468e+09	10.04	10.04	0.6368
308	Glutathione, Reduced	Amino acid	1	Ctrl_Untreated_1_4	Ctrl_Untreated_1	1.0	Glutathione	True	1.637e+08	6.131	6.131	-0.8252
107	Citrulline	Amino acid	1	Ctrl_Untreated_1_4	Ctrl_Untreated_1	1.0	Amino acids biosynthesis intermediates	True	1.325e+07	2.504	2.504	-0.3205
328	IMP	Nucleotide	1	Ctrl_Untreated_1_4	Ctrl_Untreated_1	0.285714285714286	Purine nucleotides	False			-4.53	-0.2088
706	FAD	Cofactor	1	Ctrl_Untreated_1_4	Ctrl_Untreated_1	1.0	Cofactors	True	7.081e+05	-1.722	-1.722	0.4672
735	Pyridoxamine	Cofactor	1	Ctrl_Untreated_1_4	Ctrl_Untreated_1	1.0	PLP biosynthesis and salvage	True	2.451e+06	0.06925	0.06925	0.2623
199	Serotonin	Amino acid	1	Ctrl_Untreated_1_4	Ctrl_Untreated_1	1.0	Amino acid derivatives	True	1.058e+07	2.179	2.179	0.9065
370	CMP	Nucleotide	1	Ctrl_Untreated_1_4	Ctrl_Untreated_1	1.0	Pyrimidine nucleotides	True	9.112e+06	1.963	1.963	0.1245
287	gamma-Glu-Gln	Amino acid	1	Ctrl_Untreated_1_4	Ctrl_Untreated_1	1.0	Gamma-glutamyl dipeptides	True	2.984e+07	3.675	3.675	2.834
14	UDP-Glucuronate	Carbon	1	Ctrl_Untreated_1_4	Ctrl_Untreated_1	0.857142857142857	Polysaccharide biosynthesis	True	1.438e+05	-4.022	-4.022	-2.073
229	N-Formyl-Met	Amino acid	1	Ctrl_Untreated_1_4	Ctrl_Untreated_1	1.0	N-formylated amino acids	True	1.868e+05	-3.645	-3.645	0.1772
350	3',5'-cAMP	Nucleotide	1	Ctrl_Untreated_1_4	Ctrl_Untreated_1	1.0	Purine derivatives in signaling	True	2.86e+05	-3.03	-3.03	0.5891
371	CDP	Nucleotide	1	Ctrl_Untreated_1_4	Ctrl_Untreated_1	0.571428571428571	Pyrimidine nucleotides	False			-5.544	-2.224
372	CTP	Nucleotide	1	Ctrl_Untreated_1_4	Ctrl_Untreated_1	0.571428571428571	Pyrimidine nucleotides	False			-4.713	-1.059
333	GDP	Nucleotide	1	Ctrl_Untreated_1_4	Ctrl_Untreated_1	0.571428571428571	Purine nucleotides	False			-4.484	-1.983
332	GMP	Nucleotide	1	Ctrl_Untreated_1_4	Ctrl_Untreated_1	1.0	Purine nucleotides	True	6.6e+05	-1.824	-1.824	-2.522

Metabolite ID	Name	Super Pathway	Dataset	Sample ID	Group ID	Detection Fraction	Pathway	Detected	Raw Intensity	Log2 Norm Intensity	Norm Imputed	Log2 Ctrl Norm
373	UMP	Nucleotide	1	Ctrl_Untreated_1_4	Ctrl_Untreated_1	1.0	Pyrimidine nucleotides	True	3.345e+05	-2.804	-2.804	-1.749
389	3'-CMP	Nucleotide	1	Ctrl_Untreated_1_4	Ctrl_Untreated_1	1.0	Pyrimidine derivatives in signaling	True	6.071e+05	-1.944	-1.944	-0.893
330	ADP	Nucleotide	1	Ctrl_Untreated_1_4	Ctrl_Untreated_1	0.714285714285714	Purine nucleotides	True	3.294e+05	-2.826	-2.826	-2.53
342	Hypoxanthine	Nucleotide	1	Ctrl_Untreated_1_4	Ctrl_Untreated_1	1.0	Purine bases	True	5.537e+07	4.567	4.567	0.6274
736	Pyridoxamine-P	Cofactor	1	Ctrl_Untreated_1_4	Ctrl_Untreated_1	1.0	PLP biosynthesis and salvage	True	4.541e+05	-2.363	-2.363	0.6149
148	Betaine	Amino acid	1	Ctrl_Untreated_1_4	Ctrl_Untreated_1	1.0	Amino acid derivatives	True	6.566e+07	4.813	4.813	6.90e-03
344	Xanthine	Nucleotide	1	Ctrl_Untreated_1_4	Ctrl_Untreated_1	1.0	Purine bases	True	1.457e+07	2.641	2.641	1.733
386	3-Ureidopropionate	Nucleotide	1	Ctrl_Untreated_1_4	Ctrl_Untreated_1	1.0	Pyrimidine degradation	True	1.839e+06	-0.345	-0.345	0.5743
149	DiMe-Gly	Amino acid	1	Ctrl_Untreated_1_4	Ctrl_Untreated_1	1.0	Amino acid derivatives	True	1.692e+06	-0.4653	-0.4653	0.055
703	NAD+	Cofactor	1	Ctrl_Untreated_1_4	Ctrl_Untreated_1	1.0	Cofactors	True	2.41e+07	3.367	3.367	0.6304
709	Pyridoxal-P	Cofactor	1	Ctrl_Untreated_1_4	Ctrl_Untreated_1	1.0	Cofactors	True	7.689e+05	-1.603	-1.603	0.7045
731	Thiamin (Vitamin B1)	Cofactor	1	Ctrl_Untreated_1_4	Ctrl_Untreated_1	1.0	TPP biosynthesis	True	5.213e+06	1.158	1.158	0.41
374	UDP	Nucleotide	1	Ctrl_Untreated_1_4	Ctrl_Untreated_1	0.714285714285714	Pyrimidine nucleotides	True	5.773e+04	-5.339	-5.339	-3.785
102	2-Aminoadipate	Amino acid	1	Ctrl_Untreated_1_4	Ctrl_Untreated_1	1.0	Amino acids biosynthesis intermediates	True	6.512e+05	-1.843	-1.843	0.2664
45	Fructose-6-P	Carbon	1	Ctrl_Untreated_1_4	Ctrl_Untreated_1	1.0	Glycolysis, GNG	True	1.777e+06	-0.3953	-0.3953	-0.4705
320	TMP	Nucleotide	1	Ctrl_Untreated_1_4	Ctrl_Untreated_1	0.571428571428571	Deoxy-nucleotides	False			-6.992	-2.2
341	XMP	Nucleotide	1	Ctrl_Untreated_1_4	Ctrl_Untreated_1	0.857142857142857	IMP conversion to AMP & GMP	True	3.239e+05	-2.851	-2.851	1.724
120	beta-OH-Isovalerate	Amino acid	1	Ctrl_Untreated_1_4	Ctrl_Untreated_1	0.857142857142857	Amino acids degradation intermediates	True	2.953e+05	-2.984	-2.984	0.1813
322	2'-dI	Nucleotide	1	Ctrl_Untreated_1_4	Ctrl_Untreated_1	0.857142857142857	Deoxy-nucleosides	True	1.031e+05	-4.503	-4.503	-0.3773
4	GlcNAc 6-P	Carbon	1	Ctrl_Untreated_1_4	Ctrl_Untreated_1	1.0	Aminosugar biosynthesis	True	1.272e+07	2.444	2.444	2.116
337	Xanthosine	Nucleotide	1	Ctrl_Untreated_1_4	Ctrl_Untreated_1	1.0	Purine nucleosides	True	3.184e+05	-2.876	-2.876	0.1989
188	Kynurenine	Amino acid	1	Ctrl_Untreated_1_4	Ctrl_Untreated_1	1.0	Amino acid derivatives	True	3.947e+06	0.7564	0.7564	1.448

Metabolite ID	Name	Super Pathway	Dataset	Sample ID	Group ID	Detection Fraction	Pathway	Detected	Raw Intensity	Log2 Norm Intensity	Norm Imputed	Log2 Ctrl Norm
63	6-P-Gluconate	Carbon	1	Ctrl_Untreated_1_4	Ctrl_Untreated_1	1.0	Pentose phosphate pathway (PPP)	True	9.888e+06	2.081	2.081	0.04768
40	Glucuronate	Carbon	1	Ctrl_Untreated_1_4	Ctrl_Untreated_1	1.0	Sugars and sugar alcohols	True	8.914e+05	-1.39	-1.39	0.149
108	Argininosuccinate	Amino acid	1	Ctrl_Untreated_1_4	Ctrl_Untreated_1	1.0	Amino acids biosynthesis intermediates	True	4.908e+06	1.071	1.071	1.297
710	Carnitine	Cofactor	1	Ctrl_Untreated_1_4	Ctrl_Untreated_1	1.0	Cofactors	True	3.196e+08	7.096	7.096	1.118
725	P-Pantetheine	Cofactor	1	Ctrl_Untreated_1_4	Ctrl_Untreated_1	1.0	Coenzyme A biosynthesis	True	2.547e+05	-3.198	-3.198	0.5069
48	DHAP	Carbon	1	Ctrl_Untreated_1_4	Ctrl_Untreated_1	1.0	Glycolysis, GNG	True	1.908e+07	3.03	3.03	-0.06705
17	Maltose	Carbon	1	Ctrl_Untreated_1_4	Ctrl_Untreated_1	1.0	Glycogen degradation	True	1.788e+06	-0.3862	-0.3862	0.9066
359	N1-Me-Adenosine	Nucleotide	1	Ctrl_Untreated_1_4	Ctrl_Untreated_1	0.857142857142857	Purine derivatives in RNAs	True	3.299e+06	0.4978	0.4978	1.535
159	3-Me-His	Amino acid	1	Ctrl_Untreated_1_4	Ctrl_Untreated_1	1.0	Amino acid derivatives	True	1.636e+05	-3.836	-3.836	0.399
155	4-Guanidinobutanoate	Amino acid	1	Ctrl_Untreated_1_4	Ctrl_Untreated_1	1.0	Amino acid derivatives	True	6.874e+05	-1.765	-1.765	-0.3312
164	5-OH-Lys	Amino acid	1	Ctrl_Untreated_1_4	Ctrl_Untreated_1	1.0	Amino acid derivatives	True	9.462e+05	-1.304	-1.304	0.4663
357	Adenosine-3',5'-PP	Nucleotide	1	Ctrl_Untreated_1_4	Ctrl_Untreated_1	0.857142857142857	Purine byproducts of metabolic processes	True	4.385e+05	-2.414	-2.414	1.064
104	Cystathionine	Amino acid	1	Ctrl_Untreated_1_4	Ctrl_Untreated_1	1.0	Amino acids biosynthesis intermediates	True	6.803e+06	1.542	1.542	-0.6677
113	Imidazole Lactate	Amino acid	1	Ctrl_Untreated_1_4	Ctrl_Untreated_1	0.857142857142857	Amino acids degradation intermediates	True	9.33e+04	-4.646	-4.646	-0.9699
215	N-Ac-Glu	Amino acid	1	Ctrl_Untreated_1_4	Ctrl_Untreated_1	1.0	N-acetylated amino acids	True	1.534e+07	2.715	2.715	0.9712
310	S-Lactoyl-Glutathione	Amino acid	1	Ctrl_Untreated_1_4	Ctrl_Untreated_1	0.857142857142857	Glutathione derivatives	True	2.089e+06	-0.1612	-0.1612	-0.5176
5	GlcNAc 1-P	Carbon	1	Ctrl_Untreated_1_4	Ctrl_Untreated_1	1.0	Aminosugar biosynthesis	True	1.808e+06	-0.3697	-0.3697	1.541
34	Ribitol	Carbon	1	Ctrl_Untreated_1_4	Ctrl_Untreated_1	1.0	Sugars and sugar alcohols	True	2.758e+05	-3.083	-3.083	0.3475
10	UDP-Galactose	Carbon	1	Ctrl_Untreated_1_4	Ctrl_Untreated_1	0.857142857142857	Polysaccharide biosynthesis	True	1.428e+06	-0.7099	-0.7099	-0.08347
13	Guanosine 5'-PP-Fucose	Carbon	1	Ctrl_Untreated_1_4	Ctrl_Untreated_1	1.0	Polysaccharide biosynthesis	True	6.513e+05	-1.843	-1.843	0.4564

Metabolite ID	Name	Super Pathway	Dataset	Sample ID	Group ID	Detection Fraction	Pathway	Detected	Raw Intensity	Log2 Norm Intensity	Norm Imputed	Log2 Ctrl Norm
19	Maltotetraose	Carbon	1	Ctrl_Untreated_1_4	Ctrl_Untreated_1	1.0	Glycogen degradation	True	3.582e+06	0.6164	0.6164	0.6601
233	SAM	Amino acid	1	Ctrl_Untreated_1_4	Ctrl_Untreated_1	1.0	SAM metabolism	True	2.358e+06	0.01341	0.01341	0.1697
129	5-Aminovalerate	Amino acid	1	Ctrl_Untreated_1_4	Ctrl_Untreated_1	1.0	Amino acids degradation intermediates	True	3.752e+06	0.6833	0.6833	-0.3183
741	5-Me-THF	Cofactor	1	Ctrl_Untreated_1_4	Ctrl_Untreated_1	0.714285714285714	Folate metabolism	True	1.505e+05	-3.956	-3.956	0.8014
198	Indolelactate	Amino acid	1	Ctrl_Untreated_1_4	Ctrl_Untreated_1	0.857142857142857	Amino acid derivatives	True	1.622e+05	-3.848	-3.848	-0.06123
254	Gly-Val	Amino acid	1	Ctrl_Untreated_1_4	Ctrl_Untreated_1	1.0	Dipeptides	True	7.947e+06	1.766	1.766	0.6547
291	gamma-Glu-Leu	Amino acid	1	Ctrl_Untreated_1_4	Ctrl_Untreated_1	1.0	Gamma-glutamyl dipeptides	True	2.698e+06	0.2078	0.2078	0.6965
173	Met Sulfoxide	Amino acid	1	Ctrl_Untreated_1_4	Ctrl_Untreated_1	1.0	Amino acid derivatives	True	2.079e+07	3.154	3.154	0.5862
43	Glucose	Carbon	1	Ctrl_Untreated_1_4	Ctrl_Untreated_1	1.0	Glycolysis, GNG	True	6.934e+07	4.891	4.891	-0.1601
185	Phenyllactate	Amino acid	1	Ctrl_Untreated_1_4	Ctrl_Untreated_1	0.285714285714286	Amino acid derivatives	False			-5.917	-0.1051
156	Homo-Arg	Amino acid	1	Ctrl_Untreated_1_4	Ctrl_Untreated_1	1.0	Amino acid derivatives	True	8.802e+06	1.914	1.914	0.733
135	Homocitrulline	Amino acid	1	Ctrl_Untreated_1_4	Ctrl_Untreated_1	1.0	Amino acids degradation intermediates	True	6.854e+05	-1.769	-1.769	-0.06667
719	Nicotinamide MN	Cofactor	1	Ctrl_Untreated_1_4	Ctrl_Untreated_1	1.0	NAD biosynthesis	True	1.292e+07	2.467	2.467	2.282
212	N-Ac-Asp	Amino acid	1	Ctrl_Untreated_1_4	Ctrl_Untreated_1	1.0	N-acetylated amino acids	True	2.063e+06	-0.1793	-0.1793	-0.8767
720	1-Me-Nicotinamide	Cofactor	1	Ctrl_Untreated_1_4	Ctrl_Untreated_1	1.0	Derivatives of NA, nicotinamide and NAD	True	9.294e+08	8.636	8.636	0.7452
216	N-Ac-Gly	Amino acid	1	Ctrl_Untreated_1_4	Ctrl_Untreated_1	0.714285714285714	N-acetylated amino acids	True	1.982e+05	-3.559	-3.559	-0.0194
70	Creatine	Carbon	1	Ctrl_Untreated_1_4	Ctrl_Untreated_1	1.0	Creatine energy storage	True	1.624e+09	9.441	9.441	0.02429
26	Galactonate	Carbon	1	Ctrl_Untreated_1_4	Ctrl_Untreated_1	0.857142857142857	Sugars and sugar alcohols	True	2.214e+06	-0.07798	-0.07798	0.7266
309	Glutathione, Oxidized	Amino acid	1	Ctrl_Untreated_1_4	Ctrl_Untreated_1	1.0	Glutathione	True	3.718e+06	0.6701	0.6701	-0.5226
35	Ribonate	Carbon	1	Ctrl_Untreated_1_4	Ctrl_Untreated_1	1.0	Sugars and sugar alcohols	True	1.649e+06	-0.5023	-0.5023	0.02493
160	1-Me-His	Amino acid	1	Ctrl_Untreated_1_4	Ctrl_Untreated_1	1.0	Amino acid derivatives	True	2.268e+07	3.279	3.279	-0.1623

Metabolite ID	Name	Super Pathway	Dataset	Sample ID	Group ID	Detection Fraction	Pathway	Detected	Raw Intensity	Log2 Norm Intensity	Norm Imputed	Log2 Ctrl Norm
44	Glucose 6-P	Carbon	1	Ctrl_Untreated_1_4	Ctrl_Untreated_1	0.857142857142857	Glycolysis, GNG	True	3.55e+05	-2.718	-2.718	-1.242
704	NADH	Cofactor	1	Ctrl_Untreated_1_4	Ctrl_Untreated_1	1.0	Cofactors	True	1.771e+06	-0.3998	-0.3998	0.2462
275	Thr-Phe	Amino acid	1	Ctrl_Untreated_1_4	Ctrl_Untreated_1	0.857142857142857	Dipeptides	True	1.556e+05	-3.909	-3.909	-2.255
738	Pyridoxate	Cofactor	1	Ctrl_Untreated_1_4	Ctrl_Untreated_1	1.0	PLP biosynthesis and salvage	True	1.532e+05	-3.931	-3.931	-0.5663
177	3-(4-OH-Phenyl)Lactate	Amino acid	1	Ctrl_Untreated_1_4	Ctrl_Untreated_1	1.0	Amino acid derivatives	True	3.777e+05	-2.629	-2.629	-0.1639
206	Trans-4-OH-Pro	Amino acid	1	Ctrl_Untreated_1_4	Ctrl_Untreated_1	1.0	Amino acid derivatives	True	8.681e+07	5.216	5.216	0.06032
329	AMP	Nucleotide	1	Ctrl_Untreated_1_4	Ctrl_Untreated_1	1.0	Purine nucleotides	True	1.272e+07	2.444	2.444	-1.191
11	UDP-Glucose	Carbon	1	Ctrl_Untreated_1_4	Ctrl_Untreated_1	1.0	Polysaccharide biosynthesis	True	1.735e+06	-0.4293	-0.4293	-0.3241
158	4-Imidazole-Ac	Amino acid	1	Ctrl_Untreated_1_4	Ctrl_Untreated_1	1.0	Amino acid derivatives	True	4.348e+05	-2.426	-2.426	-0.2793
111	1-Me-Imidazole-Ac	Amino acid	1	Ctrl_Untreated_1_4	Ctrl_Untreated_1	1.0	Amino acids degradation intermediates	True	6.647e+05	-1.814	-1.814	0.1203
345	Guanine	Nucleotide	1	Ctrl_Untreated_1_4	Ctrl_Untreated_1	1.0	Purine bases	True	1.695e+08	6.181	6.181	1.195
22	N-Ac-Neuraminate	Carbon	1	Ctrl_Untreated_1_4	Ctrl_Untreated_1	1.0	Aminosugar derivatives	True	2.452e+06	0.06992	0.06992	0.2053
721	N'-Methylnicotinate	Cofactor	1	Ctrl_Untreated_1_4	Ctrl_Untreated_1	1.0	Derivatives of NA, nicotinamide and NAD	True	2.335e+06	-9.25e-04	-9.25e-04	1.007
183	Phenol Sulfate	Amino acid	1	Ctrl_Untreated_1_4	Ctrl_Untreated_1	0.857142857142857	Amino acid derivatives	True	4.433e+04	-5.72	-5.72	-1.08
718	Nicotinamide Riboside	Cofactor	1	Ctrl_Untreated_1_4	Ctrl_Untreated_1	1.0	NAD biosynthesis	True	1.475e+08	5.98	5.98	4.39
297	gamma-Glu-Thr	Amino acid	1	Ctrl_Untreated_1_4	Ctrl_Untreated_1	1.0	Gamma-glutamyl dipeptides	True	5.795e+06	1.31	1.31	0.6702
295	gamma-Glu-Phe	Amino acid	1	Ctrl_Untreated_1_4	Ctrl_Untreated_1	0.428571428571429	Gamma-glutamyl dipeptides	True	7.612e+04	-4.94	-4.94	0.8452
347	Allantoic Acid	Nucleotide	1	Ctrl_Untreated_1_4	Ctrl_Untreated_1	0.571428571428571	Purine degradation	True	5.582e+04	-5.387	-5.387	0.2521
399	Pseudouridine	Nucleotide	1	Ctrl_Untreated_1_4	Ctrl_Untreated_1	1.0	Pyrimidine derivatives in RNAs	True	6.577e+05	-1.829	-1.829	0.2012
375	UTP	Nucleotide	1	Ctrl_Untreated_1_4	Ctrl_Untreated_1	0.571428571428571	Pyrimidine nucleotides	False			-5.025	-3.049
144	Glu, gamma-Me Ester	Amino acid	1	Ctrl_Untreated_1_4	Ctrl_Untreated_1	1.0	Amino acid derivatives	True	5.815e+06	1.316	1.316	0.7378

Metabolite ID	Name	Super Pathway	Dataset	Sample ID	Group ID	Detection Fraction	Pathway	Detected	Raw Intensity	Log2 Norm Intensity	Norm Imputed	Log2 Ctrl Norm
292	gamma-Glu-epsilon-Lysine	Amino acid	1	Ctrl_Untreated_1_4	Ctrl_Untreated_1	1.0	Gamma-glutamyl dipeptides	True	3.539e+06	0.5991	0.5991	0.7905
225	N-Ac-Thr	Amino acid	1	Ctrl_Untreated_1_4	Ctrl_Untreated_1	1.0	N-acetylated amino acids	True	1.199e+06	-0.9619	-0.9619	0.5407
211	N-Ac-Asn	Amino acid	1	Ctrl_Untreated_1_4	Ctrl_Untreated_1	1.0	N-acetylated amino acids	True	4.628e+05	-2.336	-2.336	0.3492
151	Phenylacetylglycine	Amino acid	1	Ctrl_Untreated_1_4	Ctrl_Untreated_1	1.0	Amino acid derivatives	True	1.108e+06	-1.077	-1.077	-0.29
217	N-Ac-His	Amino acid	1	Ctrl_Untreated_1_4	Ctrl_Untreated_1	1.0	N-acetylated amino acids	True	3.815e+05	-2.615	-2.615	0.8704
288	gamma-Glu-Gly	Amino acid	1	Ctrl_Untreated_1_4	Ctrl_Untreated_1	0.571428571428571	Gamma-glutamyl dipeptides	True	2.239e+06	-0.06119	-0.06119	1.763
222	N-Ac-Phe	Amino acid	1	Ctrl_Untreated_1_4	Ctrl_Untreated_1	0.571428571428571	N-acetylated amino acids	True	3.739e+04	-5.965	-5.965	0.3071
71	Creatine-P	Carbon	1	Ctrl_Untreated_1_4	Ctrl_Untreated_1	1.0	Creatine energy storage	True	2.259e+05	-3.371	-3.371	0.8844
210	N-Ac-Arg	Amino acid	1	Ctrl_Untreated_1_4	Ctrl_Untreated_1	1.0	N-acetylated amino acids	True	8.283e+05	-1.496	-1.496	1.18
218	N-Ac-Ile	Amino acid	1	Ctrl_Untreated_1_4	Ctrl_Untreated_1	0.428571428571429	N-acetylated amino acids	True	5.814e+04	-5.329	-5.329	0.6372
251	Gly-Leu	Amino acid	1	Ctrl_Untreated_1_4	Ctrl_Untreated_1	1.0	Dipeptides	True	1.181e+07	2.338	2.338	0.6995
290	gamma-Glu-Ile	Amino acid	1	Ctrl_Untreated_1_4	Ctrl_Untreated_1	1.0	Gamma-glutamyl dipeptides	True	2.146e+06	-0.1225	-0.1225	1.216
316	Ophthalmate	Amino acid	1	Ctrl_Untreated_1_4	Ctrl_Untreated_1	1.0	Oxidative stress markers	True	2.823e+07	3.595	3.595	1.252
125	Isovaleryl-Gly	Amino acid	1	Ctrl_Untreated_1_4	Ctrl_Untreated_1	0.857142857142857	Amino acids degradation intermediates	True	4.309e+04	-5.761	-5.761	-0.3513
368	7-Me-Guanine	Nucleotide	1	Ctrl_Untreated_1_4	Ctrl_Untreated_1	1.0	Purine derivatives in RNAs	True	9.892e+05	-1.24	-1.24	0.95
208	Pro-OH-Pro	Amino acid	1	Ctrl_Untreated_1_4	Ctrl_Untreated_1	1.0	Amino acid derivatives	True	2.277e+07	3.285	3.285	-0.04474
366	N2,N2-DiMe-Guanosine	Nucleotide	1	Ctrl_Untreated_1_4	Ctrl_Untreated_1	0.857142857142857	Purine derivatives in RNAs	True	2.117e+05	-3.464	-3.464	0.6492
352	3'-AMP	Nucleotide	1	Ctrl_Untreated_1_4	Ctrl_Untreated_1	1.0	Purine derivatives in signaling	True	8.199e+05	-1.511	-1.511	-0.02396
363	N6-Carbamoyl-Thr-Adenosine	Nucleotide	1	Ctrl_Untreated_1_4	Ctrl_Untreated_1	0.857142857142857	Purine derivatives in RNAs	True	2.244e+05	-3.38	-3.38	1.305
314	Cys-Glutathione Disulfide	Amino acid	1	Ctrl_Untreated_1_4	Ctrl_Untreated_1	1.0	Oxidative stress markers	True	5.616e+05	-2.057	-2.057	1.227
382	Orotidine	Nucleotide	1	Ctrl_Untreated_1_4	Ctrl_Untreated_1	0.428571428571429	Pyrimidine (UMP) biosynthesis	True	1.809e+05	-3.691	-3.691	0.6006

Metabolite ID	Name	Super Pathway	Dataset	Sample ID	Group ID	Detection Fraction	Pathway	Detected	Raw Intensity	Log2 Norm Intensity	Norm Imputed	Log2 Ctrl Norm
307	Cys-Gly	Amino acid	1	Ctrl_Untreated_1_4	Ctrl_Untreated_1	1.0	Glutathione biosynthesis	True	5.414e+06	1.212	1.212	-0.3945
64	Sedoheptulose-7-P	Carbon	1	Ctrl_Untreated_1_4	Ctrl_Untreated_1	1.0	Pentose phosphate pathway (PPP)	True	2.275e+06	-0.03857	-0.03857	-0.1927
142	N-Ac-Asp-Glu	Amino acid	1	Ctrl_Untreated_1_4	Ctrl_Untreated_1	1.0	Amino acid derivatives	True	1.38e+06	-0.7593	-0.7593	0.3861
708	Thiamin-PP	Cofactor	1	Ctrl_Untreated_1_4	Ctrl_Untreated_1	0.571428571428571	Cofactors	True	4.254e+04	-5.779	-5.779	0.2043
182	P-Cresol Sulfate	Amino acid	1	Ctrl_Untreated_1_4	Ctrl_Untreated_1	0.857142857142857	Amino acid derivatives	True	2.237e+05	-3.385	-3.385	-0.1886
250	Gly-Ile	Amino acid	1	Ctrl_Untreated_1_4	Ctrl_Untreated_1	1.0	Dipeptides	True	9.169e+05	-1.349	-1.349	-0.03225
286	gamma-Glu-Glu	Amino acid	1	Ctrl_Untreated_1_4	Ctrl_Untreated_1	1.0	Gamma-glutamyl dipeptides	True	4.055e+06	0.7953	0.7953	0.7775
739	Deoxycarnitine	Cofactor	1	Ctrl_Untreated_1_4	Ctrl_Untreated_1	1.0	Carnitine biosynthesis	True	7.125e+07	4.931	4.931	1.179
203	DiMe-Arg	Amino acid	1	Ctrl_Untreated_1_4	Ctrl_Untreated_1	1.0	Amino acid derivatives	True	5.147e+08	7.783	7.783	2.151
351	2'-AMP	Nucleotide	1	Ctrl_Untreated_1_4	Ctrl_Untreated_1	1.0	Purine derivatives in signaling	True	1.627e+06	-0.5222	-0.5222	1.746
8	Cytidine 5'-P-N-Ac-Neuraminate	Carbon	1	Ctrl_Untreated_1_4	Ctrl_Untreated_1	1.0	Aminosugar biosynthesis	True	8.242e+05	-1.503	-1.503	0.2423
285	gamma-Glu-Ala	Amino acid	1	Ctrl_Untreated_1_4	Ctrl_Untreated_1	0.857142857142857	Gamma-glutamyl dipeptides	True	9.441e+05	-1.307	-1.307	1.931
224	N-Ac-Ser	Amino acid	1	Ctrl_Untreated_1_4	Ctrl_Untreated_1	1.0	N-acetylated amino acids	True	1.325e+07	2.503	2.503	0.6241
244	Ala-Leu	Amino acid	1	Ctrl_Untreated_1_4	Ctrl_Untreated_1	1.0	Dipeptides	True	2.338e+06	9.25e-04	9.25e-04	-1.137
207	N-Me-Pro	Amino acid	1	Ctrl_Untreated_1_4	Ctrl_Untreated_1	1.0	Amino acid derivatives	True	1.13e+06	-1.048	-1.048	-0.1181
171	Cys Sulfinic Acid	Amino acid	1	Ctrl_Untreated_1_4	Ctrl_Untreated_1	0.857142857142857	Amino acid derivatives	True	3.236e+05	-2.852	-2.852	0.4455
181	O-Me-Tyr	Amino acid	1	Ctrl_Untreated_1_4	Ctrl_Untreated_1	0.857142857142857	Amino acid derivatives	True	2.441e+05	-3.259	-3.259	0.1111
240	N-Ac-Putrescine	Amino acid	1	Ctrl_Untreated_1_4	Ctrl_Untreated_1	1.0	Polyamine derivatives	True	1.92e+05	-3.606	-3.606	-1.242
176	S-Me-Met	Amino acid	1	Ctrl_Untreated_1_4	Ctrl_Untreated_1	0.857142857142857	Amino acid derivatives	True	5.923e+05	-1.98	-1.98	-0.1382
339	AICAR	Nucleotide	1	Ctrl_Untreated_1_4	Ctrl_Untreated_1	0.571428571428571	IMP biosynthesis	True	1.982e+05	-3.559	-3.559	0.7417
141	gamma-Carboxy-Glu	Amino acid	1	Ctrl_Untreated_1_4	Ctrl_Untreated_1	1.0	Amino acid derivatives	True	9.976e+05	-1.228	-1.228	-0.4428

Metabolite ID	Name	Super Pathway	Dataset	Sample ID	Group ID	Detection Fraction	Pathway	Detected	Raw Intensity	Log2 Norm Intensity	Norm Imputed	Log2 Ctrl Norm
392	3'-UMP	Nucleotide	1	Ctrl_Untreated_1_4	Ctrl_Untreated_1	0.571428571428571	Pyrimidine derivatives in signaling	True	1.154e+05	-4.34	-4.34	-0.3063
355	3'-GMP	Nucleotide	1	Ctrl_Untreated_1_4	Ctrl_Untreated_1	0.857142857142857	Purine derivatives in signaling	True	4.095e+04	-5.834	-5.834	-1.897
282	Val-Leu	Amino acid	1	Ctrl_Untreated_1_4	Ctrl_Untreated_1	1.0	Dipeptides	True	1.835e+06	-0.3489	-0.3489	-1.321
140	Carboxyethyl-GABA	Amino acid	1	Ctrl_Untreated_1_4	Ctrl_Untreated_1	1.0	Amino acid derivatives	True	3.936e+06	0.7525	0.7525	2.184
258	Ile-Gly	Amino acid	1	Ctrl_Untreated_1_4	Ctrl_Untreated_1	1.0	Dipeptides	True	2.79e+07	3.578	3.578	0.266
260	Leu-Ala	Amino acid	1	Ctrl_Untreated_1_4	Ctrl_Untreated_1	1.0	Dipeptides	True	2.429e+06	0.05619	0.05619	-1.245
265	Lys-Leu	Amino acid	1	Ctrl_Untreated_1_4	Ctrl_Untreated_1	0.857142857142857	Dipeptides	True	1.969e+05	-3.569	-3.569	-0.9577
263	Leu-Gly	Amino acid	1	Ctrl_Untreated_1_4	Ctrl_Untreated_1	1.0	Dipeptides	True	4.559e+06	0.9645	0.9645	-2.057
281	Val-Gly	Amino acid	1	Ctrl_Untreated_1_4	Ctrl_Untreated_1	1.0	Dipeptides	True	5.696e+07	4.608	4.608	0.08843
270	Pro-Gly	Amino acid	1	Ctrl_Untreated_1_4	Ctrl_Untreated_1	1.0	Dipeptides	True	8.556e+06	1.873	1.873	1.126
114	Imidazole Propionate	Amino acid	1	Ctrl_Untreated_1_4	Ctrl_Untreated_1	1.0	Amino acids degradation intermediates	True	2.973e+05	-2.974	-2.974	-0.6638
267	Phe-Gly	Amino acid	1	Ctrl_Untreated_1_4	Ctrl_Untreated_1	1.0	Dipeptides	True	5.694e+07	4.607	4.607	0.5134
266	Phe-Ala	Amino acid	1	Ctrl_Untreated_1_4	Ctrl_Untreated_1	1.0	Dipeptides	True	2.419e+06	0.04986	0.04986	-1.027
278	Tyr-Gly	Amino acid	1	Ctrl_Untreated_1_4	Ctrl_Untreated_1	1.0	Dipeptides	True	1.249e+07	2.418	2.418	0.2358
255	His-Ala	Amino acid	1	Ctrl_Untreated_1_4	Ctrl_Untreated_1	1.0	Dipeptides	True	1.128e+06	-1.051	-1.051	0.2866
280	Val-Gln	Amino acid	1	Ctrl_Untreated_1_4	Ctrl_Untreated_1	1.0	Dipeptides	True	3.485e+06	0.5769	0.5769	-1.041
143	S-1-Pyrroline-5-Carboxylate	Amino acid	1	Ctrl_Untreated_1_4	Ctrl_Untreated_1	0.857142857142857	Amino acid derivatives	True	4.057e+05	-2.526	-2.526	-0.4576
232	SAH	Amino acid	1	Ctrl_Untreated_1_4	Ctrl_Untreated_1	1.0	SAM metabolism	True	2.588e+06	0.1478	0.1478	0.6332
20	Erythronate	Carbon	1	Ctrl_Untreated_1_4	Ctrl_Untreated_1	1.0	Aminosugar derivatives	True	2.926e+07	3.647	3.647	-0.09469
248	Gln-Leu	Amino acid	1	Ctrl_Untreated_1_4	Ctrl_Untreated_1	1.0	Dipeptides	True	4.594e+05	-2.346	-2.346	-1.441
276	Trp-Gly	Amino acid	1	Ctrl_Untreated_1_4	Ctrl_Untreated_1	1.0	Dipeptides	True	1.617e+06	-0.5306	-0.5306	0.5478
205	N-delta-Ac-Ornithine	Amino acid	1	Ctrl_Untreated_1_4	Ctrl_Untreated_1	1.0	Amino acid derivatives	True	1.042e+06	-1.166	-1.166	-0.04448
163	Formimino-Glu	Amino acid	1	Ctrl_Untreated_1_4	Ctrl_Untreated_1	1.0	Amino acid derivatives	True	1.38e+06	-0.7591	-0.7591	0.4807
204	N-Me-Arg	Amino acid	1	Ctrl_Untreated_1_4	Ctrl_Untreated_1	1.0	Amino acid derivatives	True	4.611e+07	4.303	4.303	1.716
242	Guanidino-Ac	Amino acid	1	Ctrl_Untreated_1_4	Ctrl_Untreated_1	0.857142857142857	Creatine biosynthesis	True	1.601e+05	-3.867	-3.867	0.01311

Metabolite ID	Name	Super Pathway	Dataset	Sample ID	Group ID	Detection Fraction	Pathway	Detected	Raw Intensity	Log2 Norm Intensity	Norm Imputed	Log2 Ctrl Norm
300	gamma-Glu-Val	Amino acid	1	Ctrl_Untreated_1_4	Ctrl_Untreated_1	0.857142857142857	Gamma-glutamyl dipeptides	True	8.41e+06	1.848	1.848	1.227
53	Ac-CoA	Carbon	1	Ctrl_Untreated_1_4	Ctrl_Untreated_1	0.285714285714286	Acetyl-CoA	False			-7.274	-0.5114
18	Maltotriose	Carbon	1	Ctrl_Untreated_1_4	Ctrl_Untreated_1	1.0	Glycogen degradation	True	4.101e+06	0.8116	0.8116	0.9694
294	gamma-Glu-Met	Amino acid	1	Ctrl_Untreated_1_4	Ctrl_Untreated_1	0.714285714285714	Gamma-glutamyl dipeptides	True	1.247e+06	-0.9058	-0.9058	2.576
174	Met Sulfone	Amino acid	1	Ctrl_Untreated_1_4	Ctrl_Untreated_1	1.0	Amino acid derivatives	True	3.107e+05	-2.911	-2.911	0.1678
175	N-Ac-Met Sulfoxide	Amino acid	1	Ctrl_Untreated_1_4	Ctrl_Untreated_1	1.0	Amino acid derivatives	True	4.726e+06	1.016	1.016	1.04
25	Mannitol/Sorbitol	Carbon	1	Ctrl_Untreated_1_4	Ctrl_Untreated_1	1.0	Sugars and sugar alcohols	True	1.179e+07	2.335	2.335	0.1102
6	UDP-GlcNAc	Carbon	1	Ctrl_Untreated_1_4	Ctrl_Untreated_1	0.428571428571429	Aminosugar biosynthesis	False			-3.589	-0.4814
145	Pyro-Gln	Amino acid	1	Ctrl_Untreated_1_4	Ctrl_Untreated_1	1.0	Amino acid derivatives	True	4.033e+06	0.7876	0.7876	-0.05216
705	Coenzyme A	Cofactor	1	Ctrl_Untreated_1_4	Ctrl_Untreated_1	0.714285714285714	Cofactors	True	8.33e+04	-4.81	-4.81	-0.8508
319	2'-dAMP	Nucleotide	1	Ctrl_Untreated_1_4	Ctrl_Untreated_1	0.571428571428571	Deoxy-nucleotides	False			-4.337	-0.6568
119	alpha-OH-Isovalerate	Amino acid	1	Ctrl_Untreated_1_4	Ctrl_Untreated_1	0.714285714285714	Amino acids degradation intermediates	True	3.664e+05	-2.673	-2.673	0.6255
46	Fructose 1,6-PP / Glucose 1,6-PP / Inositol-1,4-PP / Inositol-1,3-PP	Carbon	1	Ctrl_Untreated_1_4	Ctrl_Untreated_1	1.0	Glycolysis, GNG	True	6.95e+07	4.895	4.895	0.06363
137	1-Me-Guanidine	Amino acid	1	Ctrl_Untreated_1_4	Ctrl_Untreated_1	0.857142857142857	Amino acids degradation intermediates	True	8.137e+04	-4.844	-4.844	0.309
23	N-GlcNAc-Asn	Carbon	1	Ctrl_Untreated_1_4	Ctrl_Untreated_1	1.0	Aminosugar derivatives	True	9.174e+06	1.973	1.973	1.26
262	Leu-Gln	Amino acid	1	Ctrl_Untreated_1_4	Ctrl_Untreated_1	1.0	Dipeptides	True	3.112e+06	0.4135	0.4135	-0.8829
24	Fructose	Carbon	1	Ctrl_Untreated_1_4	Ctrl_Untreated_1	1.0	Sugars and sugar alcohols	True	9.059e+06	1.955	1.955	6.20e-03
197	C-Glycosyl-Trp	Amino acid	1	Ctrl_Untreated_1_4	Ctrl_Untreated_1	1.0	Amino acid derivatives	True	5.28e+06	1.176	1.176	1.475
33	Arabitol/Xylitol	Carbon	1	Ctrl_Untreated_1_4	Ctrl_Untreated_1	1.0	Sugars and sugar alcohols	True	1.191e+06	-0.9724	-0.9724	0.8876

Metabolite ID	Name	Super Pathway	Dataset	Sample ID	Group ID	Detection Fraction	Pathway	Detected	Raw Intensity	Log2 Norm Intensity	Norm Imputed	Log2 Ctrl Norm
128	N2-Ac-Lys/N6-Ac-Lys	Amino acid	1	Ctrl_Untreated_1_4	Ctrl_Untreated_1	1.0	Amino acids degradation intermediates	True	1.959e+07	3.068	3.068	1.857
42	2-Me-Citrate	Carbon	1	Ctrl_Untreated_1_4	Ctrl_Untreated_1	0.857142857142857	Propionate metabolism	True	1.411e+05	-4.05	-4.05	0.3335
12	Glucuronate 1-P	Carbon	1	Ctrl_Untreated_1_4	Ctrl_Untreated_1	1.0	Polysaccharide biosynthesis	True	1.283e+06	-0.8653	-0.8653	0.3776
76	Gln	Amino acid	1	Ctrl_Untreated_1_5	Ctrl_Untreated_1	1.0	Proteinogenic amino acids	True	9.843e+08	8.714	8.714	0.0333
89	Trp	Amino acid	1	Ctrl_Untreated_1_5	Ctrl_Untreated_1	1.0	Proteinogenic amino acids	True	1.53e+08	6.029	6.029	0.353
723	beta-Ala	Cofactor	1	Ctrl_Untreated_1_5	Ctrl_Untreated_1	1.0	Coenzyme A biosynthesis	True	9.608e+06	2.035	2.035	-0.9741
75	Glu	Amino acid	1	Ctrl_Untreated_1_5	Ctrl_Untreated_1	1.0	Proteinogenic amino acids	True	3.541e+09	10.56	10.56	-0.1756
77	Gly	Amino acid	1	Ctrl_Untreated_1_5	Ctrl_Untreated_1	1.0	Proteinogenic amino acids	True	1.634e+08	6.123	6.123	0.508
80	His	Amino acid	1	Ctrl_Untreated_1_5	Ctrl_Untreated_1	1.0	Proteinogenic amino acids	True	3.881e+07	4.049	4.049	0.634
82	Leu	Amino acid	1	Ctrl_Untreated_1_5	Ctrl_Untreated_1	1.0	Proteinogenic amino acids	True	2.111e+09	9.815	9.815	0.5937
87	Phe	Amino acid	1	Ctrl_Untreated_1_5	Ctrl_Untreated_1	1.0	Proteinogenic amino acids	True	1.447e+09	9.27	9.27	0.4588
130	Glutarate	Amino acid	1	Ctrl_Untreated_1_5	Ctrl_Untreated_1	1.0	Amino acids degradation intermediates	True	6.826e+05	-1.78	-1.78	0.6467
196	5-OH-Indole-Ac	Amino acid	1	Ctrl_Untreated_1_5	Ctrl_Untreated_1	0.857142857142857	Amino acid derivatives	True	2.068e+05	-3.503	-3.503	0.5092
74	Asp	Amino acid	1	Ctrl_Untreated_1_5	Ctrl_Untreated_1	1.0	Proteinogenic amino acids	True	5.844e+08	7.962	7.962	0.06995
236	Spermidine	Amino acid	1	Ctrl_Untreated_1_5	Ctrl_Untreated_1	1.0	Polyamines	True	2.505e+07	3.417	3.417	-0.4442
73	Asn	Amino acid	1	Ctrl_Untreated_1_5	Ctrl_Untreated_1	1.0	Proteinogenic amino acids	True	3.488e+08	7.217	7.217	0.5461
243	Creatinine	Amino acid	1	Ctrl_Untreated_1_5	Ctrl_Untreated_1	1.0	Creatine degradation	True	6.502e+07	4.794	4.794	-0.2084
376	Cytidine	Nucleotide	1	Ctrl_Untreated_1_5	Ctrl_Untreated_1	0.714285714285714	Pyrimidine nucleosides	True	2.648e+06	0.176	0.176	-0.5517
41	Lactate	Carbon	1	Ctrl_Untreated_1_5	Ctrl_Untreated_1	1.0	Respiratory carbon sources	True	2.013e+08	6.424	6.424	-0.05774
58	alpha-Ketoglutarate	Carbon	1	Ctrl_Untreated_1_5	Ctrl_Untreated_1	1.0	TCA cycle	True	3.064e+06	0.3864	0.3864	0.3697
69	3-OH-Butyrate	Carbon	1	Ctrl_Untreated_1_5	Ctrl_Untreated_1	0.571428571428571	Ketone bodies	False			-3.497	-0.5127

Metabolite ID	Name	Super Pathway	Dataset	Sample ID	Group ID	Detection Fraction	Pathway	Detected	Raw Intensity	Log2 Norm Intensity	Norm Imputed	Log2 Ctrl Norm
343	Adenine	Nucleotide	1	Ctrl_Untreated_1_5	Ctrl_Untreated_1	1.0	Purine bases	True	4.202e+06	0.842	0.842	-0.6149
336	Adenosine	Nucleotide	1	Ctrl_Untreated_1_5	Ctrl_Untreated_1	1.0	Purine nucleosides	True	1.399e+07	2.577	2.577	-0.8455
722	ADP-Ribose	Cofactor	1	Ctrl_Untreated_1_5	Ctrl_Untreated_1	1.0	Derivatives of NA, nicotinamide and NAD	True	8.458e+04	-4.793	-4.793	-1.79
383	Cytosine	Nucleotide	1	Ctrl_Untreated_1_5	Ctrl_Untreated_1	0.571428571428571	Pyrimidine bases	False			-5.32	-0.4652
3	Glucosamine 6-P	Carbon	1	Ctrl_Untreated_1_5	Ctrl_Untreated_1	1.0	Aminosugar biosynthesis	True	2.586e+05	-3.18	-3.18	-1.513
717	Nicotinamide	Cofactor	1	Ctrl_Untreated_1_5	Ctrl_Untreated_1	1.0	NAD biosynthesis	True	1.077e+08	5.522	5.522	1.006
51	PEP	Carbon	1	Ctrl_Untreated_1_5	Ctrl_Untreated_1	1.0	Glycolysis, GNG	True	9.753e+06	2.057	2.057	1.006
237	Spermine	Amino acid	1	Ctrl_Untreated_1_5	Ctrl_Untreated_1	1.0	Polyamines	True	2.042e+06	-0.1994	-0.1994	1.291
385	Uracil	Nucleotide	1	Ctrl_Untreated_1_5	Ctrl_Untreated_1	1.0	Pyrimidine bases	True	3.202e+07	3.772	3.772	3.507
377	Uridine	Nucleotide	1	Ctrl_Untreated_1_5	Ctrl_Untreated_1	1.0	Pyrimidine nucleosides	True	7.407e+07	4.982	4.982	0.9329
348	Allantoin	Nucleotide	1	Ctrl_Untreated_1_5	Ctrl_Untreated_1	1.0	Purine degradation	True	2.414e+06	0.0426	0.0426	0.2182
335	Inosine	Nucleotide	1	Ctrl_Untreated_1_5	Ctrl_Untreated_1	1.0	Purine nucleosides	True	1.543e+08	6.04	6.04	0.5414
81	Ile	Amino acid	1	Ctrl_Untreated_1_5	Ctrl_Untreated_1	1.0	Proteinogenic amino acids	True	1.244e+09	9.051	9.051	0.3361
72	Ala	Amino acid	1	Ctrl_Untreated_1_5	Ctrl_Untreated_1	1.0	Proteinogenic amino acids	True	8.866e+08	8.563	8.563	0.06172
79	Thr	Amino acid	1	Ctrl_Untreated_1_5	Ctrl_Untreated_1	1.0	Proteinogenic amino acids	True	9.046e+08	8.592	8.592	0.5826
88	Tyr	Amino acid	1	Ctrl_Untreated_1_5	Ctrl_Untreated_1	1.0	Proteinogenic amino acids	True	6.921e+08	8.206	8.206	0.6822
84	Lys	Amino acid	1	Ctrl_Untreated_1_5	Ctrl_Untreated_1	1.0	Proteinogenic amino acids	True	7.773e+08	8.373	8.373	0.3832
86	Met	Amino acid	1	Ctrl_Untreated_1_5	Ctrl_Untreated_1	1.0	Proteinogenic amino acids	True	1.103e+09	8.878	8.878	0.65
61	Malate	Carbon	1	Ctrl_Untreated_1_5	Ctrl_Untreated_1	1.0	TCA cycle	True	4.588e+08	7.613	7.613	0.4409
235	Putrescine	Amino acid	1	Ctrl_Untreated_1_5	Ctrl_Untreated_1	1.0	Polyamines	True	6.861e+05	-1.772	-1.772	-1.281
324	2'-dU	Nucleotide	1	Ctrl_Untreated_1_5	Ctrl_Untreated_1	0.714285714285714	Deoxy-nucleosides	True	2.675e+05	-3.132	-3.132	1.249
49	3-P-Glycerate	Carbon	1	Ctrl_Untreated_1_5	Ctrl_Untreated_1	1.0	Glycolysis, GNG	True	3.201e+07	3.771	3.771	0.5058
189	Kynurenate	Amino acid	1	Ctrl_Untreated_1_5	Ctrl_Untreated_1	0.714285714285714	Amino acid derivatives	True	4.126e+04	-5.828	-5.828	-0.03023
234	5-Me-Thioadenosine	Amino acid	1	Ctrl_Untreated_1_5	Ctrl_Untreated_1	1.0	SAM metabolism	True	5.784e+06	1.303	1.303	-0.5825
59	Succinate	Carbon	1	Ctrl_Untreated_1_5	Ctrl_Untreated_1	1.0	TCA cycle	True	1.225e+06	-0.9366	-0.9366	-1.238

Metabolite ID	Name	Super Pathway	Dataset	Sample ID	Group ID	Detection Fraction	Pathway	Detected	Raw Intensity	Log2 Norm Intensity	Norm Imputed	Log2 Ctrl Norm
36	Ribose	Carbon	1	Ctrl_Untreated_1_5	Ctrl_Untreated_1	1.0	Sugars and sugar alcohols	True	1.855e+06	-0.3377	-0.3377	0.8957
133	Ornithine	Amino acid	1	Ctrl_Untreated_1_5	Ctrl_Untreated_1	1.0	Amino acids degradation intermediates	True	3.554e+07	3.922	3.922	-0.5571
313	5-Oxoproline	Amino acid	1	Ctrl_Untreated_1_5	Ctrl_Untreated_1	1.0	Glutathione derivatives	True	8.895e+06	1.924	1.924	-0.1635
165	N-6-Tri-Me-Lys	Amino acid	1	Ctrl_Untreated_1_5	Ctrl_Untreated_1	1.0	Amino acid derivatives	True	4.115e+07	4.134	4.134	0.37
380	Orotate	Nucleotide	1	Ctrl_Untreated_1_5	Ctrl_Untreated_1	0.857142857142857	Pyrimidine (UMP) biosynthesis	True	2.034e+05	-3.526	-3.526	0.1038
724	Pantothenate	Cofactor	1	Ctrl_Untreated_1_5	Ctrl_Untreated_1	1.0	Coenzyme A biosynthesis	True	2.031e+08	6.437	6.437	0.1269
150	N-Me-Gly	Amino acid	1	Ctrl_Untreated_1_5	Ctrl_Untreated_1	1.0	Amino acid derivatives	True	2.066e+06	-0.1819	-0.1819	0.1186
122	3-OH-Isobutyrate	Amino acid	1	Ctrl_Untreated_1_5	Ctrl_Untreated_1	0.857142857142857	Amino acids degradation intermediates	True	2.445e+05	-3.261	-3.261	-0.1051
241	4-Acetamidobutanoate	Amino acid	1	Ctrl_Untreated_1_5	Ctrl_Untreated_1	1.0	Polyamine derivatives	True	3.178e+06	0.439	0.439	-2.03e-03
711	alpha-Tocopherol	Cofactor	1	Ctrl_Untreated_1_5	Ctrl_Untreated_1	1.0	Cofactors	True	4.566e+06	0.962	0.962	0.1817
55	Citrate	Carbon	1	Ctrl_Untreated_1_5	Ctrl_Untreated_1	1.0	TCA cycle	True	8.673e+06	1.887	1.887	-0.06159
387	3-Aminoisobutyrate	Nucleotide	1	Ctrl_Untreated_1_5	Ctrl_Untreated_1	0.857142857142857	Pyrimidine degradation	True	5.456e+05	-2.103	-2.103	-0.08927
338	Guanosine	Nucleotide	1	Ctrl_Untreated_1_5	Ctrl_Untreated_1	1.0	Purine nucleosides	True	1.584e+08	6.079	6.079	0.9725
209	N-Ac-Ala	Amino acid	1	Ctrl_Untreated_1_5	Ctrl_Untreated_1	1.0	N-acetylated amino acids	True	1.292e+06	-0.8597	-0.8597	0.3415
221	N-Ac-Met	Amino acid	1	Ctrl_Untreated_1_5	Ctrl_Untreated_1	1.0	N-acetylated amino acids	True	1.082e+07	2.206	2.206	0.4621
228	N-Ac-Val	Amino acid	1	Ctrl_Untreated_1_5	Ctrl_Untreated_1	0.571428571428571	N-acetylated amino acids	True	7.272e+04	-5.011	-5.011	0.3602
346	Urate	Nucleotide	1	Ctrl_Untreated_1_5	Ctrl_Untreated_1	1.0	Purine degradation	True	1.247e+06	-0.9102	-0.9102	0.1526
90	Arg	Amino acid	1	Ctrl_Untreated_1_5	Ctrl_Untreated_1	1.0	Proteinogenic amino acids	True	2.773e+09	10.21	10.21	0.5024
60	Fumarate	Carbon	1	Ctrl_Untreated_1_5	Ctrl_Untreated_1	1.0	TCA cycle	True	7.332e+06	1.645	1.645	0.63
78	Ser	Amino acid	1	Ctrl_Untreated_1_5	Ctrl_Untreated_1	1.0	Proteinogenic amino acids	True	7.599e+08	8.341	8.341	1.083
83	Val	Amino acid	1	Ctrl_Untreated_1_5	Ctrl_Untreated_1	1.0	Proteinogenic amino acids	True	9.232e+08	8.621	8.621	0.7492
734	Pyridoxal	Cofactor	1	Ctrl_Untreated_1_5	Ctrl_Untreated_1	1.0	PLP biosynthesis and salvage	True	9.885e+06	2.076	2.076	0.2931

Metabolite ID	Name	Super Pathway	Dataset	Sample ID	Group ID	Detection Fraction	Pathway	Detected	Raw Intensity	Log2 Norm Intensity	Norm Imputed	Log2 Ctrl Norm
136	Urea	Amino acid	1	Ctrl_Untreated_1_5	Ctrl_Untreated_1	0.857142857142857	Amino acids degradation intermediates	True	2.984e+06	0.3482	0.3482	0.197
67	Ribose 1-P	Carbon	1	Ctrl_Untreated_1_5	Ctrl_Untreated_1	1.0	Pentose phosphate pathway (PPP)	True	4.654e+06	0.9893	0.9893	0.3681
284	Carnosine	Amino acid	1	Ctrl_Untreated_1_5	Ctrl_Untreated_1	1.0	Dipeptides	True	7.532e+05	-1.638	-1.638	0.4704
306	gamma-Glu-Cys	Amino acid	1	Ctrl_Untreated_1_5	Ctrl_Untreated_1	1.0	Glutathione biosynthesis	True	1.578e+06	-0.5708	-0.5708	-0.234
712	Retinol (Vit A)	Cofactor	1	Ctrl_Untreated_1_5	Ctrl_Untreated_1	1.0	Cofactors	True	4.889e+05	-2.261	-2.261	0.1719
85	Cys	Amino acid	1	Ctrl_Untreated_1_5	Ctrl_Untreated_1	1.0	Proteinogenic amino acids	True	4.571e+07	4.285	4.285	-0.2283
91	Pro	Amino acid	1	Ctrl_Untreated_1_5	Ctrl_Untreated_1	1.0	Proteinogenic amino acids	True	2.019e+09	9.751	9.751	0.3426
308	Glutathione, Reduced	Amino acid	1	Ctrl_Untreated_1_5	Ctrl_Untreated_1	1.0	Glutathione	True	1.891e+08	6.334	6.334	-0.622
107	Citrulline	Amino acid	1	Ctrl_Untreated_1_5	Ctrl_Untreated_1	1.0	Amino acids biosynthesis intermediates	True	1.201e+07	2.357	2.357	-0.4669
328	IMP	Nucleotide	1	Ctrl_Untreated_1_5	Ctrl_Untreated_1	0.285714285714286	Purine nucleotides	False			-4.53	-0.2088
706	FAD	Cofactor	1	Ctrl_Untreated_1_5	Ctrl_Untreated_1	1.0	Cofactors	True	6.463e+05	-1.859	-1.859	0.3308
735	Pyridoxamine	Cofactor	1	Ctrl_Untreated_1_5	Ctrl_Untreated_1	1.0	PLP biosynthesis and salvage	True	2.454e+06	0.06621	0.06621	0.2592
199	Serotonin	Amino acid	1	Ctrl_Untreated_1_5	Ctrl_Untreated_1	1.0	Amino acid derivatives	True	5.823e+06	1.313	1.313	0.03993
370	CMP	Nucleotide	1	Ctrl_Untreated_1_5	Ctrl_Untreated_1	1.0	Pyrimidine nucleotides	True	6.523e+06	1.476	1.476	-0.3625
287	gamma-Glu-Gln	Amino acid	1	Ctrl_Untreated_1_5	Ctrl_Untreated_1	1.0	Gamma-glutamyl dipeptides	True	1.805e+06	-0.3772	-0.3772	-1.218
14	UDP-Glucuronate	Carbon	1	Ctrl_Untreated_1_5	Ctrl_Untreated_1	0.857142857142857	Polysaccharide biosynthesis	False			-4.022	-2.073
229	N-Formyl-Met	Amino acid	1	Ctrl_Untreated_1_5	Ctrl_Untreated_1	1.0	N-formylated amino acids	True	1.476e+05	-3.99	-3.99	-0.1673
350	3',5'-cAMP	Nucleotide	1	Ctrl_Untreated_1_5	Ctrl_Untreated_1	1.0	Purine derivatives in signaling	True	1.977e+05	-3.568	-3.568	0.05155
371	CDP	Nucleotide	1	Ctrl_Untreated_1_5	Ctrl_Untreated_1	0.571428571428571	Pyrimidine nucleotides	False			-5.544	-2.224
372	CTP	Nucleotide	1	Ctrl_Untreated_1_5	Ctrl_Untreated_1	0.571428571428571	Pyrimidine nucleotides	False			-4.713	-1.059
333	GDP	Nucleotide	1	Ctrl_Untreated_1_5	Ctrl_Untreated_1	0.571428571428571	Purine nucleotides	False			-4.484	-1.983
332	GMP	Nucleotide	1	Ctrl_Untreated_1_5	Ctrl_Untreated_1	1.0	Purine nucleotides	True	3.783e+05	-2.631	-2.631	-3.33

Metabolite ID	Name	Super Pathway	Dataset	Sample ID	Group ID	Detection Fraction	Pathway	Detected	Raw Intensity	Log2 Norm Intensity	Norm Imputed	Log2 Ctrl Norm
373	UMP	Nucleotide	1	Ctrl_Untreated_1_5	Ctrl_Untreated_1	1.0	Pyrimidine nucleotides	True	9.371e+04	-4.645	-4.645	-3.59
389	3'-CMP	Nucleotide	1	Ctrl_Untreated_1_5	Ctrl_Untreated_1	1.0	Pyrimidine derivatives in signaling	True	4.508e+05	-2.379	-2.379	-1.327
330	ADP	Nucleotide	1	Ctrl_Untreated_1_5	Ctrl_Untreated_1	0.714285714285714	Purine nucleotides	False			-2.826	-2.53
342	Hypoxanthine	Nucleotide	1	Ctrl_Untreated_1_5	Ctrl_Untreated_1	1.0	Purine bases	True	6.631e+07	4.822	4.822	0.8829
736	Pyridoxamine-P	Cofactor	1	Ctrl_Untreated_1_5	Ctrl_Untreated_1	1.0	PLP biosynthesis and salvage	True	3.385e+05	-2.792	-2.792	0.1863
148	Betaine	Amino acid	1	Ctrl_Untreated_1_5	Ctrl_Untreated_1	1.0	Amino acid derivatives	True	5.59e+07	4.576	4.576	-0.23
344	Xanthine	Nucleotide	1	Ctrl_Untreated_1_5	Ctrl_Untreated_1	1.0	Purine bases	True	1.615e+07	2.784	2.784	1.876
386	3-Ureidopropionate	Nucleotide	1	Ctrl_Untreated_1_5	Ctrl_Untreated_1	1.0	Pyrimidine degradation	True	1.191e+06	-0.9763	-0.9763	-0.05692
149	DiMe-Gly	Amino acid	1	Ctrl_Untreated_1_5	Ctrl_Untreated_1	1.0	Amino acid derivatives	True	1.51e+06	-0.6348	-0.6348	-0.1145
703	NAD+	Cofactor	1	Ctrl_Untreated_1_5	Ctrl_Untreated_1	1.0	Cofactors	True	1.169e+07	2.318	2.318	-0.4186
709	Pyridoxal-P	Cofactor	1	Ctrl_Untreated_1_5	Ctrl_Untreated_1	1.0	Cofactors	True	3.9e+05	-2.587	-2.587	-0.2794
731	Thiamin (Vitamin B1)	Cofactor	1	Ctrl_Untreated_1_5	Ctrl_Untreated_1	1.0	TPP biosynthesis	True	3.383e+06	0.5291	0.5291	-0.2186
374	UDP	Nucleotide	1	Ctrl_Untreated_1_5	Ctrl_Untreated_1	0.714285714285714	Pyrimidine nucleotides	False			-5.339	-3.785
102	2-Aminoadipate	Amino acid	1	Ctrl_Untreated_1_5	Ctrl_Untreated_1	1.0	Amino acids biosynthesis intermediates	True	3.853e+05	-2.605	-2.605	-0.4955
45	Fructose-6-P	Carbon	1	Ctrl_Untreated_1_5	Ctrl_Untreated_1	1.0	Glycolysis, GNG	True	3.399e+06	0.5361	0.5361	0.4608
320	TMP	Nucleotide	1	Ctrl_Untreated_1_5	Ctrl_Untreated_1	0.571428571428571	Deoxy-nucleotides	False			-6.992	-2.2
341	XMP	Nucleotide	1	Ctrl_Untreated_1_5	Ctrl_Untreated_1	0.857142857142857	IMP conversion to AMP & GMP	True	2.551e+05	-3.2	-3.2	1.374
120	beta-OH-Isovalerate	Amino acid	1	Ctrl_Untreated_1_5	Ctrl_Untreated_1	0.857142857142857	Amino acids degradation intermediates	True	2.937e+05	-2.997	-2.997	0.1686
322	2'-dI	Nucleotide	1	Ctrl_Untreated_1_5	Ctrl_Untreated_1	0.857142857142857	Deoxy-nucleosides	True	4.921e+05	-2.252	-2.252	1.873
4	GlcNAc 6-P	Carbon	1	Ctrl_Untreated_1_5	Ctrl_Untreated_1	1.0	Aminosugar biosynthesis	True	9.034e+06	1.946	1.946	1.618
337	Xanthosine	Nucleotide	1	Ctrl_Untreated_1_5	Ctrl_Untreated_1	1.0	Purine nucleosides	True	4.142e+05	-2.501	-2.501	0.5737
188	Kynurenine	Amino acid	1	Ctrl_Untreated_1_5	Ctrl_Untreated_1	1.0	Amino acid derivatives	True	9.711e+05	-1.271	-1.271	-0.5797

Metabolite ID	Name	Super Pathway	Dataset	Sample ID	Group ID	Detection Fraction	Pathway	Detected	Raw Intensity	Log2 Norm Intensity	Norm Imputed	Log2 Ctrl Norm
63	6-P-Gluconate	Carbon	1	Ctrl_Untreated_1_5	Ctrl_Untreated_1	1.0	Pentose phosphate pathway (PPP)	True	3.437e+06	0.5519	0.5519	-1.482
40	Glucuronate	Carbon	1	Ctrl_Untreated_1_5	Ctrl_Untreated_1	1.0	Sugars and sugar alcohols	True	8.097e+05	-1.534	-1.534	5.45e-03
108	Argininosuccinate	Amino acid	1	Ctrl_Untreated_1_5	Ctrl_Untreated_1	1.0	Amino acids biosynthesis intermediates	True	3.799e+06	0.6966	0.6966	0.9228
710	Carnitine	Cofactor	1	Ctrl_Untreated_1_5	Ctrl_Untreated_1	1.0	Cofactors	True	1.093e+08	5.543	5.543	-0.4343
725	P-Pantetheine	Cofactor	1	Ctrl_Untreated_1_5	Ctrl_Untreated_1	1.0	Coenzyme A biosynthesis	True	4.89e+05	-2.261	-2.261	1.443
48	DHAP	Carbon	1	Ctrl_Untreated_1_5	Ctrl_Untreated_1	1.0	Glycolysis, GNG	True	2.375e+07	3.341	3.341	0.2443
17	Maltose	Carbon	1	Ctrl_Untreated_1_5	Ctrl_Untreated_1	1.0	Glycogen degradation	True	1.22e+06	-0.9425	-0.9425	0.3503
359	N1-Me-Adenosine	Nucleotide	1	Ctrl_Untreated_1_5	Ctrl_Untreated_1	0.857142857142857	Purine derivatives in RNAs	True	1.818e+06	-0.3664	-0.3664	0.6713
159	3-Me-His	Amino acid	1	Ctrl_Untreated_1_5	Ctrl_Untreated_1	1.0	Amino acid derivatives	True	8.859e+04	-4.726	-4.726	-0.4903
155	4-Guanidinobutanoate	Amino acid	1	Ctrl_Untreated_1_5	Ctrl_Untreated_1	1.0	Amino acid derivatives	True	7.789e+05	-1.59	-1.59	-0.1556
164	5-OH-Lys	Amino acid	1	Ctrl_Untreated_1_5	Ctrl_Untreated_1	1.0	Amino acid derivatives	True	5.572e+05	-2.073	-2.073	-0.3025
357	Adenosine-3',5'-PP	Nucleotide	1	Ctrl_Untreated_1_5	Ctrl_Untreated_1	0.857142857142857	Purine byproducts of metabolic processes	True	5.079e+05	-2.206	-2.206	1.271
104	Cystathionine	Amino acid	1	Ctrl_Untreated_1_5	Ctrl_Untreated_1	1.0	Amino acids biosynthesis intermediates	True	5.853e+06	1.32	1.32	-0.8892
113	Imidazole Lactate	Amino acid	1	Ctrl_Untreated_1_5	Ctrl_Untreated_1	0.857142857142857	Amino acids degradation intermediates	True	1.163e+05	-4.334	-4.334	-0.6571
215	N-Ac-Glu	Amino acid	1	Ctrl_Untreated_1_5	Ctrl_Untreated_1	1.0	N-acetylated amino acids	True	6.488e+06	1.469	1.469	-0.2747
310	S-Lactoyl-Glutathione	Amino acid	1	Ctrl_Untreated_1_5	Ctrl_Untreated_1	0.857142857142857	Glutathione derivatives	True	7.344e+06	1.647	1.647	1.291
5	GlcNAc 1-P	Carbon	1	Ctrl_Untreated_1_5	Ctrl_Untreated_1	1.0	Aminosugar biosynthesis	True	1.454e+06	-0.6895	-0.6895	1.221
34	Ribitol	Carbon	1	Ctrl_Untreated_1_5	Ctrl_Untreated_1	1.0	Sugars and sugar alcohols	True	2.92e+05	-3.005	-3.005	0.4249
10	UDP-Galactose	Carbon	1	Ctrl_Untreated_1_5	Ctrl_Untreated_1	0.857142857142857	Polysaccharide biosynthesis	True	9.289e+05	-1.335	-1.335	-0.7089
13	Guanosine 5'-PP-Fucose	Carbon	1	Ctrl_Untreated_1_5	Ctrl_Untreated_1	1.0	Polysaccharide biosynthesis	True	2.844e+05	-3.043	-3.043	-0.7437

Metabolite ID	Name	Super Pathway	Dataset	Sample ID	Group ID	Detection Fraction	Pathway	Detected	Raw Intensity	Log2 Norm Intensity	Norm Imputed	Log2 Ctrl Norm
19	Maltotetraose	Carbon	1	Ctrl_Untreated_1_5	Ctrl_Untreated_1	1.0	Glycogen degradation	True	6.535e+06	1.479	1.479	1.523
233	SAM	Amino acid	1	Ctrl_Untreated_1_5	Ctrl_Untreated_1	1.0	SAM metabolism	True	1.312e+06	-0.8377	-0.8377	-0.6814
129	5-Aminovalerate	Amino acid	1	Ctrl_Untreated_1_5	Ctrl_Untreated_1	1.0	Amino acids degradation intermediates	True	2.669e+06	0.1872	0.1872	-0.8144
741	5-Me-THF	Cofactor	1	Ctrl_Untreated_1_5	Ctrl_Untreated_1	0.714285714285714	Folate metabolism	True	8.587e+04	-4.771	-4.771	-0.01283
198	Indolelactate	Amino acid	1	Ctrl_Untreated_1_5	Ctrl_Untreated_1	0.857142857142857	Amino acid derivatives	True	1.785e+05	-3.715	-3.715	0.07236
254	Gly-Val	Amino acid	1	Ctrl_Untreated_1_5	Ctrl_Untreated_1	1.0	Dipeptides	True	6.245e+06	1.414	1.414	0.3023
291	gamma-Glu-Leu	Amino acid	1	Ctrl_Untreated_1_5	Ctrl_Untreated_1	1.0	Gamma-glutamyl dipeptides	True	1.614e+06	-0.5388	-0.5388	-0.05005
173	Met Sulfoxide	Amino acid	1	Ctrl_Untreated_1_5	Ctrl_Untreated_1	1.0	Amino acid derivatives	True	2.036e+07	3.119	3.119	0.5512
43	Glucose	Carbon	1	Ctrl_Untreated_1_5	Ctrl_Untreated_1	1.0	Glycolysis, GNG	True	7.771e+07	5.051	5.051	-5.53e-04
185	Phenyllactate	Amino acid	1	Ctrl_Untreated_1_5	Ctrl_Untreated_1	0.285714285714286	Amino acid derivatives	False			-5.917	-0.1051
156	Homo-Arg	Amino acid	1	Ctrl_Untreated_1_5	Ctrl_Untreated_1	1.0	Amino acid derivatives	True	4.356e+06	0.894	0.894	-0.2865
135	Homocitrulline	Amino acid	1	Ctrl_Untreated_1_5	Ctrl_Untreated_1	1.0	Amino acids degradation intermediates	True	5.857e+05	-2.001	-2.001	-0.2983
719	Nicotinamide MN	Cofactor	1	Ctrl_Untreated_1_5	Ctrl_Untreated_1	1.0	NAD biosynthesis	True	1.23e+07	2.391	2.391	2.206
212	N-Ac-Asp	Amino acid	1	Ctrl_Untreated_1_5	Ctrl_Untreated_1	1.0	N-acetylated amino acids	True	7.712e+06	1.718	1.718	1.021
720	1-Me-Nicotinamide	Cofactor	1	Ctrl_Untreated_1_5	Ctrl_Untreated_1	1.0	Derivatives of NA, nicotinamide and NAD	True	4.941e+08	7.72	7.72	-0.1709
216	N-Ac-Gly	Amino acid	1	Ctrl_Untreated_1_5	Ctrl_Untreated_1	0.714285714285714	N-acetylated amino acids	True	1.451e+05	-4.013	-4.013	-0.4736
70	Creatine	Carbon	1	Ctrl_Untreated_1_5	Ctrl_Untreated_1	1.0	Creatine energy storage	True	1.203e+09	9.003	9.003	-0.4141
26	Galactonate	Carbon	1	Ctrl_Untreated_1_5	Ctrl_Untreated_1	0.857142857142857	Sugars and sugar alcohols	False			-2.211	-1.406
309	Glutathione, Oxidized	Amino acid	1	Ctrl_Untreated_1_5	Ctrl_Untreated_1	1.0	Glutathione	True	5.431e+06	1.212	1.212	0.01956
35	Ribonate	Carbon	1	Ctrl_Untreated_1_5	Ctrl_Untreated_1	1.0	Sugars and sugar alcohols	True	1.555e+06	-0.5922	-0.5922	-0.06499
160	1-Me-His	Amino acid	1	Ctrl_Untreated_1_5	Ctrl_Untreated_1	1.0	Amino acid derivatives	True	2.05e+07	3.128	3.128	-0.3129

Metabolite ID	Name	Super Pathway	Dataset	Sample ID	Group ID	Detection Fraction	Pathway	Detected	Raw Intensity	Log2 Norm Intensity	Norm Imputed	Log2 Ctrl Norm
44	Glucose 6-P	Carbon	1	Ctrl_Untreated_1_5	Ctrl_Untreated_1	0.857142857142857	Glycolysis, GNG	True	5.131e+05	-2.192	-2.192	-0.715
704	NADH	Cofactor	1	Ctrl_Untreated_1_5	Ctrl_Untreated_1	1.0	Cofactors	True	8.173e+05	-1.52	-1.52	-0.8741
275	Thr-Phe	Amino acid	1	Ctrl_Untreated_1_5	Ctrl_Untreated_1	0.857142857142857	Dipeptides	False			-3.909	-2.255
738	Pyridoxate	Cofactor	1	Ctrl_Untreated_1_5	Ctrl_Untreated_1	1.0	PLP biosynthesis and salvage	True	1.895e+05	-3.629	-3.629	-0.2643
177	3-(4-OH-Phenyl)Lactate	Amino acid	1	Ctrl_Untreated_1_5	Ctrl_Untreated_1	1.0	Amino acid derivatives	True	4.26e+05	-2.46	-2.46	5.04e-03
206	Trans-4-OH-Pro	Amino acid	1	Ctrl_Untreated_1_5	Ctrl_Untreated_1	1.0	Amino acid derivatives	True	6.202e+07	4.726	4.726	-0.4297
329	AMP	Nucleotide	1	Ctrl_Untreated_1_5	Ctrl_Untreated_1	1.0	Purine nucleotides	True	4.816e+06	1.039	1.039	-2.596
11	UDP-Glucose	Carbon	1	Ctrl_Untreated_1_5	Ctrl_Untreated_1	1.0	Polysaccharide biosynthesis	True	1.609e+05	-3.865	-3.865	-3.76
158	4-Imidazole-Ac	Amino acid	1	Ctrl_Untreated_1_5	Ctrl_Untreated_1	1.0	Amino acid derivatives	True	3.051e+05	-2.942	-2.942	-0.7949
111	1-Me-Imidazole-Ac	Amino acid	1	Ctrl_Untreated_1_5	Ctrl_Untreated_1	1.0	Amino acids degradation intermediates	True	5.755e+05	-2.026	-2.026	-0.09235
345	Guanine	Nucleotide	1	Ctrl_Untreated_1_5	Ctrl_Untreated_1	1.0	Purine bases	True	1.642e+08	6.13	6.13	1.144
22	N-Ac-Neuraminate	Carbon	1	Ctrl_Untreated_1_5	Ctrl_Untreated_1	1.0	Aminosugar derivatives	True	2.574e+06	0.1351	0.1351	0.2705
721	N'-Methylnicotinate	Cofactor	1	Ctrl_Untreated_1_5	Ctrl_Untreated_1	1.0	Derivatives of NA, nicotinamide and NAD	True	9.041e+05	-1.374	-1.374	-0.3668
183	Phenol Sulfate	Amino acid	1	Ctrl_Untreated_1_5	Ctrl_Untreated_1	0.857142857142857	Amino acid derivatives	True	6.147e+04	-5.253	-5.253	-0.6129
718	Nicotinamide Riboside	Cofactor	1	Ctrl_Untreated_1_5	Ctrl_Untreated_1	1.0	NAD biosynthesis	True	2.049e+08	6.45	6.45	4.86
297	gamma-Glu-Thr	Amino acid	1	Ctrl_Untreated_1_5	Ctrl_Untreated_1	1.0	Gamma-glutamyl dipeptides	True	3.04e+06	0.3748	0.3748	-0.2654
295	gamma-Glu-Phe	Amino acid	1	Ctrl_Untreated_1_5	Ctrl_Untreated_1	0.428571428571429	Gamma-glutamyl dipeptides	False			-5.975	-0.1899
347	Allantoic Acid	Nucleotide	1	Ctrl_Untreated_1_5	Ctrl_Untreated_1	0.571428571428571	Purine degradation	False			-6.474	-0.8345
399	Pseudouridine	Nucleotide	1	Ctrl_Untreated_1_5	Ctrl_Untreated_1	1.0	Pyrimidine derivatives in RNAs	True	6.858e+05	-1.773	-1.773	0.2569
375	UTP	Nucleotide	1	Ctrl_Untreated_1_5	Ctrl_Untreated_1	0.571428571428571	Pyrimidine nucleotides	False			-5.025	-3.049
144	Glu, gamma-Me Ester	Amino acid	1	Ctrl_Untreated_1_5	Ctrl_Untreated_1	1.0	Amino acid derivatives	True	2.203e+06	-0.0896	-0.0896	-0.6674

Metabolite ID	Name	Super Pathway	Dataset	Sample ID	Group ID	Detection Fraction	Pathway	Detected	Raw Intensity	Log2 Norm Intensity	Norm Imputed	Log2 Ctrl Norm
292	gamma-Glu-epsilon-Lysine	Amino acid	1	Ctrl_Untreated_1_5	Ctrl_Untreated_1	1.0	Gamma-glutamyl dipeptides	True	2.85e+06	0.2818	0.2818	0.4731
225	N-Ac-Thr	Amino acid	1	Ctrl_Untreated_1_5	Ctrl_Untreated_1	1.0	N-acetylated amino acids	True	6.411e+05	-1.87	-1.87	-0.3677
211	N-Ac-Asn	Amino acid	1	Ctrl_Untreated_1_5	Ctrl_Untreated_1	1.0	N-acetylated amino acids	True	2.826e+05	-3.052	-3.052	-0.367
151	Phenylacetylglycine	Amino acid	1	Ctrl_Untreated_1_5	Ctrl_Untreated_1	1.0	Amino acid derivatives	True	1.123e+06	-1.061	-1.061	-0.2745
217	N-Ac-His	Amino acid	1	Ctrl_Untreated_1_5	Ctrl_Untreated_1	1.0	N-acetylated amino acids	True	1.673e+05	-3.808	-3.808	-0.3233
288	gamma-Glu-Gly	Amino acid	1	Ctrl_Untreated_1_5	Ctrl_Untreated_1	0.571428571428571	Gamma-glutamyl dipeptides	False			-3.092	-1.268
222	N-Ac-Phe	Amino acid	1	Ctrl_Untreated_1_5	Ctrl_Untreated_1	0.571428571428571	N-acetylated amino acids	False			-6.698	-0.4255
71	Creatine-P	Carbon	1	Ctrl_Untreated_1_5	Ctrl_Untreated_1	1.0	Creatine energy storage	True	8.364e+04	-4.809	-4.809	-0.5534
210	N-Ac-Arg	Amino acid	1	Ctrl_Untreated_1_5	Ctrl_Untreated_1	1.0	N-acetylated amino acids	True	4.413e+05	-2.409	-2.409	0.2664
218	N-Ac-Ile	Amino acid	1	Ctrl_Untreated_1_5	Ctrl_Untreated_1	0.428571428571429	N-acetylated amino acids	False			-6.52	-0.5541
251	Gly-Leu	Amino acid	1	Ctrl_Untreated_1_5	Ctrl_Untreated_1	1.0	Dipeptides	True	7.07e+06	1.593	1.593	-0.04607
290	gamma-Glu-Ile	Amino acid	1	Ctrl_Untreated_1_5	Ctrl_Untreated_1	1.0	Gamma-glutamyl dipeptides	True	9.077e+05	-1.369	-1.369	-0.03017
316	Ophthalmate	Amino acid	1	Ctrl_Untreated_1_5	Ctrl_Untreated_1	1.0	Oxidative stress markers	True	1.257e+07	2.423	2.423	0.07992
125	Isovaleryl-Gly	Amino acid	1	Ctrl_Untreated_1_5	Ctrl_Untreated_1	0.857142857142857	Amino acids degradation intermediates	True	4.029e+04	-5.862	-5.862	-0.4529
368	7-Me-Guanine	Nucleotide	1	Ctrl_Untreated_1_5	Ctrl_Untreated_1	1.0	Purine derivatives in RNAs	True	7.001e+05	-1.743	-1.743	0.4466
208	Pro-OH-Pro	Amino acid	1	Ctrl_Untreated_1_5	Ctrl_Untreated_1	1.0	Amino acid derivatives	True	2.278e+07	3.281	3.281	-0.04878
366	N2,N2-DiMe-Guanosine	Nucleotide	1	Ctrl_Untreated_1_5	Ctrl_Untreated_1	0.857142857142857	Purine derivatives in RNAs	True	1.886e+05	-3.635	-3.635	0.4783
352	3'-AMP	Nucleotide	1	Ctrl_Untreated_1_5	Ctrl_Untreated_1	1.0	Purine derivatives in signaling	True	1.112e+06	-1.076	-1.076	0.4112
363	N6-Carbamoyl-Thr-Adenosine	Nucleotide	1	Ctrl_Untreated_1_5	Ctrl_Untreated_1	0.857142857142857	Purine derivatives in RNAs	True	1.171e+05	-4.324	-4.324	0.3618
314	Cys-Glutathione Disulfide	Amino acid	1	Ctrl_Untreated_1_5	Ctrl_Untreated_1	1.0	Oxidative stress markers	True	1.591e+05	-3.881	-3.881	-0.5965
382	Orotidine	Nucleotide	1	Ctrl_Untreated_1_5	Ctrl_Untreated_1	0.428571428571429	Pyrimidine (UMP) biosynthesis	False			-5.287	-0.9952

Metabolite ID	Name	Super Pathway	Dataset	Sample ID	Group ID	Detection Fraction	Pathway	Detected	Raw Intensity	Log2 Norm Intensity	Norm Imputed	Log2 Ctrl Norm
307	Cys-Gly	Amino acid	1	Ctrl_Untreated_1_5	Ctrl_Untreated_1	1.0	Glutathione biosynthesis	True	2.251e+06	-0.05847	-0.05847	-1.665
64	Sedoheptulose-7-P	Carbon	1	Ctrl_Untreated_1_5	Ctrl_Untreated_1	1.0	Pentose phosphate pathway (PPP)	True	3.901e+06	0.7349	0.7349	0.5808
142	N-Ac-Asp-Glu	Amino acid	1	Ctrl_Untreated_1_5	Ctrl_Untreated_1	1.0	Amino acid derivatives	True	1.052e+06	-1.155	-1.155	-9.94e-03
708	Thiamin-PP	Cofactor	1	Ctrl_Untreated_1_5	Ctrl_Untreated_1	0.571428571428571	Cofactors	True	2.971e+04	-6.302	-6.302	-0.3181
182	P-Cresol Sulfate	Amino acid	1	Ctrl_Untreated_1_5	Ctrl_Untreated_1	0.857142857142857	Amino acid derivatives	True	2.604e+05	-3.17	-3.17	0.02597
250	Gly-Ile	Amino acid	1	Ctrl_Untreated_1_5	Ctrl_Untreated_1	1.0	Dipeptides	True	6.811e+05	-1.783	-1.783	-0.466
286	gamma-Glu-Glu	Amino acid	1	Ctrl_Untreated_1_5	Ctrl_Untreated_1	1.0	Gamma-glutamyl dipeptides	True	1.662e+06	-0.4965	-0.4965	-0.5143
739	Deoxycarnitine	Cofactor	1	Ctrl_Untreated_1_5	Ctrl_Untreated_1	1.0	Carnitine biosynthesis	True	2.958e+07	3.657	3.657	-0.09378
203	DiMe-Arg	Amino acid	1	Ctrl_Untreated_1_5	Ctrl_Untreated_1	1.0	Amino acid derivatives	True	3.158e+08	7.074	7.074	1.441
351	2'-AMP	Nucleotide	1	Ctrl_Untreated_1_5	Ctrl_Untreated_1	1.0	Purine derivatives in signaling	True	2.894e+06	0.3042	0.3042	2.573
8	Cytidine 5'-P-N-Ac-Neuraminate	Carbon	1	Ctrl_Untreated_1_5	Ctrl_Untreated_1	1.0	Aminosugar biosynthesis	True	9.998e+05	-1.229	-1.229	0.5161
285	gamma-Glu-Ala	Amino acid	1	Ctrl_Untreated_1_5	Ctrl_Untreated_1	0.857142857142857	Gamma-glutamyl dipeptides	False			-6.022	-2.783
224	N-Ac-Ser	Amino acid	1	Ctrl_Untreated_1_5	Ctrl_Untreated_1	1.0	N-acetylated amino acids	True	1.219e+07	2.379	2.379	0.4993
244	Ala-Leu	Amino acid	1	Ctrl_Untreated_1_5	Ctrl_Untreated_1	1.0	Dipeptides	True	1.448e+06	-0.6953	-0.6953	-1.833
207	N-Me-Pro	Amino acid	1	Ctrl_Untreated_1_5	Ctrl_Untreated_1	1.0	Amino acid derivatives	True	1.12e+06	-1.066	-1.066	-0.1362
171	Cys Sulfinic Acid	Amino acid	1	Ctrl_Untreated_1_5	Ctrl_Untreated_1	0.857142857142857	Amino acid derivatives	True	3.27e+05	-2.842	-2.842	0.4557
181	O-Me-Tyr	Amino acid	1	Ctrl_Untreated_1_5	Ctrl_Untreated_1	0.857142857142857	Amino acid derivatives	True	1.678e+05	-3.804	-3.804	-0.4344
240	N-Ac-Putrescine	Amino acid	1	Ctrl_Untreated_1_5	Ctrl_Untreated_1	1.0	Polyamine derivatives	True	3.229e+05	-2.86	-2.86	-0.4962
176	S-Me-Met	Amino acid	1	Ctrl_Untreated_1_5	Ctrl_Untreated_1	0.857142857142857	Amino acid derivatives	True	5.001e+05	-2.229	-2.229	-0.387
339	AICAR	Nucleotide	1	Ctrl_Untreated_1_5	Ctrl_Untreated_1	0.571428571428571	IMP biosynthesis	False			-4.716	-0.4151
141	gamma-Carboxy-Glu	Amino acid	1	Ctrl_Untreated_1_5	Ctrl_Untreated_1	1.0	Amino acid derivatives	True	1.002e+06	-1.226	-1.226	-0.4407

Metabolite ID	Name	Super Pathway	Dataset	Sample ID	Group ID	Detection Fraction	Pathway	Detected	Raw Intensity	Log2 Norm Intensity	Norm Imputed	Log2 Ctrl Norm
392	3'-UMP	Nucleotide	1	Ctrl_Untreated_1_5	Ctrl_Untreated_1	0.571428571428571	Pyrimidine derivatives in signaling	False			-5.207	-1.174
355	3'-GMP	Nucleotide	1	Ctrl_Untreated_1_5	Ctrl_Untreated_1	0.857142857142857	Purine derivatives in signaling	False			-5.834	-1.897
282	Val-Leu	Amino acid	1	Ctrl_Untreated_1_5	Ctrl_Untreated_1	1.0	Dipeptides	True	1.217e+06	-0.9451	-0.9451	-1.917
140	Carboxyethyl-GABA	Amino acid	1	Ctrl_Untreated_1_5	Ctrl_Untreated_1	1.0	Amino acid derivatives	True	1.082e+06	-1.116	-1.116	0.3153
258	Ile-Gly	Amino acid	1	Ctrl_Untreated_1_5	Ctrl_Untreated_1	1.0	Dipeptides	True	2.221e+07	3.244	3.244	-0.06818
260	Leu-Ala	Amino acid	1	Ctrl_Untreated_1_5	Ctrl_Untreated_1	1.0	Dipeptides	True	1.804e+06	-0.378	-0.378	-1.679
265	Lys-Leu	Amino acid	1	Ctrl_Untreated_1_5	Ctrl_Untreated_1	0.857142857142857	Dipeptides	True	1.666e+05	-3.814	-3.814	-1.203
263	Leu-Gly	Amino acid	1	Ctrl_Untreated_1_5	Ctrl_Untreated_1	1.0	Dipeptides	True	3.103e+06	0.4046	0.4046	-2.617
281	Val-Gly	Amino acid	1	Ctrl_Untreated_1_5	Ctrl_Untreated_1	1.0	Dipeptides	True	4.133e+07	4.14	4.14	-0.3791
270	Pro-Gly	Amino acid	1	Ctrl_Untreated_1_5	Ctrl_Untreated_1	1.0	Dipeptides	True	4.386e+06	0.9039	0.9039	0.1572
114	Imidazole Propionate	Amino acid	1	Ctrl_Untreated_1_5	Ctrl_Untreated_1	1.0	Amino acids degradation intermediates	True	3.052e+05	-2.941	-2.941	-0.6306
267	Phe-Gly	Amino acid	1	Ctrl_Untreated_1_5	Ctrl_Untreated_1	1.0	Dipeptides	True	4.331e+07	4.208	4.208	0.1139
266	Phe-Ala	Amino acid	1	Ctrl_Untreated_1_5	Ctrl_Untreated_1	1.0	Dipeptides	True	2.394e+06	0.03053	0.03053	-1.046
278	Tyr-Gly	Amino acid	1	Ctrl_Untreated_1_5	Ctrl_Untreated_1	1.0	Dipeptides	True	1.061e+07	2.178	2.178	-3.97e-03
255	His-Ala	Amino acid	1	Ctrl_Untreated_1_5	Ctrl_Untreated_1	1.0	Dipeptides	True	9.846e+05	-1.251	-1.251	0.08593
280	Val-Gln	Amino acid	1	Ctrl_Untreated_1_5	Ctrl_Untreated_1	1.0	Dipeptides	True	2.295e+06	-0.03053	-0.03053	-1.649
143	S-1-Pyrroline-5-Carboxylate	Amino acid	1	Ctrl_Untreated_1_5	Ctrl_Untreated_1	0.857142857142857	Amino acid derivatives	True	2.548e+05	-3.202	-3.202	-1.133
232	SAH	Amino acid	1	Ctrl_Untreated_1_5	Ctrl_Untreated_1	1.0	SAM metabolism	True	1.976e+06	-0.2464	-0.2464	0.239
20	Erythronate	Carbon	1	Ctrl_Untreated_1_5	Ctrl_Untreated_1	1.0	Aminosugar derivatives	True	3.201e+07	3.771	3.771	0.03005
248	Gln-Leu	Amino acid	1	Ctrl_Untreated_1_5	Ctrl_Untreated_1	1.0	Dipeptides	True	3.065e+05	-2.935	-2.935	-2.03
276	Trp-Gly	Amino acid	1	Ctrl_Untreated_1_5	Ctrl_Untreated_1	1.0	Dipeptides	True	1.17e+06	-1.003	-1.003	0.07523
205	N-delta-Ac-Ornithine	Amino acid	1	Ctrl_Untreated_1_5	Ctrl_Untreated_1	1.0	Amino acid derivatives	True	1.069e+06	-1.132	-1.132	-0.01125
163	Formimino-Glu	Amino acid	1	Ctrl_Untreated_1_5	Ctrl_Untreated_1	1.0	Amino acid derivatives	True	9.047e+05	-1.373	-1.373	-0.1337
204	N-Me-Arg	Amino acid	1	Ctrl_Untreated_1_5	Ctrl_Untreated_1	1.0	Amino acid derivatives	True	3.651e+07	3.961	3.961	1.374
242	Guanidino-Ac	Amino acid	1	Ctrl_Untreated_1_5	Ctrl_Untreated_1	0.857142857142857	Creatine biosynthesis	True	1.911e+05	-3.617	-3.617	0.2634

Metabolite ID	Name	Super Pathway	Dataset	Sample ID	Group ID	Detection Fraction	Pathway	Detected	Raw Intensity	Log2 Norm Intensity	Norm Imputed	Log2 Ctrl Norm
300	gamma-Glu-Val	Amino acid	1	Ctrl_Untreated_1_5	Ctrl_Untreated_1	0.857142857142857	Gamma-glutamyl dipeptides	True	3.094e+06	0.4002	0.4002	-0.2205
53	Ac-CoA	Carbon	1	Ctrl_Untreated_1_5	Ctrl_Untreated_1	0.285714285714286	Acetyl-CoA	False			-7.274	-0.5114
18	Maltotriose	Carbon	1	Ctrl_Untreated_1_5	Ctrl_Untreated_1	1.0	Glycogen degradation	True	3.991e+06	0.7676	0.7676	0.9254
294	gamma-Glu-Met	Amino acid	1	Ctrl_Untreated_1_5	Ctrl_Untreated_1	0.714285714285714	Gamma-glutamyl dipeptides	True	1.252e+05	-4.227	-4.227	-0.745
174	Met Sulfone	Amino acid	1	Ctrl_Untreated_1_5	Ctrl_Untreated_1	1.0	Amino acid derivatives	True	1.715e+05	-3.773	-3.773	-0.6941
175	N-Ac-Met Sulfoxide	Amino acid	1	Ctrl_Untreated_1_5	Ctrl_Untreated_1	1.0	Amino acid derivatives	True	3.736e+06	0.6723	0.6723	0.696
25	Mannitol/Sorbitol	Carbon	1	Ctrl_Untreated_1_5	Ctrl_Untreated_1	1.0	Sugars and sugar alcohols	True	1.764e+07	2.911	2.911	0.6868
6	UDP-GlcNAc	Carbon	1	Ctrl_Untreated_1_5	Ctrl_Untreated_1	0.428571428571429	Aminosugar biosynthesis	False			-3.589	-0.4814
145	Pyro-Gln	Amino acid	1	Ctrl_Untreated_1_5	Ctrl_Untreated_1	1.0	Amino acid derivatives	True	3.236e+06	0.4652	0.4652	-0.3746
705	Coenzyme A	Cofactor	1	Ctrl_Untreated_1_5	Ctrl_Untreated_1	0.714285714285714	Cofactors	False			-4.967	-1.008
319	2'-dAMP	Nucleotide	1	Ctrl_Untreated_1_5	Ctrl_Untreated_1	0.571428571428571	Deoxy-nucleotides	False			-4.337	-0.6568
119	alpha-OH-Isovalerate	Amino acid	1	Ctrl_Untreated_1_5	Ctrl_Untreated_1	0.714285714285714	Amino acids degradation intermediates	True	3.536e+05	-2.729	-2.729	0.5695
46	Fructose 1,6-PP / Glucose 1,6-PP / Inositol-1,4-PP / Inositol-1,3-PP	Carbon	1	Ctrl_Untreated_1_5	Ctrl_Untreated_1	1.0	Glycolysis, GNG	True	6.906e+07	4.881	4.881	0.04969
137	1-Me-Guanidine	Amino acid	1	Ctrl_Untreated_1_5	Ctrl_Untreated_1	0.857142857142857	Amino acids degradation intermediates	True	7.084e+04	-5.048	-5.048	0.1043
23	N-GlcNAc-Asn	Carbon	1	Ctrl_Untreated_1_5	Ctrl_Untreated_1	1.0	Aminosugar derivatives	True	4.013e+06	0.7758	0.7758	0.06285
262	Leu-Gln	Amino acid	1	Ctrl_Untreated_1_5	Ctrl_Untreated_1	1.0	Dipeptides	True	1.849e+06	-0.3421	-0.3421	-1.638
24	Fructose	Carbon	1	Ctrl_Untreated_1_5	Ctrl_Untreated_1	1.0	Sugars and sugar alcohols	True	1.309e+07	2.481	2.481	0.5323
197	C-Glycosyl-Trp	Amino acid	1	Ctrl_Untreated_1_5	Ctrl_Untreated_1	1.0	Amino acid derivatives	True	2.639e+06	0.1709	0.1709	0.47
33	Arabitol/Xylitol	Carbon	1	Ctrl_Untreated_1_5	Ctrl_Untreated_1	1.0	Sugars and sugar alcohols	True	4.679e+05	-2.325	-2.325	-0.4649

Metabolite ID	Name	Super Pathway	Dataset	Sample ID	Group ID	Detection Fraction	Pathway	Detected	Raw Intensity	Log2 Norm Intensity	Norm Imputed	Log2 Ctrl Norm
128	N2-Ac-Lys/N6-Ac-Lys	Amino acid	1	Ctrl_Untreated_1_5	Ctrl_Untreated_1	1.0	Amino acids degradation intermediates	True	9.068e+06	1.952	1.952	0.7415
42	2-Me-Citrate	Carbon	1	Ctrl_Untreated_1_5	Ctrl_Untreated_1	0.857142857142857	Propionate metabolism	True	8.796e+04	-4.736	-4.736	-0.353
12	Glucuronate 1-P	Carbon	1	Ctrl_Untreated_1_5	Ctrl_Untreated_1	1.0	Polysaccharide biosynthesis	True	1.053e+06	-1.154	-1.154	0.08843
76	Gln	Amino acid	1	Ctrl_Untreated_1_6	Ctrl_Untreated_1	1.0	Proteinogenic amino acids	True	7.634e+08	8.319	8.319	-0.3621
89	Trp	Amino acid	1	Ctrl_Untreated_1_6	Ctrl_Untreated_1	1.0	Proteinogenic amino acids	True	7.552e+07	4.981	4.981	-0.6949
723	beta-Ala	Cofactor	1	Ctrl_Untreated_1_6	Ctrl_Untreated_1	1.0	Coenzyme A biosynthesis	True	1.683e+07	2.815	2.815	-0.1941
75	Glu	Amino acid	1	Ctrl_Untreated_1_6	Ctrl_Untreated_1	1.0	Proteinogenic amino acids	True	3.877e+09	10.66	10.66	-0.07366
77	Gly	Amino acid	1	Ctrl_Untreated_1_6	Ctrl_Untreated_1	1.0	Proteinogenic amino acids	True	5.694e+07	4.573	4.573	-1.042
80	His	Amino acid	1	Ctrl_Untreated_1_6	Ctrl_Untreated_1	1.0	Proteinogenic amino acids	True	1.827e+07	2.933	2.933	-0.4819
82	Leu	Amino acid	1	Ctrl_Untreated_1_6	Ctrl_Untreated_1	1.0	Proteinogenic amino acids	True	8.588e+08	8.488	8.488	-0.7329
87	Phe	Amino acid	1	Ctrl_Untreated_1_6	Ctrl_Untreated_1	1.0	Proteinogenic amino acids	True	6.605e+08	8.11	8.11	-0.7019
130	Glutarate	Amino acid	1	Ctrl_Untreated_1_6	Ctrl_Untreated_1	1.0	Amino acids degradation intermediates	True	4.082e+05	-2.55	-2.55	-0.1237
196	5-OH-Indole-Ac	Amino acid	1	Ctrl_Untreated_1_6	Ctrl_Untreated_1	0.857142857142857	Amino acid derivatives	True	1.098e+05	-4.445	-4.445	-0.4333
74	Asp	Amino acid	1	Ctrl_Untreated_1_6	Ctrl_Untreated_1	1.0	Proteinogenic amino acids	True	4.709e+08	7.622	7.622	-0.2702
236	Spermidine	Amino acid	1	Ctrl_Untreated_1_6	Ctrl_Untreated_1	1.0	Polyamines	True	2.478e+07	3.373	3.373	-0.4883
73	Asn	Amino acid	1	Ctrl_Untreated_1_6	Ctrl_Untreated_1	1.0	Proteinogenic amino acids	True	1.752e+08	6.195	6.195	-0.4763
243	Creatinine	Amino acid	1	Ctrl_Untreated_1_6	Ctrl_Untreated_1	1.0	Creatine degradation	True	8.348e+07	5.126	5.126	0.1233
376	Cytidine	Nucleotide	1	Ctrl_Untreated_1_6	Ctrl_Untreated_1	0.714285714285714	Pyrimidine nucleosides	True	3.117e+06	0.3822	0.3822	-0.3455
41	Lactate	Carbon	1	Ctrl_Untreated_1_6	Ctrl_Untreated_1	1.0	Respiratory carbon sources	True	2.055e+08	6.425	6.425	-0.05649
58	alpha-Ketoglutarate	Carbon	1	Ctrl_Untreated_1_6	Ctrl_Untreated_1	1.0	TCA cycle	True	1.522e+06	-0.6518	-0.6518	-0.6685
69	3-OH-Butyrate	Carbon	1	Ctrl_Untreated_1_6	Ctrl_Untreated_1	0.571428571428571	Ketone bodies	False			-3.497	-0.5127

Metabolite ID	Name	Super Pathway	Dataset	Sample ID	Group ID	Detection Fraction	Pathway	Detected	Raw Intensity	Log2 Norm Intensity	Norm Imputed	Log2 Ctrl Norm
343	Adenine	Nucleotide	1	Ctrl_Untreated_1_6	Ctrl_Untreated_1	1.0	Purine bases	True	6.037e+06	1.336	1.336	-0.121
336	Adenosine	Nucleotide	1	Ctrl_Untreated_1_6	Ctrl_Untreated_1	1.0	Purine nucleosides	True	1.982e+07	3.051	3.051	-0.3714
722	ADP-Ribose	Cofactor	1	Ctrl_Untreated_1_6	Ctrl_Untreated_1	1.0	Derivatives of NA, nicotinamide and NAD	True	4.116e+05	-2.539	-2.539	0.4634
383	Cytosine	Nucleotide	1	Ctrl_Untreated_1_6	Ctrl_Untreated_1	0.571428571428571	Pyrimidine bases	False			-5.32	-0.4652
3	Glucosamine 6-P	Carbon	1	Ctrl_Untreated_1_6	Ctrl_Untreated_1	1.0	Aminosugar biosynthesis	True	1.698e+06	-0.494	-0.494	1.173
717	Nicotinamide	Cofactor	1	Ctrl_Untreated_1_6	Ctrl_Untreated_1	1.0	NAD biosynthesis	True	2.753e+07	3.525	3.525	-0.9899
51	PEP	Carbon	1	Ctrl_Untreated_1_6	Ctrl_Untreated_1	1.0	Glycolysis, GNG	True	2.484e+06	0.05497	0.05497	-0.9962
237	Spermine	Amino acid	1	Ctrl_Untreated_1_6	Ctrl_Untreated_1	1.0	Polyamines	True	2.953e+05	-3.018	-3.018	-1.527
385	Uracil	Nucleotide	1	Ctrl_Untreated_1_6	Ctrl_Untreated_1	1.0	Pyrimidine bases	True	6.472e+05	-1.885	-1.885	-2.15
377	Uridine	Nucleotide	1	Ctrl_Untreated_1_6	Ctrl_Untreated_1	1.0	Pyrimidine nucleosides	True	1.986e+07	3.054	3.054	-0.9952
348	Allantoin	Nucleotide	1	Ctrl_Untreated_1_6	Ctrl_Untreated_1	1.0	Purine degradation	True	2.29e+06	-0.06266	-0.06266	0.1129
335	Inosine	Nucleotide	1	Ctrl_Untreated_1_6	Ctrl_Untreated_1	1.0	Purine nucleosides	True	4.545e+07	4.248	4.248	-1.251
81	Ile	Amino acid	1	Ctrl_Untreated_1_6	Ctrl_Untreated_1	1.0	Proteinogenic amino acids	True	7.298e+08	8.254	8.254	-0.4616
72	Ala	Amino acid	1	Ctrl_Untreated_1_6	Ctrl_Untreated_1	1.0	Proteinogenic amino acids	True	6.049e+08	7.983	7.983	-0.5187
79	Thr	Amino acid	1	Ctrl_Untreated_1_6	Ctrl_Untreated_1	1.0	Proteinogenic amino acids	True	4.331e+08	7.501	7.501	-0.5088
88	Tyr	Amino acid	1	Ctrl_Untreated_1_6	Ctrl_Untreated_1	1.0	Proteinogenic amino acids	True	2.575e+08	6.751	6.751	-0.7731
84	Lys	Amino acid	1	Ctrl_Untreated_1_6	Ctrl_Untreated_1	1.0	Proteinogenic amino acids	True	3.913e+08	7.354	7.354	-0.6356
86	Met	Amino acid	1	Ctrl_Untreated_1_6	Ctrl_Untreated_1	1.0	Proteinogenic amino acids	True	3.989e+08	7.382	7.382	-0.8457
61	Malate	Carbon	1	Ctrl_Untreated_1_6	Ctrl_Untreated_1	1.0	TCA cycle	True	2.614e+08	6.773	6.773	-0.3992
235	Putrescine	Amino acid	1	Ctrl_Untreated_1_6	Ctrl_Untreated_1	1.0	Polyamines	True	1.888e+06	-0.3411	-0.3411	0.1502
324	2'-dU	Nucleotide	1	Ctrl_Untreated_1_6	Ctrl_Untreated_1	0.714285714285714	Deoxy-nucleosides	False			-5.281	-0.9009
49	3-P-Glycerate	Carbon	1	Ctrl_Untreated_1_6	Ctrl_Untreated_1	1.0	Glycolysis, GNG	True	2.712e+07	3.503	3.503	0.2378
189	Kynurenate	Amino acid	1	Ctrl_Untreated_1_6	Ctrl_Untreated_1	0.714285714285714	Amino acid derivatives	True	4.961e+04	-5.591	-5.591	0.2069
234	5-Me-Thioadenosine	Amino acid	1	Ctrl_Untreated_1_6	Ctrl_Untreated_1	1.0	SAM metabolism	True	9.746e+06	2.027	2.027	0.1415
59	Succinate	Carbon	1	Ctrl_Untreated_1_6	Ctrl_Untreated_1	1.0	TCA cycle	True	4.715e+06	0.9793	0.9793	0.6783

Metabolite ID	Name	Super Pathway	Dataset	Sample ID	Group ID	Detection Fraction	Pathway	Detected	Raw Intensity	Log2 Norm Intensity	Norm Imputed	Log2 Ctrl Norm
36	Ribose	Carbon	1	Ctrl_Untreated_1_6	Ctrl_Untreated_1	1.0	Sugars and sugar alcohols	True	6.83e+05	-1.808	-1.808	-0.5744
133	Ornithine	Amino acid	1	Ctrl_Untreated_1_6	Ctrl_Untreated_1	1.0	Amino acids degradation intermediates	True	5.556e+07	4.538	4.538	0.05872
313	5-Oxoproline	Amino acid	1	Ctrl_Untreated_1_6	Ctrl_Untreated_1	1.0	Glutathione derivatives	True	9.143e+06	1.935	1.935	-0.1526
165	N-6-Tri-Me-Lys	Amino acid	1	Ctrl_Untreated_1_6	Ctrl_Untreated_1	1.0	Amino acid derivatives	True	1.659e+07	2.795	2.795	-0.9689
380	Orotate	Nucleotide	1	Ctrl_Untreated_1_6	Ctrl_Untreated_1	0.857142857142857	Pyrimidine (UMP) biosynthesis	False			-4.969	-1.339
724	Pantothenate	Cofactor	1	Ctrl_Untreated_1_6	Ctrl_Untreated_1	1.0	Coenzyme A biosynthesis	True	2.569e+08	6.747	6.747	0.4372
150	N-Me-Gly	Amino acid	1	Ctrl_Untreated_1_6	Ctrl_Untreated_1	1.0	Amino acid derivatives	True	1.204e+06	-0.9897	-0.9897	-0.6892
122	3-OH-Isobutyrate	Amino acid	1	Ctrl_Untreated_1_6	Ctrl_Untreated_1	0.857142857142857	Amino acids degradation intermediates	True	1.601e+05	-3.901	-3.901	-0.7452
241	4-Acetamidobutanoate	Amino acid	1	Ctrl_Untreated_1_6	Ctrl_Untreated_1	1.0	Polyamine derivatives	True	3.408e+06	0.5112	0.5112	0.07022
711	alpha-Tocopherol	Cofactor	1	Ctrl_Untreated_1_6	Ctrl_Untreated_1	1.0	Cofactors	True	4.971e+06	1.056	1.056	0.2752
55	Citrate	Carbon	1	Ctrl_Untreated_1_6	Ctrl_Untreated_1	1.0	TCA cycle	True	7.883e+06	1.721	1.721	-0.2282
387	3-Aminoisobutyrate	Nucleotide	1	Ctrl_Untreated_1_6	Ctrl_Untreated_1	0.857142857142857	Pyrimidine degradation	True	1.15e+06	-1.057	-1.057	0.957
338	Guanosine	Nucleotide	1	Ctrl_Untreated_1_6	Ctrl_Untreated_1	1.0	Purine nucleosides	True	4.256e+07	4.154	4.154	-0.9524
209	N-Ac-Ala	Amino acid	1	Ctrl_Untreated_1_6	Ctrl_Untreated_1	1.0	N-acetylated amino acids	True	8.322e+05	-1.523	-1.523	-0.3216
221	N-Ac-Met	Amino acid	1	Ctrl_Untreated_1_6	Ctrl_Untreated_1	1.0	N-acetylated amino acids	True	4.956e+06	1.051	1.051	-0.6928
228	N-Ac-Val	Amino acid	1	Ctrl_Untreated_1_6	Ctrl_Untreated_1	0.571428571428571	N-acetylated amino acids	False			-5.82	-0.449
346	Urate	Nucleotide	1	Ctrl_Untreated_1_6	Ctrl_Untreated_1	1.0	Purine degradation	True	1.172e+06	-1.028	-1.028	0.03441
90	Arg	Amino acid	1	Ctrl_Untreated_1_6	Ctrl_Untreated_1	1.0	Proteinogenic amino acids	True	1.397e+09	9.19	9.19	-0.5152
60	Fumarate	Carbon	1	Ctrl_Untreated_1_6	Ctrl_Untreated_1	1.0	TCA cycle	True	3.745e+06	0.647	0.647	-0.3682
78	Ser	Amino acid	1	Ctrl_Untreated_1_6	Ctrl_Untreated_1	1.0	Proteinogenic amino acids	True	1.756e+08	6.198	6.198	-1.059
83	Val	Amino acid	1	Ctrl_Untreated_1_6	Ctrl_Untreated_1	1.0	Proteinogenic amino acids	True	2.944e+08	6.944	6.944	-0.9282
734	Pyridoxal	Cofactor	1	Ctrl_Untreated_1_6	Ctrl_Untreated_1	1.0	PLP biosynthesis and salvage	True	6.787e+06	1.505	1.505	-0.2781

Metabolite ID	Name	Super Pathway	Dataset	Sample ID	Group ID	Detection Fraction	Pathway	Detected	Raw Intensity	Log2 Norm Intensity	Norm Imputed	Log2 Ctrl Norm
136	Urea	Amino acid	1	Ctrl_Untreated_1_6	Ctrl_Untreated_1	0.857142857142857	Amino acids degradation intermediates	True	2.735e+06	0.1938	0.1938	0.04256
67	Ribose 1-P	Carbon	1	Ctrl_Untreated_1_6	Ctrl_Untreated_1	1.0	Pentose phosphate pathway (PPP)	True	2.412e+06	0.01267	0.01267	-0.6085
284	Carnosine	Amino acid	1	Ctrl_Untreated_1_6	Ctrl_Untreated_1	1.0	Dipeptides	True	5.278e+05	-2.18	-2.18	-0.07152
306	gamma-Glu-Cys	Amino acid	1	Ctrl_Untreated_1_6	Ctrl_Untreated_1	1.0	Glutathione biosynthesis	True	1.719e+06	-0.4764	-0.4764	-0.1396
712	Retinol (Vit A)	Cofactor	1	Ctrl_Untreated_1_6	Ctrl_Untreated_1	1.0	Cofactors	True	4.067e+05	-2.556	-2.556	-0.1224
85	Cys	Amino acid	1	Ctrl_Untreated_1_6	Ctrl_Untreated_1	1.0	Proteinogenic amino acids	True	2.82e+07	3.56	3.56	-0.9542
91	Pro	Amino acid	1	Ctrl_Untreated_1_6	Ctrl_Untreated_1	1.0	Proteinogenic amino acids	True	9.288e+08	8.601	8.601	-0.8066
308	Glutathione, Reduced	Amino acid	1	Ctrl_Untreated_1_6	Ctrl_Untreated_1	1.0	Glutathione	True	3.007e+08	6.974	6.974	0.01813
107	Citrulline	Amino acid	1	Ctrl_Untreated_1_6	Ctrl_Untreated_1	1.0	Amino acids biosynthesis intermediates	True	1.847e+07	2.949	2.949	0.1249
328	IMP	Nucleotide	1	Ctrl_Untreated_1_6	Ctrl_Untreated_1	0.285714285714286	Purine nucleotides	False			-4.53	-0.2088
706	FAD	Cofactor	1	Ctrl_Untreated_1_6	Ctrl_Untreated_1	1.0	Cofactors	True	5.043e+05	-2.246	-2.246	-0.056
735	Pyridoxamine	Cofactor	1	Ctrl_Untreated_1_6	Ctrl_Untreated_1	1.0	PLP biosynthesis and salvage	True	2.788e+06	0.2215	0.2215	0.4145
199	Serotonin	Amino acid	1	Ctrl_Untreated_1_6	Ctrl_Untreated_1	1.0	Amino acid derivatives	True	5.793e+06	1.276	1.276	3.76e-03
370	CMP	Nucleotide	1	Ctrl_Untreated_1_6	Ctrl_Untreated_1	1.0	Pyrimidine nucleotides	True	1.429e+07	2.579	2.579	0.7404
287	gamma-Glu-Gln	Amino acid	1	Ctrl_Untreated_1_6	Ctrl_Untreated_1	1.0	Gamma-glutamyl dipeptides	True	2.551e+06	0.09325	0.09325	-0.7477
14	UDP-Glucuronate	Carbon	1	Ctrl_Untreated_1_6	Ctrl_Untreated_1	0.857142857142857	Polysaccharide biosynthesis	True	2.698e+06	0.1742	0.1742	2.123
229	N-Formyl-Met	Amino acid	1	Ctrl_Untreated_1_6	Ctrl_Untreated_1	1.0	N-formylated amino acids	True	8.651e+04	-4.789	-4.789	-0.9665
350	3',5'-cAMP	Nucleotide	1	Ctrl_Untreated_1_6	Ctrl_Untreated_1	1.0	Purine derivatives in signaling	True	1.037e+05	-4.527	-4.527	-0.9083
371	CDP	Nucleotide	1	Ctrl_Untreated_1_6	Ctrl_Untreated_1	0.571428571428571	Pyrimidine nucleotides	True	7.983e+05	-1.583	-1.583	1.737
372	CTP	Nucleotide	1	Ctrl_Untreated_1_6	Ctrl_Untreated_1	0.571428571428571	Pyrimidine nucleotides	True	1.989e+05	-3.588	-3.588	0.06578
333	GDP	Nucleotide	1	Ctrl_Untreated_1_6	Ctrl_Untreated_1	0.571428571428571	Purine nucleotides	True	9.099e+05	-1.394	-1.394	1.106
332	GMP	Nucleotide	1	Ctrl_Untreated_1_6	Ctrl_Untreated_1	1.0	Purine nucleotides	True	2.055e+07	3.103	3.103	2.404

Metabolite ID	Name	Super Pathway	Dataset	Sample ID	Group ID	Detection Fraction	Pathway	Detected	Raw Intensity	Log2 Norm Intensity	Norm Imputed	Log2 Ctrl Norm
373	UMP	Nucleotide	1	Ctrl_Untreated_1_6	Ctrl_Untreated_1	1.0	Pyrimidine nucleotides	True	8.952e+06	1.904	1.904	2.96
389	3'-CMP	Nucleotide	1	Ctrl_Untreated_1_6	Ctrl_Untreated_1	1.0	Pyrimidine derivatives in signaling	True	2.535e+06	0.0839	0.0839	1.135
330	ADP	Nucleotide	1	Ctrl_Untreated_1_6	Ctrl_Untreated_1	0.714285714285714	Purine nucleotides	True	9.818e+06	2.038	2.038	2.334
342	Hypoxanthine	Nucleotide	1	Ctrl_Untreated_1_6	Ctrl_Untreated_1	1.0	Purine bases	True	1.875e+07	2.971	2.971	-0.9681
736	Pyridoxamine-P	Cofactor	1	Ctrl_Untreated_1_6	Ctrl_Untreated_1	1.0	PLP biosynthesis and salvage	True	2.35e+05	-3.347	-3.347	-0.3692
148	Betaine	Amino acid	1	Ctrl_Untreated_1_6	Ctrl_Untreated_1	1.0	Amino acid derivatives	True	5.972e+07	4.642	4.642	-0.1635
344	Xanthine	Nucleotide	1	Ctrl_Untreated_1_6	Ctrl_Untreated_1	1.0	Purine bases	True	3.881e+06	0.6987	0.6987	-0.2089
386	3-Ureidopropionate	Nucleotide	1	Ctrl_Untreated_1_6	Ctrl_Untreated_1	1.0	Pyrimidine degradation	True	4.34e+05	-2.462	-2.462	-1.543
149	DiMe-Gly	Amino acid	1	Ctrl_Untreated_1_6	Ctrl_Untreated_1	1.0	Amino acid derivatives	True	1.532e+06	-0.6423	-0.6423	-0.122
703	NAD+	Cofactor	1	Ctrl_Untreated_1_6	Ctrl_Untreated_1	1.0	Cofactors	True	1.716e+07	2.843	2.843	0.1066
709	Pyridoxal-P	Cofactor	1	Ctrl_Untreated_1_6	Ctrl_Untreated_1	1.0	Cofactors	True	5.954e+05	-2.006	-2.006	0.3021
731	Thiamin (Vitamin B1)	Cofactor	1	Ctrl_Untreated_1_6	Ctrl_Untreated_1	1.0	TPP biosynthesis	True	3.4e+06	0.5078	0.5078	-0.2399
374	UDP	Nucleotide	1	Ctrl_Untreated_1_6	Ctrl_Untreated_1	0.714285714285714	Pyrimidine nucleotides	True	5.237e+06	1.131	1.131	2.685
102	2-Aminoadipate	Amino acid	1	Ctrl_Untreated_1_6	Ctrl_Untreated_1	1.0	Amino acids biosynthesis intermediates	True	4.695e+05	-2.349	-2.349	-0.239
45	Fructose-6-P	Carbon	1	Ctrl_Untreated_1_6	Ctrl_Untreated_1	1.0	Glycolysis, GNG	True	4.313e+06	0.851	0.851	0.7757
320	TMP	Nucleotide	1	Ctrl_Untreated_1_6	Ctrl_Untreated_1	0.571428571428571	Deoxy-nucleotides	True	2.292e+05	-3.383	-3.383	1.408
341	XMP	Nucleotide	1	Ctrl_Untreated_1_6	Ctrl_Untreated_1	0.857142857142857	IMP conversion to AMP & GMP	False			-6.353	-1.778
120	beta-OH-Isovalerate	Amino acid	1	Ctrl_Untreated_1_6	Ctrl_Untreated_1	0.857142857142857	Amino acids degradation intermediates	True	2.262e+05	-3.402	-3.402	-0.2368
322	2'-dI	Nucleotide	1	Ctrl_Untreated_1_6	Ctrl_Untreated_1	0.857142857142857	Deoxy-nucleosides	False			-5.917	-1.791
4	GlcNAc 6-P	Carbon	1	Ctrl_Untreated_1_6	Ctrl_Untreated_1	1.0	Aminosugar biosynthesis	True	1.575e+06	-0.6023	-0.6023	-0.9309
337	Xanthosine	Nucleotide	1	Ctrl_Untreated_1_6	Ctrl_Untreated_1	1.0	Purine nucleosides	True	1.919e+05	-3.64	-3.64	-0.5652
188	Kynurenine	Amino acid	1	Ctrl_Untreated_1_6	Ctrl_Untreated_1	1.0	Amino acid derivatives	True	1.088e+06	-1.135	-1.135	-0.4439

Metabolite ID	Name	Super Pathway	Dataset	Sample ID	Group ID	Detection Fraction	Pathway	Detected	Raw Intensity	Log2 Norm Intensity	Norm Imputed	Log2 Ctrl Norm
63	6-P-Gluconate	Carbon	1	Ctrl_Untreated_1_6	Ctrl_Untreated_1	1.0	Pentose phosphate pathway (PPP)	True	1.513e+07	2.661	2.661	0.6276
40	Glucuronate	Carbon	1	Ctrl_Untreated_1_6	Ctrl_Untreated_1	1.0	Sugars and sugar alcohols	True	7.599e+05	-1.654	-1.654	-0.1149
108	Argininosuccinate	Amino acid	1	Ctrl_Untreated_1_6	Ctrl_Untreated_1	1.0	Amino acids biosynthesis intermediates	True	8.712e+05	-1.457	-1.457	-1.231
710	Carnitine	Cofactor	1	Ctrl_Untreated_1_6	Ctrl_Untreated_1	1.0	Cofactors	True	1.259e+08	5.718	5.718	-0.2592
725	P-Pantetheine	Cofactor	1	Ctrl_Untreated_1_6	Ctrl_Untreated_1	1.0	Coenzyme A biosynthesis	True	1.797e+05	-3.734	-3.734	-0.02939
48	DHAP	Carbon	1	Ctrl_Untreated_1_6	Ctrl_Untreated_1	1.0	Glycolysis, GNG	True	2.289e+07	3.259	3.259	0.1622
17	Maltose	Carbon	1	Ctrl_Untreated_1_6	Ctrl_Untreated_1	1.0	Glycogen degradation	True	6.679e+05	-1.84	-1.84	-0.5474
359	N1-Me-Adenosine	Nucleotide	1	Ctrl_Untreated_1_6	Ctrl_Untreated_1	0.857142857142857	Purine derivatives in RNAs	False			-2.032	-0.9939
159	3-Me-His	Amino acid	1	Ctrl_Untreated_1_6	Ctrl_Untreated_1	1.0	Amino acid derivatives	True	1.144e+05	-4.385	-4.385	-0.1501
155	4-Guanidinobutanoate	Amino acid	1	Ctrl_Untreated_1_6	Ctrl_Untreated_1	1.0	Amino acid derivatives	True	8.72e+05	-1.455	-1.455	-0.02138
164	5-OH-Lys	Amino acid	1	Ctrl_Untreated_1_6	Ctrl_Untreated_1	1.0	Amino acid derivatives	True	4.764e+05	-2.327	-2.327	-0.5571
357	Adenosine-3',5'-PP	Nucleotide	1	Ctrl_Untreated_1_6	Ctrl_Untreated_1	0.857142857142857	Purine byproducts of metabolic processes	True	1.069e+05	-4.484	-4.484	-1.006
104	Cystathionine	Amino acid	1	Ctrl_Untreated_1_6	Ctrl_Untreated_1	1.0	Amino acids biosynthesis intermediates	True	1.059e+07	2.147	2.147	-0.06217
113	Imidazole Lactate	Amino acid	1	Ctrl_Untreated_1_6	Ctrl_Untreated_1	0.857142857142857	Amino acids degradation intermediates	True	2.923e+05	-3.032	-3.032	0.6442
215	N-Ac-Glu	Amino acid	1	Ctrl_Untreated_1_6	Ctrl_Untreated_1	1.0	N-acetylated amino acids	True	5.193e+06	1.119	1.119	-0.6248
310	S-Lactoyl-Glutathione	Amino acid	1	Ctrl_Untreated_1_6	Ctrl_Untreated_1	0.857142857142857	Glutathione derivatives	True	4.71e+06	0.978	0.978	0.6216
5	GlcNAc 1-P	Carbon	1	Ctrl_Untreated_1_6	Ctrl_Untreated_1	1.0	Aminosugar biosynthesis	True	3.757e+05	-2.67	-2.67	-0.7593
34	Ribitol	Carbon	1	Ctrl_Untreated_1_6	Ctrl_Untreated_1	1.0	Sugars and sugar alcohols	True	2.254e+05	-3.407	-3.407	0.02298
10	UDP-Galactose	Carbon	1	Ctrl_Untreated_1_6	Ctrl_Untreated_1	0.857142857142857	Polysaccharide biosynthesis	True	3.593e+06	0.5875	0.5875	1.214
13	Guanosine 5'-PP-Fucose	Carbon	1	Ctrl_Untreated_1_6	Ctrl_Untreated_1	1.0	Polysaccharide biosynthesis	True	5.608e+05	-2.092	-2.092	0.207

Metabolite ID	Name	Super Pathway	Dataset	Sample ID	Group ID	Detection Fraction	Pathway	Detected	Raw Intensity	Log2 Norm Intensity	Norm Imputed	Log2 Ctrl Norm
19	Maltotetraose	Carbon	1	Ctrl_Untreated_1_6	Ctrl_Untreated_1	1.0	Glycogen degradation	True	2.759e+06	0.2063	0.2063	0.25
233	SAM	Amino acid	1	Ctrl_Untreated_1_6	Ctrl_Untreated_1	1.0	SAM metabolism	True	1.89e+06	-0.3392	-0.3392	-0.1828
129	5-Aminovalerate	Amino acid	1	Ctrl_Untreated_1_6	Ctrl_Untreated_1	1.0	Amino acids degradation intermediates	True	3.724e+06	0.6392	0.6392	-0.3625
741	5-Me-THF	Cofactor	1	Ctrl_Untreated_1_6	Ctrl_Untreated_1	0.714285714285714	Folate metabolism	True	5.866e+04	-5.349	-5.349	-0.5915
198	Indolelactate	Amino acid	1	Ctrl_Untreated_1_6	Ctrl_Untreated_1	0.857142857142857	Amino acid derivatives	True	1.649e+05	-3.859	-3.859	-0.07147
254	Gly-Val	Amino acid	1	Ctrl_Untreated_1_6	Ctrl_Untreated_1	1.0	Dipeptides	True	2.391e+06	0	0	-1.111
291	gamma-Glu-Leu	Amino acid	1	Ctrl_Untreated_1_6	Ctrl_Untreated_1	1.0	Gamma-glutamyl dipeptides	True	1.738e+06	-0.4605	-0.4605	0.02831
173	Met Sulfoxide	Amino acid	1	Ctrl_Untreated_1_6	Ctrl_Untreated_1	1.0	Amino acid derivatives	True	1.251e+07	2.387	2.387	-0.1803
43	Glucose	Carbon	1	Ctrl_Untreated_1_6	Ctrl_Untreated_1	1.0	Glycolysis, GNG	True	7.741e+07	5.017	5.017	-0.03481
185	Phenyllactate	Amino acid	1	Ctrl_Untreated_1_6	Ctrl_Untreated_1	0.285714285714286	Amino acid derivatives	False			-5.917	-0.1051
156	Homo-Arg	Amino acid	1	Ctrl_Untreated_1_6	Ctrl_Untreated_1	1.0	Amino acid derivatives	True	4.615e+06	0.9484	0.9484	-0.2321
135	Homocitrulline	Amino acid	1	Ctrl_Untreated_1_6	Ctrl_Untreated_1	1.0	Amino acids degradation intermediates	True	6.91e+05	-1.791	-1.791	-0.08847
719	Nicotinamide MN	Cofactor	1	Ctrl_Untreated_1_6	Ctrl_Untreated_1	1.0	NAD biosynthesis	True	2.503e+06	0.06609	0.06609	-0.1195
212	N-Ac-Asp	Amino acid	1	Ctrl_Untreated_1_6	Ctrl_Untreated_1	1.0	N-acetylated amino acids	True	2.929e+06	0.2926	0.2926	-0.4047
720	1-Me-Nicotinamide	Cofactor	1	Ctrl_Untreated_1_6	Ctrl_Untreated_1	1.0	Derivatives of NA, nicotinamide and NAD	True	5.774e+08	7.916	7.916	0.02499
216	N-Ac-Gly	Amino acid	1	Ctrl_Untreated_1_6	Ctrl_Untreated_1	0.714285714285714	N-acetylated amino acids	False			-4.155	-0.6151
70	Creatine	Carbon	1	Ctrl_Untreated_1_6	Ctrl_Untreated_1	1.0	Creatine energy storage	True	1.767e+09	9.53	9.53	0.1126
26	Galactonate	Carbon	1	Ctrl_Untreated_1_6	Ctrl_Untreated_1	0.857142857142857	Sugars and sugar alcohols	True	5.165e+05	-2.211	-2.211	-1.406
309	Glutathione, Oxidized	Amino acid	1	Ctrl_Untreated_1_6	Ctrl_Untreated_1	1.0	Glutathione	True	7.037e+06	1.557	1.557	0.3644
35	Ribonate	Carbon	1	Ctrl_Untreated_1_6	Ctrl_Untreated_1	1.0	Sugars and sugar alcohols	True	2.409e+06	0.01048	0.01048	0.5377
160	1-Me-His	Amino acid	1	Ctrl_Untreated_1_6	Ctrl_Untreated_1	1.0	Amino acid derivatives	True	2.332e+07	3.286	3.286	-0.1553

Metabolite ID	Name	Super Pathway	Dataset	Sample ID	Group ID	Detection Fraction	Pathway	Detected	Raw Intensity	Log2 Norm Intensity	Norm Imputed	Log2 Ctrl Norm
44	Glucose 6-P	Carbon	1	Ctrl_Untreated_1_6	Ctrl_Untreated_1	0.857142857142857	Glycolysis, GNG	True	1.885e+06	-0.3432	-0.3432	1.134
704	NADH	Cofactor	1	Ctrl_Untreated_1_6	Ctrl_Untreated_1	1.0	Cofactors	True	3.516e+06	0.5561	0.5561	1.202
275	Thr-Phe	Amino acid	1	Ctrl_Untreated_1_6	Ctrl_Untreated_1	0.857142857142857	Dipeptides	True	9.365e+05	-1.352	-1.352	0.3007
738	Pyridoxate	Cofactor	1	Ctrl_Untreated_1_6	Ctrl_Untreated_1	1.0	PLP biosynthesis and salvage	True	2.608e+05	-3.197	-3.197	0.1675
177	3-(4-OH-Phenyl)Lactate	Amino acid	1	Ctrl_Untreated_1_6	Ctrl_Untreated_1	1.0	Amino acid derivatives	True	3.926e+05	-2.607	-2.607	-0.1412
206	Trans-4-OH-Pro	Amino acid	1	Ctrl_Untreated_1_6	Ctrl_Untreated_1	1.0	Amino acid derivatives	True	7.756e+07	5.019	5.019	-0.1357
329	AMP	Nucleotide	1	Ctrl_Untreated_1_6	Ctrl_Untreated_1	1.0	Purine nucleotides	True	1.561e+08	6.028	6.028	2.393
11	UDP-Glucose	Carbon	1	Ctrl_Untreated_1_6	Ctrl_Untreated_1	1.0	Polysaccharide biosynthesis	True	4.991e+06	1.061	1.061	1.167
158	4-Imidazole-Ac	Amino acid	1	Ctrl_Untreated_1_6	Ctrl_Untreated_1	1.0	Amino acid derivatives	True	7.313e+05	-1.709	-1.709	0.4373
111	1-Me-Imidazole-Ac	Amino acid	1	Ctrl_Untreated_1_6	Ctrl_Untreated_1	1.0	Amino acids degradation intermediates	True	7.084e+05	-1.755	-1.755	0.1786
345	Guanine	Nucleotide	1	Ctrl_Untreated_1_6	Ctrl_Untreated_1	1.0	Purine bases	True	3.847e+07	4.008	4.008	-0.9785
22	N-Ac-Neuraminate	Carbon	1	Ctrl_Untreated_1_6	Ctrl_Untreated_1	1.0	Aminosugar derivatives	True	1.592e+06	-0.587	-0.587	-0.4516
721	N'-Methylnicotinate	Cofactor	1	Ctrl_Untreated_1_6	Ctrl_Untreated_1	1.0	Derivatives of NA, nicotinamide and NAD	True	9.967e+05	-1.263	-1.263	-0.2549
183	Phenol Sulfate	Amino acid	1	Ctrl_Untreated_1_6	Ctrl_Untreated_1	0.857142857142857	Amino acid derivatives	True	7.143e+04	-5.065	-5.065	-0.4251
718	Nicotinamide Riboside	Cofactor	1	Ctrl_Untreated_1_6	Ctrl_Untreated_1	1.0	NAD biosynthesis	True	6.484e+05	-1.883	-1.883	-3.473
297	gamma-Glu-Thr	Amino acid	1	Ctrl_Untreated_1_6	Ctrl_Untreated_1	1.0	Gamma-glutamyl dipeptides	True	7.354e+06	1.621	1.621	0.9806
295	gamma-Glu-Phe	Amino acid	1	Ctrl_Untreated_1_6	Ctrl_Untreated_1	0.428571428571429	Gamma-glutamyl dipeptides	False			-5.975	-0.1899
347	Allantoic Acid	Nucleotide	1	Ctrl_Untreated_1_6	Ctrl_Untreated_1	0.571428571428571	Purine degradation	False			-6.474	-0.8345
399	Pseudouridine	Nucleotide	1	Ctrl_Untreated_1_6	Ctrl_Untreated_1	1.0	Pyrimidine derivatives in RNAs	True	5.574e+05	-2.101	-2.101	-0.07103
375	UTP	Nucleotide	1	Ctrl_Untreated_1_6	Ctrl_Untreated_1	0.571428571428571	Pyrimidine nucleotides	True	1.601e+06	-0.5789	-0.5789	1.397
144	Glu, gamma-Me Ester	Amino acid	1	Ctrl_Untreated_1_6	Ctrl_Untreated_1	1.0	Amino acid derivatives	True	1.522e+06	-0.6515	-0.6515	-1.229

Metabolite ID	Name	Super Pathway	Dataset	Sample ID	Group ID	Detection Fraction	Pathway	Detected	Raw Intensity	Log2 Norm Intensity	Norm Imputed	Log2 Ctrl Norm
292	gamma-Glu-epsilon-Lysine	Amino acid	1	Ctrl_Untreated_1_6	Ctrl_Untreated_1	1.0	Gamma-glutamyl dipeptides	True	1.626e+06	-0.5562	-0.5562	-0.3648
225	N-Ac-Thr	Amino acid	1	Ctrl_Untreated_1_6	Ctrl_Untreated_1	1.0	N-acetylated amino acids	True	7.327e+05	-1.707	-1.707	-0.204
211	N-Ac-Asn	Amino acid	1	Ctrl_Untreated_1_6	Ctrl_Untreated_1	1.0	N-acetylated amino acids	True	1.912e+05	-3.645	-3.645	-0.9598
151	Phenylacetylglycine	Amino acid	1	Ctrl_Untreated_1_6	Ctrl_Untreated_1	1.0	Amino acid derivatives	True	1.879e+06	-0.3477	-0.3477	0.4393
217	N-Ac-His	Amino acid	1	Ctrl_Untreated_1_6	Ctrl_Untreated_1	1.0	N-acetylated amino acids	True	2.857e+05	-3.065	-3.065	0.4198
288	gamma-Glu-Gly	Amino acid	1	Ctrl_Untreated_1_6	Ctrl_Untreated_1	0.571428571428571	Gamma-glutamyl dipeptides	True	6.959e+05	-1.781	-1.781	0.04366
222	N-Ac-Phe	Amino acid	1	Ctrl_Untreated_1_6	Ctrl_Untreated_1	0.571428571428571	N-acetylated amino acids	True	3.485e+04	-6.101	-6.101	0.1719
71	Creatine-P	Carbon	1	Ctrl_Untreated_1_6	Ctrl_Untreated_1	1.0	Creatine energy storage	True	8.511e+04	-4.812	-4.812	-0.5571
210	N-Ac-Arg	Amino acid	1	Ctrl_Untreated_1_6	Ctrl_Untreated_1	1.0	N-acetylated amino acids	True	3.511e+05	-2.768	-2.768	-0.09241
218	N-Ac-Ile	Amino acid	1	Ctrl_Untreated_1_6	Ctrl_Untreated_1	0.428571428571429	N-acetylated amino acids	False			-6.52	-0.5541
251	Gly-Leu	Amino acid	1	Ctrl_Untreated_1_6	Ctrl_Untreated_1	1.0	Dipeptides	True	3.025e+06	0.339	0.339	-1.3
290	gamma-Glu-Ile	Amino acid	1	Ctrl_Untreated_1_6	Ctrl_Untreated_1	1.0	Gamma-glutamyl dipeptides	True	9.769e+05	-1.292	-1.292	0.04702
316	Ophthalmate	Amino acid	1	Ctrl_Untreated_1_6	Ctrl_Untreated_1	1.0	Oxidative stress markers	True	1.77e+07	2.888	2.888	0.5452
125	Isovaleryl-Gly	Amino acid	1	Ctrl_Untreated_1_6	Ctrl_Untreated_1	0.857142857142857	Amino acids degradation intermediates	True	7.725e+04	-4.952	-4.952	0.4573
368	7-Me-Guanine	Nucleotide	1	Ctrl_Untreated_1_6	Ctrl_Untreated_1	1.0	Purine derivatives in RNAs	True	3.177e+05	-2.912	-2.912	-0.7221
208	Pro-OH-Pro	Amino acid	1	Ctrl_Untreated_1_6	Ctrl_Untreated_1	1.0	Amino acid derivatives	True	2.097e+07	3.132	3.132	-0.1971
366	N2,N2-DiMe-Guanosine	Nucleotide	1	Ctrl_Untreated_1_6	Ctrl_Untreated_1	0.857142857142857	Purine derivatives in RNAs	True	1.088e+05	-4.457	-4.457	-0.3438
352	3'-AMP	Nucleotide	1	Ctrl_Untreated_1_6	Ctrl_Untreated_1	1.0	Purine derivatives in signaling	True	1.077e+06	-1.15	-1.15	0.3364
363	N6-Carbamoyl-Thr-Adenosine	Nucleotide	1	Ctrl_Untreated_1_6	Ctrl_Untreated_1	0.857142857142857	Purine derivatives in RNAs	True	3.716e+04	-6.008	-6.008	-1.323
314	Cys-Glutathione Disulfide	Amino acid	1	Ctrl_Untreated_1_6	Ctrl_Untreated_1	1.0	Oxidative stress markers	True	1.802e+05	-3.73	-3.73	-0.4457
382	Orotidine	Nucleotide	1	Ctrl_Untreated_1_6	Ctrl_Untreated_1	0.428571428571429	Pyrimidine (UMP) biosynthesis	False			-5.287	-0.9952

Metabolite ID	Name	Super Pathway	Dataset	Sample ID	Group ID	Detection Fraction	Pathway	Detected	Raw Intensity	Log2 Norm Intensity	Norm Imputed	Log2 Ctrl Norm
307	Cys-Gly	Amino acid	1	Ctrl_Untreated_1_6	Ctrl_Untreated_1	1.0	Glutathione biosynthesis	True	8.311e+06	1.797	1.797	0.1902
64	Sedoheptulose-7-P	Carbon	1	Ctrl_Untreated_1_6	Ctrl_Untreated_1	1.0	Pentose phosphate pathway (PPP)	True	1.947e+06	-0.2965	-0.2965	-0.4505
142	N-Ac-Asp-Glu	Amino acid	1	Ctrl_Untreated_1_6	Ctrl_Untreated_1	1.0	Amino acid derivatives	True	1.146e+06	-1.061	-1.061	0.08477
708	Thiamin-PP	Cofactor	1	Ctrl_Untreated_1_6	Ctrl_Untreated_1	0.571428571428571	Cofactors	False			-6.331	-0.3472
182	P-Cresol Sulfate	Amino acid	1	Ctrl_Untreated_1_6	Ctrl_Untreated_1	0.857142857142857	Amino acid derivatives	True	2.582e+05	-3.211	-3.211	-0.01494
250	Gly-Ile	Amino acid	1	Ctrl_Untreated_1_6	Ctrl_Untreated_1	1.0	Dipeptides	True	6.688e+05	-1.838	-1.838	-0.521
286	gamma-Glu-Glu	Amino acid	1	Ctrl_Untreated_1_6	Ctrl_Untreated_1	1.0	Gamma-glutamyl dipeptides	True	2.683e+06	0.1659	0.1659	0.1481
739	Deoxycarnitine	Cofactor	1	Ctrl_Untreated_1_6	Ctrl_Untreated_1	1.0	Carnitine biosynthesis	True	2.139e+07	3.161	3.161	-0.5904
203	DiMe-Arg	Amino acid	1	Ctrl_Untreated_1_6	Ctrl_Untreated_1	1.0	Amino acid derivatives	True	3.397e+07	3.828	3.828	-1.804
351	2'-AMP	Nucleotide	1	Ctrl_Untreated_1_6	Ctrl_Untreated_1	1.0	Purine derivatives in signaling	True	1.18e+05	-4.341	-4.341	-2.072
8	Cytidine 5'-P-N-Ac-Neuraminate	Carbon	1	Ctrl_Untreated_1_6	Ctrl_Untreated_1	1.0	Aminosugar biosynthesis	True	5.981e+05	-1.999	-1.999	-0.2538
285	gamma-Glu-Ala	Amino acid	1	Ctrl_Untreated_1_6	Ctrl_Untreated_1	0.857142857142857	Gamma-glutamyl dipeptides	True	3.097e+05	-2.949	-2.949	0.29
224	N-Ac-Ser	Amino acid	1	Ctrl_Untreated_1_6	Ctrl_Untreated_1	1.0	N-acetylated amino acids	True	3.97e+06	0.7314	0.7314	-1.148
244	Ala-Leu	Amino acid	1	Ctrl_Untreated_1_6	Ctrl_Untreated_1	1.0	Dipeptides	True	6.583e+06	1.461	1.461	0.3229
207	N-Me-Pro	Amino acid	1	Ctrl_Untreated_1_6	Ctrl_Untreated_1	1.0	Amino acid derivatives	True	1.068e+06	-1.164	-1.164	-0.2335
171	Cys Sulfinic Acid	Amino acid	1	Ctrl_Untreated_1_6	Ctrl_Untreated_1	0.857142857142857	Amino acid derivatives	True	6.458e+04	-5.211	-5.211	-1.913
181	O-Me-Tyr	Amino acid	1	Ctrl_Untreated_1_6	Ctrl_Untreated_1	0.857142857142857	Amino acid derivatives	True	1.206e+05	-4.309	-4.309	-0.9396
240	N-Ac-Putrescine	Amino acid	1	Ctrl_Untreated_1_6	Ctrl_Untreated_1	1.0	Polyamine derivatives	True	5.053e+05	-2.243	-2.243	0.1211
176	S-Me-Met	Amino acid	1	Ctrl_Untreated_1_6	Ctrl_Untreated_1	0.857142857142857	Amino acid derivatives	True	6.366e+05	-1.909	-1.909	-0.0677
339	AICAR	Nucleotide	1	Ctrl_Untreated_1_6	Ctrl_Untreated_1	0.571428571428571	IMP biosynthesis	True	1.192e+05	-4.327	-4.327	-0.02568
141	gamma-Carboxy-Glu	Amino acid	1	Ctrl_Untreated_1_6	Ctrl_Untreated_1	1.0	Amino acid derivatives	True	1.536e+06	-0.6387	-0.6387	0.1463

Metabolite ID	Name	Super Pathway	Dataset	Sample ID	Group ID	Detection Fraction	Pathway	Detected	Raw Intensity	Log2 Norm Intensity	Norm Imputed	Log2 Ctrl Norm
392	3'-UMP	Nucleotide	1	Ctrl_Untreated_1_6	Ctrl_Untreated_1	0.571428571428571	Pyrimidine derivatives in signaling	False			-5.207	-1.174
355	3'-GMP	Nucleotide	1	Ctrl_Untreated_1_6	Ctrl_Untreated_1	0.857142857142857	Purine derivatives in signaling	True	5.135e+05	-2.219	-2.219	1.718
282	Val-Leu	Amino acid	1	Ctrl_Untreated_1_6	Ctrl_Untreated_1	1.0	Dipeptides	True	7.021e+06	1.554	1.554	0.5821
140	Carboxyethyl-GABA	Amino acid	1	Ctrl_Untreated_1_6	Ctrl_Untreated_1	1.0	Amino acid derivatives	True	3.466e+05	-2.786	-2.786	-1.355
258	Ile-Gly	Amino acid	1	Ctrl_Untreated_1_6	Ctrl_Untreated_1	1.0	Dipeptides	True	1.499e+07	2.648	2.648	-0.6637
260	Leu-Ala	Amino acid	1	Ctrl_Untreated_1_6	Ctrl_Untreated_1	1.0	Dipeptides	True	6.186e+06	1.371	1.371	0.06989
265	Lys-Leu	Amino acid	1	Ctrl_Untreated_1_6	Ctrl_Untreated_1	0.857142857142857	Dipeptides	True	3.651e+05	-2.712	-2.712	-0.1003
263	Leu-Gly	Amino acid	1	Ctrl_Untreated_1_6	Ctrl_Untreated_1	1.0	Dipeptides	True	4.916e+07	4.361	4.361	1.34
281	Val-Gly	Amino acid	1	Ctrl_Untreated_1_6	Ctrl_Untreated_1	1.0	Dipeptides	True	3.427e+07	3.841	3.841	-0.6781
270	Pro-Gly	Amino acid	1	Ctrl_Untreated_1_6	Ctrl_Untreated_1	1.0	Dipeptides	True	1.249e+06	-0.9367	-0.9367	-1.683
114	Imidazole Propionate	Amino acid	1	Ctrl_Untreated_1_6	Ctrl_Untreated_1	1.0	Amino acids degradation intermediates	True	4.035e+05	-2.567	-2.567	-0.2565
267	Phe-Gly	Amino acid	1	Ctrl_Untreated_1_6	Ctrl_Untreated_1	1.0	Dipeptides	True	2.597e+07	3.441	3.441	-0.6527
266	Phe-Ala	Amino acid	1	Ctrl_Untreated_1_6	Ctrl_Untreated_1	1.0	Dipeptides	True	3.893e+06	0.7032	0.7032	-0.3735
278	Tyr-Gly	Amino acid	1	Ctrl_Untreated_1_6	Ctrl_Untreated_1	1.0	Dipeptides	True	6.552e+06	1.454	1.454	-0.7282
255	His-Ala	Amino acid	1	Ctrl_Untreated_1_6	Ctrl_Untreated_1	1.0	Dipeptides	True	5.187e+05	-2.205	-2.205	-0.8674
280	Val-Gln	Amino acid	1	Ctrl_Untreated_1_6	Ctrl_Untreated_1	1.0	Dipeptides	True	8.797e+06	1.879	1.879	0.2611
143	S-1-Pyrroline-5-Carboxylate	Amino acid	1	Ctrl_Untreated_1_6	Ctrl_Untreated_1	0.857142857142857	Amino acid derivatives	True	5.477e+05	-2.126	-2.126	-0.05812
232	SAH	Amino acid	1	Ctrl_Untreated_1_6	Ctrl_Untreated_1	1.0	SAM metabolism	True	1.078e+06	-1.149	-1.149	-0.6634
20	Erythronate	Carbon	1	Ctrl_Untreated_1_6	Ctrl_Untreated_1	1.0	Aminosugar derivatives	True	2.301e+07	3.266	3.266	-0.4752
248	Gln-Leu	Amino acid	1	Ctrl_Untreated_1_6	Ctrl_Untreated_1	1.0	Dipeptides	True	1.369e+06	-0.8052	-0.8052	0.1
276	Trp-Gly	Amino acid	1	Ctrl_Untreated_1_6	Ctrl_Untreated_1	1.0	Dipeptides	True	5.854e+05	-2.03	-2.03	-0.952
205	N-delta-Ac-Ornithine	Amino acid	1	Ctrl_Untreated_1_6	Ctrl_Untreated_1	1.0	Amino acid derivatives	True	1.208e+06	-0.9847	-0.9847	0.1363
163	Formimino-Glu	Amino acid	1	Ctrl_Untreated_1_6	Ctrl_Untreated_1	1.0	Amino acid derivatives	True	6.893e+05	-1.795	-1.795	-0.5548
204	N-Me-Arg	Amino acid	1	Ctrl_Untreated_1_6	Ctrl_Untreated_1	1.0	Amino acid derivatives	True	4.605e+06	0.9454	0.9454	-1.642
242	Guanidino-Ac	Amino acid	1	Ctrl_Untreated_1_6	Ctrl_Untreated_1	0.857142857142857	Creatine biosynthesis	True	1.588e+05	-3.913	-3.913	-0.03253

Metabolite ID	Name	Super Pathway	Dataset	Sample ID	Group ID	Detection Fraction	Pathway	Detected	Raw Intensity	Log2 Norm Intensity	Norm Imputed	Log2 Ctrl Norm
300	gamma-Glu-Val	Amino acid	1	Ctrl_Untreated_1_6	Ctrl_Untreated_1	0.857142857142857	Gamma-glutamyl dipeptides	True	4.883e+06	1.03	1.03	0.4092
53	Ac-CoA	Carbon	1	Ctrl_Untreated_1_6	Ctrl_Untreated_1	0.285714285714286	Acetyl-CoA	False			-7.274	-0.5114
18	Maltotriose	Carbon	1	Ctrl_Untreated_1_6	Ctrl_Untreated_1	1.0	Glycogen degradation	True	1.353e+06	-0.8222	-0.8222	-0.6644
294	gamma-Glu-Met	Amino acid	1	Ctrl_Untreated_1_6	Ctrl_Untreated_1	0.714285714285714	Gamma-glutamyl dipeptides	False			-4.503	-1.021
174	Met Sulfone	Amino acid	1	Ctrl_Untreated_1_6	Ctrl_Untreated_1	1.0	Amino acid derivatives	True	2.639e+05	-3.18	-3.18	-0.101
175	N-Ac-Met Sulfoxide	Amino acid	1	Ctrl_Untreated_1_6	Ctrl_Untreated_1	1.0	Amino acid derivatives	True	1.831e+06	-0.3848	-0.3848	-0.3612
25	Mannitol/Sorbitol	Carbon	1	Ctrl_Untreated_1_6	Ctrl_Untreated_1	1.0	Sugars and sugar alcohols	True	1.191e+07	2.316	2.316	0.09121
6	UDP-GlcNAc	Carbon	1	Ctrl_Untreated_1_6	Ctrl_Untreated_1	0.428571428571429	Aminosugar biosynthesis	False			-3.589	-0.4814
145	Pyro-Gln	Amino acid	1	Ctrl_Untreated_1_6	Ctrl_Untreated_1	1.0	Amino acid derivatives	True	3.981e+06	0.7353	0.7353	-0.1045
705	Coenzyme A	Cofactor	1	Ctrl_Untreated_1_6	Ctrl_Untreated_1	0.714285714285714	Cofactors	True	1.693e+05	-3.82	-3.82	0.1389
319	2'-dAMP	Nucleotide	1	Ctrl_Untreated_1_6	Ctrl_Untreated_1	0.571428571428571	Deoxy-nucleotides	True	2.596e+05	-3.204	-3.204	0.4768
119	alpha-OH-Isovalerate	Amino acid	1	Ctrl_Untreated_1_6	Ctrl_Untreated_1	0.714285714285714	Amino acids degradation intermediates	True	2.088e+05	-3.518	-3.518	-0.2191
46	Fructose 1,6-PP / Glucose 1,6-PP / Inositol-1,4-PP / Inositol-1,3-PP	Carbon	1	Ctrl_Untreated_1_6	Ctrl_Untreated_1	1.0	Glycolysis, GNG	True	1.045e+08	5.449	5.449	0.6179
137	1-Me-Guanidine	Amino acid	1	Ctrl_Untreated_1_6	Ctrl_Untreated_1	0.857142857142857	Amino acids degradation intermediates	False			-5.942	-0.7895
23	N-GlcNAc-Asn	Carbon	1	Ctrl_Untreated_1_6	Ctrl_Untreated_1	1.0	Aminosugar derivatives	True	3.687e+06	0.6245	0.6245	-0.08839
262	Leu-Gln	Amino acid	1	Ctrl_Untreated_1_6	Ctrl_Untreated_1	1.0	Dipeptides	True	7.165e+06	1.583	1.583	0.2868
24	Fructose	Carbon	1	Ctrl_Untreated_1_6	Ctrl_Untreated_1	1.0	Sugars and sugar alcohols	True	9.626e+06	2.009	2.009	0.06027
197	C-Glycosyl-Trp	Amino acid	1	Ctrl_Untreated_1_6	Ctrl_Untreated_1	1.0	Amino acid derivatives	True	1.318e+06	-0.8591	-0.8591	-0.5601
33	Arabitol/Xylitol	Carbon	1	Ctrl_Untreated_1_6	Ctrl_Untreated_1	1.0	Sugars and sugar alcohols	True	3.754e+05	-2.671	-2.671	-0.8113

Metabolite ID	Name	Super Pathway	Dataset	Sample ID	Group ID	Detection Fraction	Pathway	Detected	Raw Intensity	Log2 Norm Intensity	Norm Imputed	Log2 Ctrl Norm
128	N2-Ac-Lys/N6-Ac-Lys	Amino acid	1	Ctrl_Untreated_1_6	Ctrl_Untreated_1	1.0	Amino acids degradation intermediates	True	1.711e+06	-0.4831	-0.4831	-1.694
42	2-Me-Citrate	Carbon	1	Ctrl_Untreated_1_6	Ctrl_Untreated_1	0.857142857142857	Propionate metabolism	True	8.207e+04	-4.865	-4.865	-0.4818
12	Glucuronate 1-P	Carbon	1	Ctrl_Untreated_1_6	Ctrl_Untreated_1	1.0	Polysaccharide biosynthesis	True	8.324e+05	-1.522	-1.522	-0.2796
76	Gln	Amino acid	1	Ctrl_Untreated_1_7	Ctrl_Untreated_1	1.0	Proteinogenic amino acids	True	8.05e+08	7.934	7.934	-0.7469
89	Trp	Amino acid	1	Ctrl_Untreated_1_7	Ctrl_Untreated_1	1.0	Proteinogenic amino acids	True	1.606e+08	5.608	5.608	-0.06728
723	beta-Ala	Cofactor	1	Ctrl_Untreated_1_7	Ctrl_Untreated_1	1.0	Coenzyme A biosynthesis	True	2.416e+07	2.876	2.876	-0.1336
75	Glu	Amino acid	1	Ctrl_Untreated_1_7	Ctrl_Untreated_1	1.0	Proteinogenic amino acids	True	5.284e+09	10.65	10.65	-0.08842
77	Gly	Amino acid	1	Ctrl_Untreated_1_7	Ctrl_Untreated_1	1.0	Proteinogenic amino acids	True	1.018e+08	4.951	4.951	-0.6648
80	His	Amino acid	1	Ctrl_Untreated_1_7	Ctrl_Untreated_1	1.0	Proteinogenic amino acids	True	2.359e+07	2.841	2.841	-0.5743
82	Leu	Amino acid	1	Ctrl_Untreated_1_7	Ctrl_Untreated_1	1.0	Proteinogenic amino acids	True	1.281e+09	8.604	8.604	-0.6173
87	Phe	Amino acid	1	Ctrl_Untreated_1_7	Ctrl_Untreated_1	1.0	Proteinogenic amino acids	True	1.174e+09	8.479	8.479	-0.3328
130	Glutarate	Amino acid	1	Ctrl_Untreated_1_7	Ctrl_Untreated_1	1.0	Amino acids degradation intermediates	True	5.39e+05	-2.611	-2.611	-0.1842
196	5-OH-Indole-Ac	Amino acid	1	Ctrl_Untreated_1_7	Ctrl_Untreated_1	0.857142857142857	Amino acid derivatives	True	4.536e+05	-2.859	-2.859	1.152
74	Asp	Amino acid	1	Ctrl_Untreated_1_7	Ctrl_Untreated_1	1.0	Proteinogenic amino acids	True	6.805e+08	7.691	7.691	-0.2003
236	Spermidine	Amino acid	1	Ctrl_Untreated_1_7	Ctrl_Untreated_1	1.0	Polyamines	True	5.584e+07	4.084	4.084	0.2227
73	Asn	Amino acid	1	Ctrl_Untreated_1_7	Ctrl_Untreated_1	1.0	Proteinogenic amino acids	True	2.123e+08	6.011	6.011	-0.6602
243	Creatinine	Amino acid	1	Ctrl_Untreated_1_7	Ctrl_Untreated_1	1.0	Creatine degradation	True	1.368e+08	5.377	5.377	0.3748
376	Cytidine	Nucleotide	1	Ctrl_Untreated_1_7	Ctrl_Untreated_1	0.714285714285714	Pyrimidine nucleosides	True	3.27e+06	-9.74e-03	-9.74e-03	-0.7374
41	Lactate	Carbon	1	Ctrl_Untreated_1_7	Ctrl_Untreated_1	1.0	Respiratory carbon sources	True	2.619e+08	6.314	6.314	-0.1683
58	alpha-Ketoglutarate	Carbon	1	Ctrl_Untreated_1_7	Ctrl_Untreated_1	1.0	TCA cycle	True	3.315e+06	9.74e-03	9.74e-03	-6.96e-03
69	3-OH-Butyrate	Carbon	1	Ctrl_Untreated_1_7	Ctrl_Untreated_1	0.571428571428571	Ketone bodies	True	7.64e+05	-2.107	-2.107	0.877

Metabolite ID	Name	Super Pathway	Dataset	Sample ID	Group ID	Detection Fraction	Pathway	Detected	Raw Intensity	Log2 Norm Intensity	Norm Imputed	Log2 Ctrl Norm
343	Adenine	Nucleotide	1	Ctrl_Untreated_1_7	Ctrl_Untreated_1	1.0	Purine bases	True	1.596e+07	2.277	2.277	0.8201
336	Adenosine	Nucleotide	1	Ctrl_Untreated_1_7	Ctrl_Untreated_1	1.0	Purine nucleosides	True	1.056e+08	5.003	5.003	1.58
722	ADP-Ribose	Cofactor	1	Ctrl_Untreated_1_7	Ctrl_Untreated_1	1.0	Derivatives of NA, nicotinamide and NAD	True	1.92e+06	-0.778	-0.778	2.224
383	Cytosine	Nucleotide	1	Ctrl_Untreated_1_7	Ctrl_Untreated_1	0.571428571428571	Pyrimidine bases	True	9.202e+04	-5.161	-5.161	-0.3057
3	Glucosamine 6-P	Carbon	1	Ctrl_Untreated_1_7	Ctrl_Untreated_1	1.0	Aminosugar biosynthesis	True	6.975e+06	1.083	1.083	2.751
717	Nicotinamide	Cofactor	1	Ctrl_Untreated_1_7	Ctrl_Untreated_1	1.0	NAD biosynthesis	True	1.047e+08	4.991	4.991	0.476
51	PEP	Carbon	1	Ctrl_Untreated_1_7	Ctrl_Untreated_1	1.0	Glycolysis, GNG	True	1.939e+06	-0.7636	-0.7636	-1.815
237	Spermine	Amino acid	1	Ctrl_Untreated_1_7	Ctrl_Untreated_1	1.0	Polyamines	True	5.237e+05	-2.652	-2.652	-1.162
385	Uracil	Nucleotide	1	Ctrl_Untreated_1_7	Ctrl_Untreated_1	1.0	Pyrimidine bases	True	5.259e+05	-2.646	-2.646	-2.911
377	Uridine	Nucleotide	1	Ctrl_Untreated_1_7	Ctrl_Untreated_1	1.0	Pyrimidine nucleosides	True	3.37e+07	3.356	3.356	-0.6933
348	Allantoin	Nucleotide	1	Ctrl_Untreated_1_7	Ctrl_Untreated_1	1.0	Purine degradation	True	3.542e+06	0.1053	0.1053	0.2809
335	Inosine	Nucleotide	1	Ctrl_Untreated_1_7	Ctrl_Untreated_1	1.0	Purine nucleosides	True	2.62e+08	6.314	6.314	0.8152
81	Ile	Amino acid	1	Ctrl_Untreated_1_7	Ctrl_Untreated_1	1.0	Proteinogenic amino acids	True	1.134e+09	8.428	8.428	-0.2872
72	Ala	Amino acid	1	Ctrl_Untreated_1_7	Ctrl_Untreated_1	1.0	Proteinogenic amino acids	True	9.417e+08	8.16	8.16	-0.3413
79	Thr	Amino acid	1	Ctrl_Untreated_1_7	Ctrl_Untreated_1	1.0	Proteinogenic amino acids	True	5.257e+08	7.319	7.319	-0.6905
88	Tyr	Amino acid	1	Ctrl_Untreated_1_7	Ctrl_Untreated_1	1.0	Proteinogenic amino acids	True	3.461e+08	6.716	6.716	-0.8075
84	Lys	Amino acid	1	Ctrl_Untreated_1_7	Ctrl_Untreated_1	1.0	Proteinogenic amino acids	True	6.623e+08	7.652	7.652	-0.3378
86	Met	Amino acid	1	Ctrl_Untreated_1_7	Ctrl_Untreated_1	1.0	Proteinogenic amino acids	True	6.41e+08	7.605	7.605	-0.6226
61	Malate	Carbon	1	Ctrl_Untreated_1_7	Ctrl_Untreated_1	1.0	TCA cycle	True	3.264e+08	6.631	6.631	-0.5403
235	Putrescine	Amino acid	1	Ctrl_Untreated_1_7	Ctrl_Untreated_1	1.0	Polyamines	True	1.246e+07	1.92	1.92	2.411
324	2'-dU	Nucleotide	1	Ctrl_Untreated_1_7	Ctrl_Untreated_1	0.714285714285714	Deoxy-nucleosides	True	2.1e+05	-3.971	-3.971	0.4098
49	3-P-Glycerate	Carbon	1	Ctrl_Untreated_1_7	Ctrl_Untreated_1	1.0	Glycolysis, GNG	True	1.668e+07	2.341	2.341	-0.9241
189	Kynurenate	Amino acid	1	Ctrl_Untreated_1_7	Ctrl_Untreated_1	0.714285714285714	Amino acid derivatives	True	7.039e+04	-5.548	-5.548	0.2503
234	5-Me-Thioadenosine	Amino acid	1	Ctrl_Untreated_1_7	Ctrl_Untreated_1	1.0	SAM metabolism	True	1.983e+07	2.59	2.59	0.7048
59	Succinate	Carbon	1	Ctrl_Untreated_1_7	Ctrl_Untreated_1	1.0	TCA cycle	True	1.962e+07	2.575	2.575	2.274

Metabolite ID	Name	Super Pathway	Dataset	Sample ID	Group ID	Detection Fraction	Pathway	Detected	Raw Intensity	Log2 Norm Intensity	Norm Imputed	Log2 Ctrl Norm
36	Ribose	Carbon	1	Ctrl_Untreated_1_7	Ctrl_Untreated_1	1.0	Sugars and sugar alcohols	True	5.899e+05	-2.481	-2.481	-1.247
133	Ornithine	Amino acid	1	Ctrl_Untreated_1_7	Ctrl_Untreated_1	1.0	Amino acids degradation intermediates	True	1.098e+08	5.059	5.059	0.5798
313	5-Oxoproline	Amino acid	1	Ctrl_Untreated_1_7	Ctrl_Untreated_1	1.0	Glutathione derivatives	True	1.504e+07	2.191	2.191	0.104
165	N-6-Tri-Me-Lys	Amino acid	1	Ctrl_Untreated_1_7	Ctrl_Untreated_1	1.0	Amino acid derivatives	True	3.246e+07	3.302	3.302	-0.4621
380	Orotate	Nucleotide	1	Ctrl_Untreated_1_7	Ctrl_Untreated_1	0.857142857142857	Pyrimidine (UMP) biosynthesis	True	3.243e+05	-3.344	-3.344	0.2864
724	Pantothenate	Cofactor	1	Ctrl_Untreated_1_7	Ctrl_Untreated_1	1.0	Coenzyme A biosynthesis	True	2.709e+08	6.362	6.362	0.05219
150	N-Me-Gly	Amino acid	1	Ctrl_Untreated_1_7	Ctrl_Untreated_1	1.0	Amino acid derivatives	True	3.028e+06	-0.1207	-0.1207	0.1797
122	3-OH-Isobutyrate	Amino acid	1	Ctrl_Untreated_1_7	Ctrl_Untreated_1	0.857142857142857	Amino acids degradation intermediates	True	6.547e+05	-2.33	-2.33	0.8258
241	4-Acetamidobutanoate	Amino acid	1	Ctrl_Untreated_1_7	Ctrl_Untreated_1	1.0	Polyamine derivatives	True	6.976e+06	1.083	1.083	0.6423
711	alpha-Tocopherol	Cofactor	1	Ctrl_Untreated_1_7	Ctrl_Untreated_1	1.0	Cofactors	True	3.07e+06	-0.1007	-0.1007	-0.881
55	Citrate	Carbon	1	Ctrl_Untreated_1_7	Ctrl_Untreated_1	1.0	TCA cycle	True	1.61e+07	2.29	2.29	0.3408
387	3-Aminoisobutyrate	Nucleotide	1	Ctrl_Untreated_1_7	Ctrl_Untreated_1	0.857142857142857	Pyrimidine degradation	True	6.132e+05	-2.425	-2.425	-0.4109
338	Guanosine	Nucleotide	1	Ctrl_Untreated_1_7	Ctrl_Untreated_1	1.0	Purine nucleosides	True	7.143e+07	4.439	4.439	-0.6665
209	N-Ac-Ala	Amino acid	1	Ctrl_Untreated_1_7	Ctrl_Untreated_1	1.0	N-acetylated amino acids	True	9.577e+05	-1.781	-1.781	-0.5802
221	N-Ac-Met	Amino acid	1	Ctrl_Untreated_1_7	Ctrl_Untreated_1	1.0	N-acetylated amino acids	True	1.09e+07	1.727	1.727	-0.01747
228	N-Ac-Val	Amino acid	1	Ctrl_Untreated_1_7	Ctrl_Untreated_1	0.571428571428571	N-acetylated amino acids	True	7.547e+04	-5.447	-5.447	-0.0762
346	Urate	Nucleotide	1	Ctrl_Untreated_1_7	Ctrl_Untreated_1	1.0	Purine degradation	True	2.053e+06	-0.6816	-0.6816	0.3812
90	Arg	Amino acid	1	Ctrl_Untreated_1_7	Ctrl_Untreated_1	1.0	Proteinogenic amino acids	True	1.64e+09	8.96	8.96	-0.7454
60	Fumarate	Carbon	1	Ctrl_Untreated_1_7	Ctrl_Untreated_1	1.0	TCA cycle	True	4.549e+06	0.4665	0.4665	-0.5487
78	Ser	Amino acid	1	Ctrl_Untreated_1_7	Ctrl_Untreated_1	1.0	Proteinogenic amino acids	True	1.918e+08	5.864	5.864	-1.393
83	Val	Amino acid	1	Ctrl_Untreated_1_7	Ctrl_Untreated_1	1.0	Proteinogenic amino acids	True	4.638e+08	7.138	7.138	-0.7339
734	Pyridoxal	Cofactor	1	Ctrl_Untreated_1_7	Ctrl_Untreated_1	1.0	PLP biosynthesis and salvage	True	2.223e+07	2.756	2.756	0.9724

Metabolite ID	Name	Super Pathway	Dataset	Sample ID	Group ID	Detection Fraction	Pathway	Detected	Raw Intensity	Log2 Norm Intensity	Norm Imputed	Log2 Ctrl Norm
136	Urea	Amino acid	1	Ctrl_Untreated_1_7	Ctrl_Untreated_1	0.857142857142857	Amino acids degradation intermediates	True	5.463e+06	0.7307	0.7307	0.5795
67	Ribose 1-P	Carbon	1	Ctrl_Untreated_1_7	Ctrl_Untreated_1	1.0	Pentose phosphate pathway (PPP)	True	9.684e+06	1.556	1.556	0.9353
284	Carnosine	Amino acid	1	Ctrl_Untreated_1_7	Ctrl_Untreated_1	1.0	Dipeptides	True	6.607e+05	-2.317	-2.317	-0.2088
306	gamma-Glu-Cys	Amino acid	1	Ctrl_Untreated_1_7	Ctrl_Untreated_1	1.0	Glutathione biosynthesis	True	4.395e+06	0.4169	0.4169	0.7537
712	Retinol (Vit A)	Cofactor	1	Ctrl_Untreated_1_7	Ctrl_Untreated_1	1.0	Cofactors	True	6.022e+05	-2.451	-2.451	-0.01753
85	Cys	Amino acid	1	Ctrl_Untreated_1_7	Ctrl_Untreated_1	1.0	Proteinogenic amino acids	True	2.233e+08	6.084	6.084	1.57
91	Pro	Amino acid	1	Ctrl_Untreated_1_7	Ctrl_Untreated_1	1.0	Proteinogenic amino acids	True	1.991e+09	9.24	9.24	-0.1678
308	Glutathione, Reduced	Amino acid	1	Ctrl_Untreated_1_7	Ctrl_Untreated_1	1.0	Glutathione	True	7.942e+08	7.914	7.914	0.958
107	Citrulline	Amino acid	1	Ctrl_Untreated_1_7	Ctrl_Untreated_1	1.0	Amino acids biosynthesis intermediates	True	3.408e+07	3.372	3.372	0.5472
328	IMP	Nucleotide	1	Ctrl_Untreated_1_7	Ctrl_Untreated_1	0.285714285714286	Purine nucleotides	True	3.923e+05	-3.069	-3.069	1.253
706	FAD	Cofactor	1	Ctrl_Untreated_1_7	Ctrl_Untreated_1	1.0	Cofactors	True	6.581e+05	-2.323	-2.323	-0.1332
735	Pyridoxamine	Cofactor	1	Ctrl_Untreated_1_7	Ctrl_Untreated_1	1.0	PLP biosynthesis and salvage	True	2.54e+06	-0.3744	-0.3744	-0.1814
199	Serotonin	Amino acid	1	Ctrl_Untreated_1_7	Ctrl_Untreated_1	1.0	Amino acid derivatives	True	3.548e+06	0.108	0.108	-1.165
370	CMP	Nucleotide	1	Ctrl_Untreated_1_7	Ctrl_Untreated_1	1.0	Pyrimidine nucleotides	True	1.316e+07	1.999	1.999	0.1605
287	gamma-Glu-Gln	Amino acid	1	Ctrl_Untreated_1_7	Ctrl_Untreated_1	1.0	Gamma-glutamyl dipeptides	True	1.735e+06	-0.9241	-0.9241	-1.765
14	UDP-Glucuronate	Carbon	1	Ctrl_Untreated_1_7	Ctrl_Untreated_1	0.857142857142857	Polysaccharide biosynthesis	True	1.063e+06	-1.63	-1.63	0.3188
229	N-Formyl-Met	Amino acid	1	Ctrl_Untreated_1_7	Ctrl_Untreated_1	1.0	N-formylated amino acids	True	3.981e+05	-3.048	-3.048	0.7745
350	3',5'-cAMP	Nucleotide	1	Ctrl_Untreated_1_7	Ctrl_Untreated_1	1.0	Purine derivatives in signaling	True	2.927e+05	-3.491	-3.491	0.1278
371	CDP	Nucleotide	1	Ctrl_Untreated_1_7	Ctrl_Untreated_1	0.571428571428571	Pyrimidine nucleotides	True	9.181e+05	-1.842	-1.842	1.478
372	CTP	Nucleotide	1	Ctrl_Untreated_1_7	Ctrl_Untreated_1	0.571428571428571	Pyrimidine nucleotides	True	6.929e+05	-2.248	-2.248	1.405
333	GDP	Nucleotide	1	Ctrl_Untreated_1_7	Ctrl_Untreated_1	0.571428571428571	Purine nucleotides	True	2.055e+06	-0.6798	-0.6798	1.821
332	GMP	Nucleotide	1	Ctrl_Untreated_1_7	Ctrl_Untreated_1	1.0	Purine nucleotides	True	2.628e+07	2.997	2.997	2.298

Metabolite ID	Name	Super Pathway	Dataset	Sample ID	Group ID	Detection Fraction	Pathway	Detected	Raw Intensity	Log2 Norm Intensity	Norm Imputed	Log2 Ctrl Norm
373	UMP	Nucleotide	1	Ctrl_Untreated_1_7	Ctrl_Untreated_1	1.0	Pyrimidine nucleotides	True	5.745e+06	0.8032	0.8032	1.858
389	3'-CMP	Nucleotide	1	Ctrl_Untreated_1_7	Ctrl_Untreated_1	1.0	Pyrimidine derivatives in signaling	True	1.484e+06	-1.149	-1.149	-0.09812
330	ADP	Nucleotide	1	Ctrl_Untreated_1_7	Ctrl_Untreated_1	0.714285714285714	Purine nucleotides	True	9.37e+06	1.509	1.509	1.805
342	Hypoxanthine	Nucleotide	1	Ctrl_Untreated_1_7	Ctrl_Untreated_1	1.0	Purine bases	True	3.5e+07	3.41	3.41	-0.5289
736	Pyridoxamine-P	Cofactor	1	Ctrl_Untreated_1_7	Ctrl_Untreated_1	1.0	PLP biosynthesis and salvage	True	4.39e+05	-2.907	-2.907	0.07126
148	Betaine	Amino acid	1	Ctrl_Untreated_1_7	Ctrl_Untreated_1	1.0	Amino acid derivatives	True	1.3e+08	5.303	5.303	0.4974
344	Xanthine	Nucleotide	1	Ctrl_Untreated_1_7	Ctrl_Untreated_1	1.0	Purine bases	True	4.078e+06	0.3086	0.3086	-0.5989
386	3-Ureidopropionate	Nucleotide	1	Ctrl_Untreated_1_7	Ctrl_Untreated_1	1.0	Pyrimidine degradation	True	3.505e+06	0.09045	0.09045	1.01
149	DiMe-Gly	Amino acid	1	Ctrl_Untreated_1_7	Ctrl_Untreated_1	1.0	Amino acid derivatives	True	3.485e+06	0.08225	0.08225	0.6025
703	NAD+	Cofactor	1	Ctrl_Untreated_1_7	Ctrl_Untreated_1	1.0	Cofactors	True	2.084e+07	2.662	2.662	-0.07385
709	Pyridoxal-P	Cofactor	1	Ctrl_Untreated_1_7	Ctrl_Untreated_1	1.0	Cofactors	True	3.226e+05	-3.351	-3.351	-1.043
731	Thiamin (Vitamin B1)	Cofactor	1	Ctrl_Untreated_1_7	Ctrl_Untreated_1	1.0	TPP biosynthesis	True	7.792e+06	1.243	1.243	0.4952
374	UDP	Nucleotide	1	Ctrl_Untreated_1_7	Ctrl_Untreated_1	0.714285714285714	Pyrimidine nucleotides	True	1.028e+07	1.643	1.643	3.197
102	2-Aminoadipate	Amino acid	1	Ctrl_Untreated_1_7	Ctrl_Untreated_1	1.0	Amino acids biosynthesis intermediates	True	7.225e+05	-2.188	-2.188	-0.07852
45	Fructose-6-P	Carbon	1	Ctrl_Untreated_1_7	Ctrl_Untreated_1	1.0	Glycolysis, GNG	True	1.817e+06	-0.8573	-0.8573	-0.9326
320	TMP	Nucleotide	1	Ctrl_Untreated_1_7	Ctrl_Untreated_1	0.571428571428571	Deoxy-nucleotides	True	4.798e+05	-2.779	-2.779	2.013
341	XMP	Nucleotide	1	Ctrl_Untreated_1_7	Ctrl_Untreated_1	0.857142857142857	IMP conversion to AMP & GMP	True	4.029e+04	-6.353	-6.353	-1.778
120	beta-OH-Isovalerate	Amino acid	1	Ctrl_Untreated_1_7	Ctrl_Untreated_1	0.857142857142857	Amino acids degradation intermediates	True	9.018e+05	-1.868	-1.868	1.297
322	2'-dI	Nucleotide	1	Ctrl_Untreated_1_7	Ctrl_Untreated_1	0.857142857142857	Deoxy-nucleosides	True	5.969e+05	-2.464	-2.464	1.662
4	GlcNAc 6-P	Carbon	1	Ctrl_Untreated_1_7	Ctrl_Untreated_1	1.0	Aminosugar biosynthesis	True	1.729e+06	-0.9293	-0.9293	-1.258
337	Xanthosine	Nucleotide	1	Ctrl_Untreated_1_7	Ctrl_Untreated_1	1.0	Purine nucleosides	True	8.069e+05	-2.029	-2.029	1.046
188	Kynurenine	Amino acid	1	Ctrl_Untreated_1_7	Ctrl_Untreated_1	1.0	Amino acid derivatives	True	1.932e+06	-0.7687	-0.7687	-0.0771

Metabolite ID	Name	Super Pathway	Dataset	Sample ID	Group ID	Detection Fraction	Pathway	Detected	Raw Intensity	Log2 Norm Intensity	Norm Imputed	Log2 Ctrl Norm
63	6-P-Gluconate	Carbon	1	Ctrl_Untreated_1_7	Ctrl_Untreated_1	1.0	Pentose phosphate pathway (PPP)	True	4.595e+07	3.803	3.803	1.769
40	Glucuronate	Carbon	1	Ctrl_Untreated_1_7	Ctrl_Untreated_1	1.0	Sugars and sugar alcohols	True	1.319e+06	-1.32	-1.32	0.2193
108	Argininosuccinate	Amino acid	1	Ctrl_Untreated_1_7	Ctrl_Untreated_1	1.0	Amino acids biosynthesis intermediates	True	2.183e+06	-0.5925	-0.5925	-0.3663
710	Carnitine	Cofactor	1	Ctrl_Untreated_1_7	Ctrl_Untreated_1	1.0	Cofactors	True	2.27e+08	6.107	6.107	0.1299
725	P-Pantetheine	Cofactor	1	Ctrl_Untreated_1_7	Ctrl_Untreated_1	1.0	Coenzyme A biosynthesis	True	1.116e+05	-4.883	-4.883	-1.179
48	DHAP	Carbon	1	Ctrl_Untreated_1_7	Ctrl_Untreated_1	1.0	Glycolysis, GNG	True	9.877e+06	1.585	1.585	-1.512
17	Maltose	Carbon	1	Ctrl_Untreated_1_7	Ctrl_Untreated_1	1.0	Glycogen degradation	True	3.538e+05	-3.218	-3.218	-1.925
359	N1-Me-Adenosine	Nucleotide	1	Ctrl_Untreated_1_7	Ctrl_Untreated_1	0.857142857142857	Purine derivatives in RNAs	True	1.619e+06	-1.024	-1.024	0.01372
159	3-Me-His	Amino acid	1	Ctrl_Untreated_1_7	Ctrl_Untreated_1	1.0	Amino acid derivatives	True	2.989e+05	-3.462	-3.462	0.7738
155	4-Guanidinobutanoate	Amino acid	1	Ctrl_Untreated_1_7	Ctrl_Untreated_1	1.0	Amino acid derivatives	True	1.509e+06	-1.126	-1.126	0.3084
164	5-OH-Lys	Amino acid	1	Ctrl_Untreated_1_7	Ctrl_Untreated_1	1.0	Amino acid derivatives	True	1.558e+06	-1.08	-1.08	0.6907
357	Adenosine-3',5'-PP	Nucleotide	1	Ctrl_Untreated_1_7	Ctrl_Untreated_1	0.857142857142857	Purine byproducts of metabolic processes	False			-5.09	-1.612
104	Cystathionine	Amino acid	1	Ctrl_Untreated_1_7	Ctrl_Untreated_1	1.0	Amino acids biosynthesis intermediates	True	4.05e+07	3.621	3.621	1.411
113	Imidazole Lactate	Amino acid	1	Ctrl_Untreated_1_7	Ctrl_Untreated_1	0.857142857142857	Amino acids degradation intermediates	True	4.994e+05	-2.721	-2.721	0.9556
215	N-Ac-Glu	Amino acid	1	Ctrl_Untreated_1_7	Ctrl_Untreated_1	1.0	N-acetylated amino acids	True	1.129e+07	1.777	1.777	0.03394
310	S-Lactoyl-Glutathione	Amino acid	1	Ctrl_Untreated_1_7	Ctrl_Untreated_1	0.857142857142857	Glutathione derivatives	False			-3.157	-3.514
5	GlcNAc 1-P	Carbon	1	Ctrl_Untreated_1_7	Ctrl_Untreated_1	1.0	Aminosugar biosynthesis	True	5.998e+05	-2.456	-2.456	-0.5456
34	Ribitol	Carbon	1	Ctrl_Untreated_1_7	Ctrl_Untreated_1	1.0	Sugars and sugar alcohols	True	2.855e+05	-3.528	-3.528	-0.09757
10	UDP-Galactose	Carbon	1	Ctrl_Untreated_1_7	Ctrl_Untreated_1	0.857142857142857	Polysaccharide biosynthesis	True	1.453e+06	-1.18	-1.18	-0.5533
13	Guanosine 5'-PP-Fucose	Carbon	1	Ctrl_Untreated_1_7	Ctrl_Untreated_1	1.0	Polysaccharide biosynthesis	True	9.314e+05	-1.822	-1.822	0.4777

Metabolite ID	Name	Super Pathway	Dataset	Sample ID	Group ID	Detection Fraction	Pathway	Detected	Raw Intensity	Log2 Norm Intensity	Norm Imputed	Log2 Ctrl Norm
19	Maltotetraose	Carbon	1	Ctrl_Untreated_1_7	Ctrl_Untreated_1	1.0	Glycogen degradation	True	3.484e+05	-3.24	-3.24	-3.197
233	SAM	Amino acid	1	Ctrl_Untreated_1_7	Ctrl_Untreated_1	1.0	SAM metabolism	True	5.902e+06	0.8422	0.8422	0.9985
129	5-Aminovalerate	Amino acid	1	Ctrl_Untreated_1_7	Ctrl_Untreated_1	1.0	Amino acids degradation intermediates	True	8.07e+06	1.293	1.293	0.2917
741	5-Me-THF	Cofactor	1	Ctrl_Untreated_1_7	Ctrl_Untreated_1	0.714285714285714	Folate metabolism	True	2.358e+05	-3.804	-3.804	0.9542
198	Indolelactate	Amino acid	1	Ctrl_Untreated_1_7	Ctrl_Untreated_1	0.857142857142857	Amino acid derivatives	True	3.529e+05	-3.222	-3.222	0.5653
254	Gly-Val	Amino acid	1	Ctrl_Untreated_1_7	Ctrl_Untreated_1	1.0	Dipeptides	True	1.07e+07	1.701	1.701	0.5895
291	gamma-Glu-Leu	Amino acid	1	Ctrl_Untreated_1_7	Ctrl_Untreated_1	1.0	Gamma-glutamyl dipeptides	True	5.804e+06	0.818	0.818	1.307
173	Met Sulfoxide	Amino acid	1	Ctrl_Untreated_1_7	Ctrl_Untreated_1	1.0	Amino acid derivatives	True	1.082e+07	1.717	1.717	-0.8508
43	Glucose	Carbon	1	Ctrl_Untreated_1_7	Ctrl_Untreated_1	1.0	Glycolysis, GNG	True	1.012e+08	4.942	4.942	-0.1097
185	Phenyllactate	Amino acid	1	Ctrl_Untreated_1_7	Ctrl_Untreated_1	0.285714285714286	Amino acid derivatives	True	6.206e+04	-5.729	-5.729	0.08243
156	Homo-Arg	Amino acid	1	Ctrl_Untreated_1_7	Ctrl_Untreated_1	1.0	Amino acid derivatives	True	1.136e+07	1.787	1.787	0.6069
135	Homocitrulline	Amino acid	1	Ctrl_Untreated_1_7	Ctrl_Untreated_1	1.0	Amino acids degradation intermediates	True	1.546e+06	-1.091	-1.091	0.6118
719	Nicotinamide MN	Cofactor	1	Ctrl_Untreated_1_7	Ctrl_Untreated_1	1.0	NAD biosynthesis	True	8.397e+05	-1.971	-1.971	-2.157
212	N-Ac-Asp	Amino acid	1	Ctrl_Untreated_1_7	Ctrl_Untreated_1	1.0	N-acetylated amino acids	True	3.752e+06	0.1886	0.1886	-0.5087
720	1-Me-Nicotinamide	Cofactor	1	Ctrl_Untreated_1_7	Ctrl_Untreated_1	1.0	Derivatives of NA, nicotinamide and NAD	True	5.812e+08	7.464	7.464	-0.4269
216	N-Ac-Gly	Amino acid	1	Ctrl_Untreated_1_7	Ctrl_Untreated_1	0.714285714285714	N-acetylated amino acids	True	6.403e+05	-2.362	-2.362	1.178
70	Creatine	Carbon	1	Ctrl_Untreated_1_7	Ctrl_Untreated_1	1.0	Creatine energy storage	True	2.519e+09	9.58	9.58	0.1626
26	Galactonate	Carbon	1	Ctrl_Untreated_1_7	Ctrl_Untreated_1	0.857142857142857	Sugars and sugar alcohols	True	4.966e+06	0.593	0.593	1.398
309	Glutathione, Oxidized	Amino acid	1	Ctrl_Untreated_1_7	Ctrl_Untreated_1	1.0	Glutathione	True	8.411e+06	1.353	1.353	0.1604
35	Ribonate	Carbon	1	Ctrl_Untreated_1_7	Ctrl_Untreated_1	1.0	Sugars and sugar alcohols	True	1.754e+06	-0.908	-0.908	-0.3808
160	1-Me-His	Amino acid	1	Ctrl_Untreated_1_7	Ctrl_Untreated_1	1.0	Amino acid derivatives	True	5.757e+07	4.128	4.128	0.687

Metabolite ID	Name	Super Pathway	Dataset	Sample ID	Group ID	Detection Fraction	Pathway	Detected	Raw Intensity	Log2 Norm Intensity	Norm Imputed	Log2 Ctrl Norm
44	Glucose 6-P	Carbon	1	Ctrl_Untreated_1_7	Ctrl_Untreated_1	0.857142857142857	Glycolysis, GNG	True	2.421e+06	-0.4434	-0.4434	1.033
704	NADH	Cofactor	1	Ctrl_Untreated_1_7	Ctrl_Untreated_1	1.0	Cofactors	True	4.319e+05	-2.93	-2.93	-2.284
275	Thr-Phe	Amino acid	1	Ctrl_Untreated_1_7	Ctrl_Untreated_1	0.857142857142857	Dipeptides	True	8.503e+06	1.369	1.369	3.022
738	Pyridoxate	Cofactor	1	Ctrl_Untreated_1_7	Ctrl_Untreated_1	1.0	PLP biosynthesis and salvage	True	2.89e+05	-3.51	-3.51	-0.1456
177	3-(4-OH-Phenyl)Lactate	Amino acid	1	Ctrl_Untreated_1_7	Ctrl_Untreated_1	1.0	Amino acid derivatives	True	8.81e+05	-1.902	-1.902	0.5634
206	Trans-4-OH-Pro	Amino acid	1	Ctrl_Untreated_1_7	Ctrl_Untreated_1	1.0	Amino acid derivatives	True	1.411e+08	5.422	5.422	0.2664
329	AMP	Nucleotide	1	Ctrl_Untreated_1_7	Ctrl_Untreated_1	1.0	Purine nucleotides	True	1.097e+08	5.058	5.058	1.423
11	UDP-Glucose	Carbon	1	Ctrl_Untreated_1_7	Ctrl_Untreated_1	1.0	Polysaccharide biosynthesis	True	5.488e+06	0.7373	0.7373	0.8425
158	4-Imidazole-Ac	Amino acid	1	Ctrl_Untreated_1_7	Ctrl_Untreated_1	1.0	Amino acid derivatives	True	1.004e+06	-1.714	-1.714	0.4326
111	1-Me-Imidazole-Ac	Amino acid	1	Ctrl_Untreated_1_7	Ctrl_Untreated_1	1.0	Amino acids degradation intermediates	True	1.183e+06	-1.476	-1.476	0.4573
345	Guanine	Nucleotide	1	Ctrl_Untreated_1_7	Ctrl_Untreated_1	1.0	Purine bases	True	2.968e+07	3.172	3.172	-1.814
22	N-Ac-Neuraminate	Carbon	1	Ctrl_Untreated_1_7	Ctrl_Untreated_1	1.0	Aminosugar derivatives	True	2.68e+06	-0.297	-0.297	-0.1616
721	N'-Methylnicotinate	Cofactor	1	Ctrl_Untreated_1_7	Ctrl_Untreated_1	1.0	Derivatives of NA, nicotinamide and NAD	True	1.202e+06	-1.454	-1.454	-0.446
183	Phenol Sulfate	Amino acid	1	Ctrl_Untreated_1_7	Ctrl_Untreated_1	0.857142857142857	Amino acid derivatives	True	4.849e+05	-2.763	-2.763	1.877
718	Nicotinamide Riboside	Cofactor	1	Ctrl_Untreated_1_7	Ctrl_Untreated_1	1.0	NAD biosynthesis	True	6.911e+05	-2.252	-2.252	-3.842
297	gamma-Glu-Thr	Amino acid	1	Ctrl_Untreated_1_7	Ctrl_Untreated_1	1.0	Gamma-glutamyl dipeptides	True	1.375e+07	2.062	2.062	1.422
295	gamma-Glu-Phe	Amino acid	1	Ctrl_Untreated_1_7	Ctrl_Untreated_1	0.428571428571429	Gamma-glutamyl dipeptides	True	6.417e+04	-5.681	-5.681	0.104
347	Allantoic Acid	Nucleotide	1	Ctrl_Untreated_1_7	Ctrl_Untreated_1	0.571428571428571	Purine degradation	True	1.265e+05	-4.702	-4.702	0.9378
399	Pseudouridine	Nucleotide	1	Ctrl_Untreated_1_7	Ctrl_Untreated_1	1.0	Pyrimidine derivatives in RNAs	True	9.927e+05	-1.73	-1.73	0.3003
375	UTP	Nucleotide	1	Ctrl_Untreated_1_7	Ctrl_Untreated_1	0.571428571428571	Pyrimidine nucleotides	True	7.593e+06	1.206	1.206	3.182
144	Glu, gamma-Me Ester	Amino acid	1	Ctrl_Untreated_1_7	Ctrl_Untreated_1	1.0	Amino acid derivatives	True	1.426e+07	2.115	2.115	1.537

Metabolite ID	Name	Super Pathway	Dataset	Sample ID	Group ID	Detection Fraction	Pathway	Detected	Raw Intensity	Log2 Norm Intensity	Norm Imputed	Log2 Ctrl Norm
292	gamma-Glu-epsilon-Lysine	Amino acid	1	Ctrl_Untreated_1_7	Ctrl_Untreated_1	1.0	Gamma-glutamyl dipeptides	True	3.33e+06	0.01662	0.01662	0.208
225	N-Ac-Thr	Amino acid	1	Ctrl_Untreated_1_7	Ctrl_Untreated_1	1.0	N-acetylated amino acids	True	1.081e+06	-1.607	-1.607	-0.1043
211	N-Ac-Asn	Amino acid	1	Ctrl_Untreated_1_7	Ctrl_Untreated_1	1.0	N-acetylated amino acids	True	6.193e+05	-2.41	-2.41	0.2748
151	Phenylacetylglycine	Amino acid	1	Ctrl_Untreated_1_7	Ctrl_Untreated_1	1.0	Amino acid derivatives	True	1.624e+06	-1.02	-1.02	-0.2329
217	N-Ac-His	Amino acid	1	Ctrl_Untreated_1_7	Ctrl_Untreated_1	1.0	N-acetylated amino acids	True	2.089e+05	-3.978	-3.978	-0.4935
288	gamma-Glu-Gly	Amino acid	1	Ctrl_Untreated_1_7	Ctrl_Untreated_1	0.571428571428571	Gamma-glutamyl dipeptides	True	2.374e+06	-0.472	-0.472	1.353
222	N-Ac-Phe	Amino acid	1	Ctrl_Untreated_1_7	Ctrl_Untreated_1	0.571428571428571	N-acetylated amino acids	True	4.636e+04	-6.15	-6.15	0.1225
71	Creatine-P	Carbon	1	Ctrl_Untreated_1_7	Ctrl_Untreated_1	1.0	Creatine energy storage	True	1.774e+05	-4.214	-4.214	0.04114
210	N-Ac-Arg	Amino acid	1	Ctrl_Untreated_1_7	Ctrl_Untreated_1	1.0	N-acetylated amino acids	True	2.022e+05	-4.025	-4.025	-1.35
218	N-Ac-Ile	Amino acid	1	Ctrl_Untreated_1_7	Ctrl_Untreated_1	0.428571428571429	N-acetylated amino acids	True	8.02e+04	-5.359	-5.359	0.6066
251	Gly-Leu	Amino acid	1	Ctrl_Untreated_1_7	Ctrl_Untreated_1	1.0	Dipeptides	True	2.101e+07	2.674	2.674	1.035
290	gamma-Glu-Ile	Amino acid	1	Ctrl_Untreated_1_7	Ctrl_Untreated_1	1.0	Gamma-glutamyl dipeptides	True	2.424e+06	-0.4416	-0.4416	0.897
316	Ophthalmate	Amino acid	1	Ctrl_Untreated_1_7	Ctrl_Untreated_1	1.0	Oxidative stress markers	True	2.854e+07	3.116	3.116	0.7733
125	Isovaleryl-Gly	Amino acid	1	Ctrl_Untreated_1_7	Ctrl_Untreated_1	0.857142857142857	Amino acids degradation intermediates	True	9.549e+04	-5.108	-5.108	0.302
368	7-Me-Guanine	Nucleotide	1	Ctrl_Untreated_1_7	Ctrl_Untreated_1	1.0	Purine derivatives in RNAs	True	7.874e+05	-2.064	-2.064	0.1261
208	Pro-OH-Pro	Amino acid	1	Ctrl_Untreated_1_7	Ctrl_Untreated_1	1.0	Amino acid derivatives	True	5.139e+07	3.964	3.964	0.6351
366	N2,N2-DiMe-Guanosine	Nucleotide	1	Ctrl_Untreated_1_7	Ctrl_Untreated_1	0.857142857142857	Purine derivatives in RNAs	True	2.82e+05	-3.546	-3.546	0.5681
352	3'-AMP	Nucleotide	1	Ctrl_Untreated_1_7	Ctrl_Untreated_1	1.0	Purine derivatives in signaling	True	5.615e+05	-2.552	-2.552	-1.065
363	N6-Carbamoyl-Thr-Adenosine	Nucleotide	1	Ctrl_Untreated_1_7	Ctrl_Untreated_1	0.857142857142857	Purine derivatives in RNAs	True	1.763e+05	-4.223	-4.223	0.4627
314	Cys-Glutathione Disulfide	Amino acid	1	Ctrl_Untreated_1_7	Ctrl_Untreated_1	1.0	Oxidative stress markers	True	3.358e+06	0.02836	0.02836	3.312
382	Orotidine	Nucleotide	1	Ctrl_Untreated_1_7	Ctrl_Untreated_1	0.428571428571429	Pyrimidine (UMP) biosynthesis	True	9.189e+05	-1.841	-1.841	2.451

Metabolite ID	Name	Super Pathway	Dataset	Sample ID	Group ID	Detection Fraction	Pathway	Detected	Raw Intensity	Log2 Norm Intensity	Norm Imputed	Log2 Ctrl Norm
307	Cys-Gly	Amino acid	1	Ctrl_Untreated_1_7	Ctrl_Untreated_1	1.0	Glutathione biosynthesis	True	2.786e+07	3.081	3.081	1.474
64	Sedoheptulose-7-P	Carbon	1	Ctrl_Untreated_1_7	Ctrl_Untreated_1	1.0	Pentose phosphate pathway (PPP)	True	6.602e+06	1.004	1.004	0.8498
142	N-Ac-Asp-Glu	Amino acid	1	Ctrl_Untreated_1_7	Ctrl_Untreated_1	1.0	Amino acid derivatives	True	1.411e+06	-1.222	-1.222	-0.07688
708	Thiamin-PP	Cofactor	1	Ctrl_Untreated_1_7	Ctrl_Untreated_1	0.571428571428571	Cofactors	True	9.007e+04	-5.192	-5.192	0.7917
182	P-Cresol Sulfate	Amino acid	1	Ctrl_Untreated_1_7	Ctrl_Untreated_1	0.857142857142857	Amino acid derivatives	True	4.842e+05	-2.765	-2.765	0.4308
250	Gly-Ile	Amino acid	1	Ctrl_Untreated_1_7	Ctrl_Untreated_1	1.0	Dipeptides	True	2.797e+06	-0.2351	-0.2351	1.082
286	gamma-Glu-Glu	Amino acid	1	Ctrl_Untreated_1_7	Ctrl_Untreated_1	1.0	Gamma-glutamyl dipeptides	True	1.445e+06	-1.188	-1.188	-1.206
739	Deoxycarnitine	Cofactor	1	Ctrl_Untreated_1_7	Ctrl_Untreated_1	1.0	Carnitine biosynthesis	True	4.296e+07	3.706	3.706	-0.04553
203	DiMe-Arg	Amino acid	1	Ctrl_Untreated_1_7	Ctrl_Untreated_1	1.0	Amino acid derivatives	True	4.945e+07	3.909	3.909	-1.724
351	2'-AMP	Nucleotide	1	Ctrl_Untreated_1_7	Ctrl_Untreated_1	1.0	Purine derivatives in signaling	True	3.808e+05	-3.112	-3.112	-0.8433
8	Cytidine 5'-P-N-Ac-Neuraminate	Carbon	1	Ctrl_Untreated_1_7	Ctrl_Untreated_1	1.0	Aminosugar biosynthesis	True	8.292e+05	-1.989	-1.989	-0.2438
285	gamma-Glu-Ala	Amino acid	1	Ctrl_Untreated_1_7	Ctrl_Untreated_1	0.857142857142857	Gamma-glutamyl dipeptides	True	3.053e+05	-3.431	-3.431	-0.1922
224	N-Ac-Ser	Amino acid	1	Ctrl_Untreated_1_7	Ctrl_Untreated_1	1.0	N-acetylated amino acids	True	1.39e+07	2.078	2.078	0.1984
244	Ala-Leu	Amino acid	1	Ctrl_Untreated_1_7	Ctrl_Untreated_1	1.0	Dipeptides	True	4.085e+07	3.633	3.633	2.495
207	N-Me-Pro	Amino acid	1	Ctrl_Untreated_1_7	Ctrl_Untreated_1	1.0	Amino acid derivatives	True	2.86e+06	-0.2031	-0.2031	0.7269
171	Cys Sulfinic Acid	Amino acid	1	Ctrl_Untreated_1_7	Ctrl_Untreated_1	0.857142857142857	Amino acid derivatives	False			-5.211	-1.913
181	O-Me-Tyr	Amino acid	1	Ctrl_Untreated_1_7	Ctrl_Untreated_1	0.857142857142857	Amino acid derivatives	True	5.909e+05	-2.478	-2.478	0.8916
240	N-Ac-Putrescine	Amino acid	1	Ctrl_Untreated_1_7	Ctrl_Untreated_1	1.0	Polyamine derivatives	True	2.713e+06	-0.2794	-0.2794	2.084
176	S-Me-Met	Amino acid	1	Ctrl_Untreated_1_7	Ctrl_Untreated_1	0.857142857142857	Amino acid derivatives	True	1.523e+06	-1.112	-1.112	0.7297
339	AICAR	Nucleotide	1	Ctrl_Untreated_1_7	Ctrl_Untreated_1	0.571428571428571	IMP biosynthesis	True	1.809e+05	-4.186	-4.186	0.1151
141	gamma-Carboxy-Glu	Amino acid	1	Ctrl_Untreated_1_7	Ctrl_Untreated_1	1.0	Amino acid derivatives	True	2.034e+06	-0.6947	-0.6947	0.09034

Metabolite ID	Name	Super Pathway	Dataset	Sample ID	Group ID	Detection Fraction	Pathway	Detected	Raw Intensity	Log2 Norm Intensity	Norm Imputed	Log2 Ctrl Norm
392	3'-UMP	Nucleotide	1	Ctrl_Untreated_1_7	Ctrl_Untreated_1	0.571428571428571	Pyrimidine derivatives in signaling	True	8.165e+05	-2.012	-2.012	2.022
355	3'-GMP	Nucleotide	1	Ctrl_Untreated_1_7	Ctrl_Untreated_1	0.857142857142857	Purine derivatives in signaling	True	4.837e+05	-2.767	-2.767	1.17
282	Val-Leu	Amino acid	1	Ctrl_Untreated_1_7	Ctrl_Untreated_1	1.0	Dipeptides	True	5.51e+07	4.065	4.065	3.093
140	Carboxyethyl-GABA	Amino acid	1	Ctrl_Untreated_1_7	Ctrl_Untreated_1	1.0	Amino acid derivatives	True	6.582e+05	-2.323	-2.323	-0.8914
258	Ile-Gly	Amino acid	1	Ctrl_Untreated_1_7	Ctrl_Untreated_1	1.0	Dipeptides	True	6.505e+07	4.304	4.304	0.9923
260	Leu-Ala	Amino acid	1	Ctrl_Untreated_1_7	Ctrl_Untreated_1	1.0	Dipeptides	True	4.688e+07	3.832	3.832	2.531
265	Lys-Leu	Amino acid	1	Ctrl_Untreated_1_7	Ctrl_Untreated_1	0.857142857142857	Dipeptides	True	3.76e+06	0.1916	0.1916	2.803
263	Leu-Gly	Amino acid	1	Ctrl_Untreated_1_7	Ctrl_Untreated_1	1.0	Dipeptides	True	8.873e+07	4.752	4.752	1.731
281	Val-Gly	Amino acid	1	Ctrl_Untreated_1_7	Ctrl_Untreated_1	1.0	Dipeptides	True	1.488e+08	5.498	5.498	0.9792
270	Pro-Gly	Amino acid	1	Ctrl_Untreated_1_7	Ctrl_Untreated_1	1.0	Dipeptides	True	1.082e+07	1.716	1.716	0.9697
114	Imidazole Propionate	Amino acid	1	Ctrl_Untreated_1_7	Ctrl_Untreated_1	1.0	Amino acids degradation intermediates	True	1.348e+06	-1.289	-1.289	1.022
267	Phe-Gly	Amino acid	1	Ctrl_Untreated_1_7	Ctrl_Untreated_1	1.0	Dipeptides	True	9.791e+07	4.894	4.894	0.8005
266	Phe-Ala	Amino acid	1	Ctrl_Untreated_1_7	Ctrl_Untreated_1	1.0	Dipeptides	True	3.343e+07	3.344	3.344	2.267
278	Tyr-Gly	Amino acid	1	Ctrl_Untreated_1_7	Ctrl_Untreated_1	1.0	Dipeptides	True	3.015e+07	3.195	3.195	1.013
255	His-Ala	Amino acid	1	Ctrl_Untreated_1_7	Ctrl_Untreated_1	1.0	Dipeptides	True	2.679e+06	-0.2976	-0.2976	1.04
280	Val-Gln	Amino acid	1	Ctrl_Untreated_1_7	Ctrl_Untreated_1	1.0	Dipeptides	True	4.673e+07	3.827	3.827	2.209
143	S-1-Pyrroline-5-Carboxylate	Amino acid	1	Ctrl_Untreated_1_7	Ctrl_Untreated_1	0.857142857142857	Amino acid derivatives	True	1.186e+06	-1.473	-1.473	0.5956
232	SAH	Amino acid	1	Ctrl_Untreated_1_7	Ctrl_Untreated_1	1.0	SAM metabolism	True	2.2e+06	-0.5818	-0.5818	-0.09643
20	Erythronate	Carbon	1	Ctrl_Untreated_1_7	Ctrl_Untreated_1	1.0	Aminosugar derivatives	True	5.639e+07	4.098	4.098	0.3571
248	Gln-Leu	Amino acid	1	Ctrl_Untreated_1_7	Ctrl_Untreated_1	1.0	Dipeptides	True	9.834e+06	1.579	1.579	2.484
276	Trp-Gly	Amino acid	1	Ctrl_Untreated_1_7	Ctrl_Untreated_1	1.0	Dipeptides	True	3.066e+06	-0.1027	-0.1027	0.9756
205	N-delta-Ac-Ornithine	Amino acid	1	Ctrl_Untreated_1_7	Ctrl_Untreated_1	1.0	Amino acid derivatives	True	2.358e+06	-0.4815	-0.4815	0.6395
163	Formimino-Glu	Amino acid	1	Ctrl_Untreated_1_7	Ctrl_Untreated_1	1.0	Amino acid derivatives	True	1.603e+06	-1.039	-1.039	0.2011
204	N-Me-Arg	Amino acid	1	Ctrl_Untreated_1_7	Ctrl_Untreated_1	1.0	Amino acid derivatives	True	4.693e+06	0.5113	0.5113	-2.076
242	Guanidino-Ac	Amino acid	1	Ctrl_Untreated_1_7	Ctrl_Untreated_1	0.857142857142857	Creatine biosynthesis	True	2.708e+05	-3.604	-3.604	0.2768

Metabolite ID	Name	Super Pathway	Dataset	Sample ID	Group ID	Detection Fraction	Pathway	Detected	Raw Intensity	Log2 Norm Intensity	Norm Imputed	Log2 Ctrl Norm
300	gamma-Glu-Val	Amino acid	1	Ctrl_Untreated_1_7	Ctrl_Untreated_1	0.857142857142857	Gamma-glutamyl dipeptides	True	8.095e+06	1.298	1.298	0.6771
53	Ac-CoA	Carbon	1	Ctrl_Untreated_1_7	Ctrl_Untreated_1	0.285714285714286	Acetyl-CoA	True	8.445e+04	-5.285	-5.285	1.478
18	Maltotriose	Carbon	1	Ctrl_Untreated_1_7	Ctrl_Untreated_1	1.0	Glycogen degradation	True	1.244e+06	-1.404	-1.404	-1.246
294	gamma-Glu-Met	Amino acid	1	Ctrl_Untreated_1_7	Ctrl_Untreated_1	0.714285714285714	Gamma-glutamyl dipeptides	False			-4.503	-1.021
174	Met Sulfone	Amino acid	1	Ctrl_Untreated_1_7	Ctrl_Untreated_1	1.0	Amino acid derivatives	True	4.723e+05	-2.801	-2.801	0.2773
175	N-Ac-Met Sulfoxide	Amino acid	1	Ctrl_Untreated_1_7	Ctrl_Untreated_1	1.0	Amino acid derivatives	True	1.568e+06	-1.07	-1.07	-1.047
25	Mannitol/Sorbitol	Carbon	1	Ctrl_Untreated_1_7	Ctrl_Untreated_1	1.0	Sugars and sugar alcohols	True	1.592e+07	2.274	2.274	0.04914
6	UDP-GlcNAc	Carbon	1	Ctrl_Untreated_1_7	Ctrl_Untreated_1	0.428571428571429	Aminosugar biosynthesis	True	6.221e+05	-2.404	-2.404	0.7038
145	Pyro-Gln	Amino acid	1	Ctrl_Untreated_1_7	Ctrl_Untreated_1	1.0	Amino acid derivatives	True	6.04e+06	0.8755	0.8755	0.03573
705	Coenzyme A	Cofactor	1	Ctrl_Untreated_1_7	Ctrl_Untreated_1	0.714285714285714	Cofactors	True	1.729e+06	-0.9292	-0.9292	3.03
319	2'-dAMP	Nucleotide	1	Ctrl_Untreated_1_7	Ctrl_Untreated_1	0.571428571428571	Deoxy-nucleotides	True	2.928e+05	-3.491	-3.491	0.189
119	alpha-OH-Isovalerate	Amino acid	1	Ctrl_Untreated_1_7	Ctrl_Untreated_1	0.714285714285714	Amino acids degradation intermediates	True	8.223e+05	-2.001	-2.001	1.297
46	Fructose 1,6-PP / Glucose 1,6-PP / Inositol-1,4-PP / Inositol-1,3-PP	Carbon	1	Ctrl_Untreated_1_7	Ctrl_Untreated_1	1.0	Glycolysis, GNG	True	1.257e+07	1.933	1.933	-2.898
137	1-Me-Guanidine	Amino acid	1	Ctrl_Untreated_1_7	Ctrl_Untreated_1	0.857142857142857	Amino acids degradation intermediates	True	1.005e+05	-5.034	-5.034	0.1191
23	N-GlcNAc-Asn	Carbon	1	Ctrl_Untreated_1_7	Ctrl_Untreated_1	1.0	Aminosugar derivatives	True	3.028e+06	-0.1208	-0.1208	-0.8337
262	Leu-Gln	Amino acid	1	Ctrl_Untreated_1_7	Ctrl_Untreated_1	1.0	Dipeptides	True	5.015e+07	3.929	3.929	2.633
24	Fructose	Carbon	1	Ctrl_Untreated_1_7	Ctrl_Untreated_1	1.0	Sugars and sugar alcohols	True	1.851e+07	2.491	2.491	0.542
197	C-Glycosyl-Trp	Amino acid	1	Ctrl_Untreated_1_7	Ctrl_Untreated_1	1.0	Amino acid derivatives	True	1.874e+06	-0.8131	-0.8131	-0.514
33	Arabitol/Xylitol	Carbon	1	Ctrl_Untreated_1_7	Ctrl_Untreated_1	1.0	Sugars and sugar alcohols	True	4.308e+06	0.388	0.388	2.248

Metabolite ID	Name	Super Pathway	Dataset	Sample ID	Group ID	Detection Fraction	Pathway	Detected	Raw Intensity	Log2 Norm Intensity	Norm Imputed	Log2 Ctrl Norm
128	N2-Ac-Lys/N6-Ac-Lys	Amino acid	1	Ctrl_Untreated_1_7	Ctrl_Untreated_1	1.0	Amino acids degradation intermediates	True	3.514e+06	0.09384	0.09384	-1.117
42	2-Me-Citrate	Carbon	1	Ctrl_Untreated_1_7	Ctrl_Untreated_1	0.857142857142857	Propionate metabolism	True	2.233e+05	-3.882	-3.882	0.5008
12	Glucuronate 1-P	Carbon	1	Ctrl_Untreated_1_7	Ctrl_Untreated_1	1.0	Polysaccharide biosynthesis	True	7.154e+05	-2.202	-2.202	-0.9594
76	Gln	Amino acid	1	Ctrl_Untreated_1_8	Ctrl_Untreated_1	1.0	Proteinogenic amino acids	True	2.01e+09	9.622	9.622	0.9417
89	Trp	Amino acid	1	Ctrl_Untreated_1_8	Ctrl_Untreated_1	1.0	Proteinogenic amino acids	True	1.286e+08	5.656	5.656	-0.02005
723	beta-Ala	Cofactor	1	Ctrl_Untreated_1_8	Ctrl_Untreated_1	1.0	Coenzyme A biosynthesis	True	4.652e+07	4.189	4.189	1.18
75	Glu	Amino acid	1	Ctrl_Untreated_1_8	Ctrl_Untreated_1	1.0	Proteinogenic amino acids	True	5.06e+09	10.95	10.95	0.2175
77	Gly	Amino acid	1	Ctrl_Untreated_1_8	Ctrl_Untreated_1	1.0	Proteinogenic amino acids	True	1.533e+08	5.91	5.91	0.2946
80	His	Amino acid	1	Ctrl_Untreated_1_8	Ctrl_Untreated_1	1.0	Proteinogenic amino acids	True	3.288e+07	3.689	3.689	0.2735
82	Leu	Amino acid	1	Ctrl_Untreated_1_8	Ctrl_Untreated_1	1.0	Proteinogenic amino acids	True	1.64e+09	9.329	9.329	0.108
87	Phe	Amino acid	1	Ctrl_Untreated_1_8	Ctrl_Untreated_1	1.0	Proteinogenic amino acids	True	1.215e+09	8.896	8.896	0.08435
130	Glutarate	Amino acid	1	Ctrl_Untreated_1_8	Ctrl_Untreated_1	1.0	Amino acids degradation intermediates	True	5.273e+05	-2.274	-2.274	0.1528
196	5-OH-Indole-Ac	Amino acid	1	Ctrl_Untreated_1_8	Ctrl_Untreated_1	0.857142857142857	Amino acid derivatives	True	9.792e+04	-4.703	-4.703	-0.6911
74	Asp	Amino acid	1	Ctrl_Untreated_1_8	Ctrl_Untreated_1	1.0	Proteinogenic amino acids	True	6.413e+08	7.974	7.974	0.08256
236	Spermidine	Amino acid	1	Ctrl_Untreated_1_8	Ctrl_Untreated_1	1.0	Polyamines	True	2.581e+07	3.339	3.339	-0.5224
73	Asn	Amino acid	1	Ctrl_Untreated_1_8	Ctrl_Untreated_1	1.0	Proteinogenic amino acids	True	2.652e+08	6.7	6.7	0.02942
243	Creatinine	Amino acid	1	Ctrl_Untreated_1_8	Ctrl_Untreated_1	1.0	Creatine degradation	True	8.815e+07	5.111	5.111	0.1091
376	Cytidine	Nucleotide	1	Ctrl_Untreated_1_8	Ctrl_Untreated_1	0.714285714285714	Pyrimidine nucleosides	False			-1.507	-2.235
41	Lactate	Carbon	1	Ctrl_Untreated_1_8	Ctrl_Untreated_1	1.0	Respiratory carbon sources	True	3.098e+08	6.925	6.925	0.4427
58	alpha-Ketoglutarate	Carbon	1	Ctrl_Untreated_1_8	Ctrl_Untreated_1	1.0	TCA cycle	True	3.575e+06	0.4875	0.4875	0.4708
69	3-OH-Butyrate	Carbon	1	Ctrl_Untreated_1_8	Ctrl_Untreated_1	0.571428571428571	Ketone bodies	True	3.321e+05	-2.941	-2.941	0.0436

Metabolite ID	Name	Super Pathway	Dataset	Sample ID	Group ID	Detection Fraction	Pathway	Detected	Raw Intensity	Log2 Norm Intensity	Norm Imputed	Log2 Ctrl Norm
343	Adenine	Nucleotide	1	Ctrl_Untreated_1_8	Ctrl_Untreated_1	1.0	Purine bases	True	7.434e+06	1.544	1.544	0.08663
336	Adenosine	Nucleotide	1	Ctrl_Untreated_1_8	Ctrl_Untreated_1	1.0	Purine nucleosides	True	3.477e+07	3.769	3.769	0.3462
722	ADP-Ribose	Cofactor	1	Ctrl_Untreated_1_8	Ctrl_Untreated_1	1.0	Derivatives of NA, nicotinamide and NAD	True	6.214e+05	-2.037	-2.037	0.965
383	Cytosine	Nucleotide	1	Ctrl_Untreated_1_8	Ctrl_Untreated_1	0.571428571428571	Pyrimidine bases	False			-5.32	-0.4652
3	Glucosamine 6-P	Carbon	1	Ctrl_Untreated_1_8	Ctrl_Untreated_1	1.0	Aminosugar biosynthesis	True	1.239e+06	-1.042	-1.042	0.6254
717	Nicotinamide	Cofactor	1	Ctrl_Untreated_1_8	Ctrl_Untreated_1	1.0	NAD biosynthesis	True	5.241e+07	4.361	4.361	-0.1541
51	PEP	Carbon	1	Ctrl_Untreated_1_8	Ctrl_Untreated_1	1.0	Glycolysis, GNG	True	8.497e+06	1.736	1.736	0.6852
237	Spermine	Amino acid	1	Ctrl_Untreated_1_8	Ctrl_Untreated_1	1.0	Polyamines	True	1.929e+06	-0.4029	-0.4029	1.088
385	Uracil	Nucleotide	1	Ctrl_Untreated_1_8	Ctrl_Untreated_1	1.0	Pyrimidine bases	True	1.385e+06	-0.881	-0.881	-1.146
377	Uridine	Nucleotide	1	Ctrl_Untreated_1_8	Ctrl_Untreated_1	1.0	Pyrimidine nucleosides	True	3.446e+07	3.756	3.756	-0.2925
348	Allantoin	Nucleotide	1	Ctrl_Untreated_1_8	Ctrl_Untreated_1	1.0	Purine degradation	True	2.635e+06	0.04729	0.04729	0.2229
335	Inosine	Nucleotide	1	Ctrl_Untreated_1_8	Ctrl_Untreated_1	1.0	Purine nucleosides	True	1.088e+08	5.415	5.415	-0.08408
81	Ile	Amino acid	1	Ctrl_Untreated_1_8	Ctrl_Untreated_1	1.0	Proteinogenic amino acids	True	1.058e+09	8.696	8.696	-0.01923
72	Ala	Amino acid	1	Ctrl_Untreated_1_8	Ctrl_Untreated_1	1.0	Proteinogenic amino acids	True	1.449e+09	9.15	9.15	0.6489
79	Thr	Amino acid	1	Ctrl_Untreated_1_8	Ctrl_Untreated_1	1.0	Proteinogenic amino acids	True	7.709e+08	8.24	8.24	0.2302
88	Tyr	Amino acid	1	Ctrl_Untreated_1_8	Ctrl_Untreated_1	1.0	Proteinogenic amino acids	True	5.235e+08	7.682	7.682	0.1579
84	Lys	Amino acid	1	Ctrl_Untreated_1_8	Ctrl_Untreated_1	1.0	Proteinogenic amino acids	True	7.336e+08	8.168	8.168	0.1782
86	Met	Amino acid	1	Ctrl_Untreated_1_8	Ctrl_Untreated_1	1.0	Proteinogenic amino acids	True	9.033e+08	8.468	8.468	0.2408
61	Malate	Carbon	1	Ctrl_Untreated_1_8	Ctrl_Untreated_1	1.0	TCA cycle	True	4.186e+08	7.359	7.359	0.187
235	Putrescine	Amino acid	1	Ctrl_Untreated_1_8	Ctrl_Untreated_1	1.0	Polyamines	True	4.409e+05	-2.532	-2.532	-2.041
324	2'-dU	Nucleotide	1	Ctrl_Untreated_1_8	Ctrl_Untreated_1	0.714285714285714	Deoxy-nucleosides	False			-5.281	-0.9009
49	3-P-Glycerate	Carbon	1	Ctrl_Untreated_1_8	Ctrl_Untreated_1	1.0	Glycolysis, GNG	True	2.681e+07	3.394	3.394	0.1287
189	Kynurenate	Amino acid	1	Ctrl_Untreated_1_8	Ctrl_Untreated_1	0.714285714285714	Amino acid derivatives	False			-6.319	-0.5206
234	5-Me-Thioadenosine	Amino acid	1	Ctrl_Untreated_1_8	Ctrl_Untreated_1	1.0	SAM metabolism	True	9.763e+06	1.937	1.937	0.05116
59	Succinate	Carbon	1	Ctrl_Untreated_1_8	Ctrl_Untreated_1	1.0	TCA cycle	True	2.526e+06	-0.01396	-0.01396	-0.315

Metabolite ID	Name	Super Pathway	Dataset	Sample ID	Group ID	Detection Fraction	Pathway	Detected	Raw Intensity	Log2 Norm Intensity	Norm Imputed	Log2 Ctrl Norm
36	Ribose	Carbon	1	Ctrl_Untreated_1_8	Ctrl_Untreated_1	1.0	Sugars and sugar alcohols	True	1.204e+06	-1.083	-1.083	0.1502
133	Ornithine	Amino acid	1	Ctrl_Untreated_1_8	Ctrl_Untreated_1	1.0	Amino acids degradation intermediates	True	9.222e+07	5.176	5.176	0.6969
313	5-Oxoproline	Amino acid	1	Ctrl_Untreated_1_8	Ctrl_Untreated_1	1.0	Glutathione derivatives	True	1.119e+07	2.133	2.133	0.04601
165	N-6-Tri-Me-Lys	Amino acid	1	Ctrl_Untreated_1_8	Ctrl_Untreated_1	1.0	Amino acid derivatives	True	3.222e+07	3.659	3.659	-0.1045
380	Orotate	Nucleotide	1	Ctrl_Untreated_1_8	Ctrl_Untreated_1	0.857142857142857	Pyrimidine (UMP) biosynthesis	True	7.835e+05	-1.703	-1.703	1.928
724	Pantothenate	Cofactor	1	Ctrl_Untreated_1_8	Ctrl_Untreated_1	1.0	Coenzyme A biosynthesis	True	2.032e+08	6.316	6.316	5.60e-03
150	N-Me-Gly	Amino acid	1	Ctrl_Untreated_1_8	Ctrl_Untreated_1	1.0	Amino acid derivatives	True	2.241e+06	-0.1862	-0.1862	0.1143
122	3-OH-Isobutyrate	Amino acid	1	Ctrl_Untreated_1_8	Ctrl_Untreated_1	0.857142857142857	Amino acids degradation intermediates	True	2.721e+05	-3.229	-3.229	-0.07261
241	4-Acetamidobutanoate	Amino acid	1	Ctrl_Untreated_1_8	Ctrl_Untreated_1	1.0	Polyamine derivatives	True	3.89e+06	0.6092	0.6092	0.1682
711	alpha-Tocopherol	Cofactor	1	Ctrl_Untreated_1_8	Ctrl_Untreated_1	1.0	Cofactors	True	7.219e+06	1.501	1.501	0.7209
55	Citrate	Carbon	1	Ctrl_Untreated_1_8	Ctrl_Untreated_1	1.0	TCA cycle	True	7.863e+06	1.624	1.624	-0.3246
387	3-Aminoisobutyrate	Nucleotide	1	Ctrl_Untreated_1_8	Ctrl_Untreated_1	0.857142857142857	Pyrimidine degradation	True	4.253e+05	-2.584	-2.584	-0.5702
338	Guanosine	Nucleotide	1	Ctrl_Untreated_1_8	Ctrl_Untreated_1	1.0	Purine nucleosides	True	8.438e+07	5.048	5.048	-0.05769
209	N-Ac-Ala	Amino acid	1	Ctrl_Untreated_1_8	Ctrl_Untreated_1	1.0	N-acetylated amino acids	True	1.968e+06	-0.3737	-0.3737	0.8275
221	N-Ac-Met	Amino acid	1	Ctrl_Untreated_1_8	Ctrl_Untreated_1	1.0	N-acetylated amino acids	True	1.01e+07	1.986	1.986	0.2416
228	N-Ac-Val	Amino acid	1	Ctrl_Untreated_1_8	Ctrl_Untreated_1	0.571428571428571	N-acetylated amino acids	False			-5.82	-0.449
346	Urate	Nucleotide	1	Ctrl_Untreated_1_8	Ctrl_Untreated_1	1.0	Purine degradation	True	1.159e+06	-1.138	-1.138	-0.07477
90	Arg	Amino acid	1	Ctrl_Untreated_1_8	Ctrl_Untreated_1	1.0	Proteinogenic amino acids	True	2.44e+09	9.902	9.902	0.1961
60	Fumarate	Carbon	1	Ctrl_Untreated_1_8	Ctrl_Untreated_1	1.0	TCA cycle	True	4.899e+06	0.9418	0.9418	-0.07337
78	Ser	Amino acid	1	Ctrl_Untreated_1_8	Ctrl_Untreated_1	1.0	Proteinogenic amino acids	True	5.179e+08	7.666	7.666	0.4086
83	Val	Amino acid	1	Ctrl_Untreated_1_8	Ctrl_Untreated_1	1.0	Proteinogenic amino acids	True	6.279e+08	7.944	7.944	0.07165
734	Pyridoxal	Cofactor	1	Ctrl_Untreated_1_8	Ctrl_Untreated_1	1.0	PLP biosynthesis and salvage	True	6.796e+06	1.414	1.414	-0.369

Metabolite ID	Name	Super Pathway	Dataset	Sample ID	Group ID	Detection Fraction	Pathway	Detected	Raw Intensity	Log2 Norm Intensity	Norm Imputed	Log2 Ctrl Norm
136	Urea	Amino acid	1	Ctrl_Untreated_1_8	Ctrl_Untreated_1	0.857142857142857	Amino acids degradation intermediates	True	2.55e+06	0	0	-0.1512
67	Ribose 1-P	Carbon	1	Ctrl_Untreated_1_8	Ctrl_Untreated_1	1.0	Pentose phosphate pathway (PPP)	True	2.737e+06	0.1022	0.1022	-0.519
284	Carnosine	Amino acid	1	Ctrl_Untreated_1_8	Ctrl_Untreated_1	1.0	Dipeptides	True	7.553e+05	-1.755	-1.755	0.3528
306	gamma-Glu-Cys	Amino acid	1	Ctrl_Untreated_1_8	Ctrl_Untreated_1	1.0	Glutathione biosynthesis	True	2.121e+06	-0.2659	-0.2659	0.07085
712	Retinol (Vit A)	Cofactor	1	Ctrl_Untreated_1_8	Ctrl_Untreated_1	1.0	Cofactors	True	4.198e+05	-2.603	-2.603	-0.1695
85	Cys	Amino acid	1	Ctrl_Untreated_1_8	Ctrl_Untreated_1	1.0	Proteinogenic amino acids	True	4.753e+07	4.22	4.22	-0.2935
91	Pro	Amino acid	1	Ctrl_Untreated_1_8	Ctrl_Untreated_1	1.0	Proteinogenic amino acids	True	1.962e+09	9.587	9.587	0.1794
308	Glutathione, Reduced	Amino acid	1	Ctrl_Untreated_1_8	Ctrl_Untreated_1	1.0	Glutathione	True	5.923e+08	7.86	7.86	0.9035
107	Citrulline	Amino acid	1	Ctrl_Untreated_1_8	Ctrl_Untreated_1	1.0	Amino acids biosynthesis intermediates	True	2.326e+07	3.189	3.189	0.3645
328	IMP	Nucleotide	1	Ctrl_Untreated_1_8	Ctrl_Untreated_1	0.285714285714286	Purine nucleotides	False			-4.53	-0.2088
706	FAD	Cofactor	1	Ctrl_Untreated_1_8	Ctrl_Untreated_1	1.0	Cofactors	True	5.511e+05	-2.21	-2.21	-0.02072
735	Pyridoxamine	Cofactor	1	Ctrl_Untreated_1_8	Ctrl_Untreated_1	1.0	PLP biosynthesis and salvage	True	1.79e+06	-0.5105	-0.5105	-0.3175
199	Serotonin	Amino acid	1	Ctrl_Untreated_1_8	Ctrl_Untreated_1	1.0	Amino acid derivatives	True	7.342e+06	1.526	1.526	0.2528
370	CMP	Nucleotide	1	Ctrl_Untreated_1_8	Ctrl_Untreated_1	1.0	Pyrimidine nucleotides	True	8.14e+06	1.674	1.674	-0.1646
287	gamma-Glu-Gln	Amino acid	1	Ctrl_Untreated_1_8	Ctrl_Untreated_1	1.0	Gamma-glutamyl dipeptides	True	5.668e+06	1.152	1.152	0.3113
14	UDP-Glucuronate	Carbon	1	Ctrl_Untreated_1_8	Ctrl_Untreated_1	0.857142857142857	Polysaccharide biosynthesis	True	1.169e+06	-1.125	-1.125	0.8241
229	N-Formyl-Met	Amino acid	1	Ctrl_Untreated_1_8	Ctrl_Untreated_1	1.0	N-formylated amino acids	True	1.77e+05	-3.849	-3.849	-0.02636
350	3',5'-cAMP	Nucleotide	1	Ctrl_Untreated_1_8	Ctrl_Untreated_1	1.0	Purine derivatives in signaling	True	2.119e+05	-3.589	-3.589	0.03013
371	CDP	Nucleotide	1	Ctrl_Untreated_1_8	Ctrl_Untreated_1	0.571428571428571	Pyrimidine nucleotides	True	6.24e+05	-2.031	-2.031	1.289
372	CTP	Nucleotide	1	Ctrl_Untreated_1_8	Ctrl_Untreated_1	0.571428571428571	Pyrimidine nucleotides	True	2.775e+05	-3.2	-3.2	0.454
333	GDP	Nucleotide	1	Ctrl_Untreated_1_8	Ctrl_Untreated_1	0.571428571428571	Purine nucleotides	True	9.128e+05	-1.482	-1.482	1.018
332	GMP	Nucleotide	1	Ctrl_Untreated_1_8	Ctrl_Untreated_1	1.0	Purine nucleotides	True	8.851e+06	1.795	1.795	1.096

Metabolite ID	Name	Super Pathway	Dataset	Sample ID	Group ID	Detection Fraction	Pathway	Detected	Raw Intensity	Log2 Norm Intensity	Norm Imputed	Log2 Ctrl Norm
373	UMP	Nucleotide	1	Ctrl_Untreated_1_8	Ctrl_Untreated_1	1.0	Pyrimidine nucleotides	True	2.99e+06	0.2296	0.2296	1.285
389	3'-CMP	Nucleotide	1	Ctrl_Untreated_1_8	Ctrl_Untreated_1	1.0	Pyrimidine derivatives in signaling	True	2.761e+06	0.1145	0.1145	1.166
330	ADP	Nucleotide	1	Ctrl_Untreated_1_8	Ctrl_Untreated_1	0.714285714285714	Purine nucleotides	True	5.11e+06	1.003	1.003	1.299
342	Hypoxanthine	Nucleotide	1	Ctrl_Untreated_1_8	Ctrl_Untreated_1	1.0	Purine bases	True	4.609e+07	4.176	4.176	0.2365
736	Pyridoxamine-P	Cofactor	1	Ctrl_Untreated_1_8	Ctrl_Untreated_1	1.0	PLP biosynthesis and salvage	True	1.775e+05	-3.845	-3.845	-0.8667
148	Betaine	Amino acid	1	Ctrl_Untreated_1_8	Ctrl_Untreated_1	1.0	Amino acid derivatives	True	7.739e+07	4.923	4.923	0.1176
344	Xanthine	Nucleotide	1	Ctrl_Untreated_1_8	Ctrl_Untreated_1	1.0	Purine bases	True	2.905e+06	0.1878	0.1878	-0.7198
386	3-Ureidopropionate	Nucleotide	1	Ctrl_Untreated_1_8	Ctrl_Untreated_1	1.0	Pyrimidine degradation	True	1.646e+06	-0.6314	-0.6314	0.2879
149	DiMe-Gly	Amino acid	1	Ctrl_Untreated_1_8	Ctrl_Untreated_1	1.0	Amino acid derivatives	True	1.877e+06	-0.4423	-0.4423	0.07797
703	NAD+	Cofactor	1	Ctrl_Untreated_1_8	Ctrl_Untreated_1	1.0	Cofactors	True	2.293e+07	3.169	3.169	0.4325
709	Pyridoxal-P	Cofactor	1	Ctrl_Untreated_1_8	Ctrl_Untreated_1	1.0	Cofactors	True	6.024e+05	-2.082	-2.082	0.2261
731	Thiamin (Vitamin B1)	Cofactor	1	Ctrl_Untreated_1_8	Ctrl_Untreated_1	1.0	TPP biosynthesis	True	4.236e+06	0.7322	0.7322	-0.01555
374	UDP	Nucleotide	1	Ctrl_Untreated_1_8	Ctrl_Untreated_1	0.714285714285714	Pyrimidine nucleotides	True	4.075e+06	0.6762	0.6762	2.23
102	2-Aminoadipate	Amino acid	1	Ctrl_Untreated_1_8	Ctrl_Untreated_1	1.0	Amino acids biosynthesis intermediates	True	1.049e+06	-1.282	-1.282	0.8273
45	Fructose-6-P	Carbon	1	Ctrl_Untreated_1_8	Ctrl_Untreated_1	1.0	Glycolysis, GNG	True	4.248e+06	0.7363	0.7363	0.661
320	TMP	Nucleotide	1	Ctrl_Untreated_1_8	Ctrl_Untreated_1	0.571428571428571	Deoxy-nucleotides	True	1.927e+05	-3.726	-3.726	1.066
341	XMP	Nucleotide	1	Ctrl_Untreated_1_8	Ctrl_Untreated_1	0.857142857142857	IMP conversion to AMP & GMP	True	9.003e+04	-4.824	-4.824	-0.2497
120	beta-OH-Isovalerate	Amino acid	1	Ctrl_Untreated_1_8	Ctrl_Untreated_1	0.857142857142857	Amino acids degradation intermediates	True	2.867e+05	-3.153	-3.153	0.01246
322	2'-dI	Nucleotide	1	Ctrl_Untreated_1_8	Ctrl_Untreated_1	0.857142857142857	Deoxy-nucleosides	True	6.683e+04	-5.254	-5.254	-1.129
4	GlcNAc 6-P	Carbon	1	Ctrl_Untreated_1_8	Ctrl_Untreated_1	1.0	Aminosugar biosynthesis	True	1.672e+06	-0.6092	-0.6092	-0.9378
337	Xanthosine	Nucleotide	1	Ctrl_Untreated_1_8	Ctrl_Untreated_1	1.0	Purine nucleosides	True	2.263e+05	-3.494	-3.494	-0.4199
188	Kynurenine	Amino acid	1	Ctrl_Untreated_1_8	Ctrl_Untreated_1	1.0	Amino acid derivatives	True	1.807e+06	-0.4968	-0.4968	0.1948

Metabolite ID	Name	Super Pathway	Dataset	Sample ID	Group ID	Detection Fraction	Pathway	Detected	Raw Intensity	Log2 Norm Intensity	Norm Imputed	Log2 Ctrl Norm
63	6-P-Gluconate	Carbon	1	Ctrl_Untreated_1_8	Ctrl_Untreated_1	1.0	Pentose phosphate pathway (PPP)	True	1.515e+07	2.571	2.571	0.537
40	Glucuronate	Carbon	1	Ctrl_Untreated_1_8	Ctrl_Untreated_1	1.0	Sugars and sugar alcohols	True	7.013e+05	-1.862	-1.862	-0.3233
108	Argininosuccinate	Amino acid	1	Ctrl_Untreated_1_8	Ctrl_Untreated_1	1.0	Amino acids biosynthesis intermediates	True	2.455e+06	-0.05493	-0.05493	0.1712
710	Carnitine	Cofactor	1	Ctrl_Untreated_1_8	Ctrl_Untreated_1	1.0	Cofactors	True	2.455e+08	6.589	6.589	0.6114
725	P-Pantetheine	Cofactor	1	Ctrl_Untreated_1_8	Ctrl_Untreated_1	1.0	Coenzyme A biosynthesis	True	2.098e+05	-3.604	-3.604	0.1008
48	DHAP	Carbon	1	Ctrl_Untreated_1_8	Ctrl_Untreated_1	1.0	Glycolysis, GNG	True	2.518e+07	3.304	3.304	0.207
17	Maltose	Carbon	1	Ctrl_Untreated_1_8	Ctrl_Untreated_1	1.0	Glycogen degradation	True	2.067e+06	-0.3032	-0.3032	0.9895
359	N1-Me-Adenosine	Nucleotide	1	Ctrl_Untreated_1_8	Ctrl_Untreated_1	0.857142857142857	Purine derivatives in RNAs	True	8.261e+05	-1.626	-1.626	-0.5885
159	3-Me-His	Amino acid	1	Ctrl_Untreated_1_8	Ctrl_Untreated_1	1.0	Amino acid derivatives	True	1.168e+05	-4.448	-4.448	-0.2126
155	4-Guanidinobutanoate	Amino acid	1	Ctrl_Untreated_1_8	Ctrl_Untreated_1	1.0	Amino acid derivatives	True	1.063e+06	-1.263	-1.263	0.1713
164	5-OH-Lys	Amino acid	1	Ctrl_Untreated_1_8	Ctrl_Untreated_1	1.0	Amino acid derivatives	True	1.316e+06	-0.9545	-0.9545	0.8159
357	Adenosine-3',5'-PP	Nucleotide	1	Ctrl_Untreated_1_8	Ctrl_Untreated_1	0.857142857142857	Purine byproducts of metabolic processes	True	1.55e+05	-4.041	-4.041	-0.5633
104	Cystathionine	Amino acid	1	Ctrl_Untreated_1_8	Ctrl_Untreated_1	1.0	Amino acids biosynthesis intermediates	True	1.555e+07	2.608	2.608	0.3986
113	Imidazole Lactate	Amino acid	1	Ctrl_Untreated_1_8	Ctrl_Untreated_1	0.857142857142857	Amino acids degradation intermediates	True	2.477e+05	-3.364	-3.364	0.3126
215	N-Ac-Glu	Amino acid	1	Ctrl_Untreated_1_8	Ctrl_Untreated_1	1.0	N-acetylated amino acids	True	1e+07	1.972	1.972	0.2281
310	S-Lactoyl-Glutathione	Amino acid	1	Ctrl_Untreated_1_8	Ctrl_Untreated_1	0.857142857142857	Glutathione derivatives	True	7.604e+06	1.576	1.576	1.22
5	GlcNAc 1-P	Carbon	1	Ctrl_Untreated_1_8	Ctrl_Untreated_1	1.0	Aminosugar biosynthesis	True	4.335e+05	-2.557	-2.557	-0.6458
34	Ribitol	Carbon	1	Ctrl_Untreated_1_8	Ctrl_Untreated_1	1.0	Sugars and sugar alcohols	True	2.18e+05	-3.548	-3.548	-0.1179
10	UDP-Galactose	Carbon	1	Ctrl_Untreated_1_8	Ctrl_Untreated_1	0.857142857142857	Polysaccharide biosynthesis	False			-1.452	-0.8254
13	Guanosine 5'-PP-Fucose	Carbon	1	Ctrl_Untreated_1_8	Ctrl_Untreated_1	1.0	Polysaccharide biosynthesis	True	5.039e+05	-2.34	-2.34	-0.04024

Metabolite ID	Name	Super Pathway	Dataset	Sample ID	Group ID	Detection Fraction	Pathway	Detected	Raw Intensity	Log2 Norm Intensity	Norm Imputed	Log2 Ctrl Norm
19	Maltotetraose	Carbon	1	Ctrl_Untreated_1_8	Ctrl_Untreated_1	1.0	Glycogen degradation	True	5.191e+06	1.025	1.025	1.069
233	SAM	Amino acid	1	Ctrl_Untreated_1_8	Ctrl_Untreated_1	1.0	SAM metabolism	True	2.982e+06	0.2256	0.2256	0.3819
129	5-Aminovalerate	Amino acid	1	Ctrl_Untreated_1_8	Ctrl_Untreated_1	1.0	Amino acids degradation intermediates	True	4.028e+06	0.6593	0.6593	-0.3423
741	5-Me-THF	Cofactor	1	Ctrl_Untreated_1_8	Ctrl_Untreated_1	0.714285714285714	Folate metabolism	False			-5.366	-0.6085
198	Indolelactate	Amino acid	1	Ctrl_Untreated_1_8	Ctrl_Untreated_1	0.857142857142857	Amino acid derivatives	True	1.314e+05	-4.279	-4.279	-0.492
254	Gly-Val	Amino acid	1	Ctrl_Untreated_1_8	Ctrl_Untreated_1	1.0	Dipeptides	True	5.579e+06	1.129	1.129	0.01809
291	gamma-Glu-Leu	Amino acid	1	Ctrl_Untreated_1_8	Ctrl_Untreated_1	1.0	Gamma-glutamyl dipeptides	True	1.133e+06	-1.171	-1.171	-0.6823
173	Met Sulfoxide	Amino acid	1	Ctrl_Untreated_1_8	Ctrl_Untreated_1	1.0	Amino acid derivatives	True	1.43e+07	2.487	2.487	-0.07984
43	Glucose	Carbon	1	Ctrl_Untreated_1_8	Ctrl_Untreated_1	1.0	Glycolysis, GNG	True	5.966e+07	4.548	4.548	-0.5033
185	Phenyllactate	Amino acid	1	Ctrl_Untreated_1_8	Ctrl_Untreated_1	0.285714285714286	Amino acid derivatives	False			-5.917	-0.1051
156	Homo-Arg	Amino acid	1	Ctrl_Untreated_1_8	Ctrl_Untreated_1	1.0	Amino acid derivatives	True	4.927e+06	0.9501	0.9501	-0.2305
135	Homocitrulline	Amino acid	1	Ctrl_Untreated_1_8	Ctrl_Untreated_1	1.0	Amino acids degradation intermediates	True	8.673e+05	-1.556	-1.556	0.1466
719	Nicotinamide MN	Cofactor	1	Ctrl_Untreated_1_8	Ctrl_Untreated_1	1.0	NAD biosynthesis	True	1.282e+06	-0.9923	-0.9923	-1.178
212	N-Ac-Asp	Amino acid	1	Ctrl_Untreated_1_8	Ctrl_Untreated_1	1.0	N-acetylated amino acids	True	7.463e+06	1.549	1.549	0.8519
720	1-Me-Nicotinamide	Cofactor	1	Ctrl_Untreated_1_8	Ctrl_Untreated_1	1.0	Derivatives of NA, nicotinamide and NAD	True	5.836e+08	7.838	7.838	-0.05229
216	N-Ac-Gly	Amino acid	1	Ctrl_Untreated_1_8	Ctrl_Untreated_1	0.714285714285714	N-acetylated amino acids	True	1.595e+05	-3.999	-3.999	-0.4589
70	Creatine	Carbon	1	Ctrl_Untreated_1_8	Ctrl_Untreated_1	1.0	Creatine energy storage	True	1.804e+09	9.466	9.466	0.04906
26	Galactonate	Carbon	1	Ctrl_Untreated_1_8	Ctrl_Untreated_1	0.857142857142857	Sugars and sugar alcohols	True	2.874e+06	0.1727	0.1727	0.9773
309	Glutathione, Oxidized	Amino acid	1	Ctrl_Untreated_1_8	Ctrl_Untreated_1	1.0	Glutathione	True	6.176e+06	1.276	1.276	0.08335
35	Ribonate	Carbon	1	Ctrl_Untreated_1_8	Ctrl_Untreated_1	1.0	Sugars and sugar alcohols	True	2.12e+06	-0.2664	-0.2664	0.2608
160	1-Me-His	Amino acid	1	Ctrl_Untreated_1_8	Ctrl_Untreated_1	1.0	Amino acid derivatives	True	3.202e+07	3.65	3.65	0.2093

Metabolite ID	Name	Super Pathway	Dataset	Sample ID	Group ID	Detection Fraction	Pathway	Detected	Raw Intensity	Log2 Norm Intensity	Norm Imputed	Log2 Ctrl Norm
44	Glucose 6-P	Carbon	1	Ctrl_Untreated_1_8	Ctrl_Untreated_1	0.857142857142857	Glycolysis, GNG	True	1.487e+06	-0.7782	-0.7782	0.6985
704	NADH	Cofactor	1	Ctrl_Untreated_1_8	Ctrl_Untreated_1	1.0	Cofactors	True	2.765e+06	0.1169	0.1169	0.7629
275	Thr-Phe	Amino acid	1	Ctrl_Untreated_1_8	Ctrl_Untreated_1	0.857142857142857	Dipeptides	True	1.119e+06	-1.189	-1.189	0.4645
738	Pyridoxate	Cofactor	1	Ctrl_Untreated_1_8	Ctrl_Untreated_1	1.0	PLP biosynthesis and salvage	True	2.712e+05	-3.233	-3.233	0.131
177	3-(4-OH-Phenyl)Lactate	Amino acid	1	Ctrl_Untreated_1_8	Ctrl_Untreated_1	1.0	Amino acid derivatives	True	5.22e+05	-2.289	-2.289	0.1768
206	Trans-4-OH-Pro	Amino acid	1	Ctrl_Untreated_1_8	Ctrl_Untreated_1	1.0	Amino acid derivatives	True	1.257e+08	5.624	5.624	0.4684
329	AMP	Nucleotide	1	Ctrl_Untreated_1_8	Ctrl_Untreated_1	1.0	Purine nucleotides	True	5.995e+07	4.555	4.555	0.92
11	UDP-Glucose	Carbon	1	Ctrl_Untreated_1_8	Ctrl_Untreated_1	1.0	Polysaccharide biosynthesis	True	5.842e+06	1.196	1.196	1.301
158	4-Imidazole-Ac	Amino acid	1	Ctrl_Untreated_1_8	Ctrl_Untreated_1	1.0	Amino acid derivatives	True	4.147e+05	-2.62	-2.62	-0.4737
111	1-Me-Imidazole-Ac	Amino acid	1	Ctrl_Untreated_1_8	Ctrl_Untreated_1	1.0	Amino acids degradation intermediates	True	6.71e+05	-1.926	-1.926	7.64e-03
345	Guanine	Nucleotide	1	Ctrl_Untreated_1_8	Ctrl_Untreated_1	1.0	Purine bases	True	9.876e+07	5.275	5.275	0.2889
22	N-Ac-Neuraminate	Carbon	1	Ctrl_Untreated_1_8	Ctrl_Untreated_1	1.0	Aminosugar derivatives	True	2.847e+06	0.1586	0.1586	0.294
721	N'-Methylnicotinate	Cofactor	1	Ctrl_Untreated_1_8	Ctrl_Untreated_1	1.0	Derivatives of NA, nicotinamide and NAD	True	1.698e+06	-0.5869	-0.5869	0.4207
183	Phenol Sulfate	Amino acid	1	Ctrl_Untreated_1_8	Ctrl_Untreated_1	0.857142857142857	Amino acid derivatives	True	6.248e+04	-5.351	-5.351	-0.7108
718	Nicotinamide Riboside	Cofactor	1	Ctrl_Untreated_1_8	Ctrl_Untreated_1	1.0	NAD biosynthesis	True	8.931e+05	-1.514	-1.514	-3.104
297	gamma-Glu-Thr	Amino acid	1	Ctrl_Untreated_1_8	Ctrl_Untreated_1	1.0	Gamma-glutamyl dipeptides	True	1.93e+06	-0.4019	-0.4019	-1.042
295	gamma-Glu-Phe	Amino acid	1	Ctrl_Untreated_1_8	Ctrl_Untreated_1	0.428571428571429	Gamma-glutamyl dipeptides	False			-5.975	-0.1899
347	Allantoic Acid	Nucleotide	1	Ctrl_Untreated_1_8	Ctrl_Untreated_1	0.571428571428571	Purine degradation	True	5.655e+04	-5.495	-5.495	0.1444
399	Pseudouridine	Nucleotide	1	Ctrl_Untreated_1_8	Ctrl_Untreated_1	1.0	Pyrimidine derivatives in RNAs	True	6.013e+05	-2.084	-2.084	-0.05437
375	UTP	Nucleotide	1	Ctrl_Untreated_1_8	Ctrl_Untreated_1	0.571428571428571	Pyrimidine nucleotides	True	2.159e+06	-0.2404	-0.2404	1.736
144	Glu, gamma-Me Ester	Amino acid	1	Ctrl_Untreated_1_8	Ctrl_Untreated_1	1.0	Amino acid derivatives	True	4.469e+06	0.8094	0.8094	0.2316

Metabolite ID	Name	Super Pathway	Dataset	Sample ID	Group ID	Detection Fraction	Pathway	Detected	Raw Intensity	Log2 Norm Intensity	Norm Imputed	Log2 Ctrl Norm
292	gamma-Glu-epsilon-Lysine	Amino acid	1	Ctrl_Untreated_1_8	Ctrl_Untreated_1	1.0	Gamma-glutamyl dipeptides	True	2.419e+06	-0.07597	-0.07597	0.1154
225	N-Ac-Thr	Amino acid	1	Ctrl_Untreated_1_8	Ctrl_Untreated_1	1.0	N-acetylated amino acids	True	1.481e+06	-0.7845	-0.7845	0.7181
211	N-Ac-Asn	Amino acid	1	Ctrl_Untreated_1_8	Ctrl_Untreated_1	1.0	N-acetylated amino acids	True	5.323e+05	-2.26	-2.26	0.425
151	Phenylacetylglycine	Amino acid	1	Ctrl_Untreated_1_8	Ctrl_Untreated_1	1.0	Amino acid derivatives	True	1.468e+06	-0.7971	-0.7971	-0.01012
217	N-Ac-His	Amino acid	1	Ctrl_Untreated_1_8	Ctrl_Untreated_1	1.0	N-acetylated amino acids	True	2.277e+05	-3.485	-3.485	-3.47e-04
288	gamma-Glu-Gly	Amino acid	1	Ctrl_Untreated_1_8	Ctrl_Untreated_1	0.571428571428571	Gamma-glutamyl dipeptides	True	1.125e+06	-1.18	-1.18	0.6441
222	N-Ac-Phe	Amino acid	1	Ctrl_Untreated_1_8	Ctrl_Untreated_1	0.571428571428571	N-acetylated amino acids	False			-6.698	-0.4255
71	Creatine-P	Carbon	1	Ctrl_Untreated_1_8	Ctrl_Untreated_1	1.0	Creatine energy storage	True	1.14e+05	-4.484	-4.484	-0.2284
210	N-Ac-Arg	Amino acid	1	Ctrl_Untreated_1_8	Ctrl_Untreated_1	1.0	N-acetylated amino acids	True	4.745e+05	-2.426	-2.426	0.2495
218	N-Ac-Ile	Amino acid	1	Ctrl_Untreated_1_8	Ctrl_Untreated_1	0.428571428571429	N-acetylated amino acids	False			-6.52	-0.5541
251	Gly-Leu	Amino acid	1	Ctrl_Untreated_1_8	Ctrl_Untreated_1	1.0	Dipeptides	True	9.439e+06	1.888	1.888	0.2494
290	gamma-Glu-Ile	Amino acid	1	Ctrl_Untreated_1_8	Ctrl_Untreated_1	1.0	Gamma-glutamyl dipeptides	True	5.467e+05	-2.222	-2.222	-0.8831
316	Ophthalmate	Amino acid	1	Ctrl_Untreated_1_8	Ctrl_Untreated_1	1.0	Oxidative stress markers	True	1.083e+07	2.086	2.086	-0.2562
125	Isovaleryl-Gly	Amino acid	1	Ctrl_Untreated_1_8	Ctrl_Untreated_1	0.857142857142857	Amino acids degradation intermediates	False			-5.862	-0.4529
368	7-Me-Guanine	Nucleotide	1	Ctrl_Untreated_1_8	Ctrl_Untreated_1	1.0	Purine derivatives in RNAs	True	5.662e+05	-2.171	-2.171	0.01888
208	Pro-OH-Pro	Amino acid	1	Ctrl_Untreated_1_8	Ctrl_Untreated_1	1.0	Amino acid derivatives	True	4.183e+07	4.036	4.036	0.7065
366	N2,N2-DiMe-Guanosine	Nucleotide	1	Ctrl_Untreated_1_8	Ctrl_Untreated_1	0.857142857142857	Purine derivatives in RNAs	True	9.756e+04	-4.708	-4.708	-0.5946
352	3'-AMP	Nucleotide	1	Ctrl_Untreated_1_8	Ctrl_Untreated_1	1.0	Purine derivatives in signaling	True	1.622e+06	-0.6524	-0.6524	0.8344
363	N6-Carbamoyl-Thr-Adenosine	Nucleotide	1	Ctrl_Untreated_1_8	Ctrl_Untreated_1	0.857142857142857	Purine derivatives in RNAs	True	9.461e+04	-4.752	-4.752	-0.06706
314	Cys-Glutathione Disulfide	Amino acid	1	Ctrl_Untreated_1_8	Ctrl_Untreated_1	1.0	Oxidative stress markers	True	1.168e+05	-4.449	-4.449	-1.165
382	Orotidine	Nucleotide	1	Ctrl_Untreated_1_8	Ctrl_Untreated_1	0.428571428571429	Pyrimidine (UMP) biosynthesis	True	2.48e+05	-3.362	-3.362	0.9294

Metabolite ID	Name	Super Pathway	Dataset	Sample ID	Group ID	Detection Fraction	Pathway	Detected	Raw Intensity	Log2 Norm Intensity	Norm Imputed	Log2 Ctrl Norm
307	Cys-Gly	Amino acid	1	Ctrl_Untreated_1_8	Ctrl_Untreated_1	1.0	Glutathione biosynthesis	True	9.604e+06	1.913	1.913	0.306
64	Sedoheptulose-7-P	Carbon	1	Ctrl_Untreated_1_8	Ctrl_Untreated_1	1.0	Pentose phosphate pathway (PPP)	True	2.118e+06	-0.2676	-0.2676	-0.4217
142	N-Ac-Asp-Glu	Amino acid	1	Ctrl_Untreated_1_8	Ctrl_Untreated_1	1.0	Amino acid derivatives	True	1.324e+06	-0.9461	-0.9461	0.1993
708	Thiamin-PP	Cofactor	1	Ctrl_Untreated_1_8	Ctrl_Untreated_1	0.571428571428571	Cofactors	False			-6.331	-0.3472
182	P-Cresol Sulfate	Amino acid	1	Ctrl_Untreated_1_8	Ctrl_Untreated_1	0.857142857142857	Amino acid derivatives	True	2.286e+05	-3.48	-3.48	-0.2837
250	Gly-Ile	Amino acid	1	Ctrl_Untreated_1_8	Ctrl_Untreated_1	1.0	Dipeptides	True	1.145e+06	-1.155	-1.155	0.1619
286	gamma-Glu-Glu	Amino acid	1	Ctrl_Untreated_1_8	Ctrl_Untreated_1	1.0	Gamma-glutamyl dipeptides	True	3.307e+06	0.3748	0.3748	0.3569
739	Deoxycarnitine	Cofactor	1	Ctrl_Untreated_1_8	Ctrl_Untreated_1	1.0	Carnitine biosynthesis	True	4.539e+07	4.154	4.154	0.4026
203	DiMe-Arg	Amino acid	1	Ctrl_Untreated_1_8	Ctrl_Untreated_1	1.0	Amino acid derivatives	True	1.806e+08	6.146	6.146	0.5132
351	2'-AMP	Nucleotide	1	Ctrl_Untreated_1_8	Ctrl_Untreated_1	1.0	Purine derivatives in signaling	True	1.892e+05	-3.752	-3.752	-1.484
8	Cytidine 5'-P-N-Ac-Neuraminate	Carbon	1	Ctrl_Untreated_1_8	Ctrl_Untreated_1	1.0	Aminosugar biosynthesis	True	7.56e+05	-1.754	-1.754	-8.67e-03
285	gamma-Glu-Ala	Amino acid	1	Ctrl_Untreated_1_8	Ctrl_Untreated_1	0.857142857142857	Gamma-glutamyl dipeptides	True	3.624e+05	-2.815	-2.815	0.424
224	N-Ac-Ser	Amino acid	1	Ctrl_Untreated_1_8	Ctrl_Untreated_1	1.0	N-acetylated amino acids	True	1.099e+07	2.108	2.108	0.2288
244	Ala-Leu	Amino acid	1	Ctrl_Untreated_1_8	Ctrl_Untreated_1	1.0	Dipeptides	True	8.849e+06	1.795	1.795	0.6569
207	N-Me-Pro	Amino acid	1	Ctrl_Untreated_1_8	Ctrl_Untreated_1	1.0	Amino acid derivatives	True	1.504e+06	-0.7616	-0.7616	0.1684
171	Cys Sulfinic Acid	Amino acid	1	Ctrl_Untreated_1_8	Ctrl_Untreated_1	0.857142857142857	Amino acid derivatives	True	7.218e+05	-1.821	-1.821	1.476
181	O-Me-Tyr	Amino acid	1	Ctrl_Untreated_1_8	Ctrl_Untreated_1	0.857142857142857	Amino acid derivatives	True	2.705e+05	-3.237	-3.237	0.1328
240	N-Ac-Putrescine	Amino acid	1	Ctrl_Untreated_1_8	Ctrl_Untreated_1	1.0	Polyamine derivatives	True	6.461e+04	-5.303	-5.303	-2.939
176	S-Me-Met	Amino acid	1	Ctrl_Untreated_1_8	Ctrl_Untreated_1	0.857142857142857	Amino acid derivatives	True	9.024e+05	-1.499	-1.499	0.343
339	AICAR	Nucleotide	1	Ctrl_Untreated_1_8	Ctrl_Untreated_1	0.571428571428571	IMP biosynthesis	False			-4.716	-0.4151
141	gamma-Carboxy-Glu	Amino acid	1	Ctrl_Untreated_1_8	Ctrl_Untreated_1	1.0	Amino acid derivatives	True	1.891e+06	-0.4317	-0.4317	0.3533

Metabolite ID	Name	Super Pathway	Dataset	Sample ID	Group ID	Detection Fraction	Pathway	Detected	Raw Intensity	Log2 Norm Intensity	Norm Imputed	Log2 Ctrl Norm
392	3'-UMP	Nucleotide	1	Ctrl_Untreated_1_8	Ctrl_Untreated_1	0.571428571428571	Pyrimidine derivatives in signaling	False			-5.207	-1.174
355	3'-GMP	Nucleotide	1	Ctrl_Untreated_1_8	Ctrl_Untreated_1	0.857142857142857	Purine derivatives in signaling	True	4.647e+05	-2.456	-2.456	1.481
282	Val-Leu	Amino acid	1	Ctrl_Untreated_1_8	Ctrl_Untreated_1	1.0	Dipeptides	True	5.835e+06	1.194	1.194	0.2224
140	Carboxyethyl-GABA	Amino acid	1	Ctrl_Untreated_1_8	Ctrl_Untreated_1	1.0	Amino acid derivatives	True	1.026e+06	-1.313	-1.313	0.1181
258	Ile-Gly	Amino acid	1	Ctrl_Untreated_1_8	Ctrl_Untreated_1	1.0	Dipeptides	True	3.052e+07	3.581	3.581	0.2693
260	Leu-Ala	Amino acid	1	Ctrl_Untreated_1_8	Ctrl_Untreated_1	1.0	Dipeptides	True	9.387e+06	1.88	1.88	0.5788
265	Lys-Leu	Amino acid	1	Ctrl_Untreated_1_8	Ctrl_Untreated_1	0.857142857142857	Dipeptides	True	2.291e+05	-3.476	-3.476	-0.865
263	Leu-Gly	Amino acid	1	Ctrl_Untreated_1_8	Ctrl_Untreated_1	1.0	Dipeptides	True	4.914e+07	4.268	4.268	1.247
281	Val-Gly	Amino acid	1	Ctrl_Untreated_1_8	Ctrl_Untreated_1	1.0	Dipeptides	True	8.018e+07	4.975	4.975	0.4554
270	Pro-Gly	Amino acid	1	Ctrl_Untreated_1_8	Ctrl_Untreated_1	1.0	Dipeptides	True	5.99e+06	1.232	1.232	0.4853
114	Imidazole Propionate	Amino acid	1	Ctrl_Untreated_1_8	Ctrl_Untreated_1	1.0	Amino acids degradation intermediates	True	8.126e+05	-1.65	-1.65	0.6606
267	Phe-Gly	Amino acid	1	Ctrl_Untreated_1_8	Ctrl_Untreated_1	1.0	Dipeptides	True	5.554e+07	4.445	4.445	0.3511
266	Phe-Ala	Amino acid	1	Ctrl_Untreated_1_8	Ctrl_Untreated_1	1.0	Dipeptides	True	6.597e+06	1.371	1.371	0.2945
278	Tyr-Gly	Amino acid	1	Ctrl_Untreated_1_8	Ctrl_Untreated_1	1.0	Dipeptides	True	1.304e+07	2.354	2.354	0.172
255	His-Ala	Amino acid	1	Ctrl_Untreated_1_8	Ctrl_Untreated_1	1.0	Dipeptides	True	1.07e+06	-1.252	-1.252	0.08492
280	Val-Gln	Amino acid	1	Ctrl_Untreated_1_8	Ctrl_Untreated_1	1.0	Dipeptides	True	1.293e+07	2.342	2.342	0.7242
143	S-1-Pyrroline-5-Carboxylate	Amino acid	1	Ctrl_Untreated_1_8	Ctrl_Untreated_1	0.857142857142857	Amino acid derivatives	True	9.435e+05	-1.435	-1.435	0.6336
232	SAH	Amino acid	1	Ctrl_Untreated_1_8	Ctrl_Untreated_1	1.0	SAM metabolism	True	2.34e+06	-0.124	-0.124	0.3614
20	Erythronate	Carbon	1	Ctrl_Untreated_1_8	Ctrl_Untreated_1	1.0	Aminosugar derivatives	True	5.164e+07	4.34	4.34	0.5985
248	Gln-Leu	Amino acid	1	Ctrl_Untreated_1_8	Ctrl_Untreated_1	1.0	Dipeptides	True	2.313e+06	-0.1406	-0.1406	0.7647
276	Trp-Gly	Amino acid	1	Ctrl_Untreated_1_8	Ctrl_Untreated_1	1.0	Dipeptides	True	1.302e+06	-0.9698	-0.9698	0.1085
205	N-delta-Ac-Ornithine	Amino acid	1	Ctrl_Untreated_1_8	Ctrl_Untreated_1	1.0	Amino acid derivatives	True	8.637e+05	-1.562	-1.562	-0.441
163	Formimino-Glu	Amino acid	1	Ctrl_Untreated_1_8	Ctrl_Untreated_1	1.0	Amino acid derivatives	True	1.361e+06	-0.9062	-0.9062	0.3336
204	N-Me-Arg	Amino acid	1	Ctrl_Untreated_1_8	Ctrl_Untreated_1	1.0	Amino acid derivatives	True	2.555e+07	3.325	3.325	0.7376
242	Guanidino-Ac	Amino acid	1	Ctrl_Untreated_1_8	Ctrl_Untreated_1	0.857142857142857	Creatine biosynthesis	True	1.139e+05	-4.485	-4.485	-0.6044

Metabolite ID	Name	Super Pathway	Dataset	Sample ID	Group ID	Detection Fraction	Pathway	Detected	Raw Intensity	Log2 Norm Intensity	Norm Imputed	Log2 Ctrl Norm
300	gamma-Glu-Val	Amino acid	1	Ctrl_Untreated_1_8	Ctrl_Untreated_1	0.857142857142857	Gamma-glutamyl dipeptides	False			-0.1053	-0.726
53	Ac-CoA	Carbon	1	Ctrl_Untreated_1_8	Ctrl_Untreated_1	0.285714285714286	Acetyl-CoA	False			-7.274	-0.5114
18	Maltotriose	Carbon	1	Ctrl_Untreated_1_8	Ctrl_Untreated_1	1.0	Glycogen degradation	True	2.548e+06	-1.49e-03	-1.49e-03	0.1563
294	gamma-Glu-Met	Amino acid	1	Ctrl_Untreated_1_8	Ctrl_Untreated_1	0.714285714285714	Gamma-glutamyl dipeptides	True	2.85e+05	-3.161	-3.161	0.3207
174	Met Sulfone	Amino acid	1	Ctrl_Untreated_1_8	Ctrl_Untreated_1	1.0	Amino acid derivatives	True	6.385e+05	-1.998	-1.998	1.081
175	N-Ac-Met Sulfoxide	Amino acid	1	Ctrl_Untreated_1_8	Ctrl_Untreated_1	1.0	Amino acid derivatives	True	2.134e+06	-0.2574	-0.2574	-0.2337
25	Mannitol/Sorbitol	Carbon	1	Ctrl_Untreated_1_8	Ctrl_Untreated_1	1.0	Sugars and sugar alcohols	True	1.051e+07	2.044	2.044	-0.1809
6	UDP-GlcNAc	Carbon	1	Ctrl_Untreated_1_8	Ctrl_Untreated_1	0.428571428571429	Aminosugar biosynthesis	True	4.665e+05	-2.451	-2.451	0.6573
145	Pyro-Gln	Amino acid	1	Ctrl_Untreated_1_8	Ctrl_Untreated_1	1.0	Amino acid derivatives	True	7.442e+06	1.545	1.545	0.7054
705	Coenzyme A	Cofactor	1	Ctrl_Untreated_1_8	Ctrl_Untreated_1	0.714285714285714	Cofactors	True	8.155e+04	-4.967	-4.967	-1.008
319	2'-dAMP	Nucleotide	1	Ctrl_Untreated_1_8	Ctrl_Untreated_1	0.571428571428571	Deoxy-nucleotides	True	2.64e+05	-3.272	-3.272	0.4083
119	alpha-OH-Isovalerate	Amino acid	1	Ctrl_Untreated_1_8	Ctrl_Untreated_1	0.714285714285714	Amino acids degradation intermediates	False			-5.298	-1.999
46	Fructose 1,6-PP / Glucose 1,6-PP / Inositol-1,4-PP / Inositol-1,3-PP	Carbon	1	Ctrl_Untreated_1_8	Ctrl_Untreated_1	1.0	Glycolysis, GNG	True	1.6e+08	5.972	5.972	1.14
137	1-Me-Guanidine	Amino acid	1	Ctrl_Untreated_1_8	Ctrl_Untreated_1	0.857142857142857	Amino acids degradation intermediates	True	5.64e+04	-5.499	-5.499	-0.3461
23	N-GlcNAc-Asn	Carbon	1	Ctrl_Untreated_1_8	Ctrl_Untreated_1	1.0	Aminosugar derivatives	True	5.055e+06	0.9871	0.9871	0.2742
262	Leu-Gln	Amino acid	1	Ctrl_Untreated_1_8	Ctrl_Untreated_1	1.0	Dipeptides	True	8.646e+06	1.761	1.761	0.4651
24	Fructose	Carbon	1	Ctrl_Untreated_1_8	Ctrl_Untreated_1	1.0	Sugars and sugar alcohols	True	1.026e+07	2.008	2.008	0.05923
197	C-Glycosyl-Trp	Amino acid	1	Ctrl_Untreated_1_8	Ctrl_Untreated_1	1.0	Amino acid derivatives	True	1.96e+06	-0.3795	-0.3795	-0.08045
33	Arabitol/Xylitol	Carbon	1	Ctrl_Untreated_1_8	Ctrl_Untreated_1	1.0	Sugars and sugar alcohols	True	3.924e+05	-2.7	-2.7	-0.8404

Metabolite ID	Name	Super Pathway	Dataset	Sample ID	Group ID	Detection Fraction	Pathway	Detected	Raw Intensity	Log2 Norm Intensity	Norm Imputed	Log2 Ctrl Norm
128	N2-Ac-Lys/N6-Ac-Lys	Amino acid	1	Ctrl_Untreated_1_8	Ctrl_Untreated_1	1.0	Amino acids degradation intermediates	True	7.464e+06	1.549	1.549	0.339
42	2-Me-Citrate	Carbon	1	Ctrl_Untreated_1_8	Ctrl_Untreated_1	0.857142857142857	Propionate metabolism	False			-5.211	-0.8276
12	Glucuronate 1-P	Carbon	1	Ctrl_Untreated_1_8	Ctrl_Untreated_1	1.0	Polysaccharide biosynthesis	True	8.993e+05	-1.504	-1.504	-0.2609
76	Gln	Amino acid	1	Ctrl_Untreated_1_9	Ctrl_Untreated_1	1.0	Proteinogenic amino acids	True	1.107e+09	8.774	8.774	0.09337
89	Trp	Amino acid	1	Ctrl_Untreated_1_9	Ctrl_Untreated_1	1.0	Proteinogenic amino acids	True	1.342e+08	5.73	5.73	0.05374
723	beta-Ala	Cofactor	1	Ctrl_Untreated_1_9	Ctrl_Untreated_1	1.0	Coenzyme A biosynthesis	True	2.413e+07	3.254	3.254	0.2449
75	Glu	Amino acid	1	Ctrl_Untreated_1_9	Ctrl_Untreated_1	1.0	Proteinogenic amino acids	True	5.034e+09	10.96	10.96	0.2224
77	Gly	Amino acid	1	Ctrl_Untreated_1_9	Ctrl_Untreated_1	1.0	Proteinogenic amino acids	True	1.044e+08	5.368	5.368	-0.2477
80	His	Amino acid	1	Ctrl_Untreated_1_9	Ctrl_Untreated_1	1.0	Proteinogenic amino acids	True	2.33e+07	3.204	3.204	-0.2115
82	Leu	Amino acid	1	Ctrl_Untreated_1_9	Ctrl_Untreated_1	1.0	Proteinogenic amino acids	True	1.167e+09	8.85	8.85	-0.3717
87	Phe	Amino acid	1	Ctrl_Untreated_1_9	Ctrl_Untreated_1	1.0	Proteinogenic amino acids	True	9.929e+08	8.617	8.617	-0.1946
130	Glutarate	Amino acid	1	Ctrl_Untreated_1_9	Ctrl_Untreated_1	1.0	Amino acids degradation intermediates	True	7.257e+05	-1.801	-1.801	0.6256
196	5-OH-Indole-Ac	Amino acid	1	Ctrl_Untreated_1_9	Ctrl_Untreated_1	0.857142857142857	Amino acid derivatives	True	2.918e+05	-3.116	-3.116	0.8962
74	Asp	Amino acid	1	Ctrl_Untreated_1_9	Ctrl_Untreated_1	1.0	Proteinogenic amino acids	True	4.143e+08	7.356	7.356	-0.5359
236	Spermidine	Amino acid	1	Ctrl_Untreated_1_9	Ctrl_Untreated_1	1.0	Polyamines	True	7.206e+07	4.833	4.833	0.971
73	Asn	Amino acid	1	Ctrl_Untreated_1_9	Ctrl_Untreated_1	1.0	Proteinogenic amino acids	True	2.199e+08	6.442	6.442	-0.2289
243	Creatinine	Amino acid	1	Ctrl_Untreated_1_9	Ctrl_Untreated_1	1.0	Creatine degradation	True	1.229e+08	5.603	5.603	0.601
376	Cytidine	Nucleotide	1	Ctrl_Untreated_1_9	Ctrl_Untreated_1	0.714285714285714	Pyrimidine nucleosides	False			-1.507	-2.235
41	Lactate	Carbon	1	Ctrl_Untreated_1_9	Ctrl_Untreated_1	1.0	Respiratory carbon sources	True	2.543e+08	6.652	6.652	0.1698
58	alpha-Ketoglutarate	Carbon	1	Ctrl_Untreated_1_9	Ctrl_Untreated_1	1.0	TCA cycle	True	4.088e+06	0.6929	0.6929	0.6762
69	3-OH-Butyrate	Carbon	1	Ctrl_Untreated_1_9	Ctrl_Untreated_1	0.571428571428571	Ketone bodies	True	6.994e+05	-1.854	-1.854	1.13

Metabolite ID	Name	Super Pathway	Dataset	Sample ID	Group ID	Detection Fraction	Pathway	Detected	Raw Intensity	Log2 Norm Intensity	Norm Imputed	Log2 Ctrl Norm
343	Adenine	Nucleotide	1	Ctrl_Untreated_1_9	Ctrl_Untreated_1	1.0	Purine bases	True	6.815e+06	1.43	1.43	-0.02689
336	Adenosine	Nucleotide	1	Ctrl_Untreated_1_9	Ctrl_Untreated_1	1.0	Purine nucleosides	True	2.044e+07	3.015	3.015	-0.4083
722	ADP-Ribose	Cofactor	1	Ctrl_Untreated_1_9	Ctrl_Untreated_1	1.0	Derivatives of NA, nicotinamide and NAD	True	6.933e+05	-1.867	-1.867	1.135
383	Cytosine	Nucleotide	1	Ctrl_Untreated_1_9	Ctrl_Untreated_1	0.571428571428571	Pyrimidine bases	True	2.105e+05	-3.586	-3.586	1.269
3	Glucosamine 6-P	Carbon	1	Ctrl_Untreated_1_9	Ctrl_Untreated_1	1.0	Aminosugar biosynthesis	True	1.642e+06	-0.6235	-0.6235	1.044
717	Nicotinamide	Cofactor	1	Ctrl_Untreated_1_9	Ctrl_Untreated_1	1.0	NAD biosynthesis	True	6e+07	4.568	4.568	0.05309
51	PEP	Carbon	1	Ctrl_Untreated_1_9	Ctrl_Untreated_1	1.0	Glycolysis, GNG	True	5.083e+06	1.007	1.007	-0.04402
237	Spermine	Amino acid	1	Ctrl_Untreated_1_9	Ctrl_Untreated_1	1.0	Polyamines	True	1.719e+05	-3.879	-3.879	-2.388
385	Uracil	Nucleotide	1	Ctrl_Untreated_1_9	Ctrl_Untreated_1	1.0	Pyrimidine bases	True	5.685e+05	-2.153	-2.153	-2.418
377	Uridine	Nucleotide	1	Ctrl_Untreated_1_9	Ctrl_Untreated_1	1.0	Pyrimidine nucleosides	True	1.973e+07	2.964	2.964	-1.085
348	Allantoin	Nucleotide	1	Ctrl_Untreated_1_9	Ctrl_Untreated_1	1.0	Purine degradation	True	3.592e+06	0.5063	0.5063	0.6819
335	Inosine	Nucleotide	1	Ctrl_Untreated_1_9	Ctrl_Untreated_1	1.0	Purine nucleosides	True	4.909e+07	4.279	4.279	-1.22
81	Ile	Amino acid	1	Ctrl_Untreated_1_9	Ctrl_Untreated_1	1.0	Proteinogenic amino acids	True	1.107e+09	8.773	8.773	0.05816
72	Ala	Amino acid	1	Ctrl_Untreated_1_9	Ctrl_Untreated_1	1.0	Proteinogenic amino acids	True	1.012e+09	8.644	8.644	0.143
79	Thr	Amino acid	1	Ctrl_Untreated_1_9	Ctrl_Untreated_1	1.0	Proteinogenic amino acids	True	4.729e+08	7.547	7.547	-0.4626
88	Tyr	Amino acid	1	Ctrl_Untreated_1_9	Ctrl_Untreated_1	1.0	Proteinogenic amino acids	True	3.541e+08	7.129	7.129	-0.3944
84	Lys	Amino acid	1	Ctrl_Untreated_1_9	Ctrl_Untreated_1	1.0	Proteinogenic amino acids	True	5.812e+08	7.844	7.844	-0.1458
86	Met	Amino acid	1	Ctrl_Untreated_1_9	Ctrl_Untreated_1	1.0	Proteinogenic amino acids	True	5.337e+08	7.721	7.721	-0.5065
61	Malate	Carbon	1	Ctrl_Untreated_1_9	Ctrl_Untreated_1	1.0	TCA cycle	True	4.976e+08	7.62	7.62	0.4486
235	Putrescine	Amino acid	1	Ctrl_Untreated_1_9	Ctrl_Untreated_1	1.0	Polyamines	True	6.34e+06	1.326	1.326	1.817
324	2'-dU	Nucleotide	1	Ctrl_Untreated_1_9	Ctrl_Untreated_1	0.714285714285714	Deoxy-nucleosides	True	6.503e+04	-5.281	-5.281	-0.9009
49	3-P-Glycerate	Carbon	1	Ctrl_Untreated_1_9	Ctrl_Untreated_1	1.0	Glycolysis, GNG	True	1.93e+07	2.932	2.932	-0.3337
189	Kynurenate	Amino acid	1	Ctrl_Untreated_1_9	Ctrl_Untreated_1	0.714285714285714	Amino acid derivatives	True	9.231e+04	-4.776	-4.776	1.022
234	5-Me-Thioadenosine	Amino acid	1	Ctrl_Untreated_1_9	Ctrl_Untreated_1	1.0	SAM metabolism	True	1.324e+07	2.389	2.389	0.5032
59	Succinate	Carbon	1	Ctrl_Untreated_1_9	Ctrl_Untreated_1	1.0	TCA cycle	True	4.894e+06	0.9523	0.9523	0.6513

Metabolite ID	Name	Super Pathway	Dataset	Sample ID	Group ID	Detection Fraction	Pathway	Detected	Raw Intensity	Log2 Norm Intensity	Norm Imputed	Log2 Ctrl Norm
36	Ribose	Carbon	1	Ctrl_Untreated_1_9	Ctrl_Untreated_1	1.0	Sugars and sugar alcohols	True	9.138e+05	-1.469	-1.469	-0.2351
133	Ornithine	Amino acid	1	Ctrl_Untreated_1_9	Ctrl_Untreated_1	1.0	Amino acids degradation intermediates	True	1.035e+08	5.355	5.355	0.8757
313	5-Oxoproline	Amino acid	1	Ctrl_Untreated_1_9	Ctrl_Untreated_1	1.0	Glutathione derivatives	True	1.869e+07	2.886	2.886	0.7981
165	N-6-Tri-Me-Lys	Amino acid	1	Ctrl_Untreated_1_9	Ctrl_Untreated_1	1.0	Amino acid derivatives	True	2.743e+07	3.439	3.439	-0.3246
380	Orotate	Nucleotide	1	Ctrl_Untreated_1_9	Ctrl_Untreated_1	0.857142857142857	Pyrimidine (UMP) biosynthesis	True	2.204e+05	-3.52	-3.52	0.1097
724	Pantothenate	Cofactor	1	Ctrl_Untreated_1_9	Ctrl_Untreated_1	1.0	Coenzyme A biosynthesis	True	3.67e+08	7.181	7.181	0.8709
150	N-Me-Gly	Amino acid	1	Ctrl_Untreated_1_9	Ctrl_Untreated_1	1.0	Amino acid derivatives	True	2.277e+06	-0.1515	-0.1515	0.1489
122	3-OH-Isobutyrate	Amino acid	1	Ctrl_Untreated_1_9	Ctrl_Untreated_1	0.857142857142857	Amino acids degradation intermediates	True	4.897e+05	-2.369	-2.369	0.7874
241	4-Acetamidobutanoate	Amino acid	1	Ctrl_Untreated_1_9	Ctrl_Untreated_1	1.0	Polyamine derivatives	True	6.549e+06	1.373	1.373	0.9317
711	alpha-Tocopherol	Cofactor	1	Ctrl_Untreated_1_9	Ctrl_Untreated_1	1.0	Cofactors	True	3.875e+06	0.6155	0.6155	-0.1648
55	Citrate	Carbon	1	Ctrl_Untreated_1_9	Ctrl_Untreated_1	1.0	TCA cycle	True	7.783e+06	1.622	1.622	-0.3272
387	3-Aminoisobutyrate	Nucleotide	1	Ctrl_Untreated_1_9	Ctrl_Untreated_1	0.857142857142857	Pyrimidine degradation	True	4.909e+05	-2.365	-2.365	-0.3514
338	Guanosine	Nucleotide	1	Ctrl_Untreated_1_9	Ctrl_Untreated_1	1.0	Purine nucleosides	True	4.138e+07	4.032	4.032	-1.074
209	N-Ac-Ala	Amino acid	1	Ctrl_Untreated_1_9	Ctrl_Untreated_1	1.0	N-acetylated amino acids	True	6.838e+05	-1.887	-1.887	-0.6857
221	N-Ac-Met	Amino acid	1	Ctrl_Untreated_1_9	Ctrl_Untreated_1	1.0	N-acetylated amino acids	True	4.551e+06	0.8476	0.8476	-0.8966
228	N-Ac-Val	Amino acid	1	Ctrl_Untreated_1_9	Ctrl_Untreated_1	0.571428571428571	N-acetylated amino acids	True	7.379e+04	-5.099	-5.099	0.2717
346	Urate	Nucleotide	1	Ctrl_Untreated_1_9	Ctrl_Untreated_1	1.0	Purine degradation	True	1.835e+06	-0.4628	-0.4628	0.6
90	Arg	Amino acid	1	Ctrl_Untreated_1_9	Ctrl_Untreated_1	1.0	Proteinogenic amino acids	True	1.637e+09	9.338	9.338	-0.3678
60	Fumarate	Carbon	1	Ctrl_Untreated_1_9	Ctrl_Untreated_1	1.0	TCA cycle	True	7.285e+06	1.526	1.526	0.5112
78	Ser	Amino acid	1	Ctrl_Untreated_1_9	Ctrl_Untreated_1	1.0	Proteinogenic amino acids	True	2.158e+08	6.415	6.415	-0.8425
83	Val	Amino acid	1	Ctrl_Untreated_1_9	Ctrl_Untreated_1	1.0	Proteinogenic amino acids	True	4.884e+08	7.593	7.593	-0.2789
734	Pyridoxal	Cofactor	1	Ctrl_Untreated_1_9	Ctrl_Untreated_1	1.0	PLP biosynthesis and salvage	True	6.678e+06	1.401	1.401	-0.3823

Metabolite ID	Name	Super Pathway	Dataset	Sample ID	Group ID	Detection Fraction	Pathway	Detected	Raw Intensity	Log2 Norm Intensity	Norm Imputed	Log2 Ctrl Norm
136	Urea	Amino acid	1	Ctrl_Untreated_1_9	Ctrl_Untreated_1	0.857142857142857	Amino acids degradation intermediates	True	5.046e+06	0.9965	0.9965	0.8453
67	Ribose 1-P	Carbon	1	Ctrl_Untreated_1_9	Ctrl_Untreated_1	1.0	Pentose phosphate pathway (PPP)	True	3.811e+06	0.5914	0.5914	-0.02977
284	Carnosine	Amino acid	1	Ctrl_Untreated_1_9	Ctrl_Untreated_1	1.0	Dipeptides	True	6.682e+05	-1.92	-1.92	0.188
306	gamma-Glu-Cys	Amino acid	1	Ctrl_Untreated_1_9	Ctrl_Untreated_1	1.0	Glutathione biosynthesis	True	1.747e+06	-0.5337	-0.5337	-0.1969
712	Retinol (Vit A)	Cofactor	1	Ctrl_Untreated_1_9	Ctrl_Untreated_1	1.0	Cofactors	True	4.415e+05	-2.518	-2.518	-0.08475
85	Cys	Amino acid	1	Ctrl_Untreated_1_9	Ctrl_Untreated_1	1.0	Proteinogenic amino acids	True	4.198e+07	4.053	4.053	-0.4606
91	Pro	Amino acid	1	Ctrl_Untreated_1_9	Ctrl_Untreated_1	1.0	Proteinogenic amino acids	True	1.795e+09	9.471	9.471	0.06293
308	Glutathione, Reduced	Amino acid	1	Ctrl_Untreated_1_9	Ctrl_Untreated_1	1.0	Glutathione	True	3.127e+08	6.95	6.95	-5.97e-03
107	Citrulline	Amino acid	1	Ctrl_Untreated_1_9	Ctrl_Untreated_1	1.0	Amino acids biosynthesis intermediates	True	2.683e+07	3.407	3.407	0.5827
328	IMP	Nucleotide	1	Ctrl_Untreated_1_9	Ctrl_Untreated_1	0.285714285714286	Purine nucleotides	True	1.094e+05	-4.53	-4.53	-0.2088
706	FAD	Cofactor	1	Ctrl_Untreated_1_9	Ctrl_Untreated_1	1.0	Cofactors	True	4.863e+05	-2.379	-2.379	-0.189
735	Pyridoxamine	Cofactor	1	Ctrl_Untreated_1_9	Ctrl_Untreated_1	1.0	PLP biosynthesis and salvage	True	2.784e+06	0.1388	0.1388	0.3318
199	Serotonin	Amino acid	1	Ctrl_Untreated_1_9	Ctrl_Untreated_1	1.0	Amino acid derivatives	True	7.819e+06	1.628	1.628	0.3557
370	CMP	Nucleotide	1	Ctrl_Untreated_1_9	Ctrl_Untreated_1	1.0	Pyrimidine nucleotides	True	1.289e+07	2.35	2.35	0.5109
287	gamma-Glu-Gln	Amino acid	1	Ctrl_Untreated_1_9	Ctrl_Untreated_1	1.0	Gamma-glutamyl dipeptides	True	2.196e+06	-0.2036	-0.2036	-1.045
14	UDP-Glucuronate	Carbon	1	Ctrl_Untreated_1_9	Ctrl_Untreated_1	0.857142857142857	Polysaccharide biosynthesis	True	2.601e+06	0.04047	0.04047	1.99
229	N-Formyl-Met	Amino acid	1	Ctrl_Untreated_1_9	Ctrl_Untreated_1	1.0	N-formylated amino acids	True	2.093e+05	-3.595	-3.595	0.2274
350	3',5'-cAMP	Nucleotide	1	Ctrl_Untreated_1_9	Ctrl_Untreated_1	1.0	Purine derivatives in signaling	True	1.772e+05	-3.835	-3.835	-0.2156
371	CDP	Nucleotide	1	Ctrl_Untreated_1_9	Ctrl_Untreated_1	0.571428571428571	Pyrimidine nucleotides	True	1.138e+06	-1.152	-1.152	2.168
372	CTP	Nucleotide	1	Ctrl_Untreated_1_9	Ctrl_Untreated_1	0.571428571428571	Pyrimidine nucleotides	True	4.783e+05	-2.403	-2.403	1.251
333	GDP	Nucleotide	1	Ctrl_Untreated_1_9	Ctrl_Untreated_1	0.571428571428571	Purine nucleotides	True	1.794e+06	-0.4951	-0.4951	2.005
332	GMP	Nucleotide	1	Ctrl_Untreated_1_9	Ctrl_Untreated_1	1.0	Purine nucleotides	True	1.449e+07	2.519	2.519	1.82

Metabolite ID	Name	Super Pathway	Dataset	Sample ID	Group ID	Detection Fraction	Pathway	Detected	Raw Intensity	Log2 Norm Intensity	Norm Imputed	Log2 Ctrl Norm
373	UMP	Nucleotide	1	Ctrl_Untreated_1_9	Ctrl_Untreated_1	1.0	Pyrimidine nucleotides	True	7.283e+06	1.526	1.526	2.581
389	3'-CMP	Nucleotide	1	Ctrl_Untreated_1_9	Ctrl_Untreated_1	1.0	Pyrimidine derivatives in signaling	True	4.769e+05	-2.407	-2.407	-1.355
330	ADP	Nucleotide	1	Ctrl_Untreated_1_9	Ctrl_Untreated_1	0.714285714285714	Purine nucleotides	True	9.148e+06	1.855	1.855	2.151
342	Hypoxanthine	Nucleotide	1	Ctrl_Untreated_1_9	Ctrl_Untreated_1	1.0	Purine bases	True	1.913e+07	2.919	2.919	-1.02
736	Pyridoxamine-P	Cofactor	1	Ctrl_Untreated_1_9	Ctrl_Untreated_1	1.0	PLP biosynthesis and salvage	True	5.297e+05	-2.255	-2.255	0.7229
148	Betaine	Amino acid	1	Ctrl_Untreated_1_9	Ctrl_Untreated_1	1.0	Amino acid derivatives	True	1.077e+08	5.413	5.413	0.6071
344	Xanthine	Nucleotide	1	Ctrl_Untreated_1_9	Ctrl_Untreated_1	1.0	Purine bases	True	5.211e+06	1.043	1.043	0.1354
386	3-Ureidopropionate	Nucleotide	1	Ctrl_Untreated_1_9	Ctrl_Untreated_1	1.0	Pyrimidine degradation	True	2.529e+06	-4.76e-05	-4.76e-05	0.9193
149	DiMe-Gly	Amino acid	1	Ctrl_Untreated_1_9	Ctrl_Untreated_1	1.0	Amino acid derivatives	True	2.868e+06	0.1816	0.1816	0.7018
703	NAD+	Cofactor	1	Ctrl_Untreated_1_9	Ctrl_Untreated_1	1.0	Cofactors	True	1.767e+07	2.805	2.805	0.06831
709	Pyridoxal-P	Cofactor	1	Ctrl_Untreated_1_9	Ctrl_Untreated_1	1.0	Cofactors	True	1.031e+06	-1.295	-1.295	1.013
731	Thiamin (Vitamin B1)	Cofactor	1	Ctrl_Untreated_1_9	Ctrl_Untreated_1	1.0	TPP biosynthesis	True	5.17e+06	1.032	1.032	0.284
374	UDP	Nucleotide	1	Ctrl_Untreated_1_9	Ctrl_Untreated_1	0.714285714285714	Pyrimidine nucleotides	True	8.162e+06	1.69	1.69	3.244
102	2-Aminoadipate	Amino acid	1	Ctrl_Untreated_1_9	Ctrl_Untreated_1	1.0	Amino acids biosynthesis intermediates	True	9.004e+05	-1.49	-1.49	0.6196
45	Fructose-6-P	Carbon	1	Ctrl_Untreated_1_9	Ctrl_Untreated_1	1.0	Glycolysis, GNG	True	3.974e+06	0.6519	0.6519	0.5766
320	TMP	Nucleotide	1	Ctrl_Untreated_1_9	Ctrl_Untreated_1	0.571428571428571	Deoxy-nucleotides	True	3.95e+05	-2.679	-2.679	2.113
341	XMP	Nucleotide	1	Ctrl_Untreated_1_9	Ctrl_Untreated_1	0.857142857142857	IMP conversion to AMP & GMP	True	1.621e+05	-3.964	-3.964	0.6105
120	beta-OH-Isovalerate	Amino acid	1	Ctrl_Untreated_1_9	Ctrl_Untreated_1	0.857142857142857	Amino acids degradation intermediates	True	3.004e+05	-3.073	-3.073	0.09198
322	2'-dI	Nucleotide	1	Ctrl_Untreated_1_9	Ctrl_Untreated_1	0.857142857142857	Deoxy-nucleosides	True	4.187e+04	-5.917	-5.917	-1.791
4	GlcNAc 6-P	Carbon	1	Ctrl_Untreated_1_9	Ctrl_Untreated_1	1.0	Aminosugar biosynthesis	True	1.539e+06	-0.7165	-0.7165	-1.045
337	Xanthosine	Nucleotide	1	Ctrl_Untreated_1_9	Ctrl_Untreated_1	1.0	Purine nucleosides	True	3.251e+05	-2.96	-2.96	0.1146
188	Kynurenine	Amino acid	1	Ctrl_Untreated_1_9	Ctrl_Untreated_1	1.0	Amino acid derivatives	True	1.94e+06	-0.3827	-0.3827	0.3089

Metabolite ID	Name	Super Pathway	Dataset	Sample ID	Group ID	Detection Fraction	Pathway	Detected	Raw Intensity	Log2 Norm Intensity	Norm Imputed	Log2 Ctrl Norm
63	6-P-Gluconate	Carbon	1	Ctrl_Untreated_1_9	Ctrl_Untreated_1	1.0	Pentose phosphate pathway (PPP)	True	8.605e+06	1.767	1.767	-0.2671
40	Glucuronate	Carbon	1	Ctrl_Untreated_1_9	Ctrl_Untreated_1	1.0	Sugars and sugar alcohols	True	8.199e+05	-1.625	-1.625	-0.08599
108	Argininosuccinate	Amino acid	1	Ctrl_Untreated_1_9	Ctrl_Untreated_1	1.0	Amino acids biosynthesis intermediates	True	2.011e+06	-0.3308	-0.3308	-0.1046
710	Carnitine	Cofactor	1	Ctrl_Untreated_1_9	Ctrl_Untreated_1	1.0	Cofactors	True	9.948e+07	5.298	5.298	-0.6798
725	P-Pantetheine	Cofactor	1	Ctrl_Untreated_1_9	Ctrl_Untreated_1	1.0	Coenzyme A biosynthesis	True	2.272e+05	-3.477	-3.477	0.228
48	DHAP	Carbon	1	Ctrl_Untreated_1_9	Ctrl_Untreated_1	1.0	Glycolysis, GNG	True	2.876e+07	3.507	3.507	0.4109
17	Maltose	Carbon	1	Ctrl_Untreated_1_9	Ctrl_Untreated_1	1.0	Glycogen degradation	True	2.614e+06	0.04759	0.04759	1.34
359	N1-Me-Adenosine	Nucleotide	1	Ctrl_Untreated_1_9	Ctrl_Untreated_1	0.857142857142857	Purine derivatives in RNAs	True	9.734e+05	-1.377	-1.377	-0.3398
159	3-Me-His	Amino acid	1	Ctrl_Untreated_1_9	Ctrl_Untreated_1	1.0	Amino acid derivatives	True	3.437e+05	-2.879	-2.879	1.356
155	4-Guanidinobutanoate	Amino acid	1	Ctrl_Untreated_1_9	Ctrl_Untreated_1	1.0	Amino acid derivatives	True	1.28e+06	-0.9827	-0.9827	0.4513
164	5-OH-Lys	Amino acid	1	Ctrl_Untreated_1_9	Ctrl_Untreated_1	1.0	Amino acid derivatives	True	1.083e+06	-1.224	-1.224	0.5466
357	Adenosine-3',5'-PP	Nucleotide	1	Ctrl_Untreated_1_9	Ctrl_Untreated_1	0.857142857142857	Purine byproducts of metabolic processes	True	2.218e+05	-3.511	-3.511	-0.03376
104	Cystathionine	Amino acid	1	Ctrl_Untreated_1_9	Ctrl_Untreated_1	1.0	Amino acids biosynthesis intermediates	True	1.766e+07	2.803	2.803	0.594
113	Imidazole Lactate	Amino acid	1	Ctrl_Untreated_1_9	Ctrl_Untreated_1	0.857142857142857	Amino acids degradation intermediates	True	3.179e+05	-2.992	-2.992	0.6845
215	N-Ac-Glu	Amino acid	1	Ctrl_Untreated_1_9	Ctrl_Untreated_1	1.0	N-acetylated amino acids	True	4.162e+06	0.7186	0.7186	-1.025
310	S-Lactoyl-Glutathione	Amino acid	1	Ctrl_Untreated_1_9	Ctrl_Untreated_1	0.857142857142857	Glutathione derivatives	True	4.058e+06	0.6823	0.6823	0.3259
5	GlcNAc 1-P	Carbon	1	Ctrl_Untreated_1_9	Ctrl_Untreated_1	1.0	Aminosugar biosynthesis	True	3.926e+05	-2.688	-2.688	-0.7766
34	Ribitol	Carbon	1	Ctrl_Untreated_1_9	Ctrl_Untreated_1	1.0	Sugars and sugar alcohols	True	3.044e+05	-3.055	-3.055	0.3754
10	UDP-Galactose	Carbon	1	Ctrl_Untreated_1_9	Ctrl_Untreated_1	0.857142857142857	Polysaccharide biosynthesis	True	2.636e+06	0.05976	0.05976	0.6862
13	Guanosine 5'-PP-Fucose	Carbon	1	Ctrl_Untreated_1_9	Ctrl_Untreated_1	1.0	Polysaccharide biosynthesis	True	6.39e+05	-1.985	-1.985	0.3146

Metabolite ID	Name	Super Pathway	Dataset	Sample ID	Group ID	Detection Fraction	Pathway	Detected	Raw Intensity	Log2 Norm Intensity	Norm Imputed	Log2 Ctrl Norm
19	Maltotetraose	Carbon	1	Ctrl_Untreated_1_9	Ctrl_Untreated_1	1.0	Glycogen degradation	True	6.586e+06	1.381	1.381	1.425
233	SAM	Amino acid	1	Ctrl_Untreated_1_9	Ctrl_Untreated_1	1.0	SAM metabolism	True	2.993e+06	0.243	0.243	0.3993
129	5-Aminovalerate	Amino acid	1	Ctrl_Untreated_1_9	Ctrl_Untreated_1	1.0	Amino acids degradation intermediates	True	9.206e+06	1.864	1.864	0.8624
741	5-Me-THF	Cofactor	1	Ctrl_Untreated_1_9	Ctrl_Untreated_1	0.714285714285714	Folate metabolism	True	9.783e+04	-4.692	-4.692	0.06571
198	Indolelactate	Amino acid	1	Ctrl_Untreated_1_9	Ctrl_Untreated_1	0.857142857142857	Amino acid derivatives	True	3.464e+05	-2.868	-2.868	0.9191
254	Gly-Val	Amino acid	1	Ctrl_Untreated_1_9	Ctrl_Untreated_1	1.0	Dipeptides	True	2.91e+06	0.2023	0.2023	-0.9091
291	gamma-Glu-Leu	Amino acid	1	Ctrl_Untreated_1_9	Ctrl_Untreated_1	1.0	Gamma-glutamyl dipeptides	True	1.121e+06	-1.174	-1.174	-0.6848
173	Met Sulfoxide	Amino acid	1	Ctrl_Untreated_1_9	Ctrl_Untreated_1	1.0	Amino acid derivatives	True	1.303e+07	2.366	2.366	-0.2016
43	Glucose	Carbon	1	Ctrl_Untreated_1_9	Ctrl_Untreated_1	1.0	Glycolysis, GNG	True	9.146e+07	5.177	5.177	0.1251
185	Phenyllactate	Amino acid	1	Ctrl_Untreated_1_9	Ctrl_Untreated_1	0.285714285714286	Amino acid derivatives	True	6.12e+04	-5.369	-5.369	0.4428
156	Homo-Arg	Amino acid	1	Ctrl_Untreated_1_9	Ctrl_Untreated_1	1.0	Amino acid derivatives	True	8.507e+06	1.75	1.75	0.5696
135	Homocitrulline	Amino acid	1	Ctrl_Untreated_1_9	Ctrl_Untreated_1	1.0	Amino acids degradation intermediates	True	1.293e+06	-0.9683	-0.9683	0.7342
719	Nicotinamide MN	Cofactor	1	Ctrl_Untreated_1_9	Ctrl_Untreated_1	1.0	NAD biosynthesis	True	3.875e+05	-2.706	-2.706	-2.892
212	N-Ac-Asp	Amino acid	1	Ctrl_Untreated_1_9	Ctrl_Untreated_1	1.0	N-acetylated amino acids	True	4.045e+06	0.6777	0.6777	-0.01967
720	1-Me-Nicotinamide	Cofactor	1	Ctrl_Untreated_1_9	Ctrl_Untreated_1	1.0	Derivatives of NA, nicotinamide and NAD	True	8.046e+08	8.314	8.314	0.423
216	N-Ac-Gly	Amino acid	1	Ctrl_Untreated_1_9	Ctrl_Untreated_1	0.714285714285714	N-acetylated amino acids	True	4.363e+05	-2.535	-2.535	1.005
70	Creatine	Carbon	1	Ctrl_Untreated_1_9	Ctrl_Untreated_1	1.0	Creatine energy storage	True	2.015e+09	9.638	9.638	0.2207
26	Galactonate	Carbon	1	Ctrl_Untreated_1_9	Ctrl_Untreated_1	0.857142857142857	Sugars and sugar alcohols	True	2.423e+06	-0.06199	-0.06199	0.7426
309	Glutathione, Oxidized	Amino acid	1	Ctrl_Untreated_1_9	Ctrl_Untreated_1	1.0	Glutathione	True	5.4e+06	1.094	1.094	-0.09834
35	Ribonate	Carbon	1	Ctrl_Untreated_1_9	Ctrl_Untreated_1	1.0	Sugars and sugar alcohols	True	2.014e+06	-0.3285	-0.3285	0.1987
160	1-Me-His	Amino acid	1	Ctrl_Untreated_1_9	Ctrl_Untreated_1	1.0	Amino acid derivatives	True	5.237e+07	4.372	4.372	0.931

Metabolite ID	Name	Super Pathway	Dataset	Sample ID	Group ID	Detection Fraction	Pathway	Detected	Raw Intensity	Log2 Norm Intensity	Norm Imputed	Log2 Ctrl Norm
44	Glucose 6-P	Carbon	1	Ctrl_Untreated_1_9	Ctrl_Untreated_1	0.857142857142857	Glycolysis, GNG	True	1.864e+06	-0.44	-0.44	1.037
704	NADH	Cofactor	1	Ctrl_Untreated_1_9	Ctrl_Untreated_1	1.0	Cofactors	True	2.529e+06	4.76e-05	4.76e-05	0.6461
275	Thr-Phe	Amino acid	1	Ctrl_Untreated_1_9	Ctrl_Untreated_1	0.857142857142857	Dipeptides	True	6.123e+05	-2.046	-2.046	-0.393
738	Pyridoxate	Cofactor	1	Ctrl_Untreated_1_9	Ctrl_Untreated_1	1.0	PLP biosynthesis and salvage	True	3.782e+05	-2.741	-2.741	0.6229
177	3-(4-OH-Phenyl)Lactate	Amino acid	1	Ctrl_Untreated_1_9	Ctrl_Untreated_1	1.0	Amino acid derivatives	True	8.773e+05	-1.527	-1.527	0.9378
206	Trans-4-OH-Pro	Amino acid	1	Ctrl_Untreated_1_9	Ctrl_Untreated_1	1.0	Amino acid derivatives	True	1.169e+08	5.531	5.531	0.3758
329	AMP	Nucleotide	1	Ctrl_Untreated_1_9	Ctrl_Untreated_1	1.0	Purine nucleotides	True	1.187e+08	5.553	5.553	1.918
11	UDP-Glucose	Carbon	1	Ctrl_Untreated_1_9	Ctrl_Untreated_1	1.0	Polysaccharide biosynthesis	True	9.502e+06	1.91	1.91	2.015
158	4-Imidazole-Ac	Amino acid	1	Ctrl_Untreated_1_9	Ctrl_Untreated_1	1.0	Amino acid derivatives	True	8.796e+05	-1.524	-1.524	0.6229
111	1-Me-Imidazole-Ac	Amino acid	1	Ctrl_Untreated_1_9	Ctrl_Untreated_1	1.0	Amino acids degradation intermediates	True	1.069e+06	-1.242	-1.242	0.692
345	Guanine	Nucleotide	1	Ctrl_Untreated_1_9	Ctrl_Untreated_1	1.0	Purine bases	True	3.731e+07	3.883	3.883	-1.103
22	N-Ac-Neuraminate	Carbon	1	Ctrl_Untreated_1_9	Ctrl_Untreated_1	1.0	Aminosugar derivatives	True	2.243e+06	-0.1731	-0.1731	-0.03775
721	N'-Methylnicotinate	Cofactor	1	Ctrl_Untreated_1_9	Ctrl_Untreated_1	1.0	Derivatives of NA, nicotinamide and NAD	True	1.377e+06	-0.8773	-0.8773	0.1303
183	Phenol Sulfate	Amino acid	1	Ctrl_Untreated_1_9	Ctrl_Untreated_1	0.857142857142857	Amino acid derivatives	True	4.196e+05	-2.592	-2.592	2.049
718	Nicotinamide Riboside	Cofactor	1	Ctrl_Untreated_1_9	Ctrl_Untreated_1	1.0	NAD biosynthesis	True	8.48e+05	-1.577	-1.577	-3.167
297	gamma-Glu-Thr	Amino acid	1	Ctrl_Untreated_1_9	Ctrl_Untreated_1	1.0	Gamma-glutamyl dipeptides	True	1.527e+06	-0.7277	-0.7277	-1.368
295	gamma-Glu-Phe	Amino acid	1	Ctrl_Untreated_1_9	Ctrl_Untreated_1	0.428571428571429	Gamma-glutamyl dipeptides	True	4.021e+04	-5.975	-5.975	-0.1899
347	Allantoic Acid	Nucleotide	1	Ctrl_Untreated_1_9	Ctrl_Untreated_1	0.571428571428571	Purine degradation	True	1.141e+05	-4.47	-4.47	1.169
399	Pseudouridine	Nucleotide	1	Ctrl_Untreated_1_9	Ctrl_Untreated_1	1.0	Pyrimidine derivatives in RNAs	True	7.812e+05	-1.695	-1.695	0.3352
375	UTP	Nucleotide	1	Ctrl_Untreated_1_9	Ctrl_Untreated_1	0.571428571428571	Pyrimidine nucleotides	True	4.576e+06	0.8557	0.8557	2.832
144	Glu, gamma-Me Ester	Amino acid	1	Ctrl_Untreated_1_9	Ctrl_Untreated_1	1.0	Amino acid derivatives	True	4.077e+06	0.689	0.689	0.1112

Metabolite ID	Name	Super Pathway	Dataset	Sample ID	Group ID	Detection Fraction	Pathway	Detected	Raw Intensity	Log2 Norm Intensity	Norm Imputed	Log2 Ctrl Norm
292	gamma-Glu-epsilon-Lysine	Amino acid	1	Ctrl_Untreated_1_9	Ctrl_Untreated_1	1.0	Gamma-glutamyl dipeptides	True	2.035e+06	-0.3133	-0.3133	-0.122
225	N-Ac-Thr	Amino acid	1	Ctrl_Untreated_1_9	Ctrl_Untreated_1	1.0	N-acetylated amino acids	True	6.267e+05	-2.013	-2.013	-0.5101
211	N-Ac-Asn	Amino acid	1	Ctrl_Untreated_1_9	Ctrl_Untreated_1	1.0	N-acetylated amino acids	True	6.259e+05	-2.015	-2.015	0.6707
151	Phenylacetylglycine	Amino acid	1	Ctrl_Untreated_1_9	Ctrl_Untreated_1	1.0	Amino acid derivatives	True	1.603e+06	-0.6578	-0.6578	0.1291
217	N-Ac-His	Amino acid	1	Ctrl_Untreated_1_9	Ctrl_Untreated_1	1.0	N-acetylated amino acids	True	2.282e+05	-3.47	-3.47	0.01463
288	gamma-Glu-Gly	Amino acid	1	Ctrl_Untreated_1_9	Ctrl_Untreated_1	0.571428571428571	Gamma-glutamyl dipeptides	False			-3.092	-1.268
222	N-Ac-Phe	Amino acid	1	Ctrl_Untreated_1_9	Ctrl_Untreated_1	0.571428571428571	N-acetylated amino acids	True	5.223e+04	-5.598	-5.598	0.6749
71	Creatine-P	Carbon	1	Ctrl_Untreated_1_9	Ctrl_Untreated_1	1.0	Creatine energy storage	True	1.976e+05	-3.678	-3.678	0.577
210	N-Ac-Arg	Amino acid	1	Ctrl_Untreated_1_9	Ctrl_Untreated_1	1.0	N-acetylated amino acids	True	4.23e+05	-2.58	-2.58	0.09591
218	N-Ac-Ile	Amino acid	1	Ctrl_Untreated_1_9	Ctrl_Untreated_1	0.428571428571429	N-acetylated amino acids	True	7.939e+04	-4.993	-4.993	0.9725
251	Gly-Leu	Amino acid	1	Ctrl_Untreated_1_9	Ctrl_Untreated_1	1.0	Dipeptides	True	4.874e+06	0.9464	0.9464	-0.6923
290	gamma-Glu-Ile	Amino acid	1	Ctrl_Untreated_1_9	Ctrl_Untreated_1	1.0	Gamma-glutamyl dipeptides	True	6.774e+05	-1.901	-1.901	-0.5619
316	Ophthalmate	Amino acid	1	Ctrl_Untreated_1_9	Ctrl_Untreated_1	1.0	Oxidative stress markers	True	3.382e+06	0.4194	0.4194	-1.923
125	Isovaleryl-Gly	Amino acid	1	Ctrl_Untreated_1_9	Ctrl_Untreated_1	0.857142857142857	Amino acids degradation intermediates	True	6.92e+04	-5.192	-5.192	0.218
368	7-Me-Guanine	Nucleotide	1	Ctrl_Untreated_1_9	Ctrl_Untreated_1	1.0	Purine derivatives in RNAs	True	8.002e+05	-1.66	-1.66	0.5299
208	Pro-OH-Pro	Amino acid	1	Ctrl_Untreated_1_9	Ctrl_Untreated_1	1.0	Amino acid derivatives	True	4.43e+07	4.131	4.131	0.8014
366	N2,N2-DiMe-Guanosine	Nucleotide	1	Ctrl_Untreated_1_9	Ctrl_Untreated_1	0.857142857142857	Purine derivatives in RNAs	True	1.608e+05	-3.975	-3.975	0.1385
352	3'-AMP	Nucleotide	1	Ctrl_Untreated_1_9	Ctrl_Untreated_1	1.0	Purine derivatives in signaling	True	4.636e+05	-2.448	-2.448	-0.9608
363	N6-Carbamoyl-Thr-Adenosine	Nucleotide	1	Ctrl_Untreated_1_9	Ctrl_Untreated_1	0.857142857142857	Purine derivatives in RNAs	True	1.472e+05	-4.103	-4.103	0.5828
314	Cys-Glutathione Disulfide	Amino acid	1	Ctrl_Untreated_1_9	Ctrl_Untreated_1	1.0	Oxidative stress markers	True	8.976e+04	-4.816	-4.816	-1.532
382	Orotidine	Nucleotide	1	Ctrl_Untreated_1_9	Ctrl_Untreated_1	0.428571428571429	Pyrimidine (UMP) biosynthesis	False			-5.287	-0.9952

Metabolite ID	Name	Super Pathway	Dataset	Sample ID	Group ID	Detection Fraction	Pathway	Detected	Raw Intensity	Log2 Norm Intensity	Norm Imputed	Log2 Ctrl Norm
307	Cys-Gly	Amino acid	1	Ctrl_Untreated_1_9	Ctrl_Untreated_1	1.0	Glutathione biosynthesis	True	6.221e+06	1.299	1.299	-0.3085
64	Sedoheptulose-7-P	Carbon	1	Ctrl_Untreated_1_9	Ctrl_Untreated_1	1.0	Pentose phosphate pathway (PPP)	True	2.686e+06	0.08692	0.08692	-0.06717
142	N-Ac-Asp-Glu	Amino acid	1	Ctrl_Untreated_1_9	Ctrl_Untreated_1	1.0	Amino acid derivatives	True	1.205e+06	-1.07	-1.07	0.07557
708	Thiamin-PP	Cofactor	1	Ctrl_Untreated_1_9	Ctrl_Untreated_1	0.571428571428571	Cofactors	True	5.143e+04	-5.62	-5.62	0.3638
182	P-Cresol Sulfate	Amino acid	1	Ctrl_Untreated_1_9	Ctrl_Untreated_1	0.857142857142857	Amino acid derivatives	True	5.247e+05	-2.269	-2.269	0.9272
250	Gly-Ile	Amino acid	1	Ctrl_Untreated_1_9	Ctrl_Untreated_1	1.0	Dipeptides	True	1.017e+06	-1.314	-1.314	2.99e-03
286	gamma-Glu-Glu	Amino acid	1	Ctrl_Untreated_1_9	Ctrl_Untreated_1	1.0	Gamma-glutamyl dipeptides	True	4.054e+06	0.6809	0.6809	0.6631
739	Deoxycarnitine	Cofactor	1	Ctrl_Untreated_1_9	Ctrl_Untreated_1	1.0	Carnitine biosynthesis	True	2.954e+07	3.546	3.546	-0.2052
203	DiMe-Arg	Amino acid	1	Ctrl_Untreated_1_9	Ctrl_Untreated_1	1.0	Amino acid derivatives	True	7.182e+07	4.828	4.828	-0.805
351	2'-AMP	Nucleotide	1	Ctrl_Untreated_1_9	Ctrl_Untreated_1	1.0	Purine derivatives in signaling	True	1.574e+05	-4.006	-4.006	-1.737
8	Cytidine 5'-P-N-Ac-Neuraminate	Carbon	1	Ctrl_Untreated_1_9	Ctrl_Untreated_1	1.0	Aminosugar biosynthesis	True	7.615e+05	-1.732	-1.732	0.0138
285	gamma-Glu-Ala	Amino acid	1	Ctrl_Untreated_1_9	Ctrl_Untreated_1	0.857142857142857	Gamma-glutamyl dipeptides	True	1.766e+05	-3.84	-3.84	-0.6011
224	N-Ac-Ser	Amino acid	1	Ctrl_Untreated_1_9	Ctrl_Untreated_1	1.0	N-acetylated amino acids	True	9.108e+06	1.849	1.849	-0.03066
244	Ala-Leu	Amino acid	1	Ctrl_Untreated_1_9	Ctrl_Untreated_1	1.0	Dipeptides	True	3.512e+06	0.4738	0.4738	-0.6641
207	N-Me-Pro	Amino acid	1	Ctrl_Untreated_1_9	Ctrl_Untreated_1	1.0	Amino acid derivatives	True	2.083e+06	-0.28	-0.28	0.65
171	Cys Sulfinic Acid	Amino acid	1	Ctrl_Untreated_1_9	Ctrl_Untreated_1	0.857142857142857	Amino acid derivatives	True	4.637e+05	-2.447	-2.447	0.85
181	O-Me-Tyr	Amino acid	1	Ctrl_Untreated_1_9	Ctrl_Untreated_1	0.857142857142857	Amino acid derivatives	True	5.536e+05	-2.192	-2.192	1.178
240	N-Ac-Putrescine	Amino acid	1	Ctrl_Untreated_1_9	Ctrl_Untreated_1	1.0	Polyamine derivatives	True	1.759e+06	-0.524	-0.524	1.84
176	S-Me-Met	Amino acid	1	Ctrl_Untreated_1_9	Ctrl_Untreated_1	0.857142857142857	Amino acid derivatives	True	1.302e+06	-0.9577	-0.9577	0.884
339	AICAR	Nucleotide	1	Ctrl_Untreated_1_9	Ctrl_Untreated_1	0.571428571428571	IMP biosynthesis	False			-4.716	-0.4151
141	gamma-Carboxy-Glu	Amino acid	1	Ctrl_Untreated_1_9	Ctrl_Untreated_1	1.0	Amino acid derivatives	True	1.899e+06	-0.413	-0.413	0.372

Metabolite ID	Name	Super Pathway	Dataset	Sample ID	Group ID	Detection Fraction	Pathway	Detected	Raw Intensity	Log2 Norm Intensity	Norm Imputed	Log2 Ctrl Norm
392	3'-UMP	Nucleotide	1	Ctrl_Untreated_1_9	Ctrl_Untreated_1	0.571428571428571	Pyrimidine derivatives in signaling	True	3.272e+05	-2.95	-2.95	1.083
355	3'-GMP	Nucleotide	1	Ctrl_Untreated_1_9	Ctrl_Untreated_1	0.857142857142857	Purine derivatives in signaling	True	5.08e+04	-5.638	-5.638	-1.7
282	Val-Leu	Amino acid	1	Ctrl_Untreated_1_9	Ctrl_Untreated_1	1.0	Dipeptides	True	4.647e+06	0.8777	0.8777	-0.09401
140	Carboxyethyl-GABA	Amino acid	1	Ctrl_Untreated_1_9	Ctrl_Untreated_1	1.0	Amino acid derivatives	True	6.124e+05	-2.046	-2.046	-0.6149
258	Ile-Gly	Amino acid	1	Ctrl_Untreated_1_9	Ctrl_Untreated_1	1.0	Dipeptides	True	1.985e+07	2.973	2.973	-0.3393
260	Leu-Ala	Amino acid	1	Ctrl_Untreated_1_9	Ctrl_Untreated_1	1.0	Dipeptides	True	4.888e+06	0.9508	0.9508	-0.3504
265	Lys-Leu	Amino acid	1	Ctrl_Untreated_1_9	Ctrl_Untreated_1	0.857142857142857	Dipeptides	False			-3.814	-1.203
263	Leu-Gly	Amino acid	1	Ctrl_Untreated_1_9	Ctrl_Untreated_1	1.0	Dipeptides	True	2.649e+07	3.389	3.389	0.3678
281	Val-Gly	Amino acid	1	Ctrl_Untreated_1_9	Ctrl_Untreated_1	1.0	Dipeptides	True	5.201e+07	4.362	4.362	-0.157
270	Pro-Gly	Amino acid	1	Ctrl_Untreated_1_9	Ctrl_Untreated_1	1.0	Dipeptides	True	2.934e+06	0.2142	0.2142	-0.5326
114	Imidazole Propionate	Amino acid	1	Ctrl_Untreated_1_9	Ctrl_Untreated_1	1.0	Amino acids degradation intermediates	True	8.492e+05	-1.574	-1.574	0.7363
267	Phe-Gly	Amino acid	1	Ctrl_Untreated_1_9	Ctrl_Untreated_1	1.0	Dipeptides	True	2.298e+07	3.184	3.184	-0.9102
266	Phe-Ala	Amino acid	1	Ctrl_Untreated_1_9	Ctrl_Untreated_1	1.0	Dipeptides	True	3.836e+06	0.601	0.601	-0.4757
278	Tyr-Gly	Amino acid	1	Ctrl_Untreated_1_9	Ctrl_Untreated_1	1.0	Dipeptides	True	7.547e+06	1.577	1.577	-0.6048
255	His-Ala	Amino acid	1	Ctrl_Untreated_1_9	Ctrl_Untreated_1	1.0	Dipeptides	True	3.747e+05	-2.755	-2.755	-1.418
280	Val-Gln	Amino acid	1	Ctrl_Untreated_1_9	Ctrl_Untreated_1	1.0	Dipeptides	True	7.121e+06	1.494	1.494	-0.1246
143	S-1-Pyrroline-5-Carboxylate	Amino acid	1	Ctrl_Untreated_1_9	Ctrl_Untreated_1	0.857142857142857	Amino acid derivatives	True	1.77e+06	-0.5146	-0.5146	1.554
232	SAH	Amino acid	1	Ctrl_Untreated_1_9	Ctrl_Untreated_1	1.0	SAM metabolism	True	1.637e+06	-0.6273	-0.6273	-0.1419
20	Erythronate	Carbon	1	Ctrl_Untreated_1_9	Ctrl_Untreated_1	1.0	Aminosugar derivatives	True	5.247e+07	4.375	4.375	0.6336
248	Gln-Leu	Amino acid	1	Ctrl_Untreated_1_9	Ctrl_Untreated_1	1.0	Dipeptides	True	7.597e+05	-1.735	-1.735	-0.8298
276	Trp-Gly	Amino acid	1	Ctrl_Untreated_1_9	Ctrl_Untreated_1	1.0	Dipeptides	True	7.13e+05	-1.827	-1.827	-0.7484
205	N-delta-Ac-Ornithine	Amino acid	1	Ctrl_Untreated_1_9	Ctrl_Untreated_1	1.0	Amino acid derivatives	True	2.24e+06	-0.1752	-0.1752	0.9459
163	Formimino-Glu	Amino acid	1	Ctrl_Untreated_1_9	Ctrl_Untreated_1	1.0	Amino acid derivatives	True	1.653e+06	-0.6135	-0.6135	0.6263
204	N-Me-Arg	Amino acid	1	Ctrl_Untreated_1_9	Ctrl_Untreated_1	1.0	Amino acid derivatives	True	9.367e+06	1.889	1.889	-0.698
242	Guanidino-Ac	Amino acid	1	Ctrl_Untreated_1_9	Ctrl_Untreated_1	0.857142857142857	Creatine biosynthesis	True	3.177e+05	-2.993	-2.993	0.8877

Metabolite ID	Name	Super Pathway	Dataset	Sample ID	Group ID	Detection Fraction	Pathway	Detected	Raw Intensity	Log2 Norm Intensity	Norm Imputed	Log2 Ctrl Norm
300	gamma-Glu-Val	Amino acid	1	Ctrl_Untreated_1_9	Ctrl_Untreated_1	0.857142857142857	Gamma-glutamyl dipeptides	True	2.351e+06	-0.1053	-0.1053	-0.726
53	Ac-CoA	Carbon	1	Ctrl_Untreated_1_9	Ctrl_Untreated_1	0.285714285714286	Acetyl-CoA	True	4.922e+04	-5.683	-5.683	1.079
18	Maltotriose	Carbon	1	Ctrl_Untreated_1_9	Ctrl_Untreated_1	1.0	Glycogen degradation	True	8.745e+06	1.79	1.79	1.948
294	gamma-Glu-Met	Amino acid	1	Ctrl_Untreated_1_9	Ctrl_Untreated_1	0.714285714285714	Gamma-glutamyl dipeptides	True	1.115e+05	-4.503	-4.503	-1.021
174	Met Sulfone	Amino acid	1	Ctrl_Untreated_1_9	Ctrl_Untreated_1	1.0	Amino acid derivatives	True	4.226e+05	-2.581	-2.581	0.4977
175	N-Ac-Met Sulfoxide	Amino acid	1	Ctrl_Untreated_1_9	Ctrl_Untreated_1	1.0	Amino acid derivatives	True	1.656e+06	-0.6108	-0.6108	-0.5871
25	Mannitol/Sorbitol	Carbon	1	Ctrl_Untreated_1_9	Ctrl_Untreated_1	1.0	Sugars and sugar alcohols	True	2.078e+07	3.039	3.039	0.814
6	UDP-GlcNAc	Carbon	1	Ctrl_Untreated_1_9	Ctrl_Untreated_1	0.428571428571429	Aminosugar biosynthesis	True	4.338e+05	-2.543	-2.543	0.5644
145	Pyro-Gln	Amino acid	1	Ctrl_Untreated_1_9	Ctrl_Untreated_1	1.0	Amino acid derivatives	True	5.601e+06	1.147	1.147	0.3073
705	Coenzyme A	Cofactor	1	Ctrl_Untreated_1_9	Ctrl_Untreated_1	0.714285714285714	Cofactors	True	2.652e+05	-3.253	-3.253	0.7057
319	2'-dAMP	Nucleotide	1	Ctrl_Untreated_1_9	Ctrl_Untreated_1	0.571428571428571	Deoxy-nucleotides	True	3.672e+05	-2.784	-2.784	0.8963
119	alpha-OH-Isovalerate	Amino acid	1	Ctrl_Untreated_1_9	Ctrl_Untreated_1	0.714285714285714	Amino acids degradation intermediates	True	8.498e+05	-1.573	-1.573	1.725
46	Fructose 1,6-PP / Glucose 1,6-PP / Inositol-1,4-PP / Inositol-1,3-PP	Carbon	1	Ctrl_Untreated_1_9	Ctrl_Untreated_1	1.0	Glycolysis, GNG	True	1.573e+08	5.959	5.959	1.128
137	1-Me-Guanidine	Amino acid	1	Ctrl_Untreated_1_9	Ctrl_Untreated_1	0.857142857142857	Amino acids degradation intermediates	True	1.297e+05	-4.285	-4.285	0.8673
23	N-GlcNAc-Asn	Carbon	1	Ctrl_Untreated_1_9	Ctrl_Untreated_1	1.0	Aminosugar derivatives	True	3.787e+06	0.5826	0.5826	-0.1303
262	Leu-Gln	Amino acid	1	Ctrl_Untreated_1_9	Ctrl_Untreated_1	1.0	Dipeptides	True	4.678e+06	0.8873	0.8873	-0.409
24	Fructose	Carbon	1	Ctrl_Untreated_1_9	Ctrl_Untreated_1	1.0	Sugars and sugar alcohols	True	1.697e+07	2.746	2.746	0.7975
197	C-Glycosyl-Trp	Amino acid	1	Ctrl_Untreated_1_9	Ctrl_Untreated_1	1.0	Amino acid derivatives	True	1.867e+06	-0.4375	-0.4375	-0.1384
33	Arabitol/Xylitol	Carbon	1	Ctrl_Untreated_1_9	Ctrl_Untreated_1	1.0	Sugars and sugar alcohols	True	9.723e+05	-1.379	-1.379	0.4808

Metabolite ID	Name	Super Pathway	Dataset	Sample ID	Group ID	Detection Fraction	Pathway	Detected	Raw Intensity	Log2 Norm Intensity	Norm Imputed	Log2 Ctrl Norm
128	N2-Ac-Lys/N6-Ac-Lys	Amino acid	1	Ctrl_Untreated_1_9	Ctrl_Untreated_1	1.0	Amino acids degradation intermediates	True	2.449e+06	-0.04643	-0.04643	-1.257
42	2-Me-Citrate	Carbon	1	Ctrl_Untreated_1_9	Ctrl_Untreated_1	0.857142857142857	Propionate metabolism	True	2.346e+05	-3.43	-3.43	0.9526
12	Glucuronate 1-P	Carbon	1	Ctrl_Untreated_1_9	Ctrl_Untreated_1	1.0	Polysaccharide biosynthesis	True	1.062e+06	-1.252	-1.252	-9.30e-03
76	Gln	Amino acid	1	G6PDD_Untreated_1_1	G6PDD_Untreated_1	1.0	Proteinogenic amino acids	True	1.609e+09	9.046	9.046	0.3658
89	Trp	Amino acid	1	G6PDD_Untreated_1_1	G6PDD_Untreated_1	1.0	Proteinogenic amino acids	True	1.273e+08	5.386	5.386	-0.2894
723	beta-Ala	Cofactor	1	G6PDD_Untreated_1_1	G6PDD_Untreated_1	1.0	Coenzyme A biosynthesis	True	4.372e+07	3.845	3.845	0.8357
75	Glu	Amino acid	1	G6PDD_Untreated_1_1	G6PDD_Untreated_1	1.0	Proteinogenic amino acids	True	5.624e+09	10.85	10.85	0.1157
77	Gly	Amino acid	1	G6PDD_Untreated_1_1	G6PDD_Untreated_1	1.0	Proteinogenic amino acids	True	1.457e+08	5.582	5.582	-0.03372
80	His	Amino acid	1	G6PDD_Untreated_1_1	G6PDD_Untreated_1	1.0	Proteinogenic amino acids	True	2.955e+07	3.28	3.28	-0.1354
82	Leu	Amino acid	1	G6PDD_Untreated_1_1	G6PDD_Untreated_1	1.0	Proteinogenic amino acids	True	1.684e+09	9.113	9.113	-0.1084
87	Phe	Amino acid	1	G6PDD_Untreated_1_1	G6PDD_Untreated_1	1.0	Proteinogenic amino acids	True	1.138e+09	8.548	8.548	-0.2639
130	Glutarate	Amino acid	1	G6PDD_Untreated_1_1	G6PDD_Untreated_1	1.0	Amino acids degradation intermediates	True	5.579e+05	-2.447	-2.447	-0.02041
196	5-OH-Indole-Ac	Amino acid	1	G6PDD_Untreated_1_1	G6PDD_Untreated_1	0.75	Amino acid derivatives	False			-5.134	-1.122
74	Asp	Amino acid	1	G6PDD_Untreated_1_1	G6PDD_Untreated_1	1.0	Proteinogenic amino acids	True	6.702e+08	7.783	7.783	-0.1083
236	Spermidine	Amino acid	1	G6PDD_Untreated_1_1	G6PDD_Untreated_1	1.0	Polyamines	True	3.814e+07	3.648	3.648	-0.2135
73	Asn	Amino acid	1	G6PDD_Untreated_1_1	G6PDD_Untreated_1	1.0	Proteinogenic amino acids	True	2.823e+08	6.536	6.536	-0.1352
243	Creatinine	Amino acid	1	G6PDD_Untreated_1_1	G6PDD_Untreated_1	1.0	Creatine degradation	True	8.347e+07	4.778	4.778	-0.2242
376	Cytidine	Nucleotide	1	G6PDD_Untreated_1_1	G6PDD_Untreated_1	0.75	Pyrimidine nucleosides	True	2.736e+07	3.169	3.169	2.441
41	Lactate	Carbon	1	G6PDD_Untreated_1_1	G6PDD_Untreated_1	1.0	Respiratory carbon sources	True	2.558e+08	6.393	6.393	-0.08837
58	alpha-Ketoglutarate	Carbon	1	G6PDD_Untreated_1_1	G6PDD_Untreated_1	1.0	TCA cycle	True	5.13e+06	0.7538	0.7538	0.7371

Metabolite ID	Name	Super Pathway	Dataset	Sample ID	Group ID	Detection Fraction	Pathway	Detected	Raw Intensity	Log2 Norm Intensity	Norm Imputed	Log2 Ctrl Norm
69	3-OH-Butyrate	Carbon	1	G6PDD_Untreated_1_1	G6PDD_Untreated_1	0.75	Ketone bodies	True	2.782e+05	-3.451	-3.451	-0.4666
343	Adenine	Nucleotide	1	G6PDD_Untreated_1_1	G6PDD_Untreated_1	1.0	Purine bases	True	5.386e+06	0.8241	0.8241	-0.6328
336	Adenosine	Nucleotide	1	G6PDD_Untreated_1_1	G6PDD_Untreated_1	1.0	Purine nucleosides	True	1.021e+08	5.069	5.069	1.646
722	ADP-Ribose	Cofactor	1	G6PDD_Untreated_1_1	G6PDD_Untreated_1	1.0	Derivatives of NA, nicotinamide and NAD	True	2.594e+05	-3.552	-3.552	-0.55
383	Cytosine	Nucleotide	1	G6PDD_Untreated_1_1	G6PDD_Untreated_1	0.75	Pyrimidine bases	False			-5.32	-0.4652
3	Glucosamine 6-P	Carbon	1	G6PDD_Untreated_1_1	G6PDD_Untreated_1	1.0	Aminosugar biosynthesis	True	8.034e+05	-1.921	-1.921	-0.2537
717	Nicotinamide	Cofactor	1	G6PDD_Untreated_1_1	G6PDD_Untreated_1	1.0	NAD biosynthesis	True	3.664e+07	3.59	3.59	-0.9248
51	PEP	Carbon	1	G6PDD_Untreated_1_1	G6PDD_Untreated_1	1.0	Glycolysis, GNG	True	7.53e+06	1.307	1.307	0.2563
237	Spermine	Amino acid	1	G6PDD_Untreated_1_1	G6PDD_Untreated_1	1.0	Polyamines	True	1.337e+06	-1.186	-1.186	0.305
385	Uracil	Nucleotide	1	G6PDD_Untreated_1_1	G6PDD_Untreated_1	1.0	Pyrimidine bases	True	1.606e+07	2.401	2.401	2.136
377	Uridine	Nucleotide	1	G6PDD_Untreated_1_1	G6PDD_Untreated_1	1.0	Pyrimidine nucleosides	True	6.636e+07	4.447	4.447	0.3981
348	Allantoin	Nucleotide	1	G6PDD_Untreated_1_1	G6PDD_Untreated_1	1.0	Purine degradation	True	2.564e+06	-0.2466	-0.2466	-0.07097
335	Inosine	Nucleotide	1	G6PDD_Untreated_1_1	G6PDD_Untreated_1	1.0	Purine nucleosides	True	1.581e+08	5.699	5.699	0.2001
81	Ile	Amino acid	1	G6PDD_Untreated_1_1	G6PDD_Untreated_1	1.0	Proteinogenic amino acids	True	1.078e+09	8.469	8.469	-0.2462
72	Ala	Amino acid	1	G6PDD_Untreated_1_1	G6PDD_Untreated_1	1.0	Proteinogenic amino acids	True	1.276e+09	8.712	8.712	0.2105
79	Thr	Amino acid	1	G6PDD_Untreated_1_1	G6PDD_Untreated_1	1.0	Proteinogenic amino acids	True	8.438e+08	8.116	8.116	0.1061
88	Tyr	Amino acid	1	G6PDD_Untreated_1_1	G6PDD_Untreated_1	1.0	Proteinogenic amino acids	True	5.476e+08	7.492	7.492	-0.03179
84	Lys	Amino acid	1	G6PDD_Untreated_1_1	G6PDD_Untreated_1	1.0	Proteinogenic amino acids	True	4.912e+08	7.335	7.335	-0.6551
86	Met	Amino acid	1	G6PDD_Untreated_1_1	G6PDD_Untreated_1	1.0	Proteinogenic amino acids	True	8.146e+08	8.065	8.065	-0.1629
61	Malate	Carbon	1	G6PDD_Untreated_1_1	G6PDD_Untreated_1	1.0	TCA cycle	True	5.423e+08	7.478	7.478	0.3061

Metabolite ID	Name	Super Pathway	Dataset	Sample ID	Group ID	Detection Fraction	Pathway	Detected	Raw Intensity	Log2 Norm Intensity	Norm Imputed	Log2 Ctrl Norm
235	Putrescine	Amino acid	1	G6PDD_Untreated_1_1	G6PDD_Untreated_1	1.0	Polyamines	True	2.051e+06	-0.5684	-0.5684	-0.07724
324	2'-dU	Nucleotide	1	G6PDD_Untreated_1_1	G6PDD_Untreated_1	0.5	Deoxy-nucleosides	True	1.518e+05	-4.325	-4.325	0.05541
49	3-P-Glycerate	Carbon	1	G6PDD_Untreated_1_1	G6PDD_Untreated_1	1.0	Glycolysis, GNG	True	2.546e+07	3.065	3.065	-0.2001
189	Kynurenate	Amino acid	1	G6PDD_Untreated_1_1	G6PDD_Untreated_1	0.75	Amino acid derivatives	False			-6.319	-0.5206
234	5-Me-Thioadenosine	Amino acid	1	G6PDD_Untreated_1_1	G6PDD_Untreated_1	1.0	SAM metabolism	True	1.283e+07	2.077	2.077	0.1913
59	Succinate	Carbon	1	G6PDD_Untreated_1_1	G6PDD_Untreated_1	1.0	TCA cycle	True	3.76e+06	0.3055	0.3055	4.48e-03
36	Ribose	Carbon	1	G6PDD_Untreated_1_1	G6PDD_Untreated_1	1.0	Sugars and sugar alcohols	True	7.556e+05	-2.009	-2.009	-0.776
133	Ornithine	Amino acid	1	G6PDD_Untreated_1_1	G6PDD_Untreated_1	1.0	Amino acids degradation intermediates	True	7.781e+07	4.677	4.677	0.1972
313	5-Oxoproline	Amino acid	1	G6PDD_Untreated_1_1	G6PDD_Untreated_1	1.0	Glutathione derivatives	True	1.425e+07	2.228	2.228	0.1404
165	N-6-Tri-Me-Lys	Amino acid	1	G6PDD_Untreated_1_1	G6PDD_Untreated_1	1.0	Amino acid derivatives	True	3.494e+07	3.522	3.522	-0.2422
380	Orotate	Nucleotide	1	G6PDD_Untreated_1_1	G6PDD_Untreated_1	1.0	Pyrimidine (UMP) biosynthesis	True	2.285e+05	-3.735	-3.735	-0.105
724	Pantothenate	Cofactor	1	G6PDD_Untreated_1_1	G6PDD_Untreated_1	1.0	Coenzyme A biosynthesis	True	3.371e+08	6.792	6.792	0.4818
150	N-Me-Gly	Amino acid	1	G6PDD_Untreated_1_1	G6PDD_Untreated_1	1.0	Amino acid derivatives	True	2.398e+06	-0.3436	-0.3436	-0.04313
122	3-OH-Isobutyrate	Amino acid	1	G6PDD_Untreated_1_1	G6PDD_Untreated_1	0.5	Amino acids degradation intermediates	False			-3.901	-0.7452
241	4-Acetamidobutanoate	Amino acid	1	G6PDD_Untreated_1_1	G6PDD_Untreated_1	1.0	Polyamine derivatives	True	3.835e+06	0.334	0.334	-0.107
711	alpha-Tocopherol	Cofactor	1	G6PDD_Untreated_1_1	G6PDD_Untreated_1	1.0	Cofactors	True	8.384e+06	1.462	1.462	0.6821
55	Citrate	Carbon	1	G6PDD_Untreated_1_1	G6PDD_Untreated_1	1.0	TCA cycle	True	1.646e+07	2.435	2.435	0.4864
387	3-Aminoisobutyrate	Nucleotide	1	G6PDD_Untreated_1_1	G6PDD_Untreated_1	1.0	Pyrimidine degradation	True	4.928e+05	-2.626	-2.626	-0.6123
338	Guanosine	Nucleotide	1	G6PDD_Untreated_1_1	G6PDD_Untreated_1	1.0	Purine nucleosides	True	1.051e+08	5.11	5.11	4.07e-03
209	N-Ac-Ala	Amino acid	1	G6PDD_Untreated_1_1	G6PDD_Untreated_1	1.0	N-acetylated amino acids	True	1.223e+06	-1.315	-1.315	-0.1138

Metabolite ID	Name	Super Pathway	Dataset	Sample ID	Group ID	Detection Fraction	Pathway	Detected	Raw Intensity	Log2 Norm Intensity	Norm Imputed	Log2 Ctrl Norm
221	N-Ac-Met	Amino acid	1	G6PDD_Untreated_1_1	G6PDD_Untreated_1	1.0	N-acetylated amino acids	True	7.136e+06	1.23	1.23	-0.5143
228	N-Ac-Val	Amino acid	1	G6PDD_Untreated_1_1	G6PDD_Untreated_1	1.0	N-acetylated amino acids	True	1.004e+05	-4.921	-4.921	0.4493
346	Urate	Nucleotide	1	G6PDD_Untreated_1_1	G6PDD_Untreated_1	1.0	Purine degradation	True	8.283e+05	-1.877	-1.877	-0.8141
90	Arg	Amino acid	1	G6PDD_Untreated_1_1	G6PDD_Untreated_1	1.0	Proteinogenic amino acids	True	2.255e+09	9.534	9.534	-0.172
60	Fumarate	Carbon	1	G6PDD_Untreated_1_1	G6PDD_Untreated_1	1.0	TCA cycle	True	6.234e+06	1.035	1.035	0.01987
78	Ser	Amino acid	1	G6PDD_Untreated_1_1	G6PDD_Untreated_1	1.0	Proteinogenic amino acids	True	4.789e+08	7.298	7.298	0.04111
83	Val	Amino acid	1	G6PDD_Untreated_1_1	G6PDD_Untreated_1	1.0	Proteinogenic amino acids	True	6.215e+08	7.674	7.674	-0.1978
734	Pyridoxal	Cofactor	1	G6PDD_Untreated_1_1	G6PDD_Untreated_1	1.0	PLP biosynthesis and salvage	True	3.971e+06	0.3842	0.3842	-1.399
136	Urea	Amino acid	1	G6PDD_Untreated_1_1	G6PDD_Untreated_1	1.0	Amino acids degradation intermediates	True	1.62e+06	-0.9089	-0.9089	-1.06
67	Ribose 1-P	Carbon	1	G6PDD_Untreated_1_1	G6PDD_Untreated_1	1.0	Pentose phosphate pathway (PPP)	True	4.986e+06	0.7129	0.7129	0.0917
284	Carnosine	Amino acid	1	G6PDD_Untreated_1_1	G6PDD_Untreated_1	1.0	Dipeptides	True	1.139e+06	-1.417	-1.417	0.6913
306	gamma-Glu-Cys	Amino acid	1	G6PDD_Untreated_1_1	G6PDD_Untreated_1	1.0	Glutathione biosynthesis	True	3.749e+06	0.3015	0.3015	0.6383
712	Retinol (Vit A)	Cofactor	1	G6PDD_Untreated_1_1	G6PDD_Untreated_1	1.0	Cofactors	True	6.573e+05	-2.211	-2.211	0.2227
85	Cys	Amino acid	1	G6PDD_Untreated_1_1	G6PDD_Untreated_1	1.0	Proteinogenic amino acids	True	3.073e+07	3.337	3.337	-1.177
91	Pro	Amino acid	1	G6PDD_Untreated_1_1	G6PDD_Untreated_1	1.0	Proteinogenic amino acids	True	2.147e+09	9.463	9.463	0.055
308	Glutathione, Reduced	Amino acid	1	G6PDD_Untreated_1_1	G6PDD_Untreated_1	1.0	Glutathione	True	8.943e+08	8.2	8.2	1.243
107	Citrulline	Amino acid	1	G6PDD_Untreated_1_1	G6PDD_Untreated_1	1.0	Amino acids biosynthesis intermediates	True	2.052e+07	2.754	2.754	-0.0705
328	IMP	Nucleotide	1	G6PDD_Untreated_1_1	G6PDD_Untreated_1	0.5	Purine nucleotides	False			-4.53	-0.2088
706	FAD	Cofactor	1	G6PDD_Untreated_1_1	G6PDD_Untreated_1	1.0	Cofactors	True	8.709e+05	-1.805	-1.805	0.3849
735	Pyridoxamine	Cofactor	1	G6PDD_Untreated_1_1	G6PDD_Untreated_1	1.0	PLP biosynthesis and salvage	True	1.456e+06	-1.063	-1.063	-0.8699

Metabolite ID	Name	Super Pathway	Dataset	Sample ID	Group ID	Detection Fraction	Pathway	Detected	Raw Intensity	Log2 Norm Intensity	Norm Imputed	Log2 Ctrl Norm
199	Serotonin	Amino acid	1	G6PDD_Untreated_1_1	G6PDD_Untreated_1	1.0	Amino acid derivatives	True	7.382e+06	1.279	1.279	6.24e-03
370	CMP	Nucleotide	1	G6PDD_Untreated_1_1	G6PDD_Untreated_1	1.0	Pyrimidine nucleotides	True	7.657e+06	1.332	1.332	-0.5073
287	gamma-Glu-Gln	Amino acid	1	G6PDD_Untreated_1_1	G6PDD_Untreated_1	1.0	Gamma-glutamyl dipeptides	True	4.23e+06	0.4756	0.4756	-0.3653
14	UDP-Glucuronate	Carbon	1	G6PDD_Untreated_1_1	G6PDD_Untreated_1	1.0	Polysaccharide biosynthesis	True	9.724e+05	-1.646	-1.646	0.3037
229	N-Formyl-Met	Amino acid	1	G6PDD_Untreated_1_1	G6PDD_Untreated_1	1.0	N-formylated amino acids	True	3.22e+05	-3.24	-3.24	0.5823
350	3',5'-cAMP	Nucleotide	1	G6PDD_Untreated_1_1	G6PDD_Untreated_1	1.0	Purine derivatives in signaling	True	2.49e+05	-3.611	-3.611	8.09e-03
371	CDP	Nucleotide	1	G6PDD_Untreated_1_1	G6PDD_Untreated_1	1.0	Pyrimidine nucleotides	True	2.513e+05	-3.598	-3.598	-0.2778
372	CTP	Nucleotide	1	G6PDD_Untreated_1_1	G6PDD_Untreated_1	0.75	Pyrimidine nucleotides	True	1.265e+05	-4.587	-4.587	-0.9337
333	GDP	Nucleotide	1	G6PDD_Untreated_1_1	G6PDD_Untreated_1	0.5	Purine nucleotides	False			-4.484	-1.983
332	GMP	Nucleotide	1	G6PDD_Untreated_1_1	G6PDD_Untreated_1	1.0	Purine nucleotides	True	1.008e+06	-1.593	-1.593	-2.292
373	UMP	Nucleotide	1	G6PDD_Untreated_1_1	G6PDD_Untreated_1	1.0	Pyrimidine nucleotides	True	5.794e+05	-2.392	-2.392	-1.337
389	3'-CMP	Nucleotide	1	G6PDD_Untreated_1_1	G6PDD_Untreated_1	1.0	Pyrimidine derivatives in signaling	True	1.931e+06	-0.6555	-0.6555	0.3958
330	ADP	Nucleotide	1	G6PDD_Untreated_1_1	G6PDD_Untreated_1	1.0	Purine nucleotides	True	1.975e+06	-0.623	-0.623	-0.3266
342	Hypoxanthine	Nucleotide	1	G6PDD_Untreated_1_1	G6PDD_Untreated_1	1.0	Purine bases	True	5.809e+07	4.255	4.255	0.3159
736	Pyridoxamine-P	Cofactor	1	G6PDD_Untreated_1_1	G6PDD_Untreated_1	1.0	PLP biosynthesis and salvage	True	3.162e+05	-3.266	-3.266	-0.2881
148	Betaine	Amino acid	1	G6PDD_Untreated_1_1	G6PDD_Untreated_1	1.0	Amino acid derivatives	True	9.552e+07	4.973	4.973	0.1668
344	Xanthine	Nucleotide	1	G6PDD_Untreated_1_1	G6PDD_Untreated_1	1.0	Purine bases	True	3.486e+06	0.1964	0.1964	-0.7111
386	3-Ureidopropionate	Nucleotide	1	G6PDD_Untreated_1_1	G6PDD_Untreated_1	1.0	Pyrimidine degradation	True	1.334e+06	-1.19	-1.19	-0.2705
149	DiMe-Gly	Amino acid	1	G6PDD_Untreated_1_1	G6PDD_Untreated_1	1.0	Amino acid derivatives	True	1.762e+06	-0.7881	-0.7881	-0.2678
703	NAD+	Cofactor	1	G6PDD_Untreated_1_1	G6PDD_Untreated_1	1.0	Cofactors	True	2.861e+07	3.233	3.233	0.497
709	Pyridoxal-P	Cofactor	1	G6PDD_Untreated_1_1	G6PDD_Untreated_1	1.0	Cofactors	True	6.993e+05	-2.121	-2.121	0.1867

Metabolite ID	Name	Super Pathway	Dataset	Sample ID	Group ID	Detection Fraction	Pathway	Detected	Raw Intensity	Log2 Norm Intensity	Norm Imputed	Log2 Ctrl Norm
731	Thiamin (Vitamin B1)	Cofactor	1	G6PDD_Untreated_1_1	G6PDD_Untreated_1	1.0	TPP biosynthesis	True	5.871e+06	0.9485	0.9485	0.2008
374	UDP	Nucleotide	1	G6PDD_Untreated_1_1	G6PDD_Untreated_1	1.0	Pyrimidine nucleotides	True	7.438e+05	-2.032	-2.032	-0.4784
102	2-Aminoadipate	Amino acid	1	G6PDD_Untreated_1_1	G6PDD_Untreated_1	1.0	Amino acids biosynthesis intermediates	True	7.467e+05	-2.026	-2.026	0.0831
45	Fructose-6-P	Carbon	1	G6PDD_Untreated_1_1	G6PDD_Untreated_1	1.0	Glycolysis, GNG	True	2.552e+06	-0.2536	-0.2536	-0.3289
320	TMP	Nucleotide	1	G6PDD_Untreated_1_1	G6PDD_Untreated_1	0.5	Deoxy-nucleotides	False			-6.992	-2.2
341	XMP	Nucleotide	1	G6PDD_Untreated_1_1	G6PDD_Untreated_1	0.25	IMP conversion to AMP & GMP	True	1.573e+05	-4.273	-4.273	0.301
120	beta-OH-Isovalerate	Amino acid	1	G6PDD_Untreated_1_1	G6PDD_Untreated_1	1.0	Amino acids degradation intermediates	True	4.131e+05	-2.881	-2.881	0.2848
322	2'-dI	Nucleotide	1	G6PDD_Untreated_1_1	G6PDD_Untreated_1	0.5	Deoxy-nucleosides	False			-5.917	-1.791
4	GlcNAc 6-P	Carbon	1	G6PDD_Untreated_1_1	G6PDD_Untreated_1	1.0	Aminosugar biosynthesis	True	4.471e+06	0.5554	0.5554	0.2268
337	Xanthosine	Nucleotide	1	G6PDD_Untreated_1_1	G6PDD_Untreated_1	1.0	Purine nucleosides	True	1.913e+05	-3.991	-3.991	-0.9165
188	Kynurenine	Amino acid	1	G6PDD_Untreated_1_1	G6PDD_Untreated_1	1.0	Amino acid derivatives	True	4.702e+06	0.6282	0.6282	1.32
63	6-P-Gluconate	Carbon	1	G6PDD_Untreated_1_1	G6PDD_Untreated_1	1.0	Pentose phosphate pathway (PPP)	True	6.354e+06	1.063	1.063	-0.971
40	Glucuronate	Carbon	1	G6PDD_Untreated_1_1	G6PDD_Untreated_1	1.0	Sugars and sugar alcohols	True	1.074e+06	-1.502	-1.502	0.03719
108	Argininosuccinate	Amino acid	1	G6PDD_Untreated_1_1	G6PDD_Untreated_1	1.0	Amino acids biosynthesis intermediates	True	3.558e+06	0.2259	0.2259	0.4521
710	Carnitine	Cofactor	1	G6PDD_Untreated_1_1	G6PDD_Untreated_1	1.0	Cofactors	True	2.185e+08	6.166	6.166	0.1886
725	P-Pantetheine	Cofactor	1	G6PDD_Untreated_1_1	G6PDD_Untreated_1	0.75	Coenzyme A biosynthesis	True	2.779e+05	-3.453	-3.453	0.252
48	DHAP	Carbon	1	G6PDD_Untreated_1_1	G6PDD_Untreated_1	1.0	Glycolysis, GNG	True	2.609e+07	3.1	3.1	3.48e-03
17	Maltose	Carbon	1	G6PDD_Untreated_1_1	G6PDD_Untreated_1	1.0	Glycogen degradation	True	7.215e+05	-2.076	-2.076	-0.7832
359	N1-Me-Adenosine	Nucleotide	1	G6PDD_Untreated_1_1	G6PDD_Untreated_1	1.0	Purine derivatives in RNAs	True	1.644e+06	-0.8877	-0.8877	0.15
159	3-Me-His	Amino acid	1	G6PDD_Untreated_1_1	G6PDD_Untreated_1	1.0	Amino acid derivatives	True	2.581e+05	-3.559	-3.559	0.6763

Metabolite ID	Name	Super Pathway	Dataset	Sample ID	Group ID	Detection Fraction	Pathway	Detected	Raw Intensity	Log2 Norm Intensity	Norm Imputed	Log2 Ctrl Norm
155	4-Guanidinobutanoate	Amino acid	1	G6PDD_Untreated_1_1	G6PDD_Untreated_1	1.0	Amino acid derivatives	True	1.974e+06	-0.6239	-0.6239	0.8101
164	5-OH-Lys	Amino acid	1	G6PDD_Untreated_1_1	G6PDD_Untreated_1	1.0	Amino acid derivatives	True	4.849e+05	-2.649	-2.649	-0.8791
357	Adenosine-3',5'-PP	Nucleotide	1	G6PDD_Untreated_1_1	G6PDD_Untreated_1	1.0	Purine byproducts of metabolic processes	True	2e+05	-3.927	-3.927	-0.45
104	Cystathionine	Amino acid	1	G6PDD_Untreated_1_1	G6PDD_Untreated_1	1.0	Amino acids biosynthesis intermediates	True	1.596e+07	2.392	2.392	0.1821
113	Imidazole Lactate	Amino acid	1	G6PDD_Untreated_1_1	G6PDD_Untreated_1	1.0	Amino acids degradation intermediates	True	2.428e+05	-3.647	-3.647	0.0293
215	N-Ac-Glu	Amino acid	1	G6PDD_Untreated_1_1	G6PDD_Untreated_1	1.0	N-acetylated amino acids	True	6.008e+06	0.9819	0.9819	-0.7616
310	S-Lactoyl-Glutathione	Amino acid	1	G6PDD_Untreated_1_1	G6PDD_Untreated_1	1.0	Glutathione derivatives	True	1.013e+07	1.736	1.736	1.379
5	GlcNAc 1-P	Carbon	1	G6PDD_Untreated_1_1	G6PDD_Untreated_1	1.0	Aminosugar biosynthesis	True	9.355e+05	-1.701	-1.701	0.2096
34	Ribitol	Carbon	1	G6PDD_Untreated_1_1	G6PDD_Untreated_1	1.0	Sugars and sugar alcohols	True	3.066e+05	-3.311	-3.311	0.1194
10	UDP-Galactose	Carbon	1	G6PDD_Untreated_1_1	G6PDD_Untreated_1	0.75	Polysaccharide biosynthesis	True	3.784e+06	0.3146	0.3146	0.9411
13	Guanosine 5'-PP-Fucose	Carbon	1	G6PDD_Untreated_1_1	G6PDD_Untreated_1	1.0	Polysaccharide biosynthesis	True	5.192e+05	-2.551	-2.551	-0.2515
19	Maltotetraose	Carbon	1	G6PDD_Untreated_1_1	G6PDD_Untreated_1	1.0	Glycogen degradation	True	3.333e+06	0.1318	0.1318	0.1755
233	SAM	Amino acid	1	G6PDD_Untreated_1_1	G6PDD_Untreated_1	1.0	SAM metabolism	True	3.097e+06	0.02572	0.02572	0.182
129	5-Aminovalerate	Amino acid	1	G6PDD_Untreated_1_1	G6PDD_Untreated_1	1.0	Amino acids degradation intermediates	True	7.396e+06	1.282	1.282	0.2799
741	5-Me-THF	Cofactor	1	G6PDD_Untreated_1_1	G6PDD_Untreated_1	1.0	Folate metabolism	True	7.375e+04	-5.366	-5.366	-0.6085
198	Indolelactate	Amino acid	1	G6PDD_Untreated_1_1	G6PDD_Untreated_1	1.0	Amino acid derivatives	True	1.155e+05	-4.719	-4.719	-0.9322
254	Gly-Val	Amino acid	1	G6PDD_Untreated_1_1	G6PDD_Untreated_1	1.0	Dipeptides	True	8.035e+06	1.401	1.401	0.2898
291	gamma-Glu-Leu	Amino acid	1	G6PDD_Untreated_1_1	G6PDD_Untreated_1	1.0	Gamma-glutamyl dipeptides	True	1.592e+06	-0.934	-0.934	-0.4453
173	Met Sulfoxide	Amino acid	1	G6PDD_Untreated_1_1	G6PDD_Untreated_1	1.0	Amino acid derivatives	True	1.075e+07	1.822	1.822	-0.7456

Metabolite ID	Name	Super Pathway	Dataset	Sample ID	Group ID	Detection Fraction	Pathway	Detected	Raw Intensity	Log2 Norm Intensity	Norm Imputed	Log2 Ctrl Norm
43	Glucose	Carbon	1	G6PDD_Untreated_1_1	G6PDD_Untreated_1	1.0	Glycolysis, GNG	True	4.581e+07	3.912	3.912	-1.139
185	Phenyllactate	Amino acid	1	G6PDD_Untreated_1_1	G6PDD_Untreated_1	0.5	Amino acid derivatives	True	5.035e+04	-5.917	-5.917	-0.1051
156	Homo-Arg	Amino acid	1	G6PDD_Untreated_1_1	G6PDD_Untreated_1	1.0	Amino acid derivatives	True	4.49e+06	0.5617	0.5617	-0.6188
135	Homocitrulline	Amino acid	1	G6PDD_Untreated_1_1	G6PDD_Untreated_1	1.0	Amino acids degradation intermediates	True	1.073e+06	-1.504	-1.504	0.199
719	Nicotinamide MN	Cofactor	1	G6PDD_Untreated_1_1	G6PDD_Untreated_1	1.0	NAD biosynthesis	True	5.183e+06	0.7687	0.7687	0.5831
212	N-Ac-Asp	Amino acid	1	G6PDD_Untreated_1_1	G6PDD_Untreated_1	1.0	N-acetylated amino acids	True	2.282e+07	2.907	2.907	2.21
720	1-Me-Nicotinamide	Cofactor	1	G6PDD_Untreated_1_1	G6PDD_Untreated_1	1.0	Derivatives of NA, nicotinamide and NAD	True	5.048e+08	7.374	7.374	-0.5162
216	N-Ac-Gly	Amino acid	1	G6PDD_Untreated_1_1	G6PDD_Untreated_1	1.0	N-acetylated amino acids	True	3.082e+05	-3.303	-3.303	0.2367
70	Creatine	Carbon	1	G6PDD_Untreated_1_1	G6PDD_Untreated_1	1.0	Creatine energy storage	True	1.586e+09	9.026	9.026	-0.3909
26	Galactonate	Carbon	1	G6PDD_Untreated_1_1	G6PDD_Untreated_1	1.0	Sugars and sugar alcohols	True	2.678e+06	-0.184	-0.184	0.6206
309	Glutathione, Oxidized	Amino acid	1	G6PDD_Untreated_1_1	G6PDD_Untreated_1	1.0	Glutathione	True	9.542e+06	1.649	1.649	0.4565
35	Ribonate	Carbon	1	G6PDD_Untreated_1_1	G6PDD_Untreated_1	1.0	Sugars and sugar alcohols	True	1.937e+06	-0.6511	-0.6511	-0.1238
160	1-Me-His	Amino acid	1	G6PDD_Untreated_1_1	G6PDD_Untreated_1	1.0	Amino acid derivatives	True	3.951e+07	3.699	3.699	0.258
44	Glucose 6-P	Carbon	1	G6PDD_Untreated_1_1	G6PDD_Untreated_1	1.0	Glycolysis, GNG	True	1.123e+06	-1.438	-1.438	0.03836
704	NADH	Cofactor	1	G6PDD_Untreated_1_1	G6PDD_Untreated_1	1.0	Cofactors	True	4.108e+06	0.4332	0.4332	1.079
275	Thr-Phe	Amino acid	1	G6PDD_Untreated_1_1	G6PDD_Untreated_1	1.0	Dipeptides	True	6.277e+05	-2.277	-2.277	-0.6239
738	Pyridoxate	Cofactor	1	G6PDD_Untreated_1_1	G6PDD_Untreated_1	1.0	PLP biosynthesis and salvage	True	1.465e+05	-4.376	-4.376	-1.011
177	3-(4-OH-Phenyl)Lactate	Amino acid	1	G6PDD_Untreated_1_1	G6PDD_Untreated_1	1.0	Amino acid derivatives	True	4.855e+05	-2.648	-2.648	-0.1822
206	Trans-4-OH-Pro	Amino acid	1	G6PDD_Untreated_1_1	G6PDD_Untreated_1	1.0	Amino acid derivatives	True	1.106e+08	5.184	5.184	0.0285
329	AMP	Nucleotide	1	G6PDD_Untreated_1_1	G6PDD_Untreated_1	1.0	Purine nucleotides	True	1.283e+07	2.076	2.076	-1.559

Metabolite ID	Name	Super Pathway	Dataset	Sample ID	Group ID	Detection Fraction	Pathway	Detected	Raw Intensity	Log2 Norm Intensity	Norm Imputed	Log2 Ctrl Norm
11	UDP-Glucose	Carbon	1	G6PDD_Untreated_1_1	G6PDD_Untreated_1	1.0	Polysaccharide biosynthesis	True	2.988e+06	-0.02572	-0.02572	0.07948
158	4-Imidazole-Ac	Amino acid	1	G6PDD_Untreated_1_1	G6PDD_Untreated_1	1.0	Amino acid derivatives	True	5.818e+05	-2.386	-2.386	-0.2398
111	1-Me-Imidazole-Ac	Amino acid	1	G6PDD_Untreated_1_1	G6PDD_Untreated_1	1.0	Amino acids degradation intermediates	True	6.87e+05	-2.147	-2.147	-0.213
345	Guanine	Nucleotide	1	G6PDD_Untreated_1_1	G6PDD_Untreated_1	1.0	Purine bases	True	1.019e+08	5.066	5.066	0.07982
22	N-Ac-Neuraminate	Carbon	1	G6PDD_Untreated_1_1	G6PDD_Untreated_1	1.0	Aminosugar derivatives	True	3.228e+06	0.08559	0.08559	0.221
721	N'-Methylnicotinate	Cofactor	1	G6PDD_Untreated_1_1	G6PDD_Untreated_1	1.0	Derivatives of NA, nicotinamide and NAD	True	1.172e+06	-1.376	-1.376	-0.3688
183	Phenol Sulfate	Amino acid	1	G6PDD_Untreated_1_1	G6PDD_Untreated_1	1.0	Amino acid derivatives	True	5.923e+04	-5.683	-5.683	-1.042
718	Nicotinamide Riboside	Cofactor	1	G6PDD_Untreated_1_1	G6PDD_Untreated_1	1.0	NAD biosynthesis	True	6.471e+06	1.089	1.089	-0.5013
297	gamma-Glu-Thr	Amino acid	1	G6PDD_Untreated_1_1	G6PDD_Untreated_1	1.0	Gamma-glutamyl dipeptides	True	4.633e+06	0.6068	0.6068	-0.03338
295	gamma-Glu-Phe	Amino acid	1	G6PDD_Untreated_1_1	G6PDD_Untreated_1	0.75	Gamma-glutamyl dipeptides	True	6.595e+04	-5.528	-5.528	0.2573
347	Allantoic Acid	Nucleotide	1	G6PDD_Untreated_1_1	G6PDD_Untreated_1	0.5	Purine degradation	False			-6.474	-0.8345
399	Pseudouridine	Nucleotide	1	G6PDD_Untreated_1_1	G6PDD_Untreated_1	1.0	Pyrimidine derivatives in RNAs	True	4.201e+05	-2.856	-2.856	-0.8262
375	UTP	Nucleotide	1	G6PDD_Untreated_1_1	G6PDD_Untreated_1	1.0	Pyrimidine nucleotides	True	3.808e+05	-2.998	-2.998	-1.022
144	Glu, gamma-Me Ester	Amino acid	1	G6PDD_Untreated_1_1	G6PDD_Untreated_1	1.0	Amino acid derivatives	True	4.441e+06	0.5458	0.5458	-0.03198
292	gamma-Glu-epsilon-Lysine	Amino acid	1	G6PDD_Untreated_1_1	G6PDD_Untreated_1	1.0	Gamma-glutamyl dipeptides	True	1.219e+06	-1.319	-1.319	-1.128
225	N-Ac-Thr	Amino acid	1	G6PDD_Untreated_1_1	G6PDD_Untreated_1	1.0	N-acetylated amino acids	True	1.065e+06	-1.514	-1.514	-0.01103
211	N-Ac-Asn	Amino acid	1	G6PDD_Untreated_1_1	G6PDD_Untreated_1	1.0	N-acetylated amino acids	True	4.304e+05	-2.821	-2.821	-0.1361
151	Phenylacetylglycine	Amino acid	1	G6PDD_Untreated_1_1	G6PDD_Untreated_1	1.0	Amino acid derivatives	True	9.569e+05	-1.669	-1.669	-0.8817
217	N-Ac-His	Amino acid	1	G6PDD_Untreated_1_1	G6PDD_Untreated_1	1.0	N-acetylated amino acids	True	2.312e+05	-3.718	-3.718	-0.2333
288	gamma-Glu-Gly	Amino acid	1	G6PDD_Untreated_1_1	G6PDD_Untreated_1	0.75	Gamma-glutamyl dipeptides	True	1.43e+06	-1.089	-1.089	0.7353

Metabolite ID	Name	Super Pathway	Dataset	Sample ID	Group ID	Detection Fraction	Pathway	Detected	Raw Intensity	Log2 Norm Intensity	Norm Imputed	Log2 Ctrl Norm
222	N-Ac-Phe	Amino acid	1	G6PDD_Untreated_1_1	G6PDD_Untreated_1	1.0	N-acetylated amino acids	True	2.93e+04	-6.698	-6.698	-0.4255
71	Creatine-P	Carbon	1	G6PDD_Untreated_1_1	G6PDD_Untreated_1	1.0	Creatine energy storage	True	3.996e+05	-2.929	-2.929	1.327
210	N-Ac-Arg	Amino acid	1	G6PDD_Untreated_1_1	G6PDD_Untreated_1	0.75	N-acetylated amino acids	True	6.188e+05	-2.298	-2.298	0.378
218	N-Ac-Ile	Amino acid	1	G6PDD_Untreated_1_1	G6PDD_Untreated_1	0.5	N-acetylated amino acids	False			-6.52	-0.5541
251	Gly-Leu	Amino acid	1	G6PDD_Untreated_1_1	G6PDD_Untreated_1	1.0	Dipeptides	True	6.775e+06	1.155	1.155	-0.4836
290	gamma-Glu-Ile	Amino acid	1	G6PDD_Untreated_1_1	G6PDD_Untreated_1	1.0	Gamma-glutamyl dipeptides	True	1.176e+06	-1.371	-1.371	-0.03247
316	Ophthalmate	Amino acid	1	G6PDD_Untreated_1_1	G6PDD_Untreated_1	1.0	Oxidative stress markers	True	9.169e+06	1.592	1.592	-0.7509
125	Isovaleryl-Gly	Amino acid	1	G6PDD_Untreated_1_1	G6PDD_Untreated_1	1.0	Amino acids degradation intermediates	True	5.665e+04	-5.747	-5.747	-0.3373
368	7-Me-Guanine	Nucleotide	1	G6PDD_Untreated_1_1	G6PDD_Untreated_1	1.0	Purine derivatives in RNAs	True	6.564e+05	-2.212	-2.212	-0.0224
208	Pro-OH-Pro	Amino acid	1	G6PDD_Untreated_1_1	G6PDD_Untreated_1	1.0	Amino acid derivatives	True	2.494e+07	3.036	3.036	-0.2938
366	N2,N2-DiMe-Guanosine	Nucleotide	1	G6PDD_Untreated_1_1	G6PDD_Untreated_1	1.0	Purine derivatives in RNAs	True	1.164e+05	-4.708	-4.708	-0.5943
352	3'-AMP	Nucleotide	1	G6PDD_Untreated_1_1	G6PDD_Untreated_1	1.0	Purine derivatives in signaling	True	9.812e+05	-1.633	-1.633	-0.1457
363	N6-Carbamoyl-Thr-Adenosine	Nucleotide	1	G6PDD_Untreated_1_1	G6PDD_Untreated_1	1.0	Purine derivatives in RNAs	True	1.516e+05	-4.327	-4.327	0.3582
314	Cys-Glutathione Disulfide	Amino acid	1	G6PDD_Untreated_1_1	G6PDD_Untreated_1	0.25	Oxidative stress markers	False			-5.284	-2
382	Orotidine	Nucleotide	1	G6PDD_Untreated_1_1	G6PDD_Untreated_1	1.0	Pyrimidine (UMP) biosynthesis	True	1.058e+05	-4.846	-4.846	-0.5545
307	Cys-Gly	Amino acid	1	G6PDD_Untreated_1_1	G6PDD_Untreated_1	1.0	Glutathione biosynthesis	True	1.106e+07	1.862	1.862	0.2551
64	Sedoheptulose-7-P	Carbon	1	G6PDD_Untreated_1_1	G6PDD_Untreated_1	1.0	Pentose phosphate pathway (PPP)	True	4.087e+06	0.4259	0.4259	0.2718
142	N-Ac-Asp-Glu	Amino acid	1	G6PDD_Untreated_1_1	G6PDD_Untreated_1	1.0	Amino acid derivatives	True	3.824e+06	0.33	0.33	1.475
708	Thiamin-PP	Cofactor	1	G6PDD_Untreated_1_1	G6PDD_Untreated_1	1.0	Cofactors	True	3.779e+04	-6.331	-6.331	-0.3472
182	P-Cresol Sulfate	Amino acid	1	G6PDD_Untreated_1_1	G6PDD_Untreated_1	1.0	Amino acid derivatives	True	1.783e+05	-4.093	-4.093	-0.8968

Metabolite ID	Name	Super Pathway	Dataset	Sample ID	Group ID	Detection Fraction	Pathway	Detected	Raw Intensity	Log2 Norm Intensity	Norm Imputed	Log2 Ctrl Norm
250	Gly-Ile	Amino acid	1	G6PDD_Untreated_1_1	G6PDD_Untreated_1	1.0	Dipeptides	True	1.542e+06	-0.9807	-0.9807	0.3365
286	gamma-Glu-Glu	Amino acid	1	G6PDD_Untreated_1_1	G6PDD_Untreated_1	1.0	Gamma-glutamyl dipeptides	True	5.515e+06	0.8583	0.8583	0.8405
739	Deoxycarnitine	Cofactor	1	G6PDD_Untreated_1_1	G6PDD_Untreated_1	1.0	Carnitine biosynthesis	True	6.277e+07	4.367	4.367	0.6156
203	DiMe-Arg	Amino acid	1	G6PDD_Untreated_1_1	G6PDD_Untreated_1	1.0	Amino acid derivatives	True	1.166e+08	5.26	5.26	-0.3723
351	2'-AMP	Nucleotide	1	G6PDD_Untreated_1_1	G6PDD_Untreated_1	1.0	Purine derivatives in signaling	True	1.962e+05	-3.955	-3.955	-1.686
8	Cytidine 5'-P-N-Ac-Neuraminate	Carbon	1	G6PDD_Untreated_1_1	G6PDD_Untreated_1	1.0	Aminosugar biosynthesis	True	1.129e+06	-1.43	-1.43	0.3157
285	gamma-Glu-Ala	Amino acid	1	G6PDD_Untreated_1_1	G6PDD_Untreated_1	1.0	Gamma-glutamyl dipeptides	True	5.562e+05	-2.451	-2.451	0.7875
224	N-Ac-Ser	Amino acid	1	G6PDD_Untreated_1_1	G6PDD_Untreated_1	1.0	N-acetylated amino acids	True	1.037e+07	1.77	1.77	-0.1094
244	Ala-Leu	Amino acid	1	G6PDD_Untreated_1_1	G6PDD_Untreated_1	1.0	Dipeptides	True	3.595e+06	0.2408	0.2408	-0.8972
207	N-Me-Pro	Amino acid	1	G6PDD_Untreated_1_1	G6PDD_Untreated_1	1.0	Amino acid derivatives	True	1.345e+06	-1.178	-1.178	-0.2477
171	Cys Sulfinic Acid	Amino acid	1	G6PDD_Untreated_1_1	G6PDD_Untreated_1	0.75	Amino acid derivatives	True	2.701e+05	-3.493	-3.493	-0.1959
181	O-Me-Tyr	Amino acid	1	G6PDD_Untreated_1_1	G6PDD_Untreated_1	1.0	Amino acid derivatives	True	3.359e+05	-3.179	-3.179	0.1908
240	N-Ac-Putrescine	Amino acid	1	G6PDD_Untreated_1_1	G6PDD_Untreated_1	1.0	Polyamine derivatives	True	5.703e+05	-2.415	-2.415	-0.05169
176	S-Me-Met	Amino acid	1	G6PDD_Untreated_1_1	G6PDD_Untreated_1	1.0	Amino acid derivatives	True	7.453e+05	-2.029	-2.029	-0.1876
339	AICAR	Nucleotide	1	G6PDD_Untreated_1_1	G6PDD_Untreated_1	1.0	IMP biosynthesis	True	3.5e+05	-3.12	-3.12	1.181
141	gamma-Carboxy-Glu	Amino acid	1	G6PDD_Untreated_1_1	G6PDD_Untreated_1	1.0	Amino acid derivatives	True	1.334e+06	-1.189	-1.189	-0.404
392	3'-UMP	Nucleotide	1	G6PDD_Untreated_1_1	G6PDD_Untreated_1	0.75	Pyrimidine derivatives in signaling	False			-5.207	-1.174
355	3'-GMP	Nucleotide	1	G6PDD_Untreated_1_1	G6PDD_Untreated_1	1.0	Purine derivatives in signaling	True	1.455e+05	-4.386	-4.386	-0.4488
282	Val-Leu	Amino acid	1	G6PDD_Untreated_1_1	G6PDD_Untreated_1	1.0	Dipeptides	True	2.002e+06	-0.6035	-0.6035	-1.575
140	Carboxyethyl-GABA	Amino acid	1	G6PDD_Untreated_1_1	G6PDD_Untreated_1	1.0	Amino acid derivatives	True	1.109e+06	-1.456	-1.456	-0.02454
258	Ile-Gly	Amino acid	1	G6PDD_Untreated_1_1	G6PDD_Untreated_1	1.0	Dipeptides	True	2.092e+07	2.782	2.782	-0.53

Metabolite ID	Name	Super Pathway	Dataset	Sample ID	Group ID	Detection Fraction	Pathway	Detected	Raw Intensity	Log2 Norm Intensity	Norm Imputed	Log2 Ctrl Norm
260	Leu-Ala	Amino acid	1	G6PDD_Untreated_1_1	G6PDD_Untreated_1	1.0	Dipeptides	True	4.407e+06	0.5346	0.5346	-0.7666
265	Lys-Leu	Amino acid	1	G6PDD_Untreated_1_1	G6PDD_Untreated_1	1.0	Dipeptides	True	3.163e+05	-3.266	-3.266	-0.6543
263	Leu-Gly	Amino acid	1	G6PDD_Untreated_1_1	G6PDD_Untreated_1	1.0	Dipeptides	True	2.133e+07	2.81	2.81	-0.2116
281	Val-Gly	Amino acid	1	G6PDD_Untreated_1_1	G6PDD_Untreated_1	1.0	Dipeptides	True	4.046e+07	3.733	3.733	-0.786
270	Pro-Gly	Amino acid	1	G6PDD_Untreated_1_1	G6PDD_Untreated_1	1.0	Dipeptides	True	7.809e+06	1.36	1.36	0.6133
114	Imidazole Propionate	Amino acid	1	G6PDD_Untreated_1_1	G6PDD_Untreated_1	1.0	Amino acids degradation intermediates	True	1.099e+06	-1.469	-1.469	0.8418
267	Phe-Gly	Amino acid	1	G6PDD_Untreated_1_1	G6PDD_Untreated_1	1.0	Dipeptides	True	3.903e+07	3.681	3.681	-0.4125
266	Phe-Ala	Amino acid	1	G6PDD_Untreated_1_1	G6PDD_Untreated_1	1.0	Dipeptides	True	3.844e+06	0.3375	0.3375	-0.7392
278	Tyr-Gly	Amino acid	1	G6PDD_Untreated_1_1	G6PDD_Untreated_1	1.0	Dipeptides	True	9.858e+06	1.696	1.696	-0.4862
255	His-Ala	Amino acid	1	G6PDD_Untreated_1_1	G6PDD_Untreated_1	1.0	Dipeptides	True	8.053e+05	-1.918	-1.918	-0.5802
280	Val-Gln	Amino acid	1	G6PDD_Untreated_1_1	G6PDD_Untreated_1	1.0	Dipeptides	True	4.756e+06	0.6447	0.6447	-0.9734
143	S-1-Pyrroline-5-Carboxylate	Amino acid	1	G6PDD_Untreated_1_1	G6PDD_Untreated_1	1.0	Amino acid derivatives	True	6.004e+05	-2.341	-2.341	-0.2731
232	SAH	Amino acid	1	G6PDD_Untreated_1_1	G6PDD_Untreated_1	1.0	SAM metabolism	True	1.933e+06	-0.6545	-0.6545	-0.1691
20	Erythronate	Carbon	1	G6PDD_Untreated_1_1	G6PDD_Untreated_1	1.0	Aminosugar derivatives	True	4.73e+07	3.959	3.959	0.2174
248	Gln-Leu	Amino acid	1	G6PDD_Untreated_1_1	G6PDD_Untreated_1	1.0	Dipeptides	True	8.758e+05	-1.797	-1.797	-0.8913
276	Trp-Gly	Amino acid	1	G6PDD_Untreated_1_1	G6PDD_Untreated_1	1.0	Dipeptides	True	5.8e+05	-2.391	-2.391	-1.313
205	N-delta-Ac-Ornithine	Amino acid	1	G6PDD_Untreated_1_1	G6PDD_Untreated_1	1.0	Amino acid derivatives	True	1.73e+06	-0.8145	-0.8145	0.3065
163	Formimino-Glu	Amino acid	1	G6PDD_Untreated_1_1	G6PDD_Untreated_1	1.0	Amino acid derivatives	True	1.242e+06	-1.293	-1.293	-0.05274
204	N-Me-Arg	Amino acid	1	G6PDD_Untreated_1_1	G6PDD_Untreated_1	1.0	Amino acid derivatives	True	2.502e+07	3.04	3.04	0.4531
242	Guanidino-Ac	Amino acid	1	G6PDD_Untreated_1_1	G6PDD_Untreated_1	0.75	Creatine biosynthesis	False			-4.685	-0.8041

Metabolite ID	Name	Super Pathway	Dataset	Sample ID	Group ID	Detection Fraction	Pathway	Detected	Raw Intensity	Log2 Norm Intensity	Norm Imputed	Log2 Ctrl Norm
300	gamma-Glu-Val	Amino acid	1	G6PDD_Untreated_1_1	G6PDD_Untreated_1	0.75	Gamma-glutamyl dipeptides	True	4.514e+06	0.5692	0.5692	-0.05159
53	Ac-CoA	Carbon	1	G6PDD_Untreated_1_1	G6PDD_Untreated_1	0.5	Acetyl-CoA	False			-7.274	-0.5114
18	Maltotriose	Carbon	1	G6PDD_Untreated_1_1	G6PDD_Untreated_1	1.0	Glycogen degradation	True	1.893e+06	-0.6844	-0.6844	-0.5267
294	gamma-Glu-Met	Amino acid	1	G6PDD_Untreated_1_1	G6PDD_Untreated_1	0.5	Gamma-glutamyl dipeptides	True	2.639e+05	-3.527	-3.527	-0.04475
174	Met Sulfone	Amino acid	1	G6PDD_Untreated_1_1	G6PDD_Untreated_1	1.0	Amino acid derivatives	True	5.742e+05	-2.406	-2.406	0.6731
175	N-Ac-Met Sulfoxide	Amino acid	1	G6PDD_Untreated_1_1	G6PDD_Untreated_1	1.0	Amino acid derivatives	True	1.822e+06	-0.7393	-0.7393	-0.7157
25	Mannitol/Sorbitol	Carbon	1	G6PDD_Untreated_1_1	G6PDD_Untreated_1	1.0	Sugars and sugar alcohols	True	1.098e+07	1.851	1.851	-0.3732
6	UDP-GlcNAc	Carbon	1	G6PDD_Untreated_1_1	G6PDD_Untreated_1	0.5	Aminosugar biosynthesis	False			-3.589	-0.4814
145	Pyro-Gln	Amino acid	1	G6PDD_Untreated_1_1	G6PDD_Untreated_1	1.0	Amino acid derivatives	True	5.434e+06	0.8369	0.8369	-2.89e-03
705	Coenzyme A	Cofactor	1	G6PDD_Untreated_1_1	G6PDD_Untreated_1	1.0	Cofactors	True	1.221e+05	-4.639	-4.639	-0.6801
319	2'-dAMP	Nucleotide	1	G6PDD_Untreated_1_1	G6PDD_Untreated_1	0.75	Deoxy-nucleotides	False			-4.337	-0.6568
119	alpha-OH-Isovalerate	Amino acid	1	G6PDD_Untreated_1_1	G6PDD_Untreated_1	0.5	Amino acids degradation intermediates	True	3.965e+05	-2.94	-2.94	0.3589
46	Fructose 1,6-PP / Glucose 1,6-PP / Inositol-1,4-PP / Inositol-1,3-PP	Carbon	1	G6PDD_Untreated_1_1	G6PDD_Untreated_1	1.0	Glycolysis, GNG	True	8.182e+07	4.749	4.749	-0.08178
137	1-Me-Guanidine	Amino acid	1	G6PDD_Untreated_1_1	G6PDD_Untreated_1	1.0	Amino acids degradation intermediates	True	6.8e+04	-5.483	-5.483	-0.3307
23	N-GlcNAc-Asn	Carbon	1	G6PDD_Untreated_1_1	G6PDD_Untreated_1	1.0	Aminosugar derivatives	True	7.23e+06	1.249	1.249	0.536
262	Leu-Gln	Amino acid	1	G6PDD_Untreated_1_1	G6PDD_Untreated_1	1.0	Dipeptides	True	4.614e+06	0.6008	0.6008	-0.6955
24	Fructose	Carbon	1	G6PDD_Untreated_1_1	G6PDD_Untreated_1	1.0	Sugars and sugar alcohols	True	7.025e+06	1.207	1.207	-0.7416
197	C-Glycosyl-Trp	Amino acid	1	G6PDD_Untreated_1_1	G6PDD_Untreated_1	1.0	Amino acid derivatives	True	2.79e+06	-0.1246	-0.1246	0.1744
33	Arabitol/Xylitol	Carbon	1	G6PDD_Untreated_1_1	G6PDD_Untreated_1	1.0	Sugars and sugar alcohols	True	5.645e+05	-2.43	-2.43	-0.5701

Metabolite ID	Name	Super Pathway	Dataset	Sample ID	Group ID	Detection Fraction	Pathway	Detected	Raw Intensity	Log2 Norm Intensity	Norm Imputed	Log2 Ctrl Norm
128	N2-Ac-Lys/N6-Ac-Lys	Amino acid	1	G6PDD_Untreated_1_1	G6PDD_Untreated_1	1.0	Amino acids degradation intermediates	True	5.802e+06	0.9315	0.9315	-0.2788
42	2-Me-Citrate	Carbon	1	G6PDD_Untreated_1_1	G6PDD_Untreated_1	1.0	Propionate metabolism	True	8.216e+04	-5.211	-5.211	-0.8276
12	Glucuronate 1-P	Carbon	1	G6PDD_Untreated_1_1	G6PDD_Untreated_1	1.0	Polysaccharide biosynthesis	True	1.197e+06	-1.346	-1.346	-0.1033
76	Gln	Amino acid	1	G6PDD_Untreated_1_2	G6PDD_Untreated_1	1.0	Proteinogenic amino acids	True	1.363e+09	9.29	9.29	0.6091
89	Trp	Amino acid	1	G6PDD_Untreated_1_2	G6PDD_Untreated_1	1.0	Proteinogenic amino acids	True	1.151e+08	5.724	5.724	0.04789
723	beta-Ala	Cofactor	1	G6PDD_Untreated_1_2	G6PDD_Untreated_1	1.0	Coenzyme A biosynthesis	True	4.091e+07	4.232	4.232	1.222
75	Glu	Amino acid	1	G6PDD_Untreated_1_2	G6PDD_Untreated_1	1.0	Proteinogenic amino acids	True	4.604e+09	11.05	11.05	0.3093
77	Gly	Amino acid	1	G6PDD_Untreated_1_2	G6PDD_Untreated_1	1.0	Proteinogenic amino acids	True	1.074e+08	5.624	5.624	8.34e-03
80	His	Amino acid	1	G6PDD_Untreated_1_2	G6PDD_Untreated_1	1.0	Proteinogenic amino acids	True	1.831e+07	3.072	3.072	-0.3435
82	Leu	Amino acid	1	G6PDD_Untreated_1_2	G6PDD_Untreated_1	1.0	Proteinogenic amino acids	True	1.411e+09	9.34	9.34	0.1187
87	Phe	Amino acid	1	G6PDD_Untreated_1_2	G6PDD_Untreated_1	1.0	Proteinogenic amino acids	True	1.016e+09	8.866	8.866	0.05458
130	Glutarate	Amino acid	1	G6PDD_Untreated_1_2	G6PDD_Untreated_1	1.0	Amino acids degradation intermediates	True	2.777e+05	-2.971	-2.971	-0.5448
196	5-OH-Indole-Ac	Amino acid	1	G6PDD_Untreated_1_2	G6PDD_Untreated_1	0.75	Amino acid derivatives	True	6.203e+04	-5.134	-5.134	-1.122
74	Asp	Amino acid	1	G6PDD_Untreated_1_2	G6PDD_Untreated_1	1.0	Proteinogenic amino acids	True	5.268e+08	7.918	7.918	0.02661
236	Spermidine	Amino acid	1	G6PDD_Untreated_1_2	G6PDD_Untreated_1	1.0	Polyamines	True	2.977e+07	3.773	3.773	-0.0886
73	Asn	Amino acid	1	G6PDD_Untreated_1_2	G6PDD_Untreated_1	1.0	Proteinogenic amino acids	True	2.293e+08	6.718	6.718	0.04714
243	Creatinine	Amino acid	1	G6PDD_Untreated_1_2	G6PDD_Untreated_1	1.0	Creatine degradation	True	7.456e+07	5.098	5.098	0.09538
376	Cytidine	Nucleotide	1	G6PDD_Untreated_1_2	G6PDD_Untreated_1	0.75	Pyrimidine nucleosides	True	2.465e+07	3.501	3.501	2.773
41	Lactate	Carbon	1	G6PDD_Untreated_1_2	G6PDD_Untreated_1	1.0	Respiratory carbon sources	True	1.552e+08	6.156	6.156	-0.3263
58	alpha-Ketoglutarate	Carbon	1	G6PDD_Untreated_1_2	G6PDD_Untreated_1	1.0	TCA cycle	True	3.071e+06	0.4961	0.4961	0.4794

Metabolite ID	Name	Super Pathway	Dataset	Sample ID	Group ID	Detection Fraction	Pathway	Detected	Raw Intensity	Log2 Norm Intensity	Norm Imputed	Log2 Ctrl Norm
69	3-OH-Butyrate	Carbon	1	G6PDD_Untreated_1_2	G6PDD_Untreated_1	0.75	Ketone bodies	True	2.232e+05	-3.286	-3.286	-0.3019
343	Adenine	Nucleotide	1	G6PDD_Untreated_1_2	G6PDD_Untreated_1	1.0	Purine bases	True	7.065e+06	1.698	1.698	0.241
336	Adenosine	Nucleotide	1	G6PDD_Untreated_1_2	G6PDD_Untreated_1	1.0	Purine nucleosides	True	1.125e+08	5.691	5.691	2.268
722	ADP-Ribose	Cofactor	1	G6PDD_Untreated_1_2	G6PDD_Untreated_1	1.0	Derivatives of NA, nicotinamide and NAD	True	2.995e+05	-2.862	-2.862	0.1398
383	Cytosine	Nucleotide	1	G6PDD_Untreated_1_2	G6PDD_Untreated_1	0.75	Pyrimidine bases	True	1.21e+05	-4.17	-4.17	0.6851
3	Glucosamine 6-P	Carbon	1	G6PDD_Untreated_1_2	G6PDD_Untreated_1	1.0	Aminosugar biosynthesis	True	6.352e+05	-1.777	-1.777	-0.1101
717	Nicotinamide	Cofactor	1	G6PDD_Untreated_1_2	G6PDD_Untreated_1	1.0	NAD biosynthesis	True	3.536e+07	4.021	4.021	-0.494
51	PEP	Carbon	1	G6PDD_Untreated_1_2	G6PDD_Untreated_1	1.0	Glycolysis, GNG	True	5.315e+06	1.287	1.287	0.2361
237	Spermine	Amino acid	1	G6PDD_Untreated_1_2	G6PDD_Untreated_1	1.0	Polyamines	True	1.388e+06	-0.6494	-0.6494	0.8412
385	Uracil	Nucleotide	1	G6PDD_Untreated_1_2	G6PDD_Untreated_1	1.0	Pyrimidine bases	True	1.044e+07	2.261	2.261	1.996
377	Uridine	Nucleotide	1	G6PDD_Untreated_1_2	G6PDD_Untreated_1	1.0	Pyrimidine nucleosides	True	4.293e+07	4.301	4.301	0.2523
348	Allantoin	Nucleotide	1	G6PDD_Untreated_1_2	G6PDD_Untreated_1	1.0	Purine degradation	True	1.737e+06	-0.3262	-0.3262	-0.1506
335	Inosine	Nucleotide	1	G6PDD_Untreated_1_2	G6PDD_Untreated_1	1.0	Purine nucleosides	True	1.3e+08	5.899	5.899	0.4003
81	Ile	Amino acid	1	G6PDD_Untreated_1_2	G6PDD_Untreated_1	1.0	Proteinogenic amino acids	True	9.645e+08	8.791	8.791	0.07571
72	Ala	Amino acid	1	G6PDD_Untreated_1_2	G6PDD_Untreated_1	1.0	Proteinogenic amino acids	True	1.089e+09	8.965	8.965	0.464
79	Thr	Amino acid	1	G6PDD_Untreated_1_2	G6PDD_Untreated_1	1.0	Proteinogenic amino acids	True	6.485e+08	8.218	8.218	0.2086
88	Tyr	Amino acid	1	G6PDD_Untreated_1_2	G6PDD_Untreated_1	1.0	Proteinogenic amino acids	True	4.692e+08	7.751	7.751	0.2276
84	Lys	Amino acid	1	G6PDD_Untreated_1_2	G6PDD_Untreated_1	1.0	Proteinogenic amino acids	True	4.595e+08	7.721	7.721	-0.269
86	Met	Amino acid	1	G6PDD_Untreated_1_2	G6PDD_Untreated_1	1.0	Proteinogenic amino acids	True	6.795e+08	8.286	8.286	0.05792
61	Malate	Carbon	1	G6PDD_Untreated_1_2	G6PDD_Untreated_1	1.0	TCA cycle	True	3.395e+08	7.284	7.284	0.1126

Metabolite ID	Name	Super Pathway	Dataset	Sample ID	Group ID	Detection Fraction	Pathway	Detected	Raw Intensity	Log2 Norm Intensity	Norm Imputed	Log2 Ctrl Norm
235	Putrescine	Amino acid	1	G6PDD_Untreated_1_2	G6PDD_Untreated_1	1.0	Polyamines	True	2.006e+06	-0.1181	-0.1181	0.3731
324	2'-dU	Nucleotide	1	G6PDD_Untreated_1_2	G6PDD_Untreated_1	0.5	Deoxy-nucleosides	True	1.014e+05	-4.424	-4.424	-0.04385
49	3-P-Glycerate	Carbon	1	G6PDD_Untreated_1_2	G6PDD_Untreated_1	1.0	Glycolysis, GNG	True	2.076e+07	3.253	3.253	-0.01255
189	Kynurenate	Amino acid	1	G6PDD_Untreated_1_2	G6PDD_Untreated_1	0.75	Amino acid derivatives	True	3.357e+04	-6.019	-6.019	-0.2215
234	5-Me-Thioadenosine	Amino acid	1	G6PDD_Untreated_1_2	G6PDD_Untreated_1	1.0	SAM metabolism	True	1.256e+07	2.528	2.528	0.6422
59	Succinate	Carbon	1	G6PDD_Untreated_1_2	G6PDD_Untreated_1	1.0	TCA cycle	True	3.057e+06	0.4892	0.4892	0.1881
36	Ribose	Carbon	1	G6PDD_Untreated_1_2	G6PDD_Untreated_1	1.0	Sugars and sugar alcohols	True	5.202e+05	-2.066	-2.066	-0.8322
133	Ornithine	Amino acid	1	G6PDD_Untreated_1_2	G6PDD_Untreated_1	1.0	Amino acids degradation intermediates	True	7.141e+07	5.035	5.035	0.5557
313	5-Oxoproline	Amino acid	1	G6PDD_Untreated_1_2	G6PDD_Untreated_1	1.0	Glutathione derivatives	True	9.33e+06	2.099	2.099	0.01154
165	N-6-Tri-Me-Lys	Amino acid	1	G6PDD_Untreated_1_2	G6PDD_Untreated_1	1.0	Amino acid derivatives	True	2.767e+07	3.667	3.667	-0.09626
380	Orotate	Nucleotide	1	G6PDD_Untreated_1_2	G6PDD_Untreated_1	1.0	Pyrimidine (UMP) biosynthesis	True	2.055e+05	-3.405	-3.405	0.2249
724	Pantothenate	Cofactor	1	G6PDD_Untreated_1_2	G6PDD_Untreated_1	1.0	Coenzyme A biosynthesis	True	2.665e+08	6.935	6.935	0.6251
150	N-Me-Gly	Amino acid	1	G6PDD_Untreated_1_2	G6PDD_Untreated_1	1.0	Amino acid derivatives	True	2.12e+06	-0.03847	-0.03847	0.262
122	3-OH-Isobutyrate	Amino acid	1	G6PDD_Untreated_1_2	G6PDD_Untreated_1	0.5	Amino acids degradation intermediates	True	2.424e+05	-3.167	-3.167	-0.01138
241	4-Acetamidobutanoate	Amino acid	1	G6PDD_Untreated_1_2	G6PDD_Untreated_1	1.0	Polyamine derivatives	True	3.28e+06	0.5909	0.5909	0.1499
711	alpha-Tocopherol	Cofactor	1	G6PDD_Untreated_1_2	G6PDD_Untreated_1	1.0	Cofactors	True	8.234e+06	1.919	1.919	1.138
55	Citrate	Carbon	1	G6PDD_Untreated_1_2	G6PDD_Untreated_1	1.0	TCA cycle	True	1.358e+07	2.64	2.64	0.6913
387	3-Aminoisobutyrate	Nucleotide	1	G6PDD_Untreated_1_2	G6PDD_Untreated_1	1.0	Pyrimidine degradation	True	9.095e+05	-1.26	-1.26	0.754
338	Guanosine	Nucleotide	1	G6PDD_Untreated_1_2	G6PDD_Untreated_1	1.0	Purine nucleosides	True	9.882e+07	5.504	5.504	0.398
209	N-Ac-Ala	Amino acid	1	G6PDD_Untreated_1_2	G6PDD_Untreated_1	1.0	N-acetylated amino acids	True	7.67e+05	-1.506	-1.506	-0.3044

Metabolite ID	Name	Super Pathway	Dataset	Sample ID	Group ID	Detection Fraction	Pathway	Detected	Raw Intensity	Log2 Norm Intensity	Norm Imputed	Log2 Ctrl Norm
221	N-Ac-Met	Amino acid	1	G6PDD_Untreated_1_2	G6PDD_Untreated_1	1.0	N-acetylated amino acids	True	4.928e+06	1.178	1.178	-0.566
228	N-Ac-Val	Amino acid	1	G6PDD_Untreated_1_2	G6PDD_Untreated_1	1.0	N-acetylated amino acids	True	6.804e+04	-5	-5	0.3705
346	Urate	Nucleotide	1	G6PDD_Untreated_1_2	G6PDD_Untreated_1	1.0	Purine degradation	True	5.624e+05	-1.953	-1.953	-0.8903
90	Arg	Amino acid	1	G6PDD_Untreated_1_2	G6PDD_Untreated_1	1.0	Proteinogenic amino acids	True	2.03e+09	9.864	9.864	0.1586
60	Fumarate	Carbon	1	G6PDD_Untreated_1_2	G6PDD_Untreated_1	1.0	TCA cycle	True	3.897e+06	0.8397	0.8397	-0.1755
78	Ser	Amino acid	1	G6PDD_Untreated_1_2	G6PDD_Untreated_1	1.0	Proteinogenic amino acids	True	3.755e+08	7.43	7.43	0.1726
83	Val	Amino acid	1	G6PDD_Untreated_1_2	G6PDD_Untreated_1	1.0	Proteinogenic amino acids	True	5.153e+08	7.886	7.886	0.01414
734	Pyridoxal	Cofactor	1	G6PDD_Untreated_1_2	G6PDD_Untreated_1	1.0	PLP biosynthesis and salvage	True	4.478e+06	1.04	1.04	-0.743
136	Urea	Amino acid	1	G6PDD_Untreated_1_2	G6PDD_Untreated_1	1.0	Amino acids degradation intermediates	True	1.831e+06	-0.2504	-0.2504	-0.4017
67	Ribose 1-P	Carbon	1	G6PDD_Untreated_1_2	G6PDD_Untreated_1	1.0	Pentose phosphate pathway (PPP)	True	7.439e+06	1.772	1.772	1.151
284	Carnosine	Amino acid	1	G6PDD_Untreated_1_2	G6PDD_Untreated_1	1.0	Dipeptides	True	7.844e+05	-1.473	-1.473	0.6351
306	gamma-Glu-Cys	Amino acid	1	G6PDD_Untreated_1_2	G6PDD_Untreated_1	1.0	Glutathione biosynthesis	True	2.767e+06	0.3455	0.3455	0.6823
712	Retinol (Vit A)	Cofactor	1	G6PDD_Untreated_1_2	G6PDD_Untreated_1	1.0	Cofactors	True	4.43e+05	-2.297	-2.297	0.136
85	Cys	Amino acid	1	G6PDD_Untreated_1_2	G6PDD_Untreated_1	1.0	Proteinogenic amino acids	True	3.316e+07	3.928	3.928	-0.5853
91	Pro	Amino acid	1	G6PDD_Untreated_1_2	G6PDD_Untreated_1	1.0	Proteinogenic amino acids	True	1.717e+09	9.623	9.623	0.215
308	Glutathione, Reduced	Amino acid	1	G6PDD_Untreated_1_2	G6PDD_Untreated_1	1.0	Glutathione	True	7.36e+08	8.401	8.401	1.445
107	Citrulline	Amino acid	1	G6PDD_Untreated_1_2	G6PDD_Untreated_1	1.0	Amino acids biosynthesis intermediates	True	1.86e+07	3.094	3.094	0.2696
328	IMP	Nucleotide	1	G6PDD_Untreated_1_2	G6PDD_Untreated_1	0.5	Purine nucleotides	False			-4.53	-0.2088
706	FAD	Cofactor	1	G6PDD_Untreated_1_2	G6PDD_Untreated_1	1.0	Cofactors	True	5.965e+05	-1.868	-1.868	0.3213
735	Pyridoxamine	Cofactor	1	G6PDD_Untreated_1_2	G6PDD_Untreated_1	1.0	PLP biosynthesis and salvage	True	1.487e+06	-0.5503	-0.5503	-0.3573

Metabolite ID	Name	Super Pathway	Dataset	Sample ID	Group ID	Detection Fraction	Pathway	Detected	Raw Intensity	Log2 Norm Intensity	Norm Imputed	Log2 Ctrl Norm
199	Serotonin	Amino acid	1	G6PDD_Untreated_1_2	G6PDD_Untreated_1	1.0	Amino acid derivatives	True	5.045e+06	1.212	1.212	-0.06056
370	CMP	Nucleotide	1	G6PDD_Untreated_1_2	G6PDD_Untreated_1	1.0	Pyrimidine nucleotides	True	6.864e+06	1.656	1.656	-0.1826
287	gamma-Glu-Gln	Amino acid	1	G6PDD_Untreated_1_2	G6PDD_Untreated_1	1.0	Gamma-glutamyl dipeptides	True	3.335e+06	0.6149	0.6149	-0.2261
14	UDP-Glucuronate	Carbon	1	G6PDD_Untreated_1_2	G6PDD_Untreated_1	1.0	Polysaccharide biosynthesis	True	9.594e+05	-1.183	-1.183	0.7667
229	N-Formyl-Met	Amino acid	1	G6PDD_Untreated_1_2	G6PDD_Untreated_1	1.0	N-formylated amino acids	True	2.083e+05	-3.386	-3.386	0.4362
350	3',5'-cAMP	Nucleotide	1	G6PDD_Untreated_1_2	G6PDD_Untreated_1	1.0	Purine derivatives in signaling	True	1.772e+05	-3.619	-3.619	-1.02e-04
371	CDP	Nucleotide	1	G6PDD_Untreated_1_2	G6PDD_Untreated_1	1.0	Pyrimidine nucleotides	True	1.178e+05	-4.208	-4.208	-0.8883
372	CTP	Nucleotide	1	G6PDD_Untreated_1_2	G6PDD_Untreated_1	0.75	Pyrimidine nucleotides	True	1.086e+05	-4.326	-4.326	-0.672
333	GDP	Nucleotide	1	G6PDD_Untreated_1_2	G6PDD_Untreated_1	0.5	Purine nucleotides	False			-4.484	-1.983
332	GMP	Nucleotide	1	G6PDD_Untreated_1_2	G6PDD_Untreated_1	1.0	Purine nucleotides	True	1.723e+06	-0.3379	-0.3379	-1.037
373	UMP	Nucleotide	1	G6PDD_Untreated_1_2	G6PDD_Untreated_1	1.0	Pyrimidine nucleotides	True	8.147e+05	-1.418	-1.418	-0.3633
389	3'-CMP	Nucleotide	1	G6PDD_Untreated_1_2	G6PDD_Untreated_1	1.0	Pyrimidine derivatives in signaling	True	2.316e+06	0.0888	0.0888	1.14
330	ADP	Nucleotide	1	G6PDD_Untreated_1_2	G6PDD_Untreated_1	1.0	Purine nucleotides	True	5.132e+05	-2.085	-2.085	-1.789
342	Hypoxanthine	Nucleotide	1	G6PDD_Untreated_1_2	G6PDD_Untreated_1	1.0	Purine bases	True	3.858e+07	4.147	4.147	0.2076
736	Pyridoxamine-P	Cofactor	1	G6PDD_Untreated_1_2	G6PDD_Untreated_1	1.0	PLP biosynthesis and salvage	True	1.785e+05	-3.609	-3.609	-0.6305
148	Betaine	Amino acid	1	G6PDD_Untreated_1_2	G6PDD_Untreated_1	1.0	Amino acid derivatives	True	8.613e+07	5.306	5.306	0.4998
344	Xanthine	Nucleotide	1	G6PDD_Untreated_1_2	G6PDD_Untreated_1	1.0	Purine bases	True	3.153e+06	0.534	0.534	-0.3735
386	3-Ureidopropionate	Nucleotide	1	G6PDD_Untreated_1_2	G6PDD_Untreated_1	1.0	Pyrimidine degradation	True	1.063e+06	-1.034	-1.034	-0.1151
149	DiMe-Gly	Amino acid	1	G6PDD_Untreated_1_2	G6PDD_Untreated_1	1.0	Amino acid derivatives	True	1.641e+06	-0.4082	-0.4082	0.1121
703	NAD+	Cofactor	1	G6PDD_Untreated_1_2	G6PDD_Untreated_1	1.0	Cofactors	True	2.254e+07	3.372	3.372	0.6352
709	Pyridoxal-P	Cofactor	1	G6PDD_Untreated_1_2	G6PDD_Untreated_1	1.0	Cofactors	True	4.841e+05	-2.169	-2.169	0.1386

Metabolite ID	Name	Super Pathway	Dataset	Sample ID	Group ID	Detection Fraction	Pathway	Detected	Raw Intensity	Log2 Norm Intensity	Norm Imputed	Log2 Ctrl Norm
731	Thiamin (Vitamin B1)	Cofactor	1	G6PDD_Untreated_1_2	G6PDD_Untreated_1	1.0	TPP biosynthesis	True	4.871e+06	1.161	1.161	0.4137
374	UDP	Nucleotide	1	G6PDD_Untreated_1_2	G6PDD_Untreated_1	1.0	Pyrimidine nucleotides	True	6.008e+05	-1.858	-1.858	-0.3039
102	2-Aminoadipate	Amino acid	1	G6PDD_Untreated_1_2	G6PDD_Untreated_1	1.0	Amino acids biosynthesis intermediates	True	5.253e+05	-2.052	-2.052	0.05789
45	Fructose-6-P	Carbon	1	G6PDD_Untreated_1_2	G6PDD_Untreated_1	1.0	Glycolysis, GNG	True	2.143e+06	-0.02311	-0.02311	-0.09839
320	TMP	Nucleotide	1	G6PDD_Untreated_1_2	G6PDD_Untreated_1	0.5	Deoxy-nucleotides	False			-6.992	-2.2
341	XMP	Nucleotide	1	G6PDD_Untreated_1_2	G6PDD_Untreated_1	0.25	IMP conversion to AMP & GMP	False			-6.353	-1.778
120	beta-OH-Isovalerate	Amino acid	1	G6PDD_Untreated_1_2	G6PDD_Untreated_1	1.0	Amino acids degradation intermediates	True	8.495e+04	-4.68	-4.68	-1.515
322	2'-dI	Nucleotide	1	G6PDD_Untreated_1_2	G6PDD_Untreated_1	0.5	Deoxy-nucleosides	False			-5.917	-1.791
4	GlcNAc 6-P	Carbon	1	G6PDD_Untreated_1_2	G6PDD_Untreated_1	1.0	Aminosugar biosynthesis	True	5.57e+06	1.355	1.355	1.026
337	Xanthosine	Nucleotide	1	G6PDD_Untreated_1_2	G6PDD_Untreated_1	1.0	Purine nucleosides	True	1.673e+05	-3.702	-3.702	-0.6278
188	Kynurenine	Amino acid	1	G6PDD_Untreated_1_2	G6PDD_Untreated_1	1.0	Amino acid derivatives	True	3.941e+06	0.8558	0.8558	1.547
63	6-P-Gluconate	Carbon	1	G6PDD_Untreated_1_2	G6PDD_Untreated_1	1.0	Pentose phosphate pathway (PPP)	True	7.014e+06	1.688	1.688	-0.3461
40	Glucuronate	Carbon	1	G6PDD_Untreated_1_2	G6PDD_Untreated_1	1.0	Sugars and sugar alcohols	True	7.321e+05	-1.573	-1.573	-0.03365
108	Argininosuccinate	Amino acid	1	G6PDD_Untreated_1_2	G6PDD_Untreated_1	1.0	Amino acids biosynthesis intermediates	True	1.865e+06	-0.2234	-0.2234	2.82e-03
710	Carnitine	Cofactor	1	G6PDD_Untreated_1_2	G6PDD_Untreated_1	1.0	Cofactors	True	1.947e+08	6.483	6.483	0.5051
725	P-Pantetheine	Cofactor	1	G6PDD_Untreated_1_2	G6PDD_Untreated_1	0.75	Coenzyme A biosynthesis	True	2.742e+05	-2.99	-2.99	0.7151
48	DHAP	Carbon	1	G6PDD_Untreated_1_2	G6PDD_Untreated_1	1.0	Glycolysis, GNG	True	2.035e+07	3.225	3.225	0.1279
17	Maltose	Carbon	1	G6PDD_Untreated_1_2	G6PDD_Untreated_1	1.0	Glycogen degradation	True	7.458e+05	-1.546	-1.546	-0.2531
359	N1-Me-Adenosine	Nucleotide	1	G6PDD_Untreated_1_2	G6PDD_Untreated_1	1.0	Purine derivatives in RNAs	True	1.308e+06	-0.7351	-0.7351	0.3026
159	3-Me-His	Amino acid	1	G6PDD_Untreated_1_2	G6PDD_Untreated_1	1.0	Amino acid derivatives	True	1.805e+05	-3.592	-3.592	0.6429

Metabolite ID	Name	Super Pathway	Dataset	Sample ID	Group ID	Detection Fraction	Pathway	Detected	Raw Intensity	Log2 Norm Intensity	Norm Imputed	Log2 Ctrl Norm
155	4-Guanidinobutanoate	Amino acid	1	G6PDD_Untreated_1_2	G6PDD_Untreated_1	1.0	Amino acid derivatives	True	3.239e+06	0.5729	0.5729	2.007
164	5-OH-Lys	Amino acid	1	G6PDD_Untreated_1_2	G6PDD_Untreated_1	1.0	Amino acid derivatives	True	3.75e+05	-2.538	-2.538	-0.7676
357	Adenosine-3',5'-PP	Nucleotide	1	G6PDD_Untreated_1_2	G6PDD_Untreated_1	1.0	Purine byproducts of metabolic processes	True	1.527e+05	-3.834	-3.834	-0.3569
104	Cystathionine	Amino acid	1	G6PDD_Untreated_1_2	G6PDD_Untreated_1	1.0	Amino acids biosynthesis intermediates	True	1.326e+07	2.606	2.606	0.3964
113	Imidazole Lactate	Amino acid	1	G6PDD_Untreated_1_2	G6PDD_Untreated_1	1.0	Amino acids degradation intermediates	True	3.441e+05	-2.662	-2.662	1.015
215	N-Ac-Glu	Amino acid	1	G6PDD_Untreated_1_2	G6PDD_Untreated_1	1.0	N-acetylated amino acids	True	5.76e+06	1.403	1.403	-0.3401
310	S-Lactoyl-Glutathione	Amino acid	1	G6PDD_Untreated_1_2	G6PDD_Untreated_1	1.0	Glutathione derivatives	True	8.548e+06	1.973	1.973	1.616
5	GlcNAc 1-P	Carbon	1	G6PDD_Untreated_1_2	G6PDD_Untreated_1	1.0	Aminosugar biosynthesis	True	7.378e+05	-1.561	-1.561	0.3494
34	Ribitol	Carbon	1	G6PDD_Untreated_1_2	G6PDD_Untreated_1	1.0	Sugars and sugar alcohols	True	2.54e+05	-3.1	-3.1	0.33
10	UDP-Galactose	Carbon	1	G6PDD_Untreated_1_2	G6PDD_Untreated_1	0.75	Polysaccharide biosynthesis	True	1.524e+06	-0.5152	-0.5152	0.1113
13	Guanosine 5'-PP-Fucose	Carbon	1	G6PDD_Untreated_1_2	G6PDD_Untreated_1	1.0	Polysaccharide biosynthesis	True	4.047e+05	-2.428	-2.428	-0.1286
19	Maltotetraose	Carbon	1	G6PDD_Untreated_1_2	G6PDD_Untreated_1	1.0	Glycogen degradation	True	2.901e+06	0.4138	0.4138	0.4575
233	SAM	Amino acid	1	G6PDD_Untreated_1_2	G6PDD_Untreated_1	1.0	SAM metabolism	True	2.74e+06	0.3313	0.3313	0.4877
129	5-Aminovalerate	Amino acid	1	G6PDD_Untreated_1_2	G6PDD_Untreated_1	1.0	Amino acids degradation intermediates	True	6.314e+06	1.536	1.536	0.5341
741	5-Me-THF	Cofactor	1	G6PDD_Untreated_1_2	G6PDD_Untreated_1	1.0	Folate metabolism	True	7.864e+04	-4.791	-4.791	-0.03358
198	Indolelactate	Amino acid	1	G6PDD_Untreated_1_2	G6PDD_Untreated_1	1.0	Amino acid derivatives	True	1.301e+05	-4.065	-4.065	-0.2778
254	Gly-Val	Amino acid	1	G6PDD_Untreated_1_2	G6PDD_Untreated_1	1.0	Dipeptides	True	6.038e+06	1.471	1.471	0.36
291	gamma-Glu-Leu	Amino acid	1	G6PDD_Untreated_1_2	G6PDD_Untreated_1	1.0	Gamma-glutamyl dipeptides	True	1.608e+06	-0.4372	-0.4372	0.05158
173	Met Sulfoxide	Amino acid	1	G6PDD_Untreated_1_2	G6PDD_Untreated_1	1.0	Amino acid derivatives	True	2.974e+07	3.772	3.772	1.204

Metabolite ID	Name	Super Pathway	Dataset	Sample ID	Group ID	Detection Fraction	Pathway	Detected	Raw Intensity	Log2 Norm Intensity	Norm Imputed	Log2 Ctrl Norm
43	Glucose	Carbon	1	G6PDD_Untreated_1_2	G6PDD_Untreated_1	1.0	Glycolysis, GNG	True	8.102e+07	5.217	5.217	0.166
185	Phenyllactate	Amino acid	1	G6PDD_Untreated_1_2	G6PDD_Untreated_1	0.5	Amino acid derivatives	False			-5.917	-0.1051
156	Homo-Arg	Amino acid	1	G6PDD_Untreated_1_2	G6PDD_Untreated_1	1.0	Amino acid derivatives	True	3.599e+06	0.7249	0.7249	-0.4556
135	Homocitrulline	Amino acid	1	G6PDD_Untreated_1_2	G6PDD_Untreated_1	1.0	Amino acids degradation intermediates	True	8.072e+05	-1.432	-1.432	0.2708
719	Nicotinamide MN	Cofactor	1	G6PDD_Untreated_1_2	G6PDD_Untreated_1	1.0	NAD biosynthesis	True	4.187e+06	0.9433	0.9433	0.7577
212	N-Ac-Asp	Amino acid	1	G6PDD_Untreated_1_2	G6PDD_Untreated_1	1.0	N-acetylated amino acids	True	7.934e+06	1.865	1.865	1.168
720	1-Me-Nicotinamide	Cofactor	1	G6PDD_Untreated_1_2	G6PDD_Untreated_1	1.0	Derivatives of NA, nicotinamide and NAD	True	4.557e+08	7.709	7.709	-0.1814
216	N-Ac-Gly	Amino acid	1	G6PDD_Untreated_1_2	G6PDD_Untreated_1	1.0	N-acetylated amino acids	True	1.222e+05	-4.155	-4.155	-0.6151
70	Creatine	Carbon	1	G6PDD_Untreated_1_2	G6PDD_Untreated_1	1.0	Creatine energy storage	True	1.412e+09	9.341	9.341	-0.07636
26	Galactonate	Carbon	1	G6PDD_Untreated_1_2	G6PDD_Untreated_1	1.0	Sugars and sugar alcohols	True	1.807e+06	-0.2689	-0.2689	0.5357
309	Glutathione, Oxidized	Amino acid	1	G6PDD_Untreated_1_2	G6PDD_Untreated_1	1.0	Glutathione	True	1.092e+07	2.326	2.326	1.133
35	Ribonate	Carbon	1	G6PDD_Untreated_1_2	G6PDD_Untreated_1	1.0	Sugars and sugar alcohols	True	1.76e+06	-0.3072	-0.3072	0.22
160	1-Me-His	Amino acid	1	G6PDD_Untreated_1_2	G6PDD_Untreated_1	1.0	Amino acid derivatives	True	3.144e+07	3.852	3.852	0.4107
44	Glucose 6-P	Carbon	1	G6PDD_Untreated_1_2	G6PDD_Untreated_1	1.0	Glycolysis, GNG	True	4.842e+05	-2.169	-2.169	-0.6924
704	NADH	Cofactor	1	G6PDD_Untreated_1_2	G6PDD_Untreated_1	1.0	Cofactors	True	4.989e+06	1.196	1.196	1.842
275	Thr-Phe	Amino acid	1	G6PDD_Untreated_1_2	G6PDD_Untreated_1	1.0	Dipeptides	True	9.466e+05	-1.202	-1.202	0.4513
738	Pyridoxate	Cofactor	1	G6PDD_Untreated_1_2	G6PDD_Untreated_1	1.0	PLP biosynthesis and salvage	True	1.279e+05	-4.09	-4.09	-0.7255
177	3-(4-OH-Phenyl)Lactate	Amino acid	1	G6PDD_Untreated_1_2	G6PDD_Untreated_1	1.0	Amino acid derivatives	True	4.42e+05	-2.301	-2.301	0.1647
206	Trans-4-OH-Pro	Amino acid	1	G6PDD_Untreated_1_2	G6PDD_Untreated_1	1.0	Amino acid derivatives	True	9.377e+07	5.428	5.428	0.2731
329	AMP	Nucleotide	1	G6PDD_Untreated_1_2	G6PDD_Untreated_1	1.0	Purine nucleotides	True	1.56e+07	2.841	2.841	-0.7944

Metabolite ID	Name	Super Pathway	Dataset	Sample ID	Group ID	Detection Fraction	Pathway	Detected	Raw Intensity	Log2 Norm Intensity	Norm Imputed	Log2 Ctrl Norm
11	UDP-Glucose	Carbon	1	G6PDD_Untreated_1_2	G6PDD_Untreated_1	1.0	Polysaccharide biosynthesis	True	4.353e+06	0.9994	0.9994	1.105
158	4-Imidazole-Ac	Amino acid	1	G6PDD_Untreated_1_2	G6PDD_Untreated_1	1.0	Amino acid derivatives	True	9.146e+05	-1.252	-1.252	0.895
111	1-Me-Imidazole-Ac	Amino acid	1	G6PDD_Untreated_1_2	G6PDD_Untreated_1	1.0	Amino acids degradation intermediates	True	6.701e+05	-1.7	-1.7	0.2335
345	Guanine	Nucleotide	1	G6PDD_Untreated_1_2	G6PDD_Untreated_1	1.0	Purine bases	True	8.539e+07	5.293	5.293	0.3069
22	N-Ac-Neuraminate	Carbon	1	G6PDD_Untreated_1_2	G6PDD_Untreated_1	1.0	Aminosugar derivatives	True	1.834e+06	-0.2477	-0.2477	-0.1123
721	N'-Methylnicotinate	Cofactor	1	G6PDD_Untreated_1_2	G6PDD_Untreated_1	1.0	Derivatives of NA, nicotinamide and NAD	True	1.361e+06	-0.6781	-0.6781	0.3296
183	Phenol Sulfate	Amino acid	1	G6PDD_Untreated_1_2	G6PDD_Untreated_1	1.0	Amino acid derivatives	True	4.35e+04	-5.646	-5.646	-1.006
718	Nicotinamide Riboside	Cofactor	1	G6PDD_Untreated_1_2	G6PDD_Untreated_1	1.0	NAD biosynthesis	True	3.573e+06	0.7144	0.7144	-0.8757
297	gamma-Glu-Thr	Amino acid	1	G6PDD_Untreated_1_2	G6PDD_Untreated_1	1.0	Gamma-glutamyl dipeptides	True	3.875e+06	0.8314	0.8314	0.1912
295	gamma-Glu-Phe	Amino acid	1	G6PDD_Untreated_1_2	G6PDD_Untreated_1	0.75	Gamma-glutamyl dipeptides	True	4.321e+04	-5.655	-5.655	0.1298
347	Allantoic Acid	Nucleotide	1	G6PDD_Untreated_1_2	G6PDD_Untreated_1	0.5	Purine degradation	True	3.32e+04	-6.035	-6.035	-0.3959
399	Pseudouridine	Nucleotide	1	G6PDD_Untreated_1_2	G6PDD_Untreated_1	1.0	Pyrimidine derivatives in RNAs	True	4.053e+05	-2.426	-2.426	-0.3957
375	UTP	Nucleotide	1	G6PDD_Untreated_1_2	G6PDD_Untreated_1	1.0	Pyrimidine nucleotides	True	3.803e+05	-2.517	-2.517	-0.5415
144	Glu, gamma-Me Ester	Amino acid	1	G6PDD_Untreated_1_2	G6PDD_Untreated_1	1.0	Amino acid derivatives	True	4.353e+06	0.9993	0.9993	0.4215
292	gamma-Glu-epsilon-Lysine	Amino acid	1	G6PDD_Untreated_1_2	G6PDD_Untreated_1	1.0	Gamma-glutamyl dipeptides	True	1.079e+06	-1.012	-1.012	-0.8211
225	N-Ac-Thr	Amino acid	1	G6PDD_Untreated_1_2	G6PDD_Untreated_1	1.0	N-acetylated amino acids	True	7.453e+05	-1.547	-1.547	-0.04436
211	N-Ac-Asn	Amino acid	1	G6PDD_Untreated_1_2	G6PDD_Untreated_1	1.0	N-acetylated amino acids	True	4.19e+05	-2.378	-2.378	0.3075
151	Phenylacetylglycine	Amino acid	1	G6PDD_Untreated_1_2	G6PDD_Untreated_1	1.0	Amino acid derivatives	True	8.702e+05	-1.323	-1.323	-0.5364
217	N-Ac-His	Amino acid	1	G6PDD_Untreated_1_2	G6PDD_Untreated_1	1.0	N-acetylated amino acids	True	2.314e+05	-3.234	-3.234	0.2507
288	gamma-Glu-Gly	Amino acid	1	G6PDD_Untreated_1_2	G6PDD_Untreated_1	0.75	Gamma-glutamyl dipeptides	True	1.086e+06	-1.004	-1.004	0.8202

Metabolite ID	Name	Super Pathway	Dataset	Sample ID	Group ID	Detection Fraction	Pathway	Detected	Raw Intensity	Log2 Norm Intensity	Norm Imputed	Log2 Ctrl Norm
222	N-Ac-Phe	Amino acid	1	G6PDD_Untreated_1_2	G6PDD_Untreated_1	1.0	N-acetylated amino acids	True	3.414e+04	-5.995	-5.995	0.2775
71	Creatine-P	Carbon	1	G6PDD_Untreated_1_2	G6PDD_Untreated_1	1.0	Creatine energy storage	True	2.215e+05	-3.298	-3.298	0.9576
210	N-Ac-Arg	Amino acid	1	G6PDD_Untreated_1_2	G6PDD_Untreated_1	0.75	N-acetylated amino acids	True	4.723e+05	-2.205	-2.205	0.4707
218	N-Ac-Ile	Amino acid	1	G6PDD_Untreated_1_2	G6PDD_Untreated_1	0.5	N-acetylated amino acids	True	2.373e+04	-6.52	-6.52	-0.5541
251	Gly-Leu	Amino acid	1	G6PDD_Untreated_1_2	G6PDD_Untreated_1	1.0	Dipeptides	True	6.246e+06	1.52	1.52	-0.1185
290	gamma-Glu-Ile	Amino acid	1	G6PDD_Untreated_1_2	G6PDD_Untreated_1	1.0	Gamma-glutamyl dipeptides	True	8.713e+05	-1.322	-1.322	0.01702
316	Ophthalmate	Amino acid	1	G6PDD_Untreated_1_2	G6PDD_Untreated_1	1.0	Oxidative stress markers	True	7.983e+06	1.874	1.874	-0.4684
125	Isovaleryl-Gly	Amino acid	1	G6PDD_Untreated_1_2	G6PDD_Untreated_1	1.0	Amino acids degradation intermediates	True	3.969e+04	-5.778	-5.778	-0.3684
368	7-Me-Guanine	Nucleotide	1	G6PDD_Untreated_1_2	G6PDD_Untreated_1	1.0	Purine derivatives in RNAs	True	4.837e+05	-2.17	-2.17	0.01954
208	Pro-OH-Pro	Amino acid	1	G6PDD_Untreated_1_2	G6PDD_Untreated_1	1.0	Amino acid derivatives	True	2.093e+07	3.265	3.265	-0.06469
366	N2,N2-DiMe-Guanosine	Nucleotide	1	G6PDD_Untreated_1_2	G6PDD_Untreated_1	1.0	Purine derivatives in RNAs	True	6.761e+04	-5.009	-5.009	-0.8957
352	3'-AMP	Nucleotide	1	G6PDD_Untreated_1_2	G6PDD_Untreated_1	1.0	Purine derivatives in signaling	True	8.239e+05	-1.402	-1.402	0.08446
363	N6-Carbamoyl-Thr-Adenosine	Nucleotide	1	G6PDD_Untreated_1_2	G6PDD_Untreated_1	1.0	Purine derivatives in RNAs	True	1.521e+05	-3.84	-3.84	0.8457
314	Cys-Glutathione Disulfide	Amino acid	1	G6PDD_Untreated_1_2	G6PDD_Untreated_1	0.25	Oxidative stress markers	True	5.588e+04	-5.284	-5.284	-2
382	Orotidine	Nucleotide	1	G6PDD_Untreated_1_2	G6PDD_Untreated_1	1.0	Pyrimidine (UMP) biosynthesis	True	1.67e+05	-3.705	-3.705	0.5866
307	Cys-Gly	Amino acid	1	G6PDD_Untreated_1_2	G6PDD_Untreated_1	1.0	Glutathione biosynthesis	True	1.05e+07	2.269	2.269	0.662
64	Sedoheptulose-7-P	Carbon	1	G6PDD_Untreated_1_2	G6PDD_Untreated_1	1.0	Pentose phosphate pathway (PPP)	True	2.809e+06	0.367	0.367	0.2129
142	N-Ac-Asp-Glu	Amino acid	1	G6PDD_Untreated_1_2	G6PDD_Untreated_1	1.0	Amino acid derivatives	True	3.206e+06	0.5581	0.5581	1.703
708	Thiamin-PP	Cofactor	1	G6PDD_Untreated_1_2	G6PDD_Untreated_1	1.0	Cofactors	True	4.122e+04	-5.723	-5.723	0.2605
182	P-Cresol Sulfate	Amino acid	1	G6PDD_Untreated_1_2	G6PDD_Untreated_1	1.0	Amino acid derivatives	True	1.793e+05	-3.602	-3.602	-0.4061

Metabolite ID	Name	Super Pathway	Dataset	Sample ID	Group ID	Detection Fraction	Pathway	Detected	Raw Intensity	Log2 Norm Intensity	Norm Imputed	Log2 Ctrl Norm
250	Gly-Ile	Amino acid	1	G6PDD_Untreated_1_2	G6PDD_Untreated_1	1.0	Dipeptides	True	1.386e+06	-0.6515	-0.6515	0.6657
286	gamma-Glu-Glu	Amino acid	1	G6PDD_Untreated_1_2	G6PDD_Untreated_1	1.0	Gamma-glutamyl dipeptides	True	4.938e+06	1.181	1.181	1.163
739	Deoxycarnitine	Cofactor	1	G6PDD_Untreated_1_2	G6PDD_Untreated_1	1.0	Carnitine biosynthesis	True	4.656e+07	4.418	4.418	0.667
203	DiMe-Arg	Amino acid	1	G6PDD_Untreated_1_2	G6PDD_Untreated_1	1.0	Amino acid derivatives	True	7.392e+07	5.085	5.085	-0.5474
351	2'-AMP	Nucleotide	1	G6PDD_Untreated_1_2	G6PDD_Untreated_1	1.0	Purine derivatives in signaling	True	1.346e+05	-4.016	-4.016	-1.748
8	Cytidine 5'-P-N-Ac-Neuraminate	Carbon	1	G6PDD_Untreated_1_2	G6PDD_Untreated_1	1.0	Aminosugar biosynthesis	True	6.378e+05	-1.772	-1.772	-0.02616
285	gamma-Glu-Ala	Amino acid	1	G6PDD_Untreated_1_2	G6PDD_Untreated_1	1.0	Gamma-glutamyl dipeptides	True	5.014e+05	-2.119	-2.119	1.12
224	N-Ac-Ser	Amino acid	1	G6PDD_Untreated_1_2	G6PDD_Untreated_1	1.0	N-acetylated amino acids	True	8.768e+06	2.01	2.01	0.1303
244	Ala-Leu	Amino acid	1	G6PDD_Untreated_1_2	G6PDD_Untreated_1	1.0	Dipeptides	True	5.82e+06	1.418	1.418	0.2803
207	N-Me-Pro	Amino acid	1	G6PDD_Untreated_1_2	G6PDD_Untreated_1	1.0	Amino acid derivatives	True	1.168e+06	-0.8984	-0.8984	0.03162
171	Cys Sulfinic Acid	Amino acid	1	G6PDD_Untreated_1_2	G6PDD_Untreated_1	0.75	Amino acid derivatives	True	1.749e+05	-3.638	-3.638	-0.3406
181	O-Me-Tyr	Amino acid	1	G6PDD_Untreated_1_2	G6PDD_Untreated_1	1.0	Amino acid derivatives	True	2.172e+05	-3.326	-3.326	0.04405
240	N-Ac-Putrescine	Amino acid	1	G6PDD_Untreated_1_2	G6PDD_Untreated_1	1.0	Polyamine derivatives	True	4.573e+05	-2.252	-2.252	0.1122
176	S-Me-Met	Amino acid	1	G6PDD_Untreated_1_2	G6PDD_Untreated_1	1.0	Amino acid derivatives	True	6.259e+05	-1.799	-1.799	0.043
339	AICAR	Nucleotide	1	G6PDD_Untreated_1_2	G6PDD_Untreated_1	1.0	IMP biosynthesis	True	4.508e+05	-2.272	-2.272	2.029
141	gamma-Carboxy-Glu	Amino acid	1	G6PDD_Untreated_1_2	G6PDD_Untreated_1	1.0	Amino acid derivatives	True	1.188e+06	-0.874	-0.874	-0.08899
392	3'-UMP	Nucleotide	1	G6PDD_Untreated_1_2	G6PDD_Untreated_1	0.75	Pyrimidine derivatives in signaling	True	1.551e+05	-3.812	-3.812	0.2216
355	3'-GMP	Nucleotide	1	G6PDD_Untreated_1_2	G6PDD_Untreated_1	1.0	Purine derivatives in signaling	True	2.264e+05	-3.266	-3.266	0.6717
282	Val-Leu	Amino acid	1	G6PDD_Untreated_1_2	G6PDD_Untreated_1	1.0	Dipeptides	True	2.913e+06	0.4196	0.4196	-0.5521
140	Carboxyethyl-GABA	Amino acid	1	G6PDD_Untreated_1_2	G6PDD_Untreated_1	1.0	Amino acid derivatives	True	8.446e+05	-1.367	-1.367	0.0646
258	Ile-Gly	Amino acid	1	G6PDD_Untreated_1_2	G6PDD_Untreated_1	1.0	Dipeptides	True	2.282e+07	3.389	3.389	0.0774

Metabolite ID	Name	Super Pathway	Dataset	Sample ID	Group ID	Detection Fraction	Pathway	Detected	Raw Intensity	Log2 Norm Intensity	Norm Imputed	Log2 Ctrl Norm
260	Leu-Ala	Amino acid	1	G6PDD_Untreated_1_2	G6PDD_Untreated_1	1.0	Dipeptides	True	5.894e+06	1.436	1.436	0.1352
265	Lys-Leu	Amino acid	1	G6PDD_Untreated_1_2	G6PDD_Untreated_1	1.0	Dipeptides	True	4.67e+05	-2.221	-2.221	0.39
263	Leu-Gly	Amino acid	1	G6PDD_Untreated_1_2	G6PDD_Untreated_1	1.0	Dipeptides	True	4.536e+07	4.381	4.381	1.359
281	Val-Gly	Amino acid	1	G6PDD_Untreated_1_2	G6PDD_Untreated_1	1.0	Dipeptides	True	4.723e+07	4.439	4.439	-0.08018
270	Pro-Gly	Amino acid	1	G6PDD_Untreated_1_2	G6PDD_Untreated_1	1.0	Dipeptides	True	4.949e+06	1.184	1.184	0.4375
114	Imidazole Propionate	Amino acid	1	G6PDD_Untreated_1_2	G6PDD_Untreated_1	1.0	Amino acids degradation intermediates	True	1.047e+06	-1.057	-1.057	1.254
267	Phe-Gly	Amino acid	1	G6PDD_Untreated_1_2	G6PDD_Untreated_1	1.0	Dipeptides	True	3.998e+07	4.199	4.199	0.1047
266	Phe-Ala	Amino acid	1	G6PDD_Untreated_1_2	G6PDD_Untreated_1	1.0	Dipeptides	True	5.578e+06	1.357	1.357	0.2802
278	Tyr-Gly	Amino acid	1	G6PDD_Untreated_1_2	G6PDD_Untreated_1	1.0	Dipeptides	True	1.043e+07	2.26	2.26	0.07773
255	His-Ala	Amino acid	1	G6PDD_Untreated_1_2	G6PDD_Untreated_1	1.0	Dipeptides	True	1.05e+06	-1.052	-1.052	0.2852
280	Val-Gln	Amino acid	1	G6PDD_Untreated_1_2	G6PDD_Untreated_1	1.0	Dipeptides	True	7.254e+06	1.736	1.736	0.118
143	S-1-Pyrroline-5-Carboxylate	Amino acid	1	G6PDD_Untreated_1_2	G6PDD_Untreated_1	1.0	Amino acid derivatives	True	4.995e+05	-2.124	-2.124	-0.05623
232	SAH	Amino acid	1	G6PDD_Untreated_1_2	G6PDD_Untreated_1	1.0	SAM metabolism	True	1.504e+06	-0.5343	-0.5343	-0.04888
20	Erythronate	Carbon	1	G6PDD_Untreated_1_2	G6PDD_Untreated_1	1.0	Aminosugar derivatives	True	3.36e+07	3.948	3.948	0.2065
248	Gln-Leu	Amino acid	1	G6PDD_Untreated_1_2	G6PDD_Untreated_1	1.0	Dipeptides	True	1.696e+06	-0.3605	-0.3605	0.5447
276	Trp-Gly	Amino acid	1	G6PDD_Untreated_1_2	G6PDD_Untreated_1	1.0	Dipeptides	True	7.014e+05	-1.635	-1.635	-0.5562
205	N-delta-Ac-Ornithine	Amino acid	1	G6PDD_Untreated_1_2	G6PDD_Untreated_1	1.0	Amino acid derivatives	True	1.114e+06	-0.967	-0.967	0.154
163	Formimino-Glu	Amino acid	1	G6PDD_Untreated_1_2	G6PDD_Untreated_1	1.0	Amino acid derivatives	True	1.285e+06	-0.7615	-0.7615	0.4783
204	N-Me-Arg	Amino acid	1	G6PDD_Untreated_1_2	G6PDD_Untreated_1	1.0	Amino acid derivatives	True	1.83e+07	3.071	3.071	0.4841
242	Guanidino-Ac	Amino acid	1	G6PDD_Untreated_1_2	G6PDD_Untreated_1	0.75	Creatine biosynthesis	True	8.469e+04	-4.685	-4.685	-0.8041

Metabolite ID	Name	Super Pathway	Dataset	Sample ID	Group ID	Detection Fraction	Pathway	Detected	Raw Intensity	Log2 Norm Intensity	Norm Imputed	Log2 Ctrl Norm
300	gamma-Glu-Val	Amino acid	1	G6PDD_Untreated_1_2	G6PDD_Untreated_1	0.75	Gamma-glutamyl dipeptides	True	3.754e+06	0.7857	0.7857	0.1649
53	Ac-CoA	Carbon	1	G6PDD_Untreated_1_2	G6PDD_Untreated_1	0.5	Acetyl-CoA	False			-7.274	-0.5114
18	Maltotriose	Carbon	1	G6PDD_Untreated_1_2	G6PDD_Untreated_1	1.0	Glycogen degradation	True	3.455e+06	0.6657	0.6657	0.8235
294	gamma-Glu-Met	Amino acid	1	G6PDD_Untreated_1_2	G6PDD_Untreated_1	0.5	Gamma-glutamyl dipeptides	True	2.283e+05	-3.254	-3.254	0.2282
174	Met Sulfone	Amino acid	1	G6PDD_Untreated_1_2	G6PDD_Untreated_1	1.0	Amino acid derivatives	True	4.133e+05	-2.398	-2.398	0.6811
175	N-Ac-Met Sulfoxide	Amino acid	1	G6PDD_Untreated_1_2	G6PDD_Untreated_1	1.0	Amino acid derivatives	True	1.615e+06	-0.4316	-0.4316	-0.4079
25	Mannitol/Sorbitol	Carbon	1	G6PDD_Untreated_1_2	G6PDD_Untreated_1	1.0	Sugars and sugar alcohols	True	9.854e+06	2.178	2.178	-0.04665
6	UDP-GlcNAc	Carbon	1	G6PDD_Untreated_1_2	G6PDD_Untreated_1	0.5	Aminosugar biosynthesis	False			-3.589	-0.4814
145	Pyro-Gln	Amino acid	1	G6PDD_Untreated_1_2	G6PDD_Untreated_1	1.0	Amino acid derivatives	True	4.769e+06	1.131	1.131	0.291
705	Coenzyme A	Cofactor	1	G6PDD_Untreated_1_2	G6PDD_Untreated_1	1.0	Cofactors	True	9.361e+04	-4.54	-4.54	-0.581
319	2'-dAMP	Nucleotide	1	G6PDD_Untreated_1_2	G6PDD_Untreated_1	0.75	Deoxy-nucleotides	True	1.077e+05	-4.337	-4.337	-0.6568
119	alpha-OH-Isovalerate	Amino acid	1	G6PDD_Untreated_1_2	G6PDD_Untreated_1	0.5	Amino acids degradation intermediates	True	5.537e+04	-5.298	-5.298	-1.999
46	Fructose 1,6-PP / Glucose 1,6-PP / Inositol-1,4-PP / Inositol-1,3-PP	Carbon	1	G6PDD_Untreated_1_2	G6PDD_Untreated_1	1.0	Glycolysis, GNG	True	5.596e+07	4.684	4.684	-0.1475
137	1-Me-Guanidine	Amino acid	1	G6PDD_Untreated_1_2	G6PDD_Untreated_1	1.0	Amino acids degradation intermediates	True	3.542e+04	-5.942	-5.942	-0.7895
23	N-GlcNAc-Asn	Carbon	1	G6PDD_Untreated_1_2	G6PDD_Untreated_1	1.0	Aminosugar derivatives	True	5.568e+06	1.354	1.354	0.6414
262	Leu-Gln	Amino acid	1	G6PDD_Untreated_1_2	G6PDD_Untreated_1	1.0	Dipeptides	True	5.505e+06	1.338	1.338	0.04159
24	Fructose	Carbon	1	G6PDD_Untreated_1_2	G6PDD_Untreated_1	1.0	Sugars and sugar alcohols	True	6.783e+06	1.639	1.639	-0.3096
197	C-Glycosyl-Trp	Amino acid	1	G6PDD_Untreated_1_2	G6PDD_Untreated_1	1.0	Amino acid derivatives	True	2.213e+06	0.02311	0.02311	0.3221
33	Arabitol/Xylitol	Carbon	1	G6PDD_Untreated_1_2	G6PDD_Untreated_1	1.0	Sugars and sugar alcohols	True	4.043e+05	-2.429	-2.429	-0.5694

Metabolite ID	Name	Super Pathway	Dataset	Sample ID	Group ID	Detection Fraction	Pathway	Detected	Raw Intensity	Log2 Norm Intensity	Norm Imputed	Log2 Ctrl Norm
128	N2-Ac-Lys/N6-Ac-Lys	Amino acid	1	G6PDD_Untreated_1_2	G6PDD_Untreated_1	1.0	Amino acids degradation intermediates	True	4.429e+06	1.024	1.024	-0.1863
42	2-Me-Citrate	Carbon	1	G6PDD_Untreated_1_2	G6PDD_Untreated_1	1.0	Propionate metabolism	True	9.671e+04	-4.493	-4.493	-0.11
12	Glucuronate 1-P	Carbon	1	G6PDD_Untreated_1_2	G6PDD_Untreated_1	1.0	Polysaccharide biosynthesis	True	8.035e+05	-1.438	-1.438	-0.1956
76	Gln	Amino acid	1	G6PDD_Untreated_1_3	G6PDD_Untreated_1	1.0	Proteinogenic amino acids	True	8.214e+08	8.657	8.657	-0.02352
89	Trp	Amino acid	1	G6PDD_Untreated_1_3	G6PDD_Untreated_1	1.0	Proteinogenic amino acids	True	8.452e+07	5.376	5.376	-0.2993
723	beta-Ala	Cofactor	1	G6PDD_Untreated_1_3	G6PDD_Untreated_1	1.0	Coenzyme A biosynthesis	True	2.154e+07	3.404	3.404	0.3951
75	Glu	Amino acid	1	G6PDD_Untreated_1_3	G6PDD_Untreated_1	1.0	Proteinogenic amino acids	True	5.532e+09	11.41	11.41	0.672
77	Gly	Amino acid	1	G6PDD_Untreated_1_3	G6PDD_Untreated_1	1.0	Proteinogenic amino acids	True	9.407e+07	5.531	5.531	-0.0844
80	His	Amino acid	1	G6PDD_Untreated_1_3	G6PDD_Untreated_1	1.0	Proteinogenic amino acids	True	1.535e+07	2.915	2.915	-0.5001
82	Leu	Amino acid	1	G6PDD_Untreated_1_3	G6PDD_Untreated_1	1.0	Proteinogenic amino acids	True	7.547e+08	8.535	8.535	-0.6864
87	Phe	Amino acid	1	G6PDD_Untreated_1_3	G6PDD_Untreated_1	1.0	Proteinogenic amino acids	True	6.334e+08	8.282	8.282	-0.5294
130	Glutarate	Amino acid	1	G6PDD_Untreated_1_3	G6PDD_Untreated_1	1.0	Amino acids degradation intermediates	True	3.77e+05	-2.432	-2.432	-5.45e-03
196	5-OH-Indole-Ac	Amino acid	1	G6PDD_Untreated_1_3	G6PDD_Untreated_1	0.75	Amino acid derivatives	True	1.543e+05	-3.721	-3.721	0.2904
74	Asp	Amino acid	1	G6PDD_Untreated_1_3	G6PDD_Untreated_1	1.0	Proteinogenic amino acids	True	5.536e+08	8.088	8.088	0.1962
236	Spermidine	Amino acid	1	G6PDD_Untreated_1_3	G6PDD_Untreated_1	1.0	Polyamines	True	6.639e+07	5.028	5.028	1.167
73	Asn	Amino acid	1	G6PDD_Untreated_1_3	G6PDD_Untreated_1	1.0	Proteinogenic amino acids	True	1.823e+08	6.485	6.485	-0.1859
243	Creatinine	Amino acid	1	G6PDD_Untreated_1_3	G6PDD_Untreated_1	1.0	Creatine degradation	True	8.172e+07	5.328	5.328	0.3256
376	Cytidine	Nucleotide	1	G6PDD_Untreated_1_3	G6PDD_Untreated_1	0.75	Pyrimidine nucleosides	False			-1.507	-2.235
41	Lactate	Carbon	1	G6PDD_Untreated_1_3	G6PDD_Untreated_1	1.0	Respiratory carbon sources	True	2.372e+08	6.865	6.865	0.3836
58	alpha-Ketoglutarate	Carbon	1	G6PDD_Untreated_1_3	G6PDD_Untreated_1	1.0	TCA cycle	True	3.828e+06	0.9118	0.9118	0.8951

Metabolite ID	Name	Super Pathway	Dataset	Sample ID	Group ID	Detection Fraction	Pathway	Detected	Raw Intensity	Log2 Norm Intensity	Norm Imputed	Log2 Ctrl Norm
69	3-OH-Butyrate	Carbon	1	G6PDD_Untreated_1_3	G6PDD_Untreated_1	0.75	Ketone bodies	True	2.861e+05	-2.83	-2.83	0.1539
343	Adenine	Nucleotide	1	G6PDD_Untreated_1_3	G6PDD_Untreated_1	1.0	Purine bases	True	9.892e+06	2.281	2.281	0.8245
336	Adenosine	Nucleotide	1	G6PDD_Untreated_1_3	G6PDD_Untreated_1	1.0	Purine nucleosides	True	1.417e+07	2.8	2.8	-0.6226
722	ADP-Ribose	Cofactor	1	G6PDD_Untreated_1_3	G6PDD_Untreated_1	1.0	Derivatives of NA, nicotinamide and NAD	True	7.11e+05	-1.517	-1.517	1.485
383	Cytosine	Nucleotide	1	G6PDD_Untreated_1_3	G6PDD_Untreated_1	0.75	Pyrimidine bases	True	2.055e+05	-3.308	-3.308	1.547
3	Glucosamine 6-P	Carbon	1	G6PDD_Untreated_1_3	G6PDD_Untreated_1	1.0	Aminosugar biosynthesis	True	1.673e+06	-0.2822	-0.2822	1.385
717	Nicotinamide	Cofactor	1	G6PDD_Untreated_1_3	G6PDD_Untreated_1	1.0	NAD biosynthesis	True	3.539e+07	4.12	4.12	-0.3949
51	PEP	Carbon	1	G6PDD_Untreated_1_3	G6PDD_Untreated_1	1.0	Glycolysis, GNG	True	3.005e+06	0.5626	0.5626	-0.4886
237	Spermine	Amino acid	1	G6PDD_Untreated_1_3	G6PDD_Untreated_1	1.0	Polyamines	True	4.15e+05	-2.294	-2.294	-0.803
385	Uracil	Nucleotide	1	G6PDD_Untreated_1_3	G6PDD_Untreated_1	1.0	Pyrimidine bases	True	3.373e+05	-2.593	-2.593	-2.858
377	Uridine	Nucleotide	1	G6PDD_Untreated_1_3	G6PDD_Untreated_1	1.0	Pyrimidine nucleosides	True	1.019e+07	2.325	2.325	-1.724
348	Allantoin	Nucleotide	1	G6PDD_Untreated_1_3	G6PDD_Untreated_1	1.0	Purine degradation	True	2.154e+06	0.0819	0.0819	0.2575
335	Inosine	Nucleotide	1	G6PDD_Untreated_1_3	G6PDD_Untreated_1	1.0	Purine nucleosides	True	3.199e+07	3.975	3.975	-1.524
81	Ile	Amino acid	1	G6PDD_Untreated_1_3	G6PDD_Untreated_1	1.0	Proteinogenic amino acids	True	7.529e+08	8.532	8.532	-0.1836
72	Ala	Amino acid	1	G6PDD_Untreated_1_3	G6PDD_Untreated_1	1.0	Proteinogenic amino acids	True	7.718e+08	8.567	8.567	0.06581
79	Thr	Amino acid	1	G6PDD_Untreated_1_3	G6PDD_Untreated_1	1.0	Proteinogenic amino acids	True	3.538e+08	7.442	7.442	-0.5674
88	Tyr	Amino acid	1	G6PDD_Untreated_1_3	G6PDD_Untreated_1	1.0	Proteinogenic amino acids	True	2.365e+08	6.861	6.861	-0.6626
84	Lys	Amino acid	1	G6PDD_Untreated_1_3	G6PDD_Untreated_1	1.0	Proteinogenic amino acids	True	3.294e+08	7.339	7.339	-0.651
86	Met	Amino acid	1	G6PDD_Untreated_1_3	G6PDD_Untreated_1	1.0	Proteinogenic amino acids	True	3.303e+08	7.343	7.343	-0.8849
61	Malate	Carbon	1	G6PDD_Untreated_1_3	G6PDD_Untreated_1	1.0	TCA cycle	True	3.243e+08	7.316	7.316	0.1445

Metabolite ID	Name	Super Pathway	Dataset	Sample ID	Group ID	Detection Fraction	Pathway	Detected	Raw Intensity	Log2 Norm Intensity	Norm Imputed	Log2 Ctrl Norm
235	Putrescine	Amino acid	1	G6PDD_Untreated_1_3	G6PDD_Untreated_1	1.0	Polyamines	True	6.556e+06	1.688	1.688	2.179
324	2'-dU	Nucleotide	1	G6PDD_Untreated_1_3	G6PDD_Untreated_1	0.5	Deoxy-nucleosides	False			-5.281	-0.9009
49	3-P-Glycerate	Carbon	1	G6PDD_Untreated_1_3	G6PDD_Untreated_1	1.0	Glycolysis, GNG	True	2.93e+07	3.848	3.848	0.5824
189	Kynurenate	Amino acid	1	G6PDD_Untreated_1_3	G6PDD_Untreated_1	0.75	Amino acid derivatives	True	2.717e+04	-6.227	-6.227	-0.4287
234	5-Me-Thioadenosine	Amino acid	1	G6PDD_Untreated_1_3	G6PDD_Untreated_1	1.0	SAM metabolism	True	1.374e+07	2.755	2.755	0.8699
59	Succinate	Carbon	1	G6PDD_Untreated_1_3	G6PDD_Untreated_1	1.0	TCA cycle	True	8.334e+06	2.034	2.034	1.733
36	Ribose	Carbon	1	G6PDD_Untreated_1_3	G6PDD_Untreated_1	1.0	Sugars and sugar alcohols	True	5.779e+05	-1.816	-1.816	-0.5825
133	Ornithine	Amino acid	1	G6PDD_Untreated_1_3	G6PDD_Untreated_1	1.0	Amino acids degradation intermediates	True	9.932e+07	5.609	5.609	1.13
313	5-Oxoproline	Amino acid	1	G6PDD_Untreated_1_3	G6PDD_Untreated_1	1.0	Glutathione derivatives	True	9.333e+06	2.197	2.197	0.11
165	N-6-Tri-Me-Lys	Amino acid	1	G6PDD_Untreated_1_3	G6PDD_Untreated_1	1.0	Amino acid derivatives	True	1.588e+07	2.964	2.964	-0.7993
380	Orotate	Nucleotide	1	G6PDD_Untreated_1_3	G6PDD_Untreated_1	1.0	Pyrimidine (UMP) biosynthesis	True	1.957e+05	-3.378	-3.378	0.2521
724	Pantothenate	Cofactor	1	G6PDD_Untreated_1_3	G6PDD_Untreated_1	1.0	Coenzyme A biosynthesis	True	2.867e+08	7.138	7.138	0.8281
150	N-Me-Gly	Amino acid	1	G6PDD_Untreated_1_3	G6PDD_Untreated_1	1.0	Amino acid derivatives	True	2.403e+06	0.2401	0.2401	0.5405
122	3-OH-Isobutyrate	Amino acid	1	G6PDD_Untreated_1_3	G6PDD_Untreated_1	0.5	Amino acids degradation intermediates	True	2.627e+05	-2.953	-2.953	0.2028
241	4-Acetamidobutanoate	Amino acid	1	G6PDD_Untreated_1_3	G6PDD_Untreated_1	1.0	Polyamine derivatives	True	4.141e+06	1.025	1.025	0.5842
711	alpha-Tocopherol	Cofactor	1	G6PDD_Untreated_1_3	G6PDD_Untreated_1	1.0	Cofactors	True	5.954e+06	1.549	1.549	0.7687
55	Citrate	Carbon	1	G6PDD_Untreated_1_3	G6PDD_Untreated_1	1.0	TCA cycle	True	9.122e+06	2.165	2.165	0.2155
387	3-Aminoisobutyrate	Nucleotide	1	G6PDD_Untreated_1_3	G6PDD_Untreated_1	1.0	Pyrimidine degradation	True	6.027e+05	-1.755	-1.755	0.2584
338	Guanosine	Nucleotide	1	G6PDD_Untreated_1_3	G6PDD_Untreated_1	1.0	Purine nucleosides	True	1.607e+07	2.982	2.982	-2.124
209	N-Ac-Ala	Amino acid	1	G6PDD_Untreated_1_3	G6PDD_Untreated_1	1.0	N-acetylated amino acids	True	5.921e+05	-1.781	-1.781	-0.5796

Metabolite ID	Name	Super Pathway	Dataset	Sample ID	Group ID	Detection Fraction	Pathway	Detected	Raw Intensity	Log2 Norm Intensity	Norm Imputed	Log2 Ctrl Norm
221	N-Ac-Met	Amino acid	1	G6PDD_Untreated_1_3	G6PDD_Untreated_1	1.0	N-acetylated amino acids	True	4.053e+06	0.9941	0.9941	-0.7501
228	N-Ac-Val	Amino acid	1	G6PDD_Untreated_1_3	G6PDD_Untreated_1	1.0	N-acetylated amino acids	True	4.324e+04	-5.556	-5.556	-0.1856
346	Urate	Nucleotide	1	G6PDD_Untreated_1_3	G6PDD_Untreated_1	1.0	Purine degradation	True	9.567e+05	-1.089	-1.089	-0.02588
90	Arg	Amino acid	1	G6PDD_Untreated_1_3	G6PDD_Untreated_1	1.0	Proteinogenic amino acids	True	1.008e+09	8.953	8.953	-0.7525
60	Fumarate	Carbon	1	G6PDD_Untreated_1_3	G6PDD_Untreated_1	1.0	TCA cycle	True	3.95e+06	0.9571	0.9571	-0.05813
78	Ser	Amino acid	1	G6PDD_Untreated_1_3	G6PDD_Untreated_1	1.0	Proteinogenic amino acids	True	1.4e+08	6.105	6.105	-1.153
83	Val	Amino acid	1	G6PDD_Untreated_1_3	G6PDD_Untreated_1	1.0	Proteinogenic amino acids	True	2.796e+08	7.102	7.102	-0.7698
734	Pyridoxal	Cofactor	1	G6PDD_Untreated_1_3	G6PDD_Untreated_1	1.0	PLP biosynthesis and salvage	True	5.183e+06	1.349	1.349	-0.434
136	Urea	Amino acid	1	G6PDD_Untreated_1_3	G6PDD_Untreated_1	1.0	Amino acids degradation intermediates	True	1.9e+06	-0.09911	-0.09911	-0.2503
67	Ribose 1-P	Carbon	1	G6PDD_Untreated_1_3	G6PDD_Untreated_1	1.0	Pentose phosphate pathway (PPP)	True	3.959e+06	0.9604	0.9604	0.3392
284	Carnosine	Amino acid	1	G6PDD_Untreated_1_3	G6PDD_Untreated_1	1.0	Dipeptides	True	5.204e+05	-1.967	-1.967	0.1411
306	gamma-Glu-Cys	Amino acid	1	G6PDD_Untreated_1_3	G6PDD_Untreated_1	1.0	Glutathione biosynthesis	True	1.648e+06	-0.3045	-0.3045	0.03231
712	Retinol (Vit A)	Cofactor	1	G6PDD_Untreated_1_3	G6PDD_Untreated_1	1.0	Cofactors	True	3.755e+05	-2.438	-2.438	-4.88e-03
85	Cys	Amino acid	1	G6PDD_Untreated_1_3	G6PDD_Untreated_1	1.0	Proteinogenic amino acids	True	3.059e+07	3.91	3.91	-0.6038
91	Pro	Amino acid	1	G6PDD_Untreated_1_3	G6PDD_Untreated_1	1.0	Proteinogenic amino acids	True	1.422e+09	9.449	9.449	0.04111
308	Glutathione, Reduced	Amino acid	1	G6PDD_Untreated_1_3	G6PDD_Untreated_1	1.0	Glutathione	True	3.772e+08	7.534	7.534	0.5781
107	Citrulline	Amino acid	1	G6PDD_Untreated_1_3	G6PDD_Untreated_1	1.0	Amino acids biosynthesis intermediates	True	2.012e+07	3.306	3.306	0.4813
328	IMP	Nucleotide	1	G6PDD_Untreated_1_3	G6PDD_Untreated_1	0.5	Purine nucleotides	True	3.183e+05	-2.676	-2.676	1.645
706	FAD	Cofactor	1	G6PDD_Untreated_1_3	G6PDD_Untreated_1	1.0	Cofactors	True	5.228e+05	-1.96	-1.96	0.2291
735	Pyridoxamine	Cofactor	1	G6PDD_Untreated_1_3	G6PDD_Untreated_1	1.0	PLP biosynthesis and salvage	True	2.374e+06	0.2224	0.2224	0.4154

Metabolite ID	Name	Super Pathway	Dataset	Sample ID	Group ID	Detection Fraction	Pathway	Detected	Raw Intensity	Log2 Norm Intensity	Norm Imputed	Log2 Ctrl Norm
199	Serotonin	Amino acid	1	G6PDD_Untreated_1_3	G6PDD_Untreated_1	1.0	Amino acid derivatives	True	4.781e+06	1.233	1.233	-0.04019
370	CMP	Nucleotide	1	G6PDD_Untreated_1_3	G6PDD_Untreated_1	1.0	Pyrimidine nucleotides	True	9.801e+06	2.268	2.268	0.4291
287	gamma-Glu-Gln	Amino acid	1	G6PDD_Untreated_1_3	G6PDD_Untreated_1	1.0	Gamma-glutamyl dipeptides	True	1.897e+06	-0.1007	-0.1007	-0.9417
14	UDP-Glucuronate	Carbon	1	G6PDD_Untreated_1_3	G6PDD_Untreated_1	1.0	Polysaccharide biosynthesis	True	2.888e+06	0.5052	0.5052	2.454
229	N-Formyl-Met	Amino acid	1	G6PDD_Untreated_1_3	G6PDD_Untreated_1	1.0	N-formylated amino acids	True	1.559e+05	-3.706	-3.706	0.1158
350	3',5'-cAMP	Nucleotide	1	G6PDD_Untreated_1_3	G6PDD_Untreated_1	1.0	Purine derivatives in signaling	True	1.029e+05	-4.306	-4.306	-0.6865
371	CDP	Nucleotide	1	G6PDD_Untreated_1_3	G6PDD_Untreated_1	1.0	Pyrimidine nucleotides	True	7.779e+05	-1.387	-1.387	1.933
372	CTP	Nucleotide	1	G6PDD_Untreated_1_3	G6PDD_Untreated_1	0.75	Pyrimidine nucleotides	True	2.872e+05	-2.825	-2.825	0.8289
333	GDP	Nucleotide	1	G6PDD_Untreated_1_3	G6PDD_Untreated_1	0.5	Purine nucleotides	True	1.105e+06	-0.8803	-0.8803	1.62
332	GMP	Nucleotide	1	G6PDD_Untreated_1_3	G6PDD_Untreated_1	1.0	Purine nucleotides	True	2.236e+07	3.458	3.458	2.76
373	UMP	Nucleotide	1	G6PDD_Untreated_1_3	G6PDD_Untreated_1	1.0	Pyrimidine nucleotides	True	6.914e+06	1.765	1.765	2.82
389	3'-CMP	Nucleotide	1	G6PDD_Untreated_1_3	G6PDD_Untreated_1	1.0	Pyrimidine derivatives in signaling	True	3.382e+06	0.733	0.733	1.784
330	ADP	Nucleotide	1	G6PDD_Untreated_1_3	G6PDD_Untreated_1	1.0	Purine nucleotides	True	1.318e+07	2.695	2.695	2.992
342	Hypoxanthine	Nucleotide	1	G6PDD_Untreated_1_3	G6PDD_Untreated_1	1.0	Purine bases	True	8.886e+06	2.127	2.127	-1.813
736	Pyridoxamine-P	Cofactor	1	G6PDD_Untreated_1_3	G6PDD_Untreated_1	1.0	PLP biosynthesis and salvage	True	3.991e+05	-2.35	-2.35	0.6282
148	Betaine	Amino acid	1	G6PDD_Untreated_1_3	G6PDD_Untreated_1	1.0	Amino acid derivatives	True	7.532e+07	5.21	5.21	0.4044
344	Xanthine	Nucleotide	1	G6PDD_Untreated_1_3	G6PDD_Untreated_1	1.0	Purine bases	True	3.037e+06	0.5776	0.5776	-0.33
386	3-Ureidopropionate	Nucleotide	1	G6PDD_Untreated_1_3	G6PDD_Untreated_1	1.0	Pyrimidine degradation	True	1.317e+06	-0.6275	-0.6275	0.2918
149	DiMe-Gly	Amino acid	1	G6PDD_Untreated_1_3	G6PDD_Untreated_1	1.0	Amino acid derivatives	True	1.651e+06	-0.3018	-0.3018	0.2185
703	NAD+	Cofactor	1	G6PDD_Untreated_1_3	G6PDD_Untreated_1	1.0	Cofactors	True	1.6e+07	2.975	2.975	0.2391
709	Pyridoxal-P	Cofactor	1	G6PDD_Untreated_1_3	G6PDD_Untreated_1	1.0	Cofactors	True	5.546e+05	-1.875	-1.875	0.4326

Metabolite ID	Name	Super Pathway	Dataset	Sample ID	Group ID	Detection Fraction	Pathway	Detected	Raw Intensity	Log2 Norm Intensity	Norm Imputed	Log2 Ctrl Norm
731	Thiamin (Vitamin B1)	Cofactor	1	G6PDD_Untreated_1_3	G6PDD_Untreated_1	1.0	TPP biosynthesis	True	4.94e+06	1.28	1.28	0.5321
374	UDP	Nucleotide	1	G6PDD_Untreated_1_3	G6PDD_Untreated_1	1.0	Pyrimidine nucleotides	True	8.165e+06	2.005	2.005	3.558
102	2-Aminoadipate	Amino acid	1	G6PDD_Untreated_1_3	G6PDD_Untreated_1	1.0	Amino acids biosynthesis intermediates	True	9.975e+05	-1.028	-1.028	1.081
45	Fructose-6-P	Carbon	1	G6PDD_Untreated_1_3	G6PDD_Untreated_1	1.0	Glycolysis, GNG	True	4.442e+06	1.126	1.126	1.051
320	TMP	Nucleotide	1	G6PDD_Untreated_1_3	G6PDD_Untreated_1	0.5	Deoxy-nucleotides	True	2.402e+05	-3.083	-3.083	1.709
341	XMP	Nucleotide	1	G6PDD_Untreated_1_3	G6PDD_Untreated_1	0.25	IMP conversion to AMP & GMP	False			-6.353	-1.778
120	beta-OH-Isovalerate	Amino acid	1	G6PDD_Untreated_1_3	G6PDD_Untreated_1	1.0	Amino acids degradation intermediates	True	3.394e+05	-2.584	-2.584	0.5816
322	2'-dI	Nucleotide	1	G6PDD_Untreated_1_3	G6PDD_Untreated_1	0.5	Deoxy-nucleosides	True	4.572e+04	-5.476	-5.476	-1.351
4	GlcNAc 6-P	Carbon	1	G6PDD_Untreated_1_3	G6PDD_Untreated_1	1.0	Aminosugar biosynthesis	True	1.097e+06	-0.8912	-0.8912	-1.22
337	Xanthosine	Nucleotide	1	G6PDD_Untreated_1_3	G6PDD_Untreated_1	1.0	Purine nucleosides	True	1.35e+05	-3.914	-3.914	-0.8394
188	Kynurenine	Amino acid	1	G6PDD_Untreated_1_3	G6PDD_Untreated_1	1.0	Amino acid derivatives	True	1.385e+06	-0.5547	-0.5547	0.1368
63	6-P-Gluconate	Carbon	1	G6PDD_Untreated_1_3	G6PDD_Untreated_1	1.0	Pentose phosphate pathway (PPP)	True	1.437e+07	2.821	2.821	0.787
40	Glucuronate	Carbon	1	G6PDD_Untreated_1_3	G6PDD_Untreated_1	1.0	Sugars and sugar alcohols	True	1.488e+06	-0.4514	-0.4514	1.088
108	Argininosuccinate	Amino acid	1	G6PDD_Untreated_1_3	G6PDD_Untreated_1	1.0	Amino acids biosynthesis intermediates	True	4.623e+05	-2.138	-2.138	-1.912
710	Carnitine	Cofactor	1	G6PDD_Untreated_1_3	G6PDD_Untreated_1	1.0	Cofactors	True	9.665e+07	5.57	5.57	-0.4076
725	P-Pantetheine	Cofactor	1	G6PDD_Untreated_1_3	G6PDD_Untreated_1	0.75	Coenzyme A biosynthesis	False			-4.883	-1.179
48	DHAP	Carbon	1	G6PDD_Untreated_1_3	G6PDD_Untreated_1	1.0	Glycolysis, GNG	True	1.321e+08	6.021	6.021	2.924
17	Maltose	Carbon	1	G6PDD_Untreated_1_3	G6PDD_Untreated_1	1.0	Glycogen degradation	True	3.259e+05	-2.642	-2.642	-1.35
359	N1-Me-Adenosine	Nucleotide	1	G6PDD_Untreated_1_3	G6PDD_Untreated_1	1.0	Purine derivatives in RNAs	True	1.09e+06	-0.9004	-0.9004	0.1373
159	3-Me-His	Amino acid	1	G6PDD_Untreated_1_3	G6PDD_Untreated_1	1.0	Amino acid derivatives	True	1.58e+05	-3.687	-3.687	0.5488

Metabolite ID	Name	Super Pathway	Dataset	Sample ID	Group ID	Detection Fraction	Pathway	Detected	Raw Intensity	Log2 Norm Intensity	Norm Imputed	Log2 Ctrl Norm
155	4-Guanidinobutanoate	Amino acid	1	G6PDD_Untreated_1_3	G6PDD_Untreated_1	1.0	Amino acid derivatives	True	1.065e+06	-0.9342	-0.9342	0.4998
164	5-OH-Lys	Amino acid	1	G6PDD_Untreated_1_3	G6PDD_Untreated_1	1.0	Amino acid derivatives	True	6.023e+05	-1.756	-1.756	0.01399
357	Adenosine-3',5'-PP	Nucleotide	1	G6PDD_Untreated_1_3	G6PDD_Untreated_1	1.0	Purine byproducts of metabolic processes	True	1.038e+05	-4.294	-4.294	-0.8161
104	Cystathionine	Amino acid	1	G6PDD_Untreated_1_3	G6PDD_Untreated_1	1.0	Amino acids biosynthesis intermediates	True	1.75e+07	3.104	3.104	0.8947
113	Imidazole Lactate	Amino acid	1	G6PDD_Untreated_1_3	G6PDD_Untreated_1	1.0	Amino acids degradation intermediates	True	2.364e+05	-3.105	-3.105	0.5713
215	N-Ac-Glu	Amino acid	1	G6PDD_Untreated_1_3	G6PDD_Untreated_1	1.0	N-acetylated amino acids	True	3.384e+06	0.734	0.734	-1.009
310	S-Lactoyl-Glutathione	Amino acid	1	G6PDD_Untreated_1_3	G6PDD_Untreated_1	1.0	Glutathione derivatives	True	4.2e+06	1.046	1.046	0.6893
5	GlcNAc 1-P	Carbon	1	G6PDD_Untreated_1_3	G6PDD_Untreated_1	1.0	Aminosugar biosynthesis	True	2.16e+05	-3.236	-3.236	-1.325
34	Ribitol	Carbon	1	G6PDD_Untreated_1_3	G6PDD_Untreated_1	1.0	Sugars and sugar alcohols	True	2.771e+05	-2.876	-2.876	0.5536
10	UDP-Galactose	Carbon	1	G6PDD_Untreated_1_3	G6PDD_Untreated_1	0.75	Polysaccharide biosynthesis	True	1.934e+06	-0.07312	-0.07312	0.5533
13	Guanosine 5'-PP-Fucose	Carbon	1	G6PDD_Untreated_1_3	G6PDD_Untreated_1	1.0	Polysaccharide biosynthesis	True	5.838e+05	-1.801	-1.801	0.4979
19	Maltotetraose	Carbon	1	G6PDD_Untreated_1_3	G6PDD_Untreated_1	1.0	Glycogen degradation	True	3.468e+05	-2.553	-2.553	-2.509
233	SAM	Amino acid	1	G6PDD_Untreated_1_3	G6PDD_Untreated_1	1.0	SAM metabolism	True	4.067e+06	0.9991	0.9991	1.155
129	5-Aminovalerate	Amino acid	1	G6PDD_Untreated_1_3	G6PDD_Untreated_1	1.0	Amino acids degradation intermediates	True	5.193e+06	1.352	1.352	0.35
741	5-Me-THF	Cofactor	1	G6PDD_Untreated_1_3	G6PDD_Untreated_1	1.0	Folate metabolism	True	8.614e+04	-4.562	-4.562	0.1958
198	Indolelactate	Amino acid	1	G6PDD_Untreated_1_3	G6PDD_Untreated_1	1.0	Amino acid derivatives	True	1.373e+05	-3.889	-3.889	-0.1021
254	Gly-Val	Amino acid	1	G6PDD_Untreated_1_3	G6PDD_Untreated_1	1.0	Dipeptides	True	1.798e+06	-0.1787	-0.1787	-1.29
291	gamma-Glu-Leu	Amino acid	1	G6PDD_Untreated_1_3	G6PDD_Untreated_1	1.0	Gamma-glutamyl dipeptides	True	1.05e+06	-0.9545	-0.9545	-0.4657
173	Met Sulfoxide	Amino acid	1	G6PDD_Untreated_1_3	G6PDD_Untreated_1	1.0	Amino acid derivatives	True	8.233e+06	2.017	2.017	-0.5508

Metabolite ID	Name	Super Pathway	Dataset	Sample ID	Group ID	Detection Fraction	Pathway	Detected	Raw Intensity	Log2 Norm Intensity	Norm Imputed	Log2 Ctrl Norm
43	Glucose	Carbon	1	G6PDD_Untreated_1_3	G6PDD_Untreated_1	1.0	Glycolysis, GNG	True	1.105e+08	5.763	5.763	0.7112
185	Phenyllactate	Amino acid	1	G6PDD_Untreated_1_3	G6PDD_Untreated_1	0.5	Amino acid derivatives	False			-5.917	-0.1051
156	Homo-Arg	Amino acid	1	G6PDD_Untreated_1_3	G6PDD_Untreated_1	1.0	Amino acid derivatives	True	3.392e+06	0.7372	0.7372	-0.4433
135	Homocitrulline	Amino acid	1	G6PDD_Untreated_1_3	G6PDD_Untreated_1	1.0	Amino acids degradation intermediates	True	7.346e+05	-1.47	-1.47	0.2327
719	Nicotinamide MN	Cofactor	1	G6PDD_Untreated_1_3	G6PDD_Untreated_1	1.0	NAD biosynthesis	True	8.617e+04	-4.561	-4.561	-4.747
212	N-Ac-Asp	Amino acid	1	G6PDD_Untreated_1_3	G6PDD_Untreated_1	1.0	N-acetylated amino acids	True	2.438e+06	0.2611	0.2611	-0.4362
720	1-Me-Nicotinamide	Cofactor	1	G6PDD_Untreated_1_3	G6PDD_Untreated_1	1.0	Derivatives of NA, nicotinamide and NAD	True	8.246e+08	8.663	8.663	0.772
216	N-Ac-Gly	Amino acid	1	G6PDD_Untreated_1_3	G6PDD_Untreated_1	1.0	N-acetylated amino acids	True	1.997e+05	-3.349	-3.349	0.1911
70	Creatine	Carbon	1	G6PDD_Untreated_1_3	G6PDD_Untreated_1	1.0	Creatine energy storage	True	1.736e+09	9.737	9.737	0.3196
26	Galactonate	Carbon	1	G6PDD_Untreated_1_3	G6PDD_Untreated_1	1.0	Sugars and sugar alcohols	True	4.346e+06	1.095	1.095	1.9
309	Glutathione, Oxidized	Amino acid	1	G6PDD_Untreated_1_3	G6PDD_Untreated_1	1.0	Glutathione	True	4.265e+06	1.068	1.068	-0.125
35	Ribonate	Carbon	1	G6PDD_Untreated_1_3	G6PDD_Untreated_1	1.0	Sugars and sugar alcohols	True	1.357e+06	-0.5842	-0.5842	-0.05697
160	1-Me-His	Amino acid	1	G6PDD_Untreated_1_3	G6PDD_Untreated_1	1.0	Amino acid derivatives	True	2.916e+07	3.841	3.841	0.3998
44	Glucose 6-P	Carbon	1	G6PDD_Untreated_1_3	G6PDD_Untreated_1	1.0	Glycolysis, GNG	True	1.512e+06	-0.428	-0.428	1.049
704	NADH	Cofactor	1	G6PDD_Untreated_1_3	G6PDD_Untreated_1	1.0	Cofactors	True	2.552e+06	0.3268	0.3268	0.9728
275	Thr-Phe	Amino acid	1	G6PDD_Untreated_1_3	G6PDD_Untreated_1	1.0	Dipeptides	True	1.855e+06	-0.1334	-0.1334	1.52
738	Pyridoxate	Cofactor	1	G6PDD_Untreated_1_3	G6PDD_Untreated_1	1.0	PLP biosynthesis and salvage	True	2.391e+05	-3.089	-3.089	0.2751
177	3-(4-OH-Phenyl)Lactate	Amino acid	1	G6PDD_Untreated_1_3	G6PDD_Untreated_1	1.0	Amino acid derivatives	True	3.661e+05	-2.475	-2.475	-9.32e-03
206	Trans-4-OH-Pro	Amino acid	1	G6PDD_Untreated_1_3	G6PDD_Untreated_1	1.0	Amino acid derivatives	True	1.055e+08	5.696	5.696	0.5408
329	AMP	Nucleotide	1	G6PDD_Untreated_1_3	G6PDD_Untreated_1	1.0	Purine nucleotides	True	1.932e+08	6.569	6.569	2.934

Metabolite ID	Name	Super Pathway	Dataset	Sample ID	Group ID	Detection Fraction	Pathway	Detected	Raw Intensity	Log2 Norm Intensity	Norm Imputed	Log2 Ctrl Norm
11	UDP-Glucose	Carbon	1	G6PDD_Untreated_1_3	G6PDD_Untreated_1	1.0	Polysaccharide biosynthesis	True	7.94e+06	1.964	1.964	2.069
158	4-Imidazole-Ac	Amino acid	1	G6PDD_Untreated_1_3	G6PDD_Untreated_1	1.0	Amino acid derivatives	True	5.24e+05	-1.957	-1.957	0.1894
111	1-Me-Imidazole-Ac	Amino acid	1	G6PDD_Untreated_1_3	G6PDD_Untreated_1	1.0	Amino acids degradation intermediates	True	6.673e+05	-1.608	-1.608	0.3254
345	Guanine	Nucleotide	1	G6PDD_Untreated_1_3	G6PDD_Untreated_1	1.0	Purine bases	True	2.14e+07	3.395	3.395	-1.591
22	N-Ac-Neuraminate	Carbon	1	G6PDD_Untreated_1_3	G6PDD_Untreated_1	1.0	Aminosugar derivatives	True	2.533e+06	0.3159	0.3159	0.4513
721	N'-Methylnicotinate	Cofactor	1	G6PDD_Untreated_1_3	G6PDD_Untreated_1	1.0	Derivatives of NA, nicotinamide and NAD	True	1.53e+06	-0.4113	-0.4113	0.5963
183	Phenol Sulfate	Amino acid	1	G6PDD_Untreated_1_3	G6PDD_Untreated_1	1.0	Amino acid derivatives	True	4.902e+04	-5.375	-5.375	-0.7351
718	Nicotinamide Riboside	Cofactor	1	G6PDD_Untreated_1_3	G6PDD_Untreated_1	1.0	NAD biosynthesis	True	5.07e+05	-2.005	-2.005	-3.595
297	gamma-Glu-Thr	Amino acid	1	G6PDD_Untreated_1_3	G6PDD_Untreated_1	1.0	Gamma-glutamyl dipeptides	True	2.012e+06	-0.01618	-0.01618	-0.6563
295	gamma-Glu-Phe	Amino acid	1	G6PDD_Untreated_1_3	G6PDD_Untreated_1	0.75	Gamma-glutamyl dipeptides	True	3.571e+04	-5.833	-5.833	-0.04752
347	Allantoic Acid	Nucleotide	1	G6PDD_Untreated_1_3	G6PDD_Untreated_1	0.5	Purine degradation	True	7.013e+04	-4.859	-4.859	0.7807
399	Pseudouridine	Nucleotide	1	G6PDD_Untreated_1_3	G6PDD_Untreated_1	1.0	Pyrimidine derivatives in RNAs	True	4.333e+05	-2.231	-2.231	-0.2013
375	UTP	Nucleotide	1	G6PDD_Untreated_1_3	G6PDD_Untreated_1	1.0	Pyrimidine nucleotides	True	3.973e+06	0.9652	0.9652	2.941
144	Glu, gamma-Me Ester	Amino acid	1	G6PDD_Untreated_1_3	G6PDD_Untreated_1	1.0	Amino acid derivatives	True	3.628e+06	0.8343	0.8343	0.2565
292	gamma-Glu-epsilon-Lysine	Amino acid	1	G6PDD_Untreated_1_3	G6PDD_Untreated_1	1.0	Gamma-glutamyl dipeptides	True	1.241e+06	-0.7138	-0.7138	-0.5224
225	N-Ac-Thr	Amino acid	1	G6PDD_Untreated_1_3	G6PDD_Untreated_1	1.0	N-acetylated amino acids	True	5.035e+05	-2.015	-2.015	-0.5121
211	N-Ac-Asn	Amino acid	1	G6PDD_Untreated_1_3	G6PDD_Untreated_1	1.0	N-acetylated amino acids	True	4.074e+05	-2.32	-2.32	0.3649
151	Phenylacetylglycine	Amino acid	1	G6PDD_Untreated_1_3	G6PDD_Untreated_1	1.0	Amino acid derivatives	True	3.332e+06	0.7116	0.7116	1.499
217	N-Ac-His	Amino acid	1	G6PDD_Untreated_1_3	G6PDD_Untreated_1	1.0	N-acetylated amino acids	True	1.352e+05	-3.911	-3.911	-0.4263
288	gamma-Glu-Gly	Amino acid	1	G6PDD_Untreated_1_3	G6PDD_Untreated_1	0.75	Gamma-glutamyl dipeptides	True	4.391e+05	-2.212	-2.212	-0.3876

Metabolite ID	Name	Super Pathway	Dataset	Sample ID	Group ID	Detection Fraction	Pathway	Detected	Raw Intensity	Log2 Norm Intensity	Norm Imputed	Log2 Ctrl Norm
222	N-Ac-Phe	Amino acid	1	G6PDD_Untreated_1_3	G6PDD_Untreated_1	1.0	N-acetylated amino acids	True	3.909e+04	-5.702	-5.702	0.5706
71	Creatine-P	Carbon	1	G6PDD_Untreated_1_3	G6PDD_Untreated_1	1.0	Creatine energy storage	True	1.294e+05	-3.975	-3.975	0.2798
210	N-Ac-Arg	Amino acid	1	G6PDD_Untreated_1_3	G6PDD_Untreated_1	0.75	N-acetylated amino acids	True	2.786e+05	-2.868	-2.868	-0.1928
218	N-Ac-Ile	Amino acid	1	G6PDD_Untreated_1_3	G6PDD_Untreated_1	0.5	N-acetylated amino acids	True	7.088e+04	-4.843	-4.843	1.123
251	Gly-Leu	Amino acid	1	G6PDD_Untreated_1_3	G6PDD_Untreated_1	1.0	Dipeptides	True	3.848e+06	0.9192	0.9192	-0.7195
290	gamma-Glu-Ile	Amino acid	1	G6PDD_Untreated_1_3	G6PDD_Untreated_1	1.0	Gamma-glutamyl dipeptides	True	5.718e+05	-1.831	-1.831	-0.4927
316	Ophthalmate	Amino acid	1	G6PDD_Untreated_1_3	G6PDD_Untreated_1	1.0	Oxidative stress markers	True	5.417e+06	1.413	1.413	-0.93
125	Isovaleryl-Gly	Amino acid	1	G6PDD_Untreated_1_3	G6PDD_Untreated_1	1.0	Amino acids degradation intermediates	True	7.018e+04	-4.858	-4.858	0.5518
368	7-Me-Guanine	Nucleotide	1	G6PDD_Untreated_1_3	G6PDD_Untreated_1	1.0	Purine derivatives in RNAs	True	5.973e+05	-1.768	-1.768	0.4218
208	Pro-OH-Pro	Amino acid	1	G6PDD_Untreated_1_3	G6PDD_Untreated_1	1.0	Amino acid derivatives	True	3.331e+07	4.033	4.033	0.7038
366	N2,N2-DiMe-Guanosine	Nucleotide	1	G6PDD_Untreated_1_3	G6PDD_Untreated_1	1.0	Purine derivatives in RNAs	True	1.159e+05	-4.134	-4.134	-0.02072
352	3'-AMP	Nucleotide	1	G6PDD_Untreated_1_3	G6PDD_Untreated_1	1.0	Purine derivatives in signaling	True	9.306e+05	-1.129	-1.129	0.3582
363	N6-Carbamoyl-Thr-Adenosine	Nucleotide	1	G6PDD_Untreated_1_3	G6PDD_Untreated_1	1.0	Purine derivatives in RNAs	True	7.583e+04	-4.746	-4.746	-0.06048
314	Cys-Glutathione Disulfide	Amino acid	1	G6PDD_Untreated_1_3	G6PDD_Untreated_1	0.25	Oxidative stress markers	False			-5.284	-2
382	Orotidine	Nucleotide	1	G6PDD_Untreated_1_3	G6PDD_Untreated_1	1.0	Pyrimidine (UMP) biosynthesis	True	1.142e+05	-4.155	-4.155	0.1365
307	Cys-Gly	Amino acid	1	G6PDD_Untreated_1_3	G6PDD_Untreated_1	1.0	Glutathione biosynthesis	True	8.753e+06	2.105	2.105	0.498
64	Sedoheptulose-7-P	Carbon	1	G6PDD_Untreated_1_3	G6PDD_Untreated_1	1.0	Pentose phosphate pathway (PPP)	True	1.466e+06	-0.4729	-0.4729	-0.6269
142	N-Ac-Asp-Glu	Amino acid	1	G6PDD_Untreated_1_3	G6PDD_Untreated_1	1.0	Amino acid derivatives	True	1.12e+06	-0.861	-0.861	0.2844
708	Thiamin-PP	Cofactor	1	G6PDD_Untreated_1_3	G6PDD_Untreated_1	1.0	Cofactors	True	8.432e+04	-4.593	-4.593	1.391
182	P-Cresol Sulfate	Amino acid	1	G6PDD_Untreated_1_3	G6PDD_Untreated_1	1.0	Amino acid derivatives	True	2.319e+05	-3.133	-3.133	0.06267

Metabolite ID	Name	Super Pathway	Dataset	Sample ID	Group ID	Detection Fraction	Pathway	Detected	Raw Intensity	Log2 Norm Intensity	Norm Imputed	Log2 Ctrl Norm
250	Gly-Ile	Amino acid	1	G6PDD_Untreated_1_3	G6PDD_Untreated_1	1.0	Dipeptides	True	7.7e+05	-1.402	-1.402	-0.08465
286	gamma-Glu-Glu	Amino acid	1	G6PDD_Untreated_1_3	G6PDD_Untreated_1	1.0	Gamma-glutamyl dipeptides	True	3.513e+06	0.788	0.788	0.7701
739	Deoxycarnitine	Cofactor	1	G6PDD_Untreated_1_3	G6PDD_Untreated_1	1.0	Carnitine biosynthesis	True	2.275e+07	3.483	3.483	-0.2684
203	DiMe-Arg	Amino acid	1	G6PDD_Untreated_1_3	G6PDD_Untreated_1	1.0	Amino acid derivatives	True	2.045e+07	3.329	3.329	-2.303
351	2'-AMP	Nucleotide	1	G6PDD_Untreated_1_3	G6PDD_Untreated_1	1.0	Purine derivatives in signaling	True	5.877e+04	-5.114	-5.114	-2.845
8	Cytidine 5'-P-N-Ac-Neuraminate	Carbon	1	G6PDD_Untreated_1_3	G6PDD_Untreated_1	1.0	Aminosugar biosynthesis	True	6.444e+05	-1.659	-1.659	0.0866
285	gamma-Glu-Ala	Amino acid	1	G6PDD_Untreated_1_3	G6PDD_Untreated_1	1.0	Gamma-glutamyl dipeptides	True	1.527e+05	-3.736	-3.736	-0.4976
224	N-Ac-Ser	Amino acid	1	G6PDD_Untreated_1_3	G6PDD_Untreated_1	1.0	N-acetylated amino acids	True	7.449e+06	1.872	1.872	-7.07e-03
244	Ala-Leu	Amino acid	1	G6PDD_Untreated_1_3	G6PDD_Untreated_1	1.0	Dipeptides	True	8.106e+06	1.994	1.994	0.8562
207	N-Me-Pro	Amino acid	1	G6PDD_Untreated_1_3	G6PDD_Untreated_1	1.0	Amino acid derivatives	True	1.295e+06	-0.6518	-0.6518	0.2782
171	Cys Sulfinic Acid	Amino acid	1	G6PDD_Untreated_1_3	G6PDD_Untreated_1	0.75	Amino acid derivatives	False			-5.211	-1.913
181	O-Me-Tyr	Amino acid	1	G6PDD_Untreated_1_3	G6PDD_Untreated_1	1.0	Amino acid derivatives	True	3.272e+05	-2.636	-2.636	0.7334
240	N-Ac-Putrescine	Amino acid	1	G6PDD_Untreated_1_3	G6PDD_Untreated_1	1.0	Polyamine derivatives	True	2.363e+06	0.2158	0.2158	2.58
176	S-Me-Met	Amino acid	1	G6PDD_Untreated_1_3	G6PDD_Untreated_1	1.0	Amino acid derivatives	True	5.204e+05	-1.967	-1.967	-0.1254
339	AICAR	Nucleotide	1	G6PDD_Untreated_1_3	G6PDD_Untreated_1	1.0	IMP biosynthesis	True	9.336e+04	-4.446	-4.446	-0.1448
141	gamma-Carboxy-Glu	Amino acid	1	G6PDD_Untreated_1_3	G6PDD_Untreated_1	1.0	Amino acid derivatives	True	2.167e+06	0.09081	0.09081	0.8758
392	3'-UMP	Nucleotide	1	G6PDD_Untreated_1_3	G6PDD_Untreated_1	0.75	Pyrimidine derivatives in signaling	True	4.193e+05	-2.279	-2.279	1.754
355	3'-GMP	Nucleotide	1	G6PDD_Untreated_1_3	G6PDD_Untreated_1	1.0	Purine derivatives in signaling	True	5.687e+05	-1.839	-1.839	2.098
282	Val-Leu	Amino acid	1	G6PDD_Untreated_1_3	G6PDD_Untreated_1	1.0	Dipeptides	True	8.358e+06	2.038	2.038	1.067
140	Carboxyethyl-GABA	Amino acid	1	G6PDD_Untreated_1_3	G6PDD_Untreated_1	1.0	Amino acid derivatives	True	1.145e+06	-0.8292	-0.8292	0.6019
258	Ile-Gly	Amino acid	1	G6PDD_Untreated_1_3	G6PDD_Untreated_1	1.0	Dipeptides	True	1.671e+07	3.038	3.038	-0.274

Metabolite ID	Name	Super Pathway	Dataset	Sample ID	Group ID	Detection Fraction	Pathway	Detected	Raw Intensity	Log2 Norm Intensity	Norm Imputed	Log2 Ctrl Norm
260	Leu-Ala	Amino acid	1	G6PDD_Untreated_1_3	G6PDD_Untreated_1	1.0	Dipeptides	True	6.486e+06	1.672	1.672	0.3712
265	Lys-Leu	Amino acid	1	G6PDD_Untreated_1_3	G6PDD_Untreated_1	1.0	Dipeptides	True	7.022e+05	-1.535	-1.535	1.076
263	Leu-Gly	Amino acid	1	G6PDD_Untreated_1_3	G6PDD_Untreated_1	1.0	Dipeptides	True	2.805e+07	3.785	3.785	0.764
281	Val-Gly	Amino acid	1	G6PDD_Untreated_1_3	G6PDD_Untreated_1	1.0	Dipeptides	True	3.435e+07	4.077	4.077	-0.4418
270	Pro-Gly	Amino acid	1	G6PDD_Untreated_1_3	G6PDD_Untreated_1	1.0	Dipeptides	True	1.638e+06	-0.3132	-0.3132	-1.06
114	Imidazole Propionate	Amino acid	1	G6PDD_Untreated_1_3	G6PDD_Untreated_1	1.0	Amino acids degradation intermediates	True	1.03e+06	-0.9821	-0.9821	1.329
267	Phe-Gly	Amino acid	1	G6PDD_Untreated_1_3	G6PDD_Untreated_1	1.0	Dipeptides	True	2.151e+07	3.402	3.402	-0.692
266	Phe-Ala	Amino acid	1	G6PDD_Untreated_1_3	G6PDD_Untreated_1	1.0	Dipeptides	True	5.09e+06	1.323	1.323	0.2461
278	Tyr-Gly	Amino acid	1	G6PDD_Untreated_1_3	G6PDD_Untreated_1	1.0	Dipeptides	True	7.286e+06	1.84	1.84	-0.342
255	His-Ala	Amino acid	1	G6PDD_Untreated_1_3	G6PDD_Untreated_1	1.0	Dipeptides	True	4.232e+05	-2.265	-2.265	-0.928
280	Val-Gln	Amino acid	1	G6PDD_Untreated_1_3	G6PDD_Untreated_1	1.0	Dipeptides	True	8.012e+06	1.977	1.977	0.3593
143	S-1-Pyrroline-5-Carboxylate	Amino acid	1	G6PDD_Untreated_1_3	G6PDD_Untreated_1	1.0	Amino acid derivatives	True	4.527e+05	-2.168	-2.168	-0.1002
232	SAH	Amino acid	1	G6PDD_Untreated_1_3	G6PDD_Untreated_1	1.0	SAM metabolism	True	1.066e+06	-0.9325	-0.9325	-0.4471
20	Erythronate	Carbon	1	G6PDD_Untreated_1_3	G6PDD_Untreated_1	1.0	Aminosugar derivatives	True	3.52e+07	4.113	4.113	0.3714
248	Gln-Leu	Amino acid	1	G6PDD_Untreated_1_3	G6PDD_Untreated_1	1.0	Dipeptides	True	1.493e+06	-0.4462	-0.4462	0.459
276	Trp-Gly	Amino acid	1	G6PDD_Untreated_1_3	G6PDD_Untreated_1	1.0	Dipeptides	True	5.964e+05	-1.77	-1.77	-0.6921
205	N-delta-Ac-Ornithine	Amino acid	1	G6PDD_Untreated_1_3	G6PDD_Untreated_1	1.0	Amino acid derivatives	True	1.111e+06	-0.8725	-0.8725	0.2486
163	Formimino-Glu	Amino acid	1	G6PDD_Untreated_1_3	G6PDD_Untreated_1	1.0	Amino acid derivatives	True	8.773e+05	-1.214	-1.214	0.02614
204	N-Me-Arg	Amino acid	1	G6PDD_Untreated_1_3	G6PDD_Untreated_1	1.0	Amino acid derivatives	True	4.632e+06	1.187	1.187	-1.4
242	Guanidino-Ac	Amino acid	1	G6PDD_Untreated_1_3	G6PDD_Untreated_1	0.75	Creatine biosynthesis	True	1.557e+05	-3.708	-3.708	0.1724

Metabolite ID	Name	Super Pathway	Dataset	Sample ID	Group ID	Detection Fraction	Pathway	Detected	Raw Intensity	Log2 Norm Intensity	Norm Imputed	Log2 Ctrl Norm
300	gamma-Glu-Val	Amino acid	1	G6PDD_Untreated_1_3	G6PDD_Untreated_1	0.75	Gamma-glutamyl dipeptides	True	2.058e+06	0.01618	0.01618	-0.6046
53	Ac-CoA	Carbon	1	G6PDD_Untreated_1_3	G6PDD_Untreated_1	0.5	Acetyl-CoA	True	1.805e+05	-3.495	-3.495	3.268
18	Maltotriose	Carbon	1	G6PDD_Untreated_1_3	G6PDD_Untreated_1	1.0	Glycogen degradation	True	1.316e+06	-0.6289	-0.6289	-0.4711
294	gamma-Glu-Met	Amino acid	1	G6PDD_Untreated_1_3	G6PDD_Untreated_1	0.5	Gamma-glutamyl dipeptides	False			-4.503	-1.021
174	Met Sulfone	Amino acid	1	G6PDD_Untreated_1_3	G6PDD_Untreated_1	1.0	Amino acid derivatives	True	3.653e+05	-2.478	-2.478	0.601
175	N-Ac-Met Sulfoxide	Amino acid	1	G6PDD_Untreated_1_3	G6PDD_Untreated_1	1.0	Amino acid derivatives	True	1.649e+06	-0.3035	-0.3035	-0.2798
25	Mannitol/Sorbitol	Carbon	1	G6PDD_Untreated_1_3	G6PDD_Untreated_1	1.0	Sugars and sugar alcohols	True	1.087e+07	2.417	2.417	0.1927
6	UDP-GlcNAc	Carbon	1	G6PDD_Untreated_1_3	G6PDD_Untreated_1	0.5	Aminosugar biosynthesis	True	2.456e+05	-3.051	-3.051	0.0572
145	Pyro-Gln	Amino acid	1	G6PDD_Untreated_1_3	G6PDD_Untreated_1	1.0	Amino acid derivatives	True	4.531e+06	1.155	1.155	0.3151
705	Coenzyme A	Cofactor	1	G6PDD_Untreated_1_3	G6PDD_Untreated_1	1.0	Cofactors	True	7.86e+05	-1.372	-1.372	2.587
319	2'-dAMP	Nucleotide	1	G6PDD_Untreated_1_3	G6PDD_Untreated_1	0.75	Deoxy-nucleotides	True	3.531e+05	-2.527	-2.527	1.153
119	alpha-OH-Isovalerate	Amino acid	1	G6PDD_Untreated_1_3	G6PDD_Untreated_1	0.5	Amino acids degradation intermediates	False			-5.298	-1.999
46	Fructose 1,6-PP / Glucose 1,6-PP / Inositol-1,4-PP / Inositol-1,3-PP	Carbon	1	G6PDD_Untreated_1_3	G6PDD_Untreated_1	1.0	Glycolysis, GNG	True	1.46e+08	6.165	6.165	1.334
137	1-Me-Guanidine	Amino acid	1	G6PDD_Untreated_1_3	G6PDD_Untreated_1	1.0	Amino acids degradation intermediates	True	4.958e+04	-5.359	-5.359	-0.2061
23	N-GlcNAc-Asn	Carbon	1	G6PDD_Untreated_1_3	G6PDD_Untreated_1	1.0	Aminosugar derivatives	True	3.926e+06	0.9482	0.9482	0.2353
262	Leu-Gln	Amino acid	1	G6PDD_Untreated_1_3	G6PDD_Untreated_1	1.0	Dipeptides	True	8.249e+06	2.019	2.019	0.723
24	Fructose	Carbon	1	G6PDD_Untreated_1_3	G6PDD_Untreated_1	1.0	Sugars and sugar alcohols	True	9.444e+06	2.215	2.215	0.2657
197	C-Glycosyl-Trp	Amino acid	1	G6PDD_Untreated_1_3	G6PDD_Untreated_1	1.0	Amino acid derivatives	True	1.244e+06	-0.7098	-0.7098	-0.4108
33	Arabitol/Xylitol	Carbon	1	G6PDD_Untreated_1_3	G6PDD_Untreated_1	1.0	Sugars and sugar alcohols	True	4.175e+05	-2.285	-2.285	-0.425

Metabolite ID	Name	Super Pathway	Dataset	Sample ID	Group ID	Detection Fraction	Pathway	Detected	Raw Intensity	Log2 Norm Intensity	Norm Imputed	Log2 Ctrl Norm
128	N2-Ac-Lys/N6-Ac-Lys	Amino acid	1	G6PDD_Untreated_1_3	G6PDD_Untreated_1	1.0	Amino acids degradation intermediates	True	1.502e+06	-0.4376	-0.4376	-1.648
42	2-Me-Citrate	Carbon	1	G6PDD_Untreated_1_3	G6PDD_Untreated_1	1.0	Propionate metabolism	True	9.545e+04	-4.414	-4.414	-0.03095
12	Glucuronate 1-P	Carbon	1	G6PDD_Untreated_1_3	G6PDD_Untreated_1	1.0	Polysaccharide biosynthesis	True	5.667e+05	-1.844	-1.844	-0.6015
76	Gln	Amino acid	1	G6PDD_Untreated_1_4	G6PDD_Untreated_1	1.0	Proteinogenic amino acids	True	4.721e+08	7.541	7.541	-1.14
89	Trp	Amino acid	1	G6PDD_Untreated_1_4	G6PDD_Untreated_1	1.0	Proteinogenic amino acids	True	8.875e+07	5.13	5.13	-0.5461
723	beta-Ala	Cofactor	1	G6PDD_Untreated_1_4	G6PDD_Untreated_1	1.0	Coenzyme A biosynthesis	True	2.099e+07	3.05	3.05	0.04028
75	Glu	Amino acid	1	G6PDD_Untreated_1_4	G6PDD_Untreated_1	1.0	Proteinogenic amino acids	True	4.866e+09	10.91	10.91	0.17
77	Gly	Amino acid	1	G6PDD_Untreated_1_4	G6PDD_Untreated_1	1.0	Proteinogenic amino acids	True	8.514e+07	5.07	5.07	-0.5455
80	His	Amino acid	1	G6PDD_Untreated_1_4	G6PDD_Untreated_1	1.0	Proteinogenic amino acids	True	1.642e+07	2.695	2.695	-0.7199
82	Leu	Amino acid	1	G6PDD_Untreated_1_4	G6PDD_Untreated_1	1.0	Proteinogenic amino acids	True	8.554e+08	8.398	8.398	-0.8228
87	Phe	Amino acid	1	G6PDD_Untreated_1_4	G6PDD_Untreated_1	1.0	Proteinogenic amino acids	True	6.938e+08	8.096	8.096	-0.715
130	Glutarate	Amino acid	1	G6PDD_Untreated_1_4	G6PDD_Untreated_1	1.0	Amino acids degradation intermediates	True	5.092e+05	-2.316	-2.316	0.111
196	5-OH-Indole-Ac	Amino acid	1	G6PDD_Untreated_1_4	G6PDD_Untreated_1	0.75	Amino acid derivatives	True	1.668e+05	-3.926	-3.926	0.08582
74	Asp	Amino acid	1	G6PDD_Untreated_1_4	G6PDD_Untreated_1	1.0	Proteinogenic amino acids	True	3.963e+08	7.289	7.289	-0.603
236	Spermidine	Amino acid	1	G6PDD_Untreated_1_4	G6PDD_Untreated_1	1.0	Polyamines	True	4.699e+07	4.212	4.212	0.3508
73	Asn	Amino acid	1	G6PDD_Untreated_1_4	G6PDD_Untreated_1	1.0	Proteinogenic amino acids	True	1.736e+08	6.098	6.098	-0.5735
243	Creatinine	Amino acid	1	G6PDD_Untreated_1_4	G6PDD_Untreated_1	1.0	Creatine degradation	True	8.354e+07	5.043	5.043	0.0403
376	Cytidine	Nucleotide	1	G6PDD_Untreated_1_4	G6PDD_Untreated_1	0.75	Pyrimidine nucleosides	True	1.609e+06	-0.6553	-0.6553	-1.383
41	Lactate	Carbon	1	G6PDD_Untreated_1_4	G6PDD_Untreated_1	1.0	Respiratory carbon sources	True	2.797e+08	6.786	6.786	0.3041
58	alpha-Ketoglutarate	Carbon	1	G6PDD_Untreated_1_4	G6PDD_Untreated_1	1.0	TCA cycle	True	4.373e+06	0.7867	0.7867	0.77

Metabolite ID	Name	Super Pathway	Dataset	Sample ID	Group ID	Detection Fraction	Pathway	Detected	Raw Intensity	Log2 Norm Intensity	Norm Imputed	Log2 Ctrl Norm
69	3-OH-Butyrate	Carbon	1	G6PDD_Untreated_1_4	G6PDD_Untreated_1	0.75	Ketone bodies	False			-3.497	-0.5127
343	Adenine	Nucleotide	1	G6PDD_Untreated_1_4	G6PDD_Untreated_1	1.0	Purine bases	True	7.53e+06	1.571	1.571	0.1138
336	Adenosine	Nucleotide	1	G6PDD_Untreated_1_4	G6PDD_Untreated_1	1.0	Purine nucleosides	True	2.635e+07	3.378	3.378	-0.04477
722	ADP-Ribose	Cofactor	1	G6PDD_Untreated_1_4	G6PDD_Untreated_1	1.0	Derivatives of NA, nicotinamide and NAD	True	1.695e+06	-0.5804	-0.5804	2.422
383	Cytosine	Nucleotide	1	G6PDD_Untreated_1_4	G6PDD_Untreated_1	0.75	Pyrimidine bases	True	1.978e+05	-3.68	-3.68	1.175
3	Glucosamine 6-P	Carbon	1	G6PDD_Untreated_1_4	G6PDD_Untreated_1	1.0	Aminosugar biosynthesis	True	3.401e+06	0.4242	0.4242	2.092
717	Nicotinamide	Cofactor	1	G6PDD_Untreated_1_4	G6PDD_Untreated_1	1.0	NAD biosynthesis	True	5.722e+07	4.497	4.497	-0.01859
51	PEP	Carbon	1	G6PDD_Untreated_1_4	G6PDD_Untreated_1	1.0	Glycolysis, GNG	True	5.474e+06	1.111	1.111	0.05961
237	Spermine	Amino acid	1	G6PDD_Untreated_1_4	G6PDD_Untreated_1	1.0	Polyamines	True	2.908e+05	-3.124	-3.124	-1.633
385	Uracil	Nucleotide	1	G6PDD_Untreated_1_4	G6PDD_Untreated_1	1.0	Pyrimidine bases	True	7.752e+05	-1.709	-1.709	-1.974
377	Uridine	Nucleotide	1	G6PDD_Untreated_1_4	G6PDD_Untreated_1	1.0	Pyrimidine nucleosides	True	2.125e+07	3.068	3.068	-0.9813
348	Allantoin	Nucleotide	1	G6PDD_Untreated_1_4	G6PDD_Untreated_1	1.0	Purine degradation	True	2.463e+06	-0.04171	-0.04171	0.1339
335	Inosine	Nucleotide	1	G6PDD_Untreated_1_4	G6PDD_Untreated_1	1.0	Purine nucleosides	True	6.609e+07	4.705	4.705	-0.7945
81	Ile	Amino acid	1	G6PDD_Untreated_1_4	G6PDD_Untreated_1	1.0	Proteinogenic amino acids	True	8.196e+08	8.337	8.337	-0.3783
72	Ala	Amino acid	1	G6PDD_Untreated_1_4	G6PDD_Untreated_1	1.0	Proteinogenic amino acids	True	7.54e+08	8.217	8.217	-0.2848
79	Thr	Amino acid	1	G6PDD_Untreated_1_4	G6PDD_Untreated_1	1.0	Proteinogenic amino acids	True	3.641e+08	7.166	7.166	-0.8431
88	Tyr	Amino acid	1	G6PDD_Untreated_1_4	G6PDD_Untreated_1	1.0	Proteinogenic amino acids	True	2.445e+08	6.592	6.592	-0.9319
84	Lys	Amino acid	1	G6PDD_Untreated_1_4	G6PDD_Untreated_1	1.0	Proteinogenic amino acids	True	3.876e+08	7.256	7.256	-0.7335
86	Met	Amino acid	1	G6PDD_Untreated_1_4	G6PDD_Untreated_1	1.0	Proteinogenic amino acids	True	3.864e+08	7.252	7.252	-0.9757
61	Malate	Carbon	1	G6PDD_Untreated_1_4	G6PDD_Untreated_1	1.0	TCA cycle	True	4.643e+08	7.517	7.517	0.3453

Metabolite ID	Name	Super Pathway	Dataset	Sample ID	Group ID	Detection Fraction	Pathway	Detected	Raw Intensity	Log2 Norm Intensity	Norm Imputed	Log2 Ctrl Norm
235	Putrescine	Amino acid	1	G6PDD_Untreated_1_4	G6PDD_Untreated_1	1.0	Polyamines	True	7.293e+06	1.525	1.525	2.016
324	2'-dU	Nucleotide	1	G6PDD_Untreated_1_4	G6PDD_Untreated_1	0.5	Deoxy-nucleosides	False			-5.281	-0.9009
49	3-P-Glycerate	Carbon	1	G6PDD_Untreated_1_4	G6PDD_Untreated_1	1.0	Glycolysis, GNG	True	3.754e+07	3.889	3.889	0.6231
189	Kynurenate	Amino acid	1	G6PDD_Untreated_1_4	G6PDD_Untreated_1	0.75	Amino acid derivatives	True	3.176e+04	-6.319	-6.319	-0.5206
234	5-Me-Thioadenosine	Amino acid	1	G6PDD_Untreated_1_4	G6PDD_Untreated_1	1.0	SAM metabolism	True	1.261e+07	2.315	2.315	0.4291
59	Succinate	Carbon	1	G6PDD_Untreated_1_4	G6PDD_Untreated_1	1.0	TCA cycle	True	7.575e+06	1.579	1.579	1.278
36	Ribose	Carbon	1	G6PDD_Untreated_1_4	G6PDD_Untreated_1	1.0	Sugars and sugar alcohols	True	7.171e+05	-1.822	-1.822	-0.5882
133	Ornithine	Amino acid	1	G6PDD_Untreated_1_4	G6PDD_Untreated_1	1.0	Amino acids degradation intermediates	True	8.351e+07	5.042	5.042	0.5624
313	5-Oxoproline	Amino acid	1	G6PDD_Untreated_1_4	G6PDD_Untreated_1	1.0	Glutathione derivatives	True	1.169e+07	2.206	2.206	0.1183
165	N-6-Tri-Me-Lys	Amino acid	1	G6PDD_Untreated_1_4	G6PDD_Untreated_1	1.0	Amino acid derivatives	True	1.628e+07	2.683	2.683	-1.081
380	Orotate	Nucleotide	1	G6PDD_Untreated_1_4	G6PDD_Untreated_1	1.0	Pyrimidine (UMP) biosynthesis	True	4.903e+05	-2.37	-2.37	1.26
724	Pantothenate	Cofactor	1	G6PDD_Untreated_1_4	G6PDD_Untreated_1	1.0	Coenzyme A biosynthesis	True	2.893e+08	6.834	6.834	0.5242
150	N-Me-Gly	Amino acid	1	G6PDD_Untreated_1_4	G6PDD_Untreated_1	1.0	Amino acid derivatives	True	1.517e+06	-0.7404	-0.7404	-0.44
122	3-OH-Isobutyrate	Amino acid	1	G6PDD_Untreated_1_4	G6PDD_Untreated_1	0.5	Amino acids degradation intermediates	False			-3.901	-0.7452
241	4-Acetamidobutanoate	Amino acid	1	G6PDD_Untreated_1_4	G6PDD_Untreated_1	1.0	Polyamine derivatives	True	4.428e+06	0.8047	0.8047	0.3637
711	alpha-Tocopherol	Cofactor	1	G6PDD_Untreated_1_4	G6PDD_Untreated_1	1.0	Cofactors	True	8.406e+06	1.73	1.73	0.9492
55	Citrate	Carbon	1	G6PDD_Untreated_1_4	G6PDD_Untreated_1	1.0	TCA cycle	True	1.178e+07	2.217	2.217	0.2676
387	3-Aminoisobutyrate	Nucleotide	1	G6PDD_Untreated_1_4	G6PDD_Untreated_1	1.0	Pyrimidine degradation	True	6.625e+05	-1.936	-1.936	0.07787
338	Guanosine	Nucleotide	1	G6PDD_Untreated_1_4	G6PDD_Untreated_1	1.0	Purine nucleosides	True	5.002e+07	4.303	4.303	-0.8034
209	N-Ac-Ala	Amino acid	1	G6PDD_Untreated_1_4	G6PDD_Untreated_1	1.0	N-acetylated amino acids	True	7.555e+05	-1.746	-1.746	-0.5451

Metabolite ID	Name	Super Pathway	Dataset	Sample ID	Group ID	Detection Fraction	Pathway	Detected	Raw Intensity	Log2 Norm Intensity	Norm Imputed	Log2 Ctrl Norm
221	N-Ac-Met	Amino acid	1	G6PDD_Untreated_1_4	G6PDD_Untreated_1	1.0	N-acetylated amino acids	True	5.187e+06	1.033	1.033	-0.7113
228	N-Ac-Val	Amino acid	1	G6PDD_Untreated_1_4	G6PDD_Untreated_1	1.0	N-acetylated amino acids	True	4.488e+04	-5.82	-5.82	-0.449
346	Urate	Nucleotide	1	G6PDD_Untreated_1_4	G6PDD_Untreated_1	1.0	Purine degradation	True	1.142e+06	-1.151	-1.151	-0.088
90	Arg	Amino acid	1	G6PDD_Untreated_1_4	G6PDD_Untreated_1	1.0	Proteinogenic amino acids	True	1.179e+09	8.861	8.861	-0.8445
60	Fumarate	Carbon	1	G6PDD_Untreated_1_4	G6PDD_Untreated_1	1.0	TCA cycle	True	6.732e+06	1.409	1.409	0.3939
78	Ser	Amino acid	1	G6PDD_Untreated_1_4	G6PDD_Untreated_1	1.0	Proteinogenic amino acids	True	1.47e+08	5.858	5.858	-1.399
83	Val	Amino acid	1	G6PDD_Untreated_1_4	G6PDD_Untreated_1	1.0	Proteinogenic amino acids	True	3.316e+08	7.031	7.031	-0.8409
734	Pyridoxal	Cofactor	1	G6PDD_Untreated_1_4	G6PDD_Untreated_1	1.0	PLP biosynthesis and salvage	True	6.369e+06	1.329	1.329	-0.454
136	Urea	Amino acid	1	G6PDD_Untreated_1_4	G6PDD_Untreated_1	1.0	Amino acids degradation intermediates	True	2.691e+06	0.08603	0.08603	-0.06519
67	Ribose 1-P	Carbon	1	G6PDD_Untreated_1_4	G6PDD_Untreated_1	1.0	Pentose phosphate pathway (PPP)	True	5.196e+06	1.035	1.035	0.4142
284	Carnosine	Amino acid	1	G6PDD_Untreated_1_4	G6PDD_Untreated_1	1.0	Dipeptides	True	5.55e+05	-2.191	-2.191	-0.08299
306	gamma-Glu-Cys	Amino acid	1	G6PDD_Untreated_1_4	G6PDD_Untreated_1	1.0	Glutathione biosynthesis	True	9.898e+05	-1.357	-1.357	-1.02
712	Retinol (Vit A)	Cofactor	1	G6PDD_Untreated_1_4	G6PDD_Untreated_1	1.0	Cofactors	True	5.826e+05	-2.121	-2.121	0.3119
85	Cys	Amino acid	1	G6PDD_Untreated_1_4	G6PDD_Untreated_1	1.0	Proteinogenic amino acids	True	4.133e+07	4.027	4.027	-0.4864
91	Pro	Amino acid	1	G6PDD_Untreated_1_4	G6PDD_Untreated_1	1.0	Proteinogenic amino acids	True	1.569e+09	9.273	9.273	-0.1346
308	Glutathione, Reduced	Amino acid	1	G6PDD_Untreated_1_4	G6PDD_Untreated_1	1.0	Glutathione	True	2.636e+08	6.7	6.7	-0.2558
107	Citrulline	Amino acid	1	G6PDD_Untreated_1_4	G6PDD_Untreated_1	1.0	Amino acids biosynthesis intermediates	True	1.526e+07	2.589	2.589	-0.2349
328	IMP	Nucleotide	1	G6PDD_Untreated_1_4	G6PDD_Untreated_1	0.5	Purine nucleotides	True	1.204e+05	-4.396	-4.396	-0.07436
706	FAD	Cofactor	1	G6PDD_Untreated_1_4	G6PDD_Untreated_1	1.0	Cofactors	True	5.433e+05	-2.222	-2.222	-0.03249
735	Pyridoxamine	Cofactor	1	G6PDD_Untreated_1_4	G6PDD_Untreated_1	1.0	PLP biosynthesis and salvage	True	2.901e+06	0.1948	0.1948	0.3878

Metabolite ID	Name	Super Pathway	Dataset	Sample ID	Group ID	Detection Fraction	Pathway	Detected	Raw Intensity	Log2 Norm Intensity	Norm Imputed	Log2 Ctrl Norm
199	Serotonin	Amino acid	1	G6PDD_Untreated_1_4	G6PDD_Untreated_1	1.0	Amino acid derivatives	True	4.082e+06	0.6873	0.6873	-0.5854
370	CMP	Nucleotide	1	G6PDD_Untreated_1_4	G6PDD_Untreated_1	1.0	Pyrimidine nucleotides	True	1.147e+07	2.178	2.178	0.3395
287	gamma-Glu-Gln	Amino acid	1	G6PDD_Untreated_1_4	G6PDD_Untreated_1	1.0	Gamma-glutamyl dipeptides	True	7.305e+05	-1.795	-1.795	-2.636
14	UDP-Glucuronate	Carbon	1	G6PDD_Untreated_1_4	G6PDD_Untreated_1	1.0	Polysaccharide biosynthesis	True	5.112e+06	1.012	1.012	2.961
229	N-Formyl-Met	Amino acid	1	G6PDD_Untreated_1_4	G6PDD_Untreated_1	1.0	N-formylated amino acids	True	1.602e+05	-3.984	-3.984	-0.1618
350	3',5'-cAMP	Nucleotide	1	G6PDD_Untreated_1_4	G6PDD_Untreated_1	1.0	Purine derivatives in signaling	True	1.44e+05	-4.137	-4.137	-0.5181
371	CDP	Nucleotide	1	G6PDD_Untreated_1_4	G6PDD_Untreated_1	1.0	Pyrimidine nucleotides	True	2.772e+05	-3.193	-3.193	0.1271
372	CTP	Nucleotide	1	G6PDD_Untreated_1_4	G6PDD_Untreated_1	0.75	Pyrimidine nucleotides	False			-4.713	-1.059
333	GDP	Nucleotide	1	G6PDD_Untreated_1_4	G6PDD_Untreated_1	0.5	Purine nucleotides	True	9.425e+05	-1.427	-1.427	1.073
332	GMP	Nucleotide	1	G6PDD_Untreated_1_4	G6PDD_Untreated_1	1.0	Purine nucleotides	True	3.241e+07	3.676	3.676	2.978
373	UMP	Nucleotide	1	G6PDD_Untreated_1_4	G6PDD_Untreated_1	1.0	Pyrimidine nucleotides	True	1.469e+07	2.535	2.535	3.59
389	3'-CMP	Nucleotide	1	G6PDD_Untreated_1_4	G6PDD_Untreated_1	1.0	Pyrimidine derivatives in signaling	True	5.74e+06	1.179	1.179	2.23
330	ADP	Nucleotide	1	G6PDD_Untreated_1_4	G6PDD_Untreated_1	1.0	Purine nucleotides	True	7.061e+06	1.478	1.478	1.774
342	Hypoxanthine	Nucleotide	1	G6PDD_Untreated_1_4	G6PDD_Untreated_1	1.0	Purine bases	True	1.868e+07	2.881	2.881	-1.058
736	Pyridoxamine-P	Cofactor	1	G6PDD_Untreated_1_4	G6PDD_Untreated_1	1.0	PLP biosynthesis and salvage	True	4.257e+05	-2.574	-2.574	0.4043
148	Betaine	Amino acid	1	G6PDD_Untreated_1_4	G6PDD_Untreated_1	1.0	Amino acid derivatives	True	7.604e+07	4.907	4.907	0.1011
344	Xanthine	Nucleotide	1	G6PDD_Untreated_1_4	G6PDD_Untreated_1	1.0	Purine bases	True	3.447e+06	0.4436	0.4436	-0.4639
386	3-Ureidopropionate	Nucleotide	1	G6PDD_Untreated_1_4	G6PDD_Untreated_1	1.0	Pyrimidine degradation	True	1.471e+06	-0.7852	-0.7852	0.1342
149	DiMe-Gly	Amino acid	1	G6PDD_Untreated_1_4	G6PDD_Untreated_1	1.0	Amino acid derivatives	True	2.009e+06	-0.3353	-0.3353	0.185
703	NAD+	Cofactor	1	G6PDD_Untreated_1_4	G6PDD_Untreated_1	1.0	Cofactors	True	1.757e+07	2.793	2.793	0.0571
709	Pyridoxal-P	Cofactor	1	G6PDD_Untreated_1_4	G6PDD_Untreated_1	1.0	Cofactors	True	5.555e+05	-2.19	-2.19	0.1179

Metabolite ID	Name	Super Pathway	Dataset	Sample ID	Group ID	Detection Fraction	Pathway	Detected	Raw Intensity	Log2 Norm Intensity	Norm Imputed	Log2 Ctrl Norm
731	Thiamin (Vitamin B1)	Cofactor	1	G6PDD_Untreated_1_4	G6PDD_Untreated_1	1.0	TPP biosynthesis	True	4.003e+06	0.659	0.659	-0.0887
374	UDP	Nucleotide	1	G6PDD_Untreated_1_4	G6PDD_Untreated_1	1.0	Pyrimidine nucleotides	True	3.669e+06	0.5336	0.5336	2.087
102	2-Aminoadipate	Amino acid	1	G6PDD_Untreated_1_4	G6PDD_Untreated_1	1.0	Amino acids biosynthesis intermediates	True	8.062e+05	-1.653	-1.653	0.4568
45	Fructose-6-P	Carbon	1	G6PDD_Untreated_1_4	G6PDD_Untreated_1	1.0	Glycolysis, GNG	True	6.654e+06	1.392	1.392	1.317
320	TMP	Nucleotide	1	G6PDD_Untreated_1_4	G6PDD_Untreated_1	0.5	Deoxy-nucleotides	True	6.688e+05	-1.922	-1.922	2.869
341	XMP	Nucleotide	1	G6PDD_Untreated_1_4	G6PDD_Untreated_1	0.25	IMP conversion to AMP & GMP	False			-6.353	-1.778
120	beta-OH-Isovalerate	Amino acid	1	G6PDD_Untreated_1_4	G6PDD_Untreated_1	1.0	Amino acids degradation intermediates	True	1.981e+05	-3.678	-3.678	-0.5126
322	2'-dI	Nucleotide	1	G6PDD_Untreated_1_4	G6PDD_Untreated_1	0.5	Deoxy-nucleosides	True	1.007e+05	-4.653	-4.653	-0.5282
4	GlcNAc 6-P	Carbon	1	G6PDD_Untreated_1_4	G6PDD_Untreated_1	1.0	Aminosugar biosynthesis	True	2.748e+06	0.1165	0.1165	-0.2121
337	Xanthosine	Nucleotide	1	G6PDD_Untreated_1_4	G6PDD_Untreated_1	1.0	Purine nucleosides	True	1.921e+05	-3.722	-3.722	-0.6475
188	Kynurenine	Amino acid	1	G6PDD_Untreated_1_4	G6PDD_Untreated_1	1.0	Amino acid derivatives	True	1.487e+06	-0.7697	-0.7697	-0.07811
63	6-P-Gluconate	Carbon	1	G6PDD_Untreated_1_4	G6PDD_Untreated_1	1.0	Pentose phosphate pathway (PPP)	True	1.59e+07	2.649	2.649	0.6156
40	Glucuronate	Carbon	1	G6PDD_Untreated_1_4	G6PDD_Untreated_1	1.0	Sugars and sugar alcohols	True	1.624e+06	-0.642	-0.642	0.8971
108	Argininosuccinate	Amino acid	1	G6PDD_Untreated_1_4	G6PDD_Untreated_1	1.0	Amino acids biosynthesis intermediates	True	8.195e+05	-1.629	-1.629	-1.403
710	Carnitine	Cofactor	1	G6PDD_Untreated_1_4	G6PDD_Untreated_1	1.0	Cofactors	True	7.13e+07	4.814	4.814	-1.164
725	P-Pantetheine	Cofactor	1	G6PDD_Untreated_1_4	G6PDD_Untreated_1	0.75	Coenzyme A biosynthesis	True	2.58e+05	-3.296	-3.296	0.4084
48	DHAP	Carbon	1	G6PDD_Untreated_1_4	G6PDD_Untreated_1	1.0	Glycolysis, GNG	True	2.388e+07	3.236	3.236	0.1395
17	Maltose	Carbon	1	G6PDD_Untreated_1_4	G6PDD_Untreated_1	1.0	Glycogen degradation	True	5.003e+05	-2.341	-2.341	-1.048
359	N1-Me-Adenosine	Nucleotide	1	G6PDD_Untreated_1_4	G6PDD_Untreated_1	1.0	Purine derivatives in RNAs	True	1.005e+06	-1.335	-1.335	-0.2977
159	3-Me-His	Amino acid	1	G6PDD_Untreated_1_4	G6PDD_Untreated_1	1.0	Amino acid derivatives	True	1.798e+05	-3.817	-3.817	0.4182

Metabolite ID	Name	Super Pathway	Dataset	Sample ID	Group ID	Detection Fraction	Pathway	Detected	Raw Intensity	Log2 Norm Intensity	Norm Imputed	Log2 Ctrl Norm
155	4-Guanidinobutanoate	Amino acid	1	G6PDD_Untreated_1_4	G6PDD_Untreated_1	1.0	Amino acid derivatives	True	1.062e+06	-1.256	-1.256	0.1783
164	5-OH-Lys	Amino acid	1	G6PDD_Untreated_1_4	G6PDD_Untreated_1	1.0	Amino acid derivatives	True	5.383e+05	-2.235	-2.235	-0.465
357	Adenosine-3',5'-PP	Nucleotide	1	G6PDD_Untreated_1_4	G6PDD_Untreated_1	1.0	Purine byproducts of metabolic processes	True	1.622e+05	-3.966	-3.966	-0.4885
104	Cystathionine	Amino acid	1	G6PDD_Untreated_1_4	G6PDD_Untreated_1	1.0	Amino acids biosynthesis intermediates	True	1.746e+07	2.784	2.784	0.5744
113	Imidazole Lactate	Amino acid	1	G6PDD_Untreated_1_4	G6PDD_Untreated_1	1.0	Amino acids degradation intermediates	True	2.469e+05	-3.36	-3.36	0.3165
215	N-Ac-Glu	Amino acid	1	G6PDD_Untreated_1_4	G6PDD_Untreated_1	1.0	N-acetylated amino acids	True	3.279e+06	0.3716	0.3716	-1.372
310	S-Lactoyl-Glutathione	Amino acid	1	G6PDD_Untreated_1_4	G6PDD_Untreated_1	1.0	Glutathione derivatives	True	3.794e+06	0.5817	0.5817	0.2253
5	GlcNAc 1-P	Carbon	1	G6PDD_Untreated_1_4	G6PDD_Untreated_1	1.0	Aminosugar biosynthesis	True	9.044e+05	-1.487	-1.487	0.424
34	Ribitol	Carbon	1	G6PDD_Untreated_1_4	G6PDD_Untreated_1	1.0	Sugars and sugar alcohols	True	2.841e+05	-3.158	-3.158	0.2724
10	UDP-Galactose	Carbon	1	G6PDD_Untreated_1_4	G6PDD_Untreated_1	0.75	Polysaccharide biosynthesis	False			-1.452	-0.8254
13	Guanosine 5'-PP-Fucose	Carbon	1	G6PDD_Untreated_1_4	G6PDD_Untreated_1	1.0	Polysaccharide biosynthesis	True	6.861e+05	-1.885	-1.885	0.4139
19	Maltotetraose	Carbon	1	G6PDD_Untreated_1_4	G6PDD_Untreated_1	1.0	Glycogen degradation	True	2.201e+06	-0.2039	-0.2039	-0.1602
233	SAM	Amino acid	1	G6PDD_Untreated_1_4	G6PDD_Untreated_1	1.0	SAM metabolism	True	2.784e+06	0.135	0.135	0.2913
129	5-Aminovalerate	Amino acid	1	G6PDD_Untreated_1_4	G6PDD_Untreated_1	1.0	Amino acids degradation intermediates	True	2.609e+06	0.04171	0.04171	-0.96
741	5-Me-THF	Cofactor	1	G6PDD_Untreated_1_4	G6PDD_Untreated_1	1.0	Folate metabolism	True	1.237e+05	-4.357	-4.357	0.4004
198	Indolelactate	Amino acid	1	G6PDD_Untreated_1_4	G6PDD_Untreated_1	1.0	Amino acid derivatives	True	1.864e+05	-3.766	-3.766	0.02139
254	Gly-Val	Amino acid	1	G6PDD_Untreated_1_4	G6PDD_Untreated_1	1.0	Dipeptides	True	2.242e+06	-0.177	-0.177	-1.288
291	gamma-Glu-Leu	Amino acid	1	G6PDD_Untreated_1_4	G6PDD_Untreated_1	1.0	Gamma-glutamyl dipeptides	True	8.068e+05	-1.652	-1.652	-1.163
173	Met Sulfoxide	Amino acid	1	G6PDD_Untreated_1_4	G6PDD_Untreated_1	1.0	Amino acid derivatives	True	9.282e+06	1.872	1.872	-0.6949

Metabolite ID	Name	Super Pathway	Dataset	Sample ID	Group ID	Detection Fraction	Pathway	Detected	Raw Intensity	Log2 Norm Intensity	Norm Imputed	Log2 Ctrl Norm
43	Glucose	Carbon	1	G6PDD_Untreated_1_4	G6PDD_Untreated_1	1.0	Glycolysis, GNG	True	8.481e+07	5.064	5.064	0.01271
185	Phenyllactate	Amino acid	1	G6PDD_Untreated_1_4	G6PDD_Untreated_1	0.5	Amino acid derivatives	True	6.614e+04	-5.26	-5.26	0.5515
156	Homo-Arg	Amino acid	1	G6PDD_Untreated_1_4	G6PDD_Untreated_1	1.0	Amino acid derivatives	True	4.057e+06	0.6785	0.6785	-0.502
135	Homocitrulline	Amino acid	1	G6PDD_Untreated_1_4	G6PDD_Untreated_1	1.0	Amino acids degradation intermediates	True	5.59e+05	-2.181	-2.181	-0.4785
719	Nicotinamide MN	Cofactor	1	G6PDD_Untreated_1_4	G6PDD_Untreated_1	1.0	NAD biosynthesis	True	8.155e+05	-1.636	-1.636	-1.822
212	N-Ac-Asp	Amino acid	1	G6PDD_Untreated_1_4	G6PDD_Untreated_1	1.0	N-acetylated amino acids	True	4.108e+06	0.6965	0.6965	-8.78e-04
720	1-Me-Nicotinamide	Cofactor	1	G6PDD_Untreated_1_4	G6PDD_Untreated_1	1.0	Derivatives of NA, nicotinamide and NAD	True	6.894e+08	8.087	8.087	0.1966
216	N-Ac-Gly	Amino acid	1	G6PDD_Untreated_1_4	G6PDD_Untreated_1	1.0	N-acetylated amino acids	True	1.658e+05	-3.934	-3.934	-0.3941
70	Creatine	Carbon	1	G6PDD_Untreated_1_4	G6PDD_Untreated_1	1.0	Creatine energy storage	True	1.549e+09	9.255	9.255	-0.1617
26	Galactonate	Carbon	1	G6PDD_Untreated_1_4	G6PDD_Untreated_1	1.0	Sugars and sugar alcohols	True	4.285e+06	0.7573	0.7573	1.562
309	Glutathione, Oxidized	Amino acid	1	G6PDD_Untreated_1_4	G6PDD_Untreated_1	1.0	Glutathione	True	4.349e+06	0.7787	0.7787	-0.414
35	Ribonate	Carbon	1	G6PDD_Untreated_1_4	G6PDD_Untreated_1	1.0	Sugars and sugar alcohols	True	2.093e+06	-0.2762	-0.2762	0.251
160	1-Me-His	Amino acid	1	G6PDD_Untreated_1_4	G6PDD_Untreated_1	1.0	Amino acid derivatives	True	2.832e+07	3.482	3.482	0.04091
44	Glucose 6-P	Carbon	1	G6PDD_Untreated_1_4	G6PDD_Untreated_1	1.0	Glycolysis, GNG	True	2.903e+06	0.1956	0.1956	1.672
704	NADH	Cofactor	1	G6PDD_Untreated_1_4	G6PDD_Untreated_1	1.0	Cofactors	True	3.708e+06	0.5487	0.5487	1.195
275	Thr-Phe	Amino acid	1	G6PDD_Untreated_1_4	G6PDD_Untreated_1	1.0	Dipeptides	True	1.555e+06	-0.7054	-0.7054	0.9478
738	Pyridoxate	Cofactor	1	G6PDD_Untreated_1_4	G6PDD_Untreated_1	1.0	PLP biosynthesis and salvage	True	3.329e+05	-2.929	-2.929	0.4356
177	3-(4-OH-Phenyl)Lactate	Amino acid	1	G6PDD_Untreated_1_4	G6PDD_Untreated_1	1.0	Amino acid derivatives	True	6.047e+05	-2.068	-2.068	0.3977
206	Trans-4-OH-Pro	Amino acid	1	G6PDD_Untreated_1_4	G6PDD_Untreated_1	1.0	Amino acid derivatives	True	9.659e+07	5.252	5.252	0.09668
329	AMP	Nucleotide	1	G6PDD_Untreated_1_4	G6PDD_Untreated_1	1.0	Purine nucleotides	True	1.766e+08	6.123	6.123	2.487

Metabolite ID	Name	Super Pathway	Dataset	Sample ID	Group ID	Detection Fraction	Pathway	Detected	Raw Intensity	Log2 Norm Intensity	Norm Imputed	Log2 Ctrl Norm
11	UDP-Glucose	Carbon	1	G6PDD_Untreated_1_4	G6PDD_Untreated_1	1.0	Polysaccharide biosynthesis	True	9.062e+06	1.838	1.838	1.943
158	4-Imidazole-Ac	Amino acid	1	G6PDD_Untreated_1_4	G6PDD_Untreated_1	1.0	Amino acid derivatives	True	3.57e+05	-2.828	-2.828	-0.6813
111	1-Me-Imidazole-Ac	Amino acid	1	G6PDD_Untreated_1_4	G6PDD_Untreated_1	1.0	Amino acids degradation intermediates	True	7.108e+05	-1.834	-1.834	0.09944
345	Guanine	Nucleotide	1	G6PDD_Untreated_1_4	G6PDD_Untreated_1	1.0	Purine bases	True	4.707e+07	4.215	4.215	-0.7715
22	N-Ac-Neuraminate	Carbon	1	G6PDD_Untreated_1_4	G6PDD_Untreated_1	1.0	Aminosugar derivatives	True	3.109e+06	0.2947	0.2947	0.4301
721	N'-Methylnicotinate	Cofactor	1	G6PDD_Untreated_1_4	G6PDD_Untreated_1	1.0	Derivatives of NA, nicotinamide and NAD	True	8.303e+05	-1.61	-1.61	-0.6025
183	Phenol Sulfate	Amino acid	1	G6PDD_Untreated_1_4	G6PDD_Untreated_1	1.0	Amino acid derivatives	True	1.155e+05	-4.456	-4.456	0.1839
718	Nicotinamide Riboside	Cofactor	1	G6PDD_Untreated_1_4	G6PDD_Untreated_1	1.0	NAD biosynthesis	True	5.098e+05	-2.314	-2.314	-3.904
297	gamma-Glu-Thr	Amino acid	1	G6PDD_Untreated_1_4	G6PDD_Untreated_1	1.0	Gamma-glutamyl dipeptides	True	9.739e+05	-1.38	-1.38	-2.02
295	gamma-Glu-Phe	Amino acid	1	G6PDD_Untreated_1_4	G6PDD_Untreated_1	0.75	Gamma-glutamyl dipeptides	False			-5.975	-0.1899
347	Allantoic Acid	Nucleotide	1	G6PDD_Untreated_1_4	G6PDD_Untreated_1	0.5	Purine degradation	False			-6.474	-0.8345
399	Pseudouridine	Nucleotide	1	G6PDD_Untreated_1_4	G6PDD_Untreated_1	1.0	Pyrimidine derivatives in RNAs	True	5.772e+05	-2.135	-2.135	-0.1048
375	UTP	Nucleotide	1	G6PDD_Untreated_1_4	G6PDD_Untreated_1	1.0	Pyrimidine nucleotides	True	9.046e+05	-1.487	-1.487	0.4894
144	Glu, gamma-Me Ester	Amino acid	1	G6PDD_Untreated_1_4	G6PDD_Untreated_1	1.0	Amino acid derivatives	True	2.855e+06	0.1718	0.1718	-0.406
292	gamma-Glu-epsilon-Lysine	Amino acid	1	G6PDD_Untreated_1_4	G6PDD_Untreated_1	1.0	Gamma-glutamyl dipeptides	True	1.512e+06	-0.7459	-0.7459	-0.5546
225	N-Ac-Thr	Amino acid	1	G6PDD_Untreated_1_4	G6PDD_Untreated_1	1.0	N-acetylated amino acids	True	5.058e+05	-2.325	-2.325	-0.8228
211	N-Ac-Asn	Amino acid	1	G6PDD_Untreated_1_4	G6PDD_Untreated_1	1.0	N-acetylated amino acids	True	4.539e+05	-2.481	-2.481	0.2038
151	Phenylacetylglycine	Amino acid	1	G6PDD_Untreated_1_4	G6PDD_Untreated_1	1.0	Amino acid derivatives	True	4.505e+06	0.8296	0.8296	1.617
217	N-Ac-His	Amino acid	1	G6PDD_Untreated_1_4	G6PDD_Untreated_1	1.0	N-acetylated amino acids	True	1.139e+05	-4.477	-4.477	-0.9917
288	gamma-Glu-Gly	Amino acid	1	G6PDD_Untreated_1_4	G6PDD_Untreated_1	0.75	Gamma-glutamyl dipeptides	False			-3.092	-1.268

Metabolite ID	Name	Super Pathway	Dataset	Sample ID	Group ID	Detection Fraction	Pathway	Detected	Raw Intensity	Log2 Norm Intensity	Norm Imputed	Log2 Ctrl Norm
222	N-Ac-Phe	Amino acid	1	G6PDD_Untreated_1_4	G6PDD_Untreated_1	1.0	N-acetylated amino acids	True	3.681e+04	-6.106	-6.106	0.1668
71	Creatine-P	Carbon	1	G6PDD_Untreated_1_4	G6PDD_Untreated_1	1.0	Creatine energy storage	True	6.087e+04	-5.38	-5.38	-1.125
210	N-Ac-Arg	Amino acid	1	G6PDD_Untreated_1_4	G6PDD_Untreated_1	0.75	N-acetylated amino acids	False			-4.025	-1.35
218	N-Ac-Ile	Amino acid	1	G6PDD_Untreated_1_4	G6PDD_Untreated_1	0.5	N-acetylated amino acids	False			-6.52	-0.5541
251	Gly-Leu	Amino acid	1	G6PDD_Untreated_1_4	G6PDD_Untreated_1	1.0	Dipeptides	True	4.874e+06	0.9431	0.9431	-0.6956
290	gamma-Glu-Ile	Amino acid	1	G6PDD_Untreated_1_4	G6PDD_Untreated_1	1.0	Gamma-glutamyl dipeptides	True	2.598e+05	-3.287	-3.287	-1.948
316	Ophthalmate	Amino acid	1	G6PDD_Untreated_1_4	G6PDD_Untreated_1	1.0	Oxidative stress markers	True	1.992e+06	-0.3475	-0.3475	-2.69
125	Isovaleryl-Gly	Amino acid	1	G6PDD_Untreated_1_4	G6PDD_Untreated_1	1.0	Amino acids degradation intermediates	True	6.246e+04	-5.343	-5.343	0.06669
368	7-Me-Guanine	Nucleotide	1	G6PDD_Untreated_1_4	G6PDD_Untreated_1	1.0	Purine derivatives in RNAs	True	5.159e+05	-2.297	-2.297	-0.1067
208	Pro-OH-Pro	Amino acid	1	G6PDD_Untreated_1_4	G6PDD_Untreated_1	1.0	Amino acid derivatives	True	3.64e+07	3.844	3.844	0.5147
366	N2,N2-DiMe-Guanosine	Nucleotide	1	G6PDD_Untreated_1_4	G6PDD_Untreated_1	1.0	Purine derivatives in RNAs	True	1.248e+05	-4.344	-4.344	-0.2308
352	3'-AMP	Nucleotide	1	G6PDD_Untreated_1_4	G6PDD_Untreated_1	1.0	Purine derivatives in signaling	True	2.087e+06	-0.2802	-0.2802	1.207
363	N6-Carbamoyl-Thr-Adenosine	Nucleotide	1	G6PDD_Untreated_1_4	G6PDD_Untreated_1	1.0	Purine derivatives in RNAs	True	5.207e+04	-5.605	-5.605	-0.92
314	Cys-Glutathione Disulfide	Amino acid	1	G6PDD_Untreated_1_4	G6PDD_Untreated_1	0.25	Oxidative stress markers	False			-5.284	-2
382	Orotidine	Nucleotide	1	G6PDD_Untreated_1_4	G6PDD_Untreated_1	1.0	Pyrimidine (UMP) biosynthesis	True	6.492e+04	-5.287	-5.287	-0.9952
307	Cys-Gly	Amino acid	1	G6PDD_Untreated_1_4	G6PDD_Untreated_1	1.0	Glutathione biosynthesis	True	6.822e+06	1.428	1.428	-0.1788
64	Sedoheptulose-7-P	Carbon	1	G6PDD_Untreated_1_4	G6PDD_Untreated_1	1.0	Pentose phosphate pathway (PPP)	True	2.322e+06	-0.1263	-0.1263	-0.2804
142	N-Ac-Asp-Glu	Amino acid	1	G6PDD_Untreated_1_4	G6PDD_Untreated_1	1.0	Amino acid derivatives	True	5.464e+05	-2.214	-2.214	-1.069
708	Thiamin-PP	Cofactor	1	G6PDD_Untreated_1_4	G6PDD_Untreated_1	1.0	Cofactors	True	4.013e+04	-5.981	-5.981	2.45e-03
182	P-Cresol Sulfate	Amino acid	1	G6PDD_Untreated_1_4	G6PDD_Untreated_1	1.0	Amino acid derivatives	True	2.998e+05	-3.08	-3.08	0.1161

Metabolite ID	Name	Super Pathway	Dataset	Sample ID	Group ID	Detection Fraction	Pathway	Detected	Raw Intensity	Log2 Norm Intensity	Norm Imputed	Log2 Ctrl Norm
250	Gly-Ile	Amino acid	1	G6PDD_Untreated_1_4	G6PDD_Untreated_1	1.0	Dipeptides	True	8.659e+05	-1.55	-1.55	-0.2324
286	gamma-Glu-Glu	Amino acid	1	G6PDD_Untreated_1_4	G6PDD_Untreated_1	1.0	Gamma-glutamyl dipeptides	True	2.762e+06	0.1236	0.1236	0.1058
739	Deoxycarnitine	Cofactor	1	G6PDD_Untreated_1_4	G6PDD_Untreated_1	1.0	Carnitine biosynthesis	True	1.782e+07	2.814	2.814	-0.9374
203	DiMe-Arg	Amino acid	1	G6PDD_Untreated_1_4	G6PDD_Untreated_1	1.0	Amino acid derivatives	True	3.166e+07	3.643	3.643	-1.99
351	2'-AMP	Nucleotide	1	G6PDD_Untreated_1_4	G6PDD_Untreated_1	1.0	Purine derivatives in signaling	True	1.71e+05	-3.89	-3.89	-1.621
8	Cytidine 5'-P-N-Ac-Neuraminate	Carbon	1	G6PDD_Untreated_1_4	G6PDD_Untreated_1	1.0	Aminosugar biosynthesis	True	9.103e+05	-1.478	-1.478	0.2679
285	gamma-Glu-Ala	Amino acid	1	G6PDD_Untreated_1_4	G6PDD_Untreated_1	1.0	Gamma-glutamyl dipeptides	True	7.088e+04	-5.16	-5.16	-1.922
224	N-Ac-Ser	Amino acid	1	G6PDD_Untreated_1_4	G6PDD_Untreated_1	1.0	N-acetylated amino acids	True	6.197e+06	1.29	1.29	-0.5895
244	Ala-Leu	Amino acid	1	G6PDD_Untreated_1_4	G6PDD_Untreated_1	1.0	Dipeptides	True	8.116e+06	1.679	1.679	0.5409
207	N-Me-Pro	Amino acid	1	G6PDD_Untreated_1_4	G6PDD_Untreated_1	1.0	Amino acid derivatives	True	1.242e+06	-1.03	-1.03	-0.09954
171	Cys Sulfinic Acid	Amino acid	1	G6PDD_Untreated_1_4	G6PDD_Untreated_1	0.75	Amino acid derivatives	True	3.112e+05	-3.026	-3.026	0.2714
181	O-Me-Tyr	Amino acid	1	G6PDD_Untreated_1_4	G6PDD_Untreated_1	1.0	Amino acid derivatives	True	3.512e+05	-2.852	-2.852	0.5182
240	N-Ac-Putrescine	Amino acid	1	G6PDD_Untreated_1_4	G6PDD_Untreated_1	1.0	Polyamine derivatives	True	2.227e+06	-0.1867	-0.1867	2.177
176	S-Me-Met	Amino acid	1	G6PDD_Untreated_1_4	G6PDD_Untreated_1	1.0	Amino acid derivatives	True	6.505e+05	-1.962	-1.962	-0.1206
339	AICAR	Nucleotide	1	G6PDD_Untreated_1_4	G6PDD_Untreated_1	1.0	IMP biosynthesis	True	1.417e+05	-4.161	-4.161	0.14
141	gamma-Carboxy-Glu	Amino acid	1	G6PDD_Untreated_1_4	G6PDD_Untreated_1	1.0	Amino acid derivatives	True	1.338e+06	-0.9216	-0.9216	-0.1366
392	3'-UMP	Nucleotide	1	G6PDD_Untreated_1_4	G6PDD_Untreated_1	0.75	Pyrimidine derivatives in signaling	True	1.484e+06	-0.7726	-0.7726	3.261
355	3'-GMP	Nucleotide	1	G6PDD_Untreated_1_4	G6PDD_Untreated_1	1.0	Purine derivatives in signaling	True	1.238e+06	-1.033	-1.033	2.904
282	Val-Leu	Amino acid	1	G6PDD_Untreated_1_4	G6PDD_Untreated_1	1.0	Dipeptides	True	8.065e+06	1.67	1.67	0.698
140	Carboxyethyl-GABA	Amino acid	1	G6PDD_Untreated_1_4	G6PDD_Untreated_1	1.0	Amino acid derivatives	True	7.364e+05	-1.783	-1.783	-0.3523
258	Ile-Gly	Amino acid	1	G6PDD_Untreated_1_4	G6PDD_Untreated_1	1.0	Dipeptides	True	2.07e+07	3.03	3.03	-0.2821

Metabolite ID	Name	Super Pathway	Dataset	Sample ID	Group ID	Detection Fraction	Pathway	Detected	Raw Intensity	Log2 Norm Intensity	Norm Imputed	Log2 Ctrl Norm
260	Leu-Ala	Amino acid	1	G6PDD_Untreated_1_4	G6PDD_Untreated_1	1.0	Dipeptides	True	1.008e+07	1.992	1.992	0.691
265	Lys-Leu	Amino acid	1	G6PDD_Untreated_1_4	G6PDD_Untreated_1	1.0	Dipeptides	True	5.759e+05	-2.138	-2.138	0.4732
263	Leu-Gly	Amino acid	1	G6PDD_Untreated_1_4	G6PDD_Untreated_1	1.0	Dipeptides	True	4.006e+07	3.982	3.982	0.9608
281	Val-Gly	Amino acid	1	G6PDD_Untreated_1_4	G6PDD_Untreated_1	1.0	Dipeptides	True	4.54e+07	4.163	4.163	-0.3565
270	Pro-Gly	Amino acid	1	G6PDD_Untreated_1_4	G6PDD_Untreated_1	1.0	Dipeptides	True	2.135e+06	-0.248	-0.248	-0.9947
114	Imidazole Propionate	Amino acid	1	G6PDD_Untreated_1_4	G6PDD_Untreated_1	1.0	Amino acids degradation intermediates	True	1.299e+06	-0.9646	-0.9646	1.346
267	Phe-Gly	Amino acid	1	G6PDD_Untreated_1_4	G6PDD_Untreated_1	1.0	Dipeptides	True	2.34e+07	3.207	3.207	-0.8872
266	Phe-Ala	Amino acid	1	G6PDD_Untreated_1_4	G6PDD_Untreated_1	1.0	Dipeptides	True	6.572e+06	1.374	1.374	0.2976
278	Tyr-Gly	Amino acid	1	G6PDD_Untreated_1_4	G6PDD_Untreated_1	1.0	Dipeptides	True	9.299e+06	1.875	1.875	-0.3071
255	His-Ala	Amino acid	1	G6PDD_Untreated_1_4	G6PDD_Untreated_1	1.0	Dipeptides	True	8.536e+05	-1.57	-1.57	-0.2329
280	Val-Gln	Amino acid	1	G6PDD_Untreated_1_4	G6PDD_Untreated_1	1.0	Dipeptides	True	9.42e+06	1.894	1.894	0.2757
143	S-1-Pyrroline-5-Carboxylate	Amino acid	1	G6PDD_Untreated_1_4	G6PDD_Untreated_1	1.0	Amino acid derivatives	True	9.123e+05	-1.474	-1.474	0.5938
232	SAH	Amino acid	1	G6PDD_Untreated_1_4	G6PDD_Untreated_1	1.0	SAM metabolism	True	1.739e+06	-0.5439	-0.5439	-0.05846
20	Erythronate	Carbon	1	G6PDD_Untreated_1_4	G6PDD_Untreated_1	1.0	Aminosugar derivatives	True	3.304e+07	3.704	3.704	-0.03694
248	Gln-Leu	Amino acid	1	G6PDD_Untreated_1_4	G6PDD_Untreated_1	1.0	Dipeptides	True	1.604e+06	-0.6598	-0.6598	0.2454
276	Trp-Gly	Amino acid	1	G6PDD_Untreated_1_4	G6PDD_Untreated_1	1.0	Dipeptides	True	7.919e+05	-1.679	-1.679	-0.6002
205	N-delta-Ac-Ornithine	Amino acid	1	G6PDD_Untreated_1_4	G6PDD_Untreated_1	1.0	Amino acid derivatives	True	8.623e+05	-1.556	-1.556	-0.4346
163	Formimino-Glu	Amino acid	1	G6PDD_Untreated_1_4	G6PDD_Untreated_1	1.0	Amino acid derivatives	True	1.139e+06	-1.154	-1.154	0.08604
204	N-Me-Arg	Amino acid	1	G6PDD_Untreated_1_4	G6PDD_Untreated_1	1.0	Amino acid derivatives	True	4.772e+06	0.9126	0.9126	-1.674
242	Guanidino-Ac	Amino acid	1	G6PDD_Untreated_1_4	G6PDD_Untreated_1	0.75	Creatine biosynthesis	True	1.93e+05	-3.715	-3.715	0.1653

Metabolite ID	Name	Super Pathway	Dataset	Sample ID	Group ID	Detection Fraction	Pathway	Detected	Raw Intensity	Log2 Norm Intensity	Norm Imputed	Log2 Ctrl Norm
300	gamma-Glu-Val	Amino acid	1	G6PDD_Untreated_1_4	G6PDD_Untreated_1	0.75	Gamma-glutamyl dipeptides	False			-0.1053	-0.726
53	Ac-CoA	Carbon	1	G6PDD_Untreated_1_4	G6PDD_Untreated_1	0.5	Acetyl-CoA	True	8.825e+04	-4.844	-4.844	1.918
18	Maltotriose	Carbon	1	G6PDD_Untreated_1_4	G6PDD_Untreated_1	1.0	Glycogen degradation	True	1.396e+06	-0.8608	-0.8608	-0.703
294	gamma-Glu-Met	Amino acid	1	G6PDD_Untreated_1_4	G6PDD_Untreated_1	0.5	Gamma-glutamyl dipeptides	False			-4.503	-1.021
174	Met Sulfone	Amino acid	1	G6PDD_Untreated_1_4	G6PDD_Untreated_1	1.0	Amino acid derivatives	True	2.494e+05	-3.345	-3.345	-0.2666
175	N-Ac-Met Sulfoxide	Amino acid	1	G6PDD_Untreated_1_4	G6PDD_Untreated_1	1.0	Amino acid derivatives	True	1.977e+06	-0.3583	-0.3583	-0.3346
25	Mannitol/Sorbitol	Carbon	1	G6PDD_Untreated_1_4	G6PDD_Untreated_1	1.0	Sugars and sugar alcohols	True	1.355e+07	2.418	2.418	0.1936
6	UDP-GlcNAc	Carbon	1	G6PDD_Untreated_1_4	G6PDD_Untreated_1	0.5	Aminosugar biosynthesis	True	2.106e+05	-3.589	-3.589	-0.4814
145	Pyro-Gln	Amino acid	1	G6PDD_Untreated_1_4	G6PDD_Untreated_1	1.0	Amino acid derivatives	True	3.832e+06	0.5961	0.5961	-0.2437
705	Coenzyme A	Cofactor	1	G6PDD_Untreated_1_4	G6PDD_Untreated_1	1.0	Cofactors	True	3.507e+05	-2.854	-2.854	1.105
319	2'-dAMP	Nucleotide	1	G6PDD_Untreated_1_4	G6PDD_Untreated_1	0.75	Deoxy-nucleotides	True	4.072e+05	-2.638	-2.638	1.042
119	alpha-OH-Isovalerate	Amino acid	1	G6PDD_Untreated_1_4	G6PDD_Untreated_1	0.5	Amino acids degradation intermediates	False			-5.298	-1.999
46	Fructose 1,6-PP / Glucose 1,6-PP / Inositol-1,4-PP / Inositol-1,3-PP	Carbon	1	G6PDD_Untreated_1_4	G6PDD_Untreated_1	1.0	Glycolysis, GNG	True	1.006e+08	5.31	5.31	0.4793
137	1-Me-Guanidine	Amino acid	1	G6PDD_Untreated_1_4	G6PDD_Untreated_1	1.0	Amino acids degradation intermediates	True	6.313e+04	-5.327	-5.327	-0.1747
23	N-GlcNAc-Asn	Carbon	1	G6PDD_Untreated_1_4	G6PDD_Untreated_1	1.0	Aminosugar derivatives	True	2.947e+06	0.2175	0.2175	-0.4954
262	Leu-Gln	Amino acid	1	G6PDD_Untreated_1_4	G6PDD_Untreated_1	1.0	Dipeptides	True	9.247e+06	1.867	1.867	0.5708
24	Fructose	Carbon	1	G6PDD_Untreated_1_4	G6PDD_Untreated_1	1.0	Sugars and sugar alcohols	True	1.055e+07	2.058	2.058	0.1088
197	C-Glycosyl-Trp	Amino acid	1	G6PDD_Untreated_1_4	G6PDD_Untreated_1	1.0	Amino acid derivatives	True	2.753e+06	0.1193	0.1193	0.4183
33	Arabitol/Xylitol	Carbon	1	G6PDD_Untreated_1_4	G6PDD_Untreated_1	1.0	Sugars and sugar alcohols	True	4.131e+05	-2.617	-2.617	-0.7575

Metabolite ID	Name	Super Pathway	Dataset	Sample ID	Group ID	Detection Fraction	Pathway	Detected	Raw Intensity	Log2 Norm Intensity	Norm Imputed	Log2 Ctrl Norm
128	N2-Ac-Lys/N6-Ac-Lys	Amino acid	1	G6PDD_Untreated_1_4	G6PDD_Untreated_1	1.0	Amino acids degradation intermediates	True	1.56e+06	-0.7002	-0.7002	-1.911
42	2-Me-Citrate	Carbon	1	G6PDD_Untreated_1_4	G6PDD_Untreated_1	1.0	Propionate metabolism	True	1.484e+05	-4.094	-4.094	0.2891
12	Glucuronate 1-P	Carbon	1	G6PDD_Untreated_1_4	G6PDD_Untreated_1	1.0	Polysaccharide biosynthesis	True	9.729e+05	-1.382	-1.382	-0.1387
