# Supplementary material for: Targeting the FNIP2-SERCA2b axis improves metabolic and mitochondrial defects in Ataxia Telangiectasia
Source: Cell Death Dis. 2026 Mar 2;17(1):290. doi: 10.1038/s41419-026-08507-5 (PMC13031930; doi:10.1038/s41419-026-08507-5)
Supplement: Supplementary file 3 — Dataset S2 Metabolomic Statistics [file 41419_2026_8507_MOESM3_ESM.pdf]

Table S4: Metabolomics Statistics Summary

Metabolite ID	Name	Comparison	Group Numerator	Group Denominator	Log2 FC	Avg Expression	t	p-value	BH adj p-value	B	-Log10 BH p	Signif.	Direction
19	Maltotetraose	AT_1	AT_Untreated_1	Ctrl_Untreated_1	2.971	1.371	4.089	0.0010	0.1105	-0.5931	0.9565	False	ns
18	Maltotriose	AT_1	AT_Untreated_1	Ctrl_Untreated_1	2.627	1.212	4.082	0.0010	0.1105	-0.6063	0.9565	False	ns
229	N-Formyl-Met	AT_1	AT_Untreated_1	Ctrl_Untreated_1	-1.254	-0.5789	-3.943	0.0014	0.1105	-0.8629	0.9565	False	ns
189	Kynurenate	AT_1	AT_Untreated_1	Ctrl_Untreated_1	1.328	0.6129	3.697	0.0023	0.1214	-1.320	0.9156	False	ns
120	beta-OH-Isovalerate	AT_1	AT_Untreated_1	Ctrl_Untreated_1	1.645	0.7593	3.536	0.0031	0.1214	-1.621	0.9156	False	ns
703	NAD+	AT_1	AT_Untreated_1	Ctrl_Untreated_1	-1.429	-0.6598	-3.462	0.0036	0.1214	-1.759	0.9156	False	ns
69	3-OH-Butyrate	AT_1	AT_Untreated_1	Ctrl_Untreated_1	1.633	0.7537	3.366	0.0044	0.1214	-1.938	0.9156	False	ns
306	gamma-Glu-Cys	AT_1	AT_Untreated_1	Ctrl_Untreated_1	-1.395	-0.6437	-3.302	0.0050	0.1214	-2.057	0.9156	False	ns
43	Glucose	AT_1	AT_Untreated_1	Ctrl_Untreated_1	1.159	0.5351	3.255	0.0055	0.1214	-2.144	0.9156	False	ns
346	Urate	AT_1	AT_Untreated_1	Ctrl_Untreated_1	1.191	0.5499	3.225	0.0059	0.1214	-2.199	0.9156	False	ns
343	Adenine	AT_1	AT_Untreated_1	Ctrl_Untreated_1	-1.071	-0.4942	-3.205	0.0061	0.1214	-2.237	0.9156	False	ns
234	5-Me-Thioadenosine	AT_1	AT_Untreated_1	Ctrl_Untreated_1	-1.401	-0.6465	-3.167	0.0066	0.1214	-2.306	0.9156	False	ns
182	P-Cresol Sulfate	AT_1	AT_Untreated_1	Ctrl_Untreated_1	1.156	0.5337	3.133	0.0071	0.1214	-2.370	0.9156	False	ns
125	Isovaleryl-Gly	AT_1	AT_Untreated_1	Ctrl_Untreated_1	0.8841	0.4081	3.106	0.0075	0.1214	-2.421	0.9156	False	ns
224	N-Ac-Ser	AT_1	AT_Untreated_1	Ctrl_Untreated_1	-1.067	-0.4923	-3.098	0.0076	0.1214	-2.435	0.9156	False	ns
316	Ophthalmate	AT_1	AT_Untreated_1	Ctrl_Untreated_1	-1.617	-0.7464	-2.918	0.0109	0.1451	-2.766	0.8383	False	ns
242	Guanidino-Ac	AT_1	AT_Untreated_1	Ctrl_Untreated_1	1.244	0.5741	2.918	0.0109	0.1451	-2.766	0.8383	False	ns
198	Indolelactate	AT_1	AT_Untreated_1	Ctrl_Untreated_1	1.117	0.5156	2.911	0.0110	0.1451	-2.778	0.8383	False	ns
337	Xanthosine	AT_1	AT_Untreated_1	Ctrl_Untreated_1	1.273	0.5875	2.876	0.0119	0.1451	-2.842	0.8383	False	ns
739	Deoxycarnitine	AT_1	AT_Untreated_1	Ctrl_Untreated_1	-1.006	-0.4641	-2.847	0.0126	0.1451	-2.896	0.8383	False	ns
383	Cytosine	AT_1	AT_Untreated_1	Ctrl_Untreated_1	1.342	0.6196	2.842	0.0127	0.1451	-2.904	0.8383	False	ns
55	Citrate	AT_1	AT_Untreated_1	Ctrl_Untreated_1	0.8855	0.4087	2.802	0.0138	0.1501	-2.978	0.8236	False	ns
142	N-Ac-Asp-Glu	AT_1	AT_Untreated_1	Ctrl_Untreated_1	-0.9156	-0.4226	-2.773	0.0146	0.1520	-3.030	0.8180	False	ns
104	Cystathionine	AT_1	AT_Untreated_1	Ctrl_Untreated_1	-1.062	-0.4901	-2.708	0.0166	0.1585	-3.147	0.7999	False	ns
188	Kynurenine	AT_1	AT_Untreated_1	Ctrl_Untreated_1	2.267	1.046	2.670	0.0179	0.1585	-3.215	0.7999	False	ns
225	N-Ac-Thr	AT_1	AT_Untreated_1	Ctrl_Untreated_1	-0.8732	-0.4030	-2.663	0.0181	0.1585	-3.227	0.7999	False	ns
17	Maltose	AT_1	AT_Untreated_1	Ctrl_Untreated_1	1.438	0.6638	2.655	0.0184	0.1585	-3.240	0.7999	False	ns
24	Fructose	AT_1	AT_Untreated_1	Ctrl_Untreated_1	1.265	0.5841	2.646	0.0187	0.1585	-3.257	0.7999	False	ns
25	Mannitol/Sorbitol	AT_1	AT_Untreated_1	Ctrl_Untreated_1	1.010	0.4662	2.635	0.0192	0.1585	-3.277	0.7999	False	ns

Metabolite ID	Name	Comparison	Group Numerator	Group Denominator	Log2 FC	Avg Expression	t	p-value	BH adj p-value	B	-Log10 BH p	Signif.	Direction
45	Fructose-6-P	AT_1	AT_Untreated_1	Ctrl_Untreated_1	-1.310	-0.6046	-2.612	0.0200	0.1603	-3.318	0.7950	False	ns
215	N-Ac-Glu	AT_1	AT_Untreated_1	Ctrl_Untreated_1	-1.053	-0.4858	-2.546	0.0228	0.1719	-3.435	0.7647	False	ns
177	3-(4-OH-Phenyl)Lactate	AT_1	AT_Untreated_1	Ctrl_Untreated_1	1.195	0.5515	2.540	0.0231	0.1719	-3.445	0.7647	False	ns
725	P-Pantetheine	AT_1	AT_Untreated_1	Ctrl_Untreated_1	1.247	0.5756	2.528	0.0236	0.1719	-3.466	0.7647	False	ns
196	5-OH-Indole-Ac	AT_1	AT_Untreated_1	Ctrl_Untreated_1	1.151	0.5311	2.462	0.0269	0.1827	-3.581	0.7384	False	ns
290	gamma-Glu-Ile	AT_1	AT_Untreated_1	Ctrl_Untreated_1	-1.181	-0.5453	-2.462	0.0269	0.1827	-3.582	0.7384	False	ns
108	Argininosuccinate	AT_1	AT_Untreated_1	Ctrl_Untreated_1	-1.194	-0.5510	-2.452	0.0274	0.1827	-3.598	0.7384	False	ns
241	4-Acetamidobutanoate	AT_1	AT_Untreated_1	Ctrl_Untreated_1	0.9755	0.4502	2.377	0.0317	0.2013	-3.729	0.6962	False	ns
291	gamma-Glu-Leu	AT_1	AT_Untreated_1	Ctrl_Untreated_1	-0.8586	-0.3963	-2.368	0.0323	0.2013	-3.744	0.6962	False	ns
399	Pseudouridine	AT_1	AT_Untreated_1	Ctrl_Untreated_1	0.7815	0.3607	2.361	0.0327	0.2013	-3.756	0.6962	False	ns
308	Glutathione, Reduced	AT_1	AT_Untreated_1	Ctrl_Untreated_1	-1.116	-0.5152	-2.328	0.0349	0.2065	-3.812	0.6852	False	ns
723	beta-Ala	AT_1	AT_Untreated_1	Ctrl_Untreated_1	-0.7330	-0.3383	-2.322	0.0353	0.2065	-3.823	0.6852	False	ns
75	Glu	AT_1	AT_Untreated_1	Ctrl_Untreated_1	-0.5775	-0.2665	-2.291	0.0374	0.2092	-3.875	0.6795	False	ns
144	Glu, gamma-Me Ester	AT_1	AT_Untreated_1	Ctrl_Untreated_1	-1.081	-0.4990	-2.278	0.0383	0.2092	-3.896	0.6795	False	ns
74	Asp	AT_1	AT_Untreated_1	Ctrl_Untreated_1	-0.6239	-0.2879	-2.274	0.0386	0.2092	-3.903	0.6795	False	ns
221	N-Ac-Met	AT_1	AT_Untreated_1	Ctrl_Untreated_1	-0.8565	-0.3953	-2.266	0.0392	0.2092	-3.917	0.6795	False	ns
285	gamma-Glu-Ala	AT_1	AT_Untreated_1	Ctrl_Untreated_1	-1.731	-0.7990	-2.230	0.0420	0.2160	-3.977	0.6655	False	ns
119	alpha-OH-Isovalerate	AT_1	AT_Untreated_1	Ctrl_Untreated_1	1.439	0.6640	2.224	0.0425	0.2160	-3.987	0.6655	False	ns
297	gamma-Glu-Thr	AT_1	AT_Untreated_1	Ctrl_Untreated_1	-1.017	-0.4695	-2.212	0.0435	0.2160	-4.007	0.6655	False	ns
70	Creatine	AT_1	AT_Untreated_1	Ctrl_Untreated_1	-0.4968	-0.2293	-2.198	0.0446	0.2160	-4.030	0.6655	False	ns
181	O-Me-Tyr	AT_1	AT_Untreated_1	Ctrl_Untreated_1	-0.8011	-0.3697	-2.194	0.0450	0.2160	-4.037	0.6655	False	ns
111	1-Me-Imidazole-Ac	AT_1	AT_Untreated_1	Ctrl_Untreated_1	0.8163	0.3768	2.156	0.0484	0.2276	-4.100	0.6429	False	ns
333	GDP	AT_1	AT_Untreated_1	Ctrl_Untreated_1	-1.611	-0.7435	-2.139	0.0499	0.2304	-4.128	0.6375	False	ns
102	2-Aminoadipate	AT_1	AT_Untreated_1	Ctrl_Untreated_1	-0.7156	-0.3303	-2.081	0.0556	0.2489	-4.221	0.6040	False	ns
107	Citrulline	AT_1	AT_Untreated_1	Ctrl_Untreated_1	0.9125	0.4211	2.077	0.0560	0.2489	-4.228	0.6040	False	ns
309	Glutathione, Oxidized	AT_1	AT_Untreated_1	Ctrl_Untreated_1	0.7515	0.3469	2.067	0.0570	0.2489	-4.244	0.6040	False	ns
128	N2-Ac-Lys/N6-Ac-Lys	AT_1	AT_Untreated_1	Ctrl_Untreated_1	-1.124	-0.5190	-2.044	0.0595	0.2551	-4.281	0.5932	False	ns
322	2'-dI	AT_1	AT_Untreated_1	Ctrl_Untreated_1	-1.347	-0.6217	-2.029	0.0613	0.2579	-4.305	0.5885	False	ns
385	Uracil	AT_1	AT_Untreated_1	Ctrl_Untreated_1	2.179	1.006	2.019	0.0624	0.2582	-4.321	0.5881	False	ns
206	Trans-4-OH-Pro	AT_1	AT_Untreated_1	Ctrl_Untreated_1	-0.5875	-0.2712	-1.977	0.0674	0.2741	-4.387	0.5621	False	ns
136	Urea	AT_1	AT_Untreated_1	Ctrl_Untreated_1	0.8567	0.3954	1.966	0.0687	0.2748	-4.404	0.5609	False	ns

Metabolite ID	Name	Comparison	Group Numerator	Group Denominator	Log2 FC	Avg Expression	t	p-value	BH adj p-value	B	-Log10 BH p	Signif.	Direction
320	TMP	AT_1	AT_Untreated_1	Ctrl_Untreated_1	-1.622	-0.7485	-1.956	0.0701	0.2756	-4.421	0.5597	False	ns
23	N-GlcNAc-Asn	AT_1	AT_Untreated_1	Ctrl_Untreated_1	-0.6936	-0.3201	-1.936	0.0726	0.2792	-4.452	0.5540	False	ns
276	Trp-Gly	AT_1	AT_Untreated_1	Ctrl_Untreated_1	-0.8514	-0.3930	-1.930	0.0734	0.2792	-4.461	0.5540	False	ns
734	Pyridoxal	AT_1	AT_Untreated_1	Ctrl_Untreated_1	0.7165	0.3307	1.919	0.0749	0.2792	-4.478	0.5540	False	ns
46	Fructose 1,6-PP / Glucose 1,6-PP / Inositol-1,4-PP / Inositol-1,3-PP	AT_1	AT_Untreated_1	Ctrl_Untreated_1	-1.487	-0.6862	-1.911	0.0760	0.2792	-4.490	0.5540	False	ns
359	N1-Me-Adenosine	AT_1	AT_Untreated_1	Ctrl_Untreated_1	-0.7390	-0.3411	-1.905	0.0768	0.2792	-4.499	0.5540	False	ns
344	Xanthine	AT_1	AT_Untreated_1	Ctrl_Untreated_1	1.075	0.4963	1.877	0.0807	0.2887	-4.542	0.5396	False	ns
717	Nicotinamide	AT_1	AT_Untreated_1	Ctrl_Untreated_1	0.8846	0.4083	1.870	0.0818	0.2887	-4.553	0.5396	False	ns
122	3-OH-Isobutyrate	AT_1	AT_Untreated_1	Ctrl_Untreated_1	0.8014	0.3699	1.828	0.0882	0.3069	-4.616	0.5131	False	ns
149	DiMe-Gly	AT_1	AT_Untreated_1	Ctrl_Untreated_1	0.6729	0.3106	1.793	0.0938	0.3216	-4.668	0.4927	False	ns
348	Allantoin	AT_1	AT_Untreated_1	Ctrl_Untreated_1	0.7077	0.3266	1.738	0.1035	0.3497	-4.750	0.4563	False	ns
357	Adenosine-3',5'-PP	AT_1	AT_Untreated_1	Ctrl_Untreated_1	-0.9025	-0.4165	-1.726	0.1055	0.3517	-4.766	0.4539	False	ns
329	AMP	AT_1	AT_Untreated_1	Ctrl_Untreated_1	-1.905	-0.8793	-1.683	0.1138	0.3742	-4.829	0.4269	False	ns
209	N-Ac-Ala	AT_1	AT_Untreated_1	Ctrl_Untreated_1	-0.5865	-0.2707	-1.673	0.1157	0.3753	-4.842	0.4256	False	ns
63	6-P-Gluconate	AT_1	AT_Untreated_1	Ctrl_Untreated_1	-1.340	-0.6185	-1.648	0.1208	0.3865	-4.877	0.4128	False	ns
350	3',5'-cAMP	AT_1	AT_Untreated_1	Ctrl_Untreated_1	-0.4679	-0.2160	-1.631	0.1244	0.3930	-4.902	0.4056	False	ns
148	Betaine	AT_1	AT_Untreated_1	Ctrl_Untreated_1	0.5156	0.2380	1.622	0.1263	0.3937	-4.914	0.4049	False	ns
114	Imidazole Propionate	AT_1	AT_Untreated_1	Ctrl_Untreated_1	0.6902	0.3186	1.612	0.1284	0.3942	-4.927	0.4043	False	ns
207	N-Me-Pro	AT_1	AT_Untreated_1	Ctrl_Untreated_1	0.5909	0.2727	1.606	0.1298	0.3942	-4.936	0.4043	False	ns
243	Creatinine	AT_1	AT_Untreated_1	Ctrl_Untreated_1	0.5364	0.2476	1.588	0.1339	0.4016	-4.961	0.3962	False	ns
61	Malate	AT_1	AT_Untreated_1	Ctrl_Untreated_1	-0.5150	-0.2377	-1.568	0.1385	0.4103	-4.989	0.3869	False	ns
13	Guanosine 5'-PP-Fucose	AT_1	AT_Untreated_1	Ctrl_Untreated_1	-1.040	-0.4800	-1.532	0.1471	0.4266	-5.037	0.3700	False	ns
44	Glucose 6-P	AT_1	AT_Untreated_1	Ctrl_Untreated_1	-0.8911	-0.4113	-1.530	0.1475	0.4266	-5.039	0.3700	False	ns
211	N-Ac-Asn	AT_1	AT_Untreated_1	Ctrl_Untreated_1	-0.5219	-0.2409	-1.497	0.1559	0.4454	-5.084	0.3512	False	ns
34	Ribitol	AT_1	AT_Untreated_1	Ctrl_Untreated_1	0.4633	0.2138	1.471	0.1627	0.4525	-5.117	0.3443	False	ns
67	Ribose 1-P	AT_1	AT_Untreated_1	Ctrl_Untreated_1	0.5371	0.2479	1.470	0.1630	0.4525	-5.119	0.3443	False	ns
738	Pyridoxate	AT_1	AT_Untreated_1	Ctrl_Untreated_1	0.5057	0.2334	1.460	0.1656	0.4525	-5.131	0.3443	False	ns
736	Pyridoxamine-P	AT_1	AT_Untreated_1	Ctrl_Untreated_1	-0.5423	-0.2503	-1.453	0.1675	0.4525	-5.140	0.3443	False	ns
375	UTP	AT_1	AT_Untreated_1	Ctrl_Untreated_1	-1.783	-0.8228	-1.452	0.1678	0.4525	-5.142	0.3443	False	ns

Metabolite ID	Name	Comparison	Group Numerator	Group Denominator	Log2 FC	Avg Expression	t	p-value	BH adj p-value	B	-Log10 BH p	Signif.	Direction
236	Spermidine	AT_1	AT_Untreated_1	Ctrl_Untreated_1	-0.8481	-0.3914	-1.443	0.1704	0.4533	-5.154	0.3436	False	ns
130	Glutarate	AT_1	AT_Untreated_1	Ctrl_Untreated_1	0.4669	0.2155	1.437	0.1719	0.4533	-5.161	0.3436	False	ns
284	Carnosine	AT_1	AT_Untreated_1	Ctrl_Untreated_1	0.5716	0.2638	1.426	0.1751	0.4567	-5.175	0.3403	False	ns
258	Ile-Gly	AT_1	AT_Untreated_1	Ctrl_Untreated_1	-0.6806	-0.3141	-1.402	0.1819	0.4694	-5.205	0.3285	False	ns
59	Succinate	AT_1	AT_Untreated_1	Ctrl_Untreated_1	0.7254	0.3348	1.367	0.1924	0.4900	-5.248	0.3098	False	ns
281	Val-Gly	AT_1	AT_Untreated_1	Ctrl_Untreated_1	-0.7141	-0.3296	-1.359	0.1951	0.4900	-5.259	0.3098	False	ns
330	ADP	AT_1	AT_Untreated_1	Ctrl_Untreated_1	-1.364	-0.6295	-1.345	0.1992	0.4900	-5.275	0.3098	False	ns
232	SAH	AT_1	AT_Untreated_1	Ctrl_Untreated_1	-0.3990	-0.1842	-1.344	0.1997	0.4900	-5.277	0.3098	False	ns
183	Phenol Sulfate	AT_1	AT_Untreated_1	Ctrl_Untreated_1	0.9215	0.4253	1.342	0.2001	0.4900	-5.279	0.3098	False	ns
71	Creatine-P	AT_1	AT_Untreated_1	Ctrl_Untreated_1	0.7514	0.3468	1.334	0.2028	0.4917	-5.289	0.3083	False	ns
704	NADH	AT_1	AT_Untreated_1	Ctrl_Untreated_1	-1.006	-0.4644	-1.315	0.2089	0.5015	-5.312	0.2998	False	ns
163	Formimino-Glu	AT_1	AT_Untreated_1	Ctrl_Untreated_1	0.4326	0.1997	1.305	0.2123	0.5045	-5.324	0.2971	False	ns
173	Met Sulfoxide	AT_1	AT_Untreated_1	Ctrl_Untreated_1	0.3965	0.1830	1.294	0.2158	0.5061	-5.336	0.2958	False	ns
709	Pyridoxal-P	AT_1	AT_Untreated_1	Ctrl_Untreated_1	-0.4730	-0.2183	-1.290	0.2172	0.5061	-5.341	0.2958	False	ns
338	Guanosine	AT_1	AT_Untreated_1	Ctrl_Untreated_1	0.5342	0.2466	1.272	0.2235	0.5124	-5.362	0.2904	False	ns
35	Ribonate	AT_1	AT_Untreated_1	Ctrl_Untreated_1	-0.3719	-0.1717	-1.270	0.2242	0.5124	-5.365	0.2904	False	ns
89	Trp	AT_1	AT_Untreated_1	Ctrl_Untreated_1	-0.5633	-0.2600	-1.255	0.2292	0.5189	-5.381	0.2849	False	ns
140	Carboxyethyl-GABA	AT_1	AT_Untreated_1	Ctrl_Untreated_1	-0.5833	-0.2692	-1.236	0.2360	0.5293	-5.403	0.2763	False	ns
710	Carnitine	AT_1	AT_Untreated_1	Ctrl_Untreated_1	-0.4233	-0.1954	-1.216	0.2435	0.5410	-5.426	0.2668	False	ns
392	3'-UMP	AT_1	AT_Untreated_1	Ctrl_Untreated_1	0.9412	0.4344	1.194	0.2515	0.5518	-5.450	0.2582	False	ns
212	N-Ac-Asp	AT_1	AT_Untreated_1	Ctrl_Untreated_1	-0.6043	-0.2789	-1.191	0.2529	0.5518	-5.454	0.2582	False	ns
267	Phe-Gly	AT_1	AT_Untreated_1	Ctrl_Untreated_1	-0.5825	-0.2689	-1.174	0.2591	0.5591	-5.472	0.2525	False	ns
724	Pantothenate	AT_1	AT_Untreated_1	Ctrl_Untreated_1	-0.3796	-0.1752	-1.170	0.2609	0.5591	-5.477	0.2525	False	ns
8	Cytidine 5'-P-N-Ac-Neuraminate	AT_1	AT_Untreated_1	Ctrl_Untreated_1	-0.2973	-0.1372	-1.163	0.2634	0.5595	-5.484	0.2522	False	ns
64	Sedoheptulose-7-P	AT_1	AT_Untreated_1	Ctrl_Untreated_1	-0.3799	-0.1754	-1.155	0.2666	0.5598	-5.493	0.2520	False	ns
286	gamma-Glu-Glu	AT_1	AT_Untreated_1	Ctrl_Untreated_1	-0.4664	-0.2153	-1.151	0.2682	0.5598	-5.497	0.2520	False	ns
217	N-Ac-His	AT_1	AT_Untreated_1	Ctrl_Untreated_1	-0.3442	-0.1589	-1.096	0.2910	0.5965	-5.555	0.2244	False	ns
76	Gln	AT_1	AT_Untreated_1	Ctrl_Untreated_1	-0.3899	-0.1799	-1.086	0.2953	0.5965	-5.566	0.2244	False	ns
77	Gly	AT_1	AT_Untreated_1	Ctrl_Untreated_1	-0.3988	-0.1841	-1.086	0.2953	0.5965	-5.566	0.2244	False	ns
91	Pro	AT_1	AT_Untreated_1	Ctrl_Untreated_1	-0.3117	-0.1438	-1.085	0.2958	0.5965	-5.567	0.2244	False	ns
210	N-Ac-Arg	AT_1	AT_Untreated_1	Ctrl_Untreated_1	-0.4492	-0.2073	-1.067	0.3036	0.6007	-5.585	0.2213	False	ns

Metabolite ID	Name	Comparison	Group Numerator	Group Denominator	Log2 FC	Avg Expression	t	p-value	BH adj p-value	B	-Log10 BH p	Signif.	Direction
41	Lactate	AT_1	AT_Untreated_1	Ctrl_Untreated_1	-0.3280	-0.1514	-1.064	0.3049	0.6007	-5.588	0.2213	False	ns
251	Gly-Leu	AT_1	AT_Untreated_1	Ctrl_Untreated_1	-0.5494	-0.2536	-1.063	0.3054	0.6007	-5.589	0.2213	False	ns
72	Ala	AT_1	AT_Untreated_1	Ctrl_Untreated_1	-0.3235	-0.1493	-1.030	0.3201	0.6245	-5.622	0.2044	False	ns
307	Cys-Gly	AT_1	AT_Untreated_1	Ctrl_Untreated_1	-0.5475	-0.2527	-1.012	0.3283	0.6355	-5.639	0.1969	False	ns
278	Tyr-Gly	AT_1	AT_Untreated_1	Ctrl_Untreated_1	-0.4256	-0.1964	-1.002	0.3327	0.6388	-5.648	0.1946	False	ns
90	Arg	AT_1	AT_Untreated_1	Ctrl_Untreated_1	0.3081	0.1422	0.9779	0.3441	0.6515	-5.671	0.1861	False	ns
203	DiMe-Arg	AT_1	AT_Untreated_1	Ctrl_Untreated_1	-0.6245	-0.2882	-0.9765	0.3448	0.6515	-5.672	0.1861	False	ns
380	Orotate	AT_1	AT_Untreated_1	Ctrl_Untreated_1	-0.5234	-0.2416	-0.9710	0.3475	0.6515	-5.678	0.1861	False	ns
721	N'-Methylnicotinate	AT_1	AT_Untreated_1	Ctrl_Untreated_1	-0.3101	-0.1431	-0.9566	0.3544	0.6543	-5.691	0.1842	False	ns
174	Met Sulfone	AT_1	AT_Untreated_1	Ctrl_Untreated_1	-0.3824	-0.1765	-0.9551	0.3552	0.6543	-5.692	0.1842	False	ns
85	Cys	AT_1	AT_Untreated_1	Ctrl_Untreated_1	-0.3583	-0.1654	-0.9511	0.3572	0.6543	-5.696	0.1842	False	ns
165	N-6-Tri-Me-Lys	AT_1	AT_Untreated_1	Ctrl_Untreated_1	-0.3211	-0.1482	-0.9382	0.3635	0.6576	-5.708	0.1821	False	ns
345	Guanine	AT_1	AT_Untreated_1	Ctrl_Untreated_1	0.5528	0.2551	0.9324	0.3664	0.6576	-5.713	0.1821	False	ns
270	Pro-Gly	AT_1	AT_Untreated_1	Ctrl_Untreated_1	-0.7535	-0.3478	-0.9297	0.3677	0.6576	-5.715	0.1821	False	ns
20	Erythronate	AT_1	AT_Untreated_1	Ctrl_Untreated_1	0.3242	0.1496	0.9254	0.3699	0.6576	-5.719	0.1821	False	ns
12	Glucuronate 1-P	AT_1	AT_Untreated_1	Ctrl_Untreated_1	-0.2990	-0.1380	-0.9149	0.3752	0.6622	-5.728	0.1790	False	ns
363	N6-Carbamoyl-Thr-Adenosine	AT_1	AT_Untreated_1	Ctrl_Untreated_1	-0.4201	-0.1939	-0.8807	0.3929	0.6882	-5.758	0.1623	False	ns
371	CDP	AT_1	AT_Untreated_1	Ctrl_Untreated_1	-0.8548	-0.3945	-0.8693	0.3988	0.6912	-5.767	0.1604	False	ns
287	gamma-Glu-Gln	AT_1	AT_Untreated_1	Ctrl_Untreated_1	-0.5831	-0.2691	-0.8665	0.4003	0.6912	-5.770	0.1604	False	ns
81	Ile	AT_1	AT_Untreated_1	Ctrl_Untreated_1	0.2244	0.1035	0.8409	0.4140	0.7098	-5.791	0.1489	False	ns
255	His-Ala	AT_1	AT_Untreated_1	Ctrl_Untreated_1	-0.4242	-0.1958	-0.8276	0.4213	0.7171	-5.802	0.1444	False	ns
42	2-Me-Citrate	AT_1	AT_Untreated_1	Ctrl_Untreated_1	0.3388	0.1564	0.8011	0.4360	0.7369	-5.823	0.1326	False	ns
332	GMP	AT_1	AT_Untreated_1	Ctrl_Untreated_1	-0.9640	-0.4449	-0.7858	0.4446	0.7462	-5.835	0.1271	False	ns
335	Inosine	AT_1	AT_Untreated_1	Ctrl_Untreated_1	-0.3149	-0.1453	-0.7777	0.4493	0.7488	-5.841	0.1257	False	ns
374	UDP	AT_1	AT_Untreated_1	Ctrl_Untreated_1	-1.177	-0.5433	-0.7718	0.4526	0.7491	-5.845	0.1254	False	ns
377	Uridine	AT_1	AT_Untreated_1	Ctrl_Untreated_1	0.3310	0.1528	0.7549	0.4624	0.7601	-5.858	0.1191	False	ns
387	3-Aminoisobutyrate	AT_1	AT_Untreated_1	Ctrl_Untreated_1	-0.2554	-0.1179	-0.7399	0.4712	0.7636	-5.869	0.1171	False	ns
237	Spermine	AT_1	AT_Untreated_1	Ctrl_Untreated_1	0.6346	0.2929	0.7370	0.4729	0.7636	-5.871	0.1171	False	ns
3	Glucosamine 6-P	AT_1	AT_Untreated_1	Ctrl_Untreated_1	-0.5894	-0.2720	-0.7350	0.4741	0.7636	-5.873	0.1171	False	ns
79	Thr	AT_1	AT_Untreated_1	Ctrl_Untreated_1	-0.2494	-0.1151	-0.7236	0.4808	0.7693	-5.881	0.1139	False	ns
735	Pyridoxamine	AT_1	AT_Untreated_1	Ctrl_Untreated_1	0.2026	0.0935	0.7025	0.4935	0.7843	-5.896	0.1055	False	ns

Metabolite ID	Name	Comparison	Group Numerator	Group Denominator	Log2 FC	Avg Expression	t	p-value	BH adj p-value	B	-Log10 BH p	Signif.	Direction
33	Arabitol/Xylitol	AT_1	AT_Untreated_1	Ctrl_Untreated_1	-0.3667	-0.1693	-0.6889	0.5018	0.7922	-5.905	0.1011	False	ns
133	Ornithine	AT_1	AT_Untreated_1	Ctrl_Untreated_1	-0.2830	-0.1306	-0.6673	0.5150	0.8079	-5.919	0.0926	False	ns
26	Galactonate	AT_1	AT_Untreated_1	Ctrl_Untreated_1	-0.3679	-0.1698	-0.6500	0.5259	0.8110	-5.931	0.0910	False	ns
250	Gly-Ile	AT_1	AT_Untreated_1	Ctrl_Untreated_1	-0.2961	-0.1367	-0.6499	0.5259	0.8110	-5.931	0.0910	False	ns
266	Phe-Ala	AT_1	AT_Untreated_1	Ctrl_Untreated_1	-0.4000	-0.1846	-0.6475	0.5274	0.8110	-5.932	0.0910	False	ns
10	UDP-Galactose	AT_1	AT_Untreated_1	Ctrl_Untreated_1	0.3150	0.1454	0.6423	0.5307	0.8110	-5.936	0.0910	False	ns
216	N-Ac-Gly	AT_1	AT_Untreated_1	Ctrl_Untreated_1	0.3233	0.1492	0.6373	0.5339	0.8110	-5.939	0.0910	False	ns
720	1-Me-Nicotinamide	AT_1	AT_Untreated_1	Ctrl_Untreated_1	-0.1764	-0.0814	-0.6222	0.5435	0.8203	-5.948	0.0860	False	ns
175	N-Ac-Met Sulfoxide	AT_1	AT_Untreated_1	Ctrl_Untreated_1	-0.2736	-0.1263	-0.6012	0.5570	0.8235	-5.961	0.0844	False	ns
370	CMP	AT_1	AT_Untreated_1	Ctrl_Untreated_1	-0.2128	-0.0982	-0.6005	0.5574	0.8235	-5.961	0.0844	False	ns
280	Val-Gln	AT_1	AT_Untreated_1	Ctrl_Untreated_1	-0.4667	-0.2154	-0.6001	0.5577	0.8235	-5.962	0.0844	False	ns
336	Adenosine	AT_1	AT_Untreated_1	Ctrl_Untreated_1	-0.2770	-0.1279	-0.5977	0.5593	0.8235	-5.963	0.0844	False	ns
40	Glucuronate	AT_1	AT_Untreated_1	Ctrl_Untreated_1	-0.1399	-0.0646	-0.5680	0.5787	0.8450	-5.980	0.0732	False	ns
254	Gly-Val	AT_1	AT_Untreated_1	Ctrl_Untreated_1	-0.2360	-0.1089	-0.5647	0.5809	0.8450	-5.982	0.0732	False	ns
151	Phenylacetylglycine	AT_1	AT_Untreated_1	Ctrl_Untreated_1	0.2453	0.1132	0.5540	0.5880	0.8501	-5.988	0.0705	False	ns
712	Retinol (Vit A)	AT_1	AT_Untreated_1	Ctrl_Untreated_1	-0.1409	-0.0650	-0.5406	0.5970	0.8580	-5.995	0.0665	False	ns
708	Thiamin-PP	AT_1	AT_Untreated_1	Ctrl_Untreated_1	0.1810	0.0836	0.5230	0.6089	0.8621	-6.005	0.0644	False	ns
60	Fumarate	AT_1	AT_Untreated_1	Ctrl_Untreated_1	-0.1663	-0.0767	-0.5222	0.6094	0.8621	-6.005	0.0644	False	ns
51	PEP	AT_1	AT_Untreated_1	Ctrl_Untreated_1	0.2780	0.1283	0.5146	0.6146	0.8621	-6.009	0.0644	False	ns
368	7-Me-Guanine	AT_1	AT_Untreated_1	Ctrl_Untreated_1	0.2004	0.0925	0.5140	0.6150	0.8621	-6.009	0.0644	False	ns
5	GlcNAc 1-P	AT_1	AT_Untreated_1	Ctrl_Untreated_1	0.2636	0.1217	0.5098	0.6179	0.8621	-6.012	0.0644	False	ns
137	1-Me-Guanidine	AT_1	AT_Untreated_1	Ctrl_Untreated_1	0.2174	0.1004	0.5035	0.6222	0.8632	-6.015	0.0639	False	ns
48	DHAP	AT_1	AT_Untreated_1	Ctrl_Untreated_1	-0.1866	-0.0861	-0.4892	0.6320	0.8717	-6.022	0.0596	False	ns
347	Allantoic Acid	AT_1	AT_Untreated_1	Ctrl_Untreated_1	0.2647	0.1222	0.4723	0.6437	0.8778	-6.030	0.0566	False	ns
84	Lys	AT_1	AT_Untreated_1	Ctrl_Untreated_1	-0.1281	-0.0591	-0.4650	0.6489	0.8778	-6.034	0.0566	False	ns
199	Serotonin	AT_1	AT_Untreated_1	Ctrl_Untreated_1	0.1761	0.0813	0.4608	0.6518	0.8778	-6.035	0.0566	False	ns
352	3'-AMP	AT_1	AT_Untreated_1	Ctrl_Untreated_1	-0.1743	-0.0805	-0.4515	0.6583	0.8778	-6.040	0.0566	False	ns
314	Cys-Glutathione Disulfide	AT_1	AT_Untreated_1	Ctrl_Untreated_1	0.3275	0.1512	0.4444	0.6633	0.8778	-6.043	0.0566	False	ns
722	ADP-Ribose	AT_1	AT_Untreated_1	Ctrl_Untreated_1	0.5226	0.2412	0.4426	0.6646	0.8778	-6.044	0.0566	False	ns
82	Leu	AT_1	AT_Untreated_1	Ctrl_Untreated_1	0.1449	0.0669	0.4369	0.6686	0.8778	-6.046	0.0566	False	ns
58	alpha-Ketoglutarate	AT_1	AT_Untreated_1	Ctrl_Untreated_1	0.1564	0.0722	0.4349	0.6700	0.8778	-6.047	0.0566	False	ns

Metabolite ID	Name	Comparison	Group Numerator	Group Denominator	Log2 FC	Avg Expression	t	p-value	BH adj p-value	B	-Log10 BH p	Signif.	Direction
263	Leu-Gly	AT_1	AT_Untreated_1	Ctrl_Untreated_1	0.4318	0.1993	0.4335	0.6710	0.8778	-6.048	0.0566	False	ns
83	Val	AT_1	AT_Untreated_1	Ctrl_Untreated_1	0.1558	0.0719	0.4260	0.6764	0.8778	-6.051	0.0566	False	ns
260	Leu-Ala	AT_1	AT_Untreated_1	Ctrl_Untreated_1	-0.3274	-0.1511	-0.4257	0.6766	0.8778	-6.051	0.0566	False	ns
706	FAD	AT_1	AT_Untreated_1	Ctrl_Untreated_1	0.1261	0.0582	0.4050	0.6914	0.8794	-6.060	0.0558	False	ns
324	2'-dU	AT_1	AT_Untreated_1	Ctrl_Untreated_1	0.2095	0.0967	0.4027	0.6930	0.8794	-6.061	0.0558	False	ns
373	UMP	AT_1	AT_Untreated_1	Ctrl_Untreated_1	-0.5168	-0.2385	-0.4001	0.6949	0.8794	-6.062	0.0558	False	ns
159	3-Me-His	AT_1	AT_Untreated_1	Ctrl_Untreated_1	-0.2220	-0.1025	-0.4001	0.6949	0.8794	-6.062	0.0558	False	ns
73	Asn	AT_1	AT_Untreated_1	Ctrl_Untreated_1	-0.1261	-0.0582	-0.3983	0.6962	0.8794	-6.063	0.0558	False	ns
235	Putrescine	AT_1	AT_Untreated_1	Ctrl_Untreated_1	-0.3511	-0.1621	-0.3822	0.7079	0.8852	-6.069	0.0530	False	ns
376	Cytidine	AT_1	AT_Untreated_1	Ctrl_Untreated_1	0.4943	0.2281	0.3793	0.7100	0.8852	-6.070	0.0530	False	ns
313	5-Oxoproline	AT_1	AT_Untreated_1	Ctrl_Untreated_1	0.1280	0.0591	0.3767	0.7118	0.8852	-6.071	0.0530	False	ns
262	Leu-Gln	AT_1	AT_Untreated_1	Ctrl_Untreated_1	-0.2620	-0.1209	-0.3680	0.7182	0.8854	-6.074	0.0528	False	ns
14	UDP-Glucuronate	AT_1	AT_Untreated_1	Ctrl_Untreated_1	0.3401	0.1570	0.3637	0.7213	0.8854	-6.076	0.0528	False	ns
240	N-Ac-Putrescine	AT_1	AT_Untreated_1	Ctrl_Untreated_1	-0.3794	-0.1751	-0.3613	0.7231	0.8854	-6.077	0.0528	False	ns
176	S-Me-Met	AT_1	AT_Untreated_1	Ctrl_Untreated_1	0.1554	0.0717	0.3344	0.7429	0.9016	-6.086	0.0450	False	ns
711	alpha-Tocopherol	AT_1	AT_Untreated_1	Ctrl_Untreated_1	0.1320	0.0609	0.3305	0.7458	0.9016	-6.087	0.0450	False	ns
292	gamma-Glu-epsilon-Lysine	AT_1	AT_Untreated_1	Ctrl_Untreated_1	0.1149	0.0530	0.3239	0.7506	0.9016	-6.090	0.0450	False	ns
88	Tyr	AT_1	AT_Untreated_1	Ctrl_Untreated_1	0.1144	0.0528	0.3207	0.7530	0.9016	-6.091	0.0450	False	ns
205	N-delta-Ac-Ornithine	AT_1	AT_Untreated_1	Ctrl_Untreated_1	0.1479	0.0683	0.3179	0.7551	0.9016	-6.092	0.0450	False	ns
265	Lys-Leu	AT_1	AT_Untreated_1	Ctrl_Untreated_1	-0.1865	-0.0861	-0.2951	0.7721	0.9034	-6.099	0.0441	False	ns
49	3-P-Glycerate	AT_1	AT_Untreated_1	Ctrl_Untreated_1	0.1178	0.0544	0.2858	0.7791	0.9034	-6.101	0.0441	False	ns
244	Ala-Leu	AT_1	AT_Untreated_1	Ctrl_Untreated_1	-0.2598	-0.1199	-0.2826	0.7815	0.9034	-6.102	0.0441	False	ns
389	3'-CMP	AT_1	AT_Untreated_1	Ctrl_Untreated_1	-0.1573	-0.0726	-0.2823	0.7817	0.9034	-6.102	0.0441	False	ns
135	Homocitrulline	AT_1	AT_Untreated_1	Ctrl_Untreated_1	-0.1061	-0.0490	-0.2805	0.7831	0.9034	-6.103	0.0441	False	ns
160	1-Me-His	AT_1	AT_Untreated_1	Ctrl_Untreated_1	0.1076	0.0497	0.2799	0.7835	0.9034	-6.103	0.0441	False	ns
141	gamma-Carboxy-Glu	AT_1	AT_Untreated_1	Ctrl_Untreated_1	-0.0721	-0.0333	-0.2791	0.7841	0.9034	-6.103	0.0441	False	ns
197	C-Glycosyl-Trp	AT_1	AT_Untreated_1	Ctrl_Untreated_1	0.1395	0.0644	0.2756	0.7867	0.9034	-6.104	0.0441	False	ns
248	Gln-Leu	AT_1	AT_Untreated_1	Ctrl_Untreated_1	-0.1809	-0.0835	-0.2702	0.7908	0.9038	-6.106	0.0439	False	ns
204	N-Me-Arg	AT_1	AT_Untreated_1	Ctrl_Untreated_1	-0.1733	-0.0800	-0.2548	0.8024	0.9068	-6.110	0.0425	False	ns
86	Met	AT_1	AT_Untreated_1	Ctrl_Untreated_1	-0.0878	-0.0405	-0.2520	0.8045	0.9068	-6.111	0.0425	False	ns
282	Val-Leu	AT_1	AT_Untreated_1	Ctrl_Untreated_1	-0.2162	-0.0998	-0.2484	0.8073	0.9068	-6.112	0.0425	False	ns

Metabolite ID	Name	Comparison	Group Numerator	Group Denominator	Log2 FC	Avg Expression	t	p-value	BH adj p-value	B	-Log10 BH p	Signif.	Direction
351	2'-AMP	AT_1	AT_Untreated_1	Ctrl_Untreated_1	0.2191	0.1011	0.2424	0.8119	0.9068	-6.113	0.0425	False	ns
150	N-Me-Gly	AT_1	AT_Untreated_1	Ctrl_Untreated_1	-0.0629	-0.0290	-0.2418	0.8123	0.9068	-6.113	0.0425	False	ns
113	Imidazole Lactate	AT_1	AT_Untreated_1	Ctrl_Untreated_1	0.1080	0.0498	0.2249	0.8252	0.9165	-6.117	0.0379	False	ns
158	4-Imidazole-Ac	AT_1	AT_Untreated_1	Ctrl_Untreated_1	-0.0735	-0.0339	-0.2202	0.8287	0.9165	-6.118	0.0379	False	ns
11	UDP-Glucose	AT_1	AT_Untreated_1	Ctrl_Untreated_1	-0.2078	-0.0959	-0.2154	0.8324	0.9165	-6.119	0.0379	False	ns
705	Coenzyme A	AT_1	AT_Untreated_1	Ctrl_Untreated_1	-0.1300	-0.0600	-0.2028	0.8421	0.9224	-6.122	0.0351	False	ns
731	Thiamin (Vitamin B1)	AT_1	AT_Untreated_1	Ctrl_Untreated_1	-0.0599	-0.0277	-0.1983	0.8456	0.9224	-6.123	0.0351	False	ns
143	S-1-Pyrroline-5-Carboxylate	AT_1	AT_Untreated_1	Ctrl_Untreated_1	-0.0837	-0.0386	-0.1865	0.8547	0.9281	-6.125	0.0324	False	ns
718	Nicotinamide Riboside	AT_1	AT_Untreated_1	Ctrl_Untreated_1	0.3367	0.1554	0.1731	0.8649	0.9351	-6.128	0.0292	False	ns
156	Homo-Arg	AT_1	AT_Untreated_1	Ctrl_Untreated_1	0.0693	0.0320	0.1562	0.8780	0.9449	-6.131	0.0246	False	ns
355	3'-GMP	AT_1	AT_Untreated_1	Ctrl_Untreated_1	0.1079	0.0498	0.1466	0.8855	0.9487	-6.132	0.0229	False	ns
80	His	AT_1	AT_Untreated_1	Ctrl_Untreated_1	-0.0412	-0.0190	-0.1285	0.8996	0.9576	-6.135	0.0188	False	ns
36	Ribose	AT_1	AT_Untreated_1	Ctrl_Untreated_1	-0.0539	-0.0249	-0.1256	0.9018	0.9576	-6.135	0.0188	False	ns
719	Nicotinamide MN	AT_1	AT_Untreated_1	Ctrl_Untreated_1	-0.1177	-0.0543	-0.1190	0.9069	0.9588	-6.136	0.0183	False	ns
4	GlcNAc 6-P	AT_1	AT_Untreated_1	Ctrl_Untreated_1	0.0930	0.0429	0.1103	0.9137	0.9618	-6.137	0.0169	False	ns
342	Hypoxanthine	AT_1	AT_Untreated_1	Ctrl_Untreated_1	0.0388	0.0179	0.1035	0.9190	0.9632	-6.138	0.0163	False	ns
386	3-Ureidopropionate	AT_1	AT_Untreated_1	Ctrl_Untreated_1	0.0483	0.0223	0.0930	0.9272	0.9670	-6.139	0.0146	False	ns
366	N2,N2-DiMe-Guanosine	AT_1	AT_Untreated_1	Ctrl_Untreated_1	0.0321	0.0148	0.0884	0.9308	0.9670	-6.139	0.0146	False	ns
208	Pro-OH-Pro	AT_1	AT_Untreated_1	Ctrl_Untreated_1	-0.0274	-0.0127	-0.0638	0.9500	0.9822	-6.141	0.0078	False	ns
275	Thr-Phe	AT_1	AT_Untreated_1	Ctrl_Untreated_1	-0.0476	-0.0220	-0.0592	0.9536	0.9822	-6.141	0.0078	False	ns
145	Pyro-Gln	AT_1	AT_Untreated_1	Ctrl_Untreated_1	0.0157	0.0072	0.0513	0.9598	0.9844	-6.142	0.0068	False	ns
310	S-Lactoyl-Glutathione	AT_1	AT_Untreated_1	Ctrl_Untreated_1	0.0276	0.0127	0.0310	0.9757	0.9923	-6.143	0.0034	False	ns
155	4-Guanidinobutanoate	AT_1	AT_Untreated_1	Ctrl_Untreated_1	-0.0140	-0.0065	-0.0309	0.9757	0.9923	-6.143	0.0034	False	ns
78	Ser	AT_1	AT_Untreated_1	Ctrl_Untreated_1	-0.0107	-0.0050	-0.0215	0.9832	0.9956	-6.143	0.0019	False	ns
164	5-OH-Lys	AT_1	AT_Untreated_1	Ctrl_Untreated_1	0.0045	0.0021	0.0095	0.9926	0.9970	-6.143	0.0013	False	ns
87	Phe	AT_1	AT_Untreated_1	Ctrl_Untreated_1	0.0020	0.0009	0.0069	0.9946	0.9970	-6.143	0.0013	False	ns
22	N-Ac-Neuraminate	AT_1	AT_Untreated_1	Ctrl_Untreated_1	-0.0011	-0.0005	-0.0039	0.9970	0.9970	-6.143	0.0013	False	ns
114	Imidazole Propionate	G6PDD_1	G6PDD_Untreated_1	Ctrl_Untreated_1	1.193	0.4336	2.912	0.0128	0.8577	-4.469	0.0667	False	ns
711	alpha-Tocopherol	G6PDD_1	G6PDD_Untreated_1	Ctrl_Untreated_1	0.8846	0.3217	2.510	0.0270	0.8577	-4.496	0.0667	False	ns

Metabolite ID	Name	Comparison	Group Numerator	Group Denominator	Log2 FC	Avg Expression	t	p-value	BH adj p-value	B	-Log10 BH p	Signif.	Direction
155	4-Guanidinobutanoate	G6PDD_1	G6PDD_Untreated_1	Ctrl_Untreated_1	0.8738	0.3177	2.460	0.0296	0.8577	-4.499	0.0667	False	ns
389	3'-CMP	G6PDD_1	G6PDD_Untreated_1	Ctrl_Untreated_1	1.388	0.5046	2.217	0.0462	0.8577	-4.516	0.0667	False	ns
704	NADH	G6PDD_1	G6PDD_Untreated_1	Ctrl_Untreated_1	1.272	0.4626	2.203	0.0474	0.8577	-4.517	0.0667	False	ns
215	N-Ac-Glu	G6PDD_1	G6PDD_Untreated_1	Ctrl_Untreated_1	-0.8708	-0.3166	-2.189	0.0486	0.8577	-4.518	0.0667	False	ns
351	2'-AMP	G6PDD_1	G6PDD_Untreated_1	Ctrl_Untreated_1	-1.975	-0.7182	-2.182	0.0492	0.8577	-4.518	0.0667	False	ns
292	gamma-Glu-epsilon-Lysine	G6PDD_1	G6PDD_Untreated_1	Ctrl_Untreated_1	-0.7564	-0.2751	-2.143	0.0528	0.8577	-4.521	0.0667	False	ns
276	Trp-Gly	G6PDD_1	G6PDD_Untreated_1	Ctrl_Untreated_1	-0.7903	-0.2874	-2.083	0.0588	0.8577	-4.525	0.0667	False	ns
316	Ophthalmate	G6PDD_1	G6PDD_Untreated_1	Ctrl_Untreated_1	-1.210	-0.4399	-2.051	0.0623	0.8577	-4.528	0.0667	False	ns
337	Xanthosine	G6PDD_1	G6PDD_Untreated_1	Ctrl_Untreated_1	-0.7578	-0.2756	-2.038	0.0637	0.8577	-4.529	0.0667	False	ns
84	Lys	G6PDD_1	G6PDD_Untreated_1	Ctrl_Untreated_1	-0.5772	-0.2099	-2.001	0.0680	0.8577	-4.531	0.0667	False	ns
734	Pyridoxal	G6PDD_1	G6PDD_Untreated_1	Ctrl_Untreated_1	-0.7575	-0.2754	-1.950	0.0744	0.8577	-4.535	0.0667	False	ns
58	alpha-Ketoglutarate	G6PDD_1	G6PDD_Untreated_1	Ctrl_Untreated_1	0.7204	0.2620	1.931	0.0768	0.8577	-4.536	0.0667	False	ns
221	N-Ac-Met	G6PDD_1	G6PDD_Untreated_1	Ctrl_Untreated_1	-0.6354	-0.2311	-1.910	0.0798	0.8577	-4.538	0.0667	False	ns
339	AICAR	G6PDD_1	G6PDD_Untreated_1	Ctrl_Untreated_1	0.8013	0.2914	1.889	0.0828	0.8577	-4.539	0.0667	False	ns
26	Galactonate	G6PDD_1	G6PDD_Untreated_1	Ctrl_Untreated_1	1.154	0.4198	1.880	0.0841	0.8577	-4.540	0.0667	False	ns
286	gamma-Glu-Glu	G6PDD_1	G6PDD_Untreated_1	Ctrl_Untreated_1	0.7199	0.2618	1.810	0.0948	0.8577	-4.545	0.0667	False	ns
119	alpha-OH-Isovalerate	G6PDD_1	G6PDD_Untreated_1	Ctrl_Untreated_1	-1.410	-0.5126	-1.809	0.0950	0.8577	-4.545	0.0667	False	ns
14	UDP-Glucuronate	G6PDD_1	G6PDD_Untreated_1	Ctrl_Untreated_1	1.622	0.5896	1.798	0.0968	0.8577	-4.545	0.0667	False	ns
724	Pantothenate	G6PDD_1	G6PDD_Untreated_1	Ctrl_Untreated_1	0.6148	0.2236	1.768	0.1019	0.8577	-4.548	0.0667	False	ns
36	Ribose	G6PDD_1	G6PDD_Untreated_1	Ctrl_Untreated_1	-0.6947	-0.2526	-1.753	0.1045	0.8577	-4.549	0.0667	False	ns
322	2'-dI	G6PDD_1	G6PDD_Untreated_1	Ctrl_Untreated_1	-1.365	-0.4965	-1.752	0.1046	0.8577	-4.549	0.0667	False	ns

Metabolite ID	Name	Comparison	Group Numerator	Group Denominator	Log2 FC	Avg Expression	t	p-value	BH adj p-value	B	-Log10 BH p	Signif.	Direction
40	Glucuronate	G6PDD_1	G6PDD_Untreated_1	Ctrl_Untreated_1	0.4971	0.1808	1.720	0.1106	0.8577	-4.551	0.0667	False	ns
203	DiMe-Arg	G6PDD_1	G6PDD_Untreated_1	Ctrl_Untreated_1	-1.303	-0.4739	-1.708	0.1127	0.8577	-4.552	0.0667	False	ns
308	Glutathione, Reduced	G6PDD_1	G6PDD_Untreated_1	Ctrl_Untreated_1	0.7526	0.2737	1.698	0.1148	0.8577	-4.552	0.0667	False	ns
85	Cys	G6PDD_1	G6PDD_Untreated_1	Ctrl_Untreated_1	-0.7132	-0.2593	-1.621	0.1303	0.8577	-4.558	0.0667	False	ns
723	beta-Ala	G6PDD_1	G6PDD_Untreated_1	Ctrl_Untreated_1	0.6233	0.2267	1.616	0.1316	0.8577	-4.558	0.0667	False	ns
55	Citrate	G6PDD_1	G6PDD_Untreated_1	Ctrl_Untreated_1	0.4152	0.1510	1.568	0.1424	0.8577	-4.561	0.0667	False	ns
234	5-Me-Thioadenosine	G6PDD_1	G6PDD_Untreated_1	Ctrl_Untreated_1	0.5331	0.1939	1.565	0.1430	0.8577	-4.561	0.0667	False	ns
188	Kynurenine	G6PDD_1	G6PDD_Untreated_1	Ctrl_Untreated_1	0.7315	0.2660	1.556	0.1450	0.8577	-4.562	0.0667	False	ns
383	Cytosine	G6PDD_1	G6PDD_Untreated_1	Ctrl_Untreated_1	0.7356	0.2675	1.547	0.1471	0.8577	-4.563	0.0667	False	ns
133	Ornithine	G6PDD_1	G6PDD_Untreated_1	Ctrl_Untreated_1	0.6113	0.2223	1.468	0.1673	0.8577	-4.568	0.0667	False	ns
128	N2-Ac-Lys/N6-Ac-Lys	G6PDD_1	G6PDD_Untreated_1	Ctrl_Untreated_1	-1.006	-0.3658	-1.457	0.1703	0.8577	-4.569	0.0667	False	ns
17	Maltose	G6PDD_1	G6PDD_Untreated_1	Ctrl_Untreated_1	-0.8586	-0.3122	-1.447	0.1730	0.8577	-4.569	0.0667	False	ns
67	Ribose 1-P	G6PDD_1	G6PDD_Untreated_1	Ctrl_Untreated_1	0.4991	0.1815	1.442	0.1744	0.8577	-4.570	0.0667	False	ns
355	3'-GMP	G6PDD_1	G6PDD_Untreated_1	Ctrl_Untreated_1	1.306	0.4750	1.434	0.1765	0.8577	-4.570	0.0667	False	ns
165	N-6-Tri-Me-Lys	G6PDD_1	G6PDD_Untreated_1	Ctrl_Untreated_1	-0.5547	-0.2017	-1.409	0.1838	0.8577	-4.572	0.0667	False	ns
212	N-Ac-Asp	G6PDD_1	G6PDD_Untreated_1	Ctrl_Untreated_1	0.7352	0.2673	1.399	0.1865	0.8577	-4.572	0.0667	False	ns
48	DHAP	G6PDD_1	G6PDD_Untreated_1	Ctrl_Untreated_1	0.7987	0.2904	1.390	0.1892	0.8577	-4.573	0.0667	False	ns
11	UDP-Glucose	G6PDD_1	G6PDD_Untreated_1	Ctrl_Untreated_1	1.299	0.4724	1.384	0.1909	0.8577	-4.573	0.0667	False	ns
156	Homo-Arg	G6PDD_1	G6PDD_Untreated_1	Ctrl_Untreated_1	-0.5049	-0.1836	-1.381	0.1918	0.8577	-4.574	0.0667	False	ns
336	Adenosine	G6PDD_1	G6PDD_Untreated_1	Ctrl_Untreated_1	0.8118	0.2952	1.358	0.1989	0.8577	-4.575	0.0667	False	ns
240	N-Ac-Putrescine	G6PDD_1	G6PDD_Untreated_1	Ctrl_Untreated_1	1.204	0.4379	1.331	0.2072	0.8577	-4.577	0.0667	False	ns

Metabolite ID	Name	Comparison	Group Numerator	Group Denominator	Log2 FC	Avg Expression	t	p-value	BH adj p-value	B	-Log10 BH p	Signif.	Direction
80	His	G6PDD_1	G6PDD_Untreated_1	Ctrl_Untreated_1	-0.4247	-0.1544	-1.327	0.2087	0.8577	-4.577	0.0667	False	ns
233	SAM	G6PDD_1	G6PDD_Untreated_1	Ctrl_Untreated_1	0.5291	0.1924	1.327	0.2087	0.8577	-4.577	0.0667	False	ns
189	Kynurenate	G6PDD_1	G6PDD_Untreated_1	Ctrl_Untreated_1	-0.4228	-0.1538	-1.299	0.2177	0.8577	-4.579	0.0667	False	ns
235	Putrescine	G6PDD_1	G6PDD_Untreated_1	Ctrl_Untreated_1	1.123	0.4083	1.296	0.2188	0.8577	-4.579	0.0667	False	ns
142	N-Ac-Asp-Glu	G6PDD_1	G6PDD_Untreated_1	Ctrl_Untreated_1	0.5986	0.2177	1.274	0.2263	0.8577	-4.580	0.0667	False	ns
267	Phe-Gly	G6PDD_1	G6PDD_Untreated_1	Ctrl_Untreated_1	-0.4718	-0.1716	-1.273	0.2265	0.8577	-4.580	0.0667	False	ns
108	Argininosuccinate	G6PDD_1	G6PDD_Untreated_1	Ctrl_Untreated_1	-0.7149	-0.2600	-1.272	0.2269	0.8577	-4.580	0.0667	False	ns
75	Glu	G6PDD_1	G6PDD_Untreated_1	Ctrl_Untreated_1	0.3168	0.1152	1.268	0.2285	0.8577	-4.581	0.0667	False	ns
310	S-Lactoyl-Glutathione	G6PDD_1	G6PDD_Untreated_1	Ctrl_Untreated_1	0.9775	0.3555	1.247	0.2356	0.8577	-4.582	0.0667	False	ns
399	Pseudouridine	G6PDD_1	G6PDD_Untreated_1	Ctrl_Untreated_1	-0.3820	-0.1389	-1.243	0.2371	0.8577	-4.582	0.0667	False	ns
86	Met	G6PDD_1	G6PDD_Untreated_1	Ctrl_Untreated_1	-0.4914	-0.1787	-1.240	0.2382	0.8577	-4.582	0.0667	False	ns
287	gamma-Glu-Gln	G6PDD_1	G6PDD_Untreated_1	Ctrl_Untreated_1	-1.042	-0.3790	-1.239	0.2384	0.8577	-4.582	0.0667	False	ns
281	Val-Gly	G6PDD_1	G6PDD_Untreated_1	Ctrl_Untreated_1	-0.4161	-0.1513	-1.236	0.2396	0.8577	-4.583	0.0667	False	ns
290	gamma-Glu-Ile	G6PDD_1	G6PDD_Untreated_1	Ctrl_Untreated_1	-0.6140	-0.2233	-1.231	0.2413	0.8577	-4.583	0.0667	False	ns
59	Succinate	G6PDD_1	G6PDD_Untreated_1	Ctrl_Untreated_1	0.8010	0.2913	1.228	0.2424	0.8577	-4.583	0.0667	False	ns
251	Gly-Leu	G6PDD_1	G6PDD_Untreated_1	Ctrl_Untreated_1	-0.5043	-0.1834	-1.211	0.2487	0.8577	-4.584	0.0667	False	ns
366	N2,N2-DiMe-Guanosine	G6PDD_1	G6PDD_Untreated_1	Ctrl_Untreated_1	-0.4354	-0.1583	-1.201	0.2524	0.8577	-4.585	0.0667	False	ns
392	3'-UMP	G6PDD_1	G6PDD_Untreated_1	Ctrl_Untreated_1	1.016	0.3693	1.199	0.2531	0.8577	-4.585	0.0667	False	ns
159	3-Me-His	G6PDD_1	G6PDD_Untreated_1	Ctrl_Untreated_1	0.5715	0.2078	1.195	0.2545	0.8577	-4.585	0.0667	False	ns
291	gamma-Glu-Leu	G6PDD_1	G6PDD_Untreated_1	Ctrl_Untreated_1	-0.5056	-0.1838	-1.189	0.2568	0.8577	-4.585	0.0667	False	ns
136	Urea	G6PDD_1	G6PDD_Untreated_1	Ctrl_Untreated_1	-0.4443	-0.1616	-1.185	0.2586	0.8577	-4.586	0.0667	False	ns

Metabolite ID	Name	Comparison	Group Numerator	Group Denominator	Log2 FC	Avg Expression	t	p-value	BH adj p-value	B	-Log10 BH p	Signif.	Direction
209	N-Ac-Ala	G6PDD_1	G6PDD_Untreated_1	Ctrl_Untreated_1	-0.3857	-0.1403	-1.174	0.2628	0.8577	-4.586	0.0667	False	ns
346	Urate	G6PDD_1	G6PDD_Untreated_1	Ctrl_Untreated_1	-0.4546	-0.1653	-1.172	0.2636	0.8577	-4.586	0.0667	False	ns
703	NAD+	G6PDD_1	G6PDD_Untreated_1	Ctrl_Untreated_1	0.3571	0.1299	1.167	0.2655	0.8577	-4.587	0.0667	False	ns
104	Cystathionine	G6PDD_1	G6PDD_Untreated_1	Ctrl_Untreated_1	0.5119	0.1861	1.166	0.2658	0.8577	-4.587	0.0667	False	ns
137	1-Me-Guanidine	G6PDD_1	G6PDD_Untreated_1	Ctrl_Untreated_1	-0.3752	-0.1365	-1.151	0.2715	0.8577	-4.588	0.0667	False	ns
90	Arg	G6PDD_1	G6PDD_Untreated_1	Ctrl_Untreated_1	-0.4026	-0.1464	-1.149	0.2726	0.8577	-4.588	0.0667	False	ns
87	Phe	G6PDD_1	G6PDD_Untreated_1	Ctrl_Untreated_1	-0.3634	-0.1322	-1.147	0.2733	0.8577	-4.588	0.0667	False	ns
718	Nicotinamide Riboside	G6PDD_1	G6PDD_Untreated_1	Ctrl_Untreated_1	-2.219	-0.8069	-1.137	0.2773	0.8577	-4.588	0.0667	False	ns
164	5-OH-Lys	G6PDD_1	G6PDD_Untreated_1	Ctrl_Untreated_1	-0.5244	-0.1907	-1.134	0.2785	0.8577	-4.589	0.0667	False	ns
102	2-Aminoadipate	G6PDD_1	G6PDD_Untreated_1	Ctrl_Untreated_1	0.4197	0.1526	1.127	0.2812	0.8577	-4.589	0.0667	False	ns
83	Val	G6PDD_1	G6PDD_Untreated_1	Ctrl_Untreated_1	-0.4486	-0.1631	-1.120	0.2840	0.8577	-4.589	0.0667	False	ns
342	Hypoxanthine	G6PDD_1	G6PDD_Untreated_1	Ctrl_Untreated_1	-0.5868	-0.2134	-1.116	0.2858	0.8577	-4.590	0.0667	False	ns
338	Guanosine	G6PDD_1	G6PDD_Untreated_1	Ctrl_Untreated_1	-0.6314	-0.2296	-1.103	0.2911	0.8577	-4.590	0.0667	False	ns
225	N-Ac-Thr	G6PDD_1	G6PDD_Untreated_1	Ctrl_Untreated_1	-0.3476	-0.1264	-1.102	0.2917	0.8577	-4.590	0.0667	False	ns
717	Nicotinamide	G6PDD_1	G6PDD_Untreated_1	Ctrl_Untreated_1	-0.4581	-0.1666	-1.097	0.2937	0.8577	-4.591	0.0667	False	ns
175	N-Ac-Met Sulfoxide	G6PDD_1	G6PDD_Untreated_1	Ctrl_Untreated_1	-0.4345	-0.1580	-1.093	0.2954	0.8577	-4.591	0.0667	False	ns
113	Imidazole Lactate	G6PDD_1	G6PDD_Untreated_1	Ctrl_Untreated_1	0.4829	0.1756	1.091	0.2962	0.8577	-4.591	0.0667	False	ns
297	gamma-Glu-Thr	G6PDD_1	G6PDD_Untreated_1	Ctrl_Untreated_1	-0.6297	-0.2290	-1.063	0.3084	0.8577	-4.593	0.0667	False	ns
34	Ribitol	G6PDD_1	G6PDD_Untreated_1	Ctrl_Untreated_1	0.3188	0.1159	1.053	0.3128	0.8577	-4.593	0.0667	False	ns
719	Nicotinamide MN	G6PDD_1	G6PDD_Untreated_1	Ctrl_Untreated_1	-1.307	-0.4753	-1.041	0.3180	0.8577	-4.594	0.0667	False	ns
82	Leu	G6PDD_1	G6PDD_Untreated_1	Ctrl_Untreated_1	-0.3747	-0.1363	-1.024	0.3254	0.8577	-4.595	0.0667	False	ns

Metabolite ID	Name	Comparison	Group Numerator	Group Denominator	Log2 FC	Avg Expression	t	p-value	BH adj p-value	B	-Log10 BH p	Signif.	Direction
217	N-Ac-His	G6PDD_1	G6PDD_Untreated_1	Ctrl_Untreated_1	-0.3501	-0.1273	-1.017	0.3287	0.8577	-4.595	0.0667	False	ns
254	Gly-Val	G6PDD_1	G6PDD_Untreated_1	Ctrl_Untreated_1	-0.4821	-0.1753	-1.009	0.3324	0.8577	-4.595	0.0667	False	ns
45	Fructose-6-P	G6PDD_1	G6PDD_Untreated_1	Ctrl_Untreated_1	0.4852	0.1764	1.009	0.3325	0.8577	-4.595	0.0667	False	ns
78	Ser	G6PDD_1	G6PDD_Untreated_1	Ctrl_Untreated_1	-0.5846	-0.2126	-1.008	0.3329	0.8577	-4.595	0.0667	False	ns
722	ADP-Ribose	G6PDD_1	G6PDD_Untreated_1	Ctrl_Untreated_1	0.8742	0.3179	1.008	0.3330	0.8577	-4.595	0.0667	False	ns
174	Met Sulfone	G6PDD_1	G6PDD_Untreated_1	Ctrl_Untreated_1	0.4222	0.1535	1.002	0.3358	0.8577	-4.596	0.0667	False	ns
183	Phenol Sulfate	G6PDD_1	G6PDD_Untreated_1	Ctrl_Untreated_1	-0.6498	-0.2363	-0.9878	0.3423	0.8577	-4.597	0.0667	False	ns
357	Adenosine-3',5'-PP	G6PDD_1	G6PDD_Untreated_1	Ctrl_Untreated_1	-0.5279	-0.1920	-0.9856	0.3434	0.8577	-4.597	0.0667	False	ns
22	N-Ac-Neuraminate	G6PDD_1	G6PDD_Untreated_1	Ctrl_Untreated_1	0.2475	0.0900	0.9680	0.3517	0.8577	-4.598	0.0667	False	ns
33	Arabitol/Xylitol	G6PDD_1	G6PDD_Untreated_1	Ctrl_Untreated_1	-0.5805	-0.2111	-0.9633	0.3540	0.8577	-4.598	0.0667	False	ns
148	Betaine	G6PDD_1	G6PDD_Untreated_1	Ctrl_Untreated_1	0.2930	0.1066	0.9609	0.3551	0.8577	-4.598	0.0667	False	ns
196	5-OH-Indole-Ac	G6PDD_1	G6PDD_Untreated_1	Ctrl_Untreated_1	-0.4669	-0.1698	-0.9506	0.3602	0.8577	-4.598	0.0667	False	ns
350	3',5'-cAMP	G6PDD_1	G6PDD_Untreated_1	Ctrl_Untreated_1	-0.2992	-0.1088	-0.9473	0.3618	0.8577	-4.599	0.0667	False	ns
89	Trp	G6PDD_1	G6PDD_Untreated_1	Ctrl_Untreated_1	-0.2717	-0.0988	-0.9432	0.3638	0.8577	-4.599	0.0667	False	ns
284	Carnosine	G6PDD_1	G6PDD_Untreated_1	Ctrl_Untreated_1	0.3461	0.1259	0.9192	0.3757	0.8577	-4.600	0.0667	False	ns
731	Thiamin (Vitamin B1)	G6PDD_1	G6PDD_Untreated_1	Ctrl_Untreated_1	0.2645	0.0962	0.8957	0.3876	0.8577	-4.601	0.0667	False	ns
151	Phenylacetylglycine	G6PDD_1	G6PDD_Untreated_1	Ctrl_Untreated_1	0.4242	0.1543	0.8916	0.3897	0.8577	-4.601	0.0667	False	ns
263	Leu-Gly	G6PDD_1	G6PDD_Untreated_1	Ctrl_Untreated_1	0.7182	0.2612	0.8914	0.3899	0.8577	-4.601	0.0667	False	ns
377	Uridine	G6PDD_1	G6PDD_Untreated_1	Ctrl_Untreated_1	-0.5138	-0.1868	-0.8913	0.3899	0.8577	-4.601	0.0667	False	ns
706	FAD	G6PDD_1	G6PDD_Untreated_1	Ctrl_Untreated_1	0.2257	0.0821	0.8844	0.3935	0.8577	-4.602	0.0667	False	ns
352	3'-AMP	G6PDD_1	G6PDD_Untreated_1	Ctrl_Untreated_1	0.3759	0.1367	0.8807	0.3954	0.8577	-4.602	0.0667	False	ns

Metabolite ID	Name	Comparison	Group Numerator	Group Denominator	Log2 FC	Avg Expression	t	p-value	BH adj p-value	B	-Log10 BH p	Signif.	Direction
198	Indolelactate	G6PDD_1	G6PDD_Untreated_1	Ctrl_Untreated_1	-0.3227	-0.1173	-0.8781	0.3968	0.8577	-4.602	0.0667	False	ns
708	Thiamin-PP	G6PDD_1	G6PDD_Untreated_1	Ctrl_Untreated_1	0.3266	0.1188	0.8754	0.3982	0.8577	-4.602	0.0667	False	ns
88	Tyr	G6PDD_1	G6PDD_Untreated_1	Ctrl_Untreated_1	-0.3497	-0.1272	-0.8657	0.4033	0.8577	-4.603	0.0667	False	ns
324	2'-dU	G6PDD_1	G6PDD_Untreated_1	Ctrl_Untreated_1	-0.4476	-0.1628	-0.8636	0.4044	0.8577	-4.603	0.0667	False	ns
181	O-Me-Tyr	G6PDD_1	G6PDD_Untreated_1	Ctrl_Untreated_1	0.3716	0.1351	0.8589	0.4069	0.8577	-4.603	0.0667	False	ns
242	Guanidino-Ac	G6PDD_1	G6PDD_Untreated_1	Ctrl_Untreated_1	-0.3176	-0.1155	-0.8578	0.4074	0.8577	-4.603	0.0667	False	ns
122	3-OH-Isobutyrate	G6PDD_1	G6PDD_Untreated_1	Ctrl_Untreated_1	-0.3247	-0.1181	-0.8508	0.4112	0.8577	-4.603	0.0667	False	ns
3	Glucosamine 6-P	G6PDD_1	G6PDD_Untreated_1	Ctrl_Untreated_1	0.7782	0.2830	0.8471	0.4131	0.8577	-4.603	0.0667	False	ns
206	Trans-4-OH-Pro	G6PDD_1	G6PDD_Untreated_1	Ctrl_Untreated_1	0.2348	0.0854	0.8215	0.4270	0.8577	-4.605	0.0667	False	ns
309	Glutathione, Oxidized	G6PDD_1	G6PDD_Untreated_1	Ctrl_Untreated_1	0.2627	0.0955	0.8155	0.4303	0.8577	-4.605	0.0667	False	ns
236	Spermidine	G6PDD_1	G6PDD_Untreated_1	Ctrl_Untreated_1	0.3038	0.1105	0.8086	0.4342	0.8577	-4.605	0.0667	False	ns
373	UMP	G6PDD_1	G6PDD_Untreated_1	Ctrl_Untreated_1	1.177	0.4281	0.8085	0.4342	0.8577	-4.605	0.0667	False	ns
61	Malate	G6PDD_1	G6PDD_Untreated_1	Ctrl_Untreated_1	0.2272	0.0826	0.8051	0.4361	0.8577	-4.605	0.0667	False	ns
335	Inosine	G6PDD_1	G6PDD_Untreated_1	Ctrl_Untreated_1	-0.4295	-0.1562	-0.8030	0.4372	0.8577	-4.605	0.0667	False	ns
171	Cys Sulfinic Acid	G6PDD_1	G6PDD_Untreated_1	Ctrl_Untreated_1	-0.5446	-0.1980	-0.7839	0.4479	0.8577	-4.606	0.0667	False	ns
255	His-Ala	G6PDD_1	G6PDD_Untreated_1	Ctrl_Untreated_1	-0.3640	-0.1323	-0.7829	0.4485	0.8577	-4.606	0.0667	False	ns
182	P-Cresol Sulfate	G6PDD_1	G6PDD_Untreated_1	Ctrl_Untreated_1	-0.2810	-0.1022	-0.7828	0.4486	0.8577	-4.606	0.0667	False	ns
278	Tyr-Gly	G6PDD_1	G6PDD_Untreated_1	Ctrl_Untreated_1	-0.2644	-0.0961	-0.7813	0.4494	0.8577	-4.606	0.0667	False	ns
372	CTP	G6PDD_1	G6PDD_Untreated_1	Ctrl_Untreated_1	-0.4589	-0.1669	-0.7808	0.4497	0.8577	-4.606	0.0667	False	ns
71	Creatine-P	G6PDD_1	G6PDD_Untreated_1	Ctrl_Untreated_1	0.3598	0.1308	0.7768	0.4520	0.8577	-4.607	0.0667	False	ns
380	Orotate	G6PDD_1	G6PDD_Untreated_1	Ctrl_Untreated_1	0.4080	0.1484	0.7639	0.4594	0.8577	-4.607	0.0667	False	ns

Metabolite ID	Name	Comparison	Group Numerator	Group Denominator	Log2 FC	Avg Expression	t	p-value	BH adj p-value	B	-Log10 BH p	Signif.	Direction
44	Glucose 6-P	G6PDD_1	G6PDD_Untreated_1	Ctrl_Untreated_1	0.5168	0.1879	0.7599	0.4617	0.8577	-4.607	0.0667	False	ns
258	Ile-Gly	G6PDD_1	G6PDD_Untreated_1	Ctrl_Untreated_1	-0.2522	-0.0917	-0.7564	0.4637	0.8577	-4.607	0.0667	False	ns
345	Guanine	G6PDD_1	G6PDD_Untreated_1	Ctrl_Untreated_1	-0.4941	-0.1797	-0.7507	0.4670	0.8577	-4.608	0.0667	False	ns
229	N-Formyl-Met	G6PDD_1	G6PDD_Untreated_1	Ctrl_Untreated_1	0.2431	0.0884	0.7404	0.4730	0.8577	-4.608	0.0667	False	ns
12	Glucuronate 1-P	G6PDD_1	G6PDD_Untreated_1	Ctrl_Untreated_1	-0.2598	-0.0945	-0.7394	0.4735	0.8577	-4.608	0.0667	False	ns
738	Pyridoxate	G6PDD_1	G6PDD_Untreated_1	Ctrl_Untreated_1	-0.2565	-0.0933	-0.7367	0.4752	0.8577	-4.608	0.0667	False	ns
160	1-Me-His	G6PDD_1	G6PDD_Untreated_1	Ctrl_Untreated_1	0.2774	0.1009	0.7356	0.4758	0.8577	-4.608	0.0667	False	ns
712	Retinol (Vit A)	G6PDD_1	G6PDD_Untreated_1	Ctrl_Untreated_1	0.1664	0.0605	0.7335	0.4770	0.8577	-4.608	0.0667	False	ns
49	3-P-Glycerate	G6PDD_1	G6PDD_Untreated_1	Ctrl_Untreated_1	0.2482	0.0902	0.7329	0.4774	0.8577	-4.608	0.0667	False	ns
79	Thr	G6PDD_1	G6PDD_Untreated_1	Ctrl_Untreated_1	-0.2740	-0.0996	-0.7260	0.4815	0.8577	-4.609	0.0667	False	ns
705	Coenzyme A	G6PDD_1	G6PDD_Untreated_1	Ctrl_Untreated_1	0.6077	0.2210	0.7228	0.4834	0.8577	-4.609	0.0667	False	ns
374	UDP	G6PDD_1	G6PDD_Untreated_1	Ctrl_Untreated_1	1.216	0.4421	0.7216	0.4840	0.8577	-4.609	0.0667	False	ns
69	3-OH-Butyrate	G6PDD_1	G6PDD_Untreated_1	Ctrl_Untreated_1	-0.2818	-0.1025	-0.7215	0.4841	0.8577	-4.609	0.0667	False	ns
300	gamma-Glu-Val	G6PDD_1	G6PDD_Untreated_1	Ctrl_Untreated_1	-0.3043	-0.1107	-0.7197	0.4852	0.8577	-4.609	0.0667	False	ns
344	Xanthine	G6PDD_1	G6PDD_Untreated_1	Ctrl_Untreated_1	-0.4696	-0.1708	-0.7053	0.4938	0.8633	-4.610	0.0638	False	ns
294	gamma-Glu-Met	G6PDD_1	G6PDD_Untreated_1	Ctrl_Untreated_1	-0.4648	-0.1690	-0.6964	0.4992	0.8633	-4.610	0.0638	False	ns
81	Ile	G6PDD_1	G6PDD_Untreated_1	Ctrl_Untreated_1	-0.1831	-0.0666	-0.6923	0.5016	0.8633	-4.610	0.0638	False	ns
204	N-Me-Arg	G6PDD_1	G6PDD_Untreated_1	Ctrl_Untreated_1	-0.5344	-0.1943	-0.6914	0.5021	0.8633	-4.610	0.0638	False	ns
347	Allantoic Acid	G6PDD_1	G6PDD_Untreated_1	Ctrl_Untreated_1	-0.3210	-0.1167	-0.6529	0.5259	0.8880	-4.612	0.0516	False	ns
275	Thr-Phe	G6PDD_1	G6PDD_Untreated_1	Ctrl_Untreated_1	0.5738	0.2086	0.6429	0.5321	0.8880	-4.612	0.0516	False	ns
307	Cys-Gly	G6PDD_1	G6PDD_Untreated_1	Ctrl_Untreated_1	0.3090	0.1124	0.6404	0.5337	0.8880	-4.612	0.0516	False	ns

Metabolite ID	Name	Comparison	Group Numerator	Group Denominator	Log2 FC	Avg Expression	t	p-value	BH adj p-value	B	-Log10 BH p	Signif.	Direction
8	Cytidine 5'-P-N-Ac-Neuraminate	G6PDD_1	G6PDD_Untreated_1	Ctrl_Untreated_1	0.1610	0.0585	0.6386	0.5348	0.8880	-4.612	0.0516	False	ns
329	AMP	G6PDD_1	G6PDD_Untreated_1	Ctrl_Untreated_1	0.7670	0.2789	0.6353	0.5369	0.8880	-4.612	0.0516	False	ns
73	Asn	G6PDD_1	G6PDD_Untreated_1	Ctrl_Untreated_1	-0.2118	-0.0770	-0.6339	0.5378	0.8880	-4.612	0.0516	False	ns
232	SAH	G6PDD_1	G6PDD_Untreated_1	Ctrl_Untreated_1	-0.1809	-0.0658	-0.6197	0.5468	0.8970	-4.613	0.0472	False	ns
241	4-Acetamidobutanoate	G6PDD_1	G6PDD_Untreated_1	Ctrl_Untreated_1	0.2477	0.0901	0.6104	0.5527	0.9008	-4.613	0.0454	False	ns
46	Fructose 1,6-PP / Glucose 1,6-PP / Inositol-1,4-PP / Inositol-1,3-PP	G6PDD_1	G6PDD_Untreated_1	Ctrl_Untreated_1	0.3960	0.1440	0.5881	0.5671	0.9184	-4.614	0.0370	False	ns
23	N-GlcNAc-Asn	G6PDD_1	G6PDD_Untreated_1	Ctrl_Untreated_1	0.2293	0.0834	0.5812	0.5716	0.9197	-4.614	0.0364	False	ns
120	beta-OH-Isovalerate	G6PDD_1	G6PDD_Untreated_1	Ctrl_Untreated_1	-0.2902	-0.1055	-0.5689	0.5797	0.9268	-4.615	0.0330	False	ns
19	Maltotetraose	G6PDD_1	G6PDD_Untreated_1	Ctrl_Untreated_1	-0.5090	-0.1851	-0.5568	0.5877	0.9324	-4.615	0.0304	False	ns
20	Erythronate	G6PDD_1	G6PDD_Untreated_1	Ctrl_Untreated_1	0.1896	0.0690	0.5510	0.5915	0.9324	-4.615	0.0304	False	ns
211	N-Ac-Asn	G6PDD_1	G6PDD_Untreated_1	Ctrl_Untreated_1	0.1850	0.0673	0.5467	0.5944	0.9324	-4.615	0.0304	False	ns
709	Pyridoxal-P	G6PDD_1	G6PDD_Untreated_1	Ctrl_Untreated_1	0.2190	0.0796	0.5401	0.5988	0.9335	-4.616	0.0299	False	ns
330	ADP	G6PDD_1	G6PDD_Untreated_1	Ctrl_Untreated_1	0.6627	0.2410	0.5278	0.6070	0.9405	-4.616	0.0266	False	ns
710	Carnitine	G6PDD_1	G6PDD_Untreated_1	Ctrl_Untreated_1	-0.2193	-0.0798	-0.5179	0.6137	0.9450	-4.616	0.0246	False	ns
250	Gly-Ile	G6PDD_1	G6PDD_Untreated_1	Ctrl_Untreated_1	0.1713	0.0623	0.5026	0.6242	0.9553	-4.617	0.0199	False	ns
222	N-Ac-Phe	G6PDD_1	G6PDD_Untreated_1	Ctrl_Untreated_1	0.1474	0.0536	0.4715	0.6456	0.9686	-4.618	0.0139	False	ns
173	Met Sulfoxide	G6PDD_1	G6PDD_Untreated_1	Ctrl_Untreated_1	-0.1967	-0.0715	-0.4686	0.6476	0.9686	-4.618	0.0139	False	ns
199	Serotonin	G6PDD_1	G6PDD_Untreated_1	Ctrl_Untreated_1	-0.1700	-0.0618	-0.4665	0.6490	0.9686	-4.618	0.0139	False	ns
319	2'-dAMP	G6PDD_1	G6PDD_Untreated_1	Ctrl_Untreated_1	0.2205	0.0802	0.4658	0.6495	0.9686	-4.618	0.0139	False	ns
42	2-Me-Citrate	G6PDD_1	G6PDD_Untreated_1	Ctrl_Untreated_1	-0.1699	-0.0618	-0.4536	0.6581	0.9686	-4.618	0.0139	False	ns

Metabolite ID	Name	Comparison	Group Numerator	Group Denominator	Log2 FC	Avg Expression	t	p-value	BH adj p-value	B	-Log10 BH p	Signif.	Direction
208	Pro-OH-Pro	G6PDD_1	G6PDD_Untreated_1	Ctrl_Untreated_1	0.2150	0.0782	0.4519	0.6592	0.9686	-4.618	0.0139	False	ns
270	Pro-Gly	G6PDD_1	G6PDD_Untreated_1	Ctrl_Untreated_1	-0.2510	-0.0913	-0.4510	0.6599	0.9686	-4.618	0.0139	False	ns
332	GMP	G6PDD_1	G6PDD_Untreated_1	Ctrl_Untreated_1	0.6022	0.2190	0.4386	0.6685	0.9702	-4.618	0.0131	False	ns
265	Lys-Leu	G6PDD_1	G6PDD_Untreated_1	Ctrl_Untreated_1	0.3213	0.1168	0.4270	0.6768	0.9702	-4.619	0.0131	False	ns
10	UDP-Galactose	G6PDD_1	G6PDD_Untreated_1	Ctrl_Untreated_1	0.1951	0.0709	0.4233	0.6794	0.9702	-4.619	0.0131	False	ns
77	Gly	G6PDD_1	G6PDD_Untreated_1	Ctrl_Untreated_1	-0.1638	-0.0596	-0.4153	0.6851	0.9702	-4.619	0.0131	False	ns
224	N-Ac-Ser	G6PDD_1	G6PDD_Untreated_1	Ctrl_Untreated_1	-0.1439	-0.0523	-0.4089	0.6897	0.9702	-4.619	0.0131	False	ns
13	Guanosine 5'-PP-Fucose	G6PDD_1	G6PDD_Untreated_1	Ctrl_Untreated_1	0.1329	0.0483	0.4028	0.6940	0.9702	-4.619	0.0131	False	ns
74	Asp	G6PDD_1	G6PDD_Untreated_1	Ctrl_Untreated_1	-0.1221	-0.0444	-0.4006	0.6956	0.9702	-4.619	0.0131	False	ns
163	Formimino-Glu	G6PDD_1	G6PDD_Untreated_1	Ctrl_Untreated_1	0.1344	0.0489	0.3979	0.6975	0.9702	-4.619	0.0131	False	ns
237	Spermine	G6PDD_1	G6PDD_Untreated_1	Ctrl_Untreated_1	-0.3225	-0.1173	-0.3935	0.7007	0.9702	-4.620	0.0131	False	ns
343	Adenine	G6PDD_1	G6PDD_Untreated_1	Ctrl_Untreated_1	0.1366	0.0497	0.3833	0.7081	0.9702	-4.620	0.0131	False	ns
72	Ala	G6PDD_1	G6PDD_Untreated_1	Ctrl_Untreated_1	0.1139	0.0414	0.3788	0.7113	0.9702	-4.620	0.0131	False	ns
210	N-Ac-Arg	G6PDD_1	G6PDD_Untreated_1	Ctrl_Untreated_1	-0.1734	-0.0631	-0.3670	0.7198	0.9702	-4.620	0.0131	False	ns
130	Glutarate	G6PDD_1	G6PDD_Untreated_1	Ctrl_Untreated_1	-0.1149	-0.0418	-0.3558	0.7280	0.9702	-4.620	0.0131	False	ns
24	Fructose	G6PDD_1	G6PDD_Untreated_1	Ctrl_Untreated_1	-0.1692	-0.0615	-0.3521	0.7307	0.9702	-4.620	0.0131	False	ns
313	5-Oxoproline	G6PDD_1	G6PDD_Untreated_1	Ctrl_Untreated_1	0.0951	0.0346	0.3488	0.7331	0.9702	-4.621	0.0131	False	ns
216	N-Ac-Gly	G6PDD_1	G6PDD_Untreated_1	Ctrl_Untreated_1	-0.1454	-0.0529	-0.3459	0.7353	0.9702	-4.621	0.0131	False	ns
107	Citrulline	G6PDD_1	G6PDD_Untreated_1	Ctrl_Untreated_1	0.1114	0.0405	0.3325	0.7451	0.9702	-4.621	0.0131	False	ns
375	UTP	G6PDD_1	G6PDD_Untreated_1	Ctrl_Untreated_1	0.4668	0.1697	0.3321	0.7454	0.9702	-4.621	0.0131	False	ns
18	Maltotriose	G6PDD_1	G6PDD_Untreated_1	Ctrl_Untreated_1	-0.2193	-0.0798	-0.3183	0.7556	0.9702	-4.621	0.0131	False	ns

Metabolite ID	Name	Comparison	Group Numerator	Group Denominator	Log2 FC	Avg Expression	t	p-value	BH adj p-value	B	-Log10 BH p	Signif.	Direction
64	Sedoheptulose-7-P	G6PDD_1	G6PDD_Untreated_1	Ctrl_Untreated_1	-0.1056	-0.0384	-0.3153	0.7578	0.9702	-4.621	0.0131	False	ns
376	Cytidine	G6PDD_1	G6PDD_Untreated_1	Ctrl_Untreated_1	0.3992	0.1451	0.3127	0.7597	0.9702	-4.621	0.0131	False	ns
145	Pyro-Gln	G6PDD_1	G6PDD_Untreated_1	Ctrl_Untreated_1	0.0899	0.0327	0.3107	0.7613	0.9702	-4.621	0.0131	False	ns
70	Creatine	G6PDD_1	G6PDD_Untreated_1	Ctrl_Untreated_1	-0.0773	-0.0281	-0.3104	0.7614	0.9702	-4.621	0.0131	False	ns
735	Pyridoxamine	G6PDD_1	G6PDD_Untreated_1	Ctrl_Untreated_1	-0.1060	-0.0386	-0.3083	0.7630	0.9702	-4.621	0.0131	False	ns
197	C-Glycosyl-Trp	G6PDD_1	G6PDD_Untreated_1	Ctrl_Untreated_1	0.1260	0.0458	0.3080	0.7632	0.9702	-4.621	0.0131	False	ns
333	GDP	G6PDD_1	G6PDD_Untreated_1	Ctrl_Untreated_1	-0.3184	-0.1158	-0.3052	0.7653	0.9702	-4.621	0.0131	False	ns
111	1-Me-Imidazole-Ac	G6PDD_1	G6PDD_Untreated_1	Ctrl_Untreated_1	0.1113	0.0405	0.3052	0.7654	0.9702	-4.621	0.0131	False	ns
387	3-Aminoisobutyrate	G6PDD_1	G6PDD_Untreated_1	Ctrl_Untreated_1	0.1195	0.0435	0.2868	0.7791	0.9815	-4.622	0.0081	False	ns
244	Ala-Leu	G6PDD_1	G6PDD_Untreated_1	Ctrl_Untreated_1	0.1951	0.0709	0.2813	0.7832	0.9815	-4.622	0.0081	False	ns
150	N-Me-Gly	G6PDD_1	G6PDD_Untreated_1	Ctrl_Untreated_1	0.0798	0.0290	0.2776	0.7860	0.9815	-4.622	0.0081	False	ns
35	Ribonate	G6PDD_1	G6PDD_Untreated_1	Ctrl_Untreated_1	0.0726	0.0264	0.2630	0.7969	0.9840	-4.622	0.0070	False	ns
41	Lactate	G6PDD_1	G6PDD_Untreated_1	Ctrl_Untreated_1	0.0683	0.0248	0.2477	0.8085	0.9840	-4.622	0.0070	False	ns
176	S-Me-Met	G6PDD_1	G6PDD_Untreated_1	Ctrl_Untreated_1	-0.0977	-0.0355	-0.2470	0.8090	0.9840	-4.622	0.0070	False	ns
262	Leu-Gln	G6PDD_1	G6PDD_Untreated_1	Ctrl_Untreated_1	0.1600	0.0582	0.2417	0.8130	0.9840	-4.622	0.0070	False	ns
177	3-(4-OH-Phenyl)Lactate	G6PDD_1	G6PDD_Untreated_1	Ctrl_Untreated_1	0.0927	0.0337	0.2378	0.8159	0.9840	-4.623	0.0070	False	ns
371	CDP	G6PDD_1	G6PDD_Untreated_1	Ctrl_Untreated_1	0.2234	0.0812	0.2201	0.8294	0.9840	-4.623	0.0070	False	ns
720	1-Me-Nicotinamide	G6PDD_1	G6PDD_Untreated_1	Ctrl_Untreated_1	0.0677	0.0246	0.2009	0.8440	0.9840	-4.623	0.0070	False	ns
306	gamma-Glu-Cys	G6PDD_1	G6PDD_Untreated_1	Ctrl_Untreated_1	0.0833	0.0303	0.1984	0.8460	0.9840	-4.623	0.0070	False	ns
141	gamma-Carboxy-Glu	G6PDD_1	G6PDD_Untreated_1	Ctrl_Untreated_1	0.0616	0.0224	0.1980	0.8463	0.9840	-4.623	0.0070	False	ns
368	7-Me-Guanine	G6PDD_1	G6PDD_Untreated_1	Ctrl_Untreated_1	0.0781	0.0284	0.1896	0.8527	0.9840	-4.623	0.0070	False	ns

Metabolite ID	Name	Comparison	Group Numerator	Group Denominator	Log2 FC	Avg Expression	t	p-value	BH adj p-value	B	-Log10 BH p	Signif.	Direction
243	Creatinine	G6PDD_1	G6PDD_Untreated_1	Ctrl_Untreated_1	0.0593	0.0216	0.1883	0.8537	0.9840	-4.623	0.0070	False	ns
205	N-delta-Ac-Ornithine	G6PDD_1	G6PDD_Untreated_1	Ctrl_Untreated_1	0.0686	0.0250	0.1759	0.8633	0.9840	-4.623	0.0070	False	ns
149	DiMe-Gly	G6PDD_1	G6PDD_Untreated_1	Ctrl_Untreated_1	0.0619	0.0225	0.1758	0.8633	0.9840	-4.623	0.0070	False	ns
43	Glucose	G6PDD_1	G6PDD_Untreated_1	Ctrl_Untreated_1	-0.0623	-0.0227	-0.1746	0.8642	0.9840	-4.623	0.0070	False	ns
359	N1-Me-Adenosine	G6PDD_1	G6PDD_Untreated_1	Ctrl_Untreated_1	0.0730	0.0266	0.1676	0.8696	0.9840	-4.623	0.0070	False	ns
260	Leu-Ala	G6PDD_1	G6PDD_Untreated_1	Ctrl_Untreated_1	0.1077	0.0392	0.1620	0.8739	0.9840	-4.623	0.0070	False	ns
285	gamma-Glu-Ala	G6PDD_1	G6PDD_Untreated_1	Ctrl_Untreated_1	-0.1279	-0.0465	-0.1593	0.8760	0.9840	-4.623	0.0070	False	ns
135	Homocitrulline	G6PDD_1	G6PDD_Untreated_1	Ctrl_Untreated_1	0.0560	0.0204	0.1588	0.8764	0.9840	-4.623	0.0070	False	ns
5	GlcNAc 1-P	G6PDD_1	G6PDD_Untreated_1	Ctrl_Untreated_1	-0.0855	-0.0311	-0.1573	0.8775	0.9840	-4.623	0.0070	False	ns
60	Fumarate	G6PDD_1	G6PDD_Untreated_1	Ctrl_Untreated_1	0.0450	0.0164	0.1523	0.8814	0.9840	-4.624	0.0070	False	ns
91	Pro	G6PDD_1	G6PDD_Untreated_1	Ctrl_Untreated_1	0.0441	0.0160	0.1487	0.8842	0.9840	-4.624	0.0070	False	ns
228	N-Ac-Val	G6PDD_1	G6PDD_Untreated_1	Ctrl_Untreated_1	0.0463	0.0168	0.1404	0.8906	0.9840	-4.624	0.0070	False	ns
140	Carboxyethyl-GABA	G6PDD_1	G6PDD_Untreated_1	Ctrl_Untreated_1	0.0724	0.0263	0.1293	0.8993	0.9840	-4.624	0.0070	False	ns
129	5-Aminovalerate	G6PDD_1	G6PDD_Untreated_1	Ctrl_Untreated_1	0.0510	0.0185	0.1263	0.9015	0.9840	-4.624	0.0070	False	ns
144	Glu, gamma-Me Ester	G6PDD_1	G6PDD_Untreated_1	Ctrl_Untreated_1	0.0600	0.0218	0.1247	0.9028	0.9840	-4.624	0.0070	False	ns
248	Gln-Leu	G6PDD_1	G6PDD_Untreated_1	Ctrl_Untreated_1	0.0894	0.0325	0.1203	0.9062	0.9840	-4.624	0.0070	False	ns
385	Uracil	G6PDD_1	G6PDD_Untreated_1	Ctrl_Untreated_1	-0.1750	-0.0636	-0.1190	0.9072	0.9840	-4.624	0.0070	False	ns
348	Allantoin	G6PDD_1	G6PDD_Untreated_1	Ctrl_Untreated_1	0.0425	0.0154	0.1157	0.9097	0.9840	-4.624	0.0070	False	ns
76	Gln	G6PDD_1	G6PDD_Untreated_1	Ctrl_Untreated_1	-0.0471	-0.0171	-0.1151	0.9102	0.9840	-4.624	0.0070	False	ns
282	Val-Leu	G6PDD_1	G6PDD_Untreated_1	Ctrl_Untreated_1	-0.0907	-0.0330	-0.1094	0.9147	0.9840	-4.624	0.0070	False	ns
158	4-Imidazole-Ac	G6PDD_1	G6PDD_Untreated_1	Ctrl_Untreated_1	0.0408	0.0148	0.1083	0.9155	0.9840	-4.624	0.0070	False	ns

Metabolite ID	Name	Comparison	Group Numerator	Group Denominator	Log2 FC	Avg Expression	t	p-value	BH adj p-value	B	-Log10 BH p	Signif.	Direction
363	N6-Carbamoyl-Thr-Adenosine	G6PDD_1	G6PDD_Untreated_1	Ctrl_Untreated_1	0.0559	0.0203	0.1043	0.9186	0.9840	-4.624	0.0070	False	ns
725	P-Pantetheine	G6PDD_1	G6PDD_Untreated_1	Ctrl_Untreated_1	0.0492	0.0179	0.0947	0.9261	0.9840	-4.624	0.0070	False	ns
280	Val-Gln	G6PDD_1	G6PDD_Untreated_1	Ctrl_Untreated_1	-0.0551	-0.0200	-0.0890	0.9305	0.9840	-4.624	0.0070	False	ns
143	S-1-Pyrroline-5-Carboxylate	G6PDD_1	G6PDD_Untreated_1	Ctrl_Untreated_1	0.0411	0.0149	0.0823	0.9357	0.9840	-4.624	0.0070	False	ns
736	Pyridoxamine-P	G6PDD_1	G6PDD_Untreated_1	Ctrl_Untreated_1	0.0285	0.0103	0.0760	0.9406	0.9840	-4.624	0.0070	False	ns
125	Isovaleryl-Gly	G6PDD_1	G6PDD_Untreated_1	Ctrl_Untreated_1	-0.0218	-0.0079	-0.0714	0.9442	0.9840	-4.624	0.0070	False	ns
4	GlcNAc 6-P	G6PDD_1	G6PDD_Untreated_1	Ctrl_Untreated_1	-0.0447	-0.0163	-0.0630	0.9507	0.9840	-4.624	0.0070	False	ns
370	CMP	G6PDD_1	G6PDD_Untreated_1	Ctrl_Untreated_1	0.0197	0.0072	0.0549	0.9571	0.9840	-4.624	0.0070	False	ns
739	Deoxycarnitine	G6PDD_1	G6PDD_Untreated_1	Ctrl_Untreated_1	0.0192	0.0070	0.0456	0.9644	0.9840	-4.624	0.0070	False	ns
266	Phe-Ala	G6PDD_1	G6PDD_Untreated_1	Ctrl_Untreated_1	0.0212	0.0077	0.0374	0.9708	0.9840	-4.624	0.0070	False	ns
288	gamma-Glu-Gly	G6PDD_1	G6PDD_Untreated_1	Ctrl_Untreated_1	-0.0250	-0.0091	-0.0367	0.9713	0.9840	-4.624	0.0070	False	ns
63	6-P-Gluconate	G6PDD_1	G6PDD_Untreated_1	Ctrl_Untreated_1	0.0214	0.0078	0.0359	0.9719	0.9840	-4.624	0.0070	False	ns
320	TMP	G6PDD_1	G6PDD_Untreated_1	Ctrl_Untreated_1	0.0446	0.0162	0.0358	0.9720	0.9840	-4.624	0.0070	False	ns
721	N'-Methylnicotinate	G6PDD_1	G6PDD_Untreated_1	Ctrl_Untreated_1	-0.0114	-0.0041	-0.0310	0.9758	0.9840	-4.624	0.0070	False	ns
51	PEP	G6PDD_1	G6PDD_Untreated_1	Ctrl_Untreated_1	0.0158	0.0058	0.0306	0.9761	0.9840	-4.624	0.0070	False	ns
741	5-Me-THF	G6PDD_1	G6PDD_Untreated_1	Ctrl_Untreated_1	-0.0115	-0.0042	-0.0299	0.9766	0.9840	-4.624	0.0070	False	ns
207	N-Me-Pro	G6PDD_1	G6PDD_Untreated_1	Ctrl_Untreated_1	-0.0094	-0.0034	-0.0271	0.9788	0.9840	-4.624	0.0070	False	ns
25	Mannitol/Sorbitol	G6PDD_1	G6PDD_Untreated_1	Ctrl_Untreated_1	-0.0084	-0.0030	-0.0204	0.9840	0.9840	-4.624	0.0070	False	ns
386	3-Ureidopropionate	G6PDD_1	G6PDD_Untreated_1	Ctrl_Untreated_1	0.0101	0.0037	0.0204	0.9840	0.9840	-4.624	0.0070	False	ns
112	trans-Urocanate	AT_2	AT_Untreated_2	Ctrl_Untreated_2	3.293	1.976	15.115	5.05e-09	7.77e-07	11.291	6.109	True	up
348	Allantoin	AT_2	AT_Untreated_2	Ctrl_Untreated_2	1.417	0.8500	6.444	3.63e-05	0.0028	2.351	2.554	True	up
18	Maltotriose	AT_2	AT_Untreated_2	Ctrl_Untreated_2	2.206	1.323	5.962	7.38e-05	0.0038	1.616	2.421	True	up

Metabolite ID	Name	Comparison	Group Numerator	Group Denominator	Log2 FC	Avg Expression	t	p-value	BH adj p-value	B	-Log10 BH p	Signif.	Direction
313	5-Oxoproline	AT_2	AT_Untreated_2	Ctrl_Untreated_2	1.475	0.8852	5.744	0.0001	0.0040	1.273	2.402	True	up
346	Urate	AT_2	AT_Untreated_2	Ctrl_Untreated_2	1.636	0.9818	5.251	0.0002	0.0058	0.4710	2.239	True	up
399	Pseudouridine	AT_2	AT_Untreated_2	Ctrl_Untreated_2	1.796	1.078	5.248	0.0002	0.0058	0.4657	2.239	True	up
116	3-Me-2-Oxo-Valerate	AT_2	AT_Untreated_2	Ctrl_Untreated_2	2.214	1.329	5.051	0.0003	0.0068	0.1373	2.167	True	up
19	Maltotetraose	AT_2	AT_Untreated_2	Ctrl_Untreated_2	1.218	0.7311	4.931	0.0004	0.0069	-0.0662	2.160	True	up
738	Pyridoxate	AT_2	AT_Untreated_2	Ctrl_Untreated_2	1.656	0.9935	4.882	0.0004	0.0069	-0.1505	2.160	True	up
24	Fructose	AT_2	AT_Untreated_2	Ctrl_Untreated_2	1.442	0.8655	4.776	0.0005	0.0069	-0.3322	2.160	True	up
74	Asp	AT_2	AT_Untreated_2	Ctrl_Untreated_2	-1.540	-0.9238	-4.767	0.0005	0.0069	-0.3466	2.160	True	down
241	4-Acetamidobutanoate	AT_2	AT_Untreated_2	Ctrl_Untreated_2	1.351	0.8109	4.710	0.0005	0.0070	-0.4458	2.156	True	up
376	Cytidine	AT_2	AT_Untreated_2	Ctrl_Untreated_2	2.446	1.467	4.653	0.0006	0.0071	-0.5455	2.148	True	up
177	3-(4-OH-Phenyl)Lactate	AT_2	AT_Untreated_2	Ctrl_Untreated_2	1.242	0.7453	4.481	0.0008	0.0088	-0.8448	2.054	True	up
100	4-Me-2-Oxo-Pentanoate	AT_2	AT_Untreated_2	Ctrl_Untreated_2	1.956	1.174	4.248	0.0012	0.0123	-1.257	1.909	True	up
151	Phenylacetylglycine	AT_2	AT_Untreated_2	Ctrl_Untreated_2	3.686	2.212	4.106	0.0015	0.0143	-1.511	1.844	True	up
73	Asn	AT_2	AT_Untreated_2	Ctrl_Untreated_2	-1.279	-0.7673	-4.091	0.0016	0.0143	-1.539	1.844	True	down
209	N-Ac-Ala	AT_2	AT_Untreated_2	Ctrl_Untreated_2	-0.9704	-0.5822	-3.977	0.0019	0.0158	-1.745	1.800	True	down
234	5-Me-Thioadenosine	AT_2	AT_Untreated_2	Ctrl_Untreated_2	-1.154	-0.6926	-3.972	0.0020	0.0158	-1.754	1.800	True	down
224	N-Ac-Ser	AT_2	AT_Untreated_2	Ctrl_Untreated_2	-1.433	-0.8595	-3.721	0.0031	0.0236	-2.209	1.628	True	down
206	Trans-4-OH-Pro	AT_2	AT_Untreated_2	Ctrl_Untreated_2	0.9447	0.5668	3.653	0.0035	0.0253	-2.332	1.596	True	up
237	Spermine	AT_2	AT_Untreated_2	Ctrl_Untreated_2	-1.552	-0.9314	-3.607	0.0038	0.0263	-2.416	1.581	True	down
136	Urea	AT_2	AT_Untreated_2	Ctrl_Untreated_2	0.9293	0.5576	3.543	0.0042	0.0271	-2.534	1.567	True	up
295	gamma-Glu-Phe	AT_2	AT_Untreated_2	Ctrl_Untreated_2	-0.9424	-0.5655	-3.543	0.0042	0.0271	-2.534	1.567	True	down
299	gamma-Glu-Tyr	AT_2	AT_Untreated_2	Ctrl_Untreated_2	-1.115	-0.6692	-3.460	0.0049	0.0293	-2.685	1.533	True	down
243	Creatinine	AT_2	AT_Untreated_2	Ctrl_Untreated_2	0.9846	0.5908	3.455	0.0049	0.0293	-2.694	1.533	True	up
703	NAD+	AT_2	AT_Untreated_2	Ctrl_Untreated_2	-0.9370	-0.5622	-3.376	0.0057	0.0326	-2.839	1.487	True	down
41	Lactate	AT_2	AT_Untreated_2	Ctrl_Untreated_2	0.8815	0.5289	3.293	0.0066	0.0362	-2.991	1.441	True	up
17	Maltose	AT_2	AT_Untreated_2	Ctrl_Untreated_2	1.017	0.6103	3.279	0.0068	0.0362	-3.016	1.441	True	up
314	Cys-Glutathione Disulfide	AT_2	AT_Untreated_2	Ctrl_Untreated_2	1.164	0.6981	3.149	0.0087	0.0444	-3.255	1.352	True	up
705	Coenzyme A	AT_2	AT_Untreated_2	Ctrl_Untreated_2	-1.409	-0.8455	-3.103	0.0094	0.0468	-3.339	1.330	True	down
704	NADH	AT_2	AT_Untreated_2	Ctrl_Untreated_2	-1.530	-0.9181	-3.006	0.0112	0.0541	-3.515	1.267	False	ns
710	Carnitine	AT_2	AT_Untreated_2	Ctrl_Untreated_2	-0.8004	-0.4802	-2.972	0.0120	0.0556	-3.576	1.255	False	ns

Metabolite ID	Name	Comparison	Group Numerator	Group Denominator	Log2 FC	Avg Expression	t	p-value	BH adj p-value	B	-Log10 BH p	Signif.	Direction
258	Ile-Gly	AT_2	AT_Untreated_2	Ctrl_Untreated_2	-1.488	-0.8930	-2.958	0.0123	0.0556	-3.602	1.255	False	ns
336	Adenosine	AT_2	AT_Untreated_2	Ctrl_Untreated_2	-1.898	-1.139	-2.943	0.0126	0.0556	-3.630	1.255	False	ns
76	Gln	AT_2	AT_Untreated_2	Ctrl_Untreated_2	-0.6820	-0.4092	-2.773	0.0173	0.0738	-3.937	1.132	False	ns
197	C-Glycosyl-Trp	AT_2	AT_Untreated_2	Ctrl_Untreated_2	0.9660	0.5796	2.682	0.0204	0.0849	-4.100	1.071	False	ns
102	2-Aminoadipate	AT_2	AT_Untreated_2	Ctrl_Untreated_2	-0.7369	-0.4422	-2.629	0.0224	0.0906	-4.193	1.043	False	ns
245	Ala-Phe	AT_2	AT_Untreated_2	Ctrl_Untreated_2	-0.9971	-0.5982	-2.593	0.0240	0.0906	-4.257	1.043	False	ns
183	Phenol Sulfate	AT_2	AT_Untreated_2	Ctrl_Untreated_2	1.928	1.157	2.571	0.0249	0.0906	-4.295	1.043	False	ns
275	Thr-Phe	AT_2	AT_Untreated_2	Ctrl_Untreated_2	-0.6430	-0.3858	-2.571	0.0249	0.0906	-4.296	1.043	False	ns
78	Ser	AT_2	AT_Untreated_2	Ctrl_Untreated_2	-0.9156	-0.5494	-2.570	0.0250	0.0906	-4.297	1.043	False	ns
47	Fructose 1,6-PP, Glucose 1,6-PP	AT_2	AT_Untreated_2	Ctrl_Untreated_2	-1.041	-0.6249	-2.564	0.0253	0.0906	-4.309	1.043	False	ns
85	Cys	AT_2	AT_Untreated_2	Ctrl_Untreated_2	0.9219	0.5531	2.517	0.0275	0.0964	-4.391	1.016	False	ns
316	Ophthalmate	AT_2	AT_Untreated_2	Ctrl_Untreated_2	-1.335	-0.8012	-2.474	0.0298	0.1019	-4.466	0.9918	False	ns
388	2',3'-cCMP	AT_2	AT_Untreated_2	Ctrl_Untreated_2	-1.708	-1.025	-2.439	0.0317	0.1063	-4.528	0.9735	False	ns
343	Adenine	AT_2	AT_Untreated_2	Ctrl_Untreated_2	-0.5936	-0.3562	-2.366	0.0362	0.1178	-4.654	0.9289	False	ns
273	Ser-Phe	AT_2	AT_Untreated_2	Ctrl_Untreated_2	-0.7209	-0.4325	-2.358	0.0367	0.1178	-4.667	0.9289	False	ns
335	Inosine	AT_2	AT_Untreated_2	Ctrl_Untreated_2	-0.6365	-0.3819	-2.292	0.0413	0.1299	-4.780	0.8864	False	ns
385	Uracil	AT_2	AT_Untreated_2	Ctrl_Untreated_2	1.308	0.7849	2.260	0.0438	0.1326	-4.834	0.8775	False	ns
261	Leu-Glu	AT_2	AT_Untreated_2	Ctrl_Untreated_2	-1.327	-0.7965	-2.258	0.0439	0.1326	-4.837	0.8775	False	ns
133	Ornithine	AT_2	AT_Untreated_2	Ctrl_Untreated_2	0.6777	0.4066	2.194	0.0493	0.1452	-4.946	0.8380	False	ns
268	Phe-Phe	AT_2	AT_Untreated_2	Ctrl_Untreated_2	-1.307	-0.7840	-2.186	0.0500	0.1452	-4.959	0.8380	False	ns
236	Spermidine	AT_2	AT_Untreated_2	Ctrl_Untreated_2	-1.202	-0.7212	-2.172	0.0512	0.1461	-4.982	0.8354	False	ns
79	Thr	AT_2	AT_Untreated_2	Ctrl_Untreated_2	-0.7092	-0.4255	-2.151	0.0532	0.1488	-5.017	0.8273	False	ns
390	2',3'-cUMP	AT_2	AT_Untreated_2	Ctrl_Untreated_2	-0.7782	-0.4669	-2.064	0.0620	0.1705	-5.160	0.7682	False	ns
72	Ala	AT_2	AT_Untreated_2	Ctrl_Untreated_2	-0.5898	-0.3539	-2.031	0.0656	0.1773	-5.213	0.7512	False	ns
68	Ribulose 5-P / Xylulose 5-P	AT_2	AT_Untreated_2	Ctrl_Untreated_2	-0.7336	-0.4401	-2.000	0.0693	0.1836	-5.263	0.7361	False	ns
259	Ile-Ser	AT_2	AT_Untreated_2	Ctrl_Untreated_2	-1.240	-0.7438	-1.991	0.0704	0.1836	-5.277	0.7361	False	ns
221	N-Ac-Met	AT_2	AT_Untreated_2	Ctrl_Untreated_2	-0.7590	-0.4554	-1.978	0.0720	0.1847	-5.297	0.7336	False	ns
169	2-OH-Butyrate	AT_2	AT_Untreated_2	Ctrl_Untreated_2	0.6735	0.4041	1.957	0.0747	0.1872	-5.331	0.7276	False	ns
188	Kynurenine	AT_2	AT_Untreated_2	Ctrl_Untreated_2	0.8102	0.4861	1.952	0.0754	0.1872	-5.340	0.7276	False	ns
734	Pyridoxal	AT_2	AT_Untreated_2	Ctrl_Untreated_2	0.8598	0.5159	1.932	0.0780	0.1885	-5.372	0.7248	False	ns

Metabolite ID	Name	Comparison	Group Numerator	Group Denominator	Log2 FC	Avg Expression	t	p-value	BH adj p-value	B	-Log10 BH p	Signif.	Direction
235	Putrescine	AT_2	AT_Untreated_2	Ctrl_Untreated_2	-1.377	-0.8262	-1.920	0.0797	0.1885	-5.391	0.7248	False	ns
345	Guanine	AT_2	AT_Untreated_2	Ctrl_Untreated_2	0.7015	0.4209	1.916	0.0802	0.1885	-5.396	0.7248	False	ns
77	Gly	AT_2	AT_Untreated_2	Ctrl_Untreated_2	-0.5988	-0.3593	-1.907	0.0814	0.1885	-5.410	0.7248	False	ns
308	Glutathione, Reduced	AT_2	AT_Untreated_2	Ctrl_Untreated_2	-0.8500	-0.5100	-1.902	0.0822	0.1885	-5.419	0.7248	False	ns
48	DHAP	AT_2	AT_Untreated_2	Ctrl_Untreated_2	-0.8043	-0.4826	-1.894	0.0832	0.1885	-5.430	0.7248	False	ns
725	P-Pantetheine	AT_2	AT_Untreated_2	Ctrl_Untreated_2	0.5438	0.3263	1.849	0.0899	0.2006	-5.500	0.6976	False	ns
724	Pantothenate	AT_2	AT_Untreated_2	Ctrl_Untreated_2	0.5221	0.3133	1.828	0.0933	0.2049	-5.533	0.6885	False	ns
277	Tyr-Ala	AT_2	AT_Untreated_2	Ctrl_Untreated_2	-0.9278	-0.5567	-1.820	0.0945	0.2049	-5.545	0.6885	False	ns
4	GlcNAc 6-P	AT_2	AT_Untreated_2	Ctrl_Untreated_2	-0.6060	-0.3636	-1.799	0.0980	0.2095	-5.577	0.6788	False	ns
34	Ribitol	AT_2	AT_Untreated_2	Ctrl_Untreated_2	0.7432	0.4459	1.769	0.1029	0.2167	-5.622	0.6642	False	ns
279	Val-Glu	AT_2	AT_Untreated_2	Ctrl_Untreated_2	-0.6478	-0.3887	-1.754	0.1055	0.2167	-5.644	0.6642	False	ns
139	GABA	AT_2	AT_Untreated_2	Ctrl_Untreated_2	-1.025	-0.6148	-1.747	0.1069	0.2167	-5.655	0.6642	False	ns
44	Glucose 6-P	AT_2	AT_Untreated_2	Ctrl_Untreated_2	-0.7481	-0.4489	-1.743	0.1077	0.2167	-5.662	0.6642	False	ns
737	Pyridoxine (Vitamin B6)	AT_2	AT_Untreated_2	Ctrl_Untreated_2	0.5070	0.3042	1.739	0.1083	0.2167	-5.667	0.6642	False	ns
244	Ala-Leu	AT_2	AT_Untreated_2	Ctrl_Untreated_2	-0.8888	-0.5333	-1.713	0.1132	0.2215	-5.706	0.6545	False	ns
257	Ile-Gln	AT_2	AT_Untreated_2	Ctrl_Untreated_2	-0.7602	-0.4561	-1.710	0.1137	0.2215	-5.710	0.6545	False	ns
269	Phe-Ser	AT_2	AT_Untreated_2	Ctrl_Untreated_2	-0.9443	-0.5666	-1.682	0.1191	0.2293	-5.751	0.6396	False	ns
86	Met	AT_2	AT_Untreated_2	Ctrl_Untreated_2	-0.3443	-0.2066	-1.633	0.1292	0.2457	-5.822	0.6097	False	ns
91	Pro	AT_2	AT_Untreated_2	Ctrl_Untreated_2	-0.4286	-0.2571	-1.541	0.1501	0.2808	-5.952	0.5516	False	ns
173	Met Sulfoxide	AT_2	AT_Untreated_2	Ctrl_Untreated_2	0.6097	0.3658	1.535	0.1514	0.2808	-5.959	0.5516	False	ns
272	Ser-Leu	AT_2	AT_Untreated_2	Ctrl_Untreated_2	-0.6191	-0.3715	-1.508	0.1581	0.2881	-5.996	0.5404	False	ns
110	N-alpha-Ac-Ornithine	AT_2	AT_Untreated_2	Ctrl_Untreated_2	-0.4644	-0.2786	-1.504	0.1590	0.2881	-6.001	0.5404	False	ns
51	PEP	AT_2	AT_Untreated_2	Ctrl_Untreated_2	0.8833	0.5300	1.491	0.1625	0.2910	-6.020	0.5361	False	ns
271	pyroGlu-Val	AT_2	AT_Untreated_2	Ctrl_Untreated_2	-0.5196	-0.3118	-1.479	0.1656	0.2925	-6.035	0.5338	False	ns
377	Uridine	AT_2	AT_Untreated_2	Ctrl_Untreated_2	-0.3147	-0.1888	-1.473	0.1672	0.2925	-6.043	0.5338	False	ns
344	Xanthine	AT_2	AT_Untreated_2	Ctrl_Untreated_2	1.037	0.6223	1.426	0.1801	0.3116	-6.106	0.5065	False	ns
282	Val-Leu	AT_2	AT_Untreated_2	Ctrl_Untreated_2	-0.5170	-0.3102	-1.414	0.1835	0.3122	-6.122	0.5056	False	ns
264	Leu-Leu	AT_2	AT_Untreated_2	Ctrl_Untreated_2	-0.5145	-0.3087	-1.405	0.1860	0.3122	-6.133	0.5056	False	ns
286	gamma-Glu-Glu	AT_2	AT_Untreated_2	Ctrl_Untreated_2	-0.6732	-0.4039	-1.404	0.1865	0.3122	-6.135	0.5056	False	ns
291	gamma-Glu-Leu	AT_2	AT_Untreated_2	Ctrl_Untreated_2	0.5833	0.3500	1.263	0.2314	0.3832	-6.311	0.4166	False	ns
256	Ile-Ala	AT_2	AT_Untreated_2	Ctrl_Untreated_2	-0.5168	-0.3101	-1.234	0.2413	0.3917	-6.344	0.4071	False	ns

Metabolite ID	Name	Comparison	Group Numerator	Group Denominator	Log2 FC	Avg Expression	t	p-value	BH adj p-value	B	-Log10 BH p	Signif.	Direction
203	DiMe-Arg	AT_2	AT_Untreated_2	Ctrl_Untreated_2	0.4632	0.2779	1.234	0.2416	0.3917	-6.345	0.4071	False	ns
49	3-P-Glycerate	AT_2	AT_Untreated_2	Ctrl_Untreated_2	0.5480	0.3288	1.226	0.2445	0.3922	-6.354	0.4065	False	ns
247	Asp-Phe	AT_2	AT_Untreated_2	Ctrl_Untreated_2	-0.3495	-0.2097	-1.203	0.2529	0.4015	-6.381	0.3963	False	ns
717	Nicotinamide	AT_2	AT_Untreated_2	Ctrl_Untreated_2	0.3156	0.1894	1.170	0.2655	0.4153	-6.419	0.3817	False	ns
80	His	AT_2	AT_Untreated_2	Ctrl_Untreated_2	-0.2349	-0.1409	-1.166	0.2670	0.4153	-6.423	0.3817	False	ns
302	Cyclo(Glu-Glu)	AT_2	AT_Untreated_2	Ctrl_Untreated_2	-0.4496	-0.2698	-1.143	0.2758	0.4227	-6.448	0.3739	False	ns
290	gamma-Glu-Ile	AT_2	AT_Untreated_2	Ctrl_Untreated_2	-0.5561	-0.3336	-1.140	0.2773	0.4227	-6.452	0.3739	False	ns
182	P-Cresol Sulfate	AT_2	AT_Untreated_2	Ctrl_Untreated_2	0.5366	0.3220	1.129	0.2817	0.4238	-6.464	0.3729	False	ns
45	Fructose-6-P	AT_2	AT_Untreated_2	Ctrl_Untreated_2	-0.5616	-0.3370	-1.124	0.2834	0.4238	-6.469	0.3729	False	ns
61	Malate	AT_2	AT_Untreated_2	Ctrl_Untreated_2	0.3749	0.2249	1.088	0.2987	0.4423	-6.509	0.3543	False	ns
208	Pro-OH-Pro	AT_2	AT_Untreated_2	Ctrl_Untreated_2	0.2726	0.1636	1.057	0.3120	0.4537	-6.541	0.3432	False	ns
729	Riboflavin (Vitamin B2)	AT_2	AT_Untreated_2	Ctrl_Untreated_2	0.3382	0.2029	1.056	0.3123	0.4537	-6.542	0.3432	False	ns
249	Gly-Gly	AT_2	AT_Untreated_2	Ctrl_Untreated_2	-0.5146	-0.3088	-0.9641	0.3545	0.5103	-6.634	0.2922	False	ns
253	Gly-Pro	AT_2	AT_Untreated_2	Ctrl_Untreated_2	0.2767	0.1660	0.9356	0.3684	0.5225	-6.661	0.2819	False	ns
742	Folate	AT_2	AT_Untreated_2	Ctrl_Untreated_2	0.2628	0.1577	0.9328	0.3698	0.5225	-6.664	0.2819	False	ns
373	UMP	AT_2	AT_Untreated_2	Ctrl_Untreated_2	-0.7383	-0.4430	-0.9131	0.3797	0.5316	-6.682	0.2745	False	ns
43	Glucose	AT_2	AT_Untreated_2	Ctrl_Untreated_2	0.2932	0.1759	0.8563	0.4091	0.5675	-6.733	0.2460	False	ns
329	AMP	AT_2	AT_Untreated_2	Ctrl_Untreated_2	-0.8346	-0.5008	-0.8463	0.4144	0.5698	-6.741	0.2443	False	ns
263	Leu-Gly	AT_2	AT_Untreated_2	Ctrl_Untreated_2	-0.7062	-0.4237	-0.8349	0.4205	0.5731	-6.751	0.2418	False	ns
63	6-P-Gluconate	AT_2	AT_Untreated_2	Ctrl_Untreated_2	-0.2258	-0.1355	-0.8048	0.4370	0.5903	-6.776	0.2289	False	ns
707	FMN	AT_2	AT_Untreated_2	Ctrl_Untreated_2	-0.2852	-0.1711	-0.7855	0.4478	0.5997	-6.792	0.2221	False	ns
251	Gly-Leu	AT_2	AT_Untreated_2	Ctrl_Untreated_2	-0.1992	-0.1195	-0.7207	0.4853	0.6443	-6.842	0.1909	False	ns
89	Trp	AT_2	AT_Untreated_2	Ctrl_Untreated_2	-0.1590	-0.0954	-0.6989	0.4983	0.6559	-6.858	0.1832	False	ns
36	Ribose	AT_2	AT_Untreated_2	Ctrl_Untreated_2	-0.3765	-0.2259	-0.6873	0.5053	0.6579	-6.866	0.1818	False	ns
59	Succinate	AT_2	AT_Untreated_2	Ctrl_Untreated_2	-0.2353	-0.1412	-0.6822	0.5084	0.6579	-6.870	0.1818	False	ns
20	Erythronate	AT_2	AT_Untreated_2	Ctrl_Untreated_2	0.2336	0.1402	0.6365	0.5367	0.6888	-6.901	0.1619	False	ns
55	Citrate	AT_2	AT_Untreated_2	Ctrl_Untreated_2	-0.2045	-0.1227	-0.5955	0.5629	0.7164	-6.928	0.1448	False	ns
83	Val	AT_2	AT_Untreated_2	Ctrl_Untreated_2	0.1207	0.0724	0.5652	0.5826	0.7355	-6.946	0.1334	False	ns
30	Sucrose	AT_2	AT_Untreated_2	Ctrl_Untreated_2	-0.1870	-0.1122	-0.5326	0.6043	0.7566	-6.965	0.1211	False	ns
90	Arg	AT_2	AT_Untreated_2	Ctrl_Untreated_2	-0.1305	-0.0783	-0.5095	0.6199	0.7646	-6.978	0.1166	False	ns
342	Hypoxanthine	AT_2	AT_Untreated_2	Ctrl_Untreated_2	0.1362	0.0817	0.5084	0.6206	0.7646	-6.978	0.1166	False	ns

Metabolite ID	Name	Comparison	Group Numerator	Group Denominator	Log2 FC	Avg Expression	t	p-value	BH adj p-value	B	-Log10 BH p	Signif.	Direction
250	Gly-Ile	AT_2	AT_Untreated_2	Ctrl_Untreated_2	0.2908	0.1745	0.5002	0.6262	0.7654	-6.983	0.1161	False	ns
81	Ile	AT_2	AT_Untreated_2	Ctrl_Untreated_2	0.1021	0.0613	0.4564	0.6565	0.7932	-7.005	0.1006	False	ns
70	Creatine	AT_2	AT_Untreated_2	Ctrl_Untreated_2	-0.0917	-0.0550	-0.4524	0.6593	0.7932	-7.007	0.1006	False	ns
352	3'-AMP	AT_2	AT_Untreated_2	Ctrl_Untreated_2	0.1139	0.0683	0.4425	0.6662	0.7953	-7.011	0.0995	False	ns
246	Asp-Leu	AT_2	AT_Untreated_2	Ctrl_Untreated_2	-0.1466	-0.0880	-0.4190	0.6828	0.8049	-7.022	0.0942	False	ns
740	3-Dehydrocarnitine	AT_2	AT_Untreated_2	Ctrl_Untreated_2	-0.1745	-0.1047	-0.4163	0.6847	0.8049	-7.023	0.0942	False	ns
84	Lys	AT_2	AT_Untreated_2	Ctrl_Untreated_2	0.0936	0.0561	0.3751	0.7143	0.8312	-7.041	0.0803	False	ns
82	Leu	AT_2	AT_Untreated_2	Ctrl_Untreated_2	-0.0736	-0.0442	-0.3702	0.7179	0.8312	-7.043	0.0803	False	ns
252	Gly-Phe	AT_2	AT_Untreated_2	Ctrl_Untreated_2	0.1400	0.0840	0.3453	0.7360	0.8458	-7.052	0.0727	False	ns
39	Threitol	AT_2	AT_Untreated_2	Ctrl_Untreated_2	0.1052	0.0631	0.2998	0.7696	0.8779	-7.068	0.0566	False	ns
310	S-Lactoyl-Glutathione	AT_2	AT_Untreated_2	Ctrl_Untreated_2	-0.1623	-0.0974	-0.2913	0.7759	0.8786	-7.071	0.0562	False	ns
720	1-Me-Nicotinamide	AT_2	AT_Untreated_2	Ctrl_Untreated_2	-0.0654	-0.0393	-0.2669	0.7942	0.8928	-7.078	0.0493	False	ns
145	Pyro-Gln	AT_2	AT_Untreated_2	Ctrl_Untreated_2	-0.0865	-0.0519	-0.2302	0.8219	0.9110	-7.088	0.0405	False	ns
254	Gly-Val	AT_2	AT_Untreated_2	Ctrl_Untreated_2	0.0550	0.0330	0.2252	0.8257	0.9110	-7.089	0.0405	False	ns
129	5-Aminovalerate	AT_2	AT_Untreated_2	Ctrl_Untreated_2	-0.0784	-0.0470	-0.2220	0.8282	0.9110	-7.090	0.0405	False	ns
60	Fumarate	AT_2	AT_Untreated_2	Ctrl_Untreated_2	0.0595	0.0357	0.2094	0.8378	0.9150	-7.093	0.0386	False	ns
170	2-Amino-Butyrate	AT_2	AT_Untreated_2	Ctrl_Untreated_2	-0.0628	-0.0377	-0.1935	0.8499	0.9217	-7.096	0.0354	False	ns
75	Glu	AT_2	AT_Untreated_2	Ctrl_Untreated_2	0.0399	0.0240	0.1646	0.8721	0.9392	-7.102	0.0273	False	ns
155	4-Guanidinobutanoate	AT_2	AT_Untreated_2	Ctrl_Untreated_2	-0.1037	-0.0622	-0.1549	0.8796	0.9406	-7.103	0.0266	False	ns
232	SAH	AT_2	AT_Untreated_2	Ctrl_Untreated_2	0.0311	0.0187	0.1399	0.8911	0.9464	-7.106	0.0239	False	ns
338	Guanosine	AT_2	AT_Untreated_2	Ctrl_Untreated_2	0.0324	0.0194	0.1169	0.9089	0.9587	-7.109	0.0183	False	ns
309	Glutathione, Oxidized	AT_2	AT_Untreated_2	Ctrl_Untreated_2	-0.0223	-0.0134	-0.1024	0.9202	0.9640	-7.111	0.0159	False	ns
731	Thiamin (Vitamin B1)	AT_2	AT_Untreated_2	Ctrl_Untreated_2	0.0198	0.0119	0.0720	0.9438	0.9773	-7.113	0.0100	False	ns
52	Pyruvate	AT_2	AT_Untreated_2	Ctrl_Untreated_2	0.0328	0.0197	0.0612	0.9523	0.9773	-7.114	0.0100	False	ns
274	Thr-Leu	AT_2	AT_Untreated_2	Ctrl_Untreated_2	-0.0200	-0.0120	-0.0486	0.9621	0.9773	-7.115	0.0100	False	ns
723	beta-Ala	AT_2	AT_Untreated_2	Ctrl_Untreated_2	0.0158	0.0095	0.0452	0.9647	0.9773	-7.115	0.0100	False	ns
88	Tyr	AT_2	AT_Untreated_2	Ctrl_Untreated_2	0.0079	0.0047	0.0374	0.9708	0.9773	-7.115	0.0100	False	ns
87	Phe	AT_2	AT_Untreated_2	Ctrl_Untreated_2	-0.0077	-0.0046	-0.0354	0.9724	0.9773	-7.116	0.0100	False	ns
22	N-Ac-Neuraminate	AT_2	AT_Untreated_2	Ctrl_Untreated_2	0.0072	0.0043	0.0291	0.9773	0.9773	-7.116	0.0100	False	ns
224	N-Ac-Ser	H2O2_Ctrl_2	Ctrl_H2O2_2	Ctrl_Untreated_2	-2.462	-1.231	-10.972	2.02e-06	0.0003	5.624	3.557	True	down
48	DHAP	H2O2_Ctrl_2	Ctrl_H2O2_2	Ctrl_Untreated_2	-2.766	-1.383	-8.992	1.02e-05	0.0003	3.992	3.526	True	down

Metabolite ID	Name	Comparison	Group Numerator	Group Denominator	Log2 FC	Avg Expression	t	p-value	BH adj p-value	B	-Log10 BH p	Signif.	Direction
77	Gly	H2O2_Ctrl_2	Ctrl_H2O2_2	Ctrl_Untreated_2	-2.041	-1.020	-8.860	1.15e-05	0.0003	3.871	3.526	True	down
703	NAD+	H2O2_Ctrl_2	Ctrl_H2O2_2	Ctrl_Untreated_2	-2.656	-1.328	-8.807	1.20e-05	0.0003	3.822	3.526	True	down
261	Leu-Glu	H2O2_Ctrl_2	Ctrl_H2O2_2	Ctrl_Untreated_2	-2.583	-1.292	-8.797	1.21e-05	0.0003	3.812	3.526	True	down
335	Inosine	H2O2_Ctrl_2	Ctrl_H2O2_2	Ctrl_Untreated_2	-2.139	-1.069	-8.566	1.50e-05	0.0003	3.596	3.526	True	down
308	Glutathione, Reduced	H2O2_Ctrl_2	Ctrl_H2O2_2	Ctrl_Untreated_2	-2.347	-1.173	-8.549	1.52e-05	0.0003	3.579	3.526	True	down
4	GlcNAc 6-P	H2O2_Ctrl_2	Ctrl_H2O2_2	Ctrl_Untreated_2	-1.868	-0.9342	-7.454	4.44e-05	0.0008	2.472	3.119	True	down
73	Asn	H2O2_Ctrl_2	Ctrl_H2O2_2	Ctrl_Untreated_2	-2.635	-1.318	-7.228	5.62e-05	0.0009	2.227	3.068	True	down
723	beta-Ala	H2O2_Ctrl_2	Ctrl_H2O2_2	Ctrl_Untreated_2	-2.569	-1.285	-6.796	8.96e-05	0.0012	1.740	2.928	True	down
197	C-Glycosyl-Trp	H2O2_Ctrl_2	Ctrl_H2O2_2	Ctrl_Untreated_2	-1.759	-0.8796	-6.744	9.49e-05	0.0012	1.680	2.928	True	down
91	Pro	H2O2_Ctrl_2	Ctrl_H2O2_2	Ctrl_Untreated_2	-1.438	-0.7191	-6.447	0.0001	0.0015	1.330	2.820	True	down
277	Tyr-Ala	H2O2_Ctrl_2	Ctrl_H2O2_2	Ctrl_Untreated_2	-1.981	-0.9907	-6.230	0.0002	0.0015	1.066	2.814	True	down
74	Asp	H2O2_Ctrl_2	Ctrl_H2O2_2	Ctrl_Untreated_2	-1.914	-0.9572	-6.219	0.0002	0.0015	1.053	2.814	True	down
236	Spermidine	H2O2_Ctrl_2	Ctrl_H2O2_2	Ctrl_Untreated_2	-1.498	-0.7491	-6.207	0.0002	0.0015	1.038	2.814	True	down
705	Coenzyme A	H2O2_Ctrl_2	Ctrl_H2O2_2	Ctrl_Untreated_2	-2.667	-1.334	-6.188	0.0002	0.0015	1.015	2.814	True	down
78	Ser	H2O2_Ctrl_2	Ctrl_H2O2_2	Ctrl_Untreated_2	-1.837	-0.9184	-6.073	0.0002	0.0017	0.8710	2.781	True	down
79	Thr	H2O2_Ctrl_2	Ctrl_H2O2_2	Ctrl_Untreated_2	-1.769	-0.8845	-5.950	0.0002	0.0018	0.7163	2.742	True	down
68	Ribulose 5-P / Xylulose 5-P	H2O2_Ctrl_2	Ctrl_H2O2_2	Ctrl_Untreated_2	-2.111	-1.056	-5.765	0.0003	0.0022	0.4798	2.667	True	down
173	Met Sulfoxide	H2O2_Ctrl_2	Ctrl_H2O2_2	Ctrl_Untreated_2	1.353	0.6764	5.681	0.0003	0.0022	0.3708	2.658	True	up
710	Carnitine	H2O2_Ctrl_2	Ctrl_H2O2_2	Ctrl_Untreated_2	-2.211	-1.106	-5.668	0.0003	0.0022	0.3529	2.658	True	down
720	1-Me-Nicotinamide	H2O2_Ctrl_2	Ctrl_H2O2_2	Ctrl_Untreated_2	-1.800	-0.9000	-5.550	0.0004	0.0024	0.1979	2.620	True	down
237	Spermine	H2O2_Ctrl_2	Ctrl_H2O2_2	Ctrl_Untreated_2	-1.939	-0.9696	-5.524	0.0004	0.0024	0.1639	2.620	True	down
313	5-Oxoproline	H2O2_Ctrl_2	Ctrl_H2O2_2	Ctrl_Untreated_2	1.564	0.7820	5.037	0.0008	0.0041	-0.5005	2.385	True	up
377	Uridine	H2O2_Ctrl_2	Ctrl_H2O2_2	Ctrl_Untreated_2	-0.8916	-0.4458	-5.036	0.0008	0.0041	-0.5020	2.385	True	down
47	Fructose 1,6-PP, Glucose 1,6-PP	H2O2_Ctrl_2	Ctrl_H2O2_2	Ctrl_Untreated_2	-1.676	-0.8382	-5.015	0.0008	0.0041	-0.5318	2.385	True	down
707	FMN	H2O2_Ctrl_2	Ctrl_H2O2_2	Ctrl_Untreated_2	-1.051	-0.5255	-4.884	0.0009	0.0047	-0.7163	2.331	True	down
343	Adenine	H2O2_Ctrl_2	Ctrl_H2O2_2	Ctrl_Untreated_2	-1.557	-0.7785	-4.867	0.0010	0.0047	-0.7409	2.331	True	down
116	3-Me-2-Oxo-Valerate	H2O2_Ctrl_2	Ctrl_H2O2_2	Ctrl_Untreated_2	1.808	0.9038	4.823	0.0010	0.0047	-0.8032	2.327	True	up
221	N-Ac-Met	H2O2_Ctrl_2	Ctrl_H2O2_2	Ctrl_Untreated_2	-1.729	-0.8647	-4.810	0.0010	0.0047	-0.8221	2.327	True	down
19	Maltotetraose	H2O2_Ctrl_2	Ctrl_H2O2_2	Ctrl_Untreated_2	1.316	0.6579	4.637	0.0013	0.0058	-1.073	2.238	True	up
234	5-Me-Thioadenosine	H2O2_Ctrl_2	Ctrl_H2O2_2	Ctrl_Untreated_2	-1.385	-0.6925	-4.610	0.0014	0.0058	-1.112	2.235	True	down

Metabolite ID	Name	Comparison	Group Numerator	Group Denominator	Log2 FC	Avg Expression	t	p-value	BH adj p-value	B	-Log10 BH p	Signif.	Direction
72	Ala	H2O2_Ctrl_2	Ctrl_H2O2_2	Ctrl_Untreated_2	-1.602	-0.8009	-4.290	0.0021	0.0089	-1.587	2.051	True	down
314	Cys-Glutathione Disulfide	H2O2_Ctrl_2	Ctrl_H2O2_2	Ctrl_Untreated_2	1.751	0.8757	4.244	0.0023	0.0092	-1.656	2.035	True	up
241	4-Acetamidobutanoate	H2O2_Ctrl_2	Ctrl_H2O2_2	Ctrl_Untreated_2	1.278	0.6390	4.171	0.0025	0.0100	-1.768	2.002	True	up
22	N-Ac-Neuraminate	H2O2_Ctrl_2	Ctrl_H2O2_2	Ctrl_Untreated_2	-1.129	-0.5644	-4.149	0.0026	0.0100	-1.801	2.000	True	down
388	2',3'-cCMP	H2O2_Ctrl_2	Ctrl_H2O2_2	Ctrl_Untreated_2	-1.245	-0.6227	-4.009	0.0032	0.0120	-2.015	1.923	True	down
247	Asp-Phe	H2O2_Ctrl_2	Ctrl_H2O2_2	Ctrl_Untreated_2	-0.9441	-0.4721	-3.945	0.0036	0.0128	-2.116	1.892	True	down
338	Guanosine	H2O2_Ctrl_2	Ctrl_H2O2_2	Ctrl_Untreated_2	-1.042	-0.5212	-3.892	0.0038	0.0133	-2.198	1.875	True	down
70	Creatine	H2O2_Ctrl_2	Ctrl_H2O2_2	Ctrl_Untreated_2	-0.7745	-0.3872	-3.884	0.0039	0.0133	-2.210	1.875	True	down
272	Ser-Leu	H2O2_Ctrl_2	Ctrl_H2O2_2	Ctrl_Untreated_2	-1.858	-0.9292	-3.787	0.0045	0.0151	-2.362	1.822	True	down
36	Ribose	H2O2_Ctrl_2	Ctrl_H2O2_2	Ctrl_Untreated_2	-1.765	-0.8823	-3.717	0.0050	0.0163	-2.471	1.787	True	down
742	Folate	H2O2_Ctrl_2	Ctrl_H2O2_2	Ctrl_Untreated_2	0.7944	0.3972	3.612	0.0059	0.0187	-2.637	1.728	True	up
43	Glucose	H2O2_Ctrl_2	Ctrl_H2O2_2	Ctrl_Untreated_2	0.9696	0.4848	3.559	0.0064	0.0199	-2.723	1.702	True	up
102	2-Aminoadipate	H2O2_Ctrl_2	Ctrl_H2O2_2	Ctrl_Untreated_2	-1.563	-0.7817	-3.526	0.0067	0.0204	-2.774	1.690	True	down
737	Pyridoxine (Vitamin B6)	H2O2_Ctrl_2	Ctrl_H2O2_2	Ctrl_Untreated_2	0.9203	0.4601	3.458	0.0075	0.0222	-2.883	1.653	True	up
17	Maltose	H2O2_Ctrl_2	Ctrl_H2O2_2	Ctrl_Untreated_2	1.088	0.5441	3.433	0.0078	0.0226	-2.923	1.646	True	up
740	3-Dehydrocarnitine	H2O2_Ctrl_2	Ctrl_H2O2_2	Ctrl_Untreated_2	-1.237	-0.6186	-3.343	0.0089	0.0255	-3.068	1.594	True	down
254	Gly-Val	H2O2_Ctrl_2	Ctrl_H2O2_2	Ctrl_Untreated_2	-0.8338	-0.4169	-3.277	0.0099	0.0273	-3.174	1.564	True	down
151	Phenylacetylglycine	H2O2_Ctrl_2	Ctrl_H2O2_2	Ctrl_Untreated_2	2.078	1.039	3.274	0.0099	0.0273	-3.179	1.564	True	up
345	Guanine	H2O2_Ctrl_2	Ctrl_H2O2_2	Ctrl_Untreated_2	1.309	0.6546	3.242	0.0105	0.0281	-3.231	1.551	True	up
718	Nicotinamide Riboside	H2O2_Ctrl_2	Ctrl_H2O2_2	Ctrl_Untreated_2	1.910	0.9550	3.086	0.0134	0.0347	-3.484	1.459	True	up
112	trans-Urocanate	H2O2_Ctrl_2	Ctrl_H2O2_2	Ctrl_Untreated_2	2.581	1.291	3.084	0.0135	0.0347	-3.487	1.459	True	up
279	Val-Glu	H2O2_Ctrl_2	Ctrl_H2O2_2	Ctrl_Untreated_2	-0.8650	-0.4325	-3.073	0.0137	0.0347	-3.505	1.459	True	down
29	Raffinose	H2O2_Ctrl_2	Ctrl_H2O2_2	Ctrl_Untreated_2	-1.720	-0.8600	-2.868	0.0190	0.0473	-3.838	1.325	True	down
89	Trp	H2O2_Ctrl_2	Ctrl_H2O2_2	Ctrl_Untreated_2	-0.5329	-0.2665	-2.852	0.0195	0.0477	-3.864	1.321	True	down
235	Putrescine	H2O2_Ctrl_2	Ctrl_H2O2_2	Ctrl_Untreated_2	-1.973	-0.9865	-2.818	0.0206	0.0495	-3.919	1.305	True	down
269	Phe-Ser	H2O2_Ctrl_2	Ctrl_H2O2_2	Ctrl_Untreated_2	-1.348	-0.6742	-2.760	0.0226	0.0534	-4.013	1.272	False	ns
253	Gly-Pro	H2O2_Ctrl_2	Ctrl_H2O2_2	Ctrl_Untreated_2	-0.6301	-0.3150	-2.592	0.0297	0.0690	-4.286	1.161	False	ns
18	Maltotriose	H2O2_Ctrl_2	Ctrl_H2O2_2	Ctrl_Untreated_2	1.088	0.5440	2.484	0.0354	0.0808	-4.459	1.092	False	ns
273	Ser-Phe	H2O2_Ctrl_2	Ctrl_H2O2_2	Ctrl_Untreated_2	-0.8017	-0.4009	-2.419	0.0393	0.0884	-4.564	1.054	False	ns
86	Met	H2O2_Ctrl_2	Ctrl_H2O2_2	Ctrl_Untreated_2	-0.5034	-0.2517	-2.365	0.0430	0.0949	-4.650	1.023	False	ns

Metabolite ID	Name	Comparison	Group Numerator	Group Denominator	Log2 FC	Avg Expression	t	p-value	BH adj p-value	B	-Log10 BH p	Signif.	Direction
299	gamma-Glu-Tyr	H2O2_Ctrl_2	Ctrl_H2O2_2	Ctrl_Untreated_2	-1.051	-0.5256	-2.163	0.0596	0.1295	-4.967	0.8876	False	ns
88	Tyr	H2O2_Ctrl_2	Ctrl_H2O2_2	Ctrl_Untreated_2	-0.3844	-0.1922	-2.128	0.0630	0.1349	-5.021	0.8700	False	ns
263	Leu-Gly	H2O2_Ctrl_2	Ctrl_H2O2_2	Ctrl_Untreated_2	-1.510	-0.7551	-2.118	0.0640	0.1349	-5.037	0.8699	False	ns
336	Adenosine	H2O2_Ctrl_2	Ctrl_H2O2_2	Ctrl_Untreated_2	-1.369	-0.6843	-2.101	0.0658	0.1365	-5.062	0.8649	False	ns
258	Ile-Gly	H2O2_Ctrl_2	Ctrl_H2O2_2	Ctrl_Untreated_2	-1.447	-0.7235	-2.059	0.0704	0.1440	-5.127	0.8418	False	ns
251	Gly-Leu	H2O2_Ctrl_2	Ctrl_H2O2_2	Ctrl_Untreated_2	-0.5095	-0.2548	-2.007	0.0765	0.1541	-5.206	0.8122	False	ns
738	Pyridoxate	H2O2_Ctrl_2	Ctrl_H2O2_2	Ctrl_Untreated_2	1.276	0.6382	1.966	0.0817	0.1623	-5.269	0.7897	False	ns
82	Leu	H2O2_Ctrl_2	Ctrl_H2O2_2	Ctrl_Untreated_2	-0.3557	-0.1779	-1.926	0.0870	0.1703	-5.328	0.7688	False	ns
85	Cys	H2O2_Ctrl_2	Ctrl_H2O2_2	Ctrl_Untreated_2	-0.6947	-0.3474	-1.906	0.0898	0.1725	-5.357	0.7631	False	ns
133	Ornithine	H2O2_Ctrl_2	Ctrl_H2O2_2	Ctrl_Untreated_2	-0.9838	-0.4919	-1.900	0.0907	0.1725	-5.366	0.7631	False	ns
243	Creatinine	H2O2_Ctrl_2	Ctrl_H2O2_2	Ctrl_Untreated_2	0.7249	0.3625	1.883	0.0933	0.1751	-5.393	0.7568	False	ns
717	Nicotinamide	H2O2_Ctrl_2	Ctrl_H2O2_2	Ctrl_Untreated_2	0.4551	0.2275	1.866	0.0957	0.1760	-5.417	0.7546	False	ns
342	Hypoxanthine	H2O2_Ctrl_2	Ctrl_H2O2_2	Ctrl_Untreated_2	-0.5321	-0.2661	-1.862	0.0963	0.1760	-5.423	0.7546	False	ns
60	Fumarate	H2O2_Ctrl_2	Ctrl_H2O2_2	Ctrl_Untreated_2	-0.8421	-0.4210	-1.789	0.1081	0.1929	-5.530	0.7147	False	ns
100	4-Me-2-Oxo-Pentanoate	H2O2_Ctrl_2	Ctrl_H2O2_2	Ctrl_Untreated_2	0.7793	0.3897	1.779	0.1097	0.1929	-5.543	0.7147	False	ns
352	3'-AMP	H2O2_Ctrl_2	Ctrl_H2O2_2	Ctrl_Untreated_2	-0.4778	-0.2389	-1.772	0.1110	0.1929	-5.554	0.7147	False	ns
61	Malate	H2O2_Ctrl_2	Ctrl_H2O2_2	Ctrl_Untreated_2	-0.8380	-0.4190	-1.771	0.1112	0.1929	-5.556	0.7147	False	ns
286	gamma-Glu-Glu	H2O2_Ctrl_2	Ctrl_H2O2_2	Ctrl_Untreated_2	-0.6591	-0.3296	-1.752	0.1145	0.1944	-5.582	0.7113	False	ns
87	Phe	H2O2_Ctrl_2	Ctrl_H2O2_2	Ctrl_Untreated_2	-0.3250	-0.1625	-1.750	0.1149	0.1944	-5.586	0.7113	False	ns
188	Kynurenine	H2O2_Ctrl_2	Ctrl_H2O2_2	Ctrl_Untreated_2	-0.8928	-0.4464	-1.707	0.1228	0.2052	-5.646	0.6879	False	ns
155	4-Guanidinobutanoate	H2O2_Ctrl_2	Ctrl_H2O2_2	Ctrl_Untreated_2	-0.8984	-0.4492	-1.656	0.1330	0.2195	-5.718	0.6585	False	ns
75	Glu	H2O2_Ctrl_2	Ctrl_H2O2_2	Ctrl_Untreated_2	-0.4009	-0.2004	-1.633	0.1377	0.2245	-5.749	0.6487	False	ns
24	Fructose	H2O2_Ctrl_2	Ctrl_H2O2_2	Ctrl_Untreated_2	0.8078	0.4039	1.610	0.1428	0.2289	-5.782	0.6404	False	ns
282	Val-Leu	H2O2_Ctrl_2	Ctrl_H2O2_2	Ctrl_Untreated_2	-0.6341	-0.3171	-1.598	0.1455	0.2289	-5.799	0.6404	False	ns
725	P-Pantetheine	H2O2_Ctrl_2	Ctrl_H2O2_2	Ctrl_Untreated_2	0.6142	0.3071	1.597	0.1457	0.2289	-5.800	0.6404	False	ns
303	Cyclo(Leu-Pro)	H2O2_Ctrl_2	Ctrl_H2O2_2	Ctrl_Untreated_2	-0.4755	-0.2378	-1.591	0.1470	0.2289	-5.808	0.6404	False	ns
63	6-P-Gluconate	H2O2_Ctrl_2	Ctrl_H2O2_2	Ctrl_Untreated_2	0.7847	0.3923	1.579	0.1496	0.2303	-5.824	0.6376	False	ns
80	His	H2O2_Ctrl_2	Ctrl_H2O2_2	Ctrl_Untreated_2	-0.2967	-0.1484	-1.527	0.1619	0.2464	-5.893	0.6083	False	ns
309	Glutathione, Oxidized	H2O2_Ctrl_2	Ctrl_H2O2_2	Ctrl_Untreated_2	-0.3978	-0.1989	-1.516	0.1648	0.2480	-5.909	0.6055	False	ns
203	DiMe-Arg	H2O2_Ctrl_2	Ctrl_H2O2_2	Ctrl_Untreated_2	0.6993	0.3496	1.440	0.1847	0.2750	-6.008	0.5606	False	ns
316	Ophthalmate	H2O2_Ctrl_2	Ctrl_H2O2_2	Ctrl_Untreated_2	-1.131	-0.5653	-1.428	0.1878	0.2767	-6.023	0.5580	False	ns

Metabolite ID	Name	Comparison	Group Numerator	Group Denominator	Log2 FC	Avg Expression	t	p-value	BH adj p-value	B	-Log10 BH p	Signif.	Direction
59	Succinate	H2O2_Ctrl_2	Ctrl_H2O2_2	Ctrl_Untreated_2	-0.4883	-0.2442	-1.392	0.1983	0.2875	-6.069	0.5413	False	ns
731	Thiamin (Vitamin B1)	H2O2_Ctrl_2	Ctrl_H2O2_2	Ctrl_Untreated_2	0.3860	0.1930	1.388	0.1994	0.2875	-6.074	0.5413	False	ns
136	Urea	H2O2_Ctrl_2	Ctrl_H2O2_2	Ctrl_Untreated_2	0.6052	0.3026	1.345	0.2125	0.3032	-6.128	0.5182	False	ns
290	gamma-Glu-Ile	H2O2_Ctrl_2	Ctrl_H2O2_2	Ctrl_Untreated_2	-0.7492	-0.3746	-1.328	0.2175	0.3072	-6.147	0.5125	False	ns
348	Allantoin	H2O2_Ctrl_2	Ctrl_H2O2_2	Ctrl_Untreated_2	0.6449	0.3225	1.229	0.2511	0.3510	-6.266	0.4547	False	ns
30	Sucrose	H2O2_Ctrl_2	Ctrl_H2O2_2	Ctrl_Untreated_2	-0.3692	-0.1846	-1.180	0.2690	0.3722	-6.322	0.4292	False	ns
268	Phe-Phe	H2O2_Ctrl_2	Ctrl_H2O2_2	Ctrl_Untreated_2	-0.9070	-0.4535	-1.168	0.2734	0.3746	-6.335	0.4265	False	ns
302	Cyclo(Glu-Glu)	H2O2_Ctrl_2	Ctrl_H2O2_2	Ctrl_Untreated_2	0.3496	0.1748	1.157	0.2777	0.3767	-6.347	0.4240	False	ns
399	Pseudouridine	H2O2_Ctrl_2	Ctrl_H2O2_2	Ctrl_Untreated_2	0.5665	0.2832	1.125	0.2903	0.3899	-6.382	0.4090	False	ns
34	Ribitol	H2O2_Ctrl_2	Ctrl_H2O2_2	Ctrl_Untreated_2	-0.5941	-0.2971	-1.103	0.2992	0.3955	-6.406	0.4029	False	ns
344	Xanthine	H2O2_Ctrl_2	Ctrl_H2O2_2	Ctrl_Untreated_2	-0.5877	-0.2938	-1.101	0.3002	0.3955	-6.409	0.4029	False	ns
145	Pyro-Gln	H2O2_Ctrl_2	Ctrl_H2O2_2	Ctrl_Untreated_2	0.4337	0.2168	1.085	0.3068	0.4003	-6.425	0.3976	False	ns
232	SAH	H2O2_Ctrl_2	Ctrl_H2O2_2	Ctrl_Untreated_2	-0.2542	-0.1271	-1.038	0.3271	0.4197	-6.474	0.3770	False	ns
256	Ile-Ala	H2O2_Ctrl_2	Ctrl_H2O2_2	Ctrl_Untreated_2	-0.7514	-0.3757	-1.036	0.3278	0.4197	-6.476	0.3770	False	ns
110	N-alpha-Ac-Ornithine	H2O2_Ctrl_2	Ctrl_H2O2_2	Ctrl_Untreated_2	-0.2169	-0.1085	-0.9761	0.3551	0.4505	-6.536	0.3463	False	ns
76	Gln	H2O2_Ctrl_2	Ctrl_H2O2_2	Ctrl_Untreated_2	0.2635	0.1318	0.9341	0.3752	0.4716	-6.576	0.3264	False	ns
182	P-Cresol Sulfate	H2O2_Ctrl_2	Ctrl_H2O2_2	Ctrl_Untreated_2	-0.2647	-0.1323	-0.6918	0.5069	0.6216	-6.778	0.2065	False	ns
246	Asp-Leu	H2O2_Ctrl_2	Ctrl_H2O2_2	Ctrl_Untreated_2	-0.2160	-0.1080	-0.6909	0.5075	0.6216	-6.779	0.2065	False	ns
44	Glucose 6-P	H2O2_Ctrl_2	Ctrl_H2O2_2	Ctrl_Untreated_2	-0.4066	-0.2033	-0.6898	0.5082	0.6216	-6.780	0.2065	False	ns
291	gamma-Glu-Leu	H2O2_Ctrl_2	Ctrl_H2O2_2	Ctrl_Untreated_2	-0.4025	-0.2013	-0.6762	0.5163	0.6260	-6.789	0.2034	False	ns
84	Lys	H2O2_Ctrl_2	Ctrl_H2O2_2	Ctrl_Untreated_2	0.1744	0.0872	0.6681	0.5213	0.6265	-6.795	0.2031	False	ns
376	Cytidine	H2O2_Ctrl_2	Ctrl_H2O2_2	Ctrl_Untreated_2	0.5223	0.2612	0.6231	0.5491	0.6447	-6.826	0.1906	False	ns
20	Erythronate	H2O2_Ctrl_2	Ctrl_H2O2_2	Ctrl_Untreated_2	-0.2804	-0.1402	-0.6218	0.5499	0.6447	-6.827	0.1906	False	ns
208	Pro-OH-Pro	H2O2_Ctrl_2	Ctrl_H2O2_2	Ctrl_Untreated_2	0.1606	0.0803	0.6206	0.5506	0.6447	-6.827	0.1906	False	ns
390	2',3'-cUMP	H2O2_Ctrl_2	Ctrl_H2O2_2	Ctrl_Untreated_2	-0.2083	-0.1042	-0.5971	0.5655	0.6566	-6.843	0.1827	False	ns
724	Pantothenate	H2O2_Ctrl_2	Ctrl_H2O2_2	Ctrl_Untreated_2	0.1530	0.0765	0.5548	0.5929	0.6826	-6.869	0.1658	False	ns
177	3-(4-OH-Phenyl)Lactate	H2O2_Ctrl_2	Ctrl_H2O2_2	Ctrl_Untreated_2	0.1519	0.0760	0.4611	0.6559	0.7488	-6.920	0.1256	False	ns
93	3-P-Ser	H2O2_Ctrl_2	Ctrl_H2O2_2	Ctrl_Untreated_2	0.5448	0.2724	0.4467	0.6659	0.7540	-6.927	0.1227	False	ns
206	Trans-4-OH-Pro	H2O2_Ctrl_2	Ctrl_H2O2_2	Ctrl_Untreated_2	0.2078	0.1039	0.4257	0.6806	0.7643	-6.937	0.1168	False	ns
385	Uracil	H2O2_Ctrl_2	Ctrl_H2O2_2	Ctrl_Untreated_2	0.1744	0.0872	0.3959	0.7016	0.7815	-6.950	0.1071	False	ns
729	Riboflavin (Vitamin B2)	H2O2_Ctrl_2	Ctrl_H2O2_2	Ctrl_Untreated_2	0.0890	0.0445	0.3862	0.7085	0.7828	-6.954	0.1063	False	ns

Metabolite ID	Name	Comparison	Group Numerator	Group Denominator	Log2 FC	Avg Expression	t	p-value	BH adj p-value	B	-Log10 BH p	Signif.	Direction
45	Fructose-6-P	H2O2_Ctrl_2	Ctrl_H2O2_2	Ctrl_Untreated_2	-0.2153	-0.1076	-0.3648	0.7239	0.7934	-6.963	0.1005	False	ns
41	Lactate	H2O2_Ctrl_2	Ctrl_H2O2_2	Ctrl_Untreated_2	-0.1288	-0.0644	-0.3520	0.7332	0.7972	-6.968	0.0985	False	ns
170	2-Amino-Butyrate	H2O2_Ctrl_2	Ctrl_H2O2_2	Ctrl_Untreated_2	-0.1220	-0.0610	-0.3016	0.7700	0.8306	-6.986	0.0806	False	ns
346	Urate	H2O2_Ctrl_2	Ctrl_H2O2_2	Ctrl_Untreated_2	0.1126	0.0563	0.2932	0.7762	0.8308	-6.988	0.0805	False	ns
90	Arg	H2O2_Ctrl_2	Ctrl_H2O2_2	Ctrl_Untreated_2	-0.0670	-0.0335	-0.2807	0.7855	0.8342	-6.992	0.0787	False	ns
252	Gly-Phe	H2O2_Ctrl_2	Ctrl_H2O2_2	Ctrl_Untreated_2	-0.0996	-0.0498	-0.2710	0.7927	0.8353	-6.995	0.0781	False	ns
55	Citrate	H2O2_Ctrl_2	Ctrl_H2O2_2	Ctrl_Untreated_2	-0.0608	-0.0304	-0.2210	0.8301	0.8682	-7.009	0.0614	False	ns
271	pyroGlu-Val	H2O2_Ctrl_2	Ctrl_H2O2_2	Ctrl_Untreated_2	0.1038	0.0519	0.1946	0.8501	0.8823	-7.015	0.0544	False	ns
81	Ile	H2O2_Ctrl_2	Ctrl_H2O2_2	Ctrl_Untreated_2	0.0365	0.0182	0.1786	0.8623	0.8883	-7.018	0.0515	False	ns
83	Val	H2O2_Ctrl_2	Ctrl_H2O2_2	Ctrl_Untreated_2	-0.0319	-0.0159	-0.1522	0.8825	0.9022	-7.023	0.0447	False	ns
51	PEP	H2O2_Ctrl_2	Ctrl_H2O2_2	Ctrl_Untreated_2	0.0697	0.0348	0.1085	0.9161	0.9256	-7.029	0.0336	False	ns
734	Pyridoxal	H2O2_Ctrl_2	Ctrl_H2O2_2	Ctrl_Untreated_2	-0.0363	-0.0181	-0.1049	0.9188	0.9256	-7.030	0.0336	False	ns
49	3-P-Glycerate	H2O2_Ctrl_2	Ctrl_H2O2_2	Ctrl_Untreated_2	-0.0315	-0.0157	-0.0674	0.9478	0.9478	-7.033	0.0233	False	ns
703	NAD+	H2O2_AT_2	AT_H2O2_2	AT_Untreated_2	-0.9483	-1.411	-3.672	0.0027	0.1645	-1.987	0.7837	False	ns
335	Inosine	H2O2_AT_2	AT_H2O2_2	AT_Untreated_2	-0.9610	-1.117	-3.660	0.0027	0.1645	-2.001	0.7837	False	ns
343	Adenine	H2O2_AT_2	AT_H2O2_2	AT_Untreated_2	-0.9893	-1.088	-3.555	0.0034	0.1645	-2.118	0.7837	False	ns
346	Urate	H2O2_AT_2	AT_H2O2_2	AT_Untreated_2	-1.004	1.134	-3.042	0.0091	0.2331	-2.713	0.6325	False	ns
22	N-Ac-Neuraminate	H2O2_AT_2	AT_H2O2_2	AT_Untreated_2	-0.8204	-0.4030	-2.892	0.0122	0.2331	-2.889	0.6325	False	ns
338	Guanosine	H2O2_AT_2	AT_H2O2_2	AT_Untreated_2	-0.9083	-0.4218	-2.772	0.0155	0.2331	-3.032	0.6325	False	ns
91	Pro	H2O2_AT_2	AT_H2O2_2	AT_Untreated_2	-0.6037	-0.7304	-2.744	0.0163	0.2331	-3.065	0.6325	False	ns
352	3'-AMP	H2O2_AT_2	AT_H2O2_2	AT_Untreated_2	-0.6587	-0.2155	-2.736	0.0166	0.2331	-3.074	0.6325	False	ns
295	gamma-Glu-Phe	H2O2_AT_2	AT_H2O2_2	AT_Untreated_2	-0.6321	-1.259	-2.690	0.0181	0.2331	-3.129	0.6325	False	ns
41	Lactate	H2O2_AT_2	AT_H2O2_2	AT_Untreated_2	-0.5989	0.5821	-2.679	0.0185	0.2331	-3.142	0.6325	False	ns
68	Ribulose 5-P / Xylulose 5-P	H2O2_AT_2	AT_H2O2_2	AT_Untreated_2	-0.8402	-1.154	-2.665	0.0190	0.2331	-3.159	0.6325	False	ns
102	2-Aminoadipate	H2O2_AT_2	AT_H2O2_2	AT_Untreated_2	-0.7026	-1.088	-2.664	0.0190	0.2331	-3.159	0.6325	False	ns
348	Allantoin	H2O2_AT_2	AT_H2O2_2	AT_Untreated_2	-0.4704	1.181	-2.515	0.0254	0.2868	-3.336	0.5425	False	ns
279	Val-Glu	H2O2_AT_2	AT_H2O2_2	AT_Untreated_2	-0.9231	-1.109	-2.405	0.0312	0.3064	-3.464	0.5137	False	ns
188	Kynurenine	H2O2_AT_2	AT_H2O2_2	AT_Untreated_2	-0.9857	0.3174	-2.404	0.0313	0.3064	-3.466	0.5137	False	ns
48	DHAP	H2O2_AT_2	AT_H2O2_2	AT_Untreated_2	-0.7953	-1.202	-2.330	0.0360	0.3160	-3.552	0.5004	False	ns
77	Gly	H2O2_AT_2	AT_H2O2_2	AT_Untreated_2	-0.6872	-0.9424	-2.319	0.0367	0.3160	-3.565	0.5004	False	ns
49	3-P-Glycerate	H2O2_AT_2	AT_H2O2_2	AT_Untreated_2	-0.6389	0.2285	-2.264	0.0408	0.3160	-3.629	0.5004	False	ns

Metabolite ID	Name	Comparison	Group Numerator	Group Denominator	Log2 FC	Avg Expression	t	p-value	BH adj p-value	B	-Log10 BH p	Signif.	Direction
197	C-Glycosyl-Trp	H2O2_AT_2	AT_H2O2_2	AT_Untreated_2	-0.9261	0.5030	-2.259	0.0411	0.3160	-3.635	0.5004	False	ns
155	4-Guanidinobutanoate	H2O2_AT_2	AT_H2O2_2	AT_Untreated_2	-1.178	-0.6928	-2.235	0.0430	0.3160	-3.662	0.5004	False	ns
720	1-Me-Nicotinamide	H2O2_AT_2	AT_H2O2_2	AT_Untreated_2	-0.5587	-0.3448	-2.160	0.0494	0.3403	-3.748	0.4682	False	ns
710	Carnitine	H2O2_AT_2	AT_H2O2_2	AT_Untreated_2	-0.6141	-1.107	-2.136	0.0516	0.3403	-3.775	0.4682	False	ns
72	Ala	H2O2_AT_2	AT_H2O2_2	AT_Untreated_2	-0.4241	-0.8019	-2.119	0.0532	0.3403	-3.794	0.4682	False	ns
734	Pyridoxal	H2O2_AT_2	AT_H2O2_2	AT_Untreated_2	-0.8684	0.4256	-2.039	0.0616	0.3677	-3.884	0.4345	False	ns
723	beta-Ala	H2O2_AT_2	AT_H2O2_2	AT_Untreated_2	-0.8847	-0.4265	-2.006	0.0655	0.3677	-3.921	0.4345	False	ns
45	Fructose-6-P	H2O2_AT_2	AT_H2O2_2	AT_Untreated_2	0.8638	-0.1297	2.005	0.0656	0.3677	-3.922	0.4345	False	ns
182	P-Cresol Sulfate	H2O2_AT_2	AT_H2O2_2	AT_Untreated_2	1.140	1.107	1.988	0.0675	0.3677	-3.941	0.4345	False	ns
272	Ser-Leu	H2O2_AT_2	AT_H2O2_2	AT_Untreated_2	-0.5938	-0.9160	-1.933	0.0746	0.3867	-4.002	0.4126	False	ns
342	Hypoxanthine	H2O2_AT_2	AT_H2O2_2	AT_Untreated_2	-0.4859	-0.1068	-1.920	0.0763	0.3867	-4.015	0.4126	False	ns
308	Glutathione, Reduced	H2O2_AT_2	AT_H2O2_2	AT_Untreated_2	-0.7916	-1.246	-1.839	0.0882	0.4320	-4.103	0.3645	False	ns
740	3-Dehydrocarnitine	H2O2_AT_2	AT_H2O2_2	AT_Untreated_2	-0.8415	-0.5953	-1.780	0.0977	0.4329	-4.166	0.3636	False	ns
263	Leu-Gly	H2O2_AT_2	AT_H2O2_2	AT_Untreated_2	-1.124	-1.268	-1.773	0.0989	0.4329	-4.173	0.3636	False	ns
221	N-Ac-Met	H2O2_AT_2	AT_H2O2_2	AT_Untreated_2	-0.6354	-1.077	-1.767	0.0999	0.4329	-4.179	0.3636	False	ns
75	Glu	H2O2_AT_2	AT_H2O2_2	AT_Untreated_2	-0.3209	-0.1205	-1.753	0.1024	0.4329	-4.194	0.3636	False	ns
249	Gly-Gly	H2O2_AT_2	AT_H2O2_2	AT_Untreated_2	-0.7649	-0.8971	-1.743	0.1042	0.4329	-4.204	0.3636	False	ns
47	Fructose 1,6-PP, Glucose 1,6-PP	H2O2_AT_2	AT_H2O2_2	AT_Untreated_2	-0.5679	-1.325	-1.733	0.1060	0.4329	-4.215	0.3636	False	ns
133	Ornithine	H2O2_AT_2	AT_H2O2_2	AT_Untreated_2	-0.4119	0.4717	-1.711	0.1100	0.4369	-4.237	0.3596	False	ns
63	6-P-Gluconate	H2O2_AT_2	AT_H2O2_2	AT_Untreated_2	0.6209	0.0846	1.636	0.1251	0.4838	-4.314	0.3154	False	ns
177	3-(4-OH-Phenyl)Lactate	H2O2_AT_2	AT_H2O2_2	AT_Untreated_2	-0.3590	1.063	-1.618	0.1290	0.4861	-4.332	0.3133	False	ns
36	Ribose	H2O2_AT_2	AT_H2O2_2	AT_Untreated_2	-0.8577	-0.8053	-1.546	0.1454	0.5318	-4.403	0.2743	False	ns
79	Thr	H2O2_AT_2	AT_H2O2_2	AT_Untreated_2	-0.4263	-0.9224	-1.533	0.1484	0.5318	-4.415	0.2743	False	ns
254	Gly-Val	H2O2_AT_2	AT_H2O2_2	AT_Untreated_2	-0.4585	-0.1743	-1.490	0.1592	0.5318	-4.456	0.2743	False	ns
329	AMP	H2O2_AT_2	AT_H2O2_2	AT_Untreated_2	-1.397	-1.533	-1.487	0.1601	0.5318	-4.460	0.2743	False	ns
258	Ile-Gly	H2O2_AT_2	AT_H2O2_2	AT_Untreated_2	-0.7622	-1.869	-1.471	0.1643	0.5318	-4.475	0.2743	False	ns
247	Asp-Phe	H2O2_AT_2	AT_H2O2_2	AT_Untreated_2	-0.4104	-0.5547	-1.457	0.1681	0.5318	-4.488	0.2743	False	ns
61	Malate	H2O2_AT_2	AT_H2O2_2	AT_Untreated_2	-0.5268	0.1115	-1.456	0.1685	0.5318	-4.489	0.2743	False	ns
51	PEP	H2O2_AT_2	AT_H2O2_2	AT_Untreated_2	-0.5199	0.6234	-1.450	0.1700	0.5318	-4.495	0.2743	False	ns
256	Ile-Ala	H2O2_AT_2	AT_H2O2_2	AT_Untreated_2	-0.5980	-0.8158	-1.331	0.2053	0.6209	-4.603	0.2070	False	ns

Metabolite ID	Name	Comparison	Group Numerator	Group Denominator	Log2 FC	Avg Expression	t	p-value	BH adj p-value	B	-Log10 BH p	Signif.	Direction
291	gamma-Glu-Leu	H2O2_AT_2	AT_H2O2_2	AT_Untreated_2	-0.4943	0.3361	-1.326	0.2070	0.6209	-4.607	0.2070	False	ns
44	Glucose 6-P	H2O2_AT_2	AT_H2O2_2	AT_Untreated_2	0.5168	-0.4897	1.302	0.2147	0.6314	-4.628	0.1997	False	ns
88	Tyr	H2O2_AT_2	AT_H2O2_2	AT_Untreated_2	-0.2350	-0.1096	-1.272	0.2250	0.6409	-4.654	0.1932	False	ns
43	Glucose	H2O2_AT_2	AT_H2O2_2	AT_Untreated_2	0.3431	0.4648	1.264	0.2276	0.6409	-4.661	0.1932	False	ns
73	Asn	H2O2_AT_2	AT_H2O2_2	AT_Untreated_2	-0.3877	-1.473	-1.254	0.2311	0.6409	-4.669	0.1932	False	ns
78	Ser	H2O2_AT_2	AT_H2O2_2	AT_Untreated_2	-0.3218	-1.076	-1.230	0.2397	0.6411	-4.689	0.1931	False	ns
251	Gly-Leu	H2O2_AT_2	AT_H2O2_2	AT_Untreated_2	-0.5338	-0.4661	-1.221	0.2431	0.6411	-4.697	0.1931	False	ns
246	Asp-Leu	H2O2_AT_2	AT_H2O2_2	AT_Untreated_2	-0.4333	-0.3632	-1.215	0.2452	0.6411	-4.702	0.1931	False	ns
399	Pseudouridine	H2O2_AT_2	AT_H2O2_2	AT_Untreated_2	0.5064	2.049	1.200	0.2508	0.6411	-4.714	0.1931	False	ns
253	Gly-Pro	H2O2_AT_2	AT_H2O2_2	AT_Untreated_2	-0.4653	0.0440	-1.195	0.2529	0.6411	-4.719	0.1931	False	ns
224	N-Ac-Ser	H2O2_AT_2	AT_H2O2_2	AT_Untreated_2	-0.4422	-1.654	-1.165	0.2643	0.6585	-4.743	0.1814	False	ns
74	Asp	H2O2_AT_2	AT_H2O2_2	AT_Untreated_2	-0.3182	-1.699	-1.138	0.2750	0.6635	-4.764	0.1782	False	ns
70	Creatine	H2O2_AT_2	AT_H2O2_2	AT_Untreated_2	-0.1782	-0.1808	-1.131	0.2778	0.6635	-4.770	0.1782	False	ns
286	gamma-Glu-Glu	H2O2_AT_2	AT_H2O2_2	AT_Untreated_2	-0.4717	-0.9090	-1.114	0.2847	0.6635	-4.783	0.1782	False	ns
377	Uridine	H2O2_AT_2	AT_H2O2_2	AT_Untreated_2	-0.2504	-0.4398	-1.113	0.2854	0.6635	-4.784	0.1782	False	ns
86	Met	H2O2_AT_2	AT_H2O2_2	AT_Untreated_2	-0.1835	-0.4361	-1.091	0.2943	0.6635	-4.800	0.1782	False	ns
30	Sucrose	H2O2_AT_2	AT_H2O2_2	AT_Untreated_2	-0.4532	-0.4136	-1.087	0.2960	0.6635	-4.803	0.1782	False	ns
34	Ribitol	H2O2_AT_2	AT_H2O2_2	AT_Untreated_2	-0.3622	0.5621	-1.083	0.2979	0.6635	-4.807	0.1782	False	ns
82	Leu	H2O2_AT_2	AT_H2O2_2	AT_Untreated_2	-0.1825	-0.1649	-1.064	0.3060	0.6636	-4.821	0.1781	False	ns
129	5-Aminovalerate	H2O2_AT_2	AT_H2O2_2	AT_Untreated_2	-0.2802	-0.2184	-1.060	0.3078	0.6636	-4.824	0.1781	False	ns
87	Phe	H2O2_AT_2	AT_H2O2_2	AT_Untreated_2	-0.2047	-0.1100	-1.048	0.3129	0.6636	-4.833	0.1781	False	ns
151	Phenylacetylglycine	H2O2_AT_2	AT_H2O2_2	AT_Untreated_2	0.9855	4.179	1.042	0.3160	0.6636	-4.838	0.1781	False	ns
145	Pyro-Gln	H2O2_AT_2	AT_H2O2_2	AT_Untreated_2	0.2525	0.0397	0.9954	0.3371	0.6855	-4.871	0.1640	False	ns
742	Folate	H2O2_AT_2	AT_H2O2_2	AT_Untreated_2	0.2156	0.3706	0.9892	0.3401	0.6855	-4.875	0.1640	False	ns
206	Trans-4-OH-Pro	H2O2_AT_2	AT_H2O2_2	AT_Untreated_2	0.2083	1.049	0.9884	0.3404	0.6855	-4.876	0.1640	False	ns
261	Leu-Glu	H2O2_AT_2	AT_H2O2_2	AT_Untreated_2	-0.4761	-1.566	-0.9617	0.3532	0.6931	-4.894	0.1592	False	ns
273	Ser-Phe	H2O2_AT_2	AT_H2O2_2	AT_Untreated_2	-0.2100	-0.8259	-0.9609	0.3536	0.6931	-4.895	0.1592	False	ns
707	FMN	H2O2_AT_2	AT_H2O2_2	AT_Untreated_2	-0.3723	-0.4714	-0.9402	0.3637	0.7035	-4.909	0.1527	False	ns
245	Ala-Phe	H2O2_AT_2	AT_H2O2_2	AT_Untreated_2	0.3198	-0.8371	0.9156	0.3760	0.7178	-4.925	0.1440	False	ns
173	Met Sulfoxide	H2O2_AT_2	AT_H2O2_2	AT_Untreated_2	0.3142	0.7668	0.8855	0.3914	0.7331	-4.945	0.1348	False	ns
59	Succinate	H2O2_AT_2	AT_H2O2_2	AT_Untreated_2	0.3114	-0.0796	0.8806	0.3940	0.7331	-4.948	0.1348	False	ns

Metabolite ID	Name	Comparison	Group Numerator	Group Denominator	Log2 FC	Avg Expression	t	p-value	BH adj p-value	B	-Log10 BH p	Signif.	Direction
277	Tyr-Ala	H2O2_AT_2	AT_H2O2_2	AT_Untreated_2	-0.4624	-1.159	-0.8518	0.4093	0.7520	-4.966	0.1238	False	ns
236	Spermidine	H2O2_AT_2	AT_H2O2_2	AT_Untreated_2	-0.4773	-1.441	-0.8362	0.4177	0.7547	-4.976	0.1222	False	ns
237	Spermine	H2O2_AT_2	AT_H2O2_2	AT_Untreated_2	-0.3576	-1.731	-0.8301	0.4210	0.7547	-4.979	0.1222	False	ns
241	4-Acetamidobutanoate	H2O2_AT_2	AT_H2O2_2	AT_Untreated_2	0.1698	1.436	0.8186	0.4273	0.7554	-4.986	0.1218	False	ns
89	Trp	H2O2_AT_2	AT_H2O2_2	AT_Untreated_2	-0.1665	-0.2422	-0.8107	0.4317	0.7554	-4.991	0.1218	False	ns
81	Ile	H2O2_AT_2	AT_H2O2_2	AT_Untreated_2	-0.1432	0.0305	-0.7795	0.4492	0.7613	-5.009	0.1184	False	ns
729	Riboflavin (Vitamin B2)	H2O2_AT_2	AT_H2O2_2	AT_Untreated_2	-0.2429	0.2168	-0.7786	0.4497	0.7613	-5.010	0.1184	False	ns
345	Guanine	H2O2_AT_2	AT_H2O2_2	AT_Untreated_2	-0.2571	0.5729	-0.7668	0.4564	0.7613	-5.016	0.1184	False	ns
80	His	H2O2_AT_2	AT_H2O2_2	AT_Untreated_2	-0.1351	-0.3024	-0.7615	0.4595	0.7613	-5.019	0.1184	False	ns
17	Maltose	H2O2_AT_2	AT_H2O2_2	AT_Untreated_2	0.2008	1.118	0.7591	0.4609	0.7613	-5.021	0.1184	False	ns
724	Pantothenate	H2O2_AT_2	AT_H2O2_2	AT_Untreated_2	-0.1648	0.4397	-0.7433	0.4701	0.7679	-5.030	0.1147	False	ns
264	Leu-Leu	H2O2_AT_2	AT_H2O2_2	AT_Untreated_2	-0.2039	-0.6164	-0.7240	0.4815	0.7720	-5.040	0.1124	False	ns
309	Glutathione, Oxidized	H2O2_AT_2	AT_H2O2_2	AT_Untreated_2	-0.1414	-0.0930	-0.7212	0.4831	0.7720	-5.042	0.1124	False	ns
208	Pro-OH-Pro	H2O2_AT_2	AT_H2O2_2	AT_Untreated_2	-0.1387	0.2032	-0.7056	0.4925	0.7785	-5.050	0.1088	False	ns
83	Val	H2O2_AT_2	AT_H2O2_2	AT_Untreated_2	-0.1188	0.0613	-0.6873	0.5036	0.7843	-5.059	0.1055	False	ns
299	gamma-Glu-Tyr	H2O2_AT_2	AT_H2O2_2	AT_Untreated_2	-0.2196	-1.225	-0.6651	0.5172	0.7843	-5.070	0.1055	False	ns
275	Thr-Phe	H2O2_AT_2	AT_H2O2_2	AT_Untreated_2	0.1655	-0.5602	0.6532	0.5247	0.7843	-5.076	0.1055	False	ns
183	Phenol Sulfate	H2O2_AT_2	AT_H2O2_2	AT_Untreated_2	0.6324	2.244	0.6343	0.5365	0.7843	-5.085	0.1055	False	ns
52	Pyruvate	H2O2_AT_2	AT_H2O2_2	AT_Untreated_2	-0.2403	-0.0873	-0.6336	0.5370	0.7843	-5.086	0.1055	False	ns
189	Kynurenate	H2O2_AT_2	AT_H2O2_2	AT_Untreated_2	0.3965	1.541	0.6301	0.5392	0.7843	-5.087	0.1055	False	ns
717	Nicotinamide	H2O2_AT_2	AT_H2O2_2	AT_Untreated_2	-0.1498	0.2407	-0.6243	0.5429	0.7843	-5.090	0.1055	False	ns
313	5-Oxoproline	H2O2_AT_2	AT_H2O2_2	AT_Untreated_2	0.1332	1.542	0.6189	0.5464	0.7843	-5.093	0.1055	False	ns
60	Fumarate	H2O2_AT_2	AT_H2O2_2	AT_Untreated_2	-0.2645	-0.0728	-0.6168	0.5477	0.7843	-5.094	0.1055	False	ns
385	Uracil	H2O2_AT_2	AT_H2O2_2	AT_Untreated_2	0.3486	1.483	0.6139	0.5496	0.7843	-5.095	0.1055	False	ns
250	Gly-Ile	H2O2_AT_2	AT_H2O2_2	AT_Untreated_2	-0.3685	0.1066	-0.5948	0.5618	0.7941	-5.104	0.1001	False	ns
314	Cys-Glutathione Disulfide	H2O2_AT_2	AT_H2O2_2	AT_Untreated_2	0.1451	1.236	0.5716	0.5771	0.8079	-5.114	0.0927	False	ns
20	Erythronate	H2O2_AT_2	AT_H2O2_2	AT_Untreated_2	-0.1585	0.1544	-0.5497	0.5916	0.8204	-5.123	0.0860	False	ns
169	2-OH-Butyrate	H2O2_AT_2	AT_H2O2_2	AT_Untreated_2	-0.1650	0.5910	-0.5228	0.6097	0.8376	-5.134	0.0770	False	ns
390	2',3'-cUMP	H2O2_AT_2	AT_H2O2_2	AT_Untreated_2	0.1406	-0.7079	0.4983	0.6264	0.8388	-5.144	0.0763	False	ns
85	Cys	H2O2_AT_2	AT_H2O2_2	AT_Untreated_2	-0.2026	0.8206	-0.4794	0.6393	0.8388	-5.151	0.0763	False	ns
55	Citrate	H2O2_AT_2	AT_H2O2_2	AT_Untreated_2	0.1809	-0.1140	0.4584	0.6540	0.8388	-5.158	0.0763	False	ns

Metabolite ID	Name	Comparison	Group Numerator	Group Denominator	Log2 FC	Avg Expression	t	p-value	BH adj p-value	B	-Log10 BH p	Signif.	Direction
24	Fructose	H2O2_AT_2	AT_H2O2_2	AT_Untreated_2	-0.1428	1.371	-0.4515	0.6588	0.8388	-5.160	0.0763	False	ns
110	N-alpha-Ac-Ornithine	H2O2_AT_2	AT_H2O2_2	AT_Untreated_2	0.1152	-0.4068	0.4475	0.6616	0.8388	-5.162	0.0763	False	ns
112	trans-Urocanate	H2O2_AT_2	AT_H2O2_2	AT_Untreated_2	-0.1050	3.241	-0.4447	0.6636	0.8388	-5.163	0.0763	False	ns
731	Thiamin (Vitamin B1)	H2O2_AT_2	AT_H2O2_2	AT_Untreated_2	-0.0987	-0.0295	-0.4360	0.6697	0.8388	-5.166	0.0763	False	ns
4	GlcNAc 6-P	H2O2_AT_2	AT_H2O2_2	AT_Untreated_2	-0.1882	-0.7001	-0.4342	0.6710	0.8388	-5.166	0.0763	False	ns
90	Arg	H2O2_AT_2	AT_H2O2_2	AT_Untreated_2	-0.0987	-0.1799	-0.4270	0.6762	0.8388	-5.169	0.0763	False	ns
705	Coenzyme A	H2O2_AT_2	AT_H2O2_2	AT_Untreated_2	-0.2104	-1.514	-0.4203	0.6809	0.8388	-5.171	0.0763	False	ns
725	P-Pantetheine	H2O2_AT_2	AT_H2O2_2	AT_Untreated_2	0.1274	0.6075	0.4130	0.6861	0.8388	-5.173	0.0763	False	ns
738	Pyridoxate	H2O2_AT_2	AT_H2O2_2	AT_Untreated_2	0.0987	1.705	0.4117	0.6871	0.8388	-5.174	0.0763	False	ns
19	Maltotetraose	H2O2_AT_2	AT_H2O2_2	AT_Untreated_2	-0.0952	1.171	-0.4087	0.6892	0.8388	-5.175	0.0763	False	ns
18	Maltotriose	H2O2_AT_2	AT_H2O2_2	AT_Untreated_2	-0.1374	2.137	-0.4069	0.6905	0.8388	-5.175	0.0763	False	ns
203	DiMe-Arg	H2O2_AT_2	AT_H2O2_2	AT_Untreated_2	0.1202	0.5233	0.3885	0.7038	0.8480	-5.181	0.0716	False	ns
376	Cytidine	H2O2_AT_2	AT_H2O2_2	AT_Untreated_2	-0.1429	2.374	-0.3592	0.7250	0.8665	-5.189	0.0623	False	ns
737	Pyridoxine (Vitamin B6)	H2O2_AT_2	AT_H2O2_2	AT_Untreated_2	0.0683	0.5411	0.3179	0.7555	0.8850	-5.200	0.0531	False	ns
302	Cyclo(Glu-Glu)	H2O2_AT_2	AT_H2O2_2	AT_Untreated_2	-0.1363	-0.5177	-0.3166	0.7564	0.8850	-5.200	0.0531	False	ns
234	5-Me-Thioadenosine	H2O2_AT_2	AT_H2O2_2	AT_Untreated_2	-0.0956	-1.202	-0.2978	0.7704	0.8850	-5.205	0.0531	False	ns
122	3-OH-Isobutyrate	H2O2_AT_2	AT_H2O2_2	AT_Untreated_2	-0.1844	1.260	-0.2872	0.7784	0.8850	-5.207	0.0531	False	ns
100	4-Me-2-Oxo-Pentanoate	H2O2_AT_2	AT_H2O2_2	AT_Untreated_2	-0.1087	1.902	-0.2849	0.7801	0.8850	-5.208	0.0531	False	ns
116	3-Me-2-Oxo-Valerate	H2O2_AT_2	AT_H2O2_2	AT_Untreated_2	-0.1119	2.158	-0.2726	0.7893	0.8850	-5.210	0.0531	False	ns
39	Threitol	H2O2_AT_2	AT_H2O2_2	AT_Untreated_2	0.0937	0.1520	0.2715	0.7901	0.8850	-5.210	0.0531	False	ns
344	Xanthine	H2O2_AT_2	AT_H2O2_2	AT_Untreated_2	-0.2106	0.9319	-0.2658	0.7944	0.8850	-5.212	0.0531	False	ns
243	Creatinine	H2O2_AT_2	AT_H2O2_2	AT_Untreated_2	-0.0665	0.9514	-0.2654	0.7947	0.8850	-5.212	0.0531	False	ns
84	Lys	H2O2_AT_2	AT_H2O2_2	AT_Untreated_2	-0.0496	0.0688	-0.2490	0.8071	0.8898	-5.215	0.0507	False	ns
76	Gln	H2O2_AT_2	AT_H2O2_2	AT_Untreated_2	-0.0485	-0.7062	-0.2432	0.8115	0.8898	-5.216	0.0507	False	ns
388	2',3'-cCMP	H2O2_AT_2	AT_H2O2_2	AT_Untreated_2	0.1511	-1.632	0.2358	0.8172	0.8898	-5.217	0.0507	False	ns
136	Urea	H2O2_AT_2	AT_H2O2_2	AT_Untreated_2	0.0472	0.9529	0.2128	0.8346	0.9014	-5.221	0.0451	False	ns
98	3-Methyl-2-Oxobutyrate	H2O2_AT_2	AT_H2O2_2	AT_Untreated_2	-0.0911	0.9853	-0.2057	0.8401	0.9014	-5.223	0.0451	False	ns
252	Gly-Phe	H2O2_AT_2	AT_H2O2_2	AT_Untreated_2	-0.0771	0.1015	-0.1920	0.8506	0.9061	-5.225	0.0428	False	ns
170	2-Amino-Butyrate	H2O2_AT_2	AT_H2O2_2	AT_Untreated_2	-0.0565	-0.0911	-0.1735	0.8649	0.9147	-5.227	0.0387	False	ns
274	Thr-Leu	H2O2_AT_2	AT_H2O2_2	AT_Untreated_2	0.0544	0.0072	0.1602	0.8751	0.9188	-5.229	0.0368	False	ns

Metabolite ID	Name	Comparison	Group Numerator	Group Denominator	Log2 FC	Avg Expression	t	p-value	BH adj p-value	B	-Log10 BH p	Signif.	Direction
271	pyroGlu-Val	H2O2_AT_2	AT_H2O2_2	AT_Untreated_2	-0.0567	-0.5480	-0.1455	0.8865	0.9242	-5.231	0.0342	False	ns
290	gamma-Glu-Ile	H2O2_AT_2	AT_H2O2_2	AT_Untreated_2	-0.0491	-0.5806	-0.1155	0.9097	0.9418	-5.234	0.0261	False	ns
282	Val-Leu	H2O2_AT_2	AT_H2O2_2	AT_Untreated_2	-0.0330	-0.5335	-0.1035	0.9191	0.9448	-5.235	0.0247	False	ns
232	SAH	H2O2_AT_2	AT_H2O2_2	AT_Untreated_2	-0.0193	0.0215	-0.0937	0.9267	0.9461	-5.236	0.0241	False	ns
336	Adenosine	H2O2_AT_2	AT_H2O2_2	AT_Untreated_2	0.0164	-1.890	0.0405	0.9683	0.9713	-5.238	0.0126	False	ns
316	Ophthalmate	H2O2_AT_2	AT_H2O2_2	AT_Untreated_2	-0.0118	-1.341	-0.0379	0.9703	0.9713	-5.238	0.0126	False	ns
706	FAD	H2O2_AT_2	AT_H2O2_2	AT_Untreated_2	-0.0159	0.7548	-0.0366	0.9713	0.9713	-5.238	0.0126	False	ns
346	Urate	AT_combined			1.534	0.7377	7.346	1.58e-07	8.30e-06	7.479	5.081	True	up
399	Pseudouridine	AT_combined			1.563	0.6723	7.284	1.81e-07	8.30e-06	7.340	5.081	True	up
74	Asp	AT_combined			-1.329	-0.5644	-7.224	2.08e-07	8.30e-06	7.208	5.081	True	down
18	Maltotriose	AT_combined			2.302	1.261	6.628	8.27e-07	2.48e-05	5.845	4.605	True	up
224	N-Ac-Ser	AT_combined			-1.349	-0.6520	-6.262	1.97e-06	4.73e-05	4.988	4.325	True	down
738	Pyridoxate	AT_combined			1.392	0.5639	6.187	2.37e-06	4.73e-05	4.810	4.325	True	up
348	Allantoin	AT_combined			1.254	0.5542	5.848	5.38e-06	9.23e-05	4.000	4.035	True	up
151	Phenylacetylglycine	AT_combined			2.897	1.026	5.736	7.09e-06	0.0001	3.728	3.995	True	up
241	4-Acetamidobutanoate	AT_combined			1.265	0.6070	5.708	7.59e-06	0.0001	3.661	3.995	True	up
24	Fructose	AT_combined			1.402	0.7064	5.495	1.28e-05	0.0002	3.144	3.815	True	up
313	5-Oxoproline	AT_combined			1.166	0.4183	5.459	1.40e-05	0.0002	3.056	3.815	True	up
189	Kynurenate	AT_combined			1.340	0.6968	5.382	1.70e-05	0.0002	2.869	3.770	True	up
234	5-Me-Thioadenosine	AT_combined			-1.211	-0.6665	-5.126	3.21e-05	0.0003	2.243	3.528	True	down
177	3-(4-OH-Phenyl)Lactate	AT_combined			1.231	0.6358	4.994	4.47e-05	0.0004	1.917	3.417	True	up
73	Asn	AT_combined			-1.014	-0.3665	-4.894	5.74e-05	0.0005	1.672	3.338	True	down
209	N-Ac-Ala	AT_combined			-0.8823	-0.4061	-4.785	7.56e-05	0.0006	1.402	3.246	True	down

Metabolite ID	Name	Comparison	Group Numerator	Group Denominator	Log2 FC	Avg Expression	t	p-value	BH adj p-value	B	-Log10 BH p	Signif.	Direction
703	NAD+	AT_combined			-1.050	-0.6174	-4.715	9.01e-05	0.0006	1.230	3.197	True	down
243	Creatinine	AT_combined			0.8818	0.3968	4.681	9.81e-05	0.0007	1.147	3.184	True	up
336	Adenosine	AT_combined			-1.526	-0.5674	-4.219	0.0003	0.0020	0.0110	2.703	True	down
258	Ile-Gly	AT_combined			-1.303	-0.5658	-4.198	0.0003	0.0020	-0.0397	2.702	True	down
316	Ophthalmate	AT_combined			-1.400	-0.7702	-4.107	0.0004	0.0024	-0.2625	2.624	True	down
122	3-OH-Isobutyrate	AT_combined			1.226	0.5618	4.083	0.0004	0.0024	-0.3221	2.617	True	up
19	Maltotetraose	AT_combined			1.621	1.093	4.052	0.0005	0.0025	-0.3966	2.603	True	up
136	Urea	AT_combined			0.9126	0.4659	4.021	0.0005	0.0026	-0.4718	2.588	True	up
102	2-Aminoadipate	AT_combined			-0.7320	-0.3789	-3.934	0.0006	0.0031	-0.6830	2.511	True	down
343	Adenine	AT_combined			-0.7031	-0.4342	-3.915	0.0007	0.0031	-0.7298	2.507	True	down
17	Maltose	AT_combined			1.114	0.6405	3.854	0.0008	0.0035	-0.8758	2.458	True	up
710	Carnitine	AT_combined			-0.7138	-0.3192	-3.787	0.0009	0.0040	-1.038	2.401	True	down
183	Phenol Sulfate	AT_combined			1.697	0.7434	3.733	0.0011	0.0044	-1.168	2.358	True	up
221	N-Ac-Met	AT_combined			-0.7814	-0.4214	-3.443	0.0022	0.0087	-1.854	2.062	True	down
734	Pyridoxal	AT_combined			0.8269	0.4112	3.419	0.0023	0.0089	-1.912	2.050	True	up
704	NADH	AT_combined			-1.410	-0.6617	-3.286	0.0032	0.0118	-2.218	1.927	True	down
76	Gln	AT_combined			-0.6149	-0.2796	-3.276	0.0033	0.0118	-2.241	1.927	True	down
34	Ribitol	AT_combined			0.6790	0.3147	3.189	0.0040	0.0142	-2.441	1.849	True	up
308	Glutathione, Reduced	AT_combined			-0.9111	-0.5129	-3.175	0.0041	0.0142	-2.473	1.847	True	down
236	Spermidine	AT_combined			-1.121	-0.5348	-3.124	0.0047	0.0156	-2.588	1.806	True	down
41	Lactate	AT_combined			0.6039	0.1444	3.072	0.0053	0.0172	-2.706	1.764	True	up

Metabolite ID	Name	Comparison	Group Numerator	Group Denominator	Log2 FC	Avg Expression	t	p-value	BH adj p-value	B	-Log10 BH p	Signif.	Direction
72	Ala	AT_combined			-0.5287	-0.2382	-3.001	0.0063	0.0198	-2.864	1.703	True	down
79	Thr	AT_combined			-0.6037	-0.2501	-2.983	0.0066	0.0200	-2.906	1.700	True	down
706	FAD	AT_combined			0.6167	0.2319	2.977	0.0067	0.0200	-2.919	1.700	True	up
705	Coenzyme A	AT_combined			-1.116	-0.4015	-2.966	0.0068	0.0200	-2.943	1.699	True	down
206	Trans-4-OH-Pro	AT_combined			0.5931	0.0932	2.865	0.0086	0.0247	-3.163	1.608	True	up
376	Cytidine	AT_combined			1.998	0.7669	2.830	0.0094	0.0262	-3.240	1.582	True	up
173	Met Sulfoxide	AT_combined			0.5608	0.2625	2.775	0.0107	0.0291	-3.359	1.537	True	up
48	DHAP	AT_combined			-0.6626	-0.2585	-2.704	0.0125	0.0331	-3.510	1.481	True	down
197	C-Glycosyl-Trp	AT_combined			0.7763	0.2884	2.696	0.0128	0.0331	-3.527	1.481	True	up
77	Gly	AT_combined			-0.5529	-0.2603	-2.689	0.0129	0.0331	-3.540	1.481	True	down
182	P-Cresol Sulfate	AT_combined			0.6789	0.4417	2.669	0.0136	0.0339	-3.583	1.470	True	up
344	Xanthine	AT_combined			1.046	0.5511	2.637	0.0146	0.0351	-3.651	1.454	True	up
725	P-Pantetheine	AT_combined			0.7053	0.4672	2.635	0.0146	0.0351	-3.653	1.454	True	up
335	Inosine	AT_combined			-0.5627	-0.2482	-2.599	0.0159	0.0374	-3.729	1.427	True	down
385	Uracil	AT_combined			1.508	0.9098	2.557	0.0174	0.0403	-3.815	1.395	True	up
85	Cys	AT_combined			0.6281	0.1470	2.532	0.0185	0.0418	-3.867	1.378	True	up
91	Pro	AT_combined			-0.4017	-0.1931	-2.520	0.0189	0.0421	-3.890	1.376	True	down
78	Ser	AT_combined			-0.7079	-0.2417	-2.469	0.0212	0.0463	-3.994	1.334	True	down
314	Cys-Glutathione Disulfide	AT_combined			0.9717	0.3890	2.448	0.0222	0.0472	-4.037	1.326	True	up
188	Kynurenine	AT_combined			1.145	0.8028	2.444	0.0224	0.0472	-4.045	1.326	True	up
44	Glucose 6-P	AT_combined			-0.7809	-0.4276	-2.381	0.0257	0.0532	-4.169	1.274	False	ns

Metabolite ID	Name	Comparison	Group Numerator	Group Denominator	Log2 FC	Avg Expression	t	p-value	BH adj p-value	B	-Log10 BH p	Signif.	Direction
286	gamma-Glu-Glu	AT_combined			-0.6257	-0.2973	-2.367	0.0265	0.0540	-4.197	1.268	False	ns
45	Fructose-6-P	AT_combined			-0.7334	-0.4882	-2.329	0.0288	0.0574	-4.271	1.241	False	ns
290	gamma-Glu-Ile	AT_combined			-0.6996	-0.4533	-2.323	0.0292	0.0574	-4.283	1.241	False	ns
43	Glucose	AT_combined			0.4920	0.3789	2.236	0.0351	0.0679	-4.449	1.168	False	ns
51	PEP	AT_combined			0.7444	0.3030	2.117	0.0450	0.0848	-4.670	1.072	False	ns
237	Spermine	AT_combined			-1.050	-0.2394	-2.115	0.0452	0.0848	-4.674	1.072	False	ns
235	Putrescine	AT_combined			-1.142	-0.4508	-2.101	0.0466	0.0860	-4.700	1.066	False	ns
345	Guanine	AT_combined			0.6674	0.3272	2.079	0.0487	0.0885	-4.739	1.053	False	ns
133	Ornithine	AT_combined			0.4572	0.1030	1.868	0.0743	0.1331	-5.107	0.8758	False	ns
717	Nicotinamide	AT_combined			0.4462	0.3131	1.775	0.0889	0.1569	-5.259	0.8045	False	ns
49	3-P-Glycerate	AT_combined			0.4493	0.1737	1.725	0.0977	0.1700	-5.339	0.7696	False	ns
86	Met	AT_combined			-0.2854	-0.1127	-1.629	0.1167	0.1979	-5.487	0.7036	False	ns
70	Creatine	AT_combined			-0.1846	-0.1535	-1.625	0.1175	0.1979	-5.492	0.7036	False	ns
724	Pantothenate	AT_combined			0.3152	0.0372	1.619	0.1187	0.1979	-5.501	0.7036	False	ns
329	AMP	AT_combined			-1.080	-0.7147	-1.562	0.1316	0.2163	-5.585	0.6649	False	ns
244	Ala-Leu	AT_combined			-0.7444	-0.2996	-1.480	0.1522	0.2469	-5.702	0.6076	False	ns
275	Thr-Phe	AT_combined			-0.5063	-0.1801	-1.228	0.2315	0.3704	-6.027	0.4313	False	ns
20	Erythronate	AT_combined			0.2544	0.1455	1.207	0.2395	0.3764	-6.052	0.4243	False	ns
80	His	AT_combined			-0.1904	-0.0720	-1.201	0.2415	0.3764	-6.059	0.4243	False	ns
63	6-P-Gluconate	AT_combined			-0.4815	-0.4085	-1.126	0.2717	0.4180	-6.144	0.3788	False	ns
89	Trp	AT_combined			-0.2518	-0.1884	-1.093	0.2853	0.4334	-6.179	0.3631	False	ns

Metabolite ID	Name	Comparison	Group Numerator	Group Denominator	Log2 FC	Avg Expression	t	p-value	BH adj p-value	B	-Log10 BH p	Signif.	Direction
375	UTP	AT_combined			-0.6795	-0.5566	-1.061	0.2993	0.4490	-6.213	0.3478	False	ns
251	Gly-Leu	AT_combined			-0.2796	-0.1953	-1.032	0.3126	0.4589	-6.244	0.3382	False	ns
36	Ribose	AT_combined			-0.3024	-0.1123	-1.030	0.3136	0.4589	-6.246	0.3382	False	ns
4	GlcNAc 6-P	AT_combined			-0.4456	-0.1338	-1.007	0.3240	0.4684	-6.268	0.3294	False	ns
282	Val-Leu	AT_combined			-0.4479	-0.1913	-0.9837	0.3353	0.4790	-6.291	0.3197	False	ns
81	Ile	AT_combined			0.1302	0.0852	0.9619	0.3459	0.4883	-6.312	0.3113	False	ns
373	UMP	AT_combined			-0.6875	-0.3274	-0.9533	0.3502	0.4886	-6.321	0.3110	False	ns
291	gamma-Glu-Leu	AT_combined			0.2524	-0.0718	0.9256	0.3641	0.5022	-6.346	0.2992	False	ns
208	Pro-OH-Pro	AT_combined			0.2037	0.0640	0.9063	0.3740	0.5100	-6.364	0.2924	False	ns
61	Malate	AT_combined			0.1706	-0.0366	0.8126	0.4246	0.5701	-6.444	0.2441	False	ns
129	5-Aminovalerate	AT_combined			-0.1787	-0.1549	-0.8074	0.4276	0.5701	-6.449	0.2441	False	ns
309	Glutathione, Oxidized	AT_combined			0.1553	0.1902	0.7977	0.4330	0.5710	-6.456	0.2434	False	ns
723	beta-Ala	AT_combined			-0.1560	-0.1871	-0.7719	0.4479	0.5842	-6.477	0.2335	False	ns
263	Leu-Gly	AT_combined			-0.4450	-0.0716	-0.7359	0.4691	0.6016	-6.504	0.2207	False	ns
377	Uridine	AT_combined			-0.1665	0.0043	-0.7322	0.4713	0.6016	-6.507	0.2207	False	ns
75	Glu	AT_combined			-0.1018	-0.1402	-0.7115	0.4838	0.6111	-6.522	0.2139	False	ns
83	Val	AT_combined			0.1288	0.0721	0.7028	0.4891	0.6114	-6.528	0.2137	False	ns
338	Guanosine	AT_combined			0.1476	0.1478	0.6524	0.5204	0.6439	-6.562	0.1912	False	ns
720	1-Me-Nicotinamide	AT_combined			-0.0909	-0.0631	-0.6101	0.5476	0.6706	-6.589	0.1736	False	ns
203	DiMe-Arg	AT_combined			0.2136	-0.0421	0.5980	0.5556	0.6734	-6.597	0.1717	False	ns
342	Hypoxanthine	AT_combined			0.1138	0.0457	0.5729	0.5721	0.6866	-6.612	0.1633	False	ns

Metabolite ID	Name	Comparison	Group Numerator	Group Denominator	Log2 FC	Avg Expression	t	p-value	BH adj p-value	B	-Log10 BH p	Signif.	Direction
250	Gly-Ile	AT_combined			0.1561	-0.0014	0.4941	0.6258	0.7435	-6.654	0.1287	False	ns
212	N-Ac-Asp	AT_combined			0.1447	-0.0617	0.4751	0.6391	0.7519	-6.664	0.1238	False	ns
232	SAH	AT_combined			-0.0676	-0.0960	-0.4374	0.6658	0.7757	-6.681	0.1103	False	ns
145	Pyro-Gln	AT_combined			-0.0631	-0.0185	-0.3241	0.7487	0.8639	-6.725	0.0635	False	ns
84	Lys	AT_combined			0.0427	-0.0090	0.2901	0.7743	0.8849	-6.736	0.0531	False	ns
155	4-Guanidinobutanoate	AT_combined			-0.0831	-0.0307	-0.2466	0.8073	0.9070	-6.748	0.0424	False	ns
310	S-Lactoyl-Glutathione	AT_combined			-0.1187	-0.0351	-0.2407	0.8119	0.9070	-6.750	0.0424	False	ns
352	3'-AMP	AT_combined			0.0477	-0.0158	0.2350	0.8163	0.9070	-6.751	0.0424	False	ns
55	Citrate	AT_combined			0.0457	0.1777	0.2156	0.8311	0.9150	-6.755	0.0386	False	ns
88	Tyr	AT_combined			0.0323	0.0319	0.1813	0.8577	0.9329	-6.762	0.0302	False	ns
90	Arg	AT_combined			-0.0298	0.0463	-0.1745	0.8629	0.9329	-6.764	0.0302	False	ns
82	Leu	AT_combined			-0.0235	0.0186	-0.1432	0.8874	0.9473	-6.769	0.0235	False	ns
718	Nicotinamide Riboside	AT_combined			0.1377	0.1083	0.1372	0.8920	0.9473	-6.770	0.0235	False	ns
254	Gly-Val	AT_combined			-0.0118	-0.0472	-0.0546	0.9569	0.9898	-6.778	0.0045	False	ns
59	Succinate	AT_combined			-0.0148	0.1279	-0.0494	0.9610	0.9898	-6.778	0.0045	False	ns
60	Fumarate	AT_combined			0.0077	-0.0278	0.0437	0.9655	0.9898	-6.778	0.0045	False	ns
87	Phe	AT_combined			-0.0055	-0.0015	-0.0375	0.9704	0.9898	-6.779	0.0045	False	ns
22	N-Ac-Neuraminate	AT_combined			0.0053	0.0016	0.0339	0.9733	0.9898	-6.779	0.0045	False	ns
33	Arabitol/Xylitol	AT_combined			0.0051	-0.0654	0.0186	0.9853	0.9927	-6.779	0.0032	False	ns
731	Thiamin (Vitamin B1)	AT_combined			0.0015	-0.0105	0.0093	0.9927	0.9927	-6.779	0.0032	False	ns
