# Supplementary material for: Targeting the FNIP2-SERCA2b axis improves metabolic and mitochondrial defects in Ataxia Telangiectasia
Source: Cell Death Dis. 2026 Mar 2;17(1):290. doi: 10.1038/s41419-026-08507-5 (PMC13031930; doi:10.1038/s41419-026-08507-5)

Uncropped Western blots for Fig. 4B

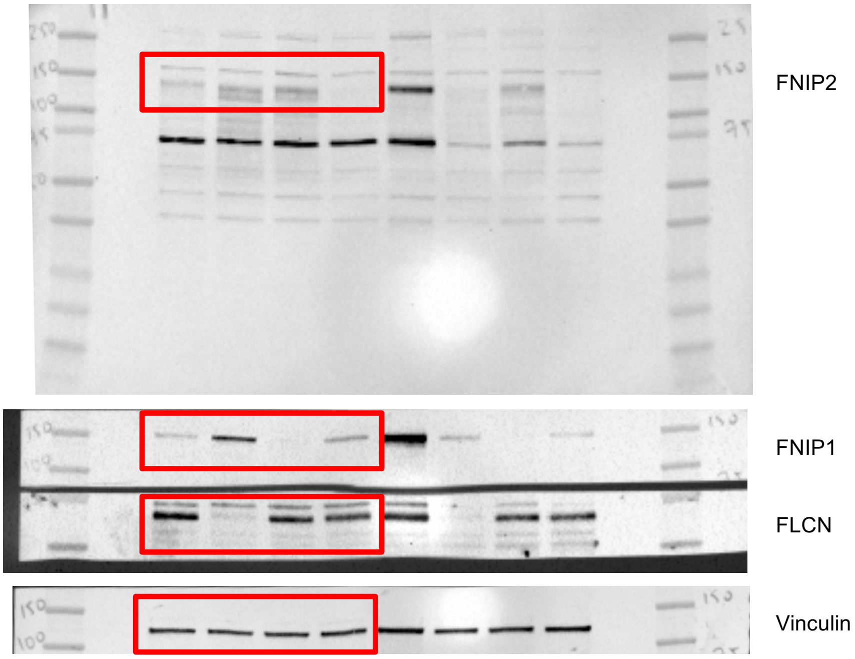

Uncropped Western blots for Fig. 4D

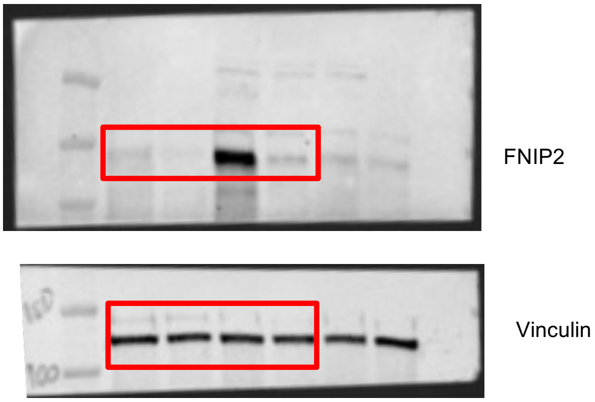

Uncropped Western blots for Fig. S7A

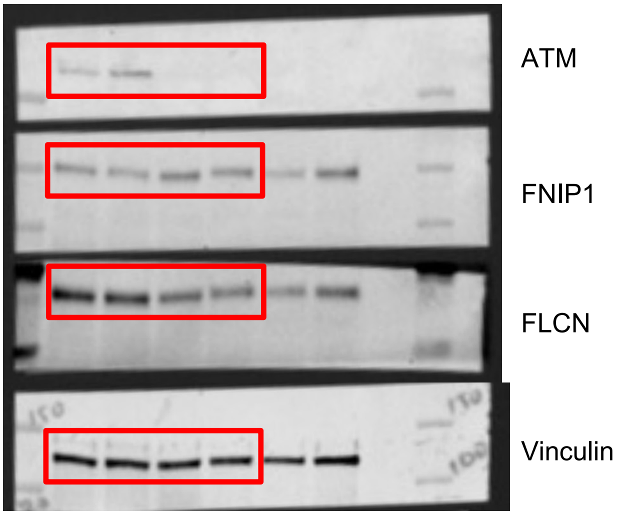

Uncropped Western blots for Fig. 6A-B

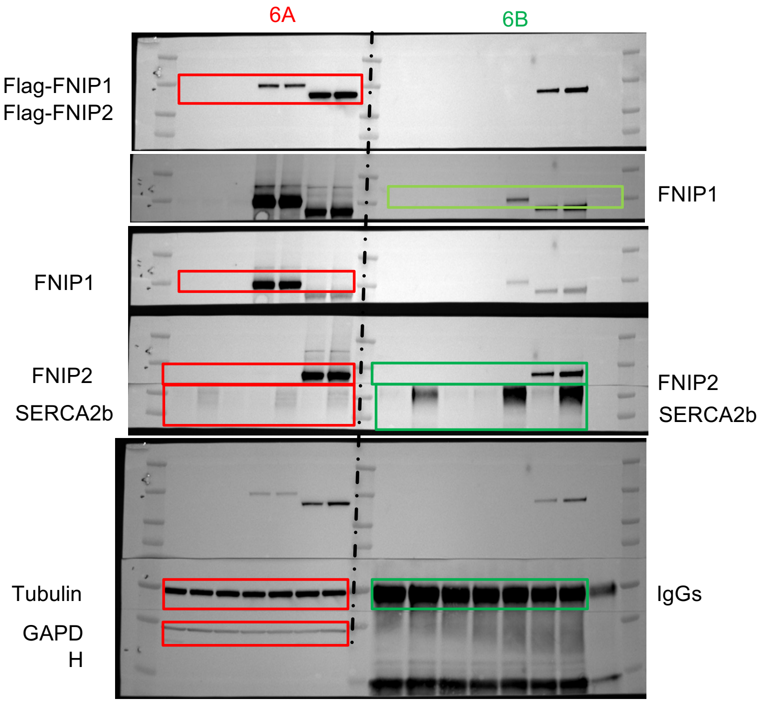

Supplement: Supplementary file 4 — Original Western Blots [file 41419_2026_8507_MOESM4_ESM.pdf]
