# Supplementary material for: Targeting the FNIP2-SERCA2b axis improves metabolic and mitochondrial defects in Ataxia Telangiectasia
Source: Cell Death Dis. 2026 Mar 2;17(1):290. doi: 10.1038/s41419-026-08507-5 (PMC13031930; doi:10.1038/s41419-026-08507-5)
Supplement: Supplementary file 5 — TableS1 [file 41419_2026_8507_MOESM5_ESM.docx]

**Table S1:** General description of individual primary cells analyzed in Set 1

**SET 1**

| **SAMPLES** | **KINSHIP** | **AGE years** | **SEX** | **Clinical Status** | **Mutation** | **Output** | **Clinical features** |
| --- | --- | --- | --- | --- | --- | --- | --- |
| C3 | A1 brother | 8 | M | Healthy | Unknown | Unknown | None |
| C4 | Mother | 37 | F | Healthy- CARRIER | 8366A>T | Truncation Codon 2756 | None |
| C5 | Sister | 11 | F | Healthy | Unknown | Unknown | None |
| C6 | Brother | 17 | M | Healthy | Unknown | Unknown | None |
| C7 | Mother | 42 | F | Healthy- CARRIER | 7913G>A | Term at Codon 2638 | None |
| C8 | A2 Father | 48 | M | Healthy- CARRIER | Unknown | Unknown | None |
| C9 | A2 Mother | 47 | F | Healthy- CARRIER | Unknown | Unknown | None |
| A1 | C3 Brother C4 Son | 7 | M | AT-patient | 8266A>T (mother Ins 4bp at 1141 (father) | Trunc at Codon 2756 Framshift+Trunc at Codon 381 | Neuro X-Ray Sens |
| A2 | C8 Daughter C9 Daughter | 14 | F | AT-patient | Unknown | Unknown | AT and Leukemia |
| A3 | Unmatched | 18 | M | AT-patient | Unknown | Compl. Group D | X-Ray Sens |
| A4 | Unmatched | 15 | F | AT-patient | Homozyg 103 C>T | Stop at Codon 35 | AT |
| A5 | Unmatched | 17 | M | AT-patient | Unknown | Unknown | AT |
| A6 | Unmatched | 8 | M | AT-patient | 19bp Del at 2251 | Framshift+Trunc Codon 750 27 aa In frame Del Codon 2191 | AT |
| G6PD | Unmatched | 12 | M | Favism | 563C>T | S188F | RBC G6PD defective |
| G6PD | Unmatched | 16 | M | Favism | 563C>T | S188F | RBC G6PD defective |
| G6PD | Unmatched | 14 | M | Favism | 563C>T | S188F | RBC G6PD defective |
| G6PD | Unmatched | 8 | M | Favism | 563C>T | S188F | RBC G6PD defective |

**Table S2:** General description of individual primary cells analyzed in Set 2

**SET 2**

| **SAMPLES** | **KINSHIP** | **AGE years** | **SEX** | **Clinical Status** | **Mutation** | **Output** | **Clinical features** |
| --- | --- | --- | --- | --- | --- | --- | --- |
| C1 | Unmatched | 35 | F | Healthy | No | None | None |
| C2 | Unmatched | 31 | M | Healthy | No | None | None |
| C3 | Unmatched | 35 | F | Healthy | No | None | None |
| C4 | Unmatched | 31 | M | Healthy | No | None | None |
| P1(A7) | Unmatched | 7 | M | AT-patient | Homozyg. 7913G>A | C-term Trunc | AT |
| P2(A8) | Unmatched | 14 | F | AT-patient | Unknown | Unknown | AT |
| P3(A9) | Unmatched | 18 | M | AT-patient | Homozyg. 7913G>A | C-term Trunc | AT |
| P4(A10) | Unmatched | 15 | F | AT-patient | Unknown | Unknown | AT |
| P5(A11) | Unmatched | 17 | F | AT-patient | Unknown | Unknown | AT |
| P6(A12) | Unmatched | 8 | M | AT-patient | Homozyg. 7913G>A | C-term Trunc | AT |
